# Complete chloroplast and ribosomal sequences for 30 accessions elucidate evolution of *Oryza* AA genome species

**DOI:** 10.1038/srep15655

**Published:** 2015-10-28

**Authors:** Kyunghee Kim, Sang-Choon Lee, Junki Lee, Yeisoo Yu, Kiwoung Yang, Beom-Soon Choi, Hee-Jong Koh, Nomar Espinosa Waminal, Hong-Il Choi, Nam-Hoon Kim, Woojong Jang, Hyun-Seung Park, Jonghoon Lee, Hyun Oh Lee, Ho Jun Joh, Hyeon Ju Lee, Jee Young Park, Sampath Perumal, Murukarthick Jayakodi, Yun Sun Lee, Backki Kim, Dario Copetti, Soonok Kim, Sunggil Kim, Ki-Byung Lim, Young-Dong Kim, Jungho Lee, Kwang-Su Cho, Beom-Seok Park, Rod A. Wing, Tae-Jin Yang

**Affiliations:** 1 Department of Plant Science, Plant Genomics and Breeding Institute, and Research Institute for Agriculture and Life Sciences, College of Agriculture and Life Sciences, Seoul National University, Seoul, 151-921, Republic of Korea; 2 Phyzen Genome Institute, 501-1, Gwanak Century Tower, Kwanak-gu, Seoul, 151-836, Republic of Korea; 3 Arizona Genomics Institute, School of Plant Sciences, The University of Arizona, Tucson, Arizona, 85721, USA; 4 Department of Horticulture, Sunchon National University, Suncheon, 540-950, Republic of Korea; 5 Biological and Genetic Resources Assessment Division, National Institute of Biological Resources, Incheon, 404-170, Republic of Korea; 6 Department of Plant Biotechnology, Biotechnology Research Institute, Chonnam National University, Gwangju, 500-757, Republic of Korea; 7 Department of Horticultural Science, Kyungpook National University, Daegu, 702-701, Republic of Korea; 8 Department of Life Science, Hallym University, Chuncheon, Kangwon-do, 200-702, Republic of Korea; 9 Green Plant Institute, #2-202 Biovalley, 89 Seoho-ro, Kwonseon-gu, Suwon, Republic of Korea; 10 Highland Agriculture Research Institute, National Institute of Crop Science, Rural Development Administration, Pyeongchang-gun, Kangwon-do, 232-955, Republic of Korea; 11 Department of Agricultural Biotechnology, National Academy of Agricultural Science, Rural Development Administration, Jeonju, 560-500, Republic of Korea

## Abstract

Cytoplasmic chloroplast (cp) genomes and nuclear ribosomal DNA (nR) are the primary sequences used to understand plant diversity and evolution. We introduce a high-throughput method to simultaneously obtain complete cp and nR sequences using Illumina platform whole-genome sequence. We applied the method to 30 rice specimens belonging to nine *Oryza* species. Concurrent phylogenomic analysis using cp and nR of several of specimens of the same *Oryza* AA genome species provides insight into the evolution and domestication of cultivated rice, clarifying three ambiguous but important issues in the evolution of wild *Oryza* species. First, cp-based trees clearly classify each lineage but can be biased by inter-subspecies cross-hybridization events during speciation. Second, *O. glumaepatula*, a South American wild rice, includes two cytoplasm types, one of which is derived from a recent interspecies hybridization with *O. longistminata*. Third, the Australian *O. rufipogan*-type rice is a perennial form of *O. meridionalis*.

Plant cells contain three genomes with different evolutionary origins and history: nuclear, mitochondrial and chloroplastic. Chloroplast (cp) genomes and nuclear ribosomal DNA (nR) units are the primary sequences used to analyze plant genetic diversity as well as evolution^1^,^2^ because mitochondrion (mt) genomes show hyper-variable structure in plant genomes^3^. The cp genomes are 120- to 217-kb circular DNA molecules containing ~30 conserved genes and relatively diverse intergenic spaces (IGSs)^4^,^5^,^6^,^7^,^8^,^9^, and maintained uni-parentally, usually via maternal inheritance^10^,^11^. Within plant nuclear genomes, nR is organized into highly abundant tandemly-repeated transcription units^12^. Due to their conserved roles in ribosome assembly and nucleolus formation, these high-copy nR units have remained homogeneous through concerted genome evolution within species. Four nR gene components usually reside in two independent chromosomal locations, namely the 5S nR (5S) and 45S nR (45S) blocks in higher plants, although some ancient plants such as *Ginkgo biloba*, moss, and algae maintain the 5S and 45S components in one tandem unit^13^,^14^. The 45S blocks include tandemly arrayed copies of the 45S cistron unit, which comprises conserved 18S, 5.8S, and 26S gene clusters, relatively variable internal transcribed spacers (ITS1 and ITS2), and IGSs^13^,^15^.

Although next-generation sequencing (NGS) technology has enabled remarkable progress in understanding nuclear genomics, sequencing of cp genomes and nR units has remained in challenging due to their high-copy characteristics. Whereas more than 500 complete cp genome sequences have been reported in GenBank, complete 45S unit sequences are known for only a few species. Most reported cp genome sequences have been obtained by conventional methods^16^,^17^ but recently, several studies have utilized NGS platforms to obtain complete cp genome sequences using isolated chloroplast DNA or reference cp-guided mapping, followed by significant efforts to fill gaps using PCR and Sanger sequencing^18^,^19^,^20^,^21^,^22^,^23^. Recently, there has been some improvement of efficiency in obtaining complete cp genome and nR sequences by optimization of reference-guided mapping using several criteria and also a computing program^24^,^25^,^26^,^27^. Reference-guided mapping is good approach for studies of related species with the known reference sequences. However, if related reference genome sequences are lacking, *de novo* assembly will be the best way to obtain the complete sequences.

Plant whole-genome shotgun (WGS) sequence data produced by NGS technologies always contains cp sequences to various levels, depending on tissue types and extraction methods used for DNA preparation. Here, we have developed an efficient method, named *de novo* assembly of low coverage WGS (dnaLCW), to assemble those short NGS reads to simultaneously obtain high-quality complete cp genome and nR units. We provide solutions for gap-filling and error correction in sequence assembly without additional efforts such as PCR and Sanger sequencing. We successfully generated new complete cp genome and nR unit sequences for more than 50 species/cultivars with a range of genome sizes. This method greatly facilitates the use of highly informative plastome and nR data to elucidate the evolution of land plants. We have applied this method to our own NGS sequences as well as to publically available NGS sequences for *Oryza* species.

Rice is the most important staple crop for human consumption worldwide. Cultivated rice includes three species/subspecies, *Oryza sativa* ssp. *japonica* (North Asian rice), *O. sativa* ssp. *indica* (South Asian rice), and *O. glaberrima* (African rice), all of which contain the *Oryza* AA-type nuclear genome. The *Oryza* AA genome group consists of eight diploid species distributed worldwide: *O. barthii* (Africa), *O. glaberrima* (Africa), *O. glumaepatula* (South America), *O. longistaminata* (Africa), *O. meridionalis* (Australia), *O. nivara* (Asia), *O. rufipogon* (Asia and Australia), and *O. sativa* (Asia and now worldwide)^28^. The AA genome group is estimated to have diverged 2–3 million years ago (MYA)^28^,^29^, and the eight species likely evolved with unique adaptive traits for each native region over time^28^,^30^. Although molecular data have improved our understanding of the phylogenetic relationships and evolutionary history of the *Oryza* AA genome group, the taxonomical classifications and evolutionary relationships for all eight species in the group have not been not fully resolved.

Here, we report cp and nR sequences for 30 *Oryza* accessions and describe the phylogenomic relationship of wild and cultivated species of the *Oryza* AA genome. We also discuss the origin of cultivated rice and some ambiguous issues for classification and evolution of wild *Oryza* AA genome species.

## Results

### *De novo* assembly of low coverage WGS

**Figure 1:**
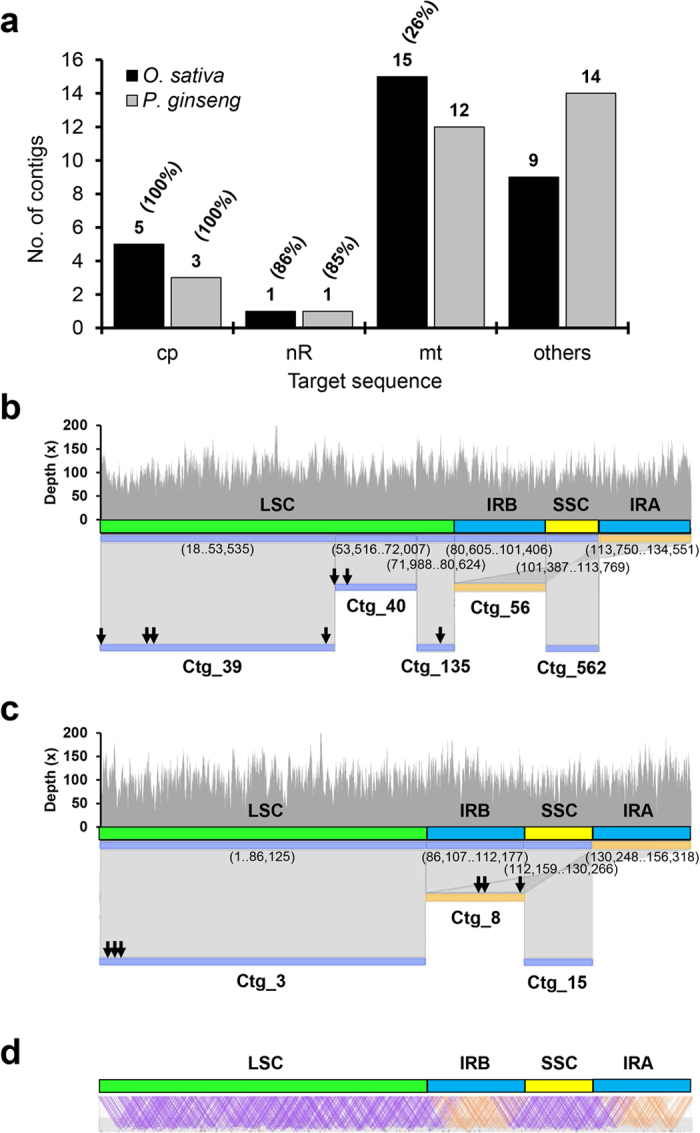
Characterization of the 30 longest contigs derived from *de novo* genome assembly using 1x and 0.05x haploid genome equivalents of rice and ginseng, respectively. (**a**) Classification based on best hit (Supplementary Tables S1 and S2 online). Number of contigs and percent coverage of cp, nR, mt and other sequences are presented above the bars. (**b,c**) Alignment of five and three contigs covering the complete cp genome sequences of rice (**b**) and ginseng (**c**), respectively. The contig numbers are indicated under the contigs and hit positions in parentheses are under the reference cp genome sequences for rice (GU592207) and ginseng (NC_006290). Sequence errors identified in the initial contigs are denoted by arrows. The overall structure of the cp genome is denoted with different colored bars: green, blue, and yellow, for LSC, IRs, and SSC, respectively. Mapping of 100x raw reads is shown above alignment. (**d**) Read mapping of 2x-depth 10-kb mate-pair reads on the assembled sequence. Purple and orange mate information indicates the proper range for 10-kb mate pairs.

We used rice reference cultivar ‘Nipponbare’ (NP)^31^ in order to test whether high-copy components such as cp, mt, and nR sequences could be assembled from low-coverage WGS data. In *de novo* assemblies of rice 1x haploid genome-equivalent WGS data, among the 30 longest assembled contigs were 5, 15, and 1 contigs representing cp, mitochondrial (mt), and nR sequences, respectively, with the remaining 9 contigs representing major repeats, mainly transposable elements (TEs) (Fig. 1a and Supplementary Table S1 online). Importantly, the five cp contigs covered the entire 134,551-bp cp genome with approximately 20-bp overlap between adjacent contigs (Fig. 1b). One 6,889-bp contig covered most of the 45S nR unit (i.e. 86%), while 15 contigs (summing to 130 kb) provided partial coverage of the mt genome (i.e. 26%). Similar results were obtained from *de novo* assembly of 151.5 Mbp *Panax ginseng* (ginseng) WGS data (0.05x whole genome coverage) where 3, 12, and 1 contigs represented cp, mt, and nR sequences, respectively, and the remaining 14 contigs were classified as unknown (Fig. 1a, and Supplementary Table S2 online). The complete cp genome was covered by three contigs that overlapped slightly (Fig. 1c) and 10 kb mate-pair read mapping showed that the three contigs were ordered properly (Fig. 1d). One 9,423-bp contig represented the 45S unit and 12 contigs (38 kb) represented the mt genome.

### Optimization of dnaLCW to obtain complete cp genome sequence

Because we obtained almost complete cp and nR sequences for rice and ginseng with 1x and 0.05x genome equivalent WGS data despite their different genome sizes (430 Mbp and 3,600 Mbp for rice and ginseng, respectively)^31^,^32^, we optimized the WGS dataset size needed to obtain complete cp genome assemblies. As NP and ginseng WGS reads included ~1.7 and 6.0% cp genome-derived reads, respectively, we extracted 10 WGS datasets with between 25x and 5,000x coverage of the cp genome for independent assembly (Supplementary Table S3 online).

We used the number of contigs covering the entire cp genome and the number of assembly errors as criteria for assessment of optimal assembly. Datasets 3–6 with 100x to 250x cp coverage, corresponding to 2–10 x haploid genome equivalents, for rice, showed the best assembly performance for cp genomes, whereas assembly errors and contig numbers in rice rapidly increased when NGS reads reached 20x whole genome coverage (~8.6 Gbp WGS sequence) (Supplementary Table S3 online). This suggests that, with higher amounts of rice input data, short NGS reads originating from nuclear or mitochondrial plastid DNAs (NMPTs; cp sequences inserted into the nuclear or mitochondrial genome) were erroneously co-assembled into cp contigs. The different assembly behavior with regard to input data could be attributable to rice having a higher NMPT content compared to ginseng in which number of assembly errors decreased as increase of cp coverage (Supplementary Table S3 online). Therefore, it is important to use the proper amount of data for assembly to minimize erroneous cp contigs caused by NMPTs.

We compared the performance of two popular genome assemblers, SOAPdenovo^33^ and the CLC *de novo* assembler (http://www.clcbio.com/products/clc-assembly-cell/), in generating small numbers of longer contigs to cover the entire cp genome using various WGS datasets of rice. The CLC *de novo* assembler outperformed SOAPdenovo (Supplementary Fig. S1 online).

### Identification and correction of *de novo* assembly errors

We could construct a single circular draft cp genome by joining the initially assembled overlapping cp contigs. However, we identified several types of assembly errors, such as the positions denoted by arrows in Fig. 1b,c, by aligning PE reads onto assembled contigs. The mis-assembled regions were typically characterized by accumulation of discordantly mapped reads or abnormally higher read mapping depth. The identified assembly errors included false gaps, false SNPs, and copy number errors for TR or monopolymers. We developed detailed *in silico* methods for identification and correction of each type of error (see Materials and Methods, Supplementary Figs S2-S5 online). We obtained a complete 134,551-bp cp genome sequence for NP that was 100% identical to the reference cp sequence of NP (GU592207), using the dnaLCW approach followed by *in silico* correction of seven errors detected in the initial assembly.

### Obtaining complete sequences for major nR units

**Figure 2:**
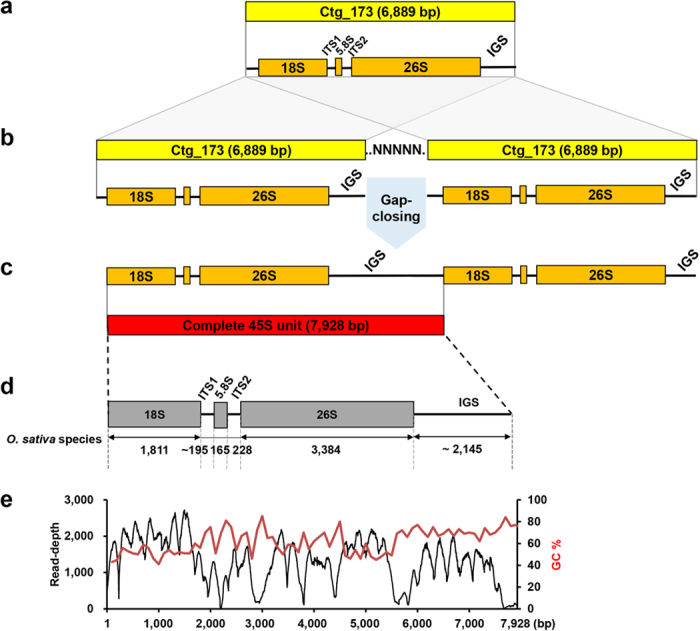
Assembly of complete 45S units. (**a–c**) Schematic diagram of the method used to obtain a complete 45S unit. (**a**) A draft single contig included the 45S transcription unit and occasionally part of the IGS. In this example, Ctg_173 assembled using a rice dataset contained a partial IGS. (**b**) To obtain the full-length IGS, a hypothetical tandem array was constructed using two copies of the contig and intervening Ns. Through a gap-closing process, the Ns were filled in by nucleotide sequences originating from IGS regions. (**c**) If the IGS remains partial, adjustment of the intervening N length and repeated gap-closing will be necessary. Ultimately, a complete 45S unit with the full-length IGS can be obtained. (**d**) Structure of the complete 45S unit of *Oryza* species. (**e**) Status of read mapping on the assembled 45S units. The Os5 dataset was mapped again to assembled single contigs covering the entire 45S unit sequence (black line). Red line indicates GC content per 100-bp unit length.

The dnaLCW assembly also generated contigs representing the 5S and 45S nR units. The initial 5S contigs contained the complete 5S units of 324 bp and 898 bp for NP and ginseng, respectively (Supplementary Figs S6 and S7 online). By contrast, the 45S contig was represented as incomplete contigs longer than 6 kb, including the main 45S transcriptional unit and part of the flanking IGS. We developed a method to extend the IGS sequences based on the highly homogeneous tandemly arrayed nature of the 45S. We generated a two-unit 45S tandem array using the initial contig and manually inserted 100 unknown nucleotides, (N)_100_, between the two units for the remaining gaps in the IGS (Fig. 2). We then applied iterative gap closing to fill the gaps between the units using Gapcloser with the raw reads. Occasionally, GC-rich regions and sub-repeat elements in IGS made gap-filling ineffective (Fig. 2d,e); however, we successfully obtained representative complete 324-bp 5S and 7,928-bp 45S units from NP that were identical to the 5S and 45S tandem array found in chromosome 11 and 9, respectively (Supplementary Fig. S6 online)^34^.

### Complete cp and nR sequences of 30 *Oryza* species

We next applied our method to generate complete cp genome and nR sequences for additional 29 *Oryza* accessions, including five *O. sativa* cultivars (one *japonica* cultivar, two *indica* cultivars, and two cultivars derived from an *indica* x *japonica* hybrid) and 24 *Oryza* wild relatives using WGS data produced by four independent groups in Korea, USA, Australia, and China (Table 1, and Supplementary Figs S8 and S9 online)^30^,^35^,^36^,^37^. The cp genomes varied from 134,296 [*O. glumaepatula* (IRGC88793)] to 134,678 bp [*O. barthii* (WAB0028903, WAB0028952)] among the *Oryza* species and representative InDels between species are shown in Supplementary Fig. S10 online. The complete 5S units varied from 302 to 499 bp due to sequence divergence in the IGS, although the coding sequence was highly conserved (Table 1 and Supplementary Fig. S7 online). The 45S units were 7,745–8,190 bp and sequence variations were more frequent in the IGS region (Table 1 and Supplementary Fig. S11 online).

### Phylogenomic analysis of cultivated rice including *indica-japonica* hybrid cultivars and their ancestors

We obtained cp and nR sequences for 30 accessions that belong to nine *Oryza* species. The nine *Oryza* species included all eight species belonging to the *Oryza* AA genome group, as well as *O. punctata*, the best outgroup *Oryza* BB-genome species for phylogenomic analysis of AA-genome species^28^,^29^.

**Figure 3:**
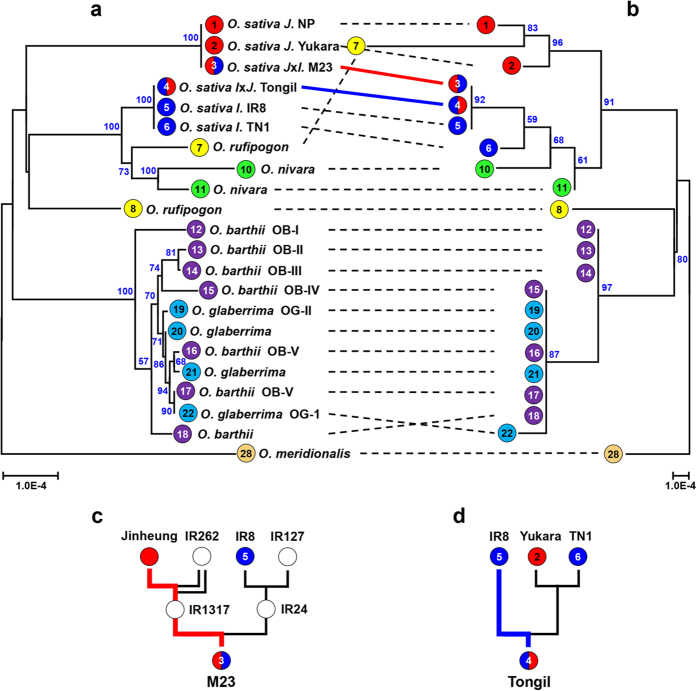
Phylogenomic tree of cultivated rice in Asia and Africa with their putative ancestor species. (**a,b**) Phylogenetic trees were built based on the complete cp genome (**a**) and 45S cistron sequences (**b**). *O. sativa* ssp. *japonica* and *indica* groups are denoted as *J* and *I*, respectively. Two cultivars, M23 (no. 3, red thick line) and Tongil (no. 4, blue thick line), derived from *japonica* x *indica* hybridization and vice versa are denoted as *J*x*I* and *I*x*J*, respectively. Different species/subspecies are indicated with different colored labels. Lines connect the positions of each accession/cultivar in the two trees. Numbers in colored circles represent accessions labeled in Table 1. The phylogenetic tree was generated using Poisson correction and the neighbor-joining (NJ) method in MEGA6. Bootstrap values calculated for 1000 replicates are shown on the branches; the values less than 50% are not shown. (**c,d**) Pedigree of two cultivars, M23 (**c**) and Tongil (**d**), bred by crossing between *O. sativa* ssp *japonica* and *indica*^38^. Red and blue thick lines indicate final maternal genotype backgrounds for M23 and Tongil, respectively.

The phylogenomic analyses based on the cp genomes and on 45S sequences agreed with each other for the most part, with the exception of the placement of *japonica*-*indica* hybrid M23 and one *O. rufipogon* accession (Fig. 3a,b). The cp genome-based tree clearly distinguished *O. sativa* subspecies *japonica* (NP, Yukara) and *indica* (IR8, TN1) from each other. Cultivars Tongil and M23, bred by crossing *O. sativa* ssp. *japonica* and *indica*, had cp genomes identical to the *indica* and *japonica* types, respectively, in accordance with their last maternal parent even though both show *indica*-like plant architecture and nuclear genome sequence (Fig. 3c,d, and Supplementary Figs. S8 and S9 online)^38^,^39^. In the 45S-based phylogenetic analysis, M23, the cultivar derived from *japonica* x *indica* hybridization, belonged to the *indica* group, in accord with its phenotype even though its cp genome was identical to those of *japonica* cultivars due to maternal inheritance^36^. Conversely, the *O. rufipogon* used in this study was grouped with *japonica* based on 45S, but with *indica* based on the cp genome (Fig. 3a,b).

The African cultivated rice *O. glaberrima* and its wild relative *O. barthi* were grouped together by both cp and nR-based trees (Fig. 3a,b). It was previously reported that *O. barthii* accessions are divided into five independent subgroups (OB-I to OB-V) and *O. glaberrima* was domesticated from OB-V^40^. Our cp-based phylogeny showed that all the *O. glaberrima* accessions are grouped with the *O. barthi* OB-V group, and the 45S-based phylogeny is in accordance with the cp-based tree although the OB-IV and OB-V groups are not distinguished (Fig. 3b).

### Phylogenomic analysis of wild *Oryza* AA genome species

**Figure 4:**
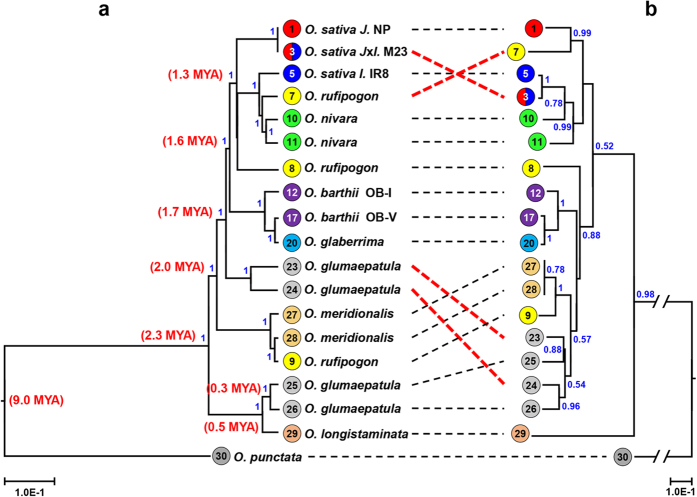
Phylogenomic tree of *Oryza* species. (**a,b**) Phylogenetic trees were built based on the complete cp genome (**a**) and 45S cistron sequences (**b**). *O. sativa* ssp. *japonica* and *indica* groups are denoted as *J* and *I*, respectively. Different species/subspecies are indicated with different colored labels. Numbers in colored circles represent accessions labeled in Table 1. Dashed lines connect the positions of each accession/cultivar in the two trees; red highlights major differences between trees. The tree was generated based on Bayesian Inference analysis using BEAST (version 1.8.1) as mentioned in Materials and Methods. Posterior probability (pp) above 0.5 are shown on the branches. Divergence time was calculated based on 9 million years ago (MYA) when *Oryza* species with AA and BB genome were estimated to be speciation^29^.

Our concurrent analysis using cp and 45S-based phylogenomic analysis revealed that the cp-based trees can be biased by one event of inter-subspecies or inter-species cross-hybridization during speciation, which was shown by one artificially bred cultivar, M23 developed by hybridization between *japonica* and *indica* rice (Fig. 3). Accordingly, we next included several different accessions as representative of each species in analysis to clarify the phylogenomic relationships using cp as well as nR sequences. The topology of the cp-based tree was well in accordance with the 45S-based tree as well as the previous reports with some exceptions (Fig. 4). In addition, phylogenetic analysis based on the maximum likelihood (ML) method (Supplementary Fig. S12 online) produced a similar topology as that in Fig. 4. All accessions belonging to same species were placed into the same clade, with two exceptions. Three *O. rufipogon* specimens were positioned independently by both cp and 45S trees. Accession no. 7 (Chinese *rufipogan*) was intermingled with Asian cultivated rice species. Accession no. 8 (Vietnamese *rufipogan*) was intermediated between Asian and African cultivated rice species. Accession no. 9 (Australian *rufipogan*) was grouped with Australian wild rice, *O. meridionalis*. We also analyzed four different *O. glumaepatula* accessions, and the four South American wild rice accessions were grouped as two independent groups by the cp-based tree. Among four *O. glumaepatula* accessions, nos. 23 and 24 were placed between *O. meridionalis* (Australian AA) and *O. barthi* (African AA), however, accessions nos. 25 and 26 were grouped with the basal AA genome species *O. longistaminata* in the cp-based tree. However, all four accessions were grouped together and independently from *O. longistaminata* by the 45S tree.

## Discussion

### The dnaLCW workflow for simultaneous determination of complete cp and nR sequences

Currently, most leading NGS-read assembly programs use a computational algorithm known as a *de Bruijn* graph, and 15 assemblers have been developed to improve genome assembly^41^. Repeat sequences generally hinder genome assembly; accordingly, many efforts have focused on removing repeat sequences to avoid the noise they create^42^,^43^,^44^,^45^. Conversely, we have developed an efficient workflow to obtain complete cp and nR sequences simultaneously by taking advantage of the high copy genomic elements and using subsequent *in silico* solutions for error correction (see Materials and Methods). We used standard procedures for DNA preparation, PE library construction and Illumina sequencing. Small amounts of NGS data from WGS reads sufficed to assemble complete cp and nR sequences using our approach.

### Origin of cultivated rice species revealed by concurrent cp and 45S-based phylogenomics

*Oryza* species are self-pollinating. The maternally inherited cp genome often most accurately represents the lineage, whereas nuclear rDNA could be intermingled by chance cross-hybridization between evolving sub-groups. However, our results demonstrated that cross hybridization between diverging groups can also produce altered genotype results in cp genome-based phylogenies, as exemplified by the biased positioning of one *japonica-indica* hybrid cultivar, M23 (no. 3 in Figs 3 and 4).

During the last four decades, there have been many breeding efforts involving inter-subspecies hybridization between *indica* and *japonica*. Two famous rice cultivars, Tong-il and M23, were bred by inter-subspecies hybridization and subsequent inbreeding and selection in Korea (Fig. 3c,d)^38^. Both cultivars have an *indica*-type appearance even though a little portion of each genotype is derived from *japonica*-type rice^39^. Our cp-based phylogenetic analysis revealed that the Tongil cp genome is identical with *indica* rice accessions. By contrast, M23 cp genome is identical to those of the *japonica* accessions rather than those of the *indica* group, a finding that is contradictory to the 45S-based tree as well as to the phenotype and genotypes (Fig. 3a,b)^39^. However, this finding is consistent with the breeding history: Tongil likely contains the *indica*-type cp genome and M23 the *japonica*-type cp genome because they were maternally inherited from the inter-subspecies hybridization (Fig. 3c,d). Based on our results, we conclude that the cp genome reports genetic diversity well for most plant species; however, the cp-based phylogeny can be sometimes be biased if there was an inter-species cross hybridization event such as in the case of M23. Similar results are observed from some accessions that were estimated to be derived from natural inter-species pollination, one *O. rufipogon* accession (no. 7), and two *O. glumaepatula* accessions (nos. 23 and 24) (red lines in Fig. 4). Our data illustrate that phylogenomic analysis based on simultaneous use of both cp and nR sequences will further promote elucidation of the relationships among closely related species over approaches using only one type of sequence.

Genome-wide analysis of large collections of *O. sativa* relatives has revealed that Asian *O. rufipogon* genomes are the most diverse and are classified into three major groups, of which Or-I/II and Or-III are thought to be ancestors of the *indica* and *japonica* types of rice, respectively, and *O. rufipogon* W1943 accession (no. 7) is classified as Or-III ecotype^36^, which coincides with our phylogenetic analysis with 45S. However, our cp-based phylogeny indicated that *O. rufipogon* W1943 is close to the *indica* type.

Although the 45S-based phylogeny did not classify the OB-IV&-V group, our cp-based phylogeny clearly distinguished the five *O. barthii* subgroups and demonstrated that the African cultivated rice was domesticated from the OB-V group, in agreement with a previous report (Fig. 3a,b)^40^.

### Phylogenetic relationships of *Oryza* AA genomes

Comparison of genome sequences of *Oryza sativa* and related five AA genome species revealed the rapid diversification of each species^30^. Phylogenetic analyses based on 53 conserved nuclear genes had *O. meridionalis* as the basal species with the AA genome^29^,^30^. By contrast, our analysis showed that *O. longistaminata* is the basal AA genome species, based on the cp genome as well as nR sequence (Fig. 4). Although there is contradictory positioning of *O. longistaminata* and *O. meridionalis* between these two analyses (Our cp-based tree vs. ref. 44), the overall topology is in agreement with other previous reports^46^,^47^,^48^,^49^,^50^,^51^. The cp-based phylogeny also showed very rapid divergence of AA genome species during the last 2.3 million years, in agreement with data based on the nuclear genome (Fig. 4)^29^,^30^.

In this work, we analyzed several different specimens as representatives of the same species, an approach that differs from the other previous studies and promotes better understanding of the divergence of wild species. Our analysis revealed that there were very recent cp genome exchanges between diverging species such as two *O. glumaepatula* accessions (nos. 23 and 24) which have *O. longistaminata*-like cp genomes even though the rDNA and phenotypes are similar to two other distinct *O. glumaepatula* accessions (nos. 25 and 26) (Fig. 4). We assume that there was a cross pollination event with some ancestor of *O. glumaepatula* (such as accessions nos. 23 and 24) as the male and *O. longistaminata* as the female between 0.5–0.3 million years ago (MYA) (Fig. 4). As described above, we identified and confirmed a similar phenomenon in one inter-subspecies hybridization-derived cultivar, M23, which was bred by cross-hybridization between *O. sativa* ssp*. japonica* x *O. sativa* ssp. *indica* according to the breeding history (Fig. 3c).

Three *O. rufipogon* specimens were placed in different groups. The Chinese *O. rufipogon* specimen (W1943, no. 7) was grouped with *O. nivara* and *O. sativa*. A wild specimen from Vietnam (AC11-1008369, no. 8) was placed independently as basal group of *O. sativa* indicating that it is a wild ancestor of *O. sativa*, in agreement with previous reports (Fig. 4a)^36^,^52^,^53^. However, another wild *O. rufipogon* specimen from Australia (AC01-1002323, no. 9) was grouped with *O. meridionalis*, indicating that the naming of the species should be reconsidered, consistent with a previous suggestion that the Australian *O. rufipogon* may be a perennial form of *O. meridionalis*^52^.

### Advantage of complete cp genome information for barcoding within species

The diversity of the cp genome within the genus or species level is an advantage to analyze domestication of crop plants and for development of barcoding markers for certain cultivars. The origin of domesticated apples has been clarified based on 47 cp genome sequence of *Malus* species^23^. The cp genomes of ginseng and American ginseng (*P. quinquefolius*) showed 138 SNPs and 40 InDels relative to one another (Supplementary Figs. S13 and S14 online). Although there is abundant polymorphism between different *Panax* species, previous PCR surveys did not detect polymorphism among *P. ginseng* cultivars in the cp intergenic regions^54^,^55^. In this study, we could identify one SNP and two InDels between the *P. ginseng* cultivars ChP and YP (Supplementary Figs. S13 and S14 online), and one SNP and three InDels between ChP and three reported *P. ginseng* specimens from China (GenBank Accession nos. KC686331, KC686332, KC686333). We further identified a total of six SNPs and six InDels by comparison of nine more complete cp genomes of *P. ginseng* cultivars or landraces^56^. By contrast, when compared with the previously reported wild *P. ginseng* cp genome (NC_006290)^57^, our *P. ginseng* cp genome sequences showed 117 SNPs and 51 InDels, likely due to differences in plant material and/or sequencing errors (Supplementary Fig. S13 online). We also applied this approach to complete cp genomes of tartary buckwheat and three onion accessions to identify useful barcoding markers to classify adjacent species^58^ and different onion cytotypes^59^.

### Evolution of nR DNA

The copy number of nR unit varies in different plant genomes. The copy numbers of each nR was estimated based on average depth coverage of 1x genome-equivalent WGS reads (Table 1) and the value was roughly in accord with, but slightly lower than, estimates derived from FISH signals, likely because FISH signals amplify the hybridization signal by rendering a two dimensional signal from the three dimensional chromosome structure (Table 1 and Supplementary Fig. S15 and Table S4 online). We found a remarkable range of estimated copy numbers for 5S and 45S. The 5S copy and 45S copy number estimates ranged from 69–6,045 and 131–2,292, respectively, among *Oryza* species (Table 1). This raises the question of whether there is any association of rDNA diversity and copy number variation with biological function.

The 5S and 45S units are found in independent chromosomal regions in rice (Supplementary Figs. S6 and S15 online) and in most genomes of higher plants. By contrast, the 45S and 5S units co-exist as one tandem repeat unit in some ancient plants^13^,^14^, and it is assumed that the single unit was divided into separate 45S and 5S units in higher plants during evolution. However, it is not clear how and when the units evolved because of the lack of complete nR unit sequences from diverse plants. Our high throughput approach can thus contribute to revealing how nR evolved in the plant kingdom.

## Methods

### Preparation of whole-genome NGS reads

Leaf samples were harvested from plants of rice and ginseng grown in a farm of Seoul National University, Suwon, Korea, and high-quality genomic DNA was extracted using a modified CTAB method^60^. A paired-end (PE) library with 500-bp insert size was constructed using the Illumina PE DNA library kit according to the manufacturer’s instructions and sequenced using an Illumina Hiseq2000 by the National Instrumentation Center and Environmental Management (NICEM, http://nicem.snu.ac.kr/, Korea) and Macrogen (http://dna.macrogen.com/, Korea) and Illumina MiSeq or NextSeq500 by LabGenomics (www.labgenomics.co.kr, Korea). Illumina Hiseq2000 reads of six *O. sativa* and eight related *Oryza* species were provided by Prof. Hee-Jong Koh (Seoul National University, Korea) and the Arizona Genomics Institute (AGI, http://www.genome.arizona.edu/, USA), respectively. WGS sequence data of additional *Oryza* species were downloaded from the SRA database and used for assembly of cp genomes and nR sequences (Table 1).

### WGS assembly and building of complete cp genome and nR sequences

Raw reads with Phred scores of 20 or less were removed from among the total NGS PE reads using the CLC-quality trim tool (quality_trim software included in CLC ASSEMBLY CELL package ver. 4.06 beta. 67189, http://www.clcbio.com/products/clc-assembly-cell/). In assemblies of WGS reads representing more than 70x genome coverage in rice and ginseng, we identified no proper long, unique cp contigs. We then tested assembly of cp genome and nR using low-coverage WGS sequences. Sub-datasets with various levels of cp genome coverage were extracted from trimmed NP and ChP WGS reads and assembled using the CLC *de novo* assembler included in the CLC ASSEMBLY CELL package or SOAPdenovo included in the SOAP package (ver. 1.12) with default parameters. Sequence gaps were filled by Gapcloser included in the SOAP package (ver. 1.12). Representative contigs for the cp genome or nRs were retrieved from the total assembled contigs using Nucmer^61^ with reference sequences. Extracted contigs were ordered and oriented based on built-in BLASTZ analysis (http://nature.snu.ac.kr/tools/blastz_v3.php)^62^ with the cp sequence of related genome and then connected into single draft sequence by joining overlapping terminal sequences.

### In silico finishing: Identification and correction of errors in dnaLCW assembly

Tentative error sites were identified by mapping raw reads to draft sequences using the CLC mapping tool (clc_ref_assemble in the CLC ASSEMBLY CELL package) and visualized using CLC viewer (clc_assembly_viewer in the CLC ASSEMBLY CELL package). The mis-assembled error sequences were characterized by accumulation of discordantly mapped reads or abnormally higher read mapping depth. The identified assembly errors were classified as false gaps, false SNPs, or copy number errors for tandem repeats (TR) or monopolymers. Each type of errors was corrected by following *in silico* manual curation and validated by PCR amplification and Sanger sequencing.

1. **False gaps:** This type of error occurs in the regions where ambiguous “N” nucleotides present in draft assembly contigs. The left and right sequence flanking an “N” are duplicated, leading to accumulation of commonly mis-mapped reads at the flanking regions (Supplementary Fig. S2 online). Such errors can be corrected by merging the common duplicated sequences flanking the “N”, and the correction validated by re-mapping reads on the edited sequence. If the edited sequence is correct, read mapping will show clear matches on the sequence.
2. **False SNPs:** DNA fragments homologous to those of the cp genome are ubiquitous in mitochondrial and nuclear genomes of rice^63^,^64^ and can interfere with cp genome assembly^44^, leading to create false SNPs (Supplementary Fig. S3a online). Each false SNP could be corrected by assigning the consensus nucleotide sequence to the false SNP location based on the reads showing the highest depth in the paired read mapping, because ~8–100-fold more reads originate from the cp genome than from the nuclear or mitochondrial genome. For example, the assembly of the Os5 dataset, which provides 4x and 200x coverage of the nuclear and cp genomes, respectively, showed two false SNPs, G/T at 51,940 nt and T/A at 51,944 nt (Supplementary Fig. S3b online). The 212 reads mapped to the region revealed clear patterns of origin, in which 186 reads (from the cp) contained T and A nucleotides at those positions, 24 reads (from the mt) contained G and T, and 2 (from the nucleus) contained T and T. Overall, false SNPs in the initial contigs can be easily corrected using read mapping followed by assigning the consensus nucleotide with the highest depth.
3. **Tandem repeat copy number error:** There are many chances for copy number error to arise during *de novo* assembly using short reads^42^,^43^,^45^. Our data show that 18-bp TR units were mis-assembled into 2 copies by default assembly options, whereas four complete copies of 18-bp TRs were correctly assembled with using a k-mer length of 64 (Supplementary Fig. S4a online). When repeats are shorter than the read length, increasing the k-mer value above the TR unit length can reduce mis-assembly. Copy number errors in the assembly can be identified by comparing read-depth at the TR and the flanking region. If raw reads map to a region incorrectly assembled with too few copies of a TR, mis-mapped reads will be abundant and abnormal high read-depth can be found at the collapsed regions (Supplementary Fig. S4b,c online). Most TR units found in cp genomes are simple and less than 100 bp, unlike those in the nuclear genome. Therefore, most errors derived from copy number variance of TRs can be fixed.
4. **Monopolymer copy number error:** A total of 95 and 91 regions contained monopolymer tracts of more than 8 nt in the cp genomes of NP and ChP, respectively. Most monopolymers were poly A or T (Supplementary Table S5 online). Monopolymer regions in the cp genome are susceptible to sequencing errors due to polymerase slippage and mis-assembly caused by interruption of homologous mitochondrial or nuclear sequences containing monopolymers of different lengths. Such monopolymer assembly error was detected at the poly T tract region at 78,424 bp in the NP cp genome (Supplementary Fig. S5a online). Similar sequences with different poly T tracts (7, 8, 9, 10, 11, 12, 15 and 17 nt long) were found in 10 chromosomal regions of the NP nuclear genome (Supplementary Fig. S5b online). The initial assembly of the Os3 dataset generated a (T)_8_ monopolymer tract assembly error caused by interruption with T monopolymers derived from sequences of rice chromosome 5, 6, 7, and 9 (Supplementary Fig. S5b online). This error could be corrected by selection of T monopolymer tracts showing the highest read-depth after raw-read mapping on hypothetical T monopolymer sequences with 100% identity. The draft sequence with the correct (T)_17_ monopolymer among the eight putative sequences showed the highest mapping depth of 33.14, as expected (Supplementary Fig. S5c online).

### Annotation and comparative analysis of cp and nR sequence

The cp genome sequence was annotated using the DOGMA program (http://dogma.ccbb.utexas.edu/)^65^ and BLAST searches. Circular and comparative maps of the cp genome were generated using OGDRAW (http://ogdraw.mpimp-golm.mpg.de/)^66^ and mVISTA (http://genome.lbl.gov/vista/mvista/submit.shtml)^67^, respectively. The gene structure of rRNAs, ITS, and IGS in assembled 45S sequences were determined by comparison with reported sequences and BLAST searches. Phylogenetic tree construction and the reliability assessment of internal branches were conducted by the neighbor-joining method with 1,000 bootstrap replicates using MEGA6^68^ (Fig. 3). The phylogenetic tree and molecular clock dating was generated based on Bayesian Inference analysis using BEAST (version 1.8.1)^69^ (Fig. 4). We performed 10 million generation of MCMC and sampled every 1000 generations, effective sample size of 592 with parameter as an uncorrelated lognormal relaxed-clock model, with Yule prior on the tree, general time reversible (GTR + I + Γ) as a substitution model and the default priors for generated random starting tree. The BEAST runs were obtained using Tracer (version 1.6) after discarding as burn-in of 10% of generations and the remaining was used to estimate the posterior probability. Numbers on each branches indicate posterior probability (pp) (>0.5). The divergence times were calculated using TreeAnnotator (version 1.8.1) and constrained to be 9 MYA as root age based on recently reported divergence time between *Oryza* AA genome and BB genome (*O. punctata*)^29^.

### Validation of polymorphic regions in cp genome sequences

Specific primers were designed from conserved sequences flanking polymorphic regions such as SNPs and InDels found among cp genomes (Supplementary Table S6 online). Genomic DNA was used as template for PCR amplification using Ex-*Taq* polymerase (Takara, Japan) and the amplified fragments were analyzed using a Fragment Analyzer (Advanced Analytical Technologies Inc., USA), according to manufacturer’s instructions. DNA fragments amplified using dCAPS primers were digested with appropriate restriction enzyme and then separated by a Fragment Analyzer (Advanced Analytical Technologies Inc., USA).

## Additional Information

**Accession codes:** Whole-genome NGS reads used in this study have been deposited in the NCBI Sequence Read Archive (SRA; http://www.ncbi.nlm.nih.gov/sra/) and final assembled cp genome and nR unit sequences from this study were deposited in the GenBank database (for accession number, see Table 1).

**How to cite this article**: Kim, K. *et al.* Complete chloroplast and ribosomal sequences for 30 accessions elucidate evolution of *Oryza* AA genome species. *Sci. Rep.* **5**, 15655; doi: 10.1038/srep15655 (2015).

## Supplementary Material

Supplementary Information

## Figures and Tables

**Table 1:** Statistics for assembly of cp and nR sequences from 30 *Oryza* species.

	Species	Genome size (Mbp)	WGS reads for cp assembly			Complete sequence (bp)			Estimated copy number^b^	
			Amount (Mbp)	Coverage (x)						
				Genome	Cp	Cp	45S^a^	5S^a^	45S	5S
1	*O. sativa J*. NP	430	860 (SRR1178954^c^)	2.0`	99	134,551 (KM088016^c^)	7,928 (KM036282^c^)	324 (KM036298^c^)	390	593
2	*O. sativa J*. Yukara	430	303 (SRR1182447)	0.7	227	134,551 (KM088017)	7,929 (KM036283)	324 (KM036299)	186	458
3	*O. sativa J*x*I*. M23	460	303 (SRR1182807)	0.7	147	134,551 (KM103382)	8,160 (KM036287)	322 (KM036303)	131	971
4	*O. sativa I*x*J*. Tongil	460	253 (SRR1182443)	0.5	69	134,502 (KM103369)	8,160 (KM036286)	322 (KM036302)	255	1,593
5	*O. sativa I*. IR8	460	327 (SRR921498)	0.7	251	134,502 (KM103367)	8,166 (KM036284)	322 (KM036300)	140	746
6	*O. sativa I*. TN1	460	303 (SRR921505)	0.7	206	134,502 (KM103368)	8,164 (KM036285)	322 (KM036301)	210	894
7	*O. rufipogon* (W1943)^36^	439	500 (ERX096841)	1.1	128	134,510 (KM103372)	8,004 (KM117266)	303 (KM036304)	275	495
8	*O. rufipogon* (AC11-1008369)^37^	380^34^	1,442 (SRX480817)	3.8	245	134,586 (Dataset S1)	6,090^d^ (Dataset S2)	322 (Dataset S3)	793	2,718
9	*O. rufipogon* (AC01-1002323)^37^	380^34^	712 (SRX480820)	1.9	685	134,572 (Dataset S1)	5,816^d^ (Dataset S2)	499 (Dataset S3)	310	354
10	*O. nivara* (IRGC88812)^30^	395^35^	571 (SRX809784)	1.5	203	134,483 (Dataset S1)	5,823^d^ (Dataset S2)	322 (Dataset S3)	439	5,561
11	*O. nivara* (IRGC100897)	448	775 (SRR1264534)	1.4	112	134,516 (KM088022)	7,904 (KM036288)	322 (KM036305)	441	1,129
12	*O. barthii* (WAB0028976)	411	159 (SRX502175)	0.4	91	134,585 (KM103378)	7,835 (KM117256)	325 (KM117247)	667	4,418
13	*O. barthii* (WAB0028903)	411	437 (SRX502171)	1.1	257	134,678 (KM103379)	7,845 (KM117257)	325 (KM117248)	748	2,380
14	*O. barthii* (WAB0028952)	411	441 (SRX502173)	1.1	319	134,678 (KM103380)	7,845 (KM117258)	325 (KM117249)	771	1,291
15	*O. barthii* (WAB0028987)	411	163 (SRX502178)	0.4	97	134,613 (KM103381)	7,836 (KM117252)	325 (KM117250)	368	1,989
16	*O. barthii* (IRGC101252)^30^	376^35^	1,113 (SRX809864)	3.0	1,277	134,598 (Dataset S1)	5,888^d^ (Dataset S2)	325 (Dataset S3)	3,769	5,984
17	*O. barthii* (IRGC100934)	411	343 (SRX502164)	0.8	113	134,598 (KM103371)	7,836 (KM117253)	325 (KM117251)	630	4,414
18	*O. barthii* (W1588)	411	375 (SRR1264535)	0.9	60	134,590 (KM088023)	7,836 (KM036290)	325 (KM036307)	617	6,045
19	*O. glaberrima* (IRGC104574)	357	330 (SRX502311)	0.9	343	134,586 (KM103377)	7,836 (KM117254)	325 (KM117246)	198	1,820
20	*O. glaberrima* (IRGC96717)	357	188 (SRR1181643)	0.5	50	134,598 (KM088021)	7,836 (KM036289)	325 (KM036306)	455	3,670
21	*O. glaberrima* (IRGC103486)^30^	370^35^	647 (SRX809780)	1.7	218	134,614 (Dataset S1)	5,899^d^ (Dataset S2)	325 (Dataset S3)	773	6,447
22	*O. glaberrima* (IRGC103937)	357	268 (SRX502309)	0.8	257	134,598 (KM103370)	7,836 (KM117255)	325 (KM117245)	358	2,374
23	*O. glumaepatula* (W1187)	400	1,536 (SRR1997915)	3.9	1,338	134,606 (KR364802)	6,427^d^ (KR364804)	440 (KR364807)	1,461	528
24	*O. glumaepatula* (IRGC88793)^30^	366^35^	397 (SRX809892)	1.1	91	134,296 (Dataset S1)	5,841^d^ (Dataset S2)	440 (Dataset S3)	554	278
25	*O. glumaepatula* (W2201)	400	1,728 (SRR1997912)	4.3	1,446	134,575 (KR364803)	5,830^d^ (KR364805)	440 (KR364806)	2,292	1,107
26	*O. glumaepatula* (GEN1233)	464	253 (SRR1264537)	0.5	78	134,575 (KM103374)	8,074 (KM036292)	460 (KM036309)	598	191
27	*O. meridionalis* (IRGC105298)^30^	388^35^	760 (SRX809898)	2.0	270	134,555 (Dataset S1)	5,839^d^ (Dataset S2)	499 (Dataset S3)	1,367	731
28	*O. meridionalis* (W2112)	435	1,000 (SRR1264536)	2.3	109	134,556 (KM103373)	8,190 (KM036291)	499 (KM036308)	461	2,525
29	*O. longistaminata* (IRGC110404)	352	563 (SRR1264538)	1.6	187	134,558 (KM088024)	7,844 (KM036293)	302 (KM036310)	200	69
30	*O. punctata* (IRGC105690)	423	250 (SRR1264539)	0.6	60	134,604 (KM103375)	7,745 (KM036294)	326 (KM036311)	307	1,317

^a^ The lengths of the most redundant and longest representative nR units are given for each species. The 45S transcription units were 5,769–5,783 bp long for *Oryza* species. We cannot rule out the presence of other nR units in each species because there is some variance in the length of the IGS.

^b^Copy numbers of 45S and 5S are based on the average depth of raw reads mapping to each sequence and were calculated based on the 1x haploid genome equivalent of raw reads.

^c^SRA and accession numbers of reads and assembled sequences deposited in GenBank.

^d^Length of 45S transcription units.
