# Supplementary material for: Complete chloroplast and ribosomal sequences for 30 accessions elucidate evolution of Oryza AA genome species
Source: Sci Rep. 2015 Oct 28;5:15655. doi: 10.1038/srep15655 (PMC4623524; doi:10.1038/srep15655)
Supplement: Supplementary Information [file srep15655-s1.pdf]

Supplementary Information

Complete chloroplast and ribosomal sequences for 30 accessions elucidate evolution of *Oryza* AA genome species

Kyunghee Kim[1,2,†], Sang-Choon Lee[1,†], Junki Lee[1,†], Yeisoo Yu[2,3,†], Kiwoung Yang[1,4], Beom-Soon Choi[2], Hee-Jong Koh[1], Nomar Espinosa Waminal[1], Hong-Il Choi[1], Nam-Hoon Kim[1], Woojong Jang[1], Hyun-Seung Park[1], Jonghoon Lee[1], Hyun Oh Lee[1,2], Ho Jun Joh[1], Hyeon Ju Lee[1], Jee Young Park[1], Sampath Perumal[1], Murukarthick Jayakodi[1], Yun Sun Lee[1], Backki Kim[1], Dario Copetti[3], Soonok Kim[5], Sunggil Kim[6], Ki-Byung Lim[7], Young-Dong Kim[8], Jungho Lee[9], Kwang-Su Cho[10], Beom-Seok Park[11], Rod A. Wing[3], and Tae-Jin Yang[1,*]

[1]Department of Plant Science, Plant Genomics and Breeding Institute, and Research Institute for Agriculture and Life Sciences, College of Agriculture and Life Sciences, Seoul National University, Seoul, 151-921, Republic of Korea; [2]Phyzen Genome Institute, 501-1, Gwanak Century Tower, Kwanak-gu, Seoul, 151-836, Republic of Korea; [3]Arizona Genomics Institute, School of Plant Sciences, The University of Arizona, Tucson, Arizona, 85721, USA; [4]Department of Horticulture, Sunchon National University, Suncheon, 540-950, Republic of Korea; [5]Biological and Genetic Resources Assessment Division, National Institute of Biological Resources, Incheon, 404-170, Republic of Korea; [6]Department of Plant Biotechnology, Biotechnology Research Institute, Chonnam National University, Gwangju, 500-757, Republic of Korea; [7]Department of Horticultural Science, Kyungpook National University, Daegu, 702-701, Republic of Korea; [8]Department of Life Science, Hallym University, Chuncheon, Kangwon-do, 200-702, Republic of Korea; [9]Green Plant Institute, #2-202 Biovalley, 89 Seoho-ro, Kwonseon-gu, Suwon, Republic of Korea; [10]Highland Agriculture Research Institute, National Institute of Crop Science, Rural Development Administration, Pyeongchang-gun, Kangwon-do, 232-955, Republic of Korea; [11]Department of Agricultural Biotechnology, National Academy of Agricultural Science, Rural Development Administration, Jeonju, 560-500, Republic of Korea

[†]These authors contributed equally to this work.

[*]Corresponding author

Correspondence and requests for materials should be addressed to T.J.Y. (email: tjyang@snu.ac.kr)

Supplementary Figures

Dataset	assembled by SOAPdenovo	assembled by CLC *de novo*
**Os2 (50x)**	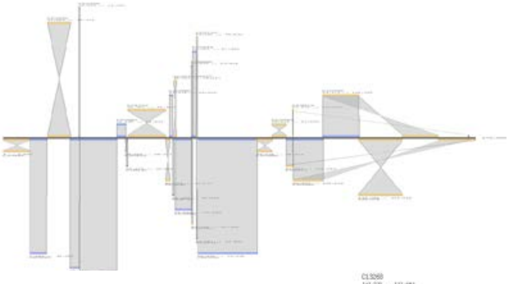	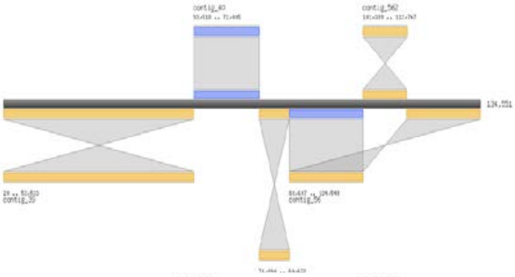
**Os3 (100x)**	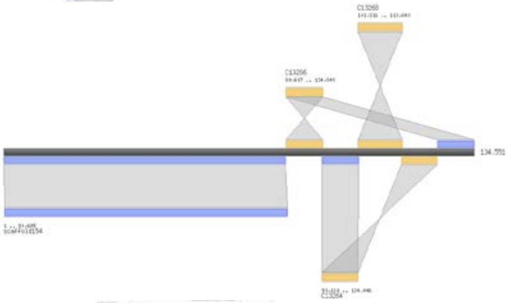	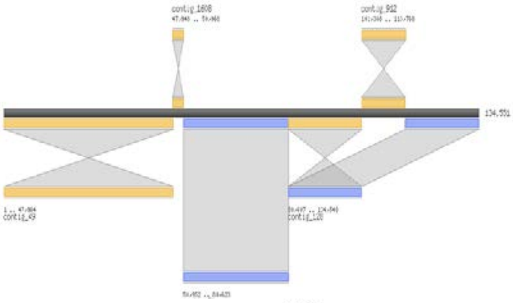
**Os6 (250x)**	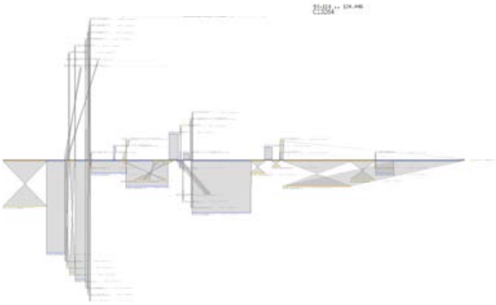	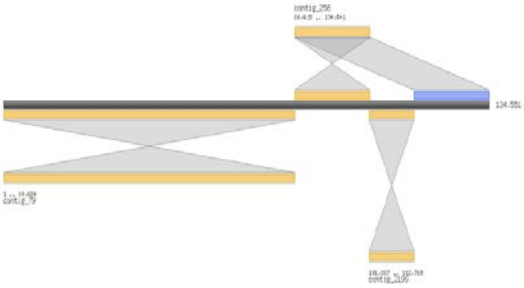

Supplementary Fig. S1. Comparison of rice cp contigs assembled by SOAPdenovo and the CLC *de novo* assembler. Assembled cp contigs from datasets Os2, Os3 and Os6, (Supplementary Table S3) were aligned to the corresponding rice cp genome sequence. Blue and orange bars indicate contigs matching to the reference sequence in forward and reverse orientations, respectively.

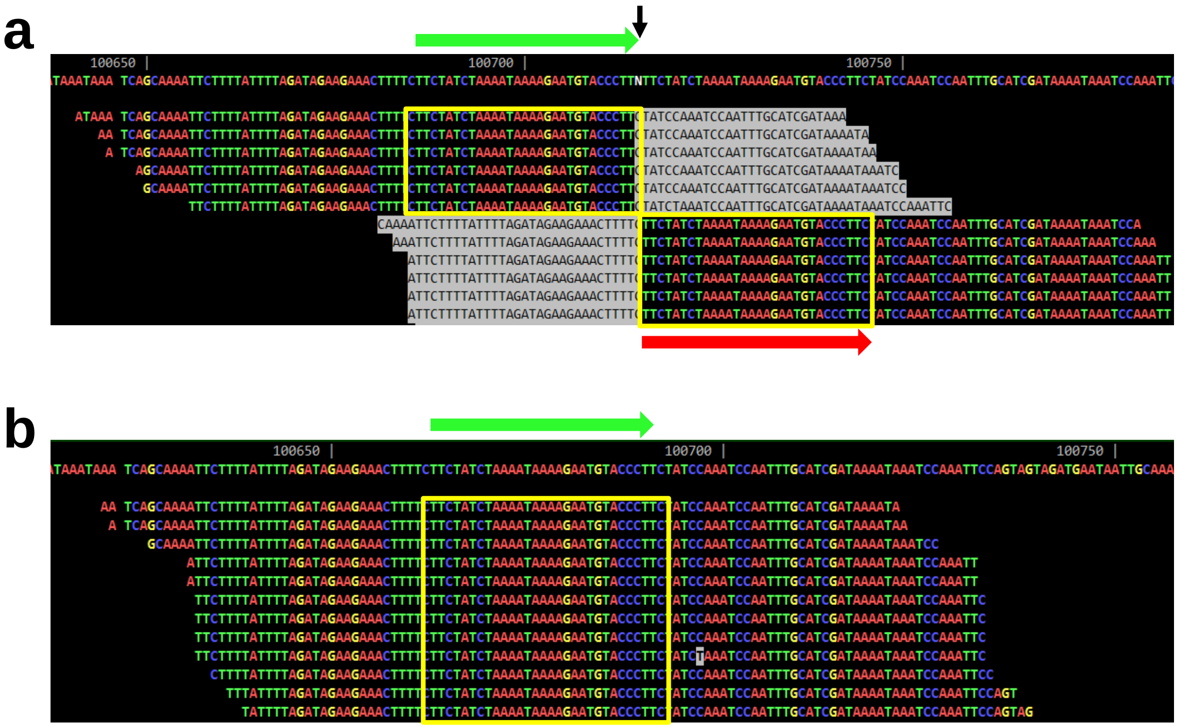

Supplementary Fig. S2. Correction of assembly error represented as a false gap. a, Raw read mapping on the mis-assembled initial contig. Many raw reads flanking an N (black arrow) were mis-mapped (gray regions) because the mis-assembled contig had a false 30-bp duplication (red arrow). **b,** Raw read mapping on the corrected cp contig sequence.

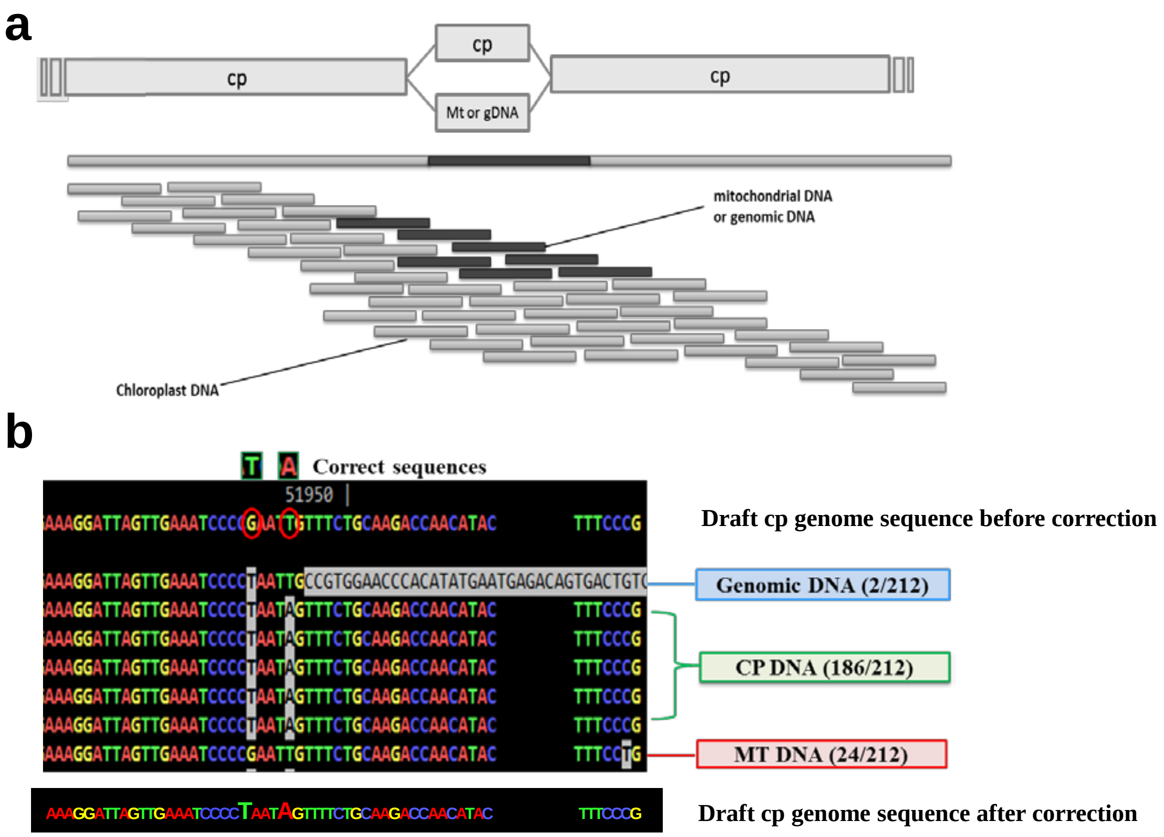

Supplementary Fig. S3. Correction of false SNP assembly error caused by interruption with homologous reads derived from the mitochondrial or nuclear genome. a, Schematic diagram of false SNP. Mis-assembly can be caused by interruption with homologous reads derived from mitochondrial and/or nuclear genomes during assembly of the cp contig. **b,** Correction of false SNP based on selection of the highest read depth. Nucleotides G and T at the 51,940 nt and 51,944 nt positions, respectively, are mis-assembled due to incorporation of mt reads instead of cp reads during assembly of the Os5 dataset (200x cp genome coverage). The correct nucleotides, T and A, instead of G and T, can be identified from the cp reads with the highest read-depth.

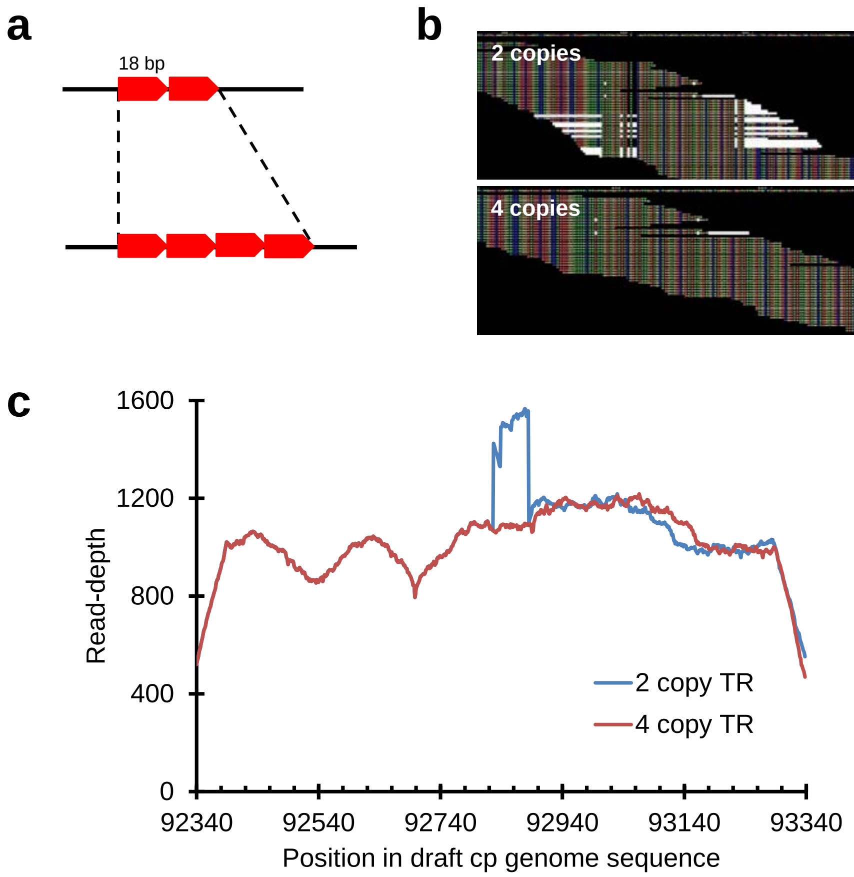

Supplementary Fig. S4. Assembly error derived from copy number reduction of tandem repeats (TRs). a, Schematic diagram showing incorrect TR copy number reduction from four to two 18-bp unit TRs. **b,** Raw read mapping on cp contigs with two (top) or four (bottom) TRs. Many instances of incorrect read mapping (white reads) are detected on the two-copy TR contig. **c,** Distribution of raw read mapping depth on cp contigs with two (blue) or four (red)-copy TRs. Abnormally high read depth is exhibited in the TR region of the two-copy TR contig because of accumulation of mis-mapping.

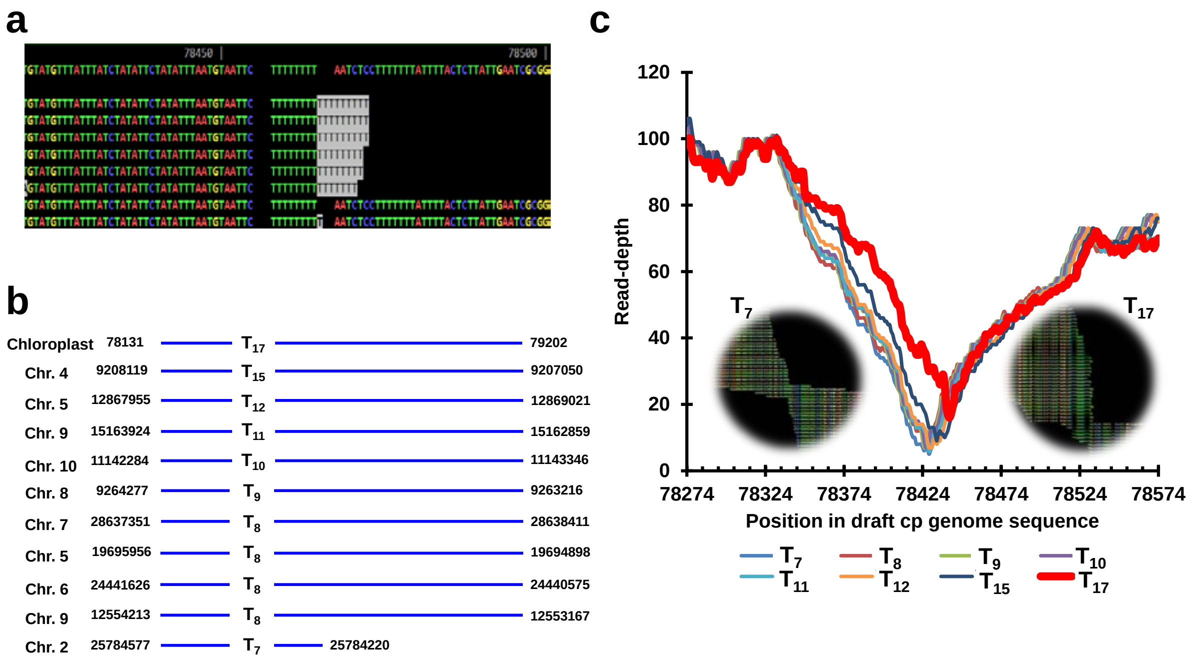

Supplementary Fig. S5. Assembly error at monopolymer regions. a, Mis-assembled poly-T tracts shown in the CLC viewer. Poly-T tracts of various length were identified from raw read mapping of the Os3 dataset (100x coverage to cp genome). **b,** The position and types of sequences containing poly-T tracts in the rice chloroplast genome and individual chromosomes. Reads from poly T tracts in chromosomes might interrupt and cause mis-assembly of the cp genome sequence. **c,** Distribution of perfect-match read-depth on assemblies with various lengths of poly-T tracts. Eight cp contigs were generated with eight lengths of poly T, T_7 ~ T_{17}, and raw reads were mapped on each cp contig with the perfect-match option. The highest number of perfect-match reads was mapped on the T_{17}-tract contig, indicating the 17 is the correct number for the poly-T tract at that position.

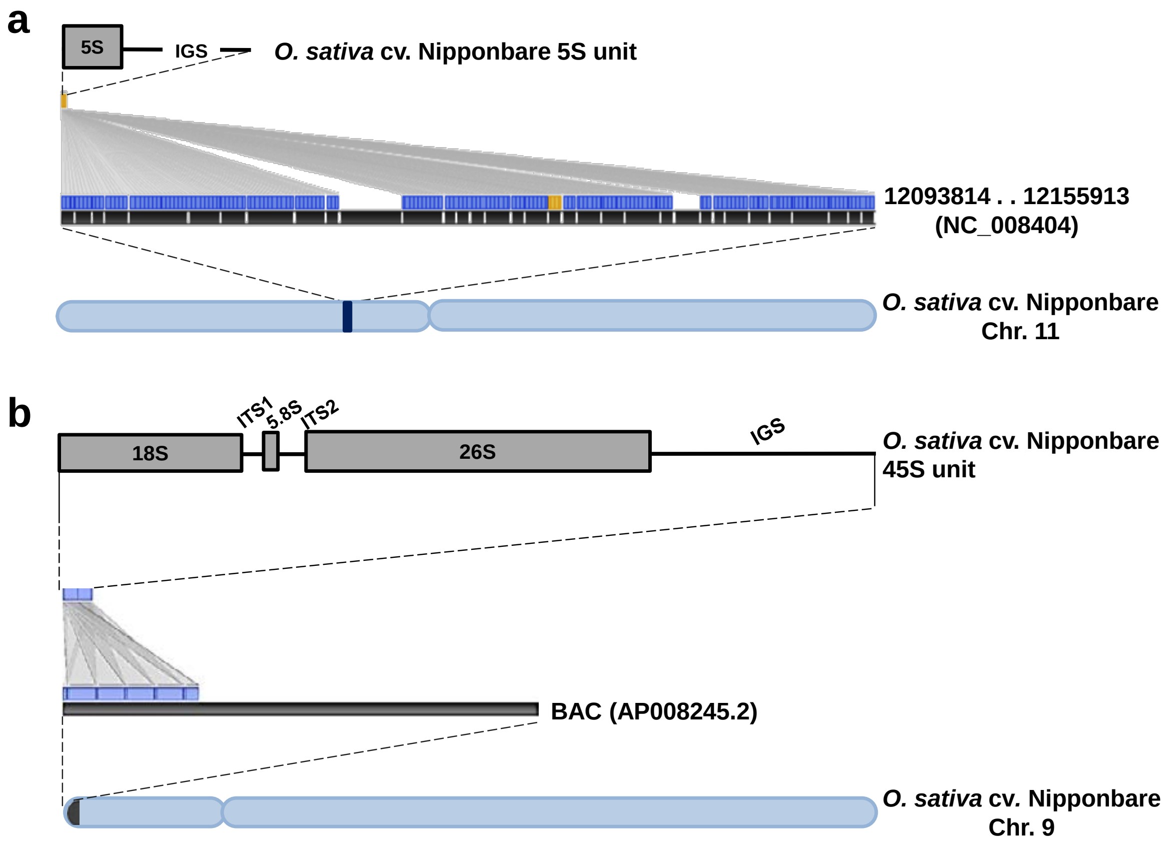

Supplementary Fig. S6. Structure of 5S and 45S contigs and comparison with their homologous sequences in rice pseudo-chromosome sequence. a, Structure of the complete 5S unit assembled in this study and tandem array of the 5S units on a region of rice chromosome 11. **b,** Structure of the complete 45S unit assembled in this study and tandem array of the 45S units on rice BAC sequence located on rice chromosome 9.

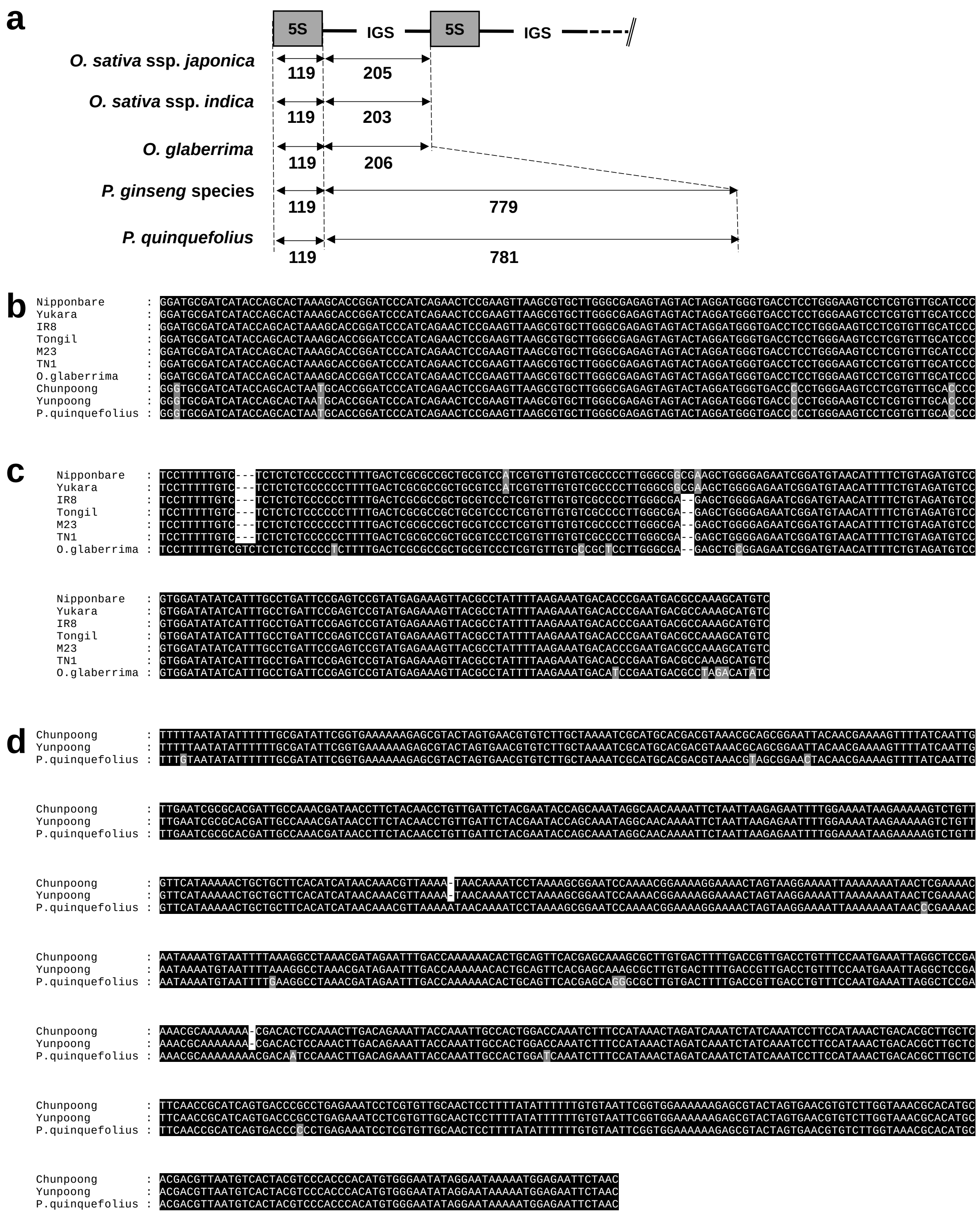

Supplementary Fig. S7. Comparison of complete 5S unit sequences in *Oryza* and *Panax* species. a, Length comparison of 5S units. **b**, Sequence comparison of 5S rRNA coding sequence. **c,** Sequence comparison of IGS of *Oryza* species. **d,** Sequence comparison of IGS of *Panax* species (GenBank nos. KM036312, KM036313, and KM036314). Nucleotide

sequences were aligned and visualized using ClustalW (http://www.genome.jp/tools/clustalw/) and GeneDoc software (http://www.nrbsc.org/gfx/genedoc/).

a

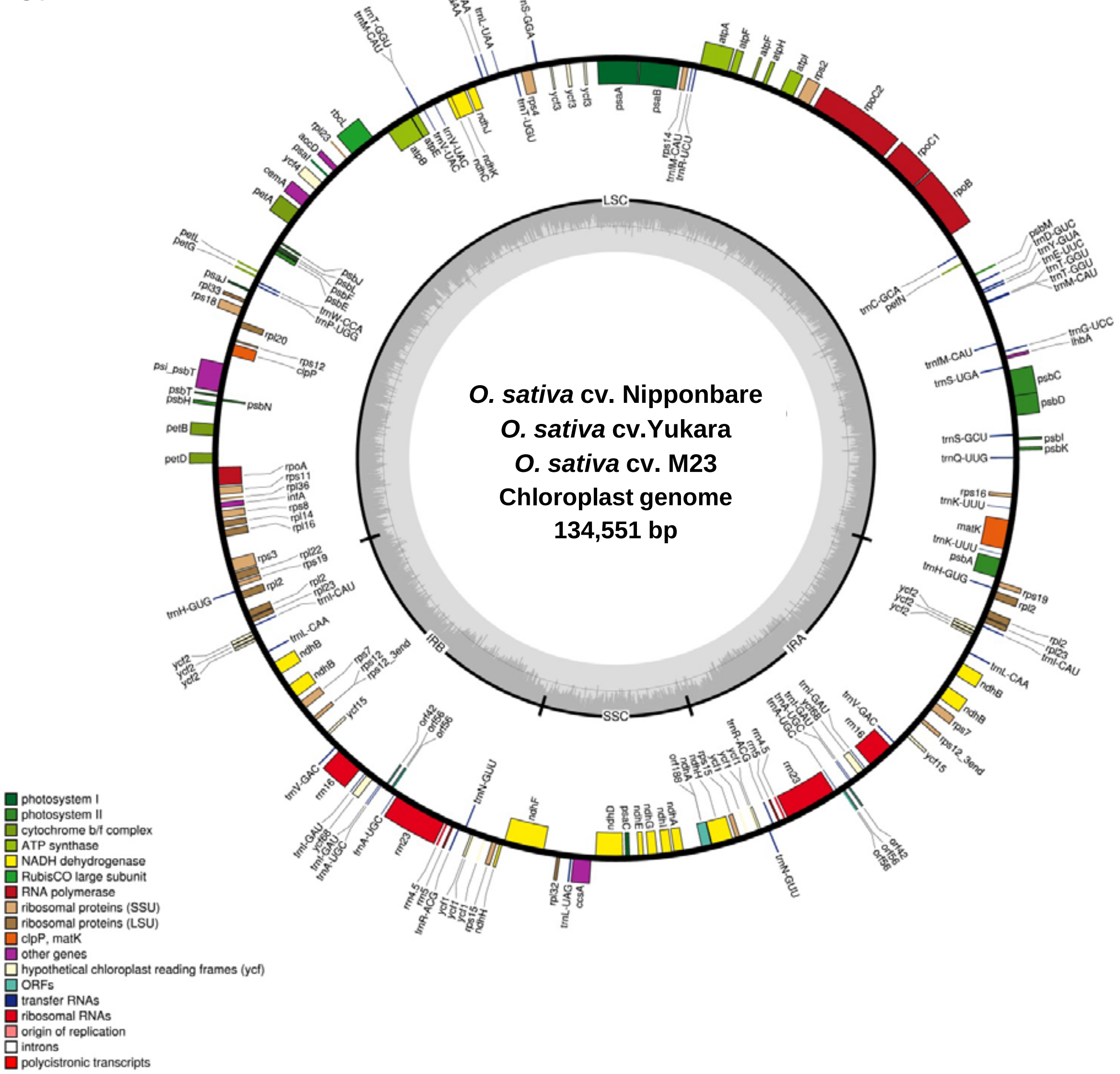

LSC
IRA
IRB
SSC
O. sativa cv. Nipponbare
O. sativa cv.Yukara
O. sativa cv. M23
Chloroplast genome
134,551 bp
photosystem I
photosystem II
cytochrome b/f complex
ATP synthase
NADH dehydrogenase
RubisCO large subunit
RNA polymerase
ribosomal proteins (SSU)
ribosomal proteins (LSU)
clpP, matK
other genes
hypothetical chloroplast reading frames (ycf)
ORFs
transfer RNAs
ribosomal RNAs
origin of replication
introns
polycistronic transcripts

b

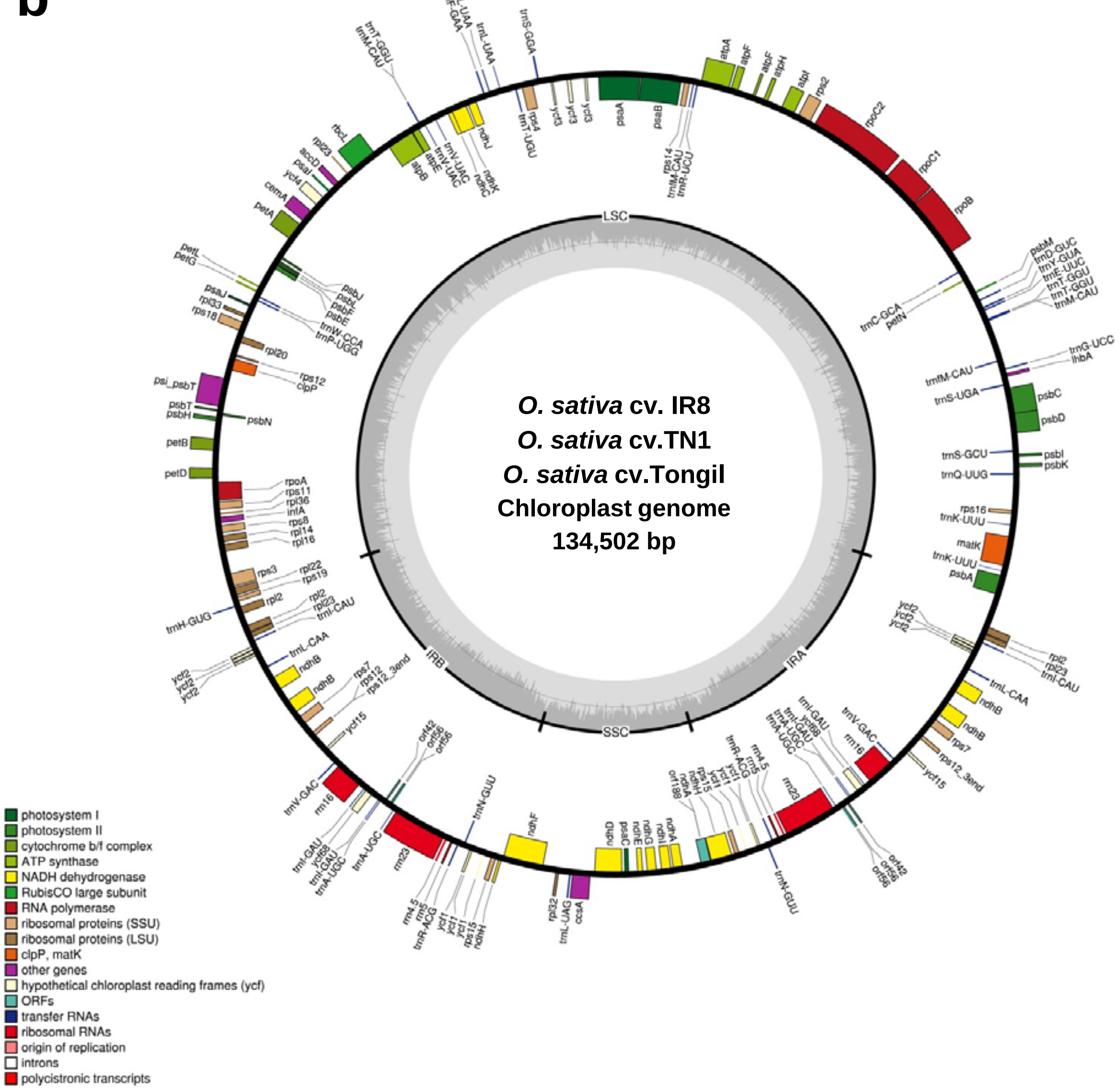

O. sativa cv. IR8
O. sativa cv.TN1
O. sativa cv.Tongil
Chloroplast genome
134,502 bp
LSC
IRA
SSC
IRB
photosystem I
photosystem II
cytochrome b/f complex
ATP synthase
NADH dehydrogenase
RubisCO large subunit
RNA polymerase
ribosomal proteins (SSU)
ribosomal proteins (LSU)
clpP, matK
other genes
hypothetical chloroplast reading frames (ycf)
ORFs
transfer RNAs
ribosomal RNAs
origin of replication
introns
polycistronic transcripts

c

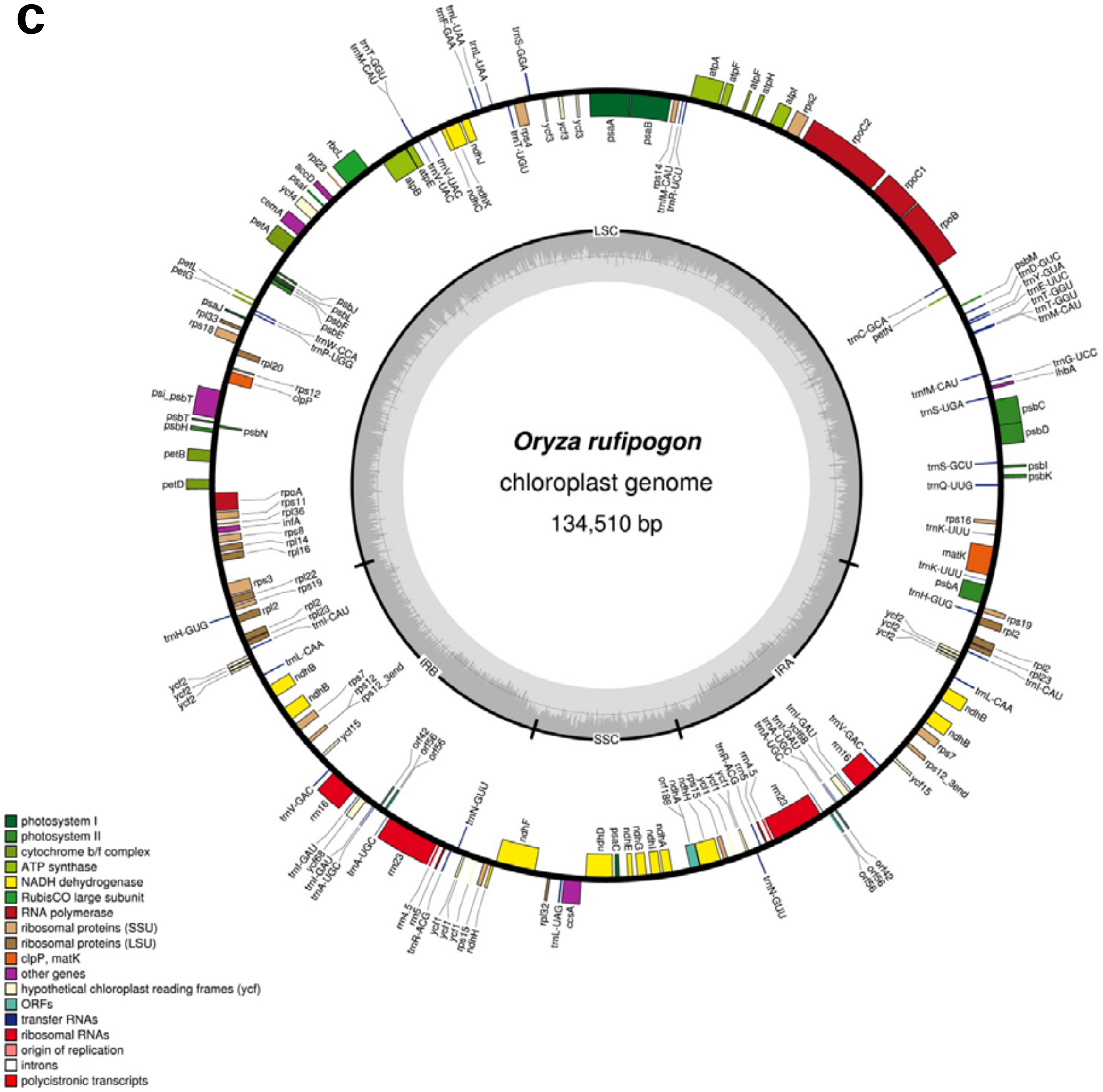

Oryza rufipogon
chloroplast genome
134,510 bp
LSC
IRA
SSC
IRB
photosystem I
photosystem II
cytochrome b/f complex
ATP synthase
NADH dehydrogenase
RubisCO large subunit
RNA polymerase
ribosomal proteins (SSU)
ribosomal proteins (LSU)
clpP, matK
other genes
hypothetical chloroplast reading frames (ycf)
ORFs
transfer RNAs
ribosomal RNAs
origin of replication
introns
polycistronic transcripts

d

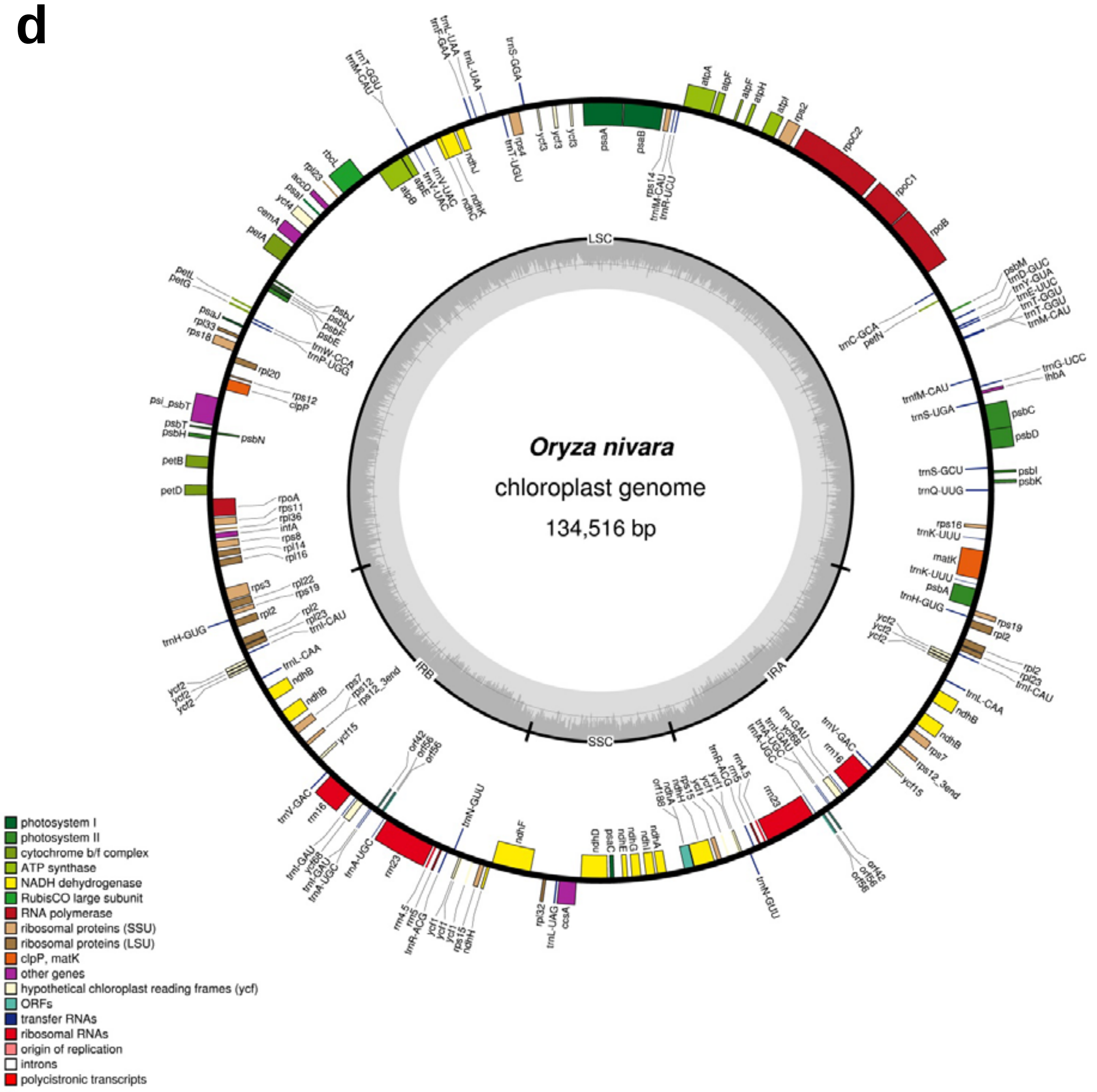
Oryza nivara
chloroplast genome
134,516 bp
LSC
IRA
SSC
IRB
photosystem I
photosystem II
cytochrome b/f complex
ATP synthase
NADH dehydrogenase
RubisCO large subunit
RNA polymerase
ribosomal proteins (SSU)
ribosomal proteins (LSU)
clpP, matK
other genes
hypothetical chloroplast reading frames (ycf)
ORFs
transfer RNAs
ribosomal RNAs
origin of replication
introns
polycistronic transcripts

e

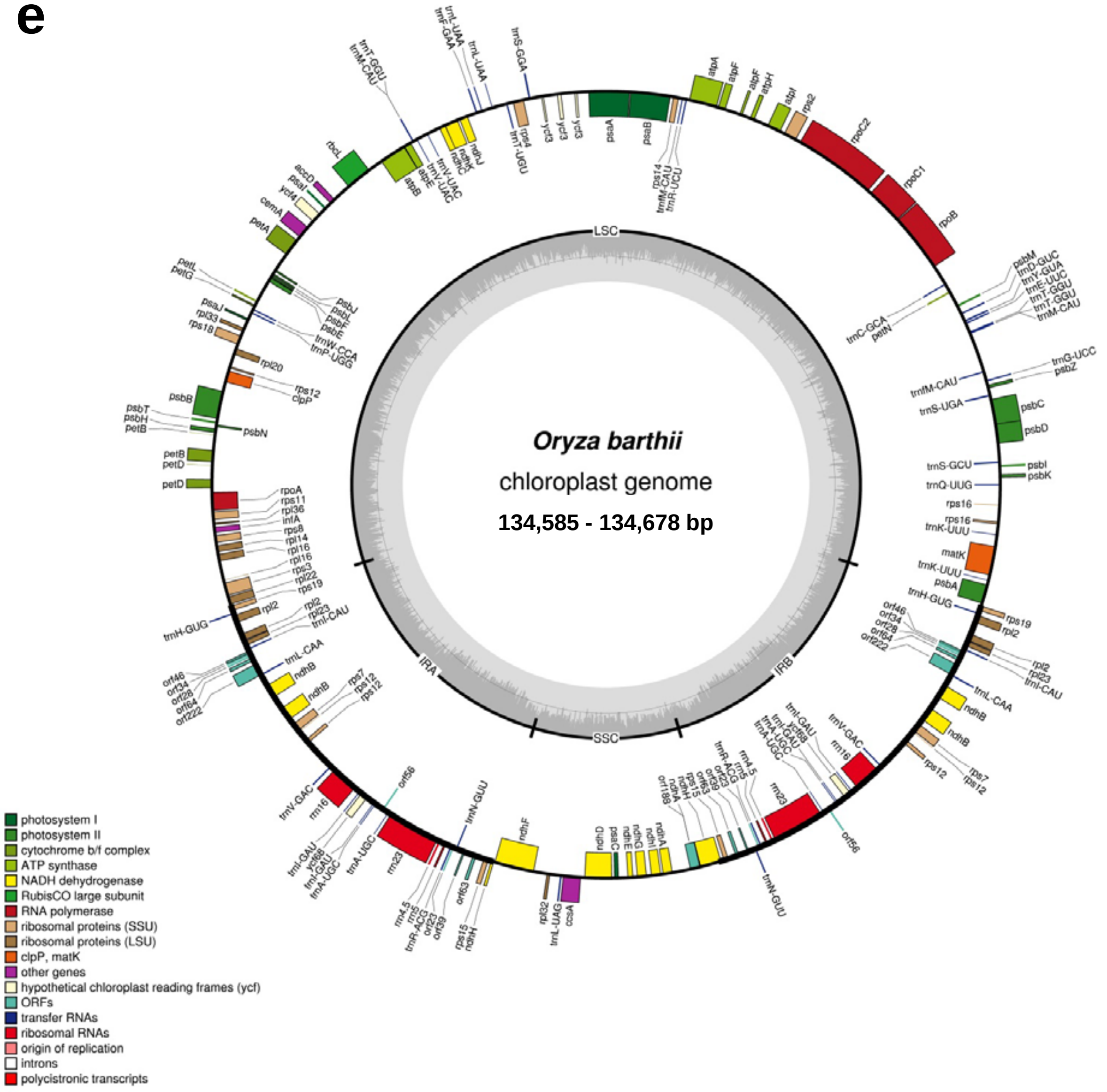

Oryza barthii
chloroplast genome
134,585 - 134,678 bp
LSC
IRA
IRB
SSC
photosystem I
photosystem II
cytochrome b/f complex
ATP synthase
NADH dehydrogenase
RubisCO large subunit
RNA polymerase
ribosomal proteins (SSU)
ribosomal proteins (LSU)
clpP, matK
other genes
hypothetical chloroplast reading frames (ycf)
ORFs
transfer RNAs
ribosomal RNAs
origin of replication
introns
polycistronic transcripts

f

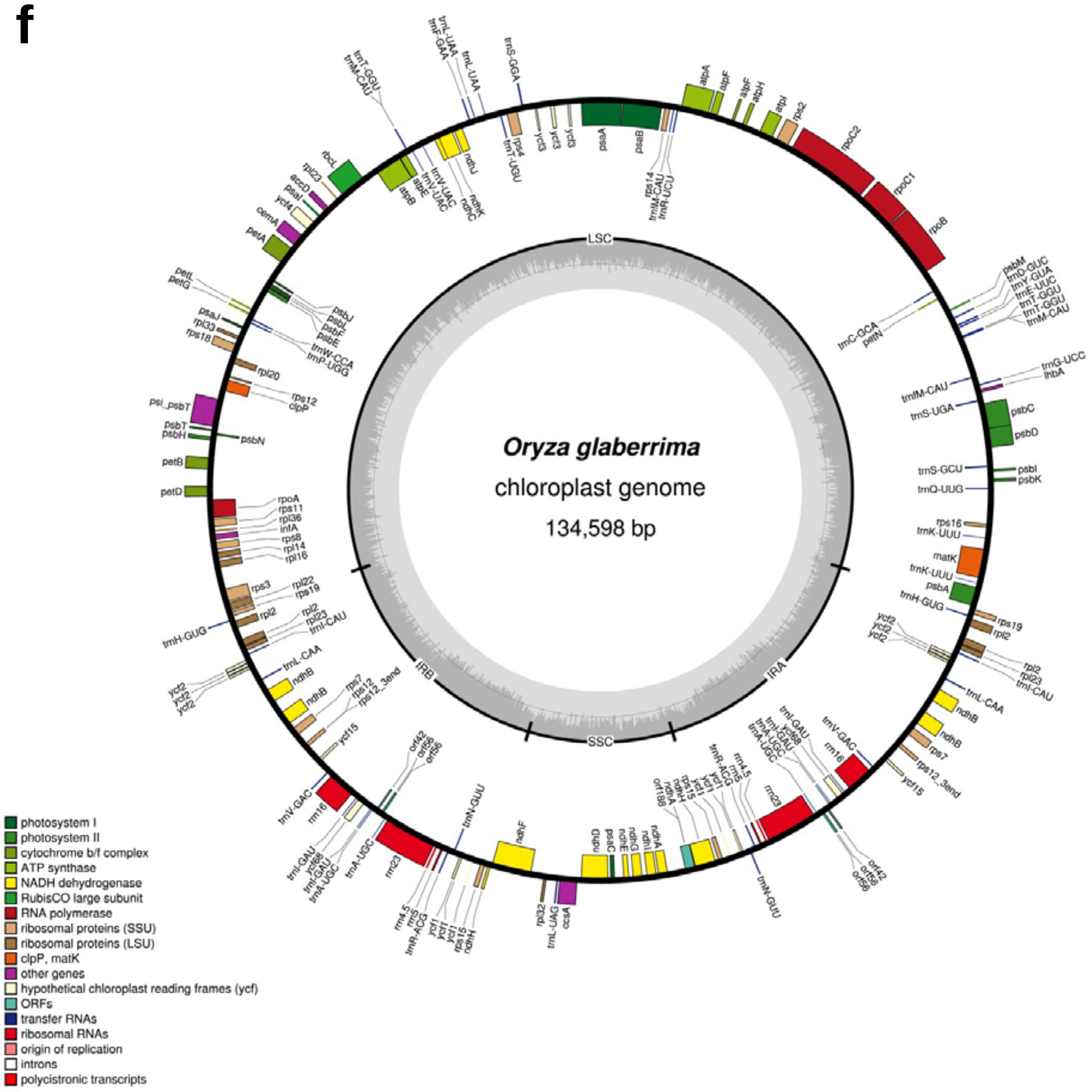

Oryza glaberrima
chloroplast genome
134,598 bp
LSC
SSC
IRA
IRB
photosystem I
photosystem II
cytochrome b/f complex
ATP synthase
NADH dehydrogenase
RubisCO large subunit
RNA polymerase
ribosomal proteins (SSU)
ribosomal proteins (LSU)
clpP, matK
other genes
hypothetical chloroplast reading frames (ycf)
ORFs
transfer RNAs
ribosomal RNAs
origin of replication
introns
polycistronic transcripts

g

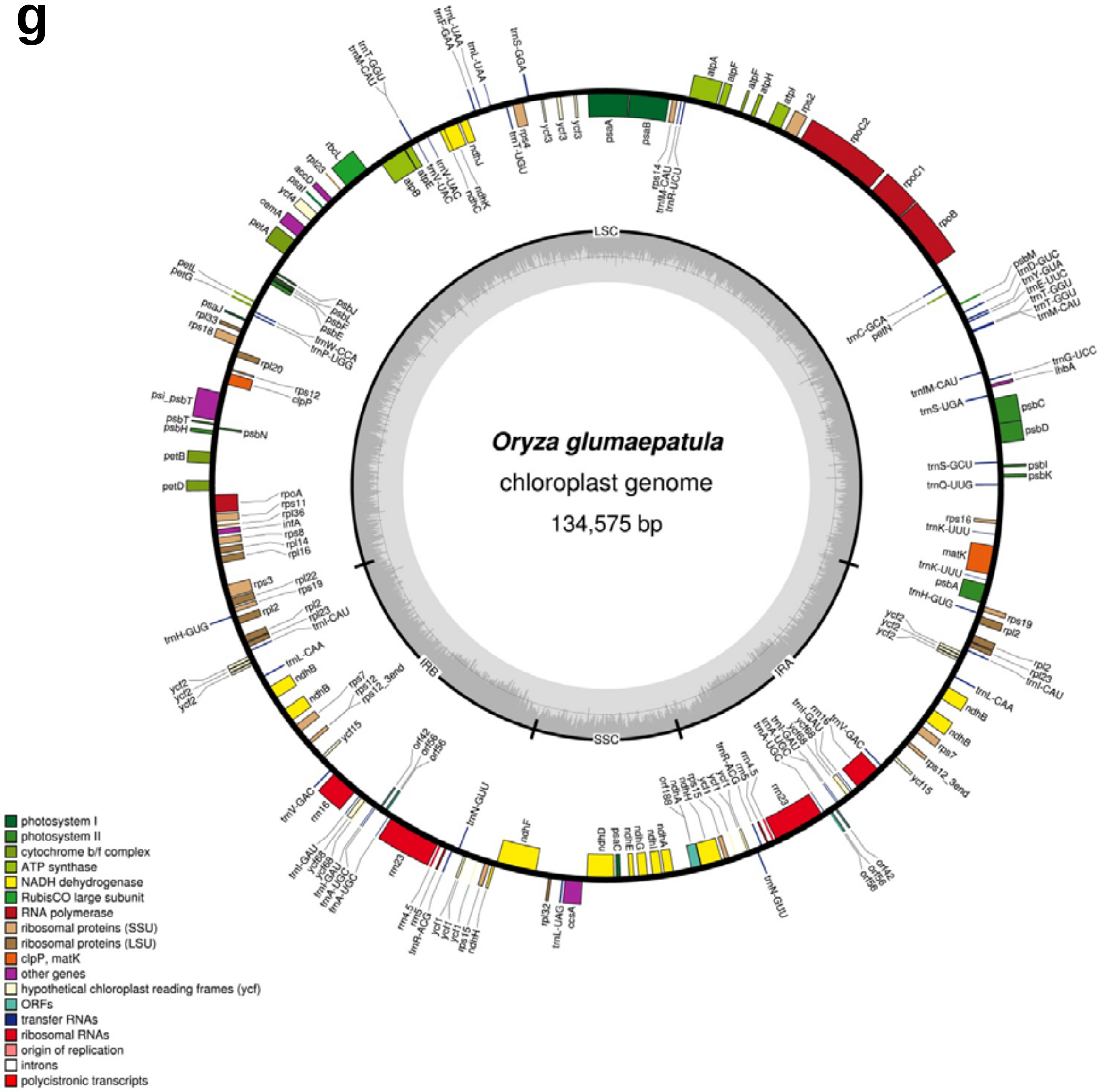

Oryza glumaepatula
chloroplast genome
134,575 bp
LSC
SSC
IRA
IRB
photosystem I
photosystem II
cytochrome b/f complex
ATP synthase
NADH dehydrogenase
RubisCO large subunit
RNA polymerase
ribosomal proteins (SSU)
ribosomal proteins (LSU)
clpP, matK
other genes
hypothetical chloroplast reading frames (ycf)
ORFs
transfer RNAs
ribosomal RNAs
origin of replication
introns
polycistronic transcripts

h

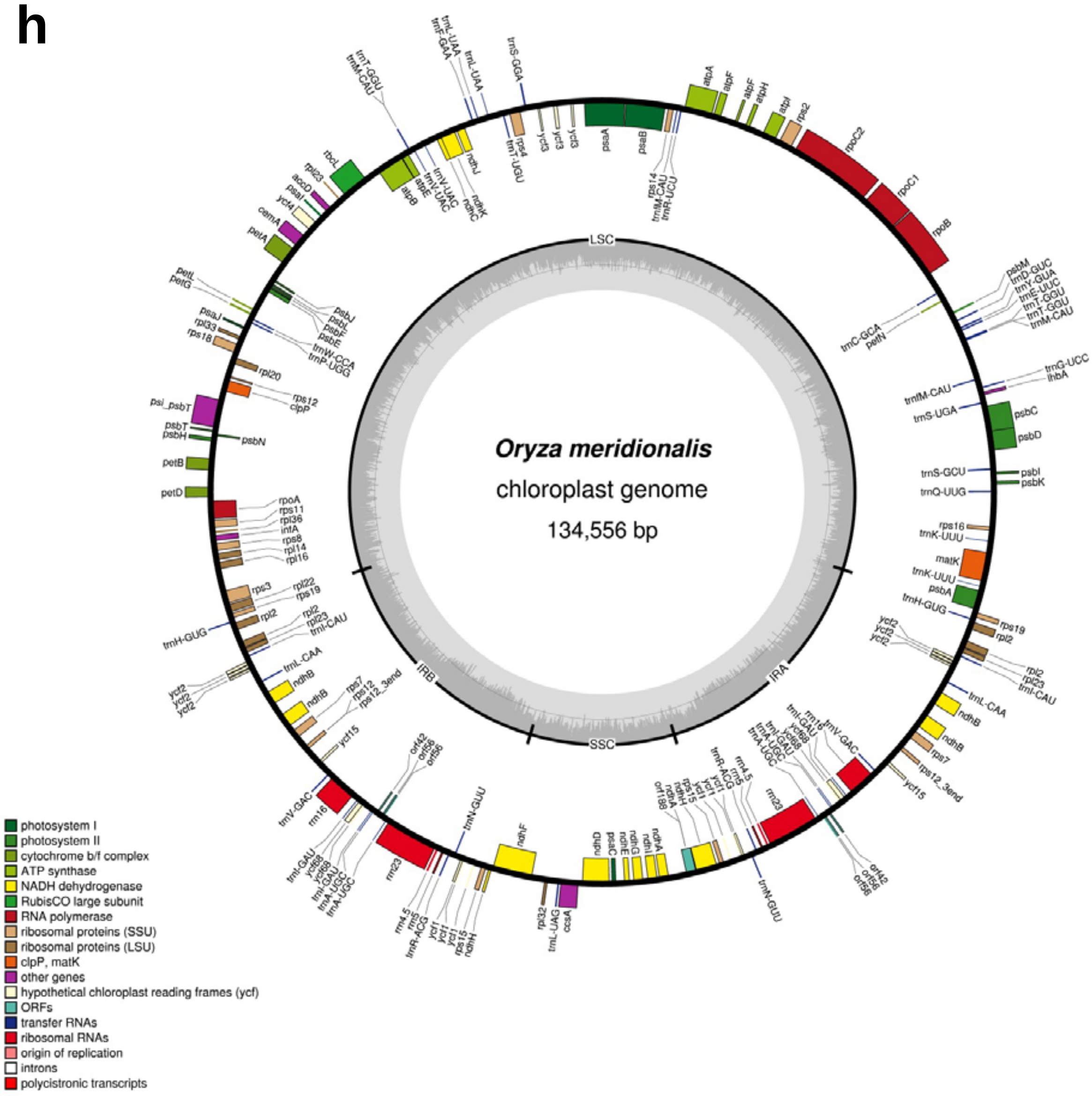
Oryza meridionalis
chloroplast genome
134,556 bp
LSC
SSC
IRA
IRB
photosystem I
photosystem II
cytochrome b/f complex
ATP synthase
NADH dehydrogenase
RubisCO large subunit
RNA polymerase
ribosomal proteins (SSU)
ribosomal proteins (LSU)
clpP, matK
other genes
hypothetical chloroplast reading frames (ycf)
ORFs
transfer RNAs
ribosomal RNAs
origin of replication
introns
polycistronic transcripts

i

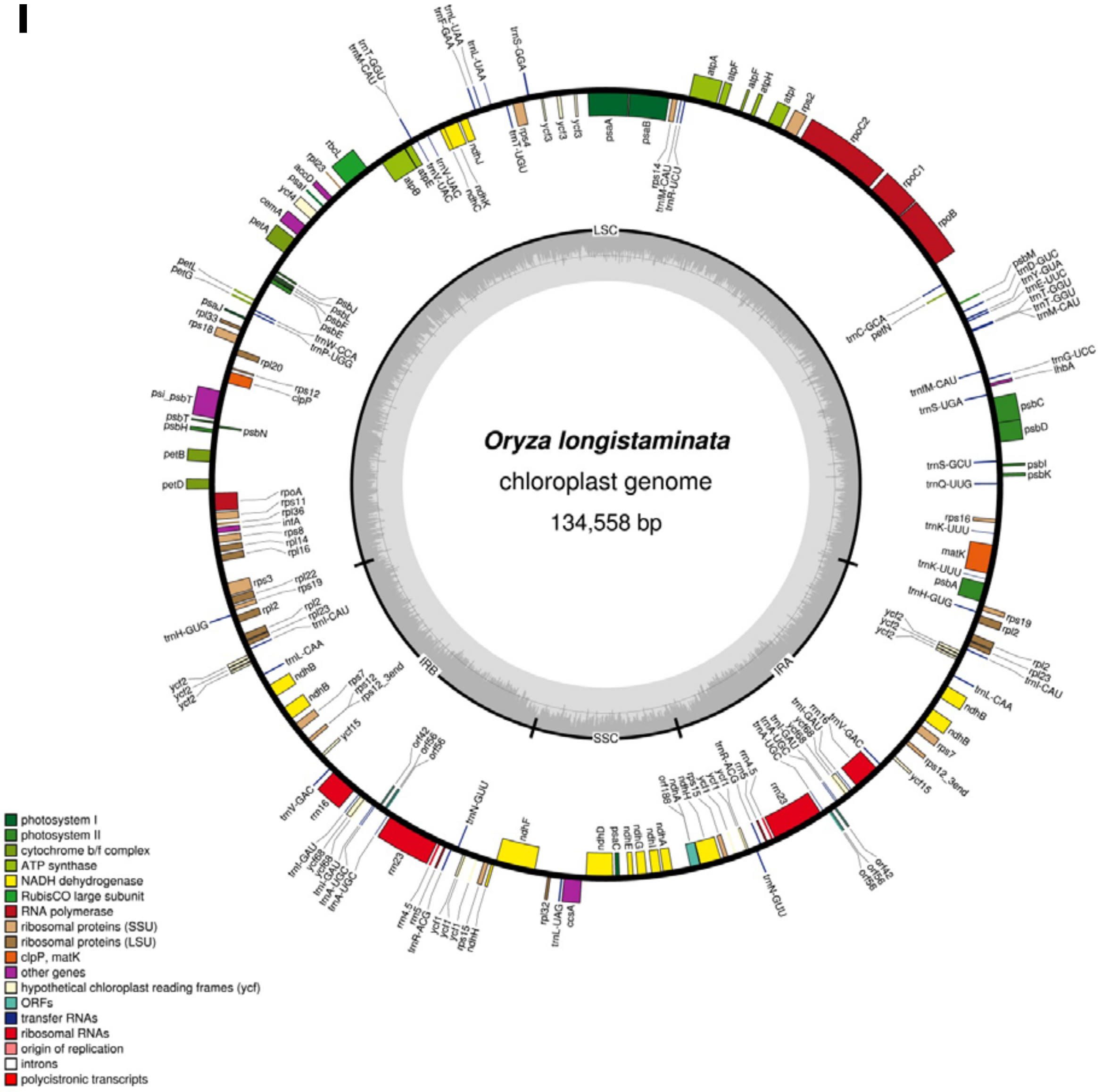

Oryza longistaminata
chloroplast genome
134,558 bp
LSC
IRA
SSC
IRB
photosystem I
photosystem II
cytochrome b/f complex
ATP synthase
NADH dehydrogenase
RubisCO large subunit
RNA polymerase
ribosomal proteins (SSU)
ribosomal proteins (LSU)
clpP, matK
other genes
hypothetical chloroplast reading frames (ycf)
ORFs
transfer RNAs
ribosomal RNAs
origin of replication
introns
polycistronic transcripts

j

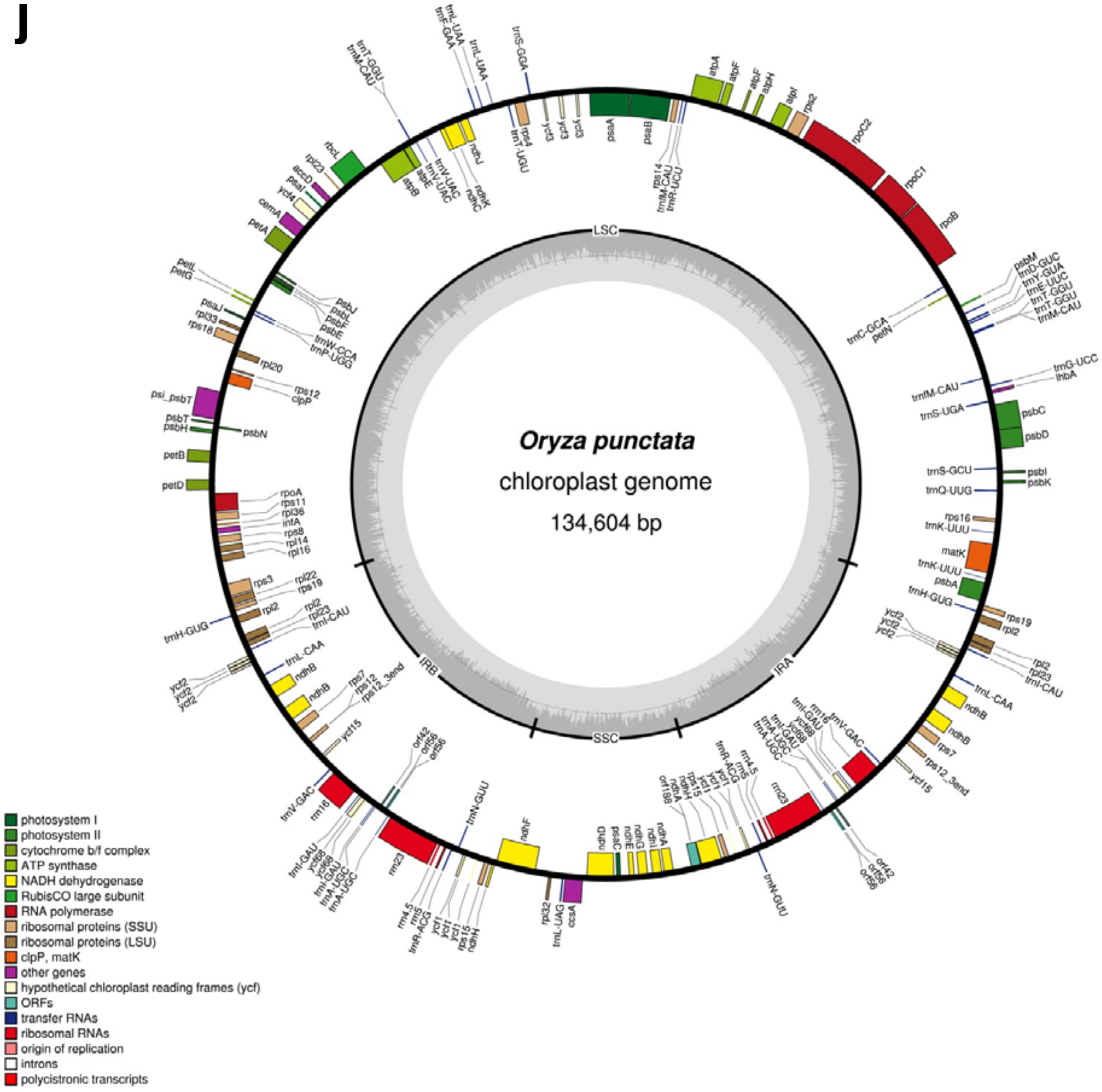

Supplementary Fig. S8. Chloroplast genome map of nine *Oryza* species. The complete cp genome sequence was generated by the dnaLCW method and annotated using the DOGMA program (http://dogma.ccbb.utexas.edu/). The map was prepared using OGDRAW (http://ogdraw.mpimp-golm.mpg.de/). Genes transcribed clockwise and counterclockwise are indicated on the outside and inside of the large circle, respectively. The four parts of the cp genome and GC content are indicated on the inner circle. **a**, *O. sativa* cv. Nipponbare, cv. Yukara, and cv. M23; **b**, *O. sativa* cv. IR28, cv. TN1, and cv. Tongil; **c**, *O. rufipogon* accessions; **d**, *O. nivara* accessions; **e**, *O. barthii* accessions; **f**, *O. glaberrima* accessions; **g**, *O.*

glumaepatula accessions; **h**, *O. meridionalis* accessions; **i**, *O. longistaminata* (IRGC110404); **j**, *O. punctata* (IRGC105690).

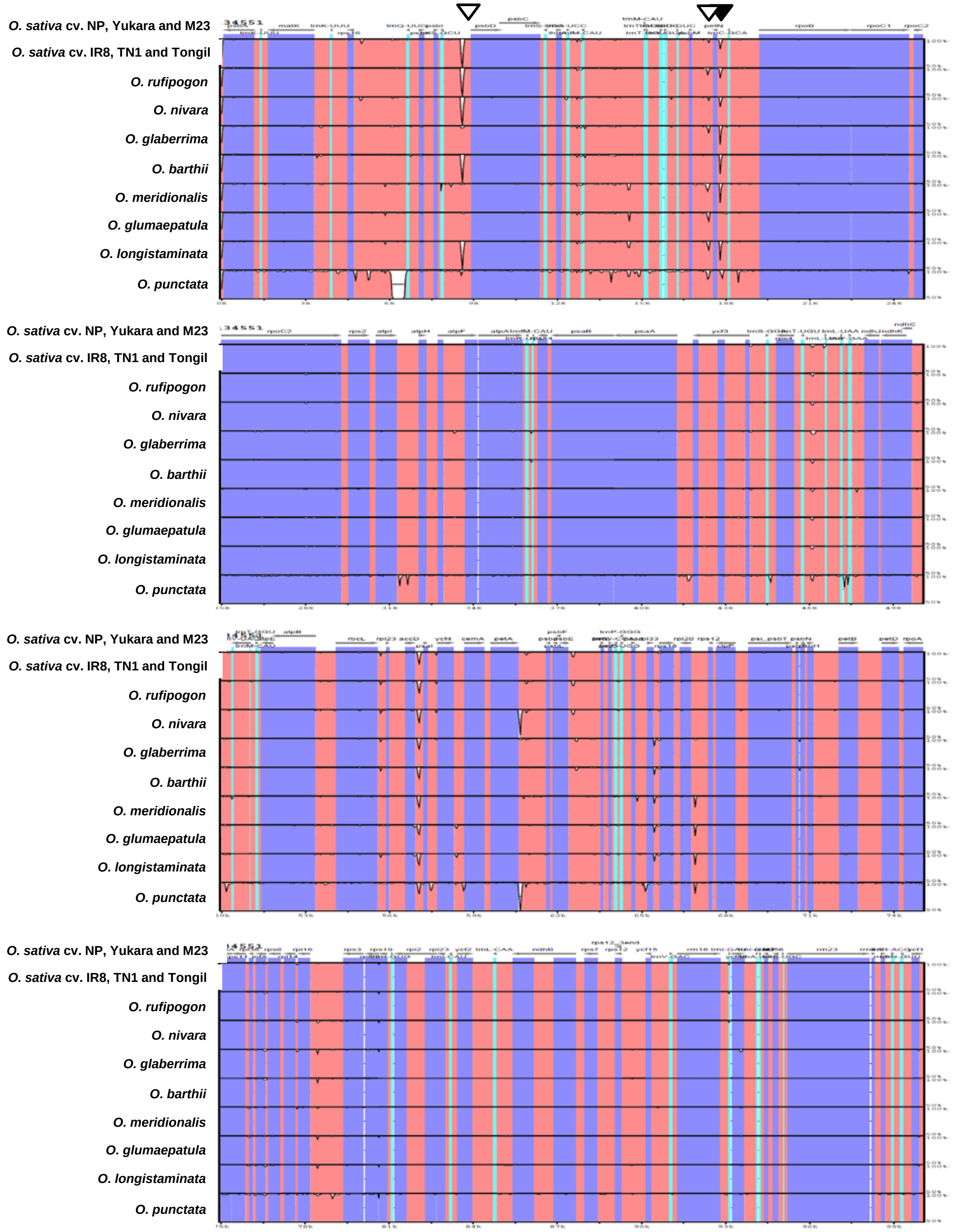
O. sativa cv. NP, Yukara and M23
O. sativa cv. IR8, TN1 and Tongil
O. rufipogon
O. nivara
O. glaberrima
O. barthii
O. meridionalis
O. glumaepatula
O. longistaminata
O. punctata
O. sativa cv. NP, Yukara and M23
O. sativa cv. IR8, TN1 and Tongil
O. rufipogon
O. nivara
O. glaberrima
O. barthii
O. meridionalis
O. glumaepatula
O. longistaminata
O. punctata
O. sativa cv. NP, Yukara and M23
O. sativa cv. IR8, TN1 and Tongil
O. rufipogon
O. nivara
O. glaberrima
O. barthii
O. meridionalis
O. glumaepatula
O. longistaminata
O. punctata
O. sativa cv. NP, Yukara and M23
O. sativa cv. IR8, TN1 and Tongil
O. rufipogon
O. nivara
O. glaberrima
O. barthii
O. meridionalis
O. glumaepatula
O. longistaminata
O. punctata

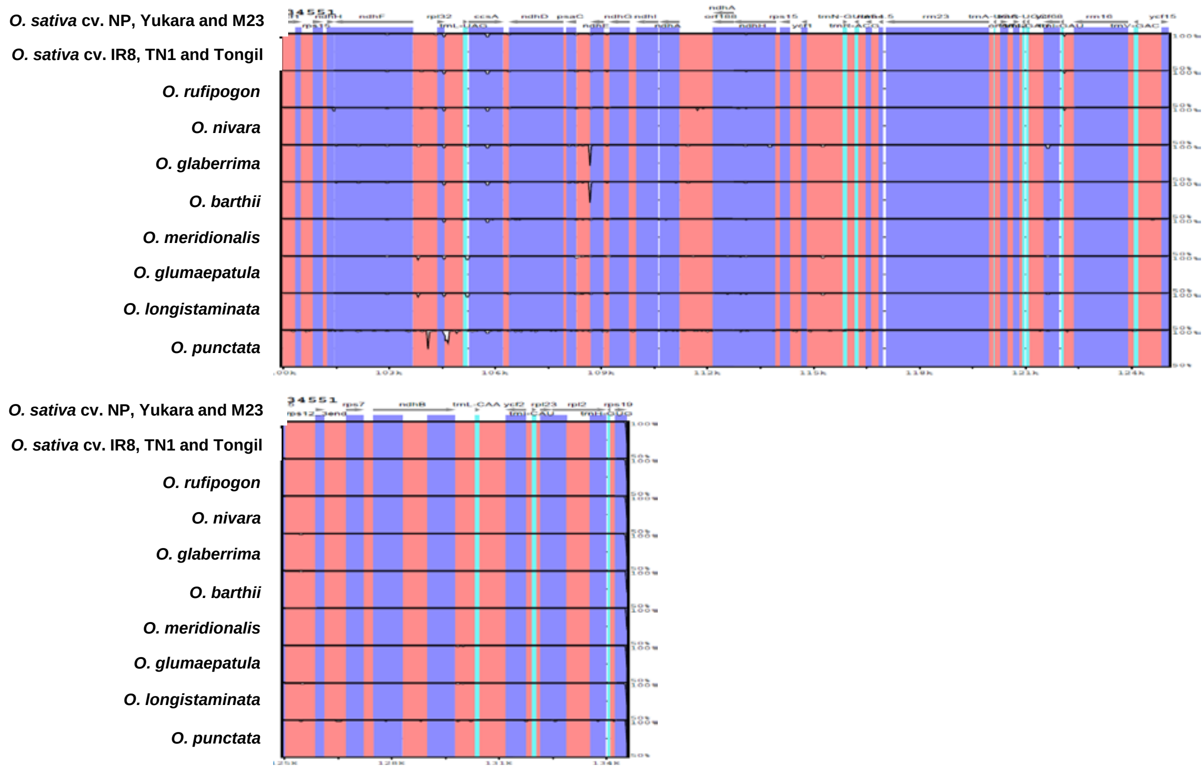

Supplementary Fig. S9. Comparison of cp genome sequences of *Oryza* species. Complete cp genomes of *Oryza* species were generated by the dnaLCW method. Genic regions were identified using the DOGMA program (http://dogma.ccbb.utexas.edu/) and the comparative map was prepared using mVISTA (http://genome.lbl.gov/vista/mvista/submit.shtml). Empty and filled arrowheads indicate deletion and insertion of nucleotides, respectively, which were analyzed in Supplementary Fig. S10.

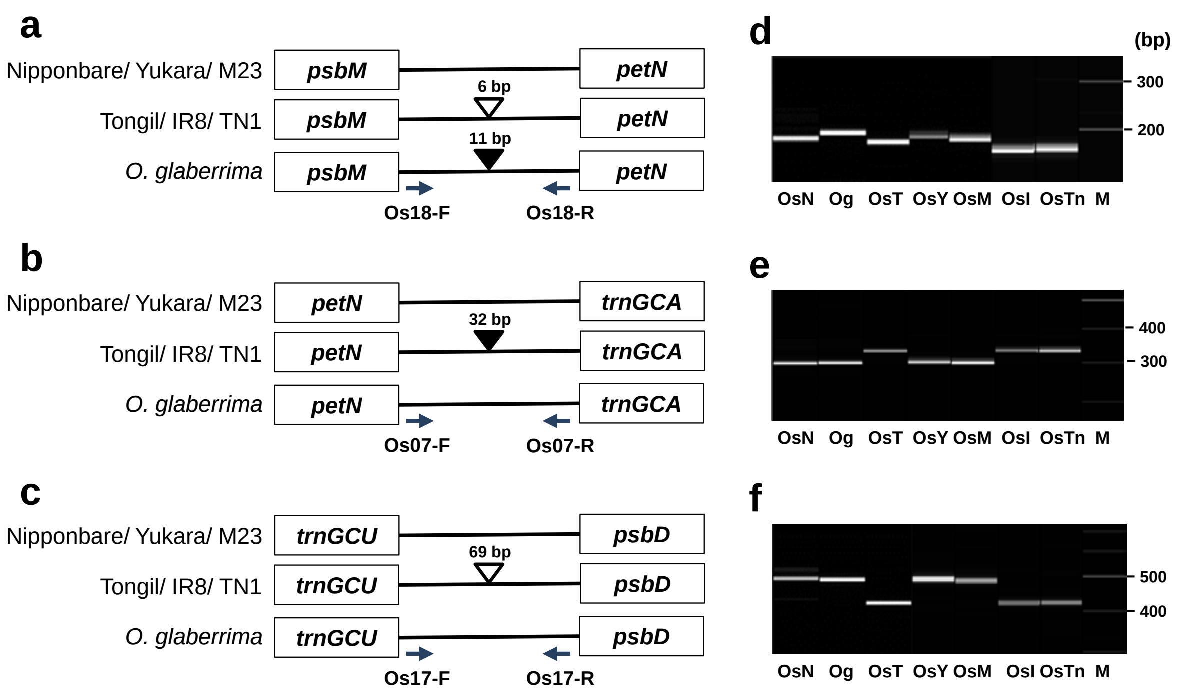

Supplementary Fig. S10. Schematic representation and validation of polymorphism for three InDel regions between *Oryza* subspecies. Complete cp genomes of seven *Oryza* species were generated and compared to identify polymorphic regions. Three InDel regions found in *psbM-petN* (**a** and **d**), *petN-trnGCA* (**b** and **e**), and *trnGCU-psbD* (**c** and **f**) were confirmed by PCR analysis. Empty and filled arrowheads indicate deletion and insertion of nucleotides, respectively, when compared with the cp genome sequence of cv. Nipponbare. Arrows indicate primer positions (Supplementary Table S6). OsN, *O. sativa* cv. Nipponbare; Og, *O. glaberrima*; OsT, cv. Tongil; OsY, cv. Yukara; OsM, cv. M23; OsI, cv. IR8; OsTn, cv. TN1; M, DNA size marker.

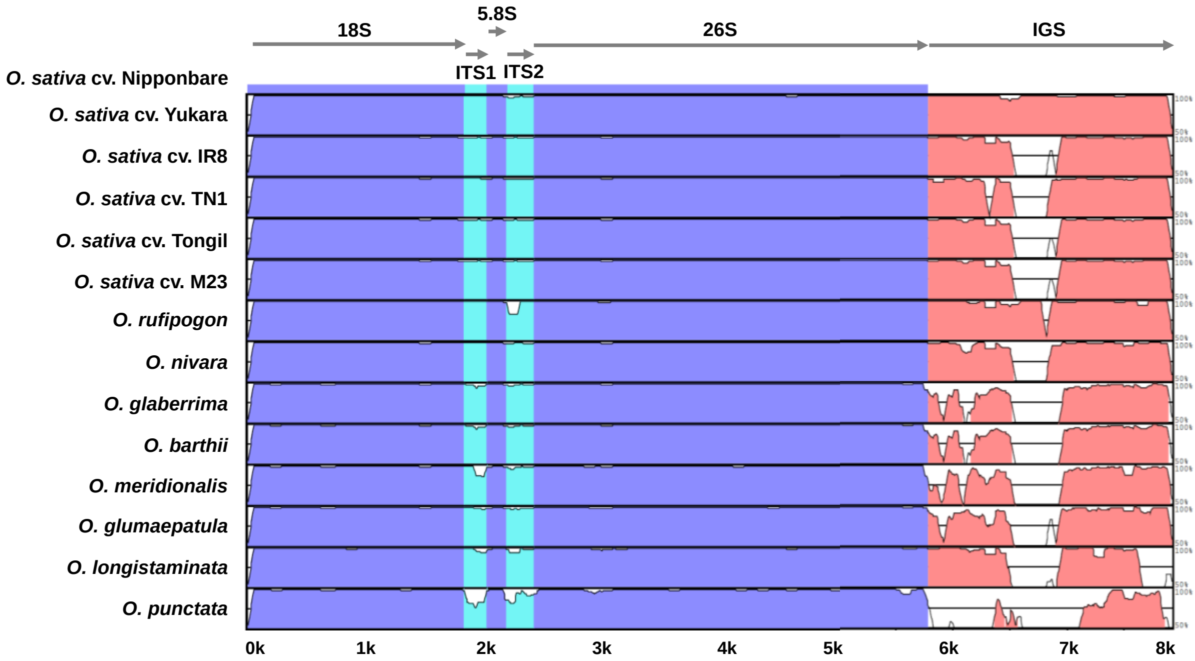

Supplementary Fig. S11. Comparison of 45S units of *Oryza* species. Complete 45S units of *Oryza* species were generated by method developed in this study and the comparative map was constructed using mVISTA (http://genome.lbl.gov/vista/mvista/submit.shtml).

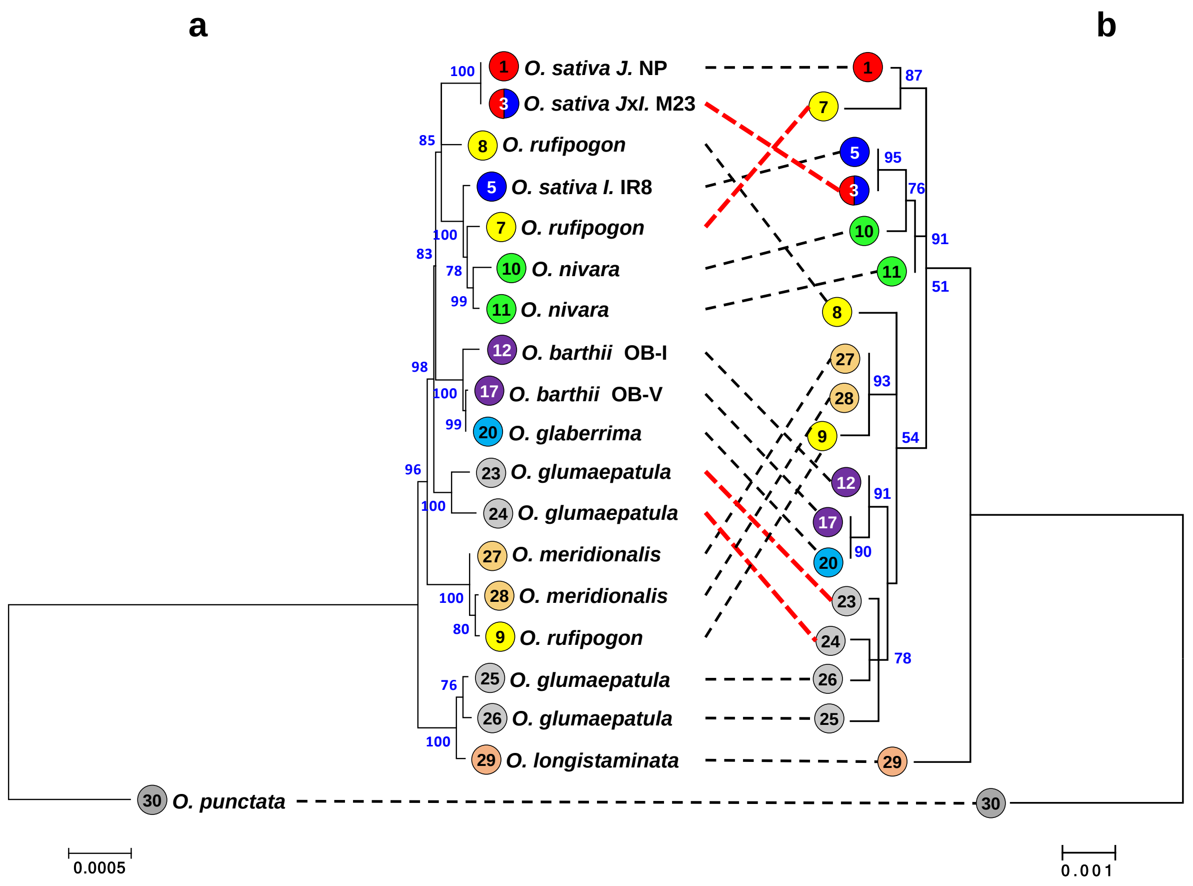

Supplementary Fig. S12. Phylogenomic tree of *Oryza* species using the maximum likelihood (ML) method. (**a**,**b**) Phylogenetic trees were built based on the complete cp genome (**a**) and 45S cistron sequences (**b**). *O. sativa* ssp. *japonica* and *indica* groups are denoted as *J* and *I*, respectively. Different species/subspecies are indicated with different colored labels. Numbers in colored circles represent accessions labeled in Table 1. Dashed lines connect the positions of each accession/cultivar in the two trees; red highlights major differences between trees. The tree was generated by the maximum likelihood (ML) method with 1,000 bootstrap replicates using MEGA6.

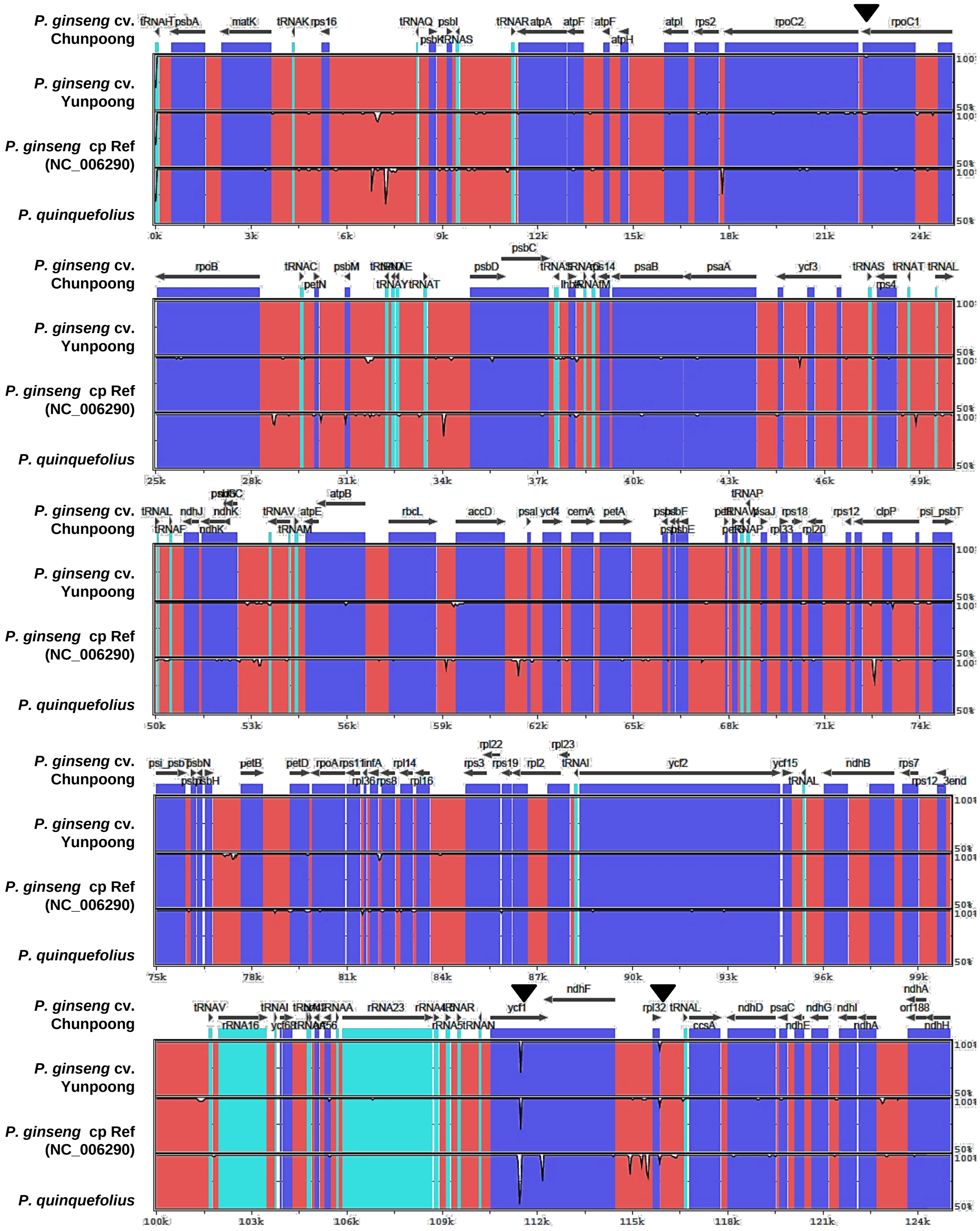
P. ginseng cv. Chunpoong
P. ginseng cv. Yunpoong
P. ginseng cp Ref (NC_006290)
P. quinquefolius

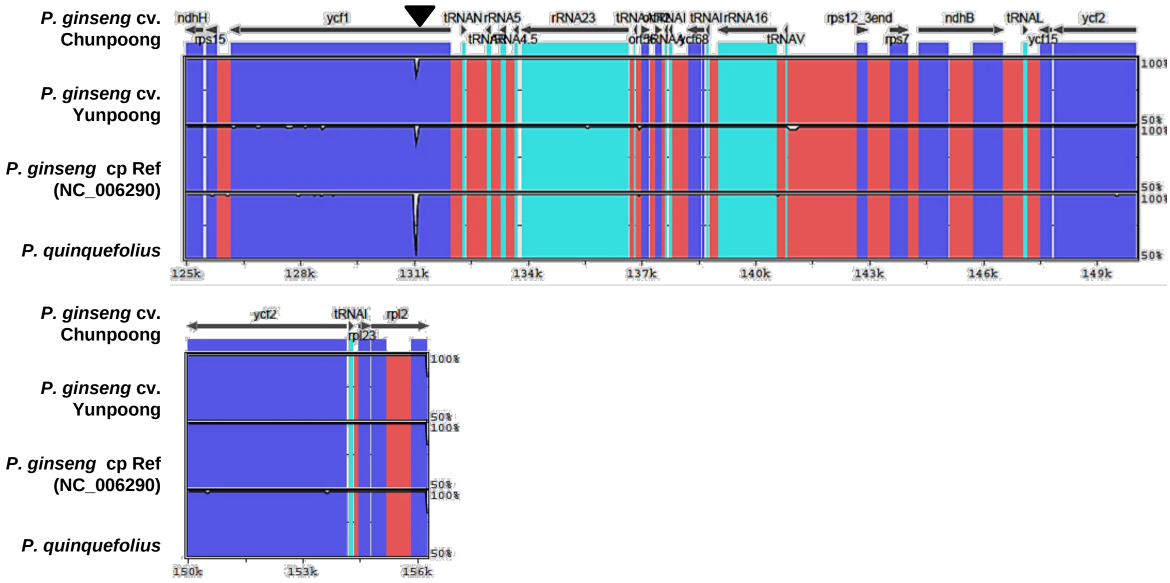

Supplementary Fig. S13. Comparison of cp genome sequences of *Panax* species. Complete cp genomes of *P. ginseng* cv. Chunpoong (GenBank Accession No. KM088019)[55], cv. Yunpoong (KM088020)[55], and *P. quinquefolius* (KM088018) were generated by the dnaLCW method and the cp genome sequence in GenBank (*P. ginseng* cv. Nees; NC_006290) was used for comparison. Genic regions were identified using the DOGMA program (http://dogma.ccbb.utexas.edu/) and the comparative map was prepared using mVISTA (http://genome.lbl.gov/vista/mvista/submit.shtml). Filled arrowheads indicate polymorphic regions analyzed in Supplementary Fig. S14.

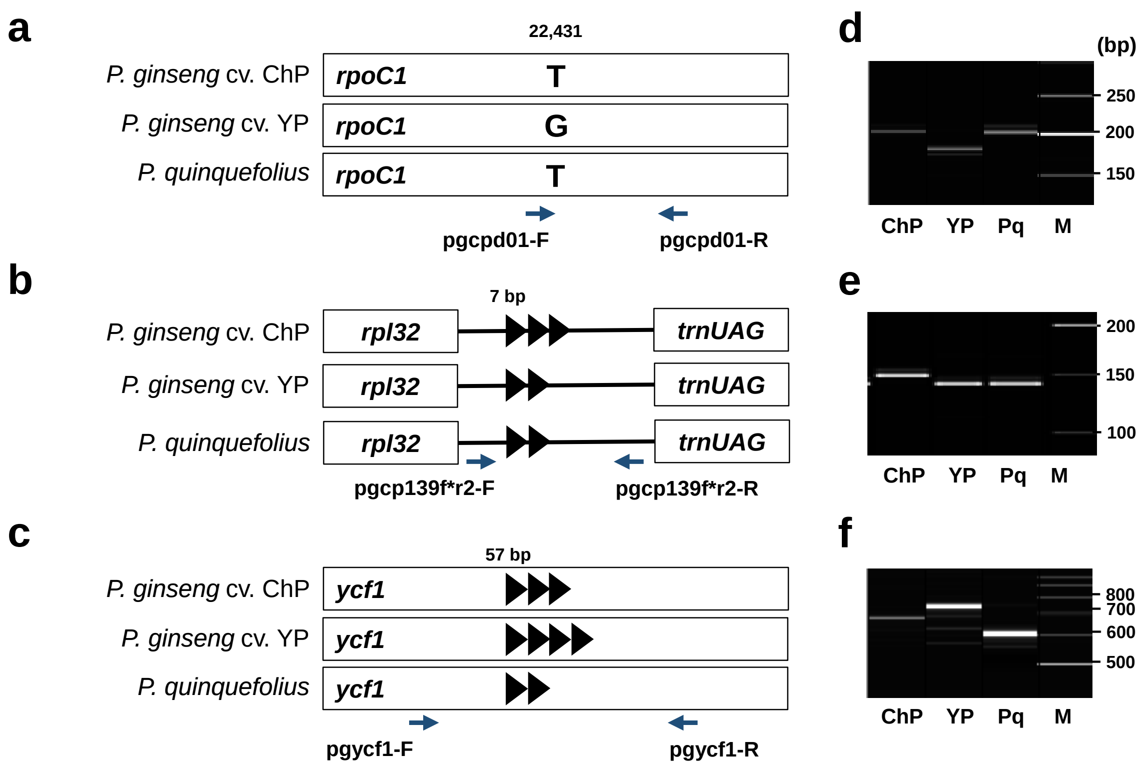

Supplementary Fig. S14. Schematic representation and validation of three polymorphic regions between cp genome sequences of two *P. ginseng* cultivars. Complete cp genomes of *P. ginseng* cv. Chunpoong (ChP), cv. Yunpoong (YP), and *P. quinquefolius* (Pq) were compared to identify SNP (**a**) and copy number variation (CNV) sites (**b** and **c**). **a** and **d,** SNP analysis using dCAPS primers designed for the SNP site of the *rpoC1* gene. *Xba*I digestion of the amplicon produced a digested fragment only in YP but not in ChP and Pq. **b** and **e**, CNV analysis of a 7-bp tandem repeat in the *rpl32-trnUAG* region. PCR amplification confirmed 2 copies of the repeat in YP and Pq, and 3 copies in ChP. **c** and **f**, CNV analysis of a 57-bp tandem repeat in the *ycf1* gene. PCR amplification confirmed 2, 3, and 4 copies of the repeat in Pq, ChP, and YP, respectively. Arrows indicate primer sites previously reported[56]. M indicates DNA size marker.

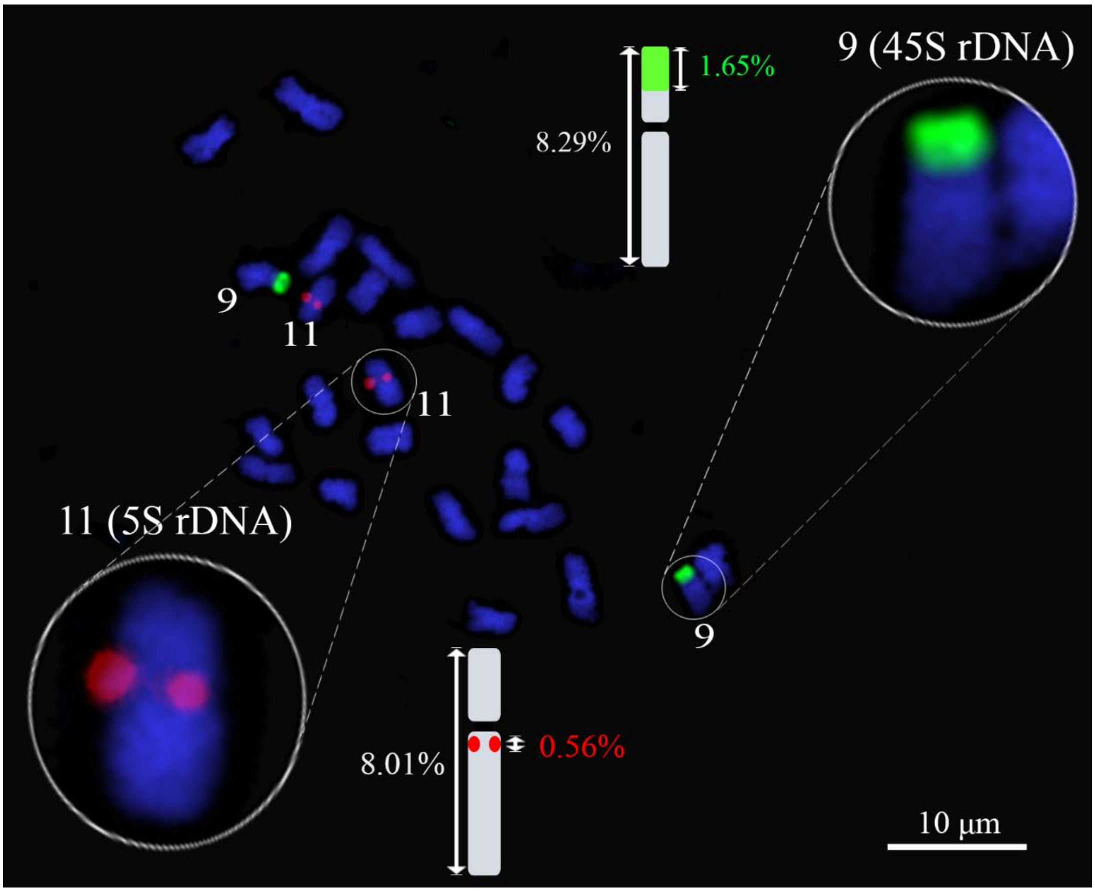

Supplementary Fig. S15. FISH analysis of 45S and 5S in *O. sativa* genome. nR content genome was estimated based on the ratio of the area covered by probe hybridization signals to the total chromosome area (Supplementary Tables S4). Values outside parentheses represent the areas of nR-bearing chromosomes or FISH signals, while values inside parentheses represent estimated genomic contents.

Supplementary Tables

Supplementary Table S1. Characterization of the 30 longest contigs in the *de novo* assembly of the rice Os2 dataset (1x genome coverage; 50x cp genome coverage).

Ctg_no.	Ctg length (bp)	Ctg Coverage (x)	Best hit in GenBank and TE DB					Description
			Acc. no.	Length (bp)	Match begin	Match end	E-value	
ctg_39	53,713	48.89	GU592207.1	134,551	18	53,535	0.0	*O. sativa japonica* chloroplast genome
ctg_56	20,802	97.14	GU592207.1	134,551	80,605	101,406	0.0	*O. sativa japonica* chloroplast genome
ctg_911	19,415	10.76	BA000029.3	490,520	173,036	183,181	0.0	*O. sativa japonica* mitochondrial genome
ctg_40	18,492	48.63	GU592207.1	134,551	53,516	72,007	0.0	*O. sativa japonica* chloroplast genome
ctg_619	16,859	12.57	BA000029.3	490,520	214,506	228,529	0.0	*O. sativa japonica* mitochondrial genome
ctg_596	16,024	10.00	BA000029.3	490,520	343,504	359,495	0.0	*O. sativa japonica* mitochondrial genome
ctg_562	12,383	47.71	GU592207.1	134,551	101,387	113,769	0.0	*O. sativa japonica* chloroplast genome
ctg_380	11,412	9.89	BA000029.3	490,520	36,168	47,528	0.0	*O. sativa japonica* mitochondrial genome
ctg_135	8,628	53.43	GU592207.1	134,551	71,988	80,624	0.0	*O. sativa japonica* chloroplast genome
ctg_72	8,112	154.94	MUDR1_OS	8,052	966	7,715	0.0	*O. sativa* MuDR-type DNA transposon
ctg_948	7,982	8.40	BA000029.3	490,520	80,797	88,762	0.0	*O. sativa japonica* mitochondrial genome
ctg_1284	7,882	8.33	BA000029.3	490,520	382,357	390,429	0.0	*O. sativa japonica* mitochondrial genome
ctg_567	7,173	29.88	MDR2	6,967	76	6,516	0.0	*O. sativa* MuDR-type DNA transposon
ctg_183	7,166	12.39	SZ-55_I	7,062	1	6,973	0.0	*O. sativa* retrotransposon SZ-55
ctg_173	6,889	342.62	M11585.1	3,377	2	3,377	0.0	*O. sativa* 25S ribosomal RNA gene
ctg_180	6,776	24.93	RIRE7_I	5,899	1,394	5,899	0.0	RIRE7 gypsy-like endogenous retrovirus
ctg_316	6,622	95.83	MuDR3_OS	8,604	97	6,666	0.0	*O. sativa* MuDR-type DNA transposon
ctg_580	6,607	8.34	BA000029.3	490,520	73,247	79,345	0.0	*O. sativa japonica* mitochondrial genome
ctg_319	6,403	108.69	RIRE1_I	5,276	800	5,275	0.0	RIRE1, a copia-like retrotransposon
ctg_343	6,331	69.87	SZ-37_I	8,831	1	5,674	0.0	*O. sativa* retrotransposon SZ-37
ctg_265	6,210	44.78	CRM-I_OS	5,933	689	5,933	0.0	*O. sativa* centromeric LTR retrotransposon
ctg_3958	6,059	7.67	BA000029.3	490,520	207,511	213,320	0.0	*O. sativa japonica* mitochondrial genome
ctg_570	5,760	9.18	BA000029.3	490,520	163,967	169,688	0.0	*O. sativa japonica* mitochondrial genome
ctg_1105	5,713	7.93	BA000029.3	490,520	256,888	262,540	0.0	*O. sativa japonica* mitochondrial genome
ctg_2119	5,635	7.67	BA000029.3	490,520	397,094	400,264	0.0	*O. sativa japonica* mitochondrial genome
ctg_675	5,512	40.40	MDR1	7,383	867	6,361	0.0	*O. sativa* MuDR-type DNA transposon
ctg_4531	5,341	8.03	BA000029.3	490,520	299,598	304,451	0.0	*O. sativa japonica* mitochondrial genome
ctg_1460	5,281	9.83	BA000029.3	490,520	53,451	58,496	0.0	*O. sativa japonica* mitochondrial genome
ctg_2770	5,280	10.04	BA000029.3	490,520	66,550	70,862	0.0	*O. sativa japonica* mitochondrial genome
ctg_1612	5,236	9.26	BA000029.3	490,520	65,707	70,862	0.0	*O. sativa japonica* mitochondrial genome

The best hit sequences were found by BlastN searches using contig sequences as queries. Contigs similar to sequences from chloroplasts, mitochondria, and ribosome are indicated by green, orange, and blue, respectively.

Supplementary Table S2. Characterization of the 30 longest contigs among the *de novo* assembly of the ginseng Pg2 dataset (0.05x genome coverage; 50x cp genome coverage).

Ctg_no.	Ctg length (bp)	Ctg Coverage (x)	Best hit in GenBank					Description
			Acc. no.	Length (bp)	Match begin	Match end	E-value	
ctg_3	86,351	49.12	AY582139.1	156,318	1	86,125	0.0	*P ginseng* chloroplast genome
ctg_8	26,153	103.15	AY582139.1	156,318	86,107	112,177	0.0	*P ginseng* chloroplast genome
ctg_15	18,122	45.15	AY582139.1	156,318	112,159	130,266	0.0	*P ginseng* chloroplast genome
ctg_30	9,423	150.85	GQ178077.1	3,362	1	3,362	0.0	*P ginseng* cv Yunpoong 26S ribosomal RNA gene
ctg_671	5,196	8.55	HQ874649.1	502,773	224,443	225,174	0.0	*Ricinus communis* mitochondrial genome
ctg_49	4,446	87.98	Copia-74_ALY-I	5,483	1,540	5,043	0.0	*Arabidopsis lyrata* LTR retrotransposon
ctg_347	4,234	12.01	Gypsy18-PTR_I	7,283	640	4,969	0.0	*Populus trichocarpa* LTR retrotransposon
ctg_269	3,671	7.49	GQ856147.1	3,792,376	277,261	278,161	0.0	*Citrullus lanatus* mitochondrial genome
ctg_41	3,555	21.38	Copia-88_VV-I	7,228	1,627	2,612	4.00E-174	*Vitis vinifera* LTR retrotransposon
ctg_658	3,462	4.13	EU365401.1	509,941	103,101	103,722	0.0	*Bambusa oldhamii* mitochondrial genome
ctg_258	3,447	4.27	HQ874649.1	502,773	223,813	224,397	0.0	*Ricinus communis* mitochondrial genome
ctg_181	3,350	43.54	EnSpm-6_STu	6,594	3,865	4,636	3.00E-50	*Solanum tuberosum* EnSpm DNA transposon
ctg_321	3,346	4.86	JQ248574	281,132	118,398	119,277	0.0	*Daucus carota* ssp. *sativus* mitochondrial genome
ctg_428	3,242	6.19	JQ248574	281,132	225,651	227,064	0.0	*Daucus carota* ssp. *sativus* mitochondrial genome
ctg_256	3,189	23.66	EnSpm2_PTr	12,422	2,262	2,393	3.00E-28	*Populus trichocarpa* EnSpm-type DNA transposon
ctg_34	3,147	6.08	JQ248574	281,132	152,185	153,281	0.0	*Daucus carota* ssp. *sativus* mitochondrial genome
ctg_359	3,142	13.20	Copia-3_CP-I	4,073	1,463	2,198	1.00E-140	*Carica papaya* LTR retrotransposon
ctg_407	3,077	16.79	MuDR-1_STu	9,408	1,590	1,687	2.00E-09	*Solanum tuberosum* MuDR-type DNA transposon
ctg_93	2,900	4.55	EU431224.1	476,890	136,810	136,903	2.00E-29	*Carica papaya* mitochondrial genome
ctg_352	2,893	39.53	GYPOT1_I	5,099	2,780	3,366	3.00E-87	*Populus trichocarpa* internal sequence of GYPOT LTR
ctg_157	2,637	4.77	JN375330.1	715,001	704,715	705,342	0.0	*Phoenix dactylifera* mitochondrial genome
ctg_101	2,609	10.09	Copia-72_VV-I	4,168	2,384	3,690	1.00E-153	*Vitis vinifera* LTR retrotransposon
ctg_935	2,556	4.15	BA000042.1	430,597	127,414	127,860	0.0	*Nicotiana tabacum* mitochondrial genome
ctg_833	2,534	5.41	Copia-4_PD-I	4,256	1,437	1,511	4.00E-07	*Phoenix dactylifera* LTR retrotransposon
ctg_631	2,466	5.16	AY061993.1	4,584	824	1,259	0.0	*Daucus carota* mitochondrial genome
ctg_420	2,460	30.33	PSAT6	607	456	553	3.00E-11	*Pisum sativum* dispersed repetitive DNA, PSAT6
ctg_240	2,385	19.22	MuDR-5_ALy	9,235	2,468	2,573	3.00E-13	*Arabidopsis lyrata* MuDR-type DNA transposon
ctg_26	2,380	34.72	Gypsy22-VV_I	5,609	1,978	2,480	1.00E-64	*Vitis vinifera* LTR retrotransposon
ctg_687	2,338	5.31	FR714868.1	396,947	135,650	136,768	0.0	*Malus x domestica* mitochondrial genome
ctg_75	2,240	120.50						No hit

The best hit sequences were found by BlastN searches using contig sequences as queries. Contigs similar to sequences from chloroplasts, mitochondria, and ribosomes are indicated by green, orange, and blue, respectively.

Supplementary Table S3. Summary of cp genome assembly using different amounts of WGS data from *O. sativa* (cv. Nipponbare) and *P. ginseng* (cv. Chunpoong)

Species	Dataset	Amount of WGS used	Coverage (x) to		No. of cp contigs [c]	No. of errors in the initial assembly				
			Genome [a]	Cp [b]		False gaps	False SNPs	Tandem repeats	Mono-polymer	Total errors
O. sativa	Os1	215,000,114	0.5	24.63	4	1	5	0	2	8
	Os2	430,000,026	1	49.31	5	5	2	0	0	7
	Os3	860,000,052	2	99.26	5	0	0	0	1	1
	Os4	1,290,000,078	3	148.67	3	4	0	0	0	4
	Os5	1,720,000,104	4	198.48	3	3	6	0	0	9
	Os6	2,150,000,130	5	248.5	3	2	0	0	0	2
	Os7	4,300,000,260	10	496.03	4	4	0	0	1	5
	Os8	8,600,000,520	20	980.42	4	7	7	1	0	15
	Os9	25,800,001,560	50	2,534.87	6	2	1	0	1	4
	Os10	44,425,734,760	100	5,006.54	10	13	38	3	1	55
P. ginseng	Pg1	75,750,000	0.025	24.49	3	6	4	3	0	13
	Pg2	151,500,000	0.05	49.89	3	9	2	1	0	12
	Pg3	303,000,000	0.1	99.06	3	4	0	2	0	6
	Pg4	454,500,000	0.15	150.38	3	3	1	2	0	6
	Pg5	606,000,000	0.2	200.54	3	3	0	2	0	5
	Pg6	757,500,000	0.25	260.13	3	3	0	2	0	5
	Pg7	1,515,000,000	0.5	512.77	3	0	0	4	0	4
	Pg8	3,030,000,000	1	1,044.53	3	4	0	2	0	6
	Pg9	7,575,000,000	2	2,564.97	3	1	0	3	0	4
	Pg10	15,150,000,000	5	5,101.95	3	2	0	2	0	4
	Pg11	30,300,000,000	10	10,008.88	2	0	2	5	0	7

[a] Coverage to genome was determined by calculation of ratio of total bases to genome size (430 Mb of *O. sativa* and 3.12 Gb of *P. ginseng*). [b] Coverage to cp genome and nR sequence were based on contents of cp and nR reads determined by mapping raw reads to reference cp genome and nR sequence. [c] Number of contigs representing the entire cp genome sequence.

Supplementary Table S4. Estimate of nR content based on FISH signal size in the rice genome

Chr. no.	Relative size (%) in the genome		Estimated DNA content (Mbp)		Estimated nR copy number
	Chromosome	nR	Chromosome	nR	
1	10.84		46.61		
2	10.03		43.12		
3	9.24		39.71		
4	9.10		39.13		
5	8.71		37.47		
6	7.78		33.46		
7	7.74		33.30		
8	7.05		30.31		
9	8.29	1.65	35.67	7.09[a]	894[c]
10	6.86		29.51		
11	8.01	0.56	34.46	2.40[b]	7,409[d]
12	6.33		27.24		
Total	100.00		429,88		

[a]45S, [b]5S, [c]based on one 45S unit of 7.928 kb, [d]based on 324 bp Nipponbare 5S unit length. Note: 5S and 45S loci are based on previous studies.

Supplementary Table S5. Number of monopolymer tracts in cp genome sequences of rice (*O. sativa J*. cv. Nipponbare)

Monopolymer type	Number
$(A)_{8-13}$	49
$(T)_{8-17}$	40
$(C)_{8-11}$	4
$(G)_{8-10}$	2
Total	95

Supplementary Table S6. Primers used for validation of cp genome polymorphism

Primer name	Primer sequence (5' → 3')	Tm (℃)
Os07-F	TGGATGAATCAGTTCAAAGAATTTA	59.1
Os07-R	GTGCCGCCTGATCAATAAAA	61.0
Os17-F	TTTCCGCTACACATAAAAATGGA	60.7
Os17-R	TCCCTTCGTAACCAGTCGTC	60.1
Os18-F	AACCATGGGTCGCTTCTAGT	58.7
Os18-R	GGGGAGGAGTGGACTCTAGG	60.1

Supplementary Datasets

Supplementary Dataset S1.

The cp genome sequences of seven *Oryza* species. WGS sequences of these seven accessions were retrieved from GenBank (Table 1) and the cp genome sequence was obtained using the dnaLCW method. The header of each FASTA sequence indicates species name and accession number.

>O.rufipogon_AC111008369_cp
cccaatatcttgcttcagcaagatattgggtatttctagctttcctttcttcaaaaattgctatatgttagcagaaaagccttatccattaagagatggaacttcaagagcag
ctaggtctagagggaagttgtgagcattacgttcgtgcattacttccataccaagattagcacggttgatgatatcagcccaagtattaataacgcgaccttggctatca
actacagattggttgaaattgaatccgtttagattgaaagccatagtactaatacctaaagcagtgaaccaaatccctactacaggccaagcagccaagaagaagtg
taaagaacgagagttgttaaaactagcatattggaagattaatcggccaaaataaccatgagcggccacaatattataagtctcttcctcttgaccaaatctgtaaccct
cattagcagattcgttttcagtggtttccctgatcaaactagaggttaccaaggaaccatgcatagcactgaatagggaaccgccgaatacaccagctacacctaac
atgtgaaatggatgcataaggatgttatgctctgcctggaatacaatcataaagttgaaagtaccagatattcctaaaggcataccatcagagaaacttccttgaccaa
tagggtaaatcaagaaaacagcagtagcagctgcaacaggagctgaatatgcaacagcaatccaaggacgcatacccagacggaaactcagttcccactcacga
cccatataacaagctacaccaagtaagaagtgtagaacaattagctcataaggaccaccattgtataaccactcatcaacagatgcagcttcccaaattgggtaaaa
gtgcaatccgatcgccgcagaagtaggaataatggcaccagagataatattgtttccgtaaagtaaagaaccagaaacaggctcacgaataccatcaatatctactg
gaggggcagcgatgaaggcgataataaatacagaagttgcggtcaataaggtagggatcatcaaaacaccgaaccatccgatgtaaagacggttttcggtgctag
ttatccagttgcagaagcgaccccacaggcttgtactttcgcgtctctctaaaattgcagtcatggtaagatcttggtttattcaaattgcaaggactcccaagcacacg
tattaactagaaagataatagaaggcttgttatttaacagtataatatagactatataccaatgtcaaccaagccagccccgacagttgtatatccatacaacaaaattta
ccaaaccaaaaaattttgtaaatgaagtgagtgaaaaatcaaaactcagattgctcctttctagtttccatatgggttgcccgggactcgaacccggaactagtcggat
ggagtagataattattccttgttacaatagagaaaaaacctctccccaaatcgtgcttgcatttttcattgcacacgactttccctatgtagaaataggctatttctattccg
aagaggaagtctactaattttttagtagtaagttgattcacttactatttattatagtacagagaacatttcagaatggaaactgtgaaagttttaccttgatcatttatcaat
catttctagtttattagttttgtttaatgattaattaagaggattcaccagatcattgatacggagaatatccaaataccaaatacgctcactgtgcgatccacggaaagaa
aagtaagttgttttggcgaacatcaaagaaaaaacttgctcttcttccgtaaaaaattcttctaaaaataccgaacccaaccattgcataaaagctcgtaccgtgcttttat
gtttacgagctaaagttctagcgcatgaaagtcgaagtatatactttagtcgatacaaagtcttctttttttgaagatccactgtgataatgaaaaagatttctacatatccg
accaaaccgatcaagaatatcccaatccgataaatcggtccaaattggtttactaataggatgccccgatccagtacaaaattgggcttttgctaaagatccaatgaga
ggagtaacagggactttggtatcgaatttttttcatttgagtatctattagaaatgaattctccagcatttgattccttactaacaaagaatttattggtacacttgaaaagtac
cccagaaaatcgaagcaagagttttctaattggtttagatggatcctttgcggttgagtccaaaaagagaaagaatattgccacaaacggacaaggtaacatttccatt
tcttcttcaaaagaaaagttccttttgatgcaagaattgcctttccttgatatcgaacataatgcataaggggatccataacgaaccatatggttttccgaaaaaaagcag
ggtacattaacccaaaatgttccatcttcctagaaaagatgattcgttccagaaaggttccggaagaagttaatcgcaagcaagaagattgtttacgaagaaacaaca
agaaaaattcatattctgatacataagagttatataggaaccgaaatagtcttttattttctttttcaaaataaaaatggatttcattgaagtaataaaactattccaattcga
gtagtagttgagaaagaatcgcaataaatgcaaggatggaacatcttggatccggtattgaaggagttgaagcaagatatccaaatggataggatagggtatttctat
atgtgctagataatgtaagtgcaaaaatttgtcttctaaaaaaggaaatattgaatgaatagatcgtaaattctgaaactttggtatttctttttcttccggacaagactgttc
tcgtagcgagaatgggatttctagaacgatcgcaaacccctcagatagaatctgagaataaaactcagaataaaaaaaaattgttgtaatccaataatcgatcttggtta
ggatgattaaccaaattaatccaaaaattctgctgatacattcgaatcattaaccgtttcacaagtagtgaactaaatttcttgttattagaaccaataatttcgacaagttc
ggaaccatttaatccataatcatgggcaaacacataaatgtactcctgaaagagtagtgggtagacgaaatattgtctaggaaatttaagtttttctgaataaccctcga
atttttccatttgtatttctacttgaatcagagagagagaaatatttctcggtttatcaaatggtgatacatagtacaatatggtcagaacagggtgttgcatttttttaatacaa
acccctggggaagaaaaggagtctaatccacggatctttttccgctccttttctatccaatttgtttatgtttgttctaattacaaaagagaacaaatcctttatttttgcagg
ccaattgctcttttgactttgggatacagtctctttatcaatatactgcttcttttacacattcaatccataacatcctttttcaatccaaaatcaagaataattaggatttctaaa
aaaaaaaagaaaaaatcaaaggtctactcataggaaaaccagcttttccctacatcaggcactaatctatttttaacgtctaattagatcagggagttcttccaattaaga
agttaagctcgttgctttttgttttaccagaattggagccaggctctatccatttattcattagacccagaaaatcagaatttttttattccattccaaaaatccaaaataaga
aattgattttattacgacatgctatttttttccattcattacccttgaggatcagtcgcggtcttatagactctaccaagagtctggacgaatttttttgcttcatccaaatgtgta
aaagatcatagtcgcacttaaaagccgagtactctaccattgagttagcaacccagataaactaggatcttagatacgatcgaaatccaaaaatcaatggaattacac
cgcacaccccctgtcaaaatcttaaaatagcaagacattaaaagaaagattttatcaccattgaaaacactcagataccaaaaggaacgggtctggttaaatttcactaa
ggttaaaagtggcaccaatcacgatcgtaaaattgtcatttttttagcatttttatttaaataaataaataaatcttgtatgagagtacaaacaagagggacaaccctacca
tttgagcaaagtgtaggcaaaaaacctaatagggagtgaggataaagagacttatccatctacaaattctagatgttcaatggacctttgtcaatggaaatacaatggt
aagaaaaaaattagatagaaaaactcaaaaaaataaaggcttatgttggattggcacgacataaatccagtcaaaaataggattaagaaagaggcaaattatttctaa
atagttagacaacaagggatactagtgagcctctcctagttttttattgatttagttcttcaattaactcaaagttctttctttttctttaaagaattccgccttccttaaaatatc
agaaactgttcttgtaggttgagcaccttttttcaaggaaatagagaatagctggaacatttaaacaagtttgattctttatcggatcataaaaacctacttttcgaagatct
cttccttctcttcgagatcgaacatcaattgcaacgattcgatagacagcttattgggatagatgtagataaacaaagcccccccctagaaacgtataggaggttttctcc
tcatacggctcgagaatatgacttgcattaatttccgtacagaaaaaacaaatttcatttatactcatgactcaagttgactaattttgattgacagacttgaaagaaaaaa
atcctttgaaatttttttgagtcgtctctaaactcttttctttgcctcatctcgaacaaattcacttttattccttattccggtccaattctattgttgagacagttgaaaatcgtgttt
acttgttcgggaatcctttatctttgatttgtgaaatccttgggtttaaacattacttcgggaattcttattctttttttctttcaaaagagtagcaacatacccttttttcttatttcct
tcgataaagcatttccctcttctatagaaatcgaatatgagcgattgattctgatagacttttaatcaaaagagttttcccatatcttccaaaattggactttcttcttattttaa

cctttgatttctatattatttcgatttctatattaagggtagaatgacaaagttggcctaatttattagttttcactaaccctagattctttcccttgataaaaaataaattctgtc
ctctcgagctccatcgtgtactatttacttagcttacttacaaacaacccagcgaaaattcggttcgggacgaatagaacagactatgtcgagccaagagcattttcatt
actatggaaaatggtggatagcaaaatccacaatcgatcgtgtccttcaagtcgcacgttgctttctaccacatcgttttaaacgaagttttaacataacattcctctaatt
tcattgcaaagtgttatagggaattgatccaatatggatggaatcatgaatagtcattagtttcgtttttgtatactaattcaaacttgctttgctatctatggagaaatatga
ataaaagaaattaagtatttatcgggaaagactccgcaaagagccaatttatttaaacccatattctatcatatgaatgaaatatagttcgaaaaaagggaataaacaag
tttgcttaagacttatttattatggaatttccatcctcaacagaggactcgagatgatcaatccaatcctgaaatgataagagaagaattgactcttctccaacaaataaa
ctatcaacctcccgtttaattaatttaattaatatattagattagcaatctatttttccataccatttttccgtaacaaaactaattaactattaactagttaaactattgcaatga
aaagaaagtttttggtagttatagaattctcgtatttcttcgactcgaataccaaaagaaagaaaaaaatgaagtaaaaaaaacgcatttcctgtaaagtaaaattaag
gtctttgcttttacttattttttcttttacctaaaagaagcaactccaaatcaaaattgaatccattctatctaacgagcagttcttatcttatctttaccgggatggatcattctg
gatatttaaaaaatcgcggatcgagatcgttttcgcttaaccaaagaaagaaaaagaagaaggaaccttttttactaataaaatactataaaaaaaaatttatctctatcat
aaatctatctctaccataaaggaataggtctcgtttttttatacaatgttctacgtcaagtttaaaattttttcatgaaaaaaagattttcaatttgactggacttgacactggatt
atgttttctgagacagaaaatgaacgcattaggactgcatcgaatctaagagtttataagagaaaaaaattctctttaataaactttatgtctcgtgcagaatacaatacg
atttcatctttcgtttcatcagaaaaaatctgggacggaaggattcgaacctccgagtaacgggaccaaaacccgctgccttaccacttggccacgccccatttcggg
ttttatgcgacactaataaacagtattatgtttatttcttattcgtcaatcctacttcaattacataaaaatggggggtattctcttggtaggattctagacatgcgaataatat
agaatccaaaaaatgcattgatcattacatggaattctattaagatattatatgaaagtcgaatttcttccactctcatttgagagtgcgaatacaaggaggtattttgtgttt
gggaaagtccgaagaaaaaaggattttgaatcctccttttcctttttcccttagaaaaataactcaatcaaaatccaattatctactctacaagaacgaaacgcttgttatg
cctaatatacttagtttaacctgtatttgttttaattctgttatttatccgactagtttttttcttcgccaaattgcccgaagcttatgccattttcaacccaatcgtggattttatgc
ctgtcatacctgtactcttttttctattagcctttgtttggcaagctgctgtaagttttcgatgaaatctttactactctgtctgccaaattgaatcatgtattcattctaaaaaaa
ttcgaaaaatggataagagccgagaagtcttatattatgaaccttcgattctaaaattcaaattcttctacattgaatgtatagctgcagcaataaatttggatcagcctttc
tactccctgcatctacgttgagcaggtatctttaggtaaccgcacaatacctaacctaatttattgataagagtgcttattataaatcaattcttgcaatttttttcaaaaattg
atttttgcatttttaggtgtcaaaataaacaaaacccatcctagtggatttgtgtggtaaggaaaaacgggtaatctattccttaaaaaaaaatcttggagattatgtaatg
cttactctcaaactttttgtttatacagtagtgatattctttgtttccctctttatctttggattcttatctaatgatccaggacgtaatcctgggcgtgacgagtaaaaatccaa
aattttttcttacaaattggatttgtttcatacatttatctacgagaaaatccgggggtcagaattccttccaattcgaaagtcccaaacgatccgagggggcggaaaga
gagggattcgaaccctcggtacaaaaaaattgtacaacggattagcaatccgccgctttagtccactcagccatctctccccgttccaaatcgaaaggtttccgtgat
atgacagaggcaagaaataacgattgcaaaaaatccttcctttttctttcaaaagttcaaaaaaattatattgccaattccattttagttatattctttttttcttaatgttaataaa
aaaaagaagaaaattcttctttttttctttctaattctaaaattggatattggctaaaagacaatcagatagattttctcttcagcaggcatttccatataggacttgttataata
aaacaagcaggttatagaaaaaaactcttttttttattatttatcaacaaagcaaaaaggggtcttatcaaaccaacccaccccataaaattggaaagaaagataaagta
agtggacctgactccttgaatgaggcctctatccgctattctgatatataaattcgatgtagatgaaattgtataagtggatttttttgtatttccttagacttagaccacgca
aggcaagaatttctcgctatttactatttcatattcttgttactagatgttctataggaataagaagaaatcgcaacccctttccgctacacataaaaatggatttcgaaagt
caatttttcttttcaatatctttactttttttcagaatcctatttttgttcttatacccatgcaatagagagcgagtgggaaaagagaggttactttttttcatttttcccttaaaaaa
taggctttcttggaaataggaatcatggaataatcctgaattccaatgtttatttctatagtataagaaaaactaattgaatcaaattcatggatttaccacgacctcggctg
tgaccccatagataaaaatgcaaaatttctatcttcgagaccattgaaaaaaggcattgaacgagaaaaaatcgtccacagataatctatcgtatgccttggaagtgat
ataaggtgctcggaaatggttgaagtaattgaataggaggatcactatgactatagcccttggtagagttactaaagaagaaaatgatttatttgatattatggacgact
ggttacgaagggaccgttttgtttttgtaggatggtctggcctattgctttttccttgtgcttatttcgctttaggaggttggtttacagggacaacttttgtaacttcttggtat
acccatggattggcgagttcctatttggaaggttgcaatttcttaaccgcagcagtttccacccctgccaatagtttagcacactctttgttgctactatggggcccgga
agcacaaggggattttactcgttggtgtcaattaggtggtctgtggacttttgttgctctccatggggcttttgcactaataggtttcatgttacgtcaatttgaacttgctc
ggtctgttcaattgcggccttataatgcaatttcattctctggcccaatcgctgtttttgtttccgtattcctgatttatccactggggcaatccggttggttctttgcgccga
gttttggcgtagcagcgatatttcgattcatcctcttcttccaaggatttcataattggacgttgaacccatttcatatgatgggagttgccggagtattaggcgcggctct
gctatgcgctattcatggggcaaccgtggaaaacactctatttgaggacggtgatggtgcaaataccttccgcgcttttaacccaactcaagctgaagaaacttattca
atggtcaccgctaatcgcttttggtcccaaatctttggtgttgctttttccaataaacgttggttacatttctttatgctatttgtaccggtcaccggtttatggatgagtgctat
tggcgtagtcggcctggctctgaacctacgtgcctatgacttcgtttcccaggaaatccgtgcagcggaagatcctgaatttgagactttctacaccaaaaatattcttt
taaacgagggtattcgtgcgtggatggcagctcaggatcagcctcatgaaaatcttatattccctgaggaggttctaccacgtggaaacgctctttaatggaacttttgt
tttagctggtcgtgaccaagaaaccaccggttttgcttggtgggccgggaatgccagacttatcaatttgtcgggtaaactacttggagctcacgtagcccatgcagg
attaatcgtattctgggccggagcaatgaacctatttgaagtggcccatttcgtaccagaaaaacccatgtatgaacaagggttgattttacttccgcacttagctactct
aggttggggagtagggcccgggggagaagttctagatacttttccgtactttgtatctggagtacttcatctaatttcctccgcagtcttaggcttcggtggcatttatca
cgcgcttctgggaccggagactcttgaagaatcttttccattctttggttatgtgtggaaagatagaaataaaatgactacaattttgggtattcacctaattttgttaggta
taggtgcttttcttctagtactcaaagctctttattttggcggtatatatgatacctgggctcctgggggaggagatgtaagaaaaattaccaatttgacccttagccccg
gtgttatatttggttatttactaaaatccccttttgggggagagggttggattgttagtgtggatgatttagaagatataattgggggacatgtatggttgggtttcatttgtg
tatttggcggaatttggcatatcttaaccaaacccttcgcatgggctcgccgtgcatttgtatggtctggagaagcttacttgtcttatagtttaggcgctttatctgtctttg
gttttatcgcttgttgttttgtctggttcaataatacagcttatccgagtgagttttatggacctactgggccagaagcttctcaagctcaagcatttacttttctagttagaga
tcagcgtcttggagctaatgtgggatctgcccaaggacccacaggtttaggtaaatatctaatgcgttccccaacgggagaggttatttttggagggggaaactatgcg
tttttgggaccttcgtgctccatggttagaacctctaaggggccccaacggtttggacttgagtaggttgaaaaaagacatacaaccttggcaagaacgacgttcggc
agaatatatgacccatgctcctttaggctctttaaattctgtgggtggcgtagctaccgagatcaatgcagttaattatgtttctcctagaagttggttagcgacttcccatt
ttgttctaggattcttctttttttgtgggccatttgtggcatgcaggaagagcccgggctgctgcagcaggatttgaaaagggaatcgatcgtgatttggagcctgttcttt
acatgacccctcttaactaagattttcttatttatacctgttctactgtttttttctgttctggctcggttattctatctagccgagccattcattcctttctatgaaagaaagataa
ggggacagaacaaaaaaaacaaataaagaaacaaacgtattcaatacgcaaaagaaaagagagaggaaagcaaaaggagagagagggattcgaaccctcga

tagttcctagaactataccggttttcaagaccggagctatcaaccactcagccatctctcccacagcctaatccttattttattcctacaaatagaacatagccatacgaaa
tgactcactaacctctagaaacatctcaaatacaaatccctttttcgatatatttctgtatactgtatccatgtatacaggatccgctatatccgcttgtgaaataaagcataa
aaccccctcaaccccatatccaaataaaaaaaagtggtgagtaataagttttaaagagaagaatcaatggattcatgattaaacccctcctacttcttgtattttattacaat
tttgattaagtgagggatcaaatataatatgtagtcaactttatttgatggtaacttggaggattagaaatatgactattgctttccaattagctgtttttgcattaattgtgact
tcctcagtcttagtcattagtgtaccccttgtatttgcttctcctgatggttggtcaaacaataaaaacgttgtattttccggtacatcattatggattggactggtctttctgg
tagctattctaaattctctcatttcttaaatgtgtttagtatttagtagcccgatacaaaataaaaaagggccgtttattcggattgtgagacgcattaaaatgcaatttgcgc
cccgaattgattgacagacaattaaattaataaaaagaaaactcaaatagaaaatgaaacggtcgacccagacatagacggtcgacccaggcggatataccctata
aaatataggacgtagcgagcgtagttcaatggtaaaacatctccttgccaaggagaagatacgggttcgattcccgccgctcgccagcttaatttagtaaggtactat
gataaaaaaatttagtctacttttattaaagtaataggtgttagtctagtaccgtaccccttttactatcttagcccccttttgcaccccactcaaaaaaaagagcactacagcg
gcgggaatcgaactcggcaacagggttccctaaaccggggattcaccgaaacaaacaaccagcaaacagcttttaaagggaagggagatagactgtgcctttctt
tcatttatttttttcttttctgcaaggtagggggccttgagagttcctcttgtggtagcaagttacttcgcaacctgctcaatttggccttatagggtcgggaactaatgaata
aaaagggttggataccgcccaaccacccagccctctaccatatctagacaaatagaatagtccttttatacagactgctaagtgcggagacgggaatcgaacccgt
gacctcaaggttatgagcctcgtgagctaccaaactgctctactccgctctggagcgctggaaaccggtggacgaaaaaggttgaatacaggcctctaccatgtct
agacaaatagaatagttattttatacagaatggagcgggtagcgggaatcgaacccgcatcgttagcttggaaggctaggggttatagtcgacgttggttgattattat
taacgtctctaattcaaaaccgaacatgaaattttgatttcattcggctcctttatgggtattctcaccacttaacatctaagtcagcttttttgtctgaatggaaccaaggct
ctctgctttctagatgatccctaaagagtaggagatagaaatttgcctaaatctatctaatctaattacttcgttccctaatttcatttaagagatcctgaggaaaagaattg
ggtttccaccgagctgaaacaatatgctgatggttctagtaaaccaaaactaccgttttttagctatttggcttccatttcctttttttaacaaaagaagatttagttacgattg
gaaataaatttttttgtatcttcatccatagatcctttactcatattttaaaaattggaatccttaatccaatgcaaaattatgcttcgcgactctgtactcataatccaaatcct
atttgtttttggatgcaatttcaattagtctttgggtacaaatcgcgaaaatgcatattcttcctcaatatgctattgagagaaaaaggagtaaatcctttctaagaactaaa
gttttcatcggaatataaaaaaccttaaggacgccttaagtatatcatttcaaattcagttattaatagaacgaatcacacttttaccactaaactatacccgctacatgtaa
attctgataccaacgctaccctttgtcaagggtagccattcgagaaagatgctaattccctcttagttaatgaaacagagaaggttcctcacagttagcggttggtactt
cgatcgcgggcctttcctttactttcttttttgttcagaattgaacaaagaatttggggaagaaaacatcttcccccacttatcatgaaatctgggccatagagaaagagt
gagatgtttttttttatttatcatagactttccctatggcttgagagaaacaataaataacttaaagaaaaaggcacataggagccgaaggatttacttgatgtaaagaag
attctgaatgtctctgcttagtcgattctctccgtttaacttttttcttcttcttttccactcaattctagtttattagattcttgtttaaaagaatcaaagaagatgaatagaact
aagaacacacaaaaaaagagcatataggcccgagaccattaccaaaagttcttcccaataatcatattgggtatctgttcccttcctttcctattaggatggggcatgtt
ggattttccatatccatataccatcgaaccttaagggttccagaaccctcctttttttgctagttttcggaaacggaaaaccctgaaccaggagcagtataaattctactgc
ccgccttttacaagaaaattggtgataaaactccactacagtttgttcaaaatgcaccaatcagaatcccttgagaatattcaagtaccctatttctaagatatttctaaga
gaagaggtacctgctgaaaatcgcttcaacaaatccgcttaagaaaaattgcaaagttttgaactcgaaggtttttccaagggatgcctgttaaaaattgtttcaacctc
tcaccaaaacagacaagaagtatatcactgaaaattaatacccagccatatgggtatatgaagggcgcgaattcgtttataccccacccaattagaggaaataaaac
ataaatggagaaagttttcatgataagatcagccaatagaagaaaaaagtccctaatttttcagaccgttctgagcatgtgaaaagtcaatagcctaaagataaaaaac
cctatactttgtgcaagtgataagagagaatatgaaagattttcctatttttcttgattttcttaagattttttgaacctgaccatgaatagacttatatatctcgatatatacag
atataatgtacattatggagtagacctataatgggaaatgaaagtggctaattttggaattgaataagaagcccttttaactcagtggtagagtaatgccatggtaaggc
ataagtcatcggttcaaatccgataaagggcttttttacttagtggtagagtaatgccgcggtaagacgtgagtcagtggttcaaatccgatacagtacttttctactaa
attcattcactttctttctttttgaaaatttctctttttttattgaatttgataacttagtgcgagatgcatgcatttttagtttaaacactaagcgaagcaggggggtgtaaattc
caaaaaagaaattggactctttttcctattagatcaatcaaatcactacccgtactgaactaatatagaatccctttttttttattaatctattcttattccatatcctttataaacg
aatttccctaaaaagtaggggatgatccgtgaattaacctaaccatcaactaaaaaaaaatcctacaaaagcataatggaaaagtaggaaggactccttgctttggatct
agttatactcttcgagtatattgacaattccaaaaaactgctcatactatcattatagtataatgaggagcggttgtataccgccctatcgtctagtgatgcccctatcgtct
agtggttcaggacatctctctttcaaggaggcagcggggattcgacttcccctgggggtagggagtattatgaaaggaggttaatcatagattatcaaaaaccctag
aataaattcttcctgggtcgatgcccgagtggttaatggggacggactgtaaattcgttgacaatatgtctacgctggttcaaatccagctcggcccaaaaatctagg
gcttcgtgaatatgagttaaatccatttttttttcttccataaaaaagaatatttgatccatagaaataaaagaaataaaggataaaaagaaaaggggaaatatctttctaat
ctatatctctttcattcctctcttacaaacaaaagacctttttcttattggttattgaaaggtggattattatctatttttagcgataataaatcgcgacatactagttatgtcattct
cactatacccccatacgatacgggggtatgtagtatatgattcgtctatttcttagagtaggacaggcgaatattcttattcttatggttccatttaagaataagtatgccat
acgccccgcggggattgtagttcaattggtcagagcaccgccctgtcaaggcggaagctgcgggttcgagccccgtcagtcccgaactagggttcaatgaatgg
agaaattcatctttcctttttccatgaaaaaagggggggcaggaagcaagatcaaatacctatggggtacccccatttcactttttttagttcgcgtttctcagtaaagaga
ggagagtataggaatttttttatcactacttctggttgatagcgaaagacatacatatcatacgtggaagggatcctcctatgttatactattccactctcaaccatgaatt
gatttgatagatccgatattcataatattgaattgattcagtattatcagaatgcaagtcctccccttgaatttacaggataccctttttccctctccatgggattacatcccga
gttattgcgaaaaaaaaagaggttatggaagtcaatattctcgcatttattgctactgcattgttcattctagttcctactgccttttttacttattatttatgtaaaaacagtcagc
caaaatgattaattggaagttcaattaatcattgaagaaatgaaaaagggattaaataaaataaaaatccaagtcttaaatgaaaggatccggttggaatcataaagtg
tggtagaaaaaactacatatagtttttctaccacactttagagtctttctattatattatcttgaatctacatagaatagattactagattgaaatagtagtctaattcaatttct
tttttcactgcatccacttaatttcaatcaagtcaaaatgaaaaaatccatggagggagagaaaaataatatgagaatagactatagtaaaagaaaaaaagtaaaagg
aaaaaaacagcgaatctttcatgcttaaacatgtcgcgaaatgtttcaaaagagcataaaatttattttaagaactaagaataagaaaagagtataaaacaaatggaaa
atgtgcgatatgttgggaatagctccgcggaagaaaatctaaagttcttatgtatagaactttttttaaccatgggtcgcttctagtagcgattatgaattgctctcaccgct
ctttctatttctattttctattctttctatttctattctactatatagaaatagaaagaatagaaggactctttcttacaagagtttcttacaggagtgaaacaaaattaaagaaa
gagagaaagaataaagttcggcaaaatgattaatgcaaaacggtcaattaaagaaaagagttgatacaacaattcgactactcaatcaattagtagtatccctagagt
ccactcctcccccatactactagtgaaagagaaaatgtaaagactaccattaaagcagcccaagcgagacttactatatccatgtaaattatgtctcctatttctatgaa
ggaattattctactattgatgaataatcatagtagaatcaagggtacagagtcaaaaaggggttctgacctaaggctatggatgaatcagttcaaagaatttactcttaa

caaattcttagagtatttctggtagaatttaacaaattcttagagtatttctggtagaattggggagcattaagtataaatatgatacatagccctttcttattaataaaagaa
taaggaaacgctatctcatccctattggtatcggtttgggccactactgctaaaacaaaccccagtttgaggaaagaacggtgggttctcaaaatccagtatcgccga
gccttgttattctcttgccccaacttatgcggggtgcaaatttgtcgatttggatcagtactataagcctaagtattttattgatcaggcggcacccagatttgaactgggg
ataaaggatttgcagtcccctgccttaccgcttggccatgccgccaaaaaatacgatctaaaatcgagaaaagagcaagtattcatccacgtttcttactaaaactaac
tttcttttatcttaaatctaattctacttactttttccaatctttttcaaaaaatctattcatgcttttttggatccagtttcgattattctcctcaaaggattctatcttaaaacacac
attgctaacactagaaaacttccctttttctttctattgaaatgaaaaaggagaaaagtggatttctagtcacaagctacaaaattaagaacaaattggaaccattaactag
aattctctttttattttgaatttcggtattctctcccgcctgccatttaatggcataataaaagacaatggatttatgcctaatccgtatataggtaaactccaggtccgaaca
gcattattatctatggatccccccttatgtacatatctctgtggagaatcgttctttaatttttcattgcattaaatatcttgaataaaaaaaaagaaaattgactttgctatgtgga
ggtccagaactagattggcatgtacttaaaaaagtacttactttattttaggattctacaacgaaatcttatatcttatattttatagaaattctactactacgaaacaaaaaa
gaaccttcaaattctttttgaaattaaactaagcgtgctattctcaatcgaactaaagtcaaactttctagtgcttataaattattatattttggttttatcccattcatagaaag
gagaaaaaatgagaaatctttgccgtccaatctaagaatatcataaactgtaagtggcagaattttttcgaggttctaggaatgttttatcacttcattttcattccatttgg
acccctgggaaattcgaactttcgttgaaattgtctctattcatatgtatgaaatacatatatgaaatacgtatgtggagttcccgagaatttcatgtgatttagtaaacaga
atatagattccataattgctagatcgatctgtagggattgatgaagagtgagctgataatggaatttttcttcgataaacaggaaacttaagattaagatgctccggaatg
gaaatgagggaatgtccacaatacccggatttagtcagatccaattcgagggattttgtaggttcattaatcaaggcttggcagaagaacttgagaagtttccaacaat
taaagatccagatcacgaaatttcatttcaattatttgcgaaaggatatcaattgctagaaccttcgataaaagaaagggatgctgtgtatgaatcactcacctattcttcc
gaattatacgtatccgcgcgattaatttttggtttcgatgtgcaaaagcaaaccatttctattggaaacattcctataatgaattccttaggaacctttataataaatggaat
ataccgaattgtgatcaatcaaatattgctaagtcctggtatttactaccgctcggaattagaccataaaggaatttctatctacaccgggactataatatcagattgggg
aggaagatcggaattagcaattgataaaaaagaaaggatatgggctcgcgtgagtagaaaacaaaagatatctattctagttctatcatcagctatgggttcaaatcta
aaagaaattctagataatgtttcctaccctgaaattttcttgtctttccctaatgctaaggagaagaagaggattgagtcaaaagaaaaagctattttggagttttatcaac
aatttgcttgtgtaggtggggacctggtattttcggaatccttatgtgaggaattacaaaagaaattttttcaacaaaaatgtgaattaggaaggattggtcgacgaaata
tgaatcgaagactgaatcttgatatacctcagaacagcaccttcttgttaccgcgagatgtattggccgctacggatcatttgattggaatgaaatttgaaacgggtata
cttgacgatgacgatatgaatcacttgaaaaataaacgtattcgttcggttgcggatctgttacaagatcaattcggactggctcttggtcgtttacaacatgcggttcaa
aaaactatccgtagagtattcatacgtcaatcaaaaccgactccacaaactttggtaactccaacttcaacctcgattttattaataactacttacgagaccttctttggta
catatcccttatctcaagtttttgatcaaaccaatccattgacacaaacggttcatgggcgaaaagtgagttgtttgggtcctggaggattgacggggagaactgcaa
gttttcggagccgagatatccatccgagtcactatgggcgtatttgtccaattgacacgtccgaaggaatcaacgttggacttactggatctttagctattcatgcgaga
attgatcattggtggggatccgtagagagtccattttatgaaatatctgagaaagcaaagaaaaaaaaagagagacaggtggtttatttatcaccaaatagagatgag
tattatatgatagcagcaggaaattctttgtccttgaatcggggtattcaggaagaacaggttgttccagctagataccgtcaagaattcctgactattgcatgggaaca
gattcatgttagaagtatttttcctttccaatatttttctattgggggttctctcattccttttattgagcataatgatgcgaatcgagctttaatgagttctaatatgcagcgcc
aagcagttccgctttctcggtccgagaagtgcattgttggaactggattggaacgccaaacagctctagattcgagggtttctgttatagccgaacgcgagggaaag
atcatttctactaatagtcacaagatccttttatcaagtagtgggaagactataagtattcctttagttacccatcggcgctctaacaaaaatacttgtatgcaccaaaaac
ctcgggttccgcggggtaaatccattaaaaaaggacaaattttagcggagggggctgctacggttggtggggaacttgctttaggaaaaaacgtattagtagcttat
atgccatgggaaggttacaattttgaagacgcagtattaattagcgaacgtttggtatatgaggatatttatacttcttttcacatccgaaaatatgaaattcagacggata
caacaagccaaggctccgctgaaaaaatcactaaagaaataccacatctagaagaacatttactccgcaatttggacagaaatggagttgtgaagttggggtcctg
ggtagaaacaggcgatattttagtaggtaaattaacgcctcagatagcgagcgaatcgtcctatatcgcggaagctggattattacgggccatatttggtcttgaggta
tccacttcaaaagaaacttctctcaaactaccgataggtggaagaggacgcgttatcgatgtgaaatggatccagagggatcccctcgacataatggttcgtgtatat
attttacaaaaacgcgaaatcaaagttggggataaagtagccggaagacacgggaataaggggatcatttccaaaattttgcctaggcaagatatgccctatttgca
agatggaacgcctgttgatatggttttcaatcccttaggagtaccctcccgaatgaatgtgggacaaatatttgaaagctcgctcggattagcaggggatctgctaaa
gaaacattatagaatagcaccctttgatgagagatatgagcaagaggcttcaagaaaacttgtgttttcagaattatatgaagccagtaaacaaacaaaaaatccgtg
ggtatttgaacccgagtacccgggaaaaagcagaatatttgatggaagaacaggagacccccttcgaacagcctgttctaatagggaagtcctatatcttaaaattaat
tcatcaagttgatgagaaaatccacggacgctctactgggccctattcacttgttacacaacaacccgttagaggaagagccaagcaaggggggacaacgaatagg
agaaatggaagtttgggctttagaaggatttggtgttgctcatattttacaagagatacttacttataaatctgatcatcttatagctcgccaagaaatacttaacgctacg
atctggggaaaacgagtacctaatcacgaggatcctccagaatcttttcgagtgcttgttcgagaactacgatctttggctctagaactgaaccatttccttgtatctcag
aagaacttccaggttaatagggaagaagtttgatcggaataaatataaattcttttcttatttctattttatgattgaccaatataaacatcaacaacttcaaattggactcgt
ttcccctcaacaaataaaggcttgggctaacaaaacactacctaatggggaagtcgttggcgaagtcacaaggccctccacttttcattataaaaccgataaaccag
aaaaagatggattgttttgcgaaagaatctttggacccataaaaagcagaatttgtgcttgtggaaattctcgagcgagcggagctgaaaacgaagacgaaagatttt
gccaaaaatgcggagtagaatttgttgattctcggatacgaagatatcaaatgggatacatcaaactcgcatgtcccgtgactcatgtgtggtatttaaaaggtcttcct
agttatatcgcgaatcttttagataaaccccttaagaaattggagggcctagtatatggcgatttctcttttgctaggcccagtgctaaaaaaccaactttcttacgattac
gaggtttattcgaggatgaaatttcatcctgtaaccatagcatttcccccttttttctaccccaggctttacaacatttcgaaatcgggaaattgcgacaggagcaggtg
ctattagagaacaattagcagatttggatttgcgaattattttagagaattcctcggtcgaatggaaggaattagaagacgaggggtatagtggagatgaatgggaag
atagaaaaagacgaataagaaaagttttttttgattagacgcatgcaattggcgaaacattttattcaaacaaatgtagaaccagaatggatggttttgtgcttattaccag
ttcttcctcccgaattaagacccattgtttataggtctggggataaagtagtgacttcggatattaatgaactttataagagagttatccgtcggaacaacaatcttgccta
tctattaaaaagaagtgaattagcgccagcagatttagtaatgtgccaggaaaaattggtacaagaagccgtggatacacttcttgatagtgggtcccgcgggcaac
caacgagggatggtcacaataaagtatacaaatcactttcagatgtaattgaaggtaaagaggggaggtttcgcgaaactctgcttgggaaacgggtcgattactcg
gggcgttctgtcattgttgtgggtccttcactttcattacatcaatgtgggttacctctagagatagcaataaagctttttcagctatttgtaattcgcgatttaatcacgaaa
cgtgctacttctaatgttaggattgctaaaaggaaaatttgggaaaaggaacccattgtatgggaaatacttcaagaagttatgaggggacatcctgtactgttgaata
gagcacctaccctgcatagattaggcatacaggctttccaacccactttagtagaggggcgtactatttgtttacacccattagtgtgtaaaggtttcaatgcagacttt

gatggggatcaaatggctgttcatctacctttatccttggaagctcaggcggaagctcgtttacttatgttttctcatatgaatctcctatctcccgctattggggatcctat
ttgcgtaccaacccaagacatgcttatcggactttatgtattaacgattggaaaccgtcgaggtatttgtgcaaatagatataatagttgcggaaactatccaaaccaaa
aagtaaattacaataataataatcctaagtatacgaaagataaagaatctctttttctagttcctatgatgcactgggagcttatagacagaaacaaatctgtttagacag
tcccttgtggctccgatggaaactagatcaacgcgtcattgggttaagagaagttccgattgaagttcaatatgaatctttggggacttatcgtgagatttatgcccact
atctagtagtgggaaatagaaaaaaggaaatccgttctatatacattcgcaccactcttggtcatatttctttttatagagaaatagaggaagccatacaaggatttagtc
aggcctattcatacactatctaaacaaggaagttagattcggggatgcccctttcgaggggcattccgatttcgctagtatcatcatttttgccgcacgaatccagattg
agattgaggaaaggaagttaactaagttttcgaatcactgactcaggcccattgtcgaatcctactcagcaattgtcgaattatactcagccgaaaaaggggggtactta
tttatggcggaacgggccaatctggtctttcagaataaagagatagatggaactgctatgaaacgacttattagcagattaatagatcatttcggaatgggatatacat
cccatatactggatcaaataaaaacgctgggcttccatcaagccactactacatcgatttcattaggaatcgaggatcttttaacaataccctctaagggatggttagtc
caagatgcggaacaacagagttttcttttggaaaaacactattattatggggctgtacacgcggtagaaaaattacgccaatccgttgaaatctggtatgctacaagtg
aatatttgaaacacgaaatgaattcgaattttcggataacagatccttctaatccagtctatctaatgtctttttcaggagctagaggaaatgcatcgcaggtacaccaat
tagtaggtatgcgaggattaatggcggatcctcaaggacaaatgattgatttacctattcaaagcaatttacgcgagggactttctttgacagaatatataatttcctgct
acggagcccgcaaaggggttgtagatactgctgtacgaacggcggatgctggatatcttacacgtagacttgttgaagtagttcaacatattattgtgcgtagaagag
attgtggtactatccaagctatttctgtgagtcctcaaaatgggatgacggaaaaactttttgtacaaacattaattggtcgtgtattagcaaacgatatatatatcggttca
cggtgcattgccactcgaaatcaagatattggaattgggttagtcaatcgattcataaccacctttcgagcacagccatttcgagcacaaccaatatatattagaaccc
cctttacttgccggagcacatcttggatctgtcaattatgttatggtcggagttccactcatggcgatctggtcgaattgggggaagctgtaggtgttattgcgggtcaa
tctattggggagccagggactcaactaacattaagaacttttcatactggtggggtattcacaggggggtactgccgaccttgtacgatccccttcaaatggaaaaatc
caattcaatggggatttggttcaccccacacgtacccgtcatgggcagcctgcttttctatgttatatagacttgcatataactattcagagtcaggatattctacatagtg
tgactattccttcaaaaagcttgattctagtgcaaaatgatcaatatgtagaatccgaacaagtaattgcggagattcgtgccggaacgtccgctttgcattttaaagaa
aaggtacaaaaacatatttattccgaatcagatggggaaatgcactggagtaccgatgtttatcatgcgcccgaatatcaatatggtaatcttcgtcgattaccaaaaa
caagccatttatggatattgtcagtaagtatgtgcagatctagtatagcttctttttcgctccacaaggatcaagatcaaatgaatacttattccttttctgttgacggaagg
tatatctttggcctctcgatggctgatgatgaggtaagacatagactgttggatacttttggtaaaaaagatagggaaattcttgattattcaacgccggatcgaatcatg
tccaatggtcattggaattttgtctatccttctattcttcaaaataattttgatttgttagcgaaaaagcgaagaaataggttcgccattccattacagtatcatcaagaacaa
gagaaagaaccaatatcctgttttgggatttcgattgaaatacccttatgggtgttttacgtagaaatactatagttgcttattttgacgacccacgatacaaaaaagata
aaaagggttcaggaattgttaaatttagatataggaccctagaggacgaatataggactcgagagaaagactcagagaacgaatacgggagcccagaaaacgaa
tataggacccgagaggaagaatgtaaaaccctagaagacgaatataggactcgagaggaggagtatgaaaccctagaagatgaatatgggatcccagagaacg
aatatgaaaccctagaagatgaatatggaatcctagaggacgaatataggactcgagaggaggaatccgaggacgaatatgggagtccagagaacaaatatagg
ccccgagaggataaatatggcactttagaggaagactcagaggacgaacatgggactttagaggaagactcagaggaagactcagaggacgaatatgggaacc
gggaggaagattccgtcttaaaaaaggggggttttgattgagcatcgaggaacaaaagaatttagtctaaaataccaaaaagaagtagatcggtttttttcattctccaa
gaactgcatatcttgccgagatcctcatccctaaaggtacttgacaatagtattattggagtggatacacaactcacaaaaaatacaagaagtcggctgggtggattg
gttcgagtgaagagaaaaaaaagccatacggaactaaaaatcttttctggagatattcattttcctgaagaggcggataagatattaggtggcagtttgataccactag
aaagagaaaaaaaaagattcgaaggaatcaaaaaaaaagggaaaattgggtctatgttcagtggaaaaaaaattctcaagagcaaggaaaagtattttgttttggttcga
cctgcagtcgcgtatgaaatgaacgaagggagaaatttagcaacacttttcccacaggatctcttgcaggaagagggtaatctccaacttcgacttgtcaattttatttc
tcatgaaaatagcaagttaactcaaagaatttatcatacgaatagtcaattcgttcgaacttgcttagtattgaattgggaacaagaagaaaaagaggaggctcgtgct
tcccttgttgagataagagcaaatggtctgattcgcgatttcctaagaattgggttaatcaaatccactatttcgtatacacgaaaaaggtatgatagcagaagtgcagg
actgattctccataataggttagatcgcaccaataccaattccttttattccaaggcgaagattcaatcacttagccaacatcaagaagctattggtaccttgttgaatcg
aaataaagaataccaatctttgatggttttgtcggcatccaactgttctcgaattggtttttttaagaattcaaaaaatcccaatggggtaaaagaatcgaatcctagaatt
cctattccaaaatttttggggctctttcgaaatttttcgggactcttaggtactattgcacctagtatatcgaatttttcttcatcttactatttactaacgtataatcagatcctg
gtaaaaaagcatttgctccttgacaatttgaaacaaaacttcaaagtacttcaaggacttaaacactctttaataaatgaaaatcaaaggacttcaaatttcgatagtaac
atcatgttggatccattccagttaaattggcactttctccctcatgattcttgggaagagacatcagcaaaaattcaccttggacaatttatttgcgaaaatgtatgtctattt
aaatcgcacataaaaaaatcaggtcaaattttcattgtaaatatagattcctttgttataagagcagctaagccttatttggtcactacaggagcaactgttcatggtcatt
atggagaaatcctttacaaaggggataggttagttacgtttatatacgaaaaagcgagatctagtgacataacgcaaggtcttccaaaagtagaacaaatcttcgaag
cgcgttcaattgattcactatcgccgaatctcgaaaggagaattgaggattggaatgagcgtataccaagaattcttgggggtccttggggattcttgattggagctga
gttaaccatagcccaaagtcgtatctctttggttaataagatccaaaaggtttatcgatcccaaggggtacagatccataatagacatatagagattattatacgccaag
taacatcaaaagtgcgggtttccgaagatggaatgtctaatgtttttttcacctggggaattaattggactattgcgagcagagcgagcagggcgggctttggatgaat
cgatctattatcgggcaatcttattgggaataacaagggtttccctgaatacccaaagtttcatatctgaagcaagttttcaagaaactgctcgagttttagcaaaagctg
ccttacgaggtcgtattgattggttgaaaggcctgaaagaaaacgtagttctgggggggattatacctgttggtaccggattccaaaaaatttgtgcaccgttacccaca
agacaagaacctttatttcgaaattcaaaaaaaaaaaactatttgcgtcggaaatgagagatattttgtttctccatacagaattagtttcttctgattctgacgtaacaaaca
atttctatgagacatcagaatcaccatttaccccatttatatgatttaaggatacataaagcagatttttttactttaaactagatttttgaccttagaacgctaagaggttaga
ttttctatttttattttaatttaaaaagaagtttagttaattcattaaggttatgcttataccatgtagaaggttccatcggaacaattattatttatttcaagctatttcggctctttc
ttaatcttcgaaaagaaagaaatttcgtaatggaaaggtaggatgaaaaaaaagaaaaaatcaaaaggaagtgtggaaaaaatgacaagaagatattggaacatca
atttgaaagagatgatagaagcgggagttcattttggtcatggtattaagaaatggaatcctaaaatggccccttacatctcggcaaagcgtaaaggtactcatattac
aaatctcgctagaaccacccgtttttatcagaagcttgtgatttagtttttgatgcagcaagtcagggaaaaagcttcttaattgttggtaccaaaaaaagagcagcgg
atttagtagcatcagctgcaataagggctcgttgtcattatgttaataaaaagtggttcagtggtatgttaacgaattggtcgattactaaaactagactttctcaatttaga
gacttaagagcagaagaaaagatggaaaaattccaccatctcccaaaaagagatgtggcaatcttgaagagaaaattatctaccttgcaaagatatctcggcggga
tcaaatatatgacgaggttgcctgacattgtgatcgtcctcgatcagcaaaaagagtatatagctcttcgggaatgtgccattttggggattcctactatttctttagccg

atacaaattgtgacccagatctcgcgaatatatcgattccagccaacgatgacactatgacttcaattcgattgattcttaacaaattagtatttgcaatttgtgagggcc
gttctctctatataagaaatcattgattaagaagaatagttaattcttgggcaactgcgtagatttatggaatcacttactattctttttgttttgcatagaaaaaagacggg
gaatattgatatatattagagggtattgatatatattatcatctgatgtgatttcttgatatcctaaatataagattaatacttcacgttgctgagttgagaaaaagatggttga
atcaaaagaattctttttttgaagttcatttttttatcaggggacaatatgaatattataccgtgttccattaaaacactcaaggggttatacgatatatcgggcgtagaagta
ggccaacacttctattggcaaataggaggtttccaaattcatgcccaagtacttatcacttcttgggtcgtaattactatcttgctaggttcagttatcatagctgttcgcaa
tccacaaaccatcccaaccgatggtcagaatttcttcgaatatgtccttgagtttattcgagacttaagcaaaactcagattggagaagaatacggtccctgggttccct
ttattggaactatgttcctttttatttttgtttcgaactggtcgggtgctcttttaccttggaaaattatacagttaccccatggagaattagcagcgcccacgaatgatataa
atactactgttgctttagctttactcacgtcagcggcatatttttatgcgggtcttagcaaaaaaggattgagttatttcgagaaatatattaaaccaactccaatccttttac
caattaacatattagaagatttcacaaaaccattatcgcttagttttcgacttttcgggaatatattggcggatgaattagtcgttgttgttcttgtttctttagtccccttagta
gttcctataccggtcatgtttcttggattatttacaagcggtattcaagctcttatttttgcaacgttagccgcagcctatataggtgaatccatggagggtcatcattgaat
tgactagttttcaaaatagtcttttttttagcttaactcaattcatgcatggttgcggaaaattcgcttggttggaaaacaaaatagttagaattgcgtatgaatatacaatct
agagttgtagaagagagaataggctatattacggaattgccaaacaaagtatataggcattagggaggggcggagtcaggctagatctatatcctttatgtctataag
ttcagtcatcttttgtatgggtttccactttaaggaatttttttttgaatccgattcaatagaaaatgagaaaatacacaaacaaaatagaagaaacaaattgatatgggatat
tatatattcccaagttagattcattatctaatccgatatatggaatcggattccatatccaattcgatgcagcatattgttatcaattggatatcttgatttaattcctattggat
ctggattaggtcgatttccataggggttcttcctctatttcaccttttattatgaattagatgataggggaaaaaatagaaactcaaggatatcgaagaggaaagaaaga
aggatggaatgaaagatcagttggttggaaagaaagagaaatagaataatgagtacacaaacctctaatgattagaaactaaaaaggagatctcgaagcagttcgg
agaattcagattatcgtttcaatttgtactttttagttacttctgtccaatagagcttagaaatatgaatttcttggttgattgtatccttaaccatttcttttttttgacacgagga
actcatcatgaatccactaattgctgctgcttccgttattgctgctggattggccgtaggtcttgcttctattgggcctggagttggtcaaggtactgctgcaggacaag
ctgtagaaggtattgcgagacagccagaagcagaaggtaaaatacgcggtactttattgcttagtctagcttttatggaagctttaacaatttatggactagttgtggca
ctggcgcttttatttgcgaacccttttgtttaatcctaaaaaagaaaacgagtcctttagattagatactttcttctttttttagtaaattggtatttgcttctgcaattccaattat
atcaatactttactcctaatttattactcctagagttttctatttatggggacagacaataccccaccccaggaatagctgatttgaggatgatcaatttagaggatatgttc
gccgtcttgcttcccgccctttgtttagggcagtggaaagtatttttccttttattttaggaattttgggaacatttcaacaaaggaggtctttcacaggtcaaacgagatct
aagacttaatctaaaagaaattactaaattgaatctatttctattaaaaaaaaattgcattaaaaaaaaccgatcaaaaagggcgagcgaagtaagtgatcgaaaaactttg
ttctttgttcgtcctatctataagaggagagcatatgaaaaatgtaacccattctttcgtttttttagctcactggccatccgctgggagtttcgggcttaataccgatatttt
agcaacaaatctaataaatctaactgtagtggttggtgtattgatttattttggaaagggagtgtgtgcgagttgtctatttcaagaatagattggatctatccggctgcac
tttagaatattttttagtatttttcgaataaataagaaaagggtgcacgatctcgacgaattacttctgaataaatgcagaaatcatatgaaagaaccatagcatttcgcga
cctattggtaaatcaaatcaactttgattctctatagaccaataatatgagaccattaacacggttaaagctaaactgcttgaagtctaggcaaaagggggtactctttct
acaactatattagtattagtaccaaatgctttaaacaggaaatagctaatgtagaatttatctgatatagaacactcatatcgataaaatggtttgaactatttactagaag
gggcaccctgccctttttttatccaatgccgaatcgacgacctatgtataaaaaaggagaaagtttttggatttgaagaaaaaagtaggaattctatccattttcattttcca
tttttctatttagtttttcttaatgaaattgaaaattttaactaaagggcaaatacaaataaaaaaacaactttgctgcccacgatagatttttatctaggcggaagagtcctc
ttaatatttatctagtcttatatgggtttcggtatattgaaatataaacagaaaagagagggtagaggataggctcattacattaaaaaaaaatatggaagtagccatagca
aaaaaagaaaaaagggagcgtgagagccaaatgaatcgaaagattcatgtttggttcgggaagagatcataaaagttgtaaacttaatagcaaggtaatctactttc
attaaaagatttattagataatcgaaaacagaggatcttgagtactattcgaaattcggaagaattgcgtagaggaaccattgagcagctcgaaaaagctcgaattcg
attacagaaagtcgaactagaagcggatgagtatcgaatgaatggatactctgagatagaacgagaaaaagcaaatttgattaatgccacttctattagtttggaaca
attagaaaagtctaaaaacgaaaccctttattttgaaaaacaaagggcgatgaatcaggtccgacaacgggttttccaacaagccgtacaaggagctctaggaactc
tgaatagttgtttgaataccgagttacatttccgtacgattcgtgctaatattagcattctcggggccatggaatggaagagttaaattaattaggccttgaacttctacttt
cctttagaatttaggcattatttttccccttgcttccgaaaaaaaaaaatagtaaagaaacactaatggcaacccttcgagtcgacgaaattcataaaattctccgcgaac
gtattgaacaatataatagaaaagtagggattgagaatatcggtcgcgtagttcaagtgggggatgggattgctcgtattataggtcttggtgaaataatgtcaggcg
aattagtcgaatttgcggaagggactaggggtattgctctgaatttggaatccaaaaatgttgggattgtattaatgggcgatgggttgatgatacaagagggcagttt
tgtaaaagcaacaggaagaattgctcagatacccgtgagcgaggcttacttgggtcgtgttataaatgctctggctaaacctattgatgggagaggcgaaattgtag
cttcggaatctcgcttaattgaatctcctgctccgggtataatttccaggcgttctgtatatgaacccccttcaaacggggcttattgctatcgattcgatgattcctattggg
cgcggtcagcgagagttaattattggggacagacaaaccggcaaaacagcagtagctacagatacaattctcaatcaaaaagggcaagatgtaatatgtgtttatgt
agctatcggtcaaagagcatcctccgtagctcaagtagtaactactttccatgaagagggggccatggaatacactattgtagtagctgaaatggcggattcccctg
ctacattacaatacctcgctccttatacgggagcagccctggctgagtattttatgtaccgcgaacggcatactttaataatttatgatgatctctccaaacaggcacaa
gcttatcgccaaatgtcccttctattaagaagacccccccggccgcgaagcttacccaggggatgtttttttatttgcattcacgccttttagaaagagccgctaaattaaa
ttctcttttaggggaaggaagtatgactgctttaccaatagttgagactcaatctggagacgtttccgcctatattcctactaatgtaatctccattacagatggacaaata
ttcttatccgcagatctattcaatgccggaattcggcctgctattaatgtgggtatttccgtttccagagtaggatccgcggctcaaattaaagccatgaaacaagtagc
tggcaaatcaaaattggaattagctcaattcgcagagttacaagcctttgcacaattcgcctctgctctcgataaaacaagtcagaatcaattggcaaggggccgacg
attacgagaattgcttaaacaatcccaagcaaatcctcttccagtggaagagcagatagctactatttatatcggaacaagaggatatcttgattccttagaaattggac
aggtaaagaaatttctggatgagttacgtaaacacctaaaagatactaaacctcaattccaagaaattatatcttctagcaagacattcaccgaggaagcggaaatcct
tttgaaggaagctattcaggaacaactcgaacggttttcccttcaggaacaaacataaattttgcacgtctactcttgttagtagaagtagtagaggagaaatcgttgag
aaagatttttcatttgaatcatgcaaaaaagttttcttagtttttagtatagttatttaaagaatagatagaaataagattgcgtccaataggatttgaacctataccaaaggtt
tagaagacctctgtcctatccattagacaatggacgctttttttcatattttcttctttcttttcattttttatcggaccaagagaaaaaactgttagaccaaaactcttttaggaa
agaaaaacaaatgcatatacaaatgtatgatgcatatatcataaagaaggaatatggagcgggtagtgggaatcgaacccgcaaccccacggttatgagccttgtc
agctaccaaactgttctatcctgttaaactaaagagaggggaactagtggataaaaggggggttgaatacgcccctctaccatatctatacaaatagaatagtccattt
atacagaatggtaaagagggctcttctacgatcatcaattccagaaatccatacaaatacgaaagggtattttatccttaccaactggatcttgttgcacccggtaacaa

acatgcataaaccatttctcgaagtatgtgtccggatagcccaaagtctcgatagttagctctaggtcttccggtcaaaaaacaacgtcgatgaaggcgtgtcggtgc
actattacgtggcagggattgcaatttttctcgcattttcgtttttcactcaaactcaaagggtaaactttgcttcttatctttttttttgaagatcgacgaatcaaatgatattt
ctgttctaatttctgccgcttcctctctctctgaatcaaacttttttttgccataatgtgccgttcctactatctaccaagtatacggttctaatcctagatggaaaaataaata
gaaaaagaaatctaagaaggcggatcctccccctccatcaagagtaatgaactaggtgctgatacagtacaacaaaattaactaaattaaccaaacttgcctgatgtt
gaggcaatcaagaaagcggcataagtgaatatataacccacggaaaagtgagctaatcccaccaatcttgcttgcacaatggaaagagccacgggcttatctctcc
agcgaattaaattagccaaaggtgtccgttcatgagcccatgctaaagtctcaattaattcctgccaatatccacgccaggaaattaagaacataaatccagtcgccc
aaacaagatgtccaaataagaacatccacgcccataccgataaactattcatcccaaaaggattatatccattgataagttgtgaagagtttaaccataggtaatctctt
aaccatcccatcaaataagtggaggattcattaaattgtgaaacgttgccctgccataatgtgatgtgtttccaatgccaataaaaagtaacccatccaatggtatttaa
catccagaaaactgccaaataaaacgcgtcccaagcagaaatatcacaagtaccgccgcgaccagggccgtcgcaaggaaaactatatccaaaatctttttatcc
ggcattaatttggaaccgcgtgcatctaaagcaccctttactaaaatcaatgtagttgtatgcaaacctaaagcaatagcatgatgaaccaagaaatctccaggtccta
ttgttaagaaaagcgaattactattctcgttaacagcattcaaccatccgggtaaccatagggttcgacccgcattgaaagcgggaccgctcgttgaagataagagta
tatcgaacccatatgtcgtcttaccatgagccgattgtatccattgagcaaatataggttcgatcaagatttgcttttctggagtaccaaaagcaagcatgacgtcattat
gaacataaaggcccaaggtatggaatcctaggaagagactagcccaacttaaatgagatatgatagcttctttatggtctaacattcttgccaatacattatcctcattct
gttccggattgtaatctctaatgaaaaaaatagctccatgagcaaaagcccctgtcatgatgaaccctgcaatgtattggtgatgagtatataaagctgcttgagtagta
aagtcttgtgctatgaatgcataagaaggtaaagagtacatatgttgagctactaaggaagtaataacccctaaagaagctagagcaagacctaattgaaaatgaatc
gaattattgattgtgtcgtaaaggcccttatgcccacgccctaatcgacccccggaggagtatgtgcttctaaaagatctttgatactgtgcccaattccgaagttagt
tcgatacatatgaccggcaatgagaaaaataaatgcaatagctaaatgatgatgagcaatatcggtcagccacaaactttgtgtttgtggatggaatcccccaagaa
gagttagaatagcagttcccgctccttgagtggtaccaaataaatgattactcgaatcggggttttgggcataaagattccactgacccgtcagaaggggtcccaac
ccctgtggatagggtaatacatctaagaaattattccatcgaacgtactctcccctggatgctggaatagcgacatgaactaaatgtcctgtccaagccaaagaactta
ccccgaaaagtcctgacaaatgatgattgagacgagattccgcgtttttgaaccacgaaaggcttggtttccatttgggttgtagatgtaaccaacccccctattaagga
tagggtagaaagaaataatagaaaaagagctcctgtataaagatcttcattggtccgtaatccaattgtataccaccactgataaaccccagaataggcgatattcact
ggaccggcggcgcctcctcgagtaaaggcttccacagcgggttgaccaaaatgaggatcccaaatcgcatgagcaataggtcttacgtgtaaaggatcctgtatc
catgattcaaaatttccttgccaagcgacatgaaacagatttccggacgtccatagaaagattattgctaattgcccaaagtgagaagcaaaaatgttctgataaagac
gttcctcagtaatatcatcatgactttcgaaatcatgtgcggtagcaataccaaaccaaatacgacgagtagtggggtcctgagctaagccttggctaaacctgggaa
atcttaattccataatgcctttcaaatcctcctagccactatcctactgcaataattctcgctaagaagaatgcccatgttgtggcaattccacccagaaggtaatgggtt
actcctacagcacgtccttgtataatgctcaaggctctaggctgagtagcaggagcaacttttaatttgttatgagcccaaacgatagattcaatcagttcttgccaata
accacggccgctgaataaaaacattaaactgaaggcccagacaaaatgagcacctaagaaaaaaagaccatatgcggataatgaagaaccataagactgaatta
cttgggatgcctgtgcccacaagaaatctcggagccacccattaatcgtaatggaactctgtgcaaagtttccccctgtgatatgagttaccaccccttgatcacttata
gtaccccaaacatccgactgcattttccaactgaaatggaaaatgactaccgaaatggaattgtacatccagaatagacctaagaaaacatgatcccaggcggatac
ttgacatgttccccctcgcccaggcccatcgcaagggaagcgaaaaccaagatttgctttatcgggtatcaaacgggaactgcgagcaaataaaacacctttcaaa
agtattaatacagtcacatggattgtaaatgcgtgaatgtgatggactaaaaaatctgcggttcctaatggaataggtaacaaagcaactttgccgcctactgctacta
actcgccacctccccacgttaagctggtacttgttgttgcaccaggagctgttacgctaggcgcgccagcatggagattttgtacccattgagcaaagatcggttgta
attgtatggcggtatccgaaaacatatcttggggacgacctaaagcactcatggtatcattatgaatgtataaaccaaaactgtgaaaacctagaaatatacatacccca
gttaaggtgggatatgattgcatcacggtgtctaaggacacgatctaatagatcgttgtatcgagtcgttggatcatagtctcttaccataaaaatggctgcatgtgcag
cagcaccaactattagaaatccgccaatccacatgtggtgtgtgaacaaggaaagttgtgtaccatagtcagtagctaggtatggatagggggggcatagagtacat
atgatgagctacaacaatggttgtagagcctagcatagctaggttaagagataattgagcatgccatgacgttgttaggatttcatagagacctttatggccctgtcctg
taaatgggcctttatgagcctccaaaatatctttcaggccatgaccaataccccagttggtcctatacatatgacctgcgatcaggaaaagaatagcaatagctaaatg
atggtgcgcaatatcgctcaaccatagaccgccggttattggatctagccctccgcgaaaactaagaaattctgcgtatttggaccaattcaaggtgaaaaaagggg
ttgctccttcggcaaaactaggataaagttgagccaaaaggtcgcgattcaaaataaattcatgaggaagtggtatctccttaggatcaaccccagcgtcaagaaatt
ggttaatcggtaaagatacatggatttggtgccccgcccaagaaagagacccaagtcctaataaccctgctaagtgatgattcaacatggattctacgtcttggaacc
aggctaatttgggagcggctttgtgataatggaaccacccagcaaaaagcattaacgatgcaaaaatcaatgcaccgattgcggtacaatagagttgtaattcacta
gttattccggatgctcgccaaatctgaaaaaacccggaggttatttggattcctcggaaacccccgcctacatcaccattcaatatttcttgccctacgattggccaaac
tacctgagcactgggtccaatgtgagtaggatcacttagccatgcttcataattggaaaaacgggcaccatggaagtacatgccactcaaccaaagaaagataatg
gagagttgaccgaaatgagcactaaagacttttcgagagatctcctccaaatcacctgtatgactatcgaaatcgtgagcatcagcatgtaggttccagatccaagtg
gtagtatcagggcccttagctattgttcttgagaaatggccgggtctggcccattcctcaaaagatgtttttacaggatccctatccacaacaatttttacttctggttccg
gcgaacgaatcatcattaagtcctcctctttccggacaagacatacaaagagacccgccaactttttagtgaacctttgaaagatagatattatgattagtccttttcttta
ctatctaccgtccttctatttttttttagttattcactggagcaattatatattgaagtcaatctgaggcaagtgttcggatctattatgacataaggattgggtgcctaacgga
cttttttttatcttggatttctccacgtaacaaaaaaaacctttttttaatttaaaaagctagtctatttttttttaagagtataagtccctatctatatctactttccttgagcataatata
gatttttttattcgattccaaattccaagataactcattagaattattaataagatggtcctgatatattagcaatatttatattgccccttttttattcgctttattacttctattctag
accctatcgtttatccttatgaaatataatataaaatagaaggcagaggaaagagatataatgaaattcttgattcgatcttccgacctaatttatttgattaatggatcaac
aaccaaaccccccattttctgaaaaaggagagtggtcttattcaaattcaaagcgcttcgtaatcttcaaccagttctgtgcttcaatataatttcccggagtaagcgctat
agcttgtttccaatactcagcagcttgatcaaaccaagcttctgcaatttctgaatcaccctgtagaatggcctgttctccccggtcggaataggtagttccttcccctag
aaccgtacttgagagtttcctacctcatacggctcagaaattgctatcttaatttcccttgtcttaactgaattcgatttctcaaaaatcgatccaatttcttcttgggttacgc
agaagaagttaattacctaagtttcaaaccctaaattttgatcaataatcagtttgatcttttctcccacctgcagaaaaatgaagcatagatagacctatatccttcgtcc
gaattttctgaaaggtaactatctcggtttcatatatgaaatttctatagaatccttgaaaaagactttttccccataagcaagaaaaaaagaacttactatctttgggatctg
atactacaccgctgcttaatcccttagtggatcggctctattacataagcggattcctaaattttgccccatatcatgggataagtaagcagtttttttttagttgtatcgacc

cagtcggtcactaattgatctttacggtgctttctctatcaatttgagaactctatccatagagtagtataggccatactttctttcttcctattttgattctcgtgaagtgtcctt
tcttcctacagctgatagggcaaaatcgttgttttgacgatccctatgtagaaagccccctttctagtaaatactagaaaatttgatcctttcttatttttctttctatagtgga
gatagtcgcacgtaatgacagatcacggccatattattaaaagcttgtggtaagaaggggtttcgttctagtgcccggaaataatattccaaagcctttgtatgctctcc
attgcttgtgtgtataaggcctatgttatagagtatataacttcgatcatagggatcaatttctagtcgcgtagcttcataataattttgcaaagcttccgcataatttccttcg
gattgagccaacatccgttacggtcgttcgttctattcaaaaaatctccgttccaaaaccgtacatgaggttttcacctcatacggctcctcccttctgtacatagtactaa
gcgaaaaaatctagagaataaaaatagaattagttccatctcattatggaccgaaaggggctggtatttttccaagaaatctctagccaaccttcccacaagaggttttt
cttaacaccaatgaattctattaatgctagaggaaaacgatagctccaagaatttctttgttctcaacgcctcctatttagaggaattagccacttcaacgatctttgatgg
ttataggggtatccaaagtacaaacttgatggttgtttgttatcccaaccattcttcccagccctgataccaatcaggaaagggttaatttctaacaaagtttttctcttgttg
attcctatttctaggtgtagtgcttttatcccctatgctacctattagtactagtagagtaggattagcctgtaatacagaacctatcctgtaggtgtaacctttcgctcaata
ctaaaatctacaattgaagcatccgaggccgcgtcagtcgaggatacacgacagaaggagttgttagttcacctcaccttccctaagcgtgggtttcccttactaattt
ggttttctctccgcgaacccccactctttctcgtaaaaccggggtgtaggtagggctaaaaaaacaaaaaaaagagtcaaatcgcaccatctctataataagtaaatg
cccttttttccccggaggttgtcggaattattcgcaataaaatattggctacaattgagaaggtcttatcaatgaaatttccatttatacgggatctaggcataattcccaa
cccattctatcattctatatagaattcttttcattccttcacaaaataacataaaaaaaaacaaatccattcaattcttataaatcgatccctatgctccaaatggataagaga
ggtatttctgctcagcccaaattctctctttttccttctgtttgaacaagaagagatagaaaatatttgactaagattggatttcattccactttcctatttcttttctcaacaaca
acttctctcatcaactatttcgcattttcaaagtcattaatcgtcccatacccctattttctatttgattgtatggggtaggataccttatgcaaacagaattctagggttccttttt
tatcgaataagaagaattcttccattctctttttctttggtttggggaaaacccaaactaaaacttttcgagggagcggaattcctagtaaaaaaatcctggacccgtcca
cttagatgaaaaggaatttttctatctaatagaacaatggaactctcgcgcggttgtggttgtacctgtactgcaggaataggaaaactcgctattcactcagttttttttc
cataataagattatggaggagagatggccgagcggttcaaggcgtagcattggaactgctatgtagacttttgtttaccgagggttcgaatccctctctttccgtttttct
taattcagcaacgttaatgattacaatgtatcaaatcaaatgacaatttattccagcaataatacaatacctttatttaatagaaattctctataccaaattactatggtatgta
aaatacacatagaggaaagaacaaaaaacaaaaaggaatcctagggttaatccatttctgttaggtgaatgggaaaatacgaattaagagccttaggtcgatttagtt
cggggaaaggggaaggggaagaaaattctatgaacctttccgtttttccttaagttcaagtttgacgagagtaatattctacaactaacaactcatttattttgagaccg
acccacttcctatccaggattttttttactagtcctttatattgcaatgtgtcaatcgtcaaatgctttggcaatttccccgggtcggatgaagcaatagaattttgaaccag
acgttttgatctttggttatccttcgtagtaataatatctcggggtttgcaacgaaaacttggtatatcgactatacgaccattaactaaaatatgtctatggttaactaattg
ccgggcctcgggaatggttgaagccatacctaatcgaaaaaggatattatccaaacgcatttcaagtaattgtagtaaaacctgacctgttgaactttttgcttttccag
cgatatgtacatatctaagtaattgtcgttctgtcagaccataatgaaaacgcaatttctgtttttcttgaagacgaatacgatattgttctttttttcccagaatggaatttcttt
ttcagattacttccggatttaggtgtttttctagtgagtcctggtaaagctcccagacggcgtatttttttaaaacgaggtcctcgataacgggacatgaagactcctttttt
attgaaatttcattttacacaattaatttcattgtatttacattacagaatacatcgaaattaaaactgaattaaagtaaaggataaacagagtaaaatctactaaaagtacc
acaaaaaaatggaatttcatcaacatctgaatttttgtatatatattatttattttattgttttgtatctagcaaaattgtagggtagaacgacataatagatcctggcttctcca
tttaattcggagaaaaagaggtatttttgttcatggaacattgatagagaaaaaagccgactatcggatttgaaccgatgaccctcgcattacaaatgcgatgctctaa
cctctgagctaagtgggcttacataacagaaatagtgtaacaaatagaaatatgtatagtataggaaatccgtaaaatgtcagatcttaattattaatcttagctattaact
agttcgaaattggaagttctacttagaaaaaaatactagaacttcataaaataaagttagatagattttttttgaactttctttttttctctaattcgcaaatctatttttctaataga
atctattccaatttctatattgaatttgattttagatatttttaatttgatatggctcggacgaataatctaatacatggaaaagaataaataatatatatacgaaaacataataa
agagaacatgcgaatttcttgtattttcagtccatcattatagacatttttgagatattttgtttttttgttatttgctaataatttaatgattaatatttcactaaggagaacata
gaatcatagcaaatgaaattgctaattctgattagcaaaaaaaaagaatgaatatcaagcgttatagtatgattttgaatactctaaaaaaggaacgcggtaggtgggg
gagagaaaaaccttgggatatattgattcgcattgaattgcaaatacatcaacgatagaatcaattcaatgctgaattgcaataagcggagtctctcaactagagacg
aaccgctagactacatagagtaatgaattcaacgattcaaaaaaaactaacagatggaggaaattgcacaaggaatcctggtctcaaagaaaaagaaaatggggat
atggcgaaatcggtagacgctacggacttgattgtattgagccttggtatggaaacctgctaagtggcaacttccaaattcagagaaaccctggaattaaaaaaggg
gcaatcctgagccaaatccatgttttgagaaaacaagcggttctcgaactagaacccaaaggaaaaggataggtgcagagactcaatggaagctgttctaacgaat
cgagttaattacgttgtgttgctagcggaactcccttctaaattagggaaagaagggctttcgaaatctaatacacacgtatagatactggcatagcaaacgattaatc
acagaactcatatcataatataggttctttaattctttttaaaatgaaaataggaatgattatgaaatagaaaattcataattttttagaattgttgtgaatccattccaatcg
aatattgagtaatcaaatccttcaattcatagttttcgaaatctttttaaaagcggattaatcggacgaggataaagagagagtcccattctacatgtcaatactgacaac
aatgaaatttctagtaaaaggaaaatccgtcgactttctaagtcgtgagggttcaagtccctctatccccaaaccctcttttattccctaactctagtatttatcctgtttttttt
attaataggtttaagattcaatggaatacatttctttttttattatagtatcggcaaggaatgtcgattattaactcgatatttaaatattattaaataggctttctttgtacaatgc
ataggactgccccctccccatttccaaattttggatattgacatagatacaaatactctactaggatgatgcacaagaaaaggtcaggatagctcagttggtagagca
gaggactgaaaatcctcgtgtcaccagttcaaatctggttcctggcacagaaaaaagggatcttccgaataggtattgatacaaatacctcgagatgggttgggata
catattcgttaataatatagatagagtatgattttttcatctaagtagataaatctctaaatagaggcacttctttttctgcatttttgcatttcttaattttctattccgctattccg
acaaatattttttttattcttgcttatcttatcttactttcctagttgttctaagtaatgcacgcggtacaaagttcgtggtagggaacttctttgagtcatcatatttttctgttcata
cgaaggaaatgaatatgtgattttccaacgaaatgaagcccttttttgcttagtctatctgaaccttttgtataattggaattaattagaatgtaataggtattctgtttcatct
aggaacagagcgtaaaaagattccttgacttgcataaaatctggagttgtgttgtataagtgagcatgaatttcttatcattcaatgagcatcttgtatttcatagaaattg
ggggttatatagtccttacgtaagggccagcctatccaactttcaggcattaggatacgtttaaggcgtggatgattatcataagagattcccaccatatcaaaagattc
gcgttcttgaaaatcggaacttctccaaatccagaagacagatgggattctaggattatccttttgggcaaagacttttatgcatacttcttctgggttatctataccatact
gtattctcgtaagatgatacacgctagctaaagatccaccgggtgcaacgtcataagcacattgggaacgtaaataattgtaaccatatacatataaaatgacagcaa
tggaatcccaatcctctgcttttatttgcaaagtctctattcctcgatgatcgaagcccaaagatctatgaaccacctcatgtttgactagccaattagataaccaaccct
gctgcattatcttgatctctcctcctttgtataaatatttcgcagttcgaatgcaagtttgaaatattgccctgctctttctttttcggcacaaagagctcctcctaattcactaa
tttgtaggaagatactggacttttggatttgaaaaaagtttcagaagatatgtctaaagtagatggtgattgatagagcaattcttgctcataagttccagtattagtactg
cgccgaacataaagcttgtggctggtagtaaaacatcgatttttcttttgagatagagttcgatcctcaactatttctcgcgatatcttcttacgaagttttgttagggcatct

ataacagcctctggtttaggtgggcaacccggcaagtagacgtccacaggaattaacttatcaactccccgaacagtactataggaatccgtactgaacattccacc
agtaatagtacaagctcccatagcaatgacgtattttggttcaggcatttgctcatataatctcactaaagagggagccattttcattgttactgtaccggctgttaaaatt
aggtccgcttgcctaggacttgatcttggtaccaatccataacgatcaaagtcgaatcgtgagcctattaatgaagcaaattcaatgaaacaacaactggtaccatata
gaaggggccataaactagagagtcttgaccaattcgaaagatcttttagtgtagttgaaataacggaattggaacttgtttggtcaagtaggggaaactcaatcaaact
cataactgtcttaatggaatcttttccttcttttttttttttgtctgaatattcagttaagaccattccaaggctccttttcgccatgcataaactaaaccaacaactaggataag
cacgaaaatgaaagcttcgataaaaaacggatatacccaatacgtcgaaactcattgcccaagggtagagaaagaccgtttccacatcaaaaacaacaaaaactag
cgcaaacatgtaatagcgtattcggaattgtaaccaagccccccccatgggttctatacccgattcataactagaaagcttctctggtccttcacgaaccggagctaa
aagtgctgaaatccaaaatgctaaaataggaataaggcttgctattattagaaatgcccaaaaaatatcatattcgtgaagcagaaacataaatgtactcccattaatgt
ggaataggcggaactgaattagtcaattcaagtcagcattgtcaatttatacagaatttctctcttttcctcggtgaaacaaggatcggtttttctcaaaccaaagggctt
agtttagcctttgtttcctctttgccacgtcttctttaaagattcatccaatggaatcccgactccctttctttttgatttcctttctatttaggtatggtggagacataattcttat
agaaacaaaactctctcgcttcactttgtctcattttctctagaatctctagaaaaaggaataaaaacgaaaatactacgaattagagcctaattaagataggatgactaa
tgtatgcagcctaatgaggagtaattctataaaaataaagaactctatttcagaacgtagattgatttagatttaggtaatctatagatatagataagcaaagtaatatactt
caaacaaagtaggaatttgcaagatggagaacatcttgcagttgatttgatagaaattcatttttcttttcctgtctctataattttcgatgaatgagcctctggtaatcctttt
atctctattttatggcgcaggcgcctgtccagtctataaacaagtactaatagggaaatgaaaactatactaaaggaaacgtaggatatctctcctaaaatctaaaaaa
ggacatattagggctatacggattcgaaccgtagaccttctcggtaaaacagatcaaacggattattatcgaaatgattcgaactgtttcaaagacccaacatgcatttt
tttgcattgggctcttttatcaactgatagaaagatcagttagtccaccatagtttttctttacggaaagataatgagatggctccctgcgctctgattgattatttgtattatg
atctatctaagagcaataccaaagtgtttcaaaggaggattaccttgacttaggtctgcctccggcctaaattaaatcaacctaagtgaaatagagtctctatcgttccg
ctacaagagttgactatgagacttcatacaccttaaagttcatagaacgaaaagaattttttttggaggcccttatcctcattaagcctagcatttagtgggctggatattta
ccttatcaactagcaaatcaataaaggttctatttgtttaggcacctggattggtacctgaatcggactgaaccaactatttgtcaggcgactgttctcctattctctcgaa
tccatgaagtaagacattgattttgcaagaagatccactatgttcattgcataataagctcccttgaaaagcattggcgcacgtgtaaacgagttgctctaccgaactga
gctatagcccttgtcagagatatcttaatatatagagaatttcttgtcaagatgaatattctctaatagtagaggatatcctttgatctgtttactataataacataccaataa
cgaagcggtatttgcttataaaaaggattcgatctataatcgatcgaagtaaagggtcttcctttgtggtgataaattgcctacttaactcagtggttagagtattgctttca
tacggcgggagtcattggttcaaatccaatagtaggtaggtaggtagaaaaattactagatagcattggccctacttcgcttcgctatctaataattttttctacccctctt
ccctttttctttgtatcaactaaaccgttgggttgtcttcaattagatgggggaatccaattaacagcctcgactcgtatcctagctcgtctgagagctaccttcgcttcaa
ccaattctttcgtaccctcagctctactcacgttagcttcggctatttcaagtgcctgttgagcttcttccggatcaatgtcactacccagttccgcatcatttcctaaaatg
atgatctcattattaactattctggcaaaaccgctccacagaaccgccgttaaccattgatcgttgaggaggcgtattctcaagggacccatatctacagctgtgttaat
gggggcgtggtttggtaatacgccaatttggccactattagtagataaaatgatttctttcacttcacaatcccaaataattcgcttaggagtcagtacataaagatttaat
ttcatttcttcaatttgttctcctcttctaagtttatagctttcgtgctagcttcatcgatgttacccaccaaataaaaagcctgttcgggtaggccgtctaattctccggaaa
ggattagttgaaatcccctaatagtttctgcaagaccaacatactttcccggagaaccggtaaaaacttctgccacaaaaaacggttgtgataagaagcgctcaatttt
tcgtgctcttgctacagttaaacgatcctcctccgataattcatccaacccaagaattgcgataatgtcctgaagttctttgtaacgttgtaaagtttgcttaactctttgcg
cagtttcataatgttcgttgccaacgatccgaggttgtaacatagttgaggttgaatctaaaggatctactgcaggataaatccctttggaagctaatcctctggaaagt
acggtagtagcatccaaatgtgcaaatgttgtagcaggagcagggtcggtcaaatcgtccgcaggtacataaaccgcttggatcgaagttatagatccctttttagta
gaagtaattctttcttgcaaagaacccatttctgtactaagagtaggttgataacccactgcagagggcattctccctaataaggcagatacctccgatcctgcttgaac
aaaacgaaagatattatcgatgaatagaagcacgtcttgcttattaacatctcggaaatattctgccatagttagggcagtcaaaccaactctcatacgagctcctggc
ggttcattcatttggccatagactagagctacctttgattcctcaagatttttttcattaattactccagattccttcatttccatataaagatcatttccttcacgagtccgttcc
cctactccgccaaatacggatacgcccccgtgagctttagcaatattgttgattaattccatgatgagtactgttttacctactccagctcccccaaatagtccgatttttc
ctccacgccgataaggagctaaaagatcgaccaccttaataccagtttcaaagatggataatttcgtatctaactcgataaaggcgggcgcggatctatgaataggg
aatgttgcactagtatctacaggacccaaattgtcaacaggctccccaagaacgttgaaaattcgtccaagagtagctccaccgacaggaacactgagaggagctc
ccgtgtcaatcacttccattcctctcatcaacccatctgtagcactcatagctacagctctaactcgattatttcctaataattgttgtacctcacaagttacattaatttgctt
accgtcagtgtctcgactcttgactaccaaagcattataaatataaggtaacttgcccgggggaaaagtgacatccagcacgggtccaataatttgatcgatacgccc
tgtactttttttcttcaattgtagaaaccccgggacgagaagtagtaggattggttctcataattatcacataattttcaaaaaaaaaggaatttatcgaaattttgattttttct
tgttgaataatgccaaatcaacaccaaaaaaatatccaaaaatccaaaagtcaaaaggaaatgaattagttaattcaataagagagaaaaggggaccagcacttgattt
cgttgcccaaacgaatcccattcaatcgtttactcatggaatgagtccgtcggaaagttcaatcaatctttttttcatatacattttgccttttgtaaacgatttgtgcctactc
tactttcttatctaggacttcgatatacaaaatatatactactgtgaagcatagattgctgtcaacagagaattttcgtagtatttaggtatttccactcaaaataagaaaag
ggggtctattaagaacttaataaggattagaagttgatttggggttgcgctatatctattaaagagtatacaataaagatggatttggtgaatcaaatccatggtttaataa
cgaagcatgttaacttaccataacaacaactcaattcttatcgaattcctatagtagaattcctatagcatagaatgtacacagggtgtacccattatatatgaatgaaac
atattatatgaatgaaacatattcattaacttaagcatgccccccattttctttaatgagttgatattaattgaatatcttttttttaagatttttgcaaaggtttcatttacgcctaa
tccatatcgagtagaccctgtcgttgtgagaattcttaattcatgagttgtagggagggacgtatgtcaccacaaacagaaactaaagcaagtgttggatttaaagctg
gtgttaaggattataaattgacttactacaccccggagtacgaaaccaaggacactgatatcttggcagcattccgagtaactcctcagccgggggttccgcccgaa
gaagcaggggctgcagtagctgccgaatcttctactggtacatggacaactgtttggactgatggacttaccagtcttgatcgttacaaaggccgatgctatcacatc
gagcccgttgttggggaggataatcaatatatcgcttatgtagcttatccattagacctatttgaagagggttctgttactaacatgtttacttccattgtgggtaacgtattt
ggtttcaaagccctacgcgctctacgtctggaggatctgcgaattcccccctacttattcaaaaactttccaaggtccgcctcatggtatccaagttgaaagggataagt
tgaacaaatacggtcgtcctttattgggatgtactattaaaccaaaattgggattatctgcaaaaaattatggtagagcatgttatgagtgtctacgcggtggacttgattt
taccaaagatgatgaaaacgtaaactcacaaccatttatgcgttggagggaccgttttgtcttttgtgccgaagctatttataaatcacaggccgaaaccggtgaaatt
aaggggcattacttgaatgcgactgcaggtacatgcgaagaaatgattaaaagagctgtatttgcgagggaattaggggttcctattgtaatgcatgactacttaacc
gggggattcaccgcaaatactagtttggctcattattgccgcgacaacggcctacttcttcacattcaccgagcaatgcatgcagttattgatagacagaaaaatcatg

gtatgcatttccgtgtattagctaaagcattgcgtatgtctgggggagatcatatccacgctggtacagtagtaggtaagttagaaggggaacgcgaaatgactttag
gttttgttgatttattgcgcgatgattttattgaaaaagatcgtgctcgcggtatctttttcactcaggactgggtatccatgccaggtgttataccggtggcttcaggggg
tattcatgtttggcatatgccagctctgaccgaaatctttggagatgattctgtattgcaatttggtggaggaactttaggacatccttggggtaatgcacctggtgcagc
agctaatcgggtggctttagaagcctgtgtacaagctcgtaacgaagggcgcgatcttgctcgtgaaggtaatgaaattatccgatcagcttgcaaatggagtcctg
aactagccgcagcttgtgaaatatggaaagcgatcaaattcgagttcgagccggtagataaactagatagctagactaagtggataaaattagatagaaaaaaggt
ctaaataaaaaagaagagaaatagaaagatcaaaaatcagttacgaaaatgcagtaattcttctttttcttctaattgattgcaattaaactcggctcaatctttttcagat
tgagccgagtttaaatagattttgatacgatcatgagacttgacaaatcgggattcctctattctatatatttagaagatataaaggtataatacaataaataaatacaaat
atagtattatcatatgataatggaatcaaatacgcagtatttacagaaaaagttttcatttattgggaaagaatcaatgacatacaatgcattacagacgtatgatcattac
cctttaaccgggttattctattccacttctagatagagaaaaaaactaaaggagaatgaatgaaaaaagacagagtttggaagttagaccccttttctaagactctctttc
aaaaaagaggacattttgaaacttttaacaggcacaatcgtgagtcaacaagtgactcgaaatgctcgtaagaaaagagaattgattttcaaaatggtagaactagat
gacgaagttttctataaccttgatgaagaggtagttttagtttacgactccgatcaagagcgagaagtctttcatttcagattggactgctatagaatctggacccatcgt
agagacgttcaaaaggctcctgatgataagaatcatactttcgcggaactccatggggctatgggcttcaacgcggtagacgttttgttccgaatttttctggagcaaa
cctccgacccaacgatacaatgaatttttctattcacaaagaaaaaagattcggaatcgcaatgctactatacgcacccggaggaatattcggaattatattcttatata
atccatttttgcgcggggtcttactaacaggacttcgctgcacgagacgaggattcgtcgaaaagctaaggtcatccggtattttcataccctttcctttgggggtggta
tatggccttacaaagtctaaggggtagtatgagatctgtagtaggtaaaagaatttgtcccttgattgagtatgctattttccctcctttaccgcgcattattgtatatgcttc
tagaagagcacgtatgcagagaggaaattacagtttaataaaaaagcctaaaaaagtttcaactttacggcaatatcaatcaactaaaagtcctatgtatcaatccttcc
agcggatttgtggggtccgagagtggttgaataagtattgcatgtggaaggaagtagacgaaaaagattttggattcgaaataggcgcattcgactaagtcgtacttt
gaatccaatttcaagttcgattagaaggatagaaaggccgcgaggatcggaaaagaaaaatcaaatctttttaattgcttctcctttttgctattttcttattatttatataat
ccattcgattcttttttttagaatactaaagtattctaaaaaaaagtattctataaaaaatcttttttgcaaactcaaaaatacaatagtcaatattcgttataatagatatactt
aattatatcataagaatcttaagatatttttgaatagatcaaatcgaatagatagaaatagtaaatttgaatggagacacctattctatgatggattttaacttaccctctatt
ttcgtgcctttagtaggcttagtatttccggcaattgcaatggcttctttatttctttatgtgcagaaaaataagattgtctagaaccgacgggaccgaattttctcaatgtat
ttccacacaggatcataatacggatcttttgtagtgtaagtaatataatatggtatgttatgtggctctttctacacacaaatgcaaacccgctatggatggggattatgga
tgcggatataggctacgagcataaatgcatgcatatgcggaaccgggtatagcgagtttttttaagtggatcaacaaatactttttgaatagaaagtcaatgtatctaac
caattattttacaggagtatctagttggcgaaggcgatttcagaatcaaaaaaagtaaagtcaaaatcatttagcttattctctcaatttcaatcgaccgctgttagtatatc
taatatgaattggcgatcagaacacatatggatagaacttctaaaaggttctcgaaaaagaggtaatttttctgggcctgtattctttttctaggttcactaggattcttag
cggttggggcttccagttatcttggtaagaatattatatctgtacttccatctcaacaaattcttttttttccacagggggtcgtgatgtctttctacggaatcgcaggcctat
tcattagcgcttacttgtggtgcactattttgtggaatgtaggcagtggttatgaccgattcgatagaaaagagggagtagtgtgcatttttcgttggggattccctggaa
taaaacgtcgcgtcttccttcgattccttatgcgggatatccaatcaattagaattcaggttaaagaaggtctttttcctcgtcgtatcctttatatggaaatccggggcca
gggggccattcccttgactcgtactgatgagaagtttttttactccacgagaaattgaacaaaaagctgccgaattggcttatttcttgcgcataccaatggaagtattttg
agtaccaattgaatttttgaaatgaattgaatgaagaagaattggaagaagaaaagttttctcaacacgagggaaaaggtcccttcgaaattgcattattgtaagggg
attttgagtatttatctaaaggaaggaacaaatgaggataagagaaaattgcttctaatttgtccaagtgggatatatggcgtaatattcttccattttcatccgaaaggac
ttttttctatttctctattccactccatctagatctaagaaagaactcaatgcaatgaaattctactagtatagaaaaaagaggaatagatacaaggtctcaaaccttgttat
agaatttttccttcaaagaaaaagaaatatcatatagagtcagcgaataaagcggattcattaacaactcaatttacagatcaaaaatgaaaaaaaagaaagcattgcc
ttctttcctatatcttgtatttatcgtacttttgccttggggagtctctttctcttttaacaaatgtctggaactttggattaagaattggtggaataccaggcaatcccaaactc
tcttaactgctattcaagagaaaagagttctagaaagattcatggaattagaagacctttttatcttggacgaaatgataaaagagaaaccgaatacacatgtacaaaa
ccccccctataggaatacgcaaggaaataatacaattggccaaaatagataatgagggtcatctccatatcattttgcatttctcgacaaatataatctgtttggctattcta
agtggttcttttttctgggtaaagaggaacttgttattttgaattcttgggttcaggaattcttctataacttaaatgactcagtaaaagcttttttattcttttagttactgatttt
tttgttggatttcactccacccgcggttgggaactactaattcgttgggtctataacgatcttggatgggttcctaacgagctaattttcactatttttgtttgtagttttcctgt
gattctagatacatgtttgaaattttgggtcttttttttgtttaaaccgcctatctccttcgcttgtagtcatttatcattcaattagtgaagcataaactcatttgatctcctgatat
taatcaaaattagcatcctttttcctttagaaagaaagcctttttccattttagcaaaattcttttttttctatttctacctgctcaaggtattcatcattccagtacaactgttgcag
tagaatgacaacagattcgtgtatagggaactagattagcttagctacctatctaatttattgtagaaattccgggatctgtgattggacatggaaaatagaaatacttttt
cttgggtaaaggaacagatgactcgatcgatttctgtatcgatcatgatatacgtaataactcggacatctatttcaaatgcatatcccatttttgcgcagcaaggttatg
aaaacccacgagaagcaactggacgaattgtatgtgccaattgccatttagcgaataagcctgtggatattgaagttccccaagcagtgcttcccgatactgtatttga
agcagttcttcgaattccttatgatatgcaactgaaacaagttcttgctaatgggaaaaagggagggttgaatgtgggtgctgttcttattttgcccgagggattcgaatt
agcgccgcccgaccgtatttctcctgagttgaaagaaaagataggaaatctctcttttcagagttatcgtcccaataaaaaaaatattcttgtgataggccctgttcccg
gtaagaaatatagtgaaatcgtctttcccattctttcccccgaccctgctatgaagaaagacgttcatttcttaaaatatcccatatatgtgggggggaaaccgaggaag
gggacagatctatcctgatggtagcaagagtaacaatacggtctataatgcaacgtcaacaggtgtagtaagaaaaatactgcgtaaagaaaagggggggatatga
aatatccatagtcgatgcatcggatggacgccaagtgattgatcttatacctcccggtctagaacttcttgtttcagagggggaatcgatcaagcttgatcaaccattaa
caagtaatcctaatgtgggagggtttggtcagggggatgcagaaatagtgcttcaggatccattacgcgttcaaggccttttgttcttcttcgcatccgttattttggcac
aagttttttttggttctcaaaaagaaacagtttgaaaaggttcaattgtacgaaatgaatttctaggtcccgggatttcttaccatcaagttggtaaaaagccgcgatttatt
ggcgattgctagaattctctatgatccattttggaaatcttttttttgtttagactatttttttgtttgcgagatgtcttgtctgcaattccttatttctatcatctcatgcaaaacga
ggagaatgcaaggcaagggcgagaacaataaaaggaacaaatccttttaggagggattgctggtcttcttaatcctctattgggcacaagaaaaaggctttttttgcct
ttttcttgtgtcgattcttcttgtatcgaaatatgaatctttttttcttcctattcggcaaagattactatttcttctttatttgggtctgtctcttgaacctcttttgcttaggttcaggc
gatgtagtggacaaaaagagggaaagaaaatatggcgggggacaaatttttttgtgaccaaatagaattgcttgacttgttcaattaagttcaatttagaacttacagaa
attttgcaaaaaaaacggatactcttccattgaaggctgttttttatttttaggctagttgagtagttttaattaaggttttattagtttattactctaaattaaatcaatgatttac
aagagacttcctccggggaataaaatattggatcctcgattgatcctttctttctcctcgcttcataaaagtgaattaatttcattggcgaggggggttataaatcaactgat

ggattacttcactaacattattaacaaacaaaattaacaaacaaaacgaataaatagagggattctgaccatcagagcaaaggctttctctttgttattttacaaatcaaa
ataggaaacccgtttgtaggttatggaatagattaaaaaagtcgggttataagagtaagaattccgcgggtcctttccgctctaatcagataaaagggggtaaggacc
cgctaagttcctatttttcatgtttacaacctggtccctccaattactatagagatgaacccaatccagaatatgaaccgtaaaagaaaacacctattaaaccaatcaca
gcaataccggttacagtacctatcagccaaagaggaattcttccagtagtatcggccatttcccctactttcctccacattttatcaagtggtcatgctagagacaaaaa
cagtcatggatagttataaggatggtatccttccaaatggggataagagaattcttactactctctttctttctctcaattgaagaagtaattggaaaataaaacagcaagt
acaaaaatgagtaataaaccccagtatagactggtacgattcaattcaacattttgttcattcgggtttgattgtgtcatagttctatagttggaatttggtttatcgttggat
gaactgcattgctgatattgatcccaagaaaaaaacagtaggtacagctagtccgtgaacagccagccatcgcactgtaaaaataggataggttcgatctatggtca
ttgagggcctcctaaaaggatctactaaattcatcgagttgttctaaagaatcaaaacggtcggttattaacggaattccttgtcggctttctgtgaaatactcgtttggcc
gaggacttccaaacacgtcataagctaaacccgtactgacaaataaccaacccgcaatgaatagggaaggtatagtaatgctatgaataacccagtatcgaatactg
gtaataatatcagcaaaagaacgttctcccgtgcttccagacatgctgagctccccaaatttttgtacattcaaaaaaggaattgattccgtaaaagatgggatcaacc
agtaaatagaaaattactgatatttcatccttgtgagattgtcaattttgtaccaaaggtgtattttgagtataccaaattagtatagctatccttcctatggcacagcaatcc
tgtttcgcttggtctcgaaacagaattcttttttctcttctttgttccttgtctatagggtaagctatatggtattcaaggcatcaatagaaaacctcaattttgagggtcctac
ttaattttcaccggcttcggaatagtagaataattcggaatagggctcaagatcttgggaaaatctaagttaatgatcaacaggtttggataaagaatttaggaaagata
ttcttatactgacacaatacaaagagaagtagatgcgaaagctatcccttcgaatccaacctttccccttaaagaatttaattggttagcataatataatatctaataaata
gaaaatcaaatagtagataatctgttatgaaagagagaaaacattctttgaagaatcaagattcgtaatcaacccttgccttgtttactaactttcttgaccaaactgcaa
gcgtggaactttgccagttcttaggtttccgaaatagtgtaatggaaaaagaagtgcttcgaatcgactttgattggggttcgaaaaaggaattaaaaaaaagtaaatt
caggaaagatttcctttttttaggagaccctcgaggggtcgtggaatgcttttcttctcctcttattccatatggaatacaatcaattaaaataagaaggaataggggaat
attcgactgttcgctccaaaaagaaggttaaatcatcctattgaaaaagaccaaaatagaaagaactttttcaaattcaattctttatttttcactggggttagctgatctag
ttcttaatatttttactttactcaattgacagattagacagcaaatctcttgattcggaattagggactcatgccccatctgatgaatccattttcttttagacttctgtatctca
ctctatcttgtttttttagtattatctaaaataaccgatgaattatgaattttccataacttaggtaagtgctttaccaacatatgtagtgtagtaaaaaaaatggaatttaaccct
ttcatgcttactataactagttatttcggttttctattggctgctttaactctaaccctagctctatttattggcttgaacaagatacgtcttatttgaaatgaattgaatgaataa
ggcagaaaagaaaaaatctctcttttggattcctggtattctacgcactaattaccaattcttttttttcttggtcattgagattcgtggataatttagactactatttagggata
aatcgtacctcttttttttatcccctcgaacaaatcgaaatgattgaagtttttctatttggaatcgtcttaggcctaattcctattactttagcgggattattcgtgactgcgta
tttgcaatacagacgtggggatcagttggatctttgattgagtaatatttctttttttgattgccctcctctctggtctggaggagggcaaattggaattgcaatttgttttgtta
agttattttgccctgcttcgacataagatagatggaatcacgctctgtaggatttgaacctacgacatcgggttttggagacccgcgttctaccgaactgaactaagag
cgctttcaaaaatcaaatccttttctactcctaatgtgtctcacgtacgtatagtatccacaaattaaagttatacccgctttaatcgacctcctcactactgcctataaaga
agaaagaagtaataggtagggatgacaggatttgaacctgtgacattttgtacccaaaacaaacgcgctaccaagctgcgctacatcccttttccaaattgttgtaca
atgtcattgtacacaattcctatcttgttttccacatcctaattttcttgtctttttctatctatagagaatcctcgtgtctatagagaatcctcgtgtcatttcttctttttggtctca
tataatcaaggacaaggaatggtatatatctaaatccaatctaatttcacctataaaagaaagattactattccttggtaatctataggaagggattgtctttttcgtttttagt
tcggaatttcgcctaaacaaaagaaatacaaatgatcttgggcaataatatctgatcatatacgtattccaataaggaaggaggattttcaatgcgggatataaaaaca
tatctctctgtagcacccgtgctaagtactctatggtttggggctttagcaggtttattgatagaaattaatcgtttatttccagatgctttgtcattcccctttttttcattctagt
tgttgctatgtgaggaatagagttcttcgtgacatgataaaaaaattcccacttttcaatccctttttagtataggaaagaaaaagaaagaaaagatagattgggttggac
cttagagtcatgaaaaatttggtaaatctcatttttgaaaaaagaaattcaattaaaagcagtatccaagctaagtcaggcctcagaaatcagagcatagaaagaggct
gggttggctactaaaatgaaaggattttgcttcaaaatccttgctaatttgacaaggattgtattccttaattatttctctattttttattacttaattgaaaaatttccaaaaattt
ttattctaatggattttcttcttcctcttcggtttcaaaatagaggaataaaagaataagtagaagaattaagttaagtcaatccaaaaaggaaaggaggttcatggccaa
ggggaaagatgttagaatcagagttattttgcaatgtgtgagttgtgttcgaaaaggggccaatgaggagtcggcagggatttctagatatagtactcaaaagaatcg
ccacaatacacccggacaattagaattaagaaaattttgtcgttattgtcgtaagcatacgattcatgccgaaataaagaaataggagcatcttgtgttcgatctttccaa
agatcaaaaaaaataagaacttcctatttaatatatagagcatagatagaatacaaaatacaaatcaacttgtctgatttccattagatattatttcatatgtatcgagggta
ttcatctaatatatggaccaaagagagactatttcttctggatccaaaattaataaaataaacaaatcaatttttttcaattttaaaataaggaataaatcatgtatacatctaa
acaaccctttcataaatccaaacaaacttttcataaatccaagcaaacttttcgtaaatccaagcaaacttttcgtaaattcaaacaaccttttcgcaaacccaaacaacc
ttttcgtaggcgtcctcggattggcccgggggatcgaattgattatagaaacatgagtttaattaatcgatttattagtgaacaaggaaaaatattatcgagacgaataa
atagattaaccttgaaacaacaacgattaattactcttgctataaaacaggctcgtattttatctttcttaccatttcgtaactatgagaatgagaaacaatttcaagcccag
tcaatttcaataattacaggtcctagacccagaaaaaatagacatattcctccattaacacaaaagttcaattccaatcgaaacttaagaaactccaaccagactttaag
aaacaacaatcggaacttaagttccgattgttgatgttttattcgaaagggccagactatatataaagaaagtaatccaatttagattcttgggtttgttataagaaagaac
aatggggaagaaaaaatagtttttttatttattgcaacatgctcgttgattcctaccacttaatcttaatttattgtatcttcccggagttacctctccgggaattctttttttaatt
attcctgtatattactttttttatccctttaattgataatctttattttattggaaatcgtgtaaagattatttggatttaatacagctacttgtgcaaggattttacgattaagaatca
attctttcttgtaaagattgtgtattaatttactataattatcgaatactttatgtatccgcgttgctgcgtttatccgactgatccacaaacgccgaaaatccctcttttgcctg
actctatctcgatgagaggaaacaaacgctcttcttacctgttgagtaatcattcgattaagtcttaaatgagcccctctaaagtttgaggcaaatgaacgcatttttgcc
cgtcgtctccgagctatatatcctcgcggaactctggtcattgaatcaaattaaccttaatgaataactaatgatttctcttcttttagccatcctttttcccattaataacaaa
acgaattattcccatatataaaatattagttccaatggcttttgctactataaccttcccaaccacaattttctcttctactcccttcagttatttcgcacgatactaaaaaata
gtgggttccatcgtttctatggttcccttttaaacggcgaggccctctctatacaccggagccctttctttcatttcatcaaagggtattgtgaacttgtatagttcacattct
ttggctctacctatccattatagagtaaatagctcttttcacaataagagttatccatacagtgacggcatttaattatgaaagttggctaagtagctgaccctcttagtcc
gtttttttaagataaaggagcataagcctttatctttttattactatttcctccgcttaatgaatagccgtttgctaccaatgggggaattgcttcttatttccaatctagatgatt
ggatttgcaccaaaggaaaccagaaattccatataccgtagaaatctaggatagagaagctctatcctattcattggtaccgatcatggatacttcaaaaattgtcttatt
tgtttgaacccatgatccgaacgagtcgcacatacaccctagcacatgttcctcgacgctgaggacatcccttaagcgcggccgattttctagcatttcgtattggctg
tcttgcgtttctaataagttgtttaaccgttggcatgtcgtatgtatatagaaaaaatggattggtttagatcgatcttaacctgaatgattgatcattatgaagtatttccatta

aattgcataaaacccgaatttagggttgaaataaatttacaagaaatctggccactaccaatccttaaacatttctggaaaccacactggatcagtatcgcagtgctcgt
caagcatttcatcccctacaatatcgacaagtccataagctttggcttcgtctgctgacataaaaacatccctttccatgtcttcggatacaacccaaaaaggcttgcct
gttcttagtgcataaacccttgtgatcatttcgcgaactttgtgtaactcttccacttctagtaaaaattctggtgttcttgcccgataataagcactagcaggttggtgaag
cataatcctcgcgtgggggaatgctatacgcttggtgggttctcctccaagcagaatgaaggacgccatggacgcggctattccgaggcatattgtatatatatctgg
tgtcaccgtttgcatcgtatcaaaaatcgccattcctgagattaaccacccgccgggggagtttataaacaaaaaaatatcgctaattccatcttctatactgagatatac
catgagacctgtaatatgattcgtgacctcgcaacgaatctcttgacctaaaaaaagtgtcctttctcgatacataacattgtataagtcaacccaagtcgcttcttcatct
ccgggaatccggtaaggtacttttggaacaccaatgggcatattagattaatattattaaatttaagtaagaaaactacactttaatatggaaacgtaagaatggaaga
gaaagaaagaatccgcagtttattgttttcactttttttcttattctatatgaatactatagattctattaatacgtagattgaaataatacatagattgaaagattatacatataa
gtaggataagacagattgaataaagaaaaaagaatgggtgattcgaatactaaacaaaaagagatagggatctattcttcgtttttccaaataagccaagctgccca
ttgcatattggcacttatcgagtatagaatagatctgcttctctttcttcttacgaacagaattggcttcttatttttaatggaatgaaataaatattcacgctttctgacacag
aatcccctagaagggttaggtacataggatatggatagtctttgccaatgcgataaaataaagtgacatcgtgtctatttttctttgctaaaggggtatttccatgggtttg
ccttggtatcgtgttcatactgtcgtattgaatgatccgggtcgattgctttcggtgcatataatgcacacagctctagtttctggttgggctggctcgatggctttatacg
aattagcggtttttgatccctctgatcctgttctggatccaatgtggagacaaggtatgttcgtcattcctttcatgactcgtttaggaataaccaattcgtggggtggttg
gagtatttcaggaggaactgtaacgaatccgggtatttggagttatgaaggcgtggcaggggcgcatattgtgttttctggcttgtgtttcttggcagctatctggcatt
gggtatattgggacctcgaaatattctgtgatgagcggacgggaaaaccttctttggatttgcccaagatctttggaattcatttatttcttgcaggggtggcttgctttgg
ctttggcgcatttcatgtaacgggtttgtatggtcctgggatatgggtgtctgatccttatggactaactggaaaagtacaagctgtaaatccggtgtggggtgcagaa
ggttttgatccttttgttccgggggggaatagcttctcatcatattgctgcgggtacattgggcatattagcgggcctattccatcttagtgtccgtccacctcaacgtctat
acaaaggattacgtatgggcaatattgaaactgtactttccagtagtatcgctgctgttttttttgcagctttcgtagttgccggaactatgtggtatgggtcagcaactac
cccaattgaattgtttgggcctactcgttatcagtgggatcagggatactttcagcaagaaatatatcgaagagttagtgatgggttagccgaaaatctcagtttatcag
aagcttggtctaaaattcccgaaaaattagccttttatgattatattggtaataatccggcaaagggggggattattcagagcaggctcaatggacaatggggatggaat
agctgttggatggttaggacatcctatctttagagataaagaagggcgcgaactttttgtacgccgtatgcctactttttttgaaacatttccggttgttttggtagatgaa
gagggaattgtgagagcggacgttccttttagaagagcagaatccaaatatagtgttgaacaagtaggcgtaacggtggagttctatggtggcgaacttaatggagt
aagttattctgatcctgctactgtaaaaaaatatgcgaggcgttcccaattaggggaaatttttgaattagatcgggctactttgaaatcagatggtgtttttcgcagcagt
ccaaggggttggttcacttttggtcatgctacctttgctttgctcttcttttcggacacatttggcatggggctagaaccttgttccgagatgtttttgctggtattgatcca
gacttggatgctcaagtggaatttggaacattccaaaaagtcggagatccaactacgaggagacagccagtctgaggccgcattgctttggtatctttcacctctcttt
tttgatttgacatgggaaacatctcccatcccttctttgactctttttcctttttatacgggaaatgatcccaaatgacaaatgaataggtgtggaagttataattgtaaata
aaccacgatcgaatctatggaagcattggtttatacgttccttttagtttcgactttagggataattttttcgctatcttcttccgagaaccacctaaggttccgactaaaaa
agtgaaataatttaattgaagtaagaagtctccccatctgggagacttcttacttcaattagtccccgtgttcttcgaatggatctcttaattgttgagagggttgcccaaa
cgcggtatataaggcatacccagtaaagcttacaagtaaaccagatatggagatggcgactaaagttgctgtttccatttttatagaatttcaagattacaatggatcta
cgaaaagatcgtgtatttacaactacaacggaatagtatacaaagtcaacacaaatcattaaatggaatttatggctacacaaaccgttgaagatagttctagacctgg
accaagacaaactcgcgtaggtaatttattgaaacccttgaattcggaatatgggaaagtagctccgggttgggggactactccttttatgggggtcgcaatggcttta
ttcgcggtattcctatctattattttagaaatttataattcttccgttttactggacggaattttaatgaattaggtttctactaacgaaaactacgaagtcatagtttttccatcc
aaaagagcctttctactttaagctctacatttctagacattctggtagttcgaccgtggaatttttttggtttcggtatctctggaatatgagtgtgtgacttgttagaatttgct
cctattgataatacatagaaagcacctgttatctctatcaagatgattctaattcgtcggatattatttattctagtatctggaacacgaaatagatagagtggatcaagaa
aaaaaaatgaaactatgattcatattaactattcagacctcgcaaccagactgaaaaaaattcaagtagttcttaataaaaataaaaaaagaaaatttcttccttccaattt
tgtttgcccaaaaaacaactttttttctctcgattttgtcgagtcattacaccgattcaataaatgatcatcaagcggttcttattcgaagaacccttgccttttgtttagcttg
agactcaatcatcgtggctctagtatgaatctaaggttttaattgaactgattcataggatcgcaacaagataatttctaccagaaaactactccaatttttgctttatttattt
atctagtaaaacaagagtaaatctgcattacgcacaaaaaaaaagaaatccaaaatagggaagagaaaaatcaagaggcctctaatgatcaacatttgggaaagaaa
gatagacgagccaacttgagattttttggcattatcatcacaaagaagaaattctggatttttcttatttcatatcttcaaggcaaatcgacccaacccagtggctgatga
agttttgaaccttttttctaatatccgttgaaaatttgtgtgtttctgtttgagccgtacgagatgaaattctcatatacggttctcggagggggggttcgggttagttacctat
ctcaataaagtatatgattggtttgaggaacgtcttgagattcaggcaattgcagatgatataactagtaaatatgttcctcctcatgtcaacatattttattgtttaggggg
aattacacttacttgttttctagtacaagttgctaccggttttgctatgactttttactatcgcccaaccgttacagaggctttttcctcggttcaatacataatgaccgaggc
caactttggttggttaatccgatcagttcatcgatggtcagcaagtatgatggttctaatgatgatcctgcacgtatttcgtgtgtatctcacaggtgggtttaaaaaaccc
cgcgaattaacttgggtcactggtgtggttttagctgtattaactgcatcgtttggtgtaactggttattctttaccttgggatcaaattggttattgggcagtcaaaattgtg
acaggtgtgcctgacgcgattccggtaataggatcacctttagtggagttattacgtggaagtgctagtgtgggtcaatccactttgactcgtttttatagtttacatacct
ttgtacttcctctgcttactgccgtatttatgttaatgcactttctaatgatacgtaagcaaggtatttcgggccctttataaggaaggcatctcatagagagttctaattctc
atatatcatatcgggtaggttgtggtatttcattgctacaaacatgggttattctaaaataagacatgtcatttagatacttctcttcaactccgaactattgtgatacaaata
atacaaatagttgaagttaattttacgaaagaaaagaaggcggattatgggagtgtgcgacttgaattattgatttggccatgcagatagagagttggatctgccacat
tagaattcacgaccaaaggtgtctccatatccaatcaacacgtaagtcccctgtctagtaaggataggctggttcacttgaggagaatattttctatgatcatacctcaa
ccatgtcatccatgaagaggctccgtaagatcccatagagtagaaatggaataagtcatgtgacatgatccaattctctatttattacacttactttttattatagtatggaa
atgcattcattttctttgcatcgattgcgatctgcaatactatcggagtaaaagaagggatctaaggaagaacgtaggctaaactttttgatttttttattagtaacaagtaaa
tactttgtttggacgtaagaaacttgcaatattggggggggataaataccaactaatcaagagacgcgagacaatccacaaagcaattgatcatgatcaaatttgtaag
cccacttggatattgagcatttacccataagagtaggattcttttcaatgaatagttgtaggtgcaacttcggaaaatagaatctgataaagcttttcttacttagagccatt
gagccattatataccttattctattatggatcttctacggttttatttctttcattcttgctcgagccggatgatgaaaaattctcatgtccggttcctttgggggctggatctta
aagaattcacctatcccaataacaaagaaacctgacttaaacgatcctgtattaagagcaaaattagctaaagggatgggacataattattacggggaacccgcgtg
gcccaacgatcttttatatatttttccagtagtaattctaggtactattgcatgtaatgtaggtttagcggttctcgagccgtcaatgattggtgaaccggcggatccgtttg

caactcctctggaaatattacccgagtggtacttctttcccgtgtttcaaatactccgtacagtacctaataagttattgggcgttctcttaatggtttctgtgccgacggg
cttattgacagtaccttttctagagaatgtcaataaattccaaaatccatttcgtcgcccagtagctacaaccgttttttaatcggtactgcagtagctctttggttaggtat
tggagcaacattacccattgaaaaatccttaactttaggtctttttagggatttttcagtttgattcattcaatcgtgaagtaccgtgcataggtatctaggaaatagttactt
ccaagtgaatcttccctagatacctataatctattttattatgatccatttcgcgaaaatatagattgtcccaaagatgcaaaattgttttctttttcttttattctaactcgaaa
aagaagaagaggaaaaaattgcaatggatttcaaactagaacttattcttaggtaaatccattgggagatgcttctctagagtgtcccatatctgttttccatcttgcatac
gaaaactgtcaattctcatcagatcttcttccgtcttactcaaaaggtccaatagtgtatggatattggcccttttgagacaattatacgttctagaaggcagttctaattga
tcaataaaaatacaattcaatggaattctttttttgtttttctttagattagttaatctttttgaaagcttaaaagggggaagtaaacctgtttttattttcttggaaacgagta
ccctcttcctccgtgtgaagaaaaggaagaaataaatcaatcaaattacgagaagcctcataaagcgcttccttaggggttaaacttccattggtccatatttctagaaa
aagtatctcatattttgcatttccattcccacaagcaaaaatactataattcacatttcgaacaggcatggatacagcatctataggataacttccatcttgagtgttctttct
gacttccgtgtgatatccgcgatctctcttgatccgtaactcaatacggaaatccgtgggttctgtcaagttagctataggttgtgccgtatcaacgatttctacggaag
gcggtaagataatatcttgggcagttatgtatctaggacctttgacgcaaattgatgcggttctaactccatagagattacttctcaatacaatttctttcaaatttagtaaa
atttcttgtaccgattcttcaatacctgctattgtagaatattcgtgcggcacgctcccaaattttgcatgtgtgatacatgttccttctgtttctccaagtaaagctcttcgca
aggcaataccaacggtatccgcttgaccttttctaagcggggacagaatgaaacgaccataataaagacgcttactatctactcttgattcaacacacttccactgtag
tgtttgagtggatcctgctacctcctctcgaaccatatagactagtattattatttgatcattgaatcgtttatttctcttgaaaggggtttaattcttttacagacgtcttttttta
ggaggtcgacacccattatgcggcataggtgttacatcgcgtatacaacttaatcgtacaccacttttagcaatggctcgtaatgcggcatctcttccactaccagcac
cctttaccataacttctgctcgttgcaaacccactgtacgaatagcatctacagctgttctttgaccagcatagggtgatgcttttcttgagcttttgaatccacaagtacc
cgcggaggaccagaaaccacccgaccttgcggatctgtaacagttataatagtattgttgaaactagcttgaacatgaataactccttttgttattctacgtgcactctt
ccgtaaactaaaacgcgcattcctacgcaaaccaatacgtactttcctacgtgaaccaatttttggtatagcttttgtcatattttattatctcataaatatgagttagaaata
acaaaaagaaaaaaagatacaaagatatccgtttcagggtaaaatatatcctttacttgaatttttttatttggaatttggacattttcgcgggtactttttttttacttttagaa
agtcaagttctttttcgaaagattaccccctgtctttgtttatgcttcggattggaacaaatgactctaattcgtccacgcctacgaatcagtcgacattttgtacaaattttac
gaacagaagctcttattttcatatttccgtattcctttcttaaactatgaatttactcttttggaaaaaataagtctctttgtttgaattttagaactttgaatttttttaccctagaaa
aaaaaagaaaaacctaaccctttgaatctttggtatccttcaaatcttcggtatccttcgagtcttcggtacgcttcgaatccttatggggaagtctataaattatacgccc
cttgcttgaatcataacgacttacttcaattttgaccctatcccccatcagtattcgtatagaactagaccggatctttcctgaaatatagcccaggatgatggtgtcattct
ctaggcgaacgcggaacattccgttgggtagggcttccataactaaaccctcgaaagtaatttttgcttctctcgggttttttttttctctcctatttttcttttctgtcatattttt
tttctcctatttttctttttttattttcaaataaaaaaaacgttgtatttttgtttgaaaaataggaagttcaagatagaatttgggtactataactataggtggggcgattaccgct
ataggcggggcgattaccatatataacataagacttctcccccaattctgtttagtcgagcttctcgatctgtcattatacctcgagaagtagaaagaatagcaattccc
attccgcccaaaaccttaggaattccttgatagttggcataaattcgtaagccaggtcggctgatacgctttaaaaaggttctagttctatatattccctttctagtctttctc
ttttgatgtcgcaaagttgaaaccaagaaatatctgttactttcctgatgtttccgaacactttcaataaaaccctctcgtagaagtattttaacaatgttttcggtaatatttg
tagatactacccgaacagttcctttttttattcatgtccgcgtttcttatagaggttagtaaatcagcaatagtgtccttgcccataagactctaattctaggttcctcctaattt
ttctataatcaacatgttctcttttttcttttcgttttggattctaaagcatatacgtgaaacacaatctactaattttttatttgatctatatcttgtctactagtatttataatacttca
ggagctaatgaaactattttagtaaaattcaattctctcaattcctcggcgatcgcgccaaaaacgcgagttccttttggatttcctttttgatcaatgataaccgctgcatt
gtcgtcatagcgtattattataccgtcttcgcatttgaactctttacatgtacgtacaattacagctcgaattacttcggatctttctagaggcatttggggcactgcgtcttt
gattacagcaacaataacatcaccaatacgagcatatcgctgattactagcagctcctatgactcgaatacacatcaattttcgagctccactgttatctgctacatttaa
aagggtctgaggttgaatcatattattttgatttcaatttgttatttcaatgcaaaaggatgaaagaaatattgtctttccagaaagaagaacctggtttttattttcaatact
actttttttggggggttatatttctaaccgaagaaattgacttcgtatgggcattttgctggcagctatggaaatagctgctctagctacagtttcggatactccgcccattt
cataaagtattcgacctggtttaacaacggctacccaatattcgggggatccctttcccgaacccatacgtgtttcggtcggtcttattgtaaccggtttgtcgggaaat
atacgtacccagatttttccaccacgacgtgcatatcgtgtcattgctcttcgtcctgcttctatctgcctcgccgtaatccaagtgggttcaagtgcttgaagagcatat
ctaccaaaacaaatacaattgcctcggtaggattttcccttcattcttcctctatgttgtttacgaaatctggttcttttggggttatagtcgatggttctctcttagttccatct
ctactgcaaaactggacatgagagtttcttctcatccagctcctcgcgaatcaaatgagaaagtgtgcaaatttctctaattccacaatattctaaaatattacggataga
tacacattaataggaaaggttagggtttttaatattgaatttattaaaatactaaaatatatagtcaagaaagaggatctagaatatatctagatgtgtatgtttatttatctat
attctatatttaatgtaattcttttttttttttttttaatctcctttttttattttactcttattgaatcgcggtaaagtattctaattcaataaaaatttcgcgggcgaatatttactcttt
cctgtcttatttgttaattcataaccttatcaaataagacaattttttttggtttgttccgccatcccacccaatgaagtattgggattcttttcaagaaaatcctatccagtcata
ggttctgtcgttcccactgcttctcctttaatggttaggtttgaatctcgcaatggagcttccaaaaaaatttctttccgagtcaatttctcagttttattaactcgggacgctct
ttgttattccttgaaatttgtatttcttgtttcaatcaaattacgatttcgaattctctgttatttttattatattgatgctttatcacattgctttttatgatgtaattcatagaccatac
atattggaatcctatatctttcttatttctcttccttctttctatcatccttccttttatctacatcccctttagttttgcttcacaacttagaatcctattttttttaaagaaaaaaattgc
agttgctacacgtatatgatagatttactcatttatgatagatgtatttattcatatagtgactgtttcttagttaggatctcgacaatacgaagcaataggttggttattagttc
attttctataattacatagtagggttcattctatttttttttaatccccttttttgaaccctaaaaaaaaaactaacgagtcacacactaagcatagcaattttattaaaagatttctc
aattttcattaaatcttatagaaagaggtagaatttcttctttttttcagggattttagggaaaataaggctcttgtcatttttttattctattactgaacagaatgggaagacag
ggttggttattcttcgtctacgaatatccaaattttaactcctaatactccatagatagttcgaattggatagcagcaataatcaattttagcgcgaattgtttggaggggga
agtctacctttttttgatgcattcggcacgcgcaatttcttttcccgcgagacggcctgcaatttttacttttactccctttatatctgttttttttagttaattcaatggctttttttcatt
gcctttcggaatgaaactctatttttttaattggaaagctatatattctgcaagaatgttaggttgtctataaggttctttcactttttcgatcccaatattaagtctctggtttaca
gaattaacttctttttgtagatctttctctaattcttcgattgctcctttttttctttaataaattggggaatccaatatggattatgacgtggattgtatcgatttctttttgaatttcta
tatgtgtaattacttcggaacttgagcctgagtccattttttttattatgtgtaattacttcgaaacttgagtctgcttctatttttctattcgagccctttttcctattcttttgtatata
gttcttgatacaattccgtattttttttatcttcctgtagaccttcagaataatttttttggttgtgcgaaccaaaaggaatggtgattttgggttgtaccaagtctgaaaccaagt
ggatttatttttttgtcccatatttttctattctatttttttttaccgggaatcaaatcttagatggatctaaagattatttcgatttctttactatatttagtacaattgttatatgacaca
tggttttttttatgggagaactacgtcctcgagcccgaggtctgaatttattcataatagtactcctactgacttcggctttagtgatgaataaatttgctttgtcgaaatccc

tataatgagtagcatttgctgctgccgaataaaccaactttaagatgggataagatgctcgataaggcatgaggttcaatatcataacagtttcttcgtagtaacgccaa
cgaatctcatccagaactctttgtactttgaaaacggacatgtggatgcgtttttgtgctttgaaaacccgttcgaacttaagtagacgatcggttggtacttttcttgcga
gtttccttagcccactcttcttcttctttatcctaggggtatactttaccagtttgaaacttgtcataaataaggttattccccgcttacctatttctttttttttttttattttgaatct
ttctattctgaattcagttaacgacgagatttagtatcctttcttgcactttcataactcgtgaaatgccgagttggtacgaattcccccaatttgcgacctaccataggattt
gttatgtaaataggtatatgttcctttccattatgaatcgcgattgtatggccaaccattgcgggtagaatgctagatgcccgggaccacgttactattgtttctttctcctc
cttcatattgaccttttctatttttgccaataaatgatgagctacaaaaggattcgtttttttcgtgtcacagctgattactcctttttttccattttaaagagtggcattcgatgt
ccaatatctcgatcgaagtatggaggtcagaataaatagaataatgatgaatggaaaaaagagaaaatcctttagctggataaggggcggatgtagccaagtggat
caaggcagtggattgtgaatccaccatgcgcgggttcaattcccgtcgttcgcccatcccattattgcaaattccaaaaatgcaattttccatattcctagttacgtattta
cttacggcgacgaagaataaaactatcactatattttttccttttcctagttcttcttccaagcgcaggataaccccaaggggttgtgggttttttctaccaatgggggctt
tcccttcaccgcccccatgggggtggtccacagggttcataactacccctcttactacggggcgtttacctagccaacacttagacccggctctacccaaactttttg
gttcaccccaacattacccacttgtccgactgttgctaagcagttttgggataccaaacggacctccccagatggtaatcttaaagtggccgatttaccttcttttgcaat
cagtttcgctacagcacctgctgctctagctaattgcccacccccttccacgtgtgatttctatgttatgtatggccgtgcctaagggcatatcggttgaagtagattcttct
tttctctcaaaaaacccccttcccaaactgtacaagcttcttccaaagcatacggctttctagatgtatatgacgatctctagacagatggatcttatatgaatcgtatgatg
aagtaccacatgagtggatatataggaaaggaatccaaatctgccgaatcgctcatgttatgatcttctacatcctaggtctccgcgttccgtcatctggcttatgttctt
catgtagcattcagatcgaatgactctatgaaattacgtcgatacttctacatattatgggtaacgtaggagacatccctattttcccccgggggtcttaattaccactgct
tagctttcaattcgcctctgaccatcaaattaaatgtgaataacccgtcctcctctctttgaaacaaggggcgcttccggttctgtgcgtgcttcaaacaattttgtcttctc
catattaccatatctctagagtcaataattttctatgaggaactactgaactcaatcacttgctgccgttactcaacagttttctgttgaggtctatcccgtagaggtagtca
aattggatcagtgatcgatttctaggtttcgtcgtaaacctaattggttacttccaattacgtaaatcaatagttcaaaccgcactcaaaggtagggcatttcccattgata
taggaacttttgtaccagaaacaatagtatctccaattatagcccctctgggatgtaaaatatatcccttctcaccatccccatagtgtatgagacaaatgtacgcatttc
gattagggtcgtattctatggttacgattctaccagatatgtctttttgattccgtcgaaaatcgattttacggtataggcgcttatgacctccccctctatgccttgcggta
atgattcctctggaattacgacctttaccacaacggtgccgtccatggatcaaattatttcgtggattggatttcacttgcctatctatggttcccttgcgtgtgctcgggat
aggtgttttgtataaatgtttcgccgtattattaagtattctcctttagttttttctctatctagaagtggaatagaataacccggttgaagggtaatgatcatacgtctgtaat
gcattgtatgtcctagaatagggcccattcttctaccctttccgggtagtcgatggctattcacagctactaccttaacaccaaagaagagttcgacccaatgctttattt
ctgtcttagtgaatcccgattcgacattaaaagtatattgattctttcccaataaacgaagactttttctgtaaatactgcgtatttgattccatccataaatcgactttccct
cctatgctctgagttccagtatcgataagaattcgagttcttattgttcttatgttatggtatgaatataccataccaattcgttatgtatggatgatggatgagattccatgg
atagagagccagttccaatagacttatggaacgttccggttcgcgtgcatccagcaggaattgaacccgcaaatttaccaattatgagttgggcgctttaaccattca
gccatggatgcttaacagggatcatcgtacatcgtaaataaccaattttcatatagaaagacatatcatagaaaaatgaaatcgaaaatattcggagatggcaaatatt
cggagatgactatgaaaacacctctctggatcctcgaattgaaagagagattgagagggatccagaatcctaattctcgctatttggaatggatccaattctattgagt
ctgactcatagtgatcatttctctttagcaaagaatgaccttggttatcaaaggattgaacaaccgggatccgtttacttatgatacctagttgacattgataacaaggatc
taatgaattatgagtttaatagatcctctttagcagaaagacgtatattccttgctcatcacttattcccaaacctcgtatggggctaatcgttttcatttaccatctcaggga
aaatccttttcgttccgcttagccctatcgggtattttagtgataggttctataggaactggacgatcctatttggtcaaatacctaatgaaaaattcctattttcctttcatta
aggtacgagggcttcttattccacaagaaaagaacgaaagcaccttttcattctttcatatactgggggtttttacttggaaaagacaatgttccatactaaaggattcgg
gtccataaccacgagttccagtgcactagatcttgtagcacttagcaacgaggccctatgaatagacatatagaatttttggtcgggaaattcgaatgaatcattgagt
gaaaaaggagcaaagaatgacaaaagacgagactctactagtcttcactcttgtggtttcctcggtttctgttttcttattcgggatcttgcttttcatggttctcatctctgc
aactcgcgattttcgcgagagaaccaaatccaagttggtgaagatcatgatttgggctggcatagtagtgattacctttgcaattgcggttcgaatctatccgatctttat
ctttttgctcaaagaacgaataaaaccccttgtcgaagccctttatgataagcttccctggatctgggaagtttctctttcacggtattgggatcgtttgatcgatttccttg
atcgctacttatgggcgtgcgctcaaaggatacaaacagggattcgcaaacaaaaaggggaattcgtagtcactttttcctgtcgcgtaaaaaaaaaggctttacgcg
agagcaatagaggttgggatacatctatctcttctgagcaacctcttttggattcttaagaccacccttgcagtaggataccgtctgctttgggttctttattatattatctcc
ttcgagggatttttaggatcgttcaggctatatttagtctattttggcttttactgtctccttttctcagggaagtggttaaggacctcagaagatagaggagagcgccag
gcgcagatttccggaatacttctacggggaatgctcattgaatgcgcattctccgtattatgccttgaagaggactcgaacctccacgctctttagcacgagattttga
gtctcgcgtgtctaccatttcaccatcaaggcatcttgaaagtgaatcatattccatgaatatgatatctatctaatgtgatatcttgaaagtgaatcgtattccatgaatat
gatatctatctaatgtgatatatggaatatatgacaaaggtggagtcttggagtatttcgatcgatcggtcatataggcctgagtcagacatcaaatagcttcgatttgca
ttatccgtaggacaccttatatgtatcaaaatcaaaaagatgtacaatccaatttctcgattcaatagaagcccaaagaggtgcatatggtacccaaataaggatagga
tagatatgtcaaaagcaggtctgattacacctattcctaatcctaaatagaatgtaaggacgtggggatttctatgtaaacagagtatcctatttccataggctcgaatga
ccccttctcataataagaatgtgcacggtctggtccggtatggaatgaacttataatctgatgatcgagtcgattccatgattataagttcattaccctagcgcccattcc
cattttgggcggaacagatctactaattcttttattccagttagtaagagggatcttgaactaagaaatagacctagcagctaaaagagggtatcctgagcaattgcaa
gaatggggttcattgatattcctggtatagtagatgctatcacacatacagtcatactcaattcgatggaattgtttgatcttaaaggggatcttctataatttcgcacataa
ggggttatttcttggtttcgtccagtcattaataacttgactatttttagataatagtagatagaaagaacgctcgtaaggagtcctattgaaaccaagaaatataggcctg
cttgccatccacaccagaatagatagagttttccgaagaaacctgctagtggaggaaggcctcctagggataagagacatagggctaaagagagagccaaaaaa
ggatctttcgtgtataatcctgcataatctcgaatgttatcagttccggtacgtagaccaaataatacaatgcaagcaaaagttcctagattcatggagatatagaaaag
catataagttatcatgcttgcatatccatcatttgagtctccaacaattattccaataattacatatccgatttgccctatggacgaatatgcaagcatacgtttcatgcttgtt
tgagtaatagcaaggagattccccaatatcatgctaagaatagctaggatttccagaagaagatgccattcgtttgatgagaaataaaaaggaatatcgagaattcgc
gtggctgaagctgaagcagcaactttcgaagtaacagaaagaaaagcaacgactggagtgggggagtcagagtcgaaaagaggattcctcgcttctttctctcat
gcaaaaccgtgcatgagactttcatctcgcacggctcctaagtgataaaagaaagaagaacttgtcttctttctttttgattaccttcctcgcgtatgtataagaccgaat
ccattctttttcgaaatcgatttcgaaaaagaactactaatccttaacttttcgaggaatccttcatcagtggttgtgaatgactgactttttcaatcctttcgaccttggttcc
gtaggagcaagtcagaaaggttgagaaatagaaccatctgatttgattcgttcccaatagccatgagatgatcatcttagggtgatccttttgtcaacggatgctcctat

tacactcgtagtctctgaaggatgagaacccactatgtagcatctacgtcgataattcaagcattgtatacgtcattagtccgattctttgtaggaactacccgtaataac
gagcttgcaaaatggatctgtttatcataaagagattcgttgttcctgaccctgcttcaccttaattgttatttgaacaaaaagatcacaataaacttttggtaaaagttctgt
cttggtcggagtggggatagcatttctcttctgcatatctatggagttttgcaaaacccaaacacctcagagatagatatagaggtaggaatttgtcgaacgaaccaca
ctccttcgtagacgtcaggagtccattgatgaaaaggggctggggaaagcttgaacccaagtcctacagtgatggatataagcgcaattgaaattcctggggagtta
tacatttgtgtattgataagaccgttcacaatttcttgaagctcgatctcccccccagatgaaccatatagccaagagaaaccatgaaccagaatagaagagcttgccc
cacccatgagtaaatatttcatagtagcctcattagaccgtagatctctcttggtatatccagacaataggtaggaacataaactgaaacattctggagctacaaagata
gttattaaatcgttagcaccacataaaaacattcccccctagagtagctgttaatacgaataacagaaactctgttatagccatttctgtacattcaatgtactctacggata
gaggaatacataaagttgaacataataaaataagaaattgaaagatttcgttgaaattgttcgtttggaaatttcccgaaaagctaattataggttcttctctccatcggaa
caatagggccgttatgcttattactaaacttgttgaagagatgaaatagaaccaaggtctatctttttgatcagaggttaaatcgatcatcagaagaagaattaggccaa
aaattaggatacattctgggaaaatgaaacttccctggaagagaagcaaatgaaacgctttcataaaaattctcgtagaatcgagaatgaagttttcattctgtacatgc
cagatcatgaattagtaactgcatccaatctccgaaaagtcccgattgtttcgatttttggaatgggatatttacggaatccccatgaataggatcaaaccttattccatg
ctatttccataagattcctctttcttattcttaagcaagcccccgagagggcttagttgatcatgatttctgttttctctttcttttcctttttgtttgtttcgagaaagatatcgtcc
gattctccttctattgattcttttccgatcgagatgtatggatccatgtgtctacatacctagattctgttcatggattaacgaaaatgtgcaagagctctatttgcctctgcc
attctatgagtcgcttcctttttgcgtatggcacccccaccccctttggcagcatctactaattcggaacttaatttgaaagccatatttcgacccggacgcttttgggatg
cttctaataaccaacgaatggcaagtgctcttccttgtttagatcctatttcaatcggaactttccgcgtcgatcctttttttattacgtcttgtttttactcctatattgggagtta
ctctacgtattgcttgacgtaaaaccaatagtggatttgtttctgtcttttgttgaatctttttcacggctcgatagagaatttgataagccaatgatttttttccgtctttcataa
tacggttaaccaccatgttaactaatcgattacgaaaaattggatcggattttgcagttcttttttctgcagtacctcgacgtgacatgagcgtgaaagaggttcaagaat
ccgttttctttttataagggctaaaaacgaatcacttattttttttggctttttgaccccatattgtagggtggatctcgaaagataggaaagatctccctccaagccgtacat
acaactttcatcgaatacggctttccacagaattctatagggatctatgagatcgagtatggaattcagtttactcacttaaaattgagtatccgtttccctccttttcccgc
taggatcggaaatcctgtattttccatatccataccatcaagtccttaggtttccgaaatagtgtaatggaaaaagaagtgcttcgaatcattgctatttgactcggacct
gttctgaaaaagtcgaggtatttcgaattgtttgttgacacggacaaagtaagggaaaacccctgaaagaatttccatattgaccttggacatataagagttccgaatc
gaatctctttagaaagaagatcttttgtctcatggtagcctgctccagtccccttacgaaactttcgttattgggttagccatacacttcacatgtttctagcgattcacatg
gcatcatcaaatgatacaagtcttggataagaatctacaacgcactagaacgcccttgttgacgattctttactgcgacagcatctagggctcctcgaataatgcgata
tctcacaccgggtaaatccttaacccttcctcctcttactaatactacagaatgttcttgtaaattatggccaataccaggtatataagcagtgatttcaaatccagaggtt
aatcgtactctggcaactttacgtaaggcagagttgggtttttttggggttgatagtggaaaagtcgacagataagtcacccttcctgtccctctacagaaccgtacatg
agattttcacctcatacggctcctcgttcaattctttcgaagggatccttttcctcgttcgagagtctccgcccttcttccactccgtcccgaagactaactaagaccaatt
gagtcacgttttcatgttctaattgaacactttccatttatgattaaaggagaagattgttcttttaccaaacatatgcggatcaaatcacgtcttataataagaagaaatctt
tctcggtatcaatccccttgcccctcattctttgagaatcagaaggatccttttcgagtttccatttcttcatttggaatctgggctcttctatcttcgacttattttttttttgcttt
attctttatttatttcatttcgatttttccctcttcctctatccctatcctctaggtacagcgtttgcatcaatagagaaccttttcctctgtatgaatctatattattccattccaatt
tcttcccgaaacttcccaagaaaaatcccgaattggatccaaaattgacgggttaatgtgagcttatccatgcggttaggcactcttcaaataggaatccattttctaact
ggctttcgtgctttggtgagtcgtccgagatcctttcgatgacctatgttgtgttgaagggatatctatatgatccgatcgattgcataagacccgcggtagcaatagaa
cggggaaagtatacagaaaagacagttcttttcaatttcgattatctatatattagttcgtttctatttctagatatctatttctatatattagtattagttagtagtactattctatt
agttagcgatcccggctctgtgagttctttcttccgtgatgaactgtcggcaccagtcctccatttttttctctgtggaccgaggagaaagggggctcagcaggaagag
gattgtaccatgagagaagcacagaggtcaaccctcttcaaatatggaacatggattctggcaatgcaacgtagttgggtcctcatatcgatccgaatgaatcagtct
ttctacagaggtcaatctttgcctattaggcaagaggatagcaagttcgaaattctgtctcggtaggacatggatttctattactatgaaattcataaatgaaaatgaagt
agttaatgggagggctaccattatcctttttcttgtatgtgttcctaagagaaggaatttgtccatttcatgtttcgaggtctcaaaaaagggcgtggaaacagatagaaa
ctcttgaatggaaattgaaaagaaatgtagccccagttccttcggaaatggtaagatctttggcgcaagaagaaggggcgacccatatcatcttgacttggttctgctt
cccctcttttttttaagaataccgagtcgggttcttctcctaccagtatcgaatagaacatgctgaacaagatcttcttcatggaaacctgctcgatttagatcgggaaaat
cgtacagattttatgaaaccatgtgctatggctcgaatccatagtcaatcctattttcgataggaccggttgacaattgaatccaatttttcccattatttgactatccataat
agtgtggaaagaaagcccggaggaagggtggccttgagtttctcgccccctttgccttaggattcgttaattctctttctcgaggggacggggaagggatataactca
gcggtagagtgtcaccttgacgtggtggaagtcatcagttcgagcctgattatccctaaacccaatgtgagtttttttctattttgacttactcccccccgccacgatcgaa
cgggaatggataagaggcttgtgggattgacgtgatagggtagggttggctatactgctggtggcgaactccaggctaataatctgaagcgcatggatacaagttat
ccttggaaggaaagacaattccgaatccgctttgtctacgaataaggaagctataagtaatgcaactatgaatctcatggagagttcgatcctggctcaggatgaacg
ctggcggcatgcttaacacatgcaagtcgaacgggaagtggtgtttccagtggcgaacgggtgagtaacgcgtaagaacctgcccttgggaggggaacaacaa
ctggaaacggttgctaataccccgtaggctgaggagcaaaaggagaaatccgcccaaggaggggctcgcgtctgattagctagttggtgaggcaatagcttacc
aaggcgatgatcagtagctggtccgagaggatgatcagccacactgggactgagacacggcccagactcctacgggaggcagcagtggggaattttccgcaat
gggcgaaagcctgacggagcaatgccgcgtggaggtggaaggcccacgggtcgtcaacttcttttctcggagaagaaacaatgacggtatctgaggaataagc
atcggctaactctgtgccagcagccgcggtaagacagaggatgcaagcgttatccggaatgattgggcgtaaagcgtctgtaggtggcttttcaagtccgccgtca
aatcccagggctcaaccctggacaggcggtggaaactaccaagctggagtacggtaggggcagagggaatttccggtggagcggtgaaatgcattgagatcgg
aaagaacaccaacggcgaaagcactctgctgggccgacactgacactgagagacgaaagctaggggagcaaatgggattagagaccccagtagtcctagccg
taaacgatggatactaggtgctgtgcgactcgacccgtgcagtgctgtagctaacgcgttaagtatcccgcctggggagtacgttcgcaagaatgaaactcaaagg
aattgacgggggcccgcacaagcggtggagcatgtggtttaattcgatgcaaagcgaagaaccttaccagggcttgacatgccgcgaatcctcttgaaagagag
gggtgccctcgggaacgcggacacaggtggtgcatggctgtcgtcagctcgtgccgtaaggtgttgggttaagtctcgcaacgagcgcaaccctcgtgtttagttg
ccactatgagtttggaaccctgaacagaccgccggtgttaagccggaggaaggagaggatgaggccaagtcatcatgccccttatgccctgggcgacacacgtg
ctacaatgggcgggacaaagggtcgcgatctcgcgagggtgagctaactccaaaaacccgtcctcagttcggattgcaggctgcaactcgcctgcatgaagcag
gaatcgctagtaatcgccggtcagccatacggcggtgaatccgttcccgggccttgtacacaccgcccgtcacactataggagctggccatgtttgaagtcattacc

cttaaccgtaaggagggggatgcctaaggctaggcttgcgactggagtgaagtcgtaacaaggtagccgtactggaaggtgcggctggatcacctccttttcagg
gagagctaatgcttatgcttattgggtattttggtttgacactgcttcacgcccaaaaagaaggcagctacgtctgagctaaacttggatatggaagtcttctttcgtttag
ggtgaagtaagaccaagctcatgagcttattatcctaggtcggaacaaattagttgatagtgataggatccccttttttgacgtccccatgccccccccgtgtggtgtgg
cggcatggggatgtcaaaaggaaagggatggagtttttctcgcttttggcgtagcaggcctcccaaagggaggcccgcgcgacgggctattagctcagtggtaga
gcgcgcccctgataattgcgtcgttgtgcctgggctgtgagggctctcagccacatggatagttcaatgtgctcatcagcgcctgacccgaagatgtggatcatcca
aggcacattagcatggcgtactcctcctgtttgaatcggagtttgaaaccaaacaaacttctcctcaggaggatagatggggcgattcaggtgagatcccatgtagat
ctaactttctattcactcgtgggatccgggcggtccgggggggggcactacggctcctctcttctcgagaatccatacatcccttatcagtgtatggagagctatctctc
gagcacaggttgaggttcgtcctcaatgggaaaatggagcacctaacaacgcatcttcacagaccaagaactacgagatcaccctttcattctggggtgacggag
ggatcgtaccattcgagcctttttttcatgcttttcccggcggtctggagaaagcagcaatcaataggacttccctaatcctcccttcctgaaaggaagaacgtgaaatt
ctttttcctttccgcagggaccaggaggttggatctagccataagaggaatgcttggtataaataagccacttcttggtcttcgactccctaagtcactacgagcgccct
cgatcagtgcaatgggatgtggctatttatctatctcttgactcgaaatgggagcagagcaggtttgaaaaaggatcttagagtgtctagggttgggccaggagggt
ctcttaacgccttccttttctgcccatcggagttatttcccaaggacttgccatggtaagggggagaaggggaagaagcacacttgaagagcgcagtacaacgga
gagttgtatgctgcgttcgggaaggatgaatcgctcccgaaaaggagtctattgattctctcccaattggttggatcgtaggggcgatgatttacttcacgggcgagg
tctctggttcaagtccaggatggcccagctgcgccagggaaaagaatagaagaagcatctgactctttcatgcatactccacttggctcgggggatatagctcagt
tggtagagctccgctcttgcaattgggtcgttgcgattacgggttggctgtctaattgtccaggcggtaatggtagtatcttgtacctgaaccggtggctcactttttcta
agtaatggggaagaggactgaaacatgccactgaaagactctactgagacaaaaagatgggctgtcaaaaaggtagaggaggtaggatgggcagttggtcaga
tctagtatggatcgtacatggacgatagttggagtcggcggctctcctaggcttccctcatctgggatccctggggaagaggatcaagttggcccttgcgaatagctt
gatgcactatctcccttcaaccctttgagcgaaatgtggcaaaaggaaggaaaatccatggaccgaccccattatctccaccccgtaggaactacgagatcacccc
aaggacgccttcggcgtccaggggtcacggaccgaccatagaccctgttcaataagtggaacacattagccgtccgctctccggttgggcagtaagggtcggag
aagggcaatcactcgttcttaaaaccagcattcttaagtttaagatcaaagagtcgggcggaaaaaggggagagctccccgttcctggttctcctgtagctggattcc
ccggaaccacaagaatccttagaatgggattccaactcagcaccttttgttttgagattttgagaagagttgctctttggagagcacagtacgatgaaagttgtaagct
gtgttcgggggggagttattgtctatcgttggcctctatggtagaacccgtcggggaggcctgagaggcggtggtttaccctgtggcggatgtcagcggttcgagtc
cgcttatctccagcccgtgaacttagcggatactatgatagcaccgaattttgccaattcggcagttcgatctatgatttcgcattcatggacgttgataagatccttcca
tttagtagcaccttaggatggcatagccttaacgttaatggcgaggttcaaaagaggaaaggcttgcggtggatacctaggtacccagagacgaggaagggcgta
gcaagcgacgaaatgcttcggggagttgaaaataagcatagatccggagattcccaaataggtcaaccttttgaactgcctgctgaatccatgagcaggcaagag
acaacctggcgaactgaaacatcttagtagccagaggaaaagaaagcaaaagcgattcccgtagtagcggcgagcgaaatgggagcagcctaaaccgtgaaa
acggggttgtgggagagcaatacaagcgttgtgctgctaggcgaagcggttgagtgccgcaccctagatggctaaagtccagtagccgaaagcatcactagctta
cgctctgacccgagtagcatggggcacgtggaatcccgtgtgaatcagcaaggaccaccttgcaaggctaaatactcctgggtgaccgatagcgaagtagtacc
gtgagggaaaggtgaaaagaaccccagtgggtagtgaaatagaacgtgaaaccgtgctgagctcccaagcagtgggaggggaaagtgatctctgaccgcgt
gcctgttgaagaatgagccggcgactcataggcagtggcttggttaagggaacggaacccaccggagccgtagcgaaagcgagtcttcatagggcgattgtcac
tgcttatggacccgaacctgggtgatctatccatgaccaggatgaagcttggatgaaactaagcagaggtccgaaccgactgatgttgaagaatcagcggatgagt
tgtggttaggggtgaaatgccactcgaacccagagctagctggttctccccgaaatgcgttgaggcgcagcagttgactggacatctaggggtaaagcactgtttc
ggtgcgggctgcgcgagcggtaccaaatcgaggcaaactctgaatactagatatgacccaaaaataacaggggtcaaggtcggccagtgagacgatgggggat
aagcttcatcgtcgagagggaaacagcccggatcaccagctaaggcccctaaatgaccgctcagtgataaaggaggtgggggtgcaaagacagccaggaggtt
tgcctagaagcagccaccctttaaagagtgcgtaatagctcactgatcgagcgcccttgcgctgaagatgaacggggctaagcgatctgccgaagctgtgggatg
tcaaaatgcatcggtaggggagcgttccgccttagagggaagcaaccgcgaaagcgggggtcgacgaagcggaagcgagaatgtcggcttgagtaacgaaaa
cattggtgagaatccaatgccccgaaaacccaaggtttcctccgcaaggttcgtccacggagggtgagtcagggcctaagatcaggccgaaaggcgtagtcgat
ggacaacaggtcaatattcctgtactaccccttgttggtacggagggacggaggaggctaggttagccgaaagatggttataggtttaaggacacaaggtgaccct
gctttttcagggtaagaaggggtagagaaaatgcctcgagccgaggtccgagtaccaagcgctgcagcgctgaagtatgagccccgtggactagccattgcttct
ccacgaggctcataccaggcgctacggcgctgaagtatgtaacccatgccatactcccaggaaaagctcgaacgaccttcaacaaaggggtacctgtacccgaa
accgacacaggtgggtaggtagagaatacctaggggcgcgagacaactctctctaaggaactcggcaaaatagccccgtaacttcgggagaaggggtgccccc
tcgcaaaagggggtcgcagtgaccaggcccgggcgactgtttaccaaaaacacaggtctccgcaaagtcgtaagaccatgtatgggggctgacgcctgcccag
tgccggaaggtcaaggaagttggtgaactgatgacagggaagccggcgaccgaagccccggtgaacggcggccgtaactataacggtcctaaggtagcgaaa
ttccttgtcgggtaagttccgacccgcacgaaaggcgtaacgatctgggcactgtctcggagagagactcggtgaaatagacatgtctgtgaagatgcggactacc
tgcacctggacagaaagaccctatgaagctttactgttccctgggattggctttgggcctttcctgcgcagcttaggtggaaggcgaagaaggccccttccgggg
gggcccgagccatcagtgagataccactctggaagagctcggattctaaccttgtgtcagacccgcgggccaagggacagtctcaggtagacagtttctatgggg
cgtaggcctcccaaaaggtaacggaggcgtgcaaaggtttcctcgggccagacggacattggtcctcgagtgcaaaggcagaagggagcttgactgcaagact
cacccgtcgagcagagacgaaagtcggccttagtgatccgacggtgccgagtggaagggccgtcgctcaacggataaaagttactctagggataacaggctgat
cttccccaagagtccacatcgacgggaaggtttggcacctcgatgtcggctcttcgccacctggagctgtaggtggttccaagggttgggctgttcgcccattaatg
cggtacgtgagctgggttcagaacgtcgtgagacagttcggtccatatccggtgtgggcgttagagcattgagaggacctttccctagtacgagaggaccgggaa
ggacgcacctctggtgtaccagttatcgtgcctacggtaaacgctgggtagccaagtgcggagaggataactgctgaaagcatataagtagtaagcccaccccaa
gatgagtgctctctcctccgacttccctagagcctccggtagcacagccgagacagcgacgggttctccacccatacggggatggagcgacagaagcatggaaa
taggataaggtagcggcgagacgagccgtttaaataggtgtcaagtggaagtgcagtgatgtatgcagctgaggcatcctaacgaacgaacgatttgaaccttgtt
cctacacgacctgatcaaatcgatcaggcacttgccatctatcttcattgttcaactctttgatgaaaagatgaaaaaaccaaaaaaaagctctgcccttccatctcttgg
atagatagagagggagggcagaggcctttggtgtcccttccagtcaagaattggggcttcacaattactagccaatatttctctcatgcctttcctcgttcatggttcga
tattctggtgtcctaggcgtagaggaaccacaccaatccatcccgaatttggtggttaaactctactgcggtgacgatactgtaggggaggtcctgcggcaaaatag

ctcgatgccagaatgataaaaagcttaacacctcttatttgactttttcactattttgaaatacgaaaaagatccaaatccaaaatgcaaaggtcgtcttattcaaaacctc
aatcatcacatcccctctctcccacttcacacctcggaacgcactgttcttatagagagaaaggggctttcccatcttcttaacccgaaatgaaatggctgaggagag
ggaggttccttttgggggggtacccccgggaagagatccagtggagacggggtgggcctgtagctcagaggattagagcacgtggctacgaaccacggtgtcgg
gggttcgaatccctcctcgcccacagccttccaaaggggaagggcctttactttccccctgagggtaggaaaaccatgatcgggatagcggacgtaaagctattga
acttgggtatgctctttccttttgtcgaagtggaatcgtagaacagaatgtgatacgatgagataaaatgcaatagaaacaaggatagcgaacgggttacctactccta
agggtcaaagcaagccctttaattcaattctttattcttacattaaagaatgaatcaaatctccccaagtaggattcgaacctacgaccagtcagttaacagccgaccgc
tctaccactgagctactgaggaacaagggggattcgacctcctagagttcaactcccgctctcaacccatgaacaatatgagtccgaagcttctttcgtaactcccgg
aatttcttcgtagtggctccgttccatgcctcatttcataggtaagcccagagtggctctatttcattctatttcacttcctagcacttcctatcatttaatatccatccctttgg
tcttattgacataagagatgtcatttatagtctatctctttctatatatggaaagtcaagaaattctcatcgaaacatcgagaaattgtgcatatagaaaactctaaagaaa
gaaaaaaaggagacccatgccatgattttcaaatcttttctacttagtagtctaagtttctcgatgaggataattaattcggtcgttgcggtcggactctattatgggtttct
gaccacattctccatgggtccctcttagatcttctttctccaatcttggattagggaagaaggagatattcgcgactcctggtggtttcattatggggcagctcatgatctt
catatcgatctattatccacctctgcatctattctttcttagctaaacgggtggaagatccatccaatttggttatatcatggactcaaaaaacggatctgaatgtgactga
aatgcacgatcttcacaggtatcacttttcacgatacctaaaaggtggaatagcgattttcgaaccatttcctataagagaaaggtttccattactttgagaaatggattct
atatcaaactatagctattgcattaaagaagaaaagaaactaatagaagtcgaagacgcggaatggtagtgaatagagagaaagattcttctggttttcttgttcctga
aaatattctatctatctcctagacgccgtagagaattgagaattttcatgtctttcaattctcgtactcgtaattggaaagttacggaaggagatccatcattttgcaatgaa
aactacataaaaaactctggacaatttcgaaatcaggccaagcgtcttaatacatatgcaaaaaaattcattattggcccaccattgattagaagatttaacttgtatgaa
tcgctattggtttgatacgaataatggcagttgtttcagtatgttaaggatacagatgtatccacaattcatttagagttacttaatagcctatttcttataccatatctctatcc
cgtgaaattctcgagccgaaagatggatgcatatgctatgtttcattttgctaaatgatatcaattaaacggtgtatcaattccataaattggatatagcaataaataaatc
agcaaaattcttttattttagatagaagaaacttttcttctatctaaaataaaagaatgtacccttctatccaaatccaatttgcatcgataaaataaatccaaattccagtagt
agatgaataattgcaaatttttgtgtgtacgagattagaataacttcaaaataactgacataatttttatttttcctgatcagaaaaatacatgaaaaagaaaggaggtag
aaaaattttggatttatggttaaagaagaaaaagaagaaaactggggttctgttgaatttcaagtattcagtttcaccaataagatacggagacttgcttcacatttgga
attacacaaaaaagatttttcatcggaaagaggtctccgaagacttttgggaaaacgtcaacgtttgctggcttatttggcaaagaaaaatagagtacgttataagaaa
ttaatcagtcagttggatattagggagcggtaatttcatcgttcgaattttttttcttattttattagtagtcttatagtagtcttagattttgcattttgatgagcctcgttttgagg
aattcatggaataatccattttcatggaataatgaattaaggaagaaaggatatgagtctaccgcttacaagaaaagatctcatgatagtcaatatgggccctcagcac
ccatcaatgcatggtgttcttcgactgatcgttactctcgatggtgaagatgttattgattgtgaacccatattaggctatttacacagaggaatggaaaaaatcggcaa
ataggaaagctacttaggcaggagataggggaattccttaagaaagaaaaaagaataagaacacagatacataacataaaaaaaagaataaataagacgaaattc
gacctcccccctacatatttaatttcttctcctatacaaaaactagcaagacctactccattggtaattccatcaatgacacccttgtcgaaaaactgcgttagttcggttaat
cctcttatacccaaggtaaaggtcctagtatagaaaatatctatataaccgcgattatatgaccaactatatatcttttttttttagttgatggaaaaaatactttttcggaccc
ccttttacaaaggaatttattaaatccaaattctgaaaaaaagagtaagcagatccataaaacatatatgctatgaatagaccaaaaatagctagacttacagaagaaat
tgcattagtgataaattcatatgaatttatggaagaattagaactttcttggaaaaagttgattgagggagttagccactttgataatatggttaattcccctatttcattatca
aaatggattcctatagatccaataaacaaagtacaaagcagtaatataagaagaggaaatagcatagtatttcccggttcatgaggatagacaaaagtgtttttagtcc
ccaatgaagtactaaaggaccctatcctatttcctgtattaacatgaattttggatagattttgtgaaaaaaaagaaactccactcttcgctgttgataaaacgaaatctct
attgactcctttagatatcctttttccccataacgatattgaatacaacgaatcctctttagtactactgtaattttgaaaatgaacacgcaaatacccatcaaaagtaagta
aatatatccgaaacatataaaacgcagttaatcctgcagtaaaagaagctattattccaaaaaagggtgaatataaccaactattactaaggatttcatctttggaccag
aagcaagcaagaggtggaataccacaaagagaaagggtaccccataaaaaacaagttcttgtaattggaatgtattttcttaaaccacccataagaaccatattctga
cttttatctggtgaatatccaacaagaggttccattgaatgaataacagatccggatcccaagaataataaagctttcgaataagcatgagtgatcaaatggaataaag
cagcttgataagaacctatacctagagctaacatcatataacccaattgagacattgtagaataggctaagcttcttttaatatctctctgagcaagagctaaagtggct
cctaagaaaagtgttagtgtacctattaaagaaatgaaactcattatcaaaggtagggatatgaaaagaggaagaagtcgagctataagaaaaatccccgcagcaa
ccatagttgctgcgtgtataagagctgaaatgggggtgggtccttccatagcatcgggtaaccatacgtgaagagggaattgtgccgatttcgcaactgcaccaag
gaataataaaaaagcacacaaaatagtaagcaaggagttaatctcattattaggaatccagttattagctattttaaacaaatcccgaaactctaaactacctgttatcca
aaaaaaacctagaattcctaataacagaccaaaatcccctacacgattagttacaaaagctttttgacaagcactcgctgcaattggccgtgtaaaccaaaagcctat
caataaataggaacacattcccacaagctcccaaaaaaaaataaatttgtatcaaattggaactagtaaccaatcccaacatggaagtattgaaaaaacttatataaaca
aaaaatctcaaatatccctcatcgtgagacatataatcatcactataaataagaaccaggattcctacagtagtaattagtattaacataatagaagtaagcgggtcgat
taagtatccaaattctaaggaaaaatcattattgacggtccaagaccatagatattgatagatagaacttccatttatttgttgaatagacaggtgaactgagaataccat
agctatacttaaaagtaaaacactaggaaaagcccatatgcgacgaagatttttttgttgctgttggaacaagaaaaagtccaaaccccattgacataataactggaag
tgggagaagagggattacccatgcatattgatatgtatgttccataagaaaagaaattgcaatttttacttgaaaattttacttcaatttttctataaaattgaaaaaagtttc
cgattcaccaaactaattcttatctatttctgaaggaataaaaaaatactagaattcttaatttttcaaaaattttctcattgaaacaatcaaaaaataagaataggttttgttg
gttaaagtcaaaaagttaatgaaataacttcgttacctagttattacctaaagaaggacttttattaaaatacaaaaaaaagattgaatcattttactttaatattttttttgtatt
aaaatgaagcagctcccttgtttcgtaactcaaattgattggaattcaattctggaatactttaattaactatttgattgaattttcccttccttttatcctcccgtcttatatggg
ggataggcccccatacсttatatctgtatatggagagtatacttgaaatataaattgatttaaatagaaaacctttgtatatattctatattattaaaacaaagtctaaaaaaa
atatataatatgttaaaaaactcttgtcttatccgcattagacaaaatgaagtaaaaaagaattcagaatttcaatatcttttagtatctaagtataaatactaagaaaaaga
agaaagatggattgatttgcggcaatagatgtctttcacatacaactagaaaaaagtaatttcctttttgaatggcagttccaaaaaaacgtacttcgatgtcaaaaaag
cgtattcgtaaaaatctttggaagaaaaagacttatttttccatagtacaatcttattctttagcaaaatcaagatcattttctggcgtcagcgagcatccaaaaccaaagg
gtttttctcggcaacaaacaaacaaataatagggttttgggataatatgaattgacctatccccaaaaaaattccaattatttaatatgaataattaggaataattaggattaa
ttaatgagtttacttttatgtgtcgaattcctcggtacaatattcttagaacaaacctctctgatatataaaaaaagggtttttggtatactgtgacctaaatattcttttcctatc
aatgaacttttcgtaatagaatccgtataataaataaaaaaaggggattctattatgaaaagtagagtattcctgcaataagacttacaacttctacctatcttatcctaaat

taacaaaaaaattagttctatattgcaactgagaaaaaattgttccaactctttcaaagtttctattgggcaaagcaagaattttttgttaaaaaattcgcaacgatactac
caaacgaagtctattttaatgaagattctaatgtcctaaattctatggaatcttccaatctcgacgattcgcgagaaaataacttaatattcttttaataaacctgttatttcaa
cttagccgccatggtgaaattggtagacacgctgctcttaggaagcagtgctcaagcatctcggttcgagtccgagtggcggcagtctcgaaaaagaatacaatag
attataaaataaaatggattcaattcgaaatttccaattttgtaatgggaccttctccttatgctatttgcaactttagaacatatactaactcatatttctttctcaacaatttca
attgtgattacgattcatttaataaccttattagttcgtgaacttgggggattgcgtgattcgtcagaaaaaggaatgatagccactttttctgtataacaggattcttagttt
ctcgttgggcttcttcgggacattttccattaagtaatttatatgagtcattgatcttcctttcatgggctctgtatattcttcatatgattcctaagatacagaactctaaaaat
gatttaagcacaataactacgccgagtactattttaacgcaaggctttgccacgtcgggtcttttaactgaaatgcatcaatccacaatactagtacctgctctacaatct
cagtggttaatgatgcatgtcagtatgatgttactaagctatgcgactcttttgtgcggatccttattatccgccgctcttctaatgattagatttcgaaagaatttagatttct
tttcgaaaaagaagaaaaatgttttgcttaaaacatttttctttaatgagattgaatatttctatgcaaaaagaagtgctttaaaaagcaccttttttcctttatttccaaattatt
acaaatatcaattaattgagcgtttggattcttggagttatcgtgtcattagtctagggtttacccttttaaccataggtattctttgtggagcagtatgggctaatgaggcg
tggggatcctactggaattgggatcctaaagaaacttgggcatttattacttggaccatatttgcaatttatttacatagtagaacaaatccaaattggaagggtacgaa
gtcagcatttgtagcttccataggatttcttataatttggatctgttattttggtatcaatctattaggaataggtttacatagttatggttcatttacattacccatctaaatgatt
acataacataaaaccttaatgaaatggaaaaaacttccatttttgtgtttgatttgagaaccccttgaacgccttctcaaagggttctcaaaaattcgagatagatctaatt
agactcttttactttttctgaatttttagtatttccactatggaatatagagcggactagtagaagaaaaaaaatcctatttaggataataattggataacagagcctctac
cctgtcaacggatagcgagagaacaaaatctggataaataccgattcctattactggtaaaaagatacagattaaaagaaagagttctcgcgggccggaatcctca
aaattttcgtttggaacatgaaatagcttgtatccatagaacatctgtcgtaacatagataataaataaataggagttaatatcattccaattgccattacaaaagtaattag
catttttggcattaacagaaattttggactagtaattagtccaaaaaatactactaattccgcaacaaaaccactcattcctggtaaggcaagagaagccattgaaaagc
tactaaacatggtaaaaatttttggcattgggatagaaacccctcccagttcttcgagataaacaaggcgcattctatcacaagccgttcccgctaagaaaaaaagtgt
agccccaataaatccatgggataatatttgtaaaatagctccattgagtccaatgttggttatggaaccaattcctataataatgaaacccatgtgagagacggaggag
taggctattcttttttgaaattgcgttggccaagagaagttgaagctgcatagattatttgcatcgctcctattattactaaccaaggggaaaatagataatgagcatga
ggtaacaattccatattgatccgaatcaatccgtatgctcccatctttaataggattcccgctaaaagcatacatgtactgtaatgcgcttccccatgggtatctggtaac
cacgtatgtaggggtataatcggcaatttgacagcataagcaataaggaagccaaaataaaatagtatttccaatgttgcagggtatgattgattaattaatctttccaa
atctaatcttggttcgttggaaccgtataagcccatacctagaactccgattaagaaaaaaatggaaccacctgcagtatacaaaataaactttgtagctgaatagaga
cgcctcttcccccccacatggataaaagtaagtaaacaggaattaattctaactcccacatgataaaaaaaagtaaaaggtctcgcgaagaaaataatcctatttga
ccgctatacattgctagcatcaggaaatagaataatcgggaattccgggtaaccggccaagctgctaaagtagctaaagtagtcataaatcctgtcaataaaatagat
cctaatgaaagtccatcgattcccaatctccagtggaaattgaagacatctatccatttagaatcctcttttaattggattaagggatcctccaattggaaatgataacag
aatgcataagtcattagaaggaattctaataaacaaatagacatagtataccacctaacgattttgtttcccctatgaggtaaaaagaaaattaatgaacccgcaaatat
cggcaaaacaacaagtattgttaaccaaggaaaagaactcatgataaagtgataaagacaagatacgttttgaccagaaaagcccgtgctcgattattttgagcaca
ggcttcttcggtaaagaggaatcagacgattcaagtggaatttttgtaacgtatcaataagatagagccatgctgcgggttgtctcaggtcctaaataaacgcggac
acttaaaaaatctgttgggcaggcggattcgcatctcttacaacccacacaatcttcggttcttggcgcggaagcaatttgcttggctttacatccatcccaaggtatca
tttctaatacatctgttggacaagctcgtacacattgagtgcatcctatacatgtatcataaatttttacggaatgtgacattggatctataaattttccttttcaacataaaaa
ttttcgatctggtcaaaatgaaattagtactatatcaatcaaatgtattgtagacaccagacgaagcaatggtttatccaaacttcaacaaataatgcaatatatttcttaat
ccgtttgtgagaaagcatgaaaagagccaagagactttaattttgggcttcaacaatcataattatacgaattgtatatacgaattcgaattagccaataaattggctatc
gtcttttcaatataaattattgcaatattcaaattgcaatatcaatgaattgcaaaaattcaactaagtaaaaaagaatactatggaataacctactcaaaaaatagatattc
tcaaataataaatagtattcatgttaatatttcatattattattatatgtgtccctttgtttagaagattctatgtctaattattcaaaaaattagattgattgatacgagttgatttc
ctattacgatggatggaagaaagaatggataatccaatagctgcttcagcagccgcaagggctataacaaaaattgcgaaaatgtctccttttaattggcggctatca
aatagatcagaaaatgttacgagatttagattaattgaattcagtataagttcaaggcatattagagctctaaccatgtttcggcttgtgatcaatccatagataccaatcg
aaaataaatagacactcaaaaaaagtacatgctcaaacatcattaactaactccttatcaatctcgattcatttcaatatggggacaagaattgaaccgattgaattaatt
agaatagaacaattacacaacaaaagagaaaagaaggtatttgttggcagtagatgggttttactaaatcaaaattgtggttctttagtgatttattttagatttgaaattct
tataattttgactcattctaagtatttcttattgccgagccatagtaattgcacctattaaagaaactagaagaattatggaaatgagttcaaatggaagataaaaatcggtt
gctaaatgaatcccaatttgttgaacgttatttatgagaccctgttctactatttggtttgatcttgtagtccaaagaattccataccatgacgtatctgggatagtagtcatt
agtgaaaaaggaatagttatacaaacgagtgaagtgaacccatctccaatagtccaaaaattcttatctttagaccattctgagccatttacgaacattacggcaaatat
gatcaagacatttatagctcccacataaataagaagttgtgccacagctacaaagtaggaattcaataaaatatagaataaggatatacaaacaagaactaatcctag
cgaaaaagcagaaaaagttgggttggtaagtaataccacccctagacccccctagtagaagaacaaatcccccaaatagcacaagaatttcatgtattggcccaggt
aaatccattatggataagaagaaattaatagtataaatttttttcatgaactgactaaaactaaaagattcaaggaagaaaaaagggattaggaaattttttttgtatattgtat
ataagttctttctatagttagaaatcacatcacgaaaatctactctggtttaaaatcaggaataatttgcaataagcagtaggtattcgtttttctttctagttagtaaagaact
tttggattctaacaaaaaaaattctagtaatcagtaatcgttcttgaattccaagatttttcttcgtctattttactttgagttgaattcctaattgtttgaattgtgtaatctcccatt
atggagattggtaaccgactcaaagcaatttgattgtaattcaattcatgacgatcataagtagaaagttcatattcttcagtcattgataaacagtttgtcggacagtact
caacacaattaccacaaaatatacaaactccgaaatcaatactataattaagcaattgtttccttttaatatcctttttcaaatctccaatccacaagaggtagatctatagg
gcatacgcgaacacatacttcacaagcaatacatttatcaaattcaaagtggattcgcccccggaaacgctccgatgtaattgatttttcataggggtagtgaatcgtta
taggtaaacgatttgtgtgggataaggtaattatgaaactttgaccaatgtaccttgctgcgcgtattgtttgttgaccataactcatgaacccagttaccatagggaaca
tattcgaaatatctatgaaaaaggtatgtttctttctcttgtttgagaggacttttgtgttgaaaatattcttactgttattgtattatcttattttatagtgaaacaagttgggaag
aagttgttaataagagattgcccagggaaataggtaaaagaaatttccatccaagatttaataactgatccattctcatcctgggtaaagtccatcttattgtgatagaaa
tgaagagaaataaataagctttagttaatgtaataaagatactcattgtcatttctaaaattccaaccattttattcatttggaaaaatccaaaaaaggatatatagggaata
gacaaattccacccgcctaagtagagaactgttacaaataaagaggaaactaataaatttaggtaagaaacaagataaaataaaccatatttgataccagaatattcg
gtttgataacctgctactaattcttcctccgcttctggtaaatcaaagggtaatctttcacattccgccaaagaagaaattagaaaaaccagaaaacctataggctgacg

ccaaagattccatccaaaaaaaccatatttgactgtgcttcaactatatcaactgtacttgaactgttggataatcatagtcgacgataacatcacagttcccaccgcta
ttccaaaaccgtacatgaaaccttagtttcatacggctcctctatgatcagaaaaaaggaaagtactgtttcatttcgttattatcttcttgggcgtagttagaattatctaa
gataaaatcgatttcaacgtcctaaattagaccaaaggaattctgtctgctagaataataaaaaacgcttcggaattcatctcatcctttataatataatggtactttttcttt
gttcagcaataacttaatcttggaataaaacactcgttataacaattaataaacgaaaagagttgggtattagttcatgaagaattctgtatgaatatggataaacgacg
gaaagaataaataaagatctttttttttgtattgcattccatatcttttgtcctattcttctttccccgaggggtatttaaaaagaaaaaaggaataaagggttaattcgttcttg
atagccatttccttaacaagtgaatgggaacatactctggatcggaatccgaagaaagtactacttgctcatttccaccaatttcaagtccttattatgattccttttatgag
gaaaaatatctaatgctttagattccctcattactaatcctttatgtacttttagtgtttctaatccctcactaacttttgatggattccctttatgattacaactttctgtatcggga
atcccttattattgcccgcttcaagatatgatgactaatcaaaaaatctcaaccttggggtaaagaatttacaccgcttatgtttacttccatttttttcttgtacataggaaat
gagattttttctttttactacaaattaataagcagttttgtttcactcatatagctatctagtttaacttactaacctgaatatagaataagaaaaggaggataaatattcaatg
aatttcagaggaaaaagatcctattttaacgaatcgcacgtagagatattgctagtacacaaaaagttaatgggatttcataactaatagattgagcggcagctcgtag
accacctgaaaaagaatatttattatttgagctatatcctgccataagaagaccaataggagcaatacttgaaatggcaatccataaaaaaacaccaatactaagatcc
gctaaaacaaagcgatatcccaaaggaataactaaaaaacttaataaaattgatatgactgctatagacggtccaatgctaaataagggaatatcccctcgggatgg
caagatatcctctttaaaaagtagcttagttccatctgctatagcttgaagcagtcccagggggccagcatattcaggaccaatacgttgttgtatcgatgcggatattt
ctctttctaaccacacaattacgagtacttctattgtgattcccagtaagagggtcaaaatgggtagaatccatatcagtccatagacttcttttaataattccaagttcga
aaaagaattgatagcttctacctgtaccctgtctattatcatttcaacgatcaacttctcccataatgatatctatactacctaatatcgtcatgatatcagccaatttcattttt
ttgactagctgaggaagaatttgcaaattaataaaaccgggtggacgaattttccatctccaggggaaaagactatcatctcctaccagataaattcctaattcaccttttt
ggggcttccactcttgcataaagctcttgttttgacaattcaaaattgggtgaaggtttttaccaagaaatcgatattcaaaatcattccattcggaattctttgctttcttaa
agcgtcggacttctaaattctcataagggcccccaggaatttttctacagcctgttgaataattttgattgattccctcatttcaccgattcgtactaaatagcgtgctaat
gaatccccttctttttgccattggactttccaatcgaattgattgtaagactcgtaaagatcaactttacgaagatcccattgtattccagaagctcgtaacatggggccc
gataagccccaatttacagcttcttctccgctaataaaaccaactccctcaactcgttccaaaaaaaatgggattctgtgtaataagttgttgatattcaataactcctcgta
aaaaataatcacagaaatctaaacatttatcgatccatccataaggcagatcggcagcaactcctccgatgcgaaagtaattatgcatcattcgcatacctgtagcag
cttcaaatagatcatatattaattctctctctctaaaaatataaaaaaaaggagtctgcgcaccgagatctgccataaaaggtccaagccataacaagtgagaagctat
acggctcaattctaacataattaccctaatatagctggctctttggggtatttgaatattctccaagaattctggtgcatttaccgttattgcttctgtaaacatagtagctaa
ataatcccaacgtgttacataaggtaagtattgtataatagttcggttttccgcgatttttttccattcctctgtgtaaatagcctaatatgggttcacaatcaataacatcttca
ccatcgagagtaacgatcagtcgaagaacaccatgcattgatgggtgctgagggcccatattgactatcatgagatcttttcttgtaagcggtagactcatatcctttct
tccttaattcattattccatgaaaatggattattccatgaattcctcaaaacgaggctcatcaaaatgcaaaatctaagactactataagactactaataaaataagaaaa
aaaattcgaacgatgaaattaccgctccctaatatccaactgactgattaatttcttataacgtactctatttttctttgccaaataagccagcaaacgttgacgttttccca
aaagtcttcggagacctctttccgatgaaaaatctttttttgtgtaattccaaatgtgaagcaagtctccgtatcttattggtgaaactgaatacttgaaattcaacagaacc
ccagttttcttctttttcttctttaaccataaatccaaaaatttttctacctcctttctttttcatgtatttttctgatcaggaaaaataaaaaattatgtcagttattttgaagttattct
aatctcgtacacacaaaaatttgcaattattcatctactactggaatttggatttattttatcgatgcaaattggatttggatagaagggtacattcttttattttagatagaag
aaaagtttcttctatctaaaataaaagaattttgctgatttatttattgctatatccaatttatggaattgatacaccgtttaattgatatcatttagcaaaatgaaacatagcat
atgcatccatctttcggctcgagaatttcacgggatagagatatggtataagaaataggctattaagtaactctaaatgaattgtggatacatctgtatccttaacatact
gaaacaactgccattattcgtatcaaaccaatagcgattcatacaagttaaatcttctaatcaatggtgggccaataatgaattttttttgcatatgtattaagacgcttggc
ctgatttcgaaattgtccagagttttttatgtagttttcattgcaaaatgatggatctccttccgtaactttccaattacgagtacgagaattgaaagacatgaaaattctcaa
ttctctacggcgtctaggagatagatagaatattttcaggaacaagaaaaccagaagaatctttctctctattcactaccattccgcgtcttcgacttctattagtttcttttc
ttctttaatgcaatagctatagtttgatatagaatccatttctcaaagtaatggaaaccttttctcttataggaaatggttcgaaaatcgctattccaccttttaggtatcgtgaa
aagtgatacctgtgaagatcgtgcatttcagtcacattcagatccgttttttgagtccatgatataaccaaattggatggatcttccacccgtttagctaagaaagaatag
atgcagaggtggataatagatcgatatgaagatcatgagctgccccataatgaaaccaccaggagtcgcgaatatctccttcttccctaatccaagattggagaaag
aagatctaagagggacccatggagaatgtggtcagaaacccataatagagtccgaccgcaacgaccgaattaattatcctcatcgagaaacttagactactaagta
gaaaagatttgaaaatcatggcatgggtctcctttttttcttctttagagttttctatatgcacaatttctcgatgtttcgatgagaatttcttgactttccatatatagaaagag
atagactataaatgacatctcttatgtcaataagaccaaagggatggatattaaatgataggaagtgctaggaagtgaaatagaatgaaatagagccactctgggctt
acctatgaaatgaggcatggaacggagccactacgaagaaattccgggagttacgaaagaagcttcggactcatattgttcatgggttgagagcgggagttgaact
ctaggaggtcgaatccccccttgttcctcagtagctcagtggtagagcggtcggctgttaactgactggtcgtaggttcgaatcctacttggggagatttgattcattcttt
aatgtaagaataaagaattgaattaaagggcttgctttgacccttaggagtaggtaacccgttcgctatccttgtttctattgcattttatctcatcgtatcacattctgttcta
cgattccacttcgacaaaaggaaagagcatacccaagttcaatagctttacgtccgctatcccgatcatggttttcctaccctcagggggaaagtaaaggcccttccc
ctttggaaggctgtgggcgaggagggattcgaacccccgacaccgtggttcgtagccacgtgctctaatcctctgagctacaggcccaccccgtctccactggatc
tcttcccgggggtacccccaaaaggaacctccctctcctcagccatttcatttcgggttaagaagatgggaaagccccttttctctctataagaacagtgcgttccga
ggtgtgaagtgggagagaggggatgtgatgattgaggttttgaataagacgacctttgcattttggatttggatctttttcgtatttcaaaatagtgaaaaagtcaaataa
gaggtgttaagctttttatcattctggcatcgagctattttgccgcaggacctcccctacagtatcgtcaccgcagtagagtttaaccaccaaattcgggatggattggt
gtggttcctctacgcctaggacaccagaatatcgaaccatgaacgaggaaaggcatgagagaaatattggctagtaattgtgaagccccaattcttgactggaagg
gacaccaaaggcctctgccctccctctctatctatccaagagatggaagggcagagcttttttttggtttttcatcttttcatcaaagagttgaacaatgaagatagatg
gcaagtgcctgatcgatttgatcaggtcgtgtaggaacaaggttcaaatcgttcgttcgttaggatgcctcagctgcatacatcactgcacttccacttgacacctattt
aaacggctcgtctcgccgctaccttatcctatttccatgcttctgtcgctccatccccgtatgggtggagaacccgtcgctgtctcggctgtgctaccggaggctctag
ggaagtcggaggagagagcactcatcttggggtgggcttactacttatatgctttcagcagttatcctctccgcacttggctacccagcgtttaccgtaggcacgata
actggtacaccagaggtgcgtccttcccggtcctctcgtactagggaaaggtcctctcaatgctctaacgcccacaccggatatggaccgaactgtctcacgacgtt
ctgaacccagctcacgtaccgcattaatgggcgaacagcccaacccttggaaccacctacagctccaggtggcgaagagccgacatcgaggtgccaaaccttcc

cgtcgatgtggactcttggggaagatcagcctgttatccctagagtaacttttatccgttgagcgacggcccttccactcggcaccgtcggatcactaaggccgactt
tcgtctctgctcgacgggtgagtcttgcagtcaagctcccttctgcctttgcactcgaggaccaatgtccgtctggcccgaggaaacctttgcacgcctccgttacctt
ttgggaggcctacgccccatagaaactgtctacctgagactgtcccttggcccgcgggtctgacacaaggttagaatccgagctcttccagagtggtatctcactga
tggctcgggcccccccggaagggggccttcttcgccttccacctaagctgcgcaggaaaggcccaaagccaatcccagggaacagtaaagcttcatagggtcttt
ctgtccaggtgcaggtagtccgcatcttcacagacatgtctatttcaccgagtctctctccgagacagtgcccagatcgttacgcctttcgtgcgggtcggaacttacc
cgacaaggaatttcgctaccttaggaccgttatagttacggccgccgttcaccggggcttcggtcgccggcttccctgtcatcagttcaccaacttccttgaccttccg
gcactgggcaggcgtcagcccccatacatggtcttacgactttgcggagacctgtgtttttggtaaacagtcgcccgggcctggtcactgcgaccccccttttgcgag
ggggcacccccttctcccgaagttacggggctattttgccgagttccttagagagagttgtctcgcgcccctaggtattctctacctacccacctgtgtcggtttcgggt
acaggtacccctttgttgaaggtcgttcgagcttttcctgggagtatggcatgggttacatacttcagcgccgtagcgcctggtatgagcctcgtggagaagcaatgg
ctagtccacggggctcatacttcagcgctgcagcgcttggtactcggacctcggctcgaggcattttctctacccccttcttaccctgaaaaagcagggtcaccttgtgt
ccttaaacctataaccatctttcggctaacctagcctcctccgtccctccgtaccaacaaggggtagtacaggaatattgacctgttgtccatcgactacgcctttcggc
ctgatcttaggccctgactcaccctccgtggacgaaccttgcggaggaaaccttgggttttcggggcattggattctcaccaatgttttcgttactcaagccgacattct
cgcttccgcttcgtcgacccccgctttcgcggttgcttccctctaaggcggaacgctcccctaccgatgcattttgacatcccacagcttcggcagatcgcttagccc
cgttcatcttcagcgcaagggcgctcgatcagtgagctattacgcactctttaaagggtggctgcttctaggcaaacctcctggctgtctttgcacccccacctccttta
tcactgagcggtcatttaggggccttagctggtgatccgggctgtttccctctcgacgatgaagcttatcccccatcgtctcactggccgaccttgaccccctgttatttt
gggtcatatctagtattcagagtttgcctcgatttggtaccgctcgcgcagcccgcaccgaaacagtgctttaccccctagatgtccagtcaactgctgcgcctcaacg
catttcggggagaaccagctagctctgggttcgagtggcatttcacccctaaccacaactcatccgctgattcttcaacatcagtcggttcggacctctgcttagtttca
tccaagcttcatcctggtcatggatagatcacccaggttcgggtccataagcagtgacaatcgccctatgaagactcgctttcgctacggctccggtgggttccgttc
ccttaaccaagccactgcctatgagtcgccggctcattcttcaacaggcacgcggtcagagatcactttcccctcccactgcttgggagctcagcacggtttcacgtt
ctatttcactacccactggggggttcttttcacctttccctcacggtactacttcgctatcggtcacccaggagtatttagccttgcaaggtggtccttgctgattcacacgg
gattccacgtgccccatgctactcgggtcagagcgtaagctagtgatgctttcggctactggactttagccatctagggtgcggcactcaaccgcttcgcctagcag
cacaacgcttgtattgctctcccacaaccccgttttcacggtttaggctgctcccatttcgctcgccgctactacgggaatcgcttttgctttcttttcctctggctactaag
atgtttcagttcgccaggttgtctcttgcctgctcatggattcagcaggcagttcaaaaggttgacctatttgggaatctccggatctatgcttattttcaactccccgaag
catttcgtcgcttgctacgcccttcctcgtctctgggtacctaggtatccaccgcaagcctttcctcttttgaacctcgccattaacgttaaggctatgccatcctaaggtg
ctactaaatggaaggatcttatcaacgtccatgaatgcgaaatcatagatcgaactgccgaattggcaaaattcggtgctatcatagtatccgctaagttcacgggctg
gagataagcggactcgaaccgctgacatccgccacagggtaaaccaccgcctctcaggcctccccgacgggttctaccatagaggccaacgatagacaataact
ccccccgaacacagcttacaactttcatcgtactgtgctctccaaagagcaactcttctcaaaatctcaaaacaaaaggtgctgagttggaatcccattctaaggatt
cttgtggttccggggaatccagctacaggagaaccaggaacggggagctctccccttttccgcccgactctttgatcttaaacttaagaatgctggttttaagaacga
gtgattgcccttctccgacccttactgcccaaccggagagcggacggctaatgtgttccacttattgaacagggtctatggtcggtccgtgaccccctggacgccgaa
ggcgtccttggggtgatctcgtagttcctacggggtggagataatggggtcggtccatggattttcttccttttgccacatttcgctcaaagggttgaagggagatag
tgcatcaagctattcgcaagggccaacttgatcctcttccccagggatcccagatgagggaagcctaggagagccgccgactccaactatcgtccatgtacgatcc
atactagatctgaccaactgcccatcctacctcctctacctttttgacagcccatctttttgtctcagtagagtctttcagtggcatgtttcagtcctcttccccattacttaga
aaaagtgagccaccggttcaggtacaagatactaccattaccgcctggacaattagacagccaacccgtaatcgcaacgacccaattgcaagagcggagctctac
caactgagctatatccccccgagccaagtggagtatgcatgaaagagtcagatgcttcttctattcttttccctggcgcagctgggccatcctggacttgaaccagag
acctcgcccgtgaagtaaatcatcgcccctacgatccaaccaattgggagagagaatcaatagactcctttttcgggagcgattcatccttcccgaacgcagcatacaac
tctccgttgtactgcgctcttcaagtgtgcttcttcccctttctcccccttaccatggcaagtccttgggaaataactccgatgggcagaaaaaggaaggcgttaagaga
ccctcctggcccaaccctagacactctaagatcctttttcaaacctgctctgctcccatttcgagtcaagagatagataaatagccacatcccattgcactgatcgagg
gcgctcgtagtgacttagggagtcgaagaccaagaagtggcttatttataccaagcattcctcttatggctagatccaacctcctggtccctgcggaaaggaaaaag
aatttcacgttcttcctttcaggaagggaggattagggaagtcctattgattgctgctttctccagaccgccgggaaaagcatgaaaaaaaaggctcgaatggtacgat
ccctccgtcaccccagaatgaaagggtgatctcgtagttcttggtctgtgaagatgcgttgttaggtgctccattttcccattgaggacgaacctcaacctgtgctcga
gagatagctctccatacactgataagggatgtatggattctcgagaagagaggagccgtagtgcccccccggaccgcccggatcccacgagtgaatagaaagt
tagatctacatgggatctcacctgaatcgccccatctatcctcctgaggagaagtttgtttggtttcaaactccgattcaaacaggaggagtacgccatgctaatgtgc
cttggatgatccacatcttcgggtcaggcgctgatgagcacattgaactatccatgtggctgagagccctcacagcccaggcacaacgacgcaattatcaggggc
gcgctctaccactgagctaatagcccgtcgcgcgggcctccctttgggaggcctgctacgccaaaagcgagaaaaactccatccctttcctttttgacatccccatgc
cgccacaccacacgggggggggcatggggacgtcaaaaaggggatcctatcactatcaactaatttgttccgacctaggataataagctcatgagcttggtcttactt
caccctaaacgaaagaagacttccatatccaagtttagctcagacgtagctgccttcttttttgggcgtgaagcagtgtcaaaccaaaatacccaataagcataagcatt
agctctccctgaaaaggaggtgatccagccgcaccttccagtacggctaccttgttacgacttcactccagtcgcaagcctagccttaggcatccccctccttacggt
taagggtaatgacttcaaacatggccagctcctatagtgtgacgggcggtgtgtacaaggcccgggaacggattcaccgccgtatggctgaccggcgattactag
cgattcctgcttcatgcaggcgagttgcagcctgcaatccgaactgaggacgggtttttggagttagctcaccctcgcgagatcgcgaccctttgtcccgcccattgt
agcacgtgtgtcgcccagggcataaggggcatgatgacttggcctcatcctctccttcctccggcttaacaccggcggtctgttcagggttccaaactcatagtggc
aactaaacacgagggttgcgctcgttgcgagacttaacccaacaccttacggcacgagctgacgacagccatgcaccacctgtgtccgcgttcccgagggcacc
cctctctttcaagaggattcgcggcatgtcaagccctggtaaggttcttcgctttgcatcgaattaaaccacatgctccaccgcttgtgcgggcccccgtcaattccttt
gagtttcattcttgcgaacgtactccccaggcgggatacttaacgcgttagctacagcactgcacgggtcgagtcgcacagcacctagtatccatcgtttacggcta
ggactactggggtctctaatcccatttgctcccctagctttcgtctctcagtgtcagtgtcggcccagcagagtgctttcgccgttggtgttctttccgatctcaatgcatt
tcaccgctccaccggaaattccctctgcccctaccgtactccagcttggtagtttccaccgcctgtccagggttgagccctgggatttgacggcggacttgaaaagc
cacctacagacgctttacgcccaatcattccggataacgcttgcatcctctgtcttaccgcggctgctggcacagagttagccgatgcttattcctcagataccgtcatt

gtttcttctccgagaaaagaagttgacgacccgtgggccttccacctccacgcggcattgctccgtcaggctttcgcccattgcggaaaattccccactgctgcctcc
cgtaggagtctgggccgtgtctcagtcccagtgtggctgatcatcctctcggaccagctactgatcatcgccttggtaagctattgcctcaccaactagctaatcaga
cgcgagcccctccttgggcggatttctccttttgctcctcagcctacggggtattagcaaccgtttccagttgttgttcccctcccaagggcaggttcttacgcgttactc
acccgttcgccactggaaacaccacttcccgttcgacttgcatgtgttaagcatgccgccagcgttcatcctgagccaggatcgaactctccatgagattcatagttg
cattacttatagcttccttattcgtagacaaagcggattcggaattgtctttccttccaaggataacttgtatccatgcgcttcagattattagcctggagttcgccaccag
cagtatagccaaccctaccctatcacgtcaatcccacaagcctcttatccattcccgttcgatcgtggcggggggagtaagtcaaaatagaaaaaactcacattggg
tttagggataatcaggctcgaactgatgacttccaccacgtcaaggtgacactctaccgctgagttatatcccttccccgtcccctcgagaaagagaattaacgaatc
ctaaggcaaaggggcgagaaactcaaggccacccttcctccgggctttctttccacactattatggatagtcaaataatgggaaaaattggattcaattgtcaaccgg
tcctatcgaaaataggattgactatggattcgagccatagcacatggtttcataaaatctgtacgattttcccgatctaaatcgagcaggtttccatgaagaagatcttgt
tcagcatgttctattcgatactggtaggagaagaacccgactcggtattcttaaaaaaagaggggaagcagaaccaagtcaagatgatatgggtcgccccttcttctt
gcgccaaagatcttaccatttccgaaggaactggggctacatttcttttcaatttccattcaagagtttctatctgtttccacgccctttttgagacctcgaaacatgaaat
ggacaaattccttctcttaggaacacatacaagaaaaaggataatggtagccctcccattaactacttcattttcatttatgaatttcatagtaatagaaatccatgtccta
ccgagacagaatttcgaacttgctatcctcttgcctaataggcaaagattgacctctgtagaaagactgattcattcggatcgatatgaggacccaactacgttgcatt
gccagaatccatgttccatatttgaagagggttgacctctgtgcttctctcatggtacaatcctcttcctgctgagcccccttctcctcggtccacagagaaaaaatgg
aggactggtgccgacagttcatcacggaagaaagaactcacagagccgggatcgctaactaatagaatagtactactaactaatactaatatatagaaatagatatc
tagaaatagaaacgaactaatatatagataatcgaaattgaaaagaactgtcttttctgtatactttccccgttctattgctaccgcgggtcttatgcaatcgatcggatca
tatagatatcccttcaacacaacataggtcatcgaaaggatctcggacgactcaccaaagcacgaaagccagttagaaaatggattcctatttgaagagtgcctaac
cgcatggataagctcacattaacccgtcaattttggatccaattcgggatttttcttgggaagtttcgggaagaaattggaatggaataatatagattcatacagaggaa
aaggttctctattgatgcaaacgctgtacctagaggatagggatagaggaagagggaaaaatcgaaatgaaataaataaagaataaagcaaaaaaaaaaataagtc
gaagatagaagagcccagattccaaatgaagaaatggaaactcgaaaaggatccttctgattctcaaagaatgaggggcaaggggattgataccgagaaagattt
cttcttattataagacgtgatttgatccgcatatgtttggtaaaagaacaatcttctcctttaatcataaatggaaagtgttcaattagaacatgaaaacgtgactcaattgg
tcttagttagtcttcgggacggagtggaagaagggcggagactctcgaacgaggaaaaggatcccttcgaaagaattgaacgaggagccgtatgaggtgaaaat
ctcatgtacggttctgtagagggacaggaagggtgacttatctgtcgacttttccactatcaaccccaaaaaacccaactctgccttacgtaaagttgccagagtacg
attaacctctggatttgaaatcactgcttatatacctggtattggccataatttacaagaacattctgtagtattagtaagaggaggaagggttaaggatttacccggtgt
gagatatcgcattattcgaggagccctagatgctgtcgcagtaaagaatcgtcaacaagggcgttctagtgcgttgtagattcttatccaagacttgtatcatttgatga
tgccatgtgaatcgctagaaacatgtgaagtgtatggctaacccaataacgaaagtttcgtaaggggactggagcaggctaccatgagacaaaagatcttctttctaa
agagattcgattcggaactcttatatgtccaaggtcaatatggaaattctttcaggggttttcccttactttgtccgtgtcaacaaacaattcgaaatacctcgactttttca
gaacaggtccgagtcaaatagcaatgattcgaagcacttctttttccattacactatttcggaaacctaaggacttgatggtatggatatggaaaatacaggatttccga
tcctagcgggaaaaggagggaaacggatactcaattttaagtgagtaaactgaattccatactcgatctcatagatccctatagaattctgtggaaagccgtattcgat
gaaagttgtatgtacggcttggagggagatctttcctatctttcgagatccaccctacaatatggggtcaaaaagccaaaaaaataagtgattcgtttttagcccttataa
aaagaaaacggattcttgaacctctttcacgctcatgtcacgtcgaggtactgcagaaaaaagaactgcaaaatccgatccaatttttcgtaatcgattagttaacatg
gtggttaaccgtattatgaaagacggaaaaaaatcattggcttatcaaattctctatcgagccgtgaaaaagattcaacaaaagacagaaacaaatccactattggtttt
acgtcaagcaatacgtagagtaactcccaatataggagtaaaaacaagacgtaataaaaaaggatcgacgcggaaagttccgattgaaataggatctaaacaagg
aagagcacttgccattcgttggttattagaagcatcccaaaagcgtccgggtcgaaatatggctttcaaattaagttccgaattagtagatgctgccaaagggggtgg
gggtgccatacgcaaaaaggaagcgactcatagaatggcagaggcaaatagagctcttgcacattttcgttaatccatgaacagaatctaggtatgtagacacatg
gatccatacatctcgatcggaaaagaatcaatagaaggagaatcggacgatatctttctcgaaacaaacaaaaaggaaaagaaagagaaaacagaaatcatgatc
aactaagccctctcgggggcttgcttaagaataagaaagaggaatcttatggaaatagcatggaataaggtttgatcctattcatggggattccgtaaatatcccattc
caaaaatcgaaacaatcgggacttttcggagattggatgcagttactaattcatgatctggcatgtacagaatgaaaacttcattctcgattctacgagaatttttatgaa
agcgtttcatttgcttctcttccagggaagtttcattttcccagaatgtatcctaatttttggcctaattcttcttctgatgatcgatttaacctctgatcaaaaagatagacctt
ggttctatttcatctcttcaacaagtttagtaataagcataacggccctattgttccgatggagagaagaacctataattagcttttcgggaaatttccaaacgaacaattt
caacgaaatctttcaatttcttattttattatgttcaactttatgtattcctctatccgtagagtacattgaatgtacagaaatggctataacagagtttctgttattcgtattaac
agctactctaggggggaatgtttttatgtggtgctaacgatttaataactatctttgtagctccagaatgtttcagtttatgttcctacctattgtctggatataccaagagaga
tctacggtctaatgaggctactatgaaatatttactcatgggtggggcaagctcttctattctggttcatggtttctcttggctatatggttcatctgggggggagatcgag
cttcaagaaattgtgaacggtcttatcaatacacaaatgtataactccccaggaatttcaattgcgcttatatccatcactgtaggacttgggttcaagctttccccagcc
ccttttcatcaatggactcctgacgtctacgaaggagtgtggttcgttcgacaaattcctacctctatatctatctctgaggtgtttgggttttgcaaaactccatagatatg
cagaagagaaatgctatccccactccgaccaagacagaacttttaccaaaagtttattgtgatctttttgttcaaataacaattaaggtgaagcagggtcaggaacaac
gaatctctttatgataaacagatccattttgcaagctcgttattacgggtagttcctacaaagaatcggactaatgacgtatacaatgcttgaattatcgacgtagatgcta
catagtgggttctcatccttcagagactacgagtgtaataggagcatccgttgacaaaaggatcaccctaagatgatcatctcatggctattgggaacgaatcaaatc
agatggttctatttctcaacctttctgacttgctcctacggaaccaaggtcgaaaggattgaaaaagtcagtcattcacaaccactgatgaaggattcctcgaaaagtta
aggattagtagttctttttcgaaatcgatttcgaaaaagaatggattcggtcttatacatacgcgaggaaggtaatcaaaaaagaaagaagacaagttcttctttcttttat
cacttaggagccgtgcgagatgaaagtctcatgcacggttttgcatgagagaaagaagcgaggaatcctcttttcgactctgactcccccactccagtcgttgcttttc
tttctgttacttcgaaagttgctgcttcagcttcagccacgcgaattctcgatattcctttttatttctcatcaaacgaatggcatcttcttctggaaatcctagctattcttagc
atgatattggggaatctccttgctattactcaaacaagcatgaaacgtatgcttgcatattcgtccatagggcaaatcggatatgtaattattggaataattgttggagact
caaatgatggatatgcaagcatgataacttatatgcttttctatatctccatgaatctaggaacttttgcttgcattgtattatttggtctacgtaccggaactgataacattc
gagattatgcaggattatacacgaaagatcctttttggctctctctttagccctatgtctcttatccctaggaggccttcctccactagcaggtttcttcggaaaactctat
ctattctggtgtggatggcaagcaggcctatatttcttggtttcaataggactccttacgagcgttctttctatctactattatctaaaaatagtcaagttattaatgactgga

cgaaaccaagaaataaccccttatgtgcgaaattatagaagatccccttaagatcaaacaattccatcgaattgagtatgactgtatgtgtgatagcatctactatacc
aggaatatcaatgaaccccattcttgcaattgctcaggataccctcttttagctgctaggtctatttcttagttcaagatccctcttactaactggaataaaagaattagtag
atctgttccgcccaaaatgggaatgggcgctagggtaatgaacttataatcatggaatcgactcgatcatcagattataagttcattccataccggaccagaccgtgc
acattcttattatgagaaggggtcattcgagcctatggaaataggatactctgtttacatagaaatccccacgtccttacattctatttaggattaggaataggtgtaatca
gacctgcttttgacatatctatcctatccttatttgggtaccatatgcacctctttgggcttctattgaatcgagaaattggattgtacatctttttgattttgatacatataaggt
gtcctacggataatgcaaatcgaagctatttgatgtctgactcaggcctatatgaccgatcgatcgaaatactccaagactccacctttgtcatatattccatatatcaca
ttagatagatatcatattcatggaatacgattcactttcaagatatcacattagatagatatcatattcatggaatatgattcactttcaagatgccttgatggtgaaatggta
gacacgcgagactcaaaatctcgtgctaaagagcgtggaggttcgagtcctcttcaaggcataatacggagaatgcgcattcaatgagcattccccgtagaagtatt
ccggaaatctgcgcctggcgctctcctctatcttctgaggtccttaaccacttccctgagaaaaggagacagtaaaagccaaaatagactaaatatagcctgaacga
tcctaaaaatccctcgaaggagataatataataaagaacccaaagcagacggtatcctactgcaagggtggtcttaagaatccaaaagaggttgctcagaagagat
agatgtatcccaacctctattgctctcgcgtaaagcctttttttacgcgacaggaaaaagtgactacgaattccccttttgtttgcgaatccctgtttgtatcctttgagc
gcacgcccataagtagcgatcaaggaaatcgatcaaacgatcccaataccgtgaaagagaaacttcccagatccagggaagcttatcataaagggcttcgacaag
gggttttattcgttctttgagcaaaaagataaagatcggatagattcgaaccgcaattgcaaaggtaatcactactatgccagcccaaatcatgatcttcaccaacttgg
atttggttctctcgcgaaaatcgcgagttgcagagatgagaaccatgaaaagcaagatcccgaataagaaaacagaaaccgaggaaaccacaagagtgaagact
agtagagtctcgtcttttgtcattctttgctcctttttcactcaatgattcattcgaatttcccgaccaaaaattctatatgtctattcatagggcctcgttgctaagtgctacaa
gatctagtgcactggaactcgtggttatggacccgaatcctttagtatggaacattgtcttttccaagtaaaaacccccagtatatgaaagaatgaaaaggtgctttcgt
tcttttcttgtggaataagaagccctcgtaccttaatgaaaggaaaataggaatttttcattaggtatttgaccaaataggatcgtccagttcctatagaacctatcactaa
aatacccgatagggctaagcggaacgaaaaggattttccctgagatggtaaatgaaaacgattagccccatacgaggtttgggaataagtgatgagcaaggaatat
acgtctttctgctaaagaggatctattaaactcataattcattagatccttgttatcaatgtcaactaggtatcataagtaaacggatcccggttgttcaatcctttgataacc
aaggtcattctttgctaaagagaaatgatcactatgagtcagactcaatagaattggatccattccaaatagcgagaattaggattctggatccctctcaatctctctttc
aattcgaggatccagagaggtgttttcatagtcatctccgaatatttgccatctccgaatattttcgatttcatttttctatgatatgtctttctatatgaaaattggttatttacg
atgtacgatgatccctgttaagcatccatggctgaatggttaaagcgcccaactcataattggtaaatttgcgggttcaattcctgctggatgcacgcgaaccggaac
gttccataagtctattggaactggctctctatccatggaatctcatccatcatccatacataacgaattggtatggtatattcataccataacataagaacaataagaactc
gaattcttatcgatactggaactcagagcataggagggaaagtcgatttatggatggaatcaaatacgcagtatttacagaaaaaagtcttcgtttattgggaaagaat
caatatacttttaatgtcgaatcgggattcactaagacagaaataaagcattgggtcgaactcttctttggtgttaaggtagtagctgtgaatagccatcgactacccgg
aaagggtagaagaatgggccctattctaggacatacaatgcattacagacgtatgatcattacccttcaaccgggttattctattccacttctagatagagaaaaaaac
taaaggagaatacttaataatacggcgaaacatttatacaaaacacctatcccgagcacacgcaagggaaccatagataggcaagtgaaatccaatccacgaaata
atttgatccatggacggcaccgttgtggtaaaggtcgtaattccagaggaatcattaccgcaaggcatagagggggaggtcataagcgcctataccgtaaaatcga
ttttcgacggaatcaaaaagacatatctggtagaatcgtaaccatagaatacgaccctaatcgaaatgcgtacatttgtctcatacactatggggatggtgagaaggg
atatattttacatcccagaggggctataattggagatactattgtttctggtacaaaagttcctatatcaatgggaaatgccctacctttgagtgcggtttgaactattgattt
acgtaattggaagtaaccaattaggtttacgacgaaacctagaaatcgatcactgatccaatttgactacctctacgggatagacctcaacagaaaactgttgagtaa
cggcagcaagtgattgagttcagtagttcctcatagaaaattattgactctagagatatggtaatatggagaagacaaaattgtttgaagcacgcacagaaccggaa
gcgccccttgtttcaaagagaggaggacgggttattcacatttaatttgatggtcagaggcgaattgaaagctaagcagtggtaattaagacccccgggggaaaata
gggatgtctcctacgttacccataatatgtagaagtatcgacgtaatttcatagagtcattcgatctgaatgctacatgaagaacataagccagatgacggaacgcgg
agacctaggatgtagaagatcataacatgagcgattcggcagatttggattcctttcctatatatccactcatgtggtacttcatcatacgattcatataagatccatctgt
ctagagatcgtcatatacatctagaaagccgtatgctttggaagaagcttgtacagtttgggaaggggtttttttgagagaaaagaagaatctacttcaaccgatatgcc
cttaggcacggccatacataacatagaaatcacacgtggaaggggtgggcaattagctagagcagcaggtgctgtagcgaaactgattgcaaaagaaggtaaat
cggccactttaagattaccatctggggaggtccgtttggtatcccaaaactgcttagcaacagtcggacaagtgggtaatgttggggtgaaccaaaaaagtttgggt
agagccgggtctaagtgttggctaggtaaacgccccgtagtaagaggggtagttatgaaccctgtggaccacccccatgggggcggtgaagggaaagcccccca
ttggtagaaaaaaacccacaacccccttggggttatcctgcgcttggaagaagaactaggaaaaggaaaaaatatagtgatagttttattcttcgtcgccgtaagtaaat
acgtaactaggaatatggaaaattgcatttttggaatttgcaataatgggatgggcgaacgacgggaattgaacccgcgcatggtggattcacaatccactgccttga
tccacttggctacatccgccccttatccagctaaaggattttctctttttccattcatcattattctatttattctgacctccatacttcgatcgagatattggacatcgaatgc
cactctttaaaatggaaaaaaaggagtaatcagctgtgacacgaaaaaaaacgaatccttttgtagctcatcatttattggcaaaaatagaaaaggtcaatatgaagg
aggagaaagaaacaatagtaacgtggtcccgggcatctagcattctacccgcaatggttggccatacaatcgcgattcataatggaaaggaacatatacctatttac
ataacaaatcctatggtaggtcgcaaattgggggaattcgtaccaactcggcatttcacgagttatgaaagtgcaagaaaggatactaaatctcgtcgttaactgaatt
cagaatagaaagattcaaaataaaaaaaaaaaaaagaaata
>O.rufipogon_AC011002323_cp
cccaatatcttgctgaagcaagatattgggtatttctagctttcctttcttcaaaaattgctatatgttagcagaaaagccttatccattaagagatggaacttcaagagca
gctaggtctagagggaagttgtgagcattacgttcgtgcattacttccataccaagattagcacggttgatgatatcagcccaagtattaataacgcgaccttggctat
caactacagattggttgaaattgaatccgtttagattgaaagccatagtactaatacctaaagcagtgaaccaaatccctactacaggccaagcagccaagaagaag
tgtaaagaacgagagttgttaaaactagcatattggaagattaatcggccaaaataaccatgagcggccacaatattataagtctcttcctcttgaccaaatctgtaacc
ctcattagcggattcgttttcagtggtttccctgatcaaactagaggttaccaaggaaccatgcatagcactgaatagggaaccgccgaatacaccagctacacctaa
catgtgaaatggatgcataaggatgttatgctctgcctggaatacaatcataaagttgaaagtaccagatattcctaaaggcataccatcagagaaacttccttgacca
atagggtaaatcaagaaaacagcagtagcagctgcaacaggagctgaatatgcaacagcaatccaaggacgcatacccagacggaaactcagttcccactcacg
acccatataacaagctacaccaagtaagaagtgtagaacaattagctcataaggaccgccattgtataaccactcatcaacagatgcagcttcccaaattgggtaaa
agtgcaatccgatcgccgcagaagtaggaataatggcaccagagataatattgtttccgtaaagtaaagaaccagaaacaggctcacgaataccatcaatatctact

ggaggggcagcgatgaaggcgataataaatacagaagttgcggtcaataaggtagggatcatcaaaacaccgaaccatccgatgtaaagacggttttcggtgcta
gttatccagttgcagaagcgaccccacaggcttgtactttcgcgtctctctaaaattgcagtcatggtaagatcttggtttattcaaattgcaaggactcccaagcacac
gtattaactagaaagataatagaaggcttgttatttaacagtataatatagactatataccaatgtcaaccaagccagccccgacagttgtatatccatacaacaaaattt
accaaaccaaaaaattttgtaaatgaagtgagtgaaaaatcaaaactcagattgctcctttctagtttccatatgggttgcccgggactcgaacccggaactagtcgg
atggagtagataattattccttgttacaatagagaaaaaacctctccccaaatcgtgcttgcatttttcattgcacacgactttccctatgtagaaataggctatttctattcc
aaagaggaagtctactaatttttttagtagtaagttgattcacttactatttattatagtacagagaacatttcagaatggaaactgtgaaagttttaccttgatcatttatcaa
tcatttctagtttattagttttgtttaatgattaattaagaggattcaccagatcattgatacggagaatatccaaataccaaatacgctcactgtgcgatccacggaaaga
aaagtaagttgttttggcgaacatcaaagaaaaaacttgctcttcttccgtaaaaaattcttctaaaaataccgaacccaaccattgcataaaagctcgtaccgtgctttt
atgtttacgagctaaagttctagcgcatgaaagtcgaagtatatactttagtcgatacaaagtcttctttttttgaagatccactgtgataatgaaaaagatttctacatatcc
gaccaaaccgatcaagaatatcccaatccgataaatcggtccaaattggtttactaataggatgccccgatccagtacaaaattgggcttttgctaaagatccaatga
gaggagtaacagggactttggtatcgaatttttttcatttgagtatctattagaaatgaattctccagcatttgattccttactaacaaagaatttattggtacacttgaaaagt
accccagaaaatcgaagcaagagtttgctaattggtttagatggatcctttgcggttgagtccaaaaagagaaagaatattgccacaaacggacaaggtaacatttc
catttcttcttcaaaagaagagttccttttgatgcaagaattgcctttccttgatatcgaacataatgcataaggggatccataacgaaccatatggttttccgaaaaaaa
gcagggtacattaacccaaaatgttccatcttcctagaaaagatgattcgttccagaaaggttccggaagaagttaatcgcaagcaagaagattgtttacgaagaaac
aacaagaaaaattcatattctgatacataagagttatataggaaccgaaatagtcttttattttcttttttcaaaataaaaatggatttcattgaagtaataaaactattccaat
tcgagtagtagttgagaaagaatcgcaataaatgcaaggatggaacatcttggatccggtattgaaggagttgaagcaagatatccaaatggataggatagggtatt
tctatatgtgctagataatgtaagtgcaaaaatttgtcttctaaaaaaggaaatattgaatgaatagatcgtaaattctgaaactttggtatttctttttcttccggacaagac
tgttctcgtagcgagaatgggatttctacaacgatcgcaaacccctcagatagaatctgagaataaaactcagaataaaaaaaaattgttgtaatccaataatcgatcttg
gttaggatgattaaccaaattaatccaaaaattctgctgatacattcgaatcattaaccgtttcacaagtagtgaactaaatttcttgttattagaaccaagaatttcgacaa
gttcggaaccatttaatccataatcatgggcaaacacataaatgtactcctgaaagagtagtgggtagacgaaatattgtctaggaaatttaagtttttctgaataaccct
cgaatttttccatttgtatttctacttgaatcagagagagagaaatatttctcggtttatcaaatggtgatacatagtacaatatggtcagaacagggtgttgcatttttttaat
acaaacccctggggaagaaaaggagtctaatccacggatctttttccgctcctttctatccaatttgtttatgtttgttctaattacaaaagagaacaaatcctttatttttg
caggccaattgctcttttgactttgggatacagtctctttatcaatatactgcttcttttacacattcaatccataacatccttttcaatccaaaatcaagaataattaggatttc
taaaaaaaaaaagaaaaaatcaaaggtctactcataggaaaacctgcttttccctacatcaggcactaatctatttttaacgtctaattagatcagggagttcttccaatt
aagaagttaagctcgttgctttttgttttaccagaattggagccaggctctatccatttattcattagacccagaaaatcagaatttttttattccattccaaaaatccaaaat
aagaaattgattttattacgacatgctattttttccattcattacccttgaggatcagtcgcggtcttatagactctaccaagagtctggacgaatttttttgcttcatccaaat
gtgtaaaagatcatagtcgcacttaaaagccgagtactctaccattgagttagcaacccagataaactaggatcttagatacgatcgaaatccaaaaatcaatggaatt
acaccgcacacccctgtcaaaatcttaaaatagcaagacattaaaagaaagattttatcaccattgaaaacactcagataccaaaaggaacgggtctggttaaatttc
actaaggttaaaagtggcaccaatcacgatcgtaaaattgtcatttttttagcatttttatttaaataaataaataaatcttgtatgagagtacaaacaagagggacaaccc
taccatttgagcaaagtgtaggcaaaaaacctaatagggagtgaggataaagagacttatccatctacaaattctagatgttcaatggacctttgtcaatggaaataca
atggtaagaaaaaaaattagatagaaaaactcaaaaaaaataaaggcttatgttggattggcacgacataaatccagtcaaaaataggattaagaaagaggcaaattatt
tctaaatagttagacaacaagggatactagtgagcctctcctagtttttttattcatttagttcttcaattaactcaaagttctttctttttctttaaagaattccgccttccttaaa
atatcagaaacagttcttgtaggttgagcacctttttcaaggaaatagagaatagctggaacatttaaacaagtttgattctttatcggatcataaaaacctacttttcgaa
gatctcttccttctcttcgagatcgaacatcaattgcaacgattcgatagacagcttattgggatagatgtagataaacaaagccccccctagaaacgtataggaggttt
tctcctcatacggctcgagaatatgacttgcattaatttccgtacagaaaaaacaaatttcatttatactcatgactcaagttgactaattttgattgacagacttgaaagaa
aaaaatcctttgaaatttttttgagtcgtctctaaactcttttctttgcctcatctcgaacaaattcacttttattccttattccggtccaattctattgttgagacagttgaaaatc
gtgtttacttgttcgggaatcctttatctttgatttgtgaaatccttgggtttaaacattacttcgggaattcttattcttttttctttcaaaagagtagcaacatacccttttttctt
atttccttcgataaagcatttccctcttctatagaaatcgaatatgagcgattgattctgatagacttttaatcaaaagagttttcccatatcttccaaaattggactttcttctt
attttaaccttttgatttctatattatttcgatttctatattaagggtagaatgacaaagttggcctaatttattagttttcactaaccctagattctttcccttgataaaaaataaat
tctgtcctctcgagctccatcgtgtactatttacttagcttacttacaaacaacccagcgaaaattcggttcgggacgaatagaacagactatgtcgagccaagagcat
tttcattactatggaaaatggtggatagcaaaatccacaatcgatcgtgtccttcaagtcgcacgttgctttctaccacatcgttttaaacgaagttttaacataacattcct
ctaatttcattgcaaagtgttatagggaattgatccaatatggatggaatcatgaatagtcattagtttcgtttttttgtatactaattcaaacttgctttgctatctatggagaa
atatgaataaaagaaattaagtatttatcgggaaagactccgcaaagagccaatttatttaaacccatattctatcatatgaatgaaatatagttcgaaaaaagggaata
aacaagtttgcttaagacttatttattatggaatttccatcctcaacagaggactcgagatgatcaatcctgaaatgataagagaagaattgactcttctccaacaaataa
actatcaacctcccgtttaattaatttaattaatatattagattagcaatctatttttccataccatttttccgtaacaaaactaattaactattaactagttaaactattgcaatg
aaaagaaagttttttggtagttatagaattctcgtatttcttcgactcgaataccaaaagaaagaaaaaaatgaagtaaaaaaaaacgcatttcctgtaaagtaaaattaa
ggtctttgcttttacttattttttcttttacctaaaagaagcaactccaaatcaaaattgaatccattctatctaacgagcagttcttatcttatctttaccgggatggatcatttt
ggatatttaaaaaatcgcggatcgagatcgttttcgcttaaccaaagaaagaaaaagaagaaggaaccttttttactaataaaatactataaaaaaaaatttatctctatca
taaatctatctctaccataaaggaataggtctcgtttttttatacaatgttctacgtcaagtttaaaattttttcatgaaaaaaagattttcaatttgactggacttgacactggat
tatgttttctgagacagaaaatgaacgcattaggactgcatcgaatctaagagtttataagagaaaaaaaattctctttaataaactttatgtctcgtgcagaatacaatac
gatttcatctttcgtttcatcagaaaaaatctgggacggaaggattcgaacctccgagtaacgggaccaaaacccgctgccttaccgcttggccacgccccatttcg
ggttttatgcgacactaataaacagtattatgtttatttcttattcgtcaatcctacttcaattacataaaaatggggggtattctcttggtaggattctagacatgcgaataat
atagaatccaaaaaatgcattgatcattacatggaattctattaagatattatatgaaagtcgaatttcttccactctcatttgagagtgcgaatacaaggaggtattttgtg
tttgggaaagtccgaagaaaaaaggattttgaatcctccttttccttttttcccttagaaaaataactcaatcaaaatccaattatctactctacaagaacgaaacgcttgtt
atgcctaatatacttagtttaacctgtatttgttttaattctgttatttatccgactagttttttcttcgccaaattgcccgaagcttatgccattttcaacccaatcgtggattttat
gcctgtcatacctgtactctttttttctattagcctttgtttggcaagctgctgtaagttttcgatgaaatctttactactctgtctgccaaattgaatcatgtattcattctaaaaa

aattcgaaaaatggataagagccgagaagtcttatattatgaaccttcgattctaaaattcaaattcttctacattgaatgtatagctgcagcaataaatttggatcagcct
ttctactccctgcatctacgttgagcaggtatctttaggtaaccgcacaatacctaacctaatttattgataagagtgcttattataaatcaattcttgcaattttttttcaaaaa
ttgatttttgcatttttaggtgtcaaaataaacaaaacccatcctagtggatttgtgtggtaaggaaaaacgggtaatctattccttaaaaaaaaaatcttggagattatgtaa
tgcttactctcaaactttttgtttatacagtagtgatattctttgtttccctctttatctttggattcttatctaatgatccaggacgtaatcctgggcgtgacgagtaaaaatcc
aaaattttttcttacaaattggatttgtttcatacatttatctacgagaaaatccgggggtcagaattccttccaattcgaaagtcccaaacgatccgagggggcggaaa
gagagggattcgaaccctcggtacaaaaaaaattgtacaacggattagcaatccgccgctttagtccactcagccatctctccccgttccaaatcgaaaggtttccgtg
atatgacagaggcaagaaataacgattgcaaaaaatccttcctttttctttcaaaagttcaaaaaaattatattgccaattccattttagttatattctttttcttaatgttaata
aaaaaaagaagaaaattcttctttttctttctaattctaaaattggatattggctaaaagacaatcagatagattttctctttagcaggcatttccatataggacttgttataat
aaaacaagcaggttatagaaaagaaaaaaactcttttttttattatttatcaacaaagcaaaaaggggtcttatcaaaccaacccaccccataaaattggaaagaaaga
taaagtaagtggacctgactccttgaatgaggcctctatccgctattctgatatataaattcgatgtagatgaaattgtataagtggattttttttgtatttccttagacttaga
ccacgcaaggcaagaatttctcgctatttactatttcatattcttgttactagatgttctataggaataagaagaaatcgcaacccctttccgctacacataaaaatggattt
cgaaagtcaatttttcttttcaatatctttactttttttcagaatcctatttttgttcttatacccatgcaatagagagcgagtgggaaaagagaggttactttttttcatttttccc
ttaaaaaataggctttcttggaaataggaatcatggaataatcctgaattccaatgtttatttctatagtataagaaaaactaattgaatcaaattcatggatttaccacgac
ctcggctgtgaccccatagataaaaatgcaaaatttctatcttcgagaccattgaaaaaaggcattgaacgagaaaaaaatcgtccacagataatctatcgtatgccttg
gaagtgatataaggtgctcggaaatggttgaagtaattgaataggaggatcactatgactatagcccttggtagagttactaaagaagaaaatgatttatttgatattat
ggacgactggttacgaagggaccgttttgtttttgtaggatggtctggcctattgctttttccttgtgcttatttcgctttaggaggttggtttacagggacaacttttgtaac
ttcttggtatacccatggattggcgagttcctatttggaaggttgcaatttcttaaccgcagcagtttccacccctgccaatagtttagcacactctttgttgctactatggg
gcccggaagcacaaggggatttactcgttggtgtcaattaggtggtctgtggacttttgttgctctccatggggcttttgcactaataggtttcatgttacgtcaatttga
acttgctcggtctgttcaattgcggccttataatgcaatttcattctctggcccaatcgctgtttttgtttccgtattcctgatttatccactggggcaatccggttggttctttg
cgccgagttttggcgtagcagcgatatttcgattcatcctcttcttccaaggatttcataattggacgttgaacccatttcatatgatgggagttgccggagtattaggcg
cggctctgctatgcgctattcatggggcaaccgtggaaaacactctatttgaggacggtgatggtgcaaataccttccgcgcttttaacccaactcaagctgaagaaa
cttattcaatggtcaccgctaatcgcttttggtcccaaatctttggtgttgctttttccaataaacgttggttacatttctttatgctatttgtaccggtcaccggtttatggatg
agtgctattggcgtagtcggcctggctctgaacctacgtgcctatgacttcgtttcccaggaaatccgtgcagcggaagatcctgaatttgagactttctacaccaaaa
atattcttttaaacgagggtattcgtgcgtggatggcagctcaggatcagcctcatgaaaatcttatattccctgaggaggttctaccacgtggaaacgctctttaatgg
aacttttgttttagctggtcgtgaccaagaaaccaccggttttgcttggtgggccgggaatgccagacttatcaatttgtcgggtaaactacttggagctcacgtagcc
catgcaggattaatcgtattctgggccggagcaatgaacctatttgaagtggcccatttcgtaccagaaaaacccatgtatgaacaagggttgattttacttccgcactt
agctactctaggttggggagtagggcccgggggagaagttctagatacttttccgtactttgtatctggagtacttcatctaatttcctccgcagtcttaggcttcggtgg
catttatcacgcgcttctgggaccggagactcttgaagaatcttttccattctttggttatgtgtggaaagatagaaataaaatgactacaattttgggtattcacctaatttt
gttaggtataggtgcttttcttctagtactcaaagctctttattttggcggtatatatgatacctgggctcctgggggaggagatgtaagaaaaattaccaatttgaccctt
agccccggtgttatatttggttacttactaaaatccccttttgggggagagggttggattgttagtgtggatgatttagaagatataattgggggacatgtatggttgggtt
tcatttgtgtatttggcggaatttggcatatcttaaccaaacccttcgcatgggctcgccgtgcatttgtatggtctggagaagcttacttgtcttatagtttaggcgctttat
ctgtctttggttttatcgcttgttgttttgtctggttcaataatacagcttatccgagtgagttttatggacctactgggccagaagcttctcaagctcaagcatttacttttcta
gttagagatcagcgtcttggagctaatgtgggatctgcccaaggacccacaggtttaggtaaatatctaatgcgttccccaacgggagaggttatttttggaggga
aactatgcgtttttgggaccttcgtgctccatggttagaacctctaaggggccccaacggtttggacttgagtaggttgaaaaaagacatacaaccttggcaagaacg
acgttcggcagaatatatgacccatgctcctttaggctctttaaattctgtgggtggcgtagctaccgagatcaatgcagttaattatgtttctcctagaagttggttagcg
acttcccattttgttctaggattcttctttttttgtgggccatttgtggcatgcaggaagagcccgggctgctgcagcaggatttgaaaagggaatcgatcgtgatttgga
gcctgttctttacatgacccctcttaactaagattttcttatttatacctgttctactgtttttttctgttctggctcggttattctatctagccgagccattcattcctttctatgaa
agaaagataaggggacagaacaaaaaaaaacaaataaagaaacaaacgtattcaatacgcaaaagaaaagagagaggaaagcaaaaggagagagagggatt
cgaaccctcgatagttcctagaactataccggttttcaagaccggagctatcaaccactcagccatctctccacagcctaatccttattttattcctacaaatagaacata
gccatacgaaatgactcactaacctctagaaacatctcaaatacaaatccctttttcgatatatttctgtatactgtatccatgtatacaggatccgctatatctgcttgtgaa
ataaagcataaaatcccctcaaccccatatccaaataaaaaaagtggtgagtaataagttttaaagagaagaatcaatggattcatgattaaacccctcctacttcttgt
attttattacaattttgattaagtgagggatcaaatataatatgtagtcaactttatttgatggtaacttggaggattagaaatatgactattgctttccaattagctgtttttgc
attaattgtgacttcctcagtcttagtcattagtgtacccccttgtatttgcttctcctgatggttggtcaaacaataaaaacgttgtattttccggtacatcattatggattgga
ctggtctttctggtagctattctaaattctctcatttcttaaatgtgtttagtatttagtagcccgatacaaaataaaaaagggccgtttattcggattgtgagacgcattaaa
atgcaatttgcgccccgaattgattgacagacaattaaattaataaaaagaaaactcaaatagaaaatgaaacggtcgacccagacatagacggtcgacccaggcg
gatataccctataaaatataggacgtagcgagcgtagttcaatggtaaaacatctccttgccaaggagaagatacgggttcgattcccgccgctcgccagcttaattt
agtaaggtactatgataaaaaaatttagtctacttttattaaagtaataggtgttagtctagtaccgtacccctttactatcttagccccctttgcaccccactcaaaaaaaga
gcactacagcggcgggaatcgaactcggcaacagggttccctaaaccggggattcaccgaaacaaacaaccagcaaacagcttttaaagggaagggagatag
actgtgcctttctttcatttatttttcttttctgcaaggtagggggccttgagagttcctcttgtggtagcaagttacttcgcaacctgctcaatttggccttatagggtcgg
gaactaatgaataaaaagggttggataccgcccaaccacccagccctctaccatatctagacaaatagaatagtccttttatacagactgctaagtgcggagacggg
aatcgaacccgtgacctcaaggttatgagcctcgtgagctaccaaactgctctactccgctctggagcgctggaaatcggtggacgaaaaaggttgaatacaggc
ctctaccatgtctagacaaatagaatagttattttatacagaatggagcgggtagcgggaatcgaacccgcatcgttagcttggaaggctaggggttatagtcgacgt
tggttgattattattaacgtctctaattcaaaaccgaacatgaaattttgatttcattcggctcctttatgggtattctcaccacttaacatctaagtcagctttttttgtctgaatg
gaaccaaggctctctgctttctagatgatccctaaagagtaggagatagaaatttgcctaaatctatctaatctaattacttcgttccctaatttcatttaagagatcctgag
gaaaagaattgggtttccaccgagctgaaacaatatgctgatggttctagcaaaccaaaactaccgtttttttagctatttggcttccatttcctttttttaacaaaagaagatt
tagttacgattggaaataaattttttttgtatcttcatccatagatcctttactcatattttaaaaattggaatccttaatccaatgcaaaattatgcttcgcgactctgtactcata

atccaaatcctatttgtttttggatgcaatttcaattagctttgggtacaaatcgcgaaaatgcatattcttcctcaatatgctattgagagaaaaaggagtaaatcctttct
aagaactaaagttttcatcggaatataaaaaaccttaaggacgccttaagtatatcatttcaaattcagttattaatagaacgaatcacacttttaccactaaactataccc
gctacatgtaaattatgataccaacgctaccctttgtcaagggtagccattcgagaaagatgctaattccctcttagttaatgaaacagagaaggttcctcacagttagc
ggttggtacttcgatcgcgggcccttcccttactttcttttttgttcagaattgaacaaagaatttggggaagaaaacatcttcccccacttatcatgaaatctgggccata
gagaaagcgtgagatgttttttttatttatcatagactttccctatggcttgagagaaacaataaataacttaaagaaaaaggcacataggagccgaaggatttacttga
tgtaaagaagattctgaatgtctctgcttagtcgattctctccgtttaactttttcttctcttcttttccactcaattctagtttattagattcttgtttaaaagaatcaaagaagat
gaatagaactaagaacacacaaaaaagagcatataggcccgagaccattaccaaaagttcttcccaataatcatattgggtatctgttcccttccttttcctattaggat
ggggcatgttggattttccatatccatataccatcgaaccttaagggttccagaaccctcctttttgctagttttcggaaacggaaaaccctgaaccaggagcagtata
aattctactgcccgccttttacaagaaaattggtgataaaactccactacagtttgttcaaaatgcaccaatcagaatcccttgagaatattcaagtaccctatttctaaga
gaagaggtacctgctgaaaatcgcttcaacaaatccgcttaagaaaaattgcaaagttttgaactcgaaggtttttccaagggatgcctgttaaaaattgtttcaacctc
tcaccaaaacagacaagaagtatatcactgaaaattaatacccagccatatgggtatatgaagggcgcgaattcgtttataccccacccaattagaggaaataaaac
ataaatggagaaagttttcatgataagatcagccaatagaagaaaaaagtccctaatttttcagaccgttctgagcatgtgaaaagtcaatagcctaaagataaaaaac
cctatactttgtgcaagtgataagagagaatatgaaagatttcctatttttcttgattttcttaagattttttgaacctgaccatgaatagacttatatatctcgatatatacag
atataatgtacattatggagtagacctataatgggaaatgaaagtggctaattttggaattgaataagaagcccttttaactcagtggtagagtaatgccatggtaaggc
ataagtcatcggttcaaatccgataaagggcttttttacttagtggtagagtaatgccgcggtaagacgtgagtcagtggttcaaatccgatacagtacttttctactaa
attcattcactttcttcttttttgaaaatttctcttttttttattgaatttgataacttagtgcgagatgcatgcatttttagtttaaacactaagcgaagcaggggggtgtaaattg
caaaaaagaaattggactctttttcctattagatcaatcaaatcactacccgtactgaactaatatagaatcccttttattaatctattcttattccatatcctttataaacgaat
ttccctaaaaagtaggggatgatccgtgaattaacctaaccatcaactaaaaaaaaatcctacaaaagcataatggaaaagtaggaaggactccttgctttggatctag
ttatactcttcgagtatattgacaattccaaaaaactgctcatactatcattatagtataatgaggagcggttgtatacggccctatcgtctagtgatgcccctatcgtcta
gtggttcaggacatctctctttcaaggaggcagcggggattcgacttcccctgggggtagggagtattatgaaaggaggttaatcatagattatcaaaaaccctaga
ataaattcttcctgggtcgatgcccgagcggttaatggggacggactgtaaattcgttgacaatatgtctacgctggttcaaatccagctcggcccaaaaatctaggg
cttcgtgaatatgagttaaatccattttttttcttccataaaaaagaatatttgatccatagaaataaaagaaataaaggataaaaagaaaagggggaaatatctttctaatct
atatctctttcattcctctcttacaaacaaaagacctttcttattggttattgaaaggtggattcttatctatttttagcgataataaatcgcgacatactagttatgtcattctc
actataccccccatacgatacgggggtatgtagtatatgattcgtctatttcttagagtaggacaggcgaatattcttattcttatggttccatttaagaataagtatgccata
cgccccgcggggattgtagttcaattggtcagagcaccgccctgtcaaggcggaagctgcgggttcgagccccgtcagtcccgaactagggtccaatgaatgga
gaaattcatctttccttttccatgaaaaaagggggggcaggaagcaagatcaaatacctatggggtacccctatttcactttttttagttcgcgtttctcagtaaagagag
gagagtataggaattttttttatcactacttctggttgatagcgaaagacatacatatcatacgtggaagggatcctcctatgttatactattccactctcaaccatgaattg
atttgatagatccgatattcataatattgaattgattcagtattatcagaatgcaagtcctccccttgaatttacaggatacccttttccctctccatgggattacatcccgag
ttattgcgaaaaaaaaagaggttatggaagtcaatattctcgcatttattgctactgcattgttcattctagttcctactgccttttttacttattatttatgtaaaaacagtcagcc
aaaatgattaattggaagttcaattaatcattgaagaaatgaaaaagggattaaataaaaaaaaaaatccaagtcttaaatgaaaggatccggttggaatcataaagtgt
ggtagaaaaaactacatatagtttttctaccacactttagagtctttctattatattatcttgaatctacatagaatagattactagattgaaatagtagtctaattcaatttctt
ttttcactgcatccacttaatttcaatcaagtcaaaatgaaaaaatccatggagggagagaaaaataatatgagaatagactatagtaaaagaaaaaagtaaaaggaa
aaaaccagcgaatctttcatgcttaaacatgtcgcgaaatgtttcaaaagagcataaaatttattttaagaactaagaataagaaaaagagtataaaacaaatggaaaat
gtgcgatatgttgggaatagctccgcggaagaaaatctaaaattcttatgtatagaactttttttaaccatgggtcgcttctagtagcgattatgaattgctctcaccgctct
ttctattttctattctttctattctactatatagaaatagaaagaattatatagaaatagaaagaatagaaagactctttcttacaagagtttcttacaggagtgaaacaaaatt
aaagaaagagagaaagaataaagttcggcaaaatgattaatgcaaaacggtcaattaaagaaaagagttgatacaacaattcgactactcaatcaattagtagtatc
cctagagtccactcctcccccatactactagtgaaagagaaaatgtaaagactaccattaaagcagcccaagcgagacttactatatccatgtaaattatgtctcctatt
tctatgaaggaattattctactattgatgaataatcatagtagaatcaagggtacagagtcaaaaaggggttctgacctaaggctatggatgaatcagttcaaagaattt
actcttaacaaattcttagagtatttctggtagaattggggagcattaagtataaatatgatacatagccctttcttattaataaaagaataaggaaacgctatctcatccct
attggtatcggtttgggccactactgctaaaacaaaccccagtttgaggaaagaacggtgggttctcaaaatccagtatcgccgagccttgttattctcttgccccaac
ttatgcggggtgcaaatttgtcgatttggatcagtactataagcctaagtattttattgatcaggcggcacccagatttgaactggggataaaggatttgcagtccccctg
ccttaccgcttggccatgccgccaaaaaatacgatctaaaatcgagaaaagagcaagtattcatgcacgtttcttactaaaactaactttcttttatcttaaatctaattcta
cttactttttccaatctttttcaaaaaatctattcatgcttttttggatccagtttcgattattctcctcaaaggattctatcttaaaacacacattgctaacactagaaaacttc
ccttttcttctattgaaatgaaaaaggagaaaagtggatttctagtcacaagctacaaaattaagaacaaattggaaccattaactagaattctcttttattttgaatttcg
gtattctctcccgcctgccatttaatggcataataaaagacaatggatttatgcctaatccgtatataggtaaactccaggtccgaacagcattattatctatggatcccc
cttatgtacatatctctgtggagaatcgttctttaatttttcattgcattaaatatcttgaataaaaaaaagaaaattgactttgctatgtggaggtccagaactagattggca
tgtacttaaaaaagtacttactttattttaggattctacaacgaaatcttatatcttatattttatagaaattctactactacgaaacaaaaaagaaccttcaaattcttttttgaa
attaaactaagcgtgctattctcaatcgaactaaagtcaaactttctagtgcttataaattattatcttttggttttatcccattcatagaaaggagaaaaaatgagaaatcttt
gccgtccaatctaagaatatcataaactgtaagtggcagaattttttttcgaggttctaggaatgttttatcacttcattttcattccatttggtggacccctgggaaattcga
actttcgttgaaattgtctctattcatatgtatgaaatacatatatgaaatacgtatgtggagttcccgagaaatttcatgtgatttagtaaacagaatatagattccataattg
ctagatcgatctgtagggattgatgaagagtgagctgataatggaatttttcttcgataaacaggaaacttaagattaagatgctccggaatggaaatgagggaatgtc
cacaatacccggatttagtcagatccaattcgagggattttgtaggttcattaatcaaggcttggcagaagaacttgagaagtttccaacaattaaagatccagatcac
gaaatttcatttcaattatttgcgaaaggatatcaattgctagaaccttcgataaaagaaagggatgctgtgtatgaatcactcacctattcttccgaattatacgtatccg
cgcgattaatttttggtttcgatgtgcaaaagcaaaccatttctattggaaacattcctataatgaattccttaggaacctttataataaatggaatataccgaattgtgatc
aatcaaatattgctaagtcctggtatttactaccgctcggaattagaccataaaggaatttctatctacaccgggactataatatcagattggggaggaagatcggaatt
agcaattgataaaaaagaaaggatatgggctcgcgtgagtagaaaacaaaagatatctattctagttctatcatcagctatgggttcaaatctaaaagaaattctagat

aatgtttcctaccctgaaattttcttgtctttccctaatgctaaggagaagaagaggattgagtcaaaagaaaaagctattttggagttttatcaacaatttgcttgtgtagg
tggggacctggtattttcggaatccttatgtgaggaattacaaaagaaattttttcaacaaaaatgtgaattaggaaggattggtcgacgaaatatgaatcgaagactg
aatcttgatatacctcagaacagcaccttcttgttaccgcgagatgtattggccgctacggatcatttgattggaatgaaatttgaaacgggtatacttgacgatgacga
tatgaatcacttgaaaaataaacgtattcgttcggttgcggatctgttacaagatcaattcggactggctcttggtcgtttacaacatgcggttcaaaaaactatccgtag
agtattcatacgtcaatcaaaaccgactccacaaactttggtaactccaacttcaacctcgattttattaataactacttacgagaccttctttggtacatatcccttatctca
agtttttgatcaaaccaatccattgacacaaacggttcatgggcgaaaagtgagttgtttgggtcctggaggattgacggggagaactgcaagttttcggagccgag
atatccatccgagtcactatgggcgtatttgtccaattgacacgtccgaaggaatcaacgttggacttactggatctttagctattcatgcgagaattgatcattggtgg
ggatccgtagagagtccattttatgaaatatctgagaaagcaaagaaaaaaaaagagagacaggtggtttatttatcaccaaatagagatgagtattatatgatagca
gcaggaaattctttgtccttgaatcggggtattcaggaagaacaggttgttccagctagataccgtcaagaattcctgactattgcatgggaacagattcatgttagaa
gtatttttcctttccaatatttttctattgggggttctctcattccttttattgagcataatgatgcgaatcgagctttaatgagttctaatatgcagcgccaagcagttccgctt
tctcggtccgagaagtgcattgttggaactggattggaacgccaaacagctctagattcgagggtttctgttatagccgaacgcgagggaaagatcatttctactaat
agtcacaagatccttttatcaagtagtgggaagactataagtattcctttagttacccatcggcgctctaacaaaaatacttgtatgcaccaaaaacctcgggttccgcg
gggtaaatccattaaaaaaggacaaattttagcggagggggctgctacggttggtggggaacttgctttaggaaaaaacgtattagtagcttatatgccatgggaag
gttacaattttgaagacgcagtattaattagcgaacgtttggtatatgaggatatttatacttcttttcacatccgaaaatatgaaattcagacggatacaacaagccaag
gctccgctgaaaaaatcactaaagaaataccacatctagaagaacatttactccgcaatttggacagaaatggagttgtgaagttggggtcctgggtagaaacagg
cgatattttagtaggtaaattaacgcctcagatagcgagcgaatcgtcctatatcgcggaagctggattattacgggccatatttggtcttgaggtatccacttcaaaag
aaacttctctcaaactaccgataggtggaagaggacgcgttatcgatgtgaaatggatccagagggatcccctcgacataatggttcgtgtatatattttacaaaaac
gcgaaatcaaagttggggataaagtagccggaagacacgggaataaggggatcatttccaaaattttgcctaggcaagatatgccctatttgcaagatggaacgcc
tgttgatatggttttcaatcccttaggagtaccctcccgaatgaatgtgggacaaatatttgaaagctcgctcggattagcaggggatctgctaaagaaacattataga
atagcaccctttgatgagagatatgagcaagaggcttcaagaaaacttgtgttttcagaattatatgaagccagtaaacaaacaaaaaatccgtgggtatttgaaccc
gagtacccgggaaaaagcagaatatttgatggaagaacaggagaccccttcgaacagcctgttctaatagggaagtcctatatcttaaaattaattcatcaagttgat
gagaaaatccacggacgctctactgggccctattcacttgttacacaacaacccgttagaggaagagccaagcaagggggacaacgaataggagaaatggaagt
ttgggctttagaaggatttggtgttgctcatattttacaagagatacttacttataaatctgatcatcttatagctcgccaagaaatacttaacgctacgatctggggaaaa
cgagtacctaatcacgaggatcctccagaatcttttcgagtgcttgttcgagaactacgatctttggctctagaactgaaccatttccttgtatctcagaagaacttccag
gttaatagggaagaagtttgatcggaataaatctaaattcttttcttatttctattttatgattgaccaatataaacatcaacaacttcaaattggactcgtttcccctcaacaa
ataaaggcttgggctaacaaaacactacctaatggggaagtcgttggcgaagtcacaaggccctccacttttcattataaaaccgataaaccagaaaaagatggatt
gttttgcgaaagaatctttggacccataaaaagcagaatttgtgcttgtggaaattctcgagcgagcggagctgaaaacgaagacgaaagattttgccaaaaatgcg
gagtagaatttgttgattctcggatacgaagatatcaaatgggatacatcaaactcgcatgtcccgtgactcatgtgtggtatttaaaaggtcttcctagttatatcgcga
atcttttagataaaccccttaagaaattggagggcctagtatatggcgatttctcttttgctaggcccagtgctaaaaaaccaactttcttacgattacgaggtttattcga
ggatgaaatttcatcctgtaaccatagcatttcccccttttttctaccccaggctttacaacatttcgaaatcgggaaattgcgacaggagcaggtgctattagagaac
aattagcagatttggatttgcgaattattttagagaattcctcggtcgaatggaaggaattagaagacgaggggtatagtggagatgaatgggaagatagaaaaaga
cgaataagaaaagttttttgattagacgcatgcaattggcgaaacattttattcaaacaaatgtagaaccagaatggatggttttgtgcttattaccagttcttcctcccg
aattaagacccattgtttataggtctggggataaagtagtgacttcggatattaatgaactttataagagagttatccgtcggaacaacaatcttgcctatctattaaaaa
gaagtgaattagcgccagcagatttagtaatgtgccaggaaaaattggtacaagaagccgtggatacacttcttgatagtgggtcccgcgggcaaccaacgaggg
atggtcacaataaagtatacaaatcactttcagatgtaattgaaggtaaagaggggaggtttcgcgaaactctgcttgggaaacgggtcgattactcggggcgttctg
tcattgttgtgggtccttcactttcattacatcaatgtgggttacctctagagatagcaataaagctttttcagctatttgtaattcgcgatttaatcacgaaacgtgctacttc
taatgttaggattgctaaaaggaaaatttgggaaaaggaacccattgtatgggaaatacttcaagaagttatgaggggacatcctgtactgttgaatagagcacctac
cctgcatagattaggcatacaggctttccaacccactttagtagaggggcgtactatttgtttacacccattagtgtgtaaaggtttcaatgcagactttgatggggatca
aatggctgttcatctacctttatccttggaagctcaggcggaagctcgtttacttatgttttctcatatgaatctcctatctcccgctattggggatcctatttgcgtaccaac
ccaagacatgcttatcggactttatgtattaacgattggaaaccgtcgaggtatttgtgcaaatagatataatagttgcggaaactatccaaaccaaaaagtaaattaca
ataataataatcctaagtatacgaaagataaagaatctctttttttctagttcctatgatgcactgggagcttatagacagaaacaaatctgtttagacagtcccttgtggct
ccgatggaaactagatcaacgcgtcattgggttaagagaagttccgattgaagttcaatatgaatctttggggacttatcgtgagatttatgcccactatctagtagtgg
gaaatagaaaaaaggaaatccgttctatatacattcgcaccactcttggtcatatttctttttatagagaaatagaggaagccatacaaggatttagtcaggcctattcat
acactatctaaacaaggaagttagattcggggatgccccttcgaggggcattccgatttcgctagtatcatcatttttgccgcacgaatccagattgagattgaggaa
aggaagttaactaagttttcgaatcactgactcaggcccattgtcgaatcctactcagcaattgtcgaattatactcagccgaaaaagggggtacttatttatggcgga
acgggccaatctggtctttcataataaagagatagatggaactgctatgaaacgacttattagcagattaatagatcatttcggaatgggatatacatcccatatactgg
atcaaataaaaacgctgggcttccatcaagccactactacatcgatttcattaggaatcgaggatcttttaacaataccctctaagggatggttagtccaagatgcgga
acaacagagttttcttttggaaaaacactattattatggggctgtacacgcggtagaaaagttacgccaatccgttgaaatctggtatgctacaagtgaatatttgaaac
acgaaatgaattcgaattttcggataacagatccttctaatccagtctatctaatgtctttttcaggagctagaggaaatgcatcgcaggtacaccaattagtaggtatgc
gaggattaatggcggatcctcaaggacaaatgattgatttacctattcaaagcaatttacgcgagggactttctttgacagaatatataatttcctgctacggagcccgc
aaaggggttgtagatactgctgtacgaacggcggatgctggatatcttacacgtagacttgttgaagtagttcaacatattattgtgcgtagaagagattgtggtactat
ccaagctatttctgtgagtcctcaaaatgggatgacggaaaaactttttgtacaaacattaattggtcgtgtattagcaaacgatatatatatcggttcacggtgcattgc
cactcgaaatcaagatattggaattgggttagtcaatcgattcataaccacctttcgagcacagccatttcgagcacaaccaatatatattagaaccccctttacttgcc
ggagcacatcttggatctgtcaattatgttatggtcggagttccactcatggcgatctggtcgaattgggggaagctgtaggtgttattgcgggtcaatctattgggga
gccagggactcaactaacattaagaacttttcatactggtggggtattcacagggggtactgccgaccttgtacgatccccttcaaatggaaaaatccaattcaatgg
ggatttggttcaccccacacgtacccgtcatgggcagcctgcttttctatgttatatagacttggatataactattcagagtcaggatattctacatagtgtgactattcctt

caaaaagcttgattctagtgcaaaatgatcaatatgtagaatccgaacaagtaattgcggagattcgtgccggaacgtccgctttgcattttaaagaaaaggtacaaa
aacatatttattccgaatcagatggggaaatgcactggagtaccgatgtttatcatgcgcccgaatatcaatatggtaatcttcgtcgattaccaaaaacaagccatttat
ggatattgtcagtaagtatgtgcagatctagtatagcttctttttcgctccacaaggatcaagatcaaatgaatacttattccttttctgttgacggaaggtatatctttggcc
tctcgatggctgatgatgaggtaagacatagactgttggatacttttggtaaaaaagatagggaaattcttgattattcaacgccggatcgaatcatgtccaatggtcat
tggaattttgtctatccttctattcttcaaaataattttgatttgttagcgaaaaagcgaagaaataggttcgccattccattacagtatcatcaagaacaagagaaagaac
caatatcctgttttgggatttcgattgaaataccctttatgggtgttttacgtagaaatactatagttgcttattttgacgacccacgatacaaaaaagataaaaagggttca
ggaattgttaaatttagatataggaccctagaggacgaatataggactcgagagaaagactcagagaacgaatacgggagcccagaaaacgaatataggacccg
agaggataaatatggcactttagaggaagactcagaggacgaacatgggactttagaggaagactcagaggaagactcagaggacgaatatgggactcgagag
gaggagtatgaaaccctagaagatgaatatgggatcccagagaacgaatatgaaaccctagaagatgaatatggaatcctagaggacgaatatgggagtccaga
gaacaaatataggccccgagaggataaatatggcactttagaggaagactcagaggacgaacatgggactttagaggaagactcagaggaagactcagaggac
gaatatgggaacccggaggaagattccgtcttaaaaaagggggttttgattgagcatcgaggaacaaaagaatttagtctaaaataccaaaaagaagtagatcggtt
ttttttcattctccaagaactgcatatcttgccgagatcctcatccctaaaggtacttgacaatagtattattggagtggatacacaactcacaaaaaatacaagaagtcg
gctgggtggattggttcgagtgaagagaaaaaaaagccatacggaactaaaaatcttttctggagatattcattttcctgaagaggcggataagatattaggtggcag
tttgataccactagaaagagaaaaaaaagattcgaaggaatcaaaaaaaagggaaaattgggtctatgttcagtggaaaaaaattctcaagagcaaggaaaagtat
tttgttttggttcgacctgcagtcgcgtatgaaatgaacgaagggagaaatttagcaacacttttcccacaggatctcttgcaggaagagggtaatctccaacttcgac
ttgtcaattttatttctcatgaaaatagcaagttaactcaaagaatttatcatacgaatagtcaattcgttcgaacttgcttagtattgaattgggaacaagaagaaaaaga
ggaggctcgtgcttcccttgttgagataagagcaaatggtctgattcgcgatttcctaagaattgggttaatcaaatccactatttcgtatacacgaaaaaggtatgata
gcagaagtgcaggactgattctccataataggttagatcgcaccaataccaattccttttattccaaggcgaagattcaatcacttagccaacatcaagaagctattgg
taccttgttgaatcgaaataaagaataccaatctttgatggttttgtcggcatccaactgttctcgaattggtttttttaagaattccaaaaatcccaatggggtaaaagaat
cgaatcctagaattcctattccaaaatttttggggctctttcgaaatttttcgggactcttaggtactattgcacctagtatatcgaatttttcttcatcttactatttactaacgt
ataatcagatcctgttaaaaaagcatttgctccttgacaatttgaaacaaaacttcaaagtacttcaaggacttaaacactctttaataaatgaaaatcaaaggacttcaa
atttcgatagtaacatcatgttggatccattccagttaaattggcactttctccctcatgattcttgggaagagacatcagcaaaaattcaccttggacaatttatttgcgaa
aatgtatgtctatttaaatcgcacataaaaaaatcaggtcaaattttcattgtaaatatagattcctttgttataagagcagctaagccttatttggccactacaggagcaa
ctgttcatggtcattatggagaaatcctttacaaaggggataggttagttacgtttatatacgaaaaagcgagatctagtgacataacgcaaggtcttccaaaagtaga
acaaatcttcgaagcgcgttcaattgattcactatcgccgaatctcgaaaggagaattgaggattggaatgagcgtataccaagaattcttgggggtccttggggatt
cttgattggagctgagttaaccatagcccaaagtcgtatctctttggttaataagatccaaaaggtttatcgatcccaaggggtacagatccataatagacatatagag
attattatacgccaagtaacatcaaaagtgcgggtttccgaagatggaatgtctaatgtttttcacctggggaattaattggactattgcgagcagagcgagcagggc
gggctttggatgaatcgatctattatcgggcaatcttattgggaataacaagggtttccctgaatacccaaagtttcatatctgaagcaagttttcaagaaactgctcga
gttttagcaaaagctgccttacgaggtcgtattgattggttgaaaggcctgaaagaaaacgtagttctgggggggattatacctgttggtaccggattccaaaaatttgt
gcaccgttacccacaagacaagaacctttatttcgaaattcaaaaaaaaaaactatttgcgtcggaaatgagagatattttgtttctccatacagaattagtttcttctgatt
ctgacgtaacaaacaatttctatgagacatcagaatcaccatttaccccatttatatgatttaaggatacataaagcagatttttttactttaaactagatttttgaccttagaa
cgctaagaggttagattttctatttttattttaatttaaaaagaagtttagttaattcattaaggttatgcttataccatgtagaaggttccatcggaacaattattatttatttcaa
gctatttcggctctttcttaatcttcgaaaagaaagaaatttcgtaatggaaaggtaggatgaaaaaaaagaaaaaatcaaaaggaagtgtggaaaaaatgacaaga
agatattggaacatcaatttgaaagagatgatagaagcgggagttcattttggtcatggtattaagaaatggaatcctaaaatggccccttacatctcggcaaagcgta
aaggtactcatattacaaatctcgctagaaccacccgtttttttatcagaagcttgtgatttagtttttgatgcagcaagtcagggaaaaagcttcttaattgttggtaccaa
aaaaagagcagcggatttagtagcatcagctgcaataagggctcgttgtcattatgttaataaaaagtggttcagtggtatgttaacgaattggtcgattactaaaacta
gactttctcaatttagagatttaagagcagaagaaaagatggaaaaattccaccatctcccaaaaagagatgtggcaatcttgaagagaaaattatctaccttgcaaa
gatatctcggcgggatcaaatatatgacgaggttgcctgacattgtgatcgtcctcgatcagcaaaaagagtatatagctcttcgggaatgtgccattttggggattcc
tactatttctttagccgatacaaattgtgacccagatctcgcgaatatatcgattccagccaacgatgacactatgacttcaattcgattgattcttaacaaattagtatttg
caatttgtgagggccgttctctctatataagaaatcattgattaagaagaatagttaattcttgggcaactgcgtagatttatggaatcacttactattctttttttgttttgcata
gaaaaaagacggggaatattgatatatattagagggtattgatatatattatcatctgatgtgatttcttgatatcctaaatataagattaatacttcacgttgctgagttgag
aaaaagatggttgaatcaaaagaattctttttttgaagttcatttttttatcaggggacaatatgaatattataccgtgttccattaaaacactcaaggggttatacgatatatc
gggcgtagaagtaggccaacacttctattggcaaataggaggtttccaaattcatgcccaagtacttatcacttcttgggtcgtaattactatcttgctaggttcagttatc
atagctgttcgcaatccacaaaccatcccaaccgatggtcagaatttcttcgaatatgtccttgagtttattcgagacttaagcaaaactcagattggagaagaatacg
gtccctgggttccctttattggaactatgttcctttttatttttgtttcgaactggtcgggtgctcttttaccttggaaaattatacagttaccccatggagaattagcagcgcc
cacgaatgatataaatactactgttgctttagctttactcacgtcagcggcatatttttatgcgggtcttagcaaaaaaggattgagttatttcgagaaatatattaaacca
actccaatccttttaccaattaacatattagaagatttcacaaaaccattatcgcttagttttcgacttttcgggaatatattggcggatgaattagtcgttgttgttcttgtttc
tttagtccccttagtagttcctataccggtcatgtttcttggattatttacaagcggtattcaagctcttatttttgcaacgttagccgcagcctatataggtgaatccatgga
gggtcatcattgaattgactagttttcaaaatagtcttttttttagcttaactcaattcatgcatggttgcggaaaattcgcttggttggaaaacaaaatagttagaattgcgt
atgaatatacaatctagagttgtagaagagagaataggctatattacggaattgccaaacaaagtatataggcattagggaggggcggagtcaggctagatctatat
cctttatgtctataagttcagtcatcttttgtatgggtttccactttaaggaattttttttgaatccgattcaatagaaaatgagaaaatacacaaacaaaatagaagaaaca
aattgatatgggatattatatattcccaagttagattcattatctaatccgatatatggaatcggattccatatccaattcgatgcagcatattgttatcaattggatatcttga
tttaattcctattggatctggattaggtcgatttccataggggttcttcctctatttcaccttttattatgaattagatgataggggaaaaaatagaaactcaaggatatcgaa
gaggaaagaaagaaggatggaatgaaagatcagttggttggaaagaaagagaaatagaatagtgagtacacaaacctctaatgattagaaactaaaaaggagat
ctcgaagcagttcggagaattcagattatcgtttcaatttgtactttttagttacttctgtccaatagagcttagaaatatgaatttcttggttgattgtatccttaaccatttctt
ttttttgacacgaggaactcatcatgaatccactaattgctgctgcttccgttattgctgctggattggccgtaggtcttgcttctattgggcctggagttggtcaaggtac

tgctgcaggacaagctgtagaaggtattgcgagacagccagaagcagaaggtaaaatacgcggtactttattgcttagtctagcttttatggaagctttaacaatttat
ggactagttgtggcactggcgcttttatttgcgaacccttttgtttaatcctaaaaaagaaaacgagtcctttagattagatactttcttctttttttagtaaattggtatttgctt
ctgcaattccaattatatcaatactttactcctaatttattactcctagagttttctatttatggggacagacaataccccaccccaggaatagctgatttgaggatgatcaa
tttagaggatatgttcgccgtcttgcttcccgccctttgtttagggcagtggaaagtatttttccttttattttaggaattttgggaacatttcaacaaaggaggtctttcaca
ggtcaaacgagatttaagacttaatctaaaagaaattactaaattgaatctatttctattaaaaaaaaattgcattaaaaaaaccgatcaaaaagggcgagcgaagtaagt
gatcgaaaaactttgttctttgttcgtcctatctataagaggagagcatatgaaaaatgtaacccattctttcgtttttttagctcactggccatccgctgggagtttcgggc
ttaataccgatattttagcaacaaatctaataaatctaactgtagtggttggtgtattgatttattttggaaagggagtgtgtgcgagttgtctatttcaagaatagattggat
ctatccggctgcactttagaatattttttagtatttttcgaataaataagaaaagggtgcacgatctcgacgaattacttctgaataaatgcagaaatcatatgaaagaac
catagcatttcgcgacctattggtaaatcaaatcaactttgattctctatagaccaataatatgagaccattaacacggttaaagctaaactgcttgaagtctaggcaaaa
gggggtactctttctacaactatattagtattagtaccaaatgctttaaacaggaaatagctaatgtagaatttatctgatatagaacactcatatcgataaaatggtttgaa
ctatttactagaaggggcaccctgccctttttttatccaatgccgaatcgacgacctatgtataaaaaaaggagaaagtttttggatttgaagaaaaaagtaggaattctat
ccattttcattttccatttttctatttagtttttcttaatgaaattgaaaattttaactaaagggcaaatacaaataaaaaaacaactttgctgcccacgatagatttttatctagg
cggaagagtcctcttaatatttatctagtcttatatgggtttcggtatattgaaatataaacagaaaagagagggtagaggataggctcattacattaaaaaaaatatgg
aagtagccatagcaaaaaaagaaaaaagggagcgtgagagccaaatgaatcgaaagattcatgtttggttcgggaagagatcataaaaattgtaaacttaatagca
aggtaatctactttcattaaaagatttattagataatcgaaaacagaggatcttgagtactattcgaaattcggaagaattgcgtagaggaaccattgagcagctcgaa
aaagctcgaattcgattacagaaagtcgaactagaagcggatgagtatcgaatgaatggatactctgagatagaacgagaaaaagcaaatttgattaatgccacttc
tattagtttggaacaattagaaaagtctaaaaacgaaaccctttattttgaaaaacaaagggcgatgaatcaggtccgacaacgggttttccaacaagccgtacaagg
agctctaggaactctgaatagttgtttgaataccgagttacatttccgtacgattcgtgctaatattagcattctcggggccatggaatggaagagttaaattaattaggc
cttgaacttctactttcctttagaatttaggcattattttccccttgcttccgaaaaaaaaaatagtaaagaaacactaatggcaacccttcgagtcgacgaaattcataaa
attctccgcgaacgtattgaacaatataatagaaaagtagggattgagaatatcggtcgcgtagttcaagtgggggatgggattgctcgtattataggtcttggtgaaa
taatgtcaggcgaattagtcgaatttgcagaagggactaggggtattgctctgaatttggaatccaaaaatgttgggattgtattaatgggcgatgggttgatgataca
agagggcagttttgtaaaagcaacaggaagaattgctcagatacccgtgagcgaggcttacttgggtcgtgttataaatgctctggctaaacctattgatgggagag
gcgaaattgtagcttcggaatctcgcttaattgaatctcctgctccgggtataatttccaggcgttctgtatatgaaccccttcaaacggggcttattgctatcgattcgat
gattcctattgggcgcggtcagcgagagttaattattggggacagacaaaccggcaaaacagcagtagctacagatacaattctcaatcaaaaagggcaagatgta
atatgtgtttatgtagctatcggtcaaagagcatcctccgtagctcaagtagtaactactttccatgaagagggggccatggaatacactattgtagtagctgaaatgg
cggattcccctgctacattacaatacctcgctccttatacgggagcagccctggctgagtattttatgtaccgcgaacggcatactttaataatttatgatgatctctcca
aacaggcacaagcttatcgccaaatgtcccttctattaagaagacccccggccgcgaagcttacccaggggatgtttttatttgcattcacgccttttagaaagagc
cgctaaattaaattctcttttaggggaaggaagtatgactgctttaccaatagttgagactcaatctggagacgtttccgcctatattcctactaatgtaatctccattaca
gatggacaaatattcttatccgcagatctattcaatgccggaattcggcctgctattaatgtgggtatttccgtttccagagtaggatccgcggctcaaattaaagccat
gaaacaagtagctggcaaatcaaaattggaattagctcaattcgcagagttacaagcctttgcacaattcgcctctgctctcgataaaacaagtcagaatcaattggc
aaggggccgacgattacgagaattgcttaaacaatcccaagcaaatcctcttccagtggaagagcagatagctactatttatatcggaacaagaggatatcttgattc
cttagaaattggacaggtaaagaaatttctggatgagttacgtaaacacctaaaagatactaaacctcaattccaagaaattatatcttctagcaagacattcaccgag
gaagcggaaatccttttgaaggaagctattcaggaacaactcgaacggttttcccttcaggaacaaacataaattttgcacgtctactcttgttagtagaagtagtaga
ggagaaatcgttgagaaagatttttcatttgaatcatgcaaaaaagttttcttagtttttagtatagttatttaaagaatagatagaaataagattgcgtccaataggatttga
acctataccaaaggtttagaagacctctgtcctatccattagacaatggacgctttttcatattttcttctttcttttctttttttctcggaccaagagaaaaaactgttagacc
aaaactcttttaggaaagaaaaacaaatgcatatacaaatgtatgatgcatatatcataaagaaggaatatggagcgggtagtgggaatcgaacccgcaaccccac
ggttatgagccttgtcagctaccaaactgttctatcctgttaaactaaagagaggggaactagtggataaaagggggggttgaatacgcccctctaccatatctataca
aatagaatagtccatttatacagaatggtaaagagggctcttctacgatcatcaattccagaaatccatacaaatacgaaagggtattttatccttaccaactggatcttg
ttgcacccggtaacaaacatgcataaaccatttctcgaagtatgtgtccggatagcccaaagtctcgatagttagctctaggtcttccggtcaaaaaacaacgtcgat
gaaggcgtgtcggtgcactattacgtggcagggattgcaatttttctcgcattttcgtttttcactcaaactcaaagggtaaactttgcttcttatctttttttttgaagatcg
acgaatcaaatgatatttctgttctaatttctgccgcttcctctctctctgaatcaaactttttttttgccataatgtgccgttcctactatctaccaagtatacggttctaatccta
gatggaaaaataaatagaaaaagaaatctaagaaggcggatcctccccctccatcaagagtaatgaactaggtgctgatacagtacaacaaaattaactaaattaac
caaacttgcctgatgttgaggcaatcaagaaagcggcataagtgaatatataacccacggaaaagtgagctaatcccaccaatcttgcttgcacaatggaaagagc
cacgggcttatctctccagcgaattaaattagccaaaggtgtccgttcatgagcccatgctaaagtctcaattaattcctgccaatatccacgccaggaaattaagaac
ataaatccagtcgcccaaacaagatgtccaaataagaacatccacgcccataccgataaactattcatcccaaaaggattatatccattgataagttgtgaagagttta
accataggtaatctcttaaccatcccatcaaataagtggaggattcattaaattgtgaaacgttgccctgccataatgtgatgtgtttccaatgccaataaaaagtaacc
catccaatggtatttaacatccagaaaactgccaaataaaacgcgtcccaagcagaaatatcacaagtaccgccgcgaccagggccgtcgcaaggaaaactatat
ccaaaatctttttttatccggcattaatttggaaccgcgtgcatctaaagcaccctttactaaaatcaatgtagttgtatgcaaacctaaagcaatagcatgatgaaccaag
aaatctccaggtcctattgttaagaaaagcgaattactattctcgttaacagcattcaaccatccgggtaaccatagggttcgacccgcattgaaagcgggaccgctc
gttgaagataagagtatatcgaacccatatgtcgtcttaccatgagccgattgtatccattgagcaaatataggttcgatcaagatttgcttttctggagtaccaaaagca
agcatgacgtcattatgaacataaaggcccaaggtatggaatcctaggaagagactagcccaacttaaatgagatatgatagcttctttatggtctaacattcttgcca
atacattatcctcattctgttccggattgtaatctctaatgaaaaaaatagctccatgagcaaaagcccctgtcatgatgaaccctgcaatgtattggtgatgagtatata
aagctgcttgagtagtaaagtcttgtgctatgaatgcataagaaggtaaagagtacatatgttgagctactaaggaagtaataacccctaaagaagctagagcaaga
cctaattgaaaatgaatcgaattattgattgtgtcgtaaaggcccttatgcccacgccctaatcgaccccccggaggagtatgtgcttctaaaagatctttgatactgtg
cccaattccgaagttagttcgatacatatgaccggcaatgagaaaaataaatgcaatagctaaatgatgatgagcaatatcggtcagccacaaactttgtgtttgtgga
tggaatcccccaagaagagttagaatagcagttcccgctccttgagtggtaccaaataaatgattactcgaatcggggttttgggcataaagattccactgacccgtc

agaaggggtcccaacccctgtggatagggtaatacatctaagaaattattccatcgaacgtactctcccctggatgctggaatagcgacatgaactaaatgtcctgtc
caagccaaagaacttaccccgaaaagtcctgacaaatgatgattgagacgagattccgcgtttttgaaccacgaaaggcttggtttccatttgggttgtagatgtaac
caaccccctattaaggatagggtagaaagaaataatagaaaaagagctcctgtataaagatcttcattggtccgtaatccaattgtataccaccactgataaacccca
gaataggcgatattcactggaccggcggcgcctcctcgagtaaaggcttccacagcgggttgaccaaaatgaggatcccaaatcgcatgagcaataggtcttacg
tgtaaaggatcctgtatccatgattcaaaatttccttgccaagcgacatgaaacagatttccggacgtccatagaaagattattgctaattgcccaaagtgagaagcaa
aaatgttctgataaagacgttcctcagtaatatcatcatgactttcgaaatcatgtgcggtagcaataccaaaccaaatacgacgagtagtggggtcctgagctaagc
cttggctaaacctgggaaatcttaattccataatgcctttcaaatcctcctagccactatcctactgcaataattctcgctaagaagaatgcccatgttgtggcaattcca
cccagaaggtaatgggttactcctacagcacgtccttgtataatgctcaaggctctaggctgagtagcaggagcaacttttaatttgttatgagcccaaacgatagatt
caatcagttcttgccaataaccacggccgctgaataaaaacattaaactgaaggcccagacaaaatgagcacctaagaaaaaaagaccatatgcggataatgaag
aaccataagactgaattacttgggatgcctgtgcccacaagaaatctcggagccacccattaatcgtaatggaactctgtgcaaagtttcccccctgtgatatgagttac
cacccccttgatcacttatagtaccccaaacatccgactgcattttccaactgaaatggaaaatgactaccgaaatggaattgtacatccagaatagacctaagaaaac
atgatcccaggcggatacttgacatgttccccctcgcccaggcccatcgcaagggaagcgaaaaccaagatttgctttatcgggtatcaaacgggaactgcgagc
aaataaaacacctttcaaaagtattaatacagtcacatggattgtaaatgcgtgaatgtgatggactaaaaaatctgcggttcctaatggaataggtaacaaagcaact
ttgccgcctactgctactaactcgccacctccccacgttaagctggtacttgttgttgcaccaggagctgttacgctaggcgcgccagcatggagattttgtacccatt
gagcaaagatcggttgtaattgtatggcggtatccgaaaacatatcttggggacgacctaaagcactcatggtatcattatgaatgtataaaccaaaactgtgaaaac
ctagaaatatacatacccagttaaggtgggatatgattgcatcacggtgtctaaggacacgatctaatagatcgttgtatcgagtcgttggatcatagtctcttaccata
aaaatggctgcatgtgcagcagcaccaactattagaaatccgccaatccacatgtggtgtgtgaacaaggaaagttgtgtaccatagtcagtagctaggtatggata
ggggggcatagagtacatatgatgagctacaacaatggttgtagagcctagcatagctaggttaagagataattgagcatgccatgacgttgttaggatttcatagag
acctttatggccctgtcctgtaaatgggcctttatgagcctccaaaatatctttcaggccatgaccaataccccagttggtcctatacatatgacctgcgatcaggaaaa
gaatagcaatagctaaatgatggtgcgcaatatcgctcaaccatagaccgccggttattggatctagccctccgcgaaaactaagaaattctgcgtatttggaccaat
tcaaggtgaaaaaaggggttgctccttcggcaaaactaggataaagttgagccaaaaggtcgcgattcaagataaattcatgaggaagtggtatctccttaggatca
accccagcgtcaagaaattggttaatcggtaaagatacatggatttggtgccccgcccaagaaagagacccaagtcctaataaccctgctaagtgatgattcaacat
ggattctacgtcttggaaccaggctaatttgggagcggctttgtgataatggaaccacccagcaaaaagcattaacgatgcaaaaatcaatgcaccgattgcggtac
aatagagttgtaattcactagttattccggatgctcgccaaatctgaaaaaacccggaggttatttggattcctcggaaacccccgcctacatcaccattcaatatttctt
gccctacgattggccaaactacctgagcactgggtccaatgtgagtaggatcacttagccatgcttcataattggaaaaacgggcaccatggaagtacatgccactc
aaccaaagaaagataatggagagttgaccgaaatgagcactaaagacttttcgagagatctcctccaaatcacctgtatgactatcgaaatcgtgagcatcagcatg
taggttccagatccaagtggtagtatcagggcccttagctattgttcttgagaaatggccgggtctggcccattcctcaaaagatgtttttacaggatccctatccacaa
caatttttacttctggttccggcgaacgaatcatcattaagtcctcctctttccggacaagacatacaaagagacccgccaactttttagtgaacctttgaaagatagata
ttatgattagtccttttctttactatctaccgtccttctatttttttttagttattcactggagcaattatatattgaagtcaatctgaggcaagtgttcggatctattatgacataa
ggattgggtgcctaacggactttttttttatcttggatttctccacgtaacaaaaaaaaccttttttttaatttaaaaagctagtctatttttttttaagagtataagtccctatctatat
ctactttccttgagcataatatagattttttttattcgattccaaattccaagataactcattagaattattaataagatggtcctgatatattagcaatatttatattgccccttttt
attcgctttattacttctattctagaccctatcgtttatccttatgaaatataatataaaatagaaggcagaggaaagagatataatgaaattcttgattcgatcttccgacct
aatttatttgattaatggatcaacaaccaaacccccattttatgaaaaaggagagtggtcttattcaaattcaaagcgcttcgtaatcttcaaccagttctgtgcttcaatat
aatttcccggagtaagcgctatagcttgtttccaatactcagcagcttgatcaaaccaagcttctgcaatttctgaatcaccctgtagaatggcctgttctccccggtcg
gaataggtagttccttcccctagaaccgtacttgagagtttcctacctcatacggctcagaaattgctatcttaatttcccttgtcttaactgaattcgatttctcaaaaatcg
atccaatttcttcttgggttacgcagaagaagttaattacctaagtttcaaaccctaaattttgatcaataatcagtttgatcttttctcccacctgcagaaaaatgaagcat
agatagacctatatccttcgtccgaattttctgaaaggtaactatctcggtttcatatatgaaatttctatagaatccttgaaaaagactttttccccataagcaagaaaaaa
gaacttactatctttgggatctgatactacaccgctgcttaatcccttagtggatcggctctattacataagcggattcctaaattttgccccatatcatgggataagtaag
cagtttttttttagttgtatcgacccagtcggtcactaattgatctttacggtgctttctctatcaatttgagaactctatccatagagtagtataggccatactttctttcttccta
ttttgattctcgtgaagtgtcctttcttcctacagctgatagggcaaaatcgttgttttgacgatccctatgtagaaagccccccttttctagtaaatactagaaaatttgatcc
tttcttatttttctttctatagtggagatagtcgcacgtaatgacagatcacggccatattattaaaagcttgtggtaagaaggggtttcgttctagtgcccggaaataatat
tccaaagcctttgtatgctctccattgcttgtgtgtataaggcctatgttatagagtatataacttcgatcatagggatcaatttctagtcgcgtagcttcataataattttgca
aagcttccgcataatttccttcggattgagccaacatccgttacggtcgttcgttctattcaaaaaatctccgttccaaaaccgtacatgaggttttcacctcatacggctc
ctcccttctgtacatagtactaagcgaaaaaatctagagaataaaaatagaattagttccatctcattatggaccgaaaggggctggtatttttccaagaaatctctagc
caaccttcccacaagaggtttttcttaacaccaatgaattctattaatgctagaggaaaacgatagctccaagaatttctttgttctcaacgcctcctatttagaggaatta
gccacttcaacgatctttgatggttataggggtatccaaagtacaaacttgatggttgtttgttatcccaaccattcttcccagccctgataccaatcaggaaagggttaa
tttctaacaaagtttttctcttgttgattcctatttctaggtgtagtgcttttatcccctatgctacctattagtactagtagagtaggattagcctgtaatacagaacctatcct
gtaggtgtaacctttcgctcaatactaaaatctacaattgaagcatccgaggccgcgtcagtcgaggatacacgacagaaggagttgttagttcacctcaccttccct
aagcgtgggtttcccttactaatttggttttctctccgcgaacccccgctctttctcgtaaaaccggggtgtaggtagggctaaaaaaacaaaaaaaaagagtcaaatcg
caccatctctataataagtaaatgcccttttttccccggaggttgtcggaattattcgcaataaaatattggctacaattgagaaggtcttatcaatgaaatttccatttatac
gggatctaggcataattcccaacccattctatcattctatatagaattcttttcattccttcacaaaataacataaaaaaaaaaaacaaatccattcaattcttataaatcgatcc
ctatgctccaaatggataagggaggtatttctgctcagcccaaattctctctttttccttctgtttgaacaagaagagatagaaaatatttgactaagattggatttcattcc
actttcctatttcttttctcaacaacaacttctctcatcaactatttcgcattttcaaagtcattaatcgtcccataccctattttctatttgattgtatggggtaggataccttatg
caaacagaattctagggttcctttttttatcgaataagaagaattcttccattctctttttctttggtttggggaaaacccaaactaaaacttttcgagggagcggaattccta
gtaaaaaaatcctggacccgtccacttagatgaaaaggaatttttctatctaatagaacaatggaactctcgcgcggttgtggttgtacctgtactgcaggaataggaa
aactcgctattcactcagtttttttttccataataagattatggaggagagatggccgagcggttcaaggcgtagcattggaactgctatgtagacttttgtttaccgaggg

ttcgaatccctctctttccgtttttcttaattcagcaacgttaatgattacaatgtatcaaatcaaatgacaatttattccagcaataatacaatacctttatttaatagaaattct
ctataccaaattactatggtatgtaaaatacacatagaggaaagaacaaaaaacaaaaaggaatcctagggttaatccatttctgttaggtgaatgggaaaatacgaa
ttaagagccttaggtcgatttagttcggggaaaggggaaggggaagaaaattctatgaacctttccgtttttccttaagttcaagtttgacgagagtaatattctacaact
aacaactcatttattttgagaccgacccacttcctatccaggattttttttactagtcctttatattgcaatgtgtcaatcgtcaaatgctttggcaatttccccgggtcggat
gaagcaatagaattttgaaccagacgttttgatctttggttatccttcgtagtaataatatctcggggtttgcaacgaaaacttggtatatcgactatacgaccattaacta
aaatatgtctatggttaactaattgccgggcctcgggaatggttgaagccatacctaatcgaaaaaggatattatccaaacgcatttcaagtaattgtagtaaaacctga
cctgttgaacttttttgcttttccagcgatatgtacatatctaagtaattgtcgttctgtcagaccataatgaaaacgcaatttctgtttttcttgaagacgaatacgatattgttc
ttttttcccagaatggaatttctttttcagattacttccggatttaggtgtttttctagtgagtcctggtaaagctcccagacggcgtatttttttaaaacgaggtcctcgataa
cgggacatgaagactcctttttttattgaaatttcattttacacaattaatttcattgtatttacattacagaatacatcgaaattaaaactgaattaaagtaaaggataaaca
gagtaaaatctactaaaagtaccacaaaaaaatggaatttcatcaacatctgaatttttgtatatatattatttattttattgttttgtatctagcaaaattgtagggtagaacg
acataatagatcctggcttctccatttaattcggagaaaaagaggtatttttgttcatggaacattgatagagaaaaaagccgactatcggatttgaaccgatgaccctc
gcattacaaatgcgatgctctaacctctgagctaagtgggcttacataacagaaatagtgtaacaaatagaaatatgtatagtataggaaatccgtaaaatgtcagatc
ttaattattaatcttagctattaactagttcgaaattggaagttctacttagaaaaaaatactagaacttcataaaataaagttagatagatttttttgaactttctttttttctctaa
ttcgcaaatctatttttctaatagaatctattccaatttctatattgaatttgattttagatatttttaatttgatatggctcggacgaataatctaatacatggaaaagaataaat
aatatatatacgaaaacataataaagagaacatgcgaatttcttgtattttcagtccatcattatagacatttttttgagatattttgttttttttgttatttgctaataatttaatgatt
aatatttcactaaggagaacatagaatcatagcaaatgaaattgctaattctgattagcaaaaaaaaagaatgaatatcaagcgttatagtatgattttgaatactctaaa
aaaggaacgcggtaggtgggggagagaaaaaccttgggatatattgattcgcattgaattgcaaatacatcaacgatagaatcaattcaatgctgaattgcaataag
cggagtctctcaactagagacgaaccgctagactacatagagtaatgaattcaacgattcaaaaaaaactaacagatggaggaaattgcacaaggaatcctggtct
caaagaaaaagaaaatggggatatggcgaaatcggtagacgctacggacttgattgtattgagccttggtatggaaacctgctaagtggcaacttccaaattcaga
gaaaccctggaattaaaaaaggggcaatcctgagccaaatccatgttttgagaaaacaagcggttctcgaactagaacccaaaggaaaaggataggtgcagaga
ctcaatggaagctgttctaacgaatcgagttaattacgttgtgttgctagcggaactcccttctaaattagggaaagaagggctttcgaaatctaatacacacgtataga
tactggcatagcaaacgattaatcacagaactcatatcataatataggttctttaattctttttttaaaatgaaaataggaatgattatgaaatagaaaattcataattttttttag
aattgttgtgaatccattccaatcgaatattgagtaatcaaatccttcaattcatagttttcgaaatctttttaaaagcggattaatcggacgaggataaagagagagtccc
attctacatgtcaatactgacaacaatgaaatttctagtaaaaggaaaatccgtcgactttctaagtcgtgagggttcaagtccctctatccccaaaccctcttttattccc
taactctagtatttatcctgtttttttttattaataggtttaagattcaatggaatacatttctttttttattatagtatcggcaaggaatgtcgattattaactcgatatttaaatattatt
aaataggctttctttgtacaatgcataggactgccccctccccatatccaaattttggatattgacatagatacaaatactctactaggatgatgcacaagaaaaggtca
ggatagctcagttggtagagcagaggactgaaaatcctcgtgtcaccagttcaaatctggttcctggcacagaaaaaagggatcttccgaataggtattgatacaaa
tacctcgagatgggttgggatacatattcgttaataatatagatagagtatgatttttttcatctaagtagataaatctctaaatagaggcacttctttttctgcatttttgcattt
cttaattttctattccgctattccgacaaatattttttttattcttgcttatcttatcttactttcctagttgttctaagtaatgcacgcggtacaaagttcgtggtagggaacttcttt
gagtcatcatatttttctgttcatacgaaggaaatgaatatgtgattttccaacgaaatgaagccctttttttgcttagtctatctgaacctttttgtataattggaattaattagaa
tgtaataggtattctgtttcatctaggaacagagcgtaaaaatattccttgacttgcataaaatctggagttgtgttgtataagtgagcatgaatttcttatcattcaatgagc
atcttgtatttcatagaaattgggggttatatagtccttacgtaagggccagcctatccaactttcaggcattaggatacgtttaaggcgtggatgattatcataagagatt
cccaccatatcaaaagattcgcgttcttgaaaatcggaacttctccaaatccagaagacagatgggattctaggattatccttttgggcaaagacttttatgcatacttct
tctgggttatctataccatactgtattctcgtaagatgatacacgctagctaaagatccaccgggtgcaacgtcataagcacattgggaacgtaaataattgtaaccata
tacatataaaatgacagcaatggaatcccaatcctctgcttttatttgcaaagtctctattcctcgatgatcgaagcccaaagatctatgaaccacctcatgtttgactag
ccaattagataaccaaccctgctgcattatcttgatctctcctcctttgtataaatatttcgcagttcgaatgcaagtttgaaatattgccctgctctttctttttcggcacaaa
gagctcctcctaattcactaatttgtaggaagatactggacttttggatttgaaaaaagtttcagaagatatgtctaaagtagatggtgattgatagagcaattcttgctca
taagttccagtattagtactgcgccgaacataaagcttgtggctggtagtaaaacatcgatttttcttttgagatagagttcgatcctcaactatttctcgcgatatcttctta
cgaagttttgttagggcatctataacagcctctggtttaggtgggcaacccggcaagtagacgtccacaggaattaacttatcaactccccgaacagtactataggaa
tccgtactgaacattccaccagtaatagtacaagctcccatagcaatgacgtattttggttcaggcatttgctcatataatctcactaaagagggagccattttcattgtta
ctgtaccggctgttaaaattaggtccgcttgcctaggacttgatcttggtaccaatccataacgatcaaagtcgaatcgtgagcctattaatgaagcaaattcaatgaaa
caacaactggtaccatatagaaggggccataaactagagagtcttgaccaattcgaaagatcttttagtgtagttgaaataacggaattggaacttgtttggtcaagta
ggggaaactcaatcaaactcataactgtcttaatggaatcttttccttcttttttttttttgtctgaatattcagttaagaccattccaaggctccttttcgccatgcataaacta
aaccaacaactaggataagcacgaaaatgaaagcttcgataaaaacggatatacccaatacgtcgaaactcattgcccaagggtagagaaagaccgtttccacatc
aaaaacaacaaaaactagcgcaaacatgtaatagcgtattcggaattgtaaccaagcccccccccatgggttctatacccgattcataactagaaagcttctctggtcc
ttcacgaaccggagctaaaagtgctgaaatccaaaatgctaaaataggaataaggcttgctattattagaaatgcccaaaaaaatatcatattcgtgaagcagaaacat
aaatgtactcccattaatgtggaataggcggaactgaattagtcaattcaagtcagcattgtcaatttatacagaatttctctcttttcctcggtgaaacaaggatcggtttt
tctcaaaccaaagggcttagtttagcctttgtttcctctttgccacgtcttctttaaagattcatccaatggaatcccgactccctttctttttgatttcctttctatttaggtatg
gtggagacataattcttatagaaacaaaactctctcgcttcactttgtctcattttctctagaatctctagaaaaaggaataaaaacgaaaatactacgaattagagccta
attaagataggatgactaatgtatgcagcctaatgaggagtaattctataaaaataaagaactctatttcagaacgtagatcgatttagatttaggtaatctatagatatag
ataagcaaagtaatatacttcaaacaaagtaggaattcgcaagatggagaacatcttgcagttgatttgatagaaattcatttttcttttcctgtctctataattttcgatgaa
tgagcctctggtaatccttttatctctattttatggcgcaggcgcctgtccagtctataaacaagtactaatagggaaatgaaaactatactaaaggaaacgtaggatat
ctctcctaaaatctaaaaaaggacaggacatattagggctatacggattcgaaccgtagaccttctcggtaaaacagatcaaacggattattatcgaaatgattcgaa
ctgtttcaaagacccaacatgcatttttttgcattgggctcttttatcaactgatagaaagatcagttagtccaccatagtttttctttacggaaagataatgagatggctcc
ctgcgctctgattgattatttgtattatgatctatctaagagcaataccaaagtgtttcaaaggaggattaccttgacttaggtctgcctccggcctaaattaaatcaacct
aagtgaaatagagtctctatcgttccgctacaagagttgactatgagacttcatacaccttaaagttcatagaacgaaaagaattttttggaggcccttatcctcattaa

gcctagcatttagtgggctggatatttaccttatcaactagcaaatcaataaaggttctatttgtttaggcacctggattggtacctgaatcggactgaaccaactatttgt
caggcgactgttctcctattctctcgaatccatgaagtaagacattgattttgcaagaagatccactatgttcattgcataataagctcctttgaaaagcattggcgcacg
tgtaaacgagttgctctaccgaactgagctatagcccttgtcagagatatcttaatatatagagaatttcttgtcaagatgaatattctctaatagtagaggatatcctttga
tctgtttactataataacataccaataacgaagcggtatttgcttataaaaaggattcgatctataatcgatcgaagtaaagggtcttcctttgtggtgataaattgcctact
taactcagtggttagagtattgctttcatacggcgggagtcattggttcaaatccaatagtaggtaggtaggtagaaaaattactagatagcattggccctacttcgctt
cgctatctaataatttttctacccctcttcccttttctttgtatcaactaaaccgttgggttgtcttcaattagatgggggaatccaattaacagcctcgactcgtatcctag
ctcgtctgagagctaccttcgcttcaaccaattctttcgtaccctcagctctactcacgttagcttcggctatttcaagtgcctgttgagcttcttccggatcaatgtcacta
cccagttccgcatcatttcctaaaatgatgatctcattattaactattctggcaaaaccgctccacagaaccgccgttaaccattgatcgttgaggaggcgtattctcaa
gggacccatatctacagctgtgttaatgggggcgtggtttggtaatacgccaatttggccactattagtagataaaatgatttctttcacttcacaatcccaaataattcg
cttaggagtcagtacataaagatttaatttcatttcttcaatttgttctcctcttctaagtttatagctttcgtgctagcttcatcgatgttacccaccaaataaaaagcctgttc
gggtaggccgtctaattctccggaaaggattagttgaaatcccctaatagtttctgcaagaccaacatactttcccggagaaccggtaaaaacttctgccacaaaaaa
cggttgtgataagaagcgctcaatttttcgtgctcttgctacagttaaacgatcctcctccgataattcatccaacccaagaattgcgataatgtcctgaagttctttgtaa
cgttgtaaagtttgcttaactctttgcgcagtttcataatgttcgttgccaacgatccgaggttgtaacatagttgaggttgaatctaaaggatctactgcaggataaatcc
ctttggaagctaatcctctggaaagtacggtagtagcatccaaatgtgcaaatgttgtagcaggagcagggtcggtcaaatcgtccgcaggtacataaaccgcttgg
atcgaagttatagatccctttttagtagaagtaattctttcttgcaaagaacccatttctgtactaagagtaggttgataacccactgcagagggcattctccctaataagg
cagatacctccgatcctgcttgaacaaaacgaaagatattatcgatgaatagaagcacgtcttgcttattaacatctcggaaatattctgccatagttagggcagtcaa
accaactctcatacgagctcctggcggttcattcatttggccatagactagagctacctttgattcctcaagatttttttcattaattactccagattccttcatttccatataa
agatcatttccttcacgagtccgttcccctactccgccaaatacggatacgcccccgtgagctttagcaatattgttgattaattccatgatgagtactgttttacctactc
cagctcccccaaatagtccgatttttcctccacgccgataaggagctaaaagatcgaccaccttaataccagtttcaaagatggataatttcgtatctaactcgataaag
gcgggcgcggatctatgaatagggaatgttgcactagtatctacaggacccaaattgtcaacaggctccccaagaacgttgaaaattcgtccaagagtagctccac
cgacaggaacactgagaggagctcccgtgtcaatcacttccattcctctcatcaacccatctgtagcactcatagctacagctctaactcgattatttcctaataattgtt
gtacctcacaagttacattaatttgcttaccgtcagtgtctcgactcttgactaccaaagcattataaatataaggtaacttgcccgggggaaaagtgacatccagcac
gggtccaataatttgatcgatacgccctgtactttttttcttcaattgtagaaaccccgggacgagaagtagtaggattggttctcataattatcacataattttcaaaaaaa
aggaatttatcgaaattttgatttttttcttgttgaataatgccaaatcaacaccaaaaaaatatccaaaaatccaaaagtcaaaaggaaatgaattagttaattcaataag
agagaaaaggggaccagcacttgatttcgttgcccaaacgaatcccattcaatcgtttactcatggaatgagtccgtcggaaagttcaatcaatctttttttcatatacat
tttgccttttgtaaacgatttgtgcctactctactttcttatctaggacttcgatatacaaaatatatactactgtgaagcatagattgctgtcaacagagaattttcgtagtatt
taggtatttccactcaaaataagaaaagggggtctattaagaacttaataaggattagaagttgatttggggttgcgctatatctattaaagagtatacaataaagatgg
atttggtgaatcaaatccatggtttaataacgaagcatgttaacttaccataacaacaactcaattcttatcgaattcctatagtagaattcctatagcatagaatgtacac
agggtgtacccattatatatgaatgaaacatattatatgaatgaaacatattcattaacttaagcatgcccccccattttctttaatgagttgatattaattgaatatcttttttta
agatttttgcaaaggtttcatttacgcctaatccatatcgagtagaccctgtcgttgtgagaattcttaattcatgagttgtagggagggacgtatgtcaccacaaacaga
aactaaagcaagtgttggatttaaagctggtgttaaggattataaattgacttactacaccccggagtacgaaaccaaggacactgatatcttggcagcattccgagta
actcctcagccgggggttccgcccgaagaagcaggggctgcagtagctgccgaatcttctactggtacatggacaactgtttggactgatggacttaccagtcttga
tcgttacaaaggccgatgctatcacatcgagcccgttgttggggaggataatcaatatatcgcttatgtagcttatccattagacctatttgaagagggttctgttactaa
catgtttacttccattgtgggtaacgtatttggtttcaaagccctacgcgctctacgtctggaggatctgcgaattccccctacttattcaaaaactttccaaggtccgcct
catggtatccaagttgaaagggataagttgaacaaatacggtcgtcctttattgggatgtactattaaaccaaaattgggattatctgcaaaaaattatggtagagcatg
ttatgagtgtctacgcggtggacttgattttaccaaagatgatgaaaacgtaaactcacaaccatttatgcgttggagggaccgttttgtcttttgtgccgaagctatttat
aaatcacaggccgaaaccggtgaaattaaggggcattacttgaatgcgactgcaggtacatgcgaagaaatgattaaaagagctgtatttgcgagggaattaggg
gttcctattgtaatgcatgactacttaaccgggggattcaccgcaaatactagtttggctcattattgccgcgacaacggcctacttcttcacattcaccgagcaatgca
tgcagttattgatagacagaaaaatcatggtatgcatttccgtgtattagctaaagcattgcgtatgtctgggggagatcatatccacgctggtacagtagtaggtaagt
tagaaggggaacgcgaaatgactttaggttttgttgatttattgcgcgatgattttattgaaaaagatcgtgctcgcggtatctttttcactcaggactgggtatccatgcc
aggtgttataccggtggcttcagggggtattcatgtttggcatatgccagctctgaccgaaatctttggagatgattctgtattgcaatttggtggaggaactttaggac
atccttggggtaatgcgcctggtgcagcagctaatcgggtggctttagaagcctgtgtacaagctcgtaacgaagggcgcgatcttgctcgtgaaggtaatgaaatt
atccgatcagcttgcaaatggagtcctgaactagccgcagcttgtgaaatatggaaagcgatcaaattcgagttcgagccggtagataaactagatagctagactaa
gtggataaaattagatagaaaaaaggtctaaataaaaaagaagagaaatagaaagatcaaaaatcagttacgaaaatgcagtaattcttctttttttcttctaattgattgc
aattaaactcggctcaatctgaaaaaagattgagccgagtttaaatagattttgatacgatcatgagacttgacaaatcgggattcctctattctatatatttagaagatat
aaaggtataatacaataaataaatacaaatatagtattatcatatgataatggaatcaaatacgcagtatttacagaaaaagttttcatttattgggaaagaatcaatgaca
tacaatgcattacagacgtatgatcattaccctttaaccgggttattctattccacttctagatagagaaaaaaactaaaggagaatgaatgaaaaaagacagagtttg
gaagttagacccctttctaagactctctttcaaaaaagaggacattttgaaacttttaacaggcacaatcgtgagtcaacaagtgactcgaaatgctcgtaagaaaaga
gaattgattttcaaaatggtagaactagatgacgaagttttctataaccttgatgaagaggtagttttagtttacgactccgatcaagagcgagaagtctttcatttcagat
tggactgctatagaatctggacccatcgtagagacgttcaaaaggctcctgatgataagaatcatactttcgcggaactccatggggctatgggcttcaacgcggta
gacgttttgttccgaatttttctggagcaaacctccgacccaacgatacaatgaatttttttctattcacaaagaaaaaagattcggaatcgcaatgctactatacgcaccc
ggaggaatattcggaattatattcttatataatccatttttgcgcggggtcttactaacaggacttcgctgcacgagacgaggattcgtcgaaaagctaaggtcatccg
gtattttcatacccttttgggggtggtatatggccttacaaagtctaaggggtagtatgagatctgtagtaggtaaaagaatttgtcccttgattgagtatgctattttccctc
ctttaccgcgcattattgtatatgcttctagaagagcacgtatgcagagaggaaattacagtttaataaaaaagcctaaaaaagtttcaactttacggcaatatcaatca
actaaaagtcctatgtatcaatccttacagcggatttgtggggtccgagagtggttgaataagtattgcatgtggaaggaagtagacgaaaaagattttggattcgaaa
taggcgcattcgactaagtcgtactttgaatccaatttcaagttcgattagaaggatagaaaggccgcgaggatcggaaaagaaaaatcaaatctttttaattgcttctc

cttttttgctattttcttattatttatataatccattcgattctttttttttagaatactaaagtattctaaaaaaaaagtattctataaaaaaatcttttttgcaaactcaaaaatacaat
agtcaatattcgttataatagatatacttaattatatcataagaatcttaagatatttttgaatagatcaaatcgaatagatagaaatagtaaatttgaatggagacacctatt
ctatgatggattttaacttaccctctattttcgtgcctttagtaggcttagtatttccggcaattgcaatggcttctttatttctttatgtgcagaaaaataagattgtctagaac
cgacgggaccgaattttctcaatgtatttccacacaggatcataatacggatcttttgtagtgtaagtaatataatatggtatgttatgtggctctttctacacacaaatgca
aacccgctatggatggggattatggatgcggatataggctacgagcataaatgcatgcatatgcggaaccgggtatagcgagttttttaagtggatcaacaaatact
ttttgaatagaaagtcaatgtatctaaccaattattttacaggagtatctagttggcgaaggcgatttcagaatcaaaaaaagtaaagtcaaaatcatttagcttattctctc
aatttcaatcgaccgctgttagtatatctaatatgaattggcgatcagaacacatatggatagaacttctaaaaggttctcgaaaaagaggtaatttttctgggcctgtat
tctttttctaggttcactaggattcttagcggttggggcttccagttatcttggtaagaatattatatctgtacttccatctcaacaaattctttttttccacagggggtcgtga
tgtctttctacggaatcgcaggcctattcattagcgcttacttgtggtgcactattttgtggaatgtaggcagtggttatgaccgattcgatagaaaagagggagtagtgt
gcatttttcgttggggattccctggaataaaacgtcgcgtcttccttcgattccttatgcgggatatccaatcaattagaattcaggttaaagaaggtctttttcctcgtcgt
atcctttatatggaaatccggggccagggggccattcccttgactcgtactgatgagaagtttttttactccacgagaaattgaacaaaaagctgccgaattggcttattt
cttgcgcataccaatggaagtattttgagtaccaattgaatttttgaaatgaattgaatgaagaagaattggaagaagaaaagttttctcaacacgagggaaaaggtc
ccttcgaaattgcattattgtaaggggattttgagtatttatctaaaggaaggaacaaatgaggataagagaaaattgcttctaatttgtccaagtgggatatatggcgta
atattcttccattttcatccgaaaggactttttctatttctctattccactccatctagatctaagaaagaactcaatgcaatgaaattctactagtatagaaaaaagaggaa
tagatacaaggtctcaaaccttgttatagaatttttccttcaaagaaaaagaaatatcatatagagtcagcgaataaagcggattcattaacaactcaatttacagatcaa
aaatgaaaaaaaagaaagcattgccttctttcctatatcttgtatttatcgtacttttgccttggggagtctctttctcttttaacaaatgtctggaactttggattaagaattg
gtggaataccaggcaatcccaaactctcttaactgctattcaagagaaaagagttctagaaagattcatggaattagaagaccttttttatcttggacgaaatgataaaa
gagaaaccgaatacacatgtacaaaaccccccctataggaatacgcaaggaaataatacaattggccaaaatagataatgagggtcatctccatatcattttgcatttct
cgacaaatataatctgtttggctattctaagtggttctttttttctgggtaaagaggaacttgttattttgaattcttgggttcaggaattcttctataacttaaatgactcagta
aaagctttttttattcttttagttactgatttttttgttggatttcactccacccgcggttgggaactactaattcgttgggtctataacgatcttggatgggttcctaacgagct
aattttcactatttttgtttgtagttttcctgtgattctagatacatgtttgaaattttgggtctttttttgtttaaaccgcctatctccttcgcttgtagtcatttatcattcaattagtg
aagcataaactcatttgatctcctgatattaatcaaaattagcatcctttttcctttagaaagaaagccttttttccattttagcaaaattctttttttctatttctacctgctcaagg
tattcatcattccagtacaactgttgcagtagaatgacaacagattcgtgtatagggaactagattagcttagctacctatctaatttattgtagaaattccgggatctgtg
attggacatggaaaatagaaatactttttcttgggtaaaggaacagatgactcgatcgatttctgtatcgatcatgatatacgtaataactcggacatctatttcaaatgca
tatcccatttttgcgcagcaaggttatgaaaacccacgagaagcaactggacgaattgtatgtgccaattgccatttagcgaataagcctgtggatattgaagttcccc
aagcagtgcttcccgatactgtatttgaagcagttcttcgaattccttatgatatgcaactgaaacaagttcttgctaatgggaaaaagggagggttgaatgtgggtgct
gttcttattttgcccgagggattcgaattagcgccgcccgaccgtatttctcctgagttgaaagaaaagataggaaatctctcttttcagagttatcgtcccaataaaaaa
aatattcttgtgataggccctgttcccggtaagaaatatagtgaaatcgtctttcccattctttcccccgaccctgctatgaagaaagacgttcatttcttaaaatatcccat
atatgtgggggggaaaccgaggaaggggacagatctatcctgatggtagcaagagtaacaatacggtctataatgcaacgtcaacaggtgtagtaagaaaaatact
gcgtaaagaaaagggggggatatgaaatatccatagtcgatgcatcggatggacgccaagtgattgatcttatacctcccggtccagaacttcttgtttcagaggggg
aatcgatcaagcttgatcaaccattaacaagtaatcctaatgtgggagggtttggtcaggggggatgcagaaatagtgcttcaggatccattacgcgttcaaggcctttt
gttcttcttcgcatccgttattttggcacaagttttttttggttctcaaaaagaaacagtttgaaaaggttcaattgtacgaaatgaatttctaggtcccgggatttcttaccatc
aagttggtaaaaagccgcgatttattggcgattgctagaattctctatgatccattttggaaatcttttttttgtttagactattttttgtttgcgagatgtcttgtctgcaattcct
tatttctatcatctcatgcaaaacgaggagaatgcaaggcaagggcgagaacaataaaaggaacaaatccttttaggagggattgctggtcttcttaatcctctattgg
gcacaagaaaaaggctttttttgccttttttcttgtgtcgattcttcttgtatcgaaatatgaatctttttttcttcctattcggcaaagattactatttcttctttatttgggtctgtctct
tgaacctcttttgcttaggttcaggcgatgtagtggacaaaaagagggaaagaaaatatggcgggggacaaattttttgtgaccaaatagaattgcttgacttgttcaa
ttaagttcaatttagaacttacagaaattttgcaaaaaaaaatggatactcttccattgaaggctgtttttttatttttaggctagttgagtagttttaattaaggttttattagtttatt
actctaaattaaatcaatgatttacaagagacttcctccgggtaataaaatattggatcctcgattgatcctttctttctcctcgcttcataaaagtgaattaatttcattggc
gagggggttataaatcaactgatggattacttcactaacattattaacaaacaaaattaacaaacaaaacgaataaatagagggattctgaccatcagatcaaaggctt
tctctttgttatttttacaaatcaaaataggaaacccgtttgtaggttatggaatagattaaaaaagtcgggttataagagtaagaattccgcgggtcctttccgctctaatc
agataaaagggggtaaggacccgctaagttcctattttttcatgtttacaacctggtccctccaattactatagagatgaacccaatccagaatatgaaccgtaaaaga
aaacacctattaaaccaatcacagcaataccggttacagtacctatcagccaaagaggaattcttccagtagtatcggccatttcccctactttcctccacattttatcaa
gtggtcatgctagagacaaaaacagtcatggatagttataaggatggtatccttccaaatggggataagagaattcttactactctctttctttctctcaattgaagaagt
aattggaaaataaaacagcaagtacaaaaatgagtaataaaccccagtatagactggtacgattcaattcaacattttgttcattcgggtttgattgtgtcatagttctata
gttggaatttggtttatcgttggatgaactgcattgctgatattgatcccaagaaaaaaacagtaggtacagctagtccgtgaacagccagccatcgcactgtaaaaat
aggataggttcgatctatggtcattgagggcctcctaaaaggatctactaaattcatcgagttgttctaaagaatcaaaacggtcggttattaacggaattccttgtcgg
ctttctgtgaaatactcgtttggccgaggacttccaaacacgtcataagctaaacccgtactgacaaataaccaacccgcaatgaatagggaaggtatagtaatgcta
tgaataacccagtatcgaatactggtaataatatcagcaaaagaacgttctcccgtgcttccagacatgctgagctccccaaatttttgtacattcaaaaaaggaattga
ttccgtaaaagatgggatcaaccagtaaatagaaaattactgatatttcatccttgtgagattgtcaattttgtaccaaaggtgtattttgagtataccaaattagtatagct
atccttcctatggcacagcaatcctgtttcgcttggtctcgaaacagaattctttttttctcttctttgttccttgtctatagggtaagctatatggtattcaaggcatcaatag
aaaacctcaattttgagggtcctacttaattttcaccggcttcggaatagtagaataattcggaatagggctcaagatcttgggaaaatctaagttaatgatcaacaggtt
tggataaagaatttaggaaagatattcttatactgacacaatacaaagagaagtagatgcgaaagctatcccttcgaatccaacctttccccttaaagaatttaattggtt
agcataatataatatctaataaatagaaaatcaaatagtagataatctgttatgaaagagagaaaacattctttgaagaatcaagattcgtaatcaacccttgccttgttta
ctaactttcttgaccaaactgcaagcgtggaactttgccagttcttaggtttccgaaatagtgtaatggaaaaagaagtgcttcgaatcgactttgattggggttcgaaa
aaggaattaaaaaaaaggaaattcaggaaagatttcctttttttaggagaccctcgaggggtcgtggaatgcttttcttctcctcttattccatatggaatacaatcaatta
aaataagaaggaataggggaatattcgactgttcgctccaaaaagaaggttaaatcatcctattgaaaaagaccaaaatagaaagaactttttcaaattcaattctttatt

tttcactggggttagctgatctagttcttaatattattactttactcaattgacagattacacagcaaatctcttgattcggaattagggactcatgccccatctgatgaatcc
attttcttttacacttctgtatctcactctatcttgtttttagtattatctaaaataaccgatgaattatgaattttccataacttaggtaagtgctttaccaacatatgtagtgtag
taaaaaaaatggaatttaaccctttcatgcttactataactagttatttcggttttctattggctgctttaactctaaccctagctctatttattggcttgaacaagatacgtctt
atttgaaatgaattgaatgaataaggcagaaaagaaaaaatctctcttttggattcctggtattctacgcactaattaccaattcttttttcttggtcattgagattcgtggat
aatttagactactatttagggataaatcgtacctcttttttttatcccctcgaacaaatcgaaatgattgaagtttttctatttggaatcgtcttaggcctaattcctattacttta
gcgggattattcgtgactgcgtatttgcaatacagacgtggggatcagttggatctttgattgagtaatatttctttttgattgccctcctctctggtctggaggaggtcaa
attggaattgcaatttgttttgttaagttattttgccttgcttcgacataagatagatggaatcacgctctgtaggatttgaacctacgacatcgggttttggagacccgcgt
tctaccgaactgaactaagagcgctttcaaaaatcaaatccttttctactcctaatgtgtctcacgtacgtatagtatccacaaattaaagttatacccgctttaatcgacct
cctcactactgcctataaagaagaaagaagtaataggtagggatgacaggatttgaacctgtgacattttgtacccaaaacaaacgcgctaccaagctgcgctacat
ccctttttccaaattgttgtacaatgtcattgtacacaattcctatcttgttttccacatcctaattttcttgtctttttctatctatagagaatcctcgtgtcatttcttctttttggtct
catataatcaaggaatggtatatatctaaatccaatctaatttcacctataaaagaaagattactattccttggtaatctataggaagggattgtctttttcgtttttagttcgg
aatttcgcctaaacaaaagaaatacaaatgatcttgggcaataatatctgatcatatacgtattccaataaggaaggaggattttcaatgcgggatataaaaacatatct
ctctgtagcacccgtgctaagtactctatggtttggggctttagcaggtttattgatagaaattaatcgtttatttccagatgctttgtcattccccttttttcattctagttgtt
gctatgtgaggaatagagttcttcgtgacatgataaaaaaattcccaccctttttagtataggaaagaaaaagaaagaaaagatagattgggttggaccttagagtcatg
aaaaatttggtaaatctcatttttgaaaaaagaaattcaattaaaagcagtatccaagctaagtcaggcctcagaaatcagagcatagaaagaggctgggttggctac
taaaatgaaaggattttgcttcaaaatccttgctaatttgacaaggattgtattccttaattatttctctattttttattacttaattgaaaaatttccaaaaatttttattctaatgg
attttcttcttcctcttcggtttcaaaatagaggaataaaagaataagtagaagaattaagttaagtcaatccaaaaaggaaaggaggttcatggccaaggggaaagat
gttagaatcagagttattttgcaatgtgtgagttgtgttcgaaaaggggccaatgaggagtcggcagggatttctagatatagtactcaaaagaatcgccacaataca
cccggacaattagaattaagaaaattttgtcgttattgtcgtaagcatacgattcatgccgaaataaagaaataggagcatcttgtgttcgatctttccaaagatcaaaaa
gaataagaacttcctatttaatattcctatttaatatatagagcatagatagaatacaaaatacaaatcaacttgtctgatttccattagatattatttcatatgtatcgagggt
attcatctaatatatggaccaaagagagactacttcttctggatccaaaattaataaaataaacaaatcaattttttttcaattttaaaataaggaataaatcatgtatacatct
aaacaaccctttcataaatccaaacaaacttttcataaatccaagcaaacttttcgtaaatccaagcaaacttttcgtaaattcaaacaaccttttcgcaaacccaaacaa
ccttttcgtaggcgtcctcggattggcccgggggatcgaattgattatagaaacatgagtttaattaatcgatttattagtgaacaaggaaaaatattatcgagacgaat
aaatagattaaccttgaaacaacaacgattaattactcttgctataaaacaggctcgtattttatctttcttaccatttcgtaactatgagaatgagaaacaatttcaagccc
agtcaatttcaataattacaggtcctagacccagaaaaaatagacatattcctccattaacacaaaagttcaattccaatcgaaacttaagaaactccaaccagacttta
agaaacaacaatcggaacttaagttccgattgttgatgttttattcgaaagggccagactatatataaagaaagtaatccaatttagattcttgggtttgttataagaaag
aacaatggggaagaaaaaaatagtttttttatttattgcaacatgctcgttgattcctaccacttaatcttaatttattgtatcttcccggagttacctctccgggaattctttttta
attattcctgtatattactttttttatccctttaattgataatctttattttattggaaatcgtgtaaagattatttggatttaatacagctacttgtgcaaggattttacgattaagaat
caattctttcttgtaaagattgtgtattaatttactataattatcgaatactttatgtatccgcgttgctgcgtttatccgactgatccacaaacgccgaaaatccctcttttgcc
tgactctatctcgatgagaggaaacaaacgctcttcttacctgttgagtaatcattcgattaagtcttaaatgagcccctctaaagtttgaggcaaatgaacgcatttttgc
ccgtcgtctccgagctatatatcctcgcggaactctggtcattgaatcaaattaaccttaatgaataactaatgatttctcttcttttagccatcctttttcccattaataacaa
aacgaattattcccatatataaaatattagttccaatggcttttgctactataaccttcccaaccacaattttctcttctactcccttcagttatttcgcatagaaataaaaaatt
ctaacgatactaaaaaatagtgggttccatcgtttctatggttcccttttaaacggcgaggccctctctatacaccggagccctttctttcatttcatcaaagggtattgtg
aacttgtatagttcacattctttggctctacctatccattatagagtaaatagctcttttcacaataagagttatccatacagtgacggcatttaattatgaaagttggctaag
tagctgaccctcttagtccgtttttttaagataaaggagcataagcctttatctttttattactatttcctccgcttaatgaatagccgtttgctaccaatgggggaattgcttc
ttatttccaatctagatgattggatttgcaccaaaggaaaccagaaattccatataccgtagaaatctaggatagagaagctctatcctattcattggtaccgatcatgga
tacttcaaaaattgtcttatttgtttgaacccatgatccgaacgagtcgcacatacaccctagcacatgttcctcgacgctgaggacatcccttaagcgcggccgatttt
ctagcatttcgtattggctgtcttgcgtttctaataagttgtttaaccgttggcatgtcgtatgtatatagaaaaaatggattggtttagatcgatcttaacctgaatgattgat
cattatgaagtatttccattaaattgcataaaacccgaatttagggttgaaataaatttacaagaaatctggccactaccaatccttaaacatttctggaaaccacactgg
atcagtatcgcagtgctcgtcaagcatttcatcccctacaatatcgacaagtccataagctttggcttcgtctgctgacataaaaacatccctttccatgtcttcggatac
aacccaaaaaggcttgcctgttcttagtgcataaacccttgtgatcatttcgcgaactttgtgtaactcttccacttctagtaaaaattctggtgttcttgcccgataataag
cactagcaggttggtgaagcataatcctcgcgtgggggaatgctatacgcttggtgggttctcctccaagcagaatgaaggacgccatggacgcggctattccga
ggcatattgtatatatatctggtgtcaccgtttgcatcgtatcaaaaatcgccattcctgagattaaccacccgccgggggagtttataaacaaaaaaatatcgctaattc
catcttctatactgagatataccatgagacctgtaatatgattcgtgacctcgcaacgaatctcttgacctaaaaaaagtgtcctttctcgatacataacattgtataagtc
aacccaagtcgcttcttcatctccgggaatccggtaaggtacttttggaacaccaatgggcatattagattaatattattaaatttaagtaagaaaactacactttaatatg
gaaacgtaagaatggaagagaaagaaagaatccgcagtttattgttttcactttttttcttattctatatgaatactatagattctattaatacgtagattgaaataatacata
gattgaaagattatacatataagtaggataagacagattgaataaagaaaaaagaatgggtgattcgaatactaaacaaaaagagataggatctattcttcgtttttc
caaataagccaagctgcccattgcatattggcacttatcgagtatagaatagatctgcttctctttcttcttacgaacagaattggcttcttatttttaatggaatgaaataa
atattcacgctttctgacacagaatcccctagaagggttaggtacataggatatggatagtctttgccaatgcgataaaataaagtgacatcgtgtctatttttctttgcta
aaggggtatttccatgggtttgccttggtatcgtgttcatactgtcgtattgaatgatccgggtcgattgctttcggtgcatataatgcacacagctctagtttctggttgg
gctggctcgatggctttatacgaattagcggtttttgatccctctgatcctgttctggatccaatgtggagacaaggtatgttcgtcattcctttcatgactcgtttaggaat
aaccaattcgtggggtggttggagtatttcaggaggaactgtaacgaatccgggtatttggagttatgaaggcgtggcaggggcgcatattgtgttttctggcttgtgt
ttcttggcagctatctggcattgggtatattgggacctcgaaatattctgtgatgagcggacgggaaaaccttctttggatttgcccaagatctttggaattcatttatttct
tgcaggggtggcttgctttggctttggcgcatttcatgtaacgggtttgtatggtcctgggatatgggtgtctgatccttatggactaactggaaaagtacaagctgtaa
atccggcgtggggtgcagaaggttttgatccttttgttccgggggaatagcttctcatcatattgctgcgggtacattgggcatattagcgggcctattccatcttagt
gtccgtccacctcaacgtctatacaaaggattacgtatgggcaatattgaaactgtactttccagtagtatcgctgctgttttttttgcagctttcgtagttgccggaactat

gtggtatgggtcagcaactaccccaattgaattgtttgggcctactcgttatcagtgggatcagggatactttcagcaagaaatatatcgaagagttagtgatgggtta
gccgaaaatctcagtttatcagaagcttggtctaaaattcccgaaaaattagccttttatgattatattggtaataatccggcaaaggggggattattcagagcaggctc
aatggacaatggggatggaatagctgttggatggttaggacatcctatctttagagataaagaagggcgcgaactttttgtacgccgtatgcctactttttttgaaacatt
tccggttgttttggtagatgaagagggaattgtgagagcggacgttccttttagaagagcagaatccaaatatagtgttgaacaagtaggcgtaacggtggagttcta
tggtggcgaacttaatggagtaagttattctgatcctgctactgtaaaaaaatatgcgaggcgttcccaattaggggaaatttttgaattagatcgggctactttgaaatc
agatggtgtttttcgcagcagtccaaggggttggttcacttttggtcatgctacctttgctttgctcttctttttcggacacatttggcatggggctagaaccttgttccgag
atgtttttgctggtattgatccagacttggatgctcaagtggaatttggaacattccaaaaagtcggagatccaactacgaggagacagccagtctgaggccgcattg
ctttggtatctttcacctctctttttttgatttgacatgggaaacatctcccatcccttctttgactctttttcctttttttatacgggaaatgatcccaaatgacaaatgaataggtg
tggaagttataattgtaaataaaccacgatcgaatctatggaagcattggtttatacgttccttttagtttcgactttagggataatttttttcgctatcttcttccgagaacca
cctaaggttccgactaaaaaagtgaaataatttaattgaagtaagaagtctccccatctgggagacttcttacttcaattagtccccgtgttcttcgaatggatctcttaatt
gttgagagggttgcccaaacgcggtatataaggcatacccagtaaagcttacaagtaaaccagatatggagatggcgactaaagttgctgtttccatttttatagaatt
tcaagattacaatggatctacgaaaagatcgtgtatttacaactacaacggaatagtatacaaagtcaacacaaatcattaaatggaatttatggctacacaaaccgttg
aagatagttctagacctggaccaagacaaactcgcgtaggtaatttattgaaacccttgaattcggaatatgggaaagtagctccgggttgggggactactccttttat
gggggtcgcaatggctttattcgcggtattcctatctattattttagaaatttataattcttccgttttactggacggaattttaatgaattaggtttctactaacgaaaactac
gaagtcatagtttttccatccaaaagagcctttctactttaagctctacatttctagacattctggtagttcgaccgtggaatttttttggtttcggtatctctggaatatgagtg
tgtgacttgttagaatttgctcctattgataatacatagaaagcacctgttatctctatcaagatgattctaattcgtcggatattatttattctagtatctggaacacgaaata
gatagagtggatcaagaaaaaaaaaaatgaaactatgattcatattaactattcagacctcgcaaccagactgaaaaaaaattcaagtagttcttaataaaaataaaaaaa
gaaaatttcttccttccaattttgtttgcccaaaaaacaactttttttctctcgattttgtcgagtcattacaccgattcaataaatgatcatcaagcggttcttattcgaagaa
cccttgccttttgtttagcttgagactcaatcatcgtggctctagtatgaatctaaggttttaattgaactgattcataggatcgcaacaagataatttctaccagaaaacta
ctccaatttttgctttatttatttatctagtaaaacaagagtaaatctgcattacgcacaaaaaaaaagaaatccaaaatagggaagagaaaaatcaagaggcctctaatg
atcaacatttgggaaagaaagatagacgagccaacttgagatttttttggcattatcatcacaaagaagaaattctggatttttcttatttcatatcttcaaggcaaatcgac
ccaacccagtggctgatgaagttttgaacctttttctaatatccgttgaaaatttgtgtgtttctgtttgagccgtacgagatgaaattctcatatacggttctcggagggg
ggttcgggttagttacctatctcaataaagtatatgattggtttgaggaacgtcttgagattcaggcaattgcagatgatataactagtaaatatgttcctcctcatgtcaa
catattttattgtttagggggaattacacttacttgttttctagtacaagttgctaccggttttgctatgactttttactatcgcccaaccgttacagaggctttttcctcggttc
aatacataatgaccgaggccaactttggttggttaatccgatcagttcatcgatggtcagcaagtatgatggttctaatgatgatcctgcacgtatttcgtgtgtatctca
caggtgggtttaaaaaaccccgcgaattaacttgggtcactggtgtggttttagctgtattaactgcatcgtttggtgtaactggttattctttaccttgggatcaaattggt
tattgggcagtcaaaattgtgacaggtgtgcctgacgcgattccggtaataggatcacctttagtggagttattacgtggaagtgctagtgtgggtcaatccactttga
ctcgtttttatagtttacatacctttgtacttcctctgcttactgccgtatttatgttaatgcactttctaatgatacgtaagcaaggtatttcgggccctttataaggaaggcat
ctcatagagagttctaattctcatatatcatatcgggtaggttgtggtatttcattgctacaaacatgggttattctaaaataagacatgtcatttagatacttctcttcaactc
cgaactattgtgatacaaataatacaaatagttgaagttaattttacgaaagaaaagaaggcggattatgggagtgtgcgacttgaattattgatttggccatgcagata
gagagttggatctgccacattagaattcacgaccaaaggtgtctccatatccaatcaacacgtaagtcccctgtctagtaaggataggctggttcacttgaggagaat
attttctatgatcatacctcaaccatgtcatccatgaagaggctccgtaagatcccatagagtagaaatggaataagtcatgtgacatgatccaattctctatttattacac
ttactttttattatagtatggaaatgcattcattttctttgcatcgattgcgatctgcaatactatcggagtaaaagaagggatctaaggaagaacgtaggctaaactttttg
attttttattagtaacaagtaaatactttgtttggacgtaagaaacttgcaatattgggggggataaataccaactaatcaagagacgcgagacaatccacaaagcaatt
gatcatgatcaaatttgtaagcccacttggatattgagcatttacccataagagtaggattcttttcaatgaatagttgtaggtgcaacttcggaaaatagaatctgataa
agcttttcttacttagagccattgagccattatataccttattctattatggatcttctacggttttatttctttcattcttgctcgagccggatgatgaaaaattctcatgtccgg
ttcctttgggggctggatcttaaagaattcacctatcccaataacaaagaaacctgacttaaacgatcctgtattaagagcaaaattagctaaagggatgggacataat
tattacggggaacccgcgtggcccaacgatcttttatatatttttccagtagtaattctaggtactattgcatgtaatgtaggtttagcggttctcgagccgtcaatgattg
gtgaaccggcggatccgtttgcaactcctctggaaatattacccgagtggtacttctttcccgtgtttcaaatactccgtacagtacctaataagttattgggcgttctctt
aatggtttctgtgccgacgggcttattgacagtaccttttctagagaatgtcaataaattccaaaatccatttcgtcgcccagtagctacaaccgtttttttaatcggtactg
cagtagctctttggttaggtattggagcaacattacccattgaaaaatccttaactttaggtctttttttagggatttttcagtttgattcattcaatcgtgaagtaccgtgcata
ggtatctaggaaatagttacttccaagtgaatcttccctagatacctataatctattttattatgatccatttcgcgaaaatatagattgtcccaaagatgcaaaattgttttct
tttttctttttattctaactcgaaaaagaagaagaggaaaaaattgcaatggatttaaaactagaacttattcttaggtaaatccattgggagatgcttctctagagtgtccc
atatctgttttccatcttgcatacgaaaactgtcaattctcatcagatcttcttccgtcttactcaaaaggtccaatagtgtatggatattggcccttttgagacaattatacgt
tctagaaggcagttctaattgatcaataaaaaatacaattcaatggaattctttttttgtttttctttagattagttaatctttttttgaaagcttaaaagggggggaagtaaacctgt
ttttattttcttggaaacgagtaccctcttcctccgtgtgaagaaaaggaagaaataaatcaatcaaattacgagaagcctcataaagcgcttccttaggggttaaacttc
cattggtccatatttctagaaaaagtatctcatattttgcatttccattcccacaagcaaaaatactataattcacatttcgaacaggcatggatacagcatctataggata
acttccatcttgagtgttctttctgacttccgtgtgatatccgcgatctctcttgatccgtaactcaatacggaaatccgtgggttctgtcaagttagctataggttgtgccg
tatcaacgatttctacggaaggcggtaagataatatcttgggcagttatgtatctaggacctttgacgcaaattgatgcggttctaactccatagagattacttctcaata
caatttctttcaaatttagtaaaatttcttgtaccgattcttcaatacctgctattgtagaatattcgtgcggcacgctcccaaattttgcatgtgtgatacatgttccttctgttt
ctccaagtaaagctcttcgcaaggcaataccaacggtatccgcttgaccttttctaagcggggacagaatgaaacgaccataataaagacgcttactatctactcttg
attcaacacacttccactgtagtgtttgagtggatcctgctacctcctctcgaaccatatagactagtattattatttgatcattgaatcgtttatttctcttgaaaggggttta
attcttttacagacgtcttttttttaggaggtcgacacccattatgcggcataggtgttacatcgcgtatacaacttaatcgtacaccacttttagcaatggctcgtaatgcg
gcatctcttccactaccagcaccctttaccataacttctgctcgttgcaaacccactgtacgaatagcatctacagctgttctttgaccagcatagggtgatgcttttcttg
agcttttgaatccacaagtacccgcggaggaccagaaaaccacccgaccttgcggatctgtaacagttataatagtattgttgaaactagcttgaacatgaataactc
cttttgttattctacgtgcactcttccgtaaactaaaacgcgcattcctacgcaaaccaatacgtactttcctacgtgaaccaatttttggtatagcttttgtcatattttattat

ctcataaatatgagttagaaataacaaaaagaaaaaaagatacaaagatatccgtttcagggtaaaatatatcctttacttgaattttttatttggaatttggacattttcgc
gggtactttttttactttttagaaagtcaagttcttttttcgaaagattacccctgtctttgtttatgcttcggattggaacaaatgactctaattcgtccacgcctacgaatca
gtcgacattttgtacaaattttacgaacagaagctcttattttcatatttccgtattcctttcttaaactatgaatttactcttttggaaaaaataagtctctttgtttgaattttaga
actttgaatttttaccctagaaaaaaaaaagaaaaacctaaccctttgaatctttggtatccttcaaatcttcggtatccttcgagtcttcggtacgcttcgaatccttatgg
ggaagtctataaattatacgccccttgcttgaatcataacgacttacttcaattttgaccctatcccccatcagtattcgtatagaactagaccggatctttcctgaaatata
gcccaggatgatggtgtcattctctaggcgaacgcggaacattccgttgggtagggcttccataactaaaccctcgaaagtaatttttgcttctctcgggtttttttttct
ctcctatttttcttttctgtcatattttttttctcctatttttcttttttttattttcaaataaaaaaaacgttgtatttttgtttgaaaaataggaagttcaagatagaatttgggtactataa
ctataggtggggcgattaccgctataggcggggcgattaccatatataacataagacttctcccccaattctgtttagtcgagcttctcgatctgtcattatacctcgag
aagtagaaagaatagcaattcccattccgcccaaaaccttaggaattccttgatagttggcataaattcgtaagccaggtcggctgatacgctttaaaaaggttctagtt
ctatatattccctttctagtctttctcttttgatgtcgcaaagttgaaaccaagaaatatctgttactttcctgatgtttccgaacactttcaataaaaccctctcgtagaagtat
tttaacaatgttttcggtaatatttgtagatactacccgaacagttcctttttttattcatgtccgcgtttcttatagaggttagtaaatcagcaatagtgtccttgcccataaga
ctctaattctaggttcctcctaatttttctataatcaacatgttctcttttttcttttcgttttggattctaaagcatatacgtgaaacacaatctactaatttttttatttgatctatatct
tgtctactagtatttataatacttcaggagctaatgaaactattttagtaaaattcaattctctcaattcctcggcgatcgcgccaaaaacgcgagttccttttggatttccttt
ttgatcaatgataaccgctgcattgtcgtcatagcgtattattataccgtcttcgcatttgaactctttacatgtacgtacaattacagctcgaattacttcggatctttctaga
ggcatttggggcactgcgtctttgattacagcaacaataacatcaccaatacgagcatatcgctgattactagcagctcctatgactcgaatacacatcaattttcgag
ctccactgttatctgctacatttaaaagggtctgaggttgaatcatattattttgatttcaatttgttatttcaatgcaaaaggatgaaagaaatattgtctttccagaaagaa
gaacctggtttttttattttcaatactactttttgggggggttatatttctaaccgaagaaattgacttcgtatgggcattttgctggcagctatggaaatagctgctctagcta
cagtttcggatactccgcccatttcataaagtattcgacctggtttaacaacggctacccaatattcgggggatccctttcccgaacccatacgtgtttcagtcggtctta
ttgtaaccggtttgtcgggaaatatacgtacccagatttttccaccacgacgtgcatatcgtgtcattgctcttcgtcctgcttctatctgcctcgccgtaatccaagtggg
ttcaagtgcttgaagagcatatctaccaaaacaaatacaattgcctcggtaggattttcccttcattcttcctctatgttgtttacgaaatctggttcttttggggttatagtcg
atggttctctcttagttccatctctactgcaaaactggacatgagagtttcttctcatccagctcctcgcgaatcaaatgagaaagtgtgcaaatttctctaattccacaat
attctaaaatattacggatagatacacattaataggaaaggttagggtttttaatattgaatttattaaaatactaaaatatatagtcaagaaagaggatctagaatatatct
agatgtgtatgtttatttatctatattctatatttaatgtaattcttttttttttttttttgaatctcctttttttattttactcttattgaatcgcggtaaagtattctaattcaataaaaaatttc
gcgggcgaatatttactctttcctgtcttatttgttaattcataaccttatcaaataagacaatttttttggtttgttccgccatcccacccaatgaagtattgggattcttttcaa
gaaaatcctatccagtcataggttctgtcgttcccactgcttctcctttaatggttaggtttgaatctcgcaatggagcttccaaaaaatttctttccgagtcaatttctcagt
tttattaactcgggacgctctttgttattccttgaaatttgtatttcttgtttcaatcaaattacgatttcgaattctctgttatttttattatattgatgctttatcacattgctttttatg
atgtaattcatagaccatacatattggaatcctatatctttcttatttctcttccttctttctatcatccttccttttatctacatcccctttagttttgcttcacaacttagaatcctat
tttttttaaagaaaaaaattgcagttgctacacgtatatgatagatttactcatttatgatagatgtatttattcatatagtgactgtttcttagttaggatctcgacaatacgaag
caataggttggttattagttcattttctataattacatagtagggttcattctattttttttttaatccccttttttgaaccctaaaaaaaaaactaacgagtcacacactaagcatag
caattttattaaaagatttctcaattttcattaaatcttatagaaagaggtagaatttcttctttttttcagggattttagggaaaataaggctcttgtcatttttttattctattactg
aacagaatgggaagacagggttggttattcttcgtctacgaatatccaaattttaactcctaatactccatagatagttcgaattggatagcagcaataatcaattttagc
gcgaattgtttggaggggaagtctaccttttttgatgcattcggcacgcgcaatttcttttcccgcgagacggcctgcaatttttactttactccctttatatctgtttttttag
ttaattcaatggctttttttcattgcctttcggaatgaaactctatttttttaattggaaagctatatattctgcaagaatgttaggttgtctataaggttctttcacttttttcgatccc
aatattaagtctctggtttacagaattaacttctttttgtagatctttctctaattcttcgattgctcctttttttctttaataaattggggaatccaatatggattatgacgtggattg
tatcgatttctttttgaatttctatatgtgtaattacttcggaacttgagcctgagtccatttttttattatgtgtaattacttcgaaacttgagtctgcttctatttttctattcgagc
cctttttcctattcttttgtatatagttcttgatacaattccgtattttttttatcttcctgtagaccttcagaataatttttttggttgtgcgaaccaaaaggaatggtgattttgggtt
gtaccaagtctgaaaccaagtggatttatttttttgtcccatatttttctattctattttttttttaccgggaatcaaatcttagatggatctaaagattatttcgatttctttactatatt
tagtacaattgttatatgacacatggttttttttatgggagaactacgtcctcgagcccgaggtctgaatttattcataatagtactcctactgacttcggctttagtgatgaa
taaatttgctttgtcgaaatccctataatgagtagcatttgctgctgccgaataaaccaactttaagatgggataagatgctcgataaggcatgaggttcaatatcataac
agtttcttcgtagtaacgccaacgaatctcatccagaactctttgtactttgaaaacggacatgtggatgcgtttttgtgctttgaaaacccgttcgaacttaagtagacg
atcggttggtacttttcttgcgagtttccttagcccactcttcttcttctttatcctaggggtatactttaccagtttgaaacttgtcataaataaggttattccccgcttacctat
ttcttttttttttattttgaatctttctattctgaattcagttaacgacgagatttagtatcctttcttgcactttcataactcgtgaaatgccgagttggtacgaattcccccaattt
gcgacctaccataggatttgttatgtaaataggtatatgttcctttccattatgaatcgcgattgtatggccaaccattgcgggtagaatgctagatgcccgggaccacg
ttactattgtttctttctcctccttcatattgaccttttctatttttgccaataaatgatgagctacaaaaggattcgttttttttcgtgtcacagctgattactccttttttttccattta
aagagtggcattcgatgtccaatatctcgatcgaagtatggaggtcagaataaatagaataatgatgaatggaaaaaagagaaaatcctttagctggataaggggc
ggatgtagccaagtggatcaaggcagtggattgtgaatccaccatgcgcgggttcaattcccgtcgttcgcccatcccattattgcaaattccaaaaatgcaattttcc
atattcctagttacgtatttacttacggcgacgaagaataaaactatcactatatttttttcctttttcctagttcttcttccaagcgcaggataaccccaaggggttgtgggttt
ttttctaccaatgggggctttcccttcaccgcccccatgggggtggtccacagggttcataactacccctcttactacggggcgtttacctagccaacacttagacccg
gctctacccaaactttttttggttcaccccaacattacccacttgtccgactgttgctaagcagttttgggataccaaacggacctccccagatggtaatcttaaagtggc
cgatttaccttcttttgcaatcagtttcgctacagcacctgctgctctagctaattgcccacccccttccacgtgtgatttctatgttatgtatggccgtgcctaagggcatat
cggttgaagtagattcttcttttctctcaaaaaaccccttcccaaactgtacaagcttcttccaaagcatacggctttctagatgtatatgacgatctctagacagatggat
cttatatgaatcgtatgatgaagtaccacatgagtggatatataggaaaggaatccaaatctgccgaatcgctcatgttatgatcttctacatcctaggtctccgcgttcc
gtcatctggcttatgttcttcatgtagcattcagatcgaatgactctatgaaattacgtcgatacttctacatattatgggtaacgtaggagacatccctattttcccccggg
ggtcttaattaccactgcttagctttcaattcgcctctgaccatcaaattaaatgtgaataacccgtcctcctctctttgaaacaaggggcgcttccggttctgtgcgtgct
tcaaacaattttgtcttctccatattaccatatctctagagtcaataattttctatgaggaactactgaactcaatcacttgctgccgttactcaacagttttctgttgaggtct
atcccgtagaggtagtcaaattggatcagtgatcgatttctaggtttcgtcgtaaacctaattggttacttccaattacgtaaatcaatagttcaaaccgcactcaaaggt

agggcatttcccattgatataggaacttttgtaccagaaacaatagtatctccaattatagcccctctgggatgtaaaatatatcccttctcaccatccccatagtgtatga
gacaaatgtacgcatttcgattagggtcgtattctatggttacgattctaccagatatgtctttttgattccgtcgaaaatcgattttacggtataggcgcttatgacctccc
cctctatgccttgcggtaatgattcctctggaattacgacctttaccacaacggtgccgtccatggatcaaattatttcgtggattggatttcacttgcctatctatggttcc
cttgcgtgtgctcgggataggtgttttgtataaatgtttcgccgtattattaagtattctcctttagtttttttctctatctagaagtggaatagaataacccggttgaagggta
atgatcatacgtctgtaatgcattgtatgtcctagaatagggcccattcttctaccctttccgggtagtcgatggctattcacagctactaccttaacaccaaagaagagt
tcgacccaatgctttatttctgtcttagtgaatcccgattcgacattaaaagtatattgattctttcccaataaacgaagactttttctgtaaatactgcgtatttgattccatc
cataaatcgactttccctcctatgctctgagttccagtatcgataagaattcgagttcttattgttcttatgttatggtatgaatataccataccaattcgttatgtatggatgat
ggatgagattccatggatagagagccagttccaatagacttatggaacgttccggttcgcgtgcatccagcaggaattgaacccgcaaatttaccaattatgagttgg
gcgctttaaccattcagccatggatgcttaacagggatcatcgtacatcgtaaataaccaattttcatatagaaagacatatcatagaaaaatgaaatcgaaaatattcg
gagatggcaaatattcggagatgactatgaaaacacctctctggatcctcgaattgaaagagagattgagagggatccagaatcctaattctcgctatttggaatgga
tccaattctattgagtctgactcatagtgatcatttctctttagcaaagaatgaccttggttatcaaaggattgaacaaccgggatccgtttacttatgatacctagttgaca
ttgataacaaggatctaatgaattatgagtttaatagatcctctttagcagaaagacgtatattccttgctcatcacttattcccaaacctcgtatggggctaatcgttttcat
ttaccatctcagggaaaatccttttcgttccgcttagccctatcgggtattttagtgataggttctataggaactggacgatcctatttggtcaaatacctaatgaaaaattc
ctattttcctttcattaaggtacgagggcttcttattccacaagaaaagaacgaaagcaccttttcattctttcatatactgggggtttttacttggaaaagacaatgttccat
actaaaggattcgggtccataaccacgagttccagtgcactagatcttgtagcacttagcaacgaggccctatgaatagacatatagaatttttggtcgggaaattcg
aatgaatcattgagtgaaaaaggagcaaagaatgacaaaagacgagactctactagtcttcactcttgtggtttcctcggtttctgttttcttattcgggatcttgcttttca
tggttctcatctctgcaactcgcgattttcgcgagagaaccaaatccaagttggtgaagatcatgatttgggctggcatagtagtgattacctttgcaattgcggttcga
atctatccgatctttatctttttgctcaaagaacgaataaaaccccttgtcgaagccctttatgataagcttccctggatctgggaagtttctctttcacggtattgggatcg
tttgatcgatttccttgatcgctacttatgggcgtgcgctcaaaggatacaaacagggattcgcaaacaaaaaggggaattcgtagtcactttttcctgtcgcgtaaaaa
aaaggctttacgcgagagcaatagaggttgggatacatctatctcttctgagcaacctcttttggattcttaagaccacccttgcagtaggataccgtctgctttgggttc
tttattatattatctccttcgagggatttttaggatcgttcaggctatatttagtctattttggcttttactgtctcctttttctcagggaagtggttaaggacctcagaagataga
ggagagcgccaggcgcagatttccggaatacttctacggggaatgctcattgaatgcgcattctccgtattatgccttgaagaggactcgaacctccacgctctttag
cacgagattttgagtctcgcgtgtctaccatttcaccatcaaggcatcttgaaagtgaatcatattccatgaatatgatatctatctaatgtgatatcttgaaagtgaatcgt
attccatgaatatgatatctatctaatgtgatatatggaatatatgacaaaggtggagtcttggagtatttcgatcgatcggtcatataggcctgagtcagacatcaaata
gcttcgatttgcattatccgtaggacaccttatatgtatcaaaatcaaaaagatgtacaatccaatttctcgattcaatagaagcccaaagaggtgcatatggtacccaa
ataaggataggatagatatgtcaaaagcaggtctgattacacctattcctaatcctaaatagaatgtaaggacgtggggatttctatgtaaacagagtatcctatttccat
aggctcgaatgacccсttctcataataagaatgtgcacggtctggtccggtatggaatgaacttataatctgatgatcgagtcgattccatgattataagttcattaccct
agcgcccattcccattttgggcggaacagatctactaattcttttattccagttagtaagagggatcttgaactaagaaatagacctagcagctaaaagagggtatcct
gagcaattgcaagaatggggttcattgatattcctggtatagtagatgctatcacacatacagtcatactcaattcgatggaattgtttgatcttaaggggatcttctata
atttcgcacataaggggttatttcttggtttcgtccagtcattaataacttgactatttttagataatagtagatagaaagaacgctcgtaaggagtcctattgaaaccaag
aaatataggcctgcttgccatccacaccagaatagatagagttttccgaagaaacctgctagtggaggaaggcctcctagggataagagacatagggctaaagag
agagccaaaaaaggatctttcgtgtataatcctgcataatctcgaatgttatcagttccggtacgtagaccaaataatacaatgcaagcaaaagttcctagattcatgg
agatatagaaaagcatataagttatcatgcttgcatatccatcatttgagtctccaacaattattccaataattacatatccgatttgccctatggacgaatatgcaagcat
acgtttcatgcttgtttgagtaatagcaaggagattccccaatatcatgctaagaatagctaggatttccagaagaagatgccattcgtttgatgagaaataaaaagga
atatcgagaattcgcgtggctgaagctgaagcagcaactttcgaagtaacagaaagaaaagcaacgactggagtgggggagtcagagtcgaaaagaggattcct
cgcttctttctctcatgcaaaaccgtgcatgagactttcatctcgcacggctcctaagtgataaaagaaagaagaacttgtcttctttctttttttgattaccttcctcgcgtat
gtataagaccgaatccattctttttcgaaatcgatttcgaaaaagaactactaatccttaacttttcgaggaatccttcatcagtggttgtgaatgactgactttttcaatcct
ttcgaccttggttccgtaggagcaagtcagaaaggttgagaaatagaaccatctgatttgattcgttcccaatagccatgagatgatcatcttagggtgatccttttgtca
acggatgctcctattacactcgtagtctctgaaggatgagaacccactatgtagcatctacgtcgataattcaagcattgtatacgtcattagtccgattctttgtaggaa
ctacccgtaataacgagcttgcaaaatggatctgtttatcataaagagattcgttgttcctgaccctgcttcaccttaattgttatttgaacaaaaagatcacaataaacttt
tggtaaaagttctgtcttggtcggagtggggatagcatttctcttctgcatatctatggagttttgcaaaacccaaacacctcagagatagatatagaggtaggaatttgt
cgaacgaaccacactccttcgtagacgtcaggagtccattgatgaaaaggggctggggaaagcttgaacccaagtcctacagtgatggatataagcgcaattgaa
attcctggggagttatacatttgtgtattgataagaccgttcacaatttcttgaagctcgatctccccccсagatgaaccatatagccaagagaaaccatgaaccagaa
tagaagagcttgccccacccatgagtaaatatttcatagtagcctcattagaccgtagatctctcttggtatatccagacaataggtaggaacataaactgaaacattct
ggagctacaaagatagttattaaatcgttagcaccacataaaaacattccccctagagtagctgttaatacgaataacagaaactctgttatagccatttctgtacattca
atgtactctacggatagaggaatacataaagttgaacataataaaataagaaattgaaagatttcgttgaaattgttcgtttggaaatttcccgaaaagctaattataggtt
cttctctccatcggaacaatagggccgttatgcttattactaaacttgttgaagagatgaaatagaaccaaggtctatctttttgatcagaggttaaatcgatcatcagaa
gaagaattaggccaaaaattaggatacattctgggaaaatgaaacttccctggaagagaagcaaatgaaacgctttcataaaaattctcgtagaatcgagaatgaag
ttttcattctgtacatgccagatcatgaattagtaactgcatccaatctccgaaaagtcccgattgtttcgatttttggaatgggatatttacggaatccccatgaataggat
caaaccttattccatgctatttccataagattcctctttcttattcttaagcaagcccccgagagggcttagttgatcatgatttctgttttctctttcttttcctttttgtttgtttcg
agaaagatatcgtccgattctccttctattgattcttttccgatcgagatgtatggatccatgtgtctacatacctagattctgttcatggattaacgaaaatgtgcaagagc
tctatttgcctctgccattctatgagtcgcttccttttgcgtatggcacccccacccccttggcagcatctactaattcggaacttaatttgaaagccatatttcgacccg
gacgcttttgggatgcttctaataaccaacgaatggcaagtgctcttccttgtttagatcctatttcaatcggaactttccgcgtcgatcctttttattacgtcttgtttttact
cctatattgggagttactctacgtattgcttgacgtaaaaccaatagtggatttgtttctgtcttttgttgaatctttttcacggctcgatagagaatttgataagccaatgatt
tttttccgtctttcataatacggttaaccaccatgttaactaatcgattacgaaaaattggatcggattttgcagttctttttctgcagtacctcgacgtgacatgagcgtga
aagaggttcaagaatccgttttctttttataagggctaaaaacgaatcacttatttttttggctttttgaccccatattgtagggtggatctcgaaagataggaaagatctcc

ctccaagccgtacatacaactttcatcgaatacggctttccacagaattctatagggatctatgagatcgagtatggaattcagtttactcacttaaaattgagtatccgtt
tccctccttttcccgctaggatcggaaatcctgtattttccatatccataccatcaagtccttaggtttccgaaatagtgtaatggaaaaagaagtgcttcgaatcattgct
atttgactcggacctgttctgaaaaagtcgaggtatttcgaattgtttgttgacacggacaaagtaagggaaaacccctgaaagaatttccatattgaccttggacatat
aagagttccgaatcgaatctctttagaaagaagatcttttgtctcatggtagcctgctccagtccccttacgaaactttcgttattgggttagccatacacttcacatgtttc
tagcgattcacatggcatcatcaaatgatacaagtcttggataagaatctacaacgcactagaacgcccttgttgacgattctttactgcgacagcatctagggctcct
cgaataatgcgatatctcacaccgggtaaatccttaacccttcctcctcttactaatactacagaatgttcttgtaaattatggccaataccaggtatataagcagtgattt
caaatccagaggttaatcgtactctggcaactttacgtaaggcagagttgggtttttttggggttgatagtggaaaagtcgacagataagtcacccttcctgtccctctac
agaaccgtacatgagattttcacctcatacggctcctcgttcaattctttcgaagggatccttttcctcgttcgagagtctccgcccttcttccactccgtcccgaagact
aactaagaccaattgagtcacgttttcatgttctaattgaacactttccatttatgattaaaggagaagattgttcttttaccaaacatatgcggatcaaatcacgtcttataa
taagaagaaatctttctcggtatcaatccccttgcccctcattctttgagaatcagaaggatccttttcgagtttccatttcttcatttggaatctgggctcttctatcttcgac
ttattttttttttgctttattctttatttatttcatttcgatttttccctcttcctctatccctatcctctaggtacagcgtttgcatcaatagagaaccttttcctctgtatgaatctatat
tattccattccaatttcttcccgaaacttcccaagaaaaatcccgaattggatccaaaattgacgggttaatgtgagcttatccatgcggttaggcactcttcaaatagga
atccattttctaactggctttcgtgctttggtgagtcgtccgagatcctttcgatgacctatgttgtgttgaagggatatctatatgatccgatcgattgcataagacccgc
ggtagcaatagaacggggaaagtatacagaaaagacagttcttttcaatttcgattatctatatattagttcgtttctatttctagatatctatttctatatattagtattagtta
gtagtactattctattagttagcgatcccggctctgtgagttctttcttccgtgatgaactgtcggcaccagtcctccattttttctctgtggaccgaggagaaagggggc
tcagcaggaagaggattgtaccatgagagaagcacagaggtcaaccctcttcaaatatggaacatggattctggcaatgcaacgtagttgggtcctcatatcgatcc
gaatgaatcagtctttctacagaggtcaatctttgcctattaggcaagaggatagcaagttcgaaattctgtctcggtaggacatggatttctattactatgaaattcataa
atgaaaatgaagtagttaatgggagggctaccattatcctttttcttgtatgtgttcctaagagaaggaatttgtccatttcatgtttcgaggtctcaaaaaagggcgtgg
aaacagatagaaactctggaatggaaattgaaaagaaatgtagccccagttccttcggaaatggtaagatctttggcgcaagaagaaggggcgacccatatcatct
tgacttggttctgcttcccctcttttttaagaataccgagtcgggttcttctcctaccagtatcgaatagaacatgctgaacaagatcttcttcatggaaacctgctcgattt
agatcgggaaaatcgtacagattttatgaaaccatgtgctatggctcgaatccatagtcaatcctattttcgataggaccggttgacaattgaatccaatttttcccattat
ttgactatccataatagtgtggaaagaaagcccggaggaagggtggccttgagtttctcgcccctttgccttaggattcgttaattctctttctcgaggggacggggaa
gggatataactcagcggtagagtgtcaccttgacgtggtggaagtcatcagttcgagcctgattatccctaaacccaatgtgagttttttctattttgacttactcccccc
gccacgatcgaacgggaatggataagaggcttgtgggattgacgtgatagggtagggttggctatactgctggtggcgaactccaggctaataatctgaagcgca
tggatacaagttatccttggaaggaaagacaattccgaatccgctttgtctacgaataaggaagctataagtaatgcaactatgaatctcatggagagttcgatcctgg
ctcaggatgaacgctggcggcatgcttaacacatgcaagtcgaacgggaagtggtgtttccagtggcgaacgggtgagtaacgcgtaagaacctgcccttggga
ggggaacaacaactggaaacggttgctaataccccgtaggctgaggagcaaaaggagaaatccgcccaaggaggggctcgcgtctgattagctagttggtgag
gcaatagcttaccaaggcgatgatcagtagctggtccgagaggatgatcagccacactgggactgagacacggcccagactcctacgggaggcagcagtggg
gaattttccgcaatgggcgaaagcctgacggagcaatgccgcgtggaggtggaaggcccacgggtcgtcaacttcttttctcggagaagaaacaatgacggtatc
tgaggaataagcatcggctaactctgtgccagcagccgcggtaagacagaggatgcaagcgttatccggaatgattgggcgtaaagcgtctgtaggtggcttttca
agtccgccgtcaaatcccagggctcaaccctggacaggcggtggaaactaccaagctggagtacggtaggggcagagggaatttccggtggagcggtgaaat
gcattgagatcggaaagaacaccaacggcgaaagcactctgctgggccgacactgacactgagagacgaaagctaggggagcaaatgggattagagacccca
gtagtcctagccgtaaacgatggatactaggtgctgtgcgactcgacccgtgcagtgctgtagctaacgcgttaagtatcccgcctggggagtacgttcgcaagaa
tgaaactcaaaggaattgacgggggcccgcacaagcggtggagcatgtggtttaattcgatgcaaagcgaagaaccttaccagggcttgacatgccgcgaatcct
cttgaaagagaggggtgccctcgggaacgcggacacaggtggtgcatggctgtcgtcagctcgtgccgtaaggtgttgggttaagtctcgcaacgagcgcaacc
ctcgtgtttagttgccactatgagtttggaaccctgaacagaccgccggtgttaagccggaggaaggagaggatgaggccaagtcatcatgccccttatgccctgg
gcgacacacgtgctacaatgggcgggacaaagggtcgcgatctcgcgagggtgagctaactccaaaaacccgtcctcagttcggattgcaggctgcaactcgcc
tgcatgaagcaggaatcgctagtaatcgccggtcagccatacggcggtgaatccgttcccgggccttgtacacaccgcccgtcacactataggagctggccatgtt
tgaagtcattacccttaaccgtaaggagggggatgcctaaggctaggcttgcgactggagtgaagtcgtaacaaggtagccgtactggaaggtgcggctggatca
cctccttttcagggagagctaatgcttatgcttattgggtattttggtttgacactgcttcacgcccaaaaagaaggcagctacgtctgagctaaacttggatatggaag
tcttctttcgtttagggtgaagtaagaccaagctcatgagcttattatcctaggtcggaacaaattagttgatagtgataggatccccttttttgacgtccccatgcccccc
cccgtgtggtgtggcggcatggggatgtcaaaaggaaagggatggagtttttctcgcttttggcgtagcaggcctcccaaagggaggcccgcgcgacgggctatt
agctcagtggtagagcgcgcccctgataattgcgtcgttgtgcctgggctgtgagggctctcagccacatggatagttcaatgtgctcatcagcgcctgacccgaa
gatgtggatcatccaaggcacattagcatggcgtactcctcctgtttgaatcggagtttgaaaccaaacaaacttctcctcaggaggatagatggggcgattcaggtg
agatcccatgtagatctaactttctattcactcgtgggatccgggcggtccgggggggcactacggctcctctcttctcgagaatccatacatcccttatcagtgtat
ggagagctatctctcgagcacaggttgaggttcgtcctcaatgggaaaatggagcacctaacaacgcatcttcacagaccaagaactacgagatcacccctttcatt
ctggggtgacggagggatcgtaccattcgagcctttttttcatgcttttcccggcggtctggagaaagcagcaatcaataggacttccctaatcctcccttcctgaaag
gaagaacgtgaaattctttttcctttccgcagggaccaggaggttggatctagccataagaggaatgcttggtataaataagccacttcttggtcttcgactccctaagt
cactacgagcgccctcgatcagtgcaatgggatgtggctatttatctatctcttgactcgaaatgggagcagagcaggtttgaaaaaggatcttagagtgtctagggt
tgggccaggagggtctcttaacgccttccttttttctgcccatcggagttatttcccaaggacttgccatggtaagggggagaaggggaagaagcacacttgaagag
cgcagtacaacggagagttgtatgctgcgttcgggaaggatgaatcgctcccgaaaaggagtctattgattctctcccaattggttggatcgtaggggcgatgattta
cttcacgggcgaggtctctggttcaagtccaggatggcccagctgcgccagggaaaagaatagaagaagcatctgactctttcatgcatactccacttggctcggg
gggatatagctcagttggtagagctccgctcttgcaattgggtcgttgcgattacgggttggctgtctaattgtccaggcggtaatggtagtatcttgtacctgaaccg
gtggctcactttttctaagtaatggggaagaggactgaaacatgccactgaaagactctactgagacaaaaagatgggctgtcaaaaaggtagaggaggtaggat
gggcagttggtcagatctagtatggatcgtacatggacgatagttggagtcggcggctctcctaggcttccctcatctgggatccctggggaagaggatcaagttgg
cccttgcgaatagcttgatgcactatctcccttcaaccctttgagcgaaatgtggcaaaaggaaggaaaatccatggaccgaccccattatctccaccccgtaggaa

ctacgagatcaccccaaggacgccttcggcgtccaggggtcacggaccgaccatagaccctgttcaataagtggaacacattagccgtccgctctccggttgggc
agtaagggtcggagaagggcaatcactcgttcttaaaaccagcattcttaagtttaagatcaaagagtcgggcggaaaaaggggagagctccccgttcctggttct
cctgtagctggattccccggaaccacaagaatccttagaatgggattccaactcagcaccttttgttttgagattttgagaagagttgctctttggagagcacagtacg
atgaaagttgtaagctgtgttcgggggggagttattgtctatcgttggcctctatggtagaacccgtcggggaggcctgagaggcggtggtttaccctgtggcggat
gtcagcggttcgagtccgcttatctccagcccgtgaacttagcggatactatgatagcaccgaattttgccaattcggcagttcgatctatgatttcgcattcatggacg
ttgataagatccttccatttagtagcaccttaggatggcatagccttaacgttaatggcgaggttcaaaagaggaaaggcttgcggtggatacctaggtacccagaga
cgaggaagggcgtagcaagcgacgaaatgcttcggggagttgaaaataagcatagatccggagattcccaaataggtcaacctttgaactgcctgctgaatccat
gagcaggcaagagacaacctggcgaactgaaacatcttagtagccagaggaaaagaaagcaaaagcgattcccgtagtagcggcgagcgaaatgggagcag
cctaaaccgtgaaaacggggttgtgggagagcaatacaagcgttgtgctgctaggcgaagcggttgagtgccgcaccctagatggctaaagtccagtagccgaa
agcatcactagcttacgctctgacccgagtagcatggggcacgtggaatcccgtgtgaatcagcaaggaccaccttgcaaggctaaatactcctgggtgaccgat
agcgaagtagtaccgtgagggaaaggtgaaaagaaccccccagtgggtagtgaaatagaacgtgaaaccgtgctgagctcccaagcagtgggaggggaaagtg
atctctgaccgcgtgcctgttgaagaatgagccggcgactcataggcagtggcttggttaagggaacggaacccaccggagccgtagcgaaagcgagtcttcat
agggcgattgtcactgcttatggacccgaacctgggtgatctatccatgaccaggatgaagcttggatgaaactaagcagaggtccgaaccgactgatgttgaaga
atcagcggatgagttgtggttaggggtgaaatgccactcgaacccagagctagctggttctccccgaaatgcgttgaggcgcagcagttgactggacatctaggg
gtaaagcactgtttcggtgcgggctgcgcgagcggtaccaaatcgaggcaaactctgaatactagatatgacccaaaaataacaggggtcaaggtcggccagtg
agacgatgggggataagcttcatcgtcgagagggaaacagcccggatcaccagctaaggcccctaaatgaccgctcagtgataaaggaggtgggggtgcaaa
gacagccaggaggtttgcctagaagcagccaccctttaaagagtgcgtaatagctcactgatcgagcgcccttgcgctgaagatgaacggggctaagcgatctgc
cgaagctgtgggatgtcaaaatgcatcggtaggggagcgttccgccttagagggaagcaaccgcgaaagcgggggtcgacgaagcggaagcgagaatgtcg
gcttgagtaacgaaaacattggtgagaatccaatgccccgaaaacccaaggtttcctccgcaaggttcgtccacggagggtgagtcagggcctaagatcaggcc
gaaaggcgtagtcgatggacaacaggtcaatattcctgtactaccccttgttggtacggagggacggaggaggctaggttagccgaaagatggttataggtttaag
gacacaaggtgaccctgctttttcagggtaagaaggggtagagaaaatgcctcgagccgaggtccgagtaccaagcgctgcagcgctgaagtatgagccccgtg
gactagccattgcttctccacgaggctcataccaggcgctacggcgctgaagtatgtaacccatgccatactcccaggaaaagctcgaacgaccttcaacaaagg
ggtacctgtacccgaaaccgacacaggtgggtaggtagagaatacctaggggcgcgagacaactctctctaaggaactcggcaaaatagccccgtaacttcggg
agaaggggtgccccctcgcaaagggggtcgcagtgaccaggcccgggcgactgtttaccaaaaacacaggtctccgcaaagtcgtaagaccatgtatgggg
gctgacgcctgcccagtgccggaaggtcaaggaagttggtgaactgatgacagggaagccggcgaccgaagccccggtgaacggcggccgtaactataacg
gtcctaaggtagcgaaattccttgtcgggtaagttccgacccgcacgaaaggcgtaacgatctgggcactgtctcggagagagactcggtgaaatagacatgtctg
tgaagatgcggactacctgcacctggacagaaagaccctatgaagctttactgttccctgggattggctttgggcctttcctgcgcagcttaggtggaaggcgaaga
aggccccccttccggggggggcccgagccatcagtgagataccactctggaagagctcggattctaaccttgtgtcagacccgcgggccaagggacagtctcaggt
agacagtttctatggggcgtaggcctcccaaaaggtaacggaggcgtgcaaaggtttcctcgggccagacggacattggtcctcgagtgcaaaggcagaaggg
agcttgactgcaagactcacccgtcgagcagagacgaaagtcggccttagtgatccgacggtgccgagtggaagggccgtcgctcaacggataaaagttactct
agggataacaggctgatcttccccaagagtccacatcgacgggaaggtttggcacctcgatgtcggctcttcgccacctggagctgtaggtggttccaagggttgg
gctgttcgcccattaatgcggtacgtgagctgggttcagaacgtcgtgagacagttcggtccatatccggtgtgggcgttagagcattgagaggacctttccctagta
cgagaggaccgggaaggacgcacctctggtgtaccagttatcgtgcctacggtaaacgctgggtagccaagtgcggagaggataactgctgaaagcatataagt
agtaagcccaccccaagatgagtgctctctcctccgacttccctagagcctccggtagcacagccgagacagcgacgggttctccacccatacggggatggagc
gacagaagcatggaaataggataaggtagcggcgagacgagccgtttaaataggtgtcaagtggaagtgcagtgatgtatgcagctgaggcatcctaacgaacg
aacgatttgaaccttgttcctacacgacctgatcaaatcgatcaggcacttgccatctatcttcattgttcaactctttgatgaaaagatgaaaaaaccaaaaaaaagctc
tgcccttccatctcttggatagatagagagggagggcagaggcctttggtgtcccttccagtcaagaattggggcttcacaattactagccaatatttctctcatgccttt
cctcgttcatggttcgatattctggtgtcctaggcgtagaggaaccacaccaatccatcccgaatttggtggttaaactctactgcggtgacgatactgtaggggaggt
cctgcggcaaaatagctcgatgccagaatgataaaaagcttaacacctcttatttgactttttcactattttgaaatacgaaaaagatccaaatccaaaatgcaaaggtc
gtcttattcaaaacctcaatcatcacatcccctctctcccacttcacacctcggaacgcactgttcttatagagagaaaggggctttcccatcttcttaacccgaaatgaa
atggctgaggagagggaggttccttttgggggggtacccccgggaagagatccagtggagacggggtgggcctgtagctcagaggattagagcacgtggctacg
aaccacggtgtcgggggttcgaatccctcctcgcccacagccttccaaaggggaagggcctttactttcccccctgagggtaggaaaaccatgatcgggatagcgg
acgtaaagctattgaacttgggtatgctctttccttttgtcgaagtggaatcgtagaacagaatgtgatacgatgagataaaatgcaatagaaacaaggatagcgaac
gggttacctactcctaagggtcaaagcaagccctttaattcaattctttattcttacattaaagaatgaatcaaatctccccaagtaggattcgaacctacgaccagtcag
ttaacagccgaccgctctaccactgagctactgaggaacaagggggattcgacctcctagagttcaactcccgctctcaacccatgaacaatatgagtccgaagctt
ctttcgtaactcccggaatttcttcgtagtggctccgttccatgcctcatttcataggtaagcccagagtggctctatttcattctatttcacttcctagcacttcctatcattt
aatatccatccctttggtcttattgacataagagatgtcatttatagtctatctctttctatatatggaaagtcaagaaattctcatcgaaacatcgagaaattgtgcatatag
aaaactctaaagaaagaaaaaaaggagacccatgccatgattttcaaatcttttctacttagtagtctaagtttctcgatgaggataattaattcggtcgttgcggtcgga
ctctattatgggtttctgaccacattctccatgggtccctcttagatcttctttctccaatcttggattagggaagaaggagatattcgcgactcctggtggtttcattatgg
ggcagctcatgatcttcatatcgatctattatccacctctgcatctattctttcttagctaaacgggtggaagatccatccaatttggttatatcatggactcaaaaaacgg
atctgaatgtgactgaaatgcacgatcttcacaggtatcacttttcacgatacctaaaaggtggaatagcgattttcgaaccatttcctataagagaaaggtttccattac
tttgagaaatggattctatatcaaactatagctattgcattaaagaagaaaagaaactaatagaagtcgaagacgcggaatggtagtgaatagagagaaagattcttct
ggttttcttgttcctgaaaatattctatctatctcctagacgccgtagagaattgagaattttcatgtctttcaattctcgtactcgtaattggaaagttacggaaggagatcc
atcattttgcaatgaaaactacataaaaaactctggacaatttcgaaatcaggccaagcgtcttaatacatatgcaaaaaaattcattattggcccaccattgattagaag
atttaacttgtatgaatcgctattggtttgatacgaataatggcagttgtttcagtatgttaaggatacagatgtatccacaattcatttagagttacttaatagcctatttctta
taccatatctctatcccgtgaaattctcgagccgaaagatggatgcatatgctatgtttcattttgctaaatgatatcaattaaacggtgtatcaattccataaattggatat

agcaataaataaatcagcaaaattcttttattttagatagaagaaaagtttcttctatctaaaataaaagaatgtacccttctatccaaatccaatttgcatcgataaaataa
atccaaattccagtagtagatgaataattgcaaatttttgtgtgtacgagattagaataacttcaaaataactgacataattttttatttttcctgatcagaaaaatacatgaa
aaagaaaggaggtagaaaaatttttggatttatggttaaagaagaaaaagaagaaaactggggttctgttgaatttcaagtattcagtttcaccaataagatacggaga
cttgcttcacatttggaattacacaaaaaagatttttcatcggaaagaggtctccgaagacttttgggaaaacgtcaacgtttgctggcttatttggcaaagaaaaatag
agtacgttataagaaattaatcagtcagttggatattagggagcggtaatttcatcgttcgaattttttttcttattttattagtagtcttatagtagtcttagattttgcattttgat
gagcctcgttttgaggaattcatggaataatccattttcatggaataatgaattaaggaagaaaggatatgagtctaccgcttacaagaaaagatctcatgatagtcaat
atgggccctcagcacccatcaatgcatggtgttcttcgactgatcgttactctcgatggtgaagatgttattgattgtgaacccatattaggctatttacacagaggaatg
gaaaaaatcggcaaataggaaagctacttaggcaggagataggggaattccttaagaaagaaaaaagaataagaacacagatacataacataaaaaaaagaata
aataagacgaaattcgacctcccccctacatatttaatttcttctcctatacaaaaactagcaagacctactccattggtaattccatcaatgacacccttgtcgaaaaact
gcgttagttcggttaatcctcttatacccaaggtaaaggtcctagtatagaaaatatctatataaccgcgattatatgaccaactatatatctttttttttagttgatggaaaa
aatactttttcggacccccttttacaaaggaatttattaaatccaaattctgaaaaaaagagtaagcagatccataaaacatatatgctatgaatagaccaaaaatagcta
gacttacagaagaaattgcattagtgataaattcatatgaatttatggaagaattagaactttcttggaaaaagttgattgagggagttagccactttgataatatggttaa
ttcccctatttcattatcaaaatggattcctatagatccaataaacaaagtacaaagcagtaatataagaagaggaaatagcatagtatttcccggttcatgaggataga
caaaagtgtttttagtccccaatgaagtactaaaggaccctatcctatttcctgtattaacatgaattttggatagattttgtgaaaaaaaagaaactccactcttcgctgtt
gataaaacgaaatctctattgactcctttagatatcctttttccccataacgatattgaatacaacgaatcctctttagtactactgtaattttgaaaatgaacacgcaaatac
ccatcaaaagtaagtaaatatatccgaaacatataaaacgcagttaatcctgcagtaaaagaagctattattccaaaaaagggtgaatataaccaactattactaagga
tttcatctttggaccagaagcaagcaagaggtggaataccacaaagagaaagggtaccccataaaaaacaagttcttgtaattggaatgtattttcttaaaccacccat
aagaaccatattctgacttttatctggtgaatatccaacaagaggttccattgaatgaataacagatccggatcccaagaataataaagctttcgaataagcatgagtg
atcaaatggaataaagcagcttgataagaacctatacctagagctaacatcatataacccaattgagacattgtagaataggctaagcttcttttaatatctctctgagca
agagctaaagtggctcctaagaaaagtgttagtgtacctattaaagaaatgaaactcattatcaaaggtagggatatgaaaagaggaagaagtcgagctataagaa
aaatccccgcagcaaccatagttgctgcgtgtataagagctgaaatgggggtgggtccttccatagcatcgggtaaccatacgtgaagagggaattgtgccgatttc
gcaactgcaccaaggaataataaaaaagcacacaaaatagtaagcaaggagttaatctcattattaggaatccagttattagctattttaaacaaatcccgaaactcta
aactacctgttatccaaaaaaaacctagaattcctaataacagaccaaaatcccctacacgattagttacaaaagctttttgacaagcactcgctgcaattggccgtgta
aaccaaaagcctatcaataaataggaacacattcccacaagctcccaaaaaaaataaatttgtatcaaattggaactagtaaccaatcccaacatggaagtattgaaa
aaacttatataaacaaaaaatctcaaatatccctcatcgtgagacatataatcatcactataaataagaaccaggattcctacagtagtaattagtattaacataatagaa
gtaagcgggtcgattaagtatccaaattctaaggaaaaatcattattgacggtccaagaccatagatattgatagatagaacttccatttatttgttgaatagacaggtga
actgagaataccatagctatacttaaaagtaaaacactaggaaaagcccatatgcgacgaagatttttttgttgctgttggaacaagaaaaagtccaaaccccattgac
ataataactggaagtgggagaagagggattacccatgcatattgatatgtatgttccataagaaaagaaattgcaatttttacttgaaaattttacttcaatttttctataaa
attgaaaaaagtttccgattcaccaaactaattcttatctatttctgaaggaataaaaaaatactagaattcttaatttttcaaaaattttctcattgaaacaatcaaaaaataa
gaataggttttgttggttaaagtcaaaaagttaatgaaataacttcgttacctagttattacctaaagaaggacttttattaaaatacaaaaaaaagattgaatcattttactt
taatattttttgtattaaaatgaagcagctcccttgtttcgtaactcaaattgattggaattcaattctggaatactttaattaactatttgattgaattttcccttccttttatcctc
ccgtcttatatgggggataggcccccataccttatatctgtatatggagagtatacttgaaatataaattgatttaaatagaaaacctttgtatatattctatattattaaaac
aaagtctaaaaaaaatatagaatatgttaaaaaactcttgtcttatccgcattagacaaaatgaagtaaaaaagaattcagaatttcaatatcttttagtatctaagtataaa
tactaagaaaaagaagaaagatggattgatttgcggcaatagatgtctttcacatacaactagaaaaaagtaatttcctttttgaatggcagttccaaaaaaacgtactt
cgatgtcaaaaaagcgtattcgtaaaaatctttggaagaaaaagacttatttttccatagtacaatcttattctttagcaaaatcaagatcattttctggcgtcagcgagca
tccaaaaccaaagggtttttctcggcaacaaacaaacaaataatagggttttgggataatatgaattgacctatccccaaaaaattccaattatttaatatgaataattag
gaataattaggattaattaatgagtttacttttatgtgtcgaattcctcggtacaatattcttagaacaaacctctctgatatataaaaaaagggtttttggtatactgtgacct
aaatattcttttcctatcaatgaacttttcgtaatagaatccgtataataaataaaaaaaggggattctattatgaaaagtagagtattcctgcaataagacttacaacttcta
cctatcttatcctaaattaacaaaaaaattagttctatattgcaactgagaaaaaattgttccaactctttcaaagtttctattgggcaaagcaagaattttttttgttaaaaaat
tcgcaacgatactaccaaacgaagtctattttaatgaagattctaatgtcctaaattctatggaatcttccaatctcgacgattcgcgagaaaataacttaatattcttttaat
aaacctattatttcaacttagccgccatggtgaaattggtagacacgctgctcttaggaagcagtgctcaagcatctcggttcgagtccgagtggcggcagtctcgaa
aaagaatacaatagattataaaataaaatggattcaattcgaaatttccaattttgtaatgggaccttctccttatgctatttgcaactttagaacatatactaactcatatttc
tttctcaacaatttcaattgtgattacgattcatttaataaccttattagttcgtgaacttgggggattgcgtgattcgtcagaaaaaggaatgatagccactttttttctgtata
acaggattcttagtttctcgttgggcttcttcgggacattttccattaagtaatttatatgagtcattgatcttcctttcatgggctctgtatattcttcatatgattcctaagata
cagaactctaaaaatgatttaagcacaataactacgccgagtactattttaacgcaaggctttgccacgtcgggtcttttaactgaaatgcatcaatccacaatactagt
acctgctctacaatctcagtggttaatgatgcatgtcagtatgatgttactaagctatgcgactcttttgtgcggatccttattatccgccgctcttctaatgattagatttcg
aaagaatttagatttcttttcgaaaaagaagaaaaatgttttgcttaaaacatttttctttaatgagattgaatatttctatgcaaaaagaagtgctttaaaaagcacctttttttc
ctttatttccaaattattacaaatatcaattaattgagcgtttggattcttggagttctcgtgtcattagtctagggtttacccttttaaccataggtattctttgtggagcagtat
gggctaatgaggcgtggggatcctactggaattgggatcctaaagaaacttgggcatttattacttggaccatatttgcaatttatttacatagtagaacaaatccaaatt
ggaagggtacgaagtcagcatttgtagcttccataggatttcttataatttggatctgttattttggtatcaatctattaggaataggtttacatagttatggttcatttacatta
cccatctaaatgattacataacataaaaccttaatgaaatggaaaaaacttccatttttgtgtttgatttgagaacccccttgaacgccttctcaaagggttctcaaaaattc
gagatagatctaattagactcttttactttttttctgaatttttttagtatttccactatggaatatagagcggactagtagaagaaaaaaaatcctatttaggataataattggat
aacagagcctctaccctgtcaacggatagcgagagaacaaaatctggataaataccgattcctattactggtaaaaagatacagattaaaagaaagagttctcgcgg
gccggaatcctcaaaatttgcgtttggaacatgaaatagcttgtatccatagaacatctgtcgtaacatagataataaataaataggagttaatatcattccaattgccatt
acaaaagtaattagcatttttggcattaacagaaattttggactagtaatgagtccaaaaaatactactaattccgcaacaaaaccactcattcctggtaaggcaagaga
agccattgaaaagctactaaacatggtaaaaatttttggcattgggatagaaacccctcccagttcttcgagataaacaaggcgcattctatcacaagccgttcccgct

aagaaaaaaagtgtagccccaataaatccatgggataatatttgtaaaatagctccattgagtccaatgttggttatggaaccaattcctataataatgaaacccatgtg
agagacggaggagtaggctattcttttttgaaattgcgttggccaagagaagttgaagctgcatagattatttgcatcgctcctattattactaaccaaggggaaaata
gataatgagcatgaggtaacaattccatattgatccgaatcaatccgtatgctcccatctttaataggattcccgctaaaagcatacatgtactgtaatgcgcttccccat
gggtatctggtaaccacgtatgtaggggtataatcggcaatttgacagcataagcaataaggaagccaaaataaaatagtatttccaatgttgcagggtatgattgatt
aattaatctttccaaatctaatcttggttcgttggaaccgtataagcccatacctagaactccgattaagaaaaaaatggaaccacctgcagtatacaaaataaactttgt
agctgaatagagacgcctcttccccccccacatggataaaagtaagtaaacaggaattaattctaactcccacatgataaaaaaaagtaaaaggtctcgcgaagaa
aataatcctatttgaccgctatacattgctagcatcaggaaatagaataatcgggaattccgggtaaccggccaagctgctaaagtagctaaagtagtcataaatcct
gtcaataaaatagatcctaatgaaagtccatcgattcccaatctccagtggaaattgaagacatctatccatttagaatcctcttttaattggattaagggatcctccaatt
ggaaatgataacagaatgcataagtcattagaaggaattctaataaacaaatagacatagtataccacctaacgattttgtttcccctatgaggtaaaaagaaaattaat
gaacccgcaaatatcggcaaaacaacaagtattgttaaccaaggaaaagaactcatgataaagtgataaagacaagatacgttttgaccagaaaagcccgtgctcg
attattttgagcacaggcttcttcggtaaagaggaatcagacgattcaagtggaatttttgtaacgtatcaataagatagagccatgctgcgggttgtctcaggtccta
aataaacgcggacacttaaaaaatctgttgggcaggcggattcgcatctcttacaacccacacaatcttcggttcttggcgcggaagcaatttgcttggctttacatcc
atcccaaggtatcatttctaatacatctgttggacaagctcgtacacattgagtgcatcctatacatgtatcataaatttttacggaatgtgacattggatctataaattttcc
ttttcaacataaaaattttcgatctggtcaaaatgaaattagtactatatcaatcaaatgtattgtagacaccagacgaagcaatggtttatccaaacttcaacaaataatg
caatatatttcttaatccgtttgtgagaaagcatgaaaagagccaagagacttgaattttgggcttcaacaatcataattatacgaattgtatatacgaattcgaattagcc
aataaattggctatcgtcttttcaatataaattattgcaatattcaaattgcaatatcaatgaattgcaaaaattcaactaagtaaaaaagaatactatggaataacctactc
aaaaaatagatattctcaaataataaatagtattcatgttaatatttcatattattattatatgtgtccctttgtttagaagattctatgtctaattattcaaaaaattagattgattg
atacgagttgatttcctattacgatggatggaagaaagaatggataatccaatagctgcttcagcagccgcaagggctataacaaaaattgcgaaaatgtctcctttta
attggcggctatcaaatagatcagaaaatgttacgagatttagattaattgaattcagtataagttcaaggcatattagagctctaaccatgtttcggcttgtgatcaatcc
atagataccaatcgaaaataaatagacactcaaaaaaagtacatgctcaaacatcattaactaactccttatcaatctcgattcatttcaatatggggacaagaattgaa
ccgattgaattaattagaatagaacaattacacaacaaaagagaaaagaaggtatttgttggcagtagatgggttttactaaatcaaaattgtggttctttagtgatttattt
tagatttgaaattcttataattttgactcattctaagtatttcttattgccgagccatagtaattgcacctattaaagaaactagaagaattatggaaatgagttcaaatggaa
gataaaaaatcggttgctaaatgaatcccaatttgttgaacgttatttatgagaccctgttctactatttggtttgatcttgtagtccaaagaattccataccatgacgtatctg
ggatagtagtcattagtgaaaaaggaatagttatacaaacgagtgaagtgaacccatctccaatagtccaaaaattcttatctttagaccattctgagccatttacgaac
attacggcaaatatgatcaagacatttatagctcccacataaataagaagttgtgccacagctacaaagtaggaattcaataaaatatagaataaggatatacaaacaa
gaactaatcctagcgaaaaagcagaaaaagttgggttggtaagtaataccacccctagaccccctagtagaagaacaaatcccccaaatagcacaagaatttcatg
tattggcccaggtaaatccattatggataagaagaaattaatagtataaatttttcatgaactgactaaaactaaaagattcaaggaagaaaaaagggattaggaaatt
tttttgtatattgtatataagttctttctatagttagaaatcacatcacgaaaatctactctggtttaaaatcaggaataatttgcaataagcagtaggtattcgtttttctttcta
gttagtaaagaacttttggattctaacaaaaaaaattctagtaatcagtaatcgttcttgaattccaagatttttcttcgtctattttactttgagttgaattcctaattgtttgaatt
gtgtaatctcccattatggagattggtaaccgactcaaagcaatttgattgtaattcaattcatgacgatcataagtagaaagttcatattcttcagtcattgataaacagtt
tgtcggacagtactcaacacaattaccacaaaatatacaaactccgaaatcaatactataattaagcaattgtttccttttaatatccttttcaaatctccaatccacaaga
ggtagatctatagggcatacgcgaacacatacttcacaagcaatacatttatcaaattcaaagtggattcgcccccggaaacgctccgatgtaattgatttttcatagg
ggtagtgaatcgttataggtaaacgatttgtgtgggataaggtaattatgaaactttgaccaatgtaccttgctgcgcgtattgtttgttgaccataactcatgaacccagt
taccatagggaacatattcgaaatatctatgaaaaaggtatgtttctttctcttgtttgagagaacttttgtgttgaaaatattcttactgttattgtattatcttattttatagtga
aacaagttgggaagaagttgttaataagagattgcccagggaaataggtaaaagaaatttccatccaagatttaataactgatccattctcatcctgggtaaagtccat
cttattgtgatagaaatgaagagaaataaataagctttagttaatgtaataaagatactcattgtcatttctagaattccaaccattttattcatttggaaaaatccaaaaaa
ggatatatagggaatagacaaattccacccgcctaagtagagaactgttacaaataaagaggaaactaataaatttaggtaagaaacaagataaaataaaccatattt
gataccagaatattcggtttgataacctgctactaattcttcctccgcttctggtaaatcaaagggtaatctttcacattccgccaaagaagaaattagaaaaaccagaa
aacctataggctgacgccaaagattccatccaaaaaaaccatattttgactgtgcttcaactatatcaactgtacttgaactgttggataatcatagtcgacgataacatc
acagttcccaccgctattccaaaaccgtacatgaaaccttagtttcatacggctcctctatgatcagaaaaaaggaaagtactgtttcatttcgttattatcttcttgggcg
tagttagaattatctaagataaaatcgatttcaacgtcctaaattagaccaaaggaattctgtctgctagaataataaaaaacgcttcggaattcatctcatcctttataata
taatggtactttttctttgttcagcaataacttaatcttggaataaaacactcgttataacaattaataaacgaaaagagttgggtattagttcatgaagaattctgtatgaat
atggataaacgacggaaagaataaataaagatcttttttttgtattgcattccatatcttttgtcctattcttctttccccgaggggtatttaaaaagaaaaaaggaataaa
gggttaattcgttcttgatagccatttccttaacaagtgaatgggaacatactctggatcggaatccgaagaaagtactacttgctcatttccaccaatttcaagtccttat
tatgattccttttatgaggaaaaatatctaatgctttagattccctcattactaatcctttatgtactttagtgtttctaatccctcactaacttttgatggattcccttatgattaca
actttctgtatcgggaatcccttattattgcccgcttcaagatatgatgactaatcaaaaaatctcaaccttggggtaaagaatttacaccgcttatgtttacttccatttttttc
ttgtacataggaaatgagatttttttcttttttactacaaattaataagcagttttgtttcactcatatagctatctagtttaacttactaacctgaatatagaataagaaaaggag
gataaatattcaatgaatttcagaggaaaaagatcctattttaacgaatcgcacgtagagatattgctagtacacaaaaagttaatgggatttcataactaatagattgag
cggcagctcgtagaccacctgaaaaagaatatttattatttgagctatatcctgccataagaagaccaataggagcaatacttgaaatggcaatccataaaaaaaacac
caatactaagatccgctaaaacaaagcgatatcccaaaggaataactaaaaaacttaataaaattgatatgactgctatagacggtccaatgctaaataagggaatat
cccctcgggatggcaagatatcctctttaaaaagtagcttagttccatctgctatagcttgaagcagtcccagggggccagcatattcaggaccaatacgttgttgtat
cgatgcggatatttctctttctaaccacacaattacgagtacttctattgtgattcccagtaagagggtcaaaatgggtagaatccatatcagtccatagacttcttttaata
attccaagttcgaaaaagaattgatagcttctacctgtaccctgtctattatcatttcaacgatcaacttctcccataatgatatctatactacctaatatcgtcatgatatca
gccaatttcatttttttgactagctgaggaagaatttgcaaattaataaaaccgggtggacgaattttccatctccaggggaaaagactatcatctcctaccagataaatt
cctaattcaccttttggggcttccactcttgcataaagctcttgttttgacaattcaaaattgggtgaaggttttttaccaagaaatcgatattcaaaatcattccattcggaa
ttctttgctttcttaaagcgtcggacttctaaattctcataagggcccccaggaatttttctacagcctgttgaataattttgattgattccctcatttcaccgattcgtactaa

atagcgtgctaatgaatccccttctttttgccattggactttccaatcgaattgattgtaagactcgtaaagatcaactttacgaagatcccattgtattccagaagctcgta
acatggggcccgataagccccaatttacagcttcttctccgctaataaaaccaactccctcaactcgttccaaaaaaatgggattctgtgtaataagttgttgatattcaa
taactcctcgtaaaaaataatcacagaaatctaaacatttatcgatccatccataaggcagatcggcagcaactcctccgatgcgaaagtaattatgcatcattcgcat
acctgtagcagcttcaaatagatcatatattaattctctctctctaaaaatataaaaaaaaggagtctgcgcaccgagatctgccataaaaggtccaagccataacaag
tgagaagctatacggctcaattctaacataattaccctaatatagctggctctttggggtatttgaatattctccaagaattctggtgcatttaccgttattgcttctgtaaac
atagtagctaaataatcccaacgtgttacataaggtaagtattgtataatagttcggttttccgcgatttttttccattcctctgtgtaaatagcctaatatgggttcacaatca
ataacatcttcaccatcgagagtaacgatcagtcgaagaacaccatgcattgatgggtgctgagggcccatattgactatcatgagatcttttcttgtaagcggtagac
tcatatcctttcttccttaattcattattccatgaaaatggattattccatgaattcctcaaaacgaggctcatcaaaatgcaaaatctaagactactataagactactaataa
aataagaaaaaaaattcgaacgatgaaattaccgctccctaatatccaactgactgattaatttcttataacgtactctatttttctttgccaaataagccagcaaacgttg
acgttttcccaaaagtcttcggagacctctttccgatgaaaaatctttttttgtgtaattccaaatgtgaagcaagtctccgtatcttattggtgaaactgaatacttgaaattc
aacagaaccccagttttcttctttttcttctttaaccataaatccaaaaatttttctacctcctttctttttcatgtatttttctgatcaggaaaaataaaaaaattatgtcagttatttt
gaagttattctaatctcgtacacacaaaaatttgcaattattcatctactactggaatttggatttattttatcgatgcaaattggatttggatagaagggtacattcttttatttt
agatagaagaaaagtttcttctatctaaaataaaagaattttgctgatttatttattgctatatccaatttatggaattgatacaccgtttaattgatatcatttagcaaaatgaa
acatagcatatgcatccatctttcggctcgagaatttcacgggatagagatatggtataagaaataggctattaagtaactctaaatgaattgtggatacatctgtatcct
taacatactgaaacaactgccattattcgtatcaaaccaatagcgattcatacaagttaaatcttctaatcaatggtgggccaataatgaattttttttgcatatgtattaaga
cgcttggcctgatttcgaaattgtccagagtttttttatgtagttttcattgcaaaatgatggatctccttccgtaactttccaattacgagtacgagaattgaaagacatgaa
aattctcaattctctacggcgtctaggagatagatagaatattttcaggaacaagaaaaccagaagaatctttctctctattcactaccattccgcgtcttcgacttctatta
gtttcttttcttctttaatgcaatagctatagtttgatatagaatccatttctcaaagtaatggaaacctttctcttataggaaatggttcgaaaatcgctattccaccttttaggt
atcgtgaaaagtgatacctgtgaagatcgtgcatttcagtcacattcagatccgtttttttgagtccatgatataaccaaattggatggatcttccacccgtttagctaaga
aagaatagatgcagaggtggataatagatcgatatgaagatcatgagctgccccataatgaaaccaccaggagtcgcgaatatctccttcttccctaatccaagattg
gagaaagaagatctaagagggacccatggagaatgtggtcagaaacccataatagagtccgaccgcaacgaccgaattaattatcctcatcgagaaacttagact
actaagtagaaaagatttgaaaatcatggcatgggtctcctttttttctttctttagagttttctatatgcacaatttctcgatgtttcgatgagaatttcttgactttccatatata
gaaagagatagactataaatgacatctcttatgtcaataagaccaaagggatggatattaaatgataggaagtgctaggaagtgaaatagaatgaaatagagccact
ctgggcttacctatgaaatgaggcatggaacggagccactacgaagaaattccgggagttacgaaagaagcttcggactcatattgttcatgggttgagagcggga
gttgaactctaggaggtcgaatccccccttgttcctcagtagctcagtggtagagcggtcggctgttaactgactggtcgtaggttcgaatcctacttggggagatttga
ttcattctttaatgtaagaataaagaattgaattaaagggcttgctttgacccttaggagtaggtaacccgttcgctatccttgtttctattgcattttatctcatcgtatcacat
tctgttctacgattccacttcgacaaaaggaaagagcatacccaagttcaatagctttacgtccgctatcccgatcatggttttcctaccctcagggggaaagtaaagg
cccttcccctttggaaggctgtgggcgaggagggattcgaacccccgacaccgtggttcgtagccacgtgctctaatcctctgagctacaggcccaccccgtctcc
actggatctcttcccgggggtacccccaaaaggaacctccctctcctcagccatttcatttcgggttaagaagatgggaaagcccctttctctctataagaacagtg
cgttccgaggtgtgaagtgggagagaggggatgtgatgattgaggttttgaataagacgacctttgcattttggatttggatctttttcgtatttcaaaatagtgaaaaag
tcaaataagaggtgttaagctttttatcattctggcatcgagctattttgccgcaggacctcccctacagtatcgtcaccgcagtagagtttaaccaccaaattcgggat
ggattggtgtggttcctctacgcctaggacaccagaatatcgaaccatgaacgaggaaaggcatgagagaaatattggctagtaattgtgaagccccaattcttgac
tggaagggacaccaaaggcctctgccctccctctctatctatccaagagatggaagggcagagcttttttttggtttttcatcttttcatcaaagagttgaacaatgaag
atagatggcaagtgcctgatcgatttgatcaggtcgtgtaggaacaaggttcaaatcgttcgttcgttaggatgcctcagctgcatacatcactgcacttccacttgac
acctatttaaacggctcgtctcgccgctaccttatcctatttccatgcttctgtcgctccatccccgtatgggtggagaacccgtcgctgtctcggctgtgctaccggag
gctctagggaagtcggaggagagagcactcatcttggggtgggcttactacttatatgctttcagcagttatcctctccgcacttggctacccagcgtttaccgtaggc
acgataactggtacaccagaggtgcgtccttcccggtcctctcgtactagggaaaggtcctctcaatgctctaacgcccacaccggatatggaccgaactgtctcac
gacgttctgaacccagctcacgtaccgcattaatgggcgaacagcccaacccttggaaccacctacagctccaggtggcgaagagccgacatcgaggtgccaaa
ccttcccgtcgatgtggactcttggggaagatcagcctgttatccctagagtaacttttatccgttgagcgacggcccttccactcggcaccgtcggatcactaaggc
cgactttcgtctctgctcgacgggtgagtcttgcagtcaagctcccttctgcctttgcactcgaggaccaatgtccgtctggcccgaggaaacctttgcacgcctccgt
taccttttgggaggcctacgccccatagaaactgtctacctgagactgtcccttggcccgcgggtctgacacaaggttagaatccgagctcttccagagtggtatctc
actgatggctcgggcccccccggaagggggccttcttcgccttccacctaagctgcgcaggaaaggcccaaagccaatcccagggaacagtaaagcttcatag
ggtctttctgtccaggtgcaggtagtccgcatcttcacagacatgtctatttcaccgagtctctctccgagacagtgcccagatcgttacgcctttcgtgcgggtcgga
acttacccgacaaggaatttcgctaccttaggaccgttatagttacggccgccgttcaccggggcttcggtcgccggcttccctgtcatcagttcaccaacttccttga
ccttccggcactgggcaggcgtcagccccatacatggtcttacgactttgcggagacctgtgtttttggtaaacagtcgcccgggcctggtcactgcgacccccttt
tgcgagggggcaccccttctcccgaagttacggggctattttgccgagttccttagagagagttgtctcgcgcccctaggtattctctacctacccacctgtgtcggtt
tcgggtacaggtacccctttgttgaaggtcgttcgagcttttcctgggagtatggcatgggttacatacttcagcgccgtagcgcctggtatgagcctcgtggagaag
caatggctagtccacggggctcatacttcagcgctgcagcgcttggtactcggacctcggctcgaggcattttctctacccccttcttaccctgaaaaagcagggtcac
cttgtgtccttaaacctataaccatctttcggctaacctagcctcctccgtccctccgtaccaacaaggggtagtacaggaatattgacctgttgtccatcgactacgcc
tttcggcctgatcttaggccctgactcaccctccgtggacgaaccttgcggaggaaaccttgggttttcggggcattggattctcaccaatgttttcgttactcaagccg
acattctcgcttccgcttcgtcgacccccgctttcgcggttgcttccctctaaggcggaacgctcccctaccgatgcattttgacatcccacagcttcggcagatcgctt
agccccgttcatcttcagcgcaagggcgctcgatcagtgagctattacgcactctttaaagggtggctgcttctaggcaaacctcctggctgtctttgcacccccacc
tcctttatcactgagcggtcatttaggggccttagctggtgatccgggctgtttccctctcgacgatgaagcttatcccccatcgtctcactggccgaccttgacccctg
ttatttttgggtcatatctagtattcagagtttgcctcgatttggtaccgctcgcgcagcccgcaccgaaacagtgctttacccctagatgtccagtcaactgctgcgcct
caacgcatttcggggagaaccagctagctctgggttcgagtggcatttcacccctaaccacaactcatccgctgattcttcaacatcagtcggttcggacctctgctta
gtttcatccaagcttcatcctggtcatggatagatcacccaggttcgggtccataagcagtgacaatcgccctatgaagactcgctttcgctacggctccggtgggttc

cgttcccttaaccaagccactgcctatgagtcgccggctcattcttcaacaggcacgcggtcagagatcactttcccctcccactgcttgggagctcagcacggtttc
acgttctatttcactacccactgggggttcttttcacctttccctcacggtactacttcgctatcggtcacccaggagtatttagccttgcaaggtggtccttgctgattcac
acgggattccacgtgccccatgctactcgggtcagagcgtaagctagtgatgctttcggctactggactttagccatctagggtgcggcactcaaccgcttcgccta
gcagcacaacgcttgtattgctctcccacaaccccgttttcacggtttaggctgctcccatttcgctcgccgctactacgggaatcgcttttgctttcttttcctctggcta
ctaagatgtttcagttcgccaggttgtctcttgcctgctcatggattcagcaggcagttcaaaaggttgacctatttgggaatctccggatctatgcttattttcaactccc
cgaagcatttcgtcgcttgctacgcccttcctcgtctctgggtacctaggtatccaccgcaagcctttcctcttttgaacctcgccattaacgttaaggctatgccatcct
aaggtgctactaaatggaaggatcttatcaacgtccatgaatgcgaaatcatagatcgaactgccgaattggcaaaattcggtgctatcatagtatccgctaagttcac
gggctggagataagcggactcgaaccgctgacatccgccacagggtaaaccaccgcctctcaggcctccccgacgggttctaccatagaggccaacgatagac
aataactcccccccgaacacagcttacaactttcatcgtactgtgctctccaaagagcaactcttctcaaaatctcaaaacaaaaggtgctgagttggaatcccattcta
aggattcttgtggttccggggaatccagctacaggagaaccaggaacggggagctctccccttttccgcccgactctttgatcttaaacttaagaatgctggttttaa
gaacgagtgattgcccttctccgacccttactgcccaaccggagagcggacggctaatgtgttccacttattgaacagggtctatggtcggtccgtgacccctggac
gccgaaggcgtccttggggtgatctcgtagttcctacggggtggagataatggggtcggtccatggattttccttccttttgccacatttcgctcaaagggttgaaggg
agatagtgcatcaagctattcgcaagggccaacttgatcctcttccccagggatcccagatgagggaagcctaggagagccgccgactccaactatcgtccatgta
cgatccatactagatctgaccaactgcccatcctacctcctctaccttttttgacagcccatctttttgtctcagtagagtctttcagtggcatgtttcagtcctcttccccatt
acttagaaaaagtgagccaccggttcaggtacaagatactaccattaccgcctggacaattagacagccaacccgtaatcgcaacgacccaattgcaagagcgga
gctctaccaactgagctatatcccccgagccaagtggagtatgcatgaaagagtcagatgcttcttctattcttttccctggcgcagctgggccatcctggacttgaa
ccagagacctcgcccgtgaagtaaatcatcgcccctacgatccaaccaattgggagagaatcaatagactccttttcgggagcgattcatccttcccgaacgcagc
atacaactctccgttgtactgcgctcttcaagtgtgcttcttccccttctcccccttaccatggcaagtccttgggaaataactccgatgggcagaaaaaggaaggcgt
taagagaccctcctggcccaaccctagacactctaagatcctttttcaaacctgctctgctcccatttcgagtcaagagatagataaatagccacatcccattgcactg
atcgagggcgctcgtagtgacttagggagtcgaagaccaagaagtggcttatttataccaagcattcctcttatggctagatccaacctcctggtccctgcggaaag
gaaaaagaatttcacgttcttcctttcaggaagggaggattagggaagtcctattgattgctgctttctccagaccgccgggaaaagcatgaaaaaaaggctcgaat
ggtacgatccctccgtcaccccagaatgaaaggggtgatctcgtagttcttggtctgtgaagatgcgttgttaggtgctccattttcccattgaggacgaacctcaacc
tgtgctcgagagatagctctccatacactgataagggatgtatggattctcgagaagagaggagccgtagtgcccccccggaccgcccggatcccacgagtga
atagaaagttagatctacatgggatctcacctgaatcgccccatctatcctcctgaggagaagtttgtttggtttcaaactccgattcaaacaggaggagtacgccatg
ctaatgtgccttggatgatccacatcttcgggtcaggcgctgatgagcacattgaactatccatgtggctgagagccctcacagcccaggcacaacgacgcaattat
caggggcgcgctctaccactgagctaatagcccgtcgcgcgggcctccctttgggaggcctgctacgccaaaagcgagaaaaactccatccctttccttttgacat
ccccatgccgccacaccacacgggggggggcatggggacgtcaaaaaggggatcctatcactatcaactaatttgttccgacctaggataataagctcatgagctt
ggtcttacttcaccctaaacgaaagaagacttccatatccaagtttagctcagacgtagctgccttctttttgggcgtgaagcagtgtcaaaccaaaatacccaataag
cataagcattagctctccctgaaaaggaggtgatccagccgcaccttccagtacggctaccttgttacgacttcactccagtcgcaagcctagccttaggcatcccc
ctccttacggttaagggtaatgacttcaaacatggccagctcctatagtgtgacgggcggtgtgtacaaggcccgggaacggattcaccgccgtatggctgaccgg
cgattactagcgattcctgcttcatgcaggcgagttgcagcctgcaatccgaactgaggacgggtttttggagttagctcaccctcgcgagatcgcgaccctttgtcc
cgcccattgtagcacgtgtgtcgcccagggcataaggggcatgatgacttggcctcatcctctccttcctccggcttaacaccggcggtctgttcagggttccaaact
catagtggcaactaaacacgagggttgcgctcgttgcgagacttaacccaacaccttacggcacgagctgacgacagccatgcaccacctgtgtccgcgttcccg
agggcacccctctctttcaagaggattcgcggcatgtcaagccctggtaaggttcttcgctttgcatcgaattaaaccacatgctccaccgcttgtgcgggcccccgt
caattcctttgagtttcattcttgcgaacgtactccccaggcgggatacttaacgcgttagctacagcactgcacgggtcgagtcgcacagcacctagtatccatcgtt
tacggctaggactactggggtctctaatcccatttgctcccctagctttcgtctctcagtgtcagtgtcggcccagcagagtgctttcgccgttggtgttctttccgatctc
aatgcatttcaccgctccaccggaaattccctctgcccctaccgtactccagcttggtagtttccaccgcctgtccagggttgagccctgggatttgacggcggactt
gaaaagccacctacagacgctttacgcccaatcattccggataacgcttgcatcctctgtcttaccgcggctgctggcacagagttagccgatgcttattcctcagat
accgtcattgtttcttctccgagaaaagaagttgacgacccgtgggccttccacctccacgcggcattgctccgtcaggctttcgcccattgcggaaaattccccact
gctgcctcccgtaggagtctgggccgtgtctcagtcccagtgtggctgatcatcctctcggaccagctactgatcatcgccttggtaagctattgcctcaccaactag
ctaatcagacgcgagcccctccttgggcggatttctccttttgctcctcagcctacggggtattagcaaccgtttccagttgttgttcccctcccaagggcaggttctta
cgcgttactcacccgttcgccactggaaacaccacttcccgttcgacttgcatgtgttaagcatgccgccagcgttcatcctgagccaggatcgaactctccatgaga
ttcatagttgcattacttatagcttccttattcgtagacaaagcggattcggaattgtctttccttccaaggataacttgtatccatgcgcttcagattattagcctggagttc
gccaccagcagtatagccaaccctaccctatcacgtcaatcccacaagcctcttatccattcccgttcgatcgtggcgggggggagtaagtcaaaatagaaaaaactc
acattgggtttagggataatcaggctcgaactgatgacttccaccacgtcaaggtgacactctaccgctgagttatatcccttccccgtcccctcgagaaagagaatt
aacgaatcctaaggcaaaggggcgagaaactcaaggccacccttcctccgggctttctttccacactattatggatagtcaaataatgggaaaaattggattcaattg
tcaaccggtcctatcgaaaataggattgactatggattcgagccatagcacatggtttcataaaatctgtacgattttcccgatctaaatcgagcaggtttccatgaaga
agatcttgttcagcatgttctattcgatactggtaggagaagaacccgactcggtattcttaaaaaaagaggggaagcagaaccaagtcaagatgatatgggtcgcc
ccttcttcttgcgccaaagatcttaccatttccgaaggaactggggctacatttcttttcaatttccattccagagtttctatctgtttccacgcccttttttgagacctcgaaa
catgaaatggacaaattccttctcttaggaacacatacaagaaaaaggataatggtagccctcccattaactacttcattttcatttatgaatttcatagtaatagaaatcc
atgtcctaccgagacagaatttcgaacttgctatcctcttgcctaataggcaaagattgacctctgtagaaagactgattcattcggatcgatatgaggacccaactac
gttgcattgccagaatccatgttccatatttgaagagggttgacctctgtgcttctctcatggtacaatcctcttcctgctgagcccccttttctcctcggtccacagagaa
aaaatggaggactggtgccgacagttcatcacggaagaaagaactcacagagccgggatcgctaactaatagaatagtactactaactaatactaatatatagaaat
agatatctagaaatagaaacgaactaatatatagataatcgaaattgaaaagaactgtcttttctgtatactttccccgttctattgctaccgcgggtcttatgcaatcgat
cggatcatatagatatcccttcaacacaacataggtcatcgaaaggatctcggacgactcaccaaagcacgaaagccagttagaaaatggattcctatttgaagagt
gcctaaccgcatggataagctcacattaacccgtcaattttggatccaattcgggatttttcttgggaagtttcgggaagaaattggaatggaataatatagattcatac

agaggaaaaggttctctattgatgcaaacgctgtacctagaggatagggatagaggaagagggaaaaatcgaaatgaaataaataaagaataaagcaaaaaaa
aataagtcgaagatagaagagcccagattccaaatgaagaaatggaaactcgaaaaggatccttctgattctcaaagaatgaggggcaaggggattgataccgag
aaagatttcttcttattataagacgtgatttgatccgcatatgtttggtaaaagaacaatcttctcctttaatcataaatggaaagtgttcaattagaacatgaaaacgtgac
tcaattggtcttagttagtcttcgggacggagtggaagaagggcggagactctcgaacgaggaaaaggatcccttcgaaagaattgaacgaggagccgtatgag
gtgaaaatctcatgtacggttctgtagagggacaggaagggtgacttatctgtcgacttttccactatcaaccccaaaaaacccaactctgccttacgtaaagttgcca
gagtacgattaacctctggatttgaaatcactgcttatatacctggtattggccataatttacaagaacattctgtagtattagtaagaggaggaagggttaaggatttac
ccggtgtgagatatcgcattattcgaggagccctagatgctgtcgcagtaaagaatcgtcaacaagggcgttctagtgcgttgtagattcttatccaagacttgtatcat
ttgatgatgccatgtgaatcgctagaaacatgtgaagtgtatggctaacccaataacgaaagtttcgtaaggggactggagcaggctaccatgagacaaaagatctt
ctttctaaagagattcgattcggaactcttatatgtccaaggtcaatatggaaattctttcaggggttttcccttactttgtccgtgtcaacaaacaattcgaaatacctcga
ctttttcagaacaggtccgagtcaaatagcaatgattcgaagcacttctttttccattacactatttcggaaacctaaggacttgatggtatggatatggaaaatacagga
tttccgatcctagcgggaaaaggagggaaacggatactcaattttaagtgagtaaactgaattccatactcgatctcatagatccctatagaattctgtggaaagccgt
attcgatgaaagttgtatgtacggcttggagggagatctttcctatctttcgagatccaccctacaatatggggtcaaaaagccaaaaaaataagtgattcgtttttagc
ccttataaaaagaaaacggattcttgaacctctttcacgctcatgtcacgtcgaggtactgcagaaaaaagaactgcaaaatccgatccaatttttcgtaatcgattagt
taacatggtggttaaccgtattatgaaagacggaaaaaaatcattggcttatcaaattctctatcgagccgtgaaaaagattcaacaaaagacagaaacaaatccact
attggttttacgtcaagcaatacgtagagtaactcccaatataggagtaaaaacaagacgtaataaaaaaggatcgacgcggaaagttccgattgaaataggatcta
aacaaggaagagcacttgccattcgttggttattagaagcatcccaaaagcgtccgggtcgaaatatggctttcaaattaagttccgaattagtagatgctgccaaag
ggggtgggggtgccatacgcaaaaaggaagcgactcatagaatggcagaggcaaatagagctcttgcacattttcgttaatccatgaacagaatctaggtatgtag
acacatggatccatacatctcgatcggaaaagaatcaatagaaggagaatcggacgatatctttctcgaaacaaacaaaaaggaaaagaaagagaaaacagaaat
catgatcaactaagccctctcgggggcttgcttaagaataagaaagaggaatcttatggaaatagcatggaataaggtttgatcctattcatggggattccgtaaatat
cccattccaaaaatcgaaacaatcgggacttttcggagattggatgcagttactaattcatgatctggcatgtacagaatgaaaacttcattctcgattctacgagaattt
ttatgaaagcgtttcatttgcttctcttccagggaagtttcattttcccagaatgtatcctaatttttggcctaattcttcttctgatgatcgatttaacctctgatcaaaaagata
gaccttggttctatttcatctcttcaacaagtttagtaataagcataacggccctattgttccgatggagagaagaacctataattagcttttcgggaaatttccaaacgaa
caatttcaacgaaatctttcaatttcttattttattatgttcaactttatgtattcctctatccgtagagtacattgaatgtacagaaatggctataacagagtttctgttattcgta
ttaacagctactctagggggaatgtttttatgtggtgctaacgatttaataactatctttgtagctccagaatgtttcagtttatgttcctacctattgtctggatataccaaga
gagatctacggtctaatgaggctactatgaaatatttactcatgggtggggcaagctcttctattctggttcatggtttctcttggctatatggttcatctgggggggagat
cgagcttcaagaaattgtgaacggtcttatcaatacacaaatgtataactccccaggaatttcaattgcgcttatatccatcactgtaggacttgggttcaagctttcccc
agccccttttcatcaatggactcctgacgtctacgaaggagtgtggttcgttcgacaaattcctacctctatatctatctctgaggtgtttgggttttgcaaaactccatag
atatgcagaagagaaatgctatccccactccgaccaagacagaacttttaccaaaagtttattgtgatctttttgttcaaataacaattaaggtgaagcagggtcagga
acaacgaatctctttatgataaacagatccattttgcaagctcgttattacgggtagttcctacaaagaatcggactaatgacgtatacaatgcttgaattatcgacgtag
atgctacatagtgggttctcatccttcagagactacgagtgtaataggagcatccgttgacaaaaggatcaccctaagatgatcatctcatggctattgggaacgaat
caaatcagatggttctatttctcaacctttctgacttgctcctacggaaccaaggtcgaaaggattgaaaaagtcagtcattcacaaccactgatgaaggattcctcga
aaagttaaggattagtagttctttttcgaaatcgatttcgaaaaagaatggattcggtcttatacatacgcgaggaaggtaatcaaaaaagaaagaagacaagttcttct
ttcttttatcacttaggagccgtgcgagatgaaagtctcatgcacggttttgcatgagagaaagaagcgaggaatcctcttttcgactctgactcccccactccagtcgt
tgcttttctttctgttacttcgaaagttgctgcttcagcttcagccacgcgaattctcgatattcctttttatttctcatcaaacgaatggcatcttcttctggaaatcctagctat
tcttagcatgatattggggaatctccttgctattactcaaacaagcatgaaacgtatgcttgcatattcgtccatagggcaaatcggatatgtaattattggaataattgtt
ggagactcaaatgatggatatgcaagcatgataacttatatgcttttctatatctccatgaatctaggaacttttgcttgcattgtattatttggtctacgtaccggaactgat
aacattcgagattatgcaggattatacacgaaagatcctttttggctctctctttagccctatgtctcttatccctaggaggccttcctccactagcaggtttcttcggaaa
actctatctattctggtgtggatggcaagcaggcctatatttcttggtttcaataggactccttacgagcgttctttctatctactattatctaaaaatagtcaagttattaatg
actggacgaaaccaagaaataaccccttatgtgcgaaattatagaagatcccctttaagatcaaacaattccatcgaattgagtatgactgtatgtgtgatagcatctac
tataccaggaatatcaatgaaccccattcttgcaattgctcaggataccctcttttagctgctaggtctatttcttagttcaagatccctcttactaactggaataaaagaat
tagtagatctgttccgcccaaaatgggaatgggcgctagggtaatgaacttataatcatggaatcgactcgatcatcagattataagttcattccataccggaccagac
cgtgcacattcttattatgagaaggggtcattcgagcctatggaaataggatactctgtttacatagaaatccccacgtccttacattctatttaggattaggaataggtgt
aatcagacctgcttttgacatatctatcctatccttatttgggtaccatatgcacctctttgggcttctattgaatcgagaaattggattgtacatctttttgattttgatacatat
aaggtgtcctacggataatgcaaatcgaagctatttgatgtctgactcaggcctatatgaccgatcgatcgaaatactccaagactccacctttgtcatatattccatata
tcacattagatagatatcatattcatggaatacgattcactttcaagatatcacattagatagatatcatattcatggaatatgattcactttcaagatgccttgatggtgaaa
tggtagacacgcgagactcaaaatctcgtgctaaagagcgtggaggttcgagtcctcttcaaggcataatacggagaatgcgcattcaatgagcattccccgtaga
agtattccggaaatctgcgcctggcgctctcctctatcttctgaggtccttaaccacttccctgagaaaaggagacagtaaaagccaaaatagactaaatatagcctg
aacgatcctaaaaatccctcgaaggagataatataataaagaacccaaagcagacggtatcctactgcaagggtggtcttaagaatccaaaagaggttgctcagaa
gagatagatgtatcccaacctctattgctctcgcgtaaagccttttttttacgcgacaggaaaaagtgactacgaattccccttttttgtttgcgaatccctgtttgtatccttt
gagcgcacgcccataagtagcgatcaaggaaatcgatcaaacgatcccaataccgtgaaagagaaacttcccagatccagggaagcttatcataaagggcttcga
caaggggttttattcgttctttgagcaaaaagataaagatcggatagattcgaaccgcaattgcaaaggtaatcactactatgccagcccaaatcatgatcttcaccaa
cttggatttggttctctcgcgaaaatcgcgagttgcagagatgagaaccatgaaaagcaagatcccgaataagaaaacagaaaccgaggaaaccacaagagtga
agactagtagagtctcgtcttttgtcattctttgctcctttttcactcaatgattcattcgaatttcccgaccaaaaattctatatgtctattcatagggcctcgttgctaagtgc
tacaagatctagtgcactggaactcgtggttatggacccgaatcctttagtatggaacattgtcttttccaagtaaaaacccccagtatatgaaagaatgaaaaggtgc
tttcgttcttttcttgtggaataagaagccctcgtaccttaatgaaaggaaaataggaatttttcattaggtatttgaccaaataggatcgtccagttcctatagaacctatc
actaaaatacccgatagggctaagcggaacgaaaaggattttccctgagatggtaaatgaaaacgattagccccatacgaggtttgggaataagtgatgagcaag

gaatatacgtctttctgctaaagaggatctattaaactcataattcattagatccttgttatcaatgtcaactaggtatcataagtaaacggatcccggttgttcaatcctttg
ataaccaaggtcattctttgctaaagagaaatgatcactatgagtcagactcaatagaattggatccattccaaatagcgagaattaggattctggatccctctcaatct
ctctttcaattcgaggatccagagaggtgttttcatagtcatctccgaatatttgccatctccgaatattttcgatttcatttttctatgatatgtctttctatatgaaaattggtta
tttacgatgtacgatgatccctgttaagcatccatggctgaatggttaaagcgcccaactcataattggtaaatttgcgggttcaattcctgctggatgcacgcgaacc
ggaacgttccataagtctattggaactggctctctatccatggaatctcatccatcatccatacataacgaattggtatggtatattcataccataacataagaacaataa
gaactcgaattcttatcgatactggaactcagagcataggagggaaagtcgatttatggatggaatcaaatacgcagtatttacagaaaaaagtcttcgtttattggga
aagaatcaatatacttttaatgtcgaatcgggattcactaagacagaaataaagcattgggtcgaactcttctttggtgttaaggtagtagctgtgaatagccatcgact
acccggaaagggtagaagaatgggccctattctaggacatacaatgcattacagacgtatgatcattacccttcaaccgggttattctattccacttctagatagagaa
aaaaactaaaggagaatacttaataatacggcgaaacatttatacaaaacacctatcccgagcacacgcaagggaaccatagataggcaagtgaaatccaatcca
cgaaataatttgatccatggacggcaccgttgtggtaaaggtcgtaattccagaggaatcattaccgcaaggcatagaggggggaggtcataagcgcctataccgta
aaatcgattttcgacggaatcaaaaagacatatctggtagaatcgtaaccatagaatacgaccctaatcgaaatgcgtacatttgtctcatacactatggggatggtga
gaagggatatattttacatcccagaggggctataattggagatactattgtttctggtacaaaagttcctatatcaatgggaaatgccctacctttgagtgcggtttgaac
tattgatttacgtaattggaagtaaccaattaggtttacgacgaaacctagaaatcgatcactgatccaatttgactacctctacgggatagacctcaacagaaaactgt
tgagtaacggcagcaagtgattgagttcagtagttcctcatagaaaattattgactctagagatatggtaatatggagaagacaaaattgtttgaagcacgcacagaa
ccggaagcgccccttgtttcaaagagaggaggacgggttattcacatttaatttgatggtcagaggcgaattgaaagctaagcagtggtaattaagacccccgggg
gaaaatagggatgtctcctacgttacccataatatgtagaagtatcgacgtaatttcatagagtcattcgatctgaatgctacatgaagaacataagccagatgacgga
acgcggagacctaggatgtagaagatcataacatgagcgattcggcagatttggattcctttcctatatatccactcatgtggtacttcatcatacgattcatataagatc
catctgtctagagatcgtcatatacatctagaaagccgtatgctttggaagaagcttgtacagtttgggaaggggtttttgagagaaaagaagaatctacttcaaccg
atatgcccttaggcacggccatacataacatagaaatcacacgtggaaggggtgggcaattagctagagcagcaggtgctgtagcgaaactgattgcaaaagaa
ggtaaatcggccactttaagattaccatctggggaggtccgtttggtatcccaaaactgcttagcaacagtcggacaagtgggtaatgttggggtgaaccaaaaaag
tttgggtagagccgggtctaagtgttggctaggtaaacgccccgtagtaagaggggtagttatgaaccctgtggaccacccccatgggggcggtgaagggaaag
cccccattggtagaaaaaaacccacaaccccttggggttatcctgcgcttggaagaagaactaggaaaaggaaaaaatatagtgatagttttattcttcgtcgccgta
agtaaatacgtaactaggaatatggaaaattgcatttttggaatttgcaataatgggatgggcgaacgacgggaattgaacccgcgcatggtggattcacaatccact
gccttgatccacttggctacatccgccccttatccagctaaaggattttctctttttccattcatcattattctatttattctgacctccatacttcgatcgagatattggacat
cgaatgccactctttaaaatggaaaaaaaggagtaatcagctgtgacacgaaaaaaaacgaatccttttgtagctcatcatttattggcaaaaatagaaaaggtcaata
tgaaggaggagaaagaaacaatagtaacgtggtcccgggcatctagcattctacccgcaatggttggccatacaatcgcgattcataatggaaaggaacatatacc
tatttacataacaaatcctatggtaggtcgcaaattgggggaattcgtaccaactcggcatttcacgagttatgaaagtgcaagaaaggatactaaatctcgtcgttaa
ctgaattcagaatagaaagattcaaaataaaaaaaaaagaaata
>O.nivara_IRGC88812_cp
cccaatatcttgcttcagcaagatattgggtatttctagctttcctttcttcaaaaattgctatatgttagcagaaaagccttatccattaagagatggaacttcaagagcag
ctaggtctagagggaagttgtgagcattacgttcgtgcattacttccataccaagattagcacggttgatgatatcagcccaagtattaataacgcgaccttggctatca
actacagattggttgaaattgaatccgtttagattgaaagccatagtactaatacctaaagcagtgaaccaaatccctactacaggccaagcagccaagaagaagtg
taaagaacgagagttgttaaaactagcatattggaagattaatcggccaaaataaccatgagcggccacaatattataagtctcttcctcttgaccaaatctgtaaccct
cattagcagattcgttttcagtggtttccctgatcaaactagaggttaccaaggaaccatgcatagcactgaatagggaaccgccgaatacaccagctacacctaac
atgtgaaatggatgcataaggatgttatgctctgcctggaatacaatcataaagttgaaagtaccagatattcctaaaggcataccatcagagaaacttccttgaccaa
tagggtaaatcaagaaaacagcagtagcagctgcaacaggagctgaatatgcaacagcaatccaaggacgcatacccagacggaaactcagttcccactcacga
cccatataacaagctacaccaagtaagaagtgtagaacaattagctcataaggaccaccattgtataaccactcatcaacagatgcagcttcccaaattgggtaaaa
gtgcaatccgatcgccgcagaagtaggaataatggcaccagagataatattgtttccgtaaagtaaagaaccagaaacaggctcacgaataccatcaatatctactg
gaggggcagcgatgaaggcgataataaatacagaagttgcggtcaataaggtagggatcatcaaaacaccgaaccatccgatgtaaagacggttttcggtgctag
ttatccagttgcagaagcgaccccacaggcttgtactttcgcgtctctctaaaattgcagtcatggtaagatcttggtttattcaaattgcaaggactcccaagcacacg
tattaactagaaagataatagaaggcttgttatttaacagtataatatagactatataccaatgtcaaccaagccagccccgacagttgtatatccatacaacaaaattta
ccaaaccaaaaaattttgtaaatgaagtgagtgaaaaatcaaaactcagattgctcctttctagtttccatatgggttgcccgggactcgaacccggaactagtcggat
ggagtagataattattccttgttacaatagagaaaaaacctctccccaaatcgtgcttgcatttttcattgcacacgactttccctatgtagaaataggctatttctattccg
aagaggaagtctactaatttttttagtagtaagttgattcacttactatttattatagtacagagaacatttcagaatggaaactgtgaaagttttaccttgatcatttatcaat
catttctagtttattagttttgtttaatgattaattaagaggattcaccagatcattgatacggagaatatccaaataccaaatacgctcactgtgcgatccacggaaagaa
aagtaagttgttttggcgaacatcaaagaaaaaacttgctcttcttccgtaaaaaattcttctaaaaataccgaacccaaccattgcataaaagctcgtaccgtgcttttat
gtttacgagctaaagttctagcgcatgaaagtcgaagtatatactttagtcgatacaaagtcttctttttgaagatccactgtgataatgaaaaagatttctacatatccg
accaaaccgatcaagaatatcccaatccgataaatcggtccaaattggtttactaataggatgccccgatccagtacaaaattgggcttttgctaaagatccaatgaga
ggagtaacagggactttggtatcgaatttttcatttgagtatctattagaaatgaattctccagcatttgattccttactaacaaagaatttattggtacacttgaaaagtac
cccagaaaatcgaagcaagagttttctaattggtttagatggatcctttgcggttgagtccaaaaagagaaagaatattgccacaaacggacaaggtaacatttccatt
tcttcttcaaaagaagagttccttttgatgcaagaattgcctttccttgatatcgaacataatgcataaggggatccataacgaaccatatggttttccgaaaaaaagcag
ggtacattaacccaaaatgttccatcttcctagaaaagatgattcgttccagaaaggttccggaagaagttaatcgcaagcaagaagattgtttacgaagaaacaaca
agaaaaattcatattctgatacataagagttatataggaaccgaaatagtcttttattttctttttcaaaataaaaatggatttcattgaagtaataaaactattccaattcga
gtagtagttgagaaagaatcgcaataaatgcaaggatggaacatcttggatccggtattgaaggagttgaagcaagatatccaaatggataggatagggtatttctat
atgtgctagataatgtaagtgcaaaaatttgtcttctaaaaaaggaaatattgaatgaatagatcgtaaattctgaaactttggtatttctttttcttccggacaagactgttc
tcgtagcgagaatgggatttctacaacgatcgcaaacccctcagatagaatctgagaataaaactcagaataaaaaaaattgttgtaatccaataatcgatcttggtta

ggatgattaaccaaattaatccaaaaattctgctgatacattcgaatcattaaccgtttcacaagtagtgaactaaatttcttgttattagaaccaataatttcgacaagttc
ggaaccatttaatccataatcatgggcaaacacataaatgtactcctgaaagagtagtgggtagacgaaatattgtctaggaaatttaagtttttctgaataaccctcga
atttttccatttgtatttctacttgaatcagagagagagaaatatttctcggtttatcaaatggtgatacatagtacaatatggtcagaacagggtgttgcatttttttaatacaa
acccctggggaagaaaaggagtctaatccacggatctttttccgctccttttctatccaatttgtttatgtttgttctaattacaaaagagaacaaatcctttatttttgcagg
ccaattgctcttttgactttgggatacagtctctttatcaatatactgcttcttttacacattcaatccataacatcctttttcaatccaaaatcaagaataattaggatttctaaa
aaaaaaagaaaaaatcaaaggtctactcataggaaaaccagcttttccctacatcaggcactaatctatttttaacgtctaattagatcagggagttcttccaattaagaa
gttaagctcgttgctttttgttttaccagaattggagccaggctctatccatttattcattagacccagaaaatcagaatttttttattccattccaaaaatccaaaataagaa
attgattttattacgacatgctatttttccattcattacccttgaggatcagtcgcggtcttatagactctaccaagagtctggacgaatttttttgcttcatccaaatgtgtaa
aagatcatagtcgcacttaaaagccgagtactctaccattgagttagcaacccagataaactaggatcttagatacgatcgaaatccaaaaatcaatggaattacacc
gcacacccctgtcaaaatcttaaaatagcaagacattaaaagaaagattttatcaccattgaaaacactcagataccaaaaggaacgggtctggttaaatttcactaag
gttaaaagtggcaccaatcacgatcgtaaaattgtcatttttttagcatttttatttaaaaaaataaataaatcttgtatgagagtacaaacaagagggacaaccctaccat
ttgagcaaagtgtaggcaaaaaacctaatagggagtgaggataaagagacttatccatctacaaattctagatgttcaatggacctttgtcaatggaaatacaatggta
agaaaaaaattagatagaaaaactcaaaaaaataaaggcttatgttggattggcacgacataaatccagtcaaaaataggattaagaaagaggcaaattatttctaaa
tagttagacaacaagggatactagtgagcctctcctagtttttttattcatttagttcttcaattaactcaaagttctttcttttttctttaaagaattccgccttccttaaaatatca
gaaactgttcttgtaggttgagcacctttttcaaggaaatagagaatagctggaacatttaaacaagtttgattctttatcggatcataaaaacctacttttcgaagatctct
tccttctcttcgagatcgaacatcaattgcaacgattcgatagacagcttattgggatagatgtagataaacaaagccccccctagaaacgtataggaggttttctcctc
atacggctcgagaatatgacttgcattaatttccgtacagaaaaaacaaatttcatttatactcatgactcaagttgactaattttgattgacagacttgaaagaaaaaaat
cctttgaaatttttttgagtcgtctctaaactcttttctttgcctcatctcgaacaaattcacttttattccttattccggtccaattctattgttgaggttgagacagttgaaaatc
gtgtttacttgttcgggaatcctttatctttgatttgtgaaatccttgggtttaaacattacttcgggaattcttattcttttttctttcaaaagagtagcaacatacccttttttctt
atttccttcgataaagcatttccctcttctatagaaatcgaatatgagcgattgattctgatagacttttaatcaaaagagttttcccatatcttccaaaattggactttcttctt
attttaaccttttgatttctatattatttcgatttctatattaagggtagaatgacaaagttggcctaatttattagttttcactaaccctagattctttcccttgataaaaaataaat
tctgtcctctcgagctccatcgtgtactatttacttagcttacttacaaacaacccagcgaaaattcggttcgggacgaatagaacagactatgtcgagccaagagcat
tttcattactatggaaaatggtggatagcaaaatccacaatcgatcgtgtccttcaagtcgcacgttgctttctaccacatcgttttaaacgaagttttaacataacattcct
ctaatttcattgcaaagtgttatagggaattgatccaatatggatggaatcatgaatagtcattagtttcgttttttgtatactaattcaaacttgctttgctatctatggagaa
atatgaataaaagaaattaagtatttatcgggaaagactccgcaaagagccaatttatttaaacccatattctatcatatgaatgaaatatagttcgaaaaaagggaata
aacaagtttgcttaagacttatttattatggaatttccatcctcaacagaggactcgagatgatcaatccaatcctgaaatgataagagaagaattgactcttctccaaca
aataaactatcaacctcccgtttaattaatttaattaatatattagattagcaatctatttttccataccatttttccgtaacaaaactaattaactattaactagttaaactattgc
aatgaaaagaaagtttttttggtagttatagaattctcgtatttcttcgactcgaataccaaaagaaagaaaaaaaatgaagtaaaaaaaacgcatttcctgtaaagtaaaat
taaggtctttgcttttacttatttttttcttttacctaaaagaagcaactccaaatcaaaattgaatccattctatctaacgagcagttcttatcttatctttaccgggatggatcat
ctggatatttaaaaaaatcgcggatcgagatcgttttcgcttaaccaaagaaagaaaaagaagaaggaacctttttttactaataaaatactataaaaaaaaatttatctctat
cataaatctatctctaccataaaggaataggtctcgttttttatacaatgttctacgtcaagtttaaaattttttcatgaaaaaaagattttcaatttgactggacttgacactg
gattatgttttctgagacagaaaatgaacgcattaggactgcatcgaatctaagagtttataagagaaaaaaattctctttaataaactttatgtctcgtgcagaatacaat
acgatttcatctttcgtttcatcataaaaaaatctgggacggaaggattcgaacctccgagtaacgggaccaaaacccgctgccttaccacttggccacgccccatttc
gggttttatgcgacactaataaacagtattatgtttatttcttattcgtcaatcctacttcaattacataaaaaatggggggtattctcttggtaggattctagacatgcgaata
atatagaatccaaaaaatgcattgatcattacatggaattctattaagatattatatgaaagtcgaatttcttccactctcatttgagagtgcgaatacaaggaggtattttg
tgtttgggaaagtccgaagaaaaaaggattttgaatcctcctttttccttttttcccttagaaaaataactcaatcaaaatccaattatctactctacaagaacgaaacgcttg
ttatgcctaatatacttagtttaacctgtatttgttttaattctgttatttatccgactagttttttttcttcgccaaattgcccgaagcttatgccattttcaacccaatcgtggatttt
atgcctgtcatacctgtactcttttttttctattagcctttgtttggcaagctgctgtaagttttcgatgaaatctttactactctgtctgccaaattgaatcatgtattcattctaaa
aaaattcgaaaaatggataagagccgagaagtcttatattatgaaccttcgattctaaaattcaaattcttctacattgaatgtatagctgcagcaataaatttggatcagc
ctttctactccctgcatctacgttgagcaggtatctttaggtaaccgcacaatacctaacctaatttattgataagagtgcttattataaatcaattcttgcaatttttttttcaaa
aattgatttttttgcatttttaggtgtcaaaataaacaaaacccatcctagtggatttgtgtggtaaggaaaaacgggtaatctattccttaaaaaaaaaatcttggagattatgt
aatgcttactctcaaactttttgtttatacagtagtgatattctttgtttccctctttatctttggattcttatctaatgatccaggacgtaatcctgggcgtgacgagtaaaaat
ccaaaatttttttcttacaaattggatttgtttcatacatttatctacgagaaaatccgggggtcagaattccttccaattcgaaagtcccaaacgatccgagggggcgga
aagagagggattcgaaccctcggtacaaaaaaattgtacaacggattagcaatccgccgctttagtccactcagccatctctccccgttccaaatcgaaaggtttcc
gtgatatgacagaggcaagaaataacgattgcaaaaaatccttcctttttctttcaaaagttcaaaaaaattatattgccaattccattttagttatattctttttttcttaatgtta
ataaaaaaaagaagaaaattcttctttttttctttctaattctaaaattggatattggctaaaagacaatcagatagattttctcttcagcaggcatttccatataggacttgtta
taataaaacaagcaggttatagaaaaaaactctttttttttattatttatcaacaaagcaaaaaggggtcttatcaaaccaacccaccccataaaattggaaagaaagata
aagtaagtggacctgactccttgaatgaggcctctatccgctattctgatatataaattcgatgtagatgaaattgtataagtggattttttttgtatttccttagacttagacc
acgcaaggcaagaatttctcgctatttactatttcatattcttgttactagatgttctataggaataagaagaaatcgcaacccctttccgctacacataaaaatggatttc
gaaagtcaatttttcttttcaatatctttacttttttttcatttttcccttaaaaaataggctttcttggaaataggaatcatggaataatcctgaattccaatgtttatttctatagtat
aagaaaaactaattgaatcaaattcatggatttaccacgacctcggctgtgaccccatagataaaaatgcaaaatttctatcttcgagaccattgaaaaaaggcattga
acgagaaaaaatcgtccacagataatctatcgtatgccttggaagtgatataaggtgctcggaaatggttgaagtaattgaataggaggatcactatgactatagccc
ttggtagagttactaaagaagaaaatgatttatttgatattatggacgactggttacgaagggaccgttttgtttttgtaggatggtctggcctattgctttttccttgtgctta
tttcgctttaggaggttggtttacagggacaacttttgtaacttcttggtatacccatggattggcgagttcctatttggaaggttgcaatttcttaaccgcagcagtttcca
cccctgccaatagtttagcacactctttgttgctactatggggcccggaagcacaaggggattttactcgttggtgtcaattaggtggtctgtggacttttgttgctctcc
atggggcttttgcactaataggtttcatgttacgtcaatttgaacttgctcggtctgttcaattgcggccttataatgcaatttcattctctggcccaatcgctgtttttgtttcc

gtattcctgatttatccactggggcaatccggttggttctttgcgccgagttttggcgtagcagcgatatttcgattcatcctcttcttccaaggatttcataattggacgtt
gaacccatttcatatgatgggagttgccggagtattaggcgcggctctgctatgcgctattcatggggcaaccgtggaaaacactctatttgaggacggtgatggtg
caaataccttccgcgcttttaacccaactcaagctgaagaaacttattcaatggtcaccgctaatcgcttttggtcccaaatctttggtgttgcttttccaataaacgttgg
ttacatttctttatgctatttgtaccggtcaccggtttatggatgagtgctattggcgtagtcggcctggctctgaacctacgtgcctatgacttcgtttcccaggaaatcc
gtgcagcggaagatcctgaatttgagactttctacaccaaaaatattcttttaaacgagggtattcgtgcgtggatggcagctcaggatcagcctcatgaaaatcttata
ttccctgaggaggttctaccacgtggaaacgctctttaatggaacttttgttttagctggtcgtgaccaagaaaccaccggttttgcttggtgggccgggaatgccaga
cttatcaatttgtcgggtaaactacttggagctcacgtagcccatgcaggattaatcgtattctgggccggagcaatgaacctatttgaagtggcccatttcgtaccag
aaaaacccatgtatgaacaagggttgattttacttccgcacttagctactctaggttggggagtagggcccgggggagaagttctagatacttttccgtactttgtatct
ggagtacttcatctaatttcctccgcagtcttaggcttcggtggcatttatcacgcgcttctgggaccggagactcttgaagaatcttttccattctttggttatgtgtggaa
agatagaaataaaatgactacaattttgggtattcacctaattttgttaggtataggtgcttttcttctagtactcaaagctctttattttggcggtatatatgatacctgggct
cctgggggaggagatgtaagaaaaattaccaatttgacccttagccccggtgttatatttggttatttactaaaatccccttttgggggagagggttggattgttagtgtg
gatgatttagaagatataattgggggacatgtatggttgggtttcatttgtgtatttggcggaatttggcatatcttaaccaaacccttcgcatgggctcgccgtgcatttg
tatggtctggagaagcttacttgtcttatagtttaggcgctttatctgtctttggttttatcgcttgttgttttgtctggttcaataatacagcttatccgagtgagttttatggac
ctactgggccagaagcttctcaagctcaagcatttacttttctagttagagatcagcgtcttggagctaatgtgggatctgcccaaggacccacaggtttaggtaaata
tctaatgcgttccccaacgggagaggttatttttggagggaaactatgcgtttttgggaccttcgtgctccatggttagaacctctaaggggccccaacggtttggac
ttgagtaggttgaaaaaagacatacaaccttggcaagaacgacgttcggcagaatatatgacccatgctcctttaggctctttaaattctgtgggtggcgtagctaccg
agatcaatgcagttaattatgtttctcctagaagttggttagcgacttcccatttgttctaggattcttcttttttgtgggccatttgtggcatgcaggaagagcccgggct
gctgcagcaggatttgaaaagggaatcgatcgtgatttggagcctgttctttacatgacccctcttaactaagattttcttatttatacctgttctactgttttttctgttctgg
ctcggttattctatctagccgagccattcattcctttctatgaaagaaagataaggggacagaacaaaaaaaacaaataaagaaacaaacgtattcaatacgcaaaag
aaaagagagaggaaagcaaaaggagagagagagggattcgaaccctcgatagttcctagaactataccggttttcaagaccggagctatcaaccactcagccatctc
tccacagcctaatccttattttattcctacaaatagaacatagccatacgaaatgactcactaacctctagaaacatctcaaatacaaatccctttcgatatatttctgtata
ctgtatccatgtatacaggatccgctatatccgcttgtgaaataaagcataaaacccccctcaaccccatatccaaataaaaaaagtggtgagtaataagttttaaagag
aagaatcaatggattcatgattaaacccctcctacttcttgtattttattacaattttgattaagtgagggatcaaatataatatgtagtcaactttatttgatggtaacttgga
ggattagaaatatgactattgctttccaattagctgtttttgcattaattgtgacttcctcagtcttagtcattagtgtacccctttgtatttgcttctcctgatggttggtcaaac
aataaaaacgttgtattttccggtacatcattatggattggactggtctttctggtagctattctaaattctctcatttcttaaatgtgtttagtatttagtagcccgatacaaaa
taaaaaagggccgtttattcggattgtgagacgcattaaaatgcaatttgcgccccgaattgattgacagacaattaaattaataaaaagaaaactcaaatagaaaatg
aaacggtcgacccagacatagacggtcgacccaggcggatataatatacccctataaaatataggacgtagcgagcgtagttcaatggtaaaacatctccttgccaa
ggagaagatacgggttcgattcccgccgctcgccagcttaatttagtaaggtactatgataaaaaaatttagtctacttttattaaagtaataggtgttagtctagtaccgta
ccccttttactatcttagccccctttgcaccccactcaaaaaaagagcactacagcggcgggaatcgaactcggcaacagggttccctaaaccggggattcaccgaa
acaaacaaccagcaaacagcttttaaagggaagggagatagactgtgcctttctttcatttatttttctttctgcaaggtagggggccttgagagttcctcttgtggtag
caagttacttcgcaacctgctcaatttggccttatagggtcgggaactaatgaataaaaagggttggataccgcccaaccacccagccctctaccatatctagacaaa
tagaatagtccttttatacagactgctaagtgcggagacgggaatcgaacccgtgacctcaaggttatgagcctcgtgagctaccaaactgctctactccgctctgga
gcgctggaaaccggtggacgaaaaaggttgaatacaggcctctaccatgtctagacaaatagaatagttattttatacagaatggagcgggtagcgggaatcgaac
ccgcatcgttagcttggaaggctaggggttatagtcgacgttggttgattattattaacgtctctaattcaaaaccgaacatgaaattttgatttcattcggctcctttatgg
gtattctcaccacttaacatctaagtcagctttttgtctgaatggaaccaaggctctctgctttctagatgatccctaaagagtaggagatagaaatttgcctaaatctatc
taatctaattacttcgttccctaatttcatttaagagatcctgaggaaaagaattgggtttccaccgagctgaaacaatatgctgatggttctagtaaaccaaaactaccg
tttttagctatttggcttccatttcctttttaacaaaagaagatttagttacgattggaaataaatttttttgtatcttcatccatagatcctttactctcatattttaaaaattggaat
ccttaatccaatgcaaaattatgcttcgcgactctgtactcataatccaaatcctatttgtttttggatgcaatttcaattagtctttgggtacaaatcgcgaaaatgcatatt
cttcctcaatatgctattgagagaaaaaggagtaaatccctttctaagaactaaagttttcatcggaatataaaaaaccttaaggacgccttaagtatatcatttcaaattca
gttattaatagaacgaatcacacttttaccactaaactatacccgctacatgtaaattctgataccaacgctaccctttgtcaagggtagccattcgagaaagatgctaat
tccctcttagttaatgaaacagagaaggttcctcacagttagcggttggtacttcgatcgcgggcctttcctttactttctttttgttcagaattgaacaaagaatttgggg
aagaaaacatcttcccccacttatcatgaaatctgggccatagagaaagagtgagatgtttttttttatttatcatagactttccctatggcttgagagaaacaataaataa
cttaaagaaaaaggcacataggagccgaaggatttacttgatgtaaagaagattctgaatgtctctgcttagtcgattctctccgtttaactttttcttctcttcttttccact
caattctagtttattagattcttgtttaaaagaatcaaagaagatgaatagaactaagaacacacaaaaaagagcatataggcccgagaccattaccaaaagttcttcc
caataatcatattgggtatctgttcccttcctttcctattaggatggggcatgttggattttccatatccatataccatcgaaccttaagggttccagaaccctcctttttttgc
tagttttcggaaacggaaaaccctgaaccaggagcagtataaattctactgcccgcctttttacaagaaaattggtgataaaactccactacagtttgttcaaaatgcac
caatcagaatcccttgagaatattcaagtaccctatttctaagatatttctaagagaagaggtacctgctgaaaatcgcttcaacaaatccgcttaagaaaaattgcaaa
gttttgaactcgaaggtttttccaagggatgcctgttaaaaattgtttcaacctctcaccaaaacagacaagaagtatatcactgaaaattaatacccagccatatgggt
atatgaagggcgcgaattcgtttataccccacccaattagaggaaataaaacataaatggagaaagttttcatgataagatcagccaatagaagaaaaaagtcccta
atttttcagaccgttctgagcatgtgaaaagtcaatagcctaaagataaaaaaccctatactttgtgcaagtgataagagagaatatgaaagattttcctatttttcttgatt
ttcttaagattttttgaacctgaccatgaatagacttatatatctcgatatatacagatataatgtacattatggagtagacctataatgggaaatgaaagtggctaattttg
gaattgaataagaagcccttttaactcagtggtagagtaatgccatggtaaggcataagtcatcggttcaaatccgataaagggcttttttacttagtggtagagtaat
gccgcggtaagacgtgagtcagtggttcaaatccgatacagtacttttctactaaattcattcactttcttcttttttgaaaatttctctttttttattgaatttgataacttagtgc
gagatgcatgcatttttagtttaaacactaagcgaagcaggggggtgtaaattccaaaaaaagaaattgtactctttttcctattagatcaatcaaatcactacccgtactg
aactaatatagaatcccttttattaatctattcttattccatatcctttataaacgaatttccctaaaaagtaggggatgatccgtgaattaacctaaccatcaactaaaaaaa
atcctacaaaagcataatggaaaagtaggaaggactccttgctttggatctagttatactcttcgagtatattgacaattccaaaaaactgctcatactatcattatagtat

aatgaggagcggttgtataccgccctatcgtctagtgatgcccctatcgtctagtggttcaggacatctctctttcaaggaggcagcggggattcgacttcccctggg
ggtagggagtattatgaaaggaggttaatcatagattatcaaaaaccctagaataaattcttcctgggtcgatgcccgagcggttaatggggacggactgtaaattcg
ttgacaatatgtctacgctggttcaaatccagctcggcccaaaaatctagggcttcgtgaatatgagttaaatccattttttttcttccataaaaaagaatatttgatccata
gaaataaaagaaataaaggataaaaagaaaaggggaaatatctttctaatctatatctctttcattcctctcttacaaacaaaaaaaagaccttttcttattggttattgaaa
ggtggattattatctatttttagcgataataaatcgcgacatactagttatgtcattctcactataccccccatacgatacgggggtatgtagtatatgattcgtctatttcttag
agtaggacaggcgaatattcttattcttatggttccatttaagaataagtatgccatataagaataagtatgccatacgccccgcggggattgtagttcaattggtcaga
gcaccgccctgtcaaggcggaagctgcgggttcgagccccgtcagtcccgaactagggttcaatgaatggagaaattcatctttccttttccatgaaaaaagggg
ggcagaaagcaagatcaaatacctatggggtacccctatttcacttttttttagttcgcgtttctcagtaaagagaggagagtataggaattttttttatcactacttctggttg
atagcgaaagacatacatatcatacgtggaagggatcctcctatgttatactattccactctcaaccatgaattgatttgatagatccgatattcataatattgaattgattc
agtattatcagaatgcaagtcctccccttgaatttacaggatacccttttccctctccatgggattacatcccgagttattgcgaaaaaaaaagaggttatggaagtcaata
ttctcgcatttattgctactgcattgttcattctagttcctactgcctttttacttattatttatgtaaaaacagtcagccaaaatgattaattggaagttcaattaatcattgaag
aaatgaaaaagggattaaataaaataaaaatccaagtcttaaatgaaaggatccggttggaatcataaagtgtggtagaaaaaactacatatagtttttttctaccacact
ttagagtctttctattatattatcttgaatctacatagaatagattactagattgaaatagtagtctaattcaatttcttttttcactgcatccacttaatttcaatcaagtcaaaat
gaaaaaatccatggagggagagaaaaataatatgagaatagactatagtaaaagaaaaaaagtaaaaggaaaaaaccagcgaatctttcatgcttaaacatgtcgc
gaaatgtttcaaaagagcataaaatttattttaagaactaagaataagaaaagagtataaaacaaatggaaaatgtgcgatatgttgggaatagctccgcggaagaaa
atctaaagttcttatgtatagaactttttttaaccatgggtcgcttctagtagcgattatgaattgctctcaccgctctttctatttctattttctattctttctatttctatatagtaga
atagaaatagaaagaatagaaagactctttcttacaagagtttcttacaggagtgaaacaaaattaaagaaagagagaaagaataaagttcggcaaaatgattaatg
caaaacggtcaattaaagaaaagagttgatacaacaattcgactactcaatcaattagtagtatccctagagtccactcctcccccatactactagtgaaagagaaaat
gtaaagactaccattaaagcagcccaagcgagacttactatatccatgtaaattatgtctcctatttctatgaaggaattattctactattgatgaataatcatagtagaatc
aagggtacagagtcaaaaaggggttctgacctaaaactatggatgaatcagttcaaagaatttactcttaacaaattcttagagtatttctggtagaatttaacaaattctt
agagtatttctggtagaattggggagcattaagtataaatatgatacatagccctttcttattaataaaagaataaggaaacgctatctcatccctattggtatcggtttgg
gccactactgctaaaacaaaccccagtttgaggaaagaacggtgggttctcaaaatccagtatcgccgagccttgttattctcttgccccaacttatgcggggtgcaa
atttgtcgatttggatcagtactataagcctaagtattttattgatcaggcggcacccagatttgaactggggataaaggatttgcagtcccctgccttaccgcttggcca
tgccgccaaaaaatacgatctaaaatcgagaaaagagcaagtattcatccacgtttcttactaaaactaactttcttttatcttaaatctaattctacttactttttttccaatctt
tttcaaaaaatctattcatgcttttttttggatccagtttcgattattctcctcaaaggattctatcttaaaacacacattgctaacactagaaaacttcccttttcttttctattgaa
atgaaaaaggagaaaagtggatttctagtcacaagctacaaaattaagaacaaattggaaccattaactagaattctcttttttatttgaatttcggtattctctcccgcct
gccatttaatggcataataaaagacaatggatttatgcctaatccgtatataagtaaactccaggtccgaacagcattattatctatggatccccccttatgtacatatctct
gtggagaatcgttctttaatttttcattgcattaaatatcttgaataaaaaaaaagaaaattgactttgctatgtggaggtccagaactagattggcatgtacttaaaaaagta
cttactttattttaggattctacaacgaaatcttatatcttatattttatagaaattctactactacgaaacaaaaaagaaccttcaaattctttttttgaaattaaactaagcgtg
ctattctcaatcgaactaaagtcaaactttctagtgcttataaattattatattttggttttatcccattcatagaaaggagaaaaaatgagaaatctttgccgtccaatctaa
gaatatcataaactgtaagtggcagaattttttttcgaggttctaggaatgttttatcacttcattttcattccatttggacccctgggaaattcgaactttcgttgaaattgtct
ctattcatatgtatgaaatacatatatgaaatacgtatgtggagttcccgagaatttcatgtgatttagtaaacagaatatagattccataattgctagatcgatctgtaggg
attgatgaagagtgagctgataatggaatttttcttcgataaacaggaaacttaagattaagatgctccggaatggaaatgagggaatgtccacaatacccggatttag
tcagatccaattcgagggattttgtaggttcattaatcaaggcttggcagaagaacttgagaagtttccaacaattaaagatccagatcacgaaatttcatttcaattattt
gcgaaaggatatcaattgctagaaccttcgataaaagaaagggatgctgtgtatgaatcactcacctattcttccgaattatacgtatccgcgcgattaatttttggtttc
gatgtgcaaaagcaaaccatttctattggaaacattcctataatgaattccttaggaacctttataataaatggaatataccgaattgtgatcaatcaaatattgctaagtc
ctggtatttactaccgctcggaattagaccataaaggaatttctatctacaccgggactataatatcagattggggaggaagatcggaattagcaattgataaaaaaga
aaggatatgggctcgcgtgagtagaaaacaaaagatatctattctagttctatcatcagctatgggttcaaatctaaaagaaattctagataatgtttcctaccctgaaat
tttcttgtctttccctaatgctaaggagaagaagaggattgagtcaaaagaaaaagctattttggagttttatcaacaatttgcttgtgtaggtggggacctggtattttcg
gaatccttatgtgaggaattacaaaagaaatttttttcaacaaaaatgtgaattaggaaggattggtcgacgaaatatgaatcgaagactgaatcttgatatacctcagaa
cagcaccttcttgttaccgcgagatgtattggccgctacggatcatttgattggaatgaaatttgaaacgggtatacttgacgatgacgatatgaatcacttgaaaaata
aacgtattcgttcggttgcggatctgttacaagatcaattcggactggctcttggtcgtttacaacatgcggttcaaaaaactatccgtagagtattcatacgtcaatcaa
aaccgactccacaaactttggtaactccaacttcaacctcgattttattaataactacttacgagaccttctttggtacatatcccttatctcaagtttttgatcaaaccaatc
cattgacacaaacggttcatgggcgaaaagtgagttgtttgggtcctggaggattgacggggagaactgcaagttttcggagccgagatatccatccgagtcactat
gggcgtatttgtccaattgacacgtccgaaggaatcaacgttggacttactggatctttagctattcatgcgagaattgatcattggtggggatccgtagagagtccatt
ttatgaaatatctgagaaagcaaagaaaaaaaaaagagagacaggtggtttatttatcaccaaatagagatgagtattatatgatagcagcaggaaattctttgtccttg
aatcggggtattcaggaagaacaggttgttccagctagataccgtcaagaattcctgactattgcatgggaacagattcatgttagaagtatttttcctttccaatattttt
ctattgggggttctctcattccttttattgagcataatgatgcgaatcgagctttaatgagttctaatatgcagcgccaagcagttccgctttctcggtccgagaagtgcat
tgttggaactggattggaacgccaaacagctctagattcgagggtttctgttatagccgaacgcgagggaaagatcatttctactaatagtcacaagatccttttatcaa
gtagtgggaagactataagtattcctttagttacccatcggcgctctaacaaaaatacttgtatgcaccaaaaacctcgggttccgcggggtaaatccattaaaaaag
gacaaattttagcggagggggctgctacggttggtggggaacttgctttaggaaaaaacgtattagtagcttatatgccatgggaaggttacaattttgaagacgcag
tattaattagcgaacgtttggtatatgaggatatttatacttcttttcacatccgaaaatatgaaattcagacggatacaacaagccaaggctccgctgaaaaaatcacta
aagaaataccacatctagaagaacatttactccgcaatttggacagaaatggagttgtgaagttggggtcctgggtagaaacaggcgatattttagtaggtaaattaa
cgcctcagatagcgagcgaatcgtcctatatcgcggaagctggattattacgggccatatttggtcttgaggtatccacttcaaaagaaacttctctcaaactaccgat
aggtggaagaggacgcgttatcgatgtgaaatggatccagagggatcccctcgacataatggttcgtgtatatattttacaaaaacgcgaaatcaaagttggggata
aagtagccggaagacacgggaataaggggatcatttccaaaattttgcctaggcaagatatgccctatttgcaagatggaacgcctgttgatatggttttcaatccctt

aggagtaccctcccgaatgaatgtgggacaaatatttgaaagctcgctcggattagcaggggatctgctaaagaaacattatagaatagcaccctttgatgagagat
atgagcaagaggcttcaagaaaacttgtgttttcagaattatatgaagccagtaaacaaacaaaaaatccgtgggtatttgaacccgagtacccgggaaaaagcag
aatatttgatggaagaacaggagaccccttcgaacagcctgttctaatagggaagtcctatatcttaaaattaattcatcaagttgatgagaaaatccacggacgctct
actgggccctattcacttgttacacaacaacccgttagaggaagagccaagcaaggggggacaacgaataggagaaatggaagtttgggctttagaaggatttggt
gttgctcatattttacaagagatacttacttataaatctgatcatcttatagctcgccaagaaatacttaacgctacgatctggggaaaacgagtacctaatcacgaggat
cctccagaatcttttcgagtgcttgttcgagaactacgatctttggctctagaactgaaccatttccttgtatctcagaagaacttccaggttaatagggaagaagtttgat
cggaataaatataaattcttttcttatttctattttatgattgacgaatataaacatcaacaacttcaaattggactcgtttcccctcaacaaataaaggcttgggctaacaaa
acactacctaatggggaagtcgttggcgaagtcacaaggccctccacttttcattataaaaccgataaaccagaaaaagatggattgttttgcgaaagaatctttgga
cccataaaaagcagaatttgtgcttgtggaaattctcgagcgagcggagctgaaaacgaagacgaaagattttgccaaaaatgcggagtagaatttgttgattctcg
gatacgaagatatcaaatgggatacatcaaactcgcatgtcccgtgactcatgtgtggtatttaaaaggtcttcctagttatatcgcgaatcttttagataaaccccttaa
gaaattggagggcctagtatatggcgatttctcttttgctaggcccagtgctaaaaaaccaactttcttacgattacgaggtttattcgaggatgaaatttcatcctgtaac
catagcatttccccctttttttctaccccaggctttacaacatttcgaaatcgggaaattgcgacaggagcaggtgctattagagaacaattagcagatttggatttgcga
attattttagagaattcctcggtcgaatggaaggaattagaagacgaggggtatagtggagatgaatgggaagatagaaaaagacgaataagaaaagtttttttgatt
agacgcatgcaattggcgaaacattttattcaaacaaatgtagaaccagaatggatggttttgtgcttattaccagttcttcctcccgaattaagacccattgtttataggt
ctggggataaagtagtgacttcggatattaatgagctttataagagagttatccgtcggaacaacaatcttgcctatctattaaaaagaagtgaattagcgccagcaga
tttagtaatgtgccaggaaaaattggtacaagaagccgtggatacacttcttgatagtgggtcccgcgggcaaccaacgagggatggtcacaataaagtatacaaat
cactttcagatgtaattgaaggtaaagaggggaggtttcgcgaaactctgcttgggaaacgggtcgattactcggggcgttctgtcattgttgtgggtccttcactttca
ttacatcaatgtgggttacctctagagatagcaataaagctttttcagctatttgtaattcgcgatttaatcacgaaacgtgctacttctaatgttaggattgctaaaaggaa
aatttgggaaaaggaacccattgtatgggaaatacttcaagaagttatgaggggacatcctgtactgttgaatagagcacctaccctgcatagattaggcatacagg
ctttccaacccactttagtagaggggcgtactatttgtttacacccattagtgtgtaaaggtttcaatgcagactttgatggggatcaaatggctgttcatctacctttatcc
ttggaagctcaggcggaagctcgtttacttatgttttctcatatgaatctcctatctcccgctattggggatcctatttgcgtaccaacccaagacatgcttatcggacttta
tgtattaacgattggaaaccgtcgaggtatttgtgcaaatagatataatagttgcggaaactatccaaaccaaaaagtaaattacaataataataatcctaagtatacga
aagataaagaatctctttttctagttcctatgatgcactgggagcttatagacagaaacaaatctgtttagacagtcccttgtggctccgatggaaactagatcaacgc
gtcattgggttaagagaagttccgattgaagttcaatatgaatctttggggacttatcgtgagatttatgcccactatctagtagtgggaaatagaaaaaaggaaatccg
ttctatatacattcgcaccactcttggtcatatttctttttatagagaaatagaggaagccatacaaggatttagtcaggcctattcatacactatctaaacaaggaagtta
gattcggggatgcccctttcgaggggcattccgatttcgctagtatcatcatttttgccgcacgaatccagattgagattgaggaaaggaagttaactaagttttcgaat
cactgactcaggcccattgtcgaatcctactcagcaattgtcgaattatactcagcaattgtcgaattatactcagccgaaaaaggggggtacttatttatggcggaacg
ggccaatctggtctttcagaataaagagatagatggaactgctatgaaacgacttattagcagattaatagatcatttcggaatgggatatacatcccatatactggatc
aaataaaaacgctgggcttccatcaagccactactacatcgatttcattaggaatcgaggatcttttaacaataccctctaagggatggttagtccaagatgcggaaca
acagagttttcttttggaaaaacactattattatggggctgtacacgcggtagaaaaattacgccaatccgttgaaatctggtatgctacaagtgaatatttgaaacacg
aaatgaattcgaattttcggataacagatccttctaatccagtctatctaatgtctttttcaggagctagaggaaatgcatcgcaggtacaccaattagtaggtatgcgag
gattaatggcggatcctcaaggacaaatgattgatttacctattcaaagcaatttacgcgagggactttctttgacagaatatataatttcctgctacggagcccgcaaa
ggggttgtagatactgctgtacgaacggcggatgctggatatcttacacgtagacttgttgaagtagttcaacatattattgtgcgtagaagagattgtggtactatcca
agctatttctgtgagtcctcaaaatgggatgacggaaaaactttttgtacaaacattaattggtcgtgtattagcaaacgatatatatatcggttcacggtgcattgccact
cgaaatcaagatattggaattgggttagtcaatcgattcataaccacctttcgagcacagccatttcgagcacaaccaatatatattagaaccccctttacttgccggag
cacatcttggatctgtcaattatgttatggtcggagttccactcatggcgatctggtcgaattgggggaagctgtaggtgttattgcgggtcaatctattggggagcca
gggactcaactaacattaagaacttttcatactggtggggtattcacagggggtactgccgaccttgtacgatccccttcaaatggaaaaatccaattcaatggggatt
tggttcaccccacacgtacccgtcatgggcagcctgcttttctatgttatatagacttgcatataactattcagagtcaggatattctacatagtgtgactattccttcaaaa
agcttgattctagtgcaaaatgatcaatatgtagaatccgaacaagtaattgcggagattcgtgccggaacgtccgctttgcattttaaagaaaaggtacaaaaacata
tttattccgaatcagatggggaaatgcactggagtaccgatgtttatcatgcgcccgaatatcaatatggtaatcttcgtcgattaccaaaaacaagccatttatggatat
tgtcagtaagtatgtgcagatctagtatagcttctttttcgctccacaaggatcaagatcaaatgaatacttattccttttctgttgacggaaggtatatctttggcctctcga
tggctgatgatgaggtaagacatagactgttggatacttttggtaaaaaagatagggaaattcttgattattcaacgccggatcgaatcatgtccaatggtcattggaat
tttgtctatccttctattcttcaaaataattttgatttgttagcgaaaaagcgaagaaataggttcgccattccattacagtatcatcaagaacaagagaaagaaccaatat
cctgttttgggatttcgattgaaataccctttatgggtgttttacgtagaaatactatagttgcttattttgacgacccacgatacaaaaaagataaaaagggttcaggaat
tgttaaatttagatataggaccctagaggacgaatataggactcgagagaaagactcagagaacgaatacgggagcccagaaaatgaatataggacccgagagg
aagaatgtaaaaccctagaagacgaatataggactcgagaggaggagtatgaaaccctagaagatgaatatgggatcccagagaacgaatatgaaaccctagaa
gatgaatatggaatcctagaggacgaatataggactcgagaggaggaatccgaggacgaatatgggagtccagagaacaaatataggccccgagaggataaat
atggcactttagaggaagactcagaggacgaacatgggactttagaggaagactcagaggaagactcagaggacgaatatgggaacccggaggaagattccgt
cttaaaaaagggggttttgattgagcatcgaggaacaaaagaatttagtctaaaataccaaaaagaagtagatcggttttttttcattctccaagaactgcatatcttgcc
gagatcctcatccctaaaggtacttgacaatagtattattggagtggatacacaactcacaaaaaatacaagaagtcggctgggtggattggttcgagtgaagagaa
aaaaaagccatacggaactaaaaatcttttctggagatattcattttcctgaagaggcggataagatattaggtggcagtttgataccactagaaagagaaaaaaaag
attcgaaggaatcaaaaaaaagggaaaattgggtctatgttcagtggaaaaaaattctcaagagcaaggaaaagtattttgttttggttcgacctgcagtcgcgtatga
aatgaacgaagggagaaatttagcaacacttttcccacaggatctcttgcaggaagagggtaatctccaacttcgacttgtcaattttatttctcatgaaaatagcaagt
taactcaaagaatttatcatacgaatagtcaattcgttcgaacttgcttagtattgaattgggaacaagaagaaaaagaggaggctcgtgcttcccttgttgagataaga
gcaaatggtctgattcgcgatttcctaagaattgggttaatcaaatccactatttcgtatacacgaaaaaggtatgatagcagaagtgcaggactgattctccataatag
gttagatcgcaccaataccaattccttttattccaaggcgaagattcaatcacttagccaacatcaagaagctattggtaccttgttgaatcgaaataaagaataccaat

ctttgatggttttgtcggcatccaactgttctcgaattggtttttttaagaattcaaaaaatcccaatggggtaaaagaatcgaatcctagaattcctattccaaaattttttg
ggctctttcgaaatttttcgggactcttaggtactattgcacctagtatatcgaatttttcttcatcttactatttactaacgtataatcagatcctgttaaaaaagcatttgctc
cttgacaatttgaaacaaaacttcaaagtacttcaaggacttaaacactctttaataaatgaaaatcaaaggacttcaaatttcgatagtaacatcatgttggatccattcc
agttaaattggcactttctccctcatgattcttgggaagagacatcagcaaaaattcaccttggacaatttatttgcgaaaatgtatgtctatttaaatcgcacataaaaaa
atcaggtcaaattttcattgtaaatatagattcctttgttataagagcagctaagccttatttggccactacaggagcaactgttcatggtcattatggagaaatcctttaca
aaggggataggttagttacgtttatatacgaaaaagcgagatctagtgacataacgcaaggtcttccaaaagtagaacaaatcttcgaagcgcgttcaattgattcact
atcgccgaatctcgaaaggagaattgaggattggaatgagcgtataccaagaattcttgggggtccttggggattcttgattggagctgagttaaccatagcccaaa
gtcgtatctctttggttaataagatccaaaaggtttatcgatcccaaggggtacagatccataatagacatatagagattattatacgccaagtaacatcaaaagtgcgg
gtttccgaagatggaatgtctaatgtttttttcacctggggaattaattggactattgcgagcagagcgagcagggcgggctttggatgaatcgatctattatcgggcaa
tcttattgggaataacaagggtttccctgaatacccaaagtttcatatctgaagcaagttttcaagaaactgctcgagttttagcaaaagctgccttacgaggtcgtattg
attggttgaaaggcctgaaagaaaacgtagttctgggggggattatacctgttggtaccggattccaaaaatttgtgcaccgttacccacaagacaagaacctttattt
cgaaattcaaaaaaaaaaaactatttgcgtcggaaatgagagatattttgtttctccatacagaattagtttcttctgattctgacgtaacaaacaatttctatgagacatca
gaatcaccatttaccccatttatatgatttaaggatacataaagcagatttttttactttaaactagatttttgaccttagaacgctaagaggttagattttctatttttattttaatt
taaaaagaagtttagttaattcattaaggttatgcttataccatgtagaaggttccatcggaacaattattatttatttcaagctatttcggctctttcttaatcttcgaaaaga
aagaaatttcgtaatggaaaggtaggatgaaaaaaaagaaaaaatcaaaaggaagtgtggaaaaaatgacaagaagatattggaacatcaatttgaaagagatga
tagaagcgggagttcattttggtcatggtattaagaaatggaatcctaaaatggccccttacatctcggcaaagcgtaaaggtactcatattacaaatctcgctagaac
cacccgtttttttatcagaagcttgtgatttagtttttgatgcagcaagtcagggaaaaagcttcttaattgttggtaccaaaaaaagagcagcggatttagtagcatcagc
tgcaataagggctcgttgtcattatgttaataaaaagtggttcagtggtatgttaacgaattggtcgattactaaaactagactttctcaatttagagacttaagagcagaa
gaaaagatggaaaaattccaccatctcccaaaaagagatgtggcaatcttgaagagaaaattatctaccttgcaaagatatctcggcgggatcaaatatatgacgag
gttgcctgacattgtgatcgtcctcgatcagcaaaaagagtatatagctcttcgggaatgtgccattttggggattcctactatttctttagccgatacaaattgtgaccca
gatctcgcgaatatatcgattccagccaacgatgacactatgacttcaattcgattgattcttaacaaattagtatttgcaatttgtgagggccgttctctctatataagaaa
tcattgattaagaagaatagttaattcttgggcaactgcgtagatttatggaatcacttactattctttttttgttttgcatagaaaaaagacggggaatattgatatatattag
agggtattgatatatattatcatctgatgtgatttcttgatatcctaaatataagattaatacttcacgttgctgagttgagaaaaagatggttgaatcaaaagaattcttttttt
gaagttcatttttatcaggggacaatatgaatattataccgtgttccattaaaacactcaaggggttatacgatatatcgggcgtagaagtaggccaacacttctattgg
caaataggaggtttccaaattcatgcccaagtacttatcacttcttgggtcgtaattactatcttgctaggttcagttatcatagctgttcgcaatccacaaaccatcccaa
ccgatggtcagaatttcttcgaatatgtccttgagtttattcgagacttaagcaaaactcagattggagaagaatacggtccctgggttccctttattggaactatgttcct
ttttatttttgtttcgaactggtcgggtgctcttttaccttggaaaattatacagttaccccatggagaattagcagcgcccacgaatgatataaatactactgttgctttagc
tttactcacgtcagcggcatatttttatgcgggtcttagcaaaaaaggattgagttatttcgagaaatatattaaaccaactccaatcctttttaccaattaacatattagaag
atttcacaaaaccattatcgcttagttttcgacttttcgggaatatattggcggatgaattagtcgttgttgttcttgtttctttagtccccttagtagttcctataccggtcatgt
ttcttggattatttacaagcggtattcaagctcttatttttgcaacgttagccgcagcctatataggtgaatccatggagggtcatcattgaattgactagttttcaaaagag
tctttttttagcttaactcaattcatgcatggttgcggaaaattcgcttggttggaaaacaaaatagttagaattgcgtatgaatatacaatctagagttgtagaagagag
aataggctatattacggaattgccaaacaaagtatataggcattagggaggggcggagtcaggctagatctatatcctttatgtctataagttcagtcatcttttgtatgg
gtttccactttaaggaatttttttgaatccgattcaatagaaaatgagaaaatacacaaacaaaatagaagaaacaaattgatatgggatattatatattcccaagttaga
ttcattatctaatccgatatatggaatcggattccatatccaattcgatgcagcatattgttatcaattggatatcttgatttaattcctattggatctggattaggtcgatttcc
ataggggttcttcctctatttcaccttttattatgaattagatgataggggaaaaaatagaaactcaaggatatcgaagaggaaagaaagaaggatggaatgaaagat
cagttggttggaaagaaagagaaatagaataatgagtacacaaacctctaatgattagaaactaaaaaggagatctcgaagcagttcggagaattcagattatcgttt
caatttgtactttttagttacttctgtccaatagagcttagaaatatgaatttcttggttgattgtatccttaaccatttcttttttttgacacgaggaactcatcatgaatccact
aattgctgctgcttccgttattgctgctggattggccgtaggtcttgcttctattgggcctggagttggtcaaggtactgctgcaggacaagctgtagaaggtattgcga
gacagccagaagcagaaggtaaaatacgcggtactttattgcttagtctagcttttatggaagctttaacaatttatggactagttgtggcactggcgcttttatttgcga
acccttttgtttaatcctaaaaaagaaaacgagtcctttagattagatactttcttctttttttagtaaattggtatttgcttctgcaattccaattatatcaatactttactcctaat
ttattactcctagagttttctatttatggggacagacaataccccaccccaggaatagctgatttgaggatgatcaatttagaggatatgttcgccgtcttgcttcccgcc
ctttgtttagggcagtggaaagtatttttccttttattttaggaattttgggaacatttcaacaaaggaggtctttcacaggtcaaacgagatctaagacttaatctaaaaga
aattactaaattgaatctatttctattaaaaaaaaattgcattaaaaaaaaccgatcaaaaagggcgagcgaagtaagtgatcgaaaaactttgttctttgttcgtcctatctat
aagaggagagcatatgaaaaatgtaacccattctttcgttttttttagctcactggccatccgctgggagtttcgggcttaataccgatattttagcaacaaatctaataaat
ctaactgtagtggttggtgtattgatttattttggaaagggagtgtgtgcgagttgtctatttcaagaatagattggatctatccggctgcactttagaatatttttttagtatttt
tcgaataaataagaaaagggtgcacgatctcgacgaattacttctgaataaatgcagaaatcatatgaaagaaccatagcatttcgcgacctattggtaaatcaaatca
actttgattctctatagaccaataatatgagaccattaacacggttaaagctaaactgcttgaagtctaggcaaaagggggtactctttctacaactatattagtattagta
ccaaatgctttaaacaggaaatagctaatgtagaatttatctgatatagaacactcatatcgataaaatggtttgaactatttactagaaggggcaccctgcccttttttat
ccaatgccgaatcgacgacctatgtataaaaaaggagaaagtttttggatttgaagaaaaaagtaggaattctatccattttcattttccatttttctatttagtttttcttaat
gaaattgaaaattttaactaaagggcaaatacaaataaaaaaacaactttgctgcccacgatagatttttatctaggcggaagagtcctcttaatatttatctagtcttatat
gggtttcggtatattgaaatataaacagaaaagagagggtagaggataggctcattacattaaaaaaaaatatggaagtagccatagcaaaaaaagaaaaaaggga
gcgtgagagccaaatgaatcgaaagattcatgtttggttcgggaagagatcataaaagttgtaaacttaatagcaaggtaatctactttcattaaaagatttattagataa
tcgaaaacagaggatcttgagtactattcgaaattcggaagaattgcgtagaggaaccattgagcagctcgaaaaagctcgaattcgattacagaaagtcgaacta
gaagcggatgagtatcgaatgaatggatactctgagatagaacgagaaaaagcaaatttgattaatgccacttctattagtttggaacaattagaaaagtctaaaaac
gaaaccctttattttgaaaaacaaagggcgatgaatcaggtccgacaacgggttttccaacaagccgtacaaggagctctaggaactctgaatagttgtttgaatacc
gagttacatttccgtacgattcgtgctaatattagcattctcggggccatggaatggaagagttaaattaattaggccttgaacttctactttcctttagaatttaggcattat

ttttccccttgcttccgaaaaaaaaaatagtaaagaaacactaatggcaacccttcgagtcgacgaaattcataaaattctccgcgaacgtattgaacaatataatagaa
aagtagggattgagaatatcggtcgcgtagttcaagtgggggatgggattgctcgtattataggtcttggtgaaataatgtcaggcgaattagtcgaatttgcagaag
ggactaggggtattgctctgaatttggaatccaaaaatgttgggattgtattaatgggcgatgggttgatgatacaagagggcagttttgtaaaagcaacaggaagaa
ttgctcagatacccgtgagcgaggcttacttgggtcgtgttataaatgctctggctaaacctattgatgggagaggcgaaattgtagcttcggaatctcgcttaattgaa
tctcctgctccgggtataatttccaggcgttctgtatatgaaccccttcaaacggggcttattgctatcgattcgatgattcctattgggcgcggtcagcgagagttaatt
attggggacagacaaaccggcaaaacagcagtagctacagatacaattctcaatcaaaaagggcaagatgtaatatgtgtttatgtagctatcggtcaaagagcatc
ctccgtagctcaagtagtaactactttccatgaagagggggccatggaatacactattgtagtagctgaaatggcggattcccctgctacattacaatacctcgctcct
tatacgggagcagccctggctgagtattttatgtaccgcgaacggcatactttaataatttatgatgatctctccaaacaggcacaagcttatcgccaaatgtcccttct
attaagaagacccccccggccgcgaagcttacccaggggatgtttttatttgcattcacgccttttagaaagagccgctaaattaaattctcttttaggggaaggaagta
tgactgctttaccaatagttgagactcaatctggagacgtttccgcctatattcctactaatgtaatctccattacagatggacaaatattcttatccgcagatctattcaat
gccggaattcggcctgctattaatgtgggtatttccgtttccagagtaggatccgcggctcaaattaaagccatgaaacaagtagctggcaaatcaaaattggaatta
gctcaattcgcagagttacaagcctttgcacaattcgcctctgctctcgataaaacaagtcagaatcaattggcaaggggccgacgattacgagaattgcttaaacaa
tcccaagcaaatcctcttccagtggaagagcagatagctactatttatatcggaacaagaggatatcttgattccttagaaattggacaggtaaagaaatttctggatg
agttacgtaaacacctaaaagatactaaacctcaattccaagaaattatatcttctagcaagacattcaccgaggaagcggaaatcctttgaaggaagctattcagga
acaactcgaacggttttcccttcaggaacaaacataaattttgcacgtctactcttgttagtagaagtagtagaggagaaatcgttgagaaagatttttcatttgaatcat
gcaaaaaagttttcttagtttttagtatagttatttaaagaatagatagaaataagattgcgtccaataggatttgaacctataccaaaggtttagaagacctctgtcctatc
cattagacaatggacgctttttcatattttcttctttcttttcatttttatcggaccaagagaaaaaaactgttagaccaaaactcttttaggaaagaaaaacaaatgcatata
caaatgtatgatgcatatatcataaagaaggaatatggagcgggtagtgggaatcgaacccgcaaccccacggttatgagccttgtcagctaccaaactgttctatc
ctgttaaactaaagagaggggaactagtggataaaagggggggttgaatacgcccctctaccatatctatacaaatagaatagtccatttatacagaatggtaaagag
ggctcttctacgatcatcaattccagaaatccatacaaatacgaaagggtattttatccttaccaactggatcttgttgcacccggtaacaaacatgcataaaccatttct
cgaagtatgtgtccggatagcccaaagtctcgatagttagctctaggtcttccggtcaaaaaacaacgtcgatgaaggcgtgtcggtgcactattacgtggcaggga
ttgcaatttttctcgcattttcgtttttcactcaaactcaaagggtaaactttgcttcttatctttttttttgaagatcgacgaatcaaatgatatttctgttctaatttctgccgctt
cctctctctctgaatcaaactttttttttgccataatgtgccgttcctactatctaccaagtatacggttctaatcctagatggaaaaataaatagaaaaagaaatctaagaa
ggcggatcctccccctccatcaagagtaatgaactaggtgctgatacagtacaacaaaattaactaaattaaccaaacttgcctgatgttgaggcaatcaagaaagc
ggcataagtgaatatataacccacggaaaagtgagctaatcccaccaatcttgcttgcacaatggaaagagccacgggcttatctctccagcgaattaaattagcca
aaggtgtccgttcatgagcccatgctaaagtctcaattaattcctgccaatatccacgccaggaaattaagaacataaatccagtcgcccaaacaagatgtccaaata
agaacatccacgcccataccgataaactattcatcccaaaaggattatatccattgataagttgtgaagagtttaaccataggtaatctcttaaccatcccatcaaataa
gtggaggattcattaaattgtgaaacgttgccctgccataatgtgatgtgtttccaatgccaataaaaagtaacccatccaatggtatttaacatccagaaaactgccaa
ataaaacgcgtcccaagcagaaatatcacaagtaccgccgcgaccagggccgtcgcaaggaaaactatatccaaaatctttttttatccggcattaatttggaaccgc
gtgcatctaaagcaccctttactaaaatcaatgtagttgtatgcaaacctaaagcaatagcatgatgaaccaagaaatctccaggtcctattgttaagaaaagcgaatt
actattctcgttaacagcattcaaccatccgggtaaccatagggttcgacccgcattgaaagcgggaccgctcgttgaagataagagtatatcgaacccatatgtcgt
cttaccatgagccgattgtatccattgagcaaatataggttcgatcaagatttgcttttctggagtaccaaaagcaagcatgacgtcattatgaacataaaggcccaag
gtatggaatcctaggaagagactagcccaacttaaatgagatatgatagcttctttatggtctaacattcttgccaatacattatcctcattctgttccggattgtaatctct
aatgaaaaaaaatagctccatgagcaaaagcccctgtcatgatgaaccctgcaatgtattggtgatgagtatataaagctgcttgagtagtaaagtcttgtgctatgaat
gcataagaaggtaaagagtacatatgttgagctactaaggaagtaataaccccctaaagaagctagagcaagacctaattgaaaatgaatcgaattattgattgtgtcg
taaaggccccttatgcccacgccctaatcgacccccccggaggagtatgtgcttctaaaagatctttgatactgtgcccaattccgaagttagttcgatacatatgaccgg
caatgagaaaaataaatgcaatagctaaatgatgatgagcaatatcggtcagccacaaactttgtgtttgtggatggaatcccccaagaagagttagaatagcagttc
ccgctccttgagtggtaccaaataaatgattactcgaatcggggttttgggcataaagattccactgacccgtcagaaggggtcccaacccctgtggatagggtaat
acatctaagaaattattccatcgaacgtactctcccctggatgctggaatagcgacatgaactaaatgtcctgtccaagccaaagaacttaccccgaaaagtcctgac
aaatgatgattgagacgagattccgcgtttttgaaccacgaaaggcttggtttccatttgggttgtagatgtaaccaacccccctattaaggatagggtagaaagaaata
atagaaaaagagctcctgtataaagatcttcattggtccgtaatccaattgtataccaccactgataaaccccagaataggcgatattcactggaccggcggcgcctc
ctcgagtaaaggcttccacagcgggttgaccaaaatgaggatcccaaatcgcatgagcaataggtcttacgtgtaaaggatcctgtatccatgattcaaaatttccttg
ccaagcgacatgaaacagatttccggacgtccatagaaagattattgctaattgcccaaagtgagaagcaaaaatgttctgataaagacgttcctcagtaatatcatc
atgactttcgaaatcatgtgcggtagcaataccaaaccaaatacgacgagtagtggggtcctgagctaagccttggctaaacctgggaaatcttaattccataatgcc
tttcaaatcctcctagccactatcctactgcaataattctcgctaagaagaatgcccatgttgtggcaattccacccagaaggtaatgggttactcctacagcacgtcct
tgtataatgctcaaggctctaggctgagtagcaggagcaacttttaatttgttatgagcccaaacgatagattcaatcagttcttgccaataaccacggccgctgaata
aaaacattaaactgaaggcccagacaaaatgagcacctaagaaaaaaagaccatatgcggataatgaagaaccataagactgaattacttgggatgcctgtgccc
acaagaaatctcggagccacccattaatcgtaatggaactctgtgcaaagtttcccccctgtgatatgagttaccacccccttgatcacttatagtaccccaaacatccga
ctgcattttccaactgaaatggaaaatgactaccgaaatggaattgtacatccagaatagacctaagaaaacatgatcccaggcggatacttgacatgttccccctcg
cccaggcccatcgcaagggaagcgaaaaccaagatttgctttatcgggtatcaaacgggaactgcgagcaaataaaacacctttcaaaagtattaatacagtcaca
tggattgtaaatgcgtgaatgtgatggactaaaaaatctgcggttcctaatggaataggtaacaaagcaactttgccgcctactgctactaactcgccacctccccac
gttaagctggtacttgttgttgcaccaggagctgttacgctaggcgcgccagcatggagattttgtacccattgagcaaagatcggttgtaattgtatggcggtatccg
aaaacatatcttggggacgacctaaagcactcatggtatcattatgaatgtataaaccaaaactgtgaaaacctagaaatatacatacccagttaaggtgggatatgat
tgcatcacggtgtctaaggacacgatctaatagatcgttgtatcgagtcgttggatcatagtctcttaccataaaaatggctgcatgtgcagcagcaccaactattaga
aatccgccaatccacatgtggtgtgtgaacaaggaaagttgtgtaccatagtcagtagctaggtatggatagggggcatagagtacatatgatgagctacaacaat
ggttgtagagcctagcatagctaggttaagagataattgagcatgccatgacgttgttaggatttcatagagacctttatggccctgtcctgtaaatgggcctttatgag

cctccaaaatatctttcaggccatgaccaataccccagttggtcctatacatatgacctgcgatcaggaaaagaatagcaatagctaaatgatggtgcgcaatatcgc
tcaaccatagaccgccggttattggatctagccctccgcgaaaactaagaaattctgcgtatttggaccaattcaaggtgaaaaaaggggttgctccttcggcaaaa
ctaggataaagttgagccaaaaggtcgcgattcaagataaattcatgaggaagtggtatctccttaggatcaaccccagcgtcaagaaattggttaatcggtaaagat
acatggatttggtgccccgcccaagaaagagacccaagtcctaataaccctgctaagtgatgattcaacatggattctacgtcttggaaccaggctaatttgggagc
ggctttgtgataatggaaccacccagcaaaaagcattaacgatgcaaaaatcaatgcaccgattgcggtacaatagagttgtaattcactagttattccggatgctcg
ccaaatctgaaaaaacccggaggttatttggattcctcggaaacccccgcctacatcaccattcaatatttcttgccctacgattggccaaactacctgagcactgggt
ccaatgtgagtaggatcacttagccatgcttcataattggaaaaacgggcaccatggaagtacatgccactcaaccaaagaaagataatggagagttgaccgaaat
gagcactaaagacttttcgagagatctcctccaaatcacctgtatgactatcgaaatcgtgagcatcagcatgtaggttccagatccaagtggtagtatcagggccctt
agctattgttcttgagaaatggccgggtctggcccattcctcaaaagatgtttttacaggatccctatccacaacaatttttacttctggttccggcgaacgaatcatcatt
aagtcctcctctttccggacaagacatacaaagagagacccgccaactttttagtgaacctttgaaagatagatattatgattagtccttttctttactatctaccgtccttctat
tttttttagttattcactggagcaattatatattgaagtcaatctgaggcaagtgttcggatctattatgacataaggattgggtgcctaacggactttttttatcttggatttc
tccacgtaacaaaaaaaccttttttaatttaaaaagctagtctattttttttaagagtataagtccctatctatatctactttccttgagcataatatagatttttttattcgattcc
aaattccaagataactcattagaattattaataagatggtcctgatatattagcaatatttatattgccccttttttattcgctttattacttctattctagaccctatcgtttatcctt
atgaaatataatataaaatagaaggcagaggaaagagatataatgaaattcttgattcgatcttccgacctaatttatttgattaatggatcaacaaccaaaccccccattt
tctgaaaaaggagagtggtcttattcaaattcaaagcgcttcgtaatcttcaaccagttctgtgcttcaatataatttcccggagtaagcgctatagcttgtttccaatact
cagcagcttgatcaaaccaagcttctgcaatttctgaatcaccctgtagaatggcctgttctccccggtcggaataggtagttccttcccctagaaccgtacttgagag
tttcctacctcatacggctcagaaattgctatcttaatttcccttgtcttaactgaattcgatttctcaaaaatcgatccaatttcttcttgggttacgcagaagaagttaatta
cctaagtttcaaaccctaaattttgatcaataatcagtttgatcttttctcccacctgcagaaaaatgaagcatagatagacctatatccttcgtccgaattttctgaaaggt
aactatctcggtttcatatatgaaatttctatagaatccttgaaaaagactttttccccataagcaagaaaaaaagaacttactatctttgggatctgatactacaccgctgct
taatcccttagtggatcggctctattacataagcggattcctaaattttgccccatatcatgggataagtaagcagttttttttagttgtatcgacccagtcggtcactaatt
gatctttacggtgctttctctatcaatttgagaactctatccatagagtagtataggccatactttctttcttcctattttgattctcgtgaagtgtcctttcttcctacagctgat
agggcaaaatcgttgttttgacgatccctatgtagaaagccccctttttctagtaaatactagaaaatttgatcctttcttatttttcttctatagtggagatagtcgcacgta
atgacagatcacggccatattattaaaagcttgtggtaagaaggggtttcgttctagtgcccggaaataatattccaaagcctttgtatgctctccattgcttgtgtgtata
aggcctatgttatagagtatataacttcgatcatagggatcaatttctagtcgcgtagcttcataataattttgcaaagcttccgcataatttccttcggattgagccaacat
ccgttacggtcgttcgttctattcaaaaaatctccgttccaaaaccgtacatgaggttttcacctcatacggctcctcccttctgtacatagtactaagcgaaaaaatcta
gagaataaaaaatagaattagttccatctcattatggaccgaaaggggctggtatttttccaagaaatctctagccaaccttcccacaagaggtttttcttaacaccaatg
aattctattaatgctagaggaaaacgatagctccaagaatttctttgttctcaacgcctcctatttagaggaattagccacttcaacgatctttgatggttataggggtatc
caaagtacaaacttgatggttgtttgttatcccaaccattcttcccagccctgataccaatcaggaaagggttaatttctaacaaagtttttctcttgttgattcctatttctag
gtgtagtgcttttatccccctatgctacctattagtactagtagagtaggattagcctgtaatacagaacctatcctgtaggtgtaacctttcgctcaatactaaaatctacaa
ttgaagcatccgaggccgcgtcagtcgaggatacacgacagaaggagttgttagttcacctcaccttccctaagcgtgggtttcccttactaatttggtttctctccgc
gaacccccactctttctcgtaaaaccggggtgcaggtagggctaaaaaaacaaaaaaaaagagtcaaatcgcaccatctctataataagtaaatgccctttttttccccg
gaggttgtcggaattattcgcaataaaatattggctacaattgagaaggtcttatcaatgaaatttccatttatacgggatctaggcataattcccaacccattctatcattc
tatatagaattcttttcattccttcacaaaataacataaaaaaaaaaacaaatccattcaattcttataaatcgatccctatgctccaaatggataagagaggtatttctgctc
agcccaaattctctctttttccttctgtttgaacaagaagagatagaaaatatttgactaagattggatttcattccactttcctatttcttttctcaacaacaacttctctcatca
actatttcgcattttcaaagtcattaatcgtcccatacccctattttctatttgattgtatggggtaggataccttatgcaaacagaattctagggttcctttttttatcgaataaga
agaattcttccattctctttttctttggtttggggaaaacccaaactaaaacttttcgagggagcggaattcctagtaaaaaaatcctggacccgtccacttagatgaaaa
ggaatttttctatctaatagaacaatggaactctcgcgcggttgtggttgtacctgtactgcaggaataggaaaactcgctattcactcagttttttttccataataagagt
atggaggagagatggccgagcggttcaaggcgtagcattggaactgctatgtagactttgtttaccgagggttcgaatccctctctttccgtttttcttaattcagcaac
gttaatgattacaatgtatcaaatcaaatgacaatttattccagcaataatacaataccttttatttaatagaaattctctataccaaattactatggtatgtaaaatacacatag
aggaaagaacaaaaaacaaaaaggaatcctagggttaatccatttctgttaggtgaatgggaaaatacgaattaagagccttaggtcgatttagttcggggaaagg
ggaaggggaagaaaattctatgaacctttccgtttttccttaagttcaagtttgacgagagtaatattctacaactaacaactcatttattttgagaccgacccacttcctat
ccaggattttttttactagtcctttatattgcaatgtgtcaatcgtcaaatgctttggcaatttccccgggtcggatgaagcaatagaattttgaaccagacgttttgatcttt
ggttatccttcgtagtaataatatctcggggtttgcaacgaaaacttggtatatcgactatacgaccattaactaaaatatgtctatggttaactaattgccgggcctcgg
gaatggttgaagccatacctaatcgaaaaaggatattatccaaacgcatttcaagtaattgtagtaaaacctgacctgttgaactttttgcttttccagcgatatgtacata
tctaagtaattgtcgttctgtcagaccataatgaaaacgcaatttctgtttttcttgaagacgaatacgatattgttctttttcccagaatggaatttcttttttcagattacttcc
ggatttaggtgtttttctagtgagtcctggtaaagctcccagacggcgtattttttaaaacgaggtcctcgataacgggacatgaagactcctttttttattgaaatttcatt
ttacacaattaatttcattgtatttacattacagaatacatcgaaattaaaactgaattaaagtaaaggataaacagagtaaaatctactaaaagtaccacaaaaaaatgg
aatttcatcaacatctgaatttttgtatatatattatttattttattgttttgtatctagcaaaattgtagggtagaacgacataatagatcctggcttctccatttaattcggaga
aaaagaggtatttttgttcatggaacattgatagagaaaaaagccgactatcggatttgaaccgatgaccctcgcattacaaatgcgatgctctaacctctgagctaag
tgggcttacataacagaaatagtgtaacaaatagaaatatgtatagtataggaaatccgtaaaatgtcagatcttaattattaatcttagctattaactagttcgaaattgg
aagttctacttagaaaaaaatactagaacttcataaaataaagttagatagattttttgaactttcttttttctctaattcgcaaatctatttttctaatagaatctattccaatttc
tatattgaatttgattttagatatttttaatttgatatggctcggacgaataatctaatacatggaaaagaataaataatatatatacgaaaacataataaagagaacatgcg
aatttcttgtattttcagtccatcattatagacatttttttgagatattttgtttttttgttatttgctaataatttaatgattaatatttcactaaggagaacatagaatcatagcaaa
tgaaattgctaattctgattagcaaaaaaaaagaatgaatatcaagcgttatagtatgattttgaatactctaaaaaaggaacgcggtaggtgggggagagaaaaacc
ttgggatatattgattcgcattgaattgcaaatacatcaacgatagaatcaattcaatgctgaattgcaataagcggagtctctcaactagagacgaaccgctagacta
catagagtaatgaattcaacgattcaaaaaaaactaacagatggaggaaattgcacaaggaatcctggtctcaaagaaaaagaaaatggggatatggcgaaatcg

gtagacgctacggacttgattgtattgagccttggtatggaaacctgctaagtggcaacttccaaattcagagaaaccctggaattaaaaaaggggcaatcctgagc
caaatccatgttttgagaaaacaagcggttctcgaactagaacccaaaggaaaaggataggtgcagagactcaatggaagctgttctaacgaatcgagttaattacg
ttgtgttgctagcggaactcccttctaaattagggaaagaagggctttcgaaatctaatacacacgtatagatactggcatagcaaacgattaatcacagaactcatat
cataatataggttctttaattctttttaaaatgaaaataggaatgattatgaaatagaaaattcataattttttagaattgttgtgaatccattccaatcgaatattgagtaatc
aaatccttcaattcatagttttcgaaatctttttaaaagcggattaatcggacgaggataaagagagagtcccattctacatgtcaatactgacaacaatgaaatttctagt
aaaaggaaaatccgtcgactttctaagtcgtgagggttcaagtccctctatccccaaaccctcttttattccctaactctagtatttatcctgttttttttattaataggtttaac
attcaatggaatacatttctttttattatagtatcggcaaggaatgtcgattattaactcgatatttaaatattattaaataggctttctttgtacaatgcataggactgccccc
tccccatttccaaattttggatattgacatagatacaaatactctactaggatgatgcacaagaaaaggtcaggatagctcagttggtagagcagaggactgaaaatc
ctcgtgtcaccagttcaaatctggttcctggcacagaaaaaagggatcttccgaataggtattgatacaaatacctcgagatgggttgggatacatattcgttaataata
tagatagagtatgatttttcatctaagtagataaatctctaaatagaggcacttctttttctgcatttttgcatttcttaattttctattccgctattccgacaaatattttttttattct
tgcttatcttatcttactttcctagttgttctaagtaatgcacgcggtacaaagttcgtggtagggaacttctttgagtcatcatatttttctgttcatacgaaggaaatgaata
tgtgattttccaacgaaatgaagcccttttttgcttagtctatctgaaccttttgtataattggaattaattagaatgtaataggtattctgtttcatctaggaacagagcgtaa
aaatattccttgacttgcataaaatctggagttgtgttgtataagtgagcatgaatttcttatcattcaatgagcatcttgtatttcatagaaattgggggttatatagtcctta
cgtaagggccagcctatccaactttcaggcattaggatacgtttaaggcgtggatgattatcataagagattcccaccatatcaaaagattcgcgttcttgaaaatcgg
aacttctccaaatccagaagacagatgggattctaggattatccttttgggcaaagacttttatgcatacttcttctgggttatctataccatactgtattctcgtaagatgat
acacgctagctaaagatccaccgggtgcaacgtcataagcacattgggaacgtaaataattgtaaccatatacatataaaatgacagcaatggaatcccaatcctct
gcttttatttgcaaagtctctattcctcgatgatcgaagcccaaagatctatgaaccacctcatgtttgactagccaattagataaccaaccctgctgcattatcttgatctc
tcctcctttgtataaatatttcgcagttcgagtgcaagtttgaaatattgccctgctctttctttttcggcacaaagagctcctcctaattcactaatttgtaggaagatactg
gacttttggatttgaaaaaagtttcagaagatatgtctaaagtagatggtgattgatagagcaattcttgctcataagttccagtattagtactgcgccgaacataaagctt
gtggctggtagtaaaacatcgatttttcttttgagatagagttcgatcctcaactatttctcgcgatatcttcttacgaagttttgttagggcatctataacagcctctggttta
ggtgggcaacccggcaagtagacgtccacaggaattaacttatcaactccccgaacagtactataggaatccgtactgaacattccaccagtaatagtacaagctc
ccatagcaatgacgtattttggttcaggcatttgctcatataatctcactaaagagggagccattttcattgttactgtaccggctgttaaaattaggtccgcttgcctagg
acttgatcttggtaccaatccataacgatcaaagtcgaatcgtgagcctattaatgaagcaaattcaatgaaacaacaactggtaccatatagaaggggccataaact
agagagtcttgaccaattcgaaagatcttttagtgtagttgaaataacggaattggaacttgtttggtcaagtaggggaaactcaatcaaactcataactgtcttaatgg
aatcttttccttcttttttttttttgtctgaatattcagttaagaccattccaaggctccttttcgccatgcataaactaaaccaacaactaggataagcacgaaaatgaaagc
ttcgataaaaacggatatacccaatacgtcgaaactcattgcccaagggtagagaaagaccgtttccacatcaaaaacaacaaaaactagcgcaaacatgtaatag
cgtattcggaattgtaaccaagccccccccatgggttctatacccgattcataactagaaagcttctctggtccttcacgaaccggagctaaaagtgctgaaatccaa
aatgctaaaataggaataaggcttgctattattagaaatgcccaaaaaaatatcatattcgtgaagcagaaacataaatgtactcccattaatgtggaataggcggaact
gaattagtcaattcaagtcagcattgtcaatttatacagaatttctctcttttcctcggtgaaacaaggatcggtttttctcaaaccaaagggcttagtttagcctttgtttcct
ctttgccacgtcttctttaaagattcatccaatggaatcccgactccctttctttttgatttactttctatttaggtatggtggagacataattcttatagaaacaaaactctctc
gcttcactttgtctcattttctctagaatctctagaaaaaggaataaaaacgaaaatactatgaattagagcctaattaagataggatgactaatgtatgcagcctaatga
ggagtaattctataaaaataaagaactctatttcagaacgtagattgatttagatttaggtaatctatagatatagataagcaaagtaatatacttcaaacaaagtaggaat
ttgcaagatggagaacatcttgcagttgatttgatagaaattcatttttcttttcctgtctctataattttcgatgaatgagcctctggtaatccttttctctctattttatggcgc
aggcgcctgtccagtctataaacaagtactaatagggaaatgaaaactatactaaaggaaacgtaggatatctctcctaaaatctaaaaaaggacatattagggctat
acggattcgaaccgtagaccttctcggtaaaacagatcaaacggattattatcgaaatgattcgaactgtttcaaagacccaacatgcatttttttgcattgggctctttta
tcaactgatagaaagatcagttagtccaccatagtttttctttacggaaagataatgagatggctccctgcgctctgattgattatttgtattatgatctatctaagagcaat
accaaagtgtttcaaaggaggattaccttgacttaggtctgcctccggcctaaattaaatcaacctaagtgaaatagagtctctatcgttccgctacaagagttgactat
gagacttcatacaccttaaagttcatagaacgaaaagaattttttttggaggcccttatcctcattaagcctagcatttagtgggctggatatttaccttatcaactagcaaa
tcaataaaggttctatttgtttaggcacctggattggtacctgaatcggactgaaccaactatttgtcaggcgactgttctcctattctctcgaatccatgaagtaagacat
tgattttgcaagaagatccactatgttcattgcataataagctcccttgaaaagcattggcgcacgtgtaaacgagttgctctaccgaactgagctatagcccttgtcag
agatatcttaatatatagagaatttcttgtcaagatgaatattctctaatagtagaggatatcctttgatctgtttactataataacataccaataacgaagcggtatttgctta
taaaaaggattcgatctataatcgatcgaagtaaagggtcttcctttgtggtgataaattgcctacttaactcagtggttagagtattgctttcatacggcgggagtcattg
gttcaaatccaatagtaggtaggtaggtagaaaaattactagatagcattggccctacttcgcttcgctatctaataattttttctacccctcttccctttttctttgtatcaact
aaaccgttgggttgtcttcaattagatgggggaatccaattaacagcctcgactcgtatcctagctcgtctgagagctaccttcgcttcaaccaattctttcgtaccctca
gctctactcacgttagcttcggctatttcaagtgcctgttgagcttcttccggatcaatgtcactacccagttccgcatcatttcctaaaatgatgatctcattattaactatt
ctggcaaaaccgctccacagaaccgccgttaaccattgatcgttgaggaggcgtattctcaagggacccatatctacagctgtgttaatgggggcgtggtttggtaa
tacgccaatttggccactattagtagataaaatgatttctttcacttcacaatcccaaataattcgcttaggagtcagtacataaagatttaatttcatttcttcaatttgttctc
ctcttctaagtttatagctttcgtgctagcttcatcgatgttacccaccaaataaaaagcctgttcgggtaggccgtctaattctccggaaaggattagttgaaatcccctа
atagtttctgcaagaccaacatactttcccggagaaccggtaaaaacttctgccacaaaaaacggttgtgataagaagcgctcaatttttcgtgctcttgctacagttaa
acgatcctcctccgataattcatccaacccaagaattgcgataatgtcctgaagttctttgtaacgttgtaaagtttgcttaactctttgcgcagtttcataatgttcgttgcc
aacgatccgaggttgtaacatagttgaggttgaatctaaaggatctactgcaggataaatccctttggaagctaatcctctggaaagtacggtagtagcatccaaatgt
gcaaatgttgtagcaggagcagggtcggtcaaatcgtccgcaggtacataaaccgcttggatcgaagttatagatcccttttttagtagaagtaattctttcttgcaaag
aacccatttctgtactaagagtaggttgataacccactgcagagggcattctccctaataaggcagatacctccgatcctgcttgaacaaaacgaaagatattatcgat
gaatagaagcacgtcttgcttattaacatctcggaaatattctgccatagttagggcagtcaaaccaactctcatacgagctcctggcggttcattcatttggccataga
ctagagctacctttgattcctcaagatttttttcattaattactccagattccttcatttccatataaagatcatttccttcacgagtccgttcccctactccgccaaatacgga
tacgcccccgtgagctttagcaatattgttgattaattccatgatgagtactgttttacctactccagctcccccaaatagtccgatttttcctccacgccgataaggagct

aaaagatcgaccaccttaataccagtttcaaagatggataatttcgtatctaactcgataaaggcgggcgcggatctatgaatagggaatgttgcactagtatctacag
gacccaaattgtcaacaggctccccaagaacgttgaaaattcgtccaagagtagctccaccgacaggaacactgagaggagctcccgtgtcaatcacttccattcc
tctcatcaacccatctgtagcactcatagctacagctctaactcgattatttcctaataattgttgtacctcacaagttacattaatttgcttaccgtcagtgtctcgactcttg
actaccaaagcattataaatataaggtaacttgcccgggggaaaagtgacatccagcacgggtccaataatttgatcgatacgccctgtactttttttcttcaattgtaga
aaccccgggacgagaagtagtaggattggttctcataattatcacataattttcaaaaaaaaaggaatttatcgaaattttgattttttttcttgttgaataatgccaaatcaac
accaaaaaaatatccaaaaatccaaaagtcaaaaggaaatgaattagttaattcaataagagagaaaaggggaccagcacttgatttcgttgcccaaacgaatccca
ttcaatcgtttactcatggaatgagtccgtcggaaagttcaatcaatcttttttcatatacattttgccttttgtaaacgatttgtgcctactctactttcttatctaggacttcg
atatacaaaatatatactactgtgaagcatagattgctgtcaacagagaattttcgtagtatttaggtatttccactcaaaataagaaaagggggtctattaagaacttaat
aaggattagaagttgatttggggttgcgctatatctattaaagagtatacaataaagatggatttggtgaatcaaatccatggtttaataacgaagcatgttaacttaccat
aacaacaactcaattcttatcgaattcctatagtagaattcctatagcatagaatgtacacagggtgtacccattatatatgaatgaaacatattatatgaatgaaacatat
tcattaacttaagcatgccccccattttctttaatgagttgatattaattgaatatcttttttttaagatttttgcaaaggtttcatttacgcctaatccatatcgagtagaccctgt
cgttgtgagaattcttaattcatgagttgtagggagggacgtatgtcaccacaaacagaaactaaagcaagtgttggatttaaagctggtgttaaggattataaattgac
ttactacaccccggagtacgaaaccaaggacactgatatcttggcagcattccgagtaactcctcagccgggggttccgcccgaagaagcaggggctgcagtag
ctgccgaatcttctactggtacatggacaactgtttggactgatggacttaccagtcttgatcgttacaaaggccgatgctatcacatcgagcccgttgttggggagga
taatcaatatatcgcttatgtagcttatccattagacctatttgaagagggttctgttactaacatgtttacttccattgtgggtaacgtatttggtttcaaagccctacgcgct
ctacgtctggaggatctgcgaattcccccctacttattcaaaaactttccaaggtccgcctcatggtatccaagttgaaagggataagttgaacaaatacggtcgtccttt
attgggatgtactattaaaccaaaattgggattatctgcaaaaaattatggtagagcatgttatgagtgtctacgcggtggacttgattttaccaaagatgatgaaaacgt
aaactcacaaccatttatgcgttggagggaccgttttgtcttttgtgccgaagctatttataaatcacaggccgaaaccggtgaaattaaggggcattacttgaatgcg
actgcaggtacatgcgaagaaatgattaaaagagctgtatttgcgagggaattaggggttcctattgtaatgcatgactacttaaccgggggattcaccgcaaatact
agtttggctcattattgccgcgacaacggcctacttcttcacattcaccgagcaatgcatgcagttattgatagacagaaaaatcatggtatgcatttccgtgtattagct
aaagcattgcgtatgtctgggggagatcatatccacgctggtacagtagtaggtaagttagaaggggaacgcgaaatgactttaggttttgttgatttattgcgcgatg
attttattgaaaaagatcgtgctcgcggtatctttttcactcaggactgggtatccatgccaggtgttataccggtggcttcaggggggtattcatgtttggcatatgccag
ctctgaccgaaatctttggagatgattctgtattgcaatttggtggaggaactttaggacatccttggggtaatgcacctggtgcagcagctaatcgggtggctttaga
agcctgtgtacaagctcgtaacgaagggcgcgatcttgctcgtgaaggtaatgaaattatccgatcagcttgcaaatggagtcctgaactagccgcagcttgtgaaa
tatggaaagcgatcaaattcgagttcgagccggtagataaactagatagctagactaagtggataaaattagatagaaaaaaggtctaaataaaaaagaagagaaa
tagaaagatcaaaaatcagttacgaaaatgcagtaattcttctttttcttctaattgattgcaattaaactcggctcaatctgaaaaaagattgagccgagtttaaatagat
tttgatacgatcatgagacttgacaaatcgggattcctctattctatatatttagaagatataaaggtataatacaataaataaatacaaatatagtattatcatatgataatg
gaatcaaatacgcagtatttacagaaaaagttttcatttattgggaaagaatcaatgacatacaatgcattacagacgtatgatcattaccctttaaccgggttattctatt
ccacttctagatagagaaaaaaactaaaggagaatgaatgaaaaaagacagagtttggaagttagacccctttctaagactctctttcaaaaaagaggacattttgaa
acttttaacaggcacaatcgtgagtcaacaagtgactcgaaatgctcgtaagaaaagagaattgattttcaaaatggtagaactagatgacgaagttttctataaccttg
atgaagaggtagttttagtttacgactccgatcaagagcgagaagtctttcatttcagattggactgctatagaatctggacccatcgtagagacgttcaaaaggctcc
tgatgataagaatcatactttcgcggaactccatggggctatgggcttcaacgcggtagacgttttgttccgaatttttctggagcaaacctccgacccaacgatacaa
tgaatttttctattcacaaagaaaaaagattcggaatcgcaatgctactatacgcacccggaggaatattcggaattatattcttatataatccatttttgcgcggggtctt
actaacaggacttcgctgcacgagacgaggattcgtcgaaaagctaaggtcatccggtattttcatacccttttgggggtggtatatggccttacaaagtctaaggggt
agtatgagatctgtagtaggtaaaagaatttgtcccttgattgagtatgctattttccctcctttaccgcgcattattgtatatgcttctagaagagcacgtatgcagagag
gaaattacagtttaataaaaaagcctaaaaaagtttcaactttacggcaatatcaatcaactaaaagtcctatgtatcaatgcttacagcggatttgtggggtccgagag
tggttgaataagtattgcatgtggaaggaagtagacgaaaaagattttggattcgaaataggtgcattcgactaagtcgtactttgaatccaatttcaagttcgattaga
aggatagaaaggccgcgaggatcggaaaagaaaaatcaaatctttttaattgcttctcctttttgctattttcttattatttatataatccattcgattcttttttttagaatact
aaagtattctaaaaaaaagtattctatactataaaaaatctttttttgcaaactcaaaaatacaatagtcaatattcgttataatagatatacttaattatatcataagaatctta
agatatttttgaatagatcaaatcgaatagatagaaatagtaaatttgaatggagacacctattctatgatggattttaacttaccctctattttcgtgcctttagtaggctta
gtatttccggcaattgcaatggcttctttatttctttatgtgcagaaaaataagattgtctagaaccgacgggaccgaattttctcaatgtatttccacacaggatcataata
cggatcttttgtagtgtaagtaatataatatggtatgttatgtggctctttctacacacaaatgcaaacccgctatggatggggattatggatgcggatataggctacgag
cataaatgcatgcatatgcggaaccgggtatagcgagttttttaagtggatcaacaaatactttttgaatagaaagtcaatgtatctaaccaattattttacaggagtatc
tagttggcgaaggcgatttcagaatcaaaaaaagtaaagtaaagtcaaaatcatttagcttattctctcaatttcaatcgaccgctgttagtatatctaatatgaattggcg
atcagaacacatatggatagaacttctaaaaggttctcgaaaaagaggtaatttttctgggcctgtattctttttctaggttcactaggattcttagcggttggggcttcca
gttatcttggtaagaatattatatctgtacttccatctcaacaaattcttttttttccacagggggtcgtgatgtctttctacggaatcgcaggcctattcattagcgcttactt
gtggtgcactattttgtggaatgtaggcagtggttatgaccgattcgatagaaaagagggagtagtgtgcatttttcgttggggattccctggaataaaacgtcgcgtc
ttccttcgattccttatgcgggatatccaatcaattagaattcaggttaaagaaggtctttttcctcgtcgtatcctttatatggaaatccggggccaggggggccattccct
tgactcgtactgatgagaagtttttttactccacgagaaattgaacaaaaagctgccgaattggcttatttcttgcgcataccaatggaagtattttgagtaccaattgaatt
ttttgaaatgaattgaatgaagaagaattggaagaagaaaagttttctcaacacgagggaaaaggtcccttcgaaattgcattattgtaagtgggatatatggcgtaat
attcttccattttcatccgaaaggactttttttctatttctctattccactccatctagatctaagaaagaactcaatgcaatgaaattctactagtatagaaaaaagaggaata
gatacaaggtctcaaaccttgttatagaatttttccttcaaagaaaaagaaatatcatatagagtcagcgaataaagcggattcattaacaactcaatttacagatcaaaa
atgaaaaaaaagaaagcattgccttctttcctatatcttgtatttatcgtacttttgccttggggagtctctttctcttttaacaaatgtctggaactttggattaagaattggtg
gaataccaggcaatcccaaactctcttaactgctattcaagagaaaagagttctagaaagattcatggaattagaagaccttttttatcttggacgaaatgataaaagag
aaaccgaatacacatgtacaaaacccccctataggaatacgcaaggaaataatacaattggccaaaatagataatgagggtcatctccatatcattttgcatttctcga
caaatataatctgtttggctattctaagtggttctttttttctgggtaaagaggaacttgttattttgaattcttgggttcaggaattcttctataacttaaatgactcagtaaaa

gcttttttattcttttagttactgattttttgttggatttcactccacccgcggttgggaactactaattcgttgggtctataacgatcttggatgggttcctaacgagctaatt
ttcactatttttgtttgtagttttcctgtgattctagatacatgtttgaaattttgggtcttttttgtttaaaccgcctatctccttcgcttgtagtcatttatcattcaattagtgaag
cataaactcatttgatctcctgatattaatcaaaattagcatcctttttcctttagaaagaaagccttttccattttagcaaaattctttttttctatttctacctgctcaaggtatt
catcattccagtacaactgttgcagtagaatgacaacagattcgtgtatagggaactagattagcttagctacctatctaatttattgtagaaattccgggatctgtgattg
gacatggaaaatagaaatactttttcttgggtaaaggaacagatgactcgatcgatttctgtatcgatcatgatatacgtaataactcggacatctatttcaaatgcatatc
ccatttttgcgcagcaaggttatgaaaacccacgagaagcaactggacgaattgtatgtgccaattgccatttagcgaataagcctgtggatattgaagttccccaag
cagtgcttcccgatactgtatttgaagcagttcttcgaattccttatgatatgcaactgaaacaagttcttgctaatgggaaaaagggagggttgaatgtgggtgctgttc
ttattttgcccgagggattcgaattagcgccgcccgaccgtatttctcctgagttgaaagaaaagataggaaatctctcttttcagagttatcgtcccaataaaaaaaat
attcttgtgataggccctgttcccggtaagaaatatagtgaaatcgtctttcccattctttcccccgaccctgctatgaagaaagacgttcatttcttaaaatatcccatata
tgtgggggggaaaccgaggaaggggacagatctatcctgatggtagcaagagtaacaatacggtctataatgcaacgtcaacaggtgtagtaagaaaaatactgcg
taaagaaaagggggggatatgaaatatccatagtcgatgcatcggatggacgccaagtgattgatcttatacctcccggtccagaacttcttgtttcagagggggaatc
gatcaagcttgatcaaccattaacaagtaatcctaatgtgggagggtttggtcagggggatgcagaaatagtgcttcaggatccattacgcgttcaaggccttttgttc
ttcttcgcatccgttattttggcacaagttttttggttctcaaaaagaaacagtttgaaaaggttcaattgtacgaaatgaatttctaggtcccgggatttcttaccatcaag
ttggtaaaaagccgcgatttattggcgattgctagaattctctatgatccattttggaaatcttttttttgtttagactatttttttgtttgcgagatgtcttgtctgcaattccttatt
tctatcatctcatgcaaaacgaggagaatgcaaggcaagggcgagaacaataaaaggaacaaatccttttaggagggattgctggtcttcttaatcctctattgggca
caagaaaaaggctttttttgcctttttcttgtgtcgattcttcttgtattgtatcgaaatatgaatctttttttcttcctattcggcaaagattactatttcttctttatttgggtctgtctc
ttgaacctcttttgcttaggttcaggcgatgtagtggacaaaaagagggaaagaaaatatggcgggggacaaattttttgtgaccaaatagaattgcttgacttgttca
attaagttcaatttagaacttacagaaattttgcaaaaaaaacggatactcttccattgaaggctgtttttttatttttaggctagttgagtagttttaattaaggttttattagttt
attactctaaattaaatcaatgatttacaagagacttcctccggggaataaaatattggatcctcgattgatcctttctttctcctcgcttcataaaagtgaattaatttcattg
gcgagggggttataaatcaactgatggattacttcactaacattattaacaaacaaaattaacaaacaaaacgaataaatagagggattctgaccatcagagcaaag
gctttctctttgttatttttacaaatcaaaataggaaacccgtttgtaggttatggaatagattaaaaaagtcgggttataagagtaagaattccgcgggtcctttccgctct
aatcagataaaagggggtaaggacccgctaagttcctattttttcatgtttacaacctggtccctccaattactatagagatgaacccaatccagaatatgaaccgtaaa
agaaaacacctattaaaccaatcacagcaataccggttacagtacctatcagccaaagaggaattcttccagtagtatcggccatttcccctactttcctccacattttat
caagtggtcatgctagagacaaaaacagtcatggatagttataaggatggtatccttccaaatggggataagagaattcttactactctctttctttctctcaattgaaga
agtaattggaaaataaaacagcaagtacaaaaatgagtaataaaccccagtatagactggtacgattcaattcaacattttgttcattcgggtttgattgtgtcatagttct
atagttggaatttggtttatcgttggatgaactgcattgctgatattgatcccaagaaaaaaacagtaggtacagctagtccgtgaacagccagccatcgcactgtaaa
aataggataggttcgatctatggtcattgagggcctcctaaaaggatctactaaattcatcgagttgttctaaagaatcaaaacggtcggttattaacggaattccttgtc
ggctttctgtgaaatactcgtttggccgaggacttccaaacacgtcataagctaaacccgtactgacaaataaccaacccgcaatgaatagggaaggtatagtaatg
ctatgaataacccagtatcgaatactggtaataatatcagcaaaagaacgttctcccgtgcttccagacatgctgagctccccaaatttttgtacattcaaaaaaggaat
tgattccgtaaaagatgggatcaaccagtaaatagaaaattactgatatttcatccttgtgagattgtcaattttgtaccaaaggtgtattttgagtataccaaattagtata
gctatccttcctatggcacagcaatcctgtttcgagaccaagcgaaacagaattcttttttttctcttctttgttccttgtctatagggtaagctatatggtattcaaggcatca
atagaaaacctcaattttgagggtcctacttaattttcaccggcttcggaatagtagaataattcggaatagggctcaagatcttgggaaaatctaagttaatgatcaac
aggtttggataaagaatttaggaaagatattcttatactgacacaatacaaagagaagtagatgcgaaagctatcccttcgaatccaacctttccccttaaagaatttaa
ttggttagcataatataatatctaataaatagaaaatcaaatagtagataatctgttatgaaagagagaaaacattctttgaagaatcaagattcgtaatcaacccttgcct
tgtttactaactttcttgaccaaactgcaagcgtggaactttgccagttcttaggtttccgaaatagtgtaatggaaaaagaagtgcttcgaatcgactttgattggggttc
gaaaaaggaattaaaaaaaagtaaattcaggaaagatttcctttttttaggagaccctcgaggggtcgtggaatgcttttcttctcctcttattccatatggaatacaatca
attaaaataagaaggaataggggaatattcgactgttcgctccaaaaagaaggttaaatcatcctattgaaaaagaccaaaatagaaagaactttttcaaattcaattct
ttatttttcactggggttagctgatctagttcttaatatttttactttactcaattgacagattacacagcaaatctcttgattcggaattagggactcatgccccatctgatga
atccattttcttttacacttctgtatctcactctatcttgtttttttagtattatctaaaataaccgatgaattatgaattttccataacttaggtaagtgctttaccaacatatgtagt
gtagtaaaaaaaaatggaatttaaccctttcatgcttactataactagttatttcggttttctattggctgctttaactctaaccctagctctatttattggcttgaacaagatac
gtcttatttgaaatgaattgaatgaataaggcagaaaagaaaaaaatctctcttttggattcctggtattctacgcactaattaccaattcttttttttcttggtcattgagattcgt
ggataatttagactactatttagggataaatcgtacctcttttttttatcccctcgaacaaatcgaaatgattgaagtttttctatttggaatcgtcttaggcctaattcctatta
ctttagcgggattattcgtgactgcgtatttgcaatacagacgtggggatcagttggatctttgattgagtaatatttcttttttgattgccctcctctctggtctggaggag
gtcaaattggaattgcaatttgttttgttaagttattttgccctgcttcgacataagatagatggaatcacgctctgtaggatttgaacctacgacatcgggttttggagac
ccgcgttctaccgaactgaactaagagcgctttcaaaaatcaaatccttttctactcctaatgtgtctcacgtacgtatagtatccacaaattaaagttatacccgctttaa
tcgacctcctcactactgcctataaagaagaaagaagtaataggtagggatgacaggatttgaacctgtgacattttgtacccaaaacaaacgcgctaccaagctgc
gctacatcccttttccaaattgttgtacaatgtcattgtacacaattcctatcttgttttccacatcctaattttcttgtctttttctatctatagagaatcctcgtgtcatttcttcttt
ttggtctcatataatcaaggaatggtatatatctaaatccaatctaatttcacctataaaagaaagattactattccttggtaatctataggaagggattgtctttttcgtttta
gttcggaatttcgcctaaacaaaagaaatacaaatgatcttgggcaataatatctgatcatatacgtattccaataaggaaggaggattttcaatgcgggatataaaaa
catatctctctgtagcacccgtgctaagtactctatggtttggggctttagcaggtttattgatagaaattaatcgtttatttccagatgctttgtcattcccctttttttcattct
agttgttgctatgtgaggaatagagttcttcgtgacatgataaaaaaattcccacttttcaatcccttttttagtataggaaagaaaaagaaagaaaagatagattgggttgg
accttagagtcatgaaaaatttggtaaatctcatttttgaaaaaagaaattcaattaaaagcagtatccaagctaagtcaggcctcagaaatcagagcatagaaagag
gctgggttggctactaaaatgaaaggattttgcttcaaaatccttgctaatttgacaaggattgtattccttaattatttctctattttttattacttaattgaaaaatttccaaaa
atttttattctaatggattttcttcttcctcttcggtttcaaaatagaggaataaaagaataagtagaagaattaagttaagtcaatccaaaaaggaaaggaggttcatggc
caaggggaaagatgttagaatcagagttattttgcaatgtgtgagttgtgttcgaaaaggggccaatgaggagtcggcggggatttctagatatagtactcaaaaga
atcgccacaatacacccggacaattagaattaagaaaattttgtcgttattgtcgtaagcatacgattcatgccgaaataaagaaataggagcatcttgtgttcgatcttt

ccaaagatcaaaaagaataagaacttcctatttaatatatagagcatagatagaatacaaaatacaaatcaacttgtctgatttccattagatattatttcatatgtatcgag
ggtattcatctaatatatggaccaaagagagactatttcttctggatccaaaattaataaaataaacaaatcaattttttcaattttaaaataaggaataaatcatgtataca
tctaaacaaccctttcataaatccaaacaaacttttcataaatccaagcaaacttttcgtaaatccaagcaaacttttcgtaaattcaaacaaccttttcgcaaacccaaac
aaccttttcgtaggcgtcctcggattggcccgggggatcgaattgattatagaaacatgagtttaattaatcgatttattagtgaacaaggaaaaatattatcgagacga
ataaatagattaaccttgaaacaacaacgattaattactcttgctataaaacaggctcgtattttatctttcttaccatttcgtaactatgagaatgagaaacaatttcaagc
ccagtcaatttcaataattacaggtcctagacccagaaaaaatagacatattcctccattaacacaaaagttcaattccaatcgaaacttaagaaactccaaccagactt
taagaaacaacaatcggaacttaagttccgattgttgatgttttattcgaaagggccagactatatataaagaaagtaatccaatttagattcttgggtttgttataagaaa
gaacaatggggaagaaaaaatagttttttatttattgcaacatgctcgttgattcctaccacttaatcttaatttattgtatcttcccggagttacctctccgggaattcttttt
taattattcctgtatattactttttatccctttaattgataatctttattttattggaaatcgtgtaaagattatttggatttaatacagctacttgtgcaaggattttacgattaaga
atcaattctttcttgtaaagattgtgtattaatttactataattatcgaatactttatgtatccgcgttgctgcgtttatccgactgatccacaaacgccgaaaatccctcttttg
cctgactctatctcgatgagaggaaacaaacgctcttcttacctgttgagtaatcattcgattaagtcttaaatgagcccctctaaagtttgaggcaaatgaacgcattttt
gcccgtcgtctccgagctatatatcctcgcggaactctggtcattgaatcaaattaaccttaatgaataactaatgatttctcttcttttagccatccttttcccattaataac
aaaacgaattattcccatatataaaatattagttccaatggcttttgctactataaccttcccaaccacaattttctcttctactcccttcagttatttcgcacgatactaaaaa
atagtgggttccatcgtttctatggttcccttttaaacggcgaggccctctctatacaccggagccctttctttcatttcatcaaagggtattgtgaacttgtatagttcacat
tctttggctctacctatccattatagagtaaatagctcttttcacaataagagttatccatacagtgacggcatttaattatgaaagttggctaagtagctgaccctcttagt
ccgtttttttaagataaaggagcataagcctttatctttttattactatttcctccgcttaatgaatagccgtttgctaccaatgggggaattgcttcttatttccaatctagatg
attggatttgcaccaaaggaaaccagaaattccatataccgtagaaatctaggatagagaagctctatcctattcattggtaccgatcatggatacttcaaaaattgtctt
atttgtttgaacccatgatccgaacgagtcgcacatacaccctagcacatgttcctcgacgctgaggacatcccttaagcgcggccgattttctagcatttcgtattgg
ctgtcttgcgtttctaataagttgtttaaccgttggcatgtcgtatgtatatagaaaaaatggattggtttagatcgatcttaacctgaatgattgatcattatgaagtatttcc
attaaattgcataaaacccgaatttagggttgaaataaatttacaagaaatctggccactaccaatccttaaacatttctggaaaccacactggatcagtatcgcagtgc
tcgtcaagcatttcatcccctacaatatcgacaagtccataagctttggcttcgtctgctgacataaaaacatccctttccatgtcttcggatacaacccaaaaaggcttg
cctgttcttagtgcataaacccttgtgatcatttcgcgaactttgtgtaactcttccacttctagtaaaaattctggtgttcttgcccgataataagcactagcaggttggtg
aagcataatcctcgcgtgggggaatgctatacgcttggtgggttctcctccaagcagaatgaaggacgccatggacgcggctattccgaggcatattgtatatatat
ctggtgtcaccgtttgcatcgtatcaaaaatcgccattcctgagattaaccacccgccgggggagtttataaacaaaaaaatatcgctaattccatcttctatactgaga
tataccatgagacctgtaatatgattcgtgacctcgcaacgaatctcttgacctaaaaaaagtgtcctttctcgatacataacattgtataagtcaacccaagtcgcttctt
catctccgggaatccggtaaggtacttttggaacaccaatgggcatattagattaatattattaaatttaagtaagaaaactacactttaatatggaaacgtaagaatgg
aagagaaagaaagaatccgcagtttattgttttcactttttttcttattctatatgaatactatagattctattaatacgtagattgaaataatacatagattgaaagattataca
tataagtaggataagacagattgaataaagaaaaaagaatgggtgattcgaatactaaacaaaaagagatagggatctattcttcgtttttccaaataagccaagctg
cccattgcatattggcacttatcgagtatagaatagatctgcttctctttcttcttacgaacagaattggcttcttatttttaatggaatgaaataaatattcacgctttctgac
acagaatcccctagaagggttaggtacataggatatggatagtctttgccaatgcgataaaataaagtgacatcgtgtctatttttctttgctaaaggggtatttccatgg
gtttgccttggtatcgtgttcatactgtcgtattgaatgatccgggtcgattgctttcggtgcatataatgcacacagctctagtttctggttgggctggctcgatggcttta
tacgaattagcggtttttgatccctctgatcctgttctggatccaatgtggagacaaggtatgttcgtcattcctttcatgactcgtttaggaataaccaattcgtggggtg
gttggagtatttcaggaggaactgtaacgaatccgggtatttggagttatgaaggcgtggcaggggcgcatattgtgttttctggcttgtgtttcttggcagctatctgg
cattgggtatattgggacctcgaaatattctgtgatgagcggacgggaaaaccttctttggatttgcccaagatctttggaattcatttatttcttgcaggggtggcttgct
ttggctttggcgcatttcatgtaacgggtttgtatggtcctgggatatgggtgtctgatccttatggactaactggaaaagtacaagctgtaaatccggcgtggggtgc
agaaggttttgatcctttttgttccgggggggaatagcttctcatcatattgctgcgggtacattgggcatattagcgggcctattccatcttagtgtccgtccacctcaacg
tctatacaaaggattacgtatgggcaatattgaaactgtactttccagtagtatcgctgctgttttttttgcagctttcgtagttgccggaactatgtggtatgggtcagcaa
ctaccccaattgaattgtttgggcctactcgttatcagtgggatcagggatactttcagcaagaaatatatcgaagagttagtgatgggttagccgaaaatctcagtttat
cagaagcttggtctaaaattcccgaaaaattagccttttatgattatattggtaataatccggcaaagggggggattattcagagcaggctcaatggacaatggggatg
gaatagctgttggatggttaggacatcctatctttagagataaagaagggcgcgaactttttgtacgccgtatgcctaccttttttgaaacatttccggttgttttggtagat
gaagagggaattgtgagagcggacgttccttttagaagagcagaatccaaatatagtgttgaacaagtaggcgtaacggtggagttctatggtggcgaacttaatg
gagtaagttattctgatcctgctactgtaaaaaaatatgcgaggcgttcccaattaggggaaatttttgaattagatcgggctactttgaaatcagatggtgtttttcgcag
cagtccaaggggttggttcacttttggtcatgctacctttgctttgctcttctttttcggacacatttggcatggggctagaaccttgttccgagatgtttttgctggtattgat
ccagacttggatgctcaagtggaatttggaacattccaaaaagtcggagatccaactacgaggagacagccagtctgaggccgcattgctttggtatctttcacctct
ctttttgatttgacatgggaaacatctcccatcccttctttgactctttttcctttttatacgggaaatgatcccaaatgacaaatgaataggtgtggaagttataattgtaa
ataaaccacgatcgaatctatggaagcattggtttatacgttccttttagtttcgactttagggataattttttcgctatcttcttccgagaaccacctaaggttccgactaa
aaaagtgaaataatttaattgaagtaagaagtctccccatctgggagacttcttacttcaattagtccccgtgttcttcgaatggatctcttaattgttgagagggttgccc
aaacgcggtatataaggcatacccagtaaagcttacaagtaaaccagatatggagatggcgactaaagttgctgtttccatttttatagaatttcaagattacaatggat
ctacgaaaagatcgtgtatttacaactacaacggaatagtatacaaagtcaacacaaatcattaaatggaatttatggctacacaaaccgttgaagatagttctagacct
ggaccaagacaaactcgcgtaggtaatttattgaaacccttgaattcggaatatgggaaagtagctccgggttgggggactactccttttatgggggtcgcaatggct
ttattcgcggtattcctatctattattttagaaatttataattcttccgttttactggacggaattttaatgaattaggtttctactaacgaaaactacgaagtcatagtttttccat
ccaaaagagcctttctactttaagctctacatttctagacattctggtagttcgaccgtggaatttttggtttcggtatctctggaatatgagtgtgtgacttgttagaatttg
ctcctattgataatacatagaaagcacctgttatctctatcaagatgattctaattcgtcggatattatttattctagtatctggaacacgaaatagatagagtggatcaaga
aaaaaaaatgaaactatgattcatattaactattcagacctcgcaaccagactgaaaaaaattcaagtagttcttaataaaaataaaaaaagaaaatttcttccttccaatt
ttgtttgcccaaaaaacaactttttttctctcgattttgtcgagtcattacaccgattcaataaatgatcatcaagcggttcttattcgaagaacccttgccttttgtttagcttg
agactcaatcatcgtggctctagtatgaatctaaggttttaattgaactgattcataggatcgcaacaagataatttctaccagaaaactactccaatttttgctttatttattt

atctagtaaaacaagagtaaatctgcattacgcacaaaaaaaagaaatccaaaatagggaagagaaaaatcaagaggcctctaatgatcaacatttgggaaagaaa
gatagacgagccaacttgagattttttggcattatcatcacaaagaagaaattatggatttttcttatttcatatcttcaaggcaaatcgacccaacccagtggctgatga
agttttgaacctttttctaatatccgttgaaaatttgtgtgtttctgtttgagccgtacgagatgaaattctcatatacggttctcggagggggggttcgggttagttacctat
ctcaataaagtatatgattggtttgaggaacgtcttgagattcaggcaattgcagatgatataactagtaaatatgttcctcctcatgtcaacatattttattgtttaggggg
aattacacttacttgttttctagtacaagttgctaccggttttgctatgactttttactatcgcccaaccgttacagaggctttttcctcggttcaatacataatgaccgaggc
caactttggttggttaatccgatcagttcatcgatggtcagcaagtatgatggttctaatgatgatcctgcacgtatttcgtgtgtatctcacaggtgggtttaaaaaaccc
cgcgaattaacttgggtcactggtgtggttttagctgtattaactgcatcgtttggtgtaactggttattctttaccttgggatcaaattggttattgggcagtcaaaattgtg
acaggtgtgcctgacgcgattccggtaataggatcacctttagtggagttattacgtggaagtgctagtgtgggtcaatccactttgactcgtttttatagtttacatacct
ttgtacttcctctgcttactgccgtatttatgttaatgcactttctaatgatacgtaagcaaggtatttcgggccctttataaggaaggcatctcatagagagttctaattctc
atatatcatatcgggtaggttgtggtatttcattgctacaaacatgggttattctaaaataagacatgtcatttagatacttctcttcaactccgaactattgtgatacaaata
atacaaatagttgaagttaattttacgaaagaaaagaaggcggattatgggagtgtgcgacttgaattattgatttggccatgcagatagagagttggatctgccacat
tagaattcacgaccaaaggtgtctccatatccaatcaacacgtaagtcccctgtctagtaaggataggctggttcacttgaggagaatattttctatgatcatacctcaa
ccatgtcatccatgaagaggctccgtaagatcccatagagtagaaatggaataagtcatgtgacatgatccaattctctatttattacacttactttttattatagtatggaa
atgcattcattttctttgcatcgattgcgatctgcaatactatcggagtaaaagaagggatctaaggaagaacgtaggctaaactttttgatttttttattagtaacaagtaaa
tactttgtttggacgtaagaaacttgcaatattgggggggataaataccaactaatcaagagacgcgagacaatccacaaagcaattgatcatgatcaaatttgtaag
cccacttggatattgagcatttacccataagagtaggattcttttcaatgaatagttgtaggtgcaacttcggaaaatagaatctgataaagcttttcttacttagagccatt
gagccattatataccttattctattatggatcttctacggttttatttctttcattcttgctcgagccggatgatgaaaaattctcatgtccggttcctttgggggctggatctta
aagaattcacctatcccaataacaaagaaacctgacttaaacgatcctgtattaagagcaaaattagctaaagggatgggacataattattacggggaacccgcgtg
gcccaacgatcttttatatatttttccagtagtaattctaggtactattgcatgtaatgtaggtttagcggttctcgagccgtcaatgattggtgaaccggcggatccgtttg
caactcctctggaaatattacccgagtggtacttctttcccgtgtttcaaatactccgtacagtacctaataagttattgggcgttctcttaatggtttctgtgccgacggg
cttattgacagtaccttttctagagaatgtcaataaattccaaaatccatttcgtcgcccagtagctacaaccgtttttttaatcggtactgcagtagctctttggttaggtat
tggagcaacattacccattgaaaaatccttaactttaggtctttttttagggatttttcagtttgattcattcaatcgtgaagtaccgtgcataggtatctaggaaatagttactt
ccaagtgaatcttccctagatacctataatctattttattatgatccatttcgcgaaaatatagattgtcccaaagatgcaaaattgttttcttttttcttttttattctaactcgaaa
aagaagaagaggaaaaaattgcaatggatttaaaactagaacttattcttaggtaaatccattgggagatgcttctctagagtgtcccatatctgttttccatcttgcatac
gaaaactgtcaattctcatcagatcttcttccgtcttactcaaaaggtccaatagtgtatggatattggcccttttgagacaattatacgttctagaaggcagttctaattga
tcaataaaaatacaattcaatggaattctttttttgtttttctttagattagttaatctttttttgaaagcttaaaagggggggaagtaaacctgtttttattttcttggaaacgagta
ccctcttcctccgtgtgaagaaaaggaagaaataaatcaatcaaattacgagaagcctcataaagcgcttccttaggggttaaacttccattggtccatatttctagaaa
aagtatctcatattttgcatttccattcccacaagcaaaaatactataattcacatttcgaacaggcatggatacagcatctataggataacttccatcttgagtgttctttct
gacttccgtgtgatatccgcgatctctcttgatccgtaactcaatacggaaatccgtgggttctgtcaagttagctataggttgtgccgtatcaacgatttctacggaag
gcggtaagataatatcttgggcagttatgtatctaggacctttgacgcaaattgatgcggttctaactccatagagattacttctcaatacaatttctttcaaatttagtaaa
atttcttgtaccgattcttcaatacctgctattgtagaatattcgtgcggcacgctcccaaattttgcatgtgtgatacatgttccttctgtttctccaagtaaagctcttcgca
aggcaataccaacggtatccgcttgaccttttctaagcggggacagaatgaaacgaccataataaagacgcttactatctactcttgattcaacacacttccactgtag
tgtttgagtggatcctgctacctcctctcgaaccatatagactagtattattatttgatcattgaatcgtttatttctcttgaaaggggtttaattcttttacagacgtcttttttta
ggaggtcgacacccattatgcggcataggtgttacatcgcgtatacaacttaatcgtacaccacttttagcaatggctcgtaatgcggcatctcttccactaccagcac
cctttaccataacttctgctcgttgcaaacccactgtacgaatagcatctacagctgttctttgaccagcatagggtgatgcttttcttgagcttttgaatccacaagtacc
cgcggaggaccagaaaccacccgaccttgcggatctgtaacagttataatagtattgttgaaactagcttgaacatgaataactccttttgttattctacgtgcactctt
ccgtaaactaaaacgcgcattcctacgcaaaccaatacgtactttcctacgtgaaccaatttttggtatagcttttgtcatattttattatctcataaatatgagttagaaata
acaaaaagaaaaaaagatacaaagatatccgtttcagggtaaaatatatcctttacttgaattttttttatttggaatttggacattttcgcgggtactttttttttttactttttaga
aagtcaagttctttttttcgaaagattacccctgtctttgtttatgcttcggattggaacaaatgactctaattcgtccacgcctacgaatcagtcgacattttgtacaaattta
cgaacagaagctcttattttcatatttccgtattcctttcttaaactatgaatttactcttttggaaaaaataagtctctttgtttgaattttagaactttgaattttttaccctagaa
aaaaaaagaaaaacctaaccctttgaatctttggtatccttcaaatcttcggtatccttcgagtcttcggtacgcttcgaatccttatggggaagtctataaattatacgcc
ccttgcttgaatcataacgacttacttcaattttgaccctatcccccatcagtattcgtatagaactagaccggatctttcctgaaatatagcccaggatgatggtgtcatt
ctctaggcgaacgcggaacattccgttgggtagggcttccataactaaaccctcgaaagtaatttttgcttctctcgggttttttttttctctcctatttttcttttctgtcatatt
ttttttctcctatttttctttttttattttcaaataaaaaaaacgttgtatttttgtttgaaaaataggaagttcaagatagaatttgggtactataactataggtggggcgattaccg
ctataggcggggcgattaccatatataacataagacttctcccccaattctgtttagtcgagcttctcgatctgtcattatacctcgagaagtagaaagaatagcaattcc
cattccgcccaaaaccttaggaattccttgatagttggcataaattcgtaagccaggtcggctgatacgctttaaaaaggttctagttctatatattccctttctagtctttct
cttttgatgtcgcaaagttgaaaccaagaaatatctgttactttcctgatgtttccgaacactttcaataaaaccctctcgtagaagtattttaacaatgttttcggtaatattt
gtagatactacccgaacagttcctttttttattcatgtccgcgtttcttatagaggttagtaaatcagcaatagtgtccttgcccataagactctaattctaggttcctcctaatt
tttctataatcaacatgttctctttttttcttttcgttttggattctaaagcatatacgtgaaacacaatctactaatttttttatttgatctatatcttgtctactagtatttataatacttc
aggagctaatgaaactattttagtaaaattcaattctctcaattcctcggcgatcgcgccaaaaacgcgagttccttttggatttcctttttgatcaatgataaccgctgcat
tgtcgtcatagcgtattattataccgtcttcgcatttgaactctttacatgtacgtacaattacagctcgaattacttcggatctttctagaggcatttggggcactgcgtctt
tgattacagcaacaataacatcaccaatacgagcatatcgctgattactagcagctcctatgactcgaatacacatcaattttcgagctccactgttatctgctacattta
aaagggtctgaggttgaatcatattattttgatttcaatttgttatttcaatgcaaaaggatgaaagaaatattgtctttccagaaagaagaacctggtttttttattttcaatac
tactttttttttggggggttatatttctaaccgaagaaattgacttcgtatgggcattttgctggcagctatggaaatagctactctagctacagtttcggatactccgcccat
ttcataaagtattcgacctggtttaacaacggctacccaatattcgggggatccctttcccgaacccatacgtgtttcggtcggtcttattgtaaccggtttgtcgggaaa
tatacgtacccagatttttccaccacgacgtgcatatcgtgtcattgctcttcgtcctgcttctatctgcctcgccgtaatccaagtgggttcaagtgcttgaagagcatat

ctaccaaaacaaatacaattgcctcggtaggattttcccttcattcttcctctatgttgtttacgaaatctggttcttttggggttatagtcgatggttctctcttagttccatct
ctactgcaaaactggacatgagagtttcttctcatccagctcctcgcgaatcaaatgagaaagtgtgcaaatttctctaattccacaatattctaaaatattacggataga
tacacattaataggaaaggttagggtttttaatattgaatttattaaaatactaaaatatatagtcaagaaagaggatctagaatatatctagatgtgtatgtttatttatctat
attctatatttaatgtaattcttttttttttttaatctcctttttttattttactcttattgaatcgcggtaaagtattctaattcaataaaaatttcgcgggcgaatatttactctttcct
gtcttatttgttaattcataaccttatcaaataagacaattttttggtttgttccgccatcccacccaatgaagtattgggattcttttcaagaaaatcctatccagtcataggt
tctgtcgttcccactgcttctcctttaatggttaggtttgaatctcgcaatggagcttccaaaaaatttctttccgagtcaatttctcagttttattaactcgggacgctctttgt
tattccttgaaatttgtatttcttgtttcaatcaaattacgatttcgaattctctgttatttttattatattgatgctttatcacattgctttttatgatgtaattcatagaccatacatat
tggaatcctatatctttcttatttctcttccttctttctatcatccttcctttttatctacatccccctttagttttgcttcacaacttagaatcctatttttttttaaagaaaaaaattgcagt
tgctacacgtatatgatagatttactcatttatgatagatgtatttattcatatagtgactgtttcttagttaggatctcgacaatacgaagcaataggttggttattagttcatt
ttctataattacatagtagggttcattctattttttttaatccccttttttgaaccctaaaaaaaaaactaacgagtcacacactaagcatagcaattttattaaaagatttctcaa
ttttcattaaatcttatagaaagaggtagaatttcttctttttttcagggattttagggaaaataaggctcttgtcatttttttattctattactgaacagaatgggaagacaggg
ttggttattcttcgtctacgaatatccaaattttaactcctaatactccatagatagttcgaattggatagcagcaataatcaattttagcgcgaattgtttggaggggaagt
ctacctttttgatgcattcggcacgcgcaatttcttttcccgcgagacggcctgcaatttttactttactcccttatatctgttttttagttaattcaatggctttttcattgc
ctttcggaatgaaactctatttttaattggaaagctatatattctgcaagaatgttaggttgtctataaggttctttcacttttcgatcccaatattaagtctctggtttacaga
attaacttctttttgtagatctttctctaattcttcgattgctcctttttttctttaataaattggggaatccaatatggattatgacgtggattgtatcgatttcttttgaatttctata
tgtgtaattacttcggaacttgagcctgagtccatttttttattatgtgtaattacttcgaaacttgagtctgcttctattttctattcgagccctttttcctattcttttgtatatagt
tcttgatacaattccgtattttttatcttcctgtagaccttcagaataattttttggttgtgcgaaccaaaaggaatggtgattttgggttgtaccaagtctgaaaccaagtg
gatttatttttgtcccatatttttctattctattttttttaccgggaatcaaatcttagatggatctaaagattatttcgatttctttactatatttagtacaattgttatatgacacat
ggttttttttatgggagaactacgtcctcgagcccgaggtctgaatttattcataatagtactcctactgacttcggctttagtgatgaataaatttgctttgtcgaaatccct
ataatgagtagcatttgctgctgccgaataaaccaactttaagatgggataagatgctcgataaggcatgaggttcaatatcataacagtttcttcgtagtaacgccaa
cgaatctcatccagaactctttgtactttgaaaacggacatgtggatgcgtttttgtgctttgaaaacccgttcgaacttaagtagacgatcggttggtacttttcttgcga
gtttccttagcccactcttcttcttctttatcctaggggtatactttaccagtttgaaacttgtcataaataaggttattccccgcttacctatttcttttttttttttatttttgaatctttc
tattctgaattcagttaacgacgagatttagtatcctttcttgcactttcataactcgtgaaatgccgagttggtacgaattcccccaatttgcgacctaccataggatttgtt
atgtaaataggtatatgttcctttccattatgaatcgcgattgtatggccaaccattgcgggtagaatgctagatgcccgggaccacgttactattgtttctttctcctcctt
catattgaccttttctatttttgccaataaatgatgagctacaaaaggattcgttttttttcgtgtcacagctgattactcctttttttccatttaaagagtggcattcgatgtcc
aatatctcgatcgaagtatggaggtcagaataaatagaataatgatgaatggaaaaaaagagaaaatcctttagctggataaggggcggatgtagccaagtggatca
aggcagtggattgtgaatccaccatgcgcgggttcaattcccgtcgttcgcccatcccattattgcaaattccaaaaatgcaattttccatattcctagttacgtatttactt
acggcgacgaagaataaaactatcactatatttttttcctttttcctagttcttcttccaagcgcaggataaccccaaggggttgtgggtttttttctaccaatgggggctttc
ccttcaccgcccccatgggggtggtccacagggttcataactaccccctcttactacggggcgtttacctagccaacacttagacccggctctacccaaactttttggt
tcaccccaacattacccacttgtccgactgttgctaagcagttttgggataccaaacggacctccccagatggtaatcttaaagtggccgatttaccttcttttgcaatca
gtttcgctacagcacctgctgctctagctaattgcccaccccttccacgtgtgatttctatgttatgtatggccgtgcctaagggcatatcggttgaagtagattcttctttt
ctctcaaaaaaccccttcccaaactgtacaagcttcttccaaagcatacggctttctagatgtatatgacgatctctagacagatggatcttatatgaatcgtatgatgaa
gtaccacatgagtggatatataggaaaggaatccaaatctgccgaatcgctcatgttatgatcttctacatcctaggtctccgcgttccgtcatctggcttatgttcttcat
gtagcattcagatcgaatgactctatgaaattacgtcgatacttctacatattatgggtaacgtaggagacatccctattttcccccgggggtcttaattaccactgctta
gctttcaattcgcctctgaccatcaaattaaatgtgaataacccgtcctcctctctttgaaacaaggggcgcttccggttctgtgcgtgcttcaaacaattttgtcttctcca
tattaccatatctctagagtcaataattttctatgaggaactactgaactcaatcacttgctgccgttactcaacagttttctgttgaggtctatcccgtagaggtagtcaaa
ttggatcagtgatcgatttctaggtttcgtcgtaaacctaattggttacttccaattacgtaaatcaatagttcaaaccgcactcaaaggtagggcatttcccattgatata
ggaacttttgtaccagaaacaatagtatctccaattatagcccctctgggatgtaaaatatatcccttctcaccatccccatagtgtatgagacaaatgtacgcatttcga
ttagggtcgtattctatggttacgattctaccagatatgtctttttgattccgtcgaaaatcgattttacggtataggcgcttatgacctccccctctatgccttgcggtaatg
attcctctggaattacgacctttaccacaacggtgccgtccatggatcaaattatttcgtggattggatttcacttgcctatctatggttcccttgcgtgtgctcgggatag
gtgttttgtataaatgtttcgccgtattattaagtattctcctttagttttttttctctatctagaagtggaatagaataacccggttgaagggtaatgatcatacgtctgtaatgc
attgtatgtcctagaatagggcccattcttctaccctttccgggtagtcgatggctattcacagctactaccttaacaccaaagaagagttcgacccaatgctttatttctg
tcttagtgaatcccgattcgacattaaaagtatattgattctttcccaataaacgaagactttttttctgtaaatactgcgtatttgattccatcccataaatcgactttccctccta
tgctctgagttccagtatcgataagaattcgagttcttattgttcttatgttatggtatgaatataccataccaattcgttatgtatggatgatggatgagattccatggatag
agagccagttccaatagacttatggaacgttccggttcgcgtgcatccagcaggaattgaacccgcaaatttaccaattatgagttgggcgctttaaccattcagccat
ggatgcttaacagggatcatcgtacatcgtaaataaccaattttcatatagaaagacatatcatagaaaaatgaaatcgaaaatattcggagatggcaaatattcggag
atgactatgaaaacacctctctggatcctcgaattgaaagagagattgagagggatccagaatcctaattctcgctatttggaatggatccaattctattgagtctgact
catagtgatcatttctctttagcaaagaatgaccttggttatcaaaggattgaacaaccgggatccgtttacttatgatacctagttgacattgataacaaggatctaatga
attatgagtttaatagatcctctttagcagaaagacgtatattccttgctcatcacttattcccaaacctcgtatggggctaatcgttttcatttaccatctcagggaaaatcc
ttttcgttccgcttagccctatcgggtattttagtgataggttctataggaactggacgatcctatttggtcaaatacctaatgaaaaattcctattttcctttcattaaggtac
gagggcttcttattccacaagaaaagaacgaaagcaccttttcattctttcatatactgggggtttttacttggaaaagacaatgttccatactaaaggattcgggtccat
aaccacgagttccagtgcactagatcttgtagcacttagcaacgaggccctatgaatagacatatagaatttttggtcgggaaattcgaatgaatcattgagtgaaaaa
ggagcaaagaatgacaaaagacgagactctactagtcttcactcttgtggtttcctcggtttctgttttcttattcgggatcttgcttttcatggttctcatctctgcaactcg
cgattttcgcgagagaaccaaatccaagttggtgaagatcatgatttgggctggcatagtagtgattacctttgcaattgcggttcgaatctatccgatctttatctttttg
ctcaaagaacgaataaaaccccttgtcgaagccctttatgataagcttccctggatctgggaagtttctctttcacggtattgggatcgtttgatcgatttccttgatcgct
acttatgggcgtgcgctcaaaggatacaaacagggattcgcaaacaaaaaggggaattcgtagtcactttttcctgtcgcgtaaaaaaaaaggctttacgcgagagc

aatagaggttgggatacatctatctcttctgagcaacctcttttggattcttaagaccacccttgcagtaggataccgtctgctttgggttctttattatattatctccttcga
gggatttttaggatcgttcaggctatatttagtctattttggcttttactgtctccttttctcagggaagtggttaaggacctcagaagatagaggagagcgccaggcgca
gatttccggaatacttctacggggaatgctcattgaatgcgcattctccgtattatgccttgaagaggactcgaacctccacgctctttagcacgagattttgagtctcg
cgtgtctaccatttcaccatcaaggcatcttgaaagtgaatcatattccatgaatatgatatctatctaatgtgatatcttgaaagtgaatcgtattccatgaatatgatatct
atctaatgtgatatatggaatatatgacaaaggtggagtcttggagtatttcgatcgatcggtcatataggcctgagtcagacatcaaatagcttcgatttgcattatccg
taggacaccttatatgtatcaaaatcaaaaagatgtacaatccaatttctcgattcaatagaagcccaaagaggtgcatatggtacccaaataaggataggatagatat
gtcaaaagcaggtctgattacacctattcctaatcctaaatagaatgtaaggacgtggggatttctatgtaaacagagtatcctatttccataggctcgaatgacccctt
ctcataataagaatgtgcacggtctggtccggtatggaatgaacttataatctgatgatcgagtcgattccatgattataagttcattaccctagcgcccattcccattttg
ggcggaacagatctactaattcttttattccagttagtaagagggatcttgaactaagaaatagacctagcagctaaaagagggtatcctgagcaattgcaagaatgg
ggttcattgatattcctggtatagtagatgctatcacacatacagtcatactcaattcgatggaattgtttgatcttaaaggggatcttctataatttcgcacataaggggtt
atttcttggtttcgtccagtcattaataacttgactatttttagataatagtagatagaaagaacgctcgtaaggagtcctattgaaaccaagaaatataggcctgcttgcc
atccacaccagaatagatagagttttccgaagaaacctgctagtggaggaaggcctcctagggataagagacatagggctaaagagagagccaaaaaaggatct
ttcgtgtataatcctgcataatctcgaatgttatcagttccggtacgtagaccaaataatacaatgcaagcaaaagttcctagattcatggagatatagaaaagcatata
agttatcatgcttgcatatccatcatttgagtctccaacaattattccaataattacatatccgatttgccctatggacgaatatgcaagcatacgtttcatgcttgtttgagt
aatagcaaggagattccccaatatcatgctaagaatagctaggatttccagaagaagatgccattcgtttgatgagaaataaaaaggaatatcgagaattcgcgtgg
ctgaagctgaagcagcaactttcgaagtaacagaaagaaaagcaacgactggagtgggggagtcagagtcgaaaagaggattcctcgcttctttctctcatgcaa
aaccgtgcatgagactttcatctcgcacggctcctaagtgataaaagaaagaagaacttgtcttctttctttttgattaccttcctcgcgtatgtataagaccgaatccatt
ctttttcgaaatcgatttcgaaaaagaactactaatccttaacttttcgaggaatccttcatcagtggttgtgaatgactgactttttcaatcctttcgaccttggttccgtag
gagcaagtcagaaaggttgagaaatagaaccatctgatttgattcgttcccaatagccatgagatgatcatcttagggtgatccttttgtcaacggatgctcctattaca
ctcgtagtctctgaaggatgagaacccactatgtagcatctacgtcgataattcaagcattgtatacgtcattagtccgattctttgtaggaactacccgtaataacgag
cttgcaaaatggatctgtttatcataaagagattcgttgttcctgaccctgcttcaccttaattgttatttgaacaaaaagatcacaataaacttttggtaaaagttctgtcttg
gtcggagtggggatagcatttctcttctgcatatctatggagttttgcaaaacccaaacacctcagagatagatatagaggtaggaatttgtcgaacgaaccacactc
cttcgtagacgtcaggagtccattgatgaaaaggggctggggaaagcttgaacccaagtcctacagtgatggatataagcgcaattgaaattcctggggagttatac
atttgtgtattgataagaccgttcacaatttcttgaagctcgatctccccccagatgaaccatatagccaagagaaaccatgaaccagaatagaagagcttgcccca
cccatgagtaaatatttcatagtagcctcattagaccgtagatctctcttggtatatccagacaataggtaggaacataaactgaaacattctggagctacaaagatagt
tattaaatcgttagcaccacataaaaacattcccctagagtagctgttaatacgaataacagaaactctgttatagccatttctgtacattcaatgtactctacggataga
ggaatacataaagttgaacataataaaataagaaattgaaagatttcgttgaaattgttcgtttggaaatttcccgaaaagctaattataggttcttctctccatcggaaca
atagggccgttatgcttattactaaacttgttgaagagatgaaatagaaccaaggtctatctttttgatcagaggttaaatcgatcatcagaagaagaattaggccaaaa
attaggatacattctgggaaaatgaaacttccctggaagagaagcaaatgaaacgctttcataaaaattctcgtagaatcgagaatgaagttttcattctgtacatgcca
gatcatgaattagtaactgcatccaatctccgaaaagtcccgattgtttcgatttttggaatgggatatttacggaatccccatgaataggatcaaaccttattccatgcta
tttccataagattcctctttcttattcttaagcaagcccccgagagggcttagttgatcatgatttctgttttctctttcttttcctttttgtttgtttcgagaaagatatcgtccgat
tctccttctattgattcttttccgatcgagatgtatggatccatgtgtctacatacctagattctgttcatggattaacgaaaatgtgcaagagctctatttgcctctgccattc
tatgagtcgcttcctttttgcgtatggcacccccacccccctttggcagcatctactaattcggaacttaatttgaaagccatatttcgacccggacgcttttgggatgcttc
taataaccaacgaatggcaagtgctcttccttgtttagatcctatttcaatcggaactttccgcgtcgatccttttttattacgtcttgtttttactcctatattgggagttactct
acgtattgcttgacgtaaaaccaatagtggatttgtttctgtcttttgttgaatctttttcacggctcgatagagaatttgataagccaatgatttttttccgtctttcataatac
ggttaaccaccatgttaactaatcgattacgaaaaattggatcggattttgcagttcttttttctgcagtacctcgacgtgacatgagcgtgaaagaggttcaagaatcc
gttttctttttataagggctaaaaacgaatcacttattttttggctttttgaccccatattgtagggtggatctcgaaagataggaaagatctccctccaagccgtacatac
aactttcatcgaatacggctttccacagaattctatagggatctatgagatcgagtatggaattcagtttactcacttaaaattgagtatccgtttccctccttttcccgcta
ggatcggaaatcctgtattttccatatccataccatcaagtccttaggtttccgaaatagtgtaatggaaaaagaagtgcttcgaatcattgctatttgactcggacctgtt
ctgaaaaagtcgaggtatttcgaattgtttgttgacacggacaaagtaagggaaaacccctgaaagaatttccatattgaccttggacatataagagttccgaatcgaa
tctctttagaaagaagatcttttgtctcatggtagcctgctccagtccccttacgaaactttcgttattgggttagccatacacttcacatgtttctagcgattcacatggcat
catcaaatgatacaagtcttggataagaatctacaacgcactagaacgcccttgttgacgattctttactgcgacagcatctagggctcctcgaataatgcgatatctc
acaccgggtaaatccttaacccttcctcctcttactaatactacagaatgttcttgtaaattatggccaataccaggtatataagcagtgatttcaaatccagaggttaatc
gtactctggcaactttacgtaaggcagagttgggtttttttggggttgatagtggaaaagtcgacagataagtcacccttcctgtccctctacagaaccgtacatgagatt
ttcacctcatacggctcctcgttcaattctttcgaagggatccttttcctcgttcgagagtctccgcccttcttccactccgtcccgaagactaactaagaccaattgagtc
acgttttcatgttctaattgaacactttccatttatgattaaaggagaagattgttcttttaccaaacatatgcggatcaaatcacgtcttataataagaagaaatctttctcg
gtatcaatccccttgcccctcattctttgagaatcagaaggatccttttcgagtttccatttcttcatttggaatctgggctcttctatcttcgacttattttttttttgctttattctt
tatttatttcatttcgatttttccctcttcctctatccctatcctctaggtacagcgtttgcatcaatagagaaccttttcctctgtatgaatctatattattccattccaatttcttcc
cgaaacttcccaagaaaaatcccgaattggatccaaaattgacgggttaatgtgagcttatccatgcggttaggcactcttcaaataggaatccattttctaactggctt
tcgtgctttggtgagtcgtccgagatcctttcgatgacctatgttgtgttgaagggatatctatatgatccgatcgattgcataagacccgcggtagcaatagaacggg
gaaagtatacagaaaagacagttcttttcaatttcgattatctatatattagttcgtttctatttctagatatctatttctatatattagtattagttagtagtactattctattagtta
gcgatcccggctctgtgagttctttcttccgtgatgaactgtcggcaccagtcctccatttttttctctgtggaccgaggagaaagggggctcagcaggaagaggattg
taccatgagagaagcacagaggtcaaccctcttcaaatatggaacatggattctggcaatgcaacgtagttgggtcctcatatcgatccgaatgaatcagtctttctac
agaggtcaatctttgcctattaggcaagaggatagcaagttcgaaattctgtctcggtaggacatggatttctattactatgaaattcataaatgaaaatgaagtagttaa
tgggagggctaccattatcctttttcttgtatgtgttcctaagagaaggaatttgtccatttcatgtttcgaggtctcaaaaaagggcgtggaaacagatagaaactcttg
aatggaaattgaaaagaaatgtagccccagttccttcggaaatggtaagatctttggcgcaagaagaaggggcgacccatatcatcttgacttggttctgcttcccct

cttttttaagaataccgagtcgggttcttctcctaccagtatcgaatagaacatgctgaacaagatcttcttcatggaaacctgctcgatttagatcgggaaaatcgtac
agattttatgaaaccatgtgctatggctcgaatccatagtcaatcctattttcgataggaccggttgacaattgaatccaatttttcccattatttgactatccataatagtgt
ggaaagaaagcccggaggaagggtggccttgagtttctcgcccctttgccttaggattcgttaattctctttctcgaggggacggggaagggatataactcagcggt
agagtgtcaccttgacgtggtggaagtcatcagttcgagcctgattatccctaaacccaatgtgagtttttctattttgacttactccccccgccacgatcgaacggga
atggataagaggcttgtgggattgacgtgatagggtagggttggctatactgctggtggcgaactccaggctaataatctgaagcgcatggatacaagttatccttg
gaaggaaagacaattccgaatccgctttgtctacgaataaggaagctataagtaatgcaactatgaatctcatggagagttcgatcctggctcaggatgaacgctgg
cggcatgcttaacacatgcaagtcgaacgggaagtggtgtttccagtggcgaacgggtgagtaacgcgtaagaacctgcccttgggaggggaacaacaactgg
aaacggttgctaataccccgtaggctgaggagcaaaaggagaaatccgcccaaggaggggctcgcgtctgattagctagttggtgaggcaatagcttaccaagg
cgatgatcagtagctggtccgagaggatgatcagccacactgggactgagacacggcccagactcctacgggaggcagcagtggggaattttccgcaatgggc
gaaagcctgacggagcaatgccgcgtggaggtggaaggcccacgggtcgtcaacttcttttctcggagaagaaacaatgacggtatctgaggaataagcatcgg
ctaactctgtgccagcagccgcggtaagacagaggatgcaagcgttatccggaatgattgggcgtaaagcgtctgtaggtggcttttcaagtccgccgtcaaatcc
cagggctcaaccctggacaggcggtggaaactaccaagctggagtacggtaggggcagagggaatttccggtggagcggtgaaatgcattgagatcggaaag
aacaccaacggcgaaagcactctgctgggccgacactgacactgagagacgaaagctaggggagcaaatgggattagagaccccagtagtcctagccgtaaa
cgatggatactaggtgctgtgcgactcgacccgtgcagtgctgtagctaacgcgttaagtatcccgcctggggagtacgttcgcaagaatgaaactcaaaggaatt
gacgggggcccgcacaagcggtggagcatgtggtttaattcgatgcaaagcgaagaaccttaccagggcttgacatgccgcgaatcctcttgaaagagaggggt
gccctcgggaacgcggacacaggtggtgcatggctgtcgtcagctcgtgccgtaaggtgttgggttaagtctcgcaacgagcgcaaccctcgtgtttagttgccac
tatgagtttggaaccctgaacagaccgccggtgttaagccggaggaaggagaggatgaggccaagtcatcatgccccttatgccctgggcgacacacgtgctac
aatgggcgggacaaagggtcgcgatctcgcgagggtgagctaactccaaaaacccgtcctcagttcggattgcaggctgcaactcgcctgcatgaagcaggaat
cgctagtaatcgccggtcagccatacggcggtgaatccgttcccgggccttgtacacaccgcccgtcacactataggagctggccatgtttgaagtcattacccta
accgtaaggagggggatgcctaaggctaggcttgcgactggagtgaagtcgtaacaaggtagccgtactggaaggtgcggctggatcacctccttttcagggag
agctaatgcttatgcttattgggtattttggtttgacactgcttcacgcccaaaaagaaggcagctacgtctgagctaaacttggatatggaagtcttctttcgtttagggt
gaagtaagaccaagctcatgagcttattatcctaggtcggaacaaattagttgatagtgataggatcccctttttgacgtccccatgcccccccccgtgtggtgtggcg
gcatggggatgtcaaaaggaaagggatggagtttttctcgcttttggcgtagcaggcctccctttgggaggcccgcgcgacgggctattagctcagtggtagagcg
cgcccctgataattgcgtcgttgtgcctgggctgtgagggctctcagccacatggatagttcaatgtgctcatcagcgcctgacccgaagatgtggatcatccaagg
cacattagcatggcgtactcctcctgtttgaatcggagtttgaaaccaaacaaacttctcctcaggaggatagatggggcgattcaggtgagatcccatgtagatcta
actttctattcactcgtgggatccgggcggtccgggggggggcactacggctcctctcttctcgagaatccatacatcccttatcagtgtatggagagctatctctcgag
cacaggttgaggttcgtcctcaatgggaaaatggagcacctaacaacgcatcttcacagaccaagaactacgagatcaccctttcattctggggtgacggagggat
cgtaccattcgagcctttttttcatgcttttcccggcggtctggagaaagcagcaatcaataggacttccctaatcctcccttcctgaaaggaagaacgtgaaattcttttt
cctttccgcagggaccaggaggttggatctagccataagaggaatgcttggtataaataagccacttcttggtcttcgactccctaagtcactacgagcgccctcgat
cagtgcaatgggatgtggctatttatctatctcttgactcgaaatgggagcagagcaggtttgaaaaaggatcttagagtgtctagggttgggccaggagggtctctt
aacgccttcctttttctgcccatcggagttatttcccaaggacttgccatggtaagggggagaagggggaagaagcacacttgaagagcgcagtacaacggagagtt
gtatgctgcgttcgggaaggatgaatcgctcccgaaaaggagtctattgattctctcccaattggttggatcgtaggggcgatgatttacttcacgggcgaggtctct
ggttcaagtccaggatggcccagctgcgccagggaaaagaatagaagaagcatctgactctttcatgcatactccacttggctcgggggggatatagctcagttggt
agagctccgctcttgcaattgggtcgttgcgattacgggttggctgtctaattgtccaggcggtaatggtagtatcttgtacctgaaccggtggctcactttttctaagta
atggggaagaggactgaaacatgccactgaaagactctactgagacaaaaagatgggctgtcaaaaaggtagaggaggtaggatgggcagttggtcagatcta
gtatggatcgtacatggacgatagttggagtcggcggctctcctaggcttccctcatctgggatccctggggaagaggatcaagttggcccttgcgaatagcttgat
gcactatctcccttcaaccctttgagcgaaatgtggcaaaaggaaggaaaatccatggaccgaccccattatctccaccccgtaggaactacgagatcaccccaag
gacgccttcggcgtccaggggtcacggaccgaccatagaccctgttcaataagtggaacacattagccgtccgctctccggttgggcagtaagggtcggagaag
ggcaatcactcgttcttaaaaccagcattcttaagtttaagatcaaagagtcgggcggaaaaaggggagagctccccgttcctggttctcctgtagctggattccccg
gaaccacaagaatccttagaatgggattccaactcagcaccttttgttttgagattttgagaagagttgctctttggagagcacagtacgatgaaagttgtaagctgtgt
tcgggggggagttattgtctatcgttggcctctatggtagaacccgtcggggaggcctgagaggcggtggtttaccctgtggcggatgtcagcggttcgagtccgc
ttatctccagcccgtgaacttagcggatactatgatagcaccgaattttgccaattcggcagttcgatctatgatttcgcattcatggacgttgataagatccttccattta
gtagcaccttaggatggcatagccttaacgttaatggcgaggttcaaaagaggaaaggcttgcggtggatacctaggtacccagagacgaggaagggcgtagca
agcgacgaaatgcttcggggagttgaaaataagcatagatccggagattcccaaataggtcaaccttttgaactgcctgctgaatccatgagcaggcaagagacaa
cctggcgaactgaaacatcttagtagccagaggaaaagaaagcaaaagcgattcccgtagtagcggcgagcgaaatgggagcagcctaaaccgtgaaaacgg
ggttgtgggagagcaatacaagcgttgtgctgctaggcgaagcggttgagtgccgcaccctagatggctaaagtccagtagccgaaagcatcactagcttacgct
ctgacccgagtagcatggggcacgtggaatcccgtgtgaatcagcaaggaccaccttgcaaggctaaatactcctgggtgaccgatagcgaagtagtaccgtga
gggaaaggtgaaaagaaccccagtgggtagtgaaatagaacgtgaaaccgtgctgagctcccaagcagtgggaggggaaagtgatctctgaccgcgtgcctg
ttgaagaatgagccggcgactcataggcagtggcttggttaagggaacggaacccaccggagccgtagcgaaagcgagtcttcatagggcgattgtcactgctta
tggacccgaacctgggtgatctatccatgaccaggatgaagcttggatgaaactaagcagaggtccgaaccgactgatgttgaagaatcagcggatgagttgtggt
taggggtgaaatgccactcgaacccagagctagctggttctccccgaaatgcgttgaggcgcagcagttgactggacatctaggggtaaagcactgtttcggtgc
gggctgcgcgagcggtaccaaatcgaggcaaactctgaatactagatatgacccaaaaataacaggggtcaaggtcggccagtgagacgatgggggataagct
tcatcgtcgagagggaaacagcccggatcaccagctaaggcccctaaatgaccgctcagtgataaaggaggtgggggtgcaaagacagccaggaggtttgcct
agaagcagccaccctttaaagagtgcgtaatagctcactgatcgagcgcccttgcgctgaagatgaacggggctaagcgatctgccgaagctgtgggatgtcaaa
atgcatcggtaggggagcgttccgccttagagggaagcaaccgcgaaagcgggggtcgacgaagcggaagcgagaatgtcggcttgagtaacgaaaacattg
gtgagaatccaatgccccgaaaacccaaggtttcctccgcaaggttcgtccacggagggtgagtcagggcctaagatcaggccgaaaggcgtagtcgatggac

aacaggtcaatattcctgtactaccccttgttggtacggagggacggaggaggctaggttagccgaaagatggttataggtttaaggacacaaggtgaccctgctttt
tcagggtaagaaggggtagagaaaatgcctcgagccgaggtccgagtaccaagcgctgcagcgctgaagtatgagccccgtggactagccattgcttctccacg
aggctcataccaggcgctacggcgctgaagtatgtaacccatgccatactcccaggaaaagctcgaacgaccttcaacaaaggggtacctgtacccgaaaccga
cacaggtgggtaggtagagaatacctaggggcgcgagacaactctctctaaggaactcggcaaaatagccccgtaacttcgggagaaggggtgccccctcgca
aaagggggtcgcagtgaccaggcccgggcgactgtttaccaaaaacacaggtctccgcaaagtcgtaagaccatgtatgggggctgacgcctgcccagtgccg
gaaggtcaaggaagttggtgaactgatgacagggaagccggcgaccgaagccccggtgaacggcggccgtaactataacggtcctaaggtagcgaaattcctt
gtcgggtaagttccgacccgcacgaaaggcgtaacgatctgggcactgtctcggagagagactcggtgaaatagacatgtctgtgaagatgcggactacctgca
cctggacagaaagaccctatgaagctttactgttccctgggattggctttgggcctttcctgcgcagcttaggtggaaggcgaagaaggccccttccgggggggc
ccgagccatcagtgagataccactctggaagagctcggattctaaccttgtgtcagacccgcgggccaagggacagtctcaggtagacagtttctatggggcgta
ggcctcccaaaaggtaacggaggcgtgcaaaggtttcctcgggccagacggacattggtcctcgagtgcaaaggcagaagggagcttgactgcaagactcacc
cgtcgagcagagacgaaagtcggccttagtgatccgacggtgccgagtggaagggccgtcgctcaacggataaaagttactctagggataacaggctgatcttc
cccaagagtccacatcgacgggaaggtttggcacctcgatgtcggctcttcgccacctggagctgtaggtggttccaagggttgggctgttcgcccattaatgcggt
acgtgagctgggttcagaacgtcgtgagacagttcggtccatatccggtgtgggcgttagagcattgagaggacctttccctagtacgagaggaccgggaaggac
gcacctctggtgtaccagttatcgtgcctacggtaaacgctgggtagccaagtgcggagaggataactgctgaaagcatataagtagtaagcccaccccaagatg
agtgctctctcctccgacttccctagagcctccggtagcacagccgagacagcgacgggttctccacccatacggggatggagcgacagaagcatggaaatagg
ataaggtagcggcgagacgagccgtttaaataggtgtcaagtggaagtgcagtgatgtatgcagctgaggcatcctaacgaacgaacgatttgaaccttgttcctac
acgacctgatcaaatcgatcaggcacttgccatctatcttcattgttcaactctttgatgaaaagatgaaaaaaccaaaaaaaagctctgcccttccatctcttggatag
atagagagggagggcagaggcctttggtgtcccttccagtcaagaattggggcttcacaattactagccaatatttctctcatgcctttcctcgttcatggttcgatattc
tggtgtcctaggcgtagaggaaccacaccaatccatcccgaatttggtggttaaactctactgcggtgacgatactgtaggggaggtcctgcggcaaaatagctcg
atgccagaatgataaaaagcttaacacctcttatttgactttttcactattttgaaatacgaaaaagatccaaatccaaaatgcaaaggtcgtcttattcaaaacctcaatc
atcacatcccctctctcccacttcacacctcggaacgcactgttcttatagagagaaaggggctttcccatcttcttaacccgaaatgaaatggctgaggagagggag
gttccttttggggggtaccccgggaagagatccagtggagacggggtgggcctgtagctcagaggattagagcacgtggctacgaaccacggtgtcgggggtt
cgaatccctcctcgcccacagccttccaaaggggaagggcctttactttcccccctgagggtaggaaaaccatgatcgggatagcggacgtaaagctattgaacttg
ggtatgctctttccttttgtcgaagtggaatcgtagaacagaatgtgatacgatgagataaaatgcaatagaaacaaggatagcgaacgggttacctactcctaaggg
tcaaagcaagccctttaattcaattctttattcttacattaaagaatgaatcaaatctccccaagtaggattcgaacctacgaccagtcagttaacagccgaccgctctac
cactgagctactgaggaacaaggggggattcgacctcctagagttcaactcccgctctcaacccatgaacaatatgagtccgaagcttctttcgtaactcccggaattt
cttcgtagtggctccgttccatgcctcatttcataggtaagcccagagtggctctatttcattctatttcacttcctagcacttcctatcatttaatatccatccctttggtctta
ttgacataagagatgtcatttatagtctatctctttctatatatggaaagtcaagaaattctcatcgaaacatcgagaaattgtgcatatagaaaactctaaagaaagaaa
aaaaggagacccatgccatgattttcaaatcttttctacttagtagtctaagtttctcgatgaggataattaattcggtcgttgcggtcggactctattatgggtttctgacc
acattctccatgggtccctcttagatcttctttctccaatcttggattagggaagaaggagatattcgcgactcctggtggtttcattatggggcagctcatgatcttcatat
cgatctattatccacctctgcatctattctttcttagctaaacgggtggaagatccatccaatttggttatatcatggactcaaaaaacggatctgaatgtgactgaaatgc
acgatcttcacaggtatcacttttcacgatacctaaaaggtggaatagcgattttcgaaccatttcctataagagaaaggtttccattactttgagaaatggattctatatc
aaactatagctattgcattaaagaagaaaagaaactaatagaagtcgaagacgcggaatggtagtgaatagagagaaagattcttctggttttcttgttcctgaaaata
ttctatctatctcctagacgccgtagagaattgagaattttcatgtctttcaattctcgtactcgtaattggaaagttacggaaggagatccatcattttgcaatgaaaacta
cataaaaaaactctggacaatttcgaaatcaggccaagcgtcttaatacatatgcaaaaaaaattcattattggcccaccattgattagaagatttaacttgtatgaatcgct
attggtttgatacgaataatggcagttgtttcagtatgttaaggatacagatgtatccacaattcatttagagttacttaatagcctatttcttataccatatctctatcccgtg
aaattctcgagccgaaagatggatgcatatgctatgtttcattttgctaaatgatatcaattaaacggtgtatcaattccataaattggatatagcaataaataaatcagca
aaattcttttattttagatagaagaaacttttcttctatctaaaataaaagaatgtacccttctatccaaatccaatttgcatcgataaaataaatccaaattccagtagtagat
gaataattgcaaatttttgtgtgtacgagattagaataacttcaaaataactgacataatttttatttttcctgatcagaaaaatacatgaaaaagaaaggaggtagaaaa
atttttggatttatggttaaagaagaaaaagaagaaaactggggttctgttgaatttcaagtattcagtttcaccaataagatacggagacttgcttcacatttggaattac
acaaaaaagatttttcatcggaaagaggtctccgaagacttttgggaaaacgtcaacgtttgctggcttatttggcaaagaaaaatagagtacgttataagaaattaat
cagtcagttggatattagggagcggtaatttcatcgttcgaattttttttcttattttattagtagtcttatagtagtcttagattttgcattttgatgagcctcgttttgaggaatt
catggaataatccattttcatggaataatgaattaaggaagaaaggatatgagtctaccgcttacaagaaaagatctcatgatagtcaatatgggccctcagcacccat
caatgcatggtgttcttcgactgatcgttactctcgatggtgaagatgttattgattgtgaacccatattaggctatttacacagaggaatggaaaaaatcggcaaatag
gaaagctacttaggcaggagataggggaattccttaagaaagaaaaaagaataagaacacagatacataacataaaaaaaaagaataaataagacgaaattcgacc
tccccctacatatttaatttcttctcctatacaaaaactagcaagacctactccattggtaattccatcaatgacacccttgtcgaaaaactgcgttagttcggttaatcctct
tatacccaaggtaaaggtcctagtatagaaaatatctatataaccgcgattatatgaccaactatatatctttttttttagttgatggaaaaaatactttttcggaccccctttt
acaaaggaatttattaaatccaaattctgaaaaaaagagtaagcagatccataaaacatatatgctatgaatagaccaaaaatagctagacttacagaagaaattgcat
tagtgataaattcatatgaatttatggaagaattagaactttcttggaaaaagttgattgagggagttagccactttgataatatggttaattcccctatttcattatcaaaat
ggattcctatagatccaataaacaaagtacaaagcagtaatataagaagaggaaatagcatagtatttcccggttcatgaggatagacaaaagtgtttttagtccccaa
tgaagtactaaaggaccctatcctatttcctgtattaacatgaattttggatagattttgtgaaaaaaaagaaactccactcttcgctgttgataaaacgaaatctctattga
ctcctttagatatcctttttccccataacgatattgaatacaacgaatcctctttagtactactgtaattttgaaaatgaacacgcaaatacccatcaaaagtaagtaaatat
atccgaaacatataaaacgcagttaatcctgcagtaaaagaagctattattccaaaaaagggtgaatataaccaactattactaaggatttcatctttggaccagaagc
aagcaagaggtggaataccacaaagagaaagggtaccccataaaaaacaagttcttgtaattggaatgtattttcttaaaccacccataagaaccatattctgactttt
atctggtgaatatccaacaagaggttccattgaatgaataacagatccggatcccaagaataataaagctttcgaataagcatgagtgatcaaatggaataaagcag
cttgataagaacctatacctagagctaacatcatataacccaattgagacattgtagaataggctaagcttcttttaatatctctctgagcaagagctaaagtggctccta

agaaaagtgttagtgtacctattaaagaaatgaaactcattatcaaaggtagggatatgaaaagaggaagaagtcgagctataagaaaaatccccgcagcaaccat
agttgctgcgtgtataagagctgaaatgggggtgggtccttccatagcatcgggtaaccatacgtgaagagggaattgtgccgatttcgcaactgcaccaaggaat
aataaaaaaagcacacaaaatagtaagcaaggagttaatctcattattaggaatccagttattagctattttaaacaaatcccgaaactctaaactacctgttatccaaaaa
aaacctagaattcctaataacagaccaaaatcccctacacgattagttacaaaagctttttgacaagcactcgctgcaattggccgtgtaaaccaaaagcctatcaata
aataggaacacattcccacaagctcccaaaaaaaataaatttgtatcaaattggaactagtaaccaatcccaacatggaagtattgaaaaaacttatataaacaaaaa
atctcaaatatccctcatcgtgagacatataatcatcactataaataagaaccaggattcctacagtagtaattagtattaacataatagaagtaagcgggtcgattaagt
atccaaattctaaggaaaaatcattattgacggtccaagaccatagatattgatagatagaacttccatttatttgttgaatagacaggtgaactgagaataccatagcta
tacttaaaagtaaaacactaggaaaagcccatatgcgacgaagatttttgttgctgttggaacaagaaaaagtccaaaccccattgacataataactggaagtggga
gaagagggattacccatgcatattgatatgtatgttccataagaaaagaaattgcaatttttacttgaaaattttacttcaatttttctataaaattgaaaaaagtttccgattc
accaaactaattcttatctatttctgaaggaataaaaaaatactagaattcttaatttttcaaaaattttctcattgaaacaatcaaaaaaataagaataggttttgttggttaaa
gtcaaaaagttaatgaaataacttcgttacctagttattacctaaagaaggacttttattaaaatacaaaaaaagattgaatcattttactttaatattttttgtattaaaatga
agcagctcccttgtttcgtaactcaaattgattggaattcaattctggaatactttaattaactatttgattgaattttcccttccttttatcctcccgtcttatatgggggatag
gcccccatacctatatctgtatatggagagtatacttgaaatataaattgatttaaatagaaaacctttgtatatattctatattattaaaacaaagtctaaaaaaaatatata
atatgttaaaaaactcttgtcttatccgcattagacaaaatgaagtaaaaaagaattcagaatttcaatatcttttagtatctaagtataaatactaagaaaaagaagaaaa
atggattgatttgcggcaatagatgtctttcacatacaactagaaaaaagtaatttccttttgaatggcagttccaaaaaaacgtacttcgatgtcaaaaaagcgtattc
gtaaaaatctttggaagaaaaagacttatttttccatagtacaatcttattctttagcaaaatcaagatcattttctggcgtcagcgagcatccaaaaccaaagggtttttct
cggcaacaaacaaacaaataatagggttttgggataatatgaattgacctatccccaaaaaattccaattatttaatatgaataattaggaataattaggattaattaatg
agtttacttttatgtgtcgaattcctcggtacaatattcttagaacaaacctctctgatatataaaaaaaagggtttttggtatactgtgacctaaatattcttttcctatcaatga
acttttcgtaatagaatccgtataataaataaaaaaaggggattctattatgaaaagtagagtattcctgcaataagacttacaacttctacctatcttatcctaaattaaca
aaaaaattagttctatattgcaactgagaaaaaaattgttccaactctttcaaagtttctattgggcaaagcaagaatttttttgttaaaaaaattcgcaacgatactaccaaac
gaagtctattttaatgaagattctaatgtcctaaattctatggaatcttccaatctcgacgattcgcgagaaaataacttaatattcttttattttaataaacctgttatttcaact
tagccgccatggtgaaattggtagacacgctgctcttaggaagcagtgctcaagcatctcggttcgagtccgagtggcggcagtctcgaaaaagaatacaatagat
tataaaataaaatggattcaattcgaaatttccaatttgtaatgggaccttctccttatgctatttgcaactttagaacatatactaactcatatttcttctcaacaatttcaat
tgtgattacgattcatttaataaccttattagttcgtgaacttggggggattgcgcgattcgtcagaaaaaggaatgatagccacttttttctgtataacaggattcttagtttc
tcgttgggcttcttcgggacattttccattaagtaatttatatgagtcattgatcttcctttcatgggctctgtatattcttcatatgattcctaagatacagaactctaaaaatg
atttaagcacaataactacgccgagtactattttaacgcaaggctttgccacgtcgggtcttttaactgaaatgcatcaatccacaatactagtacctgctctacaatctc
agtggttaatgatgcatgtcagtatgatgttactaagctatgcgactcttttgtgcggatccttattatccgccgctcttctaatgattagatttcgaaagaatttagatttctt
ttcgaaaaagaagaaaaatgttttgcttaaaacatttttctttaatgagattgaatatttctatgcaaaaagaagtgctttaaaaagcaccttttttcctttatttccaaattatta
caaatatcaattaattgagcgtttggattcttggagttatcgtgtcattagtctagggtttacccttttaaccataggtattctttgtggagcagtatgggctaatgaggcgt
ggggatcctactggaattgggatcctaaagaaacttgggcatttattacttggaccatatttgcaatttatttacatagtagaacaaatccaaattggaagggtacgaag
tcagcatttgtagcttccataggatttcttataatttggatctgttattttggtatcaatctattaggaataggtttacatagttatggttcatttacattacccatctaaatgatta
cataacataaaaccttaatgaaatggaaaaaacttccatttttgtgtttgatttgagaaccccttgaacgccttctcaaagggttctcaaaaattcgagatagatctaatta
gactcttttacttttttctgaatttttttagtatttccactatggaatatagagcggactagtagaagaaaaaaaatcctatttaggataataattggataacagagcctctacc
ctgtcaacggatagcgagagaacaaaatctggataaataccgattcctattactggtaaaaagatacagattaaaagaaagagttctcgcgggccggaatcctcaa
aattttcgtttggaacatgaaatagcttgtatccatagaacatctgtcgtaacatagataataaataaataggagttaatatcattccaattgccattacaaaagtaattagc
atttttggcattaacagaaattttggactagtaattagtccaaaaaatactactaattccgcaacaaaaccactcattcctggtaaggcaagagaagccattgaaaagct
actaaacatggtaaaaatttttggcattgggatagaaacccctcccagttcttcgagataaacaaggcgcattctatcacaagccgttcccgctaagaaaaaaagtgt
agccccaataaatccatgggataatatttgtaaaatagctccattgagtccaatgttggttatggaaccaattcctataataatgaaacccatgtgagagacggaggag
taggctattcttttttgaaattgcgttggccaagagaagttgaagctgcatagattatttgcatcgctcctattattactaaccaaggggaaaatagataatgagcatga
ggtaacaattccatattgatccgaatcaatccgtatgctcccatctttaataggattcccgctaaaagcatacatgtactgtaatgcgcttccccatgggtatctggtaac
cacgtatgtaggggtataatcggcaatttgacagcataagcaataaggaagccaaaataaaatagtatttccaatgttgcagggtatgattgattaattaatctttccaa
atctaatcttggttcgttggaaccgtataagcccatacctagaactccgattaagaaaaaaatggaaccacctgcagtatacaaaataaactttgtagctgaatagaga
cgcctcttcccccccacatggataaaagtaagtaaacaggaattaattctaactccccacatgataaaaaaaagtaaaaggtctcgcgaagaaaataatcctatttga
ccgctatacattgctagcatcaggaaatagaataatcgggaattccgggtaaccggccaagctgctaaagtagctaaagtagtcataaatcctgtcaataaaatagat
cctaatgaaagtccatcgattcccaatctccagtggaaattgaagacatctatccatttagaatcctcttttaattggattaagggatcctccaattggaaatgataacag
aatgcataagtcattagaaggaattctaataaacaaatagacatagtataccacctaacgattttgtttcccctatgaggtaaaaagaaaattaatgaacccgcaaatat
cggcaaaacaacaagtattgttaaccaaggaaaagaactcatgataaagtgataaagacaagatacgttttgaccagaaaagcccgtgctcgattattttgagcaca
ggcttcttcggtaaagaggaatcagacgattcaagtggaatttttgtaacgtatcaataagatagagccatgctgcgggttgtctcaggtcctaaataaacgcggac
acttaaaaaatctgttgggcaggcggattcgcatctcttacaacccacacaatcttcggttcttggcgcggaagcaatttgcttggctttacatcccatcccaaggtatca
tttctaatacatctgttggacaagctcgtacacattgagtgcatcctatacatgtatcataaattttttacggaatgtgacattggatctataaattttcctttcaacataaaaa
ttttcgatctggtcaaaatgaaattagtactatatcaatcaaatgtattgtagacaccagacgaagcaatggtttatccaaacttcaacaaataatgcaatatatttcttaat
ccgtttgtgagaaagcatgaaaagagccaagagactttaattttgggcttcaacaatcataattatacgaattgtatatacgaattcgaattagccaataaattggctatc
gtcttttcaatataaattattgcaatattcaaattgcaatatcaatgaattgcaaaaattcaactaagtaaaaaagaatactatggaataacctactcaaaaaatagatattc
tcaaataataaatagtattcatgttaatatttcatattattattatatgtgtccctttgtttagaagattctatgtctaattattcaaaaaattagattgattgatacgagttgatttc
ctattacgatggatggaagaaagaatggataatccaatagctgcttcagcagccgcaagggctataacaaaaattgcgaaaatgtctccttttaattggcggctatca
aatagatcagaaaatgttacgagatttagattaattgaattcagtataagttcaaggcatattagagctctaaccatgtttcggcttgtgatcaatccatagataccaatcg

aaaataaatagacactcaaaaaaagtacatgctcaaacatcattaactaactccttatcaatctcgattcatttcaatatggggacaagaattgaaccgattgaattaatt
agaatagaacaattacacaacaaaagagaaaagaaggtatttgttggcagtagatgggttttactaaatcaaaattgtggttctttagtgatttattttagatttgaaattct
tataattttgactcattctaagtatttcttattgccgagccatagtaattgcacctattaaagaaactagaagaattatggaaatgagttcaaatggaagataaaaatcggtt
gctaaatgaatcccaatttgttgaacgttatttatgagaccctgttctactatttggtttgatcttgtagtccaaagaattccataccatgacgtatctgggatagtagtcatt
agtgaaaaaggaatagttatacaaacgagtgaagtgaacccatctccaatagtccaaaaattcttatctttagaccattctgagccatttacgaacattacggcaaatat
gatcaagacatttatagctcccacataaataagaagttgtgccacagctacaaagtaggaattcaataaaatatagaataaggatatacaaacaagaactaatcctag
cgaaaaagcagaaaaagttgggttggtaagtaataccaccccctagacccccctagtagaagaacaaatcccccaaatagcacaagaatttcatgtattggcccaggt
aaatccattatggataagaagaaattaatagtataaattttttcatgaactgactaaaactaaaagattcaaggaagaaaaaagggattaggaaattttttgtatattgtat
ataagttctttctatagttagaaatcacatcacgaaaatctactctggtttaaaatcaggaataatttgcaataagcagtaggtattcgtttttctttctagttagtaaagaact
tttggattctaacaaaaaaattctagtaatcagtaatcgttcttgaattccaagatttttcttcgtctattttactttgagttgaattcctaattgtttgaattgtgtaatctcccatt
atggagattggtaaccgactcaaagcaatttgattgtaattcaattcatgacgatcataagtagaaagttcatattcttcagtcattgataaacagtttgtcggacagtact
caacacaattaccacaaaatatacaaactccgaaatcaatactataattaagcaattgtttccttttaatatccttttcaaatctccaatccacaagaggtagatctatagg
gcatacgcgaacacatacttcacaagcaatacatttatcaaattcaaagtggattcgcccccggaaacgctccgatgtaattgatttttcataggggtagtgaatcgtta
taggtaaacgatttgtgtgggataaggtaattatgaaactttgaccaatgtaccttgctgcgcgtattgtttgttgaccataactcatgaacccagttaccatagggaaca
tattcgaaatatctatgaaaaaggtatgtttctttctcttgtttgagaggacttttgtgttgaaaatattcttactgttattgtattatcttattttatagtgaaacaagttgggaag
aagttgttaataagagattgcccagggaaataggtaaaagaaatttccatccaagatttaataactgatccattctcatcctgggtaaagtccatcttattgtgatagaaa
tgaagagaaataaataagctttagttaatgtaataaagatactcattgtcatttctaaaattctaaccattttattcatttggaaaaatccaaaaaaggatatatagggaata
gacaaattccacccgcctaagtagagaactgttacaaataaagaggaaactaataaatttaggtaagaaacaagataaaataaaccatatttgataccagaatattcg
gtttgataacctgctactaattcttcctccgcttctggtaaatcaaagggtaatctttcacattccgccaaagaagaaattagaaaaaccagaaaacctataggctgacg
ccaaagattccatccaaaaaaaccatattttgactgtgcttcaactatatcaactgtacttgaactgttggataatcatagtcgacgataacatcacagttcccaccgcta
ttccaaaaccgtacatgaaaccttagtttcatacggctcctctatgatcagaaaaaaggaaagtactgtttcatttcgttattatcttcttgggcgtagttagaattatctaa
gataaaatcgatttcaacgtcctaaattagaccaaaggaattctgtctgctagaataataaaaaacgcttcggaattcatctcatcctttataatataatggtactttttcttt
gttcagcaataacttaatcttggaataaaacactcgttataacaattaataaacgaaaagagttgggtattagttcatgaagaattctgtatgaatatggataaacgacg
gaaagaataaataaagatcttttttttgtattgcattccatatcttttgtcctattcttctttccccgaggggtatttaaaaagaaaaaaggaataaagggttaattcgttcttg
atagccatttccttaacaagtgaatgggaacatactctggatcggaatccgaagaaagtactacttgctcatttccaccaatttcaagtccttattatgattccttttatgag
gaaaaatatctaatgctttagattccctcattactaatcctttatgtactttagtgtttctaatccctcactaacttttgacggattcccttatgattacaactttctgtatcggga
atcccttattattgcccgcttcaagatatgatgactaatcaaaaaatctcaaccttggggtaaagaatttacaccgcttatgtttacttccatttttttcttgtacataggaaat
gagatttttttctttttactacaaattaataagcagttttgtttcactcatatagctatctagtttaacttactaacctgaatatagaataagaaaaggaggataaatattcaatg
aatttcagaggaaaaagatcctattttaacgaatcgcacgtagagatattgctagtacacaaaaagttaatgggatttcataactaatagattgagcggcagctcgtag
accacctgaaaaagaatatttattatttgagctatatcctgccataagaagaccaataggagcaatacttgaaatggcaatccataaaaaaacaccaatactaagatcc
gctaaaacaaagcgatatcccaaaggaataactaaaaaacttaataaaattgatatgactgctatagacggtccaatgctaaataagggaatatcccctcgggatgg
caagatatcctctttaaaaagtagcttagttccatctgctatagcttgaagcagtcccagggggccagcatattcaggaccaatacgttgttgtatcgatgcggatattt
ctctttctaaccacacaattacgagtacttctattgtgattcccagtaagagggtcaaaatgggtagaatccatatcagtccatagacttcttttaataattccaagttcga
aaaagaattgatagcttctacctgtaccctgtctattatcatttcaacgatcaacttctcccataatgatatctatactacctaatatcgtcatgatatcagccaatttcattttt
ttgactagctgaggaagaatttgcaaattaataaacccgggtggacgaattttccatctccaggggaaaagactatcatctcctaccagataaattcctaattcacctttt
ggggcttccactcttgcataaagctcttgttttgacaattcaaaattgggtgaaggtttttttaccaagaaatcgatattcaaaatcattccattcggaattctttgctttcttaa
agcgtcggacttctaaattctcataagggcccccaggaatttttttctacagcctgttgaataattttgattgattccctcatttcaccgattcgtactaaatagcgtgctaat
gaatccccttctttttgccattggactttccaatcgaattgattgtaagactcgtaaagatcaactttacgaagatcccattgtattccagaagctcgtaacatggggccc
gataagccccaatttacagcttcttctccgctaataaaaccaactccctcaactcgttccaaaaaaaatgggattctgtgtaataagttgttgatattcaataactcctcgta
aaaaataatcacagaaatctaaacatttatcgatccatccataaggcagatcggcagcaactcctccgatgcgaaagtaattatgcatcattcgcatacctgtagcag
cttcaaatagatcatatattaattctctctctctaaaaatataaaaaaaaggagtctgcgcaccgagatctgccataaaaggtccaagccataacaagtgagaagctat
acggctcaattctaacataattaccctaatatagctggctctttggggtatttgaatattctccaagaattctggtgcatttaccgttattgcttctgtaaacatagtagctaa
ataatcccaacgtgttacataaggtaagtattgtataatagttcggttttccgcgatttttttccattcctctgtgtaaatagcctaatatgggttcacaatcaataacatcttca
ccatcgagagtaacgatcagtcgaagaacaccatgcattgatgggtgctgagggcccatattgactatcatgagatcttttcttgtaagcggtagactcatatcctttct
tccttaattcattattccatgaaaatggattattccatgaattcctcaaaacgaggctcatcaaaatgcaaaatctaagactactataagactactaataaaataagaaaa
aaaattcgaacgatgaaattaccgctccctaatatccaactgactgattaatttcttataacgtactctatttttctttgccaaataagccagcaaacgttgacgttttccca
aaagtcttcggagacctctttccgatgaaaaatctttttttgtgtaattccaaatgtgaagcaagtctccgtatcttattggtgaaactgaatacttgaaattcaacagaacc
ccagttttcttctttttcttctttaaccataaatccaaaaatttttctacctcctttctttttcatgtatttttctgatcaggaaaaataaaaaattatgtcagttattttgaagttattct
aatctcgtacacacaaaaatttgcaattattcatctactactggaatttggatttattttatcgatgcaaattggatttggatagaagggtacattcttttattttagatagaag
aaaagtttcttctatctaaaataaaagaattttgctgatttatttattgctatatccaatttatggaattgatacaccgtttaattgatatcatttagcaaaatgaaacatagcat
atgcatccatctttcggctcgagaatttcacgggatagagatatggtataagaaataggctattaagtaactctaaatgaattgtggatacatctgtatccttaacatact
gaaacaactgccattattcgtatcaaaccaatagcgattcatacaagttaaatcttctaatcaatggtgggccaataatgaatttttttgcatatgtattaagacgcttggc
ctgatttcgaaattgtccagagtttttttatgtagttttcattgcaaaatgatggatctccttccgtaactttccaattacgagtacgagaattgaaagacatgaaaattctcaa
ttctctacggcgtctaggagatagatagaatattttcaggaacaagaaaaccagaagaatctttctctctattcactaccattccgcgtcttcgacttctattagtttcttttc
ttctttaatgcaatagctatagtttgatatagaatccatttctcaaagtaatggaaacctttctcttataggaaatggttcgaaaatcgctattccaccttttaggtatcgtgaa
aagtgatacctgtgaagatcgtgcatttcagtcacattcagatccgtttttttgagtccatgatataaccaaattggatggatcttccacccgtttagctaagaaagaatag

atgcagaggtggataatagatcgatatgaagatcatgagctgccccataatgaaaccaccaggagtcgcgaatatctccttcttccctaatccaagattggagaaag
aagatctaagagggacccatggagaatgtggtcagaaacccataatagagtccgaccgcaacgaccgaattaattatcctcatcgagaaacttagactactaagta
gaaaagatttgaaaatcatggcatgggtctcctttttttctttctttagagttttctatatgcacaatttctcgatgtttcgatgagaatttcttgactttccatatatagaaagag
atagactataaatgacatctcttatgtcaataagaccaaagggatggatattaaatgataggaagtgctaggaagtgaaatagaatgaaatagagccactctgggctt
acctatgaaatgaggcatggaacggagccactacgaagaaattccgggagttacgaaagaagcttcggactcatattgttcatgggttgagagcgggagttgaact
ctaggaggtcgaatccccccttgttcctcagtagctcagtggtagagcggtcggctgttaactgactggtcgtaggttcgaatcctacttggggagatttgattcattcttt
aatgtaagaataaagaattgaattaaagggcttgctttgacccttaggagtaggtaacccgttcgctatccttgtttctattgcattttatctcatcgtatcacattctgttcta
cgattccacttcgacaaaaggaaagagcatacccaagttcaatagctttacgtccgctatcccgatcatggttttcctaccctcaggggggaaagtaaaggcccttccc
ctttggaaggctgtgggcgaggagggattcgaacccccgacaccgtggttcgtagccacgtgctctaatcctctgagctacaggcccaccccgtctccactggatc
tcttcccgggggtacccccccaaaaggaacctccctctcctcagccatttcatttcgggttaagaagatgggaaagccccttctctctataagaacagtgcgttccga
ggtgtgaagtgggagagaggggatgtgatgattgaggttttgaataagacgacctttgcattttggatttggatctttttcgtatttcaaaatagtgaaaaagtcaaataa
gaggtgttaagctttttatcattctggcatcgagctattttgccgcaggacctcccctacagtatcgtcaccgcagtagagtttaaccaccaaattcgggatggattggt
gtggttcctctacgcctaggacaccagaatatcgaaccatgaacgaggaaaggcatgagagaaatattggctagtaattgtgaagccccaattcttgactggaagg
gacaccaaaggcctctgccctccctctctatctatccaagagatggaagggcagagcttttttttggtttttcatcttttcatcaaagagttgaacaatgaagatagatg
gcaagtgcctgatcgatttgatcaggtcgtgtaggaacaaggttcaaatcgttcgttcgttaggatgcctcagctgcatacatcactgcacttccacttgacacctattt
aaacggctcgtctcgccgctaccttatcctatttccatgcttctgtcgctccatccccgtatgggtggagaacccgtcgctgtctcggctgtgctaccggaggctctag
ggaagtcggaggagagagcactcatcttggggtgggcttactacttatatgctttcagcagttatcctctccgcacttggctacccagcgtttaccgtaggcacgata
actggtacaccagaggtgcgtccttcccggtcctctcgtactagggaaaggtcctctcaatgctctaacgcccacaccggatatggaccgaactgtctcacgacgtt
ctgaacccagctcacgtaccgcattaatgggcgaacagcccaacccttggaaccacctacagctccaggtggcgaagagccgacatcgaggtgccaaaccttcc
cgtcgatgtggactcttggggaagatcagcctgttatccctagagtaacttttatccgttgagcgacggcccttccactcggcaccgtcggatcactaaggccgactt
tcgtctctgctcgacgggtgagtcttgcagtcaagctcccttctgcctttgcactcgaggaccaatgtccgtctggcccgaggaaacctttgcacgcctccgttacctt
ttgggaggcctacgccccatagaaactgtctacctgagactgtcccttggcccgcgggtctgacacaaggttagaatccgagctcttccagagtggtatctcactga
tggctcgggcccccccggaagggggccttcttcgccttccacctaagctgcgcaggaaaggcccaaagccaatcccagggaacagtaaagcttcatagggtcttt
ctgtccaggtgcaggtagtccgcatcttcacagacatgtctatttcaccgagtctctctccgagacagtgcccagatcgttacgcctttcgtgcgggtcggaacttacc
cgacaaggaatttcgctaccttaggaccgttatagttacggccgccgttcaccggggcttcggtcgccggcttccctgtcatcagttcaccaacttccttgaccttccg
gcactgggcaggcgtcagccccatacatggtcttacgactttgcggagacctgtgtttttggtaaacagtcgcccgggcctggtcactgcgacccccttttgcgag
ggggcacccctcttcccgaagttacggggctattttgccgagttccttagagagagttgtctcgcgcccctaggtattctctacctacccacctgtgtcggtttcgggt
acaggtacccctttgttgaaggtcgttcgagcttttcctgggagtatggcatgggttacatacttcagcgccgtagcgcctggtatgagcctcgtggagaagcaatgg
ctagtccacggggctcatacttcagcgctgcagcgcttggtactcggacctcggctcgaggcattttctctacccctttcttaccctgaaaaagcagggtcaccttgtgt
ccttaaacctataaccatctttcggctaacctagcctcctccgtccctccgtaccaacaaggggtagtacaggaatattgacctgttgtccatcgactacgcctttcggc
ctgatcttaggccctgactcaccctccgtggacgaaccttgcggaggaaaccttgggttttcggggcattggattctcaccaatgttttcgttactcaagccgacattct
cgcttccgcttcgtcgacccccgctttcgcggttgcttccctctaaggcggaacgctcccctaccgatgcattttgacatcccacagcttcggcagatcgcttagccc
cgttcatcttcagcgcaagggcgctcgatcagtgagctattacgcactctttaaagggtggctgcttctaggcaaacctcctggctgtctttgcacccccacctccttta
tcactgagcggtcatttaggggccttagctggtgatccgggctgtttccctctcgacgatgaagcttatccccatcgtctcactggccgaccttgaccccctgttatttt
gggtcatatctagtattcagagtttgcctcgatttggtaccgctcgcgcagcccgcaccgaaacagtgctttaccccctagatgtccagtcaactgctgcgcctcaacg
catttcggggagaaccagctagctctgggttcgagtggcatttcacccctaaccacaactcatccgctgattcttcaacatcagtcggttcggacctctgcttagtttca
tccaagcttcatcctggtcatggatagatcacccaggttcgggtccataagcagtgacaatcgccctatgaagactcgctttcgctacggctccggtgggttccgttc
ccttaaccaagccactgcctatgagtcgccggctcattcttcaacaggcacgcggtcagagatcactttcccctcccactgcttgggagctcagcacggtttcacgtt
ctatttcactacccactgggggttcttttcaccctttccctcacggtactacttcgctatcggtcacccaggagtatttagccttgcaaggtggtccttgctgattcacacgg
gattccacgtgccccatgctactcgggtcagagcgtaagctagtgatgctttcggctactggactttagccatctagggtgcggcactcaaccgcttcgcctagcag
cacaacgcttgtattgctctcccacaaccccgttttcacggtttaggctgctcccatttcgctcgccgctactacgggaatcgcttttgctttcttttcctctggctactaag
atgtttcagttcgccaggttgtctcttgcctgctcatggattcagcaggcagttcaaaaggttgacctatttgggaatctccggatctatgcttattttcaactccccgaag
catttcgtcgcttgctacgcccttcctcgtctctgggtacctaggtatccaccgcaagcctttcctcttttgaacctcgccattaacgttaaggctatgccatcctaaggtg
ctactaaatggaaggatcttatcaacgtccatgaatgcgaaatcatagatcgaactgccgaattggcaaaattcggtgctatcatagtatccgctaagttcacgggctg
gagataagcggactcgaaccgctgacatccgccacagggtaaaccaccgcctctcaggcctccccgacgggttctaccatagaggccaacgatagacaataact
cccccccgaacacagcttacaactttcatcgtactgtgctctccaaagagcaactcttctcaaaatctcaaaacaaaaggtgctgagttggaatcccattctaaggatt
cttgtggttccggggaatccagctacaggagaaccaggaacggggagctctcccctttttccgcccgactctttgatcttaaacttaagaatgctggttttaagaacga
gtgattgcccttctccgacccttactgcccaaccggagagcggacggctaatgtgttccacttattgaacagggtctatggtcggtccgtgacccctggacgccgaa
ggcgtccttggggtgatctcgtagttcctacggggtggagataatggggtcggtccatggattttccttcctttgccacatttcgctcaaagggttgaagggagatag
tgcatcaagctattcgcaagggccaacttgatcctcttccccagggatcccagatgagggaagcctaggagagccgccgactccaactatcgtccatgtacgatcc
atactagatctgaccaactgcccatcctacctcctctaccttttttgacagcccatctttttgtctcagtagagtctttcagtggcatgtttcagtcctcttccccattacttaga
aaaagtgagccaccggttcaggtacaagatactaccattaccgcctggacaattagacagccaacccgtaatcgcaacgacccaattgcaagagcggagctctac
caactgagctatatccccccgagccaagtggagtatgcatgaaagagtcagatgcttcttctattcttttccctggcgcagctgggccatcctggacttgaaccagag
acctcgcccgtgaagtaaatcatcgcccctacgatccaaccaattgggagagaatcaatagactccttttcgggagcgattcatccttcccgaacgcagcatacaac
tctccgttgtactgcgctcttcaagtgtgcttcttccccttctccccccttaccatggcaagtccttgggaaataactccgatgggcagaaaaaggaaggcgttaagaga
ccctcctggcccaaccctagacactctaagatcctttttcaaacctgctctgctcccatttcgagtcaagagatagataaatagccacatcccattgcactgatcgagg

gcgctcgtagtgacttagggagtcgaagaccaagaagtggcttatttataccaagcattcctcttatggctagatccaacctcctggtccctgcggaaaggaaaaag
aatttcacgttcttcctttcaggaagggaggattagggaagtcctattgattgctgctttctccagaccgccgggaaaagcatgaaaaaaaggctcgaatggtacgat
ccctccgtcaccccagaatgaaagggtgatctcgtagttcttggtctgtgaagatgcgttgttaggtgctccattttcccattgaggacgaacctcaacctgtgctcga
gagatagctctccatacactgataagggatgtatggattctcgagaagagaggagccgtagtgcccccccggaccgcccggatcccacgagtgaatagaaagt
tagatctacatgggatctcacctgaatcgccccatctatcctcctgaggagaagtttgtttggtttcaaactccgattcaaacaggaggagtacgccatgctaatgtgc
cttggatgatccacatcttcgggtcaggcgctgatgagcacattgaactatccatgtggctgagagccctcacagcccaggcacaacgacgcaattatcaggggc
gcgctctaccactgagctaatagcccgtcgcgcgggcctcccaaagggaggcctgctacgccaaaagcgagaaaaactccatccctttccttttgacatccccatg
ccgccacaccacacgggggggggcatggggacgtcaaaaaggggatcctatcactatcaactaatttgttccgacctaggataataagctcatgagcttggtctta
cttcaccctaaacgaaagaagacttccatatccaagtttagctcagacgtagctgccttctttttgggcgtgaagcagtgtcaaaccaaaatacccaataagcataagc
attagctctccctgaaaaggaggtgatccagccgcaccttccagtacggctaccttgttacgacttcactccagtcgcaagcctagccttaggcatcccccctccttac
ggttaagggtaatgacttcaaacatggccagctcctatagtgtgacgggcggtgtgtacaaggcccgggaacggattcaccgccgtatggctgaccggcgattac
tagcgattcctgcttcatgcaggcgagttgcagcctgcaatccgaactgaggacgggtttttggagttagctcaccctcgcgagatcgcgaccctttgtcccgcccat
tgtagcacgtgtgtcgcccagggcataaggggcatgatgacttggcctcatcctctccttcctccggcttaacaccggcggtctgttcagggttccaaactcatagtg
gcaactaaacacgagggttgcgctcgttgcgagacttaacccaacaccttacggcacgagctgacgacagccatgcaccacctgtgtccgcgttcccgagggca
cccctctctttcaagaggattcgcggcatgtcaagccctggtaaggttcttcgctttgcatcgaattaaaccacatgctccaccgcttgtgcgggcccccgtcaattcc
tttgagtttcattcttgcgaacgtactccccaggcgggatacttaacgcgttagctacagcactgcacgggtcgagtcgcacagcacctagtatccatcgtttacggct
aggactactggggtctctaatcccatttgctcccctagctttcgtctctcagtgtcagtgtcggcccagcagagtgctttcgccgttggtgttctttccgatctcaatgcat
ttcaccgctccaccggaaattccctctgcccctaccgtactccagcttggtagtttccaccgcctgtccagggttgagccctgggatttgacggcggacttgaaaagc
cacctacagacgctttacgcccaatcattccggataacgcttgcatcctctgtcttaccgcggctgctggcacagagttagccgatgcttattcctcagataccgtcatt
gtttcttctccgagaaaagaagttgacgacccgtgggccttccacctccacgcggcattgctccgtcaggctttcgcccattgcggaaaattccccactgctgcctcc
cgtaggagtctgggccgtgtctcagtcccagtgtggctgatcatcctctcggaccagctactgatcatcgccttggtaagctattgcctcaccaactagctaatcaga
cgcgagcccctccttgggcggatttctccttttgctcctcagcctacggggtattagcaaccgtttccagttgttgttcccctcccaagggcaggttcttacgcgttactc
acccgttcgccactggaaacaccacttcccgttcgacttgcatgtgttaagcatgccgccagcgttcatcctgagccaggatcgaactctccatgagattcatagttg
cattacttatagcttccttattcgtagacaaagcggattcggaattgtctttccttccaaggataacttgtatccatgcgcttcagattattagcctggagttcgccaccag
cagtatagccaaccctaccctatcacgtcaatcccacaagcctcttatccattcccgttcgatcgtggcgggggggagtaagtcaaaatagaaaaactcacattggg
tttagggataatcaggctcgaactgatgacttccaccacgtcaaggtgacactctaccgctgagttatatcccttccccgtcccctcgagaaagagaattaacgaatc
ctaaggcaaaggggcgagaaactcaaggccacccttcctccgggctttctttccacactattatggatagtcaaataatgggaaaaattggattcaattgtcaaccgg
tcctatcgaaaataggattgactatggattcgagccatagcacatggtttcataaaatctgtacgattttcccgatctaaatcgagcaggtttccatgaagaagatcttgt
tcagcatgttctattcgatactggtaggagaagaacccgactcggtattcttaaaaaaagaggggaagcagaaccaagtcaagatgatatgggtcgccccttcttctt
gcgccaaagatcttaccatttccgaaggaactggggctacatttcttttcaatttccattcaagagtttctatctgtttccacgcccttttttgagacctcgaaacatgaaat
ggacaaattccttctcttaggaacacatacaagaaaaaggataatggtagccctcccattaactacttcattttcatttatgaatttcatagtaatagaaatccatgtccta
ccgagacagaatttcgaacttgctatcctcttgcctaataggcaaagattgacctctgtagaaagactgattcattcggatcgatatgaggacccaactacgttgcatt
gccagaatccatgttccatatttgaagagggttgacctctgtgcttctctcatggtacaatcctcttcctgctgagcccccttctcctcggtccacagagaaaaaatgg
aggactggtgccgacagttcatcacggaagaaagaactcacagagccgggatcgctaactaatagaatagtactactaactaatactaatatatagaaatagatatc
tagaaatagaaacgaactaatatatagataatcgaaattgaaaagaactgtcttttctgtatactttccccgttctattgctaccgcgggtcttatgcaatcgatcggatca
tatagatatcccttcaacacaacataggtcatcgaaaggatctcggacgactcaccaaagcacgaaagccagttagaaaatggattcctatttgaagagtgcctaac
cgcatggataagctcacattaacccgtcaattttggatccaattcgggatttttcttgggaagtttcgggaagaaattggaatggaataatatagattcatacagaggaa
aaggttctctattgatgcaaacgctgtacctagaggatagggatagaggaagagggaaaaatcgaaatgaaataaataaagaataaagcaaaaaaaaaataagtc
gaagatagaagagcccagattccaaatgaagaaatggaaactcgaaaaggatccttctgattctcaaagaatgaggggcaaggggattgataccgagaaagattt
cttcttattataagacgtgatttgatccgcatatgtttggtaaaagaacaatcttctcctttaatcataaatggaaagtgttcaattagaacatgaaaacgtgactcaattgg
tcttagttagtcttcgggacggagtggaagaagggcggagactctcgaacgaggaaaaggatcccttcgaaagaattgaacgaggagccgtatgaggtgaaaat
ctcatgtacggttctgtagagggacaggaagggtgacttatctgtcgacttttccactatcaaccccaaaaaacccaactctgccttacgtaaagttgccagagtacg
attaacctctggatttgaaatcactgcttatatacctggtattggccataatttacaagaacattctgtagtattagtaagaggaggaagggttaaggatttacccggtgt
gagatatcgcattattcgaggagccctagatgctgtcgcagtaaagaatcgtcaacaagggcgttctagtgcgttgtagattcttatccaagacttgtatcatttgatga
tgccatgtgaatcgctagaaacatgtgaagtgtatggctaacccaataacgaaagtttcgtaaggggactggagcaggctaccatgagacaaaagatcttctttctaa
agagattcgattcggaactcttatatgtccaaggtcaatatggaaattctttcaggggttttcccttactttgtccgtgtcaacaaacaattcgaaatacctcgactttttca
gaacaggtccgagtcaaatagcaatgattcgaagcacttctttttccattacactatttcggaaacctaaggacttgatggtatggatatggaaaatacaggatttccga
tcctagcgggaaaaggagggaaacggatactcaattttaagtgagtaaactgaattccatactcgatctcatagatccctatagaattctgtggaaagccgtattcgat
gaaagttgtatgtacggcttggagggagatctttcctatctttcgagatccaccctacaatatggggtcaaaaagccaaaaaaataagtgattcgtttttagcccttataa
aaagaaaacggattcttgaacctctttcacgctcatgtcacgtcgaggtactgcagaaaaaagaactgcaaaatccgatccaatttttcgtaatcgattagttaacatg
gtggttaaccgtattatgaaagacggaaaaaaatcattggcttatcaaattctctatcgagccgtgaaaaagattcaacaaaagacagaaacaaatccactattggtttt
acgtcaagcaatacgtagagtaactcccaatataggagtaaaaacaagacgtaataaaaaaggatcgacgcggaaagttccgattgaaataggatctaaacaagg
aagagcacttgccattcgttggttattagaagcatcccaaaagcgtccgggtcgaaatatggctttcaaattaagttccgaattagtagatgctgccaaagggggtgg
gggtgccatacgcaaaaaggaagcgactcatagaatggcagaggcaaatagagctcttgcacattttcgttaatccatgaacagaatctaggtatgtagacacatg
gatccatacatctcgatcggaaaagaatcaatagaaggagaatcggacgatatctttctcgaaacaaacaaaaaggaaaagaaagagaaaacagaaatcatgatc
aactaagccctctcgggggcttgcttaagaataagaaagaggaatcttatggaaatagcatggaataaggtttgatcctattcatggggattccgtaaatatcccattc

caaaaatcgaaacaatcgggacttttcggagattggatgcagttactaattcatgatctggcatgtacagaatgaaaacttcattctcgattctacgagaatttttatgaa
agcgtttcatttgcttctcttccagggaagtttcattttcccagaatgtatcctaatttttggcctaattcttcttctgatgatcgatttaacctctgatcaaaaagatagacctt
ggttctatttcatctcttcaacaagtttagtaataagcataacggccctattgttccgatggagagaagaacctataattagcttttcgggaaatttccaaacgaacaattt
caacgaaatctttcaatttcttattttattatgttcaactttatgtattcctctatccgtagagtacattgaatgtacagaaatggctataacagagtttctgttattcgtattaac
agctactctagggggaatgtttttatgtggtgctaacgatttaataactatctttgtagctccagaatgtttcagtttatgttcctacctattgtctggatataccaagagaga
tctacggtctaatgaggctactatgaaatatttactcatgggtggggcaagctcttctattctggttcatggtttctcttggctatatggttcatctgggggggagatcgag
cttcaagaaattgtgaacggtcttatcaatacacaaatgtataactccccaggaatttcaattgcgcttatatccatcactgtaggacttgggttcaagctttccccagcc
ccttttcatcaatggactcctgacgtctacgaaggagtgtggttcgttcgacaaattcctacctctatatctatctctgaggtgtttgggttttgcaaaactccatagatatg
cagaagagaaatgctatccccactccgaccaagacagaacttttaccaaaagtttattgtgatctttttgttcaaataacaattaaggtgaagcagggtcaggaacaac
gaatctctttatgataaacagatccattttgcaagctcgttattacgggtagttcctacaaagaatcggactaatgacgtatacaatgcttgaattatcgacgtagatgcta
catagtgggttctcatccttcagagactacgagtgtaataggagcatccgttgacaaaaggatcaccctaagatgatcatctcatggctattgggaacgaatcaaatc
agatggttctatttctcaacctttctgacttgctcctacggaaccaaggtcgaaaggattgaaaaagtcagtcattcacaaccactgatgaaggattcctcgaaaagtta
aggattagtagttctttttcgaaatcgatttcgaaaaagaatggattcggtcttatacatacgcgaggaaggtaatcaaaaaagaaagaagacaagttcttctttcttttat
cacttaggagccgtgcgagatgaaagtctcatgcacggttttgcatgagagaaagaagcgaggaatcctcttttcgactctgactcccccactccagtcgttgcttttc
tttctgttacttcgaaagttgctgcttcagcttcagccacgcgaattctcgatattcctttttatttctcatcaaacgaatggcatcttcttctggaaatcctagctattcttagc
atgatattggggaatctccttgctattactcaaacaagcatgaaacgtatgcttgcatattcgtccatagggcaaatcggatatgtaattattggaataattgttggagact
caaatgatggatatgcaagcatgataacttatatgcttttctatatctccatgaatctaggaacttttgcttgcattgtattatttggtctacgtaccggaactgataacattc
gagattatgcaggattatacacgaaagatcctttttttggctctctctttagccctatgtctcttatccctaggaggccttcctccactagcaggtttcttcggaaaactctat
ctattctggtgtggatggcaagcaggcctatatttcttggtttcaataggactccttacgagcgttctttctatctactattatctaaaaatagtcaagttattaatgactgga
cgaaaccaagaaataaccccttatgtgcgaaattatagaagatcccctttaagatcaaacaattccatcgaattgagtatgactgtatgtgtgatagcatctactatacc
aggaatatcaatgaaccccattcttgcaattgctcaggataccctcttttagctgctaggtctatttcttagttcaagatccctcttactaactggaataaaagaattagtag
atctgttccgcccaaaatgggaatgggcgctagggtaatgaacttataatcatggaatcgactcgatcatcagattataagttcattccataccggaccagaccgtgc
acattcttattatgagaaggggtcattcgagcctatggaaataggatactctgtttacatagaaatccccacgtccttacattctatttaggattaggaataggtgtaatca
gacctgcttttgacatatctatcctatccttatttgggtaccatatgcacctctttgggcttctattgaatcgagaaattggattgtacatctttttgattttgatacatataaggt
gtcctacggataatgcaaatcgaagctatttgatgtctgactcaggcctatatgaccgatcgatcgaaatactccaagactccacctttgtcatatattccatatatcaca
ttagatagatatcatattcatggaatacgattcactttcaagatatcacattagatagatatcatattcatggaatatgattcactttcaagatgccttgatggtgaaatggta
gacacgcgagactcaaaatctcgtgctaaagagcgtggaggttcgagtcctcttcaaggcataatacggagaatgcgcattcaatgagcattccccgtagaagtatt
ccggaaatctgcgcctggcgctctcctctatcttctgaggtccttaaccacttccctgagaaaaggagacagtaaaagccaaaatagactaaatatagcctgaacga
tcctaaaaatccctcgaaggagataatataataaagaacccaaagcagacggtatcctactgcaagggtggtcttaagaatccaaaagaggttgctcagaagagat
agatgtatcccaacctctattgctctcgcgtaaagcctttttttttacgcgacaggaaaaagtgactacgaattccccttttttgtttgcgaatccctgtttgtatcctttgagc
gcacgcccataagtagcgatcaaggaaatcgatcaaacgatcccaataccgtgaaagagaaacttcccagatccagggaagcttatcataaagggcttcgacaag
gggttttattcgttctttgagcaaaaagataaagatcggatagattcgaaccgcaattgcaaaggtaatcactactatgccagcccaaatcatgatcttcaccaacttgg
atttggttctctcgcgaaaatcgcgagttgcagagatgagaaccatgaaaagcaagatcccgaataagaaaacagaaaccgaggaaaccacaagagtgaagact
agtagagtctcgtcttttgtcattctttgctcctttttcactcaatgattcattcgaatttcccgaccaaaaattctatatgtctattcatagggcctcgttgctaagtgctacaa
gatctagtgcactggaactcgtggttatggacccgaatcctttagtatggaacattgtcttttccaagtaaaaacccccagtatatgaaagaatgaaaaggtgctttcgt
tcttttcttgtggaataagaagccctcgtaccttaatgaaaggaaaataggaatttttcattaggtatttgaccaaataggatcgtccagttcctatagaacctatcactaa
aatacccgatagggctaagcggaacgaaaaggattttccctgagatggtaaatgaaaacgattagccccatacgaggtttgggaataagtgatgagcaaggaatat
acgtctttctgctaaagaggatctattaaactcataattcattagatccttgttatcaatgtcaactaggtatcataagtaaacggatcccggttgttcaatcctttgataacc
aaggtcattctttgctaaagagaaatgatcactatgagtcagactcaatagaattggatccattccaaatagcgagaattaggattctggatccctctcaatctctctttc
aattcgaggatccagagaggtgttttcatagtcatctccgaatatttgccatctccgaatattttcgatttcatttttctatgatatgtctttctatatgaaaattggttatttacg
atgtacgatgatccctgttaagcatccatggctgaatggttaaagcgcccaactcataattggtaaatttgcgggttcaattcctgctggatgcacgcgaaccggaac
gttccataagtctattggaactggctctctatccatggaatctcatccatcatccatacataacgaattggtatggtatattcataccataacataagaacaataagaactc
gaattcttatcgatactggaactcagagcataggagggaaagtcgatttatggatggaatcaaatacgcagtatttacagaaaaaagtcttcgtttattgggaaagaat
caatatacttttaatgtcgaatcgggattcactaagacagaaataaagcattgggtcgaactcttctttggtgttaaggtagtagctgtgaatagccatcgactacccgg
aaagggtagaagaatgggccctattctaggacatacaatgcattacagacgtatgatcattacccttcaaccgggttattctattccacttctagatagagaaaaaaac
taaaggagaatacttaataatacggcgaaacatttatacaaaacacctatcccgagcacacgcaagggaaccatagataggcaagtgaaatccaatccacgaaata
atttgatccatggacggcaccgttgtggtaaaggtcgtaattccagaggaatcattaccgcaaggcatagagggggaggtcataagcgcctataccgtaaaatcga
ttttcgacggaatcaaaaagacatatctggtagaatcgtaaccatagaatacgaccctaatcgaaatgcgtacatttgtctcatacactatggggatggtgagaaggg
atatattttacatcccagaggggctataattggagatactattgtttctggtacaaaagttcctatatcaatgggaaatgccctacctttgagtgcggtttgaactattgattt
acgtaattggaagtaaccaattaggtttacgacgaaacctagaaatcgatcactgatccaatttgactacctctacgggatagacctcaacagaaaactgttgagtaa
cggcagcaagtgattgagttcagtagttcctcatagaaaattattgactctagagatatggtaatatggagaagacaaaattgtttgaagcacgcacagaaccggaa
gcgccccttgtttcaaagagaggaggacgggttattcacatttaatttgatggtcagaggcgaattgaaagctaagcagtggtaattaagacccccgggggaaaata
gggatgtctcctacgttacccataatatgtagaagtatcgacgtaatttcatagagtcattcgatctgaatgctacatgaagaacataagccagatgacggaacgcgg
agacctaggatgtagaagatcataacatgagcgattcggcagatttggattcctttcctatatatccactcatgtggtacttcatcatacgattcatataagatccatctgt
ctagagatcgtcatatacatctagaaagccgtatgctttggaagaagcttgtacagtttgggaaggggtttttgagagaaaagaagaatctacttcaaccgatatgcc
cttaggcacggccatacataacatagaaatcacacgtggaaggggtgggcaattagctagagcagcaggtgctgtagcgaaactgattgcaaaagaaggtaaat

cggccactttaagattaccatctggggaggtccgtttggtatcccaaaactgcttagcaacagtcggacaagtgggtaatgttggggtgaaccaaaaaagtttgggt
agagccgggtctaagtgttggctaggtaaacgccccgtagtaagaggggtagttatgaaccctgtggaccacccccatgggggcggtgaagggaaagcccccca
ttggtagaaaaaaacccacaaccccttggggttatcctgcgcttggaagaagaactaggaaaaggaaaaaatatagtgatagttttattcttcgtcgccgtaagtaaat
acgtaactaggaatatggaaaattgcatttttggaatttgcaataatgggatgggcgaacgacgggaattgaacccgcgcatggtggattcacaatccactgccttga
tccacttggctacatccgccccttatccagctaaaggattttctctttttccattcatcattattctatttattctgacctccatacttcgatcgagatattggacatcgaatgc
cactctttaaaatggaaaaaaaggagtaatcagctgtgacacgaaaaaaaacgaatccttttgtagctcatcatttattggcaaaaatagaaaaggtcaatatgaagg
aggagaaagaaacaatagtaacgtggtcccgggcatctagcattctacccgcaatggttggccatacaatcgcgattcataatggaaaggaacatatacctatttac
ataacaaatcctatggtaggtcgcaaattgggggaattcgtaccaactcggcatttcacgagttatgaaagtgcaagaaaggatactaaatctcgtcgttaactgaatt
cagaatagaaagattcaaaataaaaaaaaaaaaaagaaata
>O.barthii_IRGC101252_cp
cccaatatcttgctgaagcaagatattgggtatttctagctttcctttcttcaaaaattgctatatgttagcagaaaagccttatccattaagagatggaacttcaagagca
gctaggtctagagggaagttgtgagcattacgttcgtgcattacttccataccaagattagcacggttgatgatatcagcccaagtattaataacgcgaccttggctat
caactacagattggttgaaattgaatccgtttagattgaaagccatagtactaatacctaaagcagtgaaccaaatccctactacaggccaagcagccaagaagaag
tgtaaagaacgagagttgttaaaactagcatattggaagattaatcggccaaaataaccatgagcggccacaatattataagtctcttcctcttgaccaaatctgtaacc
ctcattagcagattcgttttcagtggtttccctgatcaaactagaggttaccaaggaaccatgcatagcactgaatagggaaccgccgaatacaccagctacacctaa
catgtgaaatggatgcataaggatgttatgctctgcctggaatacaatcataaagttgaaagtaccagatattcctaaaggcataccatcagagaaacttccttgacca
atagggtaaatcaagaaaacagcagtagcagctgcaacaggagctgaatatgcaacagcaatccaaggacgcatacccagacggaaactcagttcccactcacg
acccatataacaagctacaccaagtaagaagtgtagaacaattagctcataaggaccgccattgtataaccactcatcaacagatgcagcttcccaaattgggtaaa
agtgcaatccgatcgccgcagaagtaggaataatggcaccagagataatattgtttccgtaaagtaaagaaccagaaacaggctcacgaataccatcaatatctact
ggaggggcagcgatgaaggcgataataaatacagaagttgcggtcaataaggtagggatcatcaaaacaccgaaccatccgatgtaaagacggttttcggtgcta
gttatccagttgcagaagcgaccccacaggcttgtactttcgcgtctctctaaaattgcagtcatggtaagatcttggtttattcaaattgcaaggactcccaagcacac
gtattaactagaaagataatagaaggcttgttatttaacagtataatatagactatataccaatgtcaaccaagccagccccgacagttgtatatccatacaacaaaattt
accaaaccaaaaaattttgtaaatgaagtgagtgaaaaatcaaaactcagattgctcctttctagtttccatatgggttgcccgggactcgaacccggaactagtcgg
atggagtagataattattccttgttacaatagagaaaaaacctctccccaaatcgtgcttgcatttttcattgcacacgactttccctatgtagaaataggctatttctattcc
gaagaggaagtctactaattttttagtagtaagttgattcacttactatttattatagtacagagaacatttcagaatggaaactgtgaaagttttaccttgatcatttatcaa
tcatttctagtttattagttttgtttaatgattaattaagaggattcaccagatcattgatacggagaatatccaaataccaaatacgctcactgtgcgatccacggaaaga
aaagtaagttgttttggcgaacatcaaagaaaaaacttgctcttcttccgtaaaaaattcttctaaaaataccgaacccaaccattgcataaaagctcgtaccgtgctttt
atgtttacgagctaaagttctagcgcatgaaagtcgaagtatatactttagtcgatacaaagtcttctttttgaagatccactgtgataatgaaaaagatttctacatatcc
gaccaaaccgatcaagaatatcccaatccgataaatcggtccaaattggtttactaataggatgccccgatccagtacaaaattgggcttttgctaaagatccaatga
gaggagtaacagggactttggtatcgaatttttcatttgagtatctattagaaatgaattctccagcatttgattccttactaacaaagaatttattggtacacttgaaaagt
accccagaaaatcgaagcaagagttttctaattggtttagatggatcctttgcggttgagtccaaaaagagaaagaatattgccacaaacggacaaggtaacatttcc
atttcttcttcaaaagaagagttccttttgatgcaagaattgcctttccttgatatcgaacataatgcataaggggatccataacgaaccatatggttttccgaaaaaaag
cagggtacattaacccaaaatgttccatcttcctagaaaagatgattcgttccagaaaggttccggaagaagttaatcgcaagcaagaagattgtttacgaagaaaca
acaagaaaaattcatattctgatacataagagttatataggaaccgaaatagtcttttattttctttttcaaaataaaaatggatttcattgaagtaataaaactattccaatt
cgagtagtagttgagaaagaatcgcaataaatgcaaggatggaacatcttggatccggtattgaaggagttgaagcaagatatccaaatggataggatagggtattt
ctatatgtgctagataatgtaagtgcaaaaatttgtcttctaaaaaaggaaatattgaatgaatagatcgtaaattctgaaactttggtatttctttttcttccggacaagact
gttctcgtagcgagaatgggatttctacaacgatcgcaacccccctcagatagaatctgagaataaaactcagaataaaaaaaattgttgtaatccaataatcgatcttg
gttaggatgattaaccaaattaatccaaaaattctgctgatacattcgaatcattaaccgtttcacaagtagtgaactaaatttcttgttattagaaccaataatttcgacaa
gttcggaaccatttaatccataatcatgggcaaacacataaatgtactcctgaaagagtagtgggtagacgaaatattgtctaggaaatttaagtttttctgaataaccct
cgaatttttccatttgtatttctacttgaatcagagagagagaaatatttctcggtttatcaaatggtgatacatagtacaatatggtcagaacagggtgttgcatttttttaat
acaaacccctggggaagaaaaggagtctaatccacggatctttttccgctccttttctatccaatttgtttatgtttgttctaattacaaaagagaacaaatcctttattttttg
caggccaattgctcttttgactttgggatacagtctctttatcaatatactgcttcttttacacattcaatccataacatccttttcaatccaaaatcaagaataattaggatttc
taaaaaaaaaaaaagaaaaaatcaaaggtctactcataggaaaaccagcttttccctacatcaggcactaatctatttttaacgtctaattagatcagggagttcttcca
attaagaagttaagctcgttgctttttgttttaccagaattggagccaggctctatccatttattcattagacccagaaaatcagaatttttttattccattccaaaaatccaaa
ataagaaattgattttattacgacatgctattttttccattcattacccttgaggatcagtcgcggtcttatagactctaccaagagtctggacgaatttttttgcttcatccaaa
tgtgtaaaagatcatagtcgcacttaaaagccgagtactctaccattgagttagcaacccagataaactaggatcttagatacgatcgaaatccaaaaatcaatggaat
tacaccgcacaccccctgtcaaaatcttaaaatagcaagacattaaaagaaagattttatcaccattgaaaacactcagataccaaaaggaacgggtctggttaaatttc
actaaggttaaaagtggcaccaatcacgatcgtaaaattgtcatttttttagcatttttatttaaataaataaataaatcttgtatgagagtacaaacaagagggacaaccc
taccatttgagcaaagtgtaggcaaaaaacctaatagggagtgaggataaagagacttatccatctacaaattctagatgttcaatggacctttgtcaatggaaataca
atggtaagaaaaaaaattagatagaaaaactcaaaaaaataaaggcttatgttggattggcacgacataaatccagtcaaaaataggattaagaaagaggcaaattatt
tctaaatagttagacaacaagggatactagtgagcctctcctagttttttattcatttagttcttcaattaactcaaagttctttctttttctttaaagaattccgccttccttaaa
atatcagaaactgttcttgtaggttgagcacctttttcaaggaaatagagaatagctggaacatttaaacaagtttgattctttatcggatcataaaaacctacttttcgaa
gatctcttccttctcttcgagatcgaacatcaattgcaacgattcgatagacagcttattgggatagatgtagataaacaaagcccccccctagaaacgtataggaggttt
tctcctcatacggctcgagaatatgacttgcattaatttccgtacagaaaaaacaaatttcatttatactcatgactcaagttgactaattttgattgacagacttgaaagaa
aaaaatcctttgaaatttttgagtcgtctctaaactcttttctttgcctcatctcgaacaaattcacttttattccttattccggtccaattctattgttgagacagttgaaaatc
gtgtttacttgttcgggaatcctttatctttgatttgtgaaatccttgggtttaaacattacttcgggaattcttattcttttttctttcaaaagagtagcaacataccctttttctt

atttccttcgataaagcatttccctcttctatagaaatcgaatatgagcgattgattctgatagacttttaatcaaaagagttttcccatatcttccaaaattggactttcttctt
attttaaccttttgatttctatattatttcgatttctatattaagggtagaatgacaaagttggcctaatttattagttttcactaaccctagattctttcccttgataaaaaataaat
tctgtcctctcgagctccatcgtgtactatttacttagcttacttacaaacaacccagcgaaaattcggttcgggacgaatagaacagactatgtcgagccaagagcat
tttcattactatggaaaatggtggatagcaaaatccacaatcgatcgtgtccttcaagtcgcacgttgctttctaccacatcgttttaaacgaagttttaacataacattcct
ctaatttcattgcaaagtgttatagggaattgatccaatatggatggaatcatgaatagtcattagtttcgtttttgtatactaattcaaacttgctttgctatctatggagaa
atatgaataaaagaaattaagtatttatcgggaaagactccgcaaagagccaatttatttaaacccatattctatcatatgaatgaaatatagttcgaaaaaagggaata
aacaagtttgcttaagacttatttattatggaatttccatcctcaacagaggactcgagatgatcaatccaatcctgaaatgataagagaagaattgactcttctccaaca
aataaactatcaacctcccgtttaattaatttaattaatatattagattagcaatctatttttccataccatttttccgtaacaaaactaattaactattaactagttaaactattgc
aatgaaaagaaagtttttggtagttatagaattatcgtatttcttcgactcgaataccaaaagaaagaaaaaaatgaagtaaaaaaaacgcatttcctgtaaagtaaaat
taaggtctttgcttttacttatttttcttttacctaaaagaagcaactccaaatcaaaattgaatccattctatctaacgagcagttcttatcttatctttaccgggatggatcat
tctggatatttaaaaaatcgcggatcgagatcgttttcgcttaaccaaagaaagaaaaagaagaaggaaccttttttactaataaaatactataaaaaaaatttatctctat
cataaatctatctctaccataaaggaataggtctcgtttttatacaatgttctacgtcaagtttaaaattttttcatgaaaaaaagattttcaatttgactggacttgacactg
gattatgttttctgagacagaaaatgaacgcattaggactgcatcgaatctaagagtttataagagaaaaaaattctctttaataaactttatgtctcgtgcagaatacaat
acgatttcatctttcgtttcatcagaaaaaatctgggacggaaggattcgaacctccgagtaacgggaccaaaacccgctgccttaccacttggccacgccccatttc
gggttttatgcgacactaataaacagtattatttttatttcttattcgtcaatcctacttcaattacataaaaatggggggtattctcttggtaggattctagacatgcgaataa
tatagaatccaaaaaatgcattgatcattacatggaattctattaagatattatatgaaagtcgaatttcttccactctcatttgagagtgcgaatacaaggaggtattttgt
gtttgggaaagtccgaagaaaaaaggatttgaatcctccttttcctttttcccttagaaaaataactcaatcaaaatccaattatctactctacaagaacgaaacgcttgt
tatgcctaatatacttagtttaacctgtatttgttttaattctgttatttatccgactagtttttcttcgccaaattgcccgaagcttatgcgattttcaacccaatcgtggatttta
tgcctgtcatacctgtactctttttctattagcctttgtttggcaagctgctgtaagttttcgatgaaatctttactactctgtctgccaaattgaatcatgtattcattctaaaa
aaattcgaaaaatggataagagccgagaagtcttatattatgaaccttcgattctaaaattcaaattcttctacattgaatgtatagctgcagcaataaatttggatcagcc
tttctactccctgcatctacgttgagcaggtatctttaggtaaccgcacaatacctaacctaatttattgataagagtgcttattataaatcaattcttgcaatttttttcaaaa
attgatttttgcatttttaggtgtcaaaataaacaaaacccatcctagtggatttgtgtggtaaggaaaaacgggtaatctattccttaaaaaaaaatcttggagattatgta
atgcttactctcaaactttttgtttatacagtagtgatattctttgtttccctctttatctttggattcttatctaatgatccaggacgtaatcctgggcgtgacgagtaaaaatc
caaaatttttcttacaaattggatttgtttcatacatttatctacgagaaaatccgggggtcagaattccttccaattcgaaagtcccaaacgatccgagggggcggaa
agagagggattcgaaccctcggtacaaaaaaattgtacaacggattagcaatccgccgctttagtccactcagccatctctccccgttccaaatcgaaaggtttccgt
gatatgacagaggcaagaaataacgattgcaaaaaatccttcctttttctttcaaaagttcaaaaaaattatattgccaattccatttagttatattctttttcttaatgttaat
aaaaaaaagaagaaaattcttctttttctttctaattctaaaattggatattggctaaaagacaatcagatagattttctcttcagcaggcatttccatataggacttgttata
ataaaacaagcaggttatagaaaaaaactcttttttttattatttatcaacaaagcaaaaaggggtcttatcaaaccaacccaccccataaaattggaaagaaagataaa
gtaagtggacctgactccttgaatgaggcctctatccgctattctgatatataaattcgatgtagatgaaattgtataagtggattttttgtatttccttagacttagaccac
gcaaggcaagaatttctcgctatttactatttcatattcttgttactagatgttctataggaataagaagaaatcgcaacccctttccgctacacataaaaatggatttcga
aagtcaatttttcttttcaatatctttactttttttcagaatcctatttttgttcttatacccatgcaatagagagcgagtgggaaaagagaggttactttttttcatttttcccttaa
aaaataggctttcttggaaataggaatcatggaataatcctgaattccaatgtttatttctatagtataagaaaaactaattgaatcaaattcatggatttaccacgacctc
ggctgtgaccccatagataaaaatgcaaaatttctatcttcgagaccattgaaaaaaggcattgaacgagaaaaaatcgtccacagataatctatcgtatgccttgga
agtgatataaggtgctcggaaatggttgaagtaattgaataggaggatcactatgactatagcccttggtagagttactaaagaagaaaatgatttatttgatattatgg
acgactggttacgaagggaccgttttgtttttgtaggatggtctggcctattgctttttccttgtgcttatttcgctttaggaggttggtttacagggacaacttttgtaacttc
ttggtatacccatggattggcgagttcctatttggaaggttgcaatttcttaaccgcagcagtttccacccctgccaatagtttagcacactctttgttgctactatggggc
ccggaagcacaaggggatttactcgttggtgtcaattaggtggtctgtggacttttgttgctctccatggggcttttgcactaataggtttcatgttacgtcaatttgaact
tgctcggtctgttcaattgcggccttataatgcaatttcattctctggcccaatcgctgtttttgtttccgtattcctgatttatccactggggcaatccggttggttctttgcg
ccgagttttggcgtagcagcgatatttcgattcatcctcttcttccaaggatttcataattggacgttgaacccatttcatatgatgggagttgccggagtattaggcgcg
gctctgctatgcgctattcatggggcaaccgtggaaaacactctatttgaggacggtgatggtgcaaataccttccgcgcttttaacccaactcaagctgaagaaact
tattcaatggtcaccgctaatcgcttttggtcccaaatctttggtgttgctttttccaataaacgttggttacatttctttatgctatttgtaccggtcaccggtttatggatgag
tgctattggcgtagtcggcctggctctgaacctacgtgcctatgacttcgtttcccaggaaatccgtgcagcggaagatcctgaatttgagactttctacaccaaaaat
attcttttaaacgagggtattcgtgcgtggatggcagctcaggatcagcctcatgaaaatcttatattccctgaggaggttctaccacgtggaaacgctctttaatggaa
cttttgttttagctggtcgtgaccaagaaaccaccggttttgcttggtgggccgggaatgccagacttatcaatttgtcgggtaaactacttggagctcacgtagcccat
gcaggattaatcgtattctgggccggagcaatgaacctatttgaagtggcccatttcgtaccagaaaaacccatgtatgaacaagggttgattttacttccgcacttag
ctactctaggttggggagtagggcccgggggagaagttctagatacttttccgtactttgtatctggagtacttcatctaatttcctccgcagtcttaggcttcggtggca
tttatcacgcgcttctgggaccggagactcttgaagaatcttttccattctttggttatgtgtggaaagatagaaataaaatgactacaattttgggtattcacctaattttgt
taggtataggtgcttttcttctagtactcaaagctctttattttggcggtatatatgatacctgggctcctgggggaggagatgtaagaaaaattaccaatttgacccttag
ccccggtgttatatttggttatttactaaaatccccttttgggggagagggttggattgttagtgtggatgatttagaagatataattgggggacatgtatggttgggtttc
atttgtgtatttggcggaatttggcatatcttaaccaaacccttcgcatgggctcgccgtgcatttgtatggtctggagaagcttacttgtcttatagtttaggcgctttatct
gtctttggttttatcgcttgttgttttgtctggttcaataatacagcttatccgagtgagttttatggacctactgggccagaagcttctcaagctcaagcatttacttttctagt
tagagatcagcgtcttggagctaatgtgggatctgcccaaggacccacaggtttaggtaaatatctaatgcgttccccaacgggagaggttatttttggaggggaaa
ctatgcgtttttgggaccttcgtgctccatggttagaacctctaaggggccccaacggtttggacttgagtaggttgaaaaaagacatacaaccttggcaagaacgac
gttcggcagaatatatgacccatgctcctttaggctctttaaattctgtgggtggcgtagctaccgagatcaatgcagttaattatgtttctcctagaagttggttagcgac
ttcccattttgttctaggattcttctttttgtgggccatttgtggcatgcaggaagagcccgggctgctgcagcaggatttgaaaagggaatcgatcgtgatttggagc
ctgttctttacatgacccctcttaactaagatttcttatttatacctgttctactgtttttttctgttctggctcggttattctatctagccgagccattcattcctttctatgaaag

aaagataaggggacagaacaaaaaaaacaaataaagaaacaaacgtattcaatacgcaaaagaaaagagagaggaaagtaaaaggagagagagggattcga
accctcgatagttcctagaactataccggttttcaagaccggagctatcaaccactcagccatctctccacagcctaatccttattttattcctacaaatagaacatagcc
atacgaaatgactcactaacctctagaaacatctcaaatacaaatccctttcgatatatttctgtatactgtatccatgtatacaggatccgctatatccgcttgtgaaata
aagcataaaacccccctcaaccccatatccaaataaaaaaagtggtgagtaataagttttaaagagaagaatcaatggattcatgattaaacccctcctacttcttgtattt
tattacaattttgattaagtgagggatcaaatataatatgtagtcaactttatttgatggtaacttggaggattagaaatatgactattgctttccaattagctgtttttgcatta
attgtgacttcctcagtcttagtcattagtgtaccccttgtatttgcttctcctgatggttggtcaaacaataaaaacgttgtattttccggtacatcattatggattggactgg
tctttctggtagctattctaaattctctcatttcttaaatgtgtttagtatttagtagcccgatacaaaataaaaaagggccgtttattcggattgtgagacgcattaaaatgc
aatttgcgccccgaattgattgacagacaattaaattaataaaaagaaaactcaaatagaaaatgaaacggtcgacccagacatagacggtcgacccaggcggata
taccctataaaatataggacgtagcgagcgtagttcaatggtaaaacatctccttgccaaggagaagatacgggttcgattcccgccgctcgccagcttaatttagta
aggtactatgataaaaaatttagtctacttttattaaagtaataggtgttagtctagtaccgtacccctttactatcttagccccctttgcaccccactcaaaaaaagagca
ctacagcggcgggaatcgaactcggcaacagggttccctaaaccggggattcaccgaaacaaacaaccagcaaacagcttttaaagggaagggagatagactg
tgcctttctttcatttatttttcttttctgcaaggtagggggccttgagagttcctcttgtggtagcaagttacttcgcaacctgctcaatttggccttatagggtcgggaact
aatgaataaaaagggttggataccgcccaaccacccagccctctaccatatctagacaaatagaatagtccttttatacagactgctaagtgcggagacgggaatcg
aacccgtgacctcaaggttatgagcctcgtgagctaccaaactgctctactccgctctggagcgctggaaaccggtggacgaaaaaggttgaatacaatacaggc
ctctaccatgtctagacaaatagaatagttattttatacagaatggagcgggtagcgggaatcgaacccgcatcgttagcttggaaggctaggggttatagtcgacgt
tggttgattattattaacgtctctaattcaaaaccgaacatgaaattttgatttcattcggctcctttatgggtattctcaccacttaacatctaagtcagctttttgtctgaatg
gaaccaaggctctctgctttctagatgatccctaaagagtaggagatagaaatttgcctaaatctatctaatctaattacttcgttccctaatttcatttaagagatcctgag
gaaaagaattgggtttccaccgagctgaaacaatatgctgatggttctagtaaaccaaaactaccgtttttagctatttggcttccatttcctttttaacaaaagaagatt
tagttacgattggaaataaatttttttgtatcttcatccatagatcctttactcatattttaaaaattggaatccttaatccaatgcaaaattatgcttcgcgactctgtactcata
atccaaatcctatttgtttttggatgcaatttcaattagtctttgggtacaaatcgcgaaaatgcatattcttcctcaatatgctattgagagaaaaaggagtaaatcctttct
aagaactaaagttttcatcggaatataaaaaaccttaaggacgccttaagtatatcatttcaaattcagttattaatagaacgaatcacacttttaccactaaactataccc
gctacatgtaaattctgataccaacgctaccctttgtcaagggtagccattcgagaaagatgctaattccctcttagttaatgaaacagagaaggttcctcacagttagc
agttggtacttcgatcgcgggcctttcctttactttctttttgttcagaattgaacaaagaatttggggaagaaaacatcttcccccacctatcatgaaatctgggccata
gagaaagagtgagatgtttttttttatttatcatagactttccctatggcttgagagaaacaataaataacttaaagaaaaaggcacataggagccgaaggatttacttga
tgtaaagaagattctgaatgtctctgcttagtcgattctctccgtttaactttttcttctcttcttttccactcaattctagtttattagattcttgtttaaaagaatcaaagaagat
gaatagaactaagaacacacaaaaaagagcatataggcccgagaccattaccaaaagttcttcccaataatcatattgggtatctgttcccttcctttttcctattaggat
ggggcatgttggattttccatatccatataccatcgaaccttaagggttccagaaccctcctttttgctagttttcggaaacggaaaaccctgaaccaggagcagtata
aattctactgcccgccttttacaagaaaattggtgataaaactccactacagtttgttcaaaatgcaccaatcagaatcccttgagaatattcaagtaccctatttctaaga
tatttctaagagaagaggtacctgctgaaaatcgcttcaacaaatccgcttaagaaaaattgcaaagttttgaactcgaaggtttttccaagggatgcctgttaaaaatt
gtttcaacctctcaccaaaacagacaagaagtatatcactgaaaattaatacccagccatatgggtatatgaagggcgcaaattcgtttataccccacccaattagag
gaaataaaacataaatggagaaagttttcatgataagatcagccaatagaagaaaaaagtccctaatttttcagaccgttctgagcatgtgaaaagtcaatagcctaaa
gataaaaaaccctatactttgtgcaagtgataagagagaatatgaaagattttcctatttttcttgattttcttaagatttttttgaacctgaccatgaatagacttatatatctc
gatatatacagatataatgtacattatggagtagacctataatgggaaatgaaagtggctaattttggaattgaataagaagcccttttaactcagtggtagagtaatgc
catggtaaggcataagtcatcggttcaaatccgataaagggctttttttacttagtggtagagtaatgccgcggtaagacgtgagtcagtggttcaaatccgatacagt
acttttctactaaattcattcactttctttctttttgaaaatttctctttttttattgaatttgataacttagtgcgagatgcatgcatttttagtttaaacactaagcgaagcaggg
gggtgtaaattccaaaaaagaaattggactctttttcctattagatcaatcaaatcactacccgtactgaactaatatagaatcccttttattaatctattcttattccatatcc
tttataaacgaatttccctaaaaagtaggggatgatccgtgaattaacctaaccatcaactaaaaaaaatcctacaaaagcataatggaaaagtaggaaggactcctt
gctttggatctagttatactcttcgagtatattgacaattccaaaaaactgctcatactatcattatagtataatgaggagcggttgtataccgccctatcgtctagtgatgc
ccctatcgtctagtggttcaggacatctctctttcaaggaggcagcggggattcgacttcccctgggggtagggagtattatgaaaggaggttaatcatagattatca
aaaaccctagaataaattcttcctgggtcgatgcccgagcggttaatggggacggactgtaaattcgttgacaatatgtctacgctggttcaaatccagctcggccca
aaaatctagggcttcgtgaatatgagttaaatccattttttttcttccataaaaaagaatatttgatccatagaaataaaagaaataaaggataaaaagaaaaggggaaa
tatctttctaatctatatctctttcattcctctcttacaaacaaaagaccttttcttattggttattgaaaggtggattattatctatttttagcgataataaatcgcgacatactag
ttatgtcattctcactataccccccatacgatacgggggtatgtagtatatgattcgtctatttcttagagtaggacaggcgaatattcttattcttatggttccatttaagaata
agtatgccatacgccccgcggggattgtagttcaattggtcagagcaccgccctgtcaaggcggaagctgcgggttcgagccccgtcagtcccgaactagggttc
aatgaatggagaaattcatctttcctttttccatgaaaaaagggggggcaggaagcaagatcaaatacctatggggtaccccctatttcacttttttttagttcgcgtttctcag
taaagagaggagagtataggaattttttttatcactacttctggttgatagcgaaagacatacatatcatacgtggaagggatcctcctatgttatactattccactctcaac
catgaattgatttgatagatccgatattcataatattgaattgattcagtattatcagaatgcaagtcctccccttgaatttacaggatacccttttccctctccatgggatta
catcccgagttattgcgaaaaaaaaagaggttatggaagtcaatattctcgcatttattgctactgcattgttcattctagttcctactgcctttttacttattatttatgtaaaaa
cagtcagccaaaatgattaattggaagttcaattaatcattgaagaaatgaaaaagggattaaataaaataaaaatccaagtcttaaatgaaaggatccggttggaatc
ataaagtgtggtagaaaaaactacatatagtttttttctaccacactttagagtctttctattatattatcttgaatctacatagaatagattactagattgaaatagtagtctaat
tcaatttcttttttcactgcatccacttaatttcaatcaagtcaaaatgaaaaaatccatggagggagagaaaaataatatgagaatagactatagtaaaagaaaaaaag
taaaaggaaaaaaccagcgaatctttcatgcttaaacatgtcgcgaaatgtttcaaaagagcataaaatttattttaagaactaagaataagaaaagagtataaaacaa
atggaaaatgtgcgatatgttgggaatagctccgcggaagaaaatctaaagttcttatgtatagaactttttttaaccatgggtcgcttctagtagcgattatgaattgctct
caccgctctttctatttctattttctattctttctatttctatttctatttctatatagtagaatagaaatagaaatagaaagaatagaaagactctttcttacaagagtttcttaca
ggagtgaaacaaaattaaagaaagagagaaagaataaagttcggcaaaatgattaatgcaaaacggtcaattaaagaaaagagttgatacaacaattcgactactc
aatcaattagtagtatccctagagtccactcctcccccatactactagtgaaagagaaaatgtaaagactaccattaaagcagcccaagcgagacttactatatccatg

taaattatgtctcctatttctatgaaggaattattctactattgatgaataatcatagtagaatcaagggtacagagtcaaaaaggggttctgacctaaggctatggatga
atcagttcaaagaatttactcttaacaaattcttagagtatttctggtagaattggggagcattaagtataaatatgatacatagccctttcttattaataaaagaataagga
aacgctatctcatccctattggtatcggtttgggccactactgctaaaacaaaccccagtttgaggaaagaacggtgggttctcaaaatccagtatcgccgagccttgt
tattctcttgccccaacttatgcggggtgcaaatttgtcgatttggatcagtactataagcctaagtattttattgatcaggcggcacccagatttgaactggggataaag
gatttgcagtcccctgccttaccgcttggccatgccgccaaaaaatacgatctaaaatcgagaaaagagcaagtattcatccacgtttcttactaaaactaactttctttt
atcttaaatctaattctacttactttttccaatctttttcaaaaaatctattcatgcttttttggatccagtttcgattattctcctcaaaggattctatcttaaaacacacattgct
aacactagaaaacttccctttctttctattgaaatgaaaaaggagaaaagtggatttctagtcacaagctacaaaattaagaacaaattggaaccattaactagaattct
ctttttattttgaatttcggtattctctcccgcctgccatttaatggcataataaaagacaatggatttatgcctaatccgtatataggtaaactccaggtccgaacagcatt
attatctatggatccccccttatgtacatatctctgtggagaatcgttctttaatttttcattgcattaaatatcttgaataaaaaaaagaaaattgactttgctatgtggaggtc
cagaactagattggcatgtacttaaaaaagtacttactttattttaggattctacaacgaaatcttatatcttatattttatagaaattctactactacgaaacaaaaaagaac
cttcaaattctttttgaaattaaactaagcgtgctattctcaatcgaactaaagtcaaactttctagtgcttataaattattatattttggttttatcccattcatagaaaggaga
aaaaatgagaaatctttgccgtccaatctaagaatatcataaactgtaagtggcagaattttttcgaggttctaggaatgttttatcacttcattttcattccatttggaccc
ctgggaaattcgaactttcgttgaaattgtctctattcatatgtatgaaatacatatatgaaatacgtatgtggagttcccgagaatttcatgtgatttagtaaacagaatat
agattccataattgctagatcgatctgtagggattgatgaagagtgagctgataatggaatttttcttcgataaacaggaaacttaagattaagatgctccggaatggaa
atgagggaatgtccacaatacccggatttagtcagatccaattcgagggattttgtaggttcattaatcaaggcttggcagaagaacttgagaagtttccaacaattaa
agatccagatcacgaaatttcatttcaattatttgcgaaaggatatcaattgctagaaccttcgataaaagaaagggatgctgtgtatgaatcactcacctattcttccga
attatacgtatccgcgcgattaatttttggtttcgatgtgcaaaagcaaaccatttctattggaaacattcctataatgaattccttaggaacctttataataaatggaatata
ccgaattgtgatcaatcaaatattgctaagtcctggtatttactaccgctcggaattagaccataaaggaatttctatctacaccgggactataatatcagattggggag
gaagatcggaattagcaattgataaaaaagaaaggatatgggctcgcgtgagtagaaaacaaaagatatctattctagttctatcatcagctatgggttcaaatctaaa
agaaattctagataatgtttcctaccctgaaattttcttgtctttccctaatgctaaggagaagaagaggattgagtcaaaagaaaaagctattttggagttttatcaacaa
tttgcttgtgtaggtggggacctggtattttcggaatccttatgtgaggaattacaaaagaaatttttcaacaaaaatgtgaattaggaaggattggtcgacgaaatatg
aatcgaagactgaatcttgatatacctcagaacagcaccttcttgttaccgcgagatgtattggccgctacggatcatttgattggaatgaaatttgaaacgggtatact
tgacgatgacgatatgaatcacttgaaaaataaacgtattcgttcggttgcggatctgttacaagatcaattcggactggctcttggtcgtttacaacatgcggttcaaa
aaactatccgtagagtattcatacgtcaatcaaaaccgactccacaaactttggtaactccaacttcaacctcgattttattaataactacttacgagaccttctttggtac
atatcccttatctcaagtttttgatcaaaccaatccattgacacaaacggttcatgggcgaaaagtgagttgtttgggtcctggaggattgacggggagaactgcaagt
tttcggagccgagatatccatccgagtcactatgggcgtatttgtccaattgacacgtccgaaggaatcaacgttggacttactggatctttagctattcatgcgagaat
tgatcattggtggggatccgtagagagtccattttatgaaatatctgagaaagcaaagaaaaaaaaagagagacaggtggtttatttatcaccaaatagagatgagta
ttatatgatagcagcaggaaattctttgtccttgaatcggggtattcaggaagaacaggttgttccagctagataccgtcaagaattcctgactattgcatgggaacag
attcatgttagaagtatttttcctttccaatatttttctattgggggttctctcattccttttattgagcataatgatgcgaatcgagctttaatgagttctaatatgcagcgccaa
gcagttccgctttctcggtccgagaagtgcattgttggaactggattggaacgccaaacagctctagattcgagggtttctgttatagccgaacgcgagggaaagat
catttctactaatagtcacaagatccttttatcaagtagtgggaagactataagtattcctttagttacccatcggcgctctaacaaaaatacttgtatgcaccaaaaacct
cgggttccgcggggtaaatccattaaaaaaggacaaattttagcggaggggggctgctacggttggtggggaacttgctttaggaaaaaacgtattagtagcttatat
gccatgggaaggttacaattttgaagacgcagtattaattagcgaacgtttggtatatgaggatatttatacttcttttcacatccgaaaatatgaaattcagacggatac
aacaagccaaggctccgctgaaaaaatcactaaagaaataccacatctagaagaacatttactccgcaatttggatagaaatggagttgtgaagttggggtcctggg
tagaaacaggcgatattttagtaggtaaattaacgcctcagatagcgagcgaatcgtcctatatcgcggaagctggattattacgggccatatttggtcttgaggtatc
cacttcaaaagaaacttctctcaaactaccgataggtggaagaggacgcgttatcgatgtgaaatggatccagagggatcccctcgacataatggttcgtgtatatat
tttacaaaaacgcgaaatcaaagttggggataaagtagccggaagacacgggaataaggggatcatttccaaaattttgcctaggcaagatatgccctatttgcaag
atggaacgcctgttgatatggttttcaatcccttaggagtaccctcccgaatgaatgtgggacaaatatttgaaagctcgctcggattagcaggggatctgctaaaga
aacattatagaatagcaccctttgatgagagatatgagcaagaggcttcaagaaaacttgtgttttcagaattatatgaagccagtaaacaaacaaaaaatccgtggg
tatttgaacccgagtacccgggaaaaagcagaatatttgatggaagaacaggagaccccttcgaacagcctgttctaatagggaagtcctatatcttaaaattaattc
atcaagttgatgagaaaatccacggacgctctactgggccctattcacttgttacacaacaacccgttagaggaagagccaagcaaggggggacaacgaatagga
gaaatggaagtttgggctttagaaggatttggtgttgctcatattttacaagagatacttacttataaatctgatcatcttatagctcgccaagaaatacttaacgctacgat
ctggggaaaacgagtacctaatcacgaggatcctccagaatcttttcgagtgcttgttcgagaactacgatctttggctctagaactgaaccatttccttgtatctcaga
agaacttccaggttaatagggaagaagtttgatcggaataaatataaattcttttcttatttctattttatgattgaccaatataaacatcaacaacttcaaattggactcgttt
cccctcaacaaataaaggcttgggctaacaaaacactacctaatggggaagtcgttggcgaagtcacaaggccctccacttttcattataaaaccgataaaccagaa
aaagatggattgttttgcgaaagaatctttggacccataaaaagcagaatttgtgcttgtggaaattctcgagcgagcggagctgaaaacgaagacgaaagattttgc
caaaaatgcggagtagaatttgttgattctcggatacgaagatatcaaatgggatacatcaaactcgcatgtcccgtgactcatgtgtggtatttaaaaggtcttcctag
ttatatcgcgaatcttttagataaaccccttaagaaattggagggcctagtatatggcgatttctcttttgctaggcccagtgctaaaaaaccaactttcttacgattacga
ggtttattcgaggatgaaatttcatcctgtaaccatagcatttcccccttttttttctaccccaggctttacaacatttcgaaatcgggaaattgcgacaggagcaggtgct
attagagaacaattagcagatttggatttgcgaattattttagagaattcctcggtcgaatggaaggaattagaagacgaggggtatagtggagatgaatgggaagat
agaaaaagacgaataagaaaagtttttttgattagacgcatgcaattggcgaaacattttattcaaacaaatgtagaaccagaatggatggttttgtgcttattaccagtt
cttcctcccgaattaagacccattgtttataggtctggggataaagtagtgacttcggatattaatgaactttataagagagttatccgtcggaacaacaatcttgcctat
ctattaaaaagaagtgaattagcgccagcagatttagtaatgtgccaggaaaaattggtacaagaagccgtggatacacttcttgatagtgggtcccgcgggcaacc
aacgagggatggtcacaataaagtatacaaatcactttcagatgtaattgaaggtaaagaggggaggtttcgcgaaactctgcttgggaaacgggtcgattactcgg
ggcgttctgtcattgttgtgggtccttcactttcattacatcaatgtgggttacctctagagatagcaataaagctttttcagctatttgtaattcgcgatttaatcacgaaac
gcgctacttctaatgttaggattgctaaaaggaaaatttgggaaaaggaacccattgtatgggaaatacttcaagaagttatgaggggacatcctgtactgttgaatag

agcacctaccctgcatagattaggcatacaggctttccaacccactttagtagaggggcgtactatttgtttacacccattagtgtgtaaaggtttcaatgcagactttga
tggggatcaaatggctgttcatctacctttatccttggaagctcaggcggaagctcgtttacttatgttttctcatatgaatctcctatctcccgctattggggatcctatttg
cgtaccaacccaagacatgcttatcggactttatgtattaacgattggaaaccgtcgaggtatttgtgcaaatagatataatagttgcggaaactatccaaaccaaaaa
gtaaattacaataataataatcctaagtatacgaaagataaagaatctctttttctagttcctatgatgcactgggagcttatagacagaaacaaatctgtttagacagtc
ccttgtggctccgatggaaactagatcaacgcgtcattgggttaagagaagttccgattgaagttcaatatgaatctttggggacttatcgtgagatttatgcccactat
ctagtagtgggaaatagaaaaaaggaaatccgttctatatacattcgcaccactcttggtcatatttctttttatagagaaatagaggaagccatacaaggatttagtca
ggcctattcatacactatctaaacaaggaagttagattcggggatgcccctttcgaggggcattccgatttcgctagtatcatcatttttgccgcacgaatccagattga
gattgaggaaaggaagttaactaagttttcgaatcactgactcaggcccattgtcgaatcctactcagcaattgtcgaattatactcagccgaaaaaggggggtacttat
ttatggcggaacgggccaatctggtctttcagaataaagagatagatggaactgctatgaaacgacttattagcagattaatagatcatttcggaatgggatatacatc
ccatatactggatcaaataaaaacgctgggcttccatcaagccactactacatcgatttcattaggaatcgaggatcttttaacaataccctctaagggatggttagtcc
aagatgcggaacaacagagttttcttttggaaaaacactattattatggggctgtacacgcggtagaaaaattacgccaatccgttgaaatctggtatgctacaagtga
atatttgaaacacgaaatgaattcgaattttcggataacagatccttctaatccagtctatctaatgtctttttcaggagctagaggaaatgcatcgcaggtacaccaatt
agtaggtatgcgaggattaatggcggatcctcaaggacaaatgattgatttacctattcaaagcaatttacgcgagggactttctttgacagaatatataatttcctgcta
cggagcccgcaaaggggttgtagatactgctgtacgaacggcggatgctggatatcttacacgtagacttgttgaagtagttcaacatattattgtgcgtagaagaga
ttgtggtactatccaagctatttctgtgagtcctcaaaatgggatgacggaaaaactttttgtacaaacattaattggtcgtgtattagcaaacgatatatatatcggttcac
ggtgcattgccactcgaaatcaagatattggaattgggttagtcaatcgattcataaccacctttcgagcacagccatttcgagcacaaccaatatatattagaacccc
ctttacttgccggagcacatcttggatctgtcaattatgttatggtcggagttccactcatggcgatctggtcgaattgggggaagctgtaggtgttattgcgggtcaat
ctattggggagccagggactcaactaacattaagaacttttcatactggtggggtattcacagggggtactgccgaccttgtacgatccccttcaaatggaaaaatcc
aattcaatggggatttggttcaccccacacgtacccgtcatgggcagcctgcttttctatgttatatagacttgcatataactattcagagtcaggatattctacatagtgt
gactattccttcaaaaagcttgattctagtgcaaaatgatcaatatgtagaatccgaacaagtaattgcggagattcgtgccggaacgtccgctttgcattttaaagaaa
aggtacaaaaacatatttattccgaatcagatggggaaatgcactggagtaccgatgtttatcatgcgcccgaatatcaatatggtaatcttcgtcgattaccaaaaac
aagccatttatggatattgtcagtaagtatgtgcagagctagtatagcttctttttcgctccacaaggatcaagatcaaatgaatacttattccttttctgttgacggaaggt
atatctttggcctctcgatggctgatgatgaggtaagacatagactgttggatacttttggtaaaaaagatagggaaattcttgattattcaacgccggatcgaatcatgt
ccaatggtcattggaattttgtctatccttctattcttcaaaataatttggatttgttagcgaaaaagcgaagaaataggttcgccattccattacagtatcatcaagaacaa
gagaaagaaccaatatcctgttttgggatttcgattgaaatacccttttatgggtgttttacgtagaaatactatagttgcttattttgacgacccacgatacaaaaaagata
aaaagggttcaggaattgttaaatttagatataggaccctagaggacgaatataggactcgagagaaagactcagagaacgaatacgggagcccagaaaacgaa
tataggacccgagaggaagaatgtaaaaccctagaagacgaatataggactcgagaggaggagtatgaaaccctagaagatgaatatgggatcccagagaacg
aatatgaaaccctagaagatgaatatggaatcctagaggacgaatataggactcgagaggaggaatccgaggacgaatatgggagtccagagaacaaatatagg
ccccgagaggataaatatggcactttagaggaagactcagaggacgaacatgggactttagaggaagactcagaggaagactcagaggacgaatatgggaacc
cggaggaagattccgtcttaaaaaaggggggttttgattgagcatcgaggaacaaaagaatttagtctaaaataccaaaaagaagtagatcggtttttttttcattctccaa
gaactgcatatcttgccgagatcctcatccctaaaggtacttgacaatagtattattggagtggatacacaactcacaaaaaaatacaagaagtcggctgggtggattg
gttcgagtgaagagaaaaaaaaagccatacggaactaaaaatcttttctggagatattcattttcctgaagaggcggataagatattaggtggcagtttgataccactag
aaagagaaaaaaaaagattcgaaggaatcaaaaaaaaagggaaaattgggtctatgttcagtggaaaaaaaattctcaagagcaaggaaaagtattttgttttggttcga
cctgcagtcgcgtatgaaatgaacgaagggagaaatttagcaacacttttcccacaggatctcttgcaggaagagggtaatctccaacttcgacttgtcaattttatttc
tcatgaaaatagcaagttaactcaaagaatttatcatacgaatagtcaattcgttcgaacttgcttagtattgaattgggaacaagaagaaaaagaggaggctcgtgct
tcccttgttgagataagagcaaatggtctgattcgcgatttcctaagaattgggttaatcaaatccactatttcgtatacacgaaaaaggtatgatagcagaagtgcagg
actgattctccataataggttagatcgcaccaataccaattccttttattccaaggcgaagattcaatcacttagccaacatcaagaagctattggtaccttgttgaatcg
aaataaagaataccaatctttgatggttttgtcggcatccaactgttctcgaattggtttttttaagaattcaaaaaatcccaatggggtaaaagaatcgaatcctagaatt
cctattccaaaatttttggggctctttcgaaatttttcgggactcttaggtactattgcacctagtatatcgaatttttcttcatcttactatttactaacgtataatcagatcctg
ttaaaaaagcatttgctccttgacaatttgaaacaaaacttcaaagtacttcaaggacttaaacactctttaataaatgaaaatcaaaggacttcaaatttcgatagtaaca
tcatgttggatccattccagttaaattggcactttctccctcatgattcttgggaagagacatcagcaaaaattcaccttggacaatttatttgcgaaaatgtatgtctattta
aatcgcacataaaaaaatcaggtcaaattttcattgtaaatatagattcctttgttataagagcagctaagccttatttggccactacaggagcaactgttcatggtcatta
tggagaaatcctttacaaaggggataggttagttacgtttatatacgaaaaagcgagatctagtgacataacgcaaggtcttccaaaagtagaacaaatcttcgaagc
gcgttcaattgattcactatcgccgaatctcgaaaggagaattgaggattggaatgagcgtataccaagaattcttgggggtccttggggattcttgattggagctgag
ttaaccatagcccaaagtcgtatctctttggttaataagatccaaaaggtttatcgatcccaaggggtacagatccataatagacatatagagattattatacgccaagt
aacatcaaaagtgcgggtttccgaagatggaatgtctaatgtttttttcacctggggaattaattggactattgcgagcagagcgagcagggcgggctttggatgaatc
gatctattatcgggcaatcttattgggaataacaagggtttccctgaatacccaaagtttcatatctgaagcaagttttcaagaaactgctcgagttttagcaaaagctgc
cttacgaggtcgtattgattggttgaaaggcctgaaagaaaacgtagttctgggggggattatacctgttggtaccggattccaaaaatttgtgcaccgttacccacaa
gacaagaacctttatttcgaaattcaaaaaaaaaaaactatttgcgtcggaaatgagagatattttgtttctccatacagaattagtttcttctgattctgacgtaacaaacaa
tttctatgagacatcagaatcaccatttaccccatttatatgatttaaggatacataaagcagatttttttactttaaactagatttttgaccttagaacgctaagaggttagatt
ttctatttttattttaatttaaaaagaagtttagttaattcattaaggttatgcttataccatgtagaaggttccatcggaacaattattatttatttcaagctatttcggctctttctt
aatcttcgaaaagaaagaaatttcgtaatggaaaggtaggatgaaaaaaaagaaaaaatcaaaaggaagtgtggaaaaaatgacaagaagatattggaacatcaa
tttgaaagagatgatagaagcgggagttcattttggtcatggtattaagaaatggaatcctaaaatggccccttacatctcggcaaagcgtaaaggtactcatattaca
aatctcgctagaaccacccgtttttttatcagaagcttgtgatttagtttttgatgcagcaagtcagggaaaaagcttcttaattgttggtaccaaaaaaagagcagcgga
tttagtagcatcagctgcaataagggctcgttgtcattatgttaataaaaagtggttcagtggtatgttaacgaattggtcgattactaaaactagactttctcaatttagag
acttaagagcagaagaaaagatggaaaaattccaccatctcccaaaaagagatgtggcaatcttgaagagaaaattatctaccttgcaaagatatctcggcgggatc

aaatatatgacgaggttgcctgacattgtgatcgtcctcgatcagcaaaaagagtatatagctcttcgggaatgtgccattttggggattcctactatttctttagccgat
acaaattgtgacccagatctcgcgaatatatcgattccagccaacgatgacactatgacttcaattcgattgattcttaacaaattagtatttgcaatttgtgagggccgtt
ctctctatataagaaatcattgattaagaagaatagttaattcttgggcaactgcgtagatttatggaatcacttactattctttttgttttgcatagaaaaaagacgggga
atattgatatatattagagggtattgatatatattatcatctgatgtgatttcttgatatcctaaatataagattaatacttcacgttgctgagttgagaaaaagatggttgaat
caaaagaattctttttttgaagttcatttttatcaggggacaatatgaatattataccgtgttccattaaaacactcaaggggttatacgatatatcgggcgtagaagtag
gccaacacttctattggcaaataggaggtttccaaattcatgcccaagtacttatcacttcttgggtcgtaattactatcttgctaggttcagttatcatagctgttcgcaat
ccacaaaccattccaaccgatggtcagaatttcttcgaatatgtccttgagtttattcgagacttaagcaaaactcagattggagaagaatacggtccctgggttccctt
tattggaactatgttccttttatttttgtttcgaactggtcgggtgctcttttaccttggaaaattatacagttaccccatggagaattagcagcgcccacgaatgatataaa
tactactgttgctttagctttactcacgtcagcggcatatttttatgcgggtcttagcaaaaaaggattgagttatttcgagaaatatattaaaccaactccaatccttttacc
aattaacatattagaagatttcacaaaaccattatcgcttagttttcgacttttcgggaatatattggcggatgaattagtcgttgttgttcttgtttctttagtccccttagtag
ttcctataccggtcatgtttcttggattatttacaagcggtattcaagctcttatttttgcaacgttagccgcagcctatataggtgaatccatggagggtcatcattgaatt
gactagttttcaaaatagtcttttttttagcttaactcaattcatgcatggttgcggaaaattcgcttggttggaaaacaaaatagttagaattgcgtatgaatatacaatcta
gagttgtagaagagagaataggctatattacggaattgccaaacaaagtatataggcattagggaggggcggagtcaggctagatctatatcctttatgtctataagtt
cagtcatcttttgtatgggtttccactttaaggaattttttttgaatccgattcaatagaaaatgagaaaatacacaaacaaaatagaagaaacaaattgatatgggatatt
atatattcccaagttagattcattatctaatccgatatatggaatcggattccatatccaattcgatgcagcatattgttatcaattggatatcttgatttaattcctattggatc
tggattaggtcgatttccataggggttcttcctctatttcaccttttattatgaattagatgataggggaaaaaatagaaactcaaggatatcgaagaggaaagaaagaa
ggatggaatgaaagatcagttggttggaaagaaagagaaatagaataatgagtacacaaacctctaatgattagaaactaaaaaggagatctcgaagcagttcgga
gaattcagattatcgtttcaatttgtactttttagttacttctgtccaatagagcttagaaatatgaatttcttggttgattgtatccttaaccatttcttttttttgacacgaggaa
ctcatcatgaatccactaattgctgctgcttccgttattgctgctggattggccgtaggtcttgcttctattgggcctggagttggtcaaggtactgctgcaggacaagct
gtagaaggtattgcgagacagccagaagcagaaggtaaaatacgcggtactttattgcttagtctagcttttatggaagctttaacaatttatggactagttgtggcact
ggcgcttttatttgcgaacccttttgtttaatcctaaaaaagaaaacgagtcctttagattagatactttcttctttttttagtaaattggtatttgcttctgcaattccaattatat
caatactttactcctaatttattactcctagagttttctatttatggggacagacaataccccaccccaggaatagctgatttgaggatgatcaatttagaggatatgttcg
ccgtcttgcttcccgccctttgtttagggcagtggaaagtatttttccttttattttaggaattttgggaacatttcaacaaaggaggtctttcacaggtcaaacgagatcta
agacttaatctaaaagaaattactaaattgaatctatttctattaaaaaaaaattgcattaaaaaaaccgatcaaaaagggcgagcgaagtaagtgatcgaaaaactttgt
tctttgttcgtcctatctataagaggagagcatatgaaaaatgtaacccattctttcgttttttagctcactggccatccgctgggagtttcgggcttaataccgatattta
gcaacaaatctaataaatctaactgtagtggttggtgtattgatttattttggaaagggagtgtgtgcgagttgtctatttcaagaatagattggatctatccggctgcactt
tagaatatttttagtatttttcgaataaataagaaaagggtgcacgatctcgacgaattacttctgaataaatgcagaaatcatatgaaagaaccatagcatttcgcgac
ctattggtaaatcaaatcaactttgattctctatagaccaataatatgagaccattaacacggttaaagctaaactgcttgaagtctaggcaaaagggggtactctttcta
caactatattagtattagtaccaaatgctttaaacaggaaatagctaatgtagaatttatctgatatagaacactcatatcgataaaatggtttgaactatttactagaagg
ggcaccctgccctttttatccaatgccgaatcgacgacctatgtataaaaaaggagaaagtttttggatttgaagaaaaaagtaggaattctatccattttcattttccatt
tttctatttagtttttcttaatgaaattgaaaattttaactaaagggcaaatacaaataaaaaaacaactttgctgcccacgatagatttttatctaggcggaagagtcctctt
aatatttatctagtcttatatgggtttcggtatattgaaatataaacagaaaagagagggtagaggataggctcattacattaaaaaaaatatggaagtagccatagcaa
aaaaagaaaaaagggagcgtgagagccaaatgaatcgaaagattcatgtttggttcgggaagagatcataaaagttgtaaacttaatagcaaggtaatctactttcat
taaaagatttattagataatcgaaaacagaggatcttgagtactattcgaaattcggaagaattgcgtagaggaaccattgagcagctcgaaaaagctcgaattcgatt
acagaaagtcgaactagaagcggatgagtatcgaatgaatggatactctgagatagaacgagaaaaagcaaatttgattaatgccacttctattagtttggaacaatt
agaaaagtctaaaaacgaaaccctttattttgaaaaacaaagggcgatgaatcaggtccgacaacgggttttccaacaagccgtacaaggagctctaggaactctg
aatagttgtttgaataccgagttacatttccgtacgattcgtgctaatattagcattctcggggccatggaatggaagagttaaattaattaggccttgaacttctactttcc
tttagaatttaggcattatttttccccttgcttccgaaaaaaaaaatagtaaagaaacactaatggcaacccttcgagtcgacgaaattcataaaattctccgcgaacgtat
tgaacaatataatagaaaagtagggattgagaatatcggtcgcgtagttcaagtgggggatgggattgctcgtattataggtcttggtgaaataatgtcaggcgaatta
gtcgaatttgcagaagggactaggggtattgctctgaatttggaatccaaaaatgttgggattgtattaatgggcgatgggttgatgatacaagagggcagttttgtaa
aagcaacaggaagaattgctcagatacccgtgagcgaggcttacttgggtcgtgttataaatgctctggctaaacctattgatgggagaggcgaaattgtagcttcg
gaatctcgcttaattgaatctcctgctccgggtataatttccaggcgttctgtatatgaaccccttcaaacggggcttattgctatcgattcgatgattcctattgggcgcg
gtcagcgagagttaattattggggacagacaaaccggcaaaacagcagtagctacagatacaattctcaatcaaaaagggcaagatgtaatatgtgtttatgtagct
atcggtcaaagagcatcctccgtagctcaagtagtaactactttccatgaagagggggccatggaatacactattgtagtagctgaaatggcggattcccctgctaca
ttacaatacctcgctccttatacgggagcagccctggctgagtattttatgtaccgcgaacggcatactttaataatttatgatgatctctccaaacaggcacaagcttat
cgccaaatgtcccttctattaagaagacccccggccgcgaagcttacccaggggatgtttttatttgcattcacgccttttagaaagagccgctaaattaaattctctt
ttaggggaaggaagtatgactgctttaccaatagttgagactcaatctggagacgtttccgcctatattcctactaatgtaatctccattacagatggacaaatattcttat
ccgcagatctattcaatgccggaattcggcctgctattaatgtgggtatttccgtttccagagtaggatccgcggctcaaattaaagccatgaaacaagtagctggca
aatcaaaattggaattagctcaattcgcagagttacaagcctttgcacaattcgcctctgctctcgataaaacaagtcagaatcaattggcaaggggccgacgattac
gagaattgcttaaacaatcccaagcaaatcctcttccagtggaagagcagatagctactatttatatcggaacaagaggatatcttgattccttagaaattggacaggt
aaagaaatttctggatgagttacgtaaacacctaaaagatactaaacctcaattccaagaaattatatcttctagcaagacattcaccgaggaagcggaaatccttttga
aggaagctattcaggaacaactcgaacggttttcccttcaggaacaaacataaattttgcacgtctactcttgttagtagaagtagtagaggagaaatcgttgagaaa
gatttttcatttgaatcatgcaaaaaagttttcttagtttttagtatagttatttaaagaatagatagaaataagattgcgtccaataggatttgaacctataccaaaggtttag
aagacctctgtcctatccattagacaatggacgctttttcatatttcttctttcttttcatttttatcggaccaagagaaaaaactgttagaccaaaactcttttaggaaag
aaaaacaaatgcatatacaaatgtatgatgcatatatcataaagaaggaatatatatggagcgggtagtgggaatcgaacccgcaaccccacggttatgagccttgt
cagctaccaaactgttctatcctgttaaactaaagagaggggaactagtggataaaggggggttgaatacgcccctctaccatatctatacaaatagaatagtccat

ttatacagaatggtaaagagggctcttctacgatcatcaattccagaaatccatacaaatacgaaagggtattttatccttaccaactggatcttgttgcacccggtaac
aaacatgcataaaccatttctcgaagtatgtgtccggatagcccaaagtctcgatagttagctctaggtcttccggtcaaaaaacaacgtcgatgaaggcgtgtcggt
gcactattacgtggcagggattgcaatttttctcgcattttcgtttttcactcaaactcaaagggtaaactttgcttcttatcttttttttgaagatcgacgaatcaaatgata
tttctgttctaatttctgccgcttcctctctctctgaatcaaacttttttttgccataatgtgccgttcctactatctaccaagtatacggttctaatcctagatggaaaaataaat
agaaaaagaaatctaagaaggcggatcctccccctccatcaagagtaatgaactaggtgctgatacagtacaacaaaattaactaaattaaccaaacttgcctgatgt
tgaggcaatcaagaaagcggcataagtgaatatataacccacggaaaagtgagctaatcccaccaatcttgcttgcacaatggaaagagccacgggcttatctctc
cagcgaattaaattagccaaaggtgtccgttcatgagcccatgctaaagtctcaattaattcctgccaatatccacgccaggaaattaagaacataaatccagtcgcc
caaacaagatgtccaaataagaacatccacgcccataccgataaactattcatcccaaaaggattatatccattgataagttgtgaagagtttaaccataggtaatctct
taaccatcccatcaaataagtggaggattcattaaattgtgaaacgttgccctgccataatgtgatgtgtttccaatgccaataaaaagtaacccatccaatggtatttaa
catccagaaaactgccaaataaaacgcgtcccaagcagaaatatcacaagtaccgccgcgaccagggccgtcgcaaggaaaactatatccaaaatctttttttatcc
ggcattaatttggaaccgcgtgcatctaaagcaccctttactaaaatcaatgtagttgtatgcaaacctaaagcaatagcatgatgaaccaagaaatctccaggtccta
ttgttaagaaaagcgaattactattctcgttaacagcattcaaccatccgggtaaccatagggttcgacccgcattgaaagcgggaccgctcgttgaagataagagta
tatcgaacccatatgtcgtcttaccatgagccgattgtatccattgagcaaatataggttcgatcaagatttgcttttctggagtaccaaaagcaagcatgacgtcattat
gaacataaaggcccaaggtatggaatcctaggaagagactagcccaacttaaatgagatatgatagcttctttatggtctaacattcttgccaatacattatcctcattct
gttccggattgtaatctctaatgaaaaaaatagctccatgagcaaaagcccctgtcatgatgaaccctgcaatgtattggtgatgagtatataaagctgcttgagtagta
aagtcttgtgctatgaatgcataagaaggtaaagagtacatatgttgagctactaaggaagtaataacccctaaagaagctagagcaagacctaattgaaaatgaatc
gaattattgattgtgtcgtaaaggcccttatgcccacgccctaatcgacccccccggaggagtatgtgcttctaaaagatctttgatactgtgcccaattccgaagttagt
tcgatacatatgaccggcaatgagaaaaataaatgcaatagctaaatgatgatgagcaatatcggtcagccacaaactttgtgtttgtggatggaatcccccaagaa
gagttagaatagcagttcccgctccttgagtggtaccaaataaatgattactcgaatcggggttttgggcataaagattccactgacccgtcagaaggggtcccaac
ccctgtggatagggtaatacatctaagaaattattccatcgaacgtactctcccctggatgctggaatagcgacatgaactaaatgtcctgtccaagccaaagaactta
ccccgaaaagtcctgacaaatgatgattgagacgagattccgcgtttttgaaccacgaaaggcttggtttccatttgggttgtagatgtaaccaacccccctattaagga
tagggtagaaagaaataatagaaaaagagctcctgtataaagatcttcattggtccgtaatccaattgtataccaccactgataaaccccagaataggcgatattcact
ggaccggcggcgcctcctcgagtaaaggcttccacagcgggttgaccaaaatgaggatcccaaatcgcatgagcaataggtcttacgtgtaaaggatcctgtatc
catgattcaaaatttccttgccaagcgacatgaaacagatttccggacgtccatagaaagattattgctaattgcccaaagtgagaagcaaaaatgttctgataaagac
gttcctcagtaatatcatcatgactttcgaaatcatgtgcggtagcaataccaaaccaaatacgacgagtagtggggtcctgagctaagccttggctaaacctgggaa
atcttaattccataatgcctttcaaatcctcctagccactatcctactgcaataattctcgctaagaagaatgcccatgttgtggcaattccacccagaaggtaatgggtt
actcctacagcacgtccttgtataatgctcaaggctctaggctgagtagcaggagcaacttttaatttgttatgagcccaaacgatagattcaatcagttcttgccaata
accacggccgctgaataaaaacattaaactgaaggcccagacaaaatgagcacctaagaaaaaaagaccatatgcggataatgaagaaccataagactgaatta
cttgggatgcctgtgcccacaagaaatctcggagccacccattaatcgtaatggaactctgtgcaaagtttcccccctgtgatatgagttaccaccccttgatcacttata
gtaccccaaacatccgactgcattttccaactgaaatggaaaatgactaccgaaatggaattgtacatccagaatagacctaagaaaacatgatcccaggcggatac
ttgacatgttccccctcgcccaggcccatcgcaagggaagcgaaaaccaagatttgctttatcgggtatcaaacgggaactgcgagcaaataaaacacctttcaaa
agtattaatacagtcacatggattgtaaatgcgtgaatgtgatggactaaaaaatctgcggttcctaatggaataggtaacaaagcaactttgccgcctactgctacta
actcgccacctccccacgttaagctggtacttgttgttgcaccaggagctgttacgctaggcgcgccagcatggagattttgtacccattgagcaaagatcggttgta
attgtatggcggtatccgaaaacatatcttggggacgacctaaagcactcatggtatcattatgaatgtataaaccaaaactgtgaaaacctagaaatatacataccca
gttaaggtgggatatgattgcatcacggtgtctaaggacacgatctaatagatcgttgtatcgagtcgttggatcatagtctcttaccataaaaatggctgcatgtgcag
cagcaccaactattagaaatccgccaatccacatgtggtgtgtgaacaaggaaagttgtgtaccatagtcagtagctaggtatggatagggggggcatagagtacat
atgatgagctacaacaatggttgtagagcctagcatagctaggttaagagataattgagcatgccatgacgttgttaggatttcatagagacctttatggccctgtcctg
taaatgggcctttatgagcctccaaaatatctttcaggccatgaccaataccccagttggtcctatacatatgacctgcgatcaggaaaagaatagcaatagctaaatg
atggtgcgcaatatcgctcaaccatagaccgccggttattggatctagccctccgcgaaaactaagaaattctgcgtatttggaccaattcaaggtgaaaaaagggg
ttgctccttcggcaaaactaggataaagttgagccaaaaggtcgcgattcaagataaattcatgaggaagtggtatctccttaggatcaaccccagcgtcaagaaatt
ggttaatcggtaaagatacgtggatttggtgccccgcccaagaaagagacccaagtcctaataaccctgctaagtgatgattcaacatggattctacgtcttggaacc
aggctaatttgggagcggctttgtgataatggaaccacccagcaaaaagcattaacgatgcaaaaatcaatgcaccgattgcggtacaatagagttgtaattcacta
gttattccggatgctcgccaaatctgaaaaaacccggaggttatttggattcctcggaaacccccgcctacatcaccattcaatatttcttgccctacgattggccaaac
tacctgagcactgggtccaatgtgagtaggatcacttagccatgcttcataattggaaaaacgggcaccatggaagtacatgccactcaaccaaagaaagataatg
gagagttgaccgaaatgagcactaaagacttttcgagagatctcctccaaatcacctgtatgactatcgaaatcgtgagcatcagcatgtaggttccagatccaagtg
gtagtatcagggcccttagctattgttcttgagaaatggccgggtctggcccattcctcaaaagatgtttttacaggatccctatccacaacaatttttacttctggttccg
gcgaacgaatcatcattaagtcctcctctttccggacaagacatacaaagagacccgccaactttttagtgaacctttgaaagatagatattatgattagtccttttcttta
ctatctaccgtccttctattttttttttagttattcactggagcaattatatattgaagtcaatctgaggcaagtgttcggatctattatgacataaggattgggtgcctaacgg
acttttttatcttggatttctccacgtaacaaaaaaacctttttttaatttaaaaagctagtctattttttttaagagtataagtccctatctatatctactttccttgagcataatat
agattttttattcgattccaaattccaagataactcattagaattattaataagatggtcctgatatattagcaatatttatattgccccttttttattcgctttattacttctattcta
gaccctatcgtttatccttatgaaatataatataaaatagaaggcagaggaaagagatataatgaaattcttgattcgatcttccgacctaatttatttgattaatggatcaa
caaccaaaccccccatttctgaaaaaggagagtggtcttattcaaattcaaagcgcttcgtaatcttcaaccagttctgtgcttcaatataatttcccggagtaagcgcta
tagcttgtttccaatactcagcagcttgatcaaaccaagcttctgcaatttctgaatcaccctgtagaatggcctgttctccccggtcggaataggtagttccttcccta
gaaccgtacttgagagtttcctacctcatacggctcagaaattgctatcttaatttcccttgtcttaactgaattcgatttctcaaaaatcgatccaatttcttcttgggttacg
cagaagaagttaattacctaagtttcaaaccctaaattttgatcaataatcagtttgatcttttctcccacctgcagaaaaatgaagcatagatagacctatatccttcgtc
cgaattttctgaaaggtaactatctcggtttcatatatgaaatttctatagaatccttgaaaaagactttttccccataagcaagaaaaaagaacttactatctttgggatct

gatactacaccgctgcttaatcccttagtggatcggctctattacataagcggattcctaaattttgccccatatcatgggataagtaagcagtttttttagttgtatcgac
ccagtcggtcactaattgatctttacggtgctttctctatcaatttgagaactctatccatagagtagtataggccatactttctttcttcctattttgattctcgtgaagtgtcc
tttcttcctacagctgatagggcaaaatcgttgttttgacgatccctatgtagaaagccccctttctagtaaatactagaaaatttgatcctttcttatttttctttctatagtg
gagatagtcgcacgtaatgacagatcacggccatattattaaaagcttgtggtaagaaggggtttcgttctagtgcccggaaataatattccaaagcctttgtatgctct
ccattgcttgtgtgtataaggcctatgttatagagtatataacttcgatcatagggatcaatttctagtcgcgtagcttcataataattttgcaaagcttccgcataatttcctt
cggattgagccaacatccgttacggtcgttcgttctattcaaaaaatctccgttccaaaaccgtacatgaggttttcacctcatacggctcctcccttctgtacatagtact
aagcgaaaaaatctagagaataaaaatagaattagttccatctcattatggaccgaaaggggctggtatttttccaagaaatctctagccaaccttcccacaagaggtt
tttcttaacaccaatgaattctattaatgctagaggaaaacgatagctccaagaatttctttgttctcaacgcctcctatttagaggaattagccacttcaacgatctttgat
ggttataggggtatccaaagtacaaacttgatggttgtttgttatcccaaccattcttcccagccctgataccaatcaggaaagggttaatttctaacaaagtttttctcttg
ttgattcctatttctaggtgtagtgcttttatcccctatgctacctattagtactagtagagtaggattagcctgtaatacagaacctatcctgtaggtgtaacctttcgctca
atactaaaatctacaattgaagcatccgaggccgcgtcagtcgaggatacacgacagaaggagttgttagttcacctcaccttccctaagcgtgggtttcccttacta
atttggttttctctccgcgaaccccccgctctttctcgtaaaaccggggtgtaggtagggctaaaaaaacaaaaaaaaagagtcaaatcgcaccatctctataataagtaa
atgcccttttttccccggaggttgtcggaattattcgcaataaaatattggctacaattgagaaggtcttatcaatgaaatttccatttatacgggatctaggcataattccc
aacccattctatcattctatatagaattcttttcattccttcacaaaataacataaaaaaaaaaacaaatccattcaattcttataaatcgatccctatgctccaaatggataag
agaggtatttctgctcagcccaaattctctctttttccttctgtttgaacaagaagagatagaaaatatttgactaagattggatttcattccactttcctatttcttttctcaac
aacaacttctctcatcaactatttcgcattttcaaagtcattaatcgtcccataccctattttctatttgattgtatggggtaggataccttatgcaaacagaattctagggttc
cttttttatcgaataagaagaattcttccattctctttttctttggtttggggaaaacccaaactaaaacttttcgagggagcggaattcctagtaaaaaaatcctggaccc
gtccacttagatgaaaaggaatttttctatctaatagaacaatggaactctcgcgcggttgtggttgtacctgtactgcaggaataggaaaactcgctattcactcagttt
tttttccataataagattatggaggagagatggccgagcggttcaaggcgtagcattggaactgctatgtagacttttgtttaccgagggttcgaatccctctctttccgt
ttttcttaattcagcaacgttaatgattacaatgtatcaaatcaaatgacaatttattccagcaataatacaataccttttatttaatagaaattctctataccaaattactatggt
atgtaaaatacacatagaggaaagaacaaaaaacaaaaaggaatcctagggttaatccatttctgttaggtgaatgggaaaatacgaattaagagccttaggtcgatt
tagttcggggaaaggggaaggggaagaaaattctatgaacctttccgtttttccttaagttcaagtttgacgagagtaatattctacaactaacaactcatttattttgaga
ccgacccacttcctatccaggattttttttactagtcctttatattgcaatgtgtcaatcgtcaaatgctttggcaatttccccgggtcggatgaagcaatagaattttgaac
cagacgttttgatctttggttatccttcgtagtaataatatctcggggtttgcaacgaaaacttggtatatcgactatacgaccattaactaaaatatgtctatggttaactaa
ttgccgggcctcgggaatggttgaagccatacctaatcgaaaaaggatattatccaaacgcatttcaagtaattgtagtaaaacctgacctgttgaactttttgcttttcc
agcgatatgtacatatctaagtaattgtcgttctgtcagaccataatgaaaacgcaatttctgtttttcttgaagacgaatacgatattgttcttttttcccagaatggaatttc
tttttcagattacttccggatttaggtgtttttctagtgagtcctggtaaagctcccagacggcgtattttttttaaaacgaggtcctcgataacgggacatgaagactccttt
ttttattgaaatttcattttacacaattaatttcattgtatttacattacagaatacatcgaaattaaaactgaattaaagtaaaggataaacagagtaaaatctactaaaagta
ccacaaaaaaatggaatttcatcaacatctgaatttttgtatatatattatttattttattgttttgtatctagcaaaattgtagggtagaacgacataatagatcctggcttctc
catttaattcggagaaaaagaggtatttttgttcatggaacattgatagagaaaaaagccgactatcggatttgaaccgatgaccctcgcattacaaatgcgatgctct
aacctctgagctaagtgggcttacataacagaaatagtgtaacaaatagaaatatgtatagtataggaaatccgtaaaatgtcagatcttaattattaatcttagctattaa
ctagttcgaaattggaagttctacttagaaaaaaatactagaacttcataaaataaagttagatagattttttttgaactttctttttttctctaattcgcaaatctatttttctaata
gaatctattccaatttctatattgaatttgattttagatatttttaatttgatatggctcggacgaataatctaatacatggataaagaataaataatatatatacgaaaacata
ataaagagaacatgcgaatttcttgtattttcagtccatcattatagacatttttttgagatattttgttttttttgttatttgctaataatttaatgattaatatttcactaaggagaa
catagaatcatagcaaatgaaattgctaattctgattagcaaaaaaaaaagaatgaatatcaagcgttatagtatgattttgaatactctaaaaaaggaacgcggtaggt
gggggagagaaaaaccttgggatatattgattcgcattgaattgcaaatacatcaacgatagaatcaattcaatgctgaattgcaataagcggagtctctcaactaga
gacgaaccgctagactacatagagtaatgaattcaacgattcaaaaaaaaactaacagatggaggaaattgcacaaggaatcctggtctcaaagaaaaagaaaatg
gggatatggcgaaatcggtagacgctacggacttgattgtattgagccttggtatggaaacctgctaagtggcaacttccaaattcagagaaaccctggaattaaaa
aaggggcaatcctgagccaaatccatgttttgagaaaacaagcggttctcgaactagaacccaaaggaaaaggataggtgcagagactcaatggaagctgttcta
acgaatcgagttaattacgttgtgttgctagcggaactcccttctaaattagggaaagaagggctttcgaaatctaatacacacgtatagatactggcatagcaaacga
ttaatcacagaactcatatcataatataggttctttaattcttttttaaaatgaaaataggaatgattatgaaatagaaaattcataattttttttagaattgttgtgaatccattcc
aatcgaatattgagtaatcaaatccttcaattcatagttttcgaaatctttttaaaagcggattaatcggacgaggataaagagagagtcccattctacatgtcaatactg
acaacaatgaaatttctagtaaaaggaaaatccgtcgactttctaagtcgtgagggttcaagtccctctatccccaaaccctcttttattccctaactctagtatttatcctg
tttttttttattaataggtttaagattcaatggaatacatttctttttttattatagtatcggcaaggaatgtcgattattaactcgatatttaaatattattaaataggctttctttgtac
aatgcataggactgccccctccccatttccaaattttggatattgacatagatacaaatactctactaggatgatgcacaagaaaaggtcaggatagctcagttggtag
agcagaggactgaaaatcctcgtgtcaccagttcaaatctggttcctggcacagaaaaaagggatcttccgaataggtattgatacaaatacctcgagatgggttgg
gatacatattcgttaataatatagatagagtatgattttttcatctaagtagataaatctctaaatagaggcacttctttttctgcatttttgaatttcttaattttctattccgctatt
ccgacaaatattttttttattcttgcttatcttatcttactttcctagttgttctaagtaatgcacgcggtacaaagttcgtggtagggaacttctttgagtcatcatatttttctgtt
catacgaaggaaatgaatatgtgattttccaacgaaatgaagccctttttttgcttagtctatctgaaccttttgtataattggaattaattagaatgtaataggtattctgtttc
atctaggaacagagcgtaaaaatattccttgacttgcataaaatctggagttgtgttgtataagtgagcatgaatttcttatcattcaatgagcatcttgtatttcatagaaa
ttgggggttatatagtccttacgtaagggccagcctatccaactttcaggcattaggatacgtttaaggcgtggatgattatcataagagattcccaccatatcaaaaga
ttcgcgttcttgaaaatcggaacttctccaaatccagaagacagatgggattctaggattatccttttgggcaaagacttttatgcatacttcttctgggttatctataccat
actgtattctcgtaagatgatacacgctagctaaagatccaccgggtgcaacgtcataagcacattgggaacgtaaataattgtaaccatatacatataaaatgacag
caatggaatcccaatcctctgcttttatttgcaaagtctctattcctcgatgatcgaagcccaaagatctatgaaccacctcatgtttgactagccaattagataaccaac
cctgctgcattatcttgatctctcctcctttgtataaatatttcgcagttcgaatgcaagtttgaaatattgccctgctctttctttttcggcacaaagagctcctcctaattcac
taatttgtaggaagatactggacttttggatttgaaaaaagtttcagaagatatgtctaaagtagatggtgattgatagagcaattcttgctcataagttccagtattagtac

tgcgccgaacataaagcttgtggctggtagtaaaacatcgatttttcttttgagatagagttcgatcctcaactatttctcgcgatatcttcttacgaagttttgttagggca
tctataacagcctctggtttaggtgggcaacccggcaagtagacgtccacaggaattaacttatcaactccccgaacagtactataggaatccgtactgaacattcca
ccagtaatagtacaagctcccatagcaatgacgtattttggttcaggcatttgctcatataatctcactaaagagggagccattttcattgttactgtaccggctgttaaaa
ttaggtccgcttgcctaggacttgatcttggtaccaatccataacgatcaaagtcgaatcgtgagcctattaatgaagcaaattcaatgaaacaacaactggtaccata
tagaaggggccataaactagagagtcttgaccaattcgaaagatcttttagtgtagttgaaataacggaattggaacttgtttggtcaagtaggggaaactcaatcaaa
ctcataactgtcttaatggaatcttttccttctttttttttttgtctgaatattcagttaagaccattccaaggctccttttcgccatgcataaactaaaccaacaactaggata
agcacgaaaatgaaagcttcgataaaaacggatatacccaatacgtcgaaactcattgcccaagggtagagaaagaccgtttccacatcaaaaacaacaaaaact
agcgcaaacatgtaatagcgtattcggaattgtaaccaagcccccccccatgggttctatacccgattcataactagaaagcttctctggtccttcacgaaccggagct
aaaagtgctgaaatccaaaatgctaaaataggaataaggcttgctattattagaaatgcccaaaaaatatcatattcgtgaagcagaaacataaatgtactcccattaat
gtggaataggcggaactgaattagtcaattcaagtcagcattgtcaatttatacagaatttctctcttttcctcggtgaaacaaggatcggtttttctcaaaccaaagggc
ttagtttagcctttgtttcctctttgccacgtcttctttaaagattcatccaatggaatcccgactccctttctttttgatttcctttctatttaggtatggtggagacataattctta
tagaaacaaaactctctcgcttcactttgtctcattttctctagaatctctagaaaaaggaataaaaacgaaaatactacgaattagagcctaattaagataggatgacta
atgtatgcagcctaatgaggagtaattctataaaaataaagaactctatttcagaacgtagatcgatttagatttaggtaatctatagatatagataagcaaagtaatata
cttcaaacaaagtaggaattcgcaagatggagaacatcttgcagttgatttgatagaaattcatttttcttttcctgtctctataattttcgatgaatgagcctctggtaatcc
ttttatctctattttatggcgcaggcgcctgtccagtctataaacaagtactaatagggaaatgaaaactatactaaaggaaacgtaggatatctctcctaaaatctaaaa
aaggacatattagggctatacggattcgaaccgtagaccttctcggtaaaacagatcaaacggattattatcgaaatgattcgaactgtttcaaagacccaacatgcat
ttttttgcattgggctcttttatcaactgatagaaagatcagttagtccaccatagtttttctttacggaaagataatgagatggctccctgcgctctgattgattatttgtatta
tgatctatctaagagcaataccaaagtgtttcaaaggaggattaccttgacttaggtctgcctccggcctaaattaaatcaacctaagtgaaatagagtctctatcgttcc
gctacaagagttgactatgagacttcatacaccttaaagttcatagaacgaaaagaattttttttggaggcccttatcctcattaagcctagcatttagtgggctggatattt
accttatcaactagcaaatcaataaaggttctatttgtttaggcacctggattggtacctgaatcggactgaaccaactatttgtcaggcgactgttctcctattctctcga
atccatgaagtaagacattgattttgcaagaagatccactatgttcattgcataataagctcccttgaaaagcattggcgcacgtgtaaacgagttgctctaccgaactg
agctatagcccttgtcagagatatcttaatatatagagaatttcttgtcaagatgaatattctctaatagtagaggatatcctttgatctgtttactataataacataccaata
acgaagcggtatttgcttataaaaaggattcgatctataatcgatcgaagtaaagggtcttcctttgtggtgataaattgcctacttaactcagtggttagagtattgctttc
atacggcgggagtcattggttcaaatccaatagtaggtaggtaggtagaaaaattactagatagcattggccctacttcgcttcgctatctaataatttttttctacccctct
tccctttttctttgtatcaactaaaccgttgggttgtcttcaattagatgggggaatccaattaacagcctcgactcgtatcctagctcgtctgagagctaccttcgcttcaa
ccaattctttcgtaccctcagctctactcacgttagcttcggctatttcaagtgcctgttgagcttcttccggatcaatgtcactacccagttccgcatcatttcctaaaatg
atgatctcattattaactattctggcaaaaccgctccacagaaccgccgttaaccattgatcgttgaggaggcgtattctcaagggacccatatctacagctgtgttaat
gggggcgtggtttggtaatacgccaatttggccactattagtagataaaatgatttctttcacttcacaatcccaaataattcgcttaggagtcagtacataaagatttaat
ttcatttcttcaatttgttctcctcttctaagtttatagctttcgtgctagcttcatcgatgttacccaccaaataaaaagcctgttcgggtaggccgtctaattctccggaaa
ggattagttgaaatcccctaatagtttctgcaagaccaacatactttcccggagaaccggtaaaaacttctgccacaaaaaacggttgtgataagaagcgctcaatttt
tcgtgctcttgctacagttaaacgatcctcctccgataattcatccaacccaagaattgcgataatgtcctgaagttctttgtaacgttgtaaagtttgcttaactctttgcg
cagtttcataatgttcgttgccaacgatccgaggttgtaacatagttgaggttgaatctaaaggatctactgcaggataaatccctttggaagctaatcctctggaaagt
acggtagtagcatccaaatgtgcaaatgttgtagcaggagcagggtcggtcaaatcgtccgcaggtacataaaccgcttggatcgaagttatagatcccttttttagta
gaagtaattctttcttgcaaagaacccatttctgtactaagagtaggttgataacccactgcagagggcattctccctaataaggcagatacctccgatcctgcttgaac
aaaacgaaagatattatcgatgaatagaagcacgtcttgcttattaacatctcggaaatattctgccatagttagggcagtcaaaccaactctcatacgagctcctggc
ggttcattcatttggccatagactagagctacctttgattcctcaagatttttttcattaattactccagattccttcatttccatataaagatcatttccttcacgagtccgttcc
cctactccgccaaatacggatacgcccccgtgagctttagcaatattgttgattaattccatgatgagtactgttttacctactccagctcccccaaatagtccgatttttc
ctccacgccgataaggagctaaaagatcgaccaccttaataccagtttcaaagatggataatttcgtatctaactcgataaaggcgggcgcggatctatgaataggg
aatgttgcactagtatctacaggacccaaattgtcaacaggctccccaagaacgttgaaaattcgtccaagagtagctccaccgacaggaacactgagaggagctc
ccgtgtcaatcacttccattcctctcatcaacccatctgtagcactcatagctacagctctaactcgattatttcctaataattgttgtacctcacaagttacattaatttgctt
accgtcagtgtctcgactcttgactaccaaagcattataaatataaggtaacttgcccgggggaaaagtgacatccagcacgggtccaataatttgatcgatacgccc
tgtactttttttcttcaattgtagaaaccccgggacgagaagtagtaggattggttctcataattatcacataattttcaaaaaaaaggaatttatcgaaattttgatttttttctt
gttgaataatgccaaatcaacaccaaaaaaaatatccaaaaatccaaaagtcaaaaggaaatgaattagttaattcaataagagagaaaaggggaccagcacttgatt
tcgttgcccaaacgaatcccattcaatcgtttactcatggaatgagtccgtcggaaagttcaatcaatctttttttcatatacattttgccttttgtaaacgatttgtgcctact
ctactttcttatctaggacttcgatatacaaaatatatactactgtgaagcatagattgctgtcaacagagaattttcgtagtatttaggtatttccactcaaaataagaaaa
gggggtctattaagaacttaataaggattagaagttgatttggggttgcgctatatctattaaagagtatacaataaagatggatttggtgaatcaaatccatggtttaata
acgaagcatgttaacttaccataacaacaactcaattcttatcgaattcctatagtagaattcctatagcatagaatgtacacagggtgtacccattatatatgaatgaaa
catattatatgaatgaaacatattcattaacttaagcatgccccccattttctttaatgagttgatattaattgaatatctttttttttaagatttttgcaaaggtttcatttacgccta
atccatatcgagtagaccctgtcgttgtgagaattcttaattcatgagttgtagggagggacgtatgtcaccacaaacagaaactaaagcaagtgttggatttaaagct
ggtgttaaggattataaattgacttactacaccccggagtacgaaaccaaggacactgatatcttggcagcattccgagtaactcctcagccgggggttccgcccga
agaagcaggggctgcagtagctgccgaatcttctactggtacatggacaactgtttggactgatggacttaccagtcttgatcgttacaaaggccgatgctatcacat
cgagcccgttgttggggaggataatcaatatatcgcttatgtagcttatccattagacctatttgaagagggttctgttactaacatgtttacttccattgtgggtaacgtat
ttggtttcaaagccctacgcgctctacgtctggaggatctgcgaattcccccctacttattcaaaaactttccaaggtccgcctcatggtatccaagttgaaagggataa
gttgaacaaatacggtcgtcctttattgggatgtactattaaaccaaaattgggattatctgcaaaaaaattatggtagagcatgttatgagtgtctacgcggtggacttga
ttttaccaaagatgatgaaaacgtaaactcacaaccatttatgcgttggagggaccgttttgtcttttgtgccgaagctatttataaatcacaggccgaaaccggtgaaa
ttaaggggcattacttgaatgcgactgcaggtacatgcgaagaaatgattaaaagagctgtatttgcgagggaattaggggttcctattgtaatgcatgactacttaac

cgggggattcaccgcaaatactagtttggctcattattgccgcgacaacggcctacttcttcacattcaccgagcaatgcatgcagttattgatagacagaaaaatcat
ggtatgcatttccgtgtattagctaaagcattgcgtatgtctgggggagatcatatccacgctggtacagtagtaggtaagttagaaggggaacgcgaaatgacttta
ggttttgttgatttattgcgcgatgattttattgaaaaagatcgtgctcgcggtatctttttcactcaggactgggtatccatgccaggtgttataccggtggcttcagggg
gtattcatgtttggcatatgccagctctgaccgaaatctttggagatgattctgtattgcaatttggtggaggaactttaggacatccttggggtaatgcacctggtgcag
cagctaatcgggtggctttagaagcctgtgtacaagctcgtaacgaagggcgcgatcttgctcgtgaaggtaatgaaattatccgatcagcttgcaaatggagtcct
gaactagccgcagcttgtgaaatatggaaagcgatcaaattcgagttcgagccggtagataaactagatagctagactaagtggataaaattagatagaaaaaagg
tctaaataaaaaagaagagaaatagaaagatcaaaaatcagttacgaaaatgcagtaattcttctttttcttctaattgattgcaattaaactcggctcaatctttttcaga
ttgagccgagtttaaatagattttgatacgatcatgagacttgacaaatcgggattcctctattctatatatttagaagatataaaggtataatacaataaataaatacaaat
atagtattatcatatgataatggaatcaaatacgcagtatttacagaaaaagttttcatttattgggaaagaatcaatgacatacaatgcattacagacgtatgatcattac
cctttaaccgggttattctattccacttctagatagagaaaaaaactaaaggagaatgaatgaaaaaagacagagtttggaagttagacccctttctaagactctctttc
aaaaaagaggacattttgaaacttttaacaggcacaatcgtgagtcaacaagtgactcgaaatgctcgtaagaaaagagaattgattttcaaaatggtagaactagat
gacgaagttttctataaccttgatgaagaggtagttttagtttacgactccgatcaagagcgagaagtctttcatttcagattggactgctatagaatctggacccatcgt
agagacgttcaaaaggctcctgatgataagaatcatactttcgcggaactccatggggctatgggcttcaacgcggtagacgttttgttccgaatttttctggagcaaa
cctccgacccaacgatacaatgaatttttctattcacaaagaaaaaagattcggaatcgcaatgctactatacgcacccggaggaatattcggaattatattcttatata
atccatttttgcgcggggtcttactaacaggacttcgctgcacgagacgaggattcgtcgaaaagctaaggtcatccggtattttcatacccttgggggtggtatatg
gccttacaaagtctaaggggtagtatgagatctgtagtaggtaaaagaatttgtcccttgattgagtatgctattttccctcctttaccgcgcattattgtatatgcttctag
aagagcacgtatgcagagaggaaattacagtttaataaaaaagcctaaaaaagtttcaactttacggcaatatcaatcaactaaaagtcctatgtatcaatccttacag
cggatttgtggggtccgagagtggttgaataagtattgcatgtggaaggaagtagacgaaaaagattttggattcgaaataggcgcattcgactaagtcgtactttga
atccaatttcaagttcgattagaaggatagaaaggccgcgaggatcggaaaagaaaaatcaaatctttttaattgcttctcctttttgctattttcttattatttatataatcc
attcgattcttttttttagaatactttagtattctaaaaaaaagtattctataaaaaatctttttttgcaaactcaaaaatacaatagtcaatattcgttataatagatatacttaat
tatatcataagaatcttaagatatttttgaatagatcaaatcgaatagatagaaatagtaaatttgaatggagacacctattctatgatggattttaacttaccctctattttc
gtgcctttagtaggcttagtatttccggcaattgcaatggcttctttatttctttatgtgcagaaaaataagattgtctagaaccgacgggaccgaattttctcaatgtatttc
cacacaggatcataatacggatcttttgtagtgtaagtaatataatatggtatgttatgtggctctttctacacacaaatgcaaacccgctatggatggggattatggatg
cggatataggctacgagcataaatgcatgcatatgcggaaccgggtatagcgagttttttttaagtggatcaacaaatactttttgaatagaaagtcaatgtatctaacca
attattttacaggagtatctagttggcgaaggcgatttcagaatcaaaaaaagtaaagtcaaaatcatttagcttattctctcaatttcaatcgaccgctgttagtatatcta
atatgaattggcgatcagaacacatatggatagaacttctaaaaggttctcgaaaaagaggtaattttttctgggcctgtattctttttctaggttcactaggattcttagcg
gttggggcttccagttatcttggtaagaatattatatctgtacttccatctcaacaaattctttttttccacagggggtcgtgatgtctttctacggaatcgcaggcctattc
attagcgcttacttgtggtgcactattttgtggaatgtaggcagtggttatgaccgattcgatagaaaagagggagtagtgtgcatttttcgttggggattccctggaata
aaacgtcgcgtcttccttcgattccttatgcgggatatccaatcaattagaattcaggttaaagaaggtctttttcctcgtcgtatcctttatatggaaatccggggccagg
gggccattcccttgactcgtactgatgagaagtttttttactccacgagaaattgaacaaaaagctgccgaattggcttatttcttgcgcataccaatggaagtattttgag
taccaattgaatttttttgaaatgaattgaatgaagaagaattggaagaagaaaagttttctcaacacgagggaaaaggtcccttcgaaattgcattattgtaaggggatt
ttgagtatttatctaaaggaaggaacaaatgaggataagagaaaattgcttctaatttgtccaagtgggatatatggcgtaatattcttccattttcatccgaaaggacttt
tttctatttctctattccactccatctagatctaagaaagaactcaatgcaatgaaattctactagtatagaaaaaagaggaatagatacaaggtctcaaaccttgttatag
aatttttccttcaaagaaaaagaaatatcatatagagtcagcgaataaagcggattcattaacaactcaatttacagatcaaaaatgaaaaaaaagaaagcattgccttc
tttcctatatcttgtatttatcgtacttttgccttggggagtctctttctcttttaacaaatgtctggaactttggattaagaattggtggaataccaggcaatcccaaactctct
taactgctattcaagagaaaagagttctagaaagattcatggaattagaagaccttttttatcttggacgaaatgataaaagagaaaccgaatacacatgtacaaaaccc
ccctataggaatacgcaaggaaataatacaattggccaaaatagataatgagggtcatctccatatcattttgcatttctcgacaaatataatctgtttggctattctaagt
ggttcttttttttctgggtaaagaggaacttgttattttgaattcttgggttcaggaattcttctataacttaaatgactcagtaaaagctttttttattcttttagttactgattttttttg
ttggatttcactccacccgcggttgggaactactaattcgttgggtctataacgatcttggatgggttcctaacgagctaattttcactatttttgtttgtagttttcctgtgatt
ctagatacatgtttgaaattttgggtcttttttttgtttaaaccgcctatctccttcgcttgtagtcatttatcattcaattagtgaagcataaactcatttgatctcctgatattaat
caaaattagcatccttttttcctttagaaagaaagcctttttccattttagcaaaattcttttttttctatttctacctgctcaaggtattcatcattccagtacaactgttgcagtag
aatgacaacagattcgtgtatagggaactagattagcttagctacctatctaatttattgtagaaattccgggatctgtgattggacatggaaaatagaaatactttttctt
gggtaaaggaacagatgactcgatcgatttctgtatcgatcatgatatacgtaataactcggacatctatttcaaatgcatatcccatttttgcgcagcaaggttatgaaa
acccacgagaagcaactggacgaattgtatgtgccaattgccatttagcgaataagcctgtggatattgaagttccccaagcagtgcttcccgatactgtatttgaag
cagttcttcgaattccttatgatatgcaactgaaacaagttcttgctaatgggaaaaagggagggttgaatgtgggtgctgttcttattttgcccgagggattcgaattag
cgccgcccgaccgtatttctcctgagttgaaagaaaagataggaaatctctcttttcagagttatcgtcccaataaaaaaaaatattcttgtgataggccctgttcccggt
aagaaatatagtgaaatcgtctttcccattctttcccccgaccctgctatgaagaaagacgttcatttcttaaaatatcccatatatgtgggggggaaaccgaggaaggg
gacagatctatcctgatggtagcaagagtaacaatacggtctataatgcaacgtcaacaggtgtagtaagaaaaaatactgcgtaaagaaaagggggggatatgaaat
atccatagtcgatgcatcggatggacgccaagtgattgatcttatacctcccggtccagaacttcttgtttcagagggggaatcgatcaagcttgatcaaccattaaca
agtaatcctaatgtgggagggtttggtcaggggggatgcagaaatagtgcttcaggatccattacgcgttcaaggccttttgttcttcttcgcatccgttattttggcacaa
gtttttttggttctcaaaaagaaacagtttgaaaaggttcaattgtacgaaatgaatttctaggtcccgggatttcttaccatcaagttggtaaaaagccgcgatttattgg
cgattgctagaattctctatgatccattttggaaatcttttttttgtttagactattttttgtttgcgagatgtcttgtctgcaattccttatttctatcatctcatgcaaaacgagga
gaatgcaaggcaagggcgagaacaataaaaggaacaaatccttttaggagggattgctggtcttcttaatcctctattgggcacaagaaaaaggctttttttgccttttt
cttgtgtcgattcttcttgtatcgaaatatgaatctttttttcttcctattcggcaaagattactatttcttctttatttgggtctgtctcttgaacctcttttgcttaggttcaggcgat
gtagtggacaaaaagagggaaagaaaatatggcgggggacaaattttttgtgaccaaatagaattgcttgacttgttcaattaagttcaatttagaacttacagaaattt
tgcaaaaaaaaacggatactcttccattgaaggctgtttttttatttttaggctagttgagtagttttaattaaggttttattagtttattactctaaattaaatcaatgatttacaag

agacttcctccggggaataaaatattggatcctcgattgatcctttctttctcctcgcttcataaaagtgaattaatttcattggcgagggggttataaatcaactgatgga
ttacttcactaacattattaacaaacaaaattaacaaacaaaacgaataaatagagggattctgaccatcagagcaaaggctttctctttgttatttttacaaatcaaaata
ggaaacccgtttgtaggttatggaatagattaaaaaagtcgggttataagagtaagaattccgcgggtcctttccgctctaatcagataaaagggggtaaggacccg
ctaagttcctattttttcatgtttacaacctggtccctccaattactatagagatgaacccaatccagaatatgaaccgtaaaagaaaacacctattaaaccaatcacagc
aataccggttacagtacctatcagccaaagaggaattcttccagtagtatcggccatttcccctactttcctccacattttatcaagtggtcatgctagagacaaaaaca
gtcatggatagttataaggatggtatccttccaaatggggataagagaattcttactactctctttctttctctcaattgaagaagtaattggaaaataaaacagcaagtac
aaaaatgagtaataaaccccagtatagactggtacgattcaattcaacattttgttcattcgggtttgattgtgtcatagttctatagttggaatttggtttatcgttggatga
actgcattgctgatattgatcccaagaaaaaaacagtaggtacagctagtccgtgaacagccagccatcgcactgtaaaaataggataggttcgatctatggtcattg
agggcctcctaaaaggatctactaaattcatcgagttgttctaaagaatcaaaacggtcggttattaacggaattccttgtcggctttctgtgaaatactcgtttggccga
ggacttccaaacacgtcataagctaaacccgtactgacaaataaccaacccgcaatgaatagggaaggtatagtaatgctatgaataacccagtatcgaatactggt
aataatatcagcaaaagaacgttctcccgtgcttccagacatgctgagctccccaaatttttgtacattcaaaaaaggaattgattccgtaaaagatgggatcaaccag
taaatagaaaattactgatatttcatccttgtgagattgtcaattttgtaccaaaggtgtattttgagtataccaaattagtatagctatccttcctatggcacagcaatcctgt
ttcgcttggtctcgaaacagaattctttttttctcttctttgttccttgtctatagggtaagctatatggtattcaaggcatcaatagaaaacctcaattttgagggtcctactta
attttcaccggcttcggatcggaatagtagaataattcggaatagggctcaagatcttgggaaaatctaagttaatgatcaacaggtttggataaagaatttaggaaag
atattcttatactgacacaatacaaagagaagtagatgcgaaagctatcccttcgaatccaacctttccccttaaagaatttaattggttagcataatataatatctaataa
atagaaaatcaaatagtagataatctgttatgaaagagagaaaacattctttgaagaatcaagattcgtaatcaacccttgccttgtttactaactttcttgaccaaactgc
aagcgtggaactttgccagttcttaggtttccgaaatagtgtaatggaaaaagaagtgcttcgaatcgactttgattggggttcgaaaaaggaattaaaaaaaagtaaa
ttcaggaaagatttcctttttttaggagaccctcgaggggtcgtggaatgcttttcttctcctcttattccatatggaatacaatcaattaaaataagaaggaataggggaa
tatttgactgttcgctccaaaaagaaggttaaatcatcctattgaaaaagaccaaaatagaaagaactttttcaaattcaattctttatttttcactggggttagctgatctag
ttcttaatattattactttactcaattgacagattacacagcaaatctcttgattcggaattagggactcatgccccatctgatgaatccattttcttttacacttctgtatctca
ctctatcttgtttttttagtattatctaaaataaccgatgaattatgaattttccataacttaggtaagtgctttaccaacatatgtagtgtagtaaaaaaaatggaatttaaccct
ttcatgcttactataactagttatttcggttttctattggctgctttaactctaaccctagctctatttattggcttgaacaagatacgtcttatttgaaatgaattgaatgaataa
ggcagaaaagaaaaaatctctcttttggattcctggtattctacgcactaattaccaattcttttttttcttggtcattgagattcgtggataatttagactactatttagggata
aatcgtacctcttttttttatcccctcgaacaaatcgaaatgattgaagtttttctatttggaatcgtcttaggcctaattcctattactttagcgggattattcgtgactgcgta
tttgcaatacagacgtggggatcagttggatctttgattgagtaatatttctttttttgattgccctcctctctggtctggaggaggtcaaattggaattgcaatttgttttgtta
agttattttgccctgcttcgacataagatagatggaatcacgctctgtaggatttgaacctacgacatcgggttttggagacccgcgttctaccgaactgaactaagag
cgctttcaaaaatcaaatccttttctactcctaatgtgtctcacgtacgtatagtatccacaaattaaagttatacccgctttaatcgacctcctcactactgcctataaaga
agaaagaagtaataggtagggatgacaggatttgaacctgtgacattttgtacccaaaacaaacgcgctaccaagctgcgctacatcccttttccaaattgttgtaca
atgtcattgtacacaattcctatcttgttttccacatcctaattttcttgtctttttctatctatagagaatcctcgtgtcatttcttctttttggtctcatataatcaaggaatggtat
atatctaaatccaatctaatttcacctataaaagaaagattactattccttggtaatctataggaagggattgtctttttcgtttttagttcggaatttcgcctaaacaaaaga
aatacaaatgatcttgggcaataatatctgatcatatacgtattccaataaggaaggaggattttcaatgcgggatataaaaacatatctctctgtagcacccgtgctaa
gtactctatggtttggggctttagcaggtttattgatagaaattaatcgtttatttccagatgctttgtcattcccctttttttcattctagttgttgctatgtgaggaatagagtt
cttcgtgacatgataaaaaaattcccacttttcaatccctttttagtataggaaagaaaaagaaagaaaagatagattgggttggaccttagagtcatgaaaaatttggta
aatctcatttttgaaaaaagaaattcaattaaaagcagtatccaagctaagtcaggcctcagaaatcagagcatagaaagaggctgggttggctactaaaatgaaag
gattttgcttcaaaatccttgctaatttgacaaggattgtattccttaattatttctctattttttattacttaattgaaaaatttccaaaaatttttattctaatggattttcttcttcct
cttcggtttcaaaatagaggaataaaagaataagtagaagaattaagttaagtcaatccaaaaaggaaaggaggttcatggccaaggggaaagatgttagaatcag
agttattttgcaatgtgtgagttgtgttcgaaaaggggccaatgaggagtcggcagggatttctagatatagtactcaaaagaatcgccacaatacacccggacaatt
agaattaagaaaattttgtcgttattgtcgtaagcatacgattcatgccgaaataaagaaataggagcatcttgtgttcgatctttccaaagatcaaaaagaataagaact
tcctatttaatattcctatttaatatatagagcatagatagaatacaaaatacaaatcaacttgtctgatttccattagatattatttcatatgtatcgagggtattcatctaatat
atggaccaaagagagactatttcttctggatccaaaattaataaaataaacaaatcaatttttttttcaattttaaaataaggaataaatcatgtatacatctaaacaaccctttt
cataaatccaaacaaacttttcataaatccaagcaaacttttcgtaaatccaagcaaacttttcgtaaattcaaacaaccttttcgcaaacccaaacaaccttttcgtagg
cgtcctcggattggcccgggggatcgaattgattatagaaacatgagtttaattaatcgatttattagtgaacaaggaaaaatattatcgagacgaataaatagattaac
cttgaaacaacaacgattaattactcttgctataaaacaggctcgtattttatctttcttaccatttcgtaactatgagaatgagaaacaatttcaagcccagtcaatttcaat
aattacaggtcctagacccagaaaaaatagacatattcctccattaacacaaaagttcaattccaatcgaaacttaagaaactccaaccagactttaagaaacaacaat
cggaacttaagttccgattgttgatgttttattcgaaagggccagactatatataaagaaagtaatccaatttagattcttgggtttgttataagaaagaacaatggggaa
gaaaaaatagtttttttatttattgcaacatgctcgttgattcctaccacttaatcttaatttattgtatcttcccggagttacctctccgggaattctttttttaattattcctgtatat
tacttttttatccctttaattgataatctttattttattggaaatcgtgtaaagattatttggatttaatacagctacttgtgcaaggattttacgattaagaatcaattctttcttgta
aagattgtgtattaatttactataattatcgaatactttatgtatccgcgttgctgcgtttatccgactgatccacaaacgccgaaaatccctcttttgcctgactctatctcg
atgagaggaaacaaacgctcttcttacctgttgagtaatcattcgattaagtcttaaatgagcccctctaaagtttgaggcaaatgaacgcatttttgcccgtcgtctccg
agctatatatcctcgcggaactctggtcattgaatcaaattaaccttaatgaataactaatgatttctcttcttttagccatcctttttcccattaataacaaaacgaattattc
ccatatataaaatattagttccaatggcttttgctactataaccttcccaaccacaattttctcttctactcccttcagttatttcgcacgatactaaaaaatagtgggttccat
cgtttctatggttcccttttaaacggcgaggccctctctatacaccggagccctttctttcatttcatcaaagggtattgtgaacttgtatagttcacattctttggctctacct
atccattatagagtaaatagctcttttcacaataagagttatccatacagtgacggcatttaattatgaaagttggctaagtagctgaccctcttagtccgttttttttaagata
aaggagcataagcctttatctttttattactatttcctccgcttaatgaatagccgtttgctaccaatgggggaattgcttcttatttccaatctagatgattggatttgcacca
aaggaaaccagaaattccatataccgtagaaatctaggatagagaagctctatcctattcattggtaccgatcatggatacttcaaaaattgtcttatttgtttgaacccat
gatccgaacgagtcgcacatacaccctagcacatgttcctcgacgctgaggacatcccttaagcgcggccgattttctagcatttcgtattggctgtcttgcgtttctaa

taagttgtttaaccgttggcatgtcgtatgtatatagaaaaaatggattggtttagatcgatcttaacctgaatgattgatcattatgaagtatttccattaaattgcataaaa
cccgaatttagggttgaaataaatttacaagaaatctggccactaccaatccttaaacatttctggaaaccacactggatcagtatcgcagtgctcgtcaagcatttcat
cccctacaatatcgacaagtccataagctttggcttcgtctgctgacataaaaacatccctttccatgtcttcggatacaacccaaaaaggcttgcctgttcttagtgcat
aaacccttgtgatcatttcgcgaactttgtgtaactcttccacttctagtaaaaattctggtgttcttgcccgataataagcactagcaggttggtgaagcataatcctcgc
gtgggggaatgctatacgcttggtgggttctcctccaagcagaatgaaggacgccatggacgcggctattccgaggcatattgtatatatatctggtgtcaccgtttg
catcgtatcaaaaatcgccattcctgagattaaccacccgccgggggagtttataaacaaaaaaatatcgctaattccatcttctatactgagatataccatgagacctg
taatatgattcgtgacctcgcaacgaatctcttgacctaaaaaaagtgtcctttctcgatacataacattgtataagtcaacccaagtcgcttcttcatctccgggaatcc
ggtaaggtacttttggaacaccaatgggcatattagattaatattattaaatttaagtaagaaaactacactttaatatggaaacgtaagaatggaagagaaagaaaga
atccgcagtttattgttttcactttttttcttattctatatgaatactatagattctattaatacgtagattgaaataatacatagattgaaagattatacatataagtaggataag
acagattgaataaagaaaaaagaatgggtgattcgaatactaaacaaaaagagatagggatctattcttcgtttttttccaaataagccaagctgcccattgcatattgg
cacttatcgagtatagaatagatctgcttctctttcttcttacgaacagaattggcttcttatttttaatggaatgaaataaatattcacgctttctgacacagaatcccctag
aagggttaggtacataggatatggatagtctttgccaatgcgataaaataaagtgacatcgtgtctatttttctttgctaaaggggtatttccatgggtttgccttggtatc
gtgttcatactgtcgtattgaatgatccgggtcgattgctttcggtgcatataatgcacacagctctagtttctggttgggctggctcgatggctttatacgaattagcggt
ttttgatccctctgatcctgttctggatccaatgtggagacaaggtatgttcgtcattcctttcatgactcgtttaggaataaccaattcgtggggtggttggagtatttcag
gaggaactgtaacgaatccgggtatttggagttatgaaggcgtggcaggggcgcatattgtgttttctggcttgtgtttcttggcagctatctggcattgggtatattgg
gacctcgaaatattctgtgatgagcggacgggaaaaccttctttggatttgcccaagatctttggaattcatttatttcttgcaggggtggcttgctttggctttggcgcat
ttcatgtaacgggtttgtatggtcctgggatatgggtgtctgatccttatggactaactggaaaagtacaagctgtaaatccggcgtggggtgcagaaggttttgatcct
tttgttccgggggggaatagcttctcatcatattgctgcgggtacattgggcatattagcgggcctattccatcttagtgtccgtccacctcaacgtctatacaaaggatta
cgtatgggcaatattgaaactgtactttccagtagtatcgctgctgtttttttttgcagctttcgtagttgccggaactatgtggtatgggtcagcaactaccccaattgaatt
gtttgggcctactcgttatcagtgggatcagggatactttcagcaagaaatatatcgaagagttagtgatgggttagccgaaaatctcagtttatcagaagcttggtcta
aaattcccgaaaaattagccttttatgattatattggtaataatccggcaaagggggggattattcagagcaggctcaatggacaatggggatggaatagctgttggatg
gttaggacatcctatctttagagataaagaagggcgcgaactttttgtacgccgtatgcctactttttttgaaacatttccggttgttttggtagatgaagagggaattgtg
agagcggacgttccttttagaagagcagaatccaaatatagtgttgaacaagtaggcgtaacggtggagttctatggtggcgaacttaatggagtaagttattctgat
cctgctactgtaaaaaaatatgcgaggcgttcccaattaggggaaatttttgaattagatcgggctactttgaaatcagatggtgtttttcgcagcagtccaaggggttg
gttcacttttggtcatgctacctttgctttgctcttctttttcggacacatttggcatggggctagaaccttgttccgagatgtttttgctggtattgatccagacttggatgct
caagtggaatttggaacattccaaaaagtcggagatccaactacgaggagacagccagtctgaggccgcattgctttggtatctttcacctctctttttttgatttgacat
gggaaacatctcccatcccttctttgactctttttcctttttttatacgggaaatgatcccaaatgacaaatgaataggtgtggaagttataattgtaaataaaccacgatcg
aatctatggaagcattggtttatacgttccttttagtttcgactttagggataatttttttcgctatcttcttccgagaaccacctaaggttccgactaaaaaagtgaaataatt
taattgaagtaagaagtctcccagatggggagacttcttacttcaattagtccccgtgttcttcgaatggatctcttaattgttgagagggttgcccaaacgcggtatata
aggcatacccagtaaagcttacaagtaaaccagatatggagatggcgactaaagttgctgtttccatttttatagaatttcaagattacaatggatctacgaaaagatcg
tgtatttacaactacaacggaatagtatacaaagtcaacacaaatcattaaatggaatttatggctacacaaaccgttgaagatagttctagacctggaccaagacaaa
ctcgcgtaggtaatttattgaaacccttgaattcggaatatgggaaagtagctccgggttgggggactactccttttatgggggtcgcaatggctttattcgcggtattc
ctatctattattttagaaatttataattcttccgttttactggacggaattttaatgaattaggtttctactaacgaaaactacgaagtcatagtttttccatccaaaagagcctt
tctactttaagctctacatttctagacattctggtagttcgaccgtggaatttttttggtttcggtatctctggaatatgagtgtgtgacttgttagaatttgctcctattgataat
acatagaaagcacctgttatctctatcaagatgattctaattcgtcggatattatttattctagtatctggaacacgaaatagatagagtggatcaagaaaaaaaaatgaa
actatgattcatattaactattcagacctcgcaaccagactgaaaaaaattcaagtagttcttaataaaaataaaaaaagaaaatttcttccttccaattttgtttgcccaaa
aaacaactttttttctctcgattttgtcgagtcattacaccgattcaataaatgatcatcaagcggttcttattcgaagaacccttgccttttgtttagcttgagactcaatcat
cgtggctctagtatgaatctaaggttttaattgaactgattcataggatcgcaacaagataatttctaccagaaaactactccaatttttgctttatttatttatctagtaaaac
aagagtaaatctgcattacgcacaaaaaaaaagaaatccaaaatagggaagagaaaaatcaagaggcctctaatgatcaacatttgggaaagaaagatagacgag
ccaacttgagatttttttggcattatcatcacaaagaagaaattctggatttttcttatttcatatcttcaaggcaaatcgacccaacccagtggctgatgaagttttgaacct
tttttctaatatccgttgaaaatttgtgtgtttctgtttgagccgtacgagatgaaattctcatatacggttctcggagggggggttcgggttagttacctatctcaataaagta
tatgattggtttgaggaacgtcttgagattcaggcaattgcagatgatataactagtaaatatgttcctcctcatgtcaacatattttattgtttaggggggaattacacttact
tgttttctagtacaagttgctaccggttttgctatgactttttactatcgcccaaccgttacagaggctttttcctcggttcaatacataatgaccgaggccaactttggttgg
ttaatccgatcagttcatcgatggtcagcaagtatgatggttctaatgatgatcctgcacgtatttcgtgtgtatctcacaggtgggtttaaaaaaccccgcgaattaactt
gggtcactggtgtggttttagctgtattaactgcatcgtttggtgtaactggttattctttaccttgggatcaaattggttattgggcagtcaaaattgtgacaggtgtgcct
gacgcgattccggtaataggatcacctttagtggagttattacgtggaagtgctagtgtgggtcaatccactttgactcgtttttatagtttacatacctttgtacttcctctg
cttactgccgtatttatgttaatgcactttctaatgatacgtaagcaaggtatttcgggccctttataaggaaggcatctcatagagagttctaattctcatatatcatatcg
ggtaggttgtggtatttcattgctacaaacatgggttattctaaaataagacatgtcatttagatacttctcttcaactccgaactattgtgatacaaataatacaaatagttg
aagttaattttacgaaagaaaagaaggcggattatgggagtgtgcgacttgaattattgatttggccatgcagatagagagttggatctgccacattagaattcacgac
caaaggtgtctccatatccaatcaacacgtaagtcccctgtctagtaaggataggctggttcacttgaggagaatattttctatgatcatacctcaaccatgtcatccatg
aagaggctccgtaagatcccatagagtagaaatggaataagtcatgtgacatgatccaattctctatttattacacttactttttattatagtatggaaatgcattcattttctt
tgcatcgattgcgatctgcaatactatcggagtaaaagaagggatctaaggaagaacgtaggctaaactttttgatttttttattagtaacaagtaaatactttgtttggac
gtaagaaacttgcaatattgggggggataaataccaactaatcaagagacgcgagacaatccacaaagcaattgatcatgatcaaatttgtaagcccacttggatatt
gagcatttacccataagagtaggattcttttcaatgaatagttgtaggtgcaacttcggaaaatagaatctgataaagcttttcttacttagagccattgagccattatata
ccttattctattatggatcttctacggttttatttctttcattcttgctcgagccggatgatgaaaaattctcatgtccggttcctttgggggctggatcttaaagaattcaccta
tcccaataacaaagaaacctgacttaaacgatcctgtattaagagcaaaattagctaaagggatgggacataattattacggggaacccgcgtggcccaacgatctt

ttatatattttccagtagtaattctaggtactattgcatgtaatgtaggtttagcggttctcgagccgtcaatgattggtgaaccggcggatccgtttgcaactcctctgga
aatattacccgagtggtacttctttcccgtgtttcaaatactccgtacagtacctaataagttattgggcgttctcttaatggtttctgtgccgacgggcttattgacagtac
cttttctagagaatgtcaataaattccaaaatccatttcgtcgcccagtagctacaaccgtttttttaatcggtactgcagtagctctttggttaggtattggagcaacatta
cccattgaaaaatccttaactttaggtctttttagggatttttcagtttgattcattcaatcgtgaagtaccgtgcataggtatctaggaaatagttacttccaagtgaatctt
ccctagatacctataatctattttattatgatccatttcgcgaaaatatagattgtcccaaagatgcaaaattgttttctttttcttttattctaactcgaaaaagaagaagag
gaaaaaattgcaatggatttaaaactagaacttattcttaggtaaatccattgggagatgcttctctagagtgtcccatatctgttttccatcttgcatacgaaaactgtcaa
ttctcatcagatcttcttccgtcttactcaaaaggtccaatagtgtatggatattggcccttttgagacaattatacgttctagaaggcagttctaattgatcaataaaaata
caattcaatggaattctttttttgtttttctttagattagttaatctttttgaaagcttaaaagggggaagtaaacctgtttttattttcttggaaacgagtaccctcttcctccg
tgtgaagaaaaggaagaaataaatcaatcaaattacgagaagcctcataaagcgcttccttaggggttaaacttccattggtccatatttctagaaaaagtatctcatat
tttgcatttccattcccacaagcaaaaatactataattcacatttcgaacaggcatggatacagcatctataggataacttccatcttgagtgttctttctgacttccgtgtg
atatccgcgatctctcttgatccgtaactcaatacggaaatccgtgggttctgtcaagttagctataggttgtgccgtatcaacgatttctacggaaggcggtaagataa
tatcttgggcagttatgtatctaggacctttgacgcaaattgatgcggttctaactccatagagattacttctcaatacaatttctttcaaatttagtaaaatttcttgtaccga
ttcttcaatacctgctattgtagaatattcgtgcggcacgctcccaaattttgcatgtgtgatacatgttccttctgtttctccaagtaaagctcttcgcaaggcaataccaa
cggtatccgcttgaccttttctaagcggggacagaatgaaacgaccataataaagacgcttactatctactcttgattcaacacacttccactgtagtgtttgagtggat
cctgctacctcctctcgaaccatatagactagtattattatttgatcattgaatcgtttatttctcttgaaaggggtttaattcttttacagacgtctttttttaggaggtcgaca
cccattatgcggcataggtgttacatcgcgtatacaacttaatcgtacaccacttttagcaatggctcgtaatgcggcatctcttccactaccagcaccctttaccataac
ttctgctcgttgcaaacccactgtacgaatagcatctacagctgttctttgaccagcatagggtgatgcttttcttgagcttttgaatccacaagtacccgcggaggacc
agaaaaccacccgaccttgcggatctgtaacagttataatagtattgttgaaactagcttgaacatgaataactccttttgttattctacgtgcactcttccgtaaactaaa
acgcgcattcctacgcaaaccaatacgtactttcctacgtgaaccaatttttggtatagcttttgtcatattttattatctcataaatatgagttagaaataacaaaaagaaa
aaaagatacaaagatatccgtttcagggtaaaatatatcctttacttgaatttttttatttggaatttggacattttcgcgggtactttttttttacttttagaaagtcaagttcttt
tttcgaaagattaccccctgtctttgtttatgcttcggattggaacaaatgactctaattcgtccacgcctacgaatcagtcgacattttgtacaaattttacgaacagaagct
cttattttcatatttccgtattcctttcttaaactatgaatttactcttttggaaaaaataagtctctttgtttgaattttagaactttgaattttttaccctagaaaaaaaaagaaaa
acctaaccctttgaatctttggtatccttcaaatcttcggtatccttcgagtcttcggtacgcttcgaatccttatggggaagtctataaattatacgccccttgcttgaatca
taacgacttacttcaattttgaccctatcccccatcagtattcgtatagaactagaccggatctttcctgaaatatagcccaggatgatggtgtcattctctaggcgaacg
cggaacattccgttgggtagggcttccataactaaaccctcgaaagtaatttttgcttctctcgggggttttttttctctcctatttttcttttctgtcatatttttttctcctattttt
ctttttttatttgaaaataaaaaaacgttgtatttttgtttgaaaaataggaagttcaagatagaatttgggtactataactataggtggggcgattaccgctataggcggg
gcgattaccatatataacataagacttctcccccaattctgtttagtcgagcttctcgatctgtcattatacctcgagaagtagaaagaatagcaattcccattccgccca
aaaccttaggaattccttgatagttggcataaattcgtaagccaggtcggctgatacgctttaaaaaggttctagttctatatattccctttctagtctttctcttttgatgtcg
caaagttgaaaccaagaaatatctgttactttcctgatgtttccgaacactttcaataaaaccctctcgtagaagtattttaacaatgttttcggtaatatttgtagatactac
ccgaacagttcctttttttattcatgtccgcgtttcttatagaggttagtaaatcagcaatagtgtccttgcccataagactctaattctaggttcctcctaatttttctataatca
acatgttctctttttttcttttcgttttggattctaaagcatatacgtgaaacacaatctactaatttttttatttgatctatatcttgtctactagtatttataatacttcaggagctaat
gaaactattttagtaaaattcaattctctcaattcctcggcgatcgcgccaaaaacgcgagttccttttggatttcctttttgatcaatgataaccgctgcattgtcgtcata
gcgtattattataccgtcttcgcatttgaactctttacatgtacgtacaattacagctcgaattacttcggatctttctagaggcatttggggcactgcgtctttgattacagc
aacaataacatcaccaatacgagcatatcgctgattactagcagctcctatgactcgaatacacatcaattttcgagctccactgttatctgctacatttaaaagggtctg
aggttgaatcatattattttgatttcaatttgttatttcaatgcaaaaggatgaaagaaatattgtctttccagaaagaagaacctggttttttattttcaatactactttttttggg
ggggttatatttctaaccgaagaaattgacttcgtatgggcattttgctggcagctatggaaatagctgctctagctacagtttcggatactccgcccatttcataaagta
ttcgacctggtttaacaacggctacccaatattcgggggatccctttcccgaacccatacgtgtttcggtcggtcttattgtaaccggtttgtcgggaaatatacgtacc
cagatttttccaccacgacgtgcatatcgtgtcattgctcttcgtcctgcttctatctgcctcgccgtaatccaagtgggttcaagtgcttgaagagcatatctaccaaaa
caaatacaattgcctcggtaggattttcccttcattcttcctctatgttgtttacgaaatctggttcttttggggttatagtcgatggttctctcttagttccatctctactgcaaa
actggacatgagagtttcttctcatccagctcctcgcgaatcaaatgagaaagtgtgcaaatttctctaattccacaatattctaaaatattacggatagatacacattaat
aggaaaggttagggtttttaatattgaatttattaaaatactaaaatatatagtcaagaaagaggatctagaatatatctagatgtgtatgtttatttatctatattctatattta
atgtaattctattttttttttaatctcctttttttattttactcttattgaatcgcggtaaagtattctaattcaataaaaaatttcgcgggcgaatatttactctttcctgtcttatttgtta
attcataaccttatcaaataagacaattttttttggtttgttccgccatcccacccaatgaagtattgggattcttttcaagaaaatcctatccagtcataggttctgtcgttcc
cactgcttctcctttaatggttaggtttgaatctcgcaatggagcttccaaaaaatttctttccgagtcaatttctcagttttattaactcgggacgctctttgttattccttgaa
atttgtatttcttgtttcaatcaaattacgatttcgaattctctgttatttttattatattgatgctttatcacattgctttttatgatgtaattcatagaccatacatattggaatcctat
atctttcttatttctcttccttctttctatcatccttccttttatctacatcccctttagttttgcttcacaacttagaatcctattttttttaaagaaaaaattgcagttgctacacgta
tatgatagatttactcatttatgatagatgtatttattcatatagtgactgtttcttagttaggatctcgacaatacgaagcaataggttggttattagttcattttctataattac
atagtagggttcattctatttttttttaatccccttttttgaaccctaaaaaaaaaactaacgagtcacacactaagcatagcaattttattaaaagatttctcaattttcattaaat
cttatagaaagaggtagaatttcttcttttttttcagggattttagggaaaataaggctcttgtcatttttttattctattactgaacagaatgggaagacagggttggttattctt
cgtctacgaatatccaaattttaactcctaatactccatagatagttcgaattggatagcagcaataatcaattttagcgcgaattgtttggaggggaagtctaccttttttg
atgcattcggcacgcgcaatttcttttcccgcgagacggcctgcaatttttactttactccctttatatctgttttttttagttaattcaatggctttttttcattgcctttcggaatg
aaactctatttttttaattggaaagctatatattctgcaagaatgttaggttgtctataaggttctttcactttttcgatcccaatattaagtctctggtttacagaattaacttctttt
tgtagatctttctctaattcttcgattgctcctttttttctttaataaattggggaatccaatatggattatgacgtggattgtatcgatttctttttgaatttctatatgtgtaattact
tcggaacttgagcctgagtccatttttttattatgtgtaattacttcgaaacttgagtctgcttctatttttctattcgagccctttttcctattcttttgtatatagttcttgatacaa
ttccgtattttttttatcttcctgtagaccttcagaataatttttttggttgtgcgaaccaaaaggaatggtgattttgggttgtaccaagtctgaaaccaagtggatttatttttttg
tcccatattttttctattctatttttttttaccgggaatcaaatcttagatggatctaaagattatttcgatttctttactatatttagtacaattgttatatgacacatggttttttttatg

ggagaactacgtcctcgagcccgaggtctgaatttattcataatagtactcctactgacttccgctttagtgatgaataaatttgctttgtcgaaatccctataatgagtag
catttgctgctgccgaataaaccaactttaagatgggataagatgctcgataaggcatgaggttcaatatcataacagtttcttcgtagtaacgccaacgaatctcatcc
agaactctttgtactttgaaaacggacatgtggatgcgtttttgtgctttgaaaacccgttcgaacttaagtagacgatcggttggtacttttcttgcgagtttccttagccc
actcttcttcttctttatcctaggggtatactttaccagtttgaaacttgtcataaataaggttattccccgcttacctatttcttttttttttatttgaatctttctattctgaattca
gttaacgacgagatttagtatcctttcttgcactttcataactcgtgaaatgccgagttggtacgaattcccccaatttgcgacctaccataggatttgttatgtaaataggt
atatgttcctttccattatgaatcgcgattgtatggccaaccattgcgggtagaatgctagatgcccgggaccacgttactattgtttctttctcctccttcatattgacctttt
ctatttttgccaataaatgatgagctacaaaaggattcgttttttttcgtgtcacagctgattactcctttttttccattttaaagagtggcattcgatgtccaatatctcgatcg
aagtatggaggtcagaataaatagaataatgatgaatggaaaaaagagaaaatcctttagctggataaggggcggatgtagccaagtggatcaaggcagtggatt
gtgaatccaccatgcgcgggttcaattcccgtcgttcgcccatcccattattgcaaattccaaaaatgcaattttccatattcctagttacgtatttacttacggcgacgaa
gaataaaactatcactatattttttccttttcctagttcttcttccaagcgcaggataaccccaaggggttgtgggtttttttctaccaatgggggctttcccttcaccgcccc
catgggggtggtccacagggttcataactacccctcttactacggggcgtttacctagccaacacttagacccggctctacccaaactttttttggttcaccccaacatt
acccacttgtccgactgttgctaagcagttttgggataccaaacggacctccccagatggtaatcttaaagtggccgatttaccttcttttgcaatcagtttcgctacagc
acctgctgctctagctaattgcccacccccttccacgtgtgatttctatgttatgtatggccgtgcctaagggcatatcggttgaagtagattcttcttttctctcaaaaaacc
ccttcccaaactgtacaagcttcttccaaagcatacggctttctagatgtatatgacgatctctagacagatggatcttatatgaatcgtatgatgaagtaccacatgagt
ggatatataggaaaggaatccaaatctgccgaatcgctcatgttatgatcttctacatcctaggtctccgcgttccgtcatctggcttatgttcttcatgtagcattcagat
cgaatgactctatgaaattacgtcgatacttctacatattatgggtaacgtaggagacatccctattttcccccgggggtcttaattaccactgcttagctttcaattcgcc
tctgaccatcaaattaaatgtgaataacccgtcctcctctctttgaaacaaggggcgcttccggttctgtgcgtgcttcaaacaattttgtcttctccatattaccatatctct
agagtcaataattttctatgaggaactactgaactcaatcacttgctgccgttactcaacagttttctgttgaggtctatcccgtagaggtagtcaaattggatcagtgatc
gatttctaggtttcgtcgtaaacctaattggttacttccaattacgtaaatcaatagttcaaaccgcactcaaaggtagggcatttcccattgatataggaacttttgtacca
gaaacaatagtatctccaattatagcccctctgggatgtaaaatatatcccttctcaccatccccatagtgtatgagacaaatgtacgcatttcgattagggtcgtattcta
tggttacgattctaccagatatgtctttttgattccgtcgaaaatcgattttacggtataggcgcttatgacctccccctctatgccttgcggtaatgattcctctggaattac
gacctttaccacaacggtgccgtccatggatcaaattatttcgtggattggatttcacttgcctatctatggttcccttgcgtgtgctcgggataggtgttttgtataaatgt
ttcgccgtattattaagtattctcctttagtttttttctctatctagaagtggaatagaataacccggttgaagggtaatgatcatacgtctgtaatgcattgtatgtcctagaa
tagggcccattcttctaccctttccgggtagtcgatggctattcacagctactaccttaacaccaaagaagagttcgacccaatgctttatttctgtcttagtgaatcccg
attcgacattaaaagtatattgattctttcccaataaacgaagactttttttctgtaaatactgcgtatttgattccatccataaatcgactttccctcctatgctctgagttcca
gtatcgataagaattcgagttcttattgttcttatgttatggtatgaatataccataccaattcgttatgtatggatgatggatgagattccatggatagagagccagttcca
atagacttatggaacgttccggttcgcgtgcatccagcaggaattgaacccgcaaatttaccaattatgagttgggcgctttaaccattcagccatggatgcttaacag
ggatcatcgtacatcgtaaataaccaattttcatatagaaagacatatcatagaaaaatgaaatcgaaaatattcggagatggcaaatattcggagatgactatgaaaa
cacctctctggatcctcgaattgaaagagagattgagagggatccagaatcctaattctcgctatttggaatggatccaattctattgagtctgactcatagtgatcattt
ctctttagcaaagaatgaccttggttatcaaaggattgaacaaccgggatccgtttacttatgatacctagttgacattgataacaaggatctaatgaattatgagtttaat
agatcctctttagcagaaagacgtatattccttgctcatcacttattcccaaacctcgtatggggctaatcgttttcatttaccatctcagggaaaatccttttcgttccgctt
agccctatcgggtattttagtgataggttctataggaactggacgatcctatttggtcaaatacctaatgaaaaattcctattttcctttcattaaggtacgagggcttcttat
tccacaagaaaagaacgaaagcaccttttcattctttcatatactgggggtttttacttggaaaagacaatgttccatactaaaggattcgggtccataaccacgagttc
cagtgcactagatcttgtagcacttagcaacgaggccctatgaatagacatatagaatttttggtcgggaaattcgaatgaatcattgagtgaaaaaggagcaaagaa
tgacaaaagacgagactctactagtcttcactcttgtggtttcctcggtttctgttttcttattcgggatcttgcttttcatggttctcatctctgcaactcgcgattttcgcga
gagaaccaaatccaagttggtgaagatcatgatttgggctggcatagtagtgattacctttgcaattgcggttcgaatctatccgatctttatctttttgctcaaagaacg
aataaaaccccttgtcgaagccctttatgataagcttccctggatctgggaagtttctctttcacggtattgggatcgtttgatcgatttccttgatcgctacttatgggcgt
gcgctcaaaggatacaaacagggattcgcaaacaaaaaggggaattcgtagtcactttttcctgtcgcgtaaaaaaaaggctttacgcgagagcaatagaggttgg
gatacatctatctcttctgagcaacctcttttggattcttaagaccacccttgcagtaggataccgtctgctttgggttctttattatattatctccttcgagggatttttaggat
cgttcaggctatatttagtctattttggcttttactgtctccttttctcagggaagtggttaaggacctcagaagatagaggagagcgccaggcgcagatttccggaata
cttctacggggaatgctcattgaatgcgcattctccgtattatgccttgaagaggactcgaacctccacgctctttagcacgagattttgagtctcgcgtgtctaccattt
caccatcaaggcatcttgaaagtgaatcatattccatgaatatgatatctatctaatgtgatatcttgaaagtgaatcgtattccatgaatatgatatctatctaatgtgatat
atggaatatatgacaaaggtggagtcttggagtatttcgatcgatcggtcatataggcctgagtcagacatcaaatagcttcgatttgcattatccgtaggacaccttat
atgtatcaaaatcaaaaagatgtacaatccaatttctcgattcaatagaagcccaaagaggtgcatatggtacccaaataaggataggatagatatgtcaaaagcag
gtctgattacacctattcctaatcctaaatagaatgtaaggacgtggggatttctatgtaaacagagtatcctatttccataggctcgaatgaccccttctcataataaga
atgtgcacggtctggtccggtatggaatgaacttataatctgatgatcgagtcgattccatgattataagttcattaccctagcgcccattcccattttgggcggaacag
atctactaattcttttattccagttagtaagagggatcttgaactaagaaatagacctagcagctaaaagagggtatcctgagcaattgcaagaatggggttcattgata
ttcctggtatagtagatgctatcacacatacagtcatactcaattcgatggaattgtttgatcttaaaggggatcttctataatttcgcacataaggggttatttcttggtttc
gtccagtcattaataacttgactatttttagataatagtagatagaaagaacgctcgtaaggagtcctattgaaaccaagaaatataggcctgcttgccatccacacca
gaatagatagagttttccgaagaaacctgctagtggaggaaggcctcctagggataagagacatagggctaaagagagagccaaaaaaggatctttcgtgtataat
cctgcataatctcgaatgttatcagttccggtacgtagaccaaataatacaatgcaagcaaaagttcctagattcatggagatatagaaaagcatataagttatcatgct
tgcatatccatcatttgagtctccaacaattattccaataattacatatccgatttgccctatggacgaatatgcaagcatacgtttcatgcttgtttgagtaatagcaagga
gattccccaatatcatgctaagaatagctaggatttccagaagaagatgccattcgtttgatgagaaataaaaaggaatatcgagaattcgcgtggctgaagctgaag
cagcaactttcgaagtaacagaaagaaaagcaacgactggagtgggggagtcagagtcgaaaagaggattcctcgcttctttctctcatgcaaaaccgtgcatga
gactttcatctcgcacggctcctaagtgataaaagaaagaagaacttgtcttctttctttttttgattaccttcctcgcgtatgtataagaccgaatccattctttttcgaaatc
gatttcgaaaaagaactactaatccttaacttttcgaggaatccttcatcagtggttgtgaatgactgactttttcaatcctttcgaccttggttccgtaggagcaagtcag

aaaggttgagaaatagaaccatctgatttgattcgttcccaatagccatgagatgatcatcttagggtgatccttttgtcaacggatgctcctattacactcgtagtctctg
aaggatgagaacccactatgtagcatctacgtcgataattcaagcattgtatacgtcattagtccgattctttgtaggaactacccgtaataacgagcttgcaaaatgga
tctgtttatcataaagagattcgttgttcctgaccctgcttcaccttaattgttatttgaacaaaaagatcacaataaacttttggtaaaagttctgtcttggtcggagtgggg
atagcatttctcttctgcatatctatggagttttgcaaaacccaaacacctcagagatagatatagaggtaggaatttgtcgaacgaaccacactccttcgtagacgtca
ggagtccattgatgaaaaggggctggggaaagcttgaacccaagtcctacagtgatggatataagcgcaattgaaattcctggggagttatacatttgtgtattgata
agaccgttcacaatttcttgaagctcgatctccccccagatgaaccatatagccaagagaaaccatgaaccagaatagaagagcttgccccacccatgagtaaat
atttcatagtagcctcattagaccgtagatctctcttggtatatccagacaataggtaggaacataaactgaaacattctggagctacaaagatagttattaaatcgttag
caccacataaaaacattcccccctagagtagctgttaatacgaataacagaaactctgttatagccatttctgtacattcaatgtactctacggatagaggaatacataaa
gttgaacataataaaataagaaattgaaagatttcgttgaaattgttcgtttggaaatttcccgaaaagctaattataggttcttctctccatcggaacaatagggccgtta
tgcttattactaaacttgttgaagagatgaaatagaaccaaggtctatctttttgatcagaggttaaatcgatcatcagaagaagaattaggccaaaaattaggatacatt
ctgggaaaatgaaacttccctggaagagaagcaaatgaaacgctttcataaaaattctcgtagaatcgagaatgaagttttcattctgtacatgccagatcatgaatta
gtaactgcatccaatctccgaaaagtcccgattgtttcgatttttggaatgggatatttacggaatccccatgaataggatcaaaccttattccatgctatttccataagatt
cctctttcttattcttaagcaagcccccgagagggcttagttgatcatgatttctgttttctctttcttttccttttttgtttgtttcgagaaagatatcgtccgattctccttctattg
attcttttccgatcgagatgtatggatccatgtgtctacatacctagattctgttcatggattaacgaaaatgtgcaagagctctatttgcctctgccattctatgagtcgctt
cctttttgcgtatggcacccccacccccctttggcagcatctactaattcggaacttaatttgaaagccatatttcgacccggacgcttttgggatgcttctaataaccaac
gaatggcaagtgctcttccttgtttagatcctatttcaatcggaactttccgcgtcgatcctttttttattacgtcttgtttttactcctatattgggagttactctacgtattgcttg
acgtaaaaccaatagtggatttgtttctgtcttttgttgaatctttttcacggctcgatagagaatttgataagccaatgatttttttccgtctttcataatacggttaaccacca
tgttaactaatcgattacgaaaaattggatcggattttgcagttctttttctgcagtacctcgacgtgacatgagcgtgaaagaggttcaagaatccgttttctttttataa
gggctaaaaacgaatcacttattttttggctttttgaccccatattgtagggtggatctcgaaagataggaaagatctccctccaagccgtacatacaactttcatcgaa
tacggctttccacagaattctatagggatctatgagatcgagtatggaattcagtttactcacttaaaattgagtatccgtttccctccttttcccgctaggatcggaaatc
ctgtattttccatatccataccatcaagtccttaggtttccgaaatagtgtaatggaaaaagaagtgcttcgaatcattgctatttgactcggacctgttctgaaaaagtcg
aggtatttcgaattgtttgttgacacggacaaagtaagggaaaacccctgaaagaatttccatattgaccttggacatataagagttccgaatcgaatctctttagaaag
aagatcttttgtctcatggtagcctgctccagtccccttacgaaactttcgttattgggttagccatacacttcacatgtttctagcgattcacatggcatcatcaaatgata
caagtcttggataagaatctacaacgcactagaacgcccttgttgacgattctttactgcgacagcatctagggctcctcgaataatgcgatatctcacaccgggtaa
atccttaacccttcctcctcttactaatactacagaatgttcttgtaaattatggccaataccaggtatataagcagtgatttcaaatccagaggttaatcgtactctggcaa
ctttacgtaaggcagagttgggtttttggggttgatagtggaaaagtcgacagataagtcacccttcctgtccctctacagaaccgtacatgagattttcacctcatac
ggctcctcgttcaattctttcgaagggatccttttcctcgttcgagagtctccgcccttcttccactccgtcccgaagactaactaagaccaattgagtcacgttttcatgt
tctaattgaacactttccatttatgattaaaggagaagattgttcttttaccaaacatatgcggatcaaatcacgtcttataataagaagaaatctttctcggtatcaatccc
cttgcccctcattctttgagaatcagaaggatccttttcgagtttccatttcttcatttggaatctgggctcttctatcttcgacttatttttttttgctttattctttatttatttcatt
tcgatttttccctcttcctctatccctatcctctaggtacagcgtttgcatcaatagagaaccttttcctctgtatgaatctatattattccattccaatttcttcccgaaacttcc
caagaaaaatcccgaattggatccaaaattgacgggttaatgtgagcttatccatgcggttaggcactcttcaaataggaatccattttctaactggctttcgtgctttgg
tgagtcgtccgagatcctttcgatgacctatgttgtgttgaagggatatctatatgatccgatcgattgcataagacccgcggtagcaatagaacggggaaagtatac
agaaaagacagttcttttcaatttcgattatctatatattagttcgtttctatttctagatatctatttctatatattagtattagttagtagtactattctattagttagcgatcccg
gctctgtgagttctttcttccgtgatgaactgtcggcaccagtcctccatttttctctgtggaccgaggagaaagggggctcagcaggaagaggattgtaccatgag
agaagcacagaggtcaaccctcttcaaatatggaacatggattctggcaatgcaacgtagttgggtcctcatatcgatccgaatgaatcagtctttctacagaggtca
atctttgcctattaggcaagaggatagcaagttcgaaattctgtctcggtaggacatggatttctattactatgaaattcataaatgaaaatgaagtagttaatgggagg
gctaccattatcctttttcttgtatgtgttcctaagagaaggaatttgtccatttcatgtttcgaggtctcaaaaaagggcgtggaaacagatagaaactcttgaatggaaa
ttgaaaagaaatgtagccccagttccttcggaaatggtaagatctttggcgcaagaagaaggggcgacccatatcatcttgacttggttctgcttcccctctttttttaag
aataccgagtcgggttcttctcctaccagtatcgaatagaacatgctgaacaagatcttcttcatggaaacctgctcgatttagatcgggaaaatcgtacagattttatg
aaaccatgtgctatggctcgaatccatagtcaatcctattttcgataggaccggttgacaattgaatccaatttttcccattatttgactatccataatagtgtggaaagaa
agcccggaggaagggtggccttgagtttctcgcccctttgccttaggattcgttaattctctttctcgaggggacggggaagggatataactcagcggtagagtgtca
ccttgacgtggtggaagtcatcagttcgagcctgattatccctaaacccaatgtgagtttttttctattttgacttactccccccgccacgatcgaacgggaatggataag
aggcttgtgggattgacgtgatagggtagggttggctatactgctggtggcgaactccaggctaataatctgaagcgcatggatacaagttatccttggaaggaaag
acaattccgaatccgctttgtctacgaataaggaagctataagtaatgcaactatgaatctcatggagagttcgatcctggctcaggatgaacgctggcggcatgctt
aacacatgcaagtcgaacgggaagtggtgtttccagtggcgaacgggtgagtaacgcgtaagaacctgcccttgggaggggaacaacaactggaaacggttgc
taataccccgtaggctgaggagcaaaaggagaaatccgcccaaggaggggctcgcgtctgattagctagttggtgaggcaatagcttaccaaggcgatgatcag
tagctggtccgagaggatgatcagccacactgggactgagacacggcccagactcctacgggaggcagcagtggggaattttccgcaatgggcgaaagcctga
cggagcaatgccgcgtggaggtggaaggcccacgggtcgtcaacttcttttctcggagaagaaacaatgacggtatctgaggaataagcatcggctaactctgtg
ccagcagccgcggtaagacagaggatgcaagcgttatccggaatgattgggcgtaaagcgtctgtaggtggcttttcaagtccgccgtcaaatcccagggctcaa
ccctggacaggcggtggaaactaccaagctggagtacggtaggggcagagggaatttccggtggagcggtgaaatgcattgagatcggaaagaacaccaacg
gcgaaagcactctgctgggccgacactgacactgagagacgaaagctaggggagcaaatgggattagagaccccagtagtcctagccgtaaacgatggatact
aggtgctgtgcgactcgacccgtgcagtgctgtagctaacgcgttaagtatcccgcctggggagtacgttcgcaagaatgaaactcaaaggaattgacgggggcc
cgcacaagcggtggagcatgtggtttaattcgatgcaaagcgaagaaccttaccagggcttgacatgccgcgaatcctcttgaaagagaggggtgccctcggga
acgcggacacaggtggtgcatggctgtcgtcagctcgtgccgtaaggtgttgggttaagtctcgcaacgagcgcaaccctcgtgtttagttgccactatgagtttgg
aaccctgaacagaccgccggtgttaagccggaggaaggagaggatgaggccaagtcatcatgccccttatgccctgggcgacacacgtgctacaatgggcgg
gacaaagggtcgcgatctcgcgagggtgagctaactccaaaaacccgtcctcagttcggattgcaggctgcaactcgcctgcatgaagcaggaatcgctagtaat

cgccggtcagccatacggcggtgaatccgttcccgggccttgtacacaccgcccgtcacactataggagctggccatgtttgaagtcattacccttaaccgtaagg
agggggatgcctaaggctaggcttgcgactggagtgaagtcgtaacaaggtagccgtactggaaggtgcggctggatcacctccttttcagggagagctaatgct
tatgcttattgggtattttggtttgacactgcttcacgcccaaaaagaaggcagctacgtctgagctaaacttggatatggaagtcttctttcgtttagggtgaagtaaga
ccaagctcatgagcttattatcctaggtcggaacaaattagttgatagtgataggatccccttttttgacgtccccatgccccccccgtgtggtgtggcggcatgggg
atgtcaaaaggaaagggatggagtttttctcgcttttggcgtagcaggcctcccaaagggaggcccgcgcgacgggctattagctcagtggtagagcgcgcccct
gataattgcgtcgttgtgcctgggctgtgagggctctcagccacatggatagttcaatgtgctcatcagcgcctgacccgaagatgtggatcatccaaggcacatta
gcatggcgtactcctcctgtttgaatcggagtttgaaaccaaacaaacttctcctcaggaggatagatggggcgattcaggtgagatcccatgtagatctaactttcta
ttcactcgtgggatccgggcggtccgggggggggcactacggctcctctcttctcgagaatccatacatcccttatcagtgtatggagagctatctctcgagcacagg
ttgaggttcgtcctcaatgggaaaatggagcacctaacaacgcatcttcacagaccaagaactacgagatcaccctttcattctggggtgacggagggatcgtacc
attcgagccttttttttcatgcttttcccggcggtctggagaaagcagcaatcaataggacttccctaatcctcccttcctgaaaggaagaacgtgaaattctttttccttc
cgcagggaccaggaggttggatctagccataagaggaatgcttggtataaataagccacttcttggtcttcgactccctaagtcactacgagcgccctcgatcagtg
caatgggatgtggctatttatctatctcttgactcgaaatgggagcagagcaggtttgaaaaaggatcttagagtgtctagggttgggccaggagggtctcttaacgc
cttcctttttctgcccatcggagttatttcccaaggacttgccatggtaagggggagaaggggaagaagcacacttgaagagcgcagtacaacggagagttgtatg
ctgcgttcgggaaggatgaatcgctcccgaaaaggagtctattgattctctcccaattggttggatcgtaggggcgatgatttacttcacgggcgaggtctctggttca
agtccaggatggcccagctgcgccagggaaaagaatagaagaagcatctgactctttcatgcatactccacttggctcgggggatatagctcagttggtagagct
ccgctcttgcaattgggtcgttgcgattacgggttggctgtctaattgtccaggcggtaatggtagtatcttgtacctgaaccggtggctcactttttctaagtaatgggg
aagaggactgaaacatgccactgaaagactctactgagacaaaaagatgggctgtcaaaaaggtagaggaggtaggatgggcagttggtcagatctagtatgga
tcgtacatggacgatagttggagtcggcggctctcctaggcttccctcatctgggatccctggggaagaggatcaagttggcccttgcgaatagcttgatgcactatc
tcccttcaaccctttgagcgaaatgtggcaaaaggaaggaaaatccatggaccgaccccattatctccaccccgtaggaactacgagatcaccccaaggacgcctt
cggcgtccaggggtcacggaccgaccatagaccctgttcaataagtggaacacattagccgtccgctctccggttgggcagtaagggtcggagaagggcaatca
ctcgttcttaaaaccagcattcttaagtttaagatcaaagagtcgggcggaaaaaggggagagctccccgttcctggttctcctgtagctggattccccggaaccaca
agaatccttagaatgggattccaactcagcaccttttgttttgagattttgagaagagttgctctttggagagcacagtacgatgaaagttgtaagctgtgttcgggggg
gagttattgtctatcgttggcctctatggtagaacccgtcggggaggcctgagaggcggtggtttaccctgtggcggatgtcagcggttcgagtccgcttatctccag
cccgtgaacttagcggatactatgatagcaccgaattttgccaattcggcagttcgatctatgatttcgcattcatggacgttgataagatccttccatttagtagcacctt
aggatggcatagccttaacgttaatggcgaggttcaaaagaggaaaggcttgcggtggatacctaggtacccagagacgaggaagggcgtagcaagcgacgaa
atgcttcggggagttgaaaataagcatagatccggagattcccaaataggtcaaccttttgaactgcctgctgaatccatgagcaggcaagagacaacctggcgaa
ctgaaacatcttagtagccagaggaaaagaaagcaaaagcgattcccgtagtagcggcgagcgaaatgggagcagcctaaaccgtgaaaacggggttgtggga
gagcaatacaagcgttgtgctgctaggcgaagcggttgagtgccgcaccctagatggctaaagtccagtagccgaaagcatcactagcttacgctctgacccgag
tagcatggggcacgtggaatcccgtgtgaatcagcaaggaccaccttgcaaggctaaatactcctgggtgaccgatagcgaagtagtaccgtgagggaaaggtg
aaaagaaccccccagtgggtagtgaaatagaacgtgaaaccgtgctgagctcccaagcagtgggaggggaaagtgatctctgaccgcgtgcctgttgaagaatga
gccggcgactcataggcagtggcttggttaagggaacggaacccaccggagccgtagcgaaagcgagtcttcatagggcgattgtcactgcttatggacccgaa
cctgggtgatctatccatgaccaggatgaagcttggatgaaactaagcagaggtccgaaccgactgatgttgaagaatcagcggatgagttgtggttaggggtgaa
atgccactcgaacccagagctagctggttctccccgaaatgcgttgaggcgcagcagttgactggacatctaggggtaaagcactgtttcggtgcgggctgcgcg
agcggtaccaaatcgaggcaaactctgaatactagatatgacccaaaaataacaggggtcaaggtcggccagtgagacgatgggggataagcttcatcgtcgag
agggaaacagcccggatcaccagctaaggcccctaaatgaccgctcagtgataaaggaggtgggggtgcaaagacagccaggaggtttgcctagaagcagcc
accctttaaagagtgcgtaatagctcactgatcgagcgcccttgcgctgaagatgaacggggctaagcgatctgccgaagctgtgggatgtcaaaatgcatcggta
ggggagcgttccgccttagagggaagcaaccgcgaaagcgggggtcgacgaagcggaagcgagaatgtcggcttgagtaacgaaaacattggtgagaatcca
atgccccgaaaacccaaggtttcctccgcaaggttcgtccacggagggtgagtcagggcctaagatcaggccgaaaggcgtagtcgatggacaacaggtcaata
ttcctgtactaccccttgttggtacggagggacggaggaggctaggttagccgaaagatggttataggtttaaggacacaaggtgaccctgctttttcagggtaaga
aggggtagagaaaatgcctcgagccgaggtccgagtaccaagcgctgcagcgctgaagtatgagccccgtggactagccattgcttctccacgaggctcatacc
aggcgctacggcgctgaagtatgtaacccatgccatactcccaggaaaagctcgaacgaccttcaacaaaggggtacctgtacccgaaaccgacacaggtgggt
aggtagagaatacctaggggcgcgagacaactctctctaaggaactcggcaaaatagccccgtaacttcgggagaaggggtgccccctcgcaaaagggggtcg
cagtgaccaggcccgggcgactgtttaccaaaaacacaggtctccgcaaagtcgtaagaccatgtatgggggctgacgcctgcccagtgccggaaggtcaagg
aagttggtgaactgatgacagggaagccggcgaccgaagccccggtgaacggcggccgtaactataacggtcctaaggtagcgaaattccttgtcgggtaagtt
ccgacccgcacgaaaggcgtaacgatctgggcactgtctcggagagagactcggtgaaatagacatgtctgtgaagatgcggactacctgcacctggacagaaa
gaccctatgaagctttactgttccctgggattggctttgggcctttcctgcgcagcttaggtggaaggcgaagaaggccccccttccgggggggcccgagccatcag
tgagataccactctggaagagctcggattctaaccttgtgtcagacccgcgggccaagggacagtctcaggtagacagtttctatggggcgtaggcctcccaaaa
ggtaacggaggcgtgcaaaggtttcctcgggccagacggacattggtcctcgagtgcaaaggcagaagggagcttgactgcaagactcacccgtcgagcagag
acgaaagtcggccttagtgatccgacggtgccgagtggaagggccgtcgctcaacggataaaagttactctagggataacaggctgatcttccccaagagtccac
atcgacgggaaggtttggcacctcgatgtcggctcttcgccacctggagctgtaggtggttccaagggttgggctgttcgcccattaatgcggtacgtgagctgggt
tcagaacgtcgtgagacagttcggtccatatccggtgtgggcgttagagcattgagaggacctttccctagtacgagaggaccgggaaggacgcacctctggtgt
accagttatcgtgcctacggtaaacgctgggtagccaagtgcggagaggataactgctgaaagcatataagtagtaagcccaccccaagatgagtgctctctcctc
cgacttccctagagcctccggtagcacagccgagacagcgacgggttctccacccatacggggatggagcgacagaagcatggaaataggataaggtagcgg
cgagacgagccgtttaaataggtgtcaagtggaagtgcagtgatgtatgcagctgaggcatcctaacgaacgaacgatttgaaccttgttcctacacgacctgatca
aatcgatcaggcacttgccatctatcttcattgttcaactctttgatgaaaagatgaaaaaaccaaaaaaaagctctgcccttccatctcttggatagatagagaggga
gggcagaggcctttggtgtcccttccagtcaagaattggggcttcacaattactagccaatatttctctcatgcctttcctcgttcatggttcgatattctggtgtcctagg

cgtagaggaaccacaccaatccatcccgaatttggtggttaaactctactgcggtgacgatactgtaggggaggtcctgcggcaaaatagctcgatgccagaatga
taaaaagcttaacacctcttatttgactttttcactattttgaaatacgaaaaagatccaaatccaaaatgcaaaggtcgtcttattcaaaacctcaatcatcacatcccctc
tctcccacttcacacctcggaacgcactgttcttatagagagaaaggggctttcccatcttcttaacccgaaatgaaatggctgaggagagggaggttccttttgggg
ggtacccccgggaagagatccagtggagacggggtgggcctgtagctcagaggattagagcacgtggctacgaaccacggtgtcgggggttcgaatccctcct
cgcccacagccttccaaaggggaagggcctttactttcccccctgagggtaggaaaaccatgatcgggatagcggacgtaaagctattgaacttgggtatgctctttc
cttttgtcgaagtggaatcgtagaacagaatgtgatacgatgagataaaatgcaatagaaacaaggatagcgaacgggttacctactcctaagggtcaaagcaagc
cctttaattcaattctttattcttacattaaagaatgaatcaaatctccccaagtaggattcgaacctacgaccagtcagttaacagccgaccgctctaccactgagctac
tgaggaacaaggggggattcgacctcctagagttcaactcccgctctcaacccatgaacaatatgagtccgaagcttctttcgtaactcccggaatttcttcgtagtggc
tccgttccatgcctcatttcataggtaagcccagagtggctctatttcattctatttcacttcctagcacttcctatcatttaatatccatccctttggtcttattgacataagag
atgtcatttatagtctatctctttctatatatggaaagtcaagaaattctcatcgaaacatcgagaaattgtgcatatagaaaactctaaagaaagaaaaaaaggagacc
catgccatgattttcaaatcttttctacttagtagtctaagtttctcgatgaggataattaattcggtcgttgcggtcggactctattatgggtttctgaccacattctccatgg
gtccctcttagatcttctttctccaatcttggattagggaagaaggagatattcgcgactcctggtggtttcattatggggcagctcatgatcttcatatcgatctattatcc
acctctgcatctattctttcttagctaaacgggtggaagatccatccaatttggttatatcatggactcaaaaaacggatctgaatgtgactgaaatgcacgatcttcaca
ggtatcacttttcacgatacctaaaaggtggaatagcgattttcgaaccatttcctataagagaaaggtttccattactttgagaaatggattctatatcaaactatagctat
tgcattaaagaagaaaagaaactaatagaagtcgaagacgcggaatggtagtgaatagagagaaagattcttctggttttcttgttcctgaaaatattctatctatctcct
agacgccgtagagaattgagaattttcatgtctttcaattctcgtactcgtaattggaaagttacggaaggagatccatcattttgcaatgaaaactacataaaaaactct
ggacaatttcgaaatcaggccaagcgtcttaatacatatgcaaaaaaattcattattggcccaccattgattagaagatttaacttgtatgaatcgctattggtttgatacg
aataatggcagttgtttcagtatgttaaggatacagatgtatccacaattcatttagagttacttaatagcctatttcttataccatatctctatcccgtgaaattctcgagcc
gaaagatggatgcatatgctatgtttcattttgctaaatgatatcaattaaacggtgtatcaattccataaattggatatagcaataaataaatcagcaaaattcttttatttta
gatagaagaaaagtttcttctatctaaaataaaagaatgtacccttctatccaaatccaatttgcatcgataaaataaatccaaattccagtagtagatgaataattgcaaa
tttttgtgtgtacgagattagaataacttcaaaataactgacataatttttttatttttcctgatcagaaaaatacatgaaaaagaaaggaggtagaaaaatttttggatttatg
gttaaagaagaaaaagaagaaaactggggttctgttgaatttcaagtattcagtttcaccaataagatacggagacttgcttcacatttggaattacacaaaaaagattt
ttcatcggaaagaggtctccgaagacttttgggaaaacgtcaacgtttgctggcttatttggcaaagaaaaatagagtacgttataagaaattaatcagtcagttggat
attagggagcggtaatttcatcgttcgaatttttttttcttattttattagtagtcttatagtagtcttagattttgcattttgatgagcctcgttttgaggaattcatggaataatcc
attttcatggaataatgaattaaggaagaaaggatatgagtctaccgcttacaagaaaagatctcatgatagtcaatatgggccctcagcacccatcaatgcatggtgt
tcttcgactgatcgttactctcgatggtgaagatgttattgattgtgaacccatattaggctatttacacagaggaatggaaaaaatcggcaaataggaaagctacttag
gcaggagataggggaattccttaagaaagaaaaaagaataagaacacagatacataacataaaaaaaaagaataaataagacgaaattcgccctcccccctacatatt
taatttcttctcctatacaaaaactagcaagacctactccattggtaattccatcaatgacacccttgtcgaaaaactgcgttagttcggttaatcctcttatacccaaggt
aaaggtcctagtatagaaaatatctatataaccgcgattatatgaccaactatatatctttttttttagttgatggaaaaaatactttttcggaccccccttttacaaaggaattt
attaaatccaaattctgaaaaaaagagtaagcagatccataaaacatatatgctatgaatagaccaaaaatagctagacttacagaagaaattgcattagtgataaatt
catatgaatttatggaagaattagaactttcttggaaaaagttgattgagggagttagccactttgataatatggttaattcccctatttcattatcaaaatggattcctatag
atccaataaacaaagtacaaagcagtaatataagaagaggaaatagcatagtatttcccggttcatgaggatagacaaaagtgtttttagtccccaatgaagtactaa
aggaccctatcctatttcctgtattaccatgaattttggatagattttgtgaaaaaaaagaaactccactcttcgctgttgataaaacgaaatctctattgactcctttagata
tcctttttccccataacgatattgaatacaacgaatcctctttagtactactgtaattttgaaaatgaacacgcaaatacccatcaaaagtaagtaaatatatccgaaacat
ataaaacgcagttaatcctgcagtaaaagaagctattattccaaaaaagggtgaatataaccaactattactaaggatttcatctttggaccagaagcaagcaagagg
tggaataccacaaagagaaagggtaccccataaaaaacaagttcttgtaattggaatgtattttcttaaaccacccataagaaccatattctgacttttatctggtgaata
tccaacaagaggttccattgaatgaataacagatccggatcccaagaataataaagctttcgaataagcatgagtgatcaaatggaataaagcagcttgataagaac
ctatacctagagctaacatcatataacccaattgagacattgtagaataggctaagcttcttttaatatctctctgagcaagagctaaagtggctcctaagaaaagtgtta
gtgtacctattaaagaaatgaaactcattatcaaaggtagggatatgaaaagaggaagaagtcgagctataagaaaaatccccgcagcaaccatagttgctgcgtgt
ataagagctgaaatgggggtgggtccttccatagcatcgggtaaccatacgtgaagagggaattgtgccgatttcgcaactgcaccaaggaataataaaaaagca
cacaaaatagtaagcaaggagttaatctcattattaggaatccagttattagctattttaaacaaatcccgaaactctaaactacctgttatccaaaaaaaaacctagaatt
cctaataacagaccaaaatcccctacacgattagttacaaaagctttttgacaagcactcgctgcaattggccgtgtaaaccaaaagcctatcaataaataggaacac
attcccacaagctcccaaaaaaaaataaatttgtatcaaattggaactagtaaccaatcccaacatggaagtattgaaaaaacttatataaacaaaaaatctcaaatatcc
ctcatcgtgagacatataatcatcactataaataagaaccaggattcctacagtagtaattagtattaacataatagaagtaagcgggtcgattaagtatccaaattctaa
ggaaaaatcattattgacggtccaagaccatagatattgatagatagaacttccatttatttgttgaatagacaggtgaactgagaataccatagctatacttaaaagtaa
aacactaggaaaagcccatatgcgacgaagatttttttgttgctgttggaacaagaaaaagtccaaaccccattgacataataactggaagtgggagaagagggatt
acccatgcatattgatatgtatgttccataagaaaagaaattgcaatttttacttgaaaattttacttcaatttttctataaaattgaaaaaagtttccgattcaccaaactaatt
cttatctatttctgaaggaataaaaaaatactagaattcttaatttttcaaaaattttctcattgaaacaatcaaaaaataagaataggttttgttggttaaagtcaaaaagtta
atgaaataacttcgttacctagttattacctaaagaaggacttttattaaaatacaaaaaaaagattgaatcattttactttaatatttttttgtattaaaatgaagcagctccct
tgtttcgtaactcaaattgattggaattcaattctggaatactttaattaactatttgattgaattttcccttccttttatcctcccgtcttatatgggggataggcccccatacct
tatatctgtatatggagagtatacttgaaatataaattgatttaaatagaaaacctttgtatatattctatattattaaaacaaagtctaaaaaaaatatataatatgttaaaaa
actcttgtcttatccgcattagacaaaatgaagtaaaaaagaattcagaatttcaatatcttttagtatctaagtataaatactaagaaaaagaagaaagatggattgattt
gcggcaatagatgtctttcacatacaactagaaaaaagtaatttccttttttgaatggcagttccaaaaaaacgtacttcgatgtcaaaaaagcgtattcgtaaaaatcttt
ggaagaaaaagacttatttttccatagtacaatcttattctttagcaaaatcaagatcattttctggcgtcagcgagcatccaaaaccaaagggtttttctcggcaacaaa
caaacaaataatagggttttgggataatatgaattgacctatccccaaaaaaattccaattatttaatatgaataattaggaataattaggattaattaatgagtttacttttat
gtgtcgaattcctcggtacaatattcttagaacaaacctctctgatatataaaaaaagggtttttggtatactgtgacctaaatattcttttcctatcaatgaacttttcgtaat

agaatccgtataataaataaaaaaaggggattctattatgaaaagtagagtattcctgcaataagacttacaacttctacctatcttatcctaaattaacaaaaaaattagt
tctatattgcaactgagaaaaaattgttccaactctttcaaagtttctattgggcaaagcaagaattttttgttaaaaaattcgcaacgatactaccaaacgaagtctatttt
aatgaagattctaatgtcctaaattctatggaatcttccaatctcgacgattcgcgagaaaataacttaatattcttttaataaacctgttatttcaacttagccgccatggtg
aaattggtagacacgctgctcttaggaagcagtgctcaagcatctcggttcgagtccgagtggcggcagtctcgaaaaagaatacaatagattataaaataaaatgg
attcaattcgaaatttccaattttgtaatgggaccttctccttatgctatttgcaactttagaacatatactaactcatatttctttctcaacaatttcaattgtgattacgattcat
ttaataaccttattagttcgtgaacttgggggattgcgtgattcgtcagaaaaaggaatgatagccactttttctgtataacaggattcttagtttctcgttgggcttcttcg
ggacattttccattaagtaatttatatgagtcattgatcttcctttcatgggctctgtatattcttcatatgattcctaagatacagaactctaaaaatgatttaagcacaataa
ctacgccgagtactattttaacgcaaggctttgccacgtcgggtcttttaactgaaatgcatcaatccacaatactagtacctgctctacaatctcagtggttaatgatgc
atgtcagtatgatgttactaagctatgcgactcttttgtgcggatccttattatccgccgctcttctaatgattagatttcgaaagaatttagatttcttttcgaaaaagaaga
aaaatgttttgcttaaaacatttttctttaatgagattgaatatttctatgcaaaaagaagtgctttaaaaagcacctttttttcctttatttccaaattattacaaatatcaattaatt
gagcgtttggattcttggagttatcgtgtcattagtctagggtttacccttttaaccataggtattctttgtggagcagtatgggctaatgaggcgtggggatcctactgg
aattgggatcctaaagaaacttgggcatttattacttggaccatatttgcaatttatttacatagtagaacaaatccaaattggaagggtacgaagtcagcatttgtagctt
ccataggatttcttataatttggatctgttattttggtatcaatctattaggaataggtttacatagttatggttcatttacattacccatctaaatgattacataacataaaacct
taatgaaatggaaaaaacttccatttttgtgtttgatttgagaaccccttgaacgccttctcaaagggttctcaaaaattcgagatagatctaattagactcttttactttttttc
tgaatttttttagtatttccactatggaatatagagcggactagtagaagaaaaaaaaatcctatttaggataataattggataacagagcctctaccctgtcaacggatag
cgagagaacaaaatctggataaataccgattcctattactggtaaaaagatacagattaaaagaaagagttctcgcgggccggaatcctcaaaattttcgtttggaac
atgaaatagcttgtatccatagaacatctgtcgtaacatagataataaataaataggagttaatatcattccaattgccattacaaaagtaattagcatttttggcattaaca
gaaattttggactagtaattagtccaaaaaatactactaattccgcaacaaaaccactcattcctggtaaggcaagagaagccattgaaaagctactaaacatggtaa
aaatttttggcattgggatagaaacccctcccagttcttcgagataaacaaggcgcattctatcacaagccgttcccgctaagaaaaaaagtgtagccccaataaatc
catgggataatatttgtaaaatagctccattgagtccaatgttggttatggaaccaattcctataataatgaaacccatgtgagagacggaggagtaggctattcttttttt
gaaattgcgttggccaagagaagttgaagctgcatagattatttgcatcgctcctattattactaaccaaggggaaaatagataatgagcatgaggtaacaattccata
ttgatccgaatcaatccgtatgctcccatctttaataggattcccgctaaaagcatacatgtactgtaatgcgcttccccatgggtatctggtaaccacgtatgtaggggt
ataatcggcaatttgacagcataagcaataaggaagccaaaataaaatagtatttccaatgttgcagggtatgattgattaattaatctttccaaatctaatcttggttcgtt
ggaaccgtataagcccatacctagaactccgattaagaaaaaaatggaaccacctgcagtatacaaaataaactttgtagctgaatagagacgcctcttcccccccc
acatggataaaagtaagtaaacaggaattaattctaactcccacatgataaaaaaaaagtaaaaggtctcgcgaagaaaataatcctatttgaccgctatacattgctag
catcaggaaatagaataatcgggaattccgggtaaccggccaagctgctaaagtagctaaagtagtcataaatcctgtcaataaaatagatcctaatgaaagtccat
cgattcccaatctccagtggaaattgaagacatctatccatttagaatcctcttttaattggattaagggatcctccaattggaaatgataacagaatgcataagtcatta
gaaggaattctaataaacaaatagacatagtataccacctaacgattttgtttcccctatgaggtaaaaagaaaattaatgaacccgcaaatatcggcaaaacaacaa
gtattgttaaccaaggaaaagaactcatgataaagtgataaagacaagatacgttttgaccagaaaagcccgtgctcgattattttgagcacaggcttcttcggtaaag
aggaatcagacgattcaagtggaatttttttgtaacgtatcaataagatagagccatgctgcgggttgtctcaggtcctaaataaacgcggacgcttaaaaaatctgttg
ggcaggcggattcgcatctcttacaacccacacaatcttcggttcttggcgcggaagcaatttgcttggctttacatccatcccaaggtatcatttctaatacatctgttg
gacaagctcgtacacattgagtgcatcctatacatgtatcataaatttttacggaatgtgacattggatctataaattttcctttcaacataaaaattttcgatctggtcaaa
atgaaattagtactatatcaatcaaatgtattgtagacaccagacgaagcaatggtttatccaaacttcaacaaataatgcaatatatttcttaatccgtttgtgagaaagc
atgaaaagagccaagagacttgaattttgggcttcaacaatcataattatacgaattgtatatacgaattcgaattagccaataaattggctatcgtcttttcaatataaatt
attgcaatattcaaattgcaatatcaatgaattgcaaaaattcaactaagtaaaaaagaatactatggaataacctactcaaaaaatagatattctcaaataataaatagta
ttcatgttaatatttcatcaaataataaatagtattcatgttaatatttcatattattattatatgtgtccctttgtttagaagattctatgtctaattattcaaaaaattagattgattg
atacgagttgatttcctattacgatggatggaagaaagaatggataatccaatagctgcttcagcagccgcaagggctataacaaaaattgcgaaaatgtctcctttta
attggcggctatcaaatagatcagaaaatgttacgagatttagattaattgaattcagtataagttcaaggcatattagagctctaaccatgtttcggcttgtgatcaatcc
atagataccaatcgaaaataaatagacactcaaaaaaagtacatgctcaaacatcattaactaactccttatcaatctcgattcatttcaatatggggacaagaattgaa
ccgattgaattaattagaatagaacaattacacaacaaaagagaaaagaaggtatttgttggcagtagatgggttttactaaatcaaaattgtggttctttagtgatttattt
tagatttgaaattcttataattttgactcattctaagtatttcttattgccgagccatagtaattgcacctattaaagaaactagaagaattatggaaatgagttcaaatggaa
gataaaaatcggttgctaaatgaatcccaatttgttgaacgttatttatgagaccctgttctactatttggtttgatcttgtagtccaaagaattccataccatgacgtatctg
ggatagtagtcattagtgaaaaaggaatagttatacaaacgagtgaagtgaacccatctccaatagtccaaaaattcttatctttagaccattctgagccatttacgaac
attacggcaaatatgatcaagacatttatagctcccacataaataagaagttgtgccacagctacaaagtaggaattcaataaaatatagaataaggatatacaaacaa
gaactaatcctagcgaaaaagcagaaaaagttgggttggtaagtaataccacccctagacccccctagtagaagaacaaatcccccaaatagcacaagaatttcatg
tattggcccaggtaaatccattatggataagaagaaattaatagtataaatttttttcatgaactgactaaaactaaaagattcaaggaagaaaaaagggattaggaaatt
tttttgtatattgtatataagttctttctatagttagaaatcacatcacgaaaatctactctggtttaaaatcaggaataatttgcaataagcagtaggtattcgtttttctttcta
gttagtaaagaacttttggattctaacaaaaaaattctagtaatcagtaatcgttcttgaattccaagatttttcttcgtctattttactttgagttgaattcctaattgtttgaatt
gtgtaatctcccattatggagattggtaaccgactcaaagcaatttgattgtaattcaattcatgacgatcataagtagaaagttcatattcttcagtcattgataaacagtt
tgtcggacagtactcaacacaattaccacaaaatatacaaactccgaaatcaatactataattaagcaattgtttccttttaatatccttttcaaatctccaatccacaaga
ggtagatctatagggcatacgcgaacacatacttcacaagcaatacatttatcaaattcaaagtggattcgcccccggaaacgctccgatgtaattgatttttcatagg
ggtagtgaatcgttataggtaaacgatttgtgtgggataaggtaattatgaaactttgaccaatgtaccttgctgcgcgtattgtttgttgaccataactcatgaacccagt
taccatagggaacatattcgaaatatctatgaaaaaggtatgtttctttctcttgtttgagaggacttttgtgttgaaaatattcttactgttattgtattatcttattttatagtga
aacaagttgggaagaagttgttaataagagattgcccagggaaataggtaaaagaaatttccatccaagatttaataactgatccattctcatcctgggtaaagtccat
cttattgtgatagaaatgaagagaaataaataagctttagttaatgtaataaagatactcattgtcatttctaaaattccaaccattttattcatttggaaaaatccaaaaaag
gatatatagggaatagacaaattccacccgcctaagtagagaactgttacaaataaagaggaaactaataaatttaggtaagaacaagataaaataaaccatatttg

ataccagaatattcggtttgataacctgctactaattcttcctccgcttctggtaaatcaaagggtaatctttcacattccgccaaagaagaaattagaaaaaccagaaa
acctatgggctgacgccaaagattccatccaaaaaaaccatattttgactgtgcttcaactatatcaactgtacttgaactgttggataatcatagtcgacgataacatca
cagttcccaccgctattccaaaaccgtacatgaaaccttagtttcatacggctcctctatgatcagaaaaaaggaaagtactgtttcatttcgttattatcttcttgggcgt
agttagaattatctaagataaaatcgatttcaacgtcctaaattagaccaaaggaattctgtctgctagaataataaaaaacgcttcggaattcatctcatcctttataatat
aaaggtactttttctttgttcagcaataacttaatcttggaataaaacactcgttataacaattaataaacgaaaagagttgggtattagttcatgaagaattctgtatgaat
atggataaacgacggaaagaataaataaagatcttttttttgtattgcattccatatcttttgtcctattcttctttccccgaggggtatttaaaaagaaaaaaggaataaa
gggttaattcgttcttgatagccatttccttaacaagtgaatgggaacatactctggatcggaatccgaagaaagtactacttgctcatttccaccaatttcaagtccttat
tatgattccttttatgaggaaaaatatctaatgctttagattccctcattactaatcctttatgtactttagtgtttctaatccctcactaacttttgatggattcccttatgattaca
actttctgtatcgggaatcccttattattgcccgcttcaagatatgatgactaatcaaaaaatctcaaccttggggtaaagaatttacaccgcttatgtttacttccattttttc
ttgtacataggaaatgagattttttctttttactacaaattaataagcagttttgtttcactcatatagctatctagtttaacttactaacctgaatatagaataagaaaaggag
gataaatattcaatgaatttcagaggaaaaagatcctattttaacgaatcgcacgtagagatattgctagtacacaaaaagttaatgggatttcataactaatagattgag
cggcagctcgtagaccacctgaaaaagaatatttattatttgagctatatcctgccataagaagaccaataggagcaatacttgaaatggcaatccataaaaaaacac
caatactaagatccgctaaaacaaagcgatatcccaaaggaataactaaaaaacttaataaaattgatatgactgctatagacggtccaatgctaaataagggaatat
cccctcgggatggcaagatatcctctttaaaaagtagcttagttccatctgctatagcttgaagcagtcccagggggccagcatattcaggaccaatacgttgttgtat
cgatgcggatatttctctttctaaccacacaattacgagtacttctattgtgattcccagtaagagggtcaaaatgggtagaatccatatcagtccatagacttcttttaata
attccaagttcgaaaaagaattgatagcttctacctgtaccctgtctattatcatttcaacgatcaacttctcccataatgatatctatactacctaatatcgtcatgatatca
gccaatttcattttttgactagctgaggaagaatttgcaaattaataaaaccgggtggacgaattttccatctccaggggaaaagactatcatctcctaccagataaatt
cctaattcaccttttggggcttccactcttgcataaagctcttgttttgacaattcaaaattgggtgaaggtttttttaccaagaaatcgatattcaaaatcattccattcggaa
ttctttgctttcttaaagcgtcggacttctaaattctcataagggccccccggaatttttttctacagcctgttgaataattttgattgattccctcatttcaccgattcgtactaa
atagcgtgctaatgaatccccttctttttgccattggactttccaatcgaattgattgtaagactcgtaaagatcaactttacgaagatcccattgtattccagaagctcgta
acatggggcccgataagccccaatttacagcttcttctccgctaataaaaccaactccctcaactcgttccaaaaaaatgggattctgtgtaataagttgttgatattcaa
taactcctcgtaaaaaataatcacagaaatctaaacatttatcgatccatccataaggcagatcggcagcaactcctccgatgcgaaagtaattatgcatcattcgcat
acctgtagcagcttcaaatagatcatatattaattctctctctctaaaaatataaaaaaaaggagtctgcgcaccgagatctgccataaaaggtccaagccataacaag
tgagaagctatacggctcaattctaacataattaccctaatatagctggctctttggggtatttgaatattctccaagaattctggtgcatttaccgttattgcttctgtaaac
atagtagctaaataatcccaacgtgttacataaggtaagtattgtataatagttcggttttccgcgatttttttccattcctctgtgtaaatagcctaatatgggttcacaatca
ataacatcttcaccatcgagagtaacgatcagtcgaagaacaccatgcattgatgggtgctgagggcccatattgactatcatgagatcttttcttgtaagcggtagac
tcatatcctttcttccttaattcattattccatgaaaatggattattccatgaattcctcaaaacgaggctcatcaaaatgcaaaatctaagactactataagactactaataa
aataagaaaaaaaattcgaacgatgaaattaccgctccctaatatccaactgactgattaatttcttataacgtactctatttttctttgccaaataagccagcaaacgttg
acgttttcccaaaagtcttcggagacctctttccgatgaaaaatctttttttgtgtaattccaaatgtgaagcaagtctccgtatcttattggtgaaactgaatacttgaaattc
aacagaaccccagttttcttctttttcttctttaaccataaatccaaaaatttttctacctcctttctttttcatgtatttttctgatcaggaaaaataaaaaaattatgtcagttatttt
gaagttattctaatctcgtacacacaaaaaatttgcaattattcatctactactggaatttggatttattttatcgatgcaaattggatttggatagaagggtacattcttttatttt
agatagaagaaaagtttcttctatctaaaataaaagaattttgctgatttatttattgctatatccaatttatggaattgatacaccgtttaattgatatcatttagcaaaatgaa
acatagcatatgcatccatctttcggctcgagaatttcacgggatagagatatggtataagaaataggctattaagtaactctaaatgaattgtggatacatctgtatcct
taacatactgaaacaactgccattattcgtatcaaaccaatagcgattcatacaagttaaatcttctaatcaatggtgggccaataatgaatttttttgcatatgtattaaga
cgcttggcctgatttcgaaattgtccagagtttttttatgtagttttcattgcaaaatgatggatctccttccgtaactttccaattacgagtacgagaattgaaagacatgaa
aattctcaattctctacggcgtctaggagatagatagaatattttcaggaacaagaaaaccagaagaatctttctctctattcactaccattccgcgtcttcgacttctatta
gtttcttttcttctttaatgcaatagctatagtttgatatagaatccatttctcaaagtaatggaaacctttctcttataggaaatggttcgaaaatcgctattccaccttttaggt
atcgtgaaaagtgatacctgtgaagatcgtgcatttcagtcacattcagatccgtttttttgagtccatgatataaccaaattggatggatcttccacccgtttagctaaga
aagaatagatgcagaggtggataatagatcgatatgaagatcatgagctgccccataatgaaaccaccaggagtcgcgaatatctccttcttccctaatccaagattg
gagaaagaagatctaagagggacccatggagaatgtggtcagaaacccataatagagtccgaccgcaacgaccgaattaattatcctcatcgagaaacttagact
actaagtagaaaagatttgaaaatcatggcatgggtctcctttttttctttctttagagttttctatatgcacaatttctcgatgtttcgatgagaatttcttgactttccatatata
gaaagagatagactataaatgacatctcttatgtcaataagaccaaagggatggatattaaatgataggaagtgctaggaagtgaaatagaatgaaatagagccact
ctgggcttacctatgaaatgaggcatggaacggagccactacgaagaaattccgggagttacgaaagaagcttcggactcatattgttcatgggttgagagcggga
gttgaactctaggaggtcgaatccccccttgttcctcagtagctcagtggtagagcggtcggctgttaactgactggtcgtaggttcgaatcctacttggggagatttga
ttcattctttaatgtaagaataaagaattgaattaaagggcttgctttgacccttaggagtaggtaacccgttcgctatccttgtttctattgcattttatctcatcgtatcacat
tctgttctacgattccacttcgacaaaaggaaagagcatacccaagttcaatagctttacgtccgctatcccgatcatggttttcctaccctcagggggaaagtaaagg
cccttcccctttggaaggctgtgggcgaggagggattcgaaccccccgacaccgtggttcgtagccacgtgctctaatcctctgagctacaggcccaccccgtctcc
actggatctcttcccgggggtacccccccaaaaggaacctccctctcctcagccatttcatttcgggttaagaagatgggaaagcccctttctctctataagaacagtg
cgttccgaggtgtgaagtgggagagaggggatgtgatgattgaggttttgaataagacgacctttgcattttggatttggatctttttcgtatttcaaaatagtgaaaaag
tcaaataagaggtgttaagctttttatcattctggcatcgagctattttgccgcaggacctcccctacagtatcgtcaccgcagtagagtttaaccaccaaattcgggat
ggattggtgtggttcctctacgcctaggacaccagaatatcgaaccatgaacgaggaaaggcatgagagaaatattggctagtaattgtgaagccccaattcttgac
tggaagggacaccaaaggcctctgccctccctctctatctatccaagagatggaagggcagagcttttttttggtttttttcatcttttcatcaaagagttgaacaatgaag
atagatggcaagtgcctgatcgatttgatcaggtcgtgtaggaacaaggttcaaatcgttcgttcgttaggatgcctcagctgcatacatcactgcacttccacttgac
acctatttaaacggctcgtctcgccgctaccttatcctatttccatgcttctgtcgctccatccccgtatgggtggagaacccgtcgctgtctcggctgtgctaccggag
gctctagggaagtcggaggagagagcactcatcttggggtgggcttactacttatatgctttcagcagttatcctctccgcacttggctacccagcgtttaccgtaggc
acgataactggtacaccagaggtgcgtccttcccggtcctctcgtactagggaaaggtcctctcaatgctctaacgcccacaccggatatggaccgaactgtctcac

gacgttctgaacccagctcacgtaccgcattaatgggcgaacagcccaacccttggaaccacctacagctccaggtggcgaagagccgacatcgaggtgccaaa
ccttcccgtcgatgtggactcttggggaagatcagcctgttatccctagagtaacttttatccgttgagcgacggcccttccactcggcaccgtcggatcactaaggc
cgactttcgtctctgctcgacgggtgagtcttgcagtcaagctcccttctgcctttgcactcgaggaccaatgtccgtctggcccgaggaaacctttgcacgcctccgt
taccttttgggaggcctacgccccatagaaactgtctacctgagactgtcccttggcccgcgggtctgacacaaggttagaatccgagctcttccagagtggtatctc
actgatggctcgggccccccggaagggggccttcttcgccttccacctaagctgcgcaggaaaggcccaaagccaatcccagggaacagtaaagcttcatag
ggtctttctgtccaggtgcaggtagtccgcatcttcacagacatgtctatttcaccgagtctctctccgagacagtgcccagatcgttacgcctttcgtgcgggtcgga
acttacccgacaaggaatttcgctaccttaggaccgttatagttacggccgccgttcaccggggcttcggtcgccggcttccctgtcatcagttcaccaacttccttga
ccttccggcactgggcaggcgtcagcccccatacatggtcttacgactttgcggagacctgtgtttttggtaaacagtcgcccgggcctggtcactgcgacccccttt
tgcgagggggcaccccttctcccgaagttacggggctattttgccgagttccttagagagagttgtctcgcgcccctaggtattctctacctacccacctgtgtcggtt
tcgggtacaggtacccctttgttgaaggtcgttcgagcttttcctgggagtatggcatgggttacatacttcagcgccgtagcgcctggtatgagcctcgtggagaag
caatggctagtccacggggctcatacttcagcgctgcagcgcttggtactcggacctcggctcgaggcattttctctacccccttcttaccctgaaaaagcagggtcac
cttgtgtccttaaacctataaccatctttcggctaacctagcctcctccgtccctccgtaccaacaaggggtagtacaggaatattgacctgttgtccatcgactacgcc
tttcggcctgatcttaggccctgactcaccctccgtggacgaaccttgcggaggaaaccttgggttttcggggcattggattctcaccaatgttttcgttactcaagccg
acattctcgcttccgcttcgtcgacccccgctttcgcggttgcttccctctaaggcggaacgctcccctaccgatgcattttgacatcccacagcttcggcagatcgctt
agccccgttcatcttcagcgcaagggcgctcgatcagtgagctattacgcactctttaaagggtggctgcttctaggcaaacctcctggctgtctttgcaccccccacc
tcctttatcactgagcggtcatttaggggccttagctggtgatccgggctgtttccctctcgacgatgaagcttatcccccatcgtctcactggccgaccttgacccctg
ttatttttgggtcatatctagtattcagagtttgcctcgatttggtaccgctcgcgcagcccgcaccgaaacagtgctttacccctagatgtccagtcaactgctgcgcct
caacgcatttcggggagaaccagctagctctgggttcgagtggcatttcaccccctaaccacaactcatccgctgattcttcaacatcagtcggttcggacctctgctta
gtttcatccaagcttcatcctggtcatggatagatcacccaggttcgggtccataagcagtgacaatcgccctatgaagactcgctttcgctacggctccggtgggttc
cgttcccttaaccaagccactgcctatgagtcgccggctcattcttcaacaggcacgcggtcagagatcactttcccctcccactgcttgggagctcagcacggtttc
acgttctatttcactacccactgggggttcttttcacctttccctcacggtactacttcgctatcggtcacccaggagtatttagccttgcaaggtggtccttgctgattcac
acgggattccacgtgccccatgctactcgggtcagagcgtaagctagtgatgctttcggctactggactttagccatctagggtgcggcactcaaccgcttcgccta
gcagcacaacgcttgtattgctctcccacaaccccgttttcacggtttaggctgctcccatttcgctcgccgctactacgggaatcgcttttgctttcttttcctctggcta
ctaagatgtttcagttcgccaggttgtctcttgcctgctcatggattcagcaggcagttcaaaaggttgacctatttgggaatctccggatctatgcttattttcaactccc
cgaagcatttcgtcgcttgctacgcccttcctcgtctctgggtacctaggtatccaccgcaagcctttcctcttttgaacctcgccattaacgttaaggctatgccatcct
aaggtgctactaaatggaaggatcttatcaacgtccatgaatgcgaaatcatagatcgaactgccgaattggcaaaattcggtgctatcatagtatccgctaagttcac
gggctggagataagcggactcgaaccgctgacatccgccacagggtaaaccaccgcctctcaggcctccccgacgggttctaccatagaggccaacgatagac
aataactcccccccgaacacagcttacaactttcatcgtactgtgctctccaaagagcaactcttctcaaaatctcaaaacaaaaggtgctgagttggaatcccattcta
aggattcttgtggttccggggaatccagctacaggagaaccaggaacggggagctctccccttttccgcccgactctttgatcttaaacttaagaatgctggttttaa
gaacgagtgattgcccttctccgacccttactgcccaaccggagagcggacggctaatgtgttccacttattgaacagggtctatggtcggtccgtgacccctggac
gccgaaggcgtccttggggtgatctcgtagttcctacggggtggagataatggggtcggtccatggattttccttccttttgccacatttcgctcaaagggttgaaggg
agatagtgcatcaagctattcgcaagggccaacttgatcctcttccccagggatcccagatgagggaagcctaggagagccgccgactccaactatcgtccatgta
cgatccatactagatctgaccaactgcccatcctacctcctctacctttttgacagcccatctttttgtctcagtagagtctttcagtggcatgtttcagtcctcttccccatt
acttagaaaaagtgagccaccggttcaggtacaagatactaccattaccgcctggacaattagacagccaacccgtaatcgcaacgacccaattgcaagagcgga
gctctaccaactgagctatatcccccgagccaagtggagtatgcatgaaagagtcagatgcttcttctattcttttccctggcgcagctgggccatcctggacttgaa
ccagagacctcgcccgtgaagtaaatcatcgcccctacgatccaaccaattgggagagaatcaatagactccttttcgggagcgattcatccttcccgaacgcagc
atacaactctccgttgtactgcgctcttcaagtgtgcttcttccccttctcccccttaccatggcaagtccttgggaaataactccgatgggcagaaaaaggaaggcgt
taagagaccctcctggcccaaccctagacactctaagatcctttttcaaacctgctctgctcccatttcgagtcaagagatagataaatagccacatcccattgcactg
atcgagggcgctcgtagtgacttagggagtcgaagaccaagaagtggcttatttataccaagcattcctcttatggctagatccaacctcctggtccctgcggaaag
gaaaaagaatttcacgttcttcctttcaggaagggaggattagggaagtcctattgattgctgctttctccagaccgccgggaaaagcatgaaaaaaaggctcgaat
ggtacgatccctccgtcaccccagaatgaaagggtgatctcgtagttcttggtctgtgaagatgcgttgttaggtgctccattttcccattgaggacgaacctcaacct
gtgctcgagagatagctctccatacactgataagggatgtatggattctcgagaagagaggagccgtagtgcccccccggaccgcccggatcccacgagtgaa
tagaaagttagatctacatgggatctcacctgaatcgccccatctatcctcctgaggagaagtttgtttggtttcaaactccgattcaaacaggaggagtacgccatgc
taatgtgccttggatgatccacatcttcgggtcaggcgctgatgagcacattgaactatccatgtggctgagagccctcacagcccaggcacaacgacgcaattatc
aggggcgcgctctaccactgagctaatagcccgtcgcgcgggcctccctttgggaggcctgctacgccaaaagcgagaaaaactccatccctttccttttgacatc
cccatgccgccacaccacacgggggggggcatggggacgtcaaaaggggatcctatcactatcaactaatttgttccgacctaggataataagctcatgagctt
ggtcttacttcaccctaaacgaaagaagacttccatatccaagtttagctcagacgtagctgccttctttttgggcgtgaagcagtgtcaaaccaaaatacccaataag
cataagcattagctctccctgaaaaggaggtgatccagccgcaccttccagtacggctaccttgttacgacttcactccagtcgcaagcctagccttaggcatcccc
ctccttacggttaagggtaatgacttcaaacatggccagctcctatagtgtgacgggcggtgtgtacaaggcccgggaacggattcaccgccgtatggctgaccgg
cgattactagcgattcctgcttcatgcaggcgagttgcagcctgcaatccgaactgaggacgggtttttggagttagctcaccctcgcgagatcgcgaccctttgtcc
cgcccattgtagcacgtgtgtcgcccagggcataaggggcatgatgacttggcctcatcctctccttcctccggcttaacaccggcggtctgttcagggttccaaact
catagtggcaactaaacacgagggttgcgctcgttgcgagacttaacccaacaccttacggcacgagctgacgacagccatgcaccacctgtgtccgcgttcccg
agggcacccctctctttcaagaggattcgcggcatgtcaagccctggtaaggttcttcgctttgcatcgaattaaaccacatgctccaccgcttgtgcgggcccccgt
caattcctttgagtttcattcttgcgaacgtactccccaggcgggatacttaacgcgttagctacagcactgcacgggtcgagtcgcacagcacctagtatccatcgtt
tacggctaggactactggggtctctaatcccatttgctcccctagctttcgtctctcagtgtcagtgtcggcccagcagagtgctttcgccgttggtgttctttccgatctc
aatgcatttcaccgctccaccggaaattccctctgcccctaccgtactccagcttggtagtttccaccgcctgtccagggttgagccctgggatttgacggcggactt

gaaaagccacctacagacgctttacgcccaatcattccggataacgcttgcatcctctgtcttaccgcggctgctggcacagagttagccgatgcttattcctcagat
accgtcattgtttcttctccgagaaaagaagttgacgacccgtgggccttccacctccacgcggcattgctccgtcaggctttcgcccattgcggaaaattccccact
gctgcctcccgtaggagtctgggccgtgtctcagtcccagtgtggctgatcatcctctcggaccagctactgatcatcgccttggtaagctattgcctcaccaactag
ctaatcagacgcgagcccctccttgggcggatttctccttttgctcctcagcctacggggtattagcaaccgtttccagttgttgttcccctcccaagggcaggttctta
cgcgttactcacccgttcgccactggaaacaccacttcccgttcgacttgcatgtgttaagcatgccgccagcgttcatcctgagccaggatcgaactctccatgaga
ttcatagttgcattacttatagcttccttattcgtagacaaagcggattcggaattgtctttccttccaaggataacttgtatccatgcgcttcagattattagcctggagttc
gccaccagcagtatagccaaccctaccctatcacgtcaatcccacaagcctcttatccattcccgttcgatcgtggcgggggggagtaagtcaaaatagaaaaaactc
acattgggtttagggataatcaggctcgaactgatgacttccaccacgtcaaggtgacactctaccgctgagttatatcccttccccgtcccctcgagaaagagaatt
aacgaatcctaaggcaaaggggcgagaaactcaaggccacccttcctccgggctttctttccacactattatggatagtcaaataatgggaaaaattggattcaattg
tcaaccggtcctatcgaaaataggattgactatggattcgagccatagcacatggtttcataaaatctgtacgattttcccgatctaaatcgagcaggtttccatgaaga
agatcttgttcagcatgttctattcgatactggtaggagaagaacccgactcggtattcttaaaaaaagaggggaagcagaaccaagtcaagatgatatgggtcgcc
ccttcttcttgcgccaaagatcttaccatttccgaaggaactggggctacatttcttttcaatttccattcaagagtttctatctgtttccacgcccttttttgagacctcgaaa
catgaaatggacaaattccttctcttaggaacacatacaagaaaaaggataatggtagccctcccattaactacttcattttcatttatgaatttcatagtaatagaaatcc
atgtcctaccgagacagaatttcgaacttgctatcctcttgcctaataggcaaagattgacctctgtagaaagactgattcattcggatcgatatgaggacccaactac
gttgcattgccagaatccatgttccatatttgaagagggttgacctctgtgcttctctcatggtacaatcctcttcctgctgagcccccttctcctcggtccacagagaa
aaaatggaggactggtgccgacagttcatcacggaagaaagaactcacagagccgggatcgctaactaatagaatagtactactaactaatactaatatatagaaat
agatatctagaaatagaaacgaactaatatatagataatcgaaattgaaaagaactgtcttttctgtatactttccccgttctattgctaccgcgggtcttatgcaatcgat
cggatcatatagatatcccttcaacacaacataggtcatcgaaaggatctcggacgactcaccaaagcacgaaagccagttagaaaatggattcctatttgaagagt
gcctaaccgcatggataagctcacattaacccgtcaattttggatccaattcgggatttttcttgggaagtttcgggaagaaattggaatggaataatatagattcatac
agaggaaaaggttctctattgatgcaaacgctgtacctagaggatagggatagaggaagagggaaaaatcgaaatgaaataaataaagaataaagcaaaaaaaa
aaataagtcgaagatagaagagcccagattccaaatgaagaaatggaaactcgaaaaggatccttctgattctcaaagaatgaggggcaaggggattgataccga
gaaagatttcttcttattataagacgtgatttgatccgcatatgtttggtaaaagaacaatcttctcctttaatcataaatggaaagtgttcaattagaacatgaaaacgtga
ctcaattggtcttagttagtcttcgggacggagtggaagaagggcggagactctcgaacgaggaaaaggatcccttcgaaagaattgaacgaggagccgtatgag
gtgaaaatctcatgtacggttctgtagagggacaggaagggtgacttatctgtcgacttttccactatcaaccccaaaaaacccaactctgccttacgtaaagttgcca
gagtacgattaacctctggatttgaaatcactgcttatatacctggtattggccataatttacaagaacattctgtagtattagtaagaggaggaagggttaaggatttac
ccggtgtgagatatcgcattattcgaggagccctagatgctgtcgcagtaaagaatcgtcaacaagggcgttctagtgcgttgtagattcttatccaagacttgtatcat
ttgatgatgccatgtgaatcgctagaaacatgtgaagtgtatggctaacccaataacgaaagtttcgtaaggggactggagcaggctaccatgagacaaaagatctt
ctttctaaagagattcgattcggaactcttatatgtccaaggtcaatatggaaattctttcaggggttttcccttactttgtccgtgtcaacaaacaattcgaaatacctcga
ctttttcagaacaggtccgagtcaaatagcaatgattcgaagcacttctttttccattacactatttcggaaacctaaggacttgatggtatggatatggaaaatacagga
tttccgatcctagcgggaaaaggagggaaacggatactcaattttaagtgagtaaactgaattccatactcgatctcatagatccctatagaattctgtggaaagccgt
attcgatgaaagttgtatgtacggcttggagggagatctttcctatctttcgagatccaccctacaatatggggtcaaaaagccaaaaaaataagtgattcgtttttagc
ccttataaaaagaaaacggattcttgaacctctttcacgctcatgtcacgtcgaggtactgcagaaaaaagaactgcaaaatccgatccaatttttcgtaatcgattagt
taacatggtggttaaccgtattatgaaagacggaaaaaaatcattggcttatcaaattctctatcgagccgtgaaaaagattcaacaaaagacagaaacaaatccact
attggttttacgtcaagcaatacgtagagtaactcccaatataggagtaaaaacaagacgtaataaaaaaggatcgacgcggaaagttccgattgaaataggatcta
aacaaggaagagcacttgccattcgttggttattagaagcatcccaaaagcgtccgggtcgaaatatggctttcaaattaagttccgaattagtagatgctgccaaag
ggggtgggggtgccatacgcaaaaaggaagcgactcatagaatggcagaggcaaatagagctcttgcacattttcgttaatccatgaacagaatctaggtatgtag
acacatggatccatacatctcgatcggaaaagaatcaatagaaggagaatcggacgatatctttctcgaaacaaacaaaaaggaaaagaaagagaaaacagaaat
catgatcaactaagccctctcgggggcttgcttaagaataagaaagaggaatcttatggaaatagcatggaataaggtttgatcctattcatggggattccgtaaatat
cccattccaaaaatcgaaacaatcgggacttttcggagattggatgcagttactaattcatgatctggcatgtacagaatgaaaacttcattctcgattctacgagaattt
ttatgaaagcgtttcatttgcttctcttccagggaagtttcattttcccagaatgtatcctaatttttggcctaattcttcttctgatgatcgatttaacctctgatcaaaaagata
gaccttggttctatttcatctcttcaacaagtttagtaataagcataacggccctattgttccgatggagagaagaacctataattagcttttcgggaaatttccaaacgaa
caatttcaacgaaatctttcaatttcttattttattatgttcaactttatgtattcctctatccgtagagtacattgaatgtacagaaatggctataacagagtttctgttattcgta
ttaacagctactctaggggggaatgtttttatgtggtgctaacgatttaataactatctttgtagctccagaatgtttcagtttatgttcctacctattgtctggatataccaaga
gagatctacggtctaatgaggctactatgaaatatttactcatgggtggggcaagctcttctattctggttcatggtttctcttggctatatggttcatctgggggggagat
cgagcttcaagaaattgtgaacggtcttatcaatacacaaatgtataactccccaggaatttcaattgcgcttatatccatcactgtaggacttgggttcaagctttcccc
agccccttttcatcaatggactcctgacgtctacgaaggagtgtggttcgttcgacaaattcctacctctatatctatctctgaggtgtttgggttttgcaaaactccatag
atatgcagaagagaaatgctatccccactccgaccaagacagaacttttaccaaaagtttattgtgatctttttgttcaaataacaattaaggtgaagcagggtcagga
acaacgaatctctttatgataaacagatccattttgcaagctcgttattacgggtagttcctacaaagaatcggactaatgacgtatacaatgcttgaattatcgacgtag
atgctacatagtgggttctcatccttcagagactacgagtgtaataggagcatccgttgacaaaaggatcaccctaagatgatcatctcatggctattgggaacgaat
caaatcagatggttctatttctcaacctttctgacttgctcctacggaaccaaggtcgaaaggattgaaaaagtcagtcattcacaaccactgatgaaggattcctcga
aaagttaaggattagtagttctttttcgaaatcgatttcgaaaaagaatggattcggtcttatacatacgcgaggaaggtaatcaaaaaagaaagaagacaagttcttct
ttcttttatcacttaggagccgtgcgagatgaaagtctcatgcacggttttgcatgagagaaagaagcgaggaatcctcttttcgactctgactcccccactccagtcgt
tgcttttctttctgttacttcgaaagttgctgcttcagcttcagccacgcgaattctcgatattcctttttatttctcatcaaacgaatggcatcttcttctggaaatcctagctat
tcttagcatgatattggggaatctccttgctattactcaaacaagcatgaaacgtatgcttgcatattcgtccatagggcaaatcggatatgtaattattggaataattgtt
ggagactcaaatgatggatatgcaagcatgataacttatatgcttttctatatctccatgaatctaggaacttttgcttgcattgtattatttggtctacgtaccggaactgat
aacattcgagattatgcaggattatacacgaaagatcctttttggctctctctttagccctatgtctcttatccctaggaggccttcctccactagcaggtttcttcggaaa

actctatctattctggtgtggatggcaagcaggcctatatttcttggtttcaataggactccttacgagcgttctttctatctactattatctaaaaatagtcaagttattaatg
actggacgaaaccaagaaataacccccttatgtgcgaaattatagaagatcccctttaagatcaaacaattccatcgaattgagtatgactgtatgtgtgatagcatctac
tataccaggaatatcaatgaaccccattcttgcaattgctcaggataccctcttttagctgctaggtctatttcttagttcaagatccctcttactaactggaataaaagaat
tagtagatctgttccgcccaaaatgggaatgggcgctagggtaatgaacttataatcatggaatcgactcgatcatcagattataagttcattccataccggaccagac
cgtgcacattcttattatgagaaggggtcattcgagcctatggaaataggatactctgtttacatagaaatccccacgtccttacattctatttaggattaggaataggtgt
aatcagacctgcttttgacatatctatcctatccttatttgggtaccatatgcacctctttgggcttctattgaatcgagaaattggattgtacatctttttgattttgatacatat
aaggtgtcctacggataatgcaaatcgaagctatttgatgtctgactcaggcctatatgaccgatcgatcgaaatactccaagactccacctttgtcatatattccatata
tcacattagatagatatcatattcatggaatacgattcactttcaagatatcacattagatagatatcatattcatggaatatgattcactttcaagatgccttgatggtgaaa
tggtagacacgcgagactcaaaatctcgtgctaaagagcgtggaggttcgagtcctcttcaaggcataatacggagaatgcgcattcaatgagcattccccgtaga
agtattccggaaatctgcgcctggcgctctcctctatcttctgaggtccttaaccacttccctgagaaaaggagacagtaaaagccaaaatagactaaatatagcctg
aacgatcctaaaaatccctcgaaggagataatataataaagaacccaaagcagacggtatcctactgcaagggtggtcttaagaatccaaaagaggttgctcagaa
gagatagatgtatcccaacctctattgctctcgcgtaaagccttttttttacgcgacaggaaaaagtgactacgaattccccttttttgtttgcgaatccctgtttgtatccttt
gagcgcacgcccataagtagcgatcaaggaaatcgatcaaacgatcccaataccgtgaaagagaaacttcccagatccagggaagcttatcataaagggcttcga
caaggggttttattcgttctttgagcaaaaagataaagatcggatagattcgaaccgcaattgcaaaggtaatcactactatgccagcccaaatcatgatcttcaccaa
cttggatttggttctctcgcgaaaatcgcgagttgcagagatgagaaccatgaaaagcaagatcccgaataagaaaacagaaaccgaggaaaccacaagagtga
agactagtagagtctcgtcttttgtcattctttgctcctttttcactcaatgattcattcgaatttcccgaccaaaaattctatatgtctattcatagggcctcgttgctaagtgc
tacaagatctagtgcactggaactcgtggttatggacccgaatcctttagtatggaacattgtcttttccaagtaaaaaccccccagtatatgaaagaatgaaaaggtgc
tttcgttcttttcttgtggaataagaagccctcgtaccttaatgaaaggaaaataggaatttttcattaggtatttgaccaaataggatcgtccagttcctatagaacctatc
actaaaatacccgatagggctaagcggaacgaaaaggattttccctgagatggtaaatgaaaacgattagccccatacgaggtttgggaataagtgatgagcaag
gaatatacgtctttctgctaaagaggatctattaaactcataattcattagatccttgttatcaatgtcaactaggtatcataagtaaacggatcccggttgttcaatcctttg
ataaccaaggtcattctttgctaaagagaaatgatcactatgagtcagactcaatagaattggatccattccaaatagcgagaattaggattctggatccctctcaatct
ctctttcaattcgaggatccagagaggtgttttcatagtcatctccgaatatttgccatctccgaatattttcgatttcatttttctatgatatgtctttctatatgaaaattggtta
tttacgatgtacgatgatccctgttaagcatccatggctgaatggttaaagcgcccaactcataattggtaaatttgcgggttcaattcctgctggatgcacgcgaacc
ggaacgttccataagtctattggaactggctctctatccatggaatctcatccatcatccatacataacgaattggtatggtatattcataccataacataagaacaataa
gaactcgaattcttatcgatactggaactcagagcataggagggaaagtcgatttatggatggaatcaaatacgcagtatttacagaaaaaagtcttcgtttattggga
aagaatcaatatacttttaatgtcgaatcgggattcactaagacagaaataaagcattgggtcgaactcttctttggtgttaaggtagtagctgtgaatagccatcgact
acccggaaagggtagaagaatgggccctattctaggacatacaatgcattacagacgtatgatcattacccttcaaccgggttattctattccacttctagatagagaa
aaaaactaaaggagaatacttaataatacggcgaaacatttatacaaaacacctatcccgagcacacgcaagggaaccatagataggcaagtgaaatccaatcca
cgaaataatttgatccatggacggcaccgttgtggtaaaggtcgtaattccagaggaatcattaccgcaaggcatagagggggaggtcataagcgcctataccgta
aaatcgattttcgacggaatcaaaaagacatatctggtagaatcgtaaccatagaatacgaccctaatcgaaatgcgtacatttgtctcatacactatggggatggtga
gaagggatatattttacatcccagaggggctataattggagatactattgtttctggtacaaaagttcctatatcaatgggaaatgccctacctttgagtgcggtttgaac
tattgatttacgtaattggaagtaaccaattaggtttacgacgaaacctagaaatcgatcactgatccaatttgactacctctacgggatagacctcaacagaaaactgt
tgagtaacggcagcaagtgattgagttcagtagttcctcatagaaaattattgactctagagatatggtaatatggagaagacaaaattgtttgaagcacgcacagaa
ccggaagcgccccttgtttcaaagagaggaggacgggttattcacatttaatttgatggtcagaggcgaattgaaagctaagcagtggtaattaagacccccgggg
gaaaatagggatgtctcctacgttacccataatatgtagaagtatcgacgtaatttcatagagtcattcgatctgaatgctacatgaagaacataagccagatgacgga
acgcggagacctaggatgtagaagatcataacatgagcgattcggcagatttggattcctttcctatatatccactcatgtggtacttcatcatacgattcatataagatc
catctgtctagagatcgtcatatacatctagaaagccgtatgctttggaagaagcttgtacagtttgggaaggggtttttttgagagaaaagaagaatctacttcaaccg
atatgcccttaggcacggccatacataacatagaaatcacacgtggaaggggtgggcaattagctagagcagcaggtgctgtagcgaaactgattgcaaaagaa
ggtaaatcggccactttaagattaccatctggggaggtccgtttggtatcccaaaactgcttagcaacagtcggacaagtgggtaatgttggggtgaaccaaaaaag
tttgggtagagccgggtctaagtgttggctaggtaaacgccccgtagtaagaggggtagttatgaaccctgtggaccacccccatgggggcggtgaagggaaag
cccccattggtagaaaaaaacccacaacccccttggggttatcctgcgcttggaagaagaactaggaaaaggaaaaaatatagtgatagttttattcttcgtcgccgta
agtaaatacgtaactaggaatatggaaaattgcatttttggaatttgcaataatgggatgggcgaacgacgggaattgaacccgcgcatggtggattcacaatccact
gccttgatccacttggctacatccgccccttatccagctaaaggattttctctttttccattcatcattattctatttattctgacctccatacttcgatcgagatattggacat
cgaatgccactctttaaaatggaaaaaaaggagtaatcagctgtgacacgaaaaaaaacgaatccttttgtagctcatcatttattggcaaaaatagaaaaggtcaata
tgaaggaggagaaagaaacaatagtaacgtggtcccgggcatctagcattctacccgcaatggttggccatacaatcgcgattcataatggaaaggaacatatacc
tatttacataacaaatcctatggtaggtcgcaaattgggggaattcgtaccaactcggcatttcacgagttatgaaagtgcaagaaaggatactaaatctcgtcgttaa
ctgaattcagaatagaaagattcaaaataaaaaaaaaaaagaaata
>O.glaberrima_IRGC103486_cp
cccaatatcttgcttcagcaagatattgggtatttctagctttcctttcttcaaaaattgctatatgttagcagaaaagccttatccattaagagatggaacttcaagagcag
ctaggtctagagggaagttgtgagcattacgttcgtgcattacttccataccaagattagcacggttgatgatatcagcccaagtattaataacgcgaccttggctatca
actacagattggttgaaattgaatccgtttagattgaaagccatagtactaatacctaaagcagtgaaccaaatccctactacaggccaagcagccaagaagaagtg
taaagaacgagagttgttaaaactagcatattggaagattaatcggccaaaataaccatgagcggccacaatattataagtctcttcctcttgaccaaatctgtaaccct
cattagcagattcgttttcagtggtttccctgatcaaactagaggttaccaaggaaccatgcatagcactgaatagggaaccgccgaatacaccagctacacctaac
atgtgaaatggatgcataaggatgttatgctctgcctggaatacaatcataaagttgaaagtaccagatattcctaaaggcataccatcagagaaacttccttgaccaa
tagggtaaatcaagaaaacagcagtagcagctgcaacaggagctgaatatgcaacagcaatccaaggacgcatacccagacggaaactcagttcccactcacga
cccatataacaagctacaccaagtaagaagtgtagaacaattagctcataaggaccgccattgtataaccactcatcaacagatgcagcttcccaaattgggtaaaa

gtgcaatccgatcgccgcagaagtaggaataatggcaccagagataatattgtttccgtaaagtaaagaaccagaaacaggctcacgaataccatcaatatctactg
gaggggcagcgatgaaggcgataataaatacagaagttgcggtcaataaggtagggatcatcaaaacaccgaaccatccgatgtaaagacggttttcggtgctag
ttatccagttgcagaagcgaccccacaggcttgtactttcgcgtctctctaaaattgcagtcatggtaagatcttggtttattcaaattgcaaggactcccaagcacacg
tattaactagaaagataatagaaggcttgttatttaacagtataatatagactatataccaatgtcaaccaagccagccccgacagttgtatatccatacaacaaaattta
ccaaaccaaaaaattttgtaaatgaagtgagtgaaaaatcaaaactcagattgctcctttctagtttccatatgggttgcccgggactcgaacccggaactagtcggat
ggagtagataattattccttgttacaatagagaaaaaacctctccccaaatcgtgcttgcatttttcattgcacacgactttccctatgtagaaataggctatttctattccg
aagaggaagtctactaattttttagtagtaagttgattcacttactatttattatagtacagagaacatttcagaatggaaactgtgaaagttttaccttgatcatttatcaat
catttctagtttattagttttgtttaatgattaattaagaggattcaccagatcattgatacggagaatatccaaataccaaatacgctcactgtgcgatccacggaaagaa
aagtaagttgttttggcgaacatcaaagaaaaaacttgctcttcttccgtaaaaaattcttctaaaaataccgaacccaaccattgcataaaagctcgtaccgtgcttttat
gtttacgagctaaagttctagcgcatgaaagtcgaagtatatactttagtcgatacaaagtcttctttttttgaagatccactgtgataatgaaaaagatttctacatatccg
accaaaccgatcaagaatatcccaatccgataaatcggtccaaattggtttactaataggatgccccgatccagtacaaaattgggcttttgctaaagatccaatgaga
ggagtaacagggactttggtatcgaatttttttcatttgagtatctattagaaatgaattctccagcatttgattccttactaacaaagaatttattggtacacttgaaaagtac
cccagaaaatcgaagcaagagttttctaattggtttagatggatcctttgcggttgagtccaaaaagagaaagaatattgccacaaacggacaaggtaacatttccatt
tcttcttcaaaagaagagttccttttgatgcaagaattgcctttccttgatatcgaacataatgcataaggggatccataacgaaccatatggttttccgaaaaaaagcag
ggtacattaacccaaaatgttccatcttcctagaaaagatgattcgttccagaaaggttccggaagaagttaatcgcaagcaagaagattgtttacgaagaaacaaca
agaaaaattcatattctgatacataagagttatataggaaccgaaatagtcttttattttctttttcaaaataaaaatggatttcattgaagtaataaaactattccaattcga
gtagtagttgagaaagaatcgcaataaatgcaaggatggaacatcttggatccggtattgaaggagttgaagcaagatatccaaatggataggatagggtatttctat
atgtgctagataatgtaagtgcaaaaatttgtcttctaaaaaaggaaatattgaatgaatagatcgtaaattctgaaactttggtatttctttttcttccggacaagactgttc
tcgtagcgagaatgggatttctacaacgatcgcaacccccctcagatagaatctgagaataaaactcagaataaaaaaaaattgttgtaatccaataatcgatcttggtta
ggatgattaaccaaattaatccaaaaattctgctgatacattcgaatcattaaccgtttcacaagtagtgaactaaatttcttgttattagaaccaataatttcgacaagttc
ggaaccatttaatccataatcatgggcaaacacataaatgtactcctgaaagagtagtgggtagacgaaatattgtctaggaaatttaagtttttctgaataaccctcga
atttttccatttgtatttctacttgaatcagagagagagaaatatttctcggtttatcaaatggtgatacatagtacaatatggtcagaacagggtgttgcatttttttaatacaa
accccctggggaagaaaaggagtctaatccacggatctttttccgctccttttctatccaatttgtttatgtttgttctaattacaaaagagaacaaatcctttatttttgcagg
ccaattgctcttttgactttgggatacagtctctttatcaatatactgcttcttttacacattcaatccataacatcctttttcaatccaaaatcaagaataattaggatttctaaa
aaaaaaaaaagaaaaaatcaaaggtctactcataggaaaaccagcttttccctacatcaggcactaatctatttttaacgtctaattagatcagggagttcttccaattaa
gaagttaagctcgttgctttttgttttaccagaattggagccaggctctatccatttattcattagacccagaaaatcagaatttttttattccattccaaaaatccaaaataa
gaaattgattttattacgacatgctattttttccattcattacccttgaggatcagtcgcggtcttatagactctaccaagagtctggacgaattttttgcttcatccaaatgtg
taaaagatcatagtcgcacttaaaagccgagtactctaccattgagttagcaacccagataaactaggatcttagatacgatcgaaatccaaaaatcaatggaattac
accgcacacccctgtcaaaatcttaaaatagcaagacattaaaagaaagattttatcaccattgaaaacactcagataccaaaaggaacgggtctggttaaatttcact
aaggttaaaagtggcaccaatcacgatcgtaaaattgtcattttttttagcatttttatttaaataaataaataaatcttgtatgagagtacaaacaagagggacaaccctac
catttgagcaaagtgtaggcaaaaaacctaatagggagtgaggataaagagacttatccatctacaaattctagatgttcaatggacctttgtcaatggaaatacaatg
gtaagaaaaaaattagatagaaaaactcaaaaaaataaaggcttatgttggattggcacgacataaatccagtcaaaaataggattaagaaagaggcaaattatttct
aaatagttagacaacaagggatactagtgagcctctcctagtttttttattcatttagttcttcaattaactcaaagttctttctttttctttaaagaattccgccttccttaaaata
tcagaaactgttcttgtaggttgagcacctttttcaaggaaatagagaatagctggaacatttaaacaagtttgattctttatcggatcataaaaacctacttttcgaagat
ctcttccttctcttcgagatcgaacatcaattgcaacgattcgatagacagcttattgggatagatgtagataaacaaagcccccccctagaaacgtataggaggttttct
cctcatacggctcgagaatatgacttgcattaatttccgtacagaaaaaacaaatttcatttatactcatgactcaagttgactaattttgattgacagacttgaaagaaaa
aaatcctttgaaatttttttgagtcgtctctaaactcttttctttgcctcatctcgaacaaattcacttttattccttattccggtccaattctattgttgagacagttgaaaatcgtg
tttacttgttcgggaatcctttatctttgatttgtgaaatccttgggtttaaacattacttcgggaattcttattcttttttctttcaaaagagtagcaacatacccttttttcttattt
ccttcgataaagcatttccctcttctatagaaatcgaatatgagcgattgattctgatagacttttaatcaaaagagttttcccatatcttccaaaattggactttcttcttatttt
aaccttttgatttctatattatttcgatttctatattaagggtagaatgacaaagttggcctaatttattagttttcactaaccctagattctttcccttgataaaaaataaattctg
tcctctcgagctccatcgtgtactatttacttagcttacttacaaacaacccagcgaaaattcggttcgggacgaatagaacagactatgtcgagccaagagcattttc
attactatggaaaatggtggatagcaaaatccacaatcgatcgtgtccttcaagtcgcacgttgctttctaccacatcgttttaaacgaagttttaacataacattcctcta
atttcattgcaaagtgttatagggaattgatccaatatggatggaatcatgaatagtcattagtttcgtttttttgtatactaattcaaacttgctttgctatctatggagaaatat
gaataaaagaaattaagtatttatcgggaaagactccgcaaagagccaatttatttaaacccatattctatcatatgaatgaaatatagttcgaaaaaagggaataaaca
agtttgcttaagacttatttattatggaatttccatcctcaacagaggactcgagatgatcaatccaatcctgaaatgataagagaagaattgactcttctccaacaaata
aactatcaacctcccgtttaattaatttaattaatatattagattagcaatctatttttccataccatttttccgtaacaaaactaattaactattaactagttaaactattgcaat
gaaaagaaagtttttttggtagttatagaattatcgtatttcttcgactcgaataccaaaagaaagaaaaaaatgaagtaaaaaaaacgcatttcctgtaaagtaaaattaa
ggtctttgcttttacttatttttttcttttacctaaaagaagcaactccaaatcaaaattgaatccattctatctaacgagcagttcttatcttatctttaccgggatggatcattct
ggatatttaaaaaatcgcggatcgagatcgttttcgcttaaccaaagaaagaaaaagaagaaggaacctttttttactaataaaatactataaaaaaaaatttatctctatca
taaatctatctctaccataaaggaataggtctcgttttttatacaatgttctacgtcaagtttaaaattttttcatgaaaaaaagattttcaatttgactggacttgacactggat
tatgttttctgagacagaaaatgaacgcattaggactgcatcgaatctaagagtttataagagaaaaaaaattctctttaataaactttatgtctcgtgcagaatacaatac
gatttcatctttcgtttcatcagaaaaaatctgggacggaaggattcgaacctccgagtaacgggaccaaaacccgctgccttaccacttggccacgccccatttcgg
gttttatgcgacactaataaacagtattatttttatttcttattcgtcaatcctacttcaattacataaaaatggggggtattctcttggtaggattctagacatgcgaataatat
agaatccaaaaaatgcattgatcattacatggaattctattaagatattatatgaaagtcgaatttcttccactctcatttgagagtgcgaatacaaggaggtattttgtgttt
gggaaagtccgaagaaaaaaggattttgaatcctccttttcctttttcccttagaaaaataactcaatcaaaatccaattatctactctacaagaacgaaacgcttgttatg
cctaatatacttagtttaacctgtatttgttttaattctgttatttatccgactagttttttcttcgccaaattgcccgaagcttatgcgattttcaacccaatcgtggattttatgc

ctgtcatacctgtactctttttctattagcctttgtttggcaagctgctgtaagttttcgatgaaatctttactactctgtctgccaaattgaatcatgtattcattctaaaaaaa
ttcgaaaaatggataagagccgagaagtcttatattatgaaccttcgattctaaaattcaaattcttctacattgaatgtatagctgcagcaataaatttggatcagcctttc
tactccctgcatctacgttgagcaggtatctttaggtaaccgcacaatacctaacctaatttattgataagagtgcttattataaatcaattcttgcaatttttttcaaaaattg
atttttgcatttttaggtgtcaaaataaacaaaacccatcctagtggatttgtgtggtaaggaaaaacgggtaatctattccttaaaaaaaaatcttggagattatgtaatg
cttactctcaaactttttgtttatacagtagtgatattctttgtttccctctttatctttggattcttatctaatgatccaggacgtaatcctgggcgtgacgagtaaaaatccaa
aattttttcttacaaattggatttgtttcatacatttatctacgagaaaatccgggggtcagaattccttccaattcgaaagtcccaaacgatccgagggggcggaaaga
gagggattcgaaccctcggtacaaaaaaattgtacaacggattagcaatccgccgctttagtccactcagccatctctccccgttccaaatcgaaaggtttccgtgat
atgacagaggcaagaaataacgattgcaaaaaatccttcctttttctttcaaaagttcaaaaaaattatattgccaattccattttagttatattctttttcttaatgttaataaa
aaaaagaagaaaattcttctttttctttctaattctaaaattggatattggctaaaagacaatcagatagattttctcttcagcaggcatttccatataggacttgttataata
aaacaagcaggttatagaaaaaaactcttttttttattatttatcaacaaagcaaaaaggggtcttatcaaaccaacccaccccataaaattggaaagaaagataaagta
agtggacctgactccttgaatgaggcctctatccgctattctgatatataaattcgatgtagatgaaattgtataagtggattttttgtatttccttagacttagaccacgca
aggcaagaatttctcgctatttactatttcatattcttgttactagatgttctataggaataagaagaaatcgcaacccctttccgctacacataaaaatggatttcgaaagt
caatttttcttttcaatatctttacttttttcagaatcctatttttgttcttatacccatgcaatagagagcgagtgggaaaagagaggttactttttttcatttttcccttaaaaaa
taggctttcttggaaataggaatcatggaataatcctgaattccaatgtttatttctatagtataagaaaaactaattgaatcaaattcatggatttaccacgacctcggctg
tgaccccatagataaaaatgcaaaatttctatcttcgagaccattgaaaaaaggcattgaacgagaaaaaaatcgtccacagataatctatcgtatgccttggaagtgat
ataaggtgctcggaaatggttgaagtaattgaataggaggatcactatgactatagcccttggtagagttactaaagaagaaaatgatttatttgatattatggacgact
ggttacgaagggaccgttttgtttttgtaggatggtctggcctattgctttttccttgtgcttatttcgctttaggaggttggtttacagggacaacttttgtaacttcttggtat
acccatggattggcgagttcctatttggaaggttgcaatttcttaaccgcagcagtttccacccctgccaatagtttagcacactctttgttgctactatggggcccgga
agcacaaggggattttactcgttggtgtcaattaggtggtctgtggacttttgttgctctccatggggcttttgcactaataggtttcatgttacgtcaatttgaacttgctc
ggtctgttcaattgcggccttataatgcaatttcattctctggcccaatcgctgtttttgtttccgtattcctgatttatccactggggcaatccggttggttctttgcgccga
gttttggcgtagcagcgatatttcgattcatcctcttcttccaaggatttcataattggacgttgaacccatttcatatgatgggagttgccggagtattaggcgcggctct
gctatgcgctattcatggggcaaccgtggaaaacactctatttgaggacggtgatggtgcaaataccttccgcgcttttaacccaactcaagctgaagaaacttattca
atggtcaccgctaatcgcttttggtcccaaatctttggtgttgctttttccaataaacgttggttacatttctttatgctatttgtaccggtcaccggtttatggatgagtgctat
tggcgtagtcggcctggctctgaacctacgtgcctatgacttcgtttcccaggaaatccgtgcagcggaagatcctgaatttgagactttctacaccaaaaatattcttt
taaacgagggtattcgtgcgtggatggcagctcaggatcagcctcatgaaaatcttatattccctgaggaggttctaccacgtggaaacgctctttaatggaacttttgt
tttagctggtcgtgaccaagaaaccaccggttttgcttggtgggccgggaatgccagacttatcaatttgtcgggtaaactacttggagctcacgtagcccatgcagg
attaatcgtattctgggccggagcaatgaacctatttgaagtggcccatttcgtaccagaaaaacccatgtatgaacaagggttgattttacttccgcacttagctactct
aggttggggagtagggcccgggggagaagttctagatacttttccgtactttgtatctggagtacttcatctaatttcctccgcagtcttaggcttcggtggcatttatca
cgcgcttctgggaccggagactcttgaagaatcttttccattctttggttatgtgtggaaagatagaaataaaatgactacaattttgggtattcacctaatttgttaggta
taggtgcttttcttctagtactcaaagctctttattttggcggtatatatgatacctgggctcctgggggaggagatgtaagaaaaattaccaatttgacccttagccccg
gtgttatatttggttatttactaaaatccccttttgggggagagggttggattgttagtgtggatgatttagaagatataattgggggacatgtatggttgggtttcatttgtg
tatttggcggaatttggcatatcttaaccaaacccttcgcatgggctcgccgtgcatttgtatggtctggagaagcttacttgtcttatagtttaggcgctttatctgtctttg
gttttatcgcttgttgttttgtctggttcaataatacagcttatccgagtgagttttatggacctactgggccagaagcttctcaagctcaagcatttacttttctagttagaga
tcagcgtcttggagctaatgtgggatctgcccaaggacccacaggtttaggtaaatatctaatgcgttccccaacgggagaggttatttttggaggggaaactatgcg
tttttgggaccttcgtgctccatggttagaacctctaaggggccccaacggtttggacttgagtaggttgaaaaaagacatacaaccttggcaagaacgacgttcggc
agaatatatgacccatgctcctttaggctctttaaattctgtgggtggcgtagctaccgagatcaatgcagttaattatgtttctcctagaagttggttagcgacttcccatt
ttgttctaggattcttctttttgtgggccatttgtggcatgcaggaagagcccgggctgctgcagcaggatttgaaaagggaatcgatcgtgatttggagcctgttcttt
acatgacccctcttaactaagattttcttatttatacctgttctactgttttttctgttctggctcggttattctatctagccgagccattcattcctttctatgaaagaaagataa
ggggacagaacaaaaaaaacaaataaagaaacaaacgtattcaatacgcaaaagaaaagagagaggaaagtaaaaggagagagagggattcgaaccctcgat
agttcctagaactataccggttttcaagaccggagctatcaaccactcagccatctctccacagcctaatccttattttattcctacaaatagaacatagccatacgaaat
gactcactaacctctagaaacatctcaaatacaaatcccttttcgatatatttctgtatactgtatccatgtatacaggatccgctatatccgcttgtgaaataaagcataaa
accccctcaaccccatatccaaataaaaaaagtggtgagtaataagttttaaagagaagaatcaatggattcatgattaaacccctcctacttcttgtattttattacaattt
tgattaagtgagggatcaaatataatatgtagtcaactttatttgatggtaacttggaggattagaaatatgactattgctttccaattagctgtttttgcattaattgtgactt
cctcagtcttagtcattagtgtaccccttgtatttgcttctcctgatggttggtcaaacaataaaaacgttgtattttccggtacatcattatggattggactggtctttctggt
agctattctaaattctctcatttcttaaatgtgtttagtatttagtagcccgatacaaaataaaaaagggccgtttattcggattgtgagacgcattaaaatgcaatttgcgc
cccgaattgattgacagacaattaaattaataaaaagaaaactcaaatagaaaatgaaacggtcgacccagacatagacggtcgacccaggcggatataccctata
aaatataggacgtagcgagcgtagttcaatggtaaaacatctccttgccaaggagaagatacgggttcgattcccgccgctcgccagcttaatttagtaaggtactat
gataaaaaaatttagtctacttttattaaagtaataggtgttagtctagtaccgtacccctttactatcttagccccctttgcaccccactcaaaaaaagagcactacagcg
gcgggaatcgaactcggcaacagggttccctaaaccggggattcaccgaaacaaacaaccagcaaacagcttttaaagggaagggagatagactgtgcctttctt
tcatttatttttcttttctgcaaggtagggggccttgagagttcctcttgtggtagcaagttacttcgcaacctgctcaatttggccttatagggtcgggaactaatgaata
aaaagggttggataccgcccaaccacccagccctctaccatatctagacaaatagaatagtccttttatacagactgctaagtgcggagacgggaatcgaacccgt
gacctcaaggttatgagcctcgtgagctaccaaactgctctactccgctctggagcgctggaaaccggtggacgaaaaaggttgaatacaatacaggcctctacca
tgtctagacaaatagaatagttattttatacagaatggagcgggtagcgggaatcgaacccgcatcgttagcttggaaggctaggggttatagtcgacgttggttgatt
attattaacgtctctaattcaaaaccgaacatgaaattttgatttcattcggctcctttatgggtattctcaccacttaacatctaagtcagctttttttgtctgaatggaaccaa
ggctctctgctttctagatgatccctaaagagtaggagatagaaatttgcctaaatctatctaatctaattacttcgttccctaatttcatttaagagatcctgaggaaaaga
attgggtttccaccgagctgaaacaatatgctgatggttctagtaaaccaaaactaccgtttttttagctatttggcttccatttcctttttaacaaaagaagatttagttacg

attggaaataaattttttgtatcttcatccatagatcctttactcatattttaaaaattggaatccttaatccaatgcaaaattatgcttcgcgactctgtactcataatccaaat
cctatttgtttttggatgcaatttcaattagtctttgggtacaaatcgcgaaaatgcatattcttcctcaatatgctattgagagaaaaaggagtaaatcctttctaagaact
aaagttttcatcggaatataaaaaaccttaaggacgccttaagtatatcatttcaaattcagttattaatagaacgaatcacacttttaccactaaactatacccgctacat
gtaaattctgataccaacgctaccctttgtcaagggtagccattcgagaaagatgctaattccctcttagttaatgaaacagagaaggttcctcacagttagcagttggt
acttcgatcgcgggcctttcctttactttctttttgttcagaattgaacaaagaatttggggaagaaaacatcttcccccacctatcatgaaatctgggccatagagaaa
gagtgagatgttttttttatttatcatagactttccctatggcttgagagaaacaataaataacttaaagaaaaaggcacataggagccgaaggatttacttgatgtaaa
gaagattctgaatgtctctgcttagtcgattctctccgtttaactttttcttctcttcttttccactcaattctagtttattagattcttgtttaaaagaatcaaagaagatgaatag
aactaagaacacacaaaaaagagcatataggcccgagaccattaccaaaagttcttcccaataatcatattgggtatctgttcccttccttttcctattaggatggggca
tgttggattttccatatccatataccatcgaaccttaagggttccagaaccctcctttttttgctagttttcggaaacggaaaaccctgaaccaggagcagtataaattcta
ctgcccgccttttacaagaaaattggtgataaaactccactacagtttgttcaaaatgcaccaatcagaatcccttgagaatattcaagtaccctatttctaagatatttct
aagagaagaggtacctgctgaaaatcgcttcaacaaatccgcttaagaaaaattgcaaagttttgaactcgaaggtttttccaagggatgcctgttaaaaattgtttcaa
cctctcaccaaaacagacaagaagtatatcactgaaaattaatacccagccatatgggtatatgaagggcgcaaattcgtttataccccacccaattagaggaaataa
aacataaatggagaaagttttcatgataagatcagccaatagaagaaaaaagtccctaatttttcagaccgttctgagcatgtgaaaagtcaatagcctaaagataaaa
aaccctatactttgtgcaagtgataagagagaatatgaaagattttcctatttttcttgattttcttaagattttttgaacctgaccatgaatagacttatatatctcgatatata
cagatataatgtacattatggagtagacctataatgggaaatgaaagtggctaattttggaattgaataagaagcccttttaactcagtggtagagtaatgccatggtaa
ggcataagtcatcggttcaaatccgataaagggctttttttacttagtggtagagtaatgccgcggtaagacgtgagtcagtggttcaaatccgatacagtacttttcta
ctaaattcattcactttctttctttttgaaaatttctctttttttattgaatttgataacttagtgcgagatgcatgcatttttagtttaaacactaagcgaagcagggggggtgtaa
attccaaaaaagaaattggactctttttcctattagatcaatcaaatcactacccgtactgaactaatatagaatcccttttattaatctattcttattccatatcctttataaac
gaatttccctaaaaagtaggggatgatccgtgaattaacctaaccatcaactaaaaaaaaatcctacaaaagcataatggaaaagtaggaaggactccttgctttggat
ctagttatactcttcgagtatattgacaattccaaaaaactgctcatactatcattatagtataatgaggagcggttgtataccgccctatcgtctagtgatgcccctatcgt
ctagtggttcaggacatctctctttcaaggaggcagcggggattcgacttcccctgggggtagggagtattatgaaaggaggttaatcatagattatcaaaaaccta
gaataaattcttcctgggtcgatgcccgagcggttaatggggacggactgtaaattcgttgacaatatgtctacgctggttcaaatccagctcggcccaaaaatctag
ggcttcgtgaatatgagttaaatccatttttttttcttccataaaaaagaatatttgatccatagaaataaaagaaataaaggataaaaagaaaaggggaaatatctttctaa
tctatatctctttcattcctctcttacaaacaaaagaccttttcttattggttattgaaaggtggattattatctatttttagcgataataaatcgcgacatactagttatgtcattc
tcactatacccccatacgatacgggggtatgtagtatatgattcgtctatttcttagagtaggacaggcgaatattcttattcttatggttccatttaagaataagtatgcca
tacgccccgcggggattgtagttcaattggtcagagcaccgccctgtcaaggcggaagctgcgggttcgagccccgtcagtcccgaactagggttcaatgaatgg
agaaattcatctttccttttccatgaaaaaagggggcaggaagcaagatcaaatacctatggggtacccctatttcactttttttagttcgcgtttctcagtaaagaga
ggagagtataggaattttttatcactacttctggttgatagcgaaagacatacatatcatacgtggaagggatcctcctatgttatactattccactctcaaccatgaatt
gatttgatagatccgatattcataatattgaattgattcagtattatcagaatgcaagtcctccccttgaatttacaggataccctttccctctccatgggattacatcccga
gttattgcgaaaaaaaagaggttatggaagtcaatattctcgcatttattgctactgcattgttcattctagttcctactgcctttttacttattatttatgtaaaaacagtcagc
caaaatgattaattggaagttcaattaatcattgaagaaatgaaaaagggattaaataaaataaaaatccaagtcttaaatgaaaggatccggttggaatcataaagtg
tggtagaaaaaactacatatagtttttctaccacactttagagtctttctattatattatcttgaatctacatagaatagattactagattgaaatagtagtctaattcaatttct
tttttcactgcatccacttaatttcaatcaagtcaaaatgaaaaaatccatggagggagagaaaaataatatgagaatagactatagtaaaagaaaaaaagtaaaagg
aaaaaaccagcgaatctttcatgcttaaacatgtcgcgaaatgtttcaaaagagcataaaatttattttaagaactaagaataagaaaagagtataaaacaaatggaaa
atgtgcgatatgttgggaatagctccgcggaagaaaatctaaagttcttatgtatagaactttttttaaccatgggtcgcttctagtagcgattatgaattgctctcaccgct
ctttctatttctattttctattctttctatttctattttctattctttctatttctatttctatttctatatagtagaatagaaatagaaagaatagaaagactctttcttacaagagtttctt
acaggagtgaaacaaaattaaagaaagagagaaagaataaagttcggcaaaatgattaatgcaaaacggtcaattaaagaaaagagttgatacaacaattcgacta
ctcaatcaattagtagtatccctagagtccactcctcccccatactactagtgaaagagaaaatgtaaagactaccattaaagcagcccaagcgagacttactatatcc
atgtaaattatgtctcctatttctatgaaggaattattctactattgatgaataatcatagtagaatcaagggtacagagtcaaaaaggggttctgacctaaggctatggat
gaatcagttcaaagaatttactcttaacaaattcttagagtatttctggtagaattggggagcattaagtataaatatgatacatagccctttcttattaataaaagaataag
gaaacgctatctcatccctattggtatcggtttgggccactactgctaaaacaaaccccagtttgaggaaagaacggtgggttctcaaaatccagtatcgccgagcct
tgttattctcttgccccaacttatgcggggtgcaaatttgtcgatttggatcagtactataagcctaagtattttattgatcaggcggcacccagatttgaactggggataa
aggatttgcagtcccctgccttaccgcttggccatgccgccaaaaaatacgatctaaaatcgagaaaagagcaagtattcatccacgtttcttactaaaactaactttct
tttatcttaaatctaattctacttactttttccaatctttttcaaaaaatctattcatgcttttttggatccagtttcgattattctcctcaaaggattctatcttaaaacacacattg
ctaacactagaaaacttcccttttctttctattgaaatgaaaaaggagaaaagtggatttctagtcacaagctacaaaattaagaacaaattggaaccattaactagaatt
ctctttttattttgaatttcggtattctctcccgcctgccatttaatggcataataaaagacaatggatttatgcctaatccgtatataggtaaactccaggtccgaacagca
ttattatctatggatccccccttatgtacatatctctgtggagaatcgttctttaatttttcattgcattaaatatcttgaataaaaaaaagaaaattgactttgctatgtggaggt
ccagaactagattggcatgtacttaaaaaagtacttactttattttaggattctacaacgaaatcttatatcttatattttatagaaattctactactacgaaacaaaaaagaa
ccttcaaattctttttgaaattaaactaagcgtgctattctcaatcgaactaaagtcaaactttctagtgcttataaattattatattttggttttatcccattcatagaaaggag
aaaaaatgagaaatctttgccgtccaatctaagaatatcataaactgtaagtggcagaattttttcgaggttctaggaatgttttatcacttcattttcattccatttggacc
cctgggaaattcgaactttcgttgaaattgtctctattcatatgtatgaaatacatatatgaaatacgtatgtggagttcccgagaatttcatgtgatttagtaaacagaata
tagattccataattgctagatcgatctgtagggattgatgaagagtgagctgataatggaatttttcttcgataaacaggaaacttaagattaagatgctccggaatgga
aatgagggaatgtccacaatacccggatttagtcagatccaattcgagggattttgtaggttcattaatcaaggcttggcagaagaacttgagaagtttccaacaatta
aagatccagatcacgaaatttcatttcaattatttgcgaaaggatatcaattgctagaaccttcgataaaagaaagggatgctgtgtatgaatcactcacctattcttccg
aattatacgtatccgcgcgattaatttttggtttcgatgtgcaaaagcaaaccatttctattggaaacattcctataatgaattccttaggaacctttataataaatggaatat
accgaattgtgatcaatcaaatattgctaagtcctggtatttactaccgctcggaattagaccataaaggaatttctatctacaccgggactataatatcagattgggga

ggaagatcggaattagcaattgataaaaaagaaaggatatgggctcgcgtgagtagaaaacaaaagatatctattctagttctatcatcagctatgggttcaaatctaa
aagaaattctagataatgtttcctaccctgaaattttcttgtctttccctaatgctaaggagaagaagaggattgagtcaaaagaaaaagctattttggagttttatcaaca
atttgcttgtgtaggtggggacctggtattttcggaatccttatgtgaggaattacaaaagaaattttttcaacaaaaatgtgaattaggaaggattggtcgacgaaatat
gaatcgaagactgaatcttgatatacctcagaacagcaccttcttgttaccgcgagatgtattggccgctacggatcatttgattggaatgaaatttgaaacgggtatac
ttgacgatgacgatatgaatcacttgaaaaataaacgtattcgttcggttgcggatctgttacaagatcaattcggactggctcttggtcgtttacaacatgcggttcaaa
aaactatccgtagagtattcatacgtcaatcaaaaccgactccacaaactttggtaactccaacttcaacctcgattttattaataactacttacgagaccttctttggtac
atatcccttatctcaagtttttgatcaaaccaatccattgacacaaacggttcatgggcgaaaagtgagttgtttgggtcctggaggattgacggggagaactgcaagt
tttcggagccgagatatccatccgagtcactatgggcgtatttgtccaattgacacgtccgaaggaatcaacgttggacttactggatctttagctattcatgcgagaat
tgatcattggtggggatccgtagagagtccattttatgaaatatctgagaaagcaaagaaaaaaaaagagagacaggtggtttatttatcaccaaatagagatgagta
ttatatgatagcagcaggaaattctttgtccttgaatcggggtattcaggaagaacaggttgttccagctagataccgtcaagaattcctgactattgcatgggaacag
attcatgttagaagtatttttcctttccaatatttttctattgggggttctctcattccttttattgagcataatgatgcgaatcgagctttaatgagttctaatatgcagcgccaa
gcagttccgctttctcggtccgagaagtgcattgttggaactggattggaacgccaaacagctctagattcgagggtttctgttatagccgaacgcgagggaaagat
catttctactaatagtcacaagatccttttatcaagtagtgggaagactataagtattcctttagttacccatcggcgctctaacaaaaatacttgtatgcaccaaaaacct
cgggttccgcggggtaaatccattaaaaaaggacaaattttagcggagggggctgctacggttggtggggaacttgctttaggaaaaaacgtattagtagcttatat
gccatgggaaggttacaattttgaagacgcagtattaattagcgaacgtttggtatatgaggatatttatacttcttttcacatccgaaaatatgaaattcagacggatac
aacaagccaaggctccgctgaaaaaatcactaaagaaataccacatctagaagaacatttactccgcaatttggatagaaatggagttgtgaagttggggtcctggg
tagaaacaggcgatattttagtaggtaaattaacgcctcagatagcgagcgaatcgtcctatatcgcggaagctggattattacgggccatatttggtcttgaggtatc
cacttcaaaagaaacttctctcaaactaccgataggtggaagaggacgcgttatcgatgtgaaatggatccagagggatcccctcgacataatggttcgtgtatatat
tttacaaaaacgcgaaatcaaagttggggataaagtagccggaagacacgggaataaggggatcatttccaaaattttgcctaggcaagatatgccctatttgcaag
atggaacgcctgttgatatggttttcaatcccttaggagtaccctcccgaatgaatgtgggacaaatatttgaaagctcgctcggattagcaggggatctgctaaaga
aacattatagaatagcaccctttgatgagagatatgagcaagaggcttcaagaaaacttgtgttttcagaattatatgaagccagtaaacaaacaaaaaatccgtggg
tatttgaacccgagtacccgggaaaaagcagaatatttgatggaagaacaggagacccccttcgaacagcctgttctaatagggaagtcctatatcttaaaattaattc
atcaagttgatgagaaaatccacggacgctctactgggccctattcacttgttacacaacaacccgttagaggaagagccaagcaaggggggacaacgaatagga
gaaatggaagtttgggctttagaaggatttggtgttgctcatattttacaagagatacttacttataaatctgatcatcttatagctcgccaagaaatacttaacgctacgat
ctggggaaaacgagtacctaatcacgaggatcctccagaatcttttcgagtgcttgttcgagaactacgatctttggctctagaactgaaccatttccttgtatctcaga
agaacttccaggttaatagggaagaagtttgatcggaataaatataaattcttttcttatttctattttatgattgaccaatataaacatcaacaacttcaaattggactcgttt
cccctcaacaaataaaggcttgggctaacaaaacactacctaatggggaagtcgttggcgaagtcacaaggccctccacttttcattataaaaccgataaaccagaa
aaagatggattgttttgcgaaagaatctttggacccataaaaagcagaatttgtgcttgtggaaattctcgagcgagcggagctgaaaacgaagacgaaagattttgc
caaaaatgcggagtagaatttgttgattctcggatacgaagatatcaaatgggatacatcaaactcgcatgtcccgtgactcatgtgtggtatttaaaaggtcttcctag
ttatatcgcgaatcttttagataaaccccttaagaaattggagggcctagtatatggcgatttctcttttgctaggcccagtgctaaaaaaccaactttcttacgattacga
ggtttattcgaggatgaaatttcatcctgtaaccatagcatttcccccttttttctaccccaggctttacaacatttcgaaatcgggaaattgcgacaggagcaggtgct
attagagaacaattagcagatttggatttgcgaattattttagagaattcctcggtcgaatggaaggaattagaagacgaggggtatagtggagatgaatgggaagat
agaaaaagacgaataagaaaagttttttgattagacgcatgcaattggcgaaacattttattcaaacaaatgtagaaccagaatggatggttttgtgcttattaccagtt
cttcctcccgaattaagacccattgtttataggtctggggataaagtagtgacttcggatattaatgaactttataagagagttatccgtcggaacaacaatcttgcctat
ctattaaaaagaagtgaattagcgccagcagatttagtaatgtgccaggaaaaattggtacaagaagccgtggatacacttcttgatagtgggtcccgcgggcaacc
aacgagggatggtcacaataaagtatacaaatcactttcagatgtaattgaaggtaaagaggggaggtttcgcgaaactctgcttgggaaacgggtcgattactcgg
ggcgttctgtcattgttgtgggtccttcactttcattacatcaatgtgggttacctctagagatagcaataaagctttttcagctatttgtaattcgcgatttaatcacgaaac
gcgctacttctaatgttaggattgctaaaaggaaaatttgggaaaaggaacccattgtatgggaaatacttcaagaagttatgaggggacatcctgtactgttgaatag
agcacctaccctgcatagattaggcatacaggctttccaacccactttagtagaggggcgtactatttgtttacacccattagtgtgtaaaggtttcaatgcagactttga
tggggatcaaatggctgttcatctacctttatccttggaagctcaggcggaagctcgtttacttatgttttctcatatgaatctcctatctcccgctattggggatcctatttg
cgtaccaacccaagacatgcttatcggactttatgtattaacgattggaaaccgtcgaggtatttgtgcaaatagatataatagttgcggaaactatccaaaccaaaaa
gtaaattacaataataataatcctaagtatacgaaagataaagaatctcttttttctagttcctatgatgcactgggagcttatagacagaaacaaatctgtttagacagtc
ccttgtggctccgatggaaactagatcaacgcgtcattgggttaagagaagttccgattgaagttcaatatgaatctttggggacttatcgtgagatttatgcccactat
ctagtagtgggaaatagaaaaaaggaaatccgttctatatacattcgcaccactcttggtcatatttctttttatagagaaatagaggaagccatacaaggatttagtca
ggcctattcatacactatctaaacaaggaagttagattcggggatgcccctttcgaggggcattccgatttcgctagtatcatcatttttgccgcacgaatccagattga
gattgaggaaaggaagttaactaagttttcgaatcactgactcaggcccattgtcgaatcctactcagcaattgtcgaattatactcagccgaaaaaggggggtacttat
ttatggcggaacgggccaatctggtctttcagaataaagagatagatggaactgctatgaaacgacttattagcagattaatagatcatttcggaatgggatatacatc
ccatatactggatcaaataaaaacgctgggcttccatcaagccactactacatcgatttcattaggaatcgaggatcttttaacaataccctctaagggatggttagtcc
aagatgcggaacaacagagttttcttttggaaaaacactattattatggggctgtacacgcggtagaaaaattacgccaatccgttgaaatctggtatgctacaagtga
atatttgaaacacgaaatgaattcgaattttcggataacagatccttctaatccagtctatctaatgtctttttcaggagctagaggaaatgcatcgcaggtacaccaatt
agtaggtatgcgaggattaatggcggatcctcaaggacaaatgattgatttacctattcaaagcaatttacgcgagggactttctttgacagaatatataatttcctgcta
cggagcccgcaaaggggttgtagatactgctgtacgaacggcggatgctggatatcttacacgtagacttgttgaagtagttcaacatattattgtgcgtagaagaga
ttgtggtactatccaagctatttctgtgagtcctcaaaatgggatgacggaaaaactttttgtacaaacattaattggtcgtgtattagcaaacgatatatatatcggttcac
ggtgcattgccactcgaaatcaagatattggaattgggttagtcaatcgattcataaccacctttcgagcacagccatttcgagcacaaccaatatatattagaacccc
ctttacttgccggagcacatcttggatctgtcaattatgttatggtcggagttccactcatggcgatctggtcgaattgggggaagctgtaggtgttattgcgggtcaat
ctattggggagccagggactcaactaacattaagaacttttcatactggtggggtattcacagggggtactgccgaccttgtacgatccccttcaaatggaaaaatcc

aattcaatggggatttggttcaccccacacgtacccgtcatgggcagcctgcttttctatgttatatagacttgcatataactattcagagtcaggatattctacatagtgt
gactattccttcaaaaagcttgattctagtgcaaaatgatcaatatgtagaatccgaacaagtaattgcggagattcgtgccggaacgtccgctttgcattttaaagaaa
aggtacaaaaacatatttattccgaatcagatggggaaatgcactggagtaccgatgtttatcatgcgcccgaatatcaatatggtaatcttcgtcgattaccaaaaac
aagccatttatggatattgtcagtaagtatgtgcagagctagtatagcttctttttcgctccacaaggatcaagatcaaatgaatacttattccttttctgttgacggaaggt
atatctttggcctctcgatggctgatgatgaggtaagacatagactgttggatacttttggtaaaaaagatagggaaattcttgattattcaacgccggatcgaatcatgt
ccaatggtcattggaattttgtctatccttctattcttcaaaataatttggatttgttagcgaaaaagcgaagaaataggttcgccattccattacagtatcatcaagaacaa
gagaaagaaccaatatcctgttttgggatttcgattgaaataccctttatgggtgttttacgtagaaatactatagttgcttattttgacgacccacgatacaaaaaagata
aaaagggttcaggaattgttaaatttagatataggaccctagaggacgaatataggactcgagagaaagactcagagaacgaatacgggagcccagaaaacgaa
tataggacccgagaggaagaatgtaaaaccctagaagacgaatataggactcgagaggaggagtatgaaaccctagaagatgaatatgggatcccagagaacg
aatatgaaaccctagaagatgaatatggaatcctagaggacgaatataggactcgagaggaggaatccgaggacgaatatgggagtccagagaacaaatatagg
ccccgagaggataaatatggcactttagaggaagactcagaggacgaacatgggactttagaggaagactcagaggaagactcagaggacgaatatgggaacc
cggaggaagattccgtcttaaaaaaggggttttgattgagcatcgaggaacaaaagaatttagtctaaaataccaaaaagaagtagatcggtttttttcattctccaa
gaactgcatatcttgccgagatcctcatccctaaaggtacttgacaatagtattattggagtggatacacaactcacaaaaaatacaagaagtcggctgggtggattg
gttcgagtgaagagaaaaaaaagccatacggaactaaaaatcttttctggagatattcattttcctgaagaggcggataagatattaggtggcagtttgataccactag
aaagagaaaaaaaagattcgaaggaatcaaaaaaaagggaaaattgggtctatgttcagtggaaaaaaattctcaagagcaaggaaaagtattttgttttggttcga
cctgcagtcgcgtatgaaatgaacgaagggagaaatttagcaacacttttcccacaggatctcttgcaggaagagggtaatctccaacttcgacttgtcaattttatttc
tcatgaaaatagcaagttaactcaaagaatttatcatacgaatagtcaattcgttcgaacttgcttagtattgaattgggaacaagaagaaaaagaggaggctcgtgct
tcccttgttgagataagagcaaatggtctgattcgcgatttcctaagaattgggttaatcaaatccactatttcgtatacacgaaaaaggtatgatagcagaagtgcagg
actgattctccataataggttagatcgcaccaataccaattccttttattccaaggcgaagattcaatcacttagccaacatcaagaagctattggtaccttgttgaatcg
aaataaagaataccaatctttgatggttttgtcggcatccaactgttctcgaattggtttttttaagaattcaaaaaatcccaatggggtaaaagaatcgaatcctagaatt
cctattccaaaatttttggggctctttcgaaatttttcgggactcttaggtactattgcacctagtatatcgaatttttcttcatcttactatttactaacgtataatcagatcctg
ttaaaaaagcatttgctccttgacaatttgaaacaaaacttcaaagtacttcaaggacttaaacactctttaataaatgaaaatcaaaggacttcaaatttcgatagtaaca
tcatgttggatccattccagttaaattggcactttctccctcatgattcttgggaagagacatcagcaaaaattcaccttggacaatttatttgcgaaaatgtatgtctattta
aatcgcacataaaaaaatcaggtcaaattttcattgtaaatatagattcctttgttataagagcagctaagccttatttggccactacaggagcaactgttcatggtcatta
tggagaaatcctttacaaaggggataggttagttacgtttatatacgaaaaagcgagatctagtgacataacgcaaggtcttccaaaagtagaacaaatcttcgaagc
gcgttcaattgattcactatcgccgaatctcgaaaggagaattgaggattggaatgagcgtataccaagaattcttgggggtccttggggattcttgattggagctgag
ttaaccatagcccaaagtcgtatctctttggttaataagatccaaaaggtttatcgatcccaaggggtacagatccataatagacatatagagattattatacgccaagt
aacatcaaaagtgcgggtttccgaagatggaatgtctaatgtttttcacctggggaattaattggactattgcgagcagagcgagcagggcgggctttggatgaatc
gatctattatcgggcaatcttattgggaataacaagggtttccctgaatacccaaagtttcatatctgaagcaagttttcaagaaactgctcgagttttagcaaaagctgc
cttacgaggtcgtattgattggttgaaaggcctgaaagaaaacgtagttctgggggggattatacctgttggtaccggattccaaaaatttgtgcaccgttacccacaa
gacaagaacctttatttcgaaattcaaaaaaaaaaactatttgcgtcggaaatgagagatattttgtttctccatacagaattagtttcttctgattctgacgtaacaaacaa
tttctatgagacatcagaatcaccatttaccccatttatatgatttaaggatacataaagcagatttttttactttaaactagatttttgaccttagaacgctaagaggttagatt
ttctatttttattttaatttaaaaagaagtttagttaattcattaaggttatgcttataccatgtagaaggttccatcggaacaattattatttatttcaagctatttcggctctttctt
aatcttcgaaaagaaagaaatttcgtaatggaaaggtaggatgaaaaaaaagaaaaaatcaaaaggaagtgtggaaaaaatgacaagaagatattggaacatcaa
tttgaaagagatgatagaagcgggagttcattttggtcatggtattaagaaatggaatcctaaaatggccccttacatctcggcaaagcgtaaaggtactcatattaca
aatctcgctagaaccacccgtttttttatcagaagcttgtgatttagtttttgatgcagcaagtcagggaaaaagcttcttaattgttggtaccaaaaaaagagcagcgga
tttagtagcatcagctgcaataagggctcgttgtcattatgttaataaaaagtggttcagtggtatgttaacgaattggtcgattactaaaactagactttctcaatttagag
acttaagagcagaagaaaagatggaaaaattccaccatctcccaaaaagagatgtggcaatcttgaagagaaaattatctaccttgcaaagatatctcggcgggatc
aaatatatgacgaggttgcctgacattgtgatcgtcctcgatcagcaaaaagagtatatagctcttcgggaatgtgccattttggggattcctactatttctttagccgat
acaaattgtgacccagatctcgcgaatatatcgattccagccaacgatgacactatgacttcaattcgattgattcttaacaaattagtatttgcaatttgtgagggccgtt
ctctctatataagaaatcattgattaagaagaatagttaattcttgggcaactgcgtagatttatggaatcacttactattctttttttgttttgcatagaaaaaagacgggga
atattgatatatattagagggtattgatatatattatcatctgatgtgatttcttgatatcctaaatataagattaatacttcacgttgctgagttgagaaaaagatggttgaat
caaaagaattctttttttgaagttcatttttttatcaggggacaatatgaatattataccgtgttccattaaaacactcaaggggttatacgatatatcgggcgtagaagtag
gccaacacttctattggcaaataggaggtttccaaattcatgcccaagtacttatcacttcttgggtcgtaattactatcttgctaggttcagttatcatagctgttcgcaat
ccacaaaccattccaaccgatggtcagaatttcttcgaatatgtccttgagtttattcgagacttaagcaaaactcagattggagaagaatacggtccctgggttccctt
tattggaactatgttccttttttatttttgtttcgaactggtcgggtgctcttttaccttggaaaattatacagttaccccatggagaattagcagcgcccacgaatgatataaa
tactactgttgctttagctttactcacgtcagcggcatatttttatgcgggtcttagcaaaaaaggattgagttatttcgagaaatatattaaaccaactccaatccttttacc
aattaacatattagaagatttcacaaaaccattatcgcttagttttcgacttttcgggaatatattggcggatgaattagtcgttgttgttcttgtttctttagtccccttagtag
ttcctataccggtcatgtttcttggattatttacaagcggtattcaagctcttatttttgcaacgttagccgcagcctatataggtgaatccatggagggtcatcattgaatt
gactagttttcaaaatagtcttttttttagcttaactcaattcatgcatggttgcggaaaattcgcttggttggaaaacaaaatagttagaattgcgtatgaatatacaatcta
gagttgtagaagagagaataggctatattacggaattgccaaacaaagtatataggcattagggaggggcggagtcaggctagatctatatcctttatgtctataagtt
cagtcatcttttgtatgggtttccactttaaggaattttttttgaatccgattcaatagaaaatgagaaaatacacaaacaaaatagaagaaacaaattgatatgggatatt
atatattcccaagttagattcattatctaatccgatatatggaatcggattccatatccaattcgatgcagcatattgttatcaattggatatcttgatttaattcctattggatc
tggattaggtcgatttccataggggttcttcctctatttcaccttttattatgaattagatgataggggaaaaaatagaaactcaaggatatcgaagaggaaagaaagaa
ggatggaatgaaagatcagttggttggaaagaaagagaaatagaataatgagtacacaaacctctaatgattagaaactaaaaaggagatctcgaagcagttcgga
gaattcagattatcgtttcaatttgtactttttagttacttctgtccaatagagcttagaaatatgaatttcttggttgattgtatccttaaccatttctttttttgacacgaggaa

ctcatcatgaatccactaattgctgctgcttccgttattgctgctggattggccgtaggtcttgcttctattgggcctggagttggtcaaggtactgctgcaggacaagct
gtagaaggtattgcgagacagccagaagcagaaggtaaaatacgcggtactttattgcttagtctagcttttatggaagctttaacaatttatggactagttgtggcact
ggcgcttttatttgcgaaccctttgtttaatcctaaaaaagaaaacgagtcctttagattagatactttcttctttttttagtaaattggtatttgcttctgcaattccaattatat
caatactttactcctaatttattactcctagagttttctatttatggggacagacaataccccaccccaggaatagctgatttgaggatgatcaatttagaggatatgttcg
ccgtcttgcttcccgccctttgtttagggcagtggaaagtatttttccttttattttaggaattttgggaacatttcaacaaaggaggtctttcacaggtcaaacgagatcta
agacttaatctaaaagaaattactaaattgaatctatttctattaaaaaaaattgcattaaaaaaaccgatcaaaaagggcgagcgaagtaagtgatcgaaaaactttgt
tctttgttcgtcctatctataagaggagagcatatgaaaaatgtaacccattctttcgttttttagctcactggccatccgctgggagtttcgggcttaataccgatattta
gcaacaaatctaataaatctaactgtagtggttggtgtattgatttattttggaaagggagtgtgtgcgagttgtctatttcaagaatagattggatctatccggctgcactt
tagaatattttttagtatttttcgaataaataagaaaagggtgcacgatctcgacgaattacttctgaataaatgcagaaatcatatgaaagaaccatagcatttcgcgac
ctattggtaaatcaaatcaactttgattctctatagaccaataatatgagaccattaacacggttaaagctaaactgcttgaagtctaggcaaaaggggtactctttcta
caactatattagtattagtaccaaatgctttaaacaggaaatagctaatgtagaatttatctgatatagaacactcatatcgataaaatggtttgaactatttactagaagg
ggcaccctgccctttttatccaatgccgaatcgacgacctatgtataaaaaaggagaaagtttttggatttgaagaaaaaagtaggaattctatccattttcattttccatt
tttctatttagtttttcttaatgaaattgaaaattttaactaaagggcaaatacaaataaaaaaacaactttgctgcccacgatagatttttatctaggcggaagagtcctctt
aatatttatctagtcttatatgggtttcggtatattgaaatataaacagaaaagagagggtagaggataggctcattacattaaaaaaaatatggaagtagccatagcaa
aaaaagaaaaaagggagcgtgagagccaaatgaatcgaaagattcatgtttggttcgggaagagatcataaaagttgtaaacttaatagcaaggtaatctactttcat
taaaagatttattagataatcgaaaacagaggatcttgagtactattcgaaattcggaagaattgcgtagaggaaccattgagcagctcgaaaaagctcgaattcgatt
acagaaagtcgaactagaagcggatgagtatcgaatgaatggatactctgagatagaacgagaaaaagcaaatttgattaatgccacttctattagtttggaacaatt
agaaaagtctaaaaacgaaaccctttattttgaaaaacaaagggcgatgaatcaggtccgacaacgggttttccaacaagccgtacaaggagctctaggaactctg
aatagttgtttgaataccgagttacatttccgtacgattcgtgctaatattagcattctcggggccatggaatggaagagttaaattaattaggccttgaacttctactttcc
tttagaatttaggcattatttttccccttgcttccgaaaaaaaaatagtaaagaaacactaatggcaacccttcgagtcgacgaaattcataaaattctccgcgaacgtat
tgaacaatataatagaaaagtagggattgagaatatcggtcgcgtagttcaagtgggggatgggattgctcgtattataggtcttggtgaaataatgtcaggcgaatta
gtcgaatttgcagaagggactaggggtattgctctgaatttggaatccaaaaatgttgggattgtattaatgggcgatgggttgatgatacaagagggcagttttgtaa
aagcaacaggaagaattgctcagatacccgtgagcgaggcttacttgggtcgtgttataaatgctctggctaaacctattgatgggagaggcgaaattgtagcttcg
gaatctcgcttaattgaatctcctgctccgggtataatttccaggcgttctgtatatgaaccccttcaaacggggcttattgctatcgattcgatgattcctattgggcgcg
gtcagcgagagttaattattggggacagacaaaccggcaaaacagcagtagctacagatacaattctcaatcaaaaagggcaagatgtaatatgtgtttatgtagct
atcggtcaaagagcatcctccgtagctcaagtagtaactactttccatgaagagggggccatggaatacactattgtagtagctgaaatggcggattcccctgctaca
ttacaatacctcgctccttatacgggagcagccctggctgagtattttatgtaccgcgaacggcatactttaataatttatgatgatctctccaaacaggcacaagcttat
cgccaaatgtcccttctattaagaagacccccggccgcgaagcttacccaggggatgtttttatttgcattcacgccttttagaaagagccgctaaattaaattctctt
ttaggggaaggaagtatgactgctttaccaatagttgagactcaatctggagacgtttccgcctatattcctactaatgtaatctccattacagatggacaaatattcttat
ccgcagatctattcaatgccggaattcggcctgctattaatgtgggtatttccgtttccagagtaggatccgcggctcaaattaaagccatgaaacaagtagctggca
aatcaaaattggaattagctcaattcgcagagttacaagcctttgcacaattcgcctctgctctcgataaaacaagtcagaatcaattggcaaggggccgacgattac
gagaattgcttaaacaatcccaagcaaatcctcttccagtggaagagcagatagctactatttatatcggaacaagaggatatcttgattccttagaaattggacaggt
aaagaaatttctggatgagttacgtaaacacctaaaagatactaaacctcaattccaagaaattatatcttctagcaagacattcaccgaggaagcggaaatccttttga
aggaagctattcaggaacaactcgaacggttttcccttcaggaacaaacataaattttgcacgtctactcttgttagtagaagtagtagaggagaaatcgttgagaaa
gatttttcatttgaatcatgcaaaaaagttttcttagtttttagtatagttatttaaagaatagatagaaataagattgcgtccaataggatttgaacctataccaaaggtttag
aagacctctgtcctatccattagacaatggacgctttttcatattttcttctttcttttcatttttatcggaccaagagaaaaaactgttagaccaaaactcttttaggaaag
aaaaacaaatgcatatacaaatgtatgatgcatatatcataaagaaggaatatatatggagcgggtagtgggaatcgaacccgcaaccccacggttatgagccttgt
cagctaccaaactgttctatcctgttaaactaaagagaggggaactagtggataaaaggggggttgaatacgcccctctaccatatctatacaaatagaatagtccat
ttatacagaatggtaaagagggctcttctacgatcatcaattccagaaatccatacaaatacgaaagggtattttatccttaccaactggatcttgttgcacccggtaac
aaacatgcataaaccatttctcgaagtatgtgtccggatagcccaaagtctcgatagttagctctaggtcttccggtcaaaaaacaacgtcgatgaaggcgtgtcggt
gcactattacgtggcagggattgcaatttttctcgcattttcgtttttcactcaaactcaaagggtaaactttgcttcttatcttttttttgaagatcgacgaatcaaatgata
tttctgttctaatttctgccgcttcctctctctctgaatcaaactttttttgccataatgtgccgttcctactatctaccaagtatacggttctaatcctagatggaaaaataaat
agaaaaagaaatctaagaaggcggatcctccccctccatcaagagtaatgaactaggtgctgatacagtacaacaaaattaactaaattaaccaaacttgcctgatgt
tgaggcaatcaagaaagcggcataagtgaatatataacccacggaaaagtgagctaatcccaccaatcttgcttgcacaatggaaagagccacgggcttatctctc
cagcgaattaaattagccaaaggtgtccgttcatgagcccatgctaaagtctcaattaattcctgccaatatccacgccaggaaattaagaacataaatccagtcgcc
caaacaagatgtccaaataagaacatccacgcccataccgataaactattcatcccaaaaggattatatccattgataagttgtgaagagtttaaccataggtaatctct
taaccatcccatcaaataagtggaggattcattaaattgtgaaacgttgccctgccataatgtgatgtgtttccaatgccaataaaaagtaacccatccaatggtatttaa
catccagaaaactgccaaataaaacgcgtcccaagcagaaatatcacaagtaccgccgcgaccagggccgtcgcaaggaaaactatatccaaaatctttttttatcc
ggcattaatttggaaccgcgtgcatctaaagcaccctttactaaaatcaatgtagttgtatgcaaacctaaagcaatagcatgatgaaccaagaaatctccaggtccta
ttgttaagaaaagcgaattactattctcgttaacagcattcaaccatccgggtaaccatagggttcgacccgcattgaaagcgggaccgctcgttgaagataagagta
tatcgaacccatatgtcgtcttaccatgagccgattgtatccattgagcaaatataggttcgatcaagatttgcttttctggagtaccaaaagcaagcatgacgtcattat
gaacataaaggcccaaggtatggaatcctaggaagagactagcccaacttaaatgagatatgatagcttctttatggtctaacattcttgccaatacattatcctcattct
gttccggattgtaatctctaatgaaaaaaatagctccatgagcaaaagcccctgtcatgatgaaccctgcaatgtattggtgatgagtatataaagctgcttgagtagta
aagtcttgtgctatgaatgcataagaaggtaaagagtacatatgttgagctactaaggaagtaataacccctaaagaagctagagcaagacctaattgaaaatgaatc
gaattattgattgtgtcgtaaaggcccttatgcccacgccctaatcgacccccggaggagtatgtgcttctaaaagatctttgatactgtgcccaattccgaagttagt
tcgatacatatgaccggcaatgagaaaaataaatgcaatagctaaatgatgatgagcaatatcggtcagccacaaactttgtgtttgtggatggaatcccccaagaa

gagttagaatagcagttcccgctccttgagtggtaccaaataaatgattactcgaatcggggttttgggcataaagattccactgacccgtcagaaggggtcccaac
ccctgtggatagggtaatacatctaagaaattattccatcgaacgtactctcccctggatgctggaatagcgacatgaactaaatgtcctgtccaagccaaagaactta
ccccgaaaagtcctgacaaatgatgattgagacgagattccgcgtttttgaaccacgaaaggcttggtttccatttgggttgtagatgtaaccaacccccctattaagga
tagggtagaaagaaataatagaaaaagagctcctgtataaagatcttcattggtccgtaatccaattgtataccaccactgataaaccccagaataggcgatattcact
ggaccggcggcgcctcctcgagtaaaggcttccacagcgggttgaccaaaatgaggatcccaaatcgcatgagcaataggtcttacgtgtaaaggatcctgtatc
catgattcaaaatttccttgccaagcgacatgaaacagatttccggacgtccatagaaagattattgctaattgcccaaagtgagaagcaaaaatgttctgataaagac
gttcctcagtaatatcatcatgactttcgaaatcatgtgcggtagcaataccaaaccaaatacgacgagtagtggggtcctgagctaagccttggctaaacctgggaa
atcttaattccataatgcctttcaaatcctcctagccactatcctactgcaataattctcgctaagaagaatgcccatgttgtggcaattccacccagaaggtaatgggtt
actcctacagcacgtccttgtataatgctcaaggctctaggctgagtagcaggagcaactttttaatttgttatgagcccaaacgatagattcaatcagttcttgccaata
accacggccgctgaataaaaacattaaactgaaggcccagacaaaatgagcacctaagaaaaaaagaccatatgcggataatgaagaaccataagactgaatta
cttgggatgcctgtgcccacaagaaatctcggagccacccattaatcgtaatggaactctgtgcaaagtttccccctgtgatatgagttaccacccccttgatcacttata
gtaccccaaacatccgactgcattttccaactgaaatggaaaatgactaccgaaatggaattgtacatccagaatagacctaagaaaacatgatcccaggcggatac
ttgacatgttccccctcgcccaggcccatcgcaagggaagcgaaaaccaagatttgctttatcgggtatcaaacgggaactgcgagcaaataaaacacctttcaaa
agtattaatacagtcacatggattgtaaatgcgtgaatgtgatggactaaaaaatctgcggttcctaatggaataggtaacaaagcaactttgccgcctactgctacta
actcgccacctccccacgttaagctggtacttgttgttgcaccaggagctgttacgctaggcgcgccagcatggagattttgtacccattgagcaaagatcggttgta
attgtatggcggtatccgaaaacatatcttggggacgacctaaagcactcatggtatcattatgaatgtataaaccaaaactgtgaaaacctagaaatatacatacca
gttaaggtgggatatgattgcatcacggtgtctaaggacacgatctaatagatcgttgtatcgagtcgttggatcatagtctcttaccataaaaatggctgcatgtgcag
cagcaccaactattagaaatccgccaatccacatgtggtgtgtgaacaaggaaagttgtgtaccatagtcagtagctaggtatggatagggggggcatagagtacat
atgatgagctacaacaatggttgtagagcctagcatagctaggttaagagataattgagcatgccatgacgttgttaggatttcatagagacctttatggccctgtcctg
taaatgggcctttatgagcctccaaaatatctttcaggccatgaccaataccccagttggtcctatacatatgacctgcgatcaggaaaagaatagcaatagctaaatg
atggtgcgcaatatcgctcaaccatagaccgccggttattggatctagccctccgcgaaaactaagaaattctgcgtatttggaccaattcaaggtgaaaaaagggg
ttgctccttcggcaaaactaggataaagttgagccaaaaggtcgcgattcaagataaattcatgaggaagtggtatctccttaggatcaaccccagcgtcaagaaatt
ggttaatcggtaaagatacgtggatttggtgccccgcccaagaaagagacccaagtcctaataaccctgctaagtgatgattcaacatggattctacgtcttggaacc
aggctaatttgggagcggctttgtgataatggaaccacccagcaaaaagcattaacgatgcaaaaatcaatgcaccgattgcggtacaatagagttgtaattcacta
gttattccggatgctcgccaaatctgaaaaaacccggaggttatttggattcctcggaaaccccccgcctacatcaccattcaatatttcttgccctacgattggccaaac
tacctgagcactgggtccaatgtgagtaggatcacttagccatgcttcataattggaaaaacgggcaccatggaagtacatgccactcaaccaaagaaagataatg
gagagttgaccgaaatgagcactaaagacttttcgagagatctcctccaaatcacctgtatgactatcgaaatcgtgagcatcagcatgtaggttccagatccaagtg
gtagtatcagggcccttagctattgttcttgagaaatggccgggtctggcccattcctcaaaagatgtttttacaggatccctatccacaacaatttttacttctggttccg
gcgaacgaatcatcattaagtcctcctctttccggacaagacatacaaagagacccgccaactttttagtgaacctttgaaagatagatattatgattagtccttttctta
ctatctaccgtccttctattttttttttagttattcactggagcaattatatattgaagtcaatctgaggcaagtgttcggatctattatgacataaggattgggtgcctaacgg
acttttttatcttggatttctccacgtaacaaaaaaaccttttttaatttaaaaagctagtctattttttttaagagtataagtccctatctatatctactttccttgagcataatat
agattttttattcgattccaaattccaagataactcattagaattattaataagatggtcctgatatattagcaatatttatattgccccttttttattcgctttattacttctattcta
gaccctatcgtttatccttatgaaatataatataaaatagaaggcagaggaaagagatataatgaaattcttgattcgatcttccgacctaatttatttgattaatggatcaa
caaccaaacccccatttctgaaaaaggagagtggtcttattcaaattcaaagcgcttcgtaatcttcaaccagttctgtgcttcaatataatttcccggagtaagcgcta
tagcttgtttccaatactcagcagcttgatcaaaccaagcttctgcaatttctgaatcaccctgtagaatggcctgttctccccggtcggaataggtagttccttcccta
gaaccgtacttgagagtttcctacctcatacggctcagaaattgctatcttaatttcccttgtcttaactgaattcgatttctcaaaaatcgatccaatttcttcttgggttacg
cagaagaagttaattacctaagtttcaaaccctaaattttgatcaataatcagtttgatcttttctcccacctgcagaaaaatgaagcatagatagacctatatccttcgtc
cgaattttctgaaaggtaactatctcggtttcatatatgaaatttctatagaatccttgaaaaagactttttccccataagcaagaaaaaagaacttactatctttgggatct
gatactacaccgctgcttaatcccttagtggatcggctctattacataagcggattcctaaattttgccccatatcatgggataagtaagcagtttttttttagttgtatcgac
ccagtcggtcactaattgatctttacggtgctttctctatcaatttgagaactctatccatagagtagtataggccatactttctttcttcctattttgattctcgtgaagtgtcc
tttcttcctacagctgatagggcaaaatcgttgttttgacgatccctatgtagaaagccccctttctagtaaatactagaaaattgatcctttcttatttttcttctatagtg
gagatagtcgcacgtaatgacagatcacggccatattattaaaagcttgtggtaagaaggggtttcgttctagtgcccggaaataatattccaaagcctttgtatgctct
ccattgcttgtgtgtataaggcctatgttatagagtatataacttcgatcatagggatcaatttctagtcgcgtagcttcataataattttgcaaagcttccgcataatttcctt
cggattgagccaacatccgttacggtcgttcgttctattcaaaaaatctccgttccaaaaccgtacatgaggttttcacctcatacggctcctcccttctgtacatagtact
aagcgaaaaaaatctagagaataaaaatagaattagttccatctcattatggaccgaaaggggctggtatttttccaagaaatctctagccaaccttcccacaagaggtt
tttcttaacaccaatgaattctattaatgctagaggaaaacgatagctccaagaatttcttgttctcaacgcctcctatttagaggaattagccacttcaacgatctttgat
ggttataggggtatccaaagtacaaacttgatggttgtttgttatcccaaccattcttcccagccctgataccaatcaggaaagggttaatttctaacaaagtttttctcttg
ttgattcctatttctaggtgtagtgcttttatccccctatgctacctattagtactagtagagtaggattagcctgtaatacagaacctatcctgtaggtgtaacctttcgctca
atactaaaatctacaattgaagcatccgaggccgcgtcagtcgaggatacacgacagaaggagttgttagttcacctcaccttccctaagcgtgggtttcccttacta
atttggtttctctccgcgaacccccgctctttctcgtaaaaccggggtgtaggtagggctaaaaaaacaaaaaaaagagtcaaatcgcaccatctctataataagtaa
atgccctttttccccggaggttgtcggaattattcgcaataaaatattggctacaattgagaaggtcttatcaatgaaatttccatttatacgggatctaggcataattccc
aacccattctatcattctatatagaattcttttcattccttcacaaaataacataaaaaaaaaaacaaatccattcaattcttataaatcgatccctatgctccaaatggataag
agaggtatttctgctcagcccaaattctctctttttccttctgtttgaacaagaagagatagaaaatatttgactaagattggatttcattccactttcctatttcttttctcaac
aacaacttctctcatcaactatttcgcattttcaaagtcattaatcgtcccataccctattttctatttgattgtatggggtaggataccttatgcaaacagaattctagggttc
cttttttatcgaataagaagaattcttccattctctttttctttggtttggggaaaacccaaactaaaactttttcgagggagcggaattcctagtaaaaaaatcctggaccc
gtccacttagatgaaaaggaatttttctatctaatagaacaatggaactctcgcgcggttgtggttgtacctgtactgcaggaataggaaaactcgctattcactcagttt

tttttccataataagattatggaggagagatggccgagcggttcaaggcgtagcattggaactgctatgtagacttttgtttaccgagggttcgaatccctctctttccgt
ttttcttaattcagcaacgttaatgattacaatgtatcaaatcaaatgacaatttattccagcaataatacaatacctttatttaatagaaattctctataccaaattactatggt
atgtaaaatacacatagaggaaagaacaaaaaacaaaaaggaatcctagggttaatccatttctgttaggtgaatgggaaaatacgaattaagagccttaggtcgatt
tagttcggggaaaggggaaggggaagaaaattctatgaacctttccgtttttccttaagttcaagtttgacgagagtaatattctacaactaacaactcatttattttgaga
ccgacccacttcctatccaggatttttttactagtcctttatattgcaatgtgtcaatcgtcaaatgctttggcaatttccccgggtcggatgaagcaatagaattttgaac
cagacgttttgatctttggttatccttcgtagtaataatatctcggggtttgcaacgaaaacttggtatatcgactatacgaccattaactaaaatatgtctatggttaactaa
ttgccgggcctcgggaatggttgaagccatacctaatcgaaaaaggatattatccaaacgcatttcaagtaattgtagtaaaacctgacctgttgaactttttgcttttcc
agcgatatgtacatatctaagtaattgtcgttctgtcagaccataatgaaaacgcaatttctgtttttcttgaagacgaatacgatattgttctttttcccagaatggaatttc
tttttcagattacttccggatttaggtgtttttctagtgagtcctggtaaagctcccagacggcgtattttttaaaacgaggtcctcgataacgggacatgaagactccttt
ttttattgaaatttcattttacacaattaatttcattgtatttacattacagaatacatcgaaattaaaactgaattaaagtaaaggataaacagagtaaaatctactaaaagta
ccacaaaaaaatggaatttcatcaacatctgaatttttgtatatatattatttattttattgttttgtatctagcaaaattgtagggtagaacgacataatagatcctggcttctc
catttaattcggagaaaaagaggtatttttgttcatggaacattgatagagaaaaaagccgactatcggatttgaaccgatgaccctcgcattacaaatgcgatgctct
aacctctgagctaagtgggcttacataacagaaatagtgtaacaaatagaaatatgtatagtataggaaatccgtaaaatgtcagatcttaattattaatcttagctattaa
ctagttcgaaattggaagttctacttagaaaaaaatactagaacttcataaaataaagttagatagattttttgaactttctttttctctaattcgcaaatctatttttctaata
gaatctattccaatttctatattgaatttgattttagatatttttaatttgatatggctcggacgaataatctaatacatggataaagaataaataatatatatacgaaaacata
ataaagagaacatgcgaatttcttgtattttcagtccatcattatagacatttttgagatattttgttttttttgttatttgctaataatttaatgattaatatttcactaaggagaa
catagaatcatagcaaatgaaattgctaattctgattagcaaaaaaaaagaatgaatatcaagcgttatagtatgattttgaatactctaaaaaaggaacgcggtaggt
gggggagagaaaaaccttgggatatattgattcgcattgaattgcaaatacatcaacgatagaatcaattcaatgctgaattgcaataagcggagtctctcaactaga
gacgaaccgctagactacatagagtaatgaattcaacgattcaaaaaaaactaacagatggaggaaattgcacaaggaatcctggtctcaaagaaaaagaaaatg
gggatatggcgaaatcggtagacgctacggacttgattgtattgagccttggtatggaaacctgctaagtggcaacttccaaattcagagaaaccctggaattaaaa
aaggggcaatcctgagccaaatccatgttttgagaaaacaagcggttctcgaactagaacccaaaggaaaaggataggtgcagagactcaatggaagctgttcta
acgaatcgagttaattacgttgtgttgctagcggaactcccttctaaattagggaaagaagggctttcgaaatctaatacacacgtatagatactggcatagcaaacga
ttaatcacagaactcatatcataatataggttctttaattctttttaaaatgaaaataggaatgattatgaaatagaaaattcataattttttagaattgttgtgaatccattcc
aatcgaatattgagtaatcaaatccttcaattcatagttttcgaaatctttttaaaagcggattaatcggacgaggataaagagagagtcccattctacatgtcaatactg
acaacaatgaaatttctagtaaaaggaaaatccgtcgactttctaagtcgtgagggttcaagtccctctatccccaaaccctcttttattccctaactctagtatttatcctg
tttttttattaataggtttaagattcaatggaatacatttctttttttattatagtatcggcaaggaatgtcgattattaactcgatatttaaatattattaaataggctttctttgtac
aatgcataggactgccccctccccatttccaaattttggatattgacatagatacaaatactctactaggatgatgcacaagaaaaggtcaggatagctcagttggtag
agcagaggactgaaaatcctcgtgtcaccagttcaaatctggttcctggcacagaaaaaagggatcttccgaataggtattgatacaaatacctcgagatgggttgg
gatacatattcgttaataatatagatagagtatgatttttttcatctaagtagataaatctctaaatagaggcacttctttttctgcatttttgaatttcttaattttctattccgctatt
ccgacaaatatttttttattcttgcttatcttatcttactttcctagttgttctaagtaatgcacgcggtacaaagttcgtggtagggaacttctttgagtcatcatatttttctgtt
catacgaaggaaatgaatatgtgattttccaacgaaatgaagcccttttttgcttagtctatctgaaccttttgtataattggaattaattagaatgtaataggtattctgtttc
atctaggaacagagcgtaaaaatattccttgacttgcataaaatctggagttgtgttgtataagtgagcatgaatttcttatcattcaatgagcatcttgtatttcatagaaa
ttgggggttatatagtccttacgtaagggccagcctatccaactttcaggcattaggatacgtttaaggcgtggatgattatcataagagattcccaccatatcaaaaga
ttcgcgttcttgaaaatcggaacttctccaaatccagaagacagatgggattctaggattatccttttgggcaaagacttttatgcatacttcttctgggttatctataccat
actgtattctcgtaagatgatacacgctagctaaagatccaccgggtgcaacgtcataagcacattgggaacgtaaataattgtaaccatatacatataaaatgacag
caatggaatcccaatcctctgcttttatttgcaaagtctctattcctcgatgatcgaagcccaaagatctatgaaccacctcatgtttgactagccaattagataaccaac
cctgctgcattatcttgatctctcctcctttgtataaatatttcgcagttcgaatgcaagtttgaaatattgccctgctctttctttttcggcacaaagagctcctcctaattcac
taatttgtaggaagatactggacttttggatttgaaaaaagtttcagaagatatgtctaaagtagatggtgattgatagagcaattcttgctcataagttccagtattagtac
tgcgccgaacataaagcttgtggctggtagtaaaacatcgatttttcttttgagatagagttcgatcctcaactatttctcgcgatatcttcttacgaagttttgttagggca
tctataacagcctctggtttaggtgggcaacccggcaagtagacgtccacaggaattaacttatcaactccccgaacagtactataggaatccgtactgaacattcca
ccagtaatagtacaagctcccatagcaatgacgtattttggttcaggcatttgctcatataatctcactaaagagggagccattttcattgttactgtaccggctgttaaaa
ttaggtccgcttgcctaggacttgatcttggtaccaatccataacgatcaaagtcgaatcgtgagcctattaatgaagcaaattcaatgaaacaacaactggtaccata
tagaaggggccataaactagagagtcttgaccaattcgaaagatcttttagtgtagttgaaataacggaattggaacttgtttggtcaagtaggggaaactcaatcaaa
ctcataactgtcttaatggaatcttttccttctttttttttttgtctgaatattcagttaagaccattccaaggctccttttcgccatgcataaactaaaccaacaactaggata
agcacgaaaatgaaagcttcgataaaaacggatatacccaatacgtcgaaactcattgcccaagggtagagaaagaccgtttccacatcaaaaacaacaaaaact
agcgcaaacatgtaatagcgtattcggaattgtaaccaagccccccccatgggttctatacccgattcataactagaaagcttctctggtccttcacgaaccggagct
aaaagtgctgaaatccaaaatgctaaaataggaataaggcttgctattattagaaatgcccaaaaaatatcatattcgtgaagcagaaacataaatgtactcccattaat
gtggaataggcggaactgaattagtcaattcaagtcagcattgtcaatttatacagaatttctctcttttcctcggtgaaacaaggatcggtttttctcaaaccaaagggc
ttagtttagcctttgtttcctctttgccacgtcttctttaaagattcatccaatggaatcccgactccctttctttttgatttcctttctatttaggtatggtggagacataattctta
tagaaacaaaactctctcgcttcactttgtctcattttctctagaatctctagaaaaaggaataaaaacgaaaatactacgaattagagcctaattaagataggatgacta
atgtatgcagcctaatgaggagtaattctataaaaataaagaactctatttcagaacgtagatcgatttagatttaggtaatctatagatatagataagcaaagtaatata
cttcaaacaaagtaggaattcgcaagatggagaacatcttgcagttgatttgatagaaattcatttttcttttcctgtctctataattttcgatgaatgagcctctggtaatcc
ttttatctctattttatggcgcaggcgcctgtccagtctataaacaagtactaatagggaaatgaaaactatactaaaggaaacgtaggatatctctcctaaaatctaaaa
aaggacatattagggctatacggattcgaaccgtagaccttctcggtaaaacagatcaaacggattattatcgaaatgattcgaactgtttcaaagacccaacatgcat
ttttttgcattgggctcttttatcaactgatagaaagatcagttagtccaccatagtttttctttacggaaagataatgagatggctccctgcgctctgattgattatttgtatta
tgatctatctaagagcaataccaaagtgtttcaaaggaggattaccttgacttaggtctgcctccggcctaaattaaatcaacctaagtgaaatagagtctctatcgttcc

gctacaagagttgactatgagacttcatacaccttaaagttcatagaacgaaaagaattttttggaggcccttatcctcattaagcctagcatttagtgggctggatattt
accttatcaactagcaaatcaataaaggttctatttgtttaggcacctggattggtacctgaatcggactgaaccaactatttgtcaggcgactgttctcctattctctcga
atccatgaagtaagacattgattttgcaagaagatccactatgttcattgcataataagctcccttgaaaagcattggcgcacgtgtaaacgagttgctctaccgaactg
agctatagcccttgtcagagatatcttaatatatagagaatttcttgtcaagatgaatattctctaatagtagaggatatcctttgatctgtttactataataacataccaata
acgaagcggtatttgcttataaaaaggattcgatctataatcgatcgaagtaaagggtcttcctttgtggtgataaattgcctacttaactcagtggttagagtattgctttc
atacggcgggagtcattggttcaaatccaatagtaggtaggtaggtagaaaaattactagatagcattggccctacttcgcttcgctatctaataattttttctacccctct
tcccttttttctttgtatcaactaaaccgttgggttgtcttcaattagatgggggaatccaattaacagcctcgactcgtatcctagctcgtctgagagctaccttcgcttcaa
ccaattctttcgtaccctcagctctactcacgttagcttcggctatttcaagtgcctgttgagcttcttccggatcaatgtcactacccagttccgcatcatttcctaaaatg
atgatctcattattaactattctggcaaaaccgctccacagaaccgccgttaaccattgatcgttgaggaggcgtattctcaagggacccatatctacagctgtgttaat
gggggcgtggtttggtaatacgccaatttggccactattagtagataaaatgatttctttcacttcacaatcccaaataattcgcttaggagtcagtacataaagatttaat
ttcatttcttcaatttgttctcctcttctaagtttatagctttcgtgctagcttcatcgatgttacccaccaaataaaaagcctgttcgggtaggccgtctaattctccggaaa
ggattagttgaaatcccctaatagtttctgcaagaccaacatactttcccggagaaccggtaaaaacttctgccacaaaaaacggttgtgataagaagcgctcaatttt
tcgtgctcttgctacagttaaacgatcctcctccgataattcatccaacccaagaattgcgataatgtcctgaagttctttgtaacgttgtaaagtttgcttaactctttgcg
cagtttcataatgttcgttgccaacgatccgaggttgtaacatagttgaggttgaatctaaaggatctactgcaggataaatccctttggaagctaatcctctggaaagt
acggtagtagcatccaaatgtgcaaatgttgtagcaggagcagggtcggtcaaatcgtccgcaggtacataaaccgcttggatcgaagttatagatcccttttagta
gaagtaattctttcttgcaaagaacccatttctgtactaagagtaggttgataacccactgcagagggcattctccctaataaggcagatacctccgatcctgcttgaac
aaaacgaaagatattatcgatgaatagaagcacgtcttgcttattaacatctcggaaatattctgccatagttagggcagtcaaaccaactctcatacgagctcctggc
ggttcattcatttggccatagactagagctacctttgattcctcaagattttttcattaattactccagattccttcatttccatataaagatcatttccttcacgagtccgttcc
cctactccgccaaatacggatacgcccccgtgagctttagcaatattgttgattaattccatgatgagtactgttttacctactccagctcccccaaatagtccgatttttc
ctccacgccgataaggagctaaaagatcgaccaccttaataccagtttcaaagatggataatttcgtatctaactcgataaaggcgggcgcggatctatgaataggg
aatgttgcactagtatctacaggacccaaattgtcaacaggctccccaagaacgttgaaaattcgtccaagagtagctccaccgacaggaacactgagaggagctc
ccgtgtcaatcacttccattcctctcatcaacccatctgtagcactcatagctacagctctaactcgattatttcctaataattgttgtacctcacaagttacattaatttgctt
accgtcagtgtctcgactcttgactaccaaagcattataaatataaggtaacttgcccgggggaaaagtgacatccagcacgggtccaataatttgatcgatacgccc
tgtactttttttcttcaattgtagaaaccccgggacgagaagtagtaggattggttctcataattatcacataattttcaaaaaaaaggaatttatcgaaattttgatttttttctt
gttgaataatgccaaatcaacaccaaaaaaaatatccaaaaatccaaaagtcaaaaggaaatgaattagttaattcaataagagagaaaaggggaccagcacttgatt
tcgttgcccaaacgaatcccattcaatcgtttactcatggaatgagtccgtcggaaagttcaatcaatctttttttcatatacattttgccttttgtaaacgatttgtgcctact
ctactttcttatctaggacttcgatatacaaaatatatactactgtgaagcatagattgctgtcaacagagaattttcgtagtatttaggtatttccactcaaaataagaaaa
gggggtctattaagaacttaataaggattagaagttgatttggggttgcgctatatctattaaagagtatacaataaagatggatttggtgaatcaaatccatggtttaata
acgaagcatgttaacttaccataacaacaactcaattcttatcgaattcctatagtagaattcctatagcatagaatgtacacagggtgtacccattatatatgaatgaaa
catattatatgaatgaaacatattcattaacttaagcatgcccccccattttctttaatgagttgatattaattgaatatcttttttttaagatttttgcaaaggtttcatttacgccta
atccatatcgagtagaccctgtcgttgtgagaattcttaattcatgagttgtagggagggacgtatgtcaccacaaacagaaactaaagcaagtgttggatttaaagct
ggtgttaaggattataaattgacttactacaccccggagtacgaaaccaaggacactgatatcttggcagcattccgagtaactcctcagccgggggttccgcccga
agaagcaggggctgcagtagctgccgaatcttctactggtacatggacaactgtttggactgatggacttaccagtcttgatcgttacaaaggccgatgctatcacat
cgagcccgttgttggggaggataatcaatatatcgcttatgtagcttatccattagacctatttgaagagggttctgttactaacatgtttacttccattgtgggtaacgtat
ttggtttcaaagccctacgcgctctacgtctggaggatctgcgaattcccctacttattcaaaaactttccaaggtccgcctcatggtatccaagttgaaagggataa
gttgaacaaatacggtcgtcctttattgggatgtactattaaaccaaaattgggattatctgcaaaaaattatggtagagcatgttatgagtgtctacgcggtggacttga
ttttaccaaagatgatgaaaacgtaaactcacaaccatttatgcgttggagggaccgttttgtcttttgtgccgaagctatttataaatcacaggccgaaaccggtgaaa
ttaaggggcattacttgaatgcgactgcaggtacatgcgaagaaatgattaaaagagctgtatttgcgagggaattaggggttcctattgtaatgcatgactacttaac
cgggggattcaccgcaaatactagtttggctcattattgccgcgacaacggcctacttcttcacattcaccgagcaatgcatgcagttattgatagacagaaaaatcat
ggtatgcatttccgtgtattagctaaagcattgcgtatgtctgggggagatcatatccacgctggtacagtagtaggtaagttagaaggggaacgcgaaatgacttta
ggttttgttgatttattgcgcgatgattttattgaaaaagatcgtgctcgcggtatctttttcactcaggactgggtatccatgccaggtgttataccggtggcttcagggg
gtattcatgtttggcatatgccagctctgaccgaaatctttggagatgattctgtattgcaatttggtggaggaactttaggacatccttggggtaatgcacctggtgcag
cagctaatcgggtggctttagaagcctgtgtacaagctcgtaacgaagggcgcgatcttgctcgtgaaggtaatgaaattatccgatcagcttgcaaatggagtcct
gaactagccgcagcttgtgaaatatggaaagcgatcaaattcgagttcgagccggtagataaactagatagctagactaagtggataaaattagatagaaaaaagg
tctaaataaaaaagaagagaaatagaaagatcaaaaatcagttacgaaaatgcagtaattcttctttttttcttctaattgattgcaattaaactcggctcaatctttttttcaga
ttgagccgagtttaaatagattttgatacgatcatgagacttgacaaatcgggattcctctattctatatatttagaagatataaaggtataatacaataaataaatacaaat
atagtattatcatatgataatggaatcaaatacgcagtatttacagaaaaagttttcatttattgggaaagaatcaatgacatacaatgcattacagacgtatgatcattac
cctttaaccgggttattctattccacttctagatagagaaaaaaactaaaggagaatgaatgaaaaaagacagagtttggaagttagacccctttctaagactctctttc
aaaaaagaggacattttgaaacttttaacaggcacaatcgtgagtcaacaagtgactcgaaatgctcgtaagaaaagagaattgattttcaaaatggtagaactagat
gacgaagttttctataaccttgatgaagaggtagttttagtttacgactccgatcaagagcgagaagtctttcatttcagattggactgctatagaatctggacccatcgt
agagacgttcaaaaggctcctgatgataagaatcatactttcgcggaactccatggggctatgggcttcaacgcggtagacgttttgttccgaatttttctggagcaaa
cctccgacccaacgatacaatgaatttttttctattcacaaagaaaaaagattcggaatcgcaatgctactatacgcacccggaggaatattcggaattatattcttatata
atccatttttgcgcggggtcttactaacaggacttcgctgcacgagacgaggattcgtcgaaaagctaaggtcatccggtattttcatacccttgggggtggtatatg
gccttacaaagtctaaggggtagtatgagatctgtagtaggtaaaagaatttgtcccttgattgagtatgctattttccctcctttaccgcgcattattgtatatgcttctag
aagagcacgtatgcagagaggaaattacagtttaataaaaaagcctaaaaaagtttcaactttacggcaatatcaatcaactaaaagtcctatgtatcaatccttacag
cggatttgtggggtccgagagtggttgaataagtattgcatgtggaaggaagtagacgaaaaagattttggattcgaaataggcgcattcgactaagtcgtactttga

atccaatttcaagttcgattagaaggatagaaaggccgcgaggatcggaaaagaaaaatcaaatctttttaattgcttctcctttttgctattttcttatttatttatataatcc
attcgattcttttttttagaatactttagtattctaaaaaaaagtattctataaaaaatctttttttgcaaactcaaaaatacaatagtcaatattcgttataatagatatacttaat
tatatcataagaatcttaagatatttttgaatagatcaaatcgaatagatagaaatagtaaatttgaatggagacacctattctatgatggattttaacttaccctctattttc
gtgcctttagtaggcttagtatttccggcaattgcaatggcttctttatttctttatgtgcagaaaaataagattgtctagaaccgacgggaccgaattttctcaatgtatttc
cacacaggatcataatacggatcttttgtagtgtaagtaatataatatggtatgttatgtggctctttctacacacaaatgcaaacccgctatggatggggattatggatg
cggatataggctacgagcataaatgcatgcatatgcggaaccgggtatagcgagttttttaagtggatcaacaaatactttttgaatagaaagtcaatgtatctaacca
attattttacaggagtatctagttggcgaaggcgatttcagaatcaaaaaaagtaaagtcaaaatcatttagcttattctctcaatttcaatcgaccgctgttagtatatcta
atatgaattggcgatcagaacacatatggatagaacttctaaaaggttctcgaaaaagaggtaattttttctgggcctgtattctttttctaggttcactaggattcttagcg
gttggggcttccagttatcttggtaagaatattatatctgtacttccatctcaacaaattcttttttttccacagggggtcgtgatgtctttctacggaatcgcaggcctattc
attagcgcttacttgtggtgcactattttgtggaatgtaggcagtggttatgaccgattcgatagaaaagagggagtagtgtgcatttttcgttggggattccctggaata
aaacgtcgcgtcttccttcgattccttatgcgggatatccaatcaattagaattcaggttaaagaaggtctttttcctcgtcgtatcctttatatggaaatccggggccagg
gggccattcccttgactcgtactgatgagaagtttttttactccacgagaaattgaacaaaaagctgccgaattggcttatttcttgcgcataccaatggaagtattttgag
taccaattgaatttttttgaaatgaattgaatgaagaagaattggaagaagaaaagttttctcaacacgagggaaaaggtcccttcgaaattgcattattgtaaggggatt
ttgagtatttatctaaaggaaggaacaaatgaggataagagaaaattgcttctaatttgtccaagtgggatatatggcgtaatattcttccattttcatccgaaaggacttt
tttctatttctctattccactccatctagatctaagaaagaactcaatgcaatgaaattctactagtatagaaaaaagaggaatagatacaaggtctcaaaccttgttatag
aatttttccttcaaagaaaaagaaatatcatatagagtcagcgaataaagcggattcattaacaactcaatttacagatcaaaaatgaaaaaaaagaaagcattgccttc
tttcctatatcttgtatttatcgtacttttgccttggggagtctctttctcttttaacaaatgtctggaactttggattaagaattggtggaataccaggcaatcccaaactctct
taactgctattcaagagaaaagagttctagaaagattcatggaattagaagacctttttatcttggacgaaatgataaaagagaaaccgaatacacatgtacaaaaccc
ccctataggaatacgcaaggaaataatacaattggccaaaatagataatgagggtcatctccatatcattttgcatttctcgacaaatataatctgtttggctattctaagt
ggttcttttttctgggtaaagaggaacttgttattttgaattcttgggttcaggaattcttctataacttaaatgactcagtaaaagctttttttattcttttagttactgatttttttg
ttggatttcactccacccgcggttgggaactactaattcgttgggtctataacgatcttggatgggttcctaacgagctaattttcactatttttgtttgtagttttcctgtgatt
ctagatacatgtttgaaattttgggtctttttttgtttaaaccgcctatctccttcgcttgtagtcatttatcattcaattagtgaagcataaactcatttgatctcctgatattaat
caaaattagcatcctttttcctttagaaagaaagccttttttccattttagcaaaattctttttttctatttctacctgctcaaggtattcatcattccagtacaactgttgcagtag
aatgacaacagattcgtgtatagggaactagattagcttagctacctatctaatttattgtagaaattccgggatctgtgattggacatggaaaatagaaatactttttctt
gggtaaaggaacagatgactcgatcgatttctgtatcgatcatgatatacgtaataactcggacatctatttcaaatgcatatcccatttttgcgcagcaaggttatgaaa
acccacgagaagcaactggacgaattgtatgtgccaattgccatttagcgaataagcctgtggatattgaagttccccaagcagtgcttcccgatactgtatttgaag
cagttcttcgaattccttatgatatgcaactgaaacaagttcttgctaatgggaaaaagggagggttgaatgtgggtgctgttcttattttgcccgagggattcgaattag
cgccgcccgaccgtatttctcctgagttgaaagaaaagataggaaatctctcttttcagagttatcgtcccaataaaaaaaaatattcttgtgataggccctgttcccggt
aagaaatatagtgaaatcgtctttcccattctttcccccgaccctgctatgaagaaagacgttcatttcttaaaatatcccatatatgtgggggaaaccgaggaaggg
gacagatctatcctgatggtagcaagagtaacaatacggtctataatgcaacgtcaacaggtgtagtaagaaaaatactgcgtaaagaaaagggggatatgaaat
atccatagtcgatgcatcggatggacgccaagtgattgatcttatacctcccggtccagaacttcttgtttcagagggggaatcgatcaagcttgatcaaccattaaca
agtaatcctaatgtgggagggtttggtcagggggatgcagaaatagtgcttcaggatccattacgcgttcaaggccttttgttcttcttcgcatccgttattttggcacaa
gttttttggttctcaaaaagaaacagtttgaaaaggttcaattgtacgaaatgaatttctaggtcccgggatttcttaccatcaagttggtaaaaagccgcgatttattgg
cgattgctagaattctctatgatccattttggaaatcttttttttgtttagactatttttttgtttgcgagatgtcttgtctgcaattccttatttctatcatctcatgcaaaacgagga
gaatgcaaggcaagggcgagaacaataaaaggaacaaatccttttaggagggattgctggtcttcttaatcctctattgggcacaagaaaaaggctttttttgccttttt
cttgtgtcgattcttcttgtatcgaaatatgaatctttttttcttcctattcggcaaagattactatttcttctttatttgggtctgtctcttgaacctcttttgcttaggttcaggcgat
gtagtggacaaaaagagggaaagaaaatatggcgggggacaaattttttgtgaccaaatagaattgcttgacttgttcaattaagttcaatttagaacttacagaaattt
tgcaaaaaaaacggatactcttccattgaaggctgttttttatttttaggctagttgagtagttttaattaaggttttattagtttattactctaaattaaatcaatgatttacaag
agacttcctccggggaataaaatattggatcctcgattgatcctttctttctcctcgcttcataaaagtgaattaatttcattggcgagggggttataaatcaactgatgga
ttacttcactaacattattaacaaacaaaattaacaaacaaaacgaataaatagagggattctgaccatcagagcaaaggctttctctttgttatttttacaaatcaaaata
ggaaacccgtttgtaggttatggaatagattaaaaaagtcgggttataagagtaagaattccgcgggtcctttccgctctaatcagataaaagggggtaaggacccg
ctaagttcctattttttcatgtttacaacctggtccctccaattactatagagatgaacccaatccagaatatgaaccgtaaaagaaaacacctattaaaccaatcacagc
aataccggttacagtacctatcagccaaagaggaattcttccagtagtatcggccatttcccctactttcctccacattttatcaagtggtcatgctagagacaaaaaca
gtcatggatagttataaggatggtatccttccaaatggggataagagaattcttactactctctttctttctctcaattgaagaagtaattggaaaataaaacagcaagtac
aaaaatgagtaataaaccccagtatagactggtacgattcaattcaacattttgttcattcgggtttgattgtgtcatagttctatagttggaatttggtttatcgttggatga
actgcattgctgatattgatcccaagaaaaaaacagtaggtacagctagtccgtgaacagccagccatcgcactgtaaaaataggataggttcgatctatggtcattg
agggcctcctaaaaggatctactaaattcatcgagttgttctaaagaatcaaaacggtcggttattaacggaattccttgtcggctttctgtgaaatactcgtttggccga
ggacttccaaacacgtcataagctaaacccgtactgacaaataaccaacccgcaatgaatagggaaggtatagtaatgctatgaataacccagtatcgaatactggt
aataatatcagcaaaagaacgttctcccgtgcttccagacatgctgagctccccaaatttttgtacattcaaaaaaggaattgattccgtaaaagatgggatcaaccag
taaatagaaaattactgatatttcatccttgtgagattgtcaattttgtaccaaaggtgtattttgagtataccaaattagtatagctatccttcctatggcacagcaatcctgt
ttcgcttggtctcgaaacagaattcttttttctcttctttgttccttgtctatagggtaagctatatggtattcaaggcatcaatagaaaacctcaattttgagggtcctactta
attttcaccggcttcggatcggaatagtagaataattcggaatagggctcaagatcttgggaaaatctaagttaatgatcaacaggtttggataaagaatttaggaaag
atattcttatactgacacaatacaaagagaagtagatgcgaaagctatcccttcgaatccaacctttccccttaaagaatttaattggttagcataatataatatctaataa
atagaaaatcaaatagtagataatctgttatgaaagagagaaaacattctttgaagaatcaagattcgtaatcaacccttgccttgtttactaactttcttgaccaaactgc
aagcgtggaactttgccagttcttaggtttccgaaatagtgtaatggaaaaagaagtgcttcgaatcgactttgattggggttcgaaaaaggaattaaaaaaaagtaaa
ttcaggaaagatttcctttttttaggagaccctcgaggggtcgtggaatgcttttcttctcctcttattccatatggaatacaatcaattaaaataagaaggaataggggaa

tatttgactgttcgctccaaaaagaaggttaaatcatcctattgaaaaagaccaaaatagaaagaactttttcaaattcaattctttatttttcactggggttagctgatctag
ttcttaatattattactttactcaattgacagattacacagcaaatctcttgattcggaattagggactcatgccccatctgatgaatccattttcttttacacttctgtatctca
ctctatcttgtttttttagtattatctaaaataaccgatgaattatgaattttccataacttaggtaagtgctttaccaacatatgtagtgtagtaaaaaaaatggaatttaaccct
ttcatgcttactataactagttatttcggttttctattggctgctttaactctaaccctagctctatttattggcttgaacaagatacgtcttatttgaaatgaattgaatgaataa
ggcagaaaagaaaaaatctctcttttggattcctggtattctacgcactaattaccaattcttttttttcttggtcattgagattcgtggataatttagactactatttagggata
aatcgtacctctttttttttatcccctcgaacaaatcgaaatgattgaagtttttctatttggaatcgtcttaggcctaattcctattactttagcgggattattcgtgactgcgta
tttgcaatacagacgtggggatcagttggatctttgattgagtaatatttctttttttgattgccctcctctctggtctggaggaggtcaaattggaattgcaatttgttttgtta
agttattttgccctgcttcgacataagatagatggaatcacgctctgtaggatttgaacctacgacatcgggttttggagacccgcgttctaccgaactgaactaagag
cgctttcaaaaatcaaatccttttctactcctaatgtgtctcacgtacgtatagtatccacaaattaaagttatacccgctttaatcgacctcctcactactgcctataaaga
agaaagaagtaataggtagggatgacaggatttgaacctgtgacattttgtacccaaaacaaacgcgctaccaagctgcgctacatcccttttccaaattgttgtaca
atgtcattgtacacaattcctatcttgttttccacatcctaattttcttgtctttttctatctatagagaatcctcgtgtcatttcttctttttggtctcatataatcaaggaatggtat
atatctaaatccaatctaatttcacctataaaagaaagattactattccttggtaatctataggaagggattgtctttttcgtttttagttcggaatttcgcctaaacaaaaga
aatacaaatgatcttgggcaataatatctgatcatatacgtattccaataaggaaggaggattttcaatgcgggatataaaaacatatctctctgtagcacccgtgctaa
gtactctatggtttggggctttagcaggtttattgatagaaattaatcgtttatttccagatgctttgtcattcccctttttttcattctagttgttgctatgtgaggaatagagtt
cttcgtgacatgataaaaaaattcccacttttcaatccctttttagtataggaaagaaaaagaaagaaaagatagattgggttggaccttagagtcatgaaaaatttggta
aatctcatttttgaaaaaagaaattcaattaaaagcagtatccaagctaagtcaggcctcagaaatcagagcatagaaagaggctgggttggctactaaaatgaaag
gattttgcttcaaaatccttgctaatttgacaaggattgtattccttaattatttctctattttttattacttaattgaaaaatttccaaaaatttttattctaatggattttcttcttcct
cttcggtttcaaaatagaggaataaaagaataagtagaagaattaagttaagtcaatccaaaaaggaaaggaggttcatggccaaggggaaagatgttagaatcag
agttattttgcaatgtgtgagttgtgttcgaaaaggggccaatgaggagtcggcagggatttctagatatagtactcaaaagaatcgccacaatacacccggacaatt
agaattaagaaaattttgtcgttattgtcgtaagcatacgattcatgccgaaataaagaaataggagcatcttgtgttcgatctttccaaagatcaaaaagaataagaact
tcctatttaatattcctatttaatatatagagcatagatagaatacaaaatacaaatcaacttgtctgatttccattagatattatttcatatgtatcgagggtattcatctaatat
atggaccaaagagagactatttcttctggatccaaaattaataaaataaacaaatcaattttttttcaattttaaaataaggaataaatcatgtatacatctaaacaaccctttt
cataaatccaaacaaacttttcataaatccaagcaaacttttcgtaaatccaagcaaacttttcgtaaattcaaacaaccttttcgcaaacccaaacaaccttttcgtagg
cgtcctcggattggcccgggggatcgaattgattatagaaacatgagtttaattaatcgatttattagtgaacaaggaaaaatattatcgagacgaataaatagattaac
cttgaaacaacaacgattaattactcttgctataaaacaggctcgtattttatctttcttaccatttcgtaactatgagaatgagaaacaatttcaagcccagtcaatttcaat
aattacaggtcctagacccagaaaaaatagacatattcctccattaacacaaaagttcaattccaatcgaaacttaagaaactccaaccagactttaagaaacaacaat
cggaacttaagttccgattgttgatgttttattcgaaagggccagactatatataaagaaagtaatccaatttagattcttgggtttgttataagaaagaacaatggggaa
gaaaaaatagtttttttatttattgcaacatgctcgttgattcctaccacttaatcttaatttattgtatcttcccggagttacctctccgggaattctttttttaattattcctgtatat
tactttttttatccctttaattgataatctttattttattggaaatcgtgtaaagattatttggatttaatacagctacttgtgcaaggattttacgattaagaatcaattctttcttgta
aagattgtgtattaatttactataattatcgaatactttatgtatccgcgttgctgcgtttatccgactgatccacaaacgccgaaaatccctcttttgcctgactctatctcg
atgagaggaaacaaacgctcttcttacctgttgagtaatcattcgattaagtcttaaatgagcccctctaaagtttgaggcaaatgaacgcatttttgcccgtcgtctccg
agctatatatcctcgcggaactctggtcattgaatcaaattaaccttaatgaataactaatgatttctcttcttttagccatcctttttcccattaataacaaaacgaattattc
ccatatataaaatattagttccaatggcttttgctactataaccttcccaaccacaattttctcttctactcccttcagttatttcgcacgatactaaaaaatagtgggttccat
cgtttctatggttcccttttaaacggcgaggccctctctatacaccggagccctttctttcatttcatcaaagggtattgtgaacttgtatagttcacattctttggctctacct
atccattatagagtaaatagctcttttcacaataagagttatccatacagtgacggcatttaattatgaaagttggctaagtagctgaccctcttagtccgtttttttaagata
aaggagcataagcctttatctttttattactatttcctccgcttaatgaatagccgtttgctaccaatgggggaattgcttcttatttccaatctagatgattggatttgcacca
aaggaaaccagaaattccatataccgtagaaatctaggatagagaagctctatcctattcattggtaccgatcatggatacttcaaaaattgtcttatttgtttgaacccat
gatccgaacgagtcgcacatacaccctagcacatgttcctcgacgctgaggacatcccttaagcgcggccgattttctagcatttcgtattggctgtcttgcgtttctaa
taagttgtttaaccgttggcatgtcgtatgtatatagaaaaaatggattggtttagatcgatcttaacctgaatgattgatcattatgaagtatttccattaaattgcataaaa
cccgaatttagggttgaaataaatttacaagaaatctggccactaccaatccttaaacatttctggaaaccacactggatcagtatcgcagtgctcgtcaagcatttcat
cccctacaatatcgacaagtccataagctttggcttcgtctgctgacataaaaacatccctttccatgtcttcggatacaacccaaaaaggcttgcctgttcttagtgcat
aaacccttgtgatcatttcgcgaactttgtgtaactcttccacttctagtaaaaattctggtgttcttgcccgataataagcactagcaggttggtgaagcataatcctcgc
gtgggggaatgctatacgcttggtgggttctcctccaagcagaatgaaggacgccatggacgcggctattccgaggcatattgtatatatatctggtgtcaccgtttg
catcgtatcaaaaatcgccattcctgagattaaccacccgccgggggagtttataaacaaaaaaatatcgctaattccatcttctatactgagatataccatgagacctg
taatatgattcgtgacctcgcaacgaatctcttgacctaaaaaaagtgtcctttctcgatacataacattgtataagtcaacccaagtcgcttcttcatctccgggaatcc
ggtaaggtacttttggaacaccaatgggcatattagattaatattattaaatttaagtaagaaaactacactttaatatggaaacgtaagaatggaagagaaagaaaga
atccgcagtttattgttttcactttttttcttattctatatgaatactatagattctattaatacgtagattgaaataatacatagattgaaagattatacatataagtaggataag
acagattgaataaagaaaaaagaatgggtgattcgaatactaaacaaaaagagatagggatctattcttcgtttttttccaaataagccaagctgcccattgcatattgg
cacttatcgagtatagaatagatctgcttctctttcttcttacgaacagaattggcttcttatttttaatggaatgaaataaatattcacgctttctgacacagaatcccctag
aagggttaggtacataggatatggatagtctttgccaatgcgataaaataaagtgacatcgtgtctatttttctttgctaaaggggtatttccatgggtttgccttggtatc
gtgttcatactgtcgtattgaatgatccgggtcgattgctttcggtgcatataatgcacacagctctagtttctggttgggctggctcgatggctttatacgaattagcggt
ttttgatccctctgatcctgttctggatccaatgtggagacaaggtatgttcgtcattcctttcatgactcgtttaggaataaccaattcgtggggtggttggagtatttcag
gaggaactgtaacgaatccgggtatttggagttatgaaggcgtggcaggggcgcatattgtgttttctggcttgtgtttcttggcagctatctggcattgggtatattgg
gacctcgaaatattctgtgatgagcggacgggaaaaccttctttggatttgcccaagatctttggaattcatttatttcttgcaggggtggcttgctttggctttggcgcat
ttcatgtaacgggtttgtatggtcctgggatatgggtgtctgatccttatggactaactggaaaagtacaagctgtaaatccggcgtggggtgcagaaggttttgatcct
tttgttccgggggggaatagcttctcatcatattgctgcgggtacattgggcatattagcgggcctattccatcttagtgtccgtccacctcaacgtctatacaaaggatta

cgtatgggcaatattgaaactgtactttccagtagtatcgctgctgttttttttgcagctttcgtagttgccggaactatgtggtatgggtcagcaactaccccaattgaatt
gtttgggcctactcgttatcagtgggatcagggatactttcagcaagaaatatatcgaagagttagtgatgggttagccgaaaatctcagtttatcagaagcttggtcta
aaattcccgaaaaattagccttttatgattatattggtaataatccggcaaaggggggattattcagagcaggctcaatggacaatggggatggaatagctgttggatg
gttaggacatcctatctttagagataaagaagggcgcgaactttttgtacgccgtatgcctactttttttgaaacatttccggttgttttggtagatgaagagggaattgtg
agagcggacgttccttttagaagagcagaatccaaatatagtgttgaacaagtaggcgtaacggtggagttctatggtggcgaacttaatggagtaagttattctgat
cctgctactgtaaaaaaatatgcgaggcgttcccaattaggggaaatttttgaattagatcgggctactttgaaatcagatggtgtttttcgcagcagtccaaggggttg
gttcacttttggtcatgctacctttgctttgctcttctttttcggacacatttggcatggggctagaaccttgttccgagatgtttttgctggtattgatccagacttggatgct
caagtggaatttggaacattccaaaaagtcggagatccaactacgaggagacagccagtctgaggccgcattgctttggtatctttcacctctctttttttgatttgacat
gggaaacatctcccatcccttctttgactctttttcctttttttatacgggaaatgatcccaaatgacaaatgaataggtgtggaagttataattgtaaataaaccacgatcg
aatctatggaagcattggtttatacgttccttttagtttcgactttagggataattttttttcgctatcttcttccgagaaccacctaaggttccgactaaaaaagtgaaataatt
taattgaagtaagaagtctcccagatggggagacttcttacttcaattagtccccgtgttcttcgaatggatctcttaattgttgagagggttgcccaaacgcggtatata
aggcatacccagtaaagcttacaagtaaaccagatatggagatggcgactaaagttgctgtttccatttttatagaatttcaagattacaatggatctacgaaaagatcg
tgtatttacaactacaacggaatagtatacaaagtcaacacaaatcattaaatggaatttatggctacacaaaccgttgaagatagttctagacctggaccaagacaaa
ctcgcgtaggtaatttattgaaacccttgaattcggaatatgggaaagtagctccgggttgggggactactccttttatggggggtcgcaatggctttattcgcggtattc
ctatctattattttagaaatttataattcttccgttttactggacggaattttaatgaattaggtttctactaacgaaaactacgaagtcatagtttttccatccaaaagagcctt
tctactttaagctctacatttctagacattctggtagttcgaccgtggaatttttttggtttcggtatctctggaatatgagtgtgtgacttgttagaatttgctcctattgataat
acatagaaagcacctgttatctctatcaagatgattctaattcgtcggatattatttattctagtatctggaacacgaaatagatagagtggatcaagaaaaaaaaaatgaa
actatgattcatattaactattcagacctcgcaaccagactgaaaaaaaattcaagtagttcttaataaaaataaaaaaagaaaatttcttccttccaattttgtttgcccaaa
aaacaactttttttctctcgattttgtcgagtcattacaccgattcaataaatgatcatcaagcggttcttattcgaagaacccttgccttttgtttagcttgagactcaatcat
cgtggctctagtatgaatctaaggttttaattgaactgattcataggatcgcaacaagataatttctaccagaaaactactccaatttttgctttatttatttatctagtaaaac
aagagtaaatctgcattacgcacaaaaaaaaagaaatccaaaatagggaagagaaaaatcaagaggcctctaatgatcaacatttgggaaagaaagatagacgag
ccaacttgagattttttggcattatcatcacaaagaagaaattctggatttttcttatttcatatcttcaaggcaaatcgacccaacccagtggctgatgaagttttgaacct
tttttctaatatccgttgaaaatttgtgtgtttctgtttgagccgtacgagatgaaattctcatatacggttctcggagggggggttcgggttagttacctatctcaataaagta
tatgattggtttgaggaacgtcttgagattcaggcaattgcagatgatataactagtaaatatgttcctcctcatgtcaacatattttattgtttaggggggaattacacttact
tgttttctagtacaagttgctaccggttttgctatgactttttactatcgcccaaccgttacagaggctttttcctcggttcaatacataatgaccgaggccaactttggttgg
ttaatccgatcagttcatcgatggtcagcaagtatgatggttctaatgatgatcctgcacgtatttcgtgtgtatctcacaggtgggtttaaaaaaccccgcgaattaactt
gggtcactggtgtggttttagctgtattaactgcatcgtttggtgtaactggttattctttaccttgggatcaaattggttattgggcagtcaaaattgtgacaggtgtgcct
gacgcgattccggtaataggatcacctttagtggagttattacgtggaagtgctagtgtgggtcaatccactttgactcgtttttatagtttacatacctttgtacttcctctg
cttactgccgtatttatgttaatgcactttctaatgatacgtaagcaaggtatttcgggccctttataaggaaggcatctcatagagagttctaattctcatatatcatatcg
ggtaggttgtggtatttcattgctacaaacatgggttattctaaaataagacatgtcatttagatacttctcttcaactccgaactattgtgatacaaataatacaaatagttg
aagttaattttacgaaagaaaagaaggcggattatgggagtgtgcgacttgaattattgatttggccatgcagatagagagttggatctgccacattagaattcacgac
caaaggtgtctccatatccaatcaacacgtaagtcccctgtctagtaaggataggctggttcacttgaggagaatattttctatgatcatacctcaaccatgtcatccatg
aagaggctccgtaagatcccatagagtagaaatggaataagtcatgtgacatgatccaattctctatttattacacttactttttattatagtatggaaatgcattcattttctt
tgcatcgattgcgatctgcaatactatcggagtaaaagaagggatctaaggaagaacgtaggctaaactttttgatttttttattagtaacaagtaaatactttgtttggac
gtaagaaacttgcaatattggggggggataaataccaactaatcaagagacgcgagacaatccacaaagcaattgatcatgatcaaatttgtaagcccacttggatatt
gagcatttacccataagagtaggattcttttcaatgaatagttgtaggtgcaacttcggaaaatagaatctgataaagcttttcttacttagagccattgagccattatata
ccttattctattatggatcttctacggttttatttctttcattcttgctcgagccggatgatgaaaaattctcatgtccggttcctttgggggctggatcttaaagaattcaccta
tcccaataacaaagaaacctgacttaaacgatcctgtattaagagcaaaattagctaaagggatgggacataattattacggggaacccgcgtggcccaacgatctt
ttatatatttttccagtagtaattctaggtactattgcatgtaatgtaggtttagcggttctcgagccgtcaatgattggtgaaccggcggatccgtttgcaactcctctgga
aatattacccgagtggtacttctttcccgtgtttcaaatactccgtacagtacctaataagttattgggcgttctcttaatggtttctgtgccgacgggcttattgacagtac
cttttctagagaatgtcaataaattccaaaatccatttcgtcgcccagtagctacaaccgttttttttaatcggtactgcagtagctctttggttaggtattggagcaacatta
cccattgaaaaatccttaactttaggtctttttttagggatttttcagtttgattcattcaatcgtgaagtaccgtgcataggtatctaggaaatagttacttccaagtgaatctt
ccctagatacctataatctattttattatgatccatttcgcgaaaatatagattgtcccaaagatgcaaaattgttttctttttttctttttattctaactcgaaaaagaagaagag
gaaaaaattgcaatggatttaaaactagaacttattcttaggtaaatccattgggagatgcttctctagagtgtcccatatctgttttccatcttgcatacgaaaactgtcaa
ttctcatcagatcttcttccgtcttactcaaaaggtccaatagtgtatggatattggcccttttgagacaattatacgttctagaaggcagttctaattgatcaataaaaata
caattcaatggaattctttttttgtttttctttagattagttaatctttttttgaaagcttaaaagggggggaagtaaacctgtttttattttcttggaaacgagtaccctcttcctccg
tgtgaagaaaaggaagaaataaatcaatcaaattacgagaagcctcataaagcgcttccttaggggttaaacttccattggtccatatttctagaaaaagtatctcatat
tttgcatttccattcccacaagcaaaaatactataattcacatttcgaacaggcatggatacagcatctataggataacttccatcttgagtgttctttctgacttccgtgtg
atatccgcgatctctcttgatccgtaactcaatacggaaatccgtgggttctgtcaagttagctataggttgtgccgtatcaacgatttctacggaaggcggtaagataa
tatcttgggcagttatgtatctaggacctttgacgcaaattgatgcggttctaactccatagagattacttctcaatacaatttctttcaaatttagtaaaatttcttgtaccga
ttcttcaatacctgctattgtagaatattcgtgcggcacgctcccaaattttgcatgtgtgatacatgttccttctgtttctccaagtaaagctcttcgcaaggcaataccaa
cggtatccgcttgaccttttctaagcggggacagaatgaaacgaccataataaagacgcttactatctactcttgattcaacacacttccactgtagtgtttgagtggat
cctgctacctcctctcgaaccatatagactagtattattatttgatcattgaatcgtttatttctcttgaaaggggtttaattcttttacagacgtcttttttttaggaggtcgaca
cccattatgcggcataggtgttacatcgcgtatacaacttaatcgtacaccacttttagcaatggctcgtaatgcggcatctcttccactaccagcaccctttaccataac
ttctgctcgttgcaaacccactgtacgaatagcatctacagctgttctttgaccagcatagggtgatgcttttcttgagcttttgaatccacaagtacccgcggaggacc
agaaaaccacccgaccttgcggatctgtaacagttataatagtattgttgaaactagcttgaacatgaataactccttttgttattctacgtgcactcttccgtaaactaaa

acgcgcattcctacgcaaaccaatacgtactttcctacgtgaaccaatttttggtatagcttttgtcatattttattatctcataaatatgagttagaaataacaaaaagaaa
aaaagatacaaagatatccgtttcagggtaaaatatatcctttacttgaattttttttatttggaatttggacattttcgcgggtactttttttttacttttttagaaagtcaagttcttt
tttcgaaagattacccctgtctttgtttatgcttcggattggaacaaatgactctaattcgtccacgcctacgaatcagtcgacattttgtacaaattttacgaacagaagct
cttattttcatatttccgtattcctttcttaaactatgaatttactcttttggaaaaaaataagtctctttgtttgaattttagaactttgaatttttttaccctagaaaaaaaaagaaaa
acctaaccctttgaatctttggtatccttcaaatcttcggtatccttcgagtcttcggtacgcttcgaatccttatggggaagtctataaattatacgccccttgcttgaatca
taacgacttacttcaattttgaccctatcccccatcagtattcgtatagaactagaccggatctttcctgaaatatagcccaggatgatggtgtcattctctaggcgaacg
cggaacattccgttgggtagggcttccataactaaaccctcgaaagtaatttttgcttctctcggggttttttttttctctcctatttttcttttctgtcatatttttttttctcctattttt
ctttttttatttgaaaataaaaaaaacgttgtatttttgtttgaaaaataggaagttcaagatagaatttgggtactataactataggtggggcgattaccgctataggcggg
gcgattaccatatataacataagacttctcccccaattctgtttagtcgagcttctcgatctgtcattatacctcgagaagtagaaagaatagcaattcccattccgccca
aaaccttaggaattccttgatagttggcataaattcgtaagccaggtcggctgatacgctttaaaaaggttctagttctatatattccctttctagtctttctcttttgatgtcg
caaagttgaaaccaagaaatatctgttactttcctgatgtttccgaacactttcaataaaaccctctcgtagaagtattttaacaatgttttcggtaatatttgtagatactac
ccgaacagttcctttttttattcatgtccgcgtttcttatagaggttagtaaatcagcaatagtgtccttgcccataagactctaattctaggttcctcctaatttttctataatca
acatgttctctttttttcttttcgttttggattctaaagcatatacgtgaaacacaatctactaatttttttatttgatctatatcttgtctactagtatttataatacttcaggagctaat
gaaactattttagtaaaattcaattctctcaattcctcggcgatcgcgccaaaaacgcgagttccttttggatttccttttttgatcaatgataaccgctgcattgtcgtcata
gcgtattattataccgtcttcgcatttgaactctttacatgtacgtacaattacagctcgaattacttcggatctttctagaggcatttggggcactgcgtctttgattacagc
aacaataacatcaccaatacgagcatatcgctgattactagcagctcctatgactcgaatacacatcaattttcgagctccactgttatctgctacatttaaaagggtctg
aggttgaatcatattattttgatttcaatttgttatttcaatgcaaaaggatgaaagaaatattgtctttccagaaagaagaacctggttttttattttcaatactactttttttggg
ggggttatatttctaaccgaagaaattgacttcgtatgggcattttgctggcagctatggaaatagctgctctagctacagtttcggatactccgcccatttcataaagta
ttcgacctggtttaacaacggctacccaatattcgggggatccctttcccgaacccatacgtgtttcggtcggtcttattgtaaccggtttgtcgggaaatatacgtacc
cagatttttccaccacgacgtgcatatcgtgtcattgctcttcgtcctgcttctatctgcctcgccgtaatccaagtgggttcaagtgcttgaagagcatatctaccaaaa
caaatacaattgcctcggtaggattttcccttcattcttcctctatgttgtttacgaaatctggttcttttggggttatagtcgatggttctctcttagttccatctctactgcaaa
actggacatgagagtttcttctcatccagctcctcgcgaatcaaatgagaaagtgtgcaaatttctctaattccacaatattctaaaatattacggatagatacacattaat
aggaaaggttagggtttttaatattgaatttattaaaatactaaaatatatagtcaagaaagaggatctagaatatatctagatgtgtatgtttatttatctatattctatattta
atgtaattctatttttttttttaatctccttttttttattttactcttattgaatcgcggtaaagtattctaattcaataaaaaatttcgcgggcgaatatttactctttcctgtcttatttgtta
attcataaccttatcaaataagacaattttttttggtttgttccgccatcccacccaatgaagtattgggattcttttcaagaaaatcctatccagtcataggttctgtcgttcc
cactgcttctcctttaatggttaggtttgaatctcgcaatggagcttccaaaaaaatttctttccgagtcaatttctcagttttattaactcgggacgctctttgttattccttgaa
atttgtatttcttgtttcaatcaaattacgatttcgaattctctgttatttttattatattgatgctttatcacattgctttttatgatgtaattcatagaccatacatattggaatcctat
atctttcttatttctcttccttctttctatcatccttccttttatctacatcccctttagttttgcttcacaacttagaatcctattttttttttaaagaaaaaaattgcagttgctacacgta
tatgatagatttactcatttatgatagatgtatttattcatatagtgactgtttcttagttaggatctcgacaatacgaagcaataggttggttattagttcattttctataattac
atagtagggttcattctatttttttttttaatccccttttttgaaccctaaaaaaaaaactaacgagtcacacactaagcatagcaattttattaaaagatttctcaattttcattaaat
cttatagaaagaggtagaatttcttctttttttttcagggattttagggaaaataaggctcttgtcatttttttattctattactgaacagaatgggaagacagggttggttattctt
cgtctacgaatatccaaattttaactcctaatactccatagatagttcgaattggatagcagcaataatcaattttagcgcgaattgtttggaggggaagtctaccttttttttg
atgcattcggcacgcgcaatttcttttcccgcgagacggcctgcaatttttactttactccctttatatctgtttttttagttaattcaatggcttttttcattgcctttcggaatg
aaactctatttttttaattggaaagctatatattctgcaagaatgttaggttgtctataaggttctttcactttttcgatcccaatattaagtctctggtttacagaattaacttctttt
tgtagatctttctctaattcttcgattgctcctttttttctttaataaattggggaatccaatatggattatgacgtggattgtatcgatttctttttgaatttctatatgtgtaattact
tcggaacttgagcctgagtccatttttttttattatgtgtaattacttcgaaacttgagtctgcttctatttttctattcgagcccttttttcctattcttttgtatatagttcttgatacaa
ttccgtatttttttttatcttcctgtagaccttcagaataatttttttggttgtgcgaaccaaaaggaatggtgattttgggttgtaccaagtctgaaaccaagtggatttattttttg
tcccatatttttctattctattttttttttaccgggaatcaaatcttagatggatctaaagattatttcgatttctttactatatttagtacaattgttatatgacacatggttttttttttatg
ggagaactacgtcctcgagcccgaggtctgaatttattcataatagtactcctactgacttccgctttagtgatgaataaatttgctttgtcgaaatccctataatgagtag
catttgctgctgccgaataaaccaactttaagatgggataagatgctcgataaggcatgaggttcaatatcataacagtttcttcgtagtaacgccaacgaatctcatcc
agaactctttgtactttgaaaacggacatgtggatgcgtttttgtgctttgaaaacccgttcgaacttaagtagacgatcggttggtacttttcttgcgagtttccttagccc
actcttcttcttctttatcctaggggtatactttaccagtttgaaacttgtcataaataaggttattccccgcttacctatttctttttttttttttatttttgaatctttctattctgaattca
gttaacgacgagatttagtatcctttcttgcactttcataactcgtgaaatgccgagttggtacgaattcccccaatttgcgacctaccataggatttgttatgtaaataggt
atatgttcctttccattatgaatcgcgattgtatggccaaccattgcgggtagaatgctagatgcccgggaccacgttactattgtttctttctcctccttcatattgaccttttt
ctatttttgccaataaatgatgagctacaaaaggattcgttttttttttcgtgtcacagctgattactcctttttttttccattttaaagagtggcattcgatgtccaatatctcgatcg
aagtatggaggtcagaataaatagaataatgatgaatggaaaaaagagaaaatcctttagctggataaggggcggatgtagccaagtggatcaaggcagtggatt
gtgaatccaccatgcgcgggttcaattcccgtcgttcgcccatcccattattgcaaattccaaaaatgcaattttccatattcctagttacgtatttacttacggcgacgaa
gaataaaactatcactatatttttttccttttcctagttcttcttccaagcgcaggataaccccaaggggttgtgggttttttttctaccaatgggggctttcccttcaccgcccc
catgggggtggtccacagggttcataactacccctcttactacggggcgtttacctagccaacacttagacccggctctacccaaactttttttggttcaccccaacatt
acccacttgtccgactgttgctaagcagttttgggataccaaacggacctccccagatggtaatcttaaagtggccgatttaccttcttttgcaatcagtttcgctacagc
acctgctgctctagctaattgcccaccccttccacgtgtgatttctatgttatgtatggccgtgcctaagggcatatcggttgaagtagattcttcttttctctcaaaaaacc
ccttcccaaactgtacaagcttcttccaaagcatacggctttctagatgtatatgacgatctctagacagatggatcttatatgaatcgtatgatgaagtaccacatgagt
ggatatataggaaaggaatccaaatctgccgaatcgctcatgttatgatcttctacatcctaggtctccgcgttccgtcatctggcttatgttcttcatgtagcattcagat
cgaatgactctatgaaattacgtcgatacttctacatattatgggtaacgtaggagacatccctattttcccccgggggtcttaattaccactgcttagctttcaattcgcc
tctgaccatcaaattaaatgtgaataacccgtcctcctctctttgaaacaaggggcgcttccggttctgtgcgtgcttcaaacaattttgtcttctccatattaccatatctct
agagtcaataattttctatgaggaactactgaactcaatcacttgctgccgttactcaacagttttctgttgaggtctatcccgtagaggtagtcaaattggatcagtgatc

gatttctaggtttcgtcgtaaacctaattggttacttccaattacgtaaatcaatagttcaaaccgcactcaaaggtagggcatttcccattgatataggaacttttgtacca
gaaacaatagtatctccaattatagcccctctgggatgtaaaatatatcccttctcaccatccccatagtgtatgagacaaatgtacgcatttcgattagggtcgtattcta
tggttacgattctaccagatatgtctttttgattccgtcgaaaatcgattttacggtataggcgcttatgacctccccctctatgccttgcggtaatgattcctctggaattac
gacctttaccacaacggtgccgtccatggatcaaattatttcgtggattggatttcacttgcctatctatggttcccttgcgtgtgctcgggataggtgttttgtataaatgt
ttcgccgtattattaagtattctcctttagtttttttctctatctagaagtggaatagaataacccggttgaagggtaatgatcatacgtctgtaatgcattgtatgtcctagaa
tagggcccattcttctaccctttccgggtagtcgatggctattcacagctactaccttaacaccaaagaagagttcgacccaatgctttatttctgtcttagtgaatcccg
attcgacattaaaagtatattgattctttcccaataaacgaagactttttctgtaaatactgcgtatttgattccatccataaatcgactttccctcctatgctctgagttcca
gtatcgataagaattcgagttcttattgttcttatgttatggtatgaatataccataccaattcgttatgtatggatgatggatgagattccatggatagagagccagttcca
atagacttatggaacgttccggttcgcgtgcatccagcaggaattgaacccgcaaatttaccaattatgagttgggcgctttaaccattcagccatggatgcttaacag
ggatcatcgtacatcgtaaataaccaattttcatatagaaagacatatcatagaaaaatgaaatcgaaaatattcggagatggcaaatattcggagatgactatgaaaa
cacctctctggatcctcgaattgaaagagagattgagagggatccagaatcctaattctcgctatttggaatggatccaattctattgagtctgactcatagtgatcattt
ctctttagcaaagaatgaccttggttatcaaaggattgaacaaccgggatccgtttacttatgatacctagttgacattgataacaaggatctaatgaattatgagtttaat
agatcctctttagcagaaagacgtatattccttgctcatcacttattcccaaacctcgtatggggctaatcgttttcatttaccatctcagggaaaatccttttcgttccgctt
agccctatcgggtattttagtgataggttctataggaactggacgatcctatttggtcaaatacctaatgaaaaattcctattttcctttcattaaggtacgagggcttcttat
tccacaagaaaagaacgaaagcaccttttcattctttcatatactgggggtttttacttggaaaagacaatgttccatactaaaggattcgggtccataaccacgagttc
cagtgcactagatcttgtagcacttagcaacgaggccctatgaatagacatatagaatttttggtcgggaaattcgaatgaatcattgagtgaaaaaggagcaaagaa
tgacaaaagacgagactctactagtcttcactcttgtggtttcctcggtttctgttttcttattcgggatcttgcttttcatggttctcatctctgcaactcgcgattttcgcga
gagaaccaaatccaagttggtgaagatcatgatttgggctggcatagtagtgattacctttgcaattgcggttcgaatctatccgatctttatctttttgctcaaagaacg
aataaaaccccttgtcgaagccctttatgataagcttccctggatctgggaagtttctctttcacggtattgggatcgtttgatcgatttccttgatcgctacttatgggcgt
gcgctcaaaggatacaaacagggattcgcaaacaaaaaggggaattcgtagtcactttttcctgtcgcgtaaaaaaaaggctttacgcgagagcaatagaggttgg
gatacatctatctcttctgagcaacctcttttggattcttaagaccacccttgcagtaggataccgtctgctttgggttctttattatattatctccttcgagggatttttaggat
cgttcaggctatatttagtctattttggcttttactgtctccttttctcagggaagtggttaaggacctcagaagatagaggagagcgccaggcgcagatttccggaata
cttctacggggaatgctcattgaatgcgcattctccgtattatgccttgaagaggactcgaacctccacgctctttagcacgagattttgagtctcgcgtgtctaccattt
caccatcaaggcatcttgaaagtgaatcatattccatgaatatgatatctatctaatgtgatatcttgaaagtgaatcgtattccatgaatatgatatctatctaatgtgatat
atggaatatatgacaaaggtggagtcttggagtatttcgatcgatcggtcatataggcctgagtcagacatcaaatagcttcgatttgcattatccgtaggacaccttat
atgtatcaaaatcaaaaagatgtacaatccaatttctcgattcaatagaagcccaaagaggtgcatatggtacccaaataaggataggatagatatgtcaaaagcag
gtctgattacacctattcctaatcctaaatagaatgtaaggacgtggggatttctatgtaaacagagtatcctatttccataggctcgaatgaccccttctcataataaga
atgtgcacggtctggtccggtatggaatgaacttataatctgatgatcgagtcgattccatgattataagttcattaccctagcgcccattcccattttgggcggaacag
atctactaattcttttattccagttagtaagagggatcttgaactaagaaatagacctagcagctaaaagagggtatcctgagcaattgcaagaatggggttcattgata
ttcctggtatagtagatgctatcacacatacagtcatactcaattcgatggaattgtttgatcttaaaggggatcttctataatttcgcacataaggggttatttcttggtttc
gtccagtcattaataacttgactatttttagataatagtagatagaaagaacgctcgtaaggagtcctattgaaaccaagaaatataggcctgcttgccatccacacca
gaatagatagagttttccgaagaaacctgctagtggaggaaggcctcctagggataagagacatagggctaaagagagagccaaaaaaggatctttcgtgtataat
cctgcataatctcgaatgttatcagttccggtacgtagaccaaataatacaatgcaagcaaaagttcctagattcatggagatatagaaaagcatataagttatcatgct
tgcatatccatcatttgagtctccaacaattattccaataattacatatccgatttgccctatggacgaatatgcaagcatacgtttcatgcttgtttgagtaatagcaagga
gattccccaatatcatgctaagaatagctaggatttccagaagaagatgccattcgtttgatgagaaataaaaaggaatatcgagaattcgcgtggctgaagctgaag
cagcaactttcgaagtaacagaaagaaaagcaacgactggagtgggggagtcagagtcgaaaagaggattcctcgcttctttctctcatgcaaaaccgtgcatga
gactttcatctcgcacggctcctaagtgataaaagaaagaagaacttgtcttctttctttttttgattaccttcctcgcgtatgtataagaccgaatccattctttttcgaaatc
gatttcgaaaaagaactactaatccttaacttttcgaggaatccttcatcagtggttgtgaatgactgactttttcaatcctttcgaccttggttccgtaggagcaagtcag
aaaggttgagaaatagaaccatctgatttgattcgttcccaatagccatgagatgatcatcttagggtgatccttttgtcaacggatgctcctattacactcgtagtctctg
aaggatgagaacccactatgtagcatctacgtcgataattcaagcattgtatacgtcattagtccgattctttgtaggaactacccgtaataacgagcttgcaaaatgga
tctgtttatcataaagagattcgttgttcctgaccctgcttcaccttaattgttatttgaacaaaaagatcacaataaacttttggtaaaagttctgtcttggtcggagtgggg
atagcatttctcttctgcatatctatggagttttgcaaaacccaaacacctcagagatagatatagaggtaggaatttgtcgaacgaaccacactccttcgtagacgtca
ggagtccattgatgaaaaggggctggggaaagcttgaacccaagtcctacagtgatggatataagcgcaattgaaattcctggggagttatacatttgtgtattgata
agaccgttcacaatttcttgaagctcgatctcccccccagatgaaccatatagccaagagaaaccatgaaccagaatagaagagcttgccccacccatgagtaaat
atttcatagtagcctcattagaccgtagatctctcttggtatatccagacaataggtaggaacataaactgaaacattctggagctacaaagatagttattaaatcgttag
caccacataaaaacattcccccctagagtagctgttaatacgaataacagaaactctgttatagccatttctgtacattcaatgtactctacggatagaggaatacataaa
gttgaacataataaaataagaaattgaaagatttcgttgaaattgttcgtttggaaatttcccgaaaagctaattataggttcttctctccatcggaacaatagggccgtta
tgcttattactaaacttgttgaagagatgaaatagaaccaaggtctatctttttgatcagaggttaaatcgatcatcagaagaagaattaggccaaaaattaggatacatt
ctgggaaaatgaaacttccctggaagagaagcaaatgaaacgctttcataaaaattctcgtagaatcgagaatgaagttttcattctgtacatgccagatcatgaatta
gtaactgcatccaatctccgaaaagtcccgattgtttcgatttttggaatgggatatttacggaatccccatgaataggatcaaaccttattccatgctatttccataagatt
cctctttcttattcttaagcaagcccccgagagggcttagttgatcatgatttctgttttctctttcttttcctttttgtttgtttcgagaaagatatcgtccgattctccttctattg
attcttttccgatcgagatgtatggatccatgtgtctacatacctagattctgttcatggattaacgaaaatgtgcaagagctctatttgcctctgccattctatgagtcgctt
cctttttgcgtatggcacccccaccccctttggcagcatctactaattcggaacttaatttgaaagccatatttcgacccggacgcttttgggatgcttctaataaccaac
gaatggcaagtgctcttccttgtttagatcctatttcaatcggaactttccgcgtcgatcctttttttattacgtcttgtttttactcctatattgggagttactctacgtattgcttg
acgtaaaaccaatagtggatttgtttctgtcttttgttgaatctttttcacggctcgatagagaatttgataagccaatgattttttccgtctttcataatacggttaaccacca
tgttaactaatcgattacgaaaaattggatcggattttgcagttctttttctgcagtacctcgacgtgacatgagcgtgaaagaggttcaagaatccgttttctttttataa

gggctaaaaacgaatcacttattttttggctttttgaccccatattgtagggtggatctcgaaagataggaaagatctccctccaagccgtacatacaactttcatcgaa
tacggctttccacagaattctatagggatctatgagatcgagtatggaattcagtttactcacttaaaattgagtatccgtttccctccttttcccgctaggatcggaaatc
ctgtattttccatatccataccatcaagtccttaggtttccgaaatagtgtaatggaaaaagaagtgcttcgaatcattgctatttgactcggacctgttctgaaaaagtcg
aggtatttcgaattgtttgttgacacggacaaagtaagggaaaacccctgaaagaatttccatattgaccttggacatataagagttccgaatcgaatctctttagaaag
aagatcttttgtctcatggtagcctgctccagtccccttacgaaactttcgttattgggttagccatacacttcacatgtttctagcgattcacatggcatcatcaaatgata
caagtcttggataagaatctacaacgcactagaacgcccttgttgacgattctttactgcgacagcatctagggctcctcgaataatgcgatatctcacaccgggtaa
atccttaacccttcctcctcttactaatactacagaatgttcttgtaaattatggccaataccaggtatataagcagtgatttcaaatccagaggttaatcgtactctggcaa
ctttacgtaaggcagagttgggtttttggggttgatagtggaaaagtcgacagataagtcacccttcctgtccctctacagaaccgtacatgagattttcacctcatac
ggctcctcgttcaattctttcgaagggatccttttcctcgttcgagagtctccgcccttcttccactccgtcccgaagactaactaagaccaattgagtcacgttttcatgt
tctaattgaacactttccatttatgattaaaggagaagattgttcttttaccaaacatatgcggatcaaatcacgtcttataataagaagaaatctttctcggtatcaatccc
cttgcccctcattctttgagaatcagaaggatccttttcgagtttccatttcttcatttggaatctgggctcttctatcttcgacttattttttttttgctttattctttatttatttcatt
tcgatttttccctcttcctctatccctatcctctaggtacagcgtttgcatcaatagagaaccttttcctctgtatgaatctatattattccattccaatttcttcccgaaacttcc
caagaaaaatcccgaattggatccaaaattgacgggttaatgtgagcttatccatgcggttaggcactcttcaaataggaatccattttctaactggctttcgtgctttgg
tgagtcgtccgagatcctttcgatgacctatgttgtgttgaagggatatctatatgatccgatcgattgcataagacccgcggtagcaatagaacggggaaagtatac
agaaaagacagttcttttcaatttcgattatctatatattagttcgtttctatttctagatatctatttctatatattagtattagttagtagtactattctattagttagcgatcccg
gctctgtgagttctttcttccgtgatgaactgtcggcaccagtcctccatttttctctgtggaccgaggagaaagggggctcagcaggaagaggattgtaccatgag
agaagcacagaggtcaaccctcttcaaatatggaacatggattctggcaatgcaacgtagttgggtcctcatatcgatccgaatgaatcagtctttctacagaggtca
atctttgcctattaggcaagaggatagcaagttcgaaattctgtctcggtaggacatggatttctattactatgaaattcataaatgaaaatgaagtagttaatgggagg
gctaccattatcctttttcttgtatgtgttcctaagagaaggaatttgtccatttcatgtttcgaggtctcaaaaaagggcgtggaaacagatagaaactcttgaatggaaa
ttgaaaagaaatgtagccccagttccttcggaaatggtaagatctttggcgcaagaagaaggggcgacccatatcatcttgacttggttctgcttcccctcttttttaag
aataccgagtcgggttcttctcctaccagtatcgaatagaacatgctgaacaagatcttcttcatggaaacctgctcgatttagatcgggaaaatcgtacagattttatg
aaaccatgtgctatggctcgaatccatagtcaatcctattttcgataggaccggttgacaattgaatccaatttttcccattatttgactatccataatagtgtggaaagaa
agcccggaggaagggtggccttgagtttctcgcccctttgccttaggattcgttaattctctttctcgaggggacggggaagggatataactcagcggtagagtgtca
ccttgacgtggtggaagtcatcagttcgagcctgattatccctaaacccaatgtgagtttttttctattttgacttactcccccccgccacgatcgaacgggaatggataag
aggcttgtgggattgacgtgatagggtagggttggctatactgctggtggcgaactccaggctaataatctgaagcgcatggatacaagttatccttggaaggaaag
acaattccgaatccgctttgtctacgaataaggaagctataagtaatgcaactatgaatctcatggagagttcgatcctggctcaggatgaacgctggcggcatgctt
aacacatgcaagtcgaacgggaagtggtgtttccagtggcgaacgggtgagtaacgcgtaagaacctgcccttgggaggggaacaacaactggaaacggttgc
taatacccccgtaggctgaggagcaaaaggagaaatccgcccaaggaggggctcgcgtctgattagctagttggtgaggcaatagcttaccaaggcgatgatcag
tagctggtccgagaggatgatcagccacactgggactgagacacggcccagactcctacgggaggcagcagtggggaattttccgcaatgggcgaaagcctga
cggagcaatgccgcgtggaggtggaaggcccacgggtcgtcaacttcttttctcggagaagaaacaatgacggtatctgaggaataagcatcggctaactctgtg
ccagcagccgcggtaagacagaggatgcaagcgttatccggaatgattgggcgtaaagcgtctgtaggtggcttttcaagtccgccgtcaaatcccagggctcaa
ccctggacaggcggtggaaactaccaagctggagtacggtaggggcagagggaatttccggtggagcggtgaaatgcattgagatcggaaagaacaccaacg
gcgaaagcactctgctgggccgacactgacactgagagacgaaagctaggggagcaaatgggattagagaccccagtagtcctagccgtaaacgatggatact
aggtgctgtgcgactcgacccgtgcagtgctgtagctaacgcgttaagtatcccgcctggggagtacgttcgcaagaatgaaactcaaaggaattgacgggggcc
cgcacaagcggtggagcatgtggtttaattcgatgcaaagcgaagaaccttaccagggcttgacatgccgcgaatcctcttgaaagagaggggtgccctcggga
acgcggacacaggtggtgcatggctgtcgtcagctcgtgccgtaaggtgttgggttaagtctcgcaacgagcgcaaccctcgtgtttagttgccactatgagtttgg
aaccctgaacagaccgccggtgttaagccggaggaaggagaggatgaggccaagtcatcatgccccttatgccctgggcgacacacgtgctacaatgggcgg
gacaaagggtcgcgatctcgcgagggtgagctaactccaaaaacccgtcctcagttcggattgcaggctgcaactcgcctgcatgaagcaggaatcgctagtaat
cgccggtcagccatacggcggtgaatccgttcccgggccttgtacacaccgcccgtcacactataggagctggccatgtttgaagtcattacccttaaccgtaagg
agggggatgcctaaggctaggcttgcgactggagtgaagtcgtaacaaggtagccgtactggaaggtgcggctggatcacctccttttcagggagagctaatgct
tatgcttattgggtattttggtttgacactgcttcacgcccaaaaagaaggcagctacgtctgagctaaacttggatatggaagtcttctttcgtttagggtgaagtaaga
ccaagctcatgagcttattatcctaggtcggaacaaattagttgatagtgataggatccccttttttgacgtccccatgcccccccccgtgtggtgtggcggcatgggg
atgtcaaaaggaaagggatggagtttttctcgcttttggcgtagcaggcctcccaaagggaggcccgcgcgacgggctattagctcagtggtagagcgcgcccct
gataattgcgtcgttgtgcctgggctgtgagggctctcagccacatggatagttcaatgtgctcatcagcgcctgacccgaagatgtggatcatccaaggcacatta
gcatggcgtactcctcctgtttgaatcggagtttgaaaccaaacaaacttctcctcaggaggatagatggggcgattcaggtgagatcccatgtagatctaactttcta
ttcactcgtgggatccgggcggtccgggggggggcactacggctcctctcttctcgagaatccatacatcccttatcagtgtatggagagctatctctcgagcacagg
ttgaggttcgtcctcaatgggaaaatggagcacctaacaacgcatcttcacagaccaagaactacgagatcaccctttcattctggggtgacggagggatcgtacc
attcgagcctttttttcatgcttttcccggcggtctggagaaagcagcaatcaataggacttccctaatcctcccttcctgaaaggaagaacgtgaaattctttttccttc
cgcagggaccaggaggttggatctagccataagaggaatgcttggtataaataagccacttcttggtcttcgactccctaagtcactacgagcgccctcgatcagtg
caatgggatgtggctatttatctatctcttgactcgaaatgggagcagagcaggtttgaaaaaggatcttagagtgtctagggttgggccaggagggtctcttaacgc
cttcctttttctgcccatcggagttatttcccaaggacttgccatggtaagggggagaaggggaagaagcacacttgaagagcgcagtacaacggagagttgtatg
ctgcgttcgggaaggatgaatcgctcccgaaaaggagtctattgattctctcccaattggttggatcgtaggggcgatgatttacttcacgggcgaggtctctggttca
agtccaggatggcccagctgcgccagggaaaagaatagaagaagcatctgactctttcatgcatactccacttggctcggggggatatagctcagttggtagagct
ccgctcttgcaattgggtcgttgcgattacgggttggctgtctaattgtccaggcggtaatggtagtatcttgtacctgaaccggtggctcactttttctaagtaatgggg
aagaggactgaaacatgccactgaaagactctactgagacaaaaagatgggctgtcaaaaaggtagaggaggtaggatgggcagttggtcagatctagtatgga
tcgtacatggacgatagttggagtcggcggctctcctaggcttccctcatctgggatccctggggaagaggatcaagttggcccttgcgaatagcttgatgcactatc

tcccttcaaccctttgagcgaaatgtggcaaaaggaaggaaaatccatggaccgaccccattatctccaccccgtaggaactacgagatcaccccaaggacgcctt
cggcgtccaggggtcacggaccgaccatagaccctgttcaataagtggaacacattagccgtccgctctccggttgggcagtaagggtcggagaagggcaatca
ctcgttcttaaaaccagcattcttaagtttaagatcaaagagtcgggcggaaaaaggggagagctccccgttcctggttctcctgtagctggattccccggaaccaca
agaatccttagaatgggattccaactcagcaccttttgttttgagattttgagaagagttgctctttggagagcacagtacgatgaaagttgtaagctgtgttcgggggg
gagttattgtctatcgttggcctctatggtagaacccgtcggggaggcctgagaggcggtggtttaccctgtggcggatgtcagcggttcgagtccgcttatctccag
cccgtgaacttagcggatactatgatagcaccgaattttgccaattcggcagttcgatctatgatttcgcattcatggacgttgataagatccttccatttagtagcacctt
aggatggcatagccttaacgttaatggcgaggttcaaaagaggaaaggcttgcggtggatacctaggtacccagagacgaggaagggcgtagcaagcgacgaa
atgcttcggggagttgaaaataagcatagatccggagattcccaaataggtcaaccttttgaactgcctgctgaatccatgagcaggcaagagacaacctggcgaa
ctgaaacatcttagtagccagaggaaaagaaagcaaaagcgattcccgtagtagcggcgagcgaaatgggagcagcctaaaccgtgaaaacggggttgtggga
gagcaatacaagcgttgtgctgctaggcgaagcggttgagtgccgcaccctagatggctaaagtccagtagccgaaagcatcactagcttacgctctgacccgag
tagcatggggcacgtggaatcccgtgtgaatcagcaaggaccaccttgcaaggctaaatactcctgggtgaccgatagcgaagtagtaccgtgagggaaaggtg
aaaagaaccccccagtgggtagtgaaatagaacgtgaaaccgtgctgagctcccaagcagtgggaggggaaagtgatctctgaccgcgtgcctgttgaagaatga
gccggcgactcataggcagtggcttggttaagggaacggaacccaccggagccgtagcgaaagcgagtcttcatagggcgattgtcactgcttatggacccgaa
cctgggtgatctatccatgaccaggatgaagcttggatgaaactaagcagaggtccgaaccgactgatgttgaagaatcagcggatgagttgtggttaggggtgaa
atgccactcgaacccagagctagctggttctccccgaaatgcgttgaggcgcagcagttgactggacatctaggggtaaagcactgtttcggtgcgggctgcgcg
agcggtaccaaatcgaggcaaactctgaatactagatatgacccaaaaataacaggggtcaaggtcggccagtgagacgatgggggataagcttcatcgtcgag
agggaaacagcccggatcaccagctaaggcccctaaatgaccgctcagtgataaaggaggtgggggtgcaaagacagccaggaggtttgcctagaagcagcc
accctttaaagagtgcgtaatagctcactgatcgagcgcccttgcgctgaagatgaacggggctaagcgatctgccgaagctgtgggatgtcaaaatgcatcggta
ggggagcgttccgccttagagggaagcaaccgcgaaagcgggggtcgacgaagcggaagcgagaatgtcggcttgagtaacgaaaacattggtgagaatcca
atgccccgaaaacccaaggtttcctccgcaaggttcgtccacggagggtgagtcagggcctaagatcaggccgaaaggcgtagtcgatggacaacaggtcaata
ttcctgtactaccccttgttggtacggagggacggaggaggctaggttagccgaaagatggttataggtttaaggacacaaggtgaccctgctttttcagggtaaga
aggggtagagaaaatgcctcgagccgaggtccgagtaccaagcgctgcagcgctgaagtatgagccccgtggactagccattgcttctccacgaggctcatacc
aggcgctacggcgctgaagtatgtaacccatgccatactcccaggaaaagctcgaacgaccttcaacaaaggggtacctgtacccgaaaccgacacaggtgggt
aggtagagaatacctaggggcgcgagacaactctctctaaggaactcggcaaaatagccccgtaacttcgggagaaggggtgccccctcgcaaaagggggtcg
cagtgaccaggcccgggcgactgtttaccaaaaacacaggtctccgcaaagtcgtaagaccatgtatgggggctgacgcctgcccagtgccggaaggtcaagg
aagttggtgaactgatgacagggaagccggcgaccgaagccccggtgaacggcggccgtaactataacggtcctaaggtagcgaaattccttgtcgggtaagtt
ccgacccgcacgaaaggcgtaacgatctgggcactgtctcggagagagactcggtgaaatagacatgtctgtgaagatgcggactacctgcacctggacagaaa
gaccctatgaagctttactgttccctgggattggctttgggcctttcctgcgcagcttaggtggaaggcgaagaaggccccccttccgggggggcccgagccatcag
tgagataccactctggaagagctcggattctaaccttgtgtcagacccgcgggccaagggacagtctcaggtagacagtttctatggggcgtaggcctcccaaaa
ggtaacggaggcgtgcaaaggtttcctcgggccagacggacattggtcctcgagtgcaaaggcagaagggagcttgactgcaagactcacccgtcgagcagag
acgaaagtcggccttagtgatccgacggtgccgagtggaagggccgtcgctcaacggataaaagttactctagggataacaggctgatcttccccaagagtccac
atcgacgggaaggtttggcacctcgatgtcggctcttcgccacctggagctgtaggtggttccaagggttgggctgttcgcccattaatgcggtacgtgagctgggt
tcagaacgtcgtgagacagttcggtccatatccggtgtgggcgttagagcattgagaggacctttccctagtacgagaggaccgggaaggacgcacctctggtgt
accagttatcgtgcctacggtaaacgctgggtagccaagtgcggagaggataactgctgaaagcatataagtagtaagcccaccccaagatgagtgctctctcctc
cgacttccctagagcctccggtagcacagccgagacagcgacgggttctccacccatacggggatggagcgacagaagcatggaaataggataaggtagcgg
cgagacgagccgtttaaataggtgtcaagtggaagtgcagtgatgtatgcagctgaggcatcctaacgaacgaacgatttgaaccttgttcctacacgacctgatca
aatcgatcaggcacttgccatctatcttcattgttcaactctttgatgaaaagatgaaaaaaccaaaaaaaagctctgcccttccatctcttggatagatagagaggga
gggcagaggcctttggtgtcccttccagtcaagaattggggcttcacaattactagccaatatttctctcatgcctttcctcgttcatggttcgatattctggtgtcctagg
cgtagaggaaccacaccaatccatcccgaatttggtggttaaactctactgcggtgacgatactgtaggggaggtcctgcggcaaaatagctcgatgccagaatga
taaaaagcttaacacctcttatttgactttttcactattttgaaatacgaaaaagatccaaatccaaaatgcaaaggtcgtcttattcaaaacctcaatcatcacatcccctc
tctcccacttcacacctcggaacgcactgttcttatagagagaaaggggctttcccatcttcttaacccgaaatgaaatggctgaggagagggaggttccttttgggg
ggtacccccgggaagagatccagtggagacggggtgggcctgtagctcagaggattagagcacgtggctacgaaccacggtgtcgggggttcgaatccctcct
cgcccacagccttccaaaggggaagggcctttactttccccctgagggtaggaaaaccatgatcgggatagcggacgtaaagctattgaacttgggtatgctctttc
cttttgtcgaagtggaatcgtagaacagaatgtgatacgatgagataaaatgcaatagaaacaaggatagcgaacgggttacctactcctaagggtcaaagcaagc
cctttaattcaattctttattcttacattaaagaatgaatcaaatctccccaagtaggattcgaacctacgaccagtcagttaacagccgaccgctctaccactgagctac
tgaggaacaagggggattcgacctcctagagttcaactcccgctctcaacccatgaacaatatgagtccgaagcttctttcgtaactcccggaatttcttcgtagtggc
tccgttccatgcctcatttcataggtaagcccagagtggctctatttcattctatttcacttcctagcacttcctatcatttaatatccatccctttggtcttattgacataagag
atgtcatttatagtctatctctttctatatatggaaagtcaagaaattctcatcgaaacatcgagaaattgtgcatatagaaaactctaaagaaagaaaaaaaggagacc
catgccatgattttcaaatcttttctacttagtagtctaagtttctcgatgaggataattaattcggtcgttgcggtcggactctattatgggtttctgaccacattctccatgg
gtccctcttagatcttctttctccaatcttggattagggaagaaggagatattcgcgactcctggtggtttcattatggggcagctcatgatcttcatatcgatctattatcc
acctctgcatctattctttcttagctaaacgggtggaagatccatccaatttggttatatcatggactcaaaaaacggatctgaatgtgactgaaatgcacgatcttcaca
ggtatcacttttcacgatacctaaaaggtggaatagcgattttcgaaccatttcctataagagaaaggtttccattactttgagaaatggattctatatcaaactatagctat
tgcattaaagaagaaaagaaactaatagaagtcgaagacgcggaatggtagtgaatagagagaaagattcttctggttttcttgttcctgaaaatattctatctatctcct
agacgccgtagagaattgagaattttcatgtctttcaattctcgtactcgtaattggaaagttacggaaggagatccatcattttgcaatgaaaactacataaaaaactct
ggacaatttcgaaatcaggccaagcgtcttaatacatatgcaaaaaaattcattattggcccaccattgattagaagatttaacttgtatgaatcgctattggtttgatacg
aataatggcagttgtttcagtatgttaaggatacagatgtatccacaattcatttagagttacttaatagcctatttcttataccatatctctatcccgtgaaattctcgagcc

gaaagatggatgcatatgctatgtttcattttgctaaatgatatcaattaaacggtgtatcaattccataaattggatatagcaataaataaatcagcaaaattcttttatttta
gatagaagaaaagtttcttctatctaaaataaaagaatgtacccttctatccaaatccaatttgcatcgataaaataaatccaaattccagtagtagatgaataattgcaaa
tttttgtgtgtacgagattagaataacttcaaaataactgacataatttttttatttttcctgatcagaaaaatacatgaaaaagaaaggaggtagaaaaatttttggatttatg
gttaaagaagaaaaagaagaaaactggggttctgttgaatttcaagtattcagtttcaccaataagatacggagacttgcttcacatttggaattacacaaaaaagattt
ttcatcggaaagaggtctccgaagacttttgggaaaacgtcaacgtttgctggcttatttggcaaagaaaaatagagtacgttataagaaattaatcagtcagttggat
attagggagcggtaatttcatcgttcgaatttttttcttattttattagtagtcttatagtagtcttagattttgcattttgatgagcctcgttttgaggaattcatggaataatcc
attttcatggaataatgaattaaggaagaaaggatatgagtctaccgcttacaagaaaagatctcatgatagtcaatatgggccctcagcacccatcaatgcatggtgt
tcttcgactgatcgttactctcgatggtgaagatgttattgattgtgaacccatattaggctatttacacagaggaatggaaaaaatcggcaaataggaaagctacttag
gcaggagataggggaattccttaagaaagaaaaaagaataagaacacagatacataacataaaaaaaagaataaataagacgaaattcgccctcccccctacatatt
taatttcttctcctatacaaaaactagcaagacctactccattggtaattccatcaatgacacccttgtcgaaaaactgcgttagttcggttaatcctcttatacccaaggt
aaaggtcctagtatagaaaatatctatataaccgcgattatatgaccaactatatatcttttttttagttgatggaaaaaatactttttcggacccccttttacaaaggaattt
attaaatccaaattctgaaaaaaagagtaagcagatccataaaacatatatgctatgaatagaccaaaaatagctagacttacagaagaaattgcattagtgataaatt
catatgaatttatggaagaattagaactttcttggaaaaagttgattgagggagttagccactttgataatatggttaattcccctatttcattatcaaaatggattcctatag
atccaataaacaaagtacaaagcagtaatataagaagaggaaatagcatagtatttcccggttcatgaggatagacaaaagtgtttttagtccccaatgaagtactaa
aggaccctatcctatttcctgtattaccatgaattttggatagattttgtgaaaaaaaagaaactccactcttcgctgttgataaaacgaaatctctattgactcctttagata
tcctttttccccataacgatattgaatacaacgaatcctctttagtactactgtaattttgaaaatgaacacgcaaatacccatcaaaagtaagtaaatatatccgaaacat
ataaaacgcagttaatcctgcagtaaaagaagctattattccaaaaaagggtgaatataaccaactattactaaggatttcatctttggaccagaagcaagcaagagg
tggaataccacaaagagaaagggtaccccataaaaaacaagttcttgtaattggaatgtattttcttaaaccacccataagaaccatattctgacttttatctggtgaata
tccaacaagaggttccattgaatgaataacagatccggatcccaagaataataaagctttcgaataagcatgagtgatcaaatggaataaagcagcttgataagaac
ctatacctagagctaacatcatataacccaattgagacattgtagaataggctaagcttcttttaatatctctctgagcaagagctaaagtggctcctaagaaaagtgtta
gtgtacctattaaagaaatgaaactcattatcaaaggtagggatatgaaaagaggaagaagtcgagctataagaaaaatccccgcagcaaccatagttgctgcgtgt
ataagagctgaaatgggggtgggtccttccatagcatcgggtaaccatacgtgaagagggaattgtgccgatttcgcaactgcaccaaggaataataaaaaagca
cacaaaatagtaagcaaggagttaatctcattattaggaatccagttattagctattttaaacaaatcccgaaactctaaactacctgttatccaaaaaaaacctagaatt
cctaataacagaccaaaatcccctacacgattagttacaaaagctttttgacaagcactcgctgcaattggccgtgtaaaccaaaagcctatcaataaataggaacac
attcccacaagctcccaaaaaaaataaatttgtatcaaattggaactagtaaccaatcccaacatggaagtattgaaaaaacttatataaacaaaaaatctcaaatatcc
ctcatcgtgagacatataatcatcactataaataagaaccaggattcctacagtagtaattagtattaacataatagaagtaagcgggtcgattaagtatccaaattctaa
ggaaaaatcattattgacggtccaagaccatagatattgatagatagaacttccatttatttgttgaatagacaggtgaactgagaataccatagctatacttaaaagtaa
aacactaggaaaagcccatatgcgacgaagatttttttgttgctgttggaacaagaaaaagtccaaaccccattgacataataactggaagtgggagaagagggatt
acccatgcatattgatatgtatgttccataagaaaagaaattgcaatttttacttgaaaattttacttcaatttttctataaaattgaaaaaagtttccgattcaccaaactaatt
cttatctatttctgaaggaataaaaaaatactagaattcttaatttttcaaaaattttctcattgaaacaatcaaaaaataagaataggttttgttggttaaagtcaaaaagtta
atgaaataacttcgttacctagttattacctaaagaaggacttttattaaaatacaaaaaaaagattgaatcattttactttaatattttttgtattaaaatgaagcagctccct
tgtttcgtaactcaaattgattggaattcaattctggaatactttaattaactatttgattgaattttcccttccttttatcctcccgtcttatatggggggataggcccccatacct
tatatctgtatatggagagtatacttgaaatataaattgatttaaatagaaaacctttgtatatattctatattattaaaacaaagtctaaaaaaaatatataatatgttaaaaa
actcttgtcttatccgcattagacaaaatgaagtaaaaaagaattcagaatttcaatatcttttagtatctaagtataaatactaagaaaaagaagaaagatggattgattt
gcggcaatagatgtctttcacatacaactagaaaaaagtaatttcctttttgaatggcagttccaaaaaaacgtacttcgatgtcaaaaaagcgtattcgtaaaaatcttt
ggaagaaaaagacttatttttccatagtacaatcttattctttagcaaaatcaagatcattttctggcgtcagcgagcatccaaaaccaaagggtttttctcggcaacaaa
caaacaaataatagggttttgggataatatgaattgacctatccccaaaaaattccaattatttaatatgaataattaggaataattaggattaattaatgagtttacttttat
gtgtcgaattcctcggtacaatattcttagaacaaacctctctgatatataaaaaaagggtttttggtatactgtgacctaaatattcttttcctatcaatgaacttttcgtaat
agaatccgtataataaataaaaaaaggggattctattatgaaaagtagagtattcctgcaataagacttacaacttctacctatcttatcctaaattaacaaaaaaattagt
tctatattgcaactgagaaaaaattgttccaactctttcaaagtttctattgggcaaagcaagaattttttttgttaaaaaattcgcaacgatactaccaaacgaagtctatttt
aatgaagattctaatgtcctaaattctatggaatcttccaatctcgacgattcgcgagaaaataacttaatattcttttaataaacctgttatttcaacttagccgccatggtg
aaattggtagacacgctgctcttaggaagcagtgctcaagcatctcggttcgagtccgagtggcggcagtctcgaaaaagaatacaatagattataaaataaaatgg
attcaattcgaaatttccaattttgtaatgggaccttctccttatgctatttgcaactttagaacatatactaactcatatttctttctcaacaatttcaattgtgattacgattcat
ttaataaccttattagttcgtgaacttgggggattgcgtgattcgtcagaaaaaggaatgatagccactttttttctgtataacaggattcttagtttctcgttgggcttcttcg
ggacattttccattaagtaatttatatgagtcattgatcttcctttcatgggctctgtatattcttcatatgattcctaagatacagaactctaaaaatgatttaagcacaataa
ctacgccgagtactattttaacgcaaggctttgccacgtcgggtcttttaactgaaatgcatcaatccacaatactagtacctgctctacaatctcagtggttaatgatgc
atgtcagtatgatgttactaagctatgcgactcttttgtgcggatccttattatccgccgctcttctaatgattagatttcgaaagaatttagatttcttttcgaaaaagaaga
aaaatgttttgcttaaaacatttttctttaatgagattgaatatttctatgcaaaaagaagtgctttaaaaagcaccttttttcctttatttccaaattattacaaatatcaattaatt
gagcgtttggattcttggagttatcgtgtcattagtctagggtttacccttttaaccataggtattctttgtggagcagtatgggctaatgaggcgtggggatcctactgg
aattgggatcctaaagaaacttgggcatttattacttggaccatatttgcaatttatttacatagtagaacaaatccaaattggaagggtacgaagtcagcatttgtagctt
ccataggatttcttataatttggatctgttattttggtatcaatctattaggaataggtttacatagttatggttcatttacattacccatctaaatgattacataacataaaacct
taatgaaatggaaaaaacttccattttgtgtttgatttgagaaccccttgaacgccttctcaaagggttctcaaaaattcgagatagatctaattagactcttttactttttttc
tgaatttttttagtatttccactatggaatatagagcggactagtagaagaaaaaaaatcctatttaggataataattggataacagagcctctaccctgtcaacggatag
cgagagaacaaaatctggataaataccgattcctattactggtaaaaagatacagattaaaagaaagagttctcgcgggccggaatcctcaaaattttcgtttggaac
atgaaatagcttgtatccatagaacatctgtcgtaacatagataataaataaataggagttaatatcattccaattgccattacaaaagtaattagcatttttggcattaaca
gaaattttggactagtaattagtccaaaaaatactactaattccgcaacaaaaccactcattcctggtaaggcaagagaagccattgaaaagctactaaacatggtaa

aaatttttggcattgggatagaaacccctcccagttcttcgagataaacaaggcgcattctatcacaagccgttcccgctaagaaaaaaagtgtagccccaataaatc
catgggataatatttgtaaaatagctccattgagtccaatgttggttatggaaccaattcctataataatgaaacccatgtgagagacggaggagtaggctattcttttttt
gaaattgcgttggccaagagaagttgaagctgcatagattatttgcatcgctcctattattactaaccaaggggaaaatagataatgagcatgaggtaacaattccata
ttgatccgaatcaatccgtatgctcccatctttaataggattcccgctaaaagcatacatgtactgtaatgcgcttccccatgggtatctggtaaccacgtatgtaggggt
ataatcggcaatttgacagcataagcaataaggaagccaaaataaaatagtatttccaatgttgcagggtatgattgattaattaatctttccaaatctaatcttggttcgtt
ggaaccgtataagcccatacctagaactccgattaagaaaaaaatggaaccacctgcagtatacaaaataaactttgtagctgaatagagacgcctcttcccccccc
acatggataaaagtaagtaaacaggaattaattctaactcccacatgataaaaaaaagtaaaaggtctcgcgaagaaaataatcctatttgaccgctatacattgctag
catcaggaaatagaataatcgggaattccgggtaccggccaagctgctaaagtagctaaagtagtcataaatcctgtcaataaaatagatcctaatgaaagtccat
cgattcccaatctccagtggaaattgaagacatctatccatttagaatcctcttttaattggattaagggatcctccaattggaaatgataacagaatgcataagtcatta
gaaggaattctaataaacaaatagacatagtataccacctaacgattttgtttcccctatgaggtaaaaagaaaattaatgaacccgcaaatatcggcaaaacaacaa
gtattgttaaccaaggaaaagaactcatgataaagtgataaagacaagatacgttttgaccagaaaagcccgtgctcgattattttgagcacaggcttcttcggtaaag
aggaatcagacgattcaagtggaatttttttgtaacgtatcaataagatagagccatgctgcgggttgtctcaggtcctaaataaacgcggacgcttaaaaaatctgttg
ggcaggcggattcgcatctcttacaacccacacaatcttcggttcttggcgcggaagcaatttgcttggctttacatccatcccaaggtatcatttctaatacatctgttg
gacaagctcgtacacattgagtgcatcctatacatgtatcataaatttttacggaatgtgacattggatctataaattttccttttcaacataaaaattttcgatctggtcaaa
atgaaattagtactatatcaatcaaatgtattgtagacaccagacgaagcaatggtttatccaaacttcaacaaataatgcaatatatttcttaatccgtttgtgagaaagc
atgaaaagagccaagagacttgaattttgggcttcaacaatcataattatacgaattgtatatacgaattcgaattagccaataaattggctatcgtcttttcaatataaatt
attgcaatattcaaattgcaatatcaatgaattgcaaaaattcaactaagtaaaaaagaatactatggaataacctactcaaaaaatagatattctcaaataataaatagta
ttcatgttaatatttcatcaaataataaatagtattcatgttaatatttcatattattattatatgtgtccctttgtttagaagattctatgtctaattattcaaaaaattagattgattg
atacgagttgatttcctattacgatggatggaagaaagaatggataatccaatagctgcttcagcagccgcaagggctataacaaaaattgcgaaaatgtctcctttta
attggcggctatcaaatagatcagaaaatgttacgagatttagattaattgaattcagtataagttcaaggcatattagagctctaaccatgtttcggcttgtgatcaatcc
atagataccaatcgaaaataaatagacactcaaaaaaagtacatgctcaaacatcattaactaactccttatcaatctcgattcatttcaatatggggacaagaattgaa
ccgattgaattaattagaatagaacaattacacaacaaaagagaaaagaaggtatttgttggcagtagatgggttttactaaatcaaaattgtggttctttagtgatttattt
tagatttgaaattcttataattttgactcattctaagtatttcttattgccgagccatagtaattgcacctattaaagaaactagaagaattatggaaatgagttcaaatggaa
gataaaaatcggttgctaaatgaatcccaatttgttgaacgttatttatgagaccctgttctactatttggtttgatcttgtagtccaaagaattccataccatgacgtatctg
ggatagtagtcattagtgaaaaaggaatagttatacaaacgagtgaagtgaacccatctccaatagtccaaaaattcttatctttagaccattctgagccatttacgaac
attacggcaaatatgatcaagacatttatagctcccacataaataagaagttgtgccacagctacaaagtaggaattcaataaaatatagaataaggatatacaaacaa
gaactaatcctagcgaaaaagcagaaaaagttgggttggtaagtaataccacccctagacccccctagtagaagaacaaatcccccaaatagcacaagaatttcatg
tattggcccaggtaaatccattatggataagaagaaattaatagtataaatttttttcatgaactgactaaaactaaaagattcaaggaagaaaaaagggattaggaaatt
tttttgtatattgtatataagttctttctatagttagaaatcacatcacgaaaatctactctggtttaaaatcaggaataatttgcaataagcagtaggtattcgtttttctttcta
gttagtaaagaacttttggattctaacaaaaaaattctagtaatcagtaatcgttcttgaattccaagatttttcttcgtctattttactttgagttgaattcctaattgtttgaatt
gtgtaatctcccattatggagattggtaaccgactcaaagcaatttgattgtaattcaattcatgacgatcataagtagaaagttcatattcttcagtcattgataaacagtt
tgtcggacagtactcaacacaattaccacaaaatatacaaactccgaaatcaatactataattaagcaattgtttccttttaatatccttttcaaatctccaatccacaaga
ggtagatctatagggcatacgcgaacacatacttcacaagcaatacatttatcaaattcaaagtggattcgcccccggaaacgctccgatgtaattgatttttcatagg
ggtagtgaatcgttataggtaaacgatttgtgtgggataaggtaattatgaaactttgaccaatgtaccttgctgcgcgtattgtttgttgaccataactcatgaacccagt
taccatagggaacatattcgaaatatctatgaaaaaggtatgtttctttctcttgtttgagaggacttttgtgttgaaaatattcttactgttattgtattatcttattttatagtga
aacaagttgggaagaagttgttaataagagattgcccagggaaataggtaaaagaaatttccatccaagatttaataactgatccattctcatcctgggtaaagtccat
cttattgtgatagaaatgaagagaaataaataagctttagttaatgtaataaagatactcattgtcatttctaaaattccaaccattttattcatttggaaaaatccaaaaaag
gatatatagggaatagacaaattccacccgcctaagtagagaactgttacaaataaagaggaaactaataaatttaggtaagaaacaagataaaataaaccatatttg
ataccagaatattcggtttgataacctgctactaattcttcctccgcttctggtaaatcaaagggtaatctttcacattccgccaaagaagaaattagaaaaaccagaaa
acctatgggctgacgccaaagattccatccaaaaaaaccatattttgactgtgcttcaactatatcaactgtacttgaactgttggataatcatagtcgacgataacatca
cagttcccaccgctattccaaaaccgtacatgaaaccttagtttcatacggctcctctatgatcagaaaaaaggaaagtactgtttcatttcgttattatcttcttgggcgt
agttagaattatctaagataaaatcgatttcaacgtcctaaattagaccaaaggaattctgtctgctagaataataaaaaacgcttcggaattcatctcatcctttataatat
aaaggtactttttctttgttcagcaataacttaatcttggaataaaacactcgttataacaattaataaacgaaaagagttgggtattagttcatgaagaattctgtatgaat
atggataaacgacggaaagaataaataaagatcttttttttgtattgcattccatatcttttgtcctattcttctttccccgaggggtatttaaaaagaaaaaaggaataaa
gggttaattcgttcttgatagccatttccttaacaagtgaatgggaacatactctggatcggaatccgaagaaagtactacttgctcatttccaccaatttcaagtccttat
tatgattccttttatgaggaaaaatatctaatgctttagattccctcattactaatcctttatgtactttagtgtttctaatccctcactaacttttgatggattcccttatgattaca
actttctgtatcgggaatcccttattattgcccgcttcaagatatgatgactaatcaaaaaatctcaaccttggggtaaagaatttacaccgcttatgtttacttccatttttttc
ttgtacataggaaatgagatttttttcttttttactacaaattaataagcagttttgtttcactcatatagctatctagtttaacttactaacctgaatatagaataagaaaaggag
gataaatattcaatgaatttcagaggaaaaagatcctattttaacgaatcgcacgtagagatattgctagtacacaaaaagttaatgggatttcataactaatagattgag
cggcagctcgtagaccacctgaaaaagaatatttattatttgagctatatcctgccataagaagaccaataggagcaatacttgaaatggcaatccataaaaaaacac
caatactaagatccgctaaaacaaagcgatatcccaaaggaataactaaaaaacttaataaaattgatatgactgctatagacggtccaatgctaaataagggaatat
cccctcgggatggcaagatatcctctttaaaaagtagcttagttccatctgctatagcttgaagcagtcccagggggccagcatattcaggaccaatacgttgttgtat
cgatgcggatatttctctttctaaccacacaattacgagtacttctattgtgattcccagtaagagggtcaaaatgggtagaatccatatcagtccatagacttcttttaata
attccaagttcgaaaaagaattgatagcttctacctgtaccctgtctattatcatttcaacgatcaacttctcccataatgatatctatactacctaatatcgtcatgatatca
gccaatttcatttttttgactagctgaggaagaatttgcaaattaataaaaccgggtggacgaattttccatctccaggggaaaagactatcatctcctaccagataaatt
cctaattcaccttttggggcttccactcttgcataaagctcttgttttgacaattcaaaattgggtgaaggtttttaccaagaaatcgatattcaaaatcattccattcggaa

ttctttgctttcttaaagcgtcggacttctaaattctcataagggccccccggaattttttctacagcctgttgaataattttgattgattccctcatttcaccgattcgtactaa
atagcgtgctaatgaatccccttctttttgccattggactttccaatcgaattgattgtaagactcgtaaagatcaactttacgaagatcccattgtattccagaagctcgta
acatggggcccgataagccccaatttacagcttcttctccgctaataaaaccaactccctcaactcgttccaaaaaaatgggattctgtgtaataagttgttgatattcaa
taactcctcgtaaaaaataatcacagaaatctaaacatttatcgatccatccataaggcagatcggcagcaactcctccgatgcgaaagtaattatgcatcattcgcat
acctgtagcagcttcaaatagatcatatattaattctctctctctaaaaatataaaaaaaaggagtctgcgcaccgagatctgccataaaaggtccaagccataacaag
tgagaagctatacggctcaattctaacataattaccctaatatagctggctctttggggtatttgaatattctccaagaattctggtgcatttaccgttattgcttctgtaaac
atagtagctaaataatcccaacgtgttacataaggtaagtattgtataatagttcggttttccgcgatttttttccattcctctgtgtaaatagcctaatatgggttcacaatca
ataacatcttcaccatcgagagtaacgatcagtcgaagaacaccatgcattgatgggtgctgagggcccatattgactatcatgagatcttttcttgtaagcggtagac
tcatatcctttcttccttaattcattattccatgaaaatggattattccatgaattcctcaaaacgaggctcatcaaaatgcaaaatctaagactactataagactactaataa
aataagaaaaaaaattcgaacgatgaaattaccgctccctaatatccaactgactgattaatttcttataacgtactctatttttctttgccaaataagccagcaaacgttg
acgttttcccaaaagtcttcggagacctctttccgatgaaaaatctttttttgtgtaattccaaatgtgaagcaagtctccgtatcttattggtgaaactgaatacttgaaattc
aacagaaccccagttttcttctttttcttctttaaccataaatccaaaaatttttctacctcctttctttttcatgtatttttctgatcaggaaaaataaaaaaattatgtcagttatttt
gaagttattctaatctcgtacacacaaaaaatttgcaattattcatctactactggaatttggatttattttatcgatgcaaattggatttggatagaagggtacattcttttatttt
agatagaagaaaagtttcttctatctaaaataaaagaattttgctgatttatttattgctatatccaatttatggaattgatacaccgtttaattgatatcatttagcaaaatgaa
acatagcatatgcatccatctttcggctcgagaatttcacgggatagagatatggtataagaaataggctattaagtaactctaaatgaattgtggatacatctgtatcct
taacatactgaaacaactgccattattcgtatcaaaccaatagcgattcatacaagttaaatcttctaatcaatggtgggccaataatgaatttttttgcatatgtattaaga
cgcttggcctgatttcgaaattgtccagagttttttatgtagttttcattgcaaaatgatggatctccttccgtaactttccaattacgagtacgagaattgaaagacatgaa
aattctcaattctctacggcgtctaggagatagatagaatattttcaggaacaagaaaaccagaagaatctttctctctattcactaccattccgcgtcttcgacttctatta
gtttcttttcttctttaatgcaatagctatagtttgatatagaatccatttctcaaagtaatggaaacctttctcttataggaaatggttcgaaaatcgctattccaccttttaggt
atcgtgaaaagtgatacctgtgaagatcgtgcatttcagtcacattcagatccgtttttttgagtccatgatataaccaaattggatggatcttccacccgtttagctaaga
aagaatagatgcagaggtggataatagatcgatatgaagatcatgagctgccccataatgaaaccaccaggagtcgcgaatatctccttcttccctaatccaagattg
gagaaagaagatctaagagggacccatggagaatgtggtcagaaacccataatagagtccgaccgcaacgaccgaattaattatcctcatcgagaaacttagact
actaagtagaaaagatttgaaaatcatggcatgggtctcctttttttctttctttagagttttctatatgcacaatttctcgatgtttcgatgagaatttcttgactttccatatata
gaaagagatagactataaatgacatctcttatgtcaataagaccaaagggatggatattaaatgataggaagtgctaggaagtgaaatagaatgaaatagagccact
ctgggcttacctatgaaatgaggcatggaacggagccactacgaagaaattccgggagttacgaaagaagcttcggactcatattgttcatgggttgagagcggga
gttgaactctaggaggtcgaatccccccttgttcctcagtagctcagtggtagagcggtcggctgttaactgactggtcgtaggttcgaatcctacttggggagatttga
ttcattctttaatgtaagaataaagaattgaattaaagggcttgctttgacccttaggagtaggtaacccgttcgctatccttgtttctattgcattttatctcatcgtatcacat
tctgttctacgattccacttcgacaaaaggaaagagcatacccaagttcaatagctttacgtccgctatcccgatcatggttttcctaccctcagggggaaagtaaagg
cccttcccctttggaaggctgtgggcgaggagggattcgaaccccccgacaccgtggttcgtagccacgtgctctaatcctctgagctacaggcccaccccgtctcc
actggatctcttcccgggggtaccccccaaaaggaacctccctctcctcagccatttcatttcgggttaagaagatgggaaagcccctttctctctataagaacagtg
cgttccgaggtgtgaagtgggagagaggggatgtgatgattgaggttttgaataagacgacctttgcattttggatttggatctttttcgtatttcaaaatagtgaaaaag
tcaaataagaggtgttaagctttttatcattctggcatcgagctattttgccgcaggacctcccctacagtatcgtcaccgcagtagagtttaaccaccaaattcgggat
ggattggtgtggttcctctacgcctaggacaccagaatatcgaaccatgaacgaggaaaggcatgagagaaatattggctagtaattgtgaagccccaattcttgac
tggaagggacaccaaaggcctctgccctccctctctatctatccaagagatggaagggcagagcttttttttggtttttttcatcttttcatcaaagagttgaacaatgaag
atagatggcaagtgcctgatcgatttgatcaggtcgtgtaggaacaaggttcaaatcgttcgttcgttaggatgcctcagctgcatacatcactgcacttccacttgac
acctatttaaacggctcgtctcgccgctaccttatcctatttccatgcttctgtcgctccatccccgtatgggtggagaacccgtcgctgtctcggctgtgctaccggag
gctctagggaagtcggaggagagagcactcatcttggggtgggcttactacttatatgctttcagcagttatcctctccgcacttggctacccagcgtttaccgtaggc
acgataactggtacaccagaggtgcgtccttcccggtcctctcgtactagggaaaggtcctctcaatgctctaacgcccacaccggatatggaccgaactgtctcac
gacgttctgaacccagctcacgtaccgcattaatgggcgaacagcccaacccttggaaccacctacagctccaggtggcgaagagccgacatcgaggtgccaaa
ccttcccgtcgatgtggactcttggggaagatcagcctgttatccctagagtaacttttatccgttgagcgacggcccttccactcggcaccgtcggatcactaaggc
cgactttcgtctctgctcgacgggtgagtcttgcagtcaagctcccttctgcctttgcactcgaggaccaatgtccgtctggcccgaggaaacctttgcacgcctccgt
taccttttgggaggcctacgccccatagaaactgtctacctgagactgtcccttggcccgcgggtctgacacaaggttagaatccgagctcttccagagtggtatctc
actgatggctcgggcccccccggaagggggccttcttcgccttccacctaagctgcgcaggaaaggcccaaagccaatcccagggaacagtaaagcttcatag
ggtctttctgtccaggtgcaggtagtccgcatcttcacagacatgtctatttcaccgagtctctctccgagacagtgcccagatcgttacgcctttcgtgcgggtcgga
acttacccgacaaggaatttcgctaccttaggaccgttatagttacggccgccgttcaccggggcttcggtcgccggcttccctgtcatcagttcaccaacttccttga
ccttccggcactgggcaggcgtcagcccccatacatggtcttacgactttgcggagacctgtgtttttggtaaacagtcgcccgggcctggtcactgcgacccccttt
tgcgagggggcaccccttctcccgaagttacggggctattttgccgagttccttagagagagttgtctcgcgccccctaggtattctctacctacccacctgtgtcggtt
tcgggtacaggtacccctttgttgaaggtcgttcgagcttttcctgggagtatggcatgggttacatacttcagcgccgtagcgcctggtatgagcctcgtggagaag
caatggctagtccacggggctcatacttcagcgctgcagcgcttggtactcggacctcggctcgaggcattttctctaccccttcttaccctgaaaaagcagggtcac
cttgtgtccttaaacctataaccatctttcggctaacctagcctcctccgtccctccgtaccaacaaggggtagtacaggaatattgacctgttgtccatcgactacgcc
tttcggcctgatcttaggccctgactcaccctccgtggacgaaccttgcggaggaaaccttgggttttcggggcattggattctcaccaatgttttcgttactcaagccg
acattctcgcttccgcttcgtcgaccccgctttcgcggttgcttccctctaaggcggaacgctcccctaccgatgcattttgacatcccacagcttcggcagatcgctt
agccccgttcatcttcagcgcaagggcgctcgatcagtgagctattacgcactctttaaagggtggctgcttctaggcaaacctcctggctgtctttgcaccccccacc
tcctttatcactgagcggtcatttaggggccttagctggtgatccgggctgtttccctctcgacgatgaagcttatcccccatcgtctcactggccgaccttgacccctg
ttatttttgggtcatatctagtattcagagtttgcctcgatttggtaccgctcgcgcagcccgcaccgaaacagtgctttacccctagatgtccagtcaactgctgcgcct
caacgcatttcggggagaaccagctagctctgggttcgagtggcatttcacccctaaccacaactcatccgctgattcttcaacatcagtcggttcggacctctgctta

gtttcatccaagcttcatcctggtcatggatagatcacccaggttcgggtccataagcagtgacaatcgccctatgaagactcgctttcgctacggctccggtgggttc
cgttcccttaaccaagccactgcctatgagtcgccggctcattcttcaacaggcacgcggtcagagatcactttcccctcccactgcttgggagctcagcacggtttc
acgttctatttcactacccactgggggttcttttcacctttccctcacggtactacttcgctatcggtcacccaggagtatttagccttgcaaggtggtccttgctgattcac
acgggattccacgtgccccatgctactcgggtcagagcgtaagctagtgatgctttcggctactggactttagccatctagggtgcggcactcaaccgcttcgccta
gcagcacaacgcttgtattgctctcccacaaccccgttttcacggtttaggctgctcccatttcgctcgccgctactacgggaatcgcttttgctttcttttcctctggcta
ctaagatgtttcagttcgccaggttgtctcttgcctgctcatggattcagcaggcagttcaaaaggttgacctatttgggaatctccggatctatgcttattttcaactccc
cgaagcatttcgtcgcttgctacgcccttcctcgtctctgggtacctaggtatccaccgcaagcctttcctcttttgaacctcgccattaacgttaaggctatgccatcct
aaggtgctactaaatggaaggatcttatcaacgtccatgaatgcgaaatcatagatcgaactgccgaattggcaaaattcggtgctatcatagtatccgctaagttcac
gggctggagataagcggactcgaaccgctgacatccgccacagggtaaaccaccgcctctcaggcctccccgacgggttctaccatagaggccaacgatagac
aataactccccccgaacacagcttacaactttcatcgtactgtgctctccaaagagcaactcttctcaaaatctcaaaacaaaaggtgctgagttggaatcccattcta
aggattcttgtggttccggggaatccagctacaggagaaccaggaacggggagctctccccttttccgcccgactctttgatcttaaacttaagaatgctggttttaa
gaacgagtgattgcccttctccgacccttactgcccaaccggagagcggacggctaatgtgttccacttattgaacagggtctatggtcggtccgtgacccctggac
gccgaaggcgtccttggggtgatctcgtagttcctacggggtggagataatggggtcggtccatggattttccttccttttgccacatttcgctcaaagggttgaaggg
agatagtgcatcaagctattcgcaagggccaacttgatcctcttccccagggatcccagatgagggaagcctaggagagccgccgactccaactatcgtccatgta
cgatccatactagatctgaccaactgcccatcctacctcctctaccttttttgacagcccatctttttgtctcagtagagtctttcagtggcatgtttcagtcctcttccccatt
acttagaaaaagtgagccaccggttcaggtacaagatactaccattaccgcctggacaattagacagccaacccgtaatcgcaacgacccaattgcaagagcgga
gctctaccaactgagctatatcccccgagccaagtggagtatgcatgaaagagtcagatgcttcttctattcttttccctggcgcagctgggccatcctggacttgaa
ccagagacctcgcccgtgaagtaaatcatcgcccctacgatccaaccaattgggagagaatcaatagactccttttcgggagcgattcatccttcccgaacgcagc
atacaactctccgttgtactgcgctcttcaagtgtgcttcttccccttctccccccttaccatggcaagtccttgggaaataactccgatgggcagaaaaaggaaggcgt
taagagaccctcctggcccaaccctagacactctaagatccttttttcaaacctgctctgctcccatttcgagtcaagagatagataaatagccacatcccattgcactg
atcgagggcgctcgtagtgacttagggagtcgaagaccaagaagtggcttatttataccaagcattcctcttatggctagatccaacctcctggtccctgcggaaag
gaaaaagaatttcacgttcttcctttcaggaagggaggattagggaagtcctattgattgctgctttctccagaccgccgggaaaagcatgaaaaaaaggctcgaat
ggtacgatccctccgtcaccccagaatgaaagggtgatctcgtagttcttggtctgtgaagatgcgttgttaggtgctccattttcccattgaggacgaacctcaacct
gtgctcgagagatagctctccatacactgataagggatgtatggattctcgagaagagaggagccgtagtgccccccccggaccgcccggatcccacgagtgaa
tagaaagttagatctacatgggatctcacctgaatcgccccatctatcctcctgaggagaagtttgtttggtttcaaactccgattcaaacaggaggagtacgccatgc
taatgtgccttggatgatccacatcttcgggtcaggcgctgatgagcacattgaactatccatgtggctgagagccctcacagcccaggcacaacgacgcaattatc
aggggcgcgctctaccactgagctaatagcccgtcgcgcgggcctccctttgggaggcctgctacgccaaaagcgagaaaaactccatccctttccttttgacatc
cccatgccgccacaccacacgggggggggcatggggacgtcaaaaggggatcctatcactatcaactaatttgttccgacctaggataataagctcatgagctt
ggtcttacttcaccctaaacgaaagaagacttccatatccaagtttagctcagacgtagctgccttctttttgggcgtgaagcagtgtcaaaccaaaatacccaataag
cataagcattagctctccctgaaaaggaggtgatccagccgcaccttccagtacggctaccttgttacgacttcactccagtcgcaagcctagccttaggcatcccc
ctccttacggttaagggtaatgacttcaaacatggccagctcctatagtgtgacgggcggtgtgtacaaggcccgggaacggattcaccgccgtatggctgaccgg
cgattactagcgattcctgcttcatgcaggcgagttgcagcctgcaatccgaactgaggacgggtttttggagttagctcaccctcgcgagatcgcgaccctttgtcc
cgcccattgtagcacgtgtgtcgcccagggcataaggggcatgatgacttggcctcatcctctccttcctccggcttaacaccggcggtctgttcagggttccaaact
catagtggcaactaaacacgagggttgcgctcgttgcgagacttaacccaacaccttacggcacgagctgacgacagccatgcaccacctgtgtccgcgttcccg
agggcacccctctctttcaagaggattcgcggcatgtcaagccctggtaaggttcttcgctttgcatcgaattaaaccacatgctccaccgcttgtgcgggcccccgt
caattcctttgagtttcattcttgcgaacgtactccccaggcgggatacttaacgcgttagctacagcactgcacgggtcgagtcgcacagcacctagtatccatcgtt
tacggctaggactactggggtctctaatcccatttgctcccctagctttcgtctctcagtgtcagtgtcggcccagcagagtgctttcgccgttggtgttctttccgatctc
aatgcatttcaccgctccaccggaaattccctctgcccctaccgtactccagcttggtagtttccaccgcctgtccagggttgagccctgggatttgacggcggactt
gaaaagccacctacagacgctttacgcccaatcattccggataacgcttgcatcctctgtcttaccgcggctgctggcacagagttagccgatgcttattcctcagat
accgtcattgtttcttctccgagaaaagaagttgacgacccgtgggccttccacctccacgcggcattgctccgtcaggctttcgcccattgcggaaaattccccact
gctgcctcccgtaggagtctgggccgtgtctcagtcccagtgtggctgatcatcctctcggaccagctactgatcatcgccttggtaagctattgcctcaccaactag
ctaatcagacgcgagcccctccttgggcggatttctccttttgctcctcagcctacggggtattagcaaccgtttccagttgttgttcccctcccaagggcaggttctta
cgcgttactcacccgttcgccactggaaacaccacttcccgttcgacttgcatgtgttaagcatgccgccagcgttcatcctgagccaggatcgaactctccatgaga
ttcatagttgcattacttatagcttccttattcgtagacaaagcggattcggaattgtctttccttccaaggataacttgtatccatgcgcttcagattattagcctggagttc
gccaccagcagtatagccaaccctaccctatcacgtcaatcccacaagcctcttatccattcccgttcgatcgtggcgggggggagtaagtcaaaatagaaaaaactc
acattgggtttagggataatcaggctcgaactgatgacttccaccacgtcaaggtgacactctaccgctgagttatatcccttccccgtcccctcgagaaagagaatt
aacgaatcctaaggcaaaggggcgagaaactcaaggccacccttcctccgggctttctttccacactattatggatagtcaaataatgggaaaaattggattcaattg
tcaaccggtcctatcgaaaataggattgactatggattcgagccatagcacatggtttcataaaatctgtacgattttcccgatctaaatcgagcaggtttccatgaaga
agatcttgttcagcatgttctattcgatactggtaggagaagaacccgactcggtattcttaaaaaaagaggggaagcagaaccaagtcaagatgatatgggtcgcc
ccttcttcttgcgccaaagatcttaccatttccgaaggaactggggctacatttcttttcaatttccattcaagagtttctatctgtttccacgcccttttttgagacctcgaaa
catgaaatggacaaattccttctcttaggaacacatacaagaaaaaggataatggtagccctcccattaactacttcattttcatttatgaatttcatagtaatagaaatcc
atgtcctaccgagacagaatttcgaacttgctatcctcttgcctaataggcaaagattgacctctgtagaaagactgattcattcggatcgatatgaggacccaactac
gttgcattgccagaatccatgttccatatttgaagagggttgacctctgtgcttctctcatggtacaatcctcttcctgctgagcccccttctcctcggtccacagagaa
aaaatggaggactggtgccgacagttcatcacggaagaaagaactcacagagccgggatcgctaactaatagaatagtactactaactaatactaatatatagaaat
agatatctagaaatagaaacgaactaatatatagataatcgaaattgaaaagaactgtcttttctgtatactttccccgttctattgctaccgcgggtcttatgcaatcgat
cggatcatatagatatcccttcaacacaacataggtcatcgaaaggatctcggacgactcaccaaagcacgaaagccagttagaaaatggattcctatttgaagagt

gcctaaccgcatggataagctcacattaacccgtcaattttggatccaattcgggatttttcttgggaagtttcgggaagaaattggaatggaataatatagattcatac
agaggaaaaggttctctattgatgcaaacgctgtacctagaggatagggatagaggaagagggaaaaatcgaaatgaaataaataaagaataaagcaaaaaaaa
aaataagtcgaagatagaagagcccagattccaaatgaagaaatggaaactcgaaaaggatccttctgattctcaaagaatgaggggcaaggggattgataccga
gaaagatttcttcttattataagacgtgatttgatccgcatatgtttggtaaaagaacaatcttctcctttaatcataaatggaaagtgttcaattagaacatgaaaacgtga
ctcaattggtcttagttagtcttcgggacggagtggaagaagggcggagactctcgaacgaggaaaaggatcccttcgaaagaattgaacgaggagccgtatgag
gtgaaaatctcatgtacggttctgtagagggacaggaagggtgacttatctgtcgacttttccactatcaaccccaaaaaacccaactctgccttacgtaaagttgcca
gagtacgattaacctctggatttgaaatcactgcttatatacctggtattggccataatttacaagaacattctgtagtattagtaagaggaggaagggttaaggatttac
ccggtgtgagatatcgcattattcgaggagccctagatgctgtcgcagtaaagaatcgtcaacaagggcgttctagtgcgttgtagattcttatccaagacttgtatcat
ttgatgatgccatgtgaatcgctagaaacatgtgaagtgtatggctaacccaataacgaaagtttcgtaaggggactggagcaggctaccatgagacaaaagatctt
ctttctaaagagattcgattcggaactcttatatgtccaaggtcaatatggaaattctttcaggggttttcccttactttgtccgtgtcaacaaacaattcgaaatacctcga
ctttttcagaacaggtccgagtcaaatagcaatgattcgaagcacttctttttccattacactatttcggaaacctaaggacttgatggtatggatatggaaaatacagga
tttccgatcctagcgggaaaaggagggaaacggatactcaattttaagtgagtaaactgaattccatactcgatctcatagatccctatagaattctgtggaaagccgt
attcgatgaaagttgtatgtacggcttggagggagatctttcctatctttcgagatccaccctacaatatggggtcaaaaagccaaaaaaataagtgattcgtttttagc
ccttataaaaagaaaacggattcttgaacctctttcacgctcatgtcacgtcgaggtactgcagaaaaaagaactgcaaaatccgatccaatttttcgtaatcgattagt
taacatggtggttaaccgtattatgaaagacggaaaaaaatcattggcttatcaaattctctatcgagccgtgaaaaagattcaacaaaagacagaaacaaatccact
attggttttacgtcaagcaatacgtagagtaactcccaatataggagtaaaaacaagacgtaataaaaaaggatcgacgcggaaagttccgattgaaataggatcta
aacaaggaagagcacttgccattcgttggttattagaagcatcccaaaagcgtccgggtcgaaatatggctttcaaattaagttccgaattagtagatgctgccaaag
ggggtgggggtgccatacgcaaaaaggaagcgactcatagaatggcagaggcaaatagagctcttgcacattttcgttaatccatgaacagaatctaggtatgtag
acacatggatccatacatctcgatcggaaaagaatcaatagaaggagaatcggacgatatctttctcgaaacaaacaaaaaggaaaagaaagagaaaacagaaat
catgatcaactaagccctctcgggggcttgcttaagaataagaaagaggaatcttatggaaatagcatggaataaggtttgatcctattcatggggattccgtaaatat
cccattccaaaaatcgaaacaatcgggacttttcggagattggatgcagttactaattcatgatctggcatgtacagaatgaaaacttcattctcgattctacgagaattt
ttatgaaagcgtttcatttgcttctcttccagggaagtttcattttcccagaatgtatcctaatttttggcctaattcttcttctgatgatcgatttaacctctgatcaaaaagata
gaccttggttctatttcatctcttcaacaagtttagtaataagcataacggccctattgttccgatggagagaagaacctataattagcttttcgggaaatttccaaacgaa
caatttcaacgaaatctttcaatttcttattttattatgttcaactttatgtattcctctatccgtagagtacattgaatgtacagaaatggctataacagagtttctgttattcgta
ttaacagctactctagggggaatgtttttatgtggtgctaacgatttaataactatctttgtagctccagaatgtttcagtttatgttcctacctattgtctggatataccaaga
gagatctacggtctaatgaggctactatgaaatatttactcatgggtggggcaagctcttctattctggttcatggtttctcttggctatatggttcatctgggggggagat
cgagcttcaagaaattgtgaacggtcttatcaatacacaaatgtataactccccaggaatttcaattgcgcttatatccatcactgtaggacttgggttcaagctttcccc
agccccttttcatcaatggactcctgacgtctacgaaggagtgtggttcgttcgacaaattcctacctctatatctatctctgaggtgtttgggttttgcaaaactccatag
atatgcagaagagaaatgctatccccactccgaccaagacagaacttttaccaaaagtttattgtgatctttttgttcaaataacaattaaggtgaagcagggtcagga
acaacgaatctctttatgataaacagatccattttgcaagctcgttattacgggtagttcctacaaagaatcggactaatgacgtatacaatgcttgaattatcgacgtag
atgctacatagtgggttctcatccttcagagactacgagtgtaataggagcatccgttgacaaaaggatcaccctaagatgatcatctcatggctattgggaacgaat
caaatcagatggttctatttctcaacctttctgacttgctcctacggaaccaaggtcgaaaggattgaaaaagtcagtcattcacaaccactgatgaaggattcctcga
aaagttaaggattagtagttctttttcgaaatcgatttcgaaaaagaatggattcggtcttatacatacgcgaggaaggtaatcaaaaaagaaagaagacaagttcttct
ttcttttatcacttaggagccgtgcgagatgaaagtctcatgcacggttttgcatgagagaaagaagcgaggaatcctcttttcgactctgactcccccactccagtcgt
tgcttttctttctgttacttcgaaagttgctgcttcagcttcagccacgcgaattctcgatattcctttttatttctcatcaaacgaatggcatcttcttctggaaatcctagctat
tcttagcatgatattggggaatctccttgctattactcaaacaagcatgaaacgtatgcttgcatattcgtccatagggcaaatcggatatgtaattattggaataattgtt
ggagactcaaatgatggatatgcaagcatgataacttatatgcttttctatatctccatgaatctaggaacttttgcttgcattgtattatttggtctacgtaccggaactgat
aacattcgagattatgcaggattatacacgaaagatcctttttggctctctctttagccctatgtctcttatccctaggaggccttcctccactagcaggtttcttcggaaa
actctatctattctggtgtggatggcaagcaggcctatatttcttggtttcaataggactccttacgagcgttctttctatctactattatctaaaaatagtcaagttattaatg
actggacgaaaccaagaaataaccccttatgtgcgaaattatagaagatcccctttaagatcaaacaattccatcgaattgagtatgactgtatgtgtgatagcatctac
tataccaggaatatcaatgaaccccattcttgcaattgctcaggataccctcttttagctgctaggtctatttcttagttcaagatccctcttactaactggaataaaagaat
tagtagatctgttccgcccaaaatgggaatgggcgctagggtaatgaacttataatcatggaatcgactcgatcatcagattataagttcattccataccggaccagac
cgtgcacattcttattatgagaaggggtcattcgagcctatggaaataggatactctgtttacatagaaatccccacgtccttacattctatttaggattaggaataggtgt
aatcagacctgcttttgacatatctatcctatccttatttgggtaccatatgcacctctttgggcttctattgaatcgagaaattggattgtacatctttttgattttgatacatat
aaggtgtcctacggataatgcaaatcgaagctatttgatgtctgactcaggcctatatgaccgatcgatcgaaatactccaagactccacctttgtcatatattccatata
tcacattagatagatatcatattcatggaatacgattcactttcaagatatcacattagatagatatcatattcatggaatatgattcactttcaagatgccttgatggtgaaa
tggtagacacgcgagactcaaaatctcgtgctaaagagcgtggaggttcgagtcctcttcaaggcataatacggagaatgcgcattcaatgagcattccccgtaga
agtattccggaaatctgcgcctggcgctctcctctatcttctgaggtccttaaccacttccctgagaaaaggagacagtaaaagccaaaatagactaaatatagcctg
aacgatcctaaaaatccctcgaaggagataatataataaagaacccaaagcagacggtatcctactgcaagggtggtcttaagaatccaaaagaggttgctcagaa
gagatagatgtatcccaacctctattgctctcgcgtaaagcctttttttttacgcgacaggaaaaagtgactacgaattccccttttttgtttgcgaatccctgtttgtatccttt
gagcgcacgcccataagtagcgatcaaggaaatcgatcaaacgatcccaataccgtgaaagagaaacttcccagatccagggaagcttatcataaagggcttcga
caaggggttttattcgttctttgagcaaaaagataaagatcggatagattcgaaccgcaattgcaaaggtaatcactactatgccagcccaaatcatgatcttcaccaa
cttggatttggttctctcgcgaaaatcgcgagttgcagagatgagaaccatgaaaagcaagatcccgaataagaaaacagaaaccgaggaaaccacaagagtga
agactagtagagtctcgtcttttgtcattctttgctcctttttcactcaatgattcattcgaatttcccgaccaaaaattctatatgtctattcatagggcctcgttgctaagtgc
tacaagatctagtgcactggaactcgtggttatggacccgaatcctttagtatggaacattgtcttttccaagtaaaaacccccagtatatgaaagaatgaaaaggtgc
tttcgttcttttcttgtggaataagaagccctcgtaccttaatgaaaggaaaataggaatttttcattaggtatttgaccaaataggatcgtccagttcctatagaacctatc

actaaaatacccgatagggctaagcggaacgaaaaggattttccctgagatggtaaatgaaaacgattagccccatacgaggtttgggaataagtgatgagcaag
gaatatacgtctttctgctaaagaggatctattaaactcataattcattagatccttgttatcaatgtcaactaggtatcataagtaaacggatcccggttgttcaatcctttg
ataaccaaggtcattctttgctaaagagaaatgatcactatgagtcagactcaatagaattggatccattccaaatagcgagaattaggattctggatccctctcaatct
ctctttcaattcgaggatccagagaggtgttttcatagtcatctccgaatatttgccatctccgaatattttcgatttcatttttctatgatatgtctttctatatgaaaattggtta
tttacgatgtacgatgatccctgttaagcatccatggctgaatggttaaagcgcccaactcataattggtaaatttgcgggttcaattcctgctggatgcacgcgaacc
ggaacgttccataagtctattggaactggctctctatccatggaatctcatccatcatccatacataacgaattggtatggtatattcataccataacataagaacaataa
gaactcgaattcttatcgatactggaactcagagcataggagggaaagtcgatttatggatggaatcaaatacgcagtatttacagaaaaaagtcttcgtttattggga
aagaatcaatatacttttaatgtcgaatcgggattcactaagacagaaataaagcattgggtcgaactcttctttggtgttaaggtagtagctgtgaatagccatcgact
acccggaaagggtagaagaatgggccctattctaggacatacaatgcattacagacgtatgatcattacccttcaaccgggttattctattccacttctagatagagaa
aaaaactaaaggagaatacttaataatacggcgaaacatttatacaaaacacctatcccgagcacacgcaagggaaccatagataggcaagtgaaatccaatcca
cgaaataatttgatccatggacggcaccgttgtggtaaaggtcgtaattccagaggaatcattaccgcaaggcatagagggggaggtcataagcgcctataccgta
aaatcgattttcgacggaatcaaaaagacatatctggtagaatcgtaaccatagaatacgaccctaatcgaaatgcgtacatttgtctcatacactatggggatggtga
gaagggatatattttacatcccagaggggctataattggagatactattgtttctggtacaaaagttcctatatcaatgggaaatgccctacctttgagtgcggtttgaac
tattgatttacgtaattggaagtaaccaattaggtttacgacgaaacctagaaatcgatcactgatccaatttgactacctctacgggatagacctcaacagaaaactgt
tgagtaacggcagcaagtgattgagttcagtagttcctcatagaaaattattgactctagagatatggtaatatggagaagacaaaattgtttgaagcacgcacagaa
ccggaagcgccccttgtttcaaagagaggaggacgggttattcacatttaatttgatggtcagaggcgaattgaaagctaagcagtggtaattaagacccccgggg
gaaaatagggatgtctcctacgttacccataatatgtagaagtatcgacgtaatttcatagagtcattcgatctgaatgctacatgaagaacataagccagatgacgga
acgcggagacctaggatgtagaagatcataacatgagcgattcggcagatttggattcctttcctatatatccactcatgtggtacttcatcatacgattcatataagatc
catctgtctagagatcgtcatatacatctagaaagccgtatgctttggaagaagcttgtacagtttgggaaggggtttttgagagaaaagaagaatctacttcaaccg
atatgcccttaggcacggccatacataacatagaaatcacacgtggaaggggtgggcaattagctagagcagcaggtgctgtagcgaaactgattgcaaaagaa
ggtaaatcggccactttaagattaccatctggggaggtccgtttggtatcccaaaactgcttagcaacagtcggacaagtgggtaatgttggggtgaaccaaaaaag
tttgggtagagccgggtctaagtgttggctaggtaaacgccccgtagtaagaggggtagttatgaaccctgtggaccacccccatgggggcggtgaagggaaag
cccccattggtagaaaaaaacccacaaccccttggggttatcctgcgcttggaagaagaactaggaaaaggaaaaaatatagtgatagttttattcttcgtcgccgta
agtaaatacgtaactaggaatatggaaaattgcatttttggaatttgcaataatgggatgggcgaacgacgggaattgaacccgcgcatggtggattcacaatccact
gccttgatccacttggctacatccgccccttatccagctaaaggattttctctttttccattcatcattattctatttattctgacctccatacttcgatcgagatattggacat
cgaatgccactctttaaaatggaaaaaaaggagtaatcagctgtgacacgaaaaaaaacgaatccttttgtagctcatcatttattggcaaaaatagaaaaggtcaata
tgaaggaggagaaagaaacaatagtaacgtggtcccgggcatctagcattctacccgcaatggttggccatacaatcgcgattcataatggaaaggaacatatacc
tatttacataacaaatcctatggtaggtcgcaaattgggggaattcgtaccaactcggcatttcacgagttatgaaagtgcaagaaaggatactaaatctcgtcgttaa
ctgaattcagaatagaaagattcaaaataaaaaaaaaaaagaaata
>O.glumaepatula_IRGC88793_cp
cccaatatcttgcttcagcaagatattgggtatttctagctttcctttcttcaaaaattgctatatgttagcagaaaagccttatccattaagagatggaacttcaagagcag
ctaggtctagagggaagttgtgagcattacgttcgtgcattacttccataccaagattagcacggttgatgatatcagcccaagtattaataacgcgaccttggctatca
actacagattggttgaaattgaatccgtttagattgaaagccatagtactaatacctaaagcagtgaaccaaatccctactacaggccaagcagccaagaagaagtg
taaagaacgagagttgttaaaactagcatattggaagattaatcggccaaaataaccatgagcggccacaatattataagtctcttcctcttgaccaaatctgtaaccct
cattagcagattcgttttcagtggtttccctgatcaaactagaggttaccaaggaaccatgcatagcactgaatagggaaccgccgaatacaccagctacacctaac
atgtgaaatggatgcataaggatgttatgctctgcctggaatacaatcataaagttgaaagtaccagatattcctaaaggcataccatcagagaaacttccttgaccaa
tagggtaaatcaagaaaacagcagtagcagctgcaacaggagctgaatatgcaacagcaatccaaggacgcatacccagacggaaactcagttcccactcacga
cccatataacaagctacaccaagtaagaagtgtagaacaattagctcataaggaccgccattgtataaccactcatcaacagatgcagcttcccaaattgggtaaaa
gtgcaatccgatcgccgcagaagtaggaataatggcaccagagataatattgtttccgtaaagtaaagaaccagaaacaggctcacgaataccatcaatatctactg
gaggggcagcgatgaaggcgataataaatacagaagttgcggtcaataaggtagggatcatcaaaacaccgaaccatccgatgtaaagacggttttcggtgctag
ttatccagttgcagaagcgaccccacaggcttgtactttcgcgtctctctaaaattgcagtcatggtaagatcttggtttattcaaattgcaaggactcccaagcacacg
tattaactagaaagataatagaaggcttgttatttaacagtataacatagactatataccaatgtcaaccaagccagccccgacagttgtatatccatacaacaaaattta
ccaaaccaaaaaattttgtaaatgaagtgagtgaaaaatcaaaactcagattgctcctttctagtttccatatgggttgcccgggactcgaacccggaactagtcggat
ggagtagataattattccttgttacaatagagaaaaaacctctccccaaatcgtgcttgcatttttcattgcacacgactttccctatgtagaaataggctatttctattccg
aagaggaagtctactaatttttttagtagtaagttgattcacttactatttattatagtacagagaacatttcagaatggaaactgtgaaagttttaccttgatcatttatcaat
catttctagtttattagttttgtttaatgattaattaagaggattcaccagatcattgatacggagaatatccaaataccaaatacgctcactgtgcgatccacggaaagaa
aagtaagttgttttggcgaacatcaaagaaaaaacttgctcttcttccgtaaaaaattcttctaaaaataccgaacccaaccattgcataaaagctcgtaccgtgcttttat
gtttacgagctaaagttctagcgcatgaaagtcgaagtatatactttagtcgatacaaagtcttctttttttgaagatccactgtgataatgaaaaagatttctacatatccg
accaaaccgatcaagaatatcccaatccgataaatcggtccaaattggtttactaataggatgccccgatccagtacaaaattgggcttttgctaaagatccaatgaga
ggagtaacagggactttggtatcgaatttttttcatttgagtatctattagaaatgaattctccagcatttgattccttactaacaaagaatttattggtacacttgaaaagtac
cccagaaaatcgaagcaagagttttctaattggtttagatggatcctttgcggttgagtccaaaaagagaaagaatattgccacaaacggacaaggtaacatttccatt
tcttcttcaaaagaagagttccttttgatgcaagaattgcctttccttgatatcgaacataatgcataaggggatccataacgaaccatatggttttccgaaaaaaagcag
ggtacattaacccaaaatgttccatcttcctagaaaagatgattcgttccagaaaggttccggaagaagttaatcgcaagcaagaagattgtttacgaagaaacaaca
agaaaaattcatattctgatacataagagttatataggaaccgaaatagtcttttattttctttttcaaaataaaaatggatttcattgaagtaataaaactattccaattcga
gtagtagttgagaaagaatcgcaataaatgcaaggatggaacatcttggatccggtattgaaggagttgaagcaagatatccaaatggataggatagggtatttctat
atgtgctagataatgtaagtgcaaaaatttgtcttctaaaaaaggaaatattgaatgaatagatcgtaaattctgaaactttggtatttctttttcttccggacaagactgttc

tcgtagcgagaatgggatttctacaacgatcgcaaacccctcagatagaatctgagaataaaactcagaataaaaaaaattgttgtaatccaataatcgatcttggtta
ggatgattaaccaaattaatccaaaaattctgctgatacattcgaatcattaaccgtttcacaagtagtgaactaaatttcttgttattagaaccaataatttcgacaagttc
ggaaccatttaatccataatcatgggcaaacacataaatgtactcctgaaagagtagtgggtagacgaaatattgtctaggaaatttaagtttttctgaataaccctcga
atttttccatttgtatttctacttgaatcagagagagagagaaatatttctcggtttatcaaatggtgatacatagtacaatatggtcagaacagggtgttgcatttttaatacaa
acccctggggaagaaaaggagtctaatccacggatctttttccgctccttttctatccaatttgtttatgtttgttctaattacaaaagagaacaaatcctttatttttgcagg
ccaattgctcttttgactttgggatacagtctctttatcaatatactgcttcttttacacattcaatccataacatccttttcaatccaaaatcaagaataattaggatttctaaa
aaaaaaaaaaaaaagaaaaaatcaaaggtctactcataggaaaaccagcttttccctacatcaggcactaatctatttttaacgtctaattagatcagggagttcttcca
attaagaagttaagctcgttgctttttgttttaccagaattggagccaggctctatccatttattcattagacccagaaaatcagaattttttttattccattccaaaaatccaaa
ataagaaattgattttattacgacatgctattttttccattcattacccttgaggatcagtcgcggtcttatagactctaccaagagtctggacgaatttttttgcttcatccaaa
tgtgtaaaagatcatagtcgcacttaaaagccgagtactctaccattgagttagcaacccagataaactaggatcttagatacgatcgaaatccaaaaatcaatggaat
tacaccgcacacccctgtcaaaatcttaaaatagcaagacattaaaagaaagattttatcaccattgaaaacactcagataccaaaaggaacgggtctggttaaatttc
actaaggttaaaagtggcaccaatcacgatcgtaaaattgtcatttttttagcatttttatttaaataaataaataaatcttgtatgagagtacaaacaagagggacaaccc
taccatttgagcaaagtgtaggcaaaaaacctaatagggagtgaggataaagagacttatccatctacaaattctagatgttcaatggacctttgtcaatggaaataca
atggtaagaaaaaaattagatagaaaaactcaaaaaaataaaggcttatgttggattggcacgacataaatccagtcaaaaataggattaagaaagaggcaaattatt
tctaaatagttagacaacaagggatactagtgagcctctcctagttttttattcatttagttcttcaattaactcaaagttctttctttttctttaaagaattccgccttccttaaa
atatcagaaactgttcttgtaggttgagcacccttttcaaggaaatagagaatagctggaacatttaaacaagtttgattctttatcggatcataaaaacctacttttcgaa
gatctcttccttctcttcgagatcgaacatcaattgcaacgattcgatagacagcttattgggatagatgtagataaacaaagccccccctagaaacgtataggaggttt
tctcctcatacggctcgagaatatgacttgcattaatttccgtacagaaaaaacaaatttcatttatactcatgactcaagttgactaattttgattgacagacttgaaagaa
aaaaatcctttgaaatttttgagtcgtctctaaactcttttctttgcctcatctcgaacaaattcacttttattccttattccggtccaattctattgttgagacagttgaaaatc
gtgtttacttgttcgggaatcctttatctttgatttgtgaaatccttgggtttaaacattacttcgggaattcttattctttttctttcaaaagagtagcaacataccctttttctt
atttccttcgataaagcatttccctcttctatagaaatcgaatatgagcgattgattctgatagacttttaatcaaaagagttttcccatatcttccaaaattggactttcttctt
attttaaccttttgatttctatattatttcgatttctatattaagggtagaatgacaaagttggcctaatttattagttttcactaaccctagattctttcccttgataaaaaataaat
tctgtcctctcgagctccatcgtgtactatttacttagcttacttacaaacaacccagcgaaaattcggttcgggacgaatagaacagactatgtcgagccaagagcat
tttcattactatggaaaatggtggatagcaaaatccacaatcgatcgtgtccttcaagtcgcacgttgctttctaccacatcgttttaaacgaagttttaacataacattcct
ctaatttcattgcaaagtgttatagggaattgatccaatatggatggaatcatgaatagtcattagtttcgtttttttgtatactaattcaaacttgctttgctatctatggagaa
atatgaataaaagaaattaagtatttatcgggaaagactccgcaaagagccaatttatttaaacccatattctatcatatgaatgaaatatagttcgaaaaaagggaata
aacaagtttgcttaagacttatttattatggaatttccatcctcaacagaggactcgagatgatcaatccaatcctgaaatgataagagaagaattgactcttctccaaca
aataaactatcaacctcccgtttaattaatttaattaatatattagattagcaatctatttttccataccatttttccgtaacaaaactaattaactattaactagttaaactattgc
aatgaaaagaaagtttttggtagttatagaattctcgtatttcttcgactcgaataccaaaagaaagaaaaaaatgaagtaaaaaaaacgcatttcctgtaaagtaaaat
taaggtctttgcttttacttatttttcttttacctaaaagaagcaactccaaatcaaaattgaatccattctatctaacgagcagttcttatcttatctttaccgggatggatcat
tctggatatttaaaaaatcgcggatcgagatcgttttcgcttaaccaaagaaagaaaaagaagaaggaacctttttactaataaaatactataaaaaaaaatttatctctat
cataaatctatctctaccataaaggaataggtctcgtttttatacaacgttctacgtcaagtttaaaatttttcatgaaaaaaagattttcaatttgactggacttgacactg
gattatgttttctgagacagaaaatgaacgcattaggactgcatcgaatctaagagtttataagagaaaaaaattctctttaataaactttatgcctcgtgcagaatacaa
tacgatttcatctttcgtttcatcagaaaaaatctgggacggaaggattcgaacctccgagtaacgggaccaaaacccgctgccttaccacttggccacgccccattt
cgggttttatgcgacactaataaacagtattatgtttatttcttattcgtcaatcctacttcaattacataaaaatggggggtattctcttggtaggattctagacatgcgaat
aatatagaatccaaaaaatgcattgatcattacatggaattctattaagatattatatgaaagtcgaatttcttccactctcatttgagagtgcgaatacaaggaggtatttt
gtgtttgggaaagtccgaagaaaaaaggattttgaatcctccttttcctttttcccttagaaaaataactcaatcaaaatccaattatctactctacaagaacgaaacgctt
gttatgcctaatatacttagtttaacctgtatttgttttaattctgttatttatccgactagtttttttcttcgccaaattgcccgaagcttatgccattttcaacccaatcgtggattt
tatgcctgtcatacctgtactctttttctattagcctttgtttggcaagctgctgtaagttttcgatgaaatctttactactctgtctgccaaattgaatcatgtattcattctaaa
aaaattcgaaaaatggataagagccgagaagtcttatattatgaaccttcgattctaaaattcaaattcttctacattgaatgtatagctgcagcaataaatttggatcagc
ctttctactccctgcatctacgttgagcaggtatctttaggtaaccgcacaatacctaacctaatttattgataagagtgcttattataaatcaattcttgcaatttttttcaaa
aattgatttttgcatttttaggtgtcaaaataaacaaaacccatcctagtggatttgtgtggtaaggaaaaacggggtaatctattccttaaaaaaaaatcttggagattat
gtaatgcttactctcaaactttttgtttatacagtagtgatattctttgtttccctctttatctttggattcttatctaatgatccaggacgtaatcctgggcgtgacgagtaaaa
atccaaaattttttcttacaaattggatttgtttcatacatttatctacgagaaaatccgggggtcagaattccttccaattcgaaagtcccaaacgatccgagggggcgg
aaagagagggattcgaaccctcggtacaaaaaaattgtacaacggattagcaatccgccgctttagtccactcagccatctctccccgttccaaatcgaaaggtttcc
gtgatatgacagaggcaagaaataacgattgcaaaaaatccttcctttttctttcaaaagttcaaaaaaattatattgccaattccattttagttatattcttttttcttaatgtta
ataaaaaaaagaagaaaattcttctttttctttctaattctaaaattggatattggctaaaagacaatcagatagattttctcttcagcaggcatttccatataggacttgtta
taataaaacaagcaggttatagaaaaaaactctttttttattatttatcaacaaagcaaaaaggggtcttatcaaaccaacccaccccataaaattggaaagaaagataa
agtaagtggacctgactccttgaatgaggcctctatccgctattctgatatataaattcgatgtagatgaaattgtataagtggattttttttgtatttccttagacttagacca
cgcaaggcaagaatttctcgctatttactatttcatattcttgttactagatgttctataggaataagaagaaatcgcaacccctttccgctacacataaaaatggatttcg
aaagtcaatttttcttttcaatatctttactttttttcagaatcctatttttgttcttatacccatgcaatagagagcgagtgggaaaagagaggttactttttttcatttttccctta
aaaaataggctttcttggaaataggaatcatggaataatcctgaattccaatgtttatttctatagtataagaaaaactaattgaatcaaattcatggatttaccacgacctc
ggctgtgaccccatagataaaaatgcaaaatttctatcttcgagaccattgaaaaaaggcattgaacgagaaaaaaatcgtccacagataatctatcgtatgccttgga
agtgatataaggtgctcggaaatggttgaagtaattgaataggaggatcactatgactatagcccttggtagagttactaaagaagaaaatgatttatttgatattatgg
acgactggttacgaagggaccgttttgtttttgtaggatggtctggcctattgctttttccttgtgcttatttcgctttaggaggttggtttacagggacaacttttgtaacttc
ttggtatacccatggattggcgagttcctatttggaaggttgcaatttcttaaccgcagcagtttccacccctgccaatagtttagcacactctttgttgctactatggggc

ccggaagcacaaggggattttactcgttggtgtcaattaggtggtctgtggacttttgttgctctccatggggcttttgcactaataggtttcatgttacgtcaatttgaact
tgctcggtctgttcaattgcggccttataatgcaatttcattctctggcccaatcgctgtttttgtttccgtattcctgatttatccactggggcaatccggttggttctttgcg
ccgagttttggcgtagcagcgatatttcgattcatcctcttcttccaaggatttcataattggacgttgaacccatttcatatgatgggagttgccggagtattaggcgcg
gctctgctatgcgctattcatggggcaaccgtggaaaacactctatttgaggacggtgatggtgcaaataccttccgcgcttttaacccaactcaagctgaagaaact
tattcaatggtcaccgctaatcgcttttggtcccaaatctttggtgttgctttttccaataaacgttggttacatttctttatgctatttgtaccggtcaccggtttatggatgag
tgctattggcgtagtcggcctggctctgaacctacgtgcctatgacttcgtttcccaggaaatccgtgcagcggaagatcctgaatttgagactttctacaccaaaaat
attcttttaaacgagggtattcgtgcgtggatggcagctcaggatcagcctcatgaaaatcttatattccctgaggaggttctaccacgtggaaacgctctttaatggaa
cttttgttttagctggtcgtgaccaagaaaccaccggttttgcttggtgggccgggaatgccagacttatcaatttgtcgggtaaactacttggagctcacgtagcccat
gcaggattaatcgtattctgggccggagcaatgaacctatttgaagtggcccatttcgtaccagaaaaacccatgtatgaacaagggttgattttacttccgcacttag
ctactctaggttggggagtagggcccgggggagaagttctagatacttttccgtactttgtatctggagtacttcatctaatttcctccgcagtcttaggcttcggtggca
tttatcacgcgcttctgggaccggagactcttgaagaatcttttccattctttggttatgtgtggaaagatagaaataaaatgactacaattttgggtattcacctaattttgt
taggtataggtgcttttcttctagtactcaaagctctttattttggcggtatatatgatacctgggctcctgggggaggagatgtaagaaaaattaccaatttgacccttag
ccccggtgttatatttggttatttactaaaatccccttttgggggagagggttggattgttagtgtggatgatttagaagatataattgggggacatgtatggttgggtttc
atttgtgtatttggcggaatttggcatatcttaaccaaacccttcgcatgggctcgccgtgcatttgtatggtctggagaagcttacttgtcttatagtttaggcgctttatct
gtctttggttttatcgcttgttgttttgtctggttcaataatacagcttatccgagtgagttttatggacctactgggccagaagcttctcaagctcaagcatttacttttctagt
tagagatcagcgtcttggagctaatgtgggatctgcccaaggacccacaggtttaggtaaatatctaatgcgttccccaacgggagaggttatttttggaggggaaa
ctatgcgtttttgggaccttcgtgctccatggttagaacctctaaggggccccaacggtttggacttgagtaggttgaaaaaagacatacaaccttggcaagaacgac
gttcggcagaatatatgacccatgctcctttaggctctttaaattctgtgggtggcgtagctaccgagatcaatgcagttaattatgtttctcctagaagttggttagcgac
ttcccattttgttctaggattcttctttttgtgggccatttgtggcatgcaggaagagcccgggctgctgcagcaggatttgaaaagggaatcgatcgtgatttggagc
ctgttctttacatgacccctcttaactaagattttcttatttatacctgttctactgtttttttctgttctggctcggttattctatctagccgagccattcattcctttctatgaaag
aaagataaggggacagaacaaaaaaaacaaataaagaaacaaacgtattcaatacgcaaaagaaaagagagaggaaagcaaaaggagagagagggattcga
accctcgatagttcctagaactataccggttttcaagaccggagctatcaaccactcagccatctctccacagcctaatccttattttattcctacaaatagaacatagcc
atacgaaatgactcactaacctctagaaacatctcaaatacaaatccctttttcgatatatttctgtatactgtatccatgtatacaggatccgctatatccgcttgtgaaata
aagcataaaacccccctcaaccccatatccaaataaaaaaagtggtgagtaataagttttaaagagaagaatcaatggattcatgattaaacccctcctacttcttgtattt
tattacaattttgattaagtgagggatcaaatataatatgtagtcaactttatttgatggtaacttggaggattagaaatatgactattgctttccaattagctgtttttgcatta
attgtgacttcctcagtcttagtcattagtgtaccccttgtatttgcttctcctgatggttggtcaaacaataaaaacgttgtattttccggtacatcattatggattggactgg
tctttctggtagctattctaaattctctcatttcttaaatgtgtttagtatttagtagcccgatacaaaataaaaaagggccgtttattcggattgtgagacgcattaaaatgc
aatttgcgccccgaattgattgacagacaattaaattaataaaaagaaaactcaaatagaaaatgaaacggtcgacccagacatagacggtcgacccaggcggata
taccctataaaatataggacgtagcgagcgtagttcaatggtaaaacatctccttgccaaggagaagatacgggttcgattcccgccgctcgccagcttaatttagta
aggtactatgagaaaaaatttagtctacttttattaaagtaataggtgttagtctagtaccgtacccctttactatcttagcccccttgcaccccactcaaaaaaagagca
ctacagcggcgggaatcgaactcggcaacagggttccctaaaccggggattcaccgaaacaaacaaccagcaaacagcttttaaagggaagggagatagactg
tgcctttctttcattatttttcttttctgcaaggtagggggccttgagagttcctcttgtggtagcaagttacttcgcaacctgctcaatttggccttatagggtcgggaact
aatgaataaaaagggttggataccgcccaaccacccagccctctaccatatctagacaaatagaatagtccttttatacagactgctaagtgcggagacgggaatcg
aacccgtgacctcaaggttatgagcctcgtgagctaccaaactgctctactccgctctggagcgctggaaaccggtggacgaaaaaggttgaatacaggcctctac
catgtctagacaaatagaatagttattttatacagaatggagcgggtagcgggaatcgaacccgcatcgttagcttggaaggctaggggttatagtcgacgttggttg
attattattaacgtctctaattcaaaaccgaacatgaaattttgatttcattcggctcctttatgggtattctcaccacttaacatctaagtcagctttttttgtctgaatggaacc
aaggctctctgctttctagatgatccctaaagagtaggagatagaaatttgcctaaatctatctaatctaattacttcgttccctaatttcatttaagagatcctgaggaaaa
gaattgggtttccaccgagctgaaacaatatgctgatggttctagtaaaccaaaactaccgtttttttagctatttggcttccatttcctttttttaacaaaagaagatttagtta
cgattggaaataaattttttttgtatcttcatccatagatcctttactcatattttaaaaattggaatccttaatccaatgcaaaattatgcttcgcgactctgtactcataatcca
aatcctatttgtttttttggatgcaatttcaattagtctttgggtacaaatcgcgaaaatgcatattcttcctcaatatgctattgagagaaaaaggagtaaatcctttctaaga
actaaagttttcatcggaatataaaaaaccttaaggacgccttaagtatatcatttcaaattcagttattaatagaacgaatcacacttttaccactaaactatacccgctac
atgtaaattctgataccaacgctaccctttgtcaagggtagccattcgagaaagatgctaattccctcttagttaatgaaacagagaaggttcctcacagttagcggttg
gtacttcgatcgcgggcctttcctttactttctttttttgttcagaattgaacaaagaatttggggaagaaaacatcttcccccacttatcatgaaatctgggccatagagaa
agagtgagatgttttttttttatttatcatagactttccctatggcttgagagaaacaataaataacttaaagaaaaaggcacataggagccgaaggatttacttgatgtaa
agaagattctgaatgtctctgcttagtcgattctctccgtttaactttttcttctcttcttttccactcaattctagtttattagattcttgtttaaaagaatcaaagaagatgaata
gaactaagaacacacaaaaaaagagcatataggcccgagaccattaccaaaagttcttcccaataatcatattgggtatctgttcccttccttttcctattaggatggggc
atgttggattttccatatccatataccatcgaaccttaagggttccagaaccctcctttttttgctagttttcggaaacggaaaaccctgaaccaggagcagtataaattct
actgcccgccttttacaagaaaattggtgataaaactccactacagtttgttcaaaatgcaccaatcagaatcccttgagaatattcaagtaccctatttctaagagaag
aggtacctgctgaaaatcgcttcaacaaatccgcttaagaaaaattgcaaagttttgaactcgaaggtttttccaagggatgcctgttaaaaattgtttcaacctctcacc
aaaacagacaagaagtatatcactgaaaattaatacccagccatatgggtatatgaagggcgcgaattcgtttataccccacccaattagaggaaataaaacataaat
ggagaaagttttcatgataagatcagccaatagaagaaaaaagtccctaatttttcagaccgttctgagcatgtgaaaagtcaatagcctaaagataaaaaaccctata
ctttgtgcaagtgataagagagaatatgaaagattttcctatttttcttgattttcttaagattttttgaacctgaccatgaatagacttatatatctcgatatatacagatataa
tgtacattatggagtagacctataatgggaaatgaaagtggctaattttggaattgaataagaagcccttttaactcagtggtagagtaatgccatggtaaggcataagt
catcggttcaaatccgataaagggctttttttacttagtggtagagtaatgccgcggtaagacgtgagtcagtggttcaaatccgatacagtacttttctactaaattcatt
cactttctttctttttgaaaatttctcttttttattgaatttgataacttagtgcgagatgcatgcatttttagtttaaacactaagcgaagcagggggggtgtaaattccaaaaa
agaaattggactctttttcctattagatcaatcaaatcactacccgtactgaactaatatagaatcccttttattaatctattcttattccatatcctttataaacgaatttcccta

aaaagtaggggatgatccgtgaattaacctaaccatcaactaaaaaaaatcctacaaaagcataatggaaaagtaggaaggactccttgctttggatctagttatact
cttcgagtatattgacaattccaaaaaactgctcatactatcattatagtataatgaggagcggttgtataccgccctatcgtctagtgatgcccctatcgtctagtggttc
aggacatctctctttcaaggaggcagcggggattcgacttcccctgggggtagggagtattatgaaaggaggttaatcatagattatcaaaaaccctagaataaattc
ttcctgggtcgatgcccgagcggttaatggggacggactgtaaattcgttgacaatatgtctacgctggttcaaatccagctcggcccaaaaatctagggcttcgtga
atatgagttaaatccatttttattcttccataaaaaagaatatttgatccatagaaataaaagaaataaaggataaaaagaaaaggggaaatatctttctaatctatatctct
ttcattcctctcttacaaacaaaagaccttttcttattggttattgaaaggtggattattatctatttttagcgataaaaaatcgcgacatactagttatgtcattctcactatac
ccccatacgatacgggggtatgtagtatatgattcgtctatttcttagagtaggacaggcgaatattcttattcttatggttccatttaagaataagtatgccatacgcccc
gcggggattgtagttcaattggtcagagcaccgccctgtcaaggcggaagctgcgggttcgagccccgtcagtcccgaactagggttcaatgaatggagaaattc
atctttcctttttccatgaaaaaagggggcaggaagcaagatcaaatacctatggggtacccctatttcacttttttagttcgcgtttctcagtaaagagaggagagta
taggaatttttttatcactacttctggttgatagcgaaagacatacatatcatacgtggaagggatcctcctatgttatactattccactctcaaccatgaattgatttgatag
atccgatattcataatattgaattgattcagtattatcagaatgcaagtcctccccttgaatttacaggatacccttttccctctccatgggattacatcccgagttattgcga
aaaaaaagaggttatggaagtcaatattctcgcatttattgctactgcattgttcattctagttcctactgcctttttacttattatttatgtaaaaacagtcagccaaaatgatt
aattggaagttcaattaatcattgaagaaatgaaaaagggattaaataaaataaaaatccaagtcttaaatgaaaggatccggttggaatcataaagtgtggtagaaa
aaactacatatagtttttctaccacactttagagtctttctattatattatcttgaatctacatagaatagattactagattgaaatagtagtctaattcaatttctttttcactgc
atccacttaatttcaatcaagtcaaaatgaaaaaatccatggagggagagaaaaataatatgagaatagactatagtaaaagaaaaaaagtaaaaggaaaaaacca
gcgaatcttccatgcttaaacatgtcgcgaaatgtttcaaaagagcataaaatttattttaagaactaagaataagaaaagagtataaaacaaatggaaaatgtgcgat
atgttgggaatagctccgcggaagaaaatctaaagttcttatgtatagaactttttaaccatgggtcgcttctagtagcgattatgaattgctctcaccgctctttctatttc
tattttctattctttctatttctatatagtagaatagaaatagaaagaatagaaagactctttcttacaagagtttcttacaggagtgaaacaaaattaaagaaagagagaa
agaataaagttcggcaaaatgattaatgcaaaacggtcaattaaagaaaagagttgatacaacaattcgactactcaatcaattagtagtatccctagagtccactcct
cccccatactactagtgaaagagaaaatgtaaagactaccattaaagcagcccaagcgagacttactatatccatgtaaattatgtctcctatttctatgaaggaattatt
ctactattgatgaataatcatagtagaatcaagggtacagagtcaaaaaggggttctgacctaaggctatggatgaatcagttcaaagaatttactcttaacaaattctt
agagtatttctggtagaattggggagcattaagtataaatatgatacatagccctttcttattaataaaagaataaggaaacgctatctcatccctattggtatcggtttgg
gccactactgctaaaacaaaccccagtttgaggaaagaacggtgggttctcaaaatccagtatcgccgagccttgttattctcttgccccaacttatgcggggtgcaa
atttgtcgatttggatcagtactataagcctaagtattttattgatcaggcggcacccagatttgaactggggataaaggatttgcagtcccctgccttaccgcttggcca
tgccgccaaaaaatacgatctaaaatcgagaaaagagcaagtattcatccacgtttcttactaaaactaactttcttttatcttaaatctaattctacttactttttccaatctt
tttcaaaaaatctattcatgcttttttggatccagtttcgattattctcctcaaaggattctatcttaaaacacacattgctaacactagaaaacttccctttctttctattgaa
atgaaaaaggagaaaagtggatttctagtcacaagctacaaaattaagaacaaattggaaccattaactagaattctctttttattttgaatttcggtattctctcccgcct
gccatttaatggcataataaaagacaatggatttatgcctaatccgtatataggtaaactccaggtccgaacagcattattatctatggatccccccttatgtacatatctct
gtggagaatcgttctttaatttttcattgcattaaatatcttgaataaaaaaaagaaaattgactttgctatgtggaggtccagaactagattggcatgtacttaaataaagt
acttactttattttaggattctacaacgaaatcttatatcttatattttatagaaattctactactacgaaacaaaaaagaaccttcaaattctttttgaaattaaactaagcgt
gctattctcaatcgaactaaagtcaaactttctagtgcttataaattattatattttggttttatctcattcatagaaaggagaaaaaatgagaaatctttgccgtccaatctaa
gaatatcataaactgtaagtggcagaattttttttcgaggttctaggaatgttttatcacttcattttcattccatttggacccctgggaaattcgaactttcgttgaaattgtct
ctattcatatgtatgaaatacatatatgaaatacgtatgtggagttcccgagaatttcatgtgatttagtaaacagaatatagattccataattgctagatcgatctgtaggg
attgatgaagagtgagctgataatggaatttttcttcgataaacaggaaacttaagattaagatgctccggaatggaaatgagggaatgtccacaatacccggatttag
tcagatccaattcgagggattttgtaggttcattaatcaaggcttggcagaagaacttgagaagtttccaacaattaaagatccagatcacgaaatttcatttcaattattt
gcgaaaggatatcaattgctagaaccttcgataaaagaaagggatgctgtgtatgaatcactcacctattcttccgaattatacgtatccgcgcgattaatttttggtttc
gatgtgcaaaagcaaaccatttctattggaaacattcctataatgaattccttaggaacctttataataaatggaatataccgaattgtgatcaatcaaatattgctaagtc
ctggtatttactaccgctcggaattagaccataaaggaatttctatctacaccgggactataatatcagattggggaggaagatcggaattagcaattgataaaaaaga
aaggatatgggctcgcgtgagtagaaaacaaaagatatctattctagttctatcatcagctatgggttcaaatctaaaagaaattctagataatgtttcctaccctgaaat
tttcttgtctttccctaatgctaaggagaagaagaggattgagtcaaaagaaaaagctattttggagttttatcaacaatttgcttgtgtaggtggggacctggtattttcg
gaatccttatgtgaggaattacaaaagaaattttttcaacaaaaatgtgaattaggaaggattggtcgacgaaatatgaatcgaagactgaatcttgatatacctcagaa
cagcaccttcttgttaccgcgagatgtattggccgctacggatcatttgattggaatgaaatttgaaacgggtatacttgacgatgacgatatgaatcacttgaaaaata
aacgtattcgttcggttgcggatctgttacaagatcaattcggactggctcttggtcgtttacaacatgcggttcaaaaaactatccgtagagtattcatacgtcaatcaa
aaccgactccacaaactttggtaactccaacttcaacctcgattttattaataactacttacgagaccttctttggtacatatcccttatctcaagtttttgatcaaaccaatc
cattgacacaaacggttcatgggcgaaaagtgagttgtttgggtcctggaggattgacggggagaactgcaagttttcggagccgagatatccatccgagtcactat
gggcgtatttgtccaattgacacgtccgaaggaatcaacgttggacttactggatctttagctattcatgcgagaattgatcattggtggggatccgtagagagtccatt
ttatgaaatatctgagaaagcaaagaaaaaaaaagagagacaggtggtttatttatcaccaaatagagatgagtattatatgatagcagcaggaaattctttgtccttg
aatcggggtattcaggaagaacaggttgttccagctagataccgtcaagaattcctgactattgcatgggaacagattcatgttagaagtatttttcctttccaatattttt
ctattgggggttctctcattccttttattgagcataatgatgcgaatcgagctttaatgagttctaatatgcagcgccaagcagttccgctttctcggtccgagaagtgcat
tgttggaactggattggaacgccaaacagctctagattcgagggtttctgttatagccgaacgcgagggaaagatcatttctactaatagtcacaagatccttttatcaa
gtagtgggaagactataagtattcctttagttacccatcggcgctctaacaaaaatacttgtatgcaccaaaaacctcgggttccgcggggtaaatccattaaaaaag
gacaaattttagcggagggggctgctacggttggtggggaacttgctttaggaaaaaacgtattagtagcttatatgccatgggaaggttacaattttgaagacgcag
tattaattagcgaacgtttggtatatgaggatatttatacttcttttcacatccgaaaatatgaaattcagacggatacaacaagccaaggctccgctgaaaaaatcacta
aagaaataccacatctagaagaacatttactccgcaatttggacagaaatggagttgtgaagttggggtcctgggtagaaacaggcgatattttagtaggtaaattaa
cgcctcagatagcgagcgaatcgtcctatatcgcggaagctggattattacgggccatatttggtcttgaggtatccacttcaaaagaaacttctctcaaactaccgat
aggtggaagaggacgcgttatcgatgtgaaatggatccagagggatcccctcgacataatggttcgtgtatatattttacaaaaacgcgaaatcaagttggggata

aagtagccggaagacacgggaataaggggatcatttccaaaattttgcctaggcaagatatgccctatttgcaagatggaacgcctgttgatatggttttcaatccctt
aggagtaccctcccgaatgaatgtgggacaaatatttgaaagctcgctcggattagcaggggatctgctaaagaaacattatagaatagcaccctttgatgagagat
atgagcaagaggcttcaagaaaacttgtgttttcagaattatatgaagccagtaaacaaacaaaaaatccgtgggtatttgaacccgagtacccgggaaaaagcag
aatatttgatggaagaacaggagacccccttcgaacagcctgttctaatagggaagtcctatatcttaaaattaattcatcaagttgatgagaaaatccacggacgctct
actgggccctattcacttgttacacaacaacccgttagaggaagagccaagcaagggggacaacgaataggagaaatggaagtttgggctttagaaggatttggt
gttgctcatattttacaagagatacttacttataaatctgatcatcttatagctcgccaagaaatacttaacgctacgatctggggaaaacgagtacctaatcacgaggat
cctccagaatcttttcgagtgcttgttcgagaactacgatctttggctctagaactgaaccatttccttgtatctcagaagaacttccaggttaatagggaagaagtttgat
cggaataaatataaattcttttcttatttctattttatgattgaccaatataaacatcaacaacttcaaattggactcgtttcccctcaacaaataaaggcttgggctaacaaa
acactacctaatggggaagtcgttggcgaagtcacaaggccctccacttttcattataaaaccgataaaccagaaaaagatggattgttttgcgaaagaatctttgga
cccataaaaagcagaatttgtgcttgtggaaattctcgagcgagcggagctgaaaacgaagacgaaagattttgccaaaaatgcggagtagaatttgttgattctcg
gatacgaagatatcaaatgggatacatcaaactcgcatgtcccgtgactcatgtgtggtatttaaaaggtcttcctagttatatcgcgaatcttttagataaaccccttaa
gaaattggagggcctagtatatggcgatttctcttttgctaggcccagtgctaaaaaaccaactttcttacgattacgaggtttattcgaggatgaaatttcatcctgtaac
catagcatttccccctttttttctaccccaggctttacaacatttcgaaatcgggaaattgcgacaggagcaggtgctattagagaacaattagcagatttggatttgcga
attattttagagaattcctcggtcgaatggaaggaattagaagacgaggggtatagtggagatgaatgggaagatagaaaaagacgaataagaaaagtttttttgatt
agacgcatgcaattggcgaaacattttattcaaacaaatgtagaaccagaatggatggttttgtgcttattaccagttcttcctcccgaattaagacccattgtttataggt
ctggggataaagtagtgacttcggatattaatgaactttataagagagttatccgtcggaacaacaatcttgcctatctattaaaaagaagtgaattagcgccagcaga
tttagtaatgtgtcaggaaaaattggtacaagaagctgtggatacacttcttgatagtgggtcccgcgggcaaccaacgagggatggtcacaataaagtatacaaat
cactttcagatgtaattgaaggtaaagaggggaggtttcgcgaaactctgcttgggaaacgggtcgattactcggggcgttctgtcattgttgtgggtccttcactttca
ttacatcaatgtgggttacctctagagatagcaataaagctttttcagctatttgtaattcgcgatttaatcacgaaacgtgctacttctaatgttaggattgctaaaaggaa
aatttgggaaaaggaacccattgtatgggaaatacttcaagaagttatgaggggacatcctgtactgttgaatagagcacctaccctgcatagattaggcatacagg
ctttccaacccactttagtagaggggcgtactatttgtttacacccattagtgtgtaaaggtttcaatgcagactttgatggggatcaaatggctgttcatctacctttatcc
ttggaagctcaggcggaagctcgtttacttatgttttctcatatgaatctcctatctcccgctattggggatcctatttgcgtaccaacccaagacatgcttatcggacttta
tgtattaacgattggaaaccgtcgaggtatttgtgcaaatagatataatagttgcggaaactatccaaaccaaaaagtaaattacaataataataatcctaagtatacga
aagataaagaatctctttttttctagttcctatgatgcactgggagcttatagacagaaacaaatctgtttagacagtcccttgtggctccgatggaaactagatcaacgc
gtcattgggttaagagaagttccgattgaagttcaatatgaatctttggggacttatcgtgagatttatgcccactatctagtagtgggaaatagaaaaaaggaaatccg
ttctatatacattcgcaccactcttggtcatatttctttttatagagaaatagaggaagccatacaaggatttagtcaggcctattcatacactatctaaacaaggaagtta
gattcggggatgccccttcgaggggcattccgatttcgctagtatcatcatttttgccgcacgaatccagattgagattgaggaaaggaagttaactaagttttcgaat
cactgactcaggcccattgtcgaatcctactcagcaattgtcgaattatactcagccgaaaaaagggggtacttatttatggcggaacgggccaatctggtctttcata
ataaagagatagatggaactgctatgaaacgacttattagcagattaatagatcatttcggaatgggatatacatcccatatactggatcaaataaaaacgctgggctt
ccatcaagccactactacatcgatttcattaggaatcgaggatcttttaacaataccctctaagggatggttagtccaagatgcggaacaacagagttttcttttggaaa
aacactattattatggggctgtacacgcggtagaaaaattacgccaatccgttgaaatctggtatgctacaagtgaatatttgaaacacgaaatgaattcgaattttcgg
ataacagatccttctaatccagtctatctaatgtctttttcaggagctagaggaaatgcatcgcaagtacaccaattagtaggtatgcgaggattaatggcggatcctca
aggacaaatgattgatttacctattcaaagcaatttacgcgagggactttctttgacagaatatataatttcctgctacggagcccgcaaaggggttgtagatactgctg
tacgaacggcggatgctggatatcttacacgtagacttgttgaagtagttcaacatattattgtgcgtagaagagattgtggtactatccaagctatttctgtgagtcctc
aaaatgggatgacggaaaaactttttgtacaaacattaattggtcgtgtattagcaaacgatatatatatcggttcacggtgcattgccactcgaaatcaagatattgga
attgggttagtcaatcgattcataaccacctttcgagcacagccatttcgagcacaaccaatatatattagaaccccctttacttgccggagcacatcttggatctgtca
attatgttatggtcggagttccactcatggcgatctggtcgaattgggggaagctgtaggtgttattgcgggtcaatctattggggagccagggactcaactaacatta
agaacttttcatactggtggggtattcacagggggtactgccgaccttgtacgatccccttcaaatggaaaaatccaattcaatggggatttggttcaccccacacgta
cccgtcatgggcagcctgcttttctatgttatatagacttgcatataactattcagagtcaggatattctacatagtgtgactattccttcaaaaagcttgattctagtgcaa
aatgatcaatatgtagaatccgaacaagtaattgcggagattcgtgccggaacgtccgctttgcattttaaagaaaaggtacaaaaacatatttattccgaatcagatg
gggaaatgcactggagtaccgatgtttatcatgcgcccgaatatcaatatggtaatcttcgtcgattaccaaaaacaagccatttatggatattgtcagtaagtatgtgc
agatctagtatagcttctttttcgctccacaaggatcaagatcaaatgaatacttattcctttctgttgacggaaggtatatctttggcctctcgatggctgatgatgaggt
aagacatagactgttggatacttttggtaaaaaagagagggaaattcttgattattcaacgccggatcgaatcatgtccaatggtcattggaattttgtctatccttctatt
cttcaaaataattttgatttgttagcgaaaaagcgaagaaataggttcgccattccattacagtatcatcaagaacaagagaaagaaccaatatcctgttttgggatttcg
attgaaataccctttatgggtgttttacgtagaaatactatagttgcttattttgacgacccacgatacaaaaaagataaaaagggttcaggaattgttaaatttagatata
ggaccctagaggacgaatataggactcgagagaaagactcagagaacgaatacgggagcccagaaaacgaatataggacccgagaggaagaatgtaaaacc
ctagaagacgaatataggactcgagaggaggagtatgaaaccctagaagatgaatatgggatcccagagaacgaatatgaaaccctagaagatgaatatggaat
cctagaggacgaatataggactcgagaggaggaatccgaggacgaatatgggagtccagagaacaaatataggccccgagaggataaatatggcactttagag
gaagactcagaggacgaacatgggactttagaggaagactcagaggaagactcagaggacgaatatgggaacccggaggaagattccgtcttaaaaaagggg
gttttgattgagcatcgaggaacaaaagaatttagtctaaaataccaaaaagaagtagatcggttttttttcattctccaagaactgcatatcttgccgagatcctcatcc
ctaaaggtacttgacaatagtattattggagtggatacacaactcacaaaaaatacaagaagtcggctgggtggattggttcgagtgaagagaaaaaaaagccata
cggaactaaaaatcttttctggagatattcattttcctgaagaggcggataagatattaggtggcagtttgataccactagaaagagaaaaaaaagattcgaaggaat
caaaaaaaagggaaaattgggtctatgttcagtggaaaaaaattctcaagagcaaggaaaagtattttgttttggttcgacctgcagtcgcgtatgaaatgaacgaag
ggagaaatttagcaacacttttcccacaggatctcttgcaggaagagggtaatctccaacttcgacttgtcaattttatttctcatgaaaatagcaagttaactcaaagaa
tttatcatacgaatagtcaattcgttcgaacttgcttagtattgaattgggaacaagaagaaaaagaggaggctcgtgcttcccttgttgagataagagcaaatggtctg
attcgcgatttcctaagaattgggttaatcaaatccactatttcgtatacacgaaaaggtatgatagcagaagtgcaggactgattctccataataggttagatcgcac

caataccaattccttttattccaaggcgaagattcaatcacttagccaacatcaagaagctattggtaccttgttgaatcgaaataaagaataccaatctttgatggttttg
tcggcatccaactgttctcgaattggtttttttaagaattcaaaaaatcccaatggggtaaaagaatcgaatcctagaattcctattccaaaatttttggggctctttcgaaa
tttttcgggactcttaggtactattgcacctagtatatcgaatttttcttcatcttactatttactaacgtataatcagatcctgttaaaaaagcatttgctccttgacaatttgaa
acaaaacttcaaagtacttcaaggacttaaacactctttaataaatgaaaatcaaaggacttcaaatttcgatagtaacatcatgttggatccattccagttaaattggca
ctttctccctcatgattcttgggaagagacatcagcaaaaattcaccttggacaatttatttgcgaaaatgtatgtctatttaaatcgcacataaaaaaatcaggtcaaattt
tcattgtaaatatagattcctttgttataagagcagctaagccttatttggccactacaggagcaactgttcatggtcattatggagaaatcctttacaaaggggataggtt
agttacgtttatatacgaaaaagcgagatctagtgacataacgcaaggtcttccaaaagtagaacaaatcttcgaagcgcgttcaattgattcactatcgccgaatctc
gaaaggagaattgaggattggaatgagcgtataccaagaattcttgggggtccttggggattcttgattggagctgagttaaccatagcccaaagtcgtatctctttgg
ttaataagatccaaaaggtttatcgatcccaaggggtacagatccataatagacatatagagattatatacgccaagtaacatcaaaagtgcgggtttccgaagatg
gaatgtctaatgtttttcacctggggaattaattggactattgcgagcagagcgagcagggcgggctttggatgaatcgatctattatcgggcaatcttattgggaata
acaagggtttccctgaatacccaaagtttcatatctgaagcaagttttcaagaaactgctcgagttttagcaaaagctgccttacgaggtcgtattgattggttgaaagg
cctgaaagaaaacgtagttctgggggggattatacctgttggtaccggattccaaaaatttgtgcaccgttacccacaagacaagaacctttatttcgaaattcaaaaa
aaaaaactatttgcgtcggaaatgagagatattttgtttctccatacagaattagtttcttctgattctgacgtaacaaacaatttctatgagacatcagaatcaccatttac
cccatttatatgatttaaggatacataaagcagattttttactttaaactagatttttgaccttagaacgctaagaggttagattttctatttttattttaatttaaaaagaagttta
gttaattcattaaggttatgcttataccatgtagaaggttccatcggaacaattattatttatttcaagctatttcggctctttcttaatcttcgaaaagaaagaaatttcgtaat
ggaaaggtaggatgaaaaaaaaagaaaaaatcaaaaggaagtgtggaaaaaatgacaagaagatattggaacatcaatttgaaagagatgatagaagcgggag
ttcattttggtcatggtattaagaaatggaatcctaaaatggccccttacatctcggcaaagcgtaaaggtactcatattacaaatctcgctagaaccacccgtttttttatc
agaagcttgtgatttagtttttgatgcagcaagtcagggaaaaagcttcttaattgttggtaccaaaaaaagagcagcggatttagtagcatcagctgcaataagggct
cgttgtcattatgttaataaaaagtggttcagtggtatgttaacgaattggtcgattactaaaactagactttctcaatttagagacttaagagcagaagaaaagatggaa
aaattccaccatctcccaaaaagagatgtggcaatcttgaagagaaaattatctaccttgcaaagatatctcggcgggatcaaatatatgacgaggttgcctgacatt
gtgatcgtcctcgatcagcaaaaagagtatatagctcttcgggaatgtgccattttggggattcctactatttctttagccgatacaaattgtgacccagatctcgcgaat
atatcgattccagccaacgatgacactatgacttcaattcgattgattcttaacaaattagtatttgcaatttgtgagggccgttctctctatataagaaatcattgattaag
aagaatagttaattcttgggcaactgcgtagatttatggaatcacttactattcttttttgttttgcatagaaaaaagacggggaatattgatatatattagagggtattgata
tatattatcatctgatgtgatttcttgatatcctaaatataagattaatacttcacgttgctgagttgagaaaaagatggttgaatcaaaagaattcttttttgaagttcatttttt
atcaggggacaatatgaatattataccgtgttccattaaaacactcaaggggttatacgatatatcgggcgtagaagtaggccaacacttctattggcaaataggagg
tttccaaattcatgcccaagtacttatcacttcttgggtcgtaattactatcttgctaggttcagttatcatagctgttcgcaatccacaaaccatcccaaccgatggtcag
aatttcttcgaatatgtccttgagtttattcgagacttaagcaaaactcagattggagaagaatacggtccctgggttccctttattggaactatgttcctttttatttttgtttc
gaactggtcgggtgctcttttaccttggaaaattatacagttaccccatggagaattagcagcgcccacgaatgatataaatactactgttgctttagctttactcacgtc
agcggcatatttttatgcgggtcttagcaaaaaaggattgagttatttcgagaaatatattaaaccaactccaatcctttttaccaattaacatattagaagatttcacaaaa
ccattatcgcttagttttcgacttttcgggaatatattggcggatgaattagtcgttgttgttcttgtttctttagtccccttagtagttcctataccggtcatgtttcttggattat
ttacaagcggtattcaagctcttatttttgcaacgttagccgcagcctatataggtgaatccatggagggtcatcattgaattgactagttttcaaaatagtcttttttttagc
ttaactcaattcatgcatggttgcggaaaattcgcttggttggaaaacaaaatagttagaattgcgtatgaatatacaatctagagttgtagaagagagaataggctata
ttacggaattgccaaacaaagtatataggcattagggaggggcggagtcaggctagatctatatcctttatgtctataagttcagtcatcttttgtatgggtttccacttta
aggaattttttttgaatccgattcaatagaaaatgagaaaatacacaaacaaaatagaagaaacaaattgatatgggatattatatattcccaagttagattcattatctaa
tccgatatatggaatccgattccatatccaattcgatgcagcatattgttatcaattggatatcttgatttaattcctattggatctggattaggtcgatttccataggggttct
tcctctatttcaccttttattatgaattagatgataggggaaaaaaatagaaactcaaggatatcgaagaggaaagaaagaaggatggaatgaaagatcagttggttgg
aaagaaagagaaatagaataatgagtacacaaacctctaatgattagaaactaaaaaggagatctcgaagcagttcggagaattcagattatcgtttcaatttgtactt
tttagttacttctgtccaatagagcttagaaatatgaatttcttggttgattgtatccttaaccatttcttttttttgacacgaggaactcatcatgaatccactaattgctgctg
cttccgttattgctgctggattggccgtaggtcttgcttctattgggcctggagttggtcaaggtactgctgcaggacaagctgtagaaggtattgcgagacagccag
aagcagaaggtaaaatacgcggtactttattgcttagtctagcttttatggaagctttaacaatttatggactagttgtggcactggcgcttttatttgcgaacccttttgttt
aatcctaaaaaagaaaacgagtcctttagattagatactttcttcttttttttagtaaattggtatttgcttctgcaattccaattatatcaatactttactcctaatttattactccta
gagttttctatttatggggacagacaataccccaccccaggaatagctgatttgaggatgatcaatttagaggatatgttcgccgtcttgcttcccgccctttgtttaggg
tagtggaaagtatttttcctttattttaggaattttgggaacatttcaacaaaggaggtctttcacaggtcaaacgagatctaagacttaatctaaaagaaattactaaatt
gaatctatttctattaaaaaaaattgcattaaaaaaaccgatcaaaaagggcgagcgaagtaagtgatcgaaaaactttgttctttgttcgtcctatctataagaggaga
gcatatgaaaaatgtaacccattctttcgtttttttagctcactggccatccgctgggagtttcgggcttaataccgatattttagcaacaaatctaataaatctaactgtag
tggttggtgtattgatttattttggaaagggagtgtgtgcgagttgtctatttcaagaatagattggatctatccggctgcactttagaatatttttagtatttttcgaataaat
aagaaaagggtgcacgatctcgacgaattacttctgaataaatgcagaaatcatatgaaagaaccatagcatttcgcgacctattggtaaatcaaatcaactttgattct
ctatagaccaataatatgagaccattaacacggttaaagctaaactgcttgaagtctaggcaaaagggggtactctttctacaactatattagtattagtaccaaatgctt
taaacaggaaatagctaatgtagaatttatctgatatagaacactcatatcgataaaatggtttgaactatttactagaaggggcaccctgccctttttttatccaatgccg
aatcgacgacctatgtataaaaaaggagaaagtttttggatttgaagaaaaaagtaggaattctatccattttcattttccatttttctatttagtttttcttaatgaaattgaaa
attttaactaaagggcaaatacaaataaaaaaacaactttgctgcccacgatagatttttatctaggcggaagagtcctcttaatatttatctagtcttatatgggtttcggt
atattgaaatataaacagaaaagagagggtagaggataggctcattacattaaaaaaaatatggaagtagccatagcaaaaaaagaaaaaagggagcgtgagag
ccaaatgaatcgaaagattcatgtttggttcgggaagagatcataaaagttgtaaacttaatagcaaggtaatctactttcattaaaagatttattagataatcgaaaaca
gaggatcttgagtactattcgaaattcggaagaattgcgtagaggaaccattgagcagctcgaaaaagctcgaattcgattacagaaagtcgaactagaagcggat
gagtatcgaatgaatggatactctgagatagaacgagaaaaagcaaatttgattaatgccacttctattagtttggaacaattagaaaagtctaaaaacgaaaccettt
atttgaaaaacaaagggcgatgaatcaggtccgacaacgggtttccaacaagccgtacaaggagctctaggaactctgaatagttgtttgaataccgagttacatt

tccgtacgattcgtgctaatattagcattctcggggccatggaatggaagagttaaattaattaggccttgaacttctactttccttagaatttaggcattatttttcccctt
gcttccgaaaaaaaaatagtaaagaaacactaatggcaacccttcgagtcgacgaaattcataaaattctccgcgaacgtattgaacaatataatagaaaagtaggg
attgagaatatcggtcgcgtagttcaagtgggggatgggattgctcgtattataggtcttggtgaaataatgtcaggcgaattagtcgaatttgcagaagggactagg
ggtattgctctgaatttggaatccaaaaatgttgggattgtattaatgggcgatgggttgatgatacaagagggcagttttgtaaaagcaacaggaagaattgctcaga
tacccgtgagcgaggcttacttgggtcgtgttataaatgctctggctaaacctattgatgggagaggcgaaattgtagcttcggaatctcgcttaattgaatctcctgct
ccgggtataatttccaggcgttctgtatatgaacccccttcaaacggggcttattgctatcgattcgatgattcctattgggcgcggtcagcgagagttaattattggga
cagacaaaccggcaaaacagcagtagctacagatacaattctcaatcaaaaagggcaagatgtaatatgtgtttatgtagctatcggtcaaagagcatcctccgtag
ctcaagtagtaactactttccatgaagagggggccatggaatacactattgtagtagctgaaatggcggattcccctgctacattacaatacctcgctccttatacggg
agcagccctggctgagtattttatgtaccgcgaacggcatactttaataatttatgatgatctctccaaacaggcacaagcttatcgccaaatgtcccttctattaagaag
accccctggccgcgaagcttacccaggggatgtttttatttgcattcacgccttttagaaagagccgctaaattaaattctcttttaggggaaggaagtatgactgcttt
accaatagttgagactcaatctggagacgtttccgcctatattcctactaatgtaatctccattacagatggacaaatattcttatccgcagatctattcaatgccggaatt
cggcctgctattaatgtgggtatttccgtttccagagtaggatccgcggctcaaattaaagccatgaaacaagtagctggcaaatcaaaattggaattagctcaattcg
cagagttacaagcctttgcacaattcgcctctgctctcgataaaacaagtcagaatcaattggcaaggggccgacgattacgagaattgcttaaacaatcccaagca
aatcctcttccagtggaagagcagatagctactatttatatcggaacaagaggatatcttgattccttagaaattggacaggtaaagaaatttctggatgagttacgtaa
acacctaaaagatactaaacctcaattccaagaaattatatcttctagcaagacattcaccgaggaagcggaaatcctttgaaggaagctattcaggaacaactcga
acggttttcccttcaggaacaaacataaattttgcacgtctactcttgttagtagaagtagtagaggagaaatcgttgagaaagatttttcatttgaatcatgcaaaaaag
tttcttagtttttagtatagttatttaaagaatagatagaaataagattgcgtccaataggatttgaacctataccaaaggtttagaagacctctgtcctatccattagacaa
tggacgctttttcatattttcttctttcttttcatttttatcggaccaagagaaaaaactgttagaccaaaactcttttaggaaagaaaaacaaatgcatatacaaatgtatg
atgcatatatcataaagaaggaatatggagcgggtagtgggaatcgaacccgcaaccccacggttatgagccttgtcagctaccaaactgttctatcctgttaaacta
aagagaggggaactagtggataaaagggggggttgaatacgcccctctaccatatctatacaaatagaatagtccatttatacagaatggtaaagagggctcttctac
gatcatcaattccagaaatccatacaaatacgaaagggtattttatccttaccaactggatcttgttgcaccccggtaacaaacatgcataaaccatttctcgaagtatgtg
tccggatagcccaaagtctcgatagttagctctaggtcttccggtcaaaaaacaacgtcgatgaaggcgtgtcggtgcactattacgtggcagggattgcaatttttct
cgcattttcgtttttcactcaaactcaaagggtaaactttgcttcttatcttttttttgaagatcgacgaatcaaatgatatttctgttctaatttctgccgcttcctctctctctg
aatcaaactttttttgccataatgtgccgttcctactatctaccaagtatacggttctaatcctagatggaaaaataaatagaaaaagaaatctaagaaggcggatcctc
cccctccatcaagagtaatgaactaggtactgatacagtacaacaaaattaactaaattaaccaaacttgcctgatgttgaggcaatcaagaaagcggcataagtga
atatataacccacggaaaagtgagctaatcccaccaatcttgcttgcacaatggaaagagccacgggcttatctctccagcgaattaaattagccaaaggtgtccgtt
catgagcccatgctaaagtctcaattaattcctgccaatatccacgccaggaaattaagaacataaatccagtcgcccaaacaagatgtccaaataagaacatccac
gcccataccgataaactattcatcccaaaaggattatatccattgataagttgtgaagagtttaaccataggtaatctcttaaccatcccatcaaataagtggaggattca
ttaaattgtgaaacgttgccctgccataatgtgatgtgtttccaatgccaataaaaagtaacccatccaatggtatttaacatccagaaaactgccaaataaaacgcgtc
ccaagcagaaatatcacaagtaccgccgcgaccagggccgtcgcaaggaaaactatatccaaaatctttttatccggcattaatttggaaccgcgtgcatctaaag
caccctttactaaaatcaatgtagttgtatgcaaacctaaagcaatagcatgatgaaccaagaaatctccaggtcctattgttaagaaaagcgaattactattctcgttaa
cagcattcaaccatccgggtaaccatagggttcgacccgcattgaaagcgggaccgctcgttgaagataagagtatatcgaacccatatgtcgtcttaccatgagcc
gattgtatccattgagcaaatataggttcgatcaagatttgcttttctggagtaccaaaagcaagcatgacgtcattatgaacataaaggcccaaggtatggaatccta
ggaagagactagcccaacttaaatgagatatgatagcttctttatggtctaacattcttgccaatacattatcctcattctgttccggattgtaatctctaatgaaaaaaata
gctccatgagcaaaagcccctgtcatgatgaaccctgcaatgtattggtgatgagtatataaagctgcttgagtagtaaagtcttgtgctatgaatgcataagaaggta
aagagtacatatgttgagctactaaggaagtaataaccccctaaagaagctagagcaagacctaattgaaaatgaatcgaattattgattgtgtcgtaaaggcccttatg
cccacgccctaatcgaccccccggaggagtatgtgcttctaaaagatctttgatactgtgcccaattccgaagttagttcgatacatatgaccggcaatgagaaaaat
aaatgcaatagctaaatgatgatgagcaatatcggtcagccacaaactttgtgtttgtggatggaatcccccaagaagagttagaatagcagttcccgctccttgagt
ggtaccaaataaatgattactcgaatcggggttttgggcataaagattccactgacccgtcagaaggggtcccaaccccctgtggatagggtaatacatctaagaaatt
attccatcgaacgtactctcccctggatgctggaatagcgacatgaactaaatgtcctgtccaagccaaagaacttaccccgaaaagtcctgacaaatgatgattga
gacgagattccgcgtttttgaaccacgaaaggcttggtttccatttgggttgtagatgtaaccaacccccctattaaggatagggtagaaagaaataatagaaaaagag
ctcctgtataaagatcttcattggtccgtaatccaattgtataccaccactgataaaccccagaataggcgatattcactggaccggcggcgcctcctcgagtaaagg
cttccacagcgggttgaccaaaatgaggatcccaaatcgcatgagcaataggtcttacgtgtaaaggatcctgtatccatgattcaaaatttccttgccaagcgacat
gaaacagatttccggacgtccatagaaagattattgctaattgcccaaagtgagaagcaaaaatgttctgataaagacgttcctcagtaatatcatcatgactttcgaa
atcatgtgcggtagcaataccaaaccaaatacgacgagtagtggggtcctgagctaagccttggctaaacctgggaaatcttaattccataatgcctttcaaatcctc
ctagccactatcctactgcaataattctcgctaagaagaatgcccatgttgtggcaattccacccagaaggtaatgggttactcctacagcacgtccttgtataatgctc
aaggctctaggctgagtagcaggagcaacttttaatttgttatgagcccaaacgatagattcaatcagttcttgccaataaccacggccgctgaataaaaacattaaac
tgaaggcccagacaaaatgagcacctaagaaaaaaagaccatatgcggataatgaagaaccataagactgaattacttgggatgcctgtgcccacaagaaatctc
ggagccacccattaatcgtaatggaactctgtgcaaagtttccccctgtgatatgagttaccaccccttgatcacttatagtaccccaaacatccgactgcattttccaa
ctgaaatggaaaatgactaccgaaatggaattgtacatccagaatagacctaagaaaacatgatcccaggcggatacttgacatgttccccctcgcccaggcccat
cgcaagggaagcgaaaaccaagatttgctttatcgggtatcaaacgggaactgcgagcaaataaaacacctttcaaaagtattaatacagtcacatggattgtaaat
gcgtgaatgtgatggactaaaaaatctgcggttcctaatggaataggtaacaaagcaactttgccgcctactgctactaactcgccacctccccacgttaagctggta
cttgttgttgcaccaggagctgttacgctaggcgcgccagcatggagattttgtacccattgagcaaagatcggttgtaattgtatggcggtatccgaaaacatatctt
ggggacgacctaaagcactcatggtatcattatgaatgtataaaccaaaactgtgaaaacctagaaatatacatacccagttaaggtgggatatgattgcatcacggt
gtctaaggacacgatctaatagatcgttgtatcgagtcgttggatcatagtctcttaccataaaaaatggctgcatgtgcagcagcaccaactattagaaatccgccaat
ccacatgtggtgtgtgaacaaggaaagttgtgtaccatagtcagtagctaggtatggatagggggggcatagagtacatatgatgagctacaacaatggttgtagagc

ctagcatagctaggttaagagataattgagcatgccatgacgttgttaggatttcatagagacctttatggccctgtcctgtaaatgggcctttatgagcctccaaaatat
ctttcaggccatgaccaataccccagttggtcctatacatatgacctgcgatcaggaaaagaatagcaatagctaaatgatggtgcgcaatatcgctcaaccataga
ccgccggttattggatctagccctccgcgaaaactaagaaattctgcgtatttggaccaattcaaggtgaaaaaaggggttgctccttcggcaaaactaggataaagt
tgagccaaaaggtcgcgattcaagataaattcatgaggaagtggtatctccttaggatcaaccccagcgtcaagaaattggttaatcggtaaagatacatggatttgg
tgccccgcccaagaaagagacccaagtcctaataaccctgctaagtgatgattcaacatggattctacgtcttggaaccaggctaatttgggagcggctttgtgataa
tggaaccacccagcaaaaagcattaacgatgcaaaaatcaatgcaccgattgcggtacaatagagttgtaattcactagttattccggatgctcgccaaatctgaaaa
aacccggaggttatttggattcctcggaaaccccgcctacatcaccattcaatatttcttgccctacgattggccaaactacctgagcactgggtccaatgtgagtag
gatcacttagccatgcttcataattggaaaaacgggcaccatggaagtacatgccactcaaccaaagaaagataatggagagttgaccgaaatgagcactaaaga
cttttcgagagatctcctccaaatcacctgtatgactatcgaaatcgtgagcatcagcatgtaggttccagatccaagtggtagtatcagggcccttagctattgttcttg
agaaatggccgggtctggcccattcctcaaaagatgtttttacaggatccctatccacaacaatttttacttctggttccggcgaacgaatcatcattaagtcctcctcttt
ccggacaagacatacaaagagacccgccaactttttagtgaacctttgaaagatagatattatgattagtccttttctttactatctaccgtccttctattttttttagttattc
actggagcaattatatattgaagtcaatctgaggcaagtgttcggatctattatgacataaggattgggtgcctaacggactttttttatcttggatttctccacgtaacaa
aaaaaccttttttaatttaaaaagctagtctattttttttaagagtataagtccctatctatatctactttccttgagcataatatagattttttttattcgattccaaattccaagat
aactcattagaattattaataagatggtcctgatatattagcaatatttatattgtcccttttttattcgctttattacttctattctagaccctatcgtttatccttatgaaatataata
taaaatagaaggcagaggaaagagatataatgaaattcttgattcgatcttccgacctaatttatttgattaatggatcaacaaccaaaccccccattttctgaaaaagga
gagtggtcttattcaaattcaaagcgcttcgtaatcttcaaccagttctgtgcttcaatataatttcccggagtaagcgctatagcttgtttccaatactcagcagcttgatc
aaaccaagcttctgcaatttctgaatcaccctgtagaatggcctgttctccccggtcggaataggtagttccttcccctagaaccgtacttgagagtttcctacctcatac
ggctcagaaattgctatcttaatttcccttgtcttaactgaattcgatttctcaaaaatcgatccaatttcttcttgggttacgcagaagaagttaattacctaagtttcaaac
cctaaattttgatcaataatcagtttgatcttttctcccacctgcagaaaaatgaagcatagatagacctatatccttcgtccgaattttctgaaaggtaactatctcggtttc
atatatgaaatttctatagaatccttgaaaaagactttttccccataagcaagaaaaaaagaacttactatctttgggatctgatactacaccgctgcttaatcccttagtgg
atcggctctattacataagcggattcctaaattttgccccatatcatgggataagtaagcagttttttttagttgtatcgacccagtcggtcactaattgatctttacggtgct
ttctctatcaatttgagaactctatccatagagtagtataggccatactttctttcttcctattttgattctcgtgaagtgtcctttcttcctacagctgatagggcaaaatcgtt
gttttgacgatccctatgtagaaagccccctttttctagtaaatactagaaaatttgatcctttcttatttttctttctatagtggagatagtcgcacgtaatgacagatcacgg
ccatattattaaaagcttgtggtaagaaggggtttcgttctagtgcccggaaataatattccaaagcctttgtatgctctccattgcttgtgtgtataaggcctatgttatag
agtatataacttcgatcatagggatcaatttctagtcgcgtagcttcataataattttgcaaagcttccgcataatttccttcggattgagccaacatccgttacggtcgttc
gttctattcaaaaaatctccgttccaaaaccgtacatgaggttttcacctcatacggctcctcccttctgtacatagtactaagcgaaaaaatctagagaataaaaataga
attagttccatctcattatggaccgaaaggggctggtatttttccaagaaatctctagccaaccttcccacaagaggtttttcttaacaccaatgaattctattaatgctag
aggaaaacgatagctccaagaatttctttgttctcaacgcctcctatttagaggaattagccacttcaacgatctttgatggttataggggtatccaaagtacaaacttga
tggttgtttgttatcccaaccattcttcccagccctgataccaatcaggaaagggttaatttctaacaaagtttttctcttgttgattcctatttctaggtgtagtgcttttatcc
cctatgctacctattagtactagtagagtaggattagcctgtaatacagaacctatcctgtaggtgtaacctttcgctcaatactaaaatctacaattgaagcatccgagg
ccgcgtcagtcgaggatacacgacagaaggagttgttagttcacctcaccttccctaagcgtgggtttcccttactaatttggttttctctccgcgaacccccgctctttc
tcgtaaaaccggggtgtaggtagggctaaaaaaacaaaaaaaagagtcaaatcgcaccatctctataataagtaaatgcccttttttccccggaggttgtcggaatta
ttcgcaataaaatattggctacaattgagaaggtcttatcaatgaaatttccatttatacgggatctaggcataattcccaacccattctatcattctatatagaattcttttca
ttccttcacaaaataacataaaaaaaaacaaatccattcaattcttataaatcgatccctatgctccaaatggataagagaggtatttctgctcagcccaaattctctctttt
tccttctgtttgaacaagaagagatagaaaatatttgactaagattggatttcattccactttcctatttcttttctcaacaacaacttctctcatcaactatttcgcattttcaaa
gtcattaatcgtcccatacccctattttctatttgattgtatggggtaggataccttatgcaaacagaattctagggttcctttttttatcgaataagaagaattcttccattctctt
tttctttggtttggggaaaacccaaactaaaacttttcgagggagcggaattcctagtaaaaaaatcctggacccgtccacttagatgaaaaggaatttttctatctaata
gaacaatggaactctcgcgcggttgtggttgtacctgtactgcaggaataggaaaactcgctattcactcagtttttttttccataataagattatggaggagagatggcc
gagcggttcaaggcgtagcattggaactgctatgtagacttttgtttaccgagggttcgaatccctctctttccgtttttcttaattcagcaacgttaatgattacaatgtat
caaatcaaatgacaatttattccagcaataatacaatacctttatttaatagaaattctctataccaaattactatggtatgtaaaatacacatagaggaaagaacaaaaaa
caaaaaggaatcctagggttaatccatttctgttaggtgaatgggaaaatacgaattaagagccttaggtcgatttagttcggggaaaggggaaggggaagaaaatt
ctatgaacctttccgtttttccttaagttcaagtttgacgagagtaatattctacaactaacaactcatttattttgagaccgacccacttcctatccaggattttttttactagt
cctttatattgcaatgtgtcaatcgtcaaatgctttggcaatttccccgggtcggatgaagcaatagaattttgaaccagacgttttgatctttggttatccttcgtagtaata
atatctcggggtttgcaacgaaaacttggtatatcgactatacgaccattaactaaaatatgtctatggttaactaattgccgggcctcgggaatggttgaagccatacc
taatcgaaaaaggatattatccaaacgcatttcaagtaattgtagtaaaacctgacctgttgaactttttgcttttccagcgatatgtacatatctaagtaattgtcgttctgt
cagaccataatgaaaacgcaatttctgtttttcttgaagacgaatacgatattgttctttttttcccagaatggaatttctttttcagattacttccggatttaggtgtttttctagt
gagtcctggtaaagctcccagacggcgtatttttttaaaacgaggtcctcgataacgggacatgaagactcctttttttattgaaatttcattttacacaattaatttcattgt
atttacattacagaatacatcgaaattaaaactgaatttaaagtaaaggataaacagagtaaaatctactaaaagtaccacaaaaaaaatggaatttcatcaacatctgaa
tttttgtatatatattatttattttattgttttgtatctagcaaaattgtagggtagaacgacataatagatcctggcttctccatttaattcggagaaaaagaggtatttttgttca
tggaacattgatagagaaaaaagccgactatcggatttgaaccgatgaccctcgcattacaaatgcgatgctctaacctctgagctaagtgggcttacataacagaa
atagtgtaacaaatagaaatatgtatagtataggaaatccgtaaaatgtcagatcttaattattaatcttagctattaactagttcgaaattggaagttctacttagaaaaaa
atactagaacttcataaaataaagttagatagattttttgaactttcttttttctctaattcgcaaatctatttttctaatagaatctattccaatttctatattgaatttgattttcga
tatttttaatttgatatggctcggacgaataatctaatacatggaaaagaataaataatatatatacgaaaacataataaagagaacatgcgaatttcttgtattttcagtcc
atcattatagacatttttttgagatattttgtttttttttgttatttgctaataatttaatgattaatatttcactaaggagaacatagaatcatagcaaatgaaattgctaattctgat
tagcaaaaaaaaaaagaatgaatatcaagcgttatagtatgattttgaatactctaaaaaaggaacgcggtaggtgggggagagaaaaaccttgggatatattgattc
gcattgaattgcaaatacatcaacgatagaatcaattcaatgctgaattgcaataagcggagtctctcaactagagacgaaccgctagactacatagagtaatgaatt

caacgattcaaaaaaaactaacagatggaggaaattgcacaaggaatcctggtctcaaagaaaaagaaaatggggatatggcgaaatcggtagacgctacggac
ttgattgtattgagccttggtatggaaacctgctaagtggcaacttccaaattcagagaaaccctggaattaaaaaaggggcaatcctgagccaaatccatgttttgag
aaaacaagcggttctcgaactagaacccaaaggaaaaggataggtgcagagactcaatggaagctgttctaacgaatcgagttaattacgttgtgttgctagcgga
actcccttctaaattagggaaagaagggctttcgaaatctaatacacacgtatagatactggcatagcaaacgattaatcacagaactcatatcataatataggttcttta
attcttttttaaaatgaaaataggaatgattatgaaatagaaaattcataattttttttagaattgttgtgaatccattccaatcgaatattgagtaatcaaatccttcaattcata
gttttcgaaatctttttaaaagcggattaatcggacgaggataaagagagagtcccattctacatgtcaatactgacaacaatgaaatttctagtaaaaggaaaatccgt
cgactttctaagtcgtgagggttcaagtccctctatccccaaaccctcttttattccctaactctagtatttatcctgttttttttattaataggtttaagattcaatggaatacat
ttctttttttattatagtatcggcaaggaatgtcgattattaactcgatatttaaatattattaaataggctttctttgtacaatgcataggactgccccctccccatttccaaattt
tggatattgacatagatacaaatactctactaggatgatgcacaagaaaaggtcaggatagctcagttggtagagcagaggactgaaaatcctcgtgtcaccagttc
aaatctggttcctggcacagaaaaaagggatcttccgaataggtattgatacaaatacctcgagatgggttgggatacatattcgttaataatatagatagagtatgatt
ttttcatctaagtagataaatctctaaatagaggcacttctttttctgcatttttgcatttcttaattttctattccgctattccgacaaatattttttttattcttgcttatcttatcttact
ttcctagttgttctaagtaatgcacgcggtacaaagttcgtggtagggaacttctttgagtcatcatatttttctgttcatacgaaggaaatgaatatgtgattttccaacga
aatgaagcccttttttgcttagtctatctgaaccttttgtataattggaattaattagaatgtaataggtattctgtttcatctaggaacagagcgtaaaaatattccttgactt
gcataaaatctggagttgtgttgtataagtgagcatgaatttcttatcattcaatgagcatcttgtatttcatagaaattgggggttatatagtccttacgtaagggccagc
ctatccaactttcaggcattaggatacgtttaaggcgtggatgattatcataagagattcccaccatatcaaaagattcgcgttcttgaaaatcggaacttctccaaatcc
agaagacagatgggattctaggattatccttttgggcaaagacttttatgcatacttcttctgggttatctataccatactgtattctcgtaagatgatacacgctagctaaa
gatccaccgggtgcaacgtcataagcacattgggaacgtaaataattgtaaccatatacatataaaatgacagcaatggaatcccaatcctctgcttttatttgcaaagt
ctctattcctcgatgatcgaagcccaaagatctatgaaccacctcatgtttgactagccaattagataaccaaccctgctgcattatcttgatctctcctcctttgtataaat
atttcgcagttcgaatgcaagtttgaaatattgccctgctctttctttttcggcacaaagagctcctcctaattcactaatttgtaggaagatactggacttttggatttgaaa
aaagtttcagaagatatgtctaaagtagatggtgattgatagagcaattcttgctcataagttccagtattagtactgcgccgaacataaagcttgtggctggtagtaaa
acatcgatttttcttttgagatagagttcgatcctcaactatttctcgcgatatcttcttacgaagttttgttagggcatctataacagcctctggtttaggtgggcaacccg
gcaagtagacgtccacaggaattaacttatcaactccccgaacagtactataggaatccgtactgaacattccaccagtaatagtacaagctcccatagcaatgacgt
attttggttcaggcatttgctcatataatctcactaaagagggagccattttcattgttactgtaccggctgttaaaattaggtccgcttgcctaggacttgatcttggtacc
aatccataacgatcaaagtcgaatcgtgagcctattaatgaagcaaattcaatgaaacaacaactggtaccatatagaaggggccataaactagagagtcttgacca
attcgaaagatcttttagtgtagttgaaataacggaattggaacttgtttggtcaagtaggggaaactcaatcaaactcataactgtcttaatggaatcttttccttcttttttt
tttttgtctgaatattcagttaagaccattccaaggctccttttcgccatgcataaactaaaccaacaactaggataagcacgaaaatgaaagcttcgataaaaacggat
atacccaatacgtcgaaactcattgcccaagggtagagaaagaccgtttccacatcaaaaacaacaaaaactagcgcaaacatgtaatagcgtattcggaattgtaa
ccaagcccccccccatgggttctatacccgattcataactagaaagcttctctggtccttcacgaaccggagctaaaagtgctgaaatccaaaatgctaaaataggaat
aaggcttgctattattagaaatgcccaaaaaaatatcatattcgtgaagcagaaacataaatgtactcccattaatgtggaataggcggaactgaattagtcaattcaagt
cagcattgtcaatttatacagaatttctctcttttcctcggtgaaacaaggatcggtttttctcaaaccaaagggcttagtttagcctttgtttcctctttgccacgtcttcttta
aagattcatccaatggaatcccgactccctttctttttgatttcctttctatttaggtatggtggagacataattcttatagaaacaaaactctctcgcttcactttgtctcatttt
ctctagaatctctagaaaaaggaataaaaacgaaaatactacgaattagagcctaattaagataggctgactaatgtatgcagcctaatgaggagtaattctataaaaa
taaagaactctatttcagaacgtagatcgatttagatttaggtaatctatagatatagataagcaaagtaatatacttcaaacaaagtaggaattcgcaagatggagaac
atcttgcagttgatttgatagaaattcatttttcttttcctgtctctataattttcgatgaatgagcctctggtaatccttttatctctattttatggcgcaggcgcctgtccagtct
ataaacaagtactaatagggaaatgaaaactatactaaaggaaacgtaggatatctctcctaaaatataaaaaaggacatattagggctatacggattcgaaccgtag
accttctcggtaaaacagatcaaacggattattatcgaaatgattcgaactgtttcaaagacccaacatgcattttttttgcattgggctcttttatcaactgatagaaagat
cagttagtccaccatagtttttctttacggaaagataatgagatggttccctgcgctctgattgattatttgtattatgatctatctaagagcaataccaaagtgtttcaaag
gaggattaccttgacttaggtctgcctccggcctaaattaaatcaacctaagtgaaatagagtctctatcgttccgctacaagagttgactatgagacttcatacacctta
aagttcatagaacgaaaagaatttttttggaggcccttatcctcattaagcctagcatttagtgggctggatatttaccttatcaactagcaaatcaataaaggttctatttg
tttaggcacctggattggtacctgaatcggactgaaccaactatttgtcaggcgactgttctcctattctctcgaatccatgaagtaagacattgattttgcaagaagatc
cactatgttcattgcataataagctcccttgaaaagcattggcgcacgtgtaaacgagttgctctaccgaactgagctatagcccttgtcagagatatcttaatatatag
agaatttcttgtcaagatgaatattctctaatagtagaggatatcctttgatctgtttactataataacataccaataacgaagcggtatttgcttataaaaggattcgatct
ataatcgatcgaagtaaagggtcttcctttgtggtgataaattgcctacttaactcagtggttagagtattgctttcatacggcgggagtcattggttcaaatccaatagta
ggtaggtaggtagaaaaattactagatagcattggccctacttcgcttcgctatctaataattttttctacccctcttccctttttctttgtatcaactaaaccgttgggttgtct
tcaattagatgggggaatccaattaacagcctcgactcgtatcctagctcgtctgagagctaccttcgcttcaaccaattctttcgtaccctcagctctactcacgttagc
ttcggctatttcaagtgcctgttgagcttcttccggatcaatgtcactacccagttccgcatcatttcctaaaatgatgatctcattattaactattctggcaaaaccgctcc
acagaaccgccgttaaccattgatcgttgaggaggcgtattctcaagggacccatatctacagctgtgttaatgggggcgtggtttggtaatacgccaatttggccac
tattagtagataaaatgatttctttcacttcacaatcccaaataattcgcttaggagtcagtacataaagatttaatttcatttcttcaatttgttctcctcttctaagtttatagct
ttcgtgctagcttcatcgatgttacccaccaaataaaaagcctgttcgggtaggccgtctaattctccggaaaggattagttgaaatcccctaatagtttctgcaagacc
aacatactttcccggagaaccggtaaaaacttctgccacaaaaaacggttgtgataagaagcgctcaatttttcgtgctcttgctacagttaaacgatcctcctccgata
attcatccaacccaagaattgcgataatgtcctgaagttctttgtaacgttgtaaagtttgcttaactctttgcgcagtttcataatgttcgttgccaacgatccgaggttgt
aacatagttgaggttgaatctaaaggatctactgcaggataaatccctttggaagctaatcctctggaaagtacggtagtagcatccaaatgtgcaaatgttgtagcag
gagcagggtcggtcaaatcgtccgcaggtacataaaccgcttggatcgaagttatagatccctttttagtagaagtaattctttcttgcaaagaacccatttctgtactaa
gagtaggttgataacccactgcagagggcattctccctaataaggcagatacctccgatcctgcttgaacaaaacgaaagatattatcgatgaatagaagcacgtctt
gcttattaacatctcggaaatattctgccatagttagggcagtcaaaccaactctcatacgagctcctggcggttcattcatttggccatagactagagctacctttgatt
cctcaagattttttcattaattactccagattccttcatttccatataaagatcatttccttcacgagtccgttcccctactccgccaaatacggatacgcccccgtgagctt

tagcaatattgttgattaattccatgatgagtactgttttacctactccagctcccccaaatagtccgatttttcctccacgccgataaggagctaaaagatcgaccacctt
aataccagtttcaaagatggataatttcgtatctaactcgataaaggcgggcgcggatctatgaatagggaatgttgcactagtatctacaggacccaaattgtcaaca
ggctccccaagaacgttgaaaattcgtccaagagtagctccaccgacaggaacactgagaggagctcccgtgtcaatcacttccattcctctcatcaacccatctgt
agcactcatagctacagctctaactcgattatttcctaataattgttgtacctcacaagttacattaatttgcttaccgtcagtgtctcgactcttgactaccaaagcattata
aatataaggtaacttgcccgggggaaaagtgacatccagcacgggtccaataatttgatcgatacgccctgtactttttttcttcaattgtagaaaccccgggacgaga
agtagtaggattggttctcataattatcacataattttcaaaaaaaaaggaatttatcgaaattttgatttttttcttgttgaataatgccaaatcaacaccaaaaaaatatcca
aaaatccaaaagtcaaaaggaaatgaattagttaattcaataagagagaaaaggggaccagcacttgatttcgttgcccaaacgaatcccattcaatcgtttactcatg
gaatgagtccgtcggaaagttcaatcaatcttttttcatatacattttgccttttgtaaacgatttgtgcctactctactttcttatctaggacttcgatatacaaaatatatact
actgtgaagcatagattgctgtcaacagagaattttcgtagtatttaggtatttccactcaaaataagaaaagggggtctattaagaacttaataaggattagaagttgat
ttggggttgcgctatatctattaaagagtatacaataaagatggatttggtgaatcaaatccatggtttaataacgaagcatgttaacttaccataacaacaactcaattct
tatcgaattcctatagtagaattcctatagcatagaatgtacacagggtgtacccattatatatgaatgaaacatattatatgaatgaaacatattcattaacttaagcatgc
cccccattttctttaatgagttgatattaattgaatatcttttttttaagatttttgcaaaggtttcatttacgcctaatccatatcgagtagaccctgtcgttgtgagaattcttaa
ttcatgagttgtagggagggacgtatgtcaccacaaacagaaactaaagcaagtgttggatttaaagctggtgttaaggattataaattgacttactacaccccggagt
acgaaaccaaggacactgatatcttggcagcattccgagtaactcctcagccgggggttccgcccgaagaagcaggggctgcagtagctgccgaatcttctactg
gtacatggacaactgtttggactgatggacttaccagtcttgatcgttacaaaggccgatgctatcacatcgagcccgttgttggggaggataatcaatatatcgcttat
gtagcttatccattagacctatttgaagagggttctgttactaacatgtttacttccattgtgggtaacgtatttggtttcaaagccctacgcgctctacgtctggaggatct
gcgaattcccccctacttattcaaaaactttccaaggtccgcctcatggtatccaagttgaaagggataagttgaacaaatacggtcgtcctttattgggatgtactattaa
accaaaattgggattatctgcaaaaaattatggtagagcatgttatgagtgtctacgcggtggacttgattttaccaaagatgatgaaaacgtaaactcacaaccattta
tgcgttggagggaccgttttgtcttttgtgccgaagctatttataaatcacaggccgaaaccggtgaaattaaggggcattacttgaatgcgactgcaggtacatgcg
aagaaatgattaaaagagctgtatttgcgagggaattaggggttcctattgtaatgcatgactacttaaccgggggattcaccgcaaatactagtttggctcattattgc
cgcgacaacggcctacttcttcacattcaccgagcaatgcatgcagttattgatagacagaaaaatcatggtatgcatttccgtgtattagctaaagcattgcgtatgtc
tgggggagatcatatccacgctggtacagtagtaggtaagttagaaggggaacgcgaaatgactttaggttttgttgatttattgcgcgatgattttattgaaaaagatc
gtgctcgcggtatctttttcactcaggactgggtatccatgccaggtgttataccggtggcttcagggggtattcatgtttggcatatgccagctctgaccgaaatctttg
gagatgattctgtattgcaatttggtggaggaactttaggacatccttggggtaatgcacctggtgcagcagctaatcgggtggctttagaagcctgtgtacaagctc
gtaacgaagggcgcgatcttgctcgtgaaggtaatgaaattatccgatcagcttgcaaatggagtcctgaactagccgcagcttgtgaaatatggaaagcgatcaa
attcgagttcgagccggtagataaactagatagctagactaagtggataaaattagatagaaaaaaggtctaaataaaaaagaagagaaatagaaagatcaaaaat
cagttacgaaaatgcagtaattcttctttttcttctaattgattgcaattaaactcggctcaatctttttcagattgagccgagtttaaatagattttgatacgatcatgagac
ttgacaaatcgggattcctctattctatatatttagaagatataaaggtataatacaataaataaatacaaatatagtattatcatatgataatggaatcaaatacgcagtatt
tacagaaaaagttttcatttattgggaaagaatcaatgacatacaatgcattacagacgtatgatcattaccctttaaccgggttattctattccacttctagatagagaaa
aaaactaaaggagaatgaatgaaaaaagacagagtttggaagttagacccccctttctaagactctctttcaaaaaagaggacattttgaaacttttaacaggcacaatc
gtgagtcaacaagtgactcgaaatgctcgtaagaaaagagaattgattttcaaaatggtagaactagatgacgaagttttctataaccttgatgaagaggtagttttagt
ttacgactccgatcaagagcgagaagtctttcatttcagattggactgctatagaatctggacccatcgtagagacgttcaaaaggctcctgatgataagaatcatact
ttcgcggaactccatggggctatgggcttcaacgcggtagacgttttgttccgaatttttctggagcaaacctccgacccaacgatacaatgaatttttttctattcacaaa
gaaaaaagattcggaatcgcaatgctactatacgcacccggaggaatattcggaattatattcttatataataatccatttttgcgcggggtcttactaacaggacttcg
ctgcacgagacgaggattcgtcgaaaagctaaggtcatccggtattttcataccctttgggggtggtatatggccttacaaagtctaaggggtagtatgagatctgta
gtaggtaaaagaatttgtcccttgattgagtatgctattttccctcctttaccgcgcattattgtatatgcttctagaagagcacgtatgcagagaggaaattacagtttaat
aaaaaagcctaaaaaagtttcaactttacggcaatatcaatcaactaaaagtcctatgtatcaatccttacagcggatttggggggtccgagagtggttgaataagtatt
gcatgtggaaggaagtagacgaaaaagattttggattcgaaataggcgcattcgactaagtcgtactttgaatccaatttcaagttcgattagaaggatagaaagggc
gcgaggatcggaaaagaaaaatcaaatctttttaattgcttctcctttttttgctattttcttatttatataatccattcgattcttttttgcaaactcaaaaatacaatagtca
atattcgttataatagatatacttaattatatcataagaatcttaagatattttttgaatagatcaaatcgaatagatagaaatagtaaatttgaatggagacacctattctatg
atggattttaacttaccctctattttcgtgcctttagtaggcttagtatttccggcaattgcaatggcttctttatttctttatgtgcagaaaaataagattgtctagaaccgac
gggaccgaattttctcaatgtatttccacacaggatcataatacggatcttttgtagtgtaagtaatataatatggtatgttatgtggctctttctacacacaaatgcaaacc
cgctatggatggggattatggatgcggatataggctacgagcataaatgcatgcatatgcggaaccgggtatagcgagttttttttaagtggatcaacaaatactttttg
aatagaaagtcaatgtatctaaccaattattttacaggagtatctagttggcgaaggcgatttcagaatcaaaaaaagtaaagtcaaaatcatttagcttattctctcaatt
tcaatcgaccgctgttagtatatctaatatgaattggcgatcagaacacatatggatagaacttctaaaaggttctcgaaaaagaggtaatttttttctgggcctgtattcttt
ttctaggttcactaggattcttagcggttggggcttccagttatcttggtaagaatattatatctgtacttccatctcaacaaattctttttttttccacagggggtcgtgatgtc
tttctacggaatcgcaggcctattcattagcgcttacttgtggtgcactattttgtggaatgtaggcagtggttatgaccgattcgatagaaaagagggagtagtgtgca
tttttcgttggggattccctggaataaaacgtcgcgtcttccttcgattccttatgcgggatatccaatcaattagaattcaggttaaagaaggtctttttcctcgtcgtatcc
tttatatggaaatccggggccagggggccattcccttgactcgtactgatgagaagtttttttactccacgagaaattgaacaaaaagctgccgaattggcttatttcttg
cgcataccaatggaagtattttgagtaccaattgaattttttgaaatgaattgaatgaagaagaattggaagaagaaaagttttctcaacacgagggaaaaggtccctt
cgaaattgcattattgtaaggggattttgagtatttatctaaaggaaggaacaaatgaggataagagaaaattgcttctaatttgtccaagtgggatatatggcgtaatat
tcttccattttcatccgaaaggactttttttctatttctctattccactccatctagatctaagaaagaactcaatgcaatgaaattctactagtatagaaaaaagaggaatag
atacaaggtctcaaaccttgttatagaatttttccttcaaagaaaaagaaatatcatatagagtcagcgaataaagcggattcattaacaactcaatttacagatcaaaaa
tgaaaaaaaagaaagcattgccttctttcctatatcttgtatttatcgtacttttgccttggggagtctctttctcttttaacaaatgtctggaactttggattaagaattggtgg
aataccaggcaatcccaaactctcttaactgctattcaagagaaaagagttctagaaagattcatggaattagaagaccttttttatcttggacgaaatgataaaagaga
aaccgaatacacatgtacaaaaccccccctataggaatacgcaaggaaataatacaattggccaaaatagataatgagggtcatctccatatcattttgcatttctcgac

aaatataatctgtttggctattctaagtggttcttttttctgggtaaagaggaacttgttattttgaattcttgggttcaggaattcttctataacttaaatgactcagtaaaag
cttttttattcttttagttactgatttttttgttggatttcactccacccgcggttgggaactactaattcgttgggtctataacgatcttggatgggttcctaacgagctaatttt
cactatttttgtttgtagttttcctgtgattctagatacatgtttgaaattttgggtctttttttgtttaaaccgcctatctccttcgcttgtagtcatttatcattcaattagtgaagc
ataaactcatttgatctcctgatattaatcaaaattagcatcctttttcctttagaaagaaagcctttttccattttagcaaaattctttttttctatttctacctgctcaaggtattc
atcattccagtacaactgttgcagtagaatgacaacagattcgtgtatagggaactagattagcttagctacctatctaatttattgtagaaattccgggatctgtgattgg
acatggaaaatagaaatactttttcttgggtaaaggaacagatgactcgatcgatttctgtatcgatcatgatatacgtaataactcggacatctatttcaaatgcatatcc
catttttgcgcagcaaggttatgaaaacccacgagaagcaactggacgaattgtatgtgccaattgccatttagcgaataagcctgtggatattgaagttccccaagc
agtgcttcccgatactgtatttgaagcagttcttcgaattccttatgatatgcaactgaaacaagttcttgctaatgggaaaaagggagggttgaatgtgggtgctgttct
tattttgcccgagggattcgaattagcgccgcccgaccgtatttctcctgagttgaaagaaaagataggaaatctctcttttcagagttatcgtcccaataaaaaaaata
ttcttgtgataggccctgttcccggtaagaaatatagtgaaatcgtctttcccattctttcccccgaccctgctatgaagaaagacgttcatttcttaaaatatcccatatat
gtggggggaaaccgaggaaggggacagatctatcctgatggtagcaagagtaacaatacggtctataatgcaacgtcaacaggtgtagtaagaaaaatactgcgt
aaagaaaagggggggatatgaaatatccatagtcgatgcatcggatggacgccaagtgattgatcttatacctcccggtccagaacttcttgtttcagagggggaatc
gatcaagcttgatcaaccattaacaagtaatcctaatgtgggagggtttggtcagggggatgcagaaatagtgcttcaggatccattacgcgttcaaggccttttgttc
ttcttcgcatccgttattttggcacaagtttttttggttctcaaaaagaaacagtttgaaaaggttcaattgtacgaaatgaatttctaggtcccgggatttcttaccatcaag
ttggtaaaaagccgcaatttattggcgattgctagaattctctatgatccattttggaaatctttttttgtttagactatttttttgtttgcgagatgtcttgtctgcaattccttatt
tctatcatctcatgcaaaacgaggagaatgcaaggcaagggcgagaacaataaaaggaacaaatccttttaggagggattgctggtcttcttaatcctctattgggca
caagaaaaaggctttttttgcctttttcttgtgtcgattcttcttgtatcgaaatatgaatcttttttcttcctattcggcaaagattactatttcttctttatttgggtctgtctcttga
acctcttttgcttaggttcaggcgatgtagtggacaaaaagagggaaagaaaatatggcgggggacaaatttttttgtgaccaaatagaattgcttgacttgttcaattaa
gttcaatttagaacttacagaaattttgcaaaaaaaacggatactcttccattgaaggctgttttttatttttaggctagttgagtagttttaattaaggttttattagtttattact
ctaaattaaatcaatgatttacaagagacttcctccggggaataaaatattggatcctcgattgatcctttctttctcctcgcttcataaaagtgaattaatttcattggcga
gggggttataaatcaactgatggattacttcactaacattattaacaaacaaaattaacaaacaaaacgaataaatagagggattctgaccatcagagcaaaggctttc
tctttgttatttttacaaatcaaaataggaaacccgtttgtaggttatggaatagattaaaaaagtcgggttataagagtaagaattccgcgggtcctttccgctctaatca
gataaagggggggtaaggacccgctaagttcctattttttcatgtttacaacctggtccctccaattactatagagatgaacccaatccagaatatgaaccgtaaaagaa
aacacctattaaaccaatcacagcaataccggttacagtacctatcagccaaagaggaattcttccagtagtatcggccatttcccctactttcctccacattttatcaag
tggtcatgctagagacaaaaacagtcatggatagttataaggatggtatccttccaaatggggataagagaattcttactactctctttctttctctcaattgaagaagta
attggaaaataaaacagcaagtacaaaaatgagtaataaaccccagtatagactggtacgattcaattcaacattttgttcattcgggtttgattgtgtcatagttctatag
ttggaatttggtttatcgttggatgaactgcattgctgatattgatcccaagaaaaaaacagtaggtacagctagtccgtgaacagccagccatcgcactgtaaaaata
ggataggttcgatctatggtcattgagggcctcctaaaaggatctactaaattcatcgagttgttctaaagaatcaaaacggtcggttattaacggaattccttgtcggct
ttctgtgaaatactcgtttggccgaggacttccaaacacgtcataagctaaacccgtactgacaaataaccaacccgcaatgaatagggaaggtatagtaatgctatg
aataacccagtatcgaatactggtaataatatcagcaaaagaacgttctcccgtgcttccagacatgctgagctccccaaatttttgtacattcaaaaaaggaattgatt
ccgtaaaagatgggatcaaccagtaaatagaaaattactgatatttcatccttgtgagattgtcaattttgtaccaaaggtgtattttgagtataccaaattagtatagctat
ccttcctatggcacagcaatcctgtttcgcttggtctcgaaacagaattcttttttttctcttctttgttccttgtctatagggtaagctatatggtattcaaggcatcaatagaa
aacctcaattttgagggtcctacttaattttcaccggcttcggaatagtagaataattcggaatagggctcaagatcttgggaaaatctaagttaatgatcaacaggtttg
gataaagaatttaggaaagatattcttatactgacacaatacaaagagaagtagatgcgaaagctatcccttcgaatccaacctttccccttaaagaatttaattggtta
gcataatataatatctaataaatagaaaatcaaatagtagataatctgttatgaaagagagaaaacattctttgaagaatcaagattcgtaatcaacccttgccttgtttac
taactttcttgaccaaactgcaagcgtggaactttgccagttcttaggtttccgaaatagtgtaatggaaaaagaagtgcttcgaatcgactttgattggggttcgaaaa
aggaattaaaaaaaagtaaattcaggaaagatttccttttttttaggagaccctcgaggggtcgtggaatgcttttcttctcctcttattccatatggaatacaatcaattaa
aataagaaggaataggggaatattcgactgttcgctccaaaaagaaggttaaatcatcctattgaaaaagaccaaaatagaaagaactttttcaaattcaattctttattt
ttcactggggttagctgatctagttcttaatattattactttactcaattgacagattacacagcaaatctcttgattcggaattagggactcatgccccatctgatgaatcc
attttcttttacacttctgtatctcactctatcttgtttttttagtattatctaaaataaccgatgaattatgaattttccataacttaggtaagtgctttaccaacatatgtagtgtag
taaaaaaaaatggaatttaaccctttcatgcttactataactagttatttcggttttctattggctgctttaactctaaccctagctctatttattggcttgaacaagatacgtctt
atttgaaatgaattgaatgaataaggcagaaaagaaaaaaatctctcttttggattcctggtattctacgcactaattaccaattctttttttcttggtcattgagattcgtggat
aatttagactactatttagggataaatcgtacctcttttttttttatcccctcgaacaaattgaaatgattgaagtttttctatttggaatcgtcttaggcctaattcctattacttta
gcgggattattcgtgactgcgtatttgcaatacagacgtggggatcagttggatctttgattgagtaatatttctttttttgattgccctcctctctggtctggaggaggtcaa
attggaattgcaatttgttttgttaagttattttgccctgcttcgacataagatagatggaatcacgctctgtaggatttgaacctacgacatcgggttttggagacccgcg
ttctaccgaactgaactaagagcgctttcaaaaatcaaatccttttctactcctaatgtgtctcacgtacgtatagtatccacaaattaaagttatacccgctttaatcgacc
tcctcactactgcctataaagaagaaagaagtaataggtagggatgacaggatttgaacctgtgacattttgtacccaaaacaaacgcgctaccaagctgcgctaca
tccctttttccaaattgttgtacaatgtcattgtacacaattcctatcttgttttccacatcctaattttcttgtctttttctatctatagagaatcctcgtgtcatttcttctttttggtct
catataatcaaggaatggtatatatctaaatccaatctaatttcacctataaaagaaagattactattccttggtaatctataggaagggattgtctttttcgtttttagttcgg
aatttcgcctaaacaaaagaaatacaaatgatcttgggcaataatatctgatcatatacgtattccaataaggaaggaggattttcaatgcgggatataaaaacatatct
ctctgtagcacccgtgctaagtactctatggtttggggctttagcaggtttattgatagaaattaatcgtttatttccagatgctttgtcattcccctttttttcattctagttgtt
gctatgtgaggaatagagttcttcgtgacatgataaaaaaattcccacttttcaatcccttttttagtataggaaagaaaaagaaagaaaagatagattgggttggacctta
gagtcatgaaaaatttggtaaatctcatttttgaaaaaagaaattcaattaaaagcagtatccaagctaagtcaggcctcagaaatcagagcatagaaagaggctggg
ttggctactaaaatgaaaggattttgaagcaaaatccttgctaatttgacaaggattgtattccttaattatttctctattttttattacttaattgaaaaatttccaaaaatttttat
tctaatggattttcttcttcctcttcggtttcaaaatagaggaataaaagaataagtagaagaattaagttaagtcaatccaaaaaggaaaggaggttcatggccaagg
ggaaagatgttagaatcagagttattttgcaatgtgtgagttgtgttcgaaaaggggccaatgaggagtcggcagggatttctagatatagtactcaaaagaatcgcc

acaatacacccggacaattagaattaagaaaattttgtcgttattgtcgtaagcatacgattcatgccgaaataaagaaataggagcatcttgtgttcgatctttccaaag
atcaaaaagaataagaacttcctatttaatattcctatttaatatatagagcatagatagaatacaaaatacaaatcaacttgtctgatttccattagatattatttcatatgtat
cgagggtattcatctaatatatggaccaaagagagactatttcttctggatccaaaattaataaaataaacaaatcaattttttttcaattttaaaataaggaataaatcatgt
atacatctaaacaaccctttcataaatccaaacaaacttttcataaatccaagcaaacttttcgtaaatccaagcaaacttttcgtaaattcaaacaaccttttcgcaaacc
caaacaaccttttcgtaggcgtcctcggattggcccgggggatcgaattgattatagaaacatgagtttaattaatcgatttattagtgaacaaggaaaaatattatcga
gacgaataaatagattaaccttgaaacaacaacgattaattactcttgctataaaacaggctcgtattttatctttcttaccatttcgtaactatgagaatgagaaacaattt
caagcccagtcaatttcaataattacaggtcctagacccagaaaaaatagacatattcctccattaacacaaaagttcaattccaatcgaaacttaagaaactccaacc
agactttaagaaacaacaatcggaacttaagttccgattgttgatgttttattcgaaagggccagactatatataaagaaagtaatccaatttagattcttgggtttgttata
agaaagaacaatggggaagaaaaaatagttttttatttattgcaacatgctcgttgattcctaccacttaatcttaatttattgtatcttcccggagttacctctccgggaat
tctttttaattattcctgtatattactttttatccctttaattgataatctttattttattggaaatcgtgtaaagattatttggatttaatacagctacttgtgcaaggattttacgat
taagaatcaattctttcttgtaaagattgtgtattaatttactataattatcgaatactttatgtatccgcgttgctgcgtttatccgactgatccacaaacgccgaaaatccct
cttttgcctgactctatctcgatgagaggaaacaaacgctcttcttacctgttgagtaatcattcgattaagtcttaaatgagcccctctaaagtttgaggcaaatgaacg
catttttgcccgtcgtctccgagctatatatcctcgcggaactctggtcattgaatcaaattaaccttaatgaataactaatgatttctcttcttttagccatcctttttcccatt
aataacaaaacgaattattcccatatataaaatattagttccaatggcttttgctactataaccttcccaaccacaattttctcttctactcccttcagttatttcgcacgatact
aaaaaatagtgggttccatcgtttctatggttccccttttaaacggcgaggccctctctatacaccggagccctttctttcatttcatcaaagggtattgtgaacttgtatagt
tcacattctttggctctacctatccattatagagtaaatagctcttttcacaataagagttatccatacagtgacggcatttaattatgaaagttggctaagtagctgaccct
cttagtccgttttttaagataaaggagcataagcctttatctttttattactatttcctccgcttaatgaatagccgtttgctaccaatggggaattgcttcttatttccaatct
agatgattggatttgcaccaaaggaaaccagaaattccatataccgtagaaatctaggatagagaagctctatcctattcattggtaccgatcatggatacttcaaaaa
ttgtcttatttgtttgaacccatgatccgaacgagtcgcacatacaccctagcacatgttcctcgacgctgaggacatcccttaagcgcggccgattttctagcatttcgt
attggctgtcttgcgtttctaataagttgtttaaccgttggcatgtcgtatgtatatagaaaaaatggattggtttagatcgatcttaacctgaatgattgatcattatgaagt
atttccattaaattgcataaaacccgaatttagggttgaaataaatttacaagaaatctggccactaccaatccttaaacatttctggaaaccacactggatcagtatcgc
agtgctcgtcaagcatttcatcccctacaatatcgacaagtccataagctttggcttcgtctgctgacataaaaacatccctttccatgtcttcggatacaacccaaaaa
ggcttgcctgttcttagtgcataaacccttgtgatcatttcgcgaactttgtgtaactcttccacttctagtaaaaattctggtgttcttgcccgataataagcactagcagg
ttggtgaagcataatcctcgcgtgggggaatgctatacgcttggtgggttctcctccaagcagaatgaaggacgccatggacgcggctattccgaggcatattgtat
atatatctggtgtcaccgtttgcatcgtatcaaaaatcgccattcctgagattaaccacccgccgggggagtttataaacaaaaaaatatcgctaattccatcttctatac
tgagatataccatgagacctgtaatatgattcgtgacctcgcaacgaatctcttgacctaaaaaaagtgtcctttctcgatacataacattgtataagtcaacccaagtc
gcttcttcatctccgggaatccggtaaggtacttttggaacaccaatgggcatattagattaatattattaaatttaagtaagaaaactacactttaatatggaaacgtaag
aatggaagagaaagaaagaatccgcagtttattgttttcactttttttcttattctatatgaatactatagattctattaatacgtagattgaaataatacatagattgaaagat
tatacatataagtaggataagacagattgaataaagaaaaaagaatgggtgattcgaatactaaacaaaaagagatagggatctattcttcgtttttccaaataagcc
aagctgcccattgcatattggcacttatcgagtatagaatagatctgcttctctttcttcttacgaacagaattggcttcttatttttaatggaatgaaataaatattcacgctt
tctgacacagaatcccctagaagggttaggtacataggatatggatagtctttgccaatgcgataaaataaagtgacatcgtgtctatttttctttgctaaaggggtattt
ccatgggtttgccttggtatcgtgttcatactgtcgtattgaatgatccgggtcgattgctttcggtgcatataatgcacacagctctagtttctggttgggctggctcgat
ggctttatacgaattagcggtttttgatccctctgatcctgttctggatccaatgtggagacaaggtatgttcgtcattcctttcatgactcgtttaggaataaccaattcgt
ggggtggttggagtatttcaggaggaactgtaacgaatccgggtatttggagttatgaaggcgtggcaggggcgcatattgtgttttctggcttgtgtttcttggcagc
tatctggcattgggtatattgggacctcgaaatattctgtgatgagcggacgggaaaaccttctttggatttgcccaagatctttggaattcatttatttcttgcaggggtg
gcttgctttggctttggcgcatttcatgtaacgggtttgtatggtcctgggatatgggtgtctgatccttatggactaactggaaaagtacaagctgtaaatccggcgtg
gggtgcagaaggttttgatccttttgttccgggggggaatagcttctcatcatattgctgcgggtacattgggcatattagcgggcctattccatcttagtgtccgtccacc
tcaacgtctatacaaaggattacgtatgggcaatattgaaactgtactttccagtagtatcgctgctgttttttttgcagctttcgtagttgccggaactatgtggtatgggt
cagcaactaccccaattgaattgtttgggcctactcgttatcagtgggatcagggatactttcagcaagaaatatatcgaagagttagtgatgggttagccgaaaatct
cagtttatcagaagcttggtctaaaattcccgaaaaattagccttttatgattatattggtaataatccggcaaagggggggattattcagagcaggctcaatggacaatg
gggatggaatagctgttggatggttaggacatcctatctttagagataaagaagggcgcgaactttttgtacgccgtatgcctactttttttgaaacatttccggttgtttt
ggtagatgaagagggaattgtgagagcggacgttccttttagaagagcagaatccaaatatagtgttgaacaagtaggcgtaacggtggagttctatggtggcgaa
cttaatggagtaagttattctgatcctgctactgtaaaaaaatatgcgaggcgttcccaattaggggaaatttttgaattagatcgggctactttgaaatcagatggtgttt
ttcgcagcagtccaaggggttggttcacttttggtcatgctacctttgctttgctcttctttttcggacacatttggcatggggctagaaccttgttccgagatgtttttgctg
gtattgatccagacttggatgctcaagtggaatttggaacattccaaaaagtcggagatccaactacgaggagacagccagtctgaggccgcattgctttggtatctt
tcacctctctttttttgatttgacatgggaaacatctcccatcccttctttgactctttttccttttttatacgggaaatgatcccaaatgacaaatgaataggtgtggaagttat
aattgtaaataaaccacgatcgaatctatggaagcattggtttatacgttccttttagtttcgactttagggataattttttttcgctatcttcttccgagaaccacctaaggttc
cgactaaaaaagtgaaataatttaattgaagtaagaagtctccccatctgggagacttcttacttcaattagtccccgtgttcttcgaatggatctcttaattgttgagagg
gttgcccaaacgcggtatataaggcatacccagtaaagcttacaagtaaaccagatatggagatggcgactaaagttgctgtttccatttttatagaatttcaagattac
aatggatctacgaaaagatcgtgtatttacaactacaacggaatagtatacaaagtcaacacaaatcattaaatggaatttatggctacacaaaccgttgaagatagtt
ctagacctggaccaagacaaactcgcgtaggtaatttattgaaacccttgaattcggaatatgggaaagtagctccgggttgggggactactccttttatgggggtcg
caatggctttattcgcggtattcctatctattattttagaaatttataattcttccgttttactggacggaattttaatgaattaggtttctactaacgaaaactacgaagtcata
gtttttccatccaaaagagcctttctactttaagctctacatttctagacattctggtagttcgaccgtggaatttttttggtttcggtatctctggaatatgagtgtgtgacttgt
tagaatttgctcctattgataatacatagaaagcacctgttatctctatcaagatgattctaattcgtcggatattatttattctagtatctggaacacgaaatagatagagtg
gatcaagaaaaaaaaatgaaactatgattcatattaactattcagacctcgcaaccagactgaaaaaaattcaagtagttcttaataaaaataaaaaaagaaaatttctt
ccttccaattttgtttgcccaaaaaacaacttttttctctcgattttgtcgagtcattacaccgattcaataaatgatcatcaagcggttcttattcgaagaacccttgcctttt

gtttagcttgagactcaatcatcgtggctctagtatgaatctaaggttttaattgaactgattcataggatcgcaacaagataatttctaccagaaaactactccaatttttg
ctttatttatttatctagtaaaacaagagtaaatctgcattacgcacaaaaaaaagaaatccaaaatagggaagagaaaaatcaagaggcctctaatgatcaacatttg
ggaaagaaagatagacgagccaacttgagattttttggcattatcatcacaaagaagaaattctggatttttcttatttcatatcttcaaggcaaatcgacccaacccagt
ggctgatgaagttttgaacctttttctaatatccgttgaaaatttgtgtgtttctgtttgagccgtacgagatgaaattctcatatacggttctcggagggggggttcgggtt
agttacctatctcaataaagtatatgattggtttgaggaacgtcttgagattcaggcaattgcagatgatataactagtaaatatgttcctcctcatgtcaacatattttattg
tttaggggggaattacacttacttgttttctagtacaagttgctaccggttttgctatgactttttactatcgcccaaccgttacagaggctttttcctcggttcaatacataatg
accgaggccaactttggttggttaatccgatcagttcatcgatggtcagcaagtatgatggttctaatgatgatcctgcacgtatttcgtgtgtatctcacaggtgggttt
aaaaaaccccgcgaattaacttgggtcactggtgtggttttagctgtattaactgcatcgtttggtgtaactggttattctttaccttgggatcaaattggttattgggcagt
caaaattgtgacaggtgtgcctgacgcgattccggtaataggatcacctttagtggagttattacgtggaagtgctagtgtgggtcaatccactttgactcgtttttatag
tttacatacctttgtacttcctttgcttactgccgtatttatgttaatgcactttctaatgatacgtaagcaaggtatttcgggccctttataaggaaggcatctcatagagagt
tctaattctcatatatcatatcgggtaggttgtggtatttcattgctacaaacatgggttattctaaaataagacatgtcatttagatacttctcttcaactccgaactattgtg
atacaaataatacaaatagttgaagttaattttacgaaagaaaagaaggcggattatgggagtgtgcgacttgaattattgatttggccatgcagatagagagttggat
ctgccacattagaattcacgaccaaaggtgtctccatatccaatcaacacgtaagtcccctgtctagtaaggataggctggttcacttgaggagaatattttctatgatc
atacctcaaccatgtcatccatgaagaggctccgtaagatcccatagagtagaaatggaataagtcatgtgacatgatccaattctctatttattacacttactttttattat
agtatggaaatgcattcattttctttgcatcgattgcgatctgcaatactatcggagtaaaagaagggatctaaggaagaacgtaggctaaactttttgatttttttattagta
acaagtaaatactttgtttggacgtaagaaacttgcaatattgggggggataaataccaactaatcaagagacgcgagacaatccacaaagcaattgatcatgatca
aatttgtaagcccacttggatattgagcatttacccataagagtaggattcttttcaatgaatagttgtaggtgcaacttcggaaaatagaatctgataaagcttttcttact
tagagccattgagccattatataccttattctattatggatcttctacggttttatttctttcattcttgctcgagccggatgatgaaaaattctcatgtccggttcctttgggg
gctggatcttaaagaattcacctatcccaataacaaagaaacctgacttaaacgatcctgtattaagagcaaaattagctaaagggatgggacataattattacgggg
aacccgcgtggcccaacgatcttttatatatttttccagtagtaattctaggtactattgcatgtaatgtaggtttagcggttctcgagccgtcaatgattggtgaaccggc
ggatccgtttgcaactcctctggaaatattacccgagtggtacttctttcccgtgtttcaaatactccgtacagtacctaataagttattgggcgttctcttaatggtttctgt
gccgacgggcttattgacagtaccttttctagagaatgtcaataaattccaaaatccatttcgtcgcccagtagctacaaccgttttttttaatcggtactgcagtagctctt
tggttaggtattggagcaacattacccattgaaaaatccttaactttaggtctttttttagggatttttcagtttgattcattcaatcgtgaagtaccgtgcataggtatctagg
aaatagttacttccaagtgaatcttccctagatacctataatctattttattatgatccatttcgcgaaaatatagattgtcccaaagatgcaaaattgttttcttttttctttttatt
ctaactcgaaaaagaagaagaggaaaaaattgcaatggatttaaaactagaacttattcttaggtaaatccattgggagatgcttctctagagtgtcccatatctgttttc
catcttgcatacgaaaactgtcaattctcatcagatcttcttccgtcttactcaaaaggtccaatagtgtatggatattggccctttttgagacaattatacgttctagaaggc
agttctaattgatcaataaaaaatacaattcaatggaattcttttttttgtttttctttagattagttaatctttttttgaaagcttaaaagggggggaagtaaacctgtttttattttcttg
gaaacgagtaccctcttcctccgtgtgaagaaaaggaagaaataaatcaatcaaattacgagaagcctcataaagcgcttccttaggggttaaacttccattggtcca
tatttctagaaaaagtatctcatattttgcatttccattcccacaagcaaaaatactataattcacatttcgaacaggcatggatacagcatctataggataacttccatctt
gagtgttctttctgacttccgtgtgatatccgcgatctctcttgatccgtaactcaatacggaaatccgtgggttctgtcaagttagctataggttgtgccgtatcaacgat
ttctacggaaggcggtaagataatatcttgggcagttatgtatctaggacctttgacgcaaattgatgcggttctaactccatagagattacttctcaatacaatttctttca
aatttagtaaaatttcttgtaccgattcttcaatacctgctattgtagaatattcgtgcggcacgctcccaaattttgcatgtgtgatacatgttccttctgtttctccaagtaa
agctcttcgcaaggcaataccaacggtatccgcttgaccttttctaagcggggacagaatgaaacgaccataataaagacgcttactatctactcttgattcaacacac
ttccactgtagtgtttgagtggatcctgctacctcctctcgaaccatatagactagtattattatttgatcattgaatcgtttatttctcttgaaaggggtttaattcttttacaga
cgtcttttttttaggaggtcgacacccattatgcggcataggtgttacatcgcgtatacaacttaatcgtacaccacttttagcaatggctcgtaatgcggcatctcttcca
ctaccagcaccctttaccataacttctgctcgttgcaaacccactgtacgaatagcatctacagctgttctttgaccagcatagggtgatgcttttcttgagcttttgaatc
cacaagtacccgcggaggaccagaaaaccacccgaccttgcggatctgtaacagttataatagtattgttgaaactagcttgaacatgaataactccttttgttattcta
cgtgcactcttccgtaaactaaaacgcgcattcctacgcaaaccaatacgtactttcctacgtgaaccaatttttggtatagcttttgtcatattttattatctcataaatatg
agttagaaataacaaaaagaaaaaaagatacaaagatatccgtttcagggtaaaatatatcctttacttgaatttttttatttggaatttggacattttcgcgggtactttttttt
tttactttttagaaagtcaagttctttttttcgaaagattacccctgtctttgtttatgcttcggattggaacaaatgactctaattcgtccacgcctacgaatcagtcgacatttt
gtacaaattttacgaacagaagctcttattttcatatttccgtattcctttcttaaactatgaatttactcttttggaaaaaataagtctctttgtttgaattttagaactttgaatttt
ttaccctagaaaaaaaaagaaaaacctaaccctttgaatctttggtatccttcaaatcttcggtatccttcgagtcttcggtacgcttcgaatccttatggggaagtctata
aattatacgccccttgcttgaatcataacgacttacttcaattttgaccctatccccccatcagtattcgtatagaactagaccggatctttcctgaaatatagcccaggatg
atggtgtcattctctaggcgaacgcggaacattccgttgggtagggcttccataactaaaccctcgaaagtaatttttgcttctctcgggtttttttttctctcctatttttctt
ttctgtcatattttttttctcctatttttcttttttttatttgaaaataaaaaaacgttgtatttttgtttgaaaaataggaagttcaagatagaatttgggtactataactataggtgg
ggcgattaccgctataggcggggcgattaccatatataacataagacttctcccccaattctgtttagtcgagcttctcgatctgtcattatacctcgagaagtagaaag
aatagcaattcccattccgcccaaaaccttaggaattccttgatagttggcataaattcgtaagccaggtcggctgatacgctttaaaaaggttctagttctatatattcc
ctttctagtctttctcttttgatgtcgcaaagttgaaaccaagaaatatctgttactttcctgatgtttccgaacactttcaataaaaccctctcgtagaagtattttaacaatgt
tttcggtaatatttgtagatactacccgaacagttcctttttttattcatgtccgcgtttcttatagaggttagtaaatcagcaatagtgtccttgcccataagactctaattcta
ggttcctcctaatttttctataatcaacatgttctctttttttctttttcgttttggattctaaagcatatacgtgaaacacaatctactaatttttttatttgatctatatcttgtctactag
tatttataatacttcaggagctaatgaaactattttagtaaaattcaattctctcaattcctcggcgatcgcgccaaaaacgcgagttccttttggatttcctttttgatcaatg
ataaccgctgcattgtcgtcatagcgtattattataccgtcttcgcatttgaactctttacatgtacgtacaattacagctcgaattacttcggatctttctagaggcatttgg
ggcactgcgtctttgattacagcaacaataacatcaccaatacgagcatatcgctgattactagcagctcctatgactcgaatacacatcaattttcgagctccactgtt
atctgctacatttaaaagggtctgaggttgaatcatattattttgatttcaatttgttatttcaatgcaaaaggatgaaagaaatattgtctttccagaaagaagaacctggtt
ttttattttcaatactactttttttggggggttatatttctaaccgaagaaattgacttcgtatgggcattttgctggcagctatggaaatagctgctctagctacagtttcgga
tactccgcccatttcataaagtattcgacctggtttaacaacggctacccaatattcgggggatccctttcccgaacccatacgtgtttcggtcggtcttattgtaaccgg

tttgtcgggaaatatacgtacccagatttttccaccacgacgtgcatatcgtgtcattgctcttcgtcctgcttctatctgcctcgccgtaatccaagtgggttcaagtgct
tgaagagcatatctaccaaaacaaatacaattgcctcggtaggattttcccttcattcttcctctatgttgtttacgaaatctggttcttttggggttatagtcgatggttctct
cttagttccatctctactgcaaaactggacatgagagtttcttctcatccagctcctcgcgaatcaaatgagaaagtgtgcaaatttctctaattccacaatattctaaaat
attacggatagatacacattaataggaaaggttagggtttttaatattgaatttattaaaatactaaaatatatagtcaagaaagaggatctagaatatatctagatgtgtat
gtttatttatctatattctatatttaatgtaattcttttttttttttaatctcctttttttatttactcttattgaatcgcggtaaagtattctaattcaataaaaaatttcgcgggcgaata
tttactctttcctgtcttatttgttaattcataaccttaacaaataagacaattttttggtttgttccgccatcccacccaatgaagtattgggattcttttcaagaaaatcctat
ccagtcataggttctgtcgttcccactgcttctcctttaatggttaggtttgaatctcgcaatggagcttccaaaaaatttcttccgagtcaatttctcagttttattaactcg
ggacgctctttgttattccttgaaatttgtatttcttgtttcaatcaaattacgatttcgaattctctgttatttttattatattgatgctttatcacattgcttttatgatgtaattcat
agaccatacatattggaatcctatatctttcttatttctcttccttctttctatcatccttcctttatctacatcccctttagtttgcttcacaacttagaatcctattttttttaaag
aaaaaattgcagttgctacacgtatatgatagatttactcatttatgatagatgtatttattcatatagtgactgtttcttagttaggatctcgacaatacgaagcaataggtt
ggttattagttcattttctataattacatagtagggttcattctattttttttaatccccttttgaaccctaaaaaaaaaactaacgagtcacacactaagcatagcaattttatt
aaaagatttctcaattttcattaaatcttatagaaagaggtagaatttttctttttttcagggatttagggaaaataaggctcttgtcatttttattctattactgaacagaatg
ggaagacagggttggttattcttcgtctacgaatatccaaattttaaccccctaataccccatagatagttcgaattggatagcagcaataatcaattttagcgcgaattgtt
tggaggggaagtctaccttttttgatgcattcggcgcgcgcaatttcttttcccgcgagacggcctgcaatttttacttttactccctttatatctgtttttttagttaattcaat
ggctttttcattgcctttcggaatgaaactctatttttaattggaaagctatatattctgcaagaatgttaggttgtctataaggttctttcactttttcgatcccaatattaagt
ctctggtttacagaattaacttcttttgtagatctttctctaattcttcgattgctcctttttttcttaataaattggggaatccaatatggattatgacgtggattgtatcgatttc
ttttgaatttctatatgtgtaattacttcggaacttgagcctgagtccattttttattatgtgtaattacttcgaaacttgagtctgcttctatttttctattcgagcccttttttccta
ttcttttgtatatagttcttgatacaattccgtatttttttatcttcctgtagaccttcagaataatttttttggttgtgcgaaccaaaaggaatggtgattttgggttgtaccaagt
ctgaaaccaagtggatttattttttgtcccatatttttctattctatttttttttaccgggaatcaaatcttagatggatctaaagattatttcgatttctttactatatttagtacaatt
gttatatgacacatggtttttttatgggagaactacgtcctcgagcccgaggtctgaatttattcataatagtactcctactgacttcggctttagtgatgaataaatttgct
ttgtcgaaatccctataatgagtagcatttgctgctgccgaataaaccaactttaagatgggataagatgctcgataaggcatgaggttcaatatcataacagtttcttcg
tagtaacgccaacgaatctcatccagaactctttgtactttgaaaacggacatgtggatgcgtttttgtgctttgaaaacccgttcgaacttaagtagacgatcggttggt
acttttcttgcgagtttccttagcccactcttcttcttctttatcctaggggtatactttaccagtttgaaacttgtcataaataaggttattccccgcttacctatttcttttttttttt
atttgaatctttctattctgaattcagttaacgacgagatttagtatcctttcttgcactttcataactcgtgaaatgccgagttggtacgaattcccccaatttgcgacctac
cataggatttgttatgtaaataggtatatgttcctttccattatgaatcgcgattgtatggccaaccattgcgggtagaatgctagatgcccgggaccacgttactattgtt
tctttctcctccttcatattgacctttctatttttgccaataaatgatgagctacaaaaggattcgtttttttcgtgtcacagctgattactcctttttttccattttaaagagtgg
cattcgatgtccaatatctcgatcgaagtatggaggtcagaataaatagaataatgatgaatggaaaaaagagaaaatcctttagctggataaggggcggatgtagc
caagtggatcaaggcagtggattgtgaatccaccatgcgcgggttcaattcccgtcgttcgcccatcccattattgcaaattccaaaaatgcaattttccatattcctag
ttacgtatttacttacggcgacgaagaataaaactatcactatatttttccttttcctagttcttcttccaagcgcaggataaccccaaggggttgtgggtttttttctacca
atgggggctttcccttcaccgcccccatgggggtggtccacagggttcataactacccctcttactacggggcgtttacctagccaacacttagacccggctctacc
caaactttttggttcaccccaacattacccacttgtccgactgttgctaagcagttttgggataccaaacggacctccccagatggtaatcttaaagtggccgatttacc
ttcttttgcaatcagtttcgctacagcacctgctgctctagctaattgcccacccccttccacgtgtgatttctatgttatgtatggccgtgcctaagggcatatcggttgaa
gtagattcttcttttctctcaaaaaaccccttcccaaactgtacaagcttcttccaaagcatacggctttctagatgtatatgacgatctctagacagatggatcttatatga
atcgtatgatgaagtaccacatgagtggatatataggaaaggaatccaaatctgccgaatcgctcatgttatgatcttctacatcctaggtctccgcgttccgtcatctg
gcttatgttcttcatgtagcattcagatcgaatgactctatgaaattacgtcgatacttctacatattatgggtaacgtaggagacatccctatttcccccgggggtcttaa
ttaccactgcttagctttcaattcgcctctgaccatcaaattaaatgtgaataacccgtcctcctctctttgaaacaaggggcgcttccggttctgtgcgtgcttcaaaca
attttgtcttctccatattaccatatctctagagtcaataattttctatgaggaactactgaactcaatcacttgctgccgttactcaacagttttctgttgaggtctatcccgta
gaggtagtcaaattggatcagtgatcgatttctaggtttcgtcgtaaacctaattggttacttccaattacgtaaatcaatagttcaaaccgcactcaaaggtagggcatt
tcccattgatataggaacttttgtaccagaaacaatagtatctccaattatagcccctctgggatgtaaaatatatcccttctcaccatccccatagtgtatgagacaaat
gtacgcatttcgattagggtcgtattctatggttacgattctaccagatatgtctttttgattccgtcgaaaatcgattttacggtataggcgcttatgacctccccctctatg
ccttgcggtaatgattcctctggaattacgacctttaccacaacggtgccgtccatggatcaaattatttcgtggattggatttcacttgcctatctatggttcccttgcgtg
tgctcgggataggtgttttgtataaatgtttcgccgtattattaagtattctccttttagttttttctctatctagaagtggaatagaataacccggttgaagggtaatgatcat
acgtctgtaatgcattgtatgtcctagaatagggcccattcttctacccttttccgggtagtcgatggctattcacagctactaccttaacaccaaagaagagttcgaccc
aatgctttatttctgtcttagtgaatcccgattcgacattaaaagtatattgattctttcccaataaacgaagactttttttctgtaaatactgcgtatttgattccatcccataaatc
gactttccctcctatgctctgagttccagtatcgataagaattcgagttcttattgttcttatgttatggtatgaatataccataccaattcgttatgtatggatgatggatgag
attccatggatagagagccagttccaatagacttatggaacgttccggttcgcgtgcatccagcaggaattgaacccgcaaatttaccaattatgagttgggcgcttta
accattcagccatggatgcttaacagggatcatcgtacatcgtaaataaccaattttcatatagaaagacatatcatagaaaaatgaaatcgaaaatattcggagatgg
caaatattcggagatgactatgaaaacacctctctggatcctcgaattgaaagagagattgagagggatccagaatcctaattctcgctatttggaatggatccaattc
tattgagtctgactcatagtgatcatttctctttagcaaagaatgaccttggttatcaaaggattgaacaaccgggatccgtttacttatgatacctagttgacattgataac
aaggatctaatgaattatgagtttaatagatcctctttagcagaaagacgtatattccttgctcatcacttattcccaaacctcgtatggggctaatcgttttcatttaccatc
tcagggaaaatccttttcgttccgcttagccctatcgggtattttagtgataggttctataggaactggacgatcctatttggtcaaatacctaatgaaaaattcctattttc
ctttcattaaggtacgagggcttcttattccacaagaaaagaacgaaagcaccttttcattctttcatatactgggggtttttacttggaaaagacaatgttccatactaaa
ggattcgggtccataaccacgagttccagtgcactagatcttgtagcacttagcaacgaggccctatgaatagacatatagaatttttggtcgggaaattcgaatgaat
cattgagtgaaaaaggagcaaagaatgacaaaagacgagactctactagtcttcactcttgtggtttcctcggtttctgttttcttattcgggatcttgcttttcatggttct
catctctgcaactcgcgatttcgcgagagaaccaaatccaagttggtgaagatcatgatttgggctggcatagtagtgattacctttgcaattgcggttcgaatctatc
cgatctttatctttttgctcaaagaacgaataaaaccccttgtcgaagccctttatgataagcttccctggatctgggaagtttctctttcacggtattgggatcgtttgatc

gatttccttgatcgctacttatgggcgtgcgctcaaaggatacaaacagggattcgcaaacaaaaaggggaattcgtagtcactttttcctgtcgcgtaaaaaaaagg
ctttacgcgagagcaatagaggttgggatacatctatctcttctgagcaacctcttttggattcttaagaccacccttgcagtaggataccgtctgctttgggttctttatta
tattatctccttcgagggatttttaggatcgttcaggctatatttagtctattttggcttttactgtctccttttctcagggaagtggttaaggacctcagaagatagaggaga
gcgccaggcgcagatttccggaatacttctacggggaatgctcattgaatgcgcattctccgtattatgccttgaagaggactcgaacctccacgctctttagcacga
gattttgagtctcgcgtgtctaccatttcaccatcaaggcatcttgaaagtgaatcgtattccatgaatatgatatctatctaatgtgatatatggaatatatgacaaaggtg
gagtcttggagtatttcgatcgatcggtcatataggcctgagtcagacatcaaatagcttcgatttgcattatccgtaggacaccttatatgtatcaaaatcaaaaagatg
tacaatccaatttctcgattcaatagaagcccaaagaggtgcatatggtacccaaataaggataggatagatatgtcaaaagcaggtctgattacacctattcctaatc
ctaaatagaatgtaaggacgtggggatttctatgtaaacagagtatcctatttccataggctcgaatgacccccttctcataataagaatgtgcacggtctggtccggtat
ggaatgaacttataatctgatgatcgagtcgattccatgattataagttcattaccctagcgcccattcccattttgggcggaacagatctactaattcttttattccagtta
gtaagagggatcttgaactaagaaatagacctagcagctaaaagagggtatcctgagcaattgcaagaatggggttcattgatattcctggtatagtagatgctatca
cacatacagtcatactcaattcgatggaattgtttgatcttaaaggggatcttctataatttcgcacataaggggttatttcttggtttcgtccagtcattaataacttgactat
ttttagataatagtagatagaaagaacgctcgtaaggagtcctattgaaaccaagaaatataggcctgcttgccatccacaccagaatagatagagttttccgaagaa
acctgctagtggaggaaggcctcctagggataagagacatagggctaaagagagagccaaaaaaggatctttcgtgtataatcctgcataatctcgaatgttatcag
ttccggtacgtagaccaaataatacaatgcaagcaaaagttcctagattcatggagatatagaaaagcatataagttatcatgcttgcatatccatcatttgagtctcca
acaattattccaataattacatatccgatttgccctatggacgaatatgcaagcatacgtttcatgcttgtttgagtaatagcaaggagattccccaatatcatgctaagaa
tagctaggatttccagaagaagatgccattcgtttgatgagaaataaaaaggaatatcgagaattcgcgtggctgaagctgaagcagcaactttcgaagtaacagaa
agaaaagcaacgactggagtgggggagtcagagtcgaaaagaggattcctcgcttctttctctcatgcaaaaccgtgcatgagactttcatctcgcacggctcctaa
gtgataaaagaaagaagaacttgtcttctttctttttttgattaccttcctcgcgtatgtataagaccgaatccattctttttcgaaatcgatttcgaaaaagaactactaatcc
ttaacttttcgaggaatccttcatcagtggttgtgaatgactgactttttcaatcctttcgaccttggttccgtaggagcaagtcagaaaggttgagaaatagaaccatct
gatttgattcgttcccaatagccatgagatgatcatcttagggtgatccttttgtcaacggatgctcctattacactcgtagtctctgaaggatgagaacccactatgtag
catctacgtcgataattcaagcattgtatacgtcattagtccgattctttgtaggaactacccgtaataacgagcttgcaaaatggatctgtttatcataaagagattcgtt
gttcctgaccctgcttcaccttaattgttatttgaacaaaaagatcacaataaacttttggtaaaagttctgtcttggtcggagtggggatagcatttctcttctgcatatcta
tggagttttgcaaaacccaaacacctcagagatagatatagaggtaggaatttgtcgaacgaaccacactccttcgtagacgtcaggagtccattgatgaaaaggg
gctggggaaagcttgaacccaagtcctacagtgatggatataagcgcaattgaaattcctggggagttatacatttgtgtattgataagaccgttcacaatttcttgaag
ctcgatctccccccccagatgaaccatatagccaagagaaaccatgaaccagaatagaagagcttgccccacccatgagtaaatatttcatagtagcctcattagacc
gtagatctctcttggtatatccagacaataggtaggaacataaactgaaacattctggagctacaaagatagttattaaatcgttagcaccacataaaaacattccccct
agagtagctgttaatacgaataacagaaactctgttatagccatttctgtacattcaatgtactctacggatagaggaatacataaagttgaacataataaaataagaaa
ttgaaagatttcgttgaaattgttcgtttggaaatttcccgaaaagctaattataggttcttctctccatcggaacaatagggccgttatgcttattactaaacttgttgaaga
gatgaaatagaaccaaggtctatctttttgatcagaggttaaatcgatcatcagaagaagaattaggccaaaaattaggatacattctgggaaaatgaaacttccctgg
aagagaagcaaatgaaacgctttcataaaaattctcgtagaatcgagaatgaagttttcattctgtacatgccagatcatgaattagtaactgcatccaatctccgaaaa
gtcccgattgtttcgatttttggaatgggatatttacggaatccccatgaataggatcaaaccttattccatgctatttccataagattcctctttcttattcttaagcaagccc
ccgagagggcttagttgatcatgatttctgttttctctttcttttcctttttgtttgtttcgagaaagatatcgtccgattctccttctattgattcttttccgatcgagatgtatgg
atccatgtgtctacatacctagattctgttcatggattaacgaaaatgtgcaagagctctatttgcctctgccattctatgagtcgcttcctttttgcgtatggcacccccac
cccctttggcagcatctactaattcggaacttaatttgaaagccatatttcgacccggacgcttttgggatgcttctaataaccaacgaatggcaagtgctcttccttgttt
agatcctatttcaatcggaactttccgcgtcgatcctttttattacgtcttgtttttactcctatattgggagttactctacgtattgcttgacgtaaaaccaatagtggatttgt
ttctgtcttttgttgaatctttttcacggctcgatagagaatttgataagccaatgattttttttccgtctttcataatacggttaaccaccatgttaactaatcgattacgaaaaa
ttggatcggattttgcagttcttttttctgcagtacctcgacgtgacatgagcgtgaaagaggttcaagaatccgttttctttttataagggctaaaaacgaatcacttatttt
tttggctttttgaccccatattgtagggtggatctcgaaagataggaaagatctccctccaagccgtacatacaactttcatcgaatacggctttccacagaattctatag
ggatctatgagatcgagtatggaattcagtttactcacttaaaattgagtatccgtttccctccttttcccgctaggatcggaaatcctgtattttccatatccataccatca
agtccttaggtttccgaaatagtgtaatggaaaaagaagtgcttcgaatcattgctatttgactcggacctgttctgaaaaagtcgaggtatttcgaattgtttgttgacac
ggacaaagtaagggaaaacccctgaaagaatttccatattgaccttggacatataagagttccgaatcgaatctctttagaaagaagatcttttgtctcatggtagcct
gctccagtccccttacgaaactttcgttattgggttagccatacacttcacatgtttctagcgattcacatggcatcatcaaatgatacaagtcttggataagaatctacaa
cgcactagaacgcccttgttgacgattctttactgcgacagcatctagggctcctcgaataatgcgatatctcacaccgggtaaatccttaacccttcctcctcttacta
atactacagaatgttcttgtaaattatggccaataccaggtatataagcagtgatttcaaatccagaggttaatcgtactctggcaactttacgtaaggcagagttgggtt
ttttggggttgatagtggaaaagtcgacagataagtcacccttcctgtccctctacagaaccgtacatgagattttcacctcatacggctcctcgttcaattctttcgaag
ggatccttttcctcgttcgagagtctccgcccttcttccactccgtcccgaagactaactaagaccaattgagtcacgttttcatgttctaattgaacactttccatttatgat
taaaggagaagattgttcttttaccaaacatatgcggatcaaatcacgtcttataataagaagaaatctttctcggtatcaatccccttgcccctcattctttgagaatcag
aaggatccttttcgagtttccatttcttcatttggaatctgggctcttctatcttcgacttattttttttttgctttattctttatttatttcatttcgatttttccctcttcctctatccctat
cctctaggtacagcgtttgcatcaatagagaaccttttcctctgtatgaatctatattattccattccaatttcttcccgaaacttcccaagaaaaatcccgaattggatcca
aaattgacgggttaatgtgagcttatccatgcggttaggcactcttcaaataggaatccattttctaactggctttcgtgctttggtgagtcgtccgagatcctttcgatga
cctatgttgtgttgaagggatatctatatgatccgatcgattgcataagacccgcggtagcaatagaacggggaaagtatacagaaaagacagttcttttcaatttcga
ttatctatatattagttcgtttctatttctagatatctatttctatatattagtattagttagtagtactattctattagttagcgatcccggctctgtgagttctttcttccgtgatga
actgtcggcaccagtcctccatttttctctgtggaccgaggagaaagggggctcagcaggaagaggattgtaccatgagagaagcacagaggtcaaccctcttca
aatatggaacatggattctggcaatgcaacgtagttgggtcctcatatcgatccgaatgaatcagtctttctacagaggtcaatctttgcctattaggcaagaggatag
caagttcgaaattctgtctcggtaggacatggatttctattactatgaaattcataaatgaaaatgaagtagttaatgggagggctaccattatcctttttcttgtatgtgttc
ctaagagaaggaatttgtccatttcatgtttcgaggtctcaaaaaagggcgtggaaacagatagaaactcttgaatggaaattgaaaagaaatgtagccccagttcct

tcggaaatggtaagatctttggcgcaagaagaaggggcgacccatatcatcttgacttggttctgcttcccctcttttttaagaataccgagtcgggttcttctcctacc
agtatcgaatagaacatgctgaacaagatcttcttcatggaaacctgctcgatttagatcgggaaaatcgtacagattttatgaaaccatgtgctatggctcgaatccat
agtcaatcctattttcgataggaccggttgacaattgaatccaatttttcccattatttgactatccataatagtgtggaaagaaagcccggaggaagggtggccttgag
tttctcgcccctttgccttaggattcgttaattctctttctcgaggggacggggaagggatataactcagcggtagagtgtcaccttgacgtggtggaagtcatcagttc
gagcctgattatccctaaacccaatgtgagtttttctattttgacttactcccccgccacgatcgaacgggaatggataagaggcttgtgggattgacgtgatagggt
agggttggctatactgctggtggcgaactccaggctaataatctgaagcgcatggatacaagttatccttggaaggaaagacaattccgaatccgctttgtctacgaa
taaggaagctataagtaatgcaactatgaatctcatggagagttcgatcctggctcaggatgaacgctggcggcatgcttaacacatgcaagtcgaacgggaagtg
gtgtttccagtggcgaacgggtgagtaacgcgtaagaacctgcccttgggaggggaacaacaactggaaacggttgctaataccccgtaggctgaggagcaaaa
ggagaaatccgcccaaggaggggctcgcgtctgattagctagttggtgaggcaatagcttaccaaggcgatgatcagtagctggtccgagaggatgatcagcca
cactgggactgagacacggcccagactcctacgggaggcagcagtggggaattttccgcaatgggcgaaagcctgacggagcaatgccgcgtggaggtggaa
ggcccacgggtcgtcaacttcttttctcggagaagaaacaatgacggtatctgaggaataagcatcggctaactctgtgccagcagccgcggtaagacagaggat
gcaagcgttatccggaatgattgggcgtaaagcgtctgtaggtggcttttcaagtccgccgtcaaatcccagggctcaaccctggacaggcggtggaaactacca
agctggagtacggtaggggcagagggaatttccggtggagcggtgaaatgcattgagatcggaaagaacaccaacggcgaaagcactctgctgggccgacac
tgacactgagagacgaaagctaggggagcaaatgggattagagaccccagtagtcctagccgtaaacgatggatactaggtgctgtgcgactcgacccgtgcag
tgctgtagctaacgcgttaagtatcccgcctggggagtacgttcgcaagaatgaaactcaaaggaattgacgggggcccgcacaagcggtggagcatgtggttta
attcgatgcaaagcgaagaaccttaccagggcttgacatgccgcgaatcctcttgaaagagaggggtgccctcgggaacgcggacacaggtggtgcatggctgt
cgtcagctcgtgccgtaaggtgttgggttaagtctcgcaacgagcgcaaccctcgtgtttagttgccactatgagtttggaaccctgaacagaccgccggtgttaag
ccggaggaaggagaggatgaggccaagtcatcatgccccttatgccctgggcgacacacgtgctacaatgggcgggacaaagggtcgcgatctcgcgagggt
gagctaactccaaaaacccgtcctcagttcggattgcaggctgcaactcgcctgcatgaagcaggaatcgctagtaatcgccggtcagccatacggcggtgaatc
cgttcccgggccttgtacacaccgcccgtcacactataggagctggccatgtttgaagtcattacccttaaccgtaaggagggggatgcctaaggctaggcttgcg
actggagtgaagtcgtaacaaggtagccgtactggaaggtgcggctggatcacctccttttcagggagagctaatgcttatgcttattgggtattttggtttgacactg
cttcacgcccaaaaagaaggcagctacgtctgagctaaacttggatatggaagtcttctttcgtttagggtgaagtaagaccaagctcatgagcttattatcctaggtc
ggaacaaattagttgatagtgataggatccccttttttgacgtccccatgccccccccgtgtggtgtggcggcatggggatgtcaaaaggaaagggatggagttttt
ctcgcttttggcgtagcaggcctcccaaagggaggcccgcgcgacgggctattagctcagtggtagagcgcgcccctgataattgcgtcgttgtgcctgggctgt
gagggctctcagccacatggatagttcaatgtgctcatcagcgcctgacccgaagatgtggatcatccaaggcacattagcatggcgtactcctcctgtttgaatcg
gagtttgaaaccaaacaaacttctcctcaggaggatagatggggcgattcaggtgagatcccatgtagatctaactttctattcactcgtgggatccgggcggtccg
gggggggcactacggctcctctcttctcgagaatccatacatcccttatcagtgtatggagagctatctctcgagcacaggttgaggttcgtcctcaatgggaaaatg
gagcacctaacaacgcatcttcacagaccaagaactacgagatcaccctttcattctggggtgacggagggatcgtaccattcgagcctttttttcatgcttttcccgg
cggtctggagaaagcagcaatcaataggacttccctaatcctcccttcctgaaaggaagaacgtgaaattctttttcctttccgcagggaccaggaggttggatctag
ccataagaggaatgcttggtataaataagccacttcttggtcttcgactccctaagtcactacgagcgccctcgatcagtgcaatgggatgtggctatttatctatctctt
gactcgaaatgggagcagagcaggtttgaaaaaggatcttagagtgtctagggttgggccaggagggtctcttaacgccttcctttttctgcccatcggagttatttcc
caaggacttgccatggtaagggggagaaggggaagaagcacacttgaagagcgcagtacaacggagagttgtatgctgcgttcgggaaggatgaatcgctccc
gaaaaggagtctattgattctctcccaattggttggatcgtaggggcgatgatttacttcacgggcgaggtctctggttcaagtccaggatggcccagctgcgccag
ggaaaagaatagaagaagcatctgactctttcatgcatactccacttggctcggggggatatagctcagttggtagagctccgctcttgcaattgggtcgttgcgatta
cgggttggctgtctaattgtccaggcggtaatggtagtatcttgtacctgaaccggtggctcactttttctaagtaatggggaagaggactgaaacatgccactgaaa
gactctactgagacaaaaagatgggctgtcaaaaaggtagaggaggtaggatgggcagttggtcagatctagtatggatcgtacatggacgatagttggagtcgg
cggctctcctaggcttccctcatctgggatccctggggaagaggatcaagttggcccttgcgaatagcttgatgcactatctcccttcaaccctttgagcgaaatgtg
gcaaaaggaaggaaaatccatggaccgaccccattatctccaccccgtaggaactacgagatcaccccaaggacgccttcggcgtccaggggtcacggaccga
ccatagaccctgttcaataagtggaacacattagccgtccgctctccggttgggcagtaagggtcggagaagggcaatcactcgttcttaaaaccagcattcttaagt
ttaagatcaaagagtcgggcggaaaaaggggagagctccccgttcctggttctcctgtagctggattccccggaaccacaagaatccttagaatgggattccaact
cagcaccttttgttttgagattttgagaagagttgctctttggagagcacagtacgatgaaagttgtaagctgtgttcgggggggagttattgtctatcgttggcctctat
ggtagaacccgtcggggaggcctgagaggcggtggtttaccctgtggcggatgtcagcggttcgagtccgcttatctccagcccgtgaacttagcggatactatg
atagcaccgaattttgccaattcggcagttcgatctatgatttcgcattcatggacgttgataagatccttccatttagtagcaccttaggatggcatagccttaacgttaa
tggcgaggttcaaaagaggaaaggcttgcggtggatacctaggtacccagagacgaggaagggcgtagcaagcgacgaaatgcttcggggagttgaaaataa
gcatagatccggagattcccaaataggtcaaccttttgaactgcctgctgaatccatgagcaggcaagagacaacctggcgaactgaaacatcttagtagccagag
gaaaagaaagcaaaagcgattcccgtagtagcggcgagcgaaatgggagcagcctaaaccgtgaaaacggggttgtgggagagcaatacaagcgttgtgctg
ctaggcgaagcggttgagtgccgcaccctagatggctaaagtccagtagccgaaagcatcactagcttacgctctgacccgagtagcatggggcacgtggaatc
ccgtgtgaatcagcaaggaccaccttgcaaggctaaatactcctgggtgaccgatagcgaagtagtaccgtgagggaaaggtgaaaagaaccccagtgggta
gtgaaatagaacgtgaaaccgtgctgagctcccaagcagtgggaggggaaagtgatctctgaccgcgtgcctgttgaagaatgagccggcgactcataggcagt
ggcttggttaagggaacggaacccaccggagccgtagcgaaagcgagtcttcatagggcgattgtcactgcttatggacccgaacctgggtgatctatccatgac
caggatgaagcttggatgaaactaagcagaggtccgaaccgactgatgttgaagaatcagcggatgagttgtggttaggggtgaaatgccactcgaacccagag
ctagctggttctccccgaaatgcgttgaggcgcagcagttgactggacatctaggggtaaagcactgtttcggtgcgggctgcgcgagcggtaccaaatcgaggc
aaactctgaatactagatatgacccaaaaataacaggggtcaaggtcggccagtgagacgatgggggataagcttcatcgtcgagagggaaacagcccggatca
ccagctaaggcccctaaatgaccgctcagtgataaaggaggtgggggtgcaaagacagccaggaggtttgcctagaagcagccaccctttaaagagtgcgtaat
agctcactgatcgagcgcccttgcgctgaagatgaacggggctaagcgatctgccgaagctgtgggatgtcaaaatgcatcggtaggggagcgttccgccttaga
gggaagcaaccgcgaaagcgggggtcgacgaagcggaagcgagaatgtcggcttgagtaacgaaaacattggtgagaatccaatgccccgaaaacccaagg

tttcctccgcaaggttcgtccacggagggtgagtcagggcctaagatcaggccgaaaggcgtagtcgatggacaacaggtcaatattcctgtactacccttgttgg
tacggagggacggaggaggctaggttagccgaaagatggttataggtttaaggacacaaggtgaccctgctttttcagggtaagaaggggtagagaaaatgcctc
gagccgaggtccgagtaccaagcgctgcagcgctgaagtatgtaacccatgccatactcccaggaaaagctcgaacgaccttcaacaaaggggtacctgtaccc
gaaaccgacacaggtgggtaggtagagaatacctaggggcgcgagacaactctctctaaggaactcggcaaaatagccccgtaacttcgggagaaggggtgcc
ccctcgcaaaagggggtcgcagtgaccaggcccgggcgactgtttaccaaaaacacaggtctccgcaaagtcgtaagaccatgtatgggggctgacgcctgcc
cagtgccggaaggtcaaggaagttggtgaactgatgacagggaagccggcgaccgaagccccggtgaacggcggccgtaactataacggtcctaaggtagcg
aaattccttgtcgggtaagttccgacccgcacgaaaggcgtaacgatctgggcactgtctcggagagagactcggtgaaatagacatgtctgtgaagatgcggact
acctgcacctggacagaaagaccctatgaagctttactgttccctgggattggctttgggcctttcctgcgcagcttaggtggaaggcgaagaaggccccccttccgg
gggggcccgagccatcagtgagataccactctggaagagctcggattctaaccttgtgtcagacccgcgggccaagggacagtctcaggtagacagtttctatgg
ggcgtaggcctcccaaaaggtaacggaggcgtgcaaaggtttcctcgggccagacggacattggtcctcgagtgcaaaggcagaagggagcttgactgcaaga
ctcacccgtcgagcagagacgaaagtcggccttagtgatccgacggtgccgagtggaagggccgtcgctcaacggataaaagttactctagggataacaggctg
atcttccccaagagtccacatcgacgggaaggtttggcacctcgatgtcggctcttcgccacctggagctgtaggtggttccaagggttgggctgttcgcccattaat
gcggtacgtgagctgggttcagaacgtcgtgagacagttcggtccatatccggtgtgggcgttagagcattgagaggacctttccctagtacgagaggaccggga
aggacgcacctctggtgtaccagttatcgtgcctacggtaaacgctgggtagccaagtgcggagaggataactgctgaaagcatataagtagtaagcccacccca
agatgagtgctctctcctccgacttccctagagcctccggtagcacagccgagacagcgacgggttctccacccatacggggatggagcgacagaagcatggaa
ataggataaggtagcggcgagacgagccgtttaaataggtgtcaagtggaagtgcagtgatgtatgcagctgaggcatcctaacgaacgaacgatttgaaccttgt
tcctacacgacctgatcaaatcgatcaggcacttgccatctatcttcattgttcaactctttgatgaaaagatgaaaaaaccaaaaaaaaagctctgcccttccatctcttg
gatagatagagagggagggcagaggcctttggtgtcccttccagtcaagaattggggcttcacaattactagccaatatttctctcatgcctttcctcgttcatggttcg
atattctggtgtcctaggcgtagaggaaccacaccaatccatcccgaatttggtggttaaactctactgcggtgacgatactgtaggggaggtcctgcggcaaaata
gctcgatgccagaatgataaaaagcttaacacctcttatttgactttttcactattttgaaatacgaaaaagatccaaatccaaaatgcaaaggtcgtcttattcaaaacct
caatcatcacatcccctctctcccacttcacacctcggaacgcactgttcttatagagagaaaggggctttcccatcttcttaacccgaaatgaaatggctgaggaga
gggaggttccttttggggggtaccccgggaagagatccagtggagacggggtgggcctgtagctcagaggattagagcacgtggctacgaaccacggtgtcg
ggggttcgaatccctcctcgcccacagccttccaaaggggaagggcctttactttcccccctgagggtaggaaaaccatgatcgggatagcggacgtaaagctattg
aacttgggtatgctctttccttttgtcgaagtggaatcgtagaacagaatgtgatacgatgagataaaatgcaatagaaacaaggatagcgaacgggttacctactcct
aagggtcaaagcaagccctttaattcaattctttattcttacattaaagaatgaatcaaatctccccaagtaggattcgaacctacgaccagtcagttaacagccgaccg
ctctaccactgagctactgaggaacaagggggattcgacctcctagagttcaactcccgctctcaacccatgaacaatatgagtccgaagcttctttcgtaactcccg
gaatttcttcgtagtggctccgttccatgcctcatttcataggtaagcccagagtggctctatttcattctatttcacttcctagcacttcctatcatttaatatccatcccttg
gtcttattgacataagagatgtcatttatagtctatctctttctatatatggaaagtcaagaaattctcatcgaaacatcgagaaattgtgcatatagaaaactctaaagaa
agaaaaaaaggagacccatgccatgattttcaaatcttttctacttagtagtctaagtttctcgatgaggataattaattcggtcgttgcggtcggactctattatgggtttc
tgaccacattctccatgggtccctcttagatcttctttctccaatcttggattagggaagaaggagatattcgcgactcctggtggtttcattatggggcagctcatgatct
tcatatcgatctattatccacctctgcatctattctttcttagctaaacgggtggaagatccatccaatttggttatatcatggactcaaaaaacggatctgaatgtgactga
aatgcacgatcttcacaggtatcacttttcacgatacctaaaaggtggaatagcgattttcgaaccatttcctataagagaaaggtttccattactttgagaaatggattct
atatcaaactatagctattgcattaaagaagaaaagaaactaatagaagtcgaagacgcggaatggtagtgaatagagagaaagattcttctggttttcttgttcctga
aaatattctatctatctcctagacgccgtagagaattgagaattttcatgtctttcaattctcgtactcgtaattggaaagttacggaaggagatccatcattttgcaatgaa
aactacataaaaaactctggacaatttcgaaatcaggccaagcgtcttaatacatatgcaaaaaaaattcattattggcccaccattgattagaagatttaacttgtatgaa
tcgctattggtttgatacgaataatggcagttgtttcagtatgttaaggatacagatgtatccacaattcatttagagttacttaatagcctatttcttataccatatctctatcc
cgtgaaattctcgagccgaaagatggatgcatatgctatgtttcattttgctaaatgatatcaattaaacggtgtatcaattccataaattggatatagcaataaataaatc
agcaaaattcttttattttagatagaagaaaagtttcttctatctaaaataaaagaatgtacccttctatccaaatccaatttgcatcgataaaataaatccaaattccagtag
tagatgaataattgcaaatttttgtgtgtacgagattagaataacttcaaaataactgacataatttttttattttcctgatcagaaaaatacatgaaaaagaaaggaggtag
aaaaatttttggatttatggttaaagaagaaaaagaagaaaactggggttctgttgaatttcaagtattcagtttcaccaataagatacggagacttgcttcacatttgga
attacacaaaaaagatttttcatcggaaagaggtctccgaagacttttgggaaaacgtcaacgtttgctggcttatttggcaaagaaaaatagagtacgttataagaaa
ttaatcagtcagttggatattagggagcggtaatttcatcgttcgaatttttttcttattttattagtagtcttatagtagtcttagattttgcattttgatgagcctcgttttgagg
aattcatggaataatccattttcatggaataatgaattaaggaagaaaggatatgagtctaccgcttacaagaaaagatctcatgatagtcaatatgggccctcagcac
ccatcaatgcatggtgttcttcgactgatcgttactctcgatggtgaagatgttattgattgtgaacccatattaggctatttacacagaggaatggaaaaaatcggcaa
ataggaaagctacttaggcaggagataggggaattccttaagaaagaaaaaagaataagaacacagatacataacataaaaaaaagaataaataagacgaaattc
gacctccccctacatatttaatttcttctcctatacaaaaactagcaagacctactccattggtaattccatcaatgacacccttgtcgaaaaactgcgttagttcggttaat
cctcttatacccaaggtaaaggtcctagtatagaaaatatctatataaccgcgattatatgaccaactatatatctttttttttagttgatggaaaaaatactttttcggaccc
ccttttacaaaggaatttattaaatccaaattctgaaaaaaagagtaagcagatccataaaacatatatgctatgaatagaccaaaaatagctagacttacagaagaaat
tgcattagtgataaattcatatgaatttatggaagaattagaactttcttggaaaaagttgattgagggagttagccactttgataatatggttaattcccctatttcattatca
aaatggattcctatagatccaataaacaaagtacaaagcagtaatataagaagaggaaatagcatagtatttcccggttcatgaggatagacaaaagtgtttttagtcc
ccaatgaagtactaaaggaccctatcctatttcctgtattaacatgaattttggatagattttgtgaaaaaaaagaaactccactcttcgctgttgataaaacgaaatctct
attgactcctttagatatcctttttccccataacgatattgaatacaacgaatcctctttagtactactgtaattttgaaaatgaacacgcaaatacccatcaaaagtaagta
aatatatccgaaacatataaaacgcagttaatcctgcagtaaaagaagctattattccaaaaaaagggtgaatataaccaactattactaaggatttcatctttggaccag
aagcaagcaagaggtggaataccacaaagagaaagggtaccccataaaaaacaagttcttgtaattggaatgtattttcttaaaccacccataagaaccatattctga
cttttatctggtgaatatccaacaagaggttccattgaatgaataacagatccggatcccaagaataataaagctttcgaataagcatgagtgatcaaatggaataaag
cagcttgataagaacctatacctagagctaacatcatataacccaattgagacattgtagaataggctaagcttcttttaatatctctctgagcaagagctaaagtggct

cctaagaaaagtgttagtgtacctattaaagaaatgaaactcattatcaaaggtagggatatgaaaagaggaagaagtcgagctataagaaaaatccccgcagcaa
ccatagttgctgcgtgtataagagctgaaatgggggtgggtccttccatagcatcgggtaaccatacgtgaagagggaattgtgccgatttcgcaactgcaccaag
gaataataaaaaagcacacaaaatagtaagcaaggagttaatctcattattaggaatccagttattagctattttaaacaaatcccgaaactctaaactacctgttatcca
aaaaaaacctagaattcctaataacagaccaaaatcccctacacgattagttacaaaagctttttgacaagcactcgctgcaattggccgtgtaaaccaaaagcctat
caataaataggaacacattcccacaagctcccaaaaaaaataaatttgtatcaaattggaactagtaaccaatcccaacatggaagtattgaaaaaacttatataaaca
aaaaatctcaaatatccctcatcgtgagacatataatcatcactataaataagaaccaggattcctacagtagtaattagtattaacataatagaagtaagcgggtcgat
taagtatccaaattctaaggaaaaatcattattgacggtccaagaccatagatattgatagatagaacttccatttatttgttgaatagacaggtgaactgagaataccat
agctatacttaaaagtaaaacactaggaaaagcccatatgcgacgaagatttttttgttgctgttggaacaagaaaaagtccaaaccccattgacataataactggaag
tgggagaagagggattacccatgcatattgatatgtatgttccataagaaaagaaattgcaatttttacttgaaaattttacttcaatttttctataaaattgaaaaaagtttc
cgattcaccaaactaattcttatctatttctgaaggaataaaaaaatactagaattcttaatttttcaaaaattttctcattgaaacaatcaaaaaataagaataggttttgttg
gttaaagtcaaaaagttaatgaaataacttcgttacctagttattacctaaagaaggacttttattaaaatacaaaaaaaagattgaatcattttactttaatattttttgtatt
aaaatgaagcagctcccttgtttcgtaactcaaattgattggaattcaattctggaatactttaattaactatttgattgaattttcccttccttttatcctcccgtcttatatggg
ggataggcccccataccttatatctgtatatggagagtatacttgaaatataaattgatttaaatagaaaacctttgtatatattctatattattaaaacaaagtctaaaaaaa
atatataatatgttaaaaaactcttgtcttatccgcattagacaaaatgaagtaaaaaagaattcagaatttcaatatcttttagtatctaagtataaatactaagaaaaaga
agaaagatggattgatttgcggcaatagatgtctttcacatacaactagaaaaaagtaatttcctttttgaatggcagttccaaaaaaacgtacttcgatgtcaaaaaag
cgtattcgtaaaaatctttggaagaaaaagacttatttttccatagtacaatcttattctttagcaaaatcaagatcattttctggcgtcagcgagtatccaaaaccaaagg
gtttttctcggcaacaaacaaacaaataatagggttttgggataatatgaattgacctatccccaaaaaattccaattatttaatatgaataattaggaataattaggattaa
ttaatgagtttacttttatgtgtcgaattcctcggtacaatattcttagaacaaacctctctgatatataaaaaaagggtttttggtatactgtgacctaaatattcttttcctatc
aatgaacttttcgtaatagaatccgtataataaataaaaaaaggggattctattatgaaaagtagagtattcctgcaataagacttacaacttctacctatcttatcctaaat
taacaaaaaaattagttctatattgcaactgagaaaaaattgttccaactctttcaaagtttctattgggcaaagcaagaattttttttgttaaaaaaattcgcaacgatactac
caaacgaagtctattttaatgaagattctaatgtcctaaattctatggaatcttccaatctcgacgattcgcgagaaaataacttaatattcttttaataaacctgttatttcaa
cttagccgccatggtgaaattggtagacacgctgctcttaggaagcagtgctcaagcatctcggttcgagtccgagtggcggcagtctcgaaaaagaatacaatag
attataaaataaaatggattcaattcgaaatttccaattttgtaatgggaccttctccttatgctatttgcaactttagaacatatactaactcatatttctttctcaacaatttca
attgtgattacgattcatttaataaccttattagttcgtgaacttggggggattgcgtgattcgtcagaaaaaggaatgatagccactttttttctgtataacaggattcttagttt
ctcgttgggcttcttcgggacattttccattaagtaatttatatgagtcattgatcttcctttcatgggctctgtatattcttcatatgattcctaagatacagaactctaaaaat
gatttaagcacaataactacgccgagtactattttaacgcaaggctttgccacgtcgggtcttttaactgaaatgcatcaatccacaatactagtacctgctctacaatct
cagtggttaatgatgcatgtcagtatgatgttactaagctatgcgactcttttgtgcggatccttattatccgccgctcttctaatgattagatttcgaaagaatttagatttct
tttcgaaaaagaagaaaaatgttttaagcaaaacatttttctttaatgagattgaatatttctatgcaaaaagaagtgctttaaaaagcacctttttttcctttatttccaaattat
tacaaatatcaattaattgagcgtttggattcttggagttatcgtgtcattagtctagggtttaccctttttaaccataggtattctttgtggagcagtatgggctaatgaggc
gtggggatcctactggaattgggatcctaaagaaacttgggcatttattacttggaccatatttgcaatttatttacatagtagaacaaatccaaattggaagggtacga
agtcagcatttgtagcttccataggatttcttataatttggatctgttattttggtatcaatctattaggaataggtttacatagttatggttcatttacattacccatctaaatga
ttacataacataaaaccttaatgaaatggaaaaaacttccatttttgtgtttgatttgagaaccccttgaacgccttctcaaagggttctcaaaaattcgagatagatctaat
tagactcttttactttttctgaatttttagtatttccactatggaatatagagcggactagtagaagaaaaaaaatcctatttaggataataattggataacagagcctcta
ccctgtcaacggatagcgagagaacaaaatctggataaataccgattcctattactggtaaaaagatacagattaaaagaaagagttctcgcgggccggaatcctc
aaaattttcgtttggaacatgaaatagcttgtatccatagaacatctgtcgtaacatagataataaataaataggagttaatatcattccaattgccattacaaaagtaatta
gcatttttggcattaacagaaattttggactagtaattagtccaaaaaatactactaattccgcaacaaaaccactcattcctggtaaggcaagagaagccattgaaaa
gctactaaacatggtaaaaatttttggcattgggatagaaacccctcccagttcttcgagataaacaaggcgcattctatcacaagccgttcccgctaagaaaaaaag
tgtagccccaataaatccatgggataatatttgtaaaatagctccattgagtccaatgttggttatggaaccaattcctataataatgaaacccatgtgagagacggag
gagtaggctattcttttttttgaaattgcgttggccaagagaagttgaagctgcatagattatttgcatcgctcctattattactaaccaaggggaaaatagataatgagca
tgaggtaacaattccatattgatccgaatcaatccgtatgctcccatctttaataggattcccgctaaaagcatacatgtactgtaatgcgcttccccatgggtatctggt
aaccacgtatgtaggggtataatcggcaatttgacagcataagcaataaggaagccaaaataaaatagtatttccaatgttgcagggtatgattgattaattaatctttc
caaatctaatcttggttcgttggaaccgtataagcccatacctagaactccgattaagaaaaaaatggaaccacctgcagtatacaaaataaactttgtagctgaatag
agacgcctcttcccccccacatggataaaagtaagtaaacaggaattaattctaactcccacatgataaaaaaaagtaaaaggtctcgcgaagaaaataatcctatt
tgaccgctatacattgctagcatcaggaaatagaataatcgggaattccgggtaaccggccaagctgctaaagtagctaaagtagtcataaatcctgtcaataaaata
gatcctaatgaaagtccatcgattcccaatctccagtggaaattgaagacatctatccatttagaatcctcttttaattggattaagggatcctccaattggaaatgataac
agaatgcataagtcattagaaggaattctaataaacaaatagacatagtataccacctaacgattttgtttcccctatgaggtaaaaagaaaattaatgaacccgcaaat
atcggcaaaacaacaagtattgttaaccaaggaaaagaactcatgataaagtgataaagacaagatacgttttgaccagaaaagcccgtgctcgattattttgagca
caggcttcttcggtaaagaggaatcagacgattcaagtggaatttttttgtaacgtatcaataagatagagccatgctgcgggttgtctcaggtcctaaataaacgcgg
acacttaaaaaatctgttgggcaggcggattcgcatctcttacaacccacacaatcttcggttcttggcgcggaagcaatttgcttggctttacatccatcccaaggtat
catttctaatacatctgttggacaagctcgtacacattgagtgcatcctatacatgtatcataaatttttacggaatgtgacattggatctataaattttccttttcaacataaa
aattttcgatctggtcaaaatgaaattagtactatatcaatcaaatgtattgtagacaccagacgaagcaatggtttatccaaacttcaacaaataatgcaatatatttctta
atccgtttgtgagaaagcatgaaaagagccaagagacttgaattttgggcttcaacaatcataattatacgaattgtatatacgaattcgaattagccaataaattggct
atcgtcttttcaatataaattattgcaatattcaaattgcaatatcaatgaattgcaaaaattcaactaagtaaaaaagaatactatggaataacctactcaaaaaatagata
ttctcaaataataaatagtattcatgttaatatttcatattattaatatatgtgtccctttgtttagaagattctatgtctaattattcaaaaaattagattgattgatacgagttgat
ttcctattacgatggatggaagaaagaatggataatccaatagctgcttcagcagccgcaagggctataacaaaaattgcgaaaatgtctccttttaattggcggctat
caaatagatcagaaaatgttacgagatttagattaattgaattcagtataagttcaaggcatattagagctctaaccatgtttcggcttgtgatcaatccatagataccaat

cgaaaataaatagacactcaaaaaaagtacatgctcaaacatcattaactaactccttatcaatctcgattcatttcaatatggggacaagaattgaaccgattgaatta
attagaatagaacaattacacaacaaaagagaaaagaaggtatttgttggcagtagatgggttttactaaatcaaaattgtggttctttagtgatttattttagatttgaaat
tcttataattttgactcattctaagtatttcttattgccgagccatagtaattgcacctattaaagaaactagaagaattatggaaatgagttcaaatggaagataaaaatcg
gttgctaaatgaatcccaatttgttgaacgttatttatgagaccctgttctactatttggtttgatcttgtagtccaaagaattccataccatgacgtatctgggatagtagtc
attagtgaaaaaggaatagttatacaaacgagtgaagtgaacccatctccaatagtccaaaaattcttatctttagaccattctgagccatttacgaacattacggcaaa
tatgatcaagacatttatagctcccacataaataagaagttgtgccacagctacaaagtaggaattcaataaaatatagaataaggatatacaaacaagaactaatcct
aacgaaaaagcagaaaaagttgggttggtaagtaataccacccctagacccccctagtagaagaacaaatcccccaaatagcacaagaatttcatgtattggcccag
gtaaatccattatggataagaagaaattaatagtataaatttttttcatgaactgactaaaactaaaagattcaaggaagaaaaaagggattaggaaatttttttgtatattgt
atataagttctttctatagttagaaatcacatcacgaaaatctactctggtttaaaatcaggaataatttgcaataagcagtaggtattcgtttttctttctagttagtaaagaa
cttttggattctaacaaaaaaattctagtaatcagtaatcgttcttgaattccaagatttttcttcgtctattttactttgagttgaattcctaattgtttgaattgtgtaatctccca
ttatggagattggtaaccgactcaaagcaatttgattgtaattcaattcatgacgatcataagtagaaagttcatattcttcagtcattgataaacagtttgtcggacagta
ctcaacacaattaccacaaaatatacaaactccgaaatcaatactataattaagcaattgtttccttttaatatccttttcaaatctccaatccacaagaggtagatctatag
ggcatacgcgaacacatacttcacaagcaatacatttatcaaattcaaagtggattcgcccccggaaacgctccgatgtaattgatttttcataggggtagtgaatcgtt
ataggtaaacgatttgtgtgggataaggtaattatgaaactttgaccaatgtaccttgctgcgcgtattgtttgttgaccataactcatgaacccagttaccatagggaac
atattcgaaatatctatgaaaaaggtatgtttctttctcttgtttgagagaacttttgtgttgaaaatattcttactgttattgtattatcttattttatagtgaaacaagttgggaa
gaagttgttaataagagattgcccagggaaataggtaaaagaaatttccatccaagatttaataactgatccattctcatcctgggtaaagtccatcttattgtgatagaa
atgaagagaaataaataagctttagttaatgtaataaagatactcattgtcatttctaaaattccaaccattttattcatttggaaaaatccaaaaaaggatatatagggaat
agacaaattccacccgcctaagtagagaactgttacaaataaagaggaaactaataaatttaggtaagaaacaagataaaataaaccatatttgataccagaatattc
ggtttgataacctgctactaattcttcctccgcttctggtaaatcaaagggtaatctttcacattccgccaaagaagaaattagaaaaaccagaaaacctataggctgac
gccaaagattccatccaaaaaaaccatattttgactgtgcttcaactatatcaactgtacttgaactgttggataatcatagtcgacgataacatcacagttcccaccgct
attccaaaaccgtacatgaaaccttagtttcatacggctcctctatgatcagaaaaaaggaaagtactgtttcatttcgttattatcttcttgggcgtagttagaattatcta
agataaaatcgatttcaacgtcctaaattagaccaaaggaattctgtctgctagaataataaaaaacgcttcggaattcatctcatcctttataatataatggtactttttctt
tgttcagcaataacttaatcttggaataaaacactcgttataacaattaataaacgaaaagagttgggtattagttcatgaagaattctgtatgaatatggataaacgacg
gaaagaataaataaagatcttttttttgtattgcattccatatcttttgtcctattcttctttccccgaggggtatttaaaaagaaaaaaggaataaagggttaattcgttcttg
atagccatttccttaacaagtgaatgggaacatactctggatcggaatccgaagaaagtactacttgctcatttccaccaatttcaagtccttattatgattccttttatgag
gaaaaatatctaatgctttagattcccctcattactaatcctttatgtactttagtgtttctaatccctcactaacttttgatggattcccttatgattacaactttctgtatcggg
aatcccttattattgcccgcttcaagatatgatgactaatcaaaaaatctcaaccttggggtaaagaatttacaccgcttatgtttacttccatttttttcttgtacataggaaat
gagattttttctttttactacaaattaataagcagttttgtttcactcatatagctatctagtttaacttactaacctgaatatagaataagaaaaggaggataaatattcaatttt
taacgaatcgcacgtagagatattgctagtacacaaaaagttaatgggatttcataactaatagattgagcggcagctcgtagaccacctgaaaaagaatatttattatt
tgagctatatcctgccataagaagaccaataggagcaatacttgaaatggcaatccataaaaaaacaccaatactaagatccgctaaaacaaagcgatatcccaaa
ggaataactaaaaaacttaataaaattgatatgactgctatagacggtccaatgctaaataagggaatatcccctcgggatggcaagatatcctctttaaaaagtagct
tagttccatctgctatagcttgaagcagtcccagggggccagcatattcaggaccaatacgttgttgtatcgatgcggatatttctctttctaaccacacaattacgagta
cttctattgtgattcccagtaagagggtcaaaatgggtagaatccatatcagtccatagacttcttttaataattccaagttcgaaaaagaattgatagcttctacctgtac
cctgtctattatcatttcaacgatcaacttctcccataatgatatctatactacctaatatcgtcatgatatcagccaatttcattttttttgactagctgaggaagaatttgcaa
attaataaaaccgggtggacgaattttccatctccaggggaaaagactatcatctcctaccagataaattcctaattcaccttttggggcttccactcttgcataaagctc
ttgttttgacaattcaaaattgggtgaaggtttttttaccaagaaatcgatattcaaaatcattccattcggaattctttgctttcttaaagcgtcggacttctaaattctcataa
gggcccccaggaattttttctacagcctgttgaataattttgattgattccctcatttcaccgattcgtactaaatagcgtgctaatgaatccccttctttttgccattggactt
tccaatcgaattgattgtaagactcgtaaagatcaactttacgaagatcccattgtattccagaagctcgtaacatggggcccgataagccccaatttacagcttcttct
ccgctaataaaaccaactccctcaactcgttccaaaaaaaatgggattctgtgtaataagttgttgatattcaataactcctcgtaaaaaataatcacagaaatctaaacat
ttatcgatccatccataaggcagatcggcagcaactcctccgatgcgaaagtaattatgcatcattcgcatacctgtagcagcttcaaatagatcatatattaattctctc
tctctaaaaatataaaaaaaaggagtctgcgcaccgagatctgccataaaaggtccaagccataacaagtgagaagctatacggctcaattctaacataattaccctа
atatagctggctctttggggtatttgaatattctccaagaattctggtgcatttaccgttattgcttctgtaaacatagtagctaaataatcccaacgtgttacataaggtaa
gtattgtataatagttcggttttccgcgatttttttccattcctctgtgtaaatagcctaatatgggttcacaatcaataacatcttcaccatcgagagtaacgatcagtcgaa
gaacaccatgcattgatgggtgctgagggcccatattgactatcatgagatcttttcttgtaagcggtagactcatatcctttcttccttaattcattattccatgaaaatgg
attattccatgaattcctcaaaacgaggctcatcaaaatgcaaaatctaagactactataagactactaataaaataagaaaaaaaaattcgaacgatgaaattaccgct
ccctaatatccaactgactgattaatttcttataacgtactctatttttctttgccaaataagccagcaaacgttgacgttttcccaaaagtcttcggagacctctttccgatg
aaaaatctttttttgtgtaattccaaatgtgaagcaagtctccgtatcttattggtgaaactgaatacttgaaattcaacagaaccccagttttcttctttttcttctttaaccata
aatccaaaaatttttctacctcctttctttttcatgtatttttctgatcaggaaaaataaaaaaattatgtcagttattttgaagttattctaatctcgtacacacaaaaatttgcaat
tattcatctactactggaatttggatttattttatcgatgcaaattggatttggatagaagggtacattcttttattttagatagaagaaaagtttcttctatctaaaataaaaga
attttgctgatttatttattgctatatccaatttatggaattgatacaccgtttaattgatatcatttagcaaaatgaaacatagcatatgcatccatctttcggctcgagaattt
cacgggatagagatatggtataagaaataggctattaagtaactctaaatgaattgtggatacatctgtatccttaacatactgaaacaactgccattattcgtatcaaac
caatagcgattcatacaagttaaatcttctaatcaatggtgggccaataatgaattttttttgcatatgtattaagacgcttggcctgatttcgaaattgtccagagttttttatg
tagttttcattgcaaaatgatggatctccttccgtaactttccaattacgagtacgagaattgaaagacatgaaaattctcaattctctacggcgtctaggagatagatag
aatattttcaggaacaagaaaaccagaagaatctttctctctattcactaccattccgcgtcttcgacttctattagtttcttttcttctttaatgcaatagctatagtttgatata
gaatccatttctcaaagtaatggaaacctttctcttataggaaatggttcgaaaatcgctattccaccttttaggtatcgtgaaaagtgatacctgtgaagatcgtgcattt
cagtcacattcagatccgtttttttgagtccatgatataaccaaattggatggatcttccacccgtttagctaagaaagaatagatgcagaggtggataatagatcgatat

gaagatcatgagctgccccataatgaaaccaccaggagtcgcgaatatctccttcttccctaatccaagattggagaaagaagatctaagagggacccatggagaa
tgtggtcagaaacccataatagagtccgaccgcaacgaccgaattaattatcctcatcgagaaacttagactactaagtagaaaagatttgaaaatcatggcatgggt
ctcctttttttctttctttagagttttctatatgcacaatttctcgatgtttcgatgagaatttcttgactttccatatatagaaagagatagactataaatgacatctcttatgtca
ataagaccaaagggatggatattaaatgataggaagtgctaggaagtgaaatagaatgaaatagagccactctgggcttacctatgaaatgaggcatggaacgga
gccactacgaagaaattccgggagttacgaaagaagcttcggactcatattgttcatgggttgagagcgggagttgaactctaggaggtcgaatccccccttgttcct
cagtagctcagtggtagagcggtcggctgttaactgactggtcgtaggttcgaatcctacttggggagatttgattcattctttaatgtaagaataaagaattgaattaaa
gggcttgctttgacccttaggagtaggtaacccgttcgctatccttgtttctattgcattttatctcatcgtatcacattctgttctacgattccacttcgacaaaaggaaag
agcatacccaagttcaatagctttacgtccgctatcccgatcatggttttcctaccctcagggggaaagtaaaggcccttcccctttggaaggctgtgggcgaggag
ggattcgaacccccgacaccgtggttcgtagccacgtgctctaatcctctgagctacaggcccaccccgtctccactggatctcttcccgggggtaccccccaaaa
ggaacctccctctcctcagccatttcatttcgggttaagaagatgggaaagcccctttctctctataagaacagtgcgttccgaggtgtgaagtgggagagaggga
tgtgatgattgaggttttgaataagacgacctttgcattttggatttggatctttttcgtatttcaaaatagtgaaaaagtcaaataagaggtgttaagctttttatcattctgg
catcgagctattttgccgcaggacctcccctacagtatcgtcaccgcagtagagtttaaccaccaaattcgggatggattggtgtggttcctctacgcctaggacacc
agaatatcgaaccatgaacgaggaaaggcatgagagaaatattggctagtaattgtgaagccccaattcttgactggaagggacaccaaaggcctctgccctccct
ctctatctatccaagagatggaagggcagagcttttttttggtttttcatcttttcatcaaagagttgaacaatgaagatagatggcaagtgcctgatcgatttgatcagg
tcgtgtaggaacaaggttcaaatcgttcgttcgttaggatgcctcagctgcatacatcactgcacttccacttgacacctatttaaacggctcgtctcgccgctaccttat
cctatttccatgcttctgtcgctccatccccgtatgggtggagaacccgtcgctgtctcggctgtgctaccggaggctctagggaagtcggaggagagagcactcat
cttggggtgggcttactacttatatgctttcagcagttatcctctccgcacttggctacccagcgtttaccgtaggcacgataactggtacaccagaggtgcgtccttcc
cggtcctctcgtactagggaaaggtcctctcaatgctctaacgcccacaccggatatggaccgaactgtctcacgacgttctgaacccagctcacgtaccgcattaa
tgggcgaacagcccaacccttggaaccacctacagctccaggtggcgaagagccgacatcgaggtgccaaaccttcccgtcgatgtggactcttggggaagatc
agcctgttatccctagagtaacttttatccgttgagcgacggcccttccactcggcaccgtcggatcactaaggccgactttcgtctctgctcgacgggtgagtcttgc
agtcaagctcccttctgcctttgcactcgaggaccaatgtccgtctggcccgaggaaacctttgcacgcctccgttaccttttgggaggcctacgccccatagaaact
gtctacctgagactgtcccttggcccgcgggtctgacacaaggttagaatccgagctcttccagagtggtatctcactgatggctcgggcccccccggaaggggg
ccttcttcgccttccacctaagctgcgcaggaaaggcccaaagccaatcccagggaacagtaaagcttcatagggtctttctgtccaggtgcaggtagtccgcatct
tcacagacatgtctatttcaccgagtctctctccgagacagtgcccagatcgttacgcctttcgtgcgggtcggaacttacccgacaaggaatttcgctaccttaggac
cgttatagttacggccgccgttcaccggggcttcggtcgccggcttccctgtcatcagttcaccaacttccttgaccttccggcactgggcaggcgtcagcccccat
acatggtcttacgactttgcggagacctgtgtttttggtaaacagtcgcccgggcctggtcactgcgaccccctttgcgagggggcacccttctcccgaagttacg
gggctattttgccgagttccttagagagagttgtctcgcgcccctaggtattctctacctacccacctgtgtcggtttcgggtacaggtacccctttgttgaaggtcgttc
gagcttttcctgggagtatggcatgggttacatacttcagcgccgtagcgcctggtatgagcctcgtggagaagcaatggctagtccacggggctcatacttcagcg
ctgcagcgcttggtactcggacctcggctcgaggcattttctctacccccttcttaccctgaaaaagcagggtcaccttgtgtccttaaacctataaccatctttcggcta
acctagcctcctccgtccctccgtaccaacaaggggtagtacaggaatattgacctgttgtccatcgactacgcctttcggcctgatcttaggccctgactcaccctcc
gtggacgaaccttgcggaggaaaccttgggttttcggggcattggattctcaccaatgttttcgttactcaagccgacattctcgcttccgcttcgtcgacccccgcttt
cgcggttgcttccctctaaggcggaacgctcccctaccgatgcattttgacatcccacagcttcggcagatcgcttagccccgttcatcttcagcgcaagggcgctc
gatcagtgagctattacgcactctttaaagggtggctgcttctaggcaaacctcctggctgtctttgcacccccacctcctttatcactgagcggtcatttaggggcctt
agctggtgatccgggctgtttccctctcgacgatgaagcttatcccccatcgtctcactggccgaccttgacccctgttatttttgggtcatatctagtattcagagtttgc
ctcgatttggtaccgctcgcgcagcccgcaccgaaacagtgctttaccccctagatgtccagtcaactgctgcgcctcaacgcatttcggggagaaccagctagctct
gggttcgagtggcatttcaccccctaaccacaactcatccgctgattcttcaacatcagtcggttcggacctctgcttagtttcatccaagcttcatcctggtcatggatag
atcacccaggttcgggtccataagcagtgacaatcgccctatgaagactcgctttcgctacggctccggtgggttccgttcccttaaccaagccactgcctatgagtc
gccggctcattcttcaacaggcacgcggtcagagatcactttcccctcccactgcttgggagctcagcacggtttcacgttctatttcactacccactgggggttctttt
cacctttccctcacggtactacttcgctatcggtcacccaggagtatttagccttgcaaggtggtccttgctgattcacacgggattccacgtgccccatgctactcgg
gtcagagcgtaagctagtgatgctttcggctactggactttagccatctagggtgcggcactcaaccgcttcgcctagcagcacaacgcttgtattgctctcccacaa
ccccgttttcacggtttaggctgctcccatttcgctcgccgctactacgggaatcgcttttgctttcttttcctctggctactaagatgtttcagttcgccaggttgtctcttg
cctgctcatggattcagcaggcagttcaaaaggttgacctatttgggaatctccggatctatgcttattttcaactccccgaagcatttcgtcgcttgctacgcccttcct
cgtctctgggtacctaggtatccaccgcaagcctttcctcttttgaacctcgccattaacgttaaggctatgccatcctaaggtgctactaaatggaaggatcttatcaa
cgtccatgaatgcgaaatcatagatcgaactgccgaattggcaaaattcggtgctatcatagtatccgctaagttcacgggctggagataagcggactcgaaccgct
gacatccgccacagggtaaaccaccgcctctcaggcctccccgacgggttctaccatagaggccaacgatagacaataactccccccgaacacagcttacaac
tttcatcgtactgtgctctccaaagagcaactcttctcaaaatctcaaaacaaaaggtgctgagttggaatcccattctaaggattcttgtggttccggggaatccagct
acaggagaaccaggaacggggagctctccccttttccgcccgactctttgatcttaaacttaagaatgctggttttaagaacgagtgattgcccttctccgaccctta
ctgcccaaccggagagcggacggctaatgtgttccacttattgaacagggtctatggtcggtccgtgacccctggacgccgaaggcgtccttggggtgatctcgta
gttcctacggggtggagataatggggtcggtccatggattttccttccttttgccacatttcgctcaaagggttgaagggagatagtgcatcaagctattcgcaagggc
caacttgatcctcttccccagggatcccagatgagggaagcctaggagagccgccgactccaactatcgtccatgtacgatccatactagatctgaccaactgccc
atcctacctcctctaccttttttgacagcccatctttttgtctcagtagagtctttcagtggcatgtttcagtcctcttccccattacttagaaaaagtgagccaccggttcag
gtacaagatactaccattaccgcctggacaattagacagccaacccgtaatcgcaacgacccaattgcaagagcggagctctaccaactgagctatatccccccga
gccaagtggagtatgcatgaaagagtcagatgcttcttctattcttttccctggcgcagctgggccatcctggacttgaaccagagacctcgcccgtgaagtaaatca
tcgcccctacgatccaaccaattgggagagaatcaatagactccttttcgggagcgattcatccttcccgaacgcagcatacaactctccgttgtactgcgctcttcaa
gtgtgcttcttccccttctcccccttaccatggcaagtccttgggaaataactccgatgggcagaaaaaggaaggcgttaagagaccctcctggcccaaccctagac
actctaagatcctttttcaaacctgctctgctcccatttcgagtcaagagatagataaatagccacatcccattgcactgatcgagggcgctcgtagtgacttagggag

tcgaagaccaagaagtggcttatttataccaagcattcctcttatggctagatccaacctcctggtccctgcggaaaggaaaaagaatttcacgttcttcctttcaggaa
gggaggattagggaagtcctattgattgctgctttctccagaccgccgggaaaagcatgaaaaaaaggctcgaatggtacgatccctccgtcaccccagaatgaa
agggtgatctcgtagttcttggtctgtgaagatgcgttgttaggtgctccattttcccattgaggacgaacctcaacctgtgctcgagagatagctctccatacactgat
aagggatgtatggattctcgagaagagaggagccgtagtgcccccccggaccgcccggatcccacgagtgaatagaaagttagatctacatgggatctcacct
gaatcgccccatctatcctcctgaggagaagtttgtttggtttcaaactccgattcaaacaggaggagtacgccatgctaatgtgccttggatgatccacatcttcggg
tcaggcgctgatgagcacattgaactatccatgtggctgagagccctcacagcccaggcacaacgacgcaattatcaggggcgcgctctaccactgagctaatag
cccgtcgcgcgggcctccctttgggaggcctgctacgccaaaagcgagaaaaactccatccctttccttttgacatccccatgccgccacaccacacggggggg
ggcatggggacgtcaaaaggggatcctatcactatcaactaatttgttccgacctaggataataagctcatgagcttggtcttacttcaccctaaacgaaagaagac
ttccatatccaagtttagctcagacgtagctgccttctttttgggcgtgaagcagtgtcaaaccaaaatacccaataagcataagcattagctctccctgaaaaggagg
tgatccagccgcaccttccagtacggctaccttgttacgacttcactccagtcgcaagcctagccttaggcatccccctccttacggttaagggtaatgacttcaaaca
tggccagctcctatagtgtgacgggcggtgtgtacaaggcccgggaacggattcaccgccgtatggctgaccggcgattactagcgattcctgcttcatgcaggc
gagttgcagcctgcaatccgaactgaggacgggtttttggagttagctcaccctcgcgagatcgcgaccctttgtcccgcccattgtagcacgtgtgtcgcccagg
gcataaggggcatgatgacttggcctcatcctctccttcctccggcttaacaccggcggtctgttcagggttccaaactcatagtggcaactaaacacgagggttgc
gctcgttgcgagacttaacccaacaccttacggcacgagctgacgacagccatgcaccacctgtgtccgcgttcccgagggcacccctctctttcaagaggattcg
cggcatgtcaagccctggtaaggttcttcgctttgcatcgaattaaaccacatgctccaccgcttgtgcgggcccccgtcaattcctttgagtttcattcttgcgaacgt
actccccaggcgggatacttaacgcgttagctacagcactgcacgggtcgagtcgcacagcacctagtatccatcgtttacggctaggactactggggtctctaatc
ccatttgctcccctagctttcgtctctcagtgtcagtgtcggcccagcagagtgctttcgccgttggtgttctttccgatctcaatgcatttcaccgctccaccggaaattc
cctctgcccctaccgtactccagcttggtagtttccaccgcctgtccagggttgagccctgggatttgacggcggacttgaaaagccacctacagacgctttacgcc
caatcattccggataacgcttgcatcctctgtcttaccgcggctgctggcacagagttagccgatgcttattcctcagataccgtcattgtttcttctccgagaaaagaa
gttgacgacccgtgggccttccacctccacgcggcattgctccgtcaggctttcgcccattgcggaaaattccccactgctgcctcccgtaggagtctgggccgtgt
ctcagtcccagtgtggctgatcatcctctcggaccagctactgatcatcgccttggtaagctattgcctcaccaactagctaatcagacgcgagcccctccttgggcg
gatttctccttttgctcctcagcctacggggtattagcaaccgtttccagttgttgttcccctcccaagggcaggttcttacgcgttactcacccgttcgccactggaaac
accacttcccgttcgacttgcatgtgttaagcatgccgccagcgttcatcctgagccaggatcgaactctccatgagattcatagttgcattacttatagcttccttattcg
tagacaaagcggattcggaattgtctttccttccaaggataacttgtatccatgcgcttcagattattagcctggagttcgccaccagcagtatagccaaccctaccta
tcacgtcaatcccacaagcctcttatccattcccgttcgatcgtggcgggggggagtaagtcaaaatagaaaaaactcacattgggtttagggataatcaggctcgaa
ctgatgacttccaccacgtcaaggtgacactctaccgctgagttatatcccttccccgtcccctcgagaaagagaattaacgaatcctaaggcaaaggggcgagaa
actcaaggccacccttcctccgggctttctttccacactattatggatagtcaaataatgggaaaaattggattcaattgtcaaccggtcctatcgaaaataggattgact
atggattcgagccatagcacatggtttcataaaatctgtacgattttcccgatctaaatcgagcaggtttccatgaagaagatcttgttcagcatgttctattcgatactgg
taggagaagaacccgactcggtattcttaaaaaaagaggggaagcagaaccaagtcaagatgatatgggtcgccccttcttcttgcgccaaagatcttaccatttcc
gaaggaactggggctacatttcttttcaatttccattcaagagtttctatctgtttccacgcccttttttgagacctcgaaacatgaaatggacaaattccttctcttaggaa
cacatacaagaaaaaggataatggtagccctcccattaactacttcattttcatttatgaatttcatagtaatagaaatccatgtcctaccgagacagaatttcgaacttgc
tatcctcttgcctaataggcaaagattgacctctgtagaaagactgattcattcggatcgatatgaggacccaactacgttgcattgccagaatccatgttccatatttga
agagggttgacctctgtgcttctctcatggtacaatcctcttcctgctgagccccctttctcctcggtccacagagaaaaaatggaggactggtgccgacagttcatca
cggaagaaagaactcacagagccgggatcgctaactaatagaatagtactactaactaatactaatatatagaaatagatatctagaaatagaaacgaactaatatat
agataatcgaaattgaaaagaactgtcttttctgtatactttccccgttctattgctaccgcgggtcttatgcaatcgatcggatcatatagatatcccttcaacacaacat
aggtcatcgaaaggatctcggacgactcaccaaagcacgaaagccagttagaaaatggattcctatttgaagagtgcctaaccgcatggataagctcacattaacc
cgtcaattttggatccaattcgggatttttcttgggaagtttcgggaagaaattggaatggaataatatagattcatacagaggaaaaggttctctattgatgcaaacgct
gtacctagaggatagggatagaggaagagggaaaaatcgaaatgaaataaataaagaataaagcaaaaaaaaaataagtcgaagatagaagagcccagattcc
aaatgaagaaatggaaactcgaaaaggatccttctgattctcaaagaatgaggggcaaggggattgataccgagaaagatttcttcttattataagacgtgatttgatc
cgcatatgtttggtaaaagaacaatcttctcctttaatcataaatggaaagtgttcaattagaacatgaaaacgtgactcaattggtcttagttagtcttcgggacggagt
ggaagaagggcggagactctcgaacgaggaaaaggatcccttcgaaagaattgaacgaggagccgtatgaggtgaaaatctcatgtacggttctgtagaggga
caggaagggtgacttatctgtcgacttttccactatcaaccccaaaaaacccaactctgccttacgtaaagttgccagagtacgattaacctctggatttgaaatcactg
cttatatacctggtattggccataatttacaagaacattctgtagtattagtaagaggaggaagggttaaggatttacccggtgtgagatatcgcattattcgaggagcc
ctagatgctgtcgcagtaaagaatcgtcaacaagggcgttctagtgcgttgtagattcttatccaagacttgtatcatttgatgatgccatgtgaatcgctagaaacatgt
gaagtgtatggctaacccaataacgaaagtttcgtaaggggactggagcaggctaccatgagacaaaagatcttctttctaaagagattcgattcggaactcttatat
gtccaaggtcaatatggaaattctttcaggggttttcccttactttgtccgtgtcaacaaacaattcgaaatacctcgactttttcagaacaggtccgagtcaaatagcaa
tgattcgaagcacttctttttccattacactatttcggaaacctaaggacttgatggtatggatatggaaaatacaggatttccgatcctagcgggaaaaggagggaaa
cggatactcaattttaagtgagtaaactgaattccatactcgatctcatagatccctatagaattctgtggaaagccgtattcgatgaaagttgtatgtacggcttggagg
gagatctttcctatctttcgagatccaccctacaatatggggtcaaaaagccaaaaaaataagtgattcgtttttagcccttataaaaagaaaacggattcttgaacctct
ttcacgctcatgtcacgtcgaggtactgcagaaaaaagaactgcaaaatccgatccaatttttcgtaatcgattagttaacatggtggttaaccgtattatgaaagacg
gaaaaaaatcattggcttatcaaattctctatcgagccgtgaaaaagattcaacaaaagacagaaacaaatccactattggttttacgtcaagcaatacgtagagtaac
tcccaatataggagtaaaaacaagacgtaataaaaaaggatcgacgcggaaagttccgattgaaataggatctaaacaaggaagagcacttgccattcgttggttat
tagaagcatcccaaaagcgtccgggtcgaaatatggctttcaaattaagttccgaattagtagatgctgccaaagggggtgggggtgccatacgcaaaaaggaag
cgactcatagaatggcagaggcaaatagagctcttgcacattttcgttaatccatgaacagaatctaggtatgtagacacatggatccatacatctcgatcggaaaag
aatcaatagaaggagaatcggacgatatctttctcgaaacaaacaaaaaggaaaagaaagagaaaacagaaatcatgatcaactaagccctctcgggggcttgctt
aagaataagaaagaggaatcttatggaaatagcatggaataaggtttgatcctattcatggggattccgtaaatatcccattccaaaaatcgaaacaatcgggactttt

cggagattggatgcagttactaattcatgatctggcatgtacagaatgaaaacttcattctcgattctacgagaatttttatgaaagcgtttcatttgcttctcttccaggga
agtttcattttcccagaatgtatcctaatttttggcctaattcttcttctgatgatcgatttaacctctgatcaaaaagatagaccttggttctatttcatctcttcaacaagttta
gtaataagcataacggccctattgttccgatggagagaagaacctataattagcttttcgggaaatttccaaacgaacaatttcaacgaaatctttcaatttcttattttatt
atgttcaactttatgtattcctctatccgtagagtacattgaatgtacagaaatggctataacagagtttctgttattcgtattaacagctactctagggggaatgtttttatgt
ggtgctaacgatttaataactatctttgtagctccagaatgtttcagtttatgttcctacctattgtctggatataccaagagagatctacggtctaatgaggctactatgaa
atatttactcatgggtggggcaagctcttctattctggttcatggtttctcttggctatatggttcatctgggggggagatcgagcttcaagaaattgtgaacggtcttatc
aatacacaaatgtataactccccaggaatttcaattgcgcttatatccatcactgtaggacttgggttcaagctttccccagccccttttcatcaatggactcctgacgtct
acgaaggagtgtggttcgttcgacaaattcctacctctatatctatctctgaggtgtttgggttttgcaaaactccatagatatgcagaagagaaatgctatccccactcc
gaccaagacagaacttttaccaaaagtttattgtgatctttttgttcaaataacaattaaggtgaagcagggtcaggaacaacgaatctctttatgataaacagatccatt
ttgcaagctcgttattacgggtagttcctacaaagaatcggactaatgacgtatacaatgcttgaattatcgacgtagatgctacatagtgggttctcatccttcagaga
ctacgagtgtaataggagcatccgttgacaaaaggatcaccctaagatgatcatctcatggctattgggaacgaatcaaatcagatggttctatttctcaacctttctga
cttgctcctacggaaccaaggtcgaaaggattgaaaaagtcagtcattcacaaccactgatgaaggattcctcgaaaagttaaggattagtagttctttttcgaaatcg
atttcgaaaaagaatggattcggtcttatacatacgcgaggaaggtaatcaaaaaagaaagaagacaagttcttctttcttttatcacttaggagccgtgcgagatgaa
agtctcatgcacggttttgcatgagagaaagaagcgaggaatcctcttttcgactctgactcccccactccagtcgttgcttttctttctgttacttcgaaagttgctgctt
cagcttcagccacgcgaattctcgatattcctttttatttctcatcaaacgaatggcatcttcttctggaaatcctagctattcttagcatgatattggggaatctccttgctat
tactcaaacaagcatgaaacgtatgcttgcatattcgtccatagggcaaatcggatatgtaattattggaataattgttggagactcaaatgatggatatgcaagcatga
taacttatatgcttttctatatctccatgaatctaggaacttttgcttgcattgtattatttggtctacgtaccggaactgataacattcgagattatgcaggattatacacgaa
agatcctttttttggctctctctttagccctatgtctcttatccctaggaggccttcctccactagcaggtttcttcggaaaactctatctattctggtgtggatggcaagcag
gcctatatttcttggtttcaataggactccttacgagcgttctttctatctactattatctaaaaatagtcaagttattaatgactggacgaaaccaagaaataaccccttatg
tgcgaaattatagaagatcccctttaagatcaaacaattccatcgaattgagtatgactgtatgtgtgatagcatctactataccaggaatatcaatgaaccccattcttg
caattgctcaggataccctcttttagctgctaggtctatttcttagttcaagatccctcttactaactggaataaaagaattagtagatctgttccgcccaaaatgggaatg
ggcgctagggtaatgaacttataatcatggaatcgactcgatcatcagattataagttcattccataccggaccagaccgtgcacattcttattatgagaaggggtcatt
cgagcctatggaaataggatactctgtttacatagaaatccccacgtccttacattctatttaggattaggaataggtgtaatcagacctgcttttgacatatctatcctatc
cttatttgggtaccatatgcacctctttgggcttctattgaatcgagaaattggattgtacatctttttgattttgatacatataaggtgtcctacggataatgcaaatcgaag
ctatttgatgtctgactcaggcctatatgaccgatcgatcgaaatactccaagactccacctttgtcatatattccatatatcacattagatagatatcatattcatggaata
cgattcactttcaagatgccttgatggtgaaatggtagacacgcgagactcaaaatctcgtgctaaagagcgtggaggttcgagtcctcttcaaggcataatacgga
gaatgcgcattcaatgagcattccccgtagaagtattccggaaatctgcgcctggcgctctcctctatcttctgaggtccttaaccacttccctgagaaaaggagaca
gtaaaagccaaaatagactaaatatagcctgaacgatcctaaaaatccctcgaaggagataatataataaagaacccaaagcagacggtatcctactgcaagggtg
gtcttaagaatccaaaagaggttgctcagaagagatagatgtatcccaacctctattgctctcgcgtaaagcctttttttttacgcgacaggaaaaagtgactacgaattc
cccttttttgtttgcgaatccctgtttgtatcctttgagcgcacgcccataagtagcgatcaaggaaatcgatcaaacgatcccaataccgtgaaagagaaacttcccag
atccagggaagcttatcataaagggcttcgacaaggggttttattcgttctttgagcaaaaagataaagatcggatagattcgaaccgcaattgcaaaggtaatcact
actatgccagcccaaatcatgatcttcaccaacttggatttggttctctcgcgaaaatcgcgagttgcagagatgagaaccatgaaaagcaagatcccgaataagaa
aacagaaaccgaggaaaccacaagagtgaagactagtagagtctcgtcttttgtcattctttgctcctttttcactcaatgattcattcgaatttcccgaccaaaaattcta
tatgtctattcatagggcctcgttgctaagtgctacaagatctagtgcactggaactcgtggttatggacccgaatcctttagtatggaacattgtcttttccaagtaaaaa
cccccagtatatgaaagaatgaaaaggtgctttcgttcttttcttgtggaataagaagccctcgtaccttaatgaaaggaaaataggaatttttcattaggtatttgacca
aataggatcgtccagttcctatagaacctatcactaaaatacccgatagggctaagcggaacgaaaaggattttccctgagatggtaaatgaaaacgattagcccca
tacgaggtttgggaataagtgatgagcaaggaatatacgtctttctgctaaagaggatctattaaactcataattcattagatccttgttatcaatgtcaactaggtatcat
aagtaaacggatcccggttgttcaatcctttgataaccaaggtcattctttgctaaagagaaatgatcactatgagtcagactcaatagaattggatccattccaaatag
cgagaattaggattctggatccctctcaatctctctttcaattcgaggatccagagaggtgttttcatagtcatctccgaatatttgccatctccgaatattttcgatttcattt
ttctatgatatgtctttctatatgaaaattggttatttacgatgtacgatgatccctgttaagcatccatggctgaatggttaaagcgcccaactcataattggtaaatttgcg
ggttcaattcctgctggatgcacgcgaaccggaacgttccataagtctattggaactggctctctatccatggaatctcatccatcatccatacataacgaattggtatg
gtatattcataccataacataagaacaataagaactcgaattcttatcgatactggaactcagagcataggagggaaagtcgatttatggatggaatcaaatacgcag
tatttacagaaaaaagtcttcgtttattgggaaagaatcaatatacttttaatgtcgaatcgggattcactaagacagaaataaagcattgggtcgaactcttctttggtgtt
aaggtagtagctgtgaatagccatcgactacccggaaagggtagaagaatgggccctattctaggacatacaatgcattacagacgtatgatcattacccttcaacc
gggttattctattccacttctagatagagaaaaaaactaaaggagaatacttaataatacggcgaaacatttatacaaaacacctatcccgagcacacgcaagggaac
catagataggcaagtgaaatccaatccacgaaataatttgatccatggacggcaccgttgtggtaaaggtcgtaattccagaggaatcattaccgcaaggcataga
gggggaggtcataagcgcctataccgtaaaatcgattttcgacggaatcaaaaagacatatctggtagaatcgtaaccatagaatacgaccctaatcgaaatgcgta
catttgtctcatacactatggggatggtgagaagggatatattttacatcccagaggggctataattggagatactattgtttctggtacaaaagttcctatatcaatggg
aaatgccctacctttgagtgcggtttgaactattgatttacgtaattggaagtaaccaattaggtttacgacgaaacctagaaatcgatcactgatccaatttgactacct
ctacgggatagacctcaacagaaaactgttgagtaacggcagcaagtgattgagttcagtagttcctcatagaaaattattgactctagagatatggtaatatggaga
agacaaaattgtttgaagcacgcacagaaccggaagcgccccttgtttcaaagagaggaggacgggttattcacatttaatttgatggtcagaggcgaattgaaag
ctaagcagtggtaattaagacccccgggggaaaatagggatgtctcctacgttacccataatatgtagaagtatcgacgtaatttcatagagtcattcgatctgaatgc
tacatgaagaacataagccagatgacggaacgcggagacctaggatgtagaagatcataacatgagcgattcggcagatttggattcctttcctatatatccactcat
gtggtacttcatcatacgattcatataagatccatctgtctagagatcgtcatatacatctagaaagccgtatgctttggaagaagcttgtacagtttgggaaggggttttt
tgagagaaaagaagaatctacttcaaccgatatgcccttaggcacggccatacataacatagaaatcacacgtggaaggggtgggcaattagctagagcagcag
gtgctgtagcgaaactgattgcaaaagaaggtaaatcggccactttaagattaccatctggggaggtccgtttggtatcccaaaactgcttagcaacagtcggacaa

gtgggtaatgttggggtgaaccaaaaaagtttgggtagagccgggtctaagtgttggctaggtaaacgccccgtagtaagaggggtagttatgaaccctgtggacc
acccccatgggggcggtgaagggaaagcccccattggtagaaaaaaacccacaaccccttggggttatcctgcgcttggaagaagaactaggaaaaggaaaaa
atatagtgatagttttattcttcgtcgccgtaagtaaatacgtaactaggaatatggaaaattgcatttttggaatttgcaataatgggatgggcgaacgacgggaattga
acccgcgcatggtggattcacaatccactgccttgatccacttggctacatccgccccttatccagctaaaggattttctctttttccattcatcattattctatttattctga
cctccatacttcgatcgagatattggacatcgaatgccactctttaaaatggaaaaaaaggagtaatcagctgtgacacgaaaaaaaacgaatccttttgtagctcatc
atttattggcaaaaatagaaaaggtcaatatgaaggaggagaaagaaacaatagtaacgtggtcccgggcatctagcattctacccgcaatggttggccatacaatc
gcgattcataatggaaaggaacatatacctatttacataacaaatcctatggtaggtcgcaaattgggggaattcgtaccaactcggcatttcacgagttatgaaagtg
caagaaaggatactaaatctcgtcgttaactgaattcagaatagaaagattcaaaataaaaaaaaaaaagaaata
>O.meridionalis_IRGC105298_cp
cccaatatcttgcttcagcaagatattgggtatttctagctttcctttcttcaaaaattgctatatgttagcagaaaagccttatccattaagagatggaacttcaagagcag
ctaggtctagagggaagttgtgagcattacgttcgtgcattacttccataccaagattagcacggttgatgatatcagcccaagtattaataacgcgaccttggctatca
actacagattggttgaaattgaatccgtttagattgaaagccatagtactaatacctaaagcagtgaaccaaatccctactacaggccaagcagccaagaagaagtg
taaagaacgagagttgttaaaactagcatattggaagattaatcggccaaaataaccatgagcggccacaatattataagtctcttcctcttgaccaaatctgtaaccct
cattagcggattcgttttcagtggtttccctgatcaaactagaggttaccaaggaaccatgcatagcactgaatagggaaccgccgaatacaccagctacacctaac
atgtgaaatggatgcataaggatgttatgctctgcctggaatacaatcataaagttgaaagtaccagatattcctaaaggcataccatcagagaaacttccttgaccaa
tagggtaaatcaagaaaacagcagtagcagctgcaacaggagctgaatatgcaacagcaatccaaggacgcatacccagacggaaactcagttcccactcacga
cccatataacaagctacaccaagtaagaagtgtagaacaattagctcataaggaccgccattgtataaccactcatcaacagatgcagcttcccaaattgggtaaaa
gtgcaatccgatcgccgcagaagtaggaataatggcaccagagataatattgtttccgtaaagtaaagaaccagaaacaggctcacgaataccatcaatatctactg
gaggggcagcgatgaaggcgataataaatacagaagttgcggtcaataaggtagggatcatcaaaacaccgaaccatccgatgtaaagacggttttcggtgctag
ttatccagttgcagaagcgaccccacaggcttgtactttcgcgtctctctaaaattgcagtcatggtaagatcttggtttattcaaattgcaaggactcccaagcacacg
tattaactagaaagataatagaaggcttgttatttaacagtataatatagactatataccaatgtcaaccaagccagccccgacagttgtatatccatacaacaaaattta
ccaaaccaaaaaattttgtaaatgaagtgagtgaaaaatcaaaactcagattgctcctttctagtttccatatgggttgcccgggactcgaacccggaactagtcggat
ggagtagataattattccttgttacaatagagaaaaaacctctccccaaatcgtgcttgcatttttcattgcacacgactttccctatgtagaaataggctatttctattcca
aagaggaagtctactaatttttttagtagtaagttgattcacttactatttattatagtacagagaacatttcagaatggaaactgtgaaagttttaccttgatcatttatcaat
catttctagtttattagttttgtttaatgattaattaagaggattcaccagatcattgatacggagaatatccaaataccaaatacgctcactgtgcgatccacggaaagaa
aagtaagttgttttggcgaacatcaaagaaaaaacttgctcttcttccgtaaaaaattcttctaaaaataccgaacccaaccattgcataaaagctcgtaccgtgcttttat
gtttacgagctaaagttctagcgcatgaaagtcgaagtatatactttagtcgatacaaagtcttctttttttgaagatccactgtgataatgaaaaagatttctacatatccg
accaaaccgatcaagaatatcccaatccgataaatcggtccaaattggtttactaataggatgccccgatccagtacaaaattgggcttttgctaaagatccaatgaga
ggagtaacagggactttggtatcgaatttttttcatttgagtatctattagaaatgaattctccagcatttgattccttactaacaaagaatttattggtacacttgaaaagtac
cccagaaaatcgaagcaagagtttgctaattggtttagatggatcctttgcggttgagtccaaaaagagaaagaatattgccacaaacggacaaggtaacatttccat
ttcttcttcaaaagaagagttccttttgatgcaagaattgcctttccttgatatcgaacataatgcataaggggatccataacgaaccatatggttttccgaaaaaaagca
gggtacattaacccaaaatgttccatcttcctagaaaagatgattcgttccagaaaggttccggaagaagttaatcgcaagcaagaagattgtttacgaagaaacaac
aagaaaaattcatattctgatacataagagttatataggaaccgaaatagtcttttattttctttttcaaaataaaaatggatttcattgaagtaataaaactattccaattcg
agtagtagttgagaaagaatcgcaataaatgcaaggatggaacatcttggatccggtattgaaggagttgaagcaagatatccaaatggataggatagggtatttct
atatgtgctagataatgtaagtgcaaaaatttgtcttctaaaaaaggaaatattgaatgaatagatcgtaaattctgaaactttggtatttctttttcttccggacaagactgt
tctcgtagcgagaatgggatttctacaacgatcgcaaacccctcagatagaatctgagaataaaactcagaataaaaaaaaattgttgtaatccaataatcgatcttggtt
aggatgattaaccaaattaatccaaaaattctgctgatacattcgaatcattaaccgtttcacaagtagtgaactaaatttcttgttattagaaccaagaatttcgacaagtt
cggaaccatttaatccataatcatgggcaaacacataaatgtactcctgaaagagtagtgggtagacgaaatattgtctaggaaatttaagtttttctgaataaccctcg
aatttttccatttgtatttctacttgaatcagagagagagaaatatttctcggtttatcaaatggtgatacatagtacaatatggtcagaacagggtgttgcatttttttaataca
aaccccctggggaagaaaaggagtctaatccacggatctttttccgctccttttctatccaatttgtttatgtttgttctaattacaaaagagaacaaatcctttatttttgcag
gccaattgctcttttgactttgggatacagtctctttatcaatatactgcttcttttacacattcaatccataacatccttttcaatccaaaatcaagaataattaggatttctaa
aaaaaaaaagaaaaaatcaaaggtctactcataggaaaacctgcttttccctacatcaggcactaatctatttttaacgtctaattagatcagggagttcttccaattaag
aagttaagctcgttgctttttgttttaccagaattggagccaggctctatccatttattcattagacccagaaaatcagaatttttttattccattccaaaaatccaaaataag
aaattgattttattacgacatgctattttttccattcattacccttgaggatcagtcgcggtcttatagactctaccaagagtctggacgaatttttttgcttcatccaaatgtgt
aaaagatcatagtcgcacttaaaagccgagtactctaccattgagttagcaacccagataaactaggatcttagatacgatcgaaatccaaaaatcaatggaattaca
ccgcacacccctgtcaaaatcttaaaatagcaagacattaaaagaaagattttatcaccattgaaaacactcagataccaaaaggaacgggtctggttaaatttcacta
aggttaaaagtggcaccaatcacgatcgtaaaattgtcatttttttagcatttttatttaaataaataaataaatcttgtatgagagtacaaacaagagggacaaccctacc
atttgagcaaagtgtaggcaaaaaacctaatagggagtgaggataaagagacttatccatctacaaattctagatgttcaatggacctttgtcaatggaaatacaatgg
taagaaaaaaattagatagaaaaactcaaaaaaataaaggcttatgttggattggcacgacataaatccagtcaaaaataggattaagaaagaggcaaattatttcta
aatagttagacaacaagggatactagtgagcctctcctagtttttttattcatttagttcttcaattaactcaaagttctttctttttctttaaagaattccgccttccttaaaatat
cagaaacagttcttgtaggttgagcaccttttttcaaggaaatagagaatagctggaacatttaaacaagtttgattctttatcggatcataaaaacctacttttcgaagatc
tcttccttctcttcgagatcgaacatcaattgcaacgattcgatagacagcttattgggatagatgtagataaacaaagcccccccctagaaacgtataggaggttttctc
ctcatacggctcgagaatatgacttgcattaatttccgtacagaaaaaacaaatttcatttatactcatgactcaagttgactaattttgattgacagacttgaaagaaaaa
aatcctttgaaatttttttgagtcgtctctaaactcttttctttgcctcatctcgaacaaattcacttttattccttattccggtccaattctattgttgagacagttgaaaatcgtgt
ttacttgttcgggaatcctttatctttgatttgtgaaatccttgggtttaaacattacttcgggaattcttattcttttttctttcaaaagagtagcaacatacccttttttcttatttc
cttcgataaagcatttccctcttctatagaaatcgaatatgagcgattgattctgatagacttttaatcaaaagagttttcccatatcttccaaaattggactttcttcttatttta

acctttgatttctatattatttcgatttctatattaagggtagaatgacaaagttggcctaatttattagttttcactaaccctagattctttcccttgataaaaaataaattctgt
cctctcgagctccatcgtgtactatttacttagcttacttacaaacaacccagcgaaaattcggttcgggacgaatagaacagactatgtcgagccaagagcattttca
ttactatggaaaatggtggatagcaaaatccacaatcgatcgtgtccttcaagtcgcacgttgctttctaccacatcgttttaaacgaagttttaacataacattcctctaa
tttcattgcaaagtgttatagggaattgatccaatatggatggaatcatgaatagtcattagtttcgtttttgtatactaattcaaacttgctttgctatctatggagaaatatg
aataaaagaaattaagtatttatcgggaaagactccgcaaagagccaatttatttaaacccatattctatcatatgaatgaaatatagttcgaaaaaagggaataaacaa
gtttgcttaagacttatttattatggaatttccatcctcaacagaggactcgagatgatcaatcctgaaatgataagagaagaattgactcttctccaacaaataaactatc
aacctcccgtttaattaatttaattaatatattagattagcaatctatttttccataccatttttccgtaacaaaactaattaactattaactagttaaactattgcaatgaaaaga
aagtttttggtagttatagaattctcgtatttcttcgactcgaataccaaaagaaagaaaaaaatgaagtaaaaaaaaacgcatttcctgtaaagtaaaattaaggtcttt
gcttttacttatttttcttttacctaaaagaagcaactccaaatcaaaattgaatccattctatctaacgagcagttcttatcttatctttaccgggatggatcattttggatatt
taaaaaatcgcggatcgagatcgttttcgcttaaccaaagaaagaaaaagaagaaggaaccttttttactaataaaatactataaaaaaaatttatctctatcataaatct
atctctaccataaaggaataggtctcgtttttttatacaatgttctacgtcaagtttaaaattttttcatgaaaaaaagattttcaatttgactggacttgacactggattatgtttt
ctgagacagaaaatgaacgcattaggactgcatcgaatctaagagtttataagagaaaaaaattctctttaataaactttatgtctcgtgcagaatacaatacgatttcat
ctttcgtttcatcagaaaaaatctgggacggaaggattcgaacctccgagtaacgggaccaaaacccgctgccttaccgcttggccacgccccatttcgggttttatg
cgacactaataaacagtattatgtttatttcttattcgtcaatcctacttcaattacataaaaatggggggtattctcttggtaggattctagacatgcgaataatatagaatc
caaaaaatgcattgatcattacatggaattctattaagatattatatgaaagtcgaatttcttccactctcatttgagagtgcgaatacaaggaggtattttgtgtttgggaa
agtccgaagaaaaaaggattttgaatcctccttttcctttttcccttagaaaaataactcaatcaaaatccaattatctactctacaagaacgaaacgcttgttatgcctaat
atacttagtttaacctgtatttgttttaattctgttatttatccgactagtttttttcttcgccaaattgcccgaagcttatgccattttcaacccaatcgtggattttatgcctgtcat
acctgtactctttttttctattagcctttgtttggcaagctgctgtaagttttcgatgaaatctttactactctgtctgccaaattgaatcatgtattcattctaaaaaaaattcgaaa
aatggataagagccgagaagtcttatattatgaaccttcgattctaaaattcaaattcttctacattgaatgtatagctgcagcaataaatttggatcagcctttctactccc
tgcatctacgttgagcaggtatctttaggtaaccgcacaatacctaacctaatttattgataagagtgcttattataaatcaattcttgcaattttttttcaaaaattgatttttgc
atttttaggtgtcaaaataaacaaaacccatcctagtggatttgtgtggtaaggaaaaacgggtaatctattccttaaaaaaaaaatcttggagattatgtaatgcttactct
caaactttttgtttatacagtagtgatattctttgtttccctctttatctttggattcttatctaatgatccaggacgtaatcctgggcgtgacgagtaaaaatccaaaattttttc
ttacaaattggatttgtttcatacatttatctacgagaaaatccgggggtcagaattccttccaattcgaaagtcccaaacgatccgagggggcggaaagagagggat
tcgaaccctcggtacaaaaaaaattgtacaacggattagcaatccgccgctttagtccactcagccatctctccccgttccaaatcgaaaggtttccgtgatatgacaga
ggcaagaaataacgattgcaaaaaatccttcctttttctttcaaaacaaaagttcaaaaaaaattatattgccaattccattttagttatattctttttttcttaatgttaataaaaa
aaagaagaaaattcttcttttttctttctaattctaaaattggatattggctaaaagacaatcagatagattttctctttagcaggcatttccatataggacttgttataataaaa
caagcaggttatagaaaagaaaaaaactctttttttttattatttatcaacaaagcaaaaaggggtcttatcaaaccaacccaccccataaaattggaaagaaagataaa
gtaagtggacctgactccttgaatgaggcctctatccgctattctgatatataaattcgatgtagatgaaattgtataagtggatttttttgtatttccttagacttagaccac
gcaaggcaagaatttctcgctatttactatttcatattcttgttactagatgttctataggaataagaagaaatcgcaacccctttccgctacacataaaaatggatttcga
aagtcaatttttcttttcaatatctttacttttttttcagaatcctatttttgttcttatacccatgcaatagagagcgagtgggaaaagagaggttacttttttttcatttttcccttaa
aaaataggctttcttggaaataggaatcatggaataatcctgaattccaatgtttatttctatagtataagaaaaactaattgaatcaaattcatggatttaccacgacctc
ggctgtgaccccatagataaaaatgcaaaatttctatcttcgagaccattgaaaaaaggcattgaacgagaaaaaatcgtccacagataatctatcgtatgccttgga
agtgatataaggtgctcggaaatggttgaagtaattgaataggaggatcactatgactatagcccttggtagagttactaaagaagaaaatgatttatttgatattatgg
acgactggttacgaagggaccgttttgtttttgtaggatggtctggcctattgctttttccttgtgcttatttcgctttaggaggttggtttacagggacaacttttgtaacttc
ttggtatacccatggattggcgagttcctatttggaaggttgcaatttcttaaccgcagcagtttccacccctgccaatagtttagcacactctttgttgctactatggggc
ccggaagcacaaggggattttactcgttggtgtcaattaggtggtctgtggacttttgttgctctccatggggcttttgcactaataggtttcatgttacgtcaatttgaact
tgctcggtctgttcaattgcggccttataatgcaatttcattctctggcccaatcgctgtttttgtttccgtattcctgatttatccactggggcaatccggttggttctttgcg
ccgagttttggcgtagcagcgatatttcgattcatcctcttcttccaaggatttcataattggacgttgaacccatttcatatgatgggagttgccggagtattaggcgcg
gctctgctatgcgctattcatggggcaaccgtggaaaacactctatttgaggacggtgatggtgcaaataccttccgcgcttttaacccaactcaagctgaagaaact
tattcaatggtcaccgctaatcgcttttggtcccaaatctttggtgttgctttttccaataaacgttggttacatttctttatgctatttgtaccggtcaccggtttatggatgag
tgctattggcgtagtcggcctggctctgaacctacgtgcctatgacttcgtttcccaggaaatccgtgcagcggaagatcctgaatttgagactttctacaccaaaaat
attcttttaaacgagggtattcgtgcgtggatggcagctcaggatcagcctcatgaaaatcttatattccctgaggaggttctaccacgtggaaacgctctttaatggaa
cttttgttttagctggtcgtgaccaagaaaccaccggttttgcttggtgggccgggaatgccagacttatcaatttgtcgggtaaactacttggagctcacgtagcccat
gcaggattaatcgtattctgggccggagcaatgaacctatttgaagtggcccatttcgtaccagaaaaacccatgtatgaacaagggttgattttacttccgcacttag
ctactctaggttggggagtagggcccgggggagaagttctagatacttttccgtactttgtatctggagtacttcatctaatttcctccgcagtcttaggcttcggtggca
tttatcacgcgcttctgggaccggagactcttgaagaatcttttccattctttggttatgtgtggaaagatagaaataaaatgactacaattttgggtattcacctaattttgt
taggtataggtgcttttcttctagtactcaaagctctttattttggcggtatatatgatacctgggctcctgggggaggagatgtaagaaaaattaccaatttgacccttag
ccccggtgttatatttggttacttactaaaatccccttttgggggagagggttggattgttagtgtggatgatttagaagatataattgggggacatgtatggttgggtttc
atttgtgtatttggcggaatttggcatatcttaaccaaacccttcgcatgggctcgccgtgcatttgtatggtctggagaagcttacttgtcttatagtttaggcgctttatct
gtctttggttttatcgcttgttgttttgtctggttcaataatacagcttatccgagtgagttttatggacctactgggccagaagcttctcaagctcaagcatttacttttctagt
tagagatcagcgtcttggagctaatgtgggatctgcccaaggacccacaggtttaggtaaatatctaatgcgttccccaacgggagaggttatttttggaggggaaa
ctatgcgtttttgggaccttcgtgctccatggttagaacctctaaggggccccaacggtttggacttgagtaggttgaaaaaagacatacaaccttggcaagaacgac
gttcggcagaatatatgacccatgctcctttaggctctttaaattctgtgggtggcgtagctaccgagatcaatgcagttaattatgtttctcctagaagttggttagcgac
ttcccatttgttctaggattcttctttttgtgggccatttgtggcatgcaggaagagcccgggctgctgcagcaggatttgaaaagggaatcgatcgtgatttggagc
ctgttctttacatgacccctcttaactaagattttcttatttatacctgttctactgttttttctgttctggctcggttattctatctagccgagccattcattcctttctatgaaag
aaagataaggggacagaacaaaaaaaaacaaataaagaaacaaacgtattcaatacgcaaaagaaaagagagaggaaagcaaaaggagagagagggattcg

aaccctcgatagttcctagaactataccggttttcaagaccggagctatcaaccactcagccatctctccacagcctaatccttattttattcctacaaatagaacatagc
catacgaaatgactcactaacctctagaaacatctcaaatacaaatcccttttcgatatatttctgtatactgtatccatgtatacaggatccgctatatctgcttgtgaaat
aaagcataaaatcccctcaaccccatatccaaataaaaaaagtggtgagtaataagttttaaagagaagaatcaatggattcatgattaaacccctcctacttcttgtatt
ttattacaattttgattaagtgagggatcaaatataatatgtagtcaactttatttgatggtaacttggaggattagaaatatgactattgctttccaattagctgtttttgcatt
aattgtgacttcctcagtcttagtcattagtgtacccttgtatttgcttctcctgatggttggtcaaacaataaaaacgttgtattttccggtacatcattatggattggactg
gtctttctggtagctattctaaattctctcatttcttaaatgtgtttagtatttagtagcccgatacaaaataaaaaagggccgtttattcggattgtgagacgcattaaaatg
caatttgcgccccgaattgattgacagacaattaaattaataaaaagaaaactcaaatagaaaatgaaacggtcgacccagacatagacggtcgacccaggcggat
ataccctataaaatataggacgtagcgagcgtagttcaatggtaaaacatctccttgccaaggagaagatacgggttcgattcccgccgctcgccagcttaatttagt
aaggtactatgataaaaaatttagtctacttttattaaagtaataggtgttagtctagtaccgtacccctttactatcttagccccctttgcaccccactcaaaaaaagagc
actacagcggcgggaatcgaactcggcaacagggttccctaaaccggggattcaccgaaacaaacaaccagcaaacagcttttaaagggaagggagatagact
gtgcctttctttcatttatttttcttttctgcaaggtagggggccttgagagttcctcttgtggtagcaagttacttcgcaacctgctcaatttggccttatagggtcgggaa
ctaatgaataaaaagggttggataccgcccaaccacccagccctctaccatatctagacaaatagaatagtccttttatacagactgctaagtgcggagacgggaat
cgaacccgtgacctcaaggttatgagcctcgtgagctaccaaactgctctactccgctctggagcgctggaaatcggtggacgaaaaaggttgaatacaggcctct
accatgtctagacaaatagaatagttattttatacagaatggagcgggtagcgggaatcgaacccgcatcgttagcttggaaggctaggggttatagtcgacgttggt
tgattattattaacgtctctaattcaaaaccgaacatgaaattttgatttcattcggctcctttatgggtattctcaccacttaacatctaagtcagctttttttgtctgaatggaa
ccaaggctctctgctttctagatgatccctaaagagtaggagatagaaatttgcctaaatctatctaatctaattacttcgttccctaatttcatttaagagatcctgaggaa
aagaattgggtttccaccgagctgaaacaatatgctgatggttctagcaaaccaaaactaccgtttttttagctatttggcttccatttcctttttttaacaaaagaagatttag
ttacgattggaaataaattttttttgtatcttcatccatagatcctttactcatattttaaaaattggaatccttaatccaatgcaaaattatgcttcgcgactctgtactcataatc
caaatcctatttgtttttttggatgcaatttcaattagctttgggtacaaatcgcgaaaatgcatattcttcctcaatatgctattgagagaaaaaggagtaaatcctttctaag
aactaaagttttcatcggaatataaaaaaccttaaggacgccttaagtatatcatttcaaattcagttattaatagaacgaatcacacttttaccactaaactatacccgcta
catgtaaattatgataccaacgctaccctttgtcaagggtagccattcgagaaagatgctaattccctcttagttaatgaaacagagaaggttcctcacagttagcggtt
ggtacttcgatcgcgggcctttcctttactttcttttttgttcagaattgaacaaagaatttggggaagaaaacatcttccccccacttatcatgaaatctgggccatagaga
aagcgtgagatgttttttttttatttatcatagactttccctatggcttgagagaaacaataaataacttaaagaaaaaggcacataggagccgaaggatttacttgatgta
aagaagattctgaatgtctctgcttagtcgattctctccgtttaactttttcttctcttcttttccactcaattctagtttattagattcttgtttaaaagaatcaaagaagatgaat
agaactaagaacacacaaaaaagagcatataggcccgagaccattaccaaaagttcttcccaataatcatattgggtatctgttcccttcctttcctattaggatgggg
catgttggattttccatatccatataccatcgaaccttaagggttccagaaccctcctttttttgctagttttcggaaacggaaaaccctgaaccaggagcagtataaattc
tactgcccgccttttacaagaaaattggtgataaaactccactacagtttgttcaaaatgcaccaatcagaatcccttgagaatattcaagtaccctatttctaagagaag
aggtacctgctgaaaatcgcttcaacaaatccgcttaagaaaaattgcaaagttttgaactcgaaggtttttccaagggatgcctgttaaaaattgtttcaacctctcacc
aaaacagacaagaagtatatcactgaaaattaatacccagccatatgggtatatgaagggcgcgaattcgtttataccccacccaattagaggaaataaaacataaat
ggagaaagttttcatgataagatcagccaatagaagaaaaaagtccctaatttttcagaccgttctgagcatgtgaaaagtcaatagcctaaagataaaaaaccctata
ctttgtgcaagtgataagagagaatatgaaagattttcctatttttcttgattttcttaagattttttttgaacctgaccatgaatagacttatatatctcgatatatacagatataa
tgtacattatggagtagacctataatgggaaatgaaagtggctaattttggaattgaataagaagcccttttaactcagtggtagagtaatgccatggtaaggcataagt
catcggttcaaatccgataaagggcttttttttacttagtggtagagtaatgccgcggtaagacgtgagtcagtggttcaaatccgatacagtacttttctactaaattcatt
cactttctttctttttgaaaatttctctttttttttattgaatttgataacttagtgcgagatgcatgcatttttagtttaaacactaagcgaagcaggggggtgtaaattgcaaaaa
agaaattggactctttttcctattagatcaatcaaatcactacccgtactgaactaatatagaatcccttttattaatctattcttattccatatcctttataaacgaatttcccta
aaaagtaggggatgatccgtgaattaacctaaccatcaactaaaaaaaaatcctacaaaagcataatggaaaagtaggaaggactccttgctttggatctagttatact
cttcgagtatattgacaattccaaaaaactgctcatactatcattatagtataatgaggagcggttgtatacggccctatcgtctagtgatgcccctatcgtctagtggttc
aggacatctctctttcaaggaggcagcggggattcgacttcccctgggggtagggagtattatgaaaggaggttaatcatagattatcaaaaaccctagaataaattc
ttcctgggtcgatgcccgagcggttaatggggacggactgtaaattcgttgacaatatgtctacgctggttcaaatccagctcggcccaaaaatctagggcttcgtga
atatgagttaaatccatttttttttcttccataaaaaagaatatttgatccatagaaataaaagaaataaaggataaaaagaaaaggggaaatatctttctaatctatatctctt
tcattcctctcttacaaacaaaagaccttttcttattggttattgaaaggtggattcttatctatttttagcgataataaatcgcgacatactagttatgtcattctcactatacc
cccatacgatacgggggtatgtagtatatgattcgtctatttcttagagtaggacaggcgaatattcttattcttatggttccatttaagaataagtatgccatacgccccg
cggggattgtagttcaattggtcagagcaccgccctgtcaaggcggaagctgcgggttcgagccccgtcagtcccgaactagggtccaatgaatggagaaattca
tctttcctttttccatgaaaaaagggggggcaggaagcaagatcaaatacctatggggtacccctatttcactttttttagttcgcgtttctcagtaaagagaggagagtat
aggaatttttttatcactacttctggttgatagcgaaagacatacatatcatacgtggaagggatcctcctatgttatactattccactctcaaccatgaattgatttgatag
atccgatattcataatattgaattgattcagtattatcagaatgcaagtcctccccttgaatttacaggataccctttccctctccatgggattacatcccgagttattgcga
aaaaaaagaggttatggaagtcaatattctcgcatttattgctactgcattgttcattctagttcctactgcctttttacttattatttatgtaaaaacagtcagccaaaatgatt
aattggaagttcaattaatcattgaagaaatgaaaaagggattaaataaaaaaaaaatccaagtcttaaatgaaaggatccggttggaatcataaagtgtggtagaaa
aaactacatatagttttttctaccacactttagagtctttctattatattatcttgaatctacatagaatagattactagattgaaatagtagtctaattcaatttctttttttcactgc
atccacttaatttcaatcaagtcaaaatgaaaaaatccatggagggagagaaaaataatatgagaatagactatagtaaaagaaaaaagtaaaaggaaaaaaccag
cgaatctttcatgcttaaacatgtcgcgaaatgtttcaaaagagcataaaatttattttaagaactaagaataagaaaagagtataaaacaaatggaaaatgtgcgatat
gttgggaatagctccgcggaagaaaatctaaaattcttatgtatagaactttttttaaccatgggtcgcttctagtagcgattatgaattgctctcaccgctctttctattttct
attctttctattctactatatagaaatagaaagaattatatagaaatagaaagaatagaaagactctttcttacaagagtttcttacaggagtgaaacaaaattaaagaaa
gagagaaagaataaagttcggcaaaatgattaatgcaaaacggtcaattaaagaaaagagttgatacaacaattcgactactcaatcaattagtagtatccctagagt
ccactcctcccccatactactagtgaaagagaaaatgtaaagactaccattaaagcagcccaagcgagacttactatatccatgtaaattatgtctcctatttctatgaa
ggaattattctactattgatgaataatcatagtagaatcaagggtacagagtcaaaaaggggttctgacctaaggctatggatgaatcagttcaaagaatttactcttaa

caaattcttagagtatttctggtagaattggggagcattaagtataaatatgatacatagccctttcttattaataaaagaataaggaaacgctatctcatccctattggtat
cggtttgggccactactgctaaaacaaaccccagtttgaggaaagaacggtgggttctcaaaatccagtatcgccgagccttgttattctcttgccccaacttatgcg
gggtgcaaatttgtcgatttggatcagtactataagcctaagtattttattgatcaggcggcacccagatttgaactggggataaaggatttgcagtcccctgccttacc
gcttggccatgccgccaaaaaatacgatctaaaatcgagaaaagagcaagtattcatgcacgtttcttactaaaactaactttcttttatcttaaatctaattctacttacttt
tttccaatctttttcaaaaaatctattcatgcttttttggatccagtttcgattattctcctcaaaggattctatcttaaaacacacattgctaacactagaaaacttccctttttct
ttctattgaaatgaaaaaggagaaaagtggatttctagtcacaagctacaaaattaagaacaaattggaaccattaactagaattctctttttattttgaatttcggtattctc
tcccgcctgccatttaatggcataataaaagacaatggatttatgcctaatccgtatataggtaaactccaggtccgaacagcattattatctatggatccccccttatgta
catatctctgtggagaatcgttctttaatttttcattgcattaaatatcttgaataaaaaaaagaaaattgactttgctatgtggaggtccagaactagattggcatgtactta
aaaaagtacttactttattttaggattctacaacgaaatcttatatcttatattttatagaaattctactactacgaaacaaaaaagaaccttcaaattctttttgaaattaaact
aagcgtgctattctcaatcgaactaaagtcaaactttctagtgcttataaattattatcttttggttttatcccattcatagaaaggagaaaaaatgagaaatctttgccgtcc
aatctaagaatatcataaactgtaagtggcagaatttttttcgaggttctaggaatgttttatcacttcattttcattccatttggtggacccctgggaaattcgaactttcgtt
gaaattgtctctattcatatgtatgaaatacatatatgaaatacgtatgtggagttcccgagaatttcatgtgatttagtaaacagaatatagattccataattgctagatcg
atctgtagggattgatgaagagtgagctgataatggaatttttcttcgataaacaggaaacttaagattaagatgctccggaatggaaatgagggaatgtccacaata
cccggatttagtcagatccaattcgagggattttgtaggttcattaatcaaggcttggcagaagaacttgagaagtttccaacaattaaagatccagatcacgaaatttc
atttcaattatttgcgaaaggatatcaattgctagaaccttcgataaaagaaagggatgctgtgtatgaatcactcacctattcttccgaattatacgtatccgcgcgatta
atttttggtttcgatgtgcaaaagcaaaccatttctattggaaacattcctataatgaattccttaggaacctttataataaatggaatataccgaattgtgatcaatcaaata
ttgctaagtcctggtatttactaccgctcggaattagaccataaaggaatttctatctacaccgggactataatatcagattggggaggaagatcggaattagcaattga
taaaaaagaaaggatatgggctcgcgtgagtagaaaacaaaagatatctattctagttctatcatcagctatgggttcaaatctaaaagaaattctagataatgtttccta
ccctgaaattttcttgtctttccctaatgctaaggagaagaagaggattgagtcaaaagaaaaagctattttggagttttatcaacaatttgcttgtgtaggtggggacct
ggtattttcggaatccttatgtgaggaattacaaaagaaatttttttcaacaaaaatgtgaattaggaaggattggtcgacgaaatatgaatcgaagactgaatcttgatat
acctcagaacagcaccttcttgttaccgcgagatgtattggccgctacggatcatttgattggaatgaaatttgaaacgggtatacttgacgatgacgatatgaatcact
tgaaaaataaacgtattcgttcggttgcggatctgttacaagatcaattcggactggctcttggtcgtttacaacatgcggttcaaaaaactatccgtagagtattcatac
gtcaatcaaaaccgactccacaaactttggtaactccaacttcaacctcgattttattaataactacttacgagaccttctttggtacatatcccttatctcaagtttttgatc
aaaccaatccattgacacaaacggttcatgggcgaaaagtgagttgtttgggtcctggaggattgacggggagaactgcaagttttcggagccgagatatccatcc
gagtcactatgggcgtatttgtccaattgacacgtccgaaggaatcaacgttggacttactggatctttagctattcatgcgagaattgatcattggtggggatccgtag
agagtccattttatgaaatatctgagaaagcaaagaaaaaaaaagagagacaggtggtttatttatcaccaaatagagatgagtattatatgatagcagcaggaaatt
ctttgtccttgaatcggggtattcaggaagaacaggttgttccagctagataccgtcaagaattcctgactattgcatgggaacagattcatgttagaagtatttttccttt
ccaatatttttctattgggggttctctcattccttttattgagcataatgatgcgaatcgagctttaatgagttctaatatgcagcgccaagcagttccgctttctcggtccga
gaagtgcattgttggaactggattggaacgccaaacagctctagattcgagggtttctgttatagccgaacgcgagggaaagatcatttctactaatagtcacaagat
ccttttatcaagtagtgggaagactataagtattcctttagttacccatcggcgctctaacaaaaatacttgtatgcaccaaaaacctcgggttccgcggggtaaatcca
ttaaaaaaggacaaattttagcggagggggctgctacggttggtggggaacttgctttaggaaaaaacgtattagtagcttatatgccatgggaaggttacaattttga
agacgcagtattaattagcgaacgtttggtatatgaggatatttatacttcttttcacatccgaaaatatgaaattcagacggatacaacaagccaaggctccgctgaaa
aaatcactaaagaaataccacatctagaagaacatttactccgcaatttggacagaaatggagttgtgaagttggggtcctgggtagaaacaggcgatattttagtag
gtaaattaacgcctcagatagcgagcgaatcgtcctatatcgcggaagctggattattacgggccatatttggtcttgaggtatccacttcaaaagaaacttctctcaa
actaccgataggtggaagaggacgcgttatcgatgtgaaatggatccagagggatcccctcgacataatggttcgtgtatatattttacaaaaacgcgaaatcaaag
ttggggataaagtagccggaagacacgggaataaggggatcatttccaaaattttgcctaggcaagatatgccctatttgcaagatggaacgcctgttgatatggtttt
caatcccttaggagtaccctcccgaatgaatgtgggacaaatatttgaaagctcgctcggattagcaggggatctgctaaagaaacattatagaatagcaccctttga
tgagagatatgagcaagaggcttcaagaaaacttgtgttttcagaattatatgaagccagtaaacaaacaaaaaaatccgtgggtatttgaacccgagtacccgggaa
aaagcagaatatttgatggaagaacaggagaccccttcgaacagcctgttctaatagggaagtcctatatcttaaaattaattcatcaagttgatgagaaaatccacg
gacgctctactgggccctattcacttgttacacaacaacccgttagaggaagagccaagcaagggggacaacgaataggagaaatggaagtttgggctttagaag
gatttggtgttgctcatattttacaagagatacttacttataaatctgatcatcttatagctcgccaagaaatacttaacgctacgatctggggaaaacgagtacctaatca
cgaggatcctccagaatcttttcgagtgcttgttcgagaactacgatctttggctctagaactgaaccatttccttgtatctcagaagaacttccaggttaatagggaag
aagtttgatcggaataaatctaaattcttttcttatttctattttatgattgaccaatataaacatcaacaacttcaaattggactcgtttcccctcaacaaataaaggcttggg
ctaacaaaacactacctaatggggaagtcgttggcgaagtcacaaggccctccacttttcattataaaaccgataaaccagaaaaagatggattgttttgcgaaaga
atctttggacccataaaaagcagaatttgtgcttgtggaaattctcgagcgagcggagctgaaaacgaagacgaaagattttgccaaaaatgcggagtagaatttgtt
gattctcggatacgaagatatcaaatgggatacatcaaactcgcatgtcccgtgactcatgtgtggtatttaaaaggtcttcctagttatatcgcgaatcttttagataaa
ccccttaagaaattggagggcctagtatatggcgatttctcttttgctaggcccagtgctaaaaaaccaactttcttacgattacgaggtttattcgaggatgaaatttcat
cctgtaaccatagcatttcccccttttttctaccccaggctttacaacatttcgaaatcgggaaattgcgacaggagcaggtgctattagagaacaattagcagatttg
gatttgcgaattattttagagaattcctcggtcgaatggaaggaattagaagacgaggggtatagtggagatgaatgggaagatagaaaaagacgaataagaaaag
tttttttgattagacgcatgcaattggcgaaacattttattcaaacaaatgtagaaccagaatggatggttttgtgcttattaccagttcttcctcccgaattaagacccattg
tttataggtctggggataaagtagtgacttcggatattaatgaactttataagagagttatccgtcggaacaacaatcttgcctatctattaaaaagaagtgaattagcgc
cagcagatttagtaatgtgccaggaaaaattggtacaagaagccgtggatacacttcttgatagtgggtcccgcgggcaaccaacgagggatggtcacaataaagt
atacaaatcactttcagatgtaattgaaggtaaagaggggaggtttcgcgaaactctgcttgggaaacgggtcgattactcggggcgttctgtcattgttgtgggtcct
tcactttcattacatcaatgtgggttacctctagagatagcaataaagctttttcagctatttgtaattcgcgatttaatcacgaaacgtgctacttctaatgttaggattgct
aaaaggaaaatttgggaaaaggaacccattgtatgggaaatacttcaagaagttatgaggggacatcctgtactgttgaatagagcacctaccctgcatagattagg
catacaggctttccaacccactttagtagaggggcgtactatttgtttacacccattagtgtgtaaaggtttcaatgcagactttgatggggatcaaatggctgttcatct

acctttatccttggaagctcaggcggaagctcgtttacttatgttttctcatatgaatctcctatctcccgctattggggatcctatttgcgtaccaacccaagacatgctta
tcggactttatgtattaacgattggaaaccgtcgaggtatttgtgcaaatagatataatagttgcggaaactatccaaaccaaaaagtaaattacaataataataatccta
agtatacgaaagataaagaatctctttttctagttcctatgatgcactgggagcttatagacagaaacaaatctgtttagacagtcccttgtggctccgatggaaactag
atcaacgcgtcattgggttaagagaagttccgattgaagttcaatatgaatctttggggacttatcgtgagatttatgcccactatctagtagtgggaaatagaaaaaag
gaaatccgttctatatacattcgcaccactcttggtcatatttctttttatagagaaatagaggaagccatacaaggatttagtcaggcctattcatacactatctaaacaa
ggaagttagattcggggatgcccctttcgaggggcattccgatttcgctagtatcatcatttttgccgcacgaatccagattgagattgaggaaaggaagttaactaag
ttttcgaatcactgactcaggcccattgtcgaatcctactcagcaattgtcgaattatactcagccgaaaaagggggtacttatttatggcggaacgggccaatctggt
ctttcataataaagagatagatggaactgctatgaaacgacttattagcagattaatagatcatttcggaatgggatatacatcccatatactggatcaaataaaaacgc
tgggcttccatcaagccactactacatcgatttcattaggaatcgaggatcttttaacaataccctctaagggatggttagtccaagatgcggaacaacagagttttcttt
tggaaaaacactattattatggggctgtacacgcggtagaaaagttacgccaatccgttgaaatctggtatgctacaagtgaatatttgaaacacgaaatgaattcgaa
ttttcggataacagatccttctaatccagtctatctaatgtctttttcaggagctagaggaaatgcatcgcaggtacaccaattagtaggtatgcgaggattaatggcgg
atcctcaaggacaaatgattgatttacctattcaaagcaatttacgcgagggactttctttgacagaatatataatttcctgctacggagcccgcaaaggggttgtagat
actgctgtacgaacggcggatgctggatatcttacacgtagacttgttgaagtagttcaacatattattgtgcgtagaagagattgtggtactatccaagctatttctgtg
agtcctcaaaatgggatgacggaaaaactttttgtacaaacattaattggtcgtgtattagcaaacgatatatatatcggttcacggtgcattgccactcgaaatcaaga
tattggaattgggttagtcaatcgattcataaccacctttcgagcacagccatttcgagcacaaccaatatatattagaacccccttacttgccggagcacatcttggat
ctgtcaattatgttatggtcggagttccactcatggcgatctggtcgaattgggggaagctgtaggtgttattgcgggtcaatctattggggagccagggactcaacta
acattaagaacttttcatactggtggggtattcacagggggtactgccgaccttgtacgatccccttcaaatggaaaaatccaattcaatggggatttggttcacccca
cacgtacccgtcatgggcagcctgcttttctatgttatatagacttggatataactattcagagtcaggatattctacatagtgtgactattccttcaaaaagcttgattcta
gtgcaaaatgatcaatatgtagaatccgaacaagtaattgcggagattcgtgccggaacgtccgctttgcattttaaagaaaaggtacaaaaacatatttattccgaat
cagatggggaaatgcactggagtaccgatgtttatcatgcgcccgaatatcaatatggtaatcttcgtcgattaccaaaaacaagccatttatggatattgtcagtaagt
atgtgcagatctagtatagcttctttttcgctccacaaggatcaagatcaaatgaatacttattccttttctgttgacggaaggtatatctttggcctctcgatggctgatgat
gaggtaagacatagactgttggatacttttggtaaaaaagatagggaaattcttgattattcaacgccggatcgaatcatgtccaatggtcattggaattttgtctatcctt
ctattcttcaaaataattttgatttgttagcgaaaaagcgaagaaataggttcgccattccattacagtatcatcaagaacaagagaaagaaccaatatcctgttttggga
tttcgattgaaatacccttttatgggtgttttacgtagaaatactatagttgcttattttgacgacccacgatacaaaaaagataaaaagggttcaggaattgttaaatttaga
tataggaccctagaggacgaatataggactcgagagaaagactcagagaacgaatacgggagcccagaaaacgaatataggacccgagaggaagaatgtaaa
accctagaagacgaatataggactcgagaggaggagtatgaaaccctagaagatgaatatgggatcccagagaacgaatatgaaaccctagaagatgaatatgg
aatcctagaggacgaatataggactcgagaggaggaatccgaggacgaatatgggagtccagagaacaaatataggccccgagaggataaatatggcactttag
aggaagactcagaggacgaacatgggactttagaggaagactcagaggaagactcagaggacgaatatgggaacccggaggaagattccgtcttaaaaaagg
gggttttgattgagcatcgaggaacaaaagaatttagtctaaaataccaaaaagaagtagatcggtttttttcattctccaagaactgcatatcttgccgagatcctcat
ccctaaaggtacttgacaatagtattattggagtggatacacaactcacaaaaaatacaagaagtcggctgggtggattggttcgagtgaagagaaaaaaaagcca
tacggaactaaaaatcttttctggagatattcattttcctgaagaggcggataagatattaggtggcagtttgataccactagaaagagaaaaaaaagattcgaagga
atcaaaaaaaagggaaaattgggtctatgttcagtggaaaaaaattctcaagagcaaggaaaagtattttgttttggttcgacctgcagtcgcgtatgaaatgaacga
agggagaaatttagcaacacttttcccacaggatctcttgcaggaagagggtaatctccaacttcgacttgtcaattttatttctcatgaaaatagcaagttaactcaaag
aatttatcatacgaatagtcaattcgttcgaacttgcttagtattgaattgggaacaagaagaaaaagaggaggctcgtgcttcccttgttgagataagagcaaatggt
ctgattcgcgatttcctaagaattgggttaatcaaatccactatttcgtatacacgaaaaaggtatgatagcagaagtgcaggactgattctccataataggttagatcg
caccaataccaattccttttattccaaggcgaagattcaatcacttagccaacatcaagaagctattggtaccttgttgaatcgaaataaagaataccaatctttgatggt
tttgtcggcatccaactgttctcgaattggttttttaagaattccaaaaatcccaatggggtaaaagaatcgaatcctagaattcctattccaaaatttttggggctctttcg
aaatttttcgggactcttaggtactattgcacctagtatatcgaatttttcttcatcttactatttactaacgtataatcagatcctgttaaaaaagcatttgctccttgacaattt
gaaacaaaacttcaaagtacttcaaggacttaaacactctttaataaatgaaaatcaaaggacttcaaatttcgatagtaacatcatgttggatccattccagttaaattg
gcactttctccctcatgattcttgggaagagacatcagcaaaaattcaccttggacaatttatttgcgaaaatgtatgtctatttaaatcgcacataaaaaaaatcaggtca
aattttcattgtaaatatagattcctttgttataagagcagctaagccttatttggccactacaggagcaactgttcatggtcattatggagaaatcctttacaaaggggat
aggttagttacgtttatatacgaaaaagcgagatctagtgacataacgcaaggtcttccaaaagtagaacaaatcttcgaagcgcgttcaattgattcactatcgccga
atctcgaaaggagaattgaggattggaatgagcgtataccaagaattcttgggggtccttggggattcttgattggagctgagttaaccatagcccaaagtcgtatct
ctttggttaataagatccaaaaggtttatcgatcccaaggggtacagatccataatagacatatagagattattatacgccaagtaacatcaaaagtgcgggtttccga
agatggaatgtctaatgtttttttcacctggggaattaattggactattgcgagcagagcgagcagggcgggctttggatgaatcgatctattatcgggcaatcttattgg
gaataacaagggtttccctgaatacccaaagtttcatatctgaagcaagttttcaagaaactgctcgagttttagcaaaagctgccttacgaggtcgtattgattggttg
aaaggcctgaaagaaaacgtagttctgggggggattatacctgttggtaccggattccaaaaatttgtgcaccgttacccacaagacaagaacctttatttcgaaattc
aaaaaaaaaaactatttgcgtcggaaatgagagatattttgtttctccatacagaattagtttcttctgattctgacgtaacaaacaatttctatgagacatcagaatcacc
atttaccccatttatatgatttaaggatacataaagcagatttttttactttaaactagatttttgaccttagaacgctaagaggttagattttctatttttattttaatttaaaaaga
agtttagttaattcattaaggttatgcttataccatgtagaaggttccatcggaacaattatttatttcaagctatttcggctctttcttaatcttcgaaaagaaagaaattt
cgtaatggaaaggtaggatgaaaaaaaagaaaaaatcaaaaggaagtgtggaaaaaaatgacaagaagatattggaacatcaatttgaaagagatgatagaagcg
ggagttcattttggtcatggtattaagaaatggaatcctaaaatggccccttacatctcggcaaagcgtaaaggtactcatattacaaatctcgctagaaccacccgttt
tttatcagaagcttgtgatttagtttttgatgcagcaagtcagggaaaaagcttcttaattgttggtaccaaaaaaagagcagcggatttagtagcatcagctgcaataa
gggctcgttgtcattatgttaataaaaagtggttcagtggtatgttaacgaattggtcgattactaaaactagactttctcaatttagagatttaagagcagaagaaaaga
tggaaaaattccaccatctcccaaaaagagatgtggcaatcttgaagagaaaattatctaccttgcaaagatatctcggcgggatcaaatatatgacgaggttgcctg
acattgtgatcgtcctcgatcagcaaaaagagtatatagctcttcgggaatgtgccattttggggattcctactatttctttagccgatacaaattgtgacccagatctcg

cgaatatatcgattccagccaacgatgacactatgacttcaattcgattgattcttaacaaattagtatttgcaatttgtgagggccgttctctctatataagaaatcattga
ttaagaagaatagttaattcttgggcaactgcgtagatttatggaatcacttactattctttttgttttgcatagaaaaaagacggggaatattgatatatattagagggtat
tgatatatattatcatctgatgtgatttcttgatatcctaaatataagattaatacttcacgttgctgagttgagaaaaagatggttgaatcaaaagaattctttttttgaagttc
atttttatcaggggacaatatgaatattataccgtgttccattaaaacactcaaggggttatacgatatatcgggcgtagaagtaggccaacacttctattggcaaatag
gaggtttccaaattcatgcccaagtacttatcacttcttgggtcgtaattactatcttgctaggttcagttatcatagctgttcgcaatccacaaaccatcccaaccgatgg
tcagaatttcttcgaatatgtccttgagtttattcgagacttaagcaaaactcagattggagaagaatacggtccctgggttccctttattggaactatgttcctttttattttt
gtttcgaactggtcgggtgctcttttaccttggaaaattatacagttaccccatggagaattagcagcgcccacgaatgatataaatactactgttgctttagctttactca
cgtcagcggcatatttttatgcgggtcttagcaaaaaaggattgagttatttcgagaaatatattaaaccaactccaatccttttaccaattaacatattagaagatttcac
aaaaccattatcgcttagttttcgacttttcgggaatatattggcggatgaattagtcgttgttgttcttgtttctttagtccccttagtagttcctataccggtcatgtttcttgg
attatttacaagcggtattcaagctcttatttttgcaacgttagccgcagcctatataggtgaatccatggagggtcatcattgaattgactagttttcaaaatagtcttttttt
tagcttaactcaattcatgcatggttgcggaaaattcgcttggttggaaaacaaaatagttagaattgcgtatgaatatacaatctagagttgtagaagagagaatagg
ctatattacggaattgccaaacaaagtatataggcattagggaggggcggagtcaggctagatctatatcctttatgtctataagttcagtcatcttttgtatgggtttcca
ctttaaggaattttttttgaatccgattcaatagaaaatgagaaaatacacaaacaaaatagaagaaacaaattgatatgggatattatatattcccaagttagattcattat
ctaatccgatatatggaatcggattccatatccaattcgatgcagcatattgttatcaattggatatcttgatttaattcctattggatctggattaggtcgatttccataggg
gttcttcctctatttcaccttttattatgaattagatgataggggaaaaaatagaaactcaaggatatcgaagaggaaagaaagaaggatggaatgaaagatcagttgg
ttggaaagaaagagaaatagaatagtgagtacacaaacctctaatgattagaaactaaaaaggagatctcgaagcagttcggagaattcagattatcgtttcaatttgt
actttttagttacttctgtccaatagagcttagaaatatgaatttcttggttgattgtatccttaaccatttctttttttttgacacgaggaactcatcatgaatccactaattgctg
ctgcttccgttattgctgctggattggccgtaggtcttgcttctattgggcctggagttggtcaaggtactgctgcaggacaagctgtagaaggtattgcgagacagcc
agaagcagaaggtaaaatacgcggtactttattgcttagtctagcttttatggaagctttaacaatttatggactagttgtggcactggcgcttttatttgcgaacccttttg
tttaatcctaaaaaagaaaacgagtcctttagattagatactttcttctttttttagtaaattggtatttgcttctgcaattccaattatatcaatactttactcctaatttattactc
ctagagttttctatttatggggacagacaataccccaccccaggaatagctgatttgaggatgatcaatttagaggatatgttcgccgtcttgcttcccgccctttgttta
gggcagtggaaagtatttttccttttattttaggaattttgggaacatttcaacaaaggaggtctttcacaggtcaaacgagatttaagacttaatctaaaagaaattacta
aattgaatctatttctattaaaaaaaattgcattaaaaaaaccgatcaaaaagggcgagcgaagtaagtgatcgaaaaactttgttctttgttcgtcctatctataagagg
agagcatatgaaaaatgtaacccattctttcgtttttttagctcactggccatccgctgggagtttcgggcttaataccgatattttagcaacaaatctaataaatctaactg
tagtggttggtgtattgatttattttggaaagggagtgtgtgcgagttgtctatttcaagaatagattggatctatccggctgcactttagaatatttttttagtatttttcgaata
aataagaaaagggtgcacgatctcgacgaattacttctgaataaatgcagaaatcatatgaaagaaccatagcatttcgcgacctattggtaaatcaaatcaactttga
ttctctatagaccaataatatgagaccattaacacggttaaagctaaactgcttgaagtctaggcaaaagggggtactctttctacaactatattagtattagtaccaaat
gctttaaacaggaaatagctaatgtagaatttatctgatatagaacactcatatcgataaaatggtttgaactatttactagaaggggcaccctgccctttttttatccaatg
ccgaatcgacgacctatgtataaaaaaggagaaagtttttggatttgaagaaaaaagtaggaattctatccattttcattttccatttttctatttagtttttcttaatgaaattg
aaaattttaactaaagggcaaatacaaataaaaaaacaactttgctgcccacgatagatttttatctaggcggaagagtcctcttaatatttatctagtcttatatgggtttc
ggtatattgaaatataaacagaaaagagagggtagaggataggctcattacattaaaaaaaatatggaagtagccatagcaaaaaaagaaaaaagggagcgtga
gagccaaatgaatcgaaagattcatgtttggttcgggaagagatcataaaaattgtaaacttaatagcaaggtaatctactttcattaaaagatttattagataatcgaaa
acagaggatcttgagtactattcgaaattcggaagaattgcgtagaggaaccattgagcagctcgaaaaagctcgaattcgattacagaaagtcgaactagaagcg
gatgagtatcgaatgaatggatactctgagatagaacgagaaaaagcaaatttgattaatgccacttctattagtttggaacaattagaaaagtctaaaaacgaaaccc
tttattttgaaaaacaaagggcgatgaatcaggtccgacaacgggttttccaacaagccgtacaaggagctctaggaactctgaatagttgtttgaataccgagttac
atttccgtacgattcgtgctaatattagcattctcggggccatggaatggaagagttaaattaattaggccttgaacttctactttcctttagaatttaggcattatttttcccc
ttgcttccgaaaaaaaaatagtaaagaaacactaatggcaacccttcgagtcgacgaaattcataaaattctccgcgaacgtattgaacaatataatagaaaagtagg
gattgagaatatcggtcgcgtagttcaagtgggggatgggattgctcgtattataggtcttggtgaaataatgtcaggcgaattagtcgaatttgcagaagggactag
gggtattgctctgaatttggaatccaaaaatgttgggattgtattaatgggcgatgggttgatgatacaagagggcagttttgtaaaagcaacaggaagaattgctcag
atacccgtgagcgaggcttacttgggtcgtgttataaatgctctggctaaacctattgatgggagaggcgaaattgtagcttcggaatctcgcttaattgaatctcctgc
tccgggtataatttccaggcgttctgtatatgaaccccttcaaacggggcttattgctatcgattcgatgattcctattgggcgcggtcagcgagagttaattattgggg
acagacaaaccggcaaaacagcagtagctacagatacaattctcaatcaaaaagggcaagatgtaatatgtgtttatgtagctatcggtcaaagagcatcctccgta
gctcaagtagtaactactttccatgaagagggggccatggaatacactattgtagtagctgaaatggcggattcccctgctacattacaatacctcgctccttatacgg
gagcagccctggctgagtattttatgtaccgcgaacggcatactttaataatttatgatgatctctccaaacaggcacaagcttatcgccaaatgtcccttctattaagaa
gacccccggccgcgaagcttacccaggggatgtttttatttgcattcacgccttttagaaagagccgctaaattaaattctcttttaggggaaggaagtatgactgct
ttaccaatagttgagactcaatctggagacgtttccgcctatattcctactaatgtaatctccattacagatggacaaatattcttatccgcagatctattcaatgccggaa
ttcggcctgctattaatgtgggtatttccgtttccagagtaggatccgcggctcaaattaaagccatgaaacaagtagctggcaaatcaaaattggaattagctcaattc
gcagagttacaagcctttgcacaattcgcctctgctctcgataaaacaagtcagaatcaattggcaaggggccgacgattacgagaattgcttaaacaatcccaagc
aaatcctcttccagtggaagagcagatagctactatttatatcggaacaagaggatatcttgattccttagaaattggacaggtaaagaaatttctggatgagttacgta
aacacctaaaagatactaaacctcaattccaagaaattatatcttctagcaagacattcaccgaggaagcggaaatccttttgaaggaagctattcaggaacaactcg
aacggttttcccttcaggaacaaacataaattttgcacgtctactcttgttagtagaagtagtagaggagaaatcgttgagaaagatttttcatttgaatcatgcaaaaaa
gttttcttagtttttagtatagttatttaaagaatagatagaaataagattgcgtccaataggatttgaacctataccaaaggtttagaagacctctgtcctatccattagaca
atggacgctttttcatattttcttctttcttttcatttttctcggaccaagagaaaaaactgttagaccaaaactcttttaggaaagaaaaacaaatgcatatacaaatgtat
gatgcatatatcataaagaaggaatatggagcgggtagtgggaatcgaacccgcaaccccacggttatgagccttgtcagctaccaaactgttctatcctgttaaact
aaagagaggggaactagtggataaaagggggggttgaatacgcccctctaccatatctatacaaatagaatagtccatttatacagaatggtaaagagggctcttcta
cgatcatcaattccagaaatccatacaaatacgaaagggtattttatccttaccaactggatcttgttgcacccggtaacaaacatgcataaaccatttctcgaagtatgt

gtccggatagcccaaagtctcgatagttagctctaggtcttccggtcaaaaaacaacgtcgatgaaggcgtgtcggtgcactattacgtggcagggattgcaattttt
ctcgcattttcgtttttcactcaaactcaaagggtaaactttgcttcttatctttttttttgaagatcgacgaatcaaatgatatttctgttctaatttctgccgcttcctctctctc
tgaatcaaactttttttgccataatgtgccgttcctactatctaccaagtatacggttctaatcctagatggaaaaataaatagaaaaagaaatctaagaaggcggatcc
tccccctccatcaagagtaatgaactaggtgctgatacagtacaacaaaattaactaaattaaccaaacttgcctgatgttgaggcaatcaagaaagcggcataagtg
aatatataacccacggaaaagtgagctaatcccaccaatcttgcttgcacaatggaaagagccacgggcttatctctccagcgaattaaattagccaaaggtgtccg
ttcatgagcccatgctaaagtctcaattaattcctgccaatatccacgccaggaaattaagaacataaatccagtcgcccaaacaagatgtccaaataagaacatcca
cgcccataccgataaactattcatcccaaaaggattatatccattgataagttgtgaagagtttaaccataggtaatctcttaaccatcccatcaaataagtggaggattc
attaaattgtgaaacgttgccctgccataatgtgatgtgtttccaatgccaataaaaagtaacccatccaatggtatttaacatccagaaaactgccaaataaaacgcgt
cccaagcagaaatatcacaagtaccgccgcgaccagggccgtcgcaaggaaaactatatccaaaatctttttatccggcattaatttggaaccgcgtgcatctaaa
gcaccctttactaaaatcaatgtagttgtatgcaaacctaaagcaatagcatgatgaaccaagaaatctccaggtcctattgttaagaaaagcgaattactattctcgtta
acagcattcaaccatccgggtaaccatagggttcgacccgcattgaaagcgggaccgctcgttgaagataagagtatatcgaacccatatgtcgtcttaccatgagc
cgattgtatccattgagcaaatataggttcgatcaagatttgcttttctggagtaccaaaagcaagcatgacgtcattatgaacataaaggcccaaggtatggaatcct
aggaagagactagcccaacttaaatgagatatgatagcttctttatggtctaacattcttgccaatacattatcctcattctgttccggattgtaatctctaatgaaaaaaat
agctccatgagcaaaagcccctgtcatgatgaaccctgcaatgtattggtgatgagtatataaagctgcttgagtagtaaagtcttgtgctatgaatgcataagaaggt
aaagagtacatatgttgagctactaaggaagtaataacccctaaagaagctagagcaagacctaattgaaaatgaatcgaattattgattgtgtcgtaaaggcccttat
gcccacgccctaatcgacccccggaggagtatgtgcttctaaaagatctttgatactgtgcccaattccgaagttagttcgatacatatgaccggcaatgagaaaaa
taaatgcaatagctaaatgatgatgagcaatatcggtcagccacaaactttgtgtttgtggatggaatcccccaagaagagttagaatagcagttcccgctccttgagt
ggtaccaaataaatgattactcgaatcggggttttgggcataaagattccactgacccgtcagaaggggtcccaacccctgtggatagggtaatacatctaagaaatt
attccatcgaacgtactctcccctggatgctggaatagcgacatgaactaaatgtcctgtccaagccaaagaacttaccccgaaaagtcctgacaaatgatgattga
gacgagattccgcgtttttgaaccacgaaaggcttggtttccatttgggttgtagatgtaaccaacccccctattaaggatagggtagaaagaaataatagaaaaagag
ctcctgtataaagatcttcattggtccgtaatccaattgtataccaccactgataaaccccagaataggcgatattcactggaccggcggcgcctcctcgagtaaagg
cttccacagcgggttgaccaaaatgaggatcccaaatcgcatgagcaataggtcttacgtgtaaaggatcctgtatccatgattcaaaatttccttgccaagcgacat
gaaacagatttccggacgtccatagaaagattattgctaattgcccaaagtgagaagcaaaaatgttctgataaagacgttcctcagtaatatcatcatgactttcgaa
atcatgtgcggtagcaataccaaaccaaatacgacgagtagtggggtcctgagctaagccttggctaaacctgggaaatcttaattccataatgcctttcaaatcctc
ctagccactatcctactgcaataattctcgctaagaagaatgcccatgttgtggcaattccacccagaaggtaatgggttactcctacagcacgtccttgtataatgctc
aaggctctaggctgagtagcaggagcaacttttaatttgttatgagcccaaacgatagattcaatcagttcttgccaataaccacggccgctgaataaaaacattaaac
tgaaggcccagacaaaatgagcacctaagaaaaaaagaccatatgcggataatgaagaaccataagactgaattacttgggatgcctgtgcccacaagaaatctc
ggagccacccattaatcgtaatggaactctgtgcaaagtttccccctgtgatatgagttaccaccccttgatcacttatagtaccccaaacatccgactgcattttccaa
ctgaaatggaaaatgactaccgaaatggaattgtacatccagaatagacctaagaaaacatgatcccaggcggatacttgacatgttccccctcgcccaggcccat
cgcaagggaagcgaaaaccaagatttgctttatcgggtatcaaacgggaactgcgagcaaataaaacacctttcaaaagtattaatacagtcacatggattgtaaat
gcgtgaatgtgatggactaaaaaatctgcggttcctaatggaataggtaacaaagcaactttgccgcctactgctactaactcgccacctccccacgttaagctggta
cttgttgttgcaccaggagctgttacgctaggcgcgccagcatggagattttgtacccattgagcaaagatcggttgtaattgtatggcggtatccgaaaacatatctt
ggggacgacctaaagcactcatggtatcattatgaatgtataaaccaaaactgtgaaaacctagaaatatacatacccagttaaggtgggatatgattgcatcacggt
gtctaaggacacgatctaatagatcgttgtatcgagtcgttggatcatagtctcttaccataaaaatggctgcatgtgcagcagcaccaactattagaaatccgccaat
ccacatgtggtgtgtgaacaaggaaagttgtgtaccatagtcagtagctaggtatggatagggggggcatagagtacatatgatgagctacaacaatggttgtagagc
ctagcatagctaggttaagagataattgagcatgccatgacgttgttaggatttcatagagacctttatggccctgtcctgtaaatgggcctttatgagcctccaaaatat
ctttcaggccatgaccaataccccagttggtcctatacatatgacctgcgatcaggaaaagaatagcaatagctaaatgatggtgcgcaatatcgctcaaccataga
ccgccggttattggatctagccctccgcgaaaactaagaaattctgcgtatttggaccaattcaaggtgaaaaaggggttgctccttcggcaaaactaggataaagt
tgagccaaaaggtcgcgattcaagataaattcatgaggaagtggtatctccttaggatcaaccccagcgtcaagaaattggttaatcggtaaagatacatggatttgg
tgccccgcccaagaaagagacccaagtcctaataaccctgctaagtgatgattcaacatggattctacgtcttggaaccaggctaatttgggagcggctttgtgataa
tggaaccacccagcaaaaagcattaacgatgcaaaaatcaatgcaccgattgcggtacaatagagttgtaattcactagttattccggatgctcgccaaatctgaaaa
aacccggaggttatttggattcctcggaaacccccgcctacatcaccattcaatatttcttgccctacgattggccaaactacctgagcactgggtccaatgtgagtag
gatcacttagccatgcttcataattggaaaaacgggcaccatggaagtacatgccactcaaccaaagaaagataatggagagttgaccgaaatgagcactaaaga
cttttcgagagatctcctccaaatcacctgtatgactatcgaaatcgtgagcatcagcatgtaggttccagatccaagtggtagtatcagggcccttagctattgttcttg
agaaatggccgggtctggcccattcctcaaaagatgtttttacaggatccctatccacaacaatttttacttctggttccggcgaacgaatcatcattaagtcctcctcttt
ccggacaagacatacaaagagacccgccaactttttagtgaacctttgaaagatagatattatgattagtccttttctttactatctaccgtccttctattttttttttagttattc
actggagcaattatatattgaagtcaatctgaggcaagtgttcggatctattatgacataaggattgggtgcctaacggactttttttttatcttggatttctccacgtaacaa
aaaaaccttttttttaatttaaaaagctagtctattttttttaagagtataagtccctatctatatctactttccttgagcataatatagatttttttattcgattccaaattccaagat
aactcattagaattattaataagatggtcctgatatattagcaatatttatattgccccttttttattcgctttattacttctattctagaccctatcgtttatccttatgaaatataat
ataaaatagaaggcagaggaaagagatataatgaaattcttgattcgatcttccgacctaatttatttgattaatggatcaacaaccaaacccccatttatgaaaaagg
agagtggtcttattcaaattcaaagcgcttcgtaatcttcaaccagttctgtgcttcaatataatttcccggagtaagcgctatagcttgtttccaatactcagcagcttgat
caaaccaagcttctgcaatttctgaatcaccctgtagaatggcctgttctccccggtcggaataggtagttccttcccctagaaccgtacttgagagtttcctacctcata
cggctcagaaattgctatcttaatttcccttgtcttaactgaattcgatttctcaaaaatcgatccaatttcttcttgggttacgcagaagaagttaattacctaagtttcaaa
ccctaaattttgatcaataatcagtttgatcttttctcccacctgcagaaaaatgaagcatagatagacctatatccttcgtccgaattttctgaaaggtaactatctcggttt
catatatgaaatttctatagaatccttgaaaaagactttttccccataagcaagaaaaaagaacttactatctttgggatctgatactacaccgctgcttaatcccttagtg
gatcggctctattacataagcggattcctaaattttgccccatatcatgggataagtaagcagttttttttagttgtatcgacccagtcggtcactaattgatctttacggtg

ctttctctatcaatttgagaactctatccatagagtagtataggccatactttctttcttcctattttgattctcgtgaagtgtcctttcttcctacagctgatagggcaaaatcg
ttgttttgacgatccctatgtagaaagccccctttctagtaaatactagaaaatttgatcctttcttatttttctttctatagtggagatagtcgcacgtaatgacagatcacg
gccatattattaaaagcttgtggtaagaaggggtttcgttctagtgcccggaaataatattccaaagcctttgtatgctctccattgcttgtgtgtataaggcctatgttata
gagtatataacttcgatcatagggatcaatttctagtcgcgtagcttcataataattttgcaaagcttccgcataatttccttcggattgagccaacatccgttacggtcgtt
cgttctattcaaaaaatctccgttccaaaaccgtacatgaggttttcacctcatacggctcctcccttctgtacatagtactaagcgaaaaaatctagagaataaaaatag
aattagttccatctcattatggaccgaaaggggctggtatttttccaagaaatctctagccaaccttcccacaagaggtttttcttaacaccaatgaattctattaatgcta
gaggaaaacgatagctccaagaatttctttgttctcaacgcctcctatttagaggaattagccacttcaacgatctttgatggttataggggtatccaaagtacaaacttg
atggttgtttgttatcccaaccattcttcccagccctgataccaatcaggaaagggttaatttctaacaaagtttttctcttgttgattcctatttctaggtgtagtgcttttatc
ccctatgctacctattagtactagtagagtaggattagcctgtaatacagaacctatcctgtaggtgtaacctttcgctcaatactaaaatctacaattgaagcatccgag
gccgcgtcagtcgaggatacacgacagaaggagttgttagttcacctcaccttccctaagcgtgggtttcccttactaatttggttttctctccgcgaaccccccgctctt
tctcgtaaaaccggggtgtaggtagggctaaaaaaacaaaaaaaaagagtcaaatcgcaccatctctataataagtaaatgcccttttttccccggaggttgtcggaat
tattcgcaataaaatattggctacaattgagaaggtcttatcaatgaaatttccatttatacgggatctaggcataattcccaacccattctatcattctatatagaattctttt
cattccttcacaaaataacataaaaaaaaaacaaatccattcaattcttataaatcgatccctatgctccaaatggataagagaggtatttctgctcagcccaaattctctc
tttttccttctgtttgaacaagaagagatagaaaatatttgactaagattggatttcattccactttcctatttcttttctcaacaacaacttctctcatcaactatttcgcattttc
aaagtcattaatcgtcccatacccctattttctatttgattgtatggggtaggataccttatgcaaacagaattctagggttcctttttttatcgaataagaagaattcttccattc
tctttttctttggtttggggaaaacccaaactaaaacttttcgagggagcggaattcctagtaaaaaaatcctggacccgtccacttagatgaaaaggaatttttctatcta
atagaacaatggaactctcgcgcggttgtggttgtacctgtactgcaggaataggaaaactcgctattcactcagtttttttccataataagattatggaggagagatg
gccgagcggttcaaggcgtagcattggaactgctatgtagacttttgtttaccgagggttcgaatccctctctttccgtttttcttaattcagcaacgttaatgattacaatg
tatcaaatcaaatgacaatttattccagcaataatacaataccttatttaatagaaattctctataccaaattactatggtatgtaaaatacacatagaggaaagaacaaa
aaacaaaaaggaatcctagggttaatccatttctgttaggtgaatgggaaaatacgaattaagagccttaggtcgatttagttcggggaaaggggaaggggaagaa
aattctatgaacctttccgtttttccttaagttcaagtttgacgagagtaatattctacaactaacaactcatttattttgagaccgacccacttcctatccaggattttttttact
agtcctttatattgcaatgtgtcaatcgtcaaatgctttggcaatttccccgggtcggatgaagcaatagaattttgaaccagacgttttgatctttggttatccttcgtagt
aataatatctcggggtttgcaacgaaaacttggtatatcgactatacgaccattaactaaaatatgtctatggttaactaattgccgggcctcgggaatggttgaagcca
tacctaatcgaaaaaggatattatccaaacgcatttcaagtaattgtagtaaaacctgacctgttgaactttttgcttttccagcgatatgtacatatctaagtaattgtcgtt
ctgtcagaccataatgaaaacgcaatttctgtttttcttgaagacgaatacgatattgttctttttttcccagaatggaatttctttttcagattacttccggatttaggtgtttttc
tagtgagtcctggtaaagctcccagacggcgtattttttttaaaacgaggtcctcgataacgggacatgaagactcctttttttattgaaatttcattttacacaattaatttca
ttgtatttacattacagaatacatcgaaattaaaactgaattaaagtaaaggataaacagagtaaaatctactaaaagtaccacaaaaaaatggaatttcatcaacatct
gaatttttgtatatatattatttattttattgttttgtatctagcaaaattgtagggtagaacgacataatagatcctggcttctccatttaattcggagaaaaagaggtatttttg
ttcatggaacattgatagagaaaaaagccgactatcggatttgaaccgatgaccctcgcattacaaatgcgatgctctaacctctgagctaagtgggcttacataaca
gaaatagtgtaacaaatagaaatatgtatagtataggaaatccgtaaaatgtcagatcttaattattaatcttagctattaactagttcgaaattggaagttctacttagaaa
aaaatactagaacttcataaaataaagttagatagatttttttgaactttctttttttctctaattcgcaaatctatttttctaatagaatctattccaatttctatattgaatttgatttt
agatatttttaatttgatatggctcggacgaataatctaatacatggaaaagaataaataatatatatacgaaaacataataaagagaacatgcgaatttcttgtattttca
gtccatcattatagacatttttgagatattttgtttttttttgttatttgctaataatttaatgattaatatttcactaaggagaacatagaatcatagcaaatgaaattgctaattct
gattagcaaaaaaaaaagaatgaatatcaagcgttatagtatgattttgaatactctaaaaaaggaacgcggtaggtgggggagagaaaaaccttgggatatattgatt
cgcattgaattgcaaatacatcaacgatagaatcaattcaatgctgaattgcaataagcggagtctctcaactagagacgaaccgctagactacatagagtaatgaat
tcaacgattcaaaaaaaactaacagatggaggaaattgcacaaggaatcctggtctcaaagaaaaagaaaatggggatatggcgaaatcggtagacgctacgga
cttgattgtattgagccttggtatggaaacctgctaagtggcaacttccaaattcagagaaaccctggaattaaaaaaggggcaatcctgagccaaatccatgttttga
gaaaacaagcggttctcgaactagaacccaaaggaaaaggataggtgcagagactcaatggaagctgttctaacgaatcgagttaattacgttgtgttgctagcgg
aactcccttctaaattagggaaagaagggctttcgaaatctaatacacacgtatagatactggcatagcaaacgattaatcacagaactcatatcataatataggttcttt
aattcttttttaaaatgaaaataggaatgattatgaaatagaaaattcataattttttttagaattgttgtgaatccattccaatcgaatattgagtaatcaaatccttcaattcat
agttttcgaaatctttttaaaagcggattaatcggacgaggataaagagagagtcccattctacatgtcaatactgacaacaatgaaatttctagtaaaaggaaaatcc
gtcgactttctaagtcgtgagggttcaagtccctctatccccaaaccctcttttattccctaactctagtatttatcctgttttttttattaataggtttaagattcaatggaatac
atttctttttttattatagtatcggcaaggaatgtcgattattaactcgatatttaaatattattaaataggctttctttgtacaatgcataggactgccccctccccatatccaaa
ttttggatattgacatagatacaaatactctactaggatgatgcacaagaaaaggtcaggatagctcagttggtagagcagaggactgaaaatcctcgtgtcaccagt
tcaaatctggttcctggcacagaaaaaagggatcttccgaataggtattgatacaaatacctcgagatgggttgggatacatattcgttaataatatagatagagtatga
ttttttcatctaagtagataaatctctaaatagaggcacttctttttctgcatttttgcatttcttaattttctattccgctattccgacaaatattttttttattcttgcttatcttatctta
ctttcctagttgttctaagtaatgcacgcggtacaaagttcgtggtagggaacttctttgagtcatcatatttttctgttcatacgaaggaaatgaatatgtgattttccaac
gaaatgaagccctttttttgcttagtctatctgaaccttttgtataattggaattaattagaatgtaataggtattctgtttcatctaggaacagagcgtaaaaatattccttgac
ttgcataaaatctggagttgtgttgtataagtgagcatgaatttcttatcattcaatgagcatcttgtatttcatagaaattgggggttatatagtccttacgtaagggccag
cctatccaactttcaggcattaggatacgtttaaggcgtggatgattatcataagagattcccaccatatcaaaagattcgcgttcttgaaaatcggaacttctccaaatc
cagaagacagatgggattctaggattatccttttgggcaaagacttttatgcatacttcttctgggttatctataccatactgtattctcgtaagatgatacacgctagctaa
agatccaccgggtgcaacgtcataagcacattgggaacgtaaataattgtaaccatatacatataaaatgacagcaatggaatcccaatcctctgcttttatttgcaaa
gtctctattcctcgatgatcgaagcccaaagatctatgaaccacctcatgtttgactagccaattagataaccaaccctgctgcattatcttgatctctcctcctttgtataa
atatttcgcagttcgaatgcaagtttgaaatattgccctgctctttctttttcggcacaaagagctcctcctaattcactaatttgtaggaagatactggacttttggatttga
aaaaagtttcagaagatatgtctaaagtagatggtgattgatagagcaattcttgctcataagttccagtattagtactgcgccgaacataaagcttgtggctggtagta
aaacatcgatttttcttttgagatagagttcgatcctcaactatttctcgcgatatcttcttacgaagttttgttagggcatctataacagcctctggtttaggtgggcaaccc

ggcaagtagacgtccacaggaattaacttatcaactccccgaacagtactataggaatccgtactgaacattccaccagtaatagtacaagctcccatagcaatgac
gtattttggttcaggcatttgctcatataatctcactaaagagggagccattttcattgttactgtaccggctgttaaaattaggtccgcttgcctaggacttgatcttggta
ccaatccataacgatcaaagtcgaatcgtgagcctattaatgaagcaaattcaatgaaacaacaactggtaccatatagaaggggccataaactagagagtcttgac
caattcgaaagatcttttagtgtagttgaaataacggaattggaacttgtttggtcaagtaggggaaactcaatcaaactcataactgtcttaatggaatcttttccttctttt
tttttttgtctgaatattcagttaagaccattccaaggctccttttcgccatgcataaactaaaccaacaactaggataagcacgaaaatgaaagcttcgataaaaacgg
atatacccaatacgtcgaaactcattgcccaagggtagagaaagaccgtttccacatcaaaaacaacaaaaactagcgcaaacatgtaatagcgtattcggaattgt
aaccaagccccccccatgggttctatacccgattcataactagaaagcttctctggtccttcacgaaccggagctaaaagtgctgaaatccaaaatgctaaaatagg
aataaggcttgctattattagaaatgcccaaaaaatatcatattcgtgaagcagaaacataaatgtactcccattaatgtggaataggcggaactgaattagtcaattca
agtcagcattgtcaatttatacagaatttctctcttttcctcggtgaaacaaggatcggtttttctcaaaccaaagggcttagtttagcctttgtttcctctttgccacgtcttct
ttaaagattcatccaatggaatcccgactccctttctttttgatttcctttctatttaggtatggtggagacataattcttatagaaacaaaactctctcgcttcactttgtctca
ttttctctagaatctctagaaaaaggaataaaaacgaaaatactacgaattagagcctaattaagataggatgactaatgtatgcagcctaatgaggagtaattctataa
aaataaagaactctatttcagaacgtagatcgatttagatttaggtaatctatagatatagataagcaaagtaatatacttcaaacaaagtaggaattcgcaagatggag
aacatcttgcagttgatttgatagaaattcatttttcttttcctgtctctataattttcgatgaatgagcctctggtaatccttttatctctattttatggcgcaggcgcctgtcca
gtctataaacaagtactaatagggaaatgaaaactatactaaaggaaacgtaggatatctctcctaaaatctaaaaaaggacaggacatattagggctatacggattc
gaaccgtagaccttctcggtaaaacagatcaaacggattattatcgaaatgattcgaactgtttcaaagacccaacatgcattttttttgcattgggctcttttatcaactga
tagaaagatcagttagtccaccatagtttttctttacggaaagataatgagatggctccctgcgctctgattgattatttgtattatgatctatctaagagcaataccaaagt
gtttcaaaggaggattaccttgacttaggtctgcctccggcctaaattaaatcaacctaagtgaaatagagtctctatcgttccgctacaagagttgactatgagacttc
atacaccttaaagttcatagaacgaaaagaattttttttggaggcccttatcctcattaagcctagcatttagtgggctggatatttaccttatcaactagcaaatcaataaa
ggttctatttgtttaggcacctggattggtacctgaatcggactgaaccaactatttgtcaggcgactgttctcctattctctcgaatccatgaagtaagacattgattttgc
aagaagatccactatgttcattgcataataagctcctttgaaaagcattggcgcacgtgtaaacgagttgctctaccgaactgagctatagcccttgtcagagatatctt
aatatatagagaatttcttgtcaagatgaatattctctaatagtagaggatatcctttgatctgtttactataataacataccaataacgaagcggtatttgcttataaaaag
gattcgatctataatcgatcgaagtaaagggtcttcctttgtggtgataaattgcctacttaactcagtggttagagtattgctttcatacggcgggagtcattggttcaaa
tccaatagtaggtaggtaggtagaaaaattactagatagcattggccctacttcgcttcgctatctaataatttttctacccctcttcccttttttcttgtatcaactaaaccg
ttgggttgtcttcaattagatgggggaatccaattaacagcctcgactcgtatcctagctcgtctgagagctaccttcgcttcaaccaattctttcgtaccctcagctctac
tcacgttagcttcggctatttcaagtgcctgttgagcttcttccggatcaatgtcactacccagttccgcatcatttcctaaaatgatgatctcattattaactattctggcaa
aaccgctccacagaaccgccgttaaccattgatcgttgaggaggcgtattctcaagggacccatatctacagctgtgttaatgggggcgtggtttggtaatacgcca
atttggccactattagtagataaaatgatttctttcacttcacaatcccaaataattcgcttaggagtcagtacataaagatttaatttcatttcttcaatttgttctcctcttcta
agtttatagctttcgtgctagcttcatcgatgttacccaccaaataaaaagcctgttcgggtaggccgtctaattctccggaaaggattagttgaaatcccctaatagttt
ctgcaagaccaacatactttcccggagaaccggtaaaaacttctgccacaaaaaacggttgtgataagaagcgctcaatttttcgtgctcttgctacagttaaacgatc
ctcctccgataattcatccaacccaagaattgcgataatgtcctgaagttctttgtaacgttgtaaagtttgcttaactctttgcgcagtttcataatgttcgttgccaacgat
ccgaggttgtaacatagttgaggttgaatctaaaggatctactgcaggataaatccctttggaagctaatcctctggaaagtacggtagtagcatccaaatgtgcaaat
gttgtagcaggagcagggtcggtcaaatcgtccgcaggtacataaaccgcttggatcgaagttatagatccctttttagtagaagtaattctttcttgcaaagaaccca
tttctgtactaagagtaggttgataacccactgcagagggcattctccctaataaggcagatacctccgatcctgcttgaacaaaacgaaagatattatcgatgaatag
aagcacgtcttgcttattaacatctcggaaatattctgccatagttagggcagtcaaaccaactctcatacgagctcctggcggttcattcatttggccatagactagag
ctacctttgattcctcaagattttttcattaattactccagattccttcatttccatataaagatcatttccttcacgagtccgttcccctactccgccaaatacggatacgcc
cccgtgagctttagcaatattgttgattaattccatgatgagtactgttttacctactccagctcccccaaatagtccgatttttcctccacgccgataaggagctaaaag
atcgaccaccttaataccagtttcaaagatggataatttcgtatctaactcgataaaggcgggcgcggatctatgaatagggaatgttgcactagtatctacaggaccc
aaattgtcaacaggctccccaagaacgttgaaaattcgtccaagagtagctccaccgacaggaacactgagaggagctcccgtgtcaatcacttccattcctctcat
caacccatctgtagcactcatagctacagctctaactcgattatttcctaataattgttgtacctcacaagttacattaatttgcttaccgtcagtgtctcgactcttgactac
caaagcattataaatataaggtaacttgcccgggggaaaagtgacatccagcacgggtccaataatttgatcgatacgccctgtactttttttcttcaattgtagaaaccc
cgggacgagaagtagtaggattggttctcataattatcacataattttcaaaaaaaaggaatttatcgaaattttgattttttttcttgttgaataatgccaaatcaacaccaa
aaaaatatccaaaaatccaaaagtcaaaaggaaatgaattagttaattcaataagagagaaaaggggaccagcacttgatttcgttgcccaaacgaatcccattcaat
cgtttactcatggaatgagtccgtcggaaagttcaatcaatctttttttcatatacattttgccttttgtaaacgatttgtgcctactctactttcttatctaggacttcgatatac
aaaatatatactactgtgaagcatagattgctgtcaacagagaattttcgtagtatttaggtatttccactcaaaataagaaaagggggtctattaagaacttaataagga
ttagaagttgatttggggttgcgctatatctattaaagagtatacaataaagatggatttggtgaatcaaatccatggtttaataacgaagcatgttaacttaccataacaa
caactcaattcttatcgaattcctatagtagaattcctatagcatagaatgtacacagggtgtacccattatatatgaatgaaacatattatatgaatgaaacatattcatta
acttaagcatgccccccattttctttaatgagttgatattaattgaatatcttttttttaagatttttgcaaaggtttcatttacgcctaatccatatcgagtagaccctgtcgttg
tgagaattcttaattcatgagttgtagggagggacgtatgtcaccacaaacagaaactaaagcaagtgttggatttaaagctggtgttaaggattataaattgacttact
acaccccggagtacgaaaccaaggacactgatatcttggcagcattccgagtaactcctcagccgggggttccgcccgaagaagcaggggctgcagtagctgc
cgaatcttctactggtacatggacaactgtttggactgatggacttaccagtcttgatcgttacaaaggccgatgctatcacatcgagcccgttgttggggaggataat
caatatatcgcttatgtagcttatccattagacctatttgaagagggttctgttactaacatgtttacttccattgtgggtaacgtatttggtttcaaagccctacgcgctcta
cgtctggaggatctgcgaattcccccctacttattcaaaaactttccaaggtccgcctcatggtatccaagttgaaagggataagttgaacaaatacggtcgtcctttatt
gggatgtactattaaaccaaaattgggattatctgcaaaaaattatggtagagcatgttatgagtgtctacgcggtggacttgattttaccaaagatgatgaaaacgtaa
actcacaaccatttatgcgttggagggaccgttttgtcttttgtgccgaagctatttataaatcacaggccgaaaccggtgaaattaaggggcattacttgaatgcgact
gcaggtacatgcgaagaaatgattaaaagagctgtatttgcgagggaattaggggttcctattgtaatgcatgactacttaaccgggggattcaccgcaaatactagt
ttggctcattattgccgcgacaacggcctacttcttcacattcaccgagcaatgcatgcagttattgatagacagaaaaatcatggtatgcatttccgtgtattagctaaa

gcattgcgtatgtctgggggagatcatatccacgctggtacagtagtaggtaagttagaaggggaacgcgaaatgactttaggttttgttgatttattgcgcgatgattt
tattgaaaaagatcgtgctcgcggtatctttttcactcaggactgggtatccatgccaggtgttataccggtggcttcagggggtattcatgtttggcatatgccagctct
gaccgaaatctttggagatgattctgtattgcaatttggtggaggaactttaggacatccttggggtaatgcgcctggtgcagcagctaatcgggtggctttagaagc
ctgtgtacaagctcgtaacgaagggcgcgatcttgctcgtgaaggtaatgaaattatccgatcagcttgcaaatggagtcctgaactagccgcagcttgtgaaatatg
gaaagcgatcaaattcgagttcgagccggtagataaactagatagctagactaagtggataaaattagatagaaaaaaggtctaaataaaaaagaagagaaataga
aagatcaaaaatcagttacgaaaatgcagtaattcttctttttttcttctaattgattgcaattaaactcggctcaatctttttttcagattgagccgagtttaaatagattttgata
cgatcatgagacttgacaaatcgggattcctctattctatatatttagaagatataaaggtataatacaataaataaatacaaatatagtattatcatatgataatggaatca
aatacgcagtatttacagaaaaagttttcatttattgggaaagaatcaatgacatacaatgcattacagacgtatgatcattaccctttaaccgggttattctattccacttc
tagatagagaaaaaaactaaaggagaatgaatgaaaaaagacagagtttggaagttagacccctttctaagactctctttcaaaaaagaggacattttgaaacttttaa
caggcacaatcgtgagtcaacaagtgactcgaaatgctcgtaagaaaagagaattgattttcaaaatggtagaactagatgacgaagttttctataaccttgatgaag
aggtagttttagtttacgactccgatcaagagcgagaagtctttcatttcagattggactgctatagaatctggacccatcgtagagacgttcaaaaggctcctgatgat
aagaatcatactttcgcggaactccatggggctatgggcttcaacgcggtagacgttttgttccgaatttttctggagcaaacctccgacccaacgatacaatgaatttt
ttctattcacaaagaaaaaagattcggaatcgcaatgctactatacgcacccggaggaatattcggaattatattcttatataatccatttttgcgcggggtcttactaac
aggacttcgctgcacgagacgaggattcgtcgaaaagctaaggtcatccggtattttcatacccttgggggtggtatatggccttacaaagtctaaggggtagtatg
agatctgtagtaggtaaaagaatttgtcccttgattgagtatgctattttccctcctttaccgcgcattattgtatatgcttctagaagagcacgtatgcagagaggaaatt
acagtttaataaaaaagcctaaaaaagtttcaactttacggcaatatcaatcaactaaaagtcctatgtatcaatccttacagcggatttgtggggtccgagagtggttg
aataagtattgcatgtggaaggaagtagacgaaaaagattttggattcgaaataggcgcattcgactaagtcgtactttgaatccaatttcaagttcgattagaaggat
agaaaggccgcgaggatcggaaaagaaaaatcaaatctttttaattgcttctccttttttgctattttcttattatttatataatccattcgattcttttttttagaatactaaagt
attctaaaaaaaagtattctataaaaaaatcttttttgcaaactcaaaaatacaatagtcaatattcgttataatagatatacttaattatatcataagaatcttaagatattttt
gaatagatcaaatcgaatagatagaaatagtaaatttgaatggagacacctattctatgatggattttaacttaccctctattttcgtgcctttagtaggcttagtatttccg
gcaattgcaatggcttctttatttctttatgtgcagaaaaataagattgtctagaaccgacgggaccgaattttctcaatgtatttccacacaggatcataatacggatcttt
tgtagtgtaagtaatataatatggtatgttatgtggctctttctacacacaaatgcaaacccgctatggatggggattatggatgcggatataggctacgagcataaatg
catgcatatgcggaaccgggtatagcgagttttttaagtggatcaacaaatactttttgaatagaaagtcaatgtatctaaccaattattttacaggagtatctagttggc
gaaggcgatttcagaatcaaaaaaagtaaagtcaaaatcatttagcttattctctcaatttcaatcgaccgctgttagtatatctaatatgaattggcgatcagaacacat
atggatagaacttctaaaaggttctcgaaaaagaggtaatttttctgggcctgtattctttttctaggttcactaggattcttagcggttggggcttccagttatcttggtaa
gaatattatatctgtacttccatctcaacaaattctttttttccacagggggtcgtgatgtctttctacggaatcgcaggcctattcattagcgcttacttgtggtgcactatt
ttgtggaatgtaggcagtggttatgaccgattcgatagaaaagagggagtagtgtgcatttttcgttggggattccctggaataaaacgtcgcgtcttccttcgattcctt
atgcgggatatccaatcaattagaattcaggttaaagaaggtctttttcctcgtcgtatcctttatatggaaatccggggccagggggccattcccttgactcgtactgat
gagaagtttttactccacgagaaattgaacaaaaagctgccgaattggcttatttcttgcgcataccaatggaagtattttgagtaccaattgaatttttgaaatgaattg
aatgaagaagaattggaagaagaaaagttttctcaacacgagggaaaaggtcccttcgaaattgcattattgtaaggggattttgagtatttatctaaaggaaggaac
aaatgaggataagagaaaattgcttctaatttgtccaagtgggatatatggcgtaatattcttccattttcatccgaaaggactttttctatttctctattccactccatctag
atctaagaaagaactcaatgcaatgaaattctactagtatagaaaaaagaggaatagatacaaggtctcaaaccttgttatagaatttttccttcaaagaaaaagaaata
tcatatagagtcagcgaataaagcggattcattaacaactcaatttacagatcaaaaatgaaaaaaaagaaagcattgccttctttcctatatcttgtatttatcgtactttt
gccttggggagtctctttctcttttaacaaatgtctggaactttggattaagaattggtggaataccaggcaatcccaaactctcttaactgctattcaagagaaaagagt
tctagaaagattcatggaattagaagacctttttatcttggacgaaatgataaaagagaaaccgaatacacatgtacaaaaccccccctataggaatacgcaaggaaat
aatacaattggccaaaatagataatgagggtcatctccatatcattttgcatttctcgacaaatataatctgtttggctattctaagtggttcttttttctgggtaaagagga
acttgttattttgaattcttgggttcaggaattcttctataacttaaatgactcagtaaaagctttttttattcttttagttactgatttttttgttggatttcactccacccgcggttg
ggaactactaattcgttgggtctataacgatcttggatgggttcctaacgagctaattttcactatttttgtttgtagttttcctgtgattctagatacatgtttgaaattttgggt
cttttttgtttaaaccgcctatctccttcgcttgtagtcatttatcattcaattagtgaagcataaactcatttgatctcctgatattaatcaaaattagcatcctttttcctttaga
aagaaagcctttttccattttagcaaaattctttttttctatttctacctgctcaaggtattcatcattccagtacaactgttgcagtagaatgacaacagattcgtgtataggg
aactagattagcttagctacctatctaatttattgtagaaattccgggatctgtgattggacatggaaaatagaaatactttttcttgggtaaaggaacagatgactcgatc
gatttctgtatcgatcatgatatacgtaataactcggacatctatttcaaatgcatatcccatttttgcgcagcaaggttatgaaaacccacgagaagcaactggacgaa
ttgtatgtgccaattgccatttagcgaataagcctgtggatattgaagttccccaagcagtgcttcccgatactgtatttgaagcagttcttcgaattccttatgatatgca
actgaaacaagttcttgctaatgggaaaaagggagggttgaatgtgggtgctgttcttattttgcccgagggattcgaattagcgccgcccgaccgtatttctcctgag
ttgaaagaaaagataggaaatctctcttttcagagttatcgtcccaataaaaaaaatattcttgtgataggccctgttcccggtaagaaatatagtgaaatcgtctttccc
attctttcccccgaccctgctatgaagaaagacgttcatttcttaaaatatcccatatatgtggggggaaaccgaggaaggggacagatctatcctgatggtagcaag
agtaacaatacggtctataatgcaacgtcaacaggtgtagtaagaaaaatactgcgtaaagaaaagggggggatatgaaatatccatagtcgatgcatcggatggac
gccaagtgattgatcttatacctcccggtccagaacttcttgtttcagagggggaatcgatcaagcttgatcaaccattaacaagtaatcctaatgtgggagggtttggt
cagggggatgcagaaatagtgcttcaggatccattacgcgttcaaggccttttgttcttcttcgcatccgttattttggcacaagttttttggttctcaaaaagaaacagtt
tgaaaaggttcaattgtacgaaatgaatttctaggtcccgggatttcttaccatcaagttggtaaaaagccgcgatttattggcgattgctagaattctctatgatccatttt
ggaaatcttttttttgtttagactatttttgtttgcgagatgtcttgtctgcaattccttatttctatcatctcatgcaaaacgaggagaatgcaaggcaagggcgagaaca
ataaaaggaacaaatccttttaggagggattgctggtcttcttaatcctctattgggcacaagaaaaaggctttttttgccttttcttgtgtcgattcttcttgtatcgaaatat
gaatctttttttcttcctattcggcaaagattactatttcttctttatttgggtctgtctcttgaacctcttttgcttaggttcaggcgatgtagtggacaaaaagagggaaagaa
aatatggcgggggacaaatttttttgtgaccaaatagaattgcttgacttgttcaattaagttcaatttagaacttacagaaattttgcaaaaaaaatggatactcttccattg
aaggctgttttttatttttaggctagttgagtagttttaattaaggttttattagtttattactctaaattaaatcaatgatttacaagagacttcctccgggtaataaaatattgg
atcctcgattgatcctttctttctcctcgcttcataaaagtgaattaatttcattggcgagggggttataaatcaactgatggattacttcactaacattattaacaaacaaaa

ttaacaaacaaaacgaataaatagagggattctgaccatcagatcaaaggctttctctttgttatttttacaaatcaaaataggaaacccgtttgtaggttatggaataga
ttaaaaaagtcgggttataagagtaagaattccgcgggtcctttccgctctaatcagataaaagggggtaaggacccgctaagttcctattttttcatgtttacaacctg
gtccctccaattactatagagatgaacccaatccagaatatgaaccgtaaaagaaaacacctattaaaccaatcacagcaataccggttacagtacctatcagccaa
agaggaattcttccagtagtatcggccatttcccctactttcctccacattttatcaagtggtcatgctagagacaaaaacagtcatggatagttataaggatggtatcctt
ccaaatggggataagagaattcttactactctctttctttctctcaattgaagaagtaattggaaaataaaacagcaagtacaaaaatgagtaataaaccccagtataga
ctggtacgattcaattcaacattttgttcattcgggtttgattgtgtcatagttctatagttggaatttggtttatcgttggatgaactgcattgctgatattgatcccaagaaa
aaaacagtaggtacagctagtccgtgaacagccagccatcgcactgtaaaaataggataggttcgatctatggtcattgagggcctcctaaaaggatctactaaatt
catcgagttgttctaaagaatcaaaacggtcggttattaacggaattccttgtcggctttctgtgaaatactcgtttggccgaggacttccaaacacgtcataagctaaa
cccgtactgacaaataaccaacccgcaatgaatagggaaggtatagtaatgctatgaataacccagtatcgaatactggtaataatatcagcaaaagaacgttctcc
cgtgcttccagacatgctgagctccccaaatttttgtacattcaaaaaaggaattgattccgtaaaagatgggatcaaccagtaaatagaaaattactgatatttcatcct
tgtgagattgtcaattttgtaccaaaggtgtattttgagtataccaaattagtatagctatccttcctatggcacagcaatcctgtttcgcttggtctcgaaacagaattctttt
tttctcttctttgttccttgtctatagggtaagctatatggtattcaaggcatcaatagaaaacctcaattttgagggtcctacttaattttcaccggcttcggaatagtagaat
aattcggaatagggctcaagatcttgggaaaatctaagttaatgatcaacaggtttggataaagaatttaggaaagatattcttatactgacacaatacaaagagaagt
agatgcgaaagctatcccttcgaatccaacctttccccttaaagaatttaattggttagcataatataatatctaataaatagaaaatcaaatagtagataatctgttatga
aagagagaaaacattctttgaagaatcaagattcgtaatcaacccttgccttgtttactaactttcttgaccaaactgcaagcgtggaactttgccagttcttaggtttccg
aaatagtgtaatggaaaaagaagtgcttcgaatcgactttgattggggttcgaaaaaggaattaaaaaaaaggaaattcaggaaagatttcctttttttaggagaccct
cgaggggtcgtggaatgcttttcttctcctcttattccatatggaatacaatcaattaaaataagaaggaataggggaatattcgactgttcgctccaaaaagaaggtta
aatcatcctattgaaaaagaccaaaatagaaagaactttttcaaattcaattctttatttttcactggggttagctgatctagttcttaatattattactttactcaattgacaga
ttacacagcaaatctcttgattcggaattagggactcatgccccatctgatgaatccattttcttttacacttctgtatctcactctatcttgtttttttagtattatctaaaataac
cgatgaattatgaattttccataacttaggtaagtgctttaccaacatatgtagtgtagtaaaaaaaatggaatttaaccctttcatgcttactataactagttatttcggtttt
ctattggctgctttaactctaaccctagctctatttattggcttgaacaagatacgtcttatttgaaatgaattgaatgaataaggcagaaaagaaaaaatctctcttttgga
ttcctggtattctacgcactaattaccaattctttttttcttggtcattgagattcgtggataatttagactactatttagggataaatcgtacctctttttttttatcccctcgaaca
aatcgaaatgattgaagtttttctatttggaatcgtcttaggcctaattcctattactttagcgggattattcgtgactgcgtatttgcaatacagacgtggggatcagttgg
atctttgattgagtaatatttcttttttgattgccctcctctctggtctggaggaggtcaaattggaattgcaatttgttttgttaagttattttgccttgcttcgacataagatag
atggaatcacgctctgtaggatttgaacctacgacatcgggttttggagacccgcgttctaccgaactgaactaagagcgctttcaaaaatcaaatccttttctactcct
aatgtgtctcacgtacgtatagtatccacaaattaaagttatacccgctttaatcgacctcctcactactgcctataaagaagaaagaagtaataggtagggatgacag
gatttgaacctgtgacattttgtacccaaaacaaacgcgctaccaagctgcgctacatcccttttccaaattgttgtacaatgtcattgtacacaattcctatcttgttttcc
acatcctaatttcttgtctttttctatctatagagaatcctcgtgtcatttcttctttttggtctcatataatcaaggaatggtatatatctaaatccaatctaatttcacctataaa
agaaagattactattccttggtaatctataggaagggattgtctttttcgtttttagttcggaatttcgcctaaacaaaagaaatacaaatgatcttgggcaataatatctga
tcatatacgtattccaataaggaaggaggattttcaatgcgggatataaaaacatatctctctgtagcacccgtgctaagtactctatggtttggggctttagcaggttta
ttgatagaaattaatcgtttatttccagatgctttgtcattccccttttttttcattctagttgttgctatgtgaggaatagagttcttcgtgacatgataaaaaattcccaccctttt
tagtataggaaagaaaaagaaagaaaagatagattgggttggaccttagagtcatgaaaaatttggtaaatctcatttttgaaaaaagaaattcaattaaaagcagtat
ccaagctaagtcaggcctcagaaatcagagcatagaaagaggctgggttggctactaaaatgaaaggattttgcttcaaaatccttgctaatttgacaaggattgtatt
ccttaattatttctctatttttttattacttaattgaaaaatttccaaaaatttttattctaatggattttcttcttcctcttcggtttcaaaatagaggaataaaagaataagtagaag
aattaagttaagtcaatccaaaaaggaaaggaggttcatggccaaggggaaagatgttagaatcagagttattttgcaatgtgtgagttgtgttcgaaaaggggcca
atgaggagtcggcagggatttctagatatagtactcaaaagaatcgccacaatacacccggacaattagaattaagaaaattttgtcgttattgtcgtaagcatacgat
tcatgccgaaataaagaaataggagcatcttgtgttcgatctttccaaagatcaaaaagaataagaacttcctatttaatattcctatttaatatatagagcatagatagaa
tacaaaatacaaatcaacttgtctgatttccattagatattatttcatatgtatcgagggtattcatctaatatatggaccaaagagagactacttcttctggatccaaaatta
ataaaataaacaaatcaatttttttttcaattttaaaataaggaataaatcatgtatacatctaaacaacccctttcataaatccaaacaaacttttcataaatccaagcaaactt
ttcgtaaatccaagcaaacttttcgtaaattcaaacaaccttttcgcaaacccaaacaaccttttcgtaggcgtcctcggattggcccgggggatcgaattgattataga
aacatgagtttaattaatcgatttattagtgaacaaggaaaaatattatcgagacgaataaatagattaaccttgaaacaacaacgattaattactcttgctataaaacag
gctcgtattttatctttcttaccatttcgtaactatgagaatgagaaacaatttcaagcccagtcaatttcaataattacaggtcctagacccagaaaaaatagacatattc
ctccattaacacaaaagttcaattccaatcgaaacttaagaaactccaaccagactttaagaaacaacaatcggaacttaagttccgattgttgatgttttattcgaaagg
gccagactatatataaagaaagtaatccaatttagattcttgggtttgttataagaaagaacaatggggaagaaaaaatagttttttttatttattgcaacatgctcgttgatt
cctaccacttaatcttaatttattgtatcttcccggagttacctctccgggaattctttttttaattattcctgtatattactttttttatccctttaattgataatctttattttattggaaa
tcgtgtaaagattatttggatttaatacagctacttgtgcaaggattttacgattaagaatcaattctttcttgtaaagattgtgtattaatttactataattatcgaatactttatg
tatccgcgttgctgcgtttatccgactgatccacaaacgccgaaaatccctcttttgcctgactctatctcgatgagaggaaacaaacgctcttcttacctgttgagtaat
cattcgattaagtcttaaatgagcccctctaaagtttgaggcaaatgaacgcatttttgcccgtcgtctccgagctatatatcctcgcggaactctggtcattgaatcaaa
ttaaccttaatgaataactaatgatttctcttcttttagccatcctttttcccattaataacaaaacgaattattcccatatataaaatattagttccaatggcttttgctactataa
ccttcccaaccacaattttctcttctactcccttcagttatttcgcatagaaataaaaaaattctaacgatactaaaaaatagtgggttccatcgtttctatggttcccttttaaa
cggcgaggccctctctatacaccggagccctttctttcatttcatcaaagggtattgtgaacttgtatagttcacattctttggctctacctatccattatagagtaaatagc
tcttttcacaataagagttatccatacagtgacggcatttaattatgaaagttggctaagtagctgaccctcttagtccgtttttttaagataaaggagcataagcctttatct
ttttattactatttcctccgcttaatgaatagccgtttgctaccaatgggggaattgcttcttatttccaatctagatgattggatttgcaccaaaggaaaccagaaattccat
ataccgtagaaatctaggatagagaagctctatcctattcattggtaccgatcatggatacttcaaaaattgtcttatttgtttgaacccatgatccgaacgagtcgcaca
tacaccctagcacatgttcctcgacgctgaggacatcccttaagcgcggccgattttctagcatttcgtattggctgtcttgcgtttctaataagttgtttaaccgttggca
tgtcgtatgtatatagaaaaaatggattggtttagatcgatcttaacctgaatgattgatcattatgaagtatttccattaaattgcataaaacccgaatttagggttgaaat

aaatttacaagaaatctggccactaccaatccttaaacatttctggaaaccacactggatcagtatcgcagtgctcgtcaagcatttcatcccctacaatatcgacaagt
ccataagctttggcttcgtctgctgacataaaaacatccctttccatgtcttcggatacaacccaaaaaggcttgcctgttcttagtgcataaacccttgtgatcatttcgc
gaactttgtgtaactcttccacttctagtaaaaattctggtgttcttgcccgataataagcactagcaggttggtgaagcataatcctcgcgtgggggaatgctatacgct
tggtgggttctcctccaagcagaatgaaggacgccatggacgcggctattccgaggcatattgtatatatatctggtgtcaccgtttgcatcgtatcaaaaatcgccat
tcctgagattaaccacccgccgggggagtttataaacaaaaaaaatatcgctaattccatcttctatactgagatataccatgagacctgtaatatgattcgtgacctcgc
aacgaatctcttgacctaaaaaaagtgtcctttctcgatacataacattgtataagtcaacccaagtcgcttcttcatctccgggaatccggtaaggtacttttggaacac
caatgggcatattagattaatattattaaatttaagtaagaaaactacactttaatatggaaacgtaagaatggaagagaaagaaagaatccgcagtttattgttttcactt
tttttcttattctatatgaatactatagattctattaatacgtagattgaaataatacatagattgaaagattatacatataagtaggataagacagattgaataaagaaaaaa
gaatgggtgattcgaatactaaacaaaaagagatagggatctattcttcgtttttccaaataagccaagctgcccattgcatattggcacttatcgagtatagaatagat
ctgcttctctttcttcttacgaacagaattggcttcttatttttaatggaatgaaataaatattcacgctttctgacacagaatcccctagaagggttaggtacataggatatg
gatagtctttgccaatgcgataaaataaagtgacatcgtgtctatttttctttgctaaaggggtatttccatgggtttgccttggtatcgtgttcatactgtcgtattgaatgat
ccgggtcgattgctttcggtgcatataatgcacacagctctagtttctggttgggctggctcgatggctttatacgaattagcggtttttgatccctctgatcctgttctgg
atccaatgtggagacaaggtatgttcgtcattccctttcatgactcgtttaggaataaccaattcgtggggtggttggagtatttcaggaggaactgtaacgaatccgggt
atttggagttatgaaggcgtggcaggggcgcatattgtgttttctggcttgtgtttcttggcagctatctggcattgggtatattgggacctcgaaatattctgtgatgagc
ggacgggaaaacccttctttggatttgcccaagatctttggaattcatttatttcttgcagggggtggcttgctttggctttggcgcatttcatgtaacgggtttgtatggtcct
gggatatgggtgtctgatccttatggactaactggaaaagtacaagctgtaaatccggcgtggggggtgcagaaggttttgatccttttgttccggggggaatagcttctc
atcatattgctgcgggtacattgggcatattagcgggcctattccatcttagtgtccgtccacctcaacgtctatacaaaggattacgtatgggcaatattgaaactgta
ctttccagtagtatcgctgctgttttttttgcagctttcgtagttgccggaactatgtggtatgggtcagcaactaccccaattgaattgtttgggcctactcgttatcagtg
ggatcagggatactttcagcaagaaatatatcgaagagttagtgatgggttagccgaaaatctcagtttatcagaagcttggtctaaaattcccgaaaaattagcctttt
atgattatattggtaataatccggcaaagggggggattattcagagcaggctcaatggacaatggggatggaatagctgttggatggttaggacatcctatctttagag
ataaagaagggcgcgaactttttgtacgccgtatgcctactttttttgaaacatttccggttgttttggtagatgaagagggaattgtgagagcggacgttccttttagaa
gagcagaatccaaatatagtgttgaacaagtaggcgtaacggtggagttctatggtggcgaacttaatggagtaagttattctgatcctgctactgtaaaaaaatatgc
gaggcgttcccaattaggggaaatttttgaattagatcgggctactttgaaatcagatggtgtttttcgcagcagtccaaggggttggttcacttttggtcatgctaccttt
gctttgctcttctttttcggacacatttggcatggggctagaaccttgttccgagatgtttttgctggtattgatccagacttggatgctcaagtggaatttggaacattcca
aaaagtcggagatccaactacgaggagacagccagtctgaggccgcattgctttggtatctttcacctctctttttttgatttgacatgggaaacatctcccatcccttctt
tgactctttttcctttttatacgggaaatgatcccaaatgacaaatgaataggtgtggaagttataattgtaaataaaccacgatcgaatctatggaagcattggtttatac
gttccttttagtttcgactttagggataattttttcgctatcttcttccgagaaccacctaaggttccgactaaaaaagtgaaataatttaattgaagtaagaagtctcccca
tctgggagacttcttacttcaattagtccccgtgttcttcgaatggatctcttaattgttgagagggttgcccaaacgcggtatataaggcatacccagtaaagcttacaa
gtaaaccagatatggagatggcgactaaagttgctgtttccatttttatagaatttcaagattacaatggatctacgaaaagatcgtgtatttacaactacaacggaatag
tatacaaagtcaacacaaatcattaaatggaatttatggctacacaaaccgttgaagatagttctagacctggaccaagacaaactcgcgtaggtaatttattgaaacc
cttgaattcggaatatgggaaagtagctccgggttgggggactactccttttatgggggtcgcaatggctttattcgcggtattcctatctattatttttagaaatttataatt
cttccgttttactggacggaatttttaatgaattaggtttctactaacgaaaactacgaagtcatagtttttccatccaaaagagcctttctactttaagctctacatttctaga
cattctggtagttcgaccgtggaatttttttggtttcggtatctctggaatatgagtgtgtgacttgttagaatttgctcctattgataatacatagaaagcacctgttatctcta
tcaagatgattctaattcgtcggatattatttattctagtatctggaacacgaaatagatagagtggatcaagaaaaaaaaaaatgaaactatgattcatattaactattcag
acctcgcaaccagactgaaaaaaattcaagtagttcttaataaaaataaaaaaagaaaatttcttccttccaattttgtttgcccaaaaaacaactttttttctctcgattttgt
cgagtcattacaccgattcaataaatgatcatcaagcggttcttattcgaagaacccttgccttttgtttagcttgagactcaatcatcgtggctctagtatgaatctaagg
ttttaattgaactgattcataggatcgcaacaagataatttctaccagaaaactactccaatttttgcttatttatttatctagtaaaacaagagtaaatctgcattacgcac
aaaaaaaagaaatccaaaatagggaagagaaaaatcaagaggcctctaatgatcaacatttgggaaagaaagatagacgagccaacttgagattttttggcattatc
atcacaaagaagaaattctggatttttcttatttcatatcttcaaggcaaatcgacccaacccagtggctgatgaagttttgaacctttttttctaatatccgttgaaaatttgt
gtgtttctgtttgagccgtacgagatgaaattctcatatacggttctcggagggggggttcgggttagttacctatctcaataaagtatatgattggtttgaggaacgtcttg
agattcaggcaattgcagatgatataactagtaaatatgttcctcctcatgtcaacatattttattgtttagggggaattacacttacttgttttctagtacaagttgctaccg
gttttgctatgactttttactatcgcccaaccgttacagaggctttttcctcggttcaatacataatgaccgaggccaactttggttggttaatccgatcagttcatcgatgg
tcagcaagtatgatggttctaatgatgatcctgcacgtatttcgtgtgtatctcacaggtgggtttaaaaaaccccgcgaattaacttgggtcactggtgtggttttagct
gtattaactgcatcgtttggtgtaactggttattctttaccttgggatcaaattggttattgggcagtcaaaattgtgacaggtgtgcctgacgcgattccggtaataggat
cacctttagtggagttattacgtggaagtgctagtgtgggtcaatccactttgactcgtttttatagtttacatacctttgtacttcctctgcttactgccgtatttatgttaatg
cactttctaatgatacgtaagcaaggtatttcgggccccttataaggaaggcatctcatagagagttctaattctcatatatcatatcgggtaggttgtggtatttcattgct
acaaacatgggttattctaaaataagacatgtcatttagatacttctcttcaactccgaactattgtgatacaaataatacaaatagttgaagttaattttacgaaagaaaa
gaaggcggattatgggagtgtgcgacttgaattattgatttggccatgcagatagagagttggatctgccacattagaattcacgaccaaaggtgtctccatatccaat
caacacgtaagtcccctgtctagtaaggataggctggttcacttgaggagaatattttctatgatcatacctcaaccatgtcatccatgaagaggctccgtaagatccc
atagagtagaaatggaataagtcatgtgacatgatccaattctctatttattacacttactttttattatagtatggaaatgcattcatttctttgcatcgattgcgatctgcaa
tactatcggagtaaaagaagggatctaaggaagaacgtaggctaaactttttgatttttttattagtaacaagtaaatactttgtttggacgtaagaaacttgcaatattgg
gggggataaataccaactaatcaagagacgcgagacaatccacaaagcaattgatcatgatcaaatttgtaagcccacttggatattgagcatttacccataagagt
aggattcttttcaatgaatagttgtaggtgcaacttcggaaaatagaatctgataaagctttcttacttagagccattgagccattatataccttattctattatggatcttct
acggttttatttctttcattcttgctcgagccggatgatgaaaaattctcatgtccggttcctttgggggctggatcttaaagaattcacctatcccaataacaaagaaacc
tgacttaaacgatcctgtattaagagcaaaattagctaaagggatgggacataattattacggggaacccgcgtggcccaacgatcttttatatattttccagtagtaat
tctaggtactattgcatgtaatgtaggtttagcggttctcgagccgtcaatgattggtgaaccggcggatccgtttgcaactcctctggaaatattacccgagtggtactt

ctttcccgtgtttcaaatactccgtacagtacctaataagttattgggcgttctcttaatggtttctgtgccgacgggcttattgacagtaccttttctagagaatgtcaataa
attccaaaatccatttcgtcgcccagtagctacaaccgtttttttaatcggtactgcagtagctctttggttaggtattggagcaacattacccattgaaaaatccttaactt
taggtctttttagggatttttcagtttgattcattcaatcgtgaagtaccgtgcataggtatctaggaaatagttacttccaagtgaatcttccctagatacctataatctattt
tattatgatccatttcgcgaaaatatagattgtcccaaagatgcaaaattgttttctttttctttttattctaactcgaaaaagaagaagaggaaaaaattgcaatggattta
aaactagaacttattcttaggtaaatccattgggagatgcttctctagagtgtcccatatctgttttccatcttgcatacgaaaactgtcaattctcatcagatcttcttccgt
cttactcaaaaggtccaatagtgtatggatattggcccttttgagacaattatacgttctagaaggcagttctaattgatcaataaaaatacaattcaatggaattcttttttt
gtttttctttagattagttaatctttttgaaagcttaaaagggggggaagtaaacctgtttttattttcttggaaacgagtaccctcttcctccgtgtgaagaaaaggaagaa
ataaatcaatcaaattacgagaagcctcataaagcgcttccttaggggttaaacttccattggtccatatttctagaaaaagtatctcatattttgcatttccattcccacaa
gcaaaaatactataattcacatttcgaacaggcatggatacagcatctataggataacttccatcttgagtgttctttctgacttccgtgtgatatccgcgatctctcttgat
ccgtaactcaatacggaaatccgtgggttctgtcaagttagctataggttgtgccgtatcaacgatttctacggaaggcggtaagataatatcttgggcagttatgtatc
taggacctttgacgcaaattgatgcggttctaactccatagagattacttctcaatacaatttctttcaaatttagtaaaatttcttgtaccgattcttcaatacctgctattgta
gaatattcgtgcggcacgctcccaaattttgcatgtgtgatacatgttccttctgtttctccaagtaaagctcttcgcaaggcaataccaacggtatccgcttgaccttttc
taagcggggacagaatgaaacgaccataataaagacgcttactatctactcttgattcaacacacttccactgtagtgtttgagtggatcctgctacctcctctcgaac
catatagactagtattattatttgatcattgaatcgtttatttctcttgaaaggggtttaattcttttacagacgtcttttttttaggaggtcgacacccattatgcggcataggtg
ttacatcgcgtatacaacttaatcgtacaccacttttagcaatggctcgtaatgcggcatctcttccactaccagcaccctttaccataacttctgctcgttgcaaaccca
ctgtacgaatagcatctacagctgttctttgaccagcatagggtgatgcttttcttgagcttttgaatccacaagtacccgcggaggaccagaaaaccacccgaccttg
cggatctgtaacagttataatagtattgttgaaactagcttgaacatgaataactccttttgttattctacgtgcactcttccgtaaactaaaacgcgcattcctacgcaaa
ccaatacgtactttcctacgtgaaccaatttttggtatagcttttgtcatattttattatctcataaatatgagttagaaataacaaaaagaaaaaaagatacaaagatatcc
gtttcagggtaaaatatatcctttacttgaattttttatttggaatttggacattttcgcgggtactttttttttacttttttagaaagtcaagttctttttcgaaagattacccctgtc
tttgtttatgcttcggattggaacaaatgactctaattcgtccacgcctacgaatcagtcgacattttgtacaaattttacgaacagaagctcttattttcatatttccgtattc
ctttcttaaactatgaatttactcttttggaaaaaataagtctctttgtttgaattttagaactttgaatttttttaccctagaaaaaaaaagaaaaacctaaccctttgaatctttg
gtatccttcaaatcttcggtatccttcgagtcttcggtacgcttcgaatccttatggggaagtctataaattatacgccccttgcttgaatcataacgacttacttcaattttg
accctatccccccatcagtattcgtatagaactagaccggatctttcctgaaatatagcccaggatgatggtgtcattctctaggcgaacgcggaacattccgttgggta
gggcttccataactaaaccctcgaaagtaatttttgcttctctcgggtttttttttctctcctatttttcttttctgtcatattttttttctcctatttttctttttttatttttcaaataaaaa
aacgttgtatttttgtttgaaaaataggaagttcaagatagaatttgggtactataactataggtggggcgattaccgctataggcggggcgattaccatatataacata
agacttctcccccaattctgtttagtcgagcttctcgatctgtcattatacctcgagaagtagaaagaatagcaattcccattccgcccaaaaccttaggaattccttgat
agttggcataaattcgtaagccaggtcggctgatacgctttaaaaaggttctagttctatatattccctttctagtctttctcttttgatgtcgcaaagttgaaaccaagaaa
tatctgttactttcctgatgtttccgaacactttcaataaaaccctctcgtagaagtattttaacaatgttttcggtaatatttgtagatactacccgaacagttcctttttttattc
atgtccgcgtttcttatagaggttagtaaatcagcaatagtgtccttgcccataagactctaattctaggttcctcctaatttttctataatcaacatgttctctttttttcttttcgt
tttggattctaaagcatatacgtgaaacacaatctactaatttttttatttgatctatatcttgtctactagtatttataatacttcaggagctaatgaaactattttagtaaaattc
aattctctcaattcctcggcgatcgcgccaaaaacgcgagttccttttggatttcctttttgatcaatgataaccgctgcattgtcgtcatagcgtattattataccgtcttcg
catttgaactctttacatgtacgtacaattacagctcgaattacttcggatctttctagaggcatttggggcactgcgtctttgattacagcaacaataacatcaccaatac
gagcatatcgctgattactagcagctcctatgactcgaatacacatcaattttcgagctccactgttatctgctacatttaaaagggtctgaggttgaatcatattattttga
tttcaatttgttatttcaatgcaaaaggatgaaagaaatattgtctttccagaaagaagaacctggtttttttattttcaatactactttttgggggggttatatttctaaccgaa
gaaattgacttcgtatgggcattttgctggcagctatggaaatagctgctctagctacagtttcggatactccgcccatttcataaagtattcgacctggtttaacaacgg
ctacccaatattcgggggatccctttcccgaacccatacgtgtttcagtcggtcttattgtaaccggtttgtcgggaaatatacgtacccagatttttccaccacgacgtg
catatcgtgtcattgctcttcgtcctgcttctatctgcctcgccgtaatccaagtgggttcaagtgcttgaagagcatatctaccaaaacaaatacaattgcctcggtagg
attttcccttcattcttcctctatgttgtttacgaaatctggttcttttggggttatagtcgatggttctctcttagttccatctctactgcaaaactggacatgagagtttcttctc
atccagctcctcgcgaatcaaatgagaaagtgtgcaaatttctctaattccacaatattctaaaatattacggatagatacacattaataggaaaggttagggtttttaata
ttgaatttattaaaatactaaaatatatagtcaagaaagaggatctagaatatatctagatgtgtatgtttatttatctatattctatatttaatgtaattcttttttttttttttttgaatc
tccttttttttattttactcttattgaatcgcggtaaagtattctaattcaataaaaaatttcgcgggcgaatatttactctttcctgtcttatttgttaattcataaccttatcaaataa
gacaatttttttggtttgttccgccatcccacccaatgaagtattgggattcttttcaagaaaatcctatccagtcataggttctgtcgttcccactgcttctcctttaatggtt
aggtttgaatctcgcaatggagcttccaaaaaaatttctttccgagtcaatttctcagttttattaactcgggacgctctttgttattccttgaaatttgtatttcttgtttcaatca
aattacgatttcgaattctctgttatttttattatattgatgctttatcacattgctttttatgatgtaattcatagaccatacatattggaatcctatatctttcttatttctcttccttct
ttctatcatccttccttttatctacatcccctttagttttgcttcacaacttagaatcctatttttttttaaagaaaaaattgcagttgctacacgtatatgatagatttactcatttat
gatagatgtatttattcatatagtgactgtttcttagttaggatctcgacaatacgaagcaataggttggttattagttcattttctataattacatagtagggttcattctatttttt
ttttaatccccttttttgaaccctaaaaaaaaaactaacgagtcacacactaagcatagcaattttattaaaagatttctcaattttcattaaatcttatagaaagaggtagaatt
tcttcttttttttcagggatttttagggaaaataaggctcttgtcattttttattctattactgaacagaatgggaagacagggttggttattcttcgtctacgaatatccaaatttt
aactcctaatactccatagatagttcgaattggatagcagcaataatcaattttagcgcgaattgtttggaggggaagtctacctttttttgatgcattcggcacgcgcaat
ttcttttcccgcgagacggcctgcaattttttactttttactccctttatatctgttttttttagttaattcaatggctttttttcattgcctttcggaatgaaactctatttttttaattggaa
agctatatattctgcaagaatgttaggttgtctataaggttctttcactttttcgatcccaatattaagtctctggtttacagaattaacttctttttgtagatctttctctaattctt
cgattgctcctttttttctttaataaattggggaatccaatatggattatgacgtggattgtatcgatttctttttgaatttctatatgtgtaattacttcggaacttgagcctgagt
ccatttttttattatgtgtaattacttcgaaacttgagtctgcttctatttttctattcgagccctttttcctattcttttgtatatagttcttgatacaattccgtattttttttatcttcctg
tagaccttcagaataatttttggttgtgcgaaccaaaaggaatggtgattttgggttgtaccaagtctgaaaccaagtggatttattttttgtcccatatttttctattctatttt
tttttaccgggaatcaaatcttagatggatctaaagattatttcgatttctttactatatttagtacaattgttatatgacacatggtttttttttatgggagaactacgtcctcgag
cccgaggtctgaatttattcataatagtactcctactgacttcggctttagtgatgaataaatttgctttgtcgaaatccctataatgagtagcatttgctgctgccgaataa

accaactttaagatgggataagatgctcgataaggcatgaggttcaatatcataacagtttcttcgtagtaacgccaacgaatctcatccagaactctttgtactttgaaa
acggacatgtggatgcgtttttgtgctttgaaaacccgttcgaacttaagtagacgatcggttggtacttttcttgcgagtttccttagcccactcttcttcttctttatcctag
gggtatactttaccagtttgaaacttgtcataaataaggttattccccgcttacctatttctttttttttattttgaatctttctattctgaattcagttaacgacgagatttagtat
cctttcttgcactttcataactcgtgaaatgccgagttggtacgaattcccccaatttgcgacctaccataggatttgttatgtaaataggtatatgttcctttccattatgaa
tcgcgattgtatggccaaccattgcgggtagaatgctagatgcccgggaccacgttactattgtttctttctcctccttcatattgaccttttctatttttgccaataaatgat
gagctacaaaaggattcgttttttttcgtgtcacagctgattactcctttttttccattttaaagagtggcattcgatgtccaatatctcgatcgaagtatggaggtcagaata
aatagaataatgatgaatggaaaaaagagaaaatcctttagctggataaggggcggatgtagccaagtggatcaaggcagtggattgtgaatccaccatgcgcgg
gttcaattcccgtcgttcgcccatcccattattgcaaattccaaaaatgcaattttccatattcctagttacgtatttacttacggcgacgaagaataaaactatcactatatt
ttttccttttcctagttcttcttccaagcgcaggataaccccaaggggttgtgggtttttttctaccaatgggggctttcccttcaccgcccccatgggggtggtccacag
ggttcataactacccctcttactacggggcgtttacctagccaacacttagacccggctctacccaaactttttttggttcaccccaacattacccacttgtccgactgttg
ctaagcagttttgggataccaaacggacctccccagatggtaatcttaaagtggccgatttaccttcttttgcaatcagtttcgctacagcacctgctgctctagctaatt
gcccacccccttccacgtgtgatttctatgttatgtatggccgtgcctaagggcatatcggttgaagtagattcttcttttctctcaaaaaacccccttcccaaactgtacaag
cttcttccaaagcatacggctttctagatgtatatgacgatctctagacagatggatcttatatgaatcgtatgatgaagtaccacatgagtggatatataggaaaggaa
tccaaatctgccgaatcgctcatgttatgatcttctacatcctaggtctccgcgttccgtcatctggcttatgttcttcatgtagcattcagatcgaatgactctatgaaatta
cgtcgatacttctacatattatgggtaacgtaggagacatccctattttcccccgggggtcttaattaccactgcttagctttcaattcgcctctgaccatcaaattaaatgt
gaataacccgtcctcctctctttgaaacaaggggcgcttccggttctgtgcgtgcttcaaacaattttgtcttctccatattaccatatctctagagtcaataattttctatga
ggaactactgaactcaatcacttgctgccgttactcaacagttttctgttgaggtctatcccgtagaggtagtcaaattggatcagtgatcgatttctaggtttcgtcgtaa
acctaattggttacttccaattacgtaaatcaatagttcaaaccgcactcaaaggtagggcatttcccattgatataggaacttttgtaccagaaacaatagtatctccaa
ttatagcccctctgggatgtaaaatatatcccttctcaccatccccatagtgtatgagacaaatgtacgcatttcgattagggtcgtattctatggttacgattctaccaga
tatgtctttttgattccgtcgaaaatcgattttacggtataggcgcttatgacctcccccctctatgccttgcggtaatgattcctctggaattacgacctttaccacaacggt
gccgtccatggatcaaattatttcgtggattggatttcacttgcctatctatggttcccttgcgtgtgctcgggataggtgttttgtataaatgtttcgccgtattattaagtat
tctcctttagttttttctctatctagaagtggaatagaataacccggttgaagggtaatgatcatacgtctgtaatgcattgtatgtcctagaatagggcccattcttctacc
ctttccgggtagtcgatggctattcacagctactaccttaacaccaaagaagagttcgacccaatgctttatttctgtcttagtgaatcccgattcgacattaaaagtatat
tgattctttcccaataaacgaagactttttctgtaaatactgcgtatttgattccatccataaatcgactttccctcctatgctctgagttccagtatcgataagaattcgagt
tcttattgttcttatgttatggtatgaatataccataccaattcgttatgtatggatgatggatgagattccatggatagagagccagttccaatagacttatggaacgttcc
ggttcgcgtgcatccagcaggaattgaacccgcaaatttaccaattatgagttgggcgctttaaccattcagccatggatgcttaacagggatcatcgtacatcgtaa
ataaccaattttcatatagaaagacatatcatagaaaaatgaaatcgaaaatattcggagatggcaaatattcggagatgactatgaaaacacctctctggatcctcga
attgaaagagagattgagagggatccagaatcctaattctcgctatttggaatggatccaattctattgagtctgactcatagtgatcatttctctttagcaaagaatgac
cttggttatcaaaggattgaacaaccgggatccgtttacttatgatacctagttgacattgataacaaggatctaatgaattatgagtttaatagatcctctttagcagaaa
gacgtatattccttgctcatcacttattcccaaacctcgtatggggctaatcgttttcatttaccatctcagggaaaatccttttcgttccgcttagccctatcgggtatttta
gtgataggttctataggaactggacgatcctatttggtcaaatacctaatgaaaaattcctattttcctttcattaaggtacgagggcttcttattccacaagaaaagaacg
aaagcaccttttcattctttcatatactgggggtttttacttggaaaagacaatgttccatactaaaggattcgggtccataaccacgagttccagtgcactagatcttgta
gcacttagcaacgaggccctatgaatagacatatagaatttttggtcgggaaattcgaatgaatcattgagtgaaaaaggagcaaagaatgacaaaagacgagact
ctactagtcttcactcttgtggtttcctcggtttctgttttcttattcgggatcttgcttttcatggttctcatctctgcaactcgcgattttcgcgagagaaccaaatccaagtt
ggtgaagatcatgatttgggctggcatagtagtgattacctttgcaattgcggttcgaatctatccgatctttatctttttgctcaaagaacgaataaaaccccttgtcgaa
gccctttatgataagcttccctggatctgggaagtttctctttcacggtattgggatcgtttgatcgatttccttgatcgctacttatgggcgtgcgctcaaaggatacaaa
cagggattcgcaaacaaaaaggggaattcgtagtcactttttcctgtcgcgtaaaaaaaaggctttacgcgagagcaatagaggttgggatacatctatctcttctga
gcaacctcttttggattcttaagaccacccttgcagtaggataccgtctgctttgggttctttattatattatctccttcgagggatttttaggatcgttcaggctatatttagt
ctattttggcttttactgtctccttttctcagggaagtggttaaggacctcagaagatagaggagagcgccaggcgcagatttccggaatacttctacggggaatgctc
attgaatgcgcattctccgtattatgccttgaagaggactcgaacctccacgctctttagcacgagattttgagtctcgcgtgtctaccatttcaccatcaaggcatcttg
aaagtgaatcatattccatgaatatgatatctatctaatgtgatatcttgaaagtgaatcgtattccatgaatatgatatctatctaatgtgatatatggaatatatgacaaag
gtggagtcttggagtatttcgatcgatcggtcatataggcctgagtcagacatcaaatagcttcgatttgcattatccgtaggacaccttatatgtatcaaaatcaaaaa
gatgtacaatccaatttctcgattcaatagaagcccaaagaggtgcatatggtacccaaataaggataggatagatatgtcaaaagcaggtctgattacacctattcct
aatcctaaatagaatgtaaggacgtggggatttctatgtaaacagagtatcctatttccataggctcgaatgacccccttctcataataagaatgtgcacggtctggtccg
gtatggaatgaacttataatctgatgatcgagtcgattccatgattataagttcattaccctagcgcccattcccattttgggcggaacagatctactaattcttttattcca
gttagtaagagggatcttgaactaagaaatagacctagcagctaaaagagggtatcctgagcaattgcaagaatggggttcattgatattcctggtatagtagatgct
atcacacatacagtcatactcaattcgatggaattgtttgatcttaaaggggatcttctataatttcgcacataaggggttatttcttggtttcgtccagtcattaataacttg
actatttttagataatagtagatagaaagaacgctcgtaaggagtcctattgaaaccaagaaatataggcctgcttgccatccacaccagaatagatagagttttccga
agaaacctgctagtggaggaaggcctcctagggataagagacatagggctaaagagagagccaaaaaaggatctttcgtgtataatcctgcataatctcgaatgtt
atcagttccggtacgtagaccaaataatacaatgcaagcaaaagttcctagattcatggagatatagaaaagcatataagttatcatgcttgcatatccatcatttgagt
ctccaacaattattccaataattacatatccgatttgccctatggacgaatatgcaagcatacgtttcatgcttgtttgagtaatagcaaggagattccccaatatcatgct
aagaatagctaggatttccagaagaagatgccattcgtttgatgagaaataaaaaggaatatcgagaattcgcgtggctgaagctgaagcagcaactttcgaagtaa
cagaaagaaaagcaacgactggagtgggggagtcagagtcgaaaagaggattcctcgcttctttctctcatgcaaaaccgtgcatgagactttcatctcgcacggc
tcctaagtgataaaagaaagaagaacttgtcttctttctttttttgattaccttcctcgcgtatgtataagaccgaatccattctttttcgaaatcgatttcgaaaaagaactac
taatccttaacttttcgaggaatccttcatcagtggttgtgaatgactgactttttcaatcctttcgaccttggttccgtaggagcaagtcagaaaggttgagaaatagaac
catctgatttgattcgttcccaatagccatgagatgatcatcttagggtgatccttttgtcaacggatgctcctattacactcgtagtctctgaaggatgagaacccactat

gtagcatctacgtcgataattcaagcattgtatacgtcattagtccgattctttgtaggaactacccgtaataacgagcttgcaaaatggatctgtttatcataaagagatt
cgttgttcctgaccctgcttcaccttaattgttatttgaacaaaaagatcacaataaacttttggtaaaagttctgtcttggtcggagtggggatagcatttctcttctgcata
tctatggagttttgcaaaacccaaacacctcagagatagatatagaggtaggaatttgtcgaacgaaccacactccttcgtagacgtcaggagtccattgatgaaaa
ggggctggggaaagcttgaacccaagtcctacagtgatggatataagcgcaattgaaattcctggggagttatacatttgtgtattgataagaccgttcacaatttctt
gaagctcgatctccccccagatgaaccatatagccaagagaaaccatgaaccagaatagaagagcttgccccacccatgagtaaatatttcatagtagcctcatta
gaccgtagatctctcttggtatatccagacaataggtaggaacataaactgaaacattctggagctacaaagatagttattaaatcgttagcaccacataaaaacattc
cccctagagtagctgttaatacgaataacagaaactctgttatagccatttctgtacattcaatgtactctacggatagaggaatacataaagttgaacataataaaataa
gaaattgaaagatttcgttgaaattgttcgtttggaaatttcccgaaaagctaattataggttcttctctccatcggaacaatagggccgttatgcttattactaaacttgttg
aagagatgaaatagaaccaaggtctatctttttgatcagaggttaaatcgatcatcagaagaagaattaggccaaaaattaggatacattctgggaaaatgaaacttc
cctggaagagaagcaaatgaaacgctttcataaaaattctcgtagaatcgagaatgaagttttcattctgtacatgccagatcatgaattagtaactgcatccaatctcc
gaaaagtcccgattgtttcgatttttggaatgggatatttacggaatccccatgaataggatcaaaccttattccatgctatttccataagattcctctttcttattcttaagca
agcccccgagagggcttagttgatcatgatttctgttttctctttcttttcctttttgtttgtttcgagaaagatatcgtccgattctccttctattgattcttttccgatcgagatg
tatggatccatgtgtctacatacctagattctgttcatggattaacgaaaatgtgcaagagctctatttgcctctgccattctatgagtcgcttcctttttgcgtatggcacc
cccacccccctttggcagcatctactaattcggaacttaatttgaaagccatatttcgacccggacgcttttgggatgcttctaataaccaacgaatggcaagtgctcttc
cttgtttagatcctatttcaatcggaactttccgcgtcgatcctttttttattacgtcttgtttttactcctatattgggagttactctacgtattgcttgacgtaaaaccaatagtg
gatttgtttctgtcttttgttgaatctttttcacggctcgatagagaatttgataagccaatgatttttttccgtctttcataatacggttaaccaccatgttaactaatcgattac
gaaaaattggatcggattttgcagttctttttctgcagtacctcgacgtgacatgagcgtgaaagaggttcaagaatccgttttctttttataagggctaaaaacgaatc
acttatttttttggctttttgaccccatattgtagggtggatctcgaaagataggaaagatctccctccaagccgtacatacaactttcatcgaatacggctttccacagaa
ttctatagggatctatgagatcgagtatggaattcagtttactcacttaaaattgagtatccgtttccctccttttcccgctaggatcggaaatcctgtattttccatatccat
accatcaagtccttaggtttccgaaatagtgtaatggaaaaagaagtgcttcgaatcattgctatttgactcggacctgttctgaaaaagtcgaggtatttcgaattgttt
gttgacacggacaaagtaagggaaaacccctgaaagaatttccatattgaccttggacatataagagttccgaatcgaatctctttagaaagaagatcttttgtctcatg
gtagcctgctccagtccccttacgaaactttcgttattgggttagccatacacttcacatgtttctagcgattcacatggcatcatcaaatgatacaagtcttggataaga
atctacaacgcactagaacgcccttgttgacgattctttactgcgacagcatctagggctcctcgaataatgcgatatctcacaccgggtaaatccttaacccttcctcc
tcttactaatactacagaatgttcttgtaaattatggccaataccaggtatataagcagtgatttcaaatccagaggttaatcgtactctggcaactttacgtaaggcaga
gttgggtttttggggttgatagtggaaaagtcgacagataagtcacccttcctgtccctctacagaaccgtacatgagattttcacctcatacggctcctcgttcaattct
ttcgaagggatccttttcctcgttcgagagtctccgcccttcttccactccgtcccgaagactaactaagaccaattgagtcacgttttcatgttctaattgaacactttcc
atttatgattaaaggagaagattgttcttttaccaaacatatgcggatcaaatcacgtcttataataagaagaaatctttctcggtatcaatccccttgcccctcattctttga
gaatcagaaggatccttttcgagtttccatttcttcatttggaatctgggctcttctatcttcgacttattttttttttgctttattctttatttatttcatttcgatttttccctcttcctct
atccctatcctctaggtacagcgtttgcatcaatagagaaccttttcctctgtatgaatctatattattccattccaatttcttcccgaaacttcccaagaaaaatcccgaatt
ggatccaaaattgacgggttaatgtgagcttatccatgcggttaggcactcttcaaataggaatccattttctaactggctttcgtgctttggtgagtcgtccgagatcctt
tcgatgacctatgttgtgttgaagggatatctatatgatccgatcgattgcataagacccgcggtagcaatagaacggggaaagtatacagaaaagacagttcttttca
atttcgattatctatatattagttcgtttctatttctagatatctatttctatatattagtattagttagtagtactattctattagttagcgatcccggctctgtgagttctttcttccg
tgatgaactgtcggcaccagtcctccatttttctctgtggaccgaggagaaagggggctcagcaggaagaggattgtaccatgagagaagcacagaggtcaacc
ctcttcaaatatggaacatggattctggcaatgcaacgtagttgggtcctcatatcgatccgaatgaatcagtctttctacagaggtcaatctttgcctattaggcaaga
ggatagcaagttcgaaattctgtctcggtaggacatggatttctattactatgaaattcataaatgaaaatgaagtagttaatgggagggctaccattatcctttttcttgta
tgtgttcctaagagaaggaatttgtccatttcatgtttcgaggtctcaaaaaagggcgtggaaacagatagaaactctggaatggaaattgaaaagaaatgtagcccc
agttccttcggaaatggtaagatctttggcgcaagaagaaggggcgacccatatcatcttgacttggttctgcttcccctctttttttaagaataccgagtcgggttcttct
cctaccagtatcgaatagaacatgctgaacaagatcttcttcatggaaacctgctcgatttagatcgggaaaatcgtacagattttatgaaaccatgtgctatggctcga
atccatagtcaatcctattttcgataggaccggttgacaattgaatccaatttttcccattatttgactatccataatagtgtggaaagaaagcccggaggaagggtggc
cttgagtttctcgccccttttgccttaggattcgttaattctctttctcgaggggacggggaagggatataactcagcggtagagtgtcaccttgacgtggtggaagtcat
cagttcgagcctgattatccctaaacccaatgtgagtttttttctattttgacttactccccccgccacgatcgaacgggaatggataagaggcttgtgggattgacgtga
tagggtagggttggctatactgctggtggcgaactccaggctaataatctgaagcgcatggatacaagttatccttggaaggaaagacaattccgaatccgctttgtc
tacgaataaggaagctataagtaatgcaactatgaatctcatggagagttcgatcctggctcaggatgaacgctggcggcatgcttaacacatgcaagtcgaacgg
gaagtggtgtttccagtggcgaacgggtgagtaacgcgtaagaacctgcccttgggaggggaacaacaactggaaacggttgctaataccccgtaggctgagga
gcaaaaggagaaatccgcccaaggaggggctcgcgtctgattagctagttggtgaggcaatagcttaccaaggcgatgatcagtagctggtccgagaggatgat
cagccacactgggactgagacacggcccagactcctacgggaggcagcagtggggaattttccgcaatgggcgaaagcctgacggagcaatgccgcgtgga
ggtggaaggcccacgggtcgtcaacttcttttctcggagaagaaacaatgacggtatctgaggaataagcatcggctaactctgtgccagcagccgcggtaagac
agaggatgcaagcgttatccggaatgattgggcgtaaagcgtctgtaggtggcttttcaagtccgccgtcaaatcccagggctcaaccctggacaggcggtggaa
actaccaagctggagtacggtaggggcagagggaatttccggtggagcggtgaaatgcattgagatcggaaagaacaccaacggcgaaagcactctgctgggc
cgacactgacactgagagacgaaagctaggggagcaaatgggattagagaccccagtagtcctagccgtaaacgatggatactaggtgctgtgcgactcgaccc
gtgcagtgctgtagctaacgcgttaagtatcccgcctggggagtacgttcgcaagaatgaaactcaaaggaattgacgggggcccgcacaagcggtggagcatg
tggtttaattcgatgcaaagcgaagaaccttaccagggcttgacatgccgcgaatcctcttgaaagagaggggtgccctcgggaacgcggacacaggtggtgcat
ggctgtcgtcagctcgtgccgtaaggtgttgggttaagtctcgcaacgagcgcaaccctcgtgtttagttgccactatgagtttggaaccctgaacagaccgccggt
gttaagccggaggaaggagaggatgaggccaagtcatcatgccccttatgccctgggcgacacacgtgctacaatgggcgggacaaagggtcgcgatctcgcg
agggtgagctaactccaaaaacccgtcctcagttcggattgcaggctgcaactcgcctgcatgaagcaggaatcgctagtaatcgccggtcagccatacggcggt
gaatccgttcccgggccttgtacacaccgcccgtcacactataggagctggccatgtttgaagtcattacccttaaccgtaaggagggggatgcctaaggctaggc

ttgcgactggagtgaagtcgtaacaaggtagccgtactggaaggtgcggctggatcacctccttttcagggagagctaatgcttatgcttattgggtattttggtttgac
actgcttcacgcccaaaaagaaggcagctacgtctgagctaaacttggatatggaagtcttctttcgtttagggtgaagtaagaccaagctcatgagcttattatccta
ggtcggaacaaattagttgatagtgataggatccccttttttgacgtccccatgcccccccccgtgtggtgtggcggcatggggatgtcaaaaggaaagggatggag
tttttctcgcttttggcgtagcaggcctcccaaagggaggcccgcgcgacgggctattagctcagtggtagagcgcgcccctgataattgcgtcgttgtgcctgggc
tgtgagggctctcagccacatggatagttcaatgtgctcatcagcgcctgacccgaagatgtggatcatccaaggcacattagcatggcgtactcctcctgtttgaat
cggagtttgaaaccaaacaaacttctcctcaggaggatagatggggcgattcaggtgagatcccatgtagatctaactttctattcactcgtgggatccgggcggtcc
ggggggggcactacggctcctctcttctcgagaatccatacatcccttatcagtgtatggagagctatctctcgagcacaggttgaggttcgtcctcaatgggaaaat
ggagcacctaacaacgcatcttcacagaccaagaactacgagatcacccctttcattctggggtgacggagggatcgtaccattcgagcctttttttcatgcttttccc
ggcggtctggagaaagcagcaatcaataggacttccctaatcctcccttcctgaaaggaagaacgtgaaattctttttcctttccgcagggaccaggaggttggatct
agccataagaggaatgcttggtataaataagccacttcttggtcttcgactccctaagtcactacgagcgccctcgatcagtgcaatgggatgtggctatttatctatct
cttgactcgaaatgggagcagagcaggtttgaaaaaggatcttagagtgtctagggttgggccaggagggtctcttaacgccttcctttttctgcccatcggagttatt
tcccaaggacttgccatggtaagggggagaaggggaagaagcacacttgaagagcgcagtacaacggagagttgtatgctgcgttcgggaaggatgaatcgct
cccgaaaaggagtctattgattctctcccaattggttggatcgtaggggcgatgatttacttcacgggcgaggtctctggttcaagtccaggatggcccagctgcgcc
agggaaaagaatagaagaagcatctgactctttcatgcatactccacttggctcggggggatatagctcagttggtagagctccgctcttgcaattgggtcgttgcga
ttacgggttggctgtctaattgtccaggcggtaatggtagtatcttgtacctgaaccggtggctcactttttctaagtaatggggaagaggactgaaacatgccactga
aagactctactgagacaaaaagatgggctgtcaaaaaggtagaggaggtaggatgggcagttggtcagatctagtatggatcgtacatggacgatagttggagtc
ggcggctctcctaggcttccctcatctgggatccctggggaagaggatcaagttggcccttgcgaatagcttgatgcactatctcccttcaaccctttgagcgaaatg
tggcaaaaggaaggaaaatccatggaccgaccccattatctccaccccgtaggaactacgagatcaccccaaggacgccttcggcgtccaggggtcacggacc
gaccatagaccctgttcaataagtggaacacattagccgtccgctctccggttgggcagtaagggtcggagaagggcaatcactcgttcttaaaaccagcattctta
agtttaagatcaaagagtcgggcggaaaaaggggagagctccccgttcctggttctcctgtagctggattccccggaaccacaagaatccttagaatgggattcca
actcagcaccttttgttttgagattttgagaagagttgctctttggagagcacagtacgatgaaagttgtaagctgtgttcggggggggagttattgtctatcgttggcctct
atggtagaacccgtcggggaggcctgagaggcggtggtttaccctgtggcggatgtcagcggttcgagtccgcttatctccagcccgtgaacttagcggatactat
gatagcaccgaattttgccaattcggcagttcgatctatgatttcgcattcatggacgttgataagatccttccatttagtagcaccttaggatggcatagccttaacgtta
atggcgaggttcaaaagaggaaaggcttgcggtggatacctaggtacccagagacgaggaagggcgtagcaagcgacgaaatgcttcggggagttgaaaata
agcatagatccggagattcccaaataggtcaaccttttgaactgcctgctgaatccatgagcaggcaagagacaacctggcgaactgaaacatcttagtagccaga
ggaaaagaaagcaaaagcgattcccgtagtagcggcgagcgaaatgggagcagcctaaaccgtgaaaacggggttgtgggagagcaatacaagcgttgtgct
gctaggcgaagcggttgagtgccgcaccctagatggctaaagtccagtagccgaaagcatcactagcttacgctctgacccgagtagcatggggcacgtggaat
cccgtgtgaatcagcaaggaccaccttgcaaggctaaatactcctgggtgaccgatagcgaagtagtaccgtgagggaaaggtgaaaagaaccccccagtgggta
gtgaaatagaacgtgaaaccgtgctgagctcccaagcagtgggaggggaaagtgatctctgaccgcgtgcctgttgaagaatgagccggcgactcataggcagt
ggcttggttaagggaacggaacccaccggagccgtagcgaaagcgagtcttcatagggcgattgtcactgcttatggacccgaacctgggtgatctatccatgac
caggatgaagcttggatgaaactaagcagaggtccgaaccgactgatgttgaagaatcagcggatgagttgtggttaggggtgaaatgccactcgaacccagag
ctagctggttctccccgaaatgcgttgaggcgcagcagttgactggacatctaggggtaaagcactgtttcggtgcgggctgcgcgagcggtaccaaatcgaggc
aaactctgaatactagatatgacccaaaaataacaggggtcaaggtcggccagtgagacgatgggggataagcttcatcgtcgagagggaaacagcccggatca
ccagctaaggcccctaaatgaccgctcagtgataaaggaggtgggggtgcaaagacagccaggaggtttgcctagaagcagccaccctttaaagagtgcgtaat
agctcactgatcgagcgcccttgcgctgaagatgaacggggctaagcgatctgccgaagctgtgggatgtcaaaatgcatcggtaggggagcgttccgccttaga
gggaagcaaccgcgaaagcgggggtcgacgaagcggaagcgagaatgtcggcttgagtaacgaaaacattggtgagaatccaatgccccgaaaacccaagg
tttcctccgcaaggttcgtccacggagggtgagtcagggcctaagatcaggccgaaaggcgtagtcgatggacaacaggtcaatattcctgtactaccccttgttgg
tacggagggacggaggaggctaggttagccgaaagatggttataggtttaaggacacaaggtgaccctgctttttcagggtaagaaggggtagagaaaatgcctc
gagccgaggtccgagtaccaagcgctgcagcgctgaagtatgagccccgtggactagccattgcttctccacgaggctcataccaggcgctacggcgctgaagt
atgtaacccatgccatactcccaggaaaagctcgaacgaccttcaacaaaggggtacctgtacccgaaaccgacacaggtgggtaggtagagaatacctagggg
cgcgagacaactctctctaaggaactcggcaaaatagccccgtaacttcgggagaaggggtgccccctcgcaaaagggggtcgcagtgaccaggcccgggcg
actgtttaccaaaaacacaggtctccgcaaagtcgtaagaccatgtatgggggctgacgcctgcccagtgccggaaggtcaaggaagttggtgaactgatgacag
ggaagccggcgaccgaagccccggtgaacggcggccgtaactataacggtcctaaggtagcgaaattccttgtcgggtaagttccgacccgcacgaaaggcgt
aacgatctgggcactgtctcggagagagactcggtgaaatagacatgtctgtgaagatgcggactacctgcacctggacagaaagaccctatgaagctttactgtt
ccctgggattggctttgggcctttcctgcgcagcttaggtggaaggcgaagaaggccccccttccgggggggcccgagccatcagtgagataccactctggaaga
gctcggattctaaccttgtgtcagacccgcgggccaagggacagtctcaggtagacagtttctatggggcgtaggcctcccaaaaggtaacggaggcgtgcaaa
ggtttcctcgggccagacggacattggtcctcgagtgcaaaggcagaagggagcttgactgcaagactcacccgtcgagcagagacgaaagtcggccttagtga
tccgacggtgccgagtggaagggccgtcgctcaacggataaaagttactctagggataacaggctgatcttccccaagagtccacatcgacgggaaggtttggca
cctcgatgtcggctcttcgccacctggagctgtaggtggttccaagggttgggctgttcgcccattaatgcggtacgtgagctgggttcagaacgtcgtgagacagt
tcggtccatatccggtgtgggcgttagagcattgagaggacctttccctagtacgagaggaccgggaaggacgcacctctggtgtaccagttatcgtgcctacggt
aaacgctgggtagccaagtgcggagaggataactgctgaaagcatataagtagtaagcccaccccaagatgagtgctctctcctccgacttccctagagcctccg
gtagcacagccgagacagcgacgggttctccacccatacggggatggagcgacagaagcatggaaataggataaggtagcggcgagacgagccgtttaaata
ggtgtcaagtggaagtgcagtgatgtatgcagctgaggcatcctaacgaacgaacgatttgaaccttgttcctacacgacctgatcaaatcgatcaggcacttgcca
tctatcttcattgttcaactctttgatgaaaagatgaaaaaaccaaaaaaaagctctgcccttccatctcttggatagatagagagggagggcagaggcctttggtgtc
ccttccagtcaagaattggggcttcacaattactagccaatatttctctcatgcctttcctcgttcatggttcgatattctggtgtcctaggcgtagaggaaccacaccaat
ccatcccgaatttggtggttaaactctactgcggtgacgatactgtaggggaggtcctgcggcaaaatagctcgatgccagaatgataaaaagcttaacacctcttat

ttgactttttcactattttgaaatacgaaaaagatccaaatccaaaatgcaaaggtcgtcttattcaaaacctcaatcatcacatcccctctctcccacttcacacctcgga
acgcactgttcttatagagagaaaggggctttcccatcttcttaacccgaaatgaaatggctgaggagagggaggttccttttgggggtacccccgggaagagat
ccagtggagacggggtgggcctgtagctcagaggattagagcacgtggctacgaaccacggtgtcgggggttcgaatccctcctcgcccacagccttccaaag
gggaagggcctttactttcccccctgagggtaggaaaaccatgatcgggatagcggacgtaaagctattgaacttgggtatgctctttccttttgtcgaagtggaatcgt
agaacagaatgtgatacgatgagataaaatgcaatagaaacaaggatagcgaacgggttacctactcctaagggtcaaagcaagccctttaattcaattctttattctt
acattaaagaatgaatcaaatctccccaagtaggattcgaacctacgaccagtcagttaacagccgaccgctctaccactgagctactgaggaacaagggggattc
gacctcctagagttcaactcccgctctcaacccatgaacaatatgagtccgaagcttctttcgtaactcccggaatttcttcgtagtggctccgttccatgcctcatttcat
aggtaagcccagagtggctctatttcattctatttcacttcctagcacttcctatcatttaatatccatccctttggtcttattgacataagagatgtcatttatagtctatctctt
tctatatatggaaagtcaagaaattctcatcgaaacatcgagaaattgtgcatatagaaaactctaaagaaagaaaaaaaggagacccatgccatgattttcaaatcttt
tctacttagtagtctaagtttctcgatgaggataattaattcggtcgttgcggtcggactctattatgggtttctgaccacattctccatgggtccctcttagatcttctttctc
caatcttggattagggaagaaggagatattcgcgactcctggtggtttcattatggggcagctcatgatcttcatatcgatctattatccacctctgcatctattctttctta
gctaaacgggtggaagatccatccaatttggttatatcatggactcaaaaaacggatctgaatgtgactgaaatgcacgatcttcacaggtatcacttttcacgatacct
aaaaggtggaatagcgattttcgaaccatttcctataagagaaaggtttccattactttgagaaatggattctatatcaaactatagctattgcattaaagaagaaaagaa
actaatagaagtcgaagacgcggaatggtagtgaatagagagaaagattcttctggttttcttgttcctgaaaatattctatctatctcctagacgccgtagagaattga
gaattttcatgtctttcaattctcgtactcgtaattggaaagttacggaaggagatccatcattttgcaatgaaaactacataaaaaactctggacaatttcgaaatcagg
ccaagcgtcttaatacatatgcaaaaaaattcattattggcccaccattgattagaagatttaacttgtatgaatcgctattggtttgatacgaataatggcagttgtttcag
tatgttaaggatacagatgtatccacaattcatttagagttacttaatagcctatttcttataccatatctctatcccgtgaaattctcgagccgaaagatggatgcatatgc
tatgtttcattttgctaaatgatatcaattaaacggtgtatcaattccataaattggatatagcaataaataaatcagcaaaattcttttattttagatagaagaaacttttcttct
atctaaaataaaagaatgtacccttctatccaaatccaatttgcatcgataaaataaatccaaattccagtagtagatgaataattgcaaattttgtgtgtacgagattag
aataacttcaaaataactgacataatttttatttttcctgatcagaaaaatacatgaaaaagaaaggaggtagaaaaatttttggatttatggttaaagaagaaaaagaa
gaaaactggggttctgttgaatttcaagtattcagtttcaccaataagatacggagacttgcttcacatttggaattacacaaaaaagatttttcatcggaaagaggtctc
cgaagacttttgggaaaacgtcaacgtttgctggcttatttggcaaagaaaaatagagtacgttataagaaattaatcagtcagttggatattagggagcggtaatttca
tcgttcgaattttttttcttattttattagtagtcttatagtagtcttagattttgcattttgatgagcctcgttttgaggaattcatggaataatccattttcatggaataatgaatta
aggaagaaaggatatgagtctaccgcttacaagaaaagatctcatgatagtcaatatgggccctcagcacccatcaatgcatggtgttcttcgactgatcgttactctc
gatggtgaagatgttattgattgtgaacccatattaggctatttacacagaggaatggaaaaaatcggcaaataggaaagctacttaggcaggagataggggaattc
cttaagaaagaaaaaagaataagaacacagatacataacataaaaaaaagaataaataagacgaaattcgacctcccccctacatatttaatttcttctcctatacaaaa
actagcaagacctactccattggtaattccatcaatgacacccttgtcgaaaaactgcgttagttcggttaatcctcttatacccaaggtaaaggtcctagtatagaaaa
tatctatataaccgcgattatatgaccaactatatatcttttttttagttgatggaaaaaatactttttcggaccccccttttacaaaggaatttattaaatccaaattctgaaaa
aaagagtaagcagatccataaaacatatatgctatgaatagaccaaaaatagctagacttacagaagaaattgcattagtgataaattcatatgaatttatggaagaatt
agaactttcttggaaaaagttgattgagggagttagccactttgataatatggttaattcccctatttcattatcaaaatggattcctatagatccaataaacaaagtacaa
agcagtaatataagaagaggaaatagcatagtatttcccggttcatgaggatagacaaaagtgtttttagtccccaatgaagtactaaaggaccctatcctatttcctgt
attaacatgaattttggatagattttgtgaaaaaaaagaaactccactcttcgctgttgataaaacgaaatctctattgactcctttagatatccttttttccccataacgatatt
gaatacaacgaatcctctttagtactactgtaattttgaaaatgaacacgcaaatacccatcaaaagtaagtaaatatatccgaaacatataaaacgcagttaatcctgc
agtaaaagaagctattattccaaaaaagggtgaatataaccaactattactaaggatttcatctttggaccagaagcaagcaagaggtggaataccacaaagagaaa
gggtaccccataaaaaacaagttcttgtaattggaatgtattttcttaaaccacccataagaaccatattctgacttttatctggtgaatatccaacaagaggttccattga
atgaataacagatccggatcccaagaataataaagctttcgaataagcatgagtgatcaaatggaataaagcagcttgataagaacctatacctagagctaacatcat
ataacccaattgagacattgtagaataggctaagcttcttttaatatctctctgagcaagagctaaagtggctcctaagaaaagtgttagtgtacctattaaagaaatga
aactcattatcaaaggtagggatatgaaaagaggaagaagtcgagctataagaaaaatccccgcagcaaccatagttgctgcgtgtataagagctgaaatggggg
tgggtccttccatagcatcgggtaaccatacgtgaagagggaattgtgccgatttcgcaactgcaccaaggaataataaaaaagcacacaaaatagtaagcaagg
agttaatctcattattaggaatccagttattagctattttaaacaaatcccgaaactctaaactacctgttatccaaaaaaaaacctagaattcctaataacagaccaaaatc
ccctacacgattagttacaaaagctttttgacaagcactcgctgcaattggccgtgtaaaccaaaagcctatcaataaataggaacacattcccacaagctcccaaaa
aaaataaatttgtatcaaattggaactagtaaccaatcccaacatggaagtattgaaaaaacttatataaacaaaaaatctcaaatatccctcatcgtgagacatataatc
atcactataaataagaaccaggattcctacagtagtaattagtattaacataatagaagtaagcgggtcgattaagtatccaaattctaaggaaaaatcattattgacgg
tccaagaccatagatattgatagatagaacttccatttatttgttgaatagacaggtgaactgagaataccatagctatacttaaaagtaaaacactaggaaaagcccat
atgcgacgaagatttttttgttgctgttggaacaagaaaaagtccaaaccccattgacataataactggaagtgggagaagagggattacccatgcatattgatatgtat
gttccataagaaaagaaattgcaatttttacttgaaaattttacttcaatttttctataaaattgaaaaaagtttccgattcaccaaactaattcttatctatttctgaaggaata
aaaaaatactagaattcttaatttttcaaaaattttctcattgaaacaatcaaaaaataagaataggttttgttggttaaagtcaaaaagttaatgaaataacttcgttaccta
gttattacctaaagaaggacttttattaaaatacaaaaaaaagattgaatcattttactttaatattttttgtattaaaatgaagcagctcccttgtttcgtaactcaaattgatt
ggaattcaattctggaatactttaattaactatttgattgaattttcccttccttttatcctcccgtcttatatgggggataggcccccataccttatatctgtatatggagagta
tacttgaaatataaattgatttaaatagaaaacctttgtatatattctatattattaaaacaaagtctaaaaaaaatatagaatatgttaaaaaactcttgtcttatccgcattag
acaaaatgaagtaaaaaagaattcagaatttcaatatcttttagtatctaagtataaatactaagaaaaagaagaaagatggattgatttgcggcaatagatgtctttcac
atacaactagaaaaaagtaatttcctttttgaatggcagttccaaaaaaacgtacttcgatgtcaaaaaagcgtattcgtaaaaatctttggaagaaaaagacttatttttc
catagtacaatcttattctttagcaaaatcaagatcattttctggcgtcagcgagcatccaaaaccaaagggtttttctcggcaacaaacaaacaaataatagggttttg
ggataatatgaattgacctatccccaaaaaattccaattatttaatatgaataattaggaataattaggattaattaatgagtttacttttatgtgtcgaattcctcggtacaat
attcttagaacaaacctctctgatatataaaaaaagggtttttggtatactgtgacctaaatattcttttcctatcaatgaacttttcgtaatagaatccgtataataaataaaa
aaaggggattctattatgaaaagtagagtattcctgcaataagacttacaacttctacctatcttatcctaaattaacaaaaaaattagttctatattgcaactgagaaaaa

attgttccaactctttcaaagtttctattgggcaaagcaagaatttttttgttaaaaaattcgcaacgatactaccaaacgaagtctattttaatgaagattctaatgtcctaa
attctatggaatcttccaatctcgacgattcgcgagaaaataacttaatattcttttaataaacctattatttcaacttagccgccatggtgaaattggtagacacgctgctc
ttaggaagcagtgctcaagcatctcggttcgagtccgagtggcggcagtctcgaaaaagaatacaatagattataaaataaaatggattcaattcgaaatttccaattt
tgtaatgggaccttctccttatgctatttgcaactttagaacatatactaactcatatttctttctcaacaatttcaattgtgattacgattcatttaataaccttattagttcgtga
acttgggggattgcgtgattcgtcagaaaaaggaatgatagccactttttctgtataacaggattcttagtttctcgttgggcttcttcgggacattttccattaagtaattt
atatgagtcattgatcttcctttcatgggctctgtatattcttcatatgattcctaagatacagaactctaaaaatgatttaagcacaataactacgccgagtactattttaac
gcaaggctttgccacgtcgggtcttttaactgaaatgcatcaatccacaatactagtacctgctctacaatctcagtggttaatgatgcatgtcagtatgatgttactaag
ctatgcgactcttttgtgcggatccttattatccgccgctcttctaatgattagatttcgaaagaatttagatttcttttcgaaaaagaagaaaaatgttttgcttaaaacatttt
tctttaatgagattgaatatttctatgcaaaaagaagtgctttaaaaagcacctttttttcctttatttccaaattattacaaatatcaattaattgagcgtttggattcttggagtt
ctcgtgtcattagtctagggtttacccttttaaccataggtattctttgtggagcagtatgggctaatgaggcgtggggatcctactggaattgggatcctaaagaaactt
gggcatttattacttggaccatatttgcaatttatttacatagtagaacaaatccaaattggaagggtacgaagtcagcatttgtagcttccataggatttcttataatttgg
atctgttattttggtatcaatctattaggaataggtttacatagttatggttcatttacattacccatctaaatgattacataacataaaaccttaatgaaatggaaaaaacttc
catttttgtgtttgatttgagaaccccttgaacgccttctcaaagggttctcaaaaattcgagatagatctaattagactcttttactttttctgaatttttttagtatttccactat
ggaatatagagcggactagtagaagaaaaaaaatcctatttaggataataattggataacagagcctctaccctgtcaacggatagcgagagaacaaaatctggat
aaataccgattcctattactggtaaaaagatacagattaaaagaaagagttctcgcgggccggaatcctcaaaatttgcgtttggaacatgaaatagcttgtatccata
gaacatctgtcgtaacatagataataaataaataggagttaatatcattccaattgccattacaaaagtaattagcatttttggcattaacagaaattttggactagtaatg
agtccaaaaaatactactaattccgcaacaaaaccactcattcctggtaaggcaagagaagccattgaaaagctactaaacatggtaaaaatttttggcattgggata
gaaacccctcccagttcttcgagataaacaaggcgcattctatcacaagccgttcccgctaagaaaaaaagtgtagccccaataaatccatgggataatatttgtaaa
atagctccattgagtccaatgttggttatggaaccaattcctataataatgaaacccatgtgagagacggaggagtaggctattcttttttgaaattgcgttggccaaga
gaagttgaagctgcatagattatttgcatcgctcctattattactaaccaaggggaaaatagataatgagcatgaggtaacaattccatattgatccgaatcaatccgta
tgctcccatctttaataggattcccgctaaaagcatacatgtactgtaatgcgcttccccatgggtatctggtaaccacgtatgtaggggtataatcggcaatttgacag
cataagcaataaggaagccaaaataaaatagtatttccaatgttgcagggtatgattgattaattaatctttccaaatctaatcttggttcgttggaaccgtataagcccat
acctagaactccgattaagaaaaaaatggaaccacctgcagtatacaaaataaactttgtagctgaatagagacgcctcttccccccccacatggataaaagtaagt
aaacaggaattaattctaactcccacatgataaaaaaaagtaaaaggtctcgcgaagaaaataatcctatttgaccgctatacattgctagcatcaggaaatagaataa
tcgggaattccgggtaaccggccaagctgctaaagtagctaaagtagtcataaatcctgtcaataaaatagatcctaatgaaagtccatcgattcccaatctccagtg
gaaattgaagacatctatccatttagaatcctcttttaattggattaagggatcctccaattggaaatgataacagaatgcataagtcattagaaggaattctaataaaca
aatagacatagtataccacctaacgattttgtttcccctatgaggtaaaaagaaaattaatgaacccgcaaatatcggcaaaacaacaagtattgttaaccaaggaaaa
gaactcatgataaagtgataaagacaagatacgttttgaccagaaaagcccgtgctcgattattttgagcacaggcttcttcggtaaagaggaatcagacgattcaag
tggaatttttttgtaacgtatcaataagatagagccatgctgcgggttgtctcaggtcctaaataaacgcggacacttaaaaaatctgttgggcaggcggattcgcatct
cttacaacccacacaatcttcggttcttggcgcggaagcaatttgcttggctttacatccatcccaaggtatcatttctaatacatctgttggacaagctcgtacacattg
agtgcatcctatacatgtatcataaatttttacggaatgtgacattggatctataaattttccttttcaacataaaaattttcgatctggtcaaaatgaaattagtactatatca
atcaaatgtattgtagacaccagacgaagcaatggtttatccaaacttcaacaaataatgcaatatatttcttaatccgtttgtgagaaagcatgaaaagagccaagag
acttgaattttgggcttcaacaatcataattatacgaattgtatatacgaattcgaattagccaataaattggctatcgtcttttcaatataaattattgcaatattcaaattgc
aatatcaatgaattgcaaaaattcaactaagtaaaaaagaatactatggaataacctactcaaaaaatagatattctcaaataataaatagtattcatgttaatatttcatatt
attattatatgtgtccctttgtttagaagattctatgtctaattattcaaaaaattagattgattgatacgagttgatttcctattacgatggatggaagaaagaatggataatc
caatagctgcttcagcagccgcaagggctataacaaaaattgcgaaaatgtctccttttaattggcggctatcaaatagatcagaaaatgttacgagatttagattaatt
gaattcagtataagttcaaggcatattagagctctaaccatgtttcggcttgtgatcaatccatagataccaatcgaaaataaatagacactcaaaaaaagtacatgctc
aaacatcattaactaactccttatcaatctcgattcatttcaatatggggacaagaattgaaccgattgaattaattagaatagaacaattacacaacaaaagagaaaag
aaggtatttgttggcagtagatgggttttactaaatcaaaattgtggttctttagtgatttattttagatttgaaattcttataattttgactcattctaagtatttcttattgccgag
ccatagtaattgcacctattaaagaaactagaagaattatggaaatgagttcaaatggaagataaaaatcggttgctaaatgaatcccaatttgttgaacgttatttatga
gaccctgttctactatttggtttgatcttgtagtccaaagaattccataccatgacgtatctgggatagtagtcattagtgaaaaaggaatagttatacaaacgagtgaag
tgaacccatctccaatagtccaaaaattcttatctttagaccattctgagccatttacgaacattacggcaaatatgatcaagacatttatagctcccacataaataagaa
gttgtgccacagctacaaagtaggaattcaataaaatatagaataaggatatacaaacaagaactaatcctagcgaaaaagcagaaaaagttgggttggtaagtaat
accaccccctagacccccctagtagaagaacaaatcccccaaatagcacaagaatttcatgtattggcccaggtaaatccattatggataagaagaaattaatagtataa
attttttcatgaactgactaaaactaaaagattcaaggaagaaaaaagggattaggaaattttttttgtatattgtatataagttctttctatagttagaaatcacatcacgaaa
atctactctggtttaaaatcaggaataatttgcaataagcagtaggtattcgtttttctttctagttagtaaagaacttttggattctaacaaaaaaaattctagtaatcagtaat
cgttcttgaattccaagatttttcttcgtctattttactttgagttgaattcctaattgtttgaattgtgtaatctcccattatggagattggtaaccgactcaaagcaatttgatt
gtaattcaattcatgacgatcataagtagaaagttcatattcttcagtcattgataaacagtttgtcggacagtactcaacacaattaccacaaaatatacaaactccgaa
atcaatactataattaagcaattgtttccttttaatatccttttcaaatctccaatccacaagaggtagatctatagggcatacgcgaacacatacttcacaagcaatacatt
tatcaaattcaaagtggattcgcccccggaaacgctccgatgtaattgatttttcataggggtagtgaatcgttataggtaaacgatttgtgtgggataaggtaattatga
aactttgaccaatgtaccttgctgcgcgtattgtttgttgaccataactcatgaacccagttaccatagggaacatattcgaaatatctatgaaaaaggtatgtttctttctc
ttgtttgagagaacttttgtgttgaaaatattcttactgttattgtattatcttattttatagtgaaacaagttgggaagaagttgttaataagagattgcccagggaaataggt
aaaagaaatttccatccaagatttaataactgatccattctcatcctgggtaaagtccatcttattgtgatagaaatgaagagaaataaataagctttagttaatgtaataa
agatactcattgtcatttctagaattccaaccattttattcatttggaaaaatccaaaaaaggatatatagggaatagacaaattccacccgcctaagtagagaactgtta
caaataaagaggaaactaataaatttaggtaagaaacaagataaaataaaccatatttgataccagaatattcggtttgataacctgctactaattcttcctccgcttctg
gtaaatcaaagggtaatctttcacattccgccaaagaagaaattagaaaaaccagaaaacctataggctgacgccaaagattccatccaaaaaaaccatattttgact

gtgcttcaactatatcaactgtacttgaactgttggataatcatagtcgacgataacatcacagttcccaccgctattccaaaaccgtacatgaaaccttagtttcatacg
gctcctctatgatcagaaaaaaggaaagtactgtttcatttcgttattatcttcttgggcgtagttagaattatctaagataaaatcgatttcaacgtcctaaattagaccaa
aggaattctgtctgctagaataataaaaaacgcttcggaattcatctcatcctttataatataatggtactttttctttgttcagcaataacttaatcttggaataaaacactcg
ttataacaattaataaacgaaaagagttgggtattagttcatgaagaattctgtatgaatatggataaacgacggaaagaataaataaagatcttttttttgtattgcattc
catatcttttgtcctattcttctttccccgaggggtatttaaaaagaaaaaaggaataaagggttaattcgttcttgatagccatttccttaacaagtgaatgggaacatact
ctggatcggaatccgaagaaagtactacttgctcatttccaccaatttcaagtccttattatgattccttttatgaggaaaaatatctaatgctttagattccctcattactaat
cctttatgtactttagtgtttctaatccctcactaacttttgatggattcccttatgattacaactttctgtatcgggaatcccttattattgcccgcttcaagatatgatgactaa
tcaaaaaatctcaaccttggggtaaagaatttacaccgcttatgtttacttccatttttcttgtacataggaaatgagatttttttcttttactacaaattaataagcagttttgtt
tcactcatatagctatctagtttaacttactaacctgaatatagaataagaaaaggaggataaatattcaatgaatttcagaggaaaaagatcctattttaacgaatcgca
cgtagagatattgctagtacacaaaaagttaatgggatttcataactaatagattgagcggcagctcgtagaccacctgaaaaagaatatttattatttgagctatatcct
gccataagaagaccaataggagcaatacttgaaatggcaatccataaaaaaacaccaatactaagatccgctaaaacaaagcgatatcccaaaggaataactaaa
aaacttaataaaattgatatgactgctatagacggtccaatgctaaataagggaatatcccctcgggatggcaagatatcctctttaaaaagtagcttagttccatctgct
atagcttgaagcagtcccagggggccagcatattcaggaccaatacgttgttgtatcgatgcggatatttctctttctaaccacacaattacgagtacttctattgtgatt
cccagtaagagggtcaaaatgggtagaatccatatcagtccatagacttcttttaataattccaagttcgaaaaagaattgatagcttctacctgtaccctgtctattatca
tttcaacgatcaacttctcccataatgatatctatactacctaatatcgtcatgatatcagccaatttcatttttttgactagctgaggaagaatttgcaaattaataaaaccg
ggtggacgaattttccatctccaggggaaaagactatcatctcctaccagataaattcctaattcaccttttggggcttccactcttgcataaagctcttgttttgacaattc
aaaattgggtgaaggtttttaccaagaaatcgatattcaaaatcattccattcggaattctttgctttcttaaagcgtcggacttctaaattctcataagggcccccagga
attttttctacagcctgttgaataattttgattgattccctcatttcaccgattcgtactaaatagcgtgctaatgaatccccttctttttgccattggactttccaatcgaattga
ttgtaagactcgtaaagatcaactttacgaagatcccattgtattccagaagctcgtaacatggggcccgataagccccaatttacagcttcttctccgctaataaaacc
aactccctcaactcgttccaaaaaaatgggattctgtgtaataagttgttgatattcaataactcctcgtaaaaaataatcacagaaatctaaacatttatcgatccatcca
taaggcagatcggcagcaactcctccgatgcgaaagtaattatgcatcattcgcatacctgtagcagcttcaaatagatcatatattaattctctctctctaaaaatataa
aaaaaaggagtctgcgcaccgagatctgccataaaaggtccaagccataacaagtgagaagctatacggctcaattctaacataattaccctaatatagctggctctt
tggggtatttgaatattctccaagaattctggtgcatttaccgttattgcttctgtaaacatagtagctaaataatcccaacgtgttacataaggtaagtattgtataatagtt
cggttttccgcgatttttttccattcctctgtgtaaatagcctaatatgggttcacaatcaataacatcttcaccatcgagagtaacgatcagtcgaagaacaccatgcatt
gatgggtgctgagggcccatattgactatcatgagatcttttcttgtaagcggtagactcatatcctttcttccttaattcattattccatgaaaatggattattccatgaattc
ctcaaaacgaggctcatcaaaatgcaaaatctaagactactataagactactaataaaataagaaaaaaaaattcgaacgatgaaattaccgctccctaatatccaact
gactgattaatttcttataacgtactctatttttctttgccaaataagccagcaaacgttgacgttttcccaaaagtcttcggagacctctttccgatgaaaaatctttttttgtg
taattccaaatgtgaagcaagtctccgtatcttattggtgaaactgaatacttgaaattcaacagaaccccagttttcttctttttcttctttaaccataaatccaaaaattttttc
tacctcctttctttttcatgtatttttctgatcaggaaaaataaaaaaattatgtcagttattttgaagttattctaatctcgtacacacaaaaatttgcaattattcatctactactg
gaatttggatttattttatcgatgcaaattggatttggatagaagggtacattcttttattttagatagaagaaaagtttcttctatctaaaataaaagaattttgctgatttattt
attgctatatccaatttatggaattgatacaccgtttaattgatatcatttagcaaaatgaaacatagcatatgcatccatctttcggctcgagaatttcacgggatagagat
atggtataagaaataggctattaagtaactctaaatgaattgtggatacatctgtatccttaacatactgaaacaactgccattattcgtatcaaaccaatagcgattcata
caagttaaatcttctaatcaatggtgggccaataatgaattttttttgcatatgtattaagacgcttggcctgatttcgaaattgtccagagtttttttatgtagttttcattgcaaa
atgatggatctccttccgtaactttccaattacgagtacgagaattgaaagacatgaaaattctcaattctctacggcgtctaggagatagatagaatattttcaggaac
aagaaaaccagaagaatctttctctctattcactaccattccgcgtcttcgacttctattagtttcttttcttctttaatgcaatagctatagtttgatatagaatccatttctcaa
agtaatggaaacctttctcttataggaaatggttcgaaaatcgctattccaccttttaggtatcgtgaaaagtgatacctgtgaagatcgtgcatttcagtcacattcagat
ccgtttttttgagtccatgatataaccaaattggatggatcttccacccgtttagctaagaaagaatagatgcagaggtggataatagatcgatatgaagatcatgagct
gccccataatgaaaccaccaggagtcgcgaatatctccttcttccctaatccaagattggagaaagaagatctaagagggacccatggagaatgtggtcagaaacc
cataatagagtccgaccgcaacgaccgaattaattatcctcatcgagaaacttagactactaagtagaaaagatttgaaaatcatggcatgggtctcctttttttttctttctt
tagagttttctatatgcacaatttctcgatgtttcgatgagaatttcttgactttccatatatagaaagagatagactataaatgacatctcttatgtcaataagaccaaagg
gatggatattaaatgataggaagtgctaggaagtgaaatagaatgaaatagagccactctgggcttacctatgaaatgaggcatggaacggagccactacgaaga
aattccgggagttacgaaagaagcttcggactcatattgttcatgggttgagagcgggagttgaactctaggaggtcgaatcccccccttgttcctcagtagctcagtg
gtagagcggtcggctgttaactgactggtcgtaggttcgaatcctacttggggagatttgattcattctttaatgtaagaataaagaattgaattaaagggcttgctttga
cccttaggagtaggtaacccgttcgctatccttgtttctattgcattttatctcatcgtatcacattctgttctacgattccacttcgacaaaaggaaagagcatacccaagt
tcaatagctttacgtccgctatcccgatcatggttttcctaccctcagggggaaagtaaaggcccttcccctttggaaggctgtgggcgaggagggattcgaacccc
cgacaccgtggttcgtagccacgtgctctaatcctctgagctacaggcccaccccgtctccactggatctcttcccgggggtacccccaaaggaacctccctctc
ctcagccatttcatttcgggttaagaagatgggaaagcccctttctctctataagaacagtgcgttccgaggtgtgaagtgggagagaggggatgtgatgattgaggt
tttgaataagacgacctttgcattttggatttggatctttttcgtatttcaaaatagtgaaaaagtcaaataagaggtgttaagctttttatcattctggcatcgagctattttgc
cgcaggacctcccctacagtatcgtcaccgcagtagagtttaaccaccaaattcgggatggattggtgtggttcctctacgcctaggacaccagaatatcgaaccat
gaacgaggaaaggcatgagagaaatattggctagtaattgtgaagccccaattcttgactggaagggacaccaaaggcctctgccctccctctctatctatccaaga
gatggaagggcagagcttttttttggtttttttcatcttttcatcaaagagttgaacaatgaagatagatggcaagtgcctgatcgatttgatcaggtcgtgtaggaacaag
gttcaaatcgttcgttcgttaggatgcctcagctgcatacatcactgcacttccacttgacacctatttaaacggctcgtctcgccgctaccttatcctatttccatgcttct
gtcgctccatccccgtatgggtggagaacccgtcgctgtctcggctgtgctaccggaggctctagggaagtcggaggagagagcactcatcttggggtgggctta
ctacttatatgctttcagcagttatcctctccgcacttggctacccagcgtttaccgtaggcacgataactggtacaccagaggtgcgtccttcccggtcctctcgtact
agggaaaggtcctctcaatgctctaacgcccacaccggatatggaccgaactgtctcacgacgttctgaacccagctcacgtaccgcattaatgggcgaacagcc
caacccttggaaccacctacagctccaggtggcgaagagccgacatcgaggtgccaaaccttcccgtcgatgtggactcttggggaagatcagcctgttatcccta

gagtaacttttatccgttgagcgacggcccttccactcggcaccgtcggatcactaaggccgactttcgtctctgctcgacgggtgagtcttgcagtcaagctcccttc
tgcctttgcactcgaggaccaatgtccgtctggcccgaggaaacctttgcacgcctccgttaccttttgggaggcctacgccccatagaaactgtctacctgagactg
tcccttggcccgcgggtctgacacaaggttagaatccgagctcttccagagtggtatctcactgatggctcgggccccccggaagggggccttcttcgccttcca
cctaagctgcgcaggaaaggcccaaagccaatcccagggaacagtaaagcttcatagggtctttctgtccaggtgcaggtagtccgcatcttcacagacatgtcta
tttcaccgagtctctctccgagacagtgcccagatcgttacgcctttcgtgcgggtcggaacttacccgacaaggaatttcgctaccttaggaccgttatagttacggc
cgccgttcaccggggcttcggtcgccggcttccctgtcatcagttcaccaacttccttgaccttccggcactgggcaggcgtcagcccccatacatggtcttacgact
ttgcggagacctgtgtttttggtaaacagtcgcccgggcctggtcactgcgacccccttttgcgagggggcaccccttctcccgaagttacggggctattttgccga
gttccttagagagagttgtctcgcgcccctaggtattctctacctacccacctgtgtcggtttcgggtacaggtacccctttgttgaaggtcgttcgagcttttcctggga
gtatggcatgggttacatacttcagcgccgtagcgcctggtatgagcctcgtggagaagcaatggctagtccacggggctcatacttcagcgctgcagcgcttggt
actcggacctcggctcgaggcattttctctaccccttcttaccctgaaaaagcagggtcaccttgtgtccttaaacctataaccatctttcggctaacctagcctcctcc
gtccctccgtaccaacaaggggtagtacaggaatattgacctgttgtccatcgactacgcctttcggcctgatcttaggccctgactcaccctccgtggacgaacctt
gcggaggaaaccttgggttttcggggcattggattctcaccaatgttttcgttactcaagccgacattctcgcttccgcttcgtcgacccccgctttcgcggttgcttcc
ctctaaggcggaacgctcccctaccgatgcattttgacatcccacagcttcggcagatcgcttagccccgttcatcttcagcgcaagggcgctcgatcagtgagcta
ttacgcactctttaaagggtggctgcttctaggcaaacctcctggctgtctttgcacccccacctcctttatcactgagcggtcatttaggggccttagctggtgatccg
ggctgtttccctctcgacgatgaagcttatcccccatcgtctcactggccgaccttgaccccctgttatttttgggtcatatctagtattcagagtttgcctcgatttggtacc
gctcgcgcagcccgcaccgaaacagtgctttaccccctagatgtccagtcaactgctgcgcctcaacgcatttcggggagaaccagctagctctgggttcgagtgg
catttcaccccctaaccacaactcatccgctgattcttcaacatcagtcggttcggacctctgcttagtttcatccaagcttcatcctggtcatggatagatcacccaggtt
cgggtccataagcagtgacaatcgccctatgaagactcgctttcgctacggctccggtgggttccgttcccttaaccaagccactgcctatgagtcgccggctcattc
ttcaacaggcacgcggtcagagatcactttcccctcccactgcttgggagctcagcacggtttcacgttctatttcactacccactggggggttcttttcacctttccctca
cggtactacttcgctatcggtcacccaggagtatttagccttgcaaggtggtccttgctgattcacacgggattccacgtgccccatgctactcgggtcagagcgtaa
gctagtgatgctttcggctactggactttagccatctagggtgcggcactcaaccgcttcgcctagcagcacaacgcttgtattgctctcccacaaccccgttttcacg
gtttaggctgctcccatttcgctcgccgctactacgggaatcgcttttgctttcttttcctctggctactaagatgtttcagttcgccaggttgtctcttgcctgctcatggat
tcagcaggcagttcaaaaggttgacctatttgggaatctccggatctatgcttattttcaactccccgaagcatttcgtcgcttgctacgcccttcctcgtctctgggtac
ctaggtatccaccgcaagcctttcctcttttgaacctcgccattaacgttaaggctatgccatcctaaggtgctactaaatggaaggatcttatcaacgtccatgaatgc
gaaatcatagatcgaactgccgaattggcaaaattcggtgctatcatagtatccgctaagttcacgggctggagataagcggactcgaaccgctgacatccgccac
agggtaaaccaccgcctctcaggcctccccgacgggttctaccatagaggccaacgatagacaataactccccccgaacacagcttacaactttcatcgtactgt
gctctccaaagagcaactcttctcaaaatctcaaaacaaaaggtgctgagttggaatcccattctaaggattcttgtggttccggggaatccagctacaggagaacca
ggaacggggagctctccccttttccgcccgactctttgatcttaaacttaagaatgctggttttaagaacgagtgattgcccttctccgacccttactgcccaaccgga
gagcggacggctaatgtgttccacttattgaacagggtctatggtcggtccgtgaccccctggacgccgaaggcgtccttggggtgatctcgtagttcctacggggtg
gagataatggggtcggtccatggattttccttccttttgccacatttcgctcaaagggttgaagggagatagtgcatcaagctattcgcaagggccaacttgatcctctt
ccccagggatcccagatgagggaagcctaggagagccgccgactccaactatcgtccatgtacgatccatactagatctgaccaactgcccatcctacctcctcta
cctttttgacagcccatctttttgtctcagtagagtctttcagtggcatgtttcagtcctcttccccattacttagaaaaagtgagccaccggttcaggtacaagatactac
cattaccgcctggacaattagacagccaacccgtaatcgcaacgacccaattgcaagagcggagctctaccaactgagctatatcccccgagccaagtggagta
tgcatgaaagagtcagatgcttcttctattcttttccctggcgcagctgggccatcctggacttgaaccagagacctcgcccgtgaagtaaatcatcgcccctacgatc
caaccaattgggagagaatcaatagactccttttcgggagcgattcatccttcccgaacgcagcatacaactctccgttgtactgcgctcttcaagtgtgcttcttcccc
ttctccccttaccatggcaagtccttgggaaataactccgatgggcagaaaaggaaggcgttaagagaccctcctggcccaaccctagacactctaagatcctttt
ttcaaacctgctctgctcccatttcgagtcaagagatagataaatagccacatcccattgcactgatcgagggcgctcgtagtgacttagggagtcgaagaccaaga
agtggcttatttataccaagcattcctcttatggctagatccaacctcctggtccctgcggaaaggaaaaagaatttcacgttcttcctttcaggaagggaggattagg
gaagtcctattgattgctgctttctccagaccgccgggaaaagcatgaaaaaaaggctcgaatggtacgatccctccgtcaccccagaatgaaaggggtgatctcg
tagttcttggtctgtgaagatgcgttgttaggtgctccattttcccattgaggacgaacctcaacctgtgctcgagagatagctctccatacactgataagggatgtatg
gattctcgagaagagaggagccgtagtgccccccccggaccgcccggatcccacgagtgaatagaaagttagatctacatgggatctcacctgaatcgccccat
ctatcctcctgaggagaagtttgtttggtttcaaactccgattcaaacaggaggagtacgccatgctaatgtgccttggatgatccacatcttcgggtcaggcgctgat
gagcacattgaactatccatgtggctgagagccctcacagcccaggcacaacgacgcaattatcaggggcgcgctctaccactgagctaatagcccgtcgcgcg
ggcctccctttgggaggcctgctacgccaaaagcgagaaaaactccatccctttccttttgacatccccatgccgccacaccacacgggggggggcatggggac
gtcaaaaaggggatcctatcactatcaactaatttgttccgacctaggataataagctcatgagcttggtcttacttcaccctaaacgaaagaagacttccatatccaag
tttagctcagacgtagctgccttctttttgggcgtgaagcagtgtcaaaccaaaatacccaataagcataagcattagctctccctgaaaaggaggtgatccagccgc
accttccagtacggctaccttgttacgacttcactccagtcgcaagcctagccttaggcatccccctccttacggttaagggtaatgacttcaaacatggccagctcct
atagtgtgacgggcggtgtgtacaaggcccgggaacggattcaccgccgtatggctgaccggcgattactagcgattcctgcttcatgcaggcgagttgcagcct
gcaatccgaactgaggacgggtttttggagttagctcaccctcgcgagatcgcgaccctttgtcccgcccattgtagcacgtgtgtcgcccagggcataaggggca
tgatgacttggcctcatcctctccttcctccggcttaacaccggcggtctgttcagggttccaaactcatagtggcaactaaacacgagggttgcgctcgttgcgaga
cttaacccaacaccttacggcacgagctgacgacagccatgcaccacctgtgtccgcgttcccgagggcacccctctctttcaagaggattcgcggcatgtcaagc
cctggtaaggttcttcgctttgcatcgaattaaaccacatgctccaccgcttgtgcgggcccccgtcaattcctttgagtttcattcttgcgaacgtactccccaggcgg
gatacttaacgcgttagctacagcactgcacgggtcgagtcgcacagcacctagtatccatcgtttacggctaggactactggggtctctaatcccatttgctcccta
gctttcgtctctcagtgtcagtgtcggcccagcagagtgctttcgccgttggtgttctttccgatctcaatgcatttcaccgctccaccggaaattccctctgcccctacc
gtactccagcttggtagtttccaccgcctgtccagggttgagccctgggatttgacggcggacttgaaaagccacctacagacgctttacgcccaatcattccggat
aacgcttgcatcctctgtcttaccgcggctgctggcacagagttagccgatgcttattcctcagataccgtcattgtttcttctccgagaaaagaagttgacgacccgtg

ggccttccacctccacgcggcattgctccgtcaggctttcgcccattgcggaaaattccccactgctgcctcccgtaggagtctgggccgtgtctcagtcccagtgt
ggctgatcatcctctcggaccagctactgatcatcgccttggtaagctattgcctcaccaactagctaatcagacgcgagcccctccttgggcggatttctccttttgct
cctcagcctacggggtattagcaaccgtttccagttgttgttcccctcccaagggcaggttcttacgcgttactcacccgttcgccactggaaacaccacttcccgttc
gacttgcatgtgttaagcatgccgccagcgttcatcctgagccaggatcgaactctccatgagattcatagttgcattacttatagcttccttattcgtagacaaagcgg
attcggaattgtctttccttccaaggataacttgtatccatgcgcttcagattattagcctggagttcgccaccagcagtatagccaaccctaccctatcacgtcaatccc
acaagcctcttatccattcccgttcgatcgtggcgggggggagtaagtcaaaatagaaaaaactcacattgggtttagggataatcaggctcgaactgatgacttccac
cacgtcaaggtgacactctaccgctgagttatatcccttccccgtcccctcgagaaagagaattaacgaatcctaaggcaaaggggcgagaaactcaaggccacc
cttcctccgggctttctttccacactattatggatagtcaaataatgggaaaaattggattcaattgtcaaccggtcctatcgaaaataggattgactatggattcgagcc
atagcacatggtttcataaaatctgtacgattttcccgatctaaatcgagcaggtttccatgaagaagatcttgttcagcatgttctattcgatactggtaggagaagaac
ccgactcggtattcttaaaaaaagaggggaagcagaaccaagtcaagatgatatgggtcgccccttcttcttgcgccaaagatcttaccatttccgaaggaactggg
gctacatttcttttcaatttccattccagagtttctatctgtttccacgcccttttttgagacctcgaaacatgaaatggacaaattccttctcttaggaacacatacaagaaa
aaggataatggtagccctcccattaactacttcattttcatttatgaatttcatagtaatagaaatccatgtcctaccgagacagaatttcgaacttgctatcctcttgccta
ataggcaaagattgacctctgtagaaagactgattcattcggatcgatatgaggacccaactacgttgcattgccagaatccatgttccatatttgaagagggttgacc
tctgtgcttctctcatggtacaatcctcttcctgctgagccccctttctcctcggtccacagagaaaaaatggaggactggtgccgacagttcatcacggaagaaaga
actcacagagccgggatcgctaactaatagaatagtactactaactaatactaatatatagaaatagatatctagaaatagaaacgaactaatatatagataatcgaaa
ttgaaaagaactgtcttttctgtatactttccccgttctattgctaccgcgggtcttatgcaatcgatcggatcatatagatatcccttcaacacaacataggtcatcgaaa
ggatctcggacgactcaccaaagcacgaaagccagttagaaaatggattcctatttgaagagtgcctaaccgcatggataagctcacattaacccgtcaattttggat
ccaattcgggatttttcttgggaagtttcgggaagaaattggaatggaataatatagattcatacagaggaaaaggttctctattgatgcaaacgctgtacctagaggat
agggatagaggaagagggaaaaatcgaaatgaaataaataaagaataaagcaaaaaaaaaaataagtcgaagatagaagagcccagattccaaatgaagaaatg
gaaactcgaaaaggatccttctgattctcaaagaatgaggggcaaggggattgataccgagaaagatttcttcttattataagacgtgatttgatccgcatatgtttggt
aaaagaacaatcttctcctttaatcataaatggaaagtgttcaattagaacatgaaaacgtgactcaattggtcttagttagtcttcgggacggagtggaagaagggcg
gagactctcgaacgaggaaaaggatcccttcgaaagaattgaacgaggagccgtatgaggtgaaaatctcatgtacggttctgtagagggacaggaagggtgac
ttatctgtcgacttttccactatcaaccccaaaaaacccaactctgccttacgtaaagttgccagagtacgattaacctctggatttgaaatcactgcttatatacctggta
ttggccataatttacaagaacattctgtagtattagtaagaggaggaagggttaaggatttacccggtgtgagatatcgcattattcgaggagccctagatgctgtcgc
agtaaagaatcgtcaacaagggcgttctagtgcgttgtagattcttatccaagacttgtatcatttgatgatgccatgtgaatcgctagaaacatgtgaagtgtatggct
aacccaataacgaaagtttcgtaaggggactggagcaggctaccatgagacaaaagatcttctttctaaagagattcgattcggaactcttatatgtccaaggtcaat
atggaaattctttcaggggttttcccttactttgtccgtgtcaacaaacaattcgaaatacctcgactttttcagaacaggtccgagtcaaatagcaatgattcgaagcac
ttctttttccattacactatttcggaaacctaaggacttgatggtatggatatggaaaatacaggatttccgatcctagcgggaaaaggagggaaacggatactcaattt
taagtgagtaaactgaattccatactcgatctcatagatccctatagaattctgtggaaagccgtattcgatgaaagttgtatgtacggcttggagggagatctttcctat
ctttcgagatccaccctacaatatggggtcaaaaagccaaaaaaataagtgattcgtttttagcccttataaaaagaaaacggattcttgaacctctttcacgctcatgtc
acgtcgaggtactgcagaaaaaagaactgcaaaatccgatccaatttttcgtaatcgattagttaacatggtggttaaccgtattatgaaagacggaaaaaaatcattg
gcttatcaaattctctatcgagccgtgaaaaagattcaacaaaagacagaaacaaatccactattggttttacgtcaagcaatacgtagagtaactcccaatataggag
taaaaacaagacgtaataaaaaaggatcgacgcggaaagttccgattgaaataggatctaaacaaggaagagcacttgccattcgttggttattagaagcatccca
aaagcgtccgggtcgaaatatggctttcaaattaagttccgaattagtagatgctgccaaagggggtgggggtgccatacgcaaaaaggaagcgactcatagaat
ggcagaggcaaatagagctcttgcacattttcgttaatccatgaacagaatctaggtatgtagacacatggatccatacatctcgatcggaaaagaatcaatagaag
gagaatcggacgatatctttctcgaaacaaacaaaaaggaaaagaaagagaaaacagaaatcatgatcaactaagccctctcgggggcttgcttaagaataagaa
agaggaatcttatggaaatagcatggaataaggtttgatcctattcatggggattccgtaaatatcccattccaaaaatcgaaacaatcgggacttttcggagattggat
gcagttactaattcatgatctggcatgtacagaatgaaaacttcattctcgattctacgagaatttttatgaaagcgtttcatttgcttctcttccagggaagtttcattttccc
agaatgtatcctaatttttggcctaattcttcttctgatgatcgatttaacctctgatcaaaaagatagaccttggttctatttcatctcttcaacaagtttagtaataagcataa
cggccctattgttccgatggagagaagaacctataattagcttttcgggaaatttccaaacgaacaatttcaacgaaatctttcaatttcttattttattatgttcaactttatg
tattcctctatccgtagagtacattgaatgtacagaaatggctataacagagtttctgttattcgtattaacagctactctaggggggaatgtttttatgtggtgctaacgattt
aataactatctttgtagctccagaatgtttcagtttatgttcctacctattgtctggatataccaagagagatctacggtctaatgaggctactatgaaatatttactcatgg
gtggggcaagctcttctattctggttcatggtttctcttggctatatggttcatctgggggggagatcgagcttcaagaaattgtgaacggtcttatcaatacacaaatgt
ataactccccaggaatttcaattgcgcttatatccatcactgtaggacttgggttcaagctttccccagccccttttcatcaatggactcctgacgtctacgaaggagtgt
ggttcgttcgacaaattcctacctctatatctatctctgaggtgtttgggttttgcaaaactccatagatatgcagaagagaaatgctatccccactccgaccaagacag
aacttttaccaaaagtttattgtgatctttttgttcaaataacaattaaggtgaagcagggtcaggaacaacgaatctctttatgataaacagatccattttgcaagctcgtt
attacgggtagttcctacaaagaatcggactaatgacgtatacaatgcttgaattatcgacgtagatgctacatagtgggttctcatccttcagagactacgagtgtaat
aggagcatccgttgacaaaaggatcaccctaagatgatcatctcatggctattgggaacgaatcaaatcagatggttctatttctcaacctttctgacttgctcctacgg
aaccaaggtcgaaaggattgaaaaagtcagtcattcacaaccactgatgaaggattcctcgaaaagttaaggattagtagttctttttcgaaatcgatttcgaaaaaga
atggattcggtcttatacatacgcgaggaaggtaatcaaaaaagaaagaagacaagttcttctttcttttatcacttaggagccgtgcgagatgaaagtctcatgcacg
gttttgcatgagagaaagaagcgaggaatcctcttttcgactctgactcccccactccagtcgttgcttttctttctgttacttcgaaagttgctgcttcagcttcagccac
gcgaattctcgatattcctttttatttctcatcaaacgaatggcatcttcttctggaaatcctagctattcttagcatgatattggggaatctccttgctattactcaaacaagc
atgaaacgtatgcttgcatattcgtccatagggcaaatcggatatgtaattattggaataattgttggagactcaaatgatggatatgcaagcatgataacttatatgcttt
tctatatctccatgaatctaggaacttttgcttgcattgtattatttggtctacgtaccggaactgataacattcgagattatgcaggattatacacgaaagatcctttttgg
ctctctctttagccctatgtctcttatccctaggaggccttcctccactagcaggtttcttcggaaaactctatctattctggtgtggatggcaagcaggcctatatttcttg
gtttcaataggactccttacgagcgttctttctatctactattatctaaaaatagtcaagttattaatgactggacgaaaccaagaaataaccccttatgtgcgaaattata

gaagatcccctttaagatcaaacaattccatcgaattgagtatgactgtatgtgtgatagcatctactataccaggaatatcaatgaaccccattcttgcaattgctcagg
ataccctcttttagctgctaggtctatttcttagttcaagatccctcttactaactggaataaaagaattagtagatctgttccgcccaaaatgggaatgggcgctagggt
aatgaacttataatcatggaatcgactcgatcatcagattataagttcattccataccggaccagaccgtgcacattcttattatgagaaggggtcattcgagcctatgg
aaataggatactctgtttacatagaaatccccacgtccttacattctatttaggattaggaataggtgtaatcagacctgcttttgacatatctatcctatccttatttgggta
ccatatgcacctctttgggcttctattgaatcgagaaattggattgtacatctttttgattttgatacatataaggtgtcctacggataatgcaaatcgaagctatttgatgtc
tgactcaggcctatatgaccgatcgatcgaaatactccaagactccacctttgtcatatattccatatatcacattagatagatatcatattcatggaatacgattcactttc
aagatatcacattagatagatatcatattcatggaatatgattcactttcaagatgccttgatggtgaaatggtagacacgcgagactcaaaatctcgtgctaaagagc
gtggaggttcgagtcctcttcaaggcataatacggagaatgcgcattcaatgagcattccccgtagaagtattccggaaatctgcgcctggcgctctcctctatcttct
gaggtccttaaccacttccctgagaaaaggagacagtaaaagccaaaatagactaaatatagcctgaacgatcctaaaaatccctcgaaggagataatataataaa
gaacccaaagcagacggtatcctactgcaagggtggtcttaagaatccaaaagaggttgctcagaagagatagatgtatcccaacctctattgctctcgcgtaaagc
ctttttttacgcgacaggaaaaagtgactacgaattccccttttttgtttgcgaatccctgtttgtatcctttgagcgcacgcccataagtagcgatcaaggaaatcgatc
aaacgatcccaataccgtgaaagagaaacttcccagatccagggaagcttatcataaagggcttcgacaaggggttttattcgttctttgagcaaaaagataaagatc
ggatagattcgaaccgcaattgcaaaggtaatcactactatgccagcccaaatcatgatcttcaccaacttggatttggttctctcgcgaaaatcgcgagttgcagag
atgagaaccatgaaaagcaagatcccgaataagaaaacagaaaccgaggaaaccacaagagtgaagactagtagagtctcgtcttttgtcattctttgctcctttttc
actcaatgattcattcgaatttcccgaccaaaaattctatatgtctattcatagggcctcgttgctaagtgctacaagatctagtgcactggaactcgtggttatggaccc
gaatcctttagtatggaacattgtcttttccaagtaaaaacccccagtatatgaaagaatgaaaaggtgctttcgttcttttcttgtggaataagaagccctcgtaccttaa
tgaaaggaaaataggaatttttcattaggtatttgaccaaataggatcgtccagttcctatagaacctatcactaaaatacccgatagggctaagcggaacgaaaagg
attttccctgagatggtaaatgaaaacgattagccccatacgaggtttgggaataagtgatgagcaaggaatatacgtctttctgctaaagaggatctattaaactcata
attcattagatccttgttatcaatgtcaactaggtatcataagtaaacggatcccggttgttcaatcctttgataaccaaggtcattctttgctaaagagaaatgatcactat
gagtcagactcaatagaattggatccattccaaatagcgagaattaggattctggatccctctcaatctctctttcaattcgaggatccagagaggtgttttcatagtcat
ctccgaatatttgccatctccgaatattttcgatttcatttttctatgatatgtctttctatatgaaaattggttatttacgatgtacgatgatccctgttaagcatccatggctga
atggttaaagcgcccaactcataattggtaaatttgcgggttcaattcctgctggatgcacgcgaaccggaacgttccataagtctattggaactggctctctatccatg
gaatctcatccatcatccatacataacgaattggtatggtatattcataccataacataagaacaataagaactcgaattcttatcgatactggaactcagagcatagga
gggaaagtcgatttatggatggaatcaaatacgcagtatttacagaaaaaagtcttcgtttattgggaaagaatcaatatacttttaatgtcgaatcgggattcactaag
acagaaataaagcattgggtcgaactcttctttggtgttaaggtagtagctgtgaatagccatcgactacccggaaagggtagaagaatgggccctattctaggacat
acaatgcattacagacgtatgatcattacccttcaaccgggttattctattccacttctagatagagaaaaaaactaaaggagaatacttaataatacggcgaaacattt
atacaaaacacctatcccgagcacacgcaagggaaccatagataggcaagtgaaatccaatccacgaaataatttgatccatggacggcaccgttgtggtaaagg
tcgtaattccagaggaatcattaccgcaaggcatagagggggaggtcataagcgcctataccgtaaaatcgattttcgacggaatcaaaaagacatatctggtaga
atcgtaaccatagaatacgaccctaatcgaaatgcgtacatttgtctcatacactatggggatggtgagaagggatatattttacatcccagaggggctataattggag
atactattgtttctggtacaaaagttcctatatcaatgggaaatgccctacctttgagtgcggtttgaactattgatttacgtaattggaagtaaccaattaggtttacgacg
aaacctagaaatcgatcactgatccaatttgactacctctacgggatagacctcaacagaaaactgttgagtaacggcagcaagtgattgagttcagtagttcctcata
gaaaattattgactctagagatatggtaatatggagaagacaaaattgtttgaagcacgcacagaaccggaagcgccccttgtttcaaagagaggaggacgggtta
ttcacatttaatttgatggtcagaggcgaattgaaagctaagcagtggtaattaagacccccgggggaaaatagggatgtctcctacgttacccataatatgtagaagt
atcgacgtaatttcatagagtcattcgatctgaatgctacatgaagaacataagccagatgacggaacgcggagacctaggatgtagaagatcataacatgagcga
ttcggcagatttggattcctttcctatatatccactcatgtggtacttcatcatacgattcatataagatccatctgtctagagatcgtcatatacatctagaaagccgtatg
ctttggaagaagcttgtacagtttgggaaggggttttttgagagaaaagaagaatctacttcaaccgatatgcccttaggcacggccatacataacatagaaatcaca
cgtggaaggggtgggcaattagctagagcagcaggtgctgtagcgaaactgattgcaaaagaaggtaaatcggccactttaagattaccatctggggaggtccgt
ttggtatcccaaaactgcttagcaacagtcggacaagtgggtaatgttggggtgaaccaaaaaagtttgggtagagccgggtctaagtgttggctaggtaaacgcc
ccgtagtaagaggggtagttatgaaccctgtggaccacccccatgggggcggtgaagggaaagcccccattggtagaaaaaaacccacaaccccttggggttat
cctgcgcttggaagaagaactaggaaaaggaaaaaatatagtgatagttttattcttcgtcgccgtaagtaaatacgtaactaggaatatggaaaattgcatttttggaa
tttgcaataatgggatgggcgaacgacgggaattgaacccgcgcatggtggattcacaatccactgccttgatccacttggctacatccgccccttatccagctaaa
ggattttctctttttccattcatcattattctatttattctgacctccatacttcgatcgagatattggacatcgaatgccactctttaaaatggaaaaaaaggagtaatcagc
tgtgacacgaaaaaaaacgaatccttttgtagctcatcatttattggcaaaaatagaaaaggtcaatatgaaggaggagaaagaaacaatagtaacgtggtcccggg
catctagcattctacccgcaatggttggccatacaatcgcgattcataatggaaaggaacatatacctatttacataacaaatcctatggtaggtcgcaaattggggga
attcgtaccaactcggcatttcacgagttatgaaagtgcaagaaaggatactaaatctcgtcgttaactgaattcagaatagaaagattcaaaataaaaaaaaaagaa
ata

Supplementary Dataset S2.

The 45S transcription unit sequences of seven *Oryza* species. WGS sequences of these seven accessions were retrieved from GenBank (Table 1) and the 45S sequence was obtained using the dnaLCW method. The header of each FASTA sequence indicates species name and accession number.

>O.rufipogon_AC11-1008369_45S
ACCTGGTTGATCCTGCCAGTAGTCATATGCTTGTCTCAAAGATTAAGCCATGCATGTGCAAGTATGAA
CTAATTCGAACTGTGAAACTGCGAATGGCTCATTAAATCAGTTATAGTTTGTTTGATGGTACGTGCTA
CTCGGATAACCGTAGTAATTCTAGAGCTAATACGTGCAACAAACCCCGACTTCCGGGAGGGGCGCAT
TTATTAGATAAAAGGCTGACGTGGGCTCTGCCCGCTGATCCGATGATTCATGATAACTCGACGGATCG
CACGGCCCTCGTGCCGGCGACGCATCATTCAAATTTCTGCCCTATCAACTTTCGATGGTAGGATAGG
GGCCTACCATGGTGGTGACGGGTGACGGAGAATTAGGGTTCGATTCCGGAGAGGGAGCCTGAGAA
ACGGCTACCACATCCAAGGAAGGCAGCAGGCGCGCAAATTACCCAATCCTGACACGGGGAGGTAG
TGACAATAAATAACAATACCGGGCGCTTTAGTGTCTGGTAATTGGAATGAGTACAATCTAAATCCCTT
AACGAGGATCCATTGGAGGGCAAGTCTGGTGCCAGCAGCCGCGGTAATTCCAGCTCCAATAGCGTA
TATTTAAGTTGTTGCAGTTAAAAAGCTCGTAGTTGGACCTTGGGCCGGGTCGGCCGGTCCGCCTCAC
GGCGAGCACCGACCTACTCGACCCTTCTGCCGGCGATGCGCTCCTGGCCTTAACTGGCCGGGTCGT
GCCTCCGGCGCCGTTACTTTGAAGAAATTAGAGTGCTCAAAGCAAGCCATCGCTCTGGATACATTAG
CATGGGATAACATCATAGGATTCCGGTCCTATTGTGTTGGCCTTCGGGATCGGAGTAATGATTAATAG
GGACAGTCGGGGGCATTCGTATTTCATAGTCAGAGGTGAAATTCTTGGATTTATGAAAGACGAACAA
CTGCGAAAGCATTTGCCAAGGATGTTTTCATTAATCAAGAACGAAAGTTGGGGGCTCGAAGACGAT
CAGATACCGTCCTAGTCTCAACCATAAACGATGCCGACCAGGGATCGGCGGATGTTGCTTATAGGAC
TCCGCCGGCACCTTATGAGAAATCAAAGTCTTTGGGTTCCGGGGGGAGTATGGTCGCAAGGCTGAA
ACTTAAAGGAATTGACGGAAGGGCACCACCAGGCGTGGAGCCTGCGGCTTAATTTGACTCAACACG
GGGAAACTTACCAGGTCCAGACATAGCAAGGATTGACAGACTGAGAGCTCTTTCTTGATTCTATGGG
TGGTGGTGCATGGCCGTTCTTAGTTGGTGGAGCGATTTGTCTGGTTAATTCCGTTAACGAACGAGAC
CTCAGCCTGCTAACTAGCTATGCGGAGCCATCCCTCCGCAGCTAGCTTCTTAGAGGGACTATGGCCG
TTTAGGCCACGGAAGTTTGAGGCAATAACAGGTCTGTGATGCCCTTAGATGTTCTGGGCCGCACGCG
CGCTACACTGATGTATTCAACGAGTATATAGCCTTGGCCGACAGGCCCGGGTAATCTTGGGAAATTTC
ATCGTGATGGGGATAGATCATTGCAATTGTTGGTCTTCAACGAGGAATGCCTAGTAAGCGCGAGTCA
TCAGCTCGCGTTGACTACGTCCCTGCCCTTTGTACACACCGCCCGTCGCTCCTACCGATTGAATGGT
CCGGTGAAGTGTTCGGATCGCGGCGACGGGGGCGGTTCGCCGCCCCCGACGTCGCGAGAAGTCCA
TTGAACCTTATCATTTAGAGGAAGGAGAAGTCGTAACAAGGTTTCCGTAGGTGAACCTGCGGAAGG
ATCATTGTCGTGACCCTGACCAAAACAGACCGCGAACGCGTCACCCCTGCCCGCCGAGCGCTCGCG
CGCGAGGCAACCGAGGCCCCCGGGCCGCAACAGAACCCACGGCGCCGACGGCGTCAAGGAACAC
AGCGATACGCCCGCGCCGCCCGGTCGGCCCTGGCCGTCCGGCGGCGCGGCGCGATACCACGAGTTA
AATCCACACGACTCTCGGCAATGGATATCTCGGCTCTCGCATCGATGAAGAACGTAGCGAAATGCGA
TACCTGGTGTGAATTGCAGAATCCCGTGAACCATCGAGTCTTTGAACGCAAGTTGCGCCCGAGGCC
ATCCGGCCGAGGGCACGCCTGCCTGGGCGTCACGCCAAAAGACGCTCCACGCGCCCCCCCTATCCG
GGAGGGCGCGGGGACGCGGTGTCTGGCCTCCCGCGCCTCGCGGCGCGGTGGGCCGAAGCTCGGGC
TGCCGGCGAAGCGTGCCGGGCACAGCGCATGGTGGACAGCTCACGCTGGCTCTAGGCCGCAGTGC
ACCCCGGCGCGCGGCCGGCGCGATGGCCCCTCAGGACCCAAACGCACCGAGAGCGAACGCCTCGG
ACCGCGACCCCAGGTCAGGCGGGACTACCCGCTGAGTTTAAGCATATAAATAAGCGGAGGAGAAGA
AACTTACGAGGATTCCCCTAGTAACGGCGAGCGAACCGGGAGATGCCCAGCTTGAGAATCGGGCGG
CCGCGCCGCCCGAATTGTAGTCTGGAGAGGCGTCCTCAGCGACGGACCGGGCCCAAGTCCCCTGG
AAAGGGGCGCCTGGGAGGGTGAGAGCCCCGTCCGGCCCGGACCCTGTCGCCCCACGAGGCGCCGT
CAACGAGTCGGGTTGTTTGGGAATGCAGCCCAAATCGGGCGGTAAACTCCGTCCAAGGCTAAATAC
AGGCGAGAGACCGATAGCGAACAAGTACCGCGAGGGAAAGATGAAAAGGACTTTGAAAAGAGAG
TCAAAGAGTGCTTGAAATTGCCGGGAGGGAAGCGGATGGGGGCCGGCGATGCGCCCCGGCCGTAT
GCGGAACGGCTTCGGCTGGTCCGCCGATCGGCTCGGGGCGTGGACTGTTGTCGGCCGCGCCGGCG
GCCAAAGCCCGGGGGCTCCGCGCCCCCGGCAGCCGCCGTCGGCGCAGCCGGTCACCGCGCGCCTC
TGGCGCGCCCCTCGGGGCGCTGCGCTGCAACGGCCTGCGGGCTCCCCATCCGACCCGTCTTGAAAC
ACGGACCAAGGAGTCTGACATGCGTGCGAGTCGACGGGTTCTGAAACCTGGGATGCGCAAGGAAG
CTGACGAGCGGGAGGCCCTCACGGGCCGCACCGCTGGCCGACCCTGATCTTCTGTGAAGGGTTCGA
GTTGGAGCACGCCTGTCGGGACCCGAAAGATGGTGAACTATGCCTGAGCGGGGCGAAGCCAGAGG

AAACTCTGGTGGAGGCTCGAAGCGATACTGACGTGCAAATCGTTCGTCTGACTTGGGTATAGGGGC
GAAAGACTAATCGAACCATCTAGTAGCTGGTTCCCTCCGAAGTTTCCCTCAGGATAGCTGGAGCCCA
TTACGAGTTCTATCGGGTAAAGCCAATGATTAGAGGCATCGGGGGCGCAACGCCCTCGACCTATTCT
CAAACTTTAAATAGGTAGGACGGCGCGGCTGCTCCGGTGAGCCGCGCCACGGAATCGGGAGCTCCA
AGTGGGCCATTTTTGGTAAGCAGAACTGGCGATGCGGGATGAACCGGAAGCCTGGTTACGGTGCCG
AACTGCGCGCTAACCTAGAACCCACAAAGGGTGTTGGTCGATTAAGACAGCAGGACGGTGGTCATG
GAAGTCGAAATCCGCTAAGGAGTGTGTAACAACTCACCTGCCGAATCAACTAGCCCCGAAAATGGA
TGGCGCTGAAGCGCGCGACCCACACCAGGCCATCTGGGCGAGCGCCATGCCCCGATGAGTAGGAG
GGCGCGGCGGCCGCCGCAAAACCCGGGGCGCGAGCCCGGGCGGAGCGGCCGTCGGTGCAGATCTT
GGTGGTAGTAGCAAATATTCAAATGAGAACTTTGAAGGCCGAAGAGGAGAAAGGTTCCATGTGAAC
GGCACTTGCACATGGGTAAGCCGATCCTAAGGGACGGGGTAACCCCGGCAGAGAGCGCGACCACG
CGCGTGCCCCGAAAGGGAATCGGGTTAAGATTTCCCGAGCCGGGACGTGGCGGTTGACGGCGACG
TTAGGAAGTCCGGAGACGCCGGCGGGGGCCTCGGGAAGAGTTATCTTTTCTGCTTAACGGCCCGCC
AACCCTGGAAACGGTTCAGCCGGAGGTAGGGTCCAGCGGCCGGAAGAGCACCGCACGTCGCGCGG
TGTCCGGTGCGCCCCCGGCGGCCCTTGAAAATCCGGAGGACCGAGTACCGTCCACGCCCGGTCGTA
CTCATAACCGCATCAGGTCTCCAAGGTGAACAGCCTCTGGCCAATGGAACAATGTAGGCAAGGGAA
GTCGGCAAAACGGATCCGTAACTTCGGGAAAAGGATTGGCTCTGAGGGCTGGGCTCGGGGGTCCC
GGCCCCGAACCCGTCGGCTGCCGGCGGACTGCTCGAGCTGCTCGCGCGGCGAGAGCGGGCCGCCG
CGTGCCGGCCGGGGGACGGACCGGGAACGGCCCCCTCGGGGGCCTTCCCCGGGCGTCGAACAGCC
GACTCAGAACTGGTACGGACAAGGGGAATCCGACTGTTTAATTAAAACAAAGCATTGCGATGGTCC
TCGCGGATGCTGACGCAATGTGATTTCTGCCCAGTGCTCTGAATGTCAAAGTGAAGAAATTCAACCA
AGCGCGGGTAAACGGCGGGAGTAACTATGACTCTCTTAAGGTAGCCAAATGCCTCGTCATCTAATTA
GTGACGCGCATGAATGGATTAACGAGATTCCCACTGTCCCTGTCTACTATCCAGCGAAACCACAGCC
AAGGGAACGGGCTTGGCGGAATCAGCGGGGAAAGAAGACCCTGTTGAGCTTGACTCTAGTCCGAC
TTTGTGAAATGACTTGAGAGGTGTAGGATAAGTGGGAGCCCTCGGGCGCAAGTGAAATACCACTAC
TTTTAACGTTATTTACTTATTCCGTGAGTCGGAAGCGGGGCCTGGCCCCTCCTTTTGGCTCTAAGGC
CCGAGTCCCTCGGGCCGATCCGGGCGGAAGACATTGTCAGGTGGGGAGTTTGGCTGGGGCGGCAC
ATCTGTTAAAAGATAACGCAGGTGTCCTAAGATGAGCTCAACGAGAACAGAAATCTCGTGTGGAAC
AAAAGGGTAAAAGCTCGTTTGATTCTGATTTCCAGTACGAATACGAACCGTGAAAGCGTGGCCTATC
GATCCTTTAGACCTTCGGAGTTTGAAGCTAGAGGTGTCAGAAAAGTTACCACAGGGATAACTGGCT
TGTGGCAGCCAAGCGTTCATAGCGACGTTGCTTTTTGATCCTTCGATGTCGGCTCTTCCTATCATTGT
GAAGCAGAATTCACCAAGTGTTGGATTGTTCACCCACCAATAGGGAACGTGAGCTGGGTTTAGACC
GTCGTGAGACAGGTTAGTTTTACCCTACTGATGACCGTGCCGCGATAGTAATTCAACCTAGTACGAG
AGGAACCGTTGATTCACACAATTGGTCATCGCGCTTGGTTGAAAAGCCAGTGGCGCGAAGCTACCG
TGTGCCGGATTATGACTGAACGCCTCTAAGTCAGAATCCAAGCTAGCAAGCGGCGCCTGCGCCCGC
CGCCCGCCCCGACCCACGTTAGGGGCGCAAGCCCCCAAGGGCCCGTGCCACCGGCCAAGCCGGCC
CGGCCGACGCGCCGCGGCCGGCCGCCTCGAAGCTCCCTTCCCAACGGGCGGCGGGCTGAATCCTTT
GCAGACGACTTAAATACGCGACGGGGCATTGTAAGTGGCAGAGTGGCCTTGCTGCCACGATCCACT
GAGATCCAGCCCCGCGTCGCACGGATTCGTCCCTCCCCCCTCTCCCCCGCGCCCCGCGCAGGTTCCC
CCCCGAGCCCGCCCCGGTCCGGCCAAGTCCCCAGGCCTCTCTAAGTCCGCCGCGCTGGTGGGGAGG
CACGAAGGGAAAACGCGCTCGCCAAGTCCCAAGAGCCACCGGGCAGACTCCAAGGACGGACGA
CGGGCGGTTGGACGGCGCCCATGCCCACCAAGCCTCCAAGCGTGCCGCCGCACGGAACCCGCCAA
GGTCCTGAGCACGTACCGCGCGAGAGCACCCGCACCACGCCGGGTTCGGTCCACGTCCGCTCGCCC
CAGCTCCCGAGC
>O.rufipogon_AC01-1002323_45S
ACCTGGTTGATCCTGCCAGTAGTCATATGCTTGTCTCAAAGATTAAGCCATGCATGTGCAAGTATGAA
CTAATTTGAACTGTGAAACTGCGAATGGCTCATTAAATCAGTTATAGTTTGTTTGATGGTACGTGCTA
CTCGGATAACCGTAGTAATTCTAGAGCTAATACGTGCAACAAACCCCGACTTCCGGGAGGGGCGCAT
TTATTAGATAAAAGGCTGACGCGGGCTCTGCCCGCTGATCCGATGATTCATGATAACTCGACGGATCG
CACGGCCCTCGTGCCGGCGACGCATCATTCAAATTTCTGCCCTATCAACTTTCGATGGTAGGATAGG
GGCCTACCATGGTGGTGACGGGTGACGGAGAATTAGGGTTCGATTCCGGAGAGGGAGCCTGAGAA
ACGGCTACCACATCCAAGGAAGGCAGCAGGCGCGCAAATTACCCAATCCTGACACGGGGAGGTAG
TGACAATAAATAACAATACCGGGCGCTTTAGTGTCTGGTAATTGGAATGAGTACAATCTAAATCCCTT
AACGAGGATCCATTGGAGGGCAAGTCTGGTGCCAGCAGCCGCGGTAATTCCAGCTCCAATAGCGTA
TATTTAAGTTGTTGCAGTTAAAAAGCTCGTAGTTGGACCTTGGGCCGGGTCGGCCGGTCCGCCTCAC
GGCGAGCACCGACCTACTCGACCCTTCTGCCGGCGATGCGCTCCTGGCCTTAACTGGCCGGGTCGT

GCCTCCGGCGCCGTTACTTTGAAGAAATTAGAGTGCTCAAAGCAAGCCATCGCTCTGGATACATTAG
CATGGGATAACATCATAGGATTCCGGTCCTATTGTGTTGGCCTTCGGGATCGGAGTAATGATTAATAG
GGACAGTCGGGGGCATTCGTATTTCATAGTCAGAGGTGAAATTCTTGGATTTATGAAAGACGAACAA
CTGCGAAAGCATTTGCCAAGGATGTTTTCATTAATCAAGAACGAAAGTTGGGGGCTCGAAGACGAT
CAGATACCGTCCTAGTCTCAACCATAAACGATGCCGACCAGGGATCGGCGGATGTTGCTTATAGGAC
TCCGCCGGCACCTTATGAGAAATCAAAGTCTTTGGGTTCCGGGGGGAGTATGGTCGCAAGGCTGAA
ACTTAAAGGAATTGACGGAAGGGCACCACCAGGCGTGGAGCCTGCGGCTTAATTTGACTCAACACG
GGGAAACTTACCAGGTCCAGACATAGCAAGGATTGACAGACTGAGAGCTCTTTCTTGATTCTATGGG
TGGTGGTGCATGGCCGTTCTTAGTTGGTGGAGCGATTTGTCTGGTTAATTCCGTTAACGAACGAGAC
CTCAGCCTGCTAACTAGCTATGCGGAGCCATCCCTCCGCAGCTAGCTTCTTAGAGGGACTATGGCCG
TTTAGGCCACGGAAGTTTGAGGCAATAACAGGTCTGTGATGCCCTTAGATGTTCTGGGCCGCACGCG
CGCTACACTGATGTATTCAACGAGTATATAGCCTTGGCCGACAGGCCCGGGTAATCTTGGGAAATTTC
ATCGTGATGGGGATAGATCATTGCAATTGTTGGTCTTCAACGAGGAATGCCTAGTAAGCGCGAGTCA
TCAGCTCGCGTTGACTACGTCCCTGCCCTTTGTACACACCGCCCGTCGCTCCTACCGATTGAATGGT
CCGGTGAAGTGTTCGGATCGCGGCGACGGGGGCGGTTCGCCGCCCCCGACGTCGCGAGAAGTCCA
TTGAACCTTATCATTTAGAGGAAGGAGAAGTCGTAACAAGGTTTCCGTAGGTGAACCTGCGGAAGG
ATCATTGTCGTGACCCTGACCAAAACAGACCGCGAACGCGTCACCCCTGCCCGCCGAGCGCTCGCG
CGCGAGGCAACCGAGGCCCCCGGGCCGCAACAGAACCCACGGCGCCGACGGCGTCAAGGAACAC
AGCGATACGCCCGCGCCGCCCGGTCGGCCCTGGCCGTCCGGCGGCGCGGCGCGATACCACGAGTTA
ATCCACACGACTCTCGGCAACGGATATCTCGGCTCTCGCATCGATGAAGAACGTAGCGAAATGCGAT
ACCTGGTGTGAATTGCAGAATCCCGTGAACCATCGAGTCTTTGAACGCAAGTTGCGCCCGAGGCCA
TCCGGCCGAGGGCACGCCTGCCTGGGCGTCACGCCAAAAGACGCTCCACGCGCCCCCCTATCCGG
GAGGGCGCGGGGACGCGGTGTCTGGCCTCCCGCGCCTCGCGGCGCGGCGGGCCGAAGCTCGGGCT
GCCGGCGAAGCGTGCCGGGCACAGCGCATGGTGGACAGCTCACGCTGGCTCTAGGCCGCAGTGCA
CCCCGGCGCGCGGCCGGCGCGATGGCCCCTCAGGACCCAAACGCACCGAGAGCGAACGCCTCGGA
CCGCGACCCTAGGTCAGGCGGGACTACCCGCTGAGTTTAAGCATATAAATAAGCGGAGGAGAAGAA
ACTTACGAGGATTCCCCTAGTAACGGCGAGCGAACCGGGAGATGCCCAGCTTGAGAATCGGGCGGC
CGCGCCGTCCGAATTGTAGTCTGGAGAGGCGTCCTCAGCGACGGACCGGGCCCAAGTCCCCTGGA
AAGGGGCGCCTGGGAGGGTGAGAGCCCCGTCCGGCCCGGACCCTGTCGCCCCACGAGGCGCCGTC
AACGAGTCGGGTTGTTTGGGAATGCAGCCCAAATCGGGCGGTAAACTCCGTCCAAGGCTAAATACA
GGCGAGAGACCGATAGCGAACAAGTACCGCGAGGGAAAGATGAAAAGGACTTTGAAAAGAGAGT
CAAAGAGTGCTTGAAATTGCCGGGAGGGAAGCGGATGGGGGCCGGCGATGCGCCCCGGCCGTATG
CGGAACGGCTCCGGCTGGTCCGCCGATCGGCTCGGGGCGTGGACTGTTGTCGGCCGCGCCGGCGG
CCAAAGCCCGGGGGCTCCGCGCCCCCGGCAGCCGTCGTCGGCGCAGCCGGTCACCGCGCGCCTCT
GGCGCGCCCCTCGGGGCGCTGCGCTGCAACGGCCTGCGGGCTCCCCATCCGACCCGTCTTGAAACA
CGGACCAAGGAGTCTGACATGCGTGCGAGTCGACGGGTTCTGAAACCTGGGATGCGCAAGGAAGC
TGACGAGCGGGAGGCCCTCACGGGCCGCACCGCTGGCCGACCCTGATCTTCTGTGAAGGGTTCGA
GTTGGAGCACGCCTGTCGGGACCCGAAAGATGGTGAACTATGCCTGAGCGGGGCGAAGCCAGAGG
AAACTCTGGTGGAGGCTCGAAGCGATACTGACGTGCAAATCGTTCGTCTGACTTGGGTATAGGGGC
GAAAGACTAATCGAACCATCTAGTAGCTGGTTCCCTCCGAAGTTTCCCTCAGGATAGCTGGAGCCCA
TTACGAGTTCTATCGGGTAAAGCCAATGATTAGAGGCATCGGGGGCGCAACGCCCTCGACCTATTCT
CAAACTTTAAATAGGTAGGACGGCGCGGCTGCTCCGGTGAGCCGCGCCACGGAATCGGGAGCTCCA
AGTGGGCCATTTTTGGTAAGCAGAACTGGCGATGCGGGATGAACCGGAAGCCTGGTTACGGTGCCG
AACTGCGCGCTAACCTAGAACCCACAAAGGGTGTTGGTCGATTAAGACAGCAGGACGGTGGTCATG
GAAGTCGAAATCCGCTAAGGAGTGTGTAACAACTCACCTGCCGAATCAACTAGCCCCGAAAATGGA
TGGCGCTGAAGCGCGCGACCCACACCAGGCCATCTGGGCGAGCGCCATGCCCCGATGAGTAGGAG
GGCGCGGCGGCCGCCGCAAAACCCGGGGCGCGAGCCCGGGCGGAGCGGCCGTCGGTGCAGATCTT
GGTGGTAGTAGCAAATATTCAAATGAGAACTTTGAAGGCCGAAGAGGAGAAAGGTTCCATGTGAAC
GGCACTTGCACATGGGTAAGCCGATCCTAAGGGACGGGGTAACCCCGGCAGAGAGCGCGACCACG
CGCGTGCCCCGAAAGGGAATCGGGTTAAGATTTCCCGAGCCGGGACGTGGCGGTTGACGGCGACG
TTAGGAAGTCCGGAGACGCCGGCGGGGGCCTCGGGAAGAGTTATCTTTTCTGCTTAACGGCCCGCC
AACCCTGGAAACGGCTCAGCCGGAGGTAGGGTCCAGCGGCCGGAAGAGCACCGCACGTCGCGCG
GTGTCCGGTGCGCCCCCGGCGGCCCTTGAAAATCCGGAGGACCGAGTACCGTCCACGCCCGGTCGT
ACTCATAACCGCATCAGGTCTCCAAGGTGAACAGCCTCTGGCCAATGGAACAATGTAGGCAAGGGA
AGTCGGCAAAACGGATCCGTAACTTCGGGAAAAGGATTGGCTCTGAGGGCTGGGCTCGGGGGTCC
CGGCCCCGAACCCGTCGGCTGCCGGCGGACTGCTCGAGCTGCTCGCGCGGCGAGAGCGGGCCGCC

GCGTGCCGGCCGGGGGACGGACCGGGAACGGCCCCCTCGGGGGCCTTCCCCGGGCGTCGAACAGC
CGACTCAGAACTGGTACGGACAAGGGGAATCCGACTGTTTAATTAAAACAAAGCATTGCGATGGTC
CTCGCGGATGCTGACGCAATGTGATTTCTGCCCAGTGCTCTGAATGTCAAAGTGAAGAAATTCAACC
AAGCGCGGGTAAACGGCGGGAGTAACTATGACTCTCTTAAGGTAGCCAAATGCCTCGTCATCTAATT
AGTGACGCGCATGAATGGATTAACGAGATTCCCACTGTCCCTGTCTACTATCCAGCGAAACCACAGC
CAAGGGAACGGGCTTGGCGGAATCAGCGGGGAAAGAAGACCCTGTTGAGCTTGACTCTAGTCCGA
CTTTGTGAAATGACTTGAGAGGTGTAGGATAAGTGGGAGCCCTCGGGCGCAAGTGAAATACCACTA
CTTTTAACGTTATTTTACTTATTCCGTGAGTCGGAAGCGGGGCCTGGCCCCTCCTTTTGGCTCTAAGG
CCCGAGTCCCTCGGGCCGATCCGGGCGGAAGACATTGTCAGGTGGGGAGTTTGGCTGGGGCGGCA
CATCTGTTAAAAGATAACGCAGGTGTCCTAAGATGAGCTCAACGAGAACAGAAATCTCGTGTGGAA
CAAAAGGGTAAAAGCTCGTTTGATTCTGATTTCCAGTACGAATACGAACCGTGAAAGCGTGGCCTAT
CGATCCTTTAGACCTTCGGAGTTTGAAGCTAGAGGTGTCAGAAAAGTTACCACAGGGATAACTGGC
TTGTGGCAGCCAAGCGTTCATAGCGACGTTGCTTTTTGATCCTTCGATGTCGGCTCTTCCTATCATTG
TGAAGCAGAATTCACCAAGTGTTGGATTGTTCACCCACCAATAGGGAACGTGAGCTGGGTTTAGAC
CGTCGTGAGACAGGTTAGTTTTACCCTACTGATGACCGTGCCGCGATAGTAATTCAACCTAGTACGA
GAGGAACCGTTGATTCACACAATTGGTCATCGCGCTTGGTTGAAAAGCCAGTGGCGCGAAGCTACC
GTGTGCCGGATTATGACTGAACGCCTCTAAGTCAGAATCCAAGCTAGCAAGCGGCGCCTGCGCCCG
CCGCCCGCCCCGACCCACGTTAGGGGCGCAAGCCCCCAAGGGCCCGTGCCACCGGCCAAGCCGGC
CCGGCCGACGCGCCGCGGCCGGCCGCCTCGAAGCTCCCTTCCCAACGGGCGGCGGGCTGAATCCTT
TGCAGACGACTTAAATACGCGACGGGGCATTGTAAGTGGCAGAGTGGCCTTGCTGCCACGATCCAC
TGAGATCCAGCCCCGCGTCGCACGGATTCGTCCCTCCCCCCTCTCCCCCGCGCCCCGCGCAGGTTCC
C
>O.nivara_IRGC88812_45S
ACCTGGTTGATCCTGCCAGTAGTCATATGCTTGTCTCAAAGATTAAGCCATGCATGTGCAAGTATGAA
CTAATTCGAACTGTGAAACTGCGAATGGCTCATTAAATCAGTTATAGTTTGTTTGATGGTACGTGCTA
CTCGGATAACCGTAGTAATTCTAGAGCTAATACGTGCAACAAACCCCGACTTCCGGGAGGGGCGCAT
TTATTAGATAAAAGGCTGACGCGGGCTCCGCCCGCTGATCCGATGATTCATGATAACTCGACGGATC
GCACGGCCCTCGTGCCGGCGACGCATCATTCAAATTTCTGCCCTATCAACTTTCGATGGTAGGATAG
GGGCCTACCATGGTGGTGACGGGTGACGGAGAATTAGGGTTCGATTCCGGAGAGGGAGCCTGAGA
AACGGCTACCACATCCAAGGAAGGCAGCAGGCGCGCAAATTACCCAATCCTGACACGGGGAGGTA
GTGACAATAAATAACAATACCGGGCGCTTTAGTGTCTGGTAATTGGAATGAGTACAATCTAAATCCCT
TAACGAGGATCCATTGGAGGGCAAGTCTGGTGCCAGCAGCCGCGGTAATTCCAGCTCCAATAGCGT
ATATTTAAGTTGTTGCAGTTAAAAAGCTCGTAGTTGGACCTTGGGCCGGGCCGGCCGGTCCGCCTCA
CGGCGAGCACCGACCTGCTCGACCCTTCTGCCGGCGATGCGCTCCTGGCCTTAACTGGCCGGGTCG
TGCCTCCGGCGCCGTTACTTTGAAGAAATTAGAGTGCTCAAAGCAAGCCATCGCTCTGGATACATTA
GCATGGGATAACATCATAGGATTCCGGTCCTATTGTGTTGGCCTTCGGGATCGGAGTAATGATTAATA
GGGACAGTCGGGGGCATTCGTATTTCATAGTCAGAGGTGAAATTCTTGGATTTATGAAAGACGAACA
ACTGCGAAAGCATTTGCCAAGGATGTTTTCATTAATCAAGAACGAAAGTTGGGGGCTCGAAGACGA
TCAGATACCGTCCTAGTCTCAACCATAAACGATGCCGACCAGGGATCGGCGGATGTTGCTTATAGGA
CTCCGCCGGCACCTTATGAGAAATCAAAGTCTTTGGGTTCCGGGGGGAGTATGGTCGCAAGGCTGA
AACTTAAAGGAATTGACGGAAGGGCACCACCAGGCGTGGAGCCTGCGGCTTAATTTGACTCAACAC
GGGGAAACTTACCAGGTCCAGACATAGCAAGGATTGACAGACTGAGAGCTCTTTCTTGATTCTATGG
GTGGTGGTGCATGGCCGTTCTTAGTTGGTGGAGCGATTTGTCTGGTTAATTCCGTTAACGAACGAGA
CCTCAGCCTGCTAACTAGCTATGCGGAGCCATCCCTCCGCAGCTAGCTTCTTAGAGGGACTATGGCC
GTTTAGGCCACGGAAGTTTGAGGCAATAACAGGTCTGTGATGCCCTTAGATGTTCTGGGCCGCACGC
GCGCTACACTGATGTATTCAACGAGTATATAGCCTTGGCCGACAGGCCCGGGTAATCTTGGGAAATT
TCATCGTGATGGGGATAGATCATTGCAATTGTTGGTCTTCAACGAGGAATGCCTAGTAAGCGCGAGT
CATCAGCTCGCGTTGACTACGTCCCTGCCCTTTGTACACACCGCCCGTCGCTCCTACCGATTGAATG
GTCCGGTGAAGTGTTCGGATCGCGGCGACGGGGGCGGTTCGCCGCCCCCGACGTCGCGAGAAGTC
CATTGAACCTTATCATTTAGAGGAAGGAGAAGTCGTAACAAGGTTTCCGTAGGTGAACCTGCGGAA
GGATCATTGTCGTGACCCTGACCAAAACAGACCGCGAACGCGTCACCCCTGCCCGCCGAGCGCTCG
CGCGCGAGGCAACCGAGGCCCCCGGGCCGCAACAGAACCCACGGCGCCGACGGCGTCAAGGAAC
ACAGCGATACGCCCCGCGCTGGCCCGGTCGGCCCTGGCCGTCCGGCGGCGCGGCGCGATACCACGA
GCTAAATCCACACGACTCTCGGCAACGGATATCTCGGCTCTCGCATCGATGAAGAACGTAGCGAAAT
GCGATACCTGGTGTGAATTGCAGAATCCCGTGAACCATCGAGTCTTTGAACGCAAGTTGCGCCCGA
GGCCATCCGGCCGAGGGCACGCCTGCCTGGGCGTCACGCCAAAAGACGCTCCACGCGCCCCCCCT

ATCCGGGAGGGCGCGGGGACGCGGTGTCTGGCCCCCCGCGCCTCGCGGCGCGGTGGGCCGAAGCT
CGGGCTGCCGGCGAAGCGTGCCGGGCACAGCGCATGGTGGACAGCTCACGCTGGCTCTAGGCCGC
AGTGCACCCCGGCGCGCGGCCGGCGCGATGGCCCCTCAGGACCCAAACGCACCGAGAGCGAACGC
CTCGGACCGCGACCCCAGGTCAGGCGGGACTACCCGCTGAGTTTAAGCATATAAATAAGCGGAGGA
GAAGAAACTTACGAGGATTCCCCTAGTAACGGCGAGCGAACCGGGAGATGCCCAGCTTGAGAATCG
GGCGGCCGCGCCGTCCGAATTGTAGTCTGGAGAGGCGTCCTCAGCGACGGACCGGGCCCAAGTCC
CCTGGAAAGGGGCGCCTGGGAGGGTGAGAGCCCCGTCCGGCCCGGACCCTGTCGCCCCACGAGGC
GCCGTCAACGAGTCGGGTTGTTTGGGAATGCAGCCCAAATCGGGCGGTAAACTCCGTCCAAGGCTA
AATACAGGCGAGAGACCGATAGCGAACAAGTACCGCGAGGGAAAGATGAAAAGGACTTTGAAAAG
AGAGTCAAAGAGTGCTTGAAATTGCCGGGAGGGAAGCGGATGGGGGCCGGCGATGCGCCCCGGCC
GTATGCGGAACGGCTTCGGCTGGTCCGCCGATCGGCTCGGGGCGTGGACTGTTGTCGGCCGCGCTG
GCGGCCAAAGCCCGGGGGCTCCGCGCCCCCGGCAGCCGTCGTCGGCGCAGCCGGTCACCGCGCGC
CTCTGGCGCGCCCCTCGGGGCGCCGCGCTGCAACGGCCTGCGGGCTCCCCATCCGACCCGTCTTGA
AACACGGACCAAGGAGTCTGACATGCGTGCGAGTCGACGGGTTCTGAAACCTGGGATGCGCAAGG
AAGCTGACGAGCGGGAGGCCCTCACGGGCCGCACCGCTGGCCGACCCTGATCTTCTGTGAAGGGT
TCGAGTTGGAGCACGCCTGTCGGGACCCGAAAGATGGTGAACTATGCCTGAGCGGGGCGAAGCCA
GAGGAAACTCTGGTGGAGGCTCGAAGCGATACTGACGTGCAAATCGTTCGTCTGACTTGGGTATAG
GGGCGAAAGACTAATCGAACCATCTAGTAGCTGGTTCCCTCCGAAGTTTCCCTCAGGATAGCTGGAG
CCCATTACGAGTTCTATCGGGTAAAGCCAATGATTAGAGGCATCGGGGGCGCAACGCCCTCGACCTA
TTCTCAAACTTTAAATAGGTAGGACGGCGCGGCTGCTCCGGTGAGCCGCGCCACGGAATCGGGAGC
TCCAAGTGGGCCATTTTTGGTAAGCAGAACTGGCGATGCGGGATGAACCGGAAGCCTGGTTACGGT
GCCGAACTGCGCGCTAACCTAGAACCCACAAAGGGTGTTGGTCGATTAAGACAGCAGGACGGTGG
TCATGGAAGTCGAAATCCGCTAAGGAGTGTGTAACAACTCACCTGCCGAATCAACTAGCCCCGAAA
ATGGATGGCGCTGAAGCGCGCGACCCACACCAGGCCATCTGGGCGAGCGCCATGCCCCGATGAGTA
GGAGGGCGCGGCGGCCGCCGCAAAACCCGGGGCGCGAGCCCGGGCGGAGCGGCCGTCGGTGCAG
ATCTTGGTGGTAGTAGCAAATATTCAAATGAGAACTTTGAAGGCCGAAGAGGAGAAAGGTTCCATG
TGAACGGCACTTGCACATGGGTAAGCCGATCCTAAGGGACGGGGTAACCCCGGCAGAGAGCGCGA
CCACGCGCGTGCCCCGAAAGGGAATCGGGTTAAGATTTCCCGAGCCGGGACGTGGCGGTTGACGG
CGACGTTAGGAAGTCCGGAGACGCCGGCGGGGGCCTCGGGAAGAGTTATCTTTTCTGCTTAACGGC
CCGCCAACCCTGGAAACGGTTCAGCCGGAGGTAGGGTCCAGCGGCCGGAAGAGCACCGCACGTCG
CGCGGTGTCCGGTGCGCCCCCGGCGGCCCTTGAAAATCCGGAGGACCGAGTACCGTCCACGCCCG
GTCGTACTCATAACCGCATCAGGTCTCCAAGGTGAACAGCCTCTGGCCAATGGAACAATGTAGGCA
AGGGAAGTCGGCAAAACGGATCCGTAACTTCGGGAAAAGGATTGGCTCTGAGGGCTGGGCTCGGG
GGTCCCGGCCCCGAACCCGTCGGCTGCCGGCGGACTGCTCGAGCTGCTCGCGCGGCGAGAGCGGG
CCGCCGCGTGCCGGCCGGGGGACGGACCGGGAACGGCCCCCTCGGGGGCCTTCCCCGGGCGTCGA
ACAGCCGACTCAGAACTGGTACGGACAAGGGGAATCCGACTGTTTAATTAAAACAAAGCATTGCGA
TGGTCCTCGCGGATGCTGACGCAATGTGATTTCTGCCCAGTGCTCTGAATGTCAAAGTGAAGAAATT
CAACCAAGCGCGGGTAAACGGCGGGAGTAACTATGACTCTCTTAAGGTAGCCAAATGCCTCGTCAT
CTAATTAGTGACGCGCATGAATGGATTAACGAGATTCCCACTGTCCCTGTCTACTATCCAGCGAAACC
ACAGCCAAGGGAACGGGCTTGGCGGAATCAGCGGGGAAAGAAGACCCTGTTGAGCTTGACTCTAG
TCCGACTTTGTGAAATGACTTGAGAGGTGTAGGATAAGTGGGAGCCCTCGGGCGCAAGTGAAATAC
CACTACTTTTAACGTTATTTACTTATTCCGTGAGTCGGAAGCGGGGCCTGGCCCCTCCTTTTGGCTC
TAAGGCCCGAGTCCCTCGGGCCGATCCGGGCGGAAGACATTGTCAGGTGGGGAGTTTGGCTGGGG
CGGCACATCTGTTAAAAGATAACGCAGGTGTCCTAAGATGAGCTCAACGAGAACAGAAATCTCGTG
TGGAACAAAAGGGTAAAAGCTCGTTTGATTCTGATTTCCAGTACGAATACGAACCGTGAAAGCGTG
GCCTATCGATCCTTTAGACCTTCGGAGTTTGAAGCTAGAGGTGTCAGAAAAGTTACCACAGGGATAA
CTGGCTTGTGGCAGCCAAGCGTTCATAGCGACGTTGCTTTTTGATCCTTCGATGTCGGCTCTTCCTAT
CATTGTGAAGCAGAATTCACCAAGTGTTGGATTGTTCACCCACCAATAGGGAACGTGAGCTGGGTT
TAGACCGTCGTGAGACAGGTTAGTTTTACCCTACTGATGACCGTGCCGCGATAGTAATTCAACCTAG
TACGAGAGGAACCGTTGATTCACACAATTGGTCATCGCGCTTGGTTGAAAAGCCAGTGGCGCGAAG
CTACCGTGTGCCGGATTATGACTGAACGCCTCTAAGTCAGAATCCAAGCTAGCAAGCGGCGCCTGC
GCCCGCCGCCCGCCCCGACCCACGTTAGGGGCGCAAGCCCCCAAGGGCCCGTGCCACCGGCCAAG
CCGGCCCGGCCGACGCGCCGCGGCCGGCCGCCTCGAAGCTCCCTTCCCAACGGGCGGCGGGCTGA
ATCCTTTGCAGACGACTTAAATACGCGACGGGGCATTGTAAGTGGCAGAGTGGCCTTGCTGCCACG
ATCCACTGAGATCCAGCCCCGCGTCGCACGGATTCGTCCCTCCCCCCTCTCCCCCGCGCCCCGCGCA
GGTTCCCCCCCGAGCCCGCCCCGGTCCGGCCAAGTCCCCAG

>O.barthii_IRGC101252_45S
ACCTGGTTGATCCTGCCAGTAGTCATATGCTTGTCTCAAAGATTAAGCCATGCATGTGCAAGTATGAA
CTAATTCGAACTGTGAAACTGCGAATGGCTCATTAAATCAGTTATAGTTTGTTTGATGGTACGTGCTA
CTCGGATAACCGTAGTAATTCTAGAGCTAATACGTGCAACAAACCCCGACTTCCGGGAGGGGCGCAT
TTATTAGATAAAAGGCTGACGCGGGCTCTGCCCGCTGATCCGATGATTCATGATAACTCGACGGATCG
CACGGCCCTCGTGCCGGCGACGCATCATTCAAATTTCTGCCCTATCAACTTTCGATGGTAGGATAGG
GGCCTACCATGGTGGTGACGGGTGACGGAGAATTAGGGTTCGATTCCGGAGAGGGAGCCTGAGAA
ACGGCTACCACATCCAAGGAAGGCAGCAGGCGCGCAAATTACCCAATCCTGACACGGGGAGGTAG
TGACAATAAATAACAATACCGGGCGCTTTAGTGTCTGGTAATTGGAATGAGTACAATCTAAATCCCTT
AACGAGGATCCATTGGAGGGCAAGTCTGGTGCCAGCAGCCGCGGTAATTCCAGCTCCAATAGCGTA
TATTTAAGTTGTTGCAGTTAAAAAGCTCGTAGTTGGACCTTGGGCCGGGTCGGCCGGTCCGCCTCAC
GGCGAGCACCGACCTACTCGACCCTTCTGCCGGCGATGCGCTCCTGGCCTTAACTGGCCGGGTCGT
GCCTCCGGCGCCGTTACTTTGAAGAAATTAGAGTGCTCAAAGCAAGCCATCGCTCTGGATACATTAG
CATGGGATAACATCATAGGATTCCGGTCCTATTGTGTTGGCCTTCGGGATCGGAGTAATGATTAATAG
GGACAGTCGGGGGCATTCGTATTTCATAGTCAGAGGTGAAATTCTTGGATTTATGAAAGACGAACAA
CTGCGAAAGCATTTGCCAAGGATGTTTTCATTAATCAAGAACGAAAGTTGGGGGCTCGAAGACGAT
CAGATACCGTCCTAGTCTCAACCATAAACGATGCCGACCAGGGATCGGCGGATGTTGCTTATAGGAC
TCCGCCGGCACCTTATGAGAAATCAAAGTCTTTGGGTTCCGGGGGGAGTATGGTCGCAAGGCTGAA
ACTTAAAGGAATTGACGGAAGGGCACCACCAGGCGTGGAGCCTGCGGCTTAATTTGACTCAACACG
GGGAAACTTACCAGGTCCAGACATAGCAAGGATTGACAGACTGAGAGCTCTTTCTTGATTCTATGGG
TGGTGGTGCATGGCCGTTCTTAGTTGGTGGAGCGATTTGTCTGGTTAATTCCGTTAACGAACGAGAC
CTCAGCCTGCTAACTAGCTATGCGGAGCCATCCCTCCGCAGCTAGCTTCTTAGAGGGACTATGGCCG
TTTAGGCCACGGAAGTTTGAGGCAATAACAGGTCTGTGATGCCCTTAGATGTTCTGGGCCGCACGCG
CGCTACACTGATGTATTCAACGAGTATATAGCCTTGGCCGACAGGCCCGGGTAATCTTGGGAAATTTC
ATCGTGATGGGGATAGATCATTGCAATTGTTGGTCTTCAACGAGGAATGCCTAGTAAGCGCGAGTCA
TCAGCTCGCGTTGACTACGTCCCTGCCCTTTGTACACACCGCCCGTCGCTCCTACCGATTGAATGGT
CCGGTGAAGTGTTCGGATCGCGGCGACGGGGGCGGTTCGCCGCCCCCGACGTCGCGAGAAGTCCA
TTGAACCTTATCATTTAGAGGAAGGAGAAGTCGTAACAAGGTTTCCGTAGGTGAACCTGCGGAAGG
ATCATTGTCGTGACCCTGACCAAAACAGACCGCGAACGCGTCACCCCTGCCCGCCGAGCGCTCGCG
CGCTCGGCAACCGAGGCCCCCGGGCCGCAACAGAACCCACGGCGCCGACGGCGTCAAGGAACAC
AGCGATACGCCCGCGCCGCTCGGTCGGCCCTGGCCGTCCGGCGGCGCGGCGCGATACCACGAGTTA
AATCCACACGACTCTCGGCAACGGATATCTCGGCTCTCGCATCGATGAAGAACGTAGCGAAATGCG
ATACCTGGTGTGAATTGCAGAATCCCGTGAACCATCGAGTCTTTGAACGCAAGTTGCGCCCGAGGC
CATCCGGCCGAGGGCACGCCTGCCTGGGCGTCACGCCAAAAGACGCTCCACGCGCCCCTCCTATCC
GGGAGGGCGCGGGGACGCGGTGTCTGGCCTCCCGCGCCTCGCGGCGCGGTGGGCCGAAGCTCGGG
CTGCCGGCGAAGCGTGCCGGGCACAGCGCATGGTGGACAGCTCACGCTGGCTCTAGGCCGCAGTG
CACCCCGGCGCGCGGCCGGCGCGATGGCCCCTCAGGACCCAAACGCACCGAGAGCGAACGCCTCG
GACCGCGACCCCAGGTCAGGCGGGACTACCCGCTGAGTTTAAGCATATAAATAAGCGGAGGAGAAG
AAACTTACGAGGATTCCCCTAGTAACGGCGAGCGAACCGGGAGATGCCCAGCTTGAGAATCGGGCG
GCCGCGCCGTCCGAATTGTAGTCTGGAGAGGCGTCCTCAGCGACGGACCGGGCCCAAGTCCCCTGG
AAAGGGGCGCCTGGGAGGGTGAGAGCCCCGTCCGGCCCGGACCCTGTCGCCCCACGAGGCGCCGT
CAACGAGTCGGGTTGTTTGGGAATGCAGCCCAAATCGGGCGGTAAACTCCGTCCAAGGCTAAATAC
AGGCGAGAGACCGATAGCGAACAAGTACCGCGAGGGAAAGATGAAAAGGACTTTGAAAAGAGAG
TCAAAGAGTGCTTGAAATTGCCGGGAGGGAAGCGGATGGGGGCCGGCGATGCGCCCCGGCCGTAT
GCGGAACGGCTTCGGCTGGTCCGCCGATCGGCTCGGGGCGTGGACTGTTGTCGGCCGCGCCGGCG
GCCAAAGCCCGGGGGCTCCGCGCCCCCGGCAGCCGTCGTCGGCGCAGCCGGTCACCGCGCGCCTC
TGGCGCGCCCCTCGGGGCGCTGCGCTGCAACGGCCTGCGGGCTCCCCATCCGACCCGTCTTGAAAC
ACGGACCAAGGAGTCTGACATGCGTGCGAGTCGACGGGTTCTGAAACCTGGGATGCGCAAGGAAG
CTGACGAGCGGGAGGCCCTCACGGGCCGCACCGCTGGCCGACCCTGATCTTCTGTGAAGGGTTCGA
GTTGGAGCACGCCTGTCGGGACCCGAAAGATGGTGAACTATGCCTGAGCGGGGCGAAGCCAGAGG
AAACTCTGGTGGAGGCTCGAAGCGATACTGACGTGCAAATCGTTCGTCTGACTTGGGTATAGGGGC
GAAAGACTAATCGAACCATCTAGTAGCTGGTTCCCTCCGAAGTTTCCCTCAGGATAGCTGGAGCCCA
TTACGAGTTCTATCGGGTAAAGCCAATGATTAGAGGCATCGGGGGCGCAACGCCCTCGACCTATTCT
CAAACTTTAAATAGGTAGGACGGCGCGGCTGCTCCGGTGAGCCGCGCCACGGAATCGGGAGCTCCA
AGTGGGCCATTTTTGGTAAGCAGAACTGGCGATGCGGGATGAACCGGAAGCCTGGTTACGGTGCCG
AACTGCGCGCTAACCTAGAACCCACAAAGGGTGTTGGTCGATTAAGACAGCAGGACGGTGGTCATG

GAAGTCGAAATCCGCTAAGGAGTGTGTAACAACTCACCTGCCGAATCAACTAGCCCCGAAAATGGA
TGGCGCTGAAGCGCGCGACCCACACCAGGCCATCTGGGCGAGCGCCATGCCCCGATGAGTAGGAG
GGCGCGGCGGCCGCCGCAAAACCCGGGGCGCGAGCCCGGGCGGAGCGGCCGTCGGTGCAGATCTT
GGTGGTAGTAGCAAATATTCAAATGAGAACTTTGAAGGCCGAAGAGGAGAAAGGTTCCATGTGAAC
GGCACTTGCACATGGGTAAGCCGATCCTAAGGGACGGGGTAACCCCGGCAGAGAGCGCGACCACG
CGCGTGCCCCGAAAGGGAATCGGGTTAAGATTTCCCGAGCCGGGACGTGGCGGTTGACGGCGACG
TTAGGAAGTCCGGAGACGCCGGCGGGGGCCTCGGGAAGAGTTATCTTTTCTGCTTAACGGCCCGCC
AACCCTGGAAACGGTTCAGCCGGAGGTAGGGTCCAGCGGCCGGAAGAGCACCGCACGTCGCGCGG
TGTCCGGTGCGCCCCCGGCGGCCCTTGAAAATCCGGAGGACCGAGTACCGTCCACGCCCGGTCGTA
CTCATAACCGCATCAGGTCTCCAAGGTGAACAGCCTCTGGCCAATGGAACAATGTAGGCAAGGGAA
GTCGGCAAAACGGATCCGTAACTTCGGGAAAAGGATTGGCTCTGAGGGCTGGGCTCGGGGGTCCC
GGCCCCGAACCCGTCGGCTGCCGGCGGACTGCTCGAGCTGCTCGCGCGGCGAGAGCGGGCCGCCG
CGTGCCGGCCGGGGGACGGACCGGGAACGGCCCCCTCGGGGGCCTTCCCCGGGCGTCGAACAGCC
GACTCAGAACTGGTACGGACAAGGGGAATCCGACTGTTTAATTAAAACAAAGCATTGCGATGGTCC
TCGCGGATGCTGACGCAATGTGATTTCTGCCCAGTGCTCTGAATGTCAAAGTGAAGAAATTCAACCA
AGCGCGGGTAAACGGCGGGAGTAACTATGACTCTCTTAAGGTAGCCAAATGCCTCGTCATCTAATTA
GTGACGCGCATGAATGGATTAACGAGATTCCCACTGTCCCTGTCTACTATCCAGCGAAACCACAGCC
AAGGGAACGGGCTTGGCGGAATCAGCGGGGAAAGAAGACCCTGTTGAGCTTGACTCTAGTCCGAC
TTTGTGAAATGACTTGAGAGGTGTAGGATAAGTGGGAGCCCTCGGGCGCAAGTGAAATACCACTAC
TTTTAACGTTATTTTACTTATTCCGTGAGTCGGAAGCGGGGCCTGGCCCCTCCTTTTGGCTCTAAGGC
CCGAGTCCCTCGGGCCGATCCGGGCGGAAGACATTGTCAGGTGGGGAGTTTGGCTGGGGCGGCAC
ATCTGTTAAAAGATAACGCAGGTGTCCTAAGATGAGCTCAACGAGAACAGAAATCTCGTGTGGAAC
AAAAGGGTAAAAGCTCGTTTGATTCTGATTTCCAGTACGAATACGAACCGTGAAAGCGTGGCCTATC
GATCCTTTAGACCTTCGGAGTTTGAAGCTAGAGGTGTCAGAAAAGTTACCACAGGGATAACTGGCT
TGTGGCAGCCAAGCGTTCATAGCGACGTTGCTTTTTGATCCTTCGATGTCGGCTCTTCCTATCATTGT
GAAGCAGAATTCACCAAGTGTTGGATTGTTCACCCACCAATAGGGAACGTGAGCTGGGTTTAGACC
GTCGTGAGACAGGTTAGTTTTACCCTACTGATGACCGTGCCGCGATAGTAATTCAACCTAGTACGAG
AGGAACCGTTGATTCACACAATTGGTCATCGCGCTTGGTTGAAAAGCCAGTGGCGCGAAGCTACCG
TGTGCCGGATTATGACTGAACGCCTCTAAGTCAGAATCCAAGCTAGCAAGCGGCGCCTGCGCCCGC
CGCCCGCCCCGACCCACGTTAGGGGCGCAAGCCCCCAAGGGCCCGTGCCACTGGCCAAGCCGGCC
CGGCCGACGCGCCGCGGCCGGCCGCCTCGAAGCTCCCTTCCCAACGGGCGGCGGGCTGAATCCTTT
GCAGACGACTTAAATACGCGACGGGGCATTGTAAGTGGCAGAGTGGCCTTGCTGCCACGATCCACT
GAGATCCAGCCCCGCGTCGCACGGATTCGTCCCTCCCCCCACACACCCTCTCCCCCGCACCCCGCG
CAGGTTCTCCCCCCCGAGCCCGCCCCAGTCCCGCCGAGTCCCCAAGTCTCTCTAAGTCCGCTGCGTT
GG
>O.glaberrima_IRGC103486_45S
ACCTGGTTGATCCTGCCAGTAGTCATATGCTTGTCTCAAAGATTAAGCCATGCATGTGCAAGTATGAA
CTAATTCGAACTGTGAAACTGCGAATGGCTCATTAAATCAGTTATAGTTTGTTTGATGGTACGTGCTA
CTCGGATAACCGTAGTAATTCTAGAGCTAATACGTGCAACAAACCCCGACTTCCGGGAGGGGCGCAT
TTATTAGATAAAAGGCTGACGCGGGCTCTGCCCGCTGATCCGATGATTCATGATAACTCGACGGATCG
CACGGCCCTCGTGCCGGCGACGCATCATTCAAATTTCTGCCCTATCAACTTTCGATGGTAGGATAGG
GGCCTACCATGGTGGTGACGGGTGACGGAGAATTAGGGTTCGATTCCGGAGAGGGAGCCTGAGAA
ACGGCTACCACATCCAAGGAAGGCAGCAGGCGCGCAAATTACCCAATCCTGACACGGGGAGGTAG
TGACAATAAATAACAATACCGGGCGCTTTAGTGTCTGGTAATTGGAATGAGTACAATCTAAATCCCTT
AACGAGGATCCATTGGAGGGCAAGTCTGGTGCCAGCAGCCGCGGTAATTCCAGCTCCAATAGCGTA
TATTTAAGTTGTTGCAGTTAAAAAGCTCGTAGTTGGACCTTGGGCCGGGTCGGCCGGTCCGCCTCAC
GGCGAGCACCGACCTACTCGACCCTTCTGCCGGCGATGCGCTCCTGGCCTTAACTGGCCGGGTCGT
GCCTCCGGCGCCGTTACTTTGAAGAAATTAGAGTGCTCAAAGCAAGCCATCGCTCTGGATACATTAG
CATGGGATAACATCATAGGATTCCGGTCCTATTGTGTTGGCCTTCGGGATCGGAGTAATGATTAATAG
GGACAGTCGGGGGCATTCGTATTTCATAGTCAGAGGTGAAATTCTTGGATTTATGAAAGACGAACAA
CTGCGAAAGCATTTGCCAAGGATGTTTTCATTAATCAAGAACGAAAGTTGGGGGCTCGAAGACGAT
CAGATACCGTCCTAGTCTCAACCATAAACGATGCCGACCAGGGATCGGCGGATGTTGCTTATAGGAC
TCCGCCGGCACCTTATGAGAAATCAAAGTCTTTGGGTTCCGGGGGGAGTATGGTCGCAAGGCTGAA
ACTTAAAGGAATTGACGGAAGGGCACCACCAGGCGTGGAGCCTGCGGCTTAATTTGACTCAACACG
GGGAAACTTACCAGGTCCAGACATAGCAAGGATTGACAGACTGAGAGCTCTTTCTTGATTCTATGGG
TGGTGGTGCATGGCCGTTCTTAGTTGGTGGAGCGATTTGTCTGGTTAATTCCGTTAACGAACGAGAC

CTCAGCCTGCTAACTAGCTATGCGGAGCCATCCCTCCGCAGCTAGCTTCTTAGAGGGACTATGGCCG
TTTAGGCCACGGAAGTTTGAGGCAATAACAGGTCTGTGATGCCCTTAGATGTTCTGGGCCGCACGCG
CGCTACACTGATGTATTCAACGAGTATATAGCCTTGGCCGACAGGCCCGGGTAATCTTGGGAAATTTC
ATCGTGATGGGGATAGATCATTGCAATTGTTGGTCTTCAACGAGGAATGCCTAGTAAGCGCGAGTCA
TCAGCTCGCGTTGACTACGTCCCTGCCCTTTGTACACACCGCCCGTCGCTCCTACCGATTGAATGGT
CCGGTGAAGTGTTCGGATCGCGGCGACGGGGGCGGTTCGCCGCCCCCGACGTCGCGAGAAGTCCA
TTGAACCTTATCATTTAGAGGAAGGAGAAGTCGTAACAAGGTTTCCGTAGGTGAACCTGCGGAAGG
ATCATTGTCGTGACCCTGACCAAAACAGACCGCGAACGCGTCACCCCTGCCCGCCGAGCGCTCGCG
CGCTCGGCAACCGAGGCCCCCGGGCCGCAACAGAACCCACGGCGCCGACGGCGTCAAGGAACAC
AGCGATACGCCCGCGCCGCTCGGTCGGCCCTGGCCGTCCGGCGGCGCGGCGCGATACCACGAGTTA
AATCCACACGACTCTCGGCAACGGATATCTCGGCTCTCGCATCGATGAAGAACGTAGCGAAATGCG
ATACCTGGTGTGAATTGCAGAATCCCGTGAACCATCGAGTCTTTGAACGCAAGTTGCGCCCGAGGC
CATCCGGCCGAGGGCACGCCTGCCTGGGCGTCACGCCAAAAGACGCTCCACGCGCCCCTCCTATCC
GGGAGGGCGCGGGGACGCGGTGTCTGGCCTCCCGCGCCTCGCGGCGCGGTGGGCCGAAGCTCGGG
CTGCCGGCGAAGCGTGCCGGGCACAGCGCATGGTGGACAGCTCACGCTGGCTCTAGGCCGCAGTG
CACCCCGGCGCGCGGCCGGCGCGATGGCCCCTCAGGACCCAAACGCACCGAGAGCGAACGCCTCG
GACCGCGACCCCAGGTCAGGCGGGACTACCCGCTGAGTTTAAGCATATAAATAAGCGGAGGAGAAG
AAACTTACGAGGATTCCCCTAGTAACGGCGAGCGAACCGGGAGATGCCCAGCTTGAGAATCGGGCG
GCCGCGCCGTCCGAATTGTAGTCTGGAGAGGCGTCCTCAGCGACGGACCGGGCCCAAGTCCCCTGG
AAAGGGGCGCCTGGGAGGGTGAGAGCCCCGTCCGGCCCGGACCCTGTCGCCCCACGAGGCGCCGT
CAACGAGTCGGGTTGTTTGGGAATGCAGCCCAAATCGGGCGGTAAACTCCGTCCAAGGCTAAATAC
AGGCGAGAGACCGATAGCGAACAAGTACCGCGAGGGAAAGATGAAAAGGACTTTGAAAAGAGAG
TCAAAGAGTGCTTGAAATTGCCGGGAGGGAAGCGGATGGGGGCCGGCGATGCGCCCCGGCCGTAT
GCGGAACGGCTTCGGCTGGTCCGCCGATCGGCTCGGGGCGTGGACTGTTGTCGGCCGCGCCGGCG
GCCAAAGCCCGGGGGCTCCGCGCCCCCGGCAGCCGTCGTCGGCGCAGCCGGTCACCGCGCGCCTC
TGGCGCGCCCCTCGGGGCGCTGCGCTGCAACGGCCTGCGGGCTCCCCATCCGACCCGTCTTGAAAC
ACGGACCAAGGAGTCTGACATGCGTGCGAGTCGACGGGTTCTGAAACCTGGGATGCGCAAGGAAG
CTGACGAGCGGGAGGCCCTCACGGGCCGCACCGCTGGCCGACCCTGATCTTCTGTGAAGGGTTCGA
GTTGGAGCACGCCTGTCGGGACCCGAAAGATGGTGAACTATGCCTGAGCGGGGCGAAGCCAGAGG
AAACTCTGGTGGAGGCTCGAAGCGATACTGACGTGCAAATCGTTCGTCTGACTTGGGTATAGGGGC
GAAAGACTAATCGAACCATCTAGTAGCTGGTTCCCTCCGAAGTTTCCCTCAGGATAGCTGGAGCCCA
TTACGAGTTCTATCGGGTAAAGCCAATGATTAGAGGCATCGGGGGCGCAACGCCCTCGACCTATTCT
CAAACTTTAAATAGGTAGGACGGCGCGGCTGCTCCGGTGAGCCGCGCCACGGAATCGGGAGCTCCA
AGTGGGCCATTTTTGGTAAGCAGAACTGGCGATGCGGGATGAACCGGAAGCCTGGTTACGGTGCCG
AACTGCGCGCTAACCTAGAACCCACAAAGGGTGTTGGTCGATTAAGACAGCAGGACGGTGGTCATG
GAAGTCGAAATCCGCTAAGGAGTGTGTAACAACTCACCTGCCGAATCAACTAGCCCCGAAAATGGA
TGGCGCTGAAGCGCGCGACCCACACCAGGCCATCTGGGCGAGCGCCATGCCCCGATGAGTAGGAG
GGCGCGGCGGCCGCCGCAAAACCCGGGGCGCGAGCCCGGGCGGAGCGGCCGTCGGTGCAGATCTT
GGTGGTAGTAGCAAATATTCAAATGAGAACTTTGAAGGCCGAAGAGGAGAAAGGTTCCATGTGAAC
GGCACTTGCACATGGGTAAGCCGATCCTAAGGGACGGGGTAACCCCGGCAGAGAGCGCGACCACG
CGCGTGCCCCGAAAGGGAATCGGGTTAAGATTTCCCGAGCCGGGACGTGGCGGTTGACGGCGACG
TTAGGAAGTCCGGAGACGCCGGCGGGGGCCTCGGGAAGAGTTATCTTTTCTGCTTAACGGCCCGCC
AACCCTGGAAACGGTTCAGCCGGAGGTAGGGTCCAGCGGCCGGAAGAGCACCGCACGTCGCGCGG
TGTCCGGTGCGCCCCCGGCGGCCCTTGAAAATCCGGAGGACCGAGTACCGTCCACGCCCGGTCGTA
CTCATAACCGCATCAGGTCTCCAAGGTGAACAGCCTCTGGCCAATGGAACAATGTAGGCAAGGGAA
GTCGGCAAAACGGATCCGTAACTTCGGGAAAAGGATTGGCTCTGAGGGCTGGGCTCGGGGGTCCC
GGCCCCGAACCCGTCGGCTGCCGGCGGACTGCTCGAGCTGCTCGCGCGGCGAGAGCGGGCCGCCG
CGTGCCGGCCGGGGGACGGACCGGGAACGGCCCCCTCGGGGGCCTTCCCCGGGCGTCGAACAGCC
GACTCAGAACTGGTACGGACAAGGGGAATCCGACTGTTTAATTAAAACAAAGCATTGCGATGGTCC
TCGCGGATGCTGACGCAATGTGATTTCTGCCCAGTGCTCTGAATGTCAAAGTGAAGAAATTCAACCA
AGCGCGGGTAAACGGCGGGAGTAACTATGACTCTCTTAAGGTAGCCAAATGCCTCGTCATCTAATTA
GTGACGCGCATGAATGGATTAACGAGATTCCCACTGTCCCTGTCTACTATCCAGCGAAACCACAGCC
AAGGGAACGGGCTTGGCGGAATCAGCGGGGAAAGAAGACCCTGTTGAGCTTGACTCTAGTCCGAC
TTTGTGAAATGACTTGAGAGGTGTAGGATAAGTGGGAGCCCTCGGGCGCAAGTGAAATACCACTAC
TTTTAACGTTATTTTACTTATTCCGTGAGTCGGAAGCGGGGCCTGGCCCCTCCTTTTGGCTCTAAGGC
CCGAGTCCCTCGGGCCGATCCGGGCGGAAGACATTGTCAGGTGGGGAGTTTGGCTGGGGCGGCAC

ATCTGTTAAAAGATAACGCAGGTGTCCTAAGATGAGCTCAACGAGAACAGAAATCTCGTGTGGAAC
AAAAGGGTAAAAGCTCGTTTGATTCTGATTTCCAGTACGAATACGAACCGTGAAAGCGTGGCCTATC
GATCCTTTAGACCTTCGGAGTTTGAAGCTAGAGGTGTCAGAAAAGTTACCACAGGGATAACTGGCT
TGTGGCAGCCAAGCGTTCATAGCGACGTTGCTTTTTGATCCTTCGATGTCGGCTCTTCCTATCATTGT
GAAGCAGAATTCACCAAGTGTTGGATTGTTCACCCACCAATAGGGAACGTGAGCTGGGTTTAGACC
GTCGTGAGACAGGTTAGTTTTACCCTACTGATGACCGTGCCGCGATAGTAATTCAACCTAGTACGAG
AGGAACCGTTGATTCACACAATTGGTCATCGCGCTTGGTTGAAAAGCCAGTGGCGCGAAGCTACCG
TGTGCCGGATTATGACTGAACGCCTCTAAGTCAGAATCCAAGCTAGCAAGCGGCGCCTGCGCCCGC
CGCCCGCCCCGACCCACGTTAGGGGCGCAAGCCCCCAAGGGCCCGTGCCACTGGCCAAGCCGGCC
CGGCCGACGCGCCGCGGCCGGCCGCCTCGAAGCTCCCTTCCCAACGGGCGGCGGGCTGAATCCTTT
GCAGACGACTTAAATACGCGACGGGGCATTGTAAGTGGCAGAGTGGCCTTGCTGCCACGATCCACT
GAGATCCAGCCCCGCGTCGCACGGATTCGTCCCTCCCCCCACACACCCTCTCCCCCGCACCCCGCG
CAGGTTCTCCCCCCCGAGCCCGCCCCAGTCCCGCCGAGTCCCCAAGTCTCTCTAAGTCCGCTGCGTT
GGTGCCAAGGCGCGAA
>O.glumaepatula_IRGC88793_45S
ACCTGGTTGATCCTGCCAGTAGTCATATGCTTGTCTCAAAGATTAAGCCATGCATGTGCAAGTATGAA
CTAATTCGAACTGTGAAACTGCGAATGGCTCATTAAATCAGTTATAGTTTGTTTGATGGTACGTGCTA
CTCGGATAACCGTAGTAATTCTAGAGCTAATACGTGCAACAAACCCCGACTTCCGGGAGGGGCGCAT
TTATTAGATAAAAGGCTGACGCGGGCTCTGCCCGCTGATCCGATGATTCATGATAACTCGACGGATCG
CACGGCCCTCGTGCCGGCGACGCATCATTCAAATTTCTGCCCTATCAACTTTCGATGGTAGGATAGG
GGCCTACCATGGTGGTGACGGGTGACGGAGAATTAGGGTTCGATTCCGGAGAGGGAGCCTGAGAA
ACGGCTACCACATCCAAGGAAGGCAGCAGGCGCGCAAATTACCCAATCCTGACACGGGGAGGTAG
TGACAATAAATAACAATACCGGGCGCTTTAGTGTCTGGTAATTGGAATGAGTACAATCTAAATCCCTT
AACGAGGATCCATTGGAGGGCAAGTCTGGTGCCAGCAGCCGCGGTAATTCCAGCTCCAATAGCGTA
TATTTAAGTTGTTGCAGTTAAAAAGCTCGTAGTTGGACCTTGGGCCGGGTCGGCCGGTCCGCCTCAC
GGCGAGCACCGACCTACTCGACCCTTCTGCCGGCGATGCGCTCCTGGCCTTAACTGGCCGGGTCGT
GCCTCCGGCGCCGTTACTTTGAAGAAATTAGAGTGCTCAAAGCAAGCCATCGCTCTGGATACATTAG
CATGGGATAACATCATAGGATTCCGGTCCTATTGTGTTGGCCTTCGGGATCGGAGTAATGATTAATAG
GGACAGTCGGGGGCATTCGTATTTCATAGTCAGAGGTGAAATTCTTGGATTTATGAAAGACGAACAA
CTGCGAAAGCATTTGCCAAGGATGTTTTCATTAATCAAGAACGAAAGTTGGGGGCTCGAAGACGAT
CAGATACCGTCCTAGTCTCAACCATAAACGATGCCGACCAGGGATCGGCGGATGTTGCTTATAGGAC
TCCGCCGGCACCTTATGAGAAATCAAAGTCTTTGGGTTCCGGGGGGAGTATGGTCGCAAGGCTGAA
ACTTAAAGGAATTGACGGAAGGGCACCACCAGGCGTGGAGCCTGCGGCTTAATTTGACTCAACACG
GGGAAACTTACCAGGTCCAGACATAGCAAGGATTGACAGACTGAGAGCTCTTTCTTGATTCTATGGG
TGGTGGTGCATGGCCGTTCTTAGTTGGTGGAGCGATTTGTCTGGTTAATTCCGTTAACGAACGAGAC
CTCAGCCTGCTAACTAGCTATGCGGAGCCATCCCTCCGCAGCTAGCTTCTTAGAGGGACTATGGCCG
TTTAGGCCACGGAAGTTTGAGGCAATAACAGGTCTGTGATGCCCTTAGATGTTCTGGGCCGCACGCG
CGCTACACTGATGTATTCAACGAGTATATAGCCTTGGCCGACAGGCCCGGGTAATCTTGGGAAATTTC
ATCGTGATGGGGATAGATCATTGCAATTGTTGGTCTTCAACGAGGAATGCCTAGTAAGCGCGAGTCA
TCAGCTCGCGTTGACTACGTCCCTGCCCTTTGTACACACCGCCCGTCGCTCCTACCGATTGAATGGT
CCGGTGAAGTGTTCGGATCGCGGCGACGGGGGCGGTTCGCCGCCCCCGACGTCGCGAGAAGTCCA
TTGAACCTTATCATTTAGAGGAAGGAGAAGTCGTAACAAGGTTTCCGTAGGTGAACCTGCGGAAGG
ATCATTGTCGTGACCCTGACCAAAACAGACCGCGAACGCGTCACCCCTGCCCGCCGAGCGCTCGCG
CGCGAGGCAACCGAGGCCCCCGGGCCGCAACAGAACCCACGGCGCCGACGGCGTCAAGGAACAC
AGCGATACGCCCGCGCCGCCCGGTCGGCCCTGGCCATCCGGCGGCGCGGCGCGATACCACGAGTTA
ATCCACACGACTCTCGGCAACGGATATCTCGGCTCTCGCATCGATGAAGAACGTAGCGAAATGCGAT
ACCTGGTGTGAATTGCAGAATCCCGTGAACCATCGAGTCTTTGAACGCAAGTTGCGCCCGAGGCCA
TCCGGCCGAGGGCACGCCTGCCTGGGCGTCACGCCAAAAGACGCTCCACGCGCCCCCCCTATCCGG
GAGGGCGCGGGGACGCGGTGTCTGGCCTCCCGCGCCTCGCGGCGCGGCGGGCCGAAGCTCGGGCT
GCCGGCGAAGCGTGCCGGGCACAGCGCATGGTGGACAGCTCACGCTGGCTCTAGGCCGCAGTGCA
CCCCGGCGCGCGGCCGGCGCGATGGCCCCTCAGGACCCAAACGCACCGAGAGCGAACGCCTCGGA
CCGCGACCCCAGGTCAGGCGGGACTACCCGCTGAGTTTAAGCATATAAATAAGCGGAGGAGAAGAA
ACTTACGAGGATTCCCCTAGTAACGGCGAGCGAACCGGGAGATGCCCAGCTTGAGAATCGGGCGGC
CGCGCCGTCCGAATTGTAGTCTGGAGAGGCGTCCTCAGCGACGGACCGGGCCCAAGTCCCCTGGA
AAGGGGCGCCTGGGAGGGTGAGAGCCCCGTCCGGCCCGGACCCTGTCGCCCCACGAGGCGCCGTC
AACGAGTCGGGTTGTTTGGGAATGCAGCCCAAATCGGGCGGTAAACTCCGTCCAAGGCTAAATACA

GGCGAGAGACCGATAGCGAACAAGTACCGCGAGGGAAAGATGAAAAGGACTTTGAAAAGAGAGT
CAAAGAGTGCTTGAAATTGCCGGGAGGGAAGCGGATGGGGGCCGGCGATGCGCCCCGGCCGTATG
CGGAACGGCTTCGGCTGGTCCGCCGATCGGCTCGGGGCGTGGACTGTTGTCGGCCGCGCCGGCGGC
CAAAGCCCGGGGGCTCCGCGCCCCCGGCAGCCGTCGTCGGCGCAGCCGGTCACCGCGCGCCTCTG
GCGCGCCCCTCGGGGCGCTGCGCTGCAACGGCCTGCGGGCTCCCCATCCGACCCGTCTTGAAACAC
GGACCAAGGAGTCTGACATGCGTGCGAGTCGACGGGTTCTGAAACCTGGGATGCGCAAGGAAGCT
GACGAGCGGGAGGCCCTCACGGGCCGCACCGCTGGCCGACCCTGATCTTCTGTGAAGGGTTCGAG
TTGGAGCACGCCTGTCGGGACCCGAAAGATGGTGAACTATGCCTGAGCGGGGCGAAGCCAGAGGA
AACTCTGGTGGAGGCTCGAAGCGATACTGACGTGCAAATCGTTCGTCTGACTTGGGTATAGGGGCG
AAAGACTAATCGAACCATCTAGTAGCTGGTTCCCTCCGAAGTTTCCCTCAGGATAGCTGGAGCCCAT
TACGAGTTCTATCGGGTAAAGCCAATGATTAGAGGCATCGGGGGCGCAACGCCCTCGACCTATTCTC
AAACTTTAAATAGGTAGGACGGCGCGGCTGCTCCGGTGAGCCGCGCCACGGAATCGGGAGCTCCAA
GTGGGCCATTTTTGGTAAGCAGAACTGGCGATGCGGGATGAACCGGAAGCCTGGTTACGGTGCCGA
ACTGCGCGCTAACCTAGAACCCACAAAGGGTGTTGGTCGATTAAGACAGCAGGACGGTGGTCATGG
AAGTCGAAATCCGCTAAGGAGTGTGTAACAACTCACCTGCCGAATCAACTAGCCCCGAAAATGGAT
GGCGCTGAAGCGCGCGACCCACACCAGGCCATCTGGGCGAGCGCCAAGCCCCGATGAGTAGGAGG
GCGCGGCGGCCGCCGCAAAACCCGGGGCGCGAGCCCGGGCGGAGCGGCCGTCGGTGCAGATCTTG
GTGGTAGTAGCAAATATTCAAATGAGAACTTTGAAGGCCGAAGAGGAGAAAGGTTCCATGTGAACG
GCACTTGCACATGGGTAAGCCGATCCTAAGGGACGGGGTAACCCCGGCAGAGAGCGCGACCACGC
GCGTGCCCCGAAAGGGAATCGGGTTAAGATTTCCCGAGCCGGGACGTGGCGGTTGACGGCGACGT
TAGGAAGTCCGGAGACGCCGGCGGGGGCCTCGGGAAGAGTTATCTTTTCTGCTTAACGGCCCGCCA
ACCCTGGAAACGGTTCAGCCGGAGGTAGGGTCCAGCGGCCGGAAGAGCACCGCACGTCGCGCGGT
GTCCGGTGCGCCCCCGGCGGCCCTTGAAAATCCGGAGGACCGAGTACCGTCCACGCCCGGTCGTAC
TCATAACCGCATCAGGTCTCCAAGGTGAACAGCCTCTGGCCAATGGAACAATGTAGGCAAGGGAAG
TCGGCAAAACGGATCCGTAACTTCGGGAAAAGGATTGGCTCTGAGGGCTGGGCTCGGGGGTCCCG
GCCCCGAACCCGTCGGCTGCCGGCGGACTGCTCGAGCTGCTCGCGCGGCGAGAGCGGGCCGCCGC
GTGCCGGCCGGGGGACGGACCGGGAACGGCCCCCTCGGGGGCCTTCCCCGGGCGTCGAACAGCCG
ACTCAGAACTGGTACGGACAAGGGGAATCCGACTGTTTAATTAAAACAAAGCATTGCGATGGTCCT
CGCGGATGCTGACGCAATGTGATTTCTGCCCAGTGCTCTGAATGTCAAAGTGAAGAAATTCAACCA
AGCGCGGGTAAACGGCGGGAGTAACTATGACTCTCTTAAGGTAGCCAAATGCCTCGTCATCTAATTA
GTGACGCGCATGAATGGATTAACGAGATTCCCACTGTCCCTGTCTACTATCCAGCGAAACCACAGCC
AAGGGAACGGGCTTGGCGGAATCAGCGGGGAAAGAAGACCCTGTTGAGCTTGACTCTAGTCCGAC
TTTGTGAAATGACTTGAGAGGTGTAGGATAAGTGGGAGCCCTCGGGCGCAAGTGAAATACCACTAC
TTTTAACGTTATTTTACTTATTCCGTGAGTCGGAAGCGGGGCCTGGCCCCTCCTTTTGGCTCTAAGGC
CCGAGTCCCTCGGGCCGATCCGGGCGGAAGACATTGTCAGGTGGGGAGTTTGGCTGGGGCGGCAC
ATCTGTTAAAAGATAACGCAGGTGTCCTAAGATGAGCTCAACGAGAACAGAAATCTCGTGTGGAAC
AAAAGGGTAAAAGCTCGTTTGATTCTGATTTCCAGTACGAATACGAACCGTGAAAGCGTGGCCTATC
GATCCTTTAGACCTTCGGAGTTTGAAGCTAGAGGTGTCAGAAAAGTTACCACAGGGATAACTGGCT
TGTGGCAGCCAAGCGTTCATAGCGACGTTGCTTTTTGATCCTTCGATGTCGGCTCTTCCTATCATTGT
GAAGCAGAATTCACCAAGTGTTGGATTGTTCACCCACCAATAGGGAACGTGAGCTGGGTTTAGACC
GTCGTGAGACAGGTTAGTTTTACCCTACTGATGACCGTGCCGCGATAGTAATTCAACCTAGTACGAG
AGGAACCGTTGATTCACACAATTGGTCATCGCGCTTGGTTGAAAAGCCAGTGGCGCGAAGCTACCG
TGTGCCGGATTATGACTGAACGCCTCTAAGTCAGAATCCAAGCTAGCAAGCGGCGCCTGCGCCCGC
CGCCCGCCCCGACCCACGTTAGGGGCGCAAGCCCCCAAGGGCCCGTGCCACTGGCCAAGCCGGCC
CGGCCGACGCGCCGCGGCCGGCCGCCTCGAAGCTCCCTTCCCAACGGGCGGCGGGCTGAATCCTTT
GCAGACGACTTAAATACGCGACGGGGCATTGTAAGTGGCAGAGTGGCCTTGCTGCCACGATCCACT
GAGATCCAGCCCCGCGTCGCACGGATTCGTCCCTCCCCCTCTCCCCCGCGCCCCGCGCAGGTTCTCC
CCCCCGAGCCCGCCCCAATCCCGCCGAGTCCCCAGGTCTCTCTAG
>O.meridionalis_IRGC105298_45S
ACCTGGTTGATCCTGCCAGTAGTCATATGCTTGTCTCAAAGATTAAGCCATGCATGTGCAAGTATGAA
CTAATTCGAACTGTGAAACTGCGAATGGCTCATTAAATCAGTTATAGTTTGTTTGATGGTACGTGCTA
CTCGGATAACCGTAGTAATTCTAGAGCTAATACGTGCAACAAACCCCGACTTCCGGGAGGGGCGCAT
TTATTAGATAAAAGGCTGACGCGGGCTCTGCCCGCTGATCCGATGATTCATGATAACTCGACGGATCG
CACGGCCCTCGTGCCGGCGACGCATCATTCAAATTTCTGCCCTATCAACTTTCGATGGTAGGATAGG
GGCCTACCATGGTGGTGACGGGTGACGGAGAATTAGGGTTCGATTCCGGAGAGGGAGCCTGAGAA
ACGGCTACCACATCCAAGGAAGGCAGCAGGCGCGCAAATTACCCAATCCTGACACGGGGAGGTAG

TGACAATAAATAACAATACCGGGCGCTTTAGTGTCTGGTAATTGGAATGAGTACAATCTAAATCCCTT
AACGAGGATCCATTGGAGGGCAAGTCTGGTGCCAGCAGCCGCGGTAATTCCAGCTCCAATAGCGTA
TATTTAAGTTGTTGCAGTTAAAAAGCTCGTAGTTGGACCTTGGGCCGGGTCGGCCGGTCCGCCTCAC
GGCGAGCACCGACCTACTCGACCCTTCTGCCGGCGATGCGCTCCTGGCCTTAACTGGCCGGGTCGT
GCCTCCGGCGCCGTTACTTTGAAGAAATTAGAGTGCTCAAAGCAAGCCATCGCTCTGGATACATTAG
CATGGGATAACATCATAGGATTCCGGTCCTATTGTGTTGGCCTTCGGGATCGGAGTAATGATTAATAG
GGACAGTCGGGGGCATTCGTATTTCATAGTCAGAGGTGAAATTCTTGGATTTATGAAAGACGAACAA
CTGCGAAAGCATTTGCCAAGGATGTTTTCATTAATCAAGAACGAAAGTTGGGGGCTCGAAGACGAT
CAGATACCGTCCTAGTCTCAACCATAAACGATGCCGACCAGGGATCGGCGGATGTTGCTTATAGGAC
TCCGCCGGCACCTTATGAGAAATCAAAGTCTTTGGGTTCCGGGGGGAGTATGGTCGCAAGGCTGAA
ACTTAAAGGAATTGACGGAAGGGCACCACCAGGCGTGGAGCCTGCGGCTTAATTTGACTCAACACG
GGGAAACTTACCAGGTCCAGACATAGCAAGGATTGACAGACTGAGAGCTCTTTCTTGATTCTATGGG
TGGTGGTGCATGGCCGTTCTTAGTTGGTGGAGCGATTTGTCTGGTTAATTCCGTTAACGAACGAGAC
CTCAGCCTGCTAACTAGCTATGCGGAGCCATCCCTCCGCAGCTAGCTTCTTAGAGGGACTATGGCCG
TTTAGGCCACGGAAGTTTGAGGCAATAACAGGTCTGTGATGCCCTTAGATGTTCTGGGCCGCACGCG
CGCTACACTGATGTATTCAACGAGTATATAGCCTTGGCCGACAGGCCCGGGTAATCTTGGGAAATTTC
ATCGTGATGGGGATAGATCATTGCAATTGTTGGTCTTCAACGAGGAATGCCTAGTAAGCGCGAGTCA
TCAGCTCGCGTTGACTACGTCCCTGCCCTTTGTACACACCGCCCGTCGCTCCTACCGATTGAATGGT
CCGGTGAAGTGTTCGGATCGCGGCGACGGGGGCGGTTCGCCGCCCCCGACGTCGCGAGAAGTCCA
TTGAACCTTATCATTTAGAGGAAGGAGAAGTCGTAACAAGGTTTCCGTAGGTGAACCTGCGGAAGG
ATCATTGTCGTGACCCTGACCAAAACAGACCGCGAACGCGTCACCCCTGCCCGCCGAGCGCTCGCG
CGCGAGGCAACCGAGGCCCCCGGGCCGCAACAGAACCCACGGCGCCGACGGCGTCAAGGAACAC
AGCGATACGCCCGCGCCGCCCGGTCGGCCCTGGCCGTCCGGCGGCGCGGCGCGATACCACGAGTTA
ATCCACACGACTCTCGGCAACGGATATCTCGGCTCTCGCATCGATGAAGAACGTAGCGAAATGCGAT
ACCTGGTGTGAATTGCAGAATCCCGTGAACCATCGAGTCTTTGAACGCAAGTTGCGCCCGAGGCCA
TCCGGCCGAGGGCACGCCTGCCTGGGCGTCACGCCAAAAGACGCTCCACGCGCCCCCCTATCCGGG
AGGGCGCGGGGACGCGGTGTCTGGCCTCCCGCGCCTCGCGGCGCGGCGGGCCGAAGCTCGGGCTG
CCGGCGAAGCGTGCCGGGCACAGCGCATGGTGGACAGCTCACGCTGGCTCTAGGCCGCAGTGCAC
CCCGGCGCGCGGCCGGCGCGATGGCCCCTCAGGACCCAAACGCACCGAGAGCGAACGCCTCGGAC
CGCGACCCCAGGTCAGGCGGGACTACCCGCTGAGTTTAAGCATATAAATAAGCGGAGGAGAAGAAA
CTTACGAGGATTCCCCTAGTAACGGCGAGCGAACCGGGAGATGCCCAGCTTGAGAATCGGGCGGCC
GCGCCGTCCGAATTGTAGTCTGGAGAGGCGTCCTCAGCGACGGACCGGGCCCAAGTCCCCTGGAA
AGGGGCGCCTGGGAGGGTGAGAGCCCCGTCCGGCCCGGACCCTGTCGCCCCACGAGGCGCCGTCA
ACGAGTCGGGTTGTTTGGGAATGCAGCCCAAATCGGGCGGTAAACTCCGTCCAAGGCTAAATACAG
GCGAGAGACCGATAGCGAACAAGTACCGCGAGGGAAAGATGAAAAGGACTTTGAAAAGAGAGTC
AAAGAGTGCTTGAAATTGCCGGGAGGGAAGCGGATGGGGGCCGGCGATGCGCCCCGGCCGTATGC
GGAACGGCTCCGGCTGGTCCGCCGATCGGCTCGGGGCGTGGACTGTTGTCGGCCGCGCCGGCGGC
CAAAGCCCGGGGGCTCCGCGCCCCCGGCAGCCGTCGTCGGCGCAGCCGGTCACCGCGCGCCTCTG
GCGCGCCCCTCGGGGCGCTGCGCTGCAACGGCCTGCGGGCTCCCCATCCGACCCGTCTTGAAACAC
GGACCAAGGAGTCTGACATGCGTGCGAGTCGACGGGTTCTGAAACCTGGGATGCGCAAGGAAGCT
GACGAGCGGGAGGCCCTCACGGGCCGCACCGCTGGCCGACCCTGATCTTCTGTGAAGGGTTCGAG
TTGGAGCACGCCTGTCGGGACCCGAAAGATGGTGAACTATGCCTGAGCGGGGCGAAGCCAGAGGA
AACTCTGGTGGAGGCTCGAAGCGATACTGACGTGCAAATCGTTCGTCTGACTTGGGTATAGGGGCG
AAAGACTAATCGAACCATCTAGTAGCTGGTTCCCTCCGAAGTTTCCCTCAGGATAGCTGGAGCCCAT
TACGAGTTCTATCGGGTAAAGCCAATGATTAGAGGCATCGGGGGCGCAACGCCCTCGACCTATTCTC
AAACTTTAAATAGGTAGGACGGCGCGGCTGCTCCGGTGAGCCGCGCCACGGAATCGGGAGCTCCAA
GTGGGCCATTTTTGGTAAGCAGAACTGGCGATGCGGGATGAACCGGAAGCCTGGTTACGGTGCCGA
ACTGCGCGCTAACCTAGAACCCACAAAGGGTGTTGGTCGATTAAGACAGCAGGACGGTGGTCATGG
AAGTCGAAATCCGCTAAGGAGTGTGTAACAACTCACCTGCCGAATCAACTAGCCCCGAAAATGGAT
GGCGCTGAAGCGCGCGACCCACACCAGGCCATCTGGGCGAGCGCCATGCCCCGATGAGTAGGAGG
GCGCGGCGGCCGCCGCAAAACCCGGGGCGCGAGCCCGGGCGGAGCGGCCGTCGGTGCAGATCTTG
GTGGTAGTAGCAAATATTCAAATGAGAACTTTGAAGGCCGAAGAGGAGAAAGGTTCCATGTGAACG
GCACTTGCACATGGGTAAGCCGATCCTAAGGGACGGGGTAACCCCGGCAGAGAGCGCGACCACGC
GCGTGCCCCGAAAGGGAATCGGGTTAAGATTTCCCGAGCCGGGACGTGGCGGTTGACGGCGACGT
TAGGAAGTCCGGAGACGCCGGCGGGGGCCTCGGGAAGAGTTATCTTTTCTGCTTAACGGCCCGCCA
ACCCTGGAAACGGCTCAGCCGGAGGTAGGGTCCAGCGGCCGGAAGAGCACCGCACGTCGCGCGGT

GTCCGGTGCGCCCCCGGCGGCCCTTGAAAATCCGGAGGACCGAGTACCGTCCACGCCCGGTCGTAC
TCATAACCGCATCAGGTCTCCAAGGTGAACAGCCTCTGGCCAATGGAACAATGTAGGCAAGGGAAG
TCGGCAAAACGGATCCGTAACTTCGGGAAAAGGATTGGCTCTGAGGGCTGGGCTCGGGGGTCCCG
GCCCCGAACCCGTCGGCTGCCGGCGGACTGCTCGAGCTGCTCGCGCGGCGAGAGCGGGCCGCCGC
GTGCCGGCCGGGGGACGGACCGGGAACGGCCCCCTCGGGGGCCTTCCCCGGGCGTCGAACAGCCG
ACTCAGAACTGGTACGGACAAGGGGAATCCGACTGTTTAATTAAAACAAAGCATTGCGATGGTCCT
CGCGGATGCTGACGCAATGTGATTTCTGCCCAGTGCTCTGAATGTCAAAGTGAAGAAATTCAACCA
AGCGCGGGTAAACGGCGGGAGTAACTATGACTCTCTTAAGGTAGCCAAATGCCTCGTCATCTAATTA
GTGACGCGCATGAATGGATTAACGAGATTCCCACTGTCCCTGTCTACTATCCAGCGAAACCACAGCC
AAGGGAACGGGCTTGGCGGAATCAGCGGGGAAAGAAGACCCTGTTGAGCTTGACTCTAGTCCGAC
TTTGTGAAATGACTTGAGAGGTGTAGGATAAGTGGGAGCCCTCGGGCGCAAGTGAAATACCACTAC
TTTTAACGTTATTTTACTTATTCCGTGAGTCGGAAGCGGGGCCTGGCCCCTCCTTTTGGCTCTAAGGC
CCGAGTCCCTCGGGCCGATCCGGGCGGAAGACATTGTCAGGTGGGGAGTTTGGCTGGGGCGGCAC
ATCTGTTAAAAGATAACGCAGGTGTCCTAAGATGAGCTCAACGAGAACAGAAATCTCGTGTGGAAC
AAAAGGGTAAAAGCTCGTTTGATTCTGATTTCCAGTACGAATACGAACCGTGAAAGCGTGGCCTATC
GATCCTTTAGACCTTCGGAGTTTGAAGCTAGAGGTGTCAGAAAAGTTACCACAGGGATAACTGGCT
TGTGGCAGCCAAGCGTTCATAGCGACGTTGCTTTTTGATCCTTCGATGTCGGCTCTTCCTATCATTGT
GAAGCAGAATTCACCAAGTGTTGGATTGTTCACCCACCAATAGGGAACGTGAGCTGGGTTTAGACC
GTCGTGAGACAGGTTAGTTTTACCCTACTGATGACCGTGCCGCGATAGTAATTCAACCTAGTACGAG
AGGAACCGTTGATTCACACAATTGGTCATCGCGCTTGGTTGAAAAGCCAGTGGCGCGAAGCTACCG
TGTGCCGGATTATGACTGAACGCCTCTAAGTCAGAATCCAAGCTAGCAAGCGGCGCCTGCGCCCGC
CGCCCGCCCCGACCCACGTTAGGGGCGCAAGCCCCCAAGGGCCCGTGCCACCGGCCAAGCCGGCC
CGGCCGACGCGCCGCGGCCGGCCGCCTCGAAGCTCCCTTCCCAACGGGCGGCGGGCTGAATCCTTT
GCAGACGACTTAAATACGCGACGGGGCATTGTAAGTGGCAGAGTGGCCTTGCTGCCACGATCCACT
GAGATCCAGCCCCGCGTCGCACGGATTCGTCCCTCCCCCCCCCCACACCCTCTCCCCCGGACCCCGC
GCAGGTTCTCCGCCCCCCCCGAAT

Supplementary Dataset S3.

The 5S transcription unit sequences of seven *Oryza* species. WGS sequences of these seven accessions were retrieved from GenBank (Table 1) and the 5S sequence was obtained using the dnaLCW method. The header of each FASTA sequence indicates species name and accession number.

>O.rufipogon_AC11-1008369_5S
GGATGCGATCATACCAGCACTAAAGCACCGGATCCCATCAGAACTCCGAAGTTAAGCGTGCTTGGG
CGAGAGTAGTACTAGGATGGGTGACCTCCTGGGAAGTCCTCGTGTTGCATCCCTCCTTTTTGTCTCT
CTCTCCCCCCTTTTGACTCGCGCCGCTGCGTCCCTCGTGTTGTGTCGCCCCTTGGGCGAGAGCTGGG
GAGAATCGGATGTAACATTTTCTGTAGATGTCCGTGGATATATCATTTGCCTGATTCCGAGTCCGTATG
AGAAAGTTACGCCTATTTTAAGAAATGACACCCGAATGACGCCAAAGCATGTC
>O.rufipogon_AC01-1002323_5S
GGATGCGATCATACCAGCACTAAAGCACCGGATCCCATCAGAACTCCGAAGTTAAGCGTGCTTGGG
CGAGAGTAGTACTAGGATGGGTGACCTCCTGGGAAGTCCTCGTGTTGCATCCGTCCTTTTTGACTCG
CGCCGCTACATCCCTCGTGCTGTGTTGTGTCGTCCTTTTTGTCTCTCTCACCTTTTGACCCGCGCCGT
TACATCTCTCGTGCTGTGATGTGTCGTCCCACGCACGAGAGCTGGGGAGAATCGGATGTAACATTTT
CTGTAGATGTCCGTGGATATATTATTTGCCTGATTCCGAGTCCGTATGAGAAAGTTACGCCCATTTTAA
GAAATGACACCCGAATGACGCCTAGGCCGGCACAGGTTTTCGCCGGCCGAACGAGGGAGCGAGGG
CCGCCCGCGGCAGCCCGTTTCAGCAACGGGCGCACGGCTCACGCTCAGCCGCGCGCGGGGCGAGG
AGCACAAGAGGGGCTGGGGCGCAAGACATGTC
>O.nivara_IRGC88812_5S
GGATGCGATCATACCAGCACTAAAGCACCGGATCCCATCAGAACTCCGAAGTTAAGCGTGCTTGGG
CGAGAGTAGTACTAGGATGGGTGACCTCCTGGGAAGTCCTCGTGTTGCATCCCTCCTTTTTGACTCT
CTCTCCCCCCTTTTGACTCGCGCCGCTGCGTCCCCCGTGTTGTGTCGCCTCTTGGGCGAGAGCTGGG
GAGAATCGGATGTAACATTTTCTGTAGATGTCCGTGGATATATCATTTGCCTGATTCCGAGTCCGTATG
AGAAAGTTACGCCTATTTTAAGAAATGACACCCGAATGACGCGAAAGCATGTC
>O.barthii_IRGC101252_5S
GGATGCGATCATACCAGCACTAAAGCACCGGATCCCATCAGAACTCCGAAGTTAAGCGTGCTTGGG
CGAGAGTAGTACTAGGATGGGTGACCTCCTGGGAAGTCCTCGTGTTCCATCCCTCCTTTTTGTCGTC
TCTCTCTCCCCTCTTTTGACTCGCGCCGCTGCGTCCCTCGTGTTGTCTCGCTCCTTGGGCGAGAGCT
GCGGAGAATCGGATGTAACATTTTCTGTAGATGTCCGTGGATATATCATTTGCCTGATTCCGAGTCCG
TATGAGAAAGTTACGCCTATTTTAAGAAATGACATCCGAATGACGCCTAGACATATC
>O.glaberrima_IRGC103486_5S
GGATGCGATCATACCAGCACTAAAGCACCGGATCCCATCAGAACTCCGAAGTTAAGCGTGCTTGGG
CGAGAGTAGTACTAGGATGGGTGACCTCCTGGGAAGTCCTCGTGTTGCATCCCTCCTTTTTGTCGTC
TCTCTCTCCCCTCTTTTGACTCGCGCCGCTGCGTCCCTCGTGTTGTGCCGCTCCTTGGGCGAGAGCT
GCGGAGAATCGGATGTAACATTTTCTGTAGATGTCCGTGGATATATCATTTGCCTGATTCCGAGTCCG
TATGAGAAAGTTACGCCTATTTTAAGAAATGACATCCGAATGACGCCTAGACATATC
>O.glumaepatula_IRGC88793_5S
GGATGCGATCATACCAGCACTAAAGCACCGGATCCCATCAGAACTCCGAAGTTAAGCGTGCTTGGG
CGAGAGTAGTACTAGGATGGGTGACCTCCTGGGAAGTCCTCGTGTTGCATCCCTCCTTTTTTTTTTCT
CCCTCTCTCCGCTGCGTCCCTCGTGTTGTGTCGTCCCATGGGCGAGAGCTGGGGAGAATCGGATGTA
AAATTTTCTGTAGATGTCCGTGGATATATCATTTGCCTGATTCCGAGTCCGTATGAGAAAGTTACGCC
TATTTTAAGAAATGACACCCGAATGACGCCTAGGCCGGCACAGGTTTTCGCCGGCCAAACGAGGGA
GCGAGGGCCGCACGCGGCAGCCTGTTCCGGCAACGGGCGCACGGCTCACGCTCCGGCCGCGCGCG
GGGCGAGGAGCACAAGAGGGGCGGGGGCGCAAGGCATGTC
>O.meridionalis_IRGC105298_5S
GGATGCGATCATACCAGCACTAAAGCACCGGATCCCATCAGAACTCCGAAGTTAAGCGTGCTTGGG
CGAGAGTAGTACTAGGATGGGTGACCTCCTGGGAAGTCCTCGTGTTGCATCCGTCCTTTTTGACTCG
CGCCGCTACATCCCTCGTGCTGTGTTGTGTCGTCCTTTTTGTCTCTCTCACCTTTTGACTCGCGCCGT
TACATCTCTCGTGCTGTGATGTGTCGTCCCACGCACGAGAGCTGGGGAGAATCGGATGTAACATTTT
CTGTAGATGTCCGTGGATATATTATTTGCCTGATTCCGAGTCCGTATGAGAAAGTTACGCCTATTTTAA
GAAATGACACCCGAATGACGCCTAGGCCGGCACAGGTTTTCGCCGGCCGAACGAGGGAGCGAGGG
CCGCCCGCGGCAGCCCGTTTCAGCAACGGGCGCACGGCTCACGCTCAGCCGCGCGCGGGGCGAGG
AGCACAAGAGGGGCTGGGGCGCAAGACATGTC
